# Emergence of novel SARS-CoV-2 variants in the Netherlands

**DOI:** 10.1038/s41598-021-85363-7

**Published:** 2021-03-23

**Authors:** Aysun Urhan, Thomas Abeel

**Affiliations:** 1 Delft Bioinformatics Lab, Delft University of Technology Van Mourik, Broekmanweg 6, 2628 XE Delft, The Netherlands; 2 Infectious Disease and Microbiome Program, Broad Institute of MIT and Harvard, 415 Main Street, Cambridge, MA 02142 USA

**Keywords:** Computational biology and bioinformatics, Phylogeny, Bioinformatics, Comparative genomics, Viral genetics, Applied microbiology

## Abstract

Coronavirus disease 2019 (COVID-19) has emerged in December 2019 when the first case was reported in Wuhan, China and turned into a pandemic with 27 million (September 9th) cases. Currently, there are over 95,000 complete genome sequences of the severe acute respiratory syndrome coronavirus 2 (SARS-CoV-2), the virus causing COVID-19, in public databases, accompanying a growing number of studies. Nevertheless, there is still much to learn about the viral population variation when the virus is evolving as it continues to spread. We have analyzed SARS-CoV-2 genomes to identify the most variant sites, as well as the stable, conserved ones in samples collected in the Netherlands until June 2020. We identified the most frequent mutations in different geographies. We also performed a phylogenetic study focused on the Netherlands to detect novel variants emerging in the late stages of the pandemic and forming local clusters. We investigated the S and N proteins on SARS-CoV-2 genomes in the Netherlands and found the most variant and stable sites to guide development of diagnostics assays and vaccines. We observed that while the SARS-CoV-2 genome has accumulated mutations, diverging from reference sequence, the variation landscape is dominated by four mutations globally, suggesting the current reference does not represent the virus samples circulating currently. In addition, we detected novel variants of SARS-CoV-2 almost unique to the Netherlands that form localized clusters and region-specific sub-populations indicating community spread. We explored SARS-CoV-2 variants in the Netherlands until June 2020 within a global context; our results provide insight into the viral population diversity for localized efforts in tracking the transmission of COVID-19, as well as sequenced-based approaches in diagnostics and therapeutics. We emphasize that little diversity is observed globally in recent samples despite the increased number of mutations relative to the established reference sequence. We suggest sequence-based analyses should opt for a consensus representation to adequately cover the genomic variation observed to speed up diagnostics and vaccine design.

## Introduction

In late December 2019, officials had reported the first case of coronavirus disease 2019 (COVID-19) in China, caused by a novel type of coronavirus named severe acute respiratory syndrome coronavirus 2, (SARS-CoV-2)^1^. COVID-19 has consequently led to the global pandemic we are going through at the moment; according a situation report released by the World Health Organization (September 9th) there are 27.4 million cases and almost 900,000 deaths in total^2^. SARS-CoV-2 has been placed under the betacoronavirus genus, closest relatives being bat and pangolin coronaviruses^3,4^.

Despite having major commonalities with recent outbreaks of betacoronaviruses, SARS in 2002 and Middle East respiratory syndrome (MERS) in 2012, it is unprecedented not only in its ease of spread but also in the collective effort of several international scientists to investigate and understand the biology of the disease and the virus causing it since the day the first complete SARS-CoV-2 genome sequence had been published^4–7^. Early studies on the SARS-CoV-2 genome has shown its closest relative, in terms of sequence identity, to be the bat coronavirus RaTF13 with over 93.1% match in the spike (S) protein and > 96% sequence identity overall^5,8^. Immediately a reference sequence had been established^9^, paving the way for the exponential growth in both the number and the scale of studies on the SARS-CoV-2 genome^10–15^.

At the moment, the GISAID database has established the SARS-CoV-2 population consists of six major clades: G, GH, GR, L, S and V^16^. There is a growing number of studies on the genetic variability of SARS-CoV-2 relative to the reference genome^17–20^. From previous viral outbreaks, it is known that as part of the natural evolution of a virus, subpopulations of clades that can affect the severity of a disease emerge and alter the trajectory of a pandemic^21^. It has been reported that while the two major structural proteins, S and nucleocapsid (N) protein are rich in sites of episodic selection, ORF3a and ORF8 had also been shown to carry a lot of mutations^22^.

In this study, we investigate the genetic variability of SARS-CoV-2 genomes in the Netherlands until mid-2020, in the context of global viral population with a particular focus on the later stages of the first wave of the pandemic (from early April to the end of May). We have identified the most variant proteins in the SARS-CoV-2 genome, as well as the most frequent mutations in the Netherlands that also showed high dominance in the rest of the world. We found relatively conserved regions in the S and N proteins of SARS-CoV-2, as well as frequent mutations on the target regions of some RT-qPCR diagnostic tests. Tracing the viral genome since its first introduction into the Netherlands, we detected novel mutations unique to the Netherlands, and local clusters of distinct viral sub-populations emerging in different provinces. Our work provides valuable insights into the regional variance of SARS-CoV-2 populations in the Netherlands that would prove beneficial for localized efforts in tracking routes of transmission through genetic variation, primer/probe design in RT-qPCR tests targeting viral sub-populations. We recommend that emergent variants are examined when developing sequence-based diagnostics, vaccines or therapeutics against COVID-19. In order to do so, genomic surveillance needs to continue at a sufficiently high level throughout the course of the pandemic.

## Methods

Our study of SARS-COV-2 genomes in the Netherlands consists of three main steps: data retrieval, preprocessing and multiple sequence alignment, phylogenetic tree construction and sequence variation analysis. We have also analyzed the global phylogenetic tree of SARS-COV-2 genomes using additional metadata on patients and travel history.

### Data retrieval and preprocessing, and multiple sequence alignment

Complete, high quality (number of undetermined bases less than 1% of the whole sequence) genome sequences of SARS-COV-2 that were isolated from human hosts only were obtained from GISAID, NCBI and China’s National Genomics Data Center (NGDC) on June 13th^16,23,24^. The dataset contained 29,503 sequences with unique identifiers in total, including the Wuhan-Hu-1 reference sequence (accession ID NC_045512.2). The “Collection date” field was also extracted for all sequences, and it is referred to as “date” throughout this work. The acknowledgement table for GISAID sequences can be found in Supplementary file 2 and the full list of sequence identifiers for NCBI and NGDC records are provided in Supplementary file 3.

All sequences were aligned against the Wuhan-Hu-1 reference using MAFFT (v7.46) with the FFT-NS-fragment option, and the alignment was filtered to remove identical sequences to obtain 24,365 non-redundant genomes^25^.

### Sequence variation analysis

In order to determine mutations, the filtered multiple sequence alignment was trimmed to remove gaps from the Wuhan-Hu-1 reference (accession ID NC_045512.2) and used as input to the *coronapp* web application to obtain nucleotide variations^26^. Next, the trimmed alignment was used to cluster genomes according to the nomenclature on GISAID website. We assigned all 29,503 sequences to one of the clades S, L, V, G, GH and GR.

We retrieved primer/probe sequence sets released by US CDC, WHO, Institut Pasteur and China CDC, and identified mutations which overlap with these sequences^27–30^.

### Phylogenetic tree construction

The maximum likelihood phylogenetic tree for the samples in the Netherlands (1338 genomes in total) was built using IQ-TREE (v2.05) with GTR model, allowing to collapse non-zero branches, and ultrafast bootstrap with 1000 replicates^31^. A time tree was also constructed for dating branches in IQ-TREE (v2.05) and the final tree was rooted at the ancestral node of S clades in the tree using ETE Toolkit (v3.1.1)^32^. ETE was also used for visualizing tree. Collection date and region (within the Netherlands) fields of each sequence record (if available) were retrieved, and utilized to infer the spread of variants within the Netherlands.

## Results

**Figure 1:**
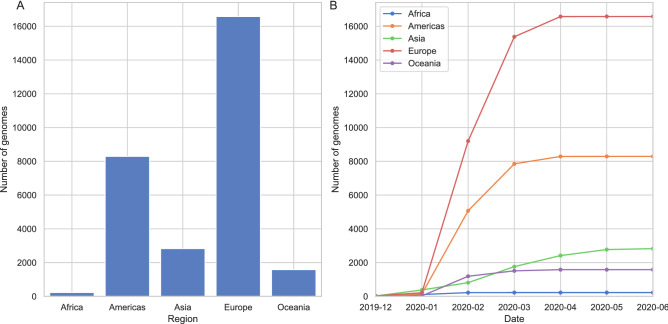
Distribution of SARS-CoV-2 genomes across five continents: (**A**) total number of genomes is shown on y-axis and the regions in x-axis, (**B**) the change in number of genomes collected over the course of pandemic, x-axis shows the collection date. See (**B**) for colors of each continent.

The global SARS-CoV-2 dataset was filtered considering only the sequence quality, hence we observe a large discrepancy in the distribution of genomes across different countries. Initially, most sequencing effort was concentrated in China and other countries where the outbreak had begun. However, at the time of data retrieval (June 13th) the dataset is dominated by samples from the UK, the USA and Australia (Table 1 and Fig. 1).

**Table 1:** 20 countries with the largest number of genomes in the dataset.

Country	Number of genomes
The UK	9641
The USA	7294
Australia	1398
The Netherlands	1338
Spain	886
India	710
China	651
Belgium	645
Denmark	581
Canada	560
Portugal	500
Iceland	481
France	376
Sweden	353
Switzerland	314
Singapore	285
Austria	247
Russia	218
Germany	209
Luxembourg	192

Since we have not corrected for sampling differences, in this section, we will provide a view of the current situation of pandemic mainly in Europe, focusing on the Netherlands, where most of the viral genomes are available today (Fig. 1). While initially many genome sequences were generated, by April virtually no sequences were determined.

### Distinct genetic patterns in the SARS-CoV-2 population emerge across the globe

**Figure 2:**
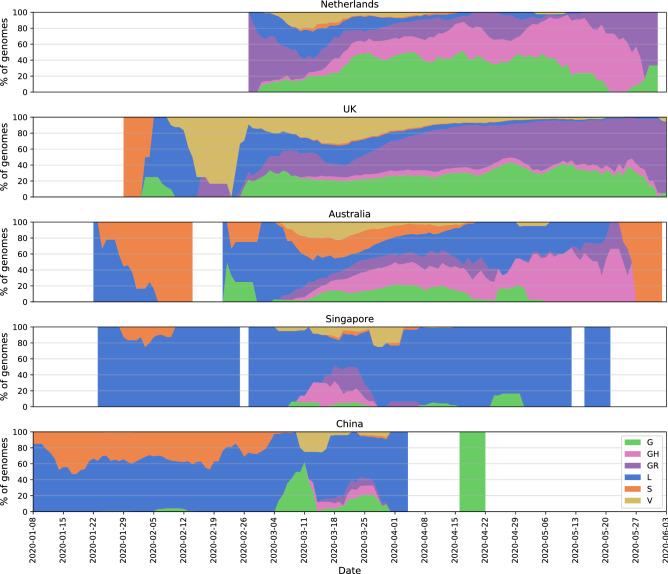
Distribution of SARS-CoV-2 clades in a selection among the 12 most sampled countries in comparison to the Netherlands: y-axis shows a 7-day moving average of the relative abundance of the six clades, and x-axis shows the collection date. (See the legend for clade names and colors) Intervals with fewer than one genome per day were discarded. See Fig. S3 for absolute number of genomes.

In order to get a broad overview of the viral diversity throughout the pandemic, we monitored changes in proportion of clades in time using the clade definitions proposed by the GISAID database^16^. We observe the distribution of different clades in the Netherlands resemble that of Australia where the first samples are genetically diverse and there is no dominating variant (subplots in Fig. 2, see Fig. S3 for absolute number of genomes). A similar pattern is seen in other European counties such as the UK and Belgium, while the USA, Canada and Denmark have distinct trajectories with GH clade dominating the population (Figs. S4 and S5). Also note that clade S has gradually faded out despite its high prevalence before April in several countries, this is particularly noticeable in Australia, China (Fig. 2), the USA, Spain and Canada (Figs. S4 and S5).

Viral diversity can be observed more clearly when put into context with less diverse populations in other countries where the outbreak had begun the earliest. For instance, China, Singapore and Italy had experienced the outbreak the earliest in the world, and there are only few of the major clades circulating (Fig. 2, Italy not shown due to small sample size, see Figs. S3 and S4 for other countries). China had opted for possibly the most severe restrictions; similarly in Singapore, the initial cases of COVID-19 had been followed up with strict precautions, preventing both the spread and new introduction of the virus. While it is tricky to formulate any clear hypothesis since there has not been any public data submission from these countries since April, it is certainly interesting to see the contrast between them and countries where COVID-19 arrived at relatively late stages of the pandemic, such as the Netherlands, the UK and Australia. However, more data is needed to form a better understanding of the population structures.

### Evolution of the SARS-CoV-2 genome and increased mutation frequency in hotspot regions

**Figure 3:**
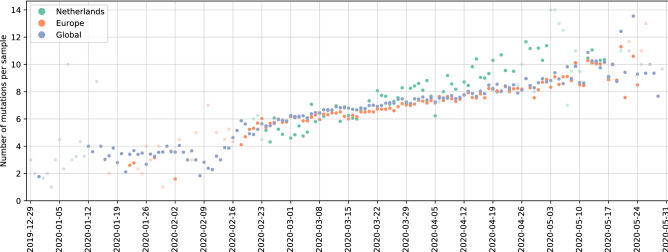
Number of mutations per sample per day over the course of pandemic in the Netherlands (green), Europe (orange) and globally (blue): each point is the average number of mutations observed in samples collected on the same date in the Netherlands (green), Europe (orange) and globally (blue), x-axis shows collection date. Data points corresponding to days with fewer than five samples are colored transparently to indicate uncertainty.

To assess the mutational landscape and its impact as the pandemic progressed, we investigated dominant mutations across time in the viral population. It is essential to monitor these changes in the SARS-CoV-2 genome to identify conserved sites relevant for designing therapeutics and vaccines, as well as to study the viral evolution during a pandemic. Currently, each new sample has on average around ten mutation sites in total compared to the Wuhan-Hu-1 reference (accession ID NC_045512.2) in the Netherlands where the trajectory has been in parallel with those in Europe and the world (Fig. 3 shows number of mutations per sample each day from December 2019 to June 2020). Clearly showing a divergence away from the original reference.

**Figure 4:**
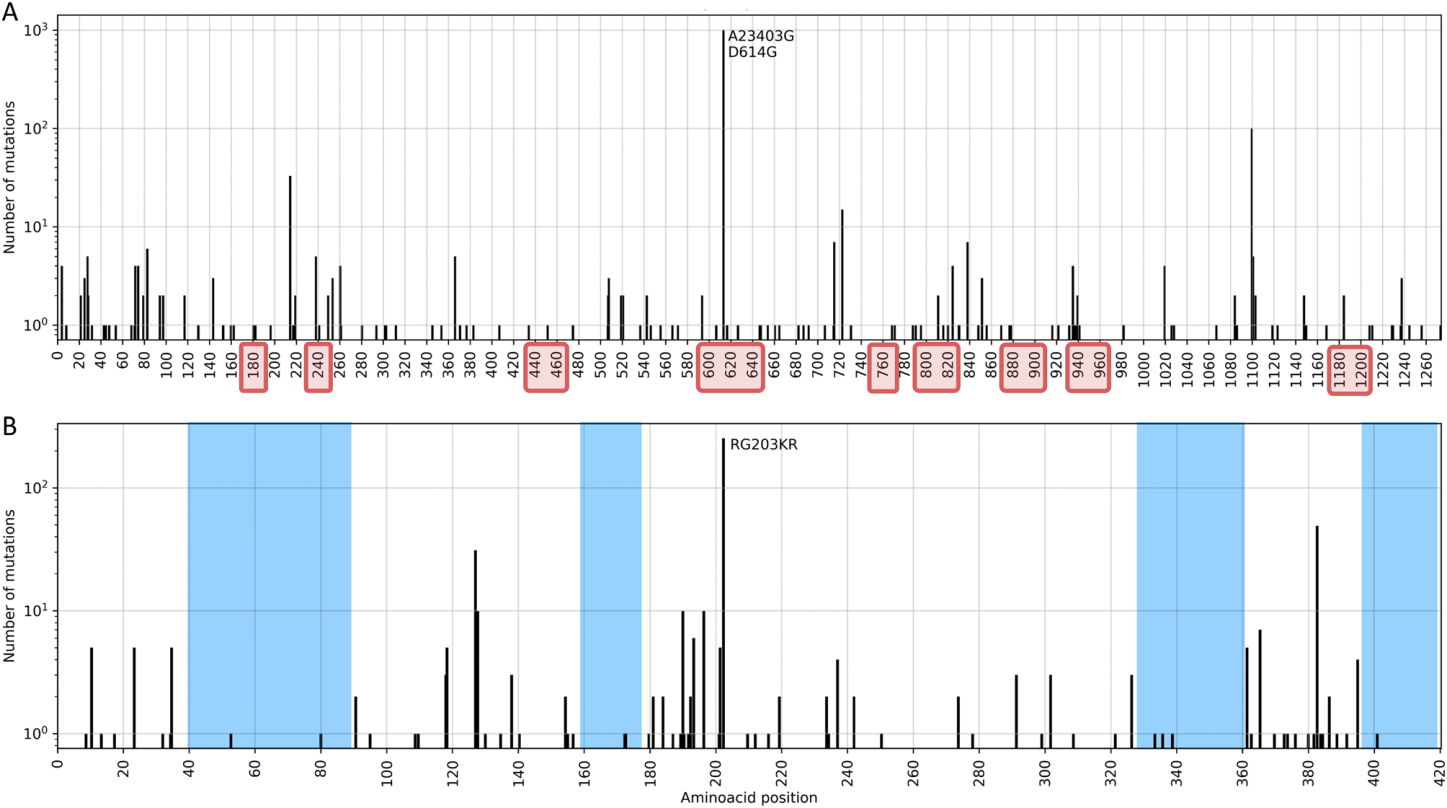
Total number of nucleotide mutations in the S (**A**) and N (**B**) proteins in samples from the Netherlands are displayed on y-axis; predicted epitope regions from^38^ are shown with red rectangles on x-axis, and conserved sites (free of mutations) on N protein are shaded in blue in (**B**). X-axis tick marks are labelled with the corresponding amino-acid position to complement the mutation annotations.

In particular, the S and N proteins have both been reported as the most variant proteins in the SARS-CoV-2 genome^22,33^. S:D614G and N:RG203KR amino acid changes comprise a large fraction of the mutation in these regions (Fig. 4); former being one of the mutation that defines G, GR and GH clades. Apart from carrying the majority of mutations observed in the populations, both proteins play an important role in RT-qPCR based diagnostic tests as well as vaccine and drug development^34^. The S protein has been investigated in great detail for its significance in binding to the host cell and a potential target for COVID-19 treatment and vaccine design^35–37^. In a recent study, prior information on the SARS-CoV S and N proteins, and their known epitopes were combined to identify regions in the SARS-CoV-2 genome that could potentially serve as epitopes for B-cells and T-cells^38^. In Fig. 4 we have highlighted the predicted epitope regions from^38^ with mutations using red rectangles on the x-axis. The authors confirmed that the most abundant mutations in these regions, S:D614G in particular, should be taken into account for vaccine design and development of treatments. We also note that the prevalence of S:D614G variant has steadily increased over the course of the pandemic: it is observed in all the sequences sampled recently in the world (Fig. S6).

In order to determine the appropriate primers to use when diagnosing patients with RT-PCR tests or when designing novel primer/probe sequences, variations in the nucleotide sequence should be considered since it plays a crucial role in achieving accurate tests^9,19^. We have identified mutations on target regions of primer/probe assay sets most used in the Netherlands. We found that assay sequences published by US CDC had fewer than ten genomes with mutations, and those from WHO had fewer than 18 out of 1338 genomes. We have also checked the assay sets from China CDC and Institut Pasteur, even though they are not in use in the Netherlands to our knowledge. We report 73 genomes (5%) with mutations on ORF1ab and 771 genomes (57%) with mutations on N protein for the sets released by China CDC, and fewer than six genomes for Institut Pasteur. Without being too specific, amino-acid positions from 40 to 90, 160–170, 330–360 and 400–420 on N protein appear to be relatively conserved sites, free of any mutations and could potentially be utilized as primer sequences (blue shaded regions in Fig. 4). The N protein is recommended as a screening assay by the WHO as well, and is utilized in many countries other than the Netherlands^28^. Further investigation of the location and frequency of mutations indicate the existence of conserved regions and show a general preference for non-silent changes in the genome (see “Supplemental Text”).

### Population of SARS-CoV-2 is dominated by four mutations globally while emergence of locally distinct variants indicates local outbreaks

**Figure 5:**
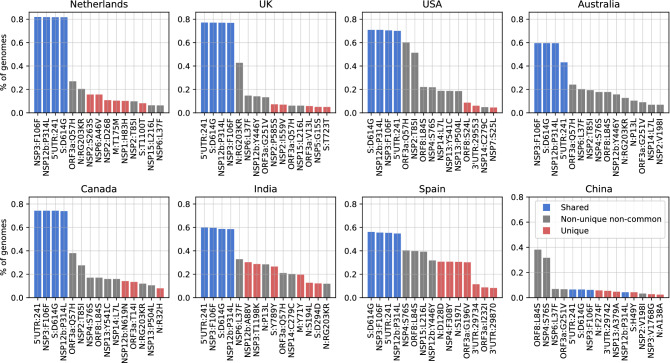
Total frequency (% of genomes) of the top 15 mutations in the most-sampled countries in our dataset: x-axes are the top 15 mutations and y-axes show the frequency (number of mutations per sample) of mutations. Blue bars are mutations shared across all these countries in the top 15, while the red bars are unique to that one country in the top 15 and the gray bars are non-unique and non-common variants where the mutation is observed in more than one country.

To study the global SARS-CoV-2 population and viral diversity in more detail, and observe the mutational landscape in the Netherlands within a global context, we have identified the most abundant mutations in our dataset. In addition to S:D614G and N:RG203KR, several other mutations, NSP12b:P314L, NSP3:F106F and 5′UTR:241 in particular, appear to dominate the most frequent mutations in the world; Fig. 5 shows the 15 most dominant SNPs in some of the most-sampled countries in our dataset. Due to over-representation of few European countries, it is difficult to comment on the geographical dominance of any mutations. However, four mutations, S:D614G, NSP12b:P314L, NSP3:F106F and 5′UTR:241 (blue bars in Fig. 5) are established within the global collection genomes, except for China where these mutations have very low frequencies.

While we observe a diverse mutational landscape in Australia, India and Spain, the viral population in China has remained relatively homogenous and with very few variants compared to the Wuhan-Hu-1 reference. The most frequent mutation is ORF8:L84S, which defines the S clade that appears to be fading out even though it had been circulating since the beginning of the pandemic along with the L clade. Recently, a possible link between two mutations, ORF8:L84S and NSP4:S76S, has been suggested, we also observed they co-occur several times outside of Europe; in China, the USA, Australia and Canada^39^. While keeping in mind that we do not have any sequences collected after April from China, we note a few region-specific mutations: first one being ORF8:L84S, which is more frequent in the USA and China and, second is NSP6:L37F which is frequent in in Australia and the USA.

**Figure 6:**
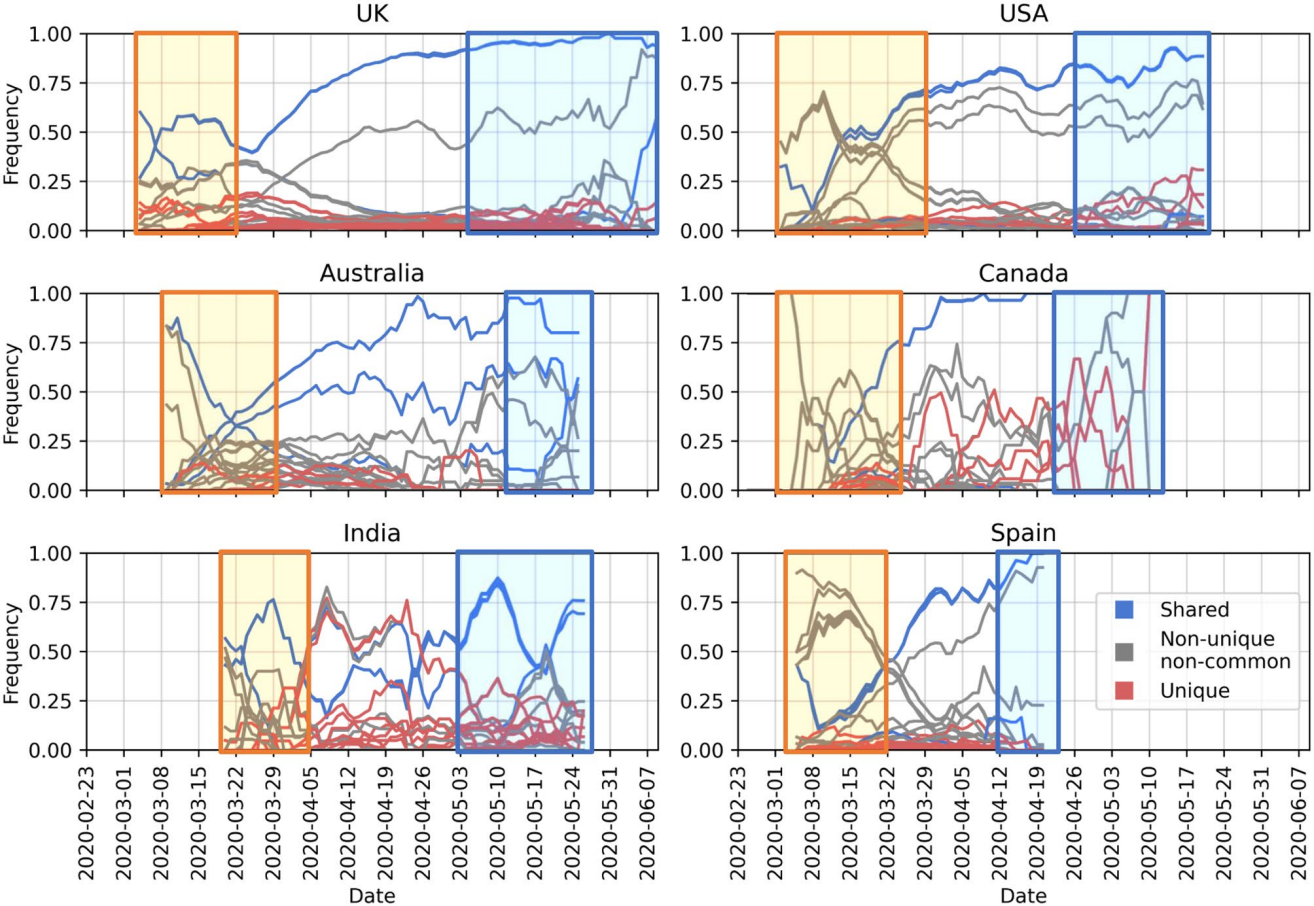
Change in frequency of the top 15 mutations in the most-sampled countries in our dataset: y-axes show mutation frequency (number of mutations per sample) averaged over a period of 7 days where periods with fewer than one sample per day were removed and x-axes show the collection date. Line colors were kept consistent with Fig. 5: blue lines are mutations shared across all these countries in the top 15, while the red lines are unique to that one country in the top 15 and the gray lines are non-unique and non-common variants where the mutation is observed in more than one country. Areas highlighted in yellow and blue as, mentioned in text, to indicate pre-lockdown and post-lockdown.

Considering the fluctuations in rate of sequencing, and over-representation of samples from the USA, the UK and Europe in general, it is difficult to comment on the geographical spread. Nevertheless, when we look into the frequency of the top four mutations, S:D614G, NSP12b:P314L, NSP3:F106F and 5′UTR:241 over the course of pandemic, we see a steady increase of their abundance in the viral population, regardless of the date of introduction in each country (Fig. 6).

A common pattern emerges in how shared and rarer mutations change in frequency in time: in the early phase of the pandemic, the viral population is diverse with relatively few mutations shared among all countries (areas highlighted in yellow in Fig. 6: first 2 weeks of March in the Netherlands, the UK, Australia, Canada and Spain^40–44^, also late March in the USA and India^45,46^). From mid-March to end of April when strict measures against travel were imposed universally, frequency of shared mutations increase more rapidly. As the pandemic progresses, the four most abundant mutations shared across each country (blue lines in Fig. 6) become well-established as part of the viral genome. In May, however, restrictions on domestic travel were slowly eased^47–51^, which we presume allowed for regional transmission, leading to again an increase in unique/rare mutations (areas highlighted in blue in Fig. 6) as they spread and form local clusters of variants. In addition, for most of the countries, number of sequences peaked in March or April, and has been on decline since then, except for India (Fig. S7). Hence it does not appear to be driving the changes in the frequency of rare/unique. Abundance of these unique variants suggests community-driven spread, which can be elaborated by monitoring such variants to detect super-spreading events.

**Figure 7:**
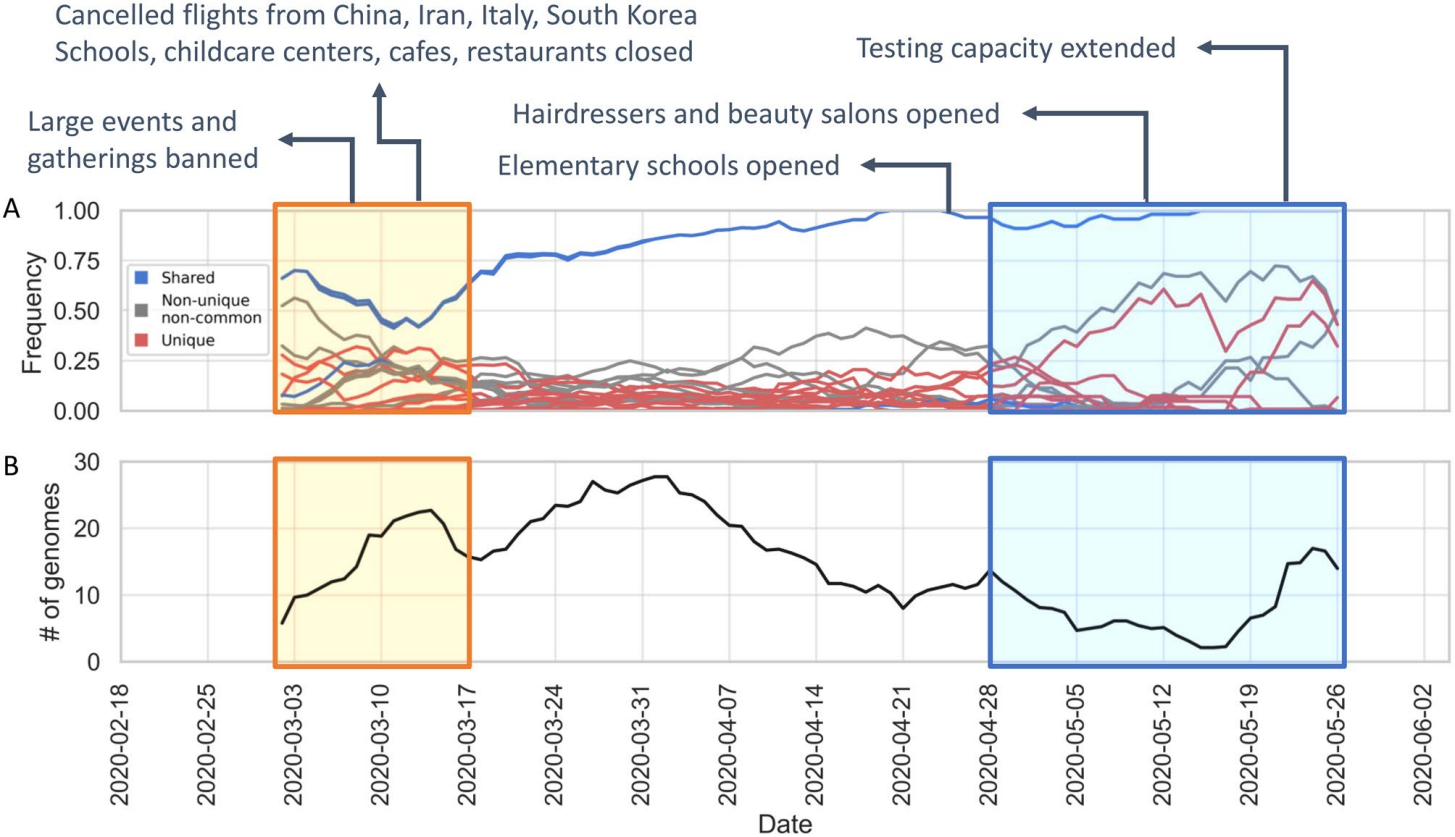
(**A**) Change in frequency of the top 15 mutations in the Netherlands, averaged over a period of 7 days and removed periods with less than one sample per day. Each line represents the abundance of a specific mutation over time. Line colors were kept consistent with Figs. 5 and 6: blue lines are mutations shared across all these countries in the top 15, while the red lines are unique to that one country in the top 15 and the gray lines are non-unique and non-common variants where the mutation is observed in more than one country. Areas highlighted in yellow and blue indicate pre-lockdown and post-lockdown respectively. Major milestones in the national response against COVID-19 are annotated at the top. (**B**) Number of submitted genome sequences in the Netherlands, averaged over a period of 7 days and removed periods with less dan one sample.

To assess the impact of lockdown attempts to control the pandemic on the viral diversity we investigated Dutch viral samples in detail. It is non-trivial to relate lockdown status to the viral population diversity across countries; while all measures to control COVID-19 have been reported throughout the pandemic, it is highly likely that there are both national and regional differences in their implementation as well as their impact on human behavior, especially in federal governments such as the USA, Australia and Canada, where regional governments play an influential role. For that reason, we focus on the Netherlands to understand this pattern better: we have placed the major milestones in national response against COVID-19 in the Netherlands along with the mutation frequencies below in Fig. 7A in comparison to number of sequences collected in Fig. 7B. We observe local/rare mutations to increase in frequency around the same time as restrictions are relaxed. One other explanation for the increase in frequency of rare mutations could be the gradual expansion of testing and sequencing capacity. Testing in the Netherlands was almost exclusively available to healthcare workers due to limited capacity until May^52^. It is conceivable that testing different groups of individuals has made it possible to collect more diverse samples of the virus.

### Introduction of COVID-19 in the Netherlands and local clusters with high genomic diversity

Next, we examined the Dutch phylogenetic tree to better understand the dynamics of COVID-19 in the Netherlands: from its introduction in the earliest samples to its further spread through localized infection clusters. We have identified multiple points of introduction in different provinces via highly diverse samples of virus. As the pandemic progresses, we see deeper branching in the tree with unique, localized mutations as well as similar patterns of evolution emerge in separate locations. While the virus population carries an increased number of mutations in general, these mutations are localized in their own clusters with little genomic diversity.

**Figure 8:**
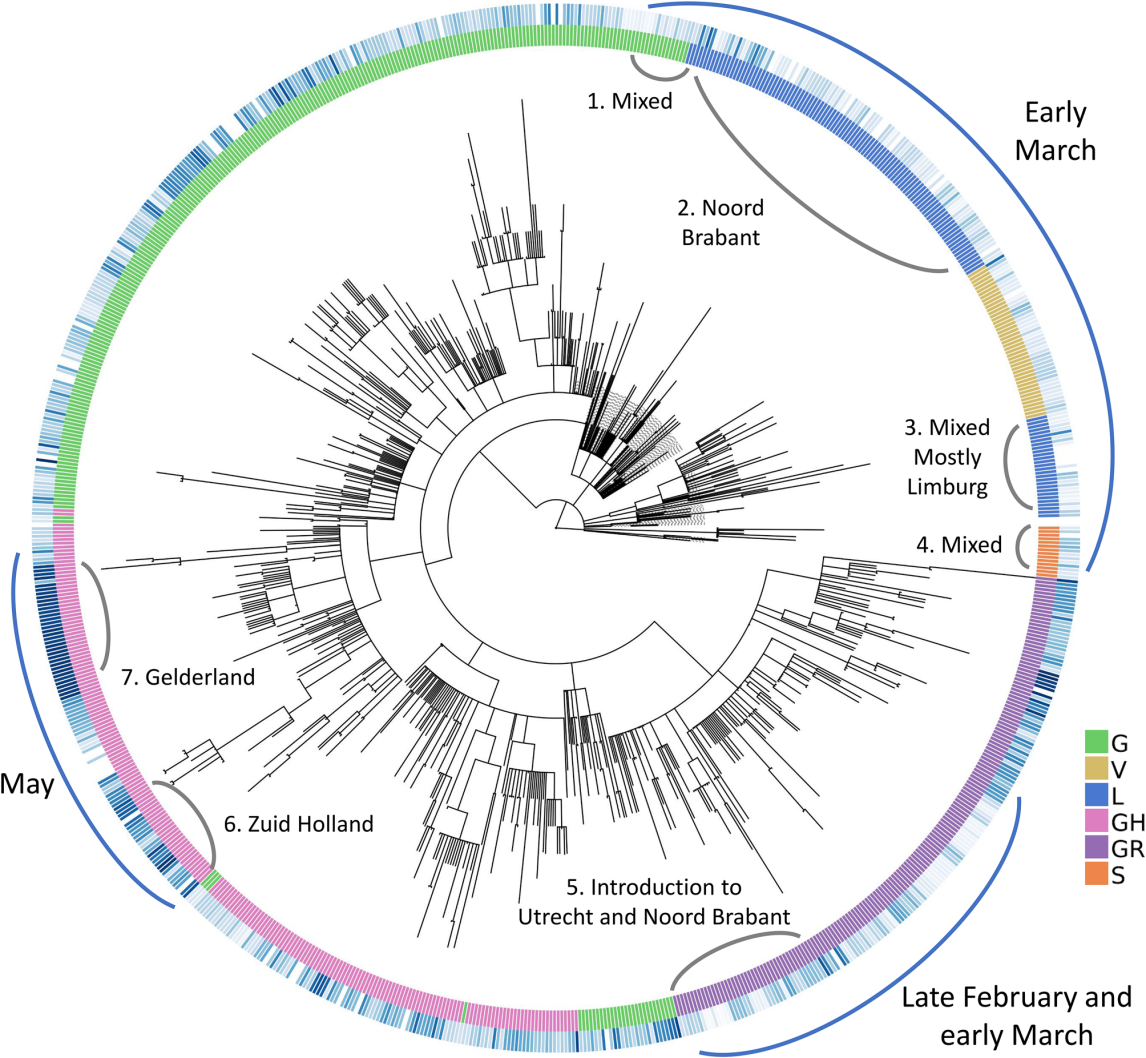
Radial representation of the Dutch phylogenetic tree: inner circle colored w.r.t. the assigned clades (see legend for clade names), outer circle is color-coded according to the sample collection date (if available), where the darker shade of blue represents more recent samples. Major points discussed in the text have been indicated with blue arches on the outer circle, along with more detailed information (numbered in clockwise direction) in gray arches on the inner circle.

We observe two separate sections on the radial tree in Fig. 8, representing the diversity of introduction to the Netherlands in terms of both the viral genome and location. First, at the top, starting from around 12 o’clock to 3 o’clock consists of some of the earliest samples from early March of V, L and S clades (denoted with a blue arch and text “Early March”). This is further broken down into four sections numbered from 1 to 4 where the second section is the early outbreak in Noord-Brabant in parallel with the first case reports^10^. However, the remaining sections are mixed in location and date as we encounter samples isolated from Limburg, Zuid-Holland, Gelderland and Utrecht, also from later into the pandemic in late March and early April.

The second point of introduction is from 4 to 6 o’clock on the tree, denoted as “late February and early March” with a blue arch. This section differs from the first one in that we observe only samples of G and GR clades, both of which are dominant in the Europe while absent in China. The earliest SARS-CoV-2 genome in our dataset with full sample collection date (accession ID EPI_IS_454750, collected on February 27) is also located in this section and it was first isolated in Utrecht (also see Supplementary file 4, rectangular Dutch tree annotated with GISAID clade assignments, collection dates and within-Netherlands location).

Recall the clade distribution over time in the Netherlands (Figs. 2 and 3) showed an initial phase of high diversity with L and GR dominating the dataset, also supported by the phylogenetic analysis. As part of the Dutch initiative to investigate transmission of COVID-19 in the Netherlands, Munnink et al. had conducted a detailed analysis on the earlier samples with patient data^10^. More recently, Sikkema et al. have published their findings on COVID-19 infection in health-care workers in early March^53^. Their studies suggest multiple introductions from Italy and Switzerland, as well as localized community transmissions in super-spreading events in late February and early March. We also note early samples from the Netherlands scattered among samples from outside the Netherlands, mostly Europe, collected around the same time in global phylogenetic tree (Supplementary file 5). In addition, the authors note the diversity of early strains even for patients with similar travel histories, also in parallel with our observations in our study. In addition to Noord-Brabant, Munnink et al. had detected local clusters in Zuid-Holland and Utrecht.

### Novel mutations appear in the later phase of pandemic

To explore local transmission clusters, we analyzed mutations that appeared after the initial pandemic response in the Netherlands. Munnink et al. have stated three phases of response to pandemic in the Netherlands in their study; (1) before the first case was reported, (2) from the first reported case to the start of screening of healthcare workers and (3) the period from the introduction of stricter measures along with events and large gatherings of people being banned until March 15th when the most strict phase of lockdown had begun as retail and catering industries were closed, as well as schools and childcare centers^40^. Since March 15th, the spread of COVID-19 has been very limited due to more stringent measures on travel and widely adopted practice of social distancing. For this reason, it is particularly interesting to investigate the deeper branching in Fig. 8 with later samples around 8–9 o’clock (denoted with a blue arch and the text “May”).

**Figure 9:**
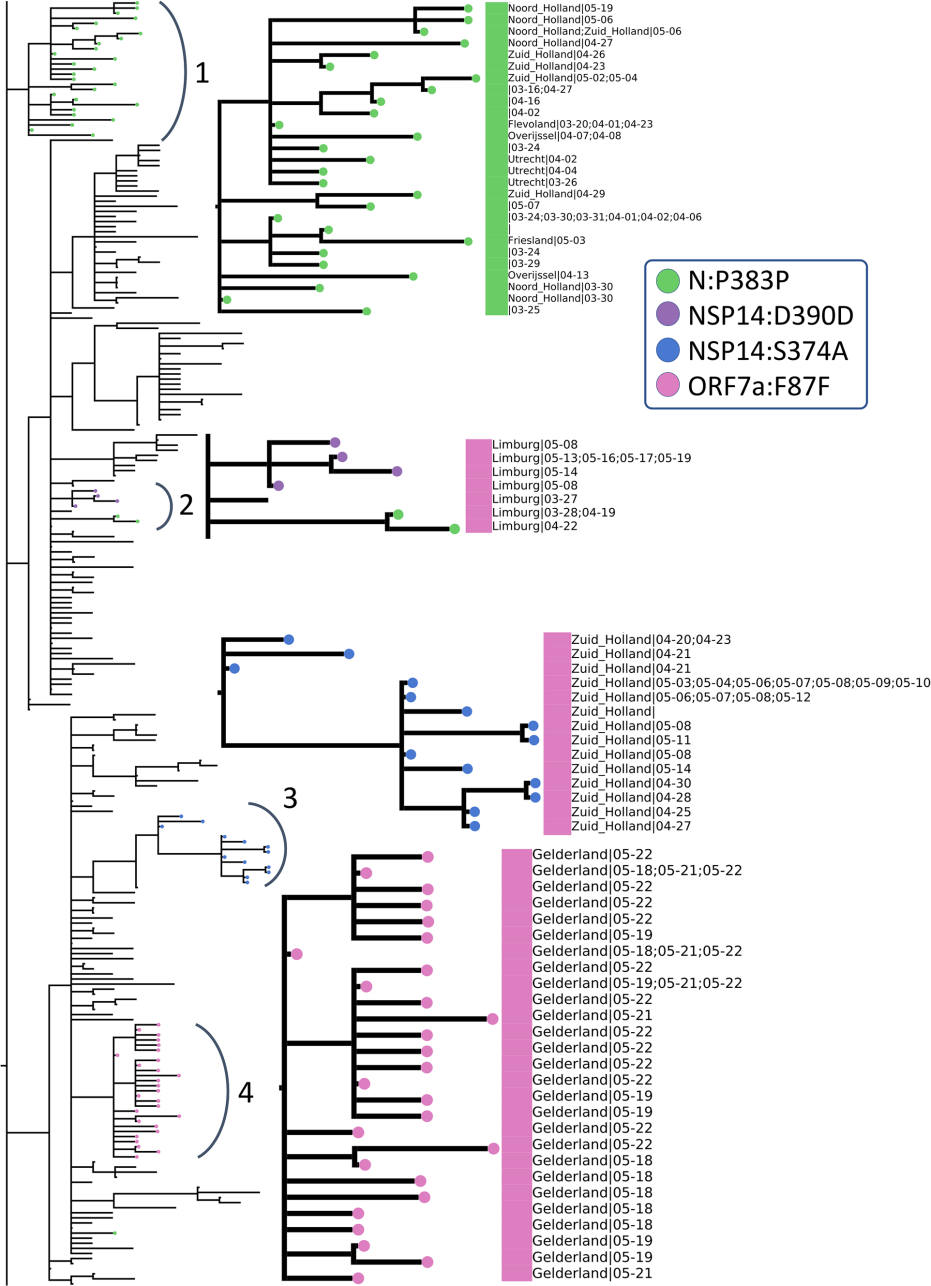
Zoomed-in view of rectangular representation of the Dutch phylogenetic tree: three regions of focus are numbered next to the corresponding arch. Newer, unique mutations that define deep branching in the tree are drawn in circles and the common mutations within Europe are rectangle (see legend for mutation annotations). Assigned clades are indicated with large rectangle aligned next to the leaves (pink: GH and green: G) and additional information about sequences (location and sequence collection date) are displayed next to the clade color, if available.

Below in Fig. 9, we have zoomed into the two “May” regions from Fig. 8 (numbered 3 and 4 in Fig. 9) as well as the remaining deep branches (numbered 1 and 2 in Fig. 9). To simplify, we have indicated the absence/presence of a mutation with a circle where the branch ends. Additional information about sample collection date and its location are also displayed aligned to the leaves, if available and in the case of duplicate sequences separated with a semicolon. Dates are expressed in format month-day. The large squares next to the leaf names are color-coded clade assignments, colors have been kept consistent throughout our study in Figs. 2, 8 and 9.

We have identified four mutations all of which have emerged after March 15th and have led to deeper branching on the phylogenetic tree and are either unique to the Netherlands or very rarely observed in the rest of the world: N:P383P, NSP14:D390D, NSP14:S374A and ORF7a:F87F. These rare mutations could be further utilized to track local transmissions of disease within the Netherlands.

N:P383P (green circles), a silent mutation on N protein is fairly unique to the Netherlands; it is present in less than five sequences in many European countries, including the most well-sampled ones Denmark and the UK, as well as the USA and Canada. Considering the sample size, it is surely intriguing that this mutation has been observed only in the Netherlands in such abundance. This mutation is also one of the oldest circulating ones since its first occurrence was in a sequence from the Switzerland on February 27th. However, we observe it for the first time in the Netherlands 2 weeks later on March 16th (province unknown). Later on, the same mutation has appeared in multiple provinces, Noord Holland, Zuid Holland, Flevoland, Utrecht and Limburg, in 50 sequences in total. Moreover, we observe it in two separate branching events in the phylogenetic tree in different provinces of the Netherlands; several provinces in arc number 1 and only in Limburg in arc number 3. In a recent study, this mutation had been detected as one of several homoplasies on the SARS-CoV-2 genome^54^. Since the Limburg branching contains only three sequences carrying the mutation, it is difficult to comment whether it is convergent or not. Given that branching with number 1 contains several provinces; it is also likely that this is a consequence of relaxations in domestic travel restrictions, rather than convergent evolution.

The second mutation, NSP14:D390D (purple circles), is tricky to interpret because it is present in only nine genomes, seven of which had been sequenced in the Netherlands and the remaining two in the UK. It has first appeared in the UK on March 24th, during strict lockdown conditions, and it has emerged in the Netherlands in May. We hypothesize this is a small cluster of variants genomes, localized in Limburg only and it has not found the chance to spread outside of the province yet.

NSP14:S374A (blue circles) is the only non-silent mutation in this list, and is very unique to Zuid Holland; it is present in 35 genomes in total, all collected in Zuid-Holland region within 3 weeks. Similar to NSP14:D390D, it is highly likely to be a small, contained cluster of individuals.

ORF7a:F87F (pink circles) is also incredibly rare since it was observed only in Gelderland in the Netherlands from late April to early May, and less than five times in any other country. It occurs in only one sequence from Canada in April 13th, twice in the USA in late March and four times in the UK in mid-April.

## Discussion

In this work, we retrieved 29,503 complete, high quality SARS-CoV-2 from publicly available databases to explore the viral population diversity In the Netherlands, within a global context. Considering the rapid increase in public data and research on this subject, our work is among the more comprehensive ones to lend insight into the genetic variation of SARS-CoV-2 in the later stages of the pandemic in April and early May.

As a consequence of the natural evolution of a virus, SARS-CoV-2 genome has been diverging from the initial reference sequence Wuhan-Hu-1 established based on viral samples from Wuhan, China. The six major clades designated by GISAID had varying distributions in different regions, at different points of time through the course of pandemic. We demonstrated that in most countries, viral population goes through an initial phase of high diversity followed by a decline in genetic variety in which the population is comprised of mostly G, GR or GH clades (Fig. 2). With increased ease of travel, COVID-19 was able to spread rapidly across the world and several studies had reported multiple introductions of a diverse viral population into many countries outside of China that lends itself to a more homogeneous population diverged from the Wuhan-Hu-1 reference^55–57^. Interesting, we have also observed that China and Singapore, both of which are countries that experienced the outbreak the earliest, harbor a markedly different viral population that remains mostly homogeneous with L being the dominant clade that also includes the Wuhan-Hu-1 reference (Fig. 2). Note that this could also be the artifact of the dramatic decline in number of sequences from China, where we do not have any sequence collected after April.

The S and N proteins in SARS-CoV-2 genome has received much attention; both have been reported as the most variant proteins^22,33^ and are also significant in RT-qPCR based diagnostic tests as well as vaccine and drug development^34^. We have identified the most variant sites on the S and N proteins in sequences from the Netherlands (Fig. 4). Koyama et al. had noted the effect of these variants on sequence-based vaccine and therapeutics against COVID-19^38^. Following their discussion, we highlight their predicted epitope regions derived from SARS and the mutations we detected on the S and N proteins in Fig. 4. In addition, Kim et al. discussed variations on SARS-CoV-2 genes targeted by diagnostic assays in^20^, and Vanaerschot et al. observed a mutation on N gene decrease the sensitivity of SARS-CoV-2 detection^58^. More recently, it was reported that a novel variant, first detected in the UK, denoted B.1.1.7, could lead to false negative results in diagnostic tests targeting the S gene^59^.

Similarly, we analyzed primer/probe sequences currently in use in the Netherlands for diagnostics targeting S and N genes (Fig. 4); we found a mutation on N protein, RG203KR (in 57% of the genomes) overlapping with the target region of China CDC diagnostic test. While there were no major mutations on target regions of tests released by US CDC, WHO or Institut Pasteur in our dataset, emerging variants should be monitored routinely to ensure the reliability of diagnostics. In our studies, we find amino-acid positions from 40 to 90, 160–170, 330–360 and 400–420 on N protein could potentially be utilized as targets (blue shaded regions in Fig. 4). Even though RT-qPCR tests contain primer/probe sets targeting multiple genes, according to the recent WHO guidelines, a single target could be used as well, particularly in areas where COVID-19 has spread widely. Hence, it is recommended that primer/probe binding sites are investigated for mismatches^60^.

When we observed the global landscape of variants, we found four mutations, S:D614G, N:RG203KR, NSP3:F106F and 5′UTR:241, are not only the most frequent ones, but also have been steadily increasing in the frequency outside of China since the beginning of pandemic. The 614G variant has been reported to exhibit increased transmissibility in human cells and animal models^61^, as well as phylodynamic studies^62^, although there are currently no known effects on the disease trajectory or clinical outcome^63^. Volz et al. also report two mutations, S:D614G and N:RG203KR, to be linked^62^. Some studies have suggested certain linked mutations which poses a different question on its own^63^. We also reported the increase in frequency of these shared mutations, regardless of the date of introduction (Fig. 6). On one hand, the abundance of these mutations might suggest that viral genome has converged to a new variant, different than the Wuhan-Hu-1 reference. On the other hand, since most of the viral sequences are from diagnostic tests performed on hospitalized patients at the moment, we are looking at only a small portion of the whole virus population in humans and we do not know clearly whether milder, or even asymptomatic cases of COVID-19 also carry these mutations or not. To our knowledge, studies have not found any significant correlation between these specific mutations and the COVID-19 disease in patients^63^. Nevertheless, it is surely interesting consider that these four mutations, linked to one another, might also influence the infection in the human host.

With our phylogenetic study in the Netherlands, we confirmed multiple introductions in distinct provinces as well as the population diversity in the initial samples. We found sequences collected from late February to early March in Noord Brabant, Limburg, Utrecht as well as Zuid Holland spread around the tree indicating genetically very diverse strains (Fig. 8). We also detected emerging local clusters, defined by four mutations, N:P383P, NSP14:D390D, NSP14:S374A and ORF7a:F87F, all of which are either entirely unique to the Netherlands or very rarely observed elsewhere (Fig. 9). N:P383P had occurred at two distinct sections in different regions, we presume this is likely a domestic travel event rather than a convergent mutation. We note the detection and monitoring of such unique mutations could be utilized for tracking the spread of virus and identifying possible routes of transmission during the outbreak. In addition, our findings are in line with previous studies in the Netherlands by Munnink et al. and Sikkema et al.; they had also observed sequence diversity in the earliest days of the outbreak as well as community transmission^10,53^.

The single most prominent pattern that we encountered in our study was that despite the continual increase in number of mutations in the genome, diverging away from the Wuhan-Hu-1 reference, there is little diversity in the new variants as we enter the later stages of the first wave of the pandemic. This suggests the current SARS-CoV-2 reference genome should be re-evaluated, perhaps replaced with a new one that represents the viral population more accurately. Further work is required to investigate implications of an inadequate reference in sequence-based analyses as well as develop alternative models. Having a good quality reference sequence is crucial in sequence-based analyses; we expect better read mapping and variant calling would improve phylogenetic studies and clade designations, and allow for reliable detection of transmission clusters and emerging variants. Improved variant detection would enable design of more accurate diagnostic assays. We assert this line of research will continue to supplement the global effort to fight COVID-19.

The major limitation of our study is the biased sampling of SARS-CoV-2 sequences. Despite our efforts to combine all genome sequences publicly available up to date, due to imbalanced sampling and dramatic changes in the frequency of genome sequencing, our dataset is over-represented by samples from the Europe and the USA and there are several gaps in time since the beginning of pandemic. In addition, most of the viral sequencing today is performed on hospitalized patients. These issues could be circumvented to some extend by stratified sampling or controlled sequencing efforts with random samples collected from individuals. Nevertheless, our findings are significant to understand the SARS-CoV-2 genome and both its national and global population diversity.

## Conclusions

In this study, we have analyzed 29,503 SARS-CoV-2 genomes retrieved from public databases to investigate genetic diversity in viral population as the pandemic progresses, with a focus on the Netherlands in particular. Our dataset contained 1338 genomes from the Netherlands, most of them sequenced in April and early May. We assert our work provides valuable information on the genetic diversity of SARS-CoV-2 and its local dynamics in the Netherlands for tracking the transmission of COVID-19, as well as localized, region-specific efforts in DNA-based therapeutic or vaccine development against COVID-19, and primer/probe design in RT-qPCR tests. Our work demonstrates the use of genomics in guiding diagnostics and outbreak investigation at a limited scale. In order to fully realize the potential of genomic epidemiology, we need routine sequencing of viral DNA established in parallel with COVID-19 testing. We emphasize the little diversity observed globally in recent samples despite the increased number of mutations relative to the established reference sequence, suggesting the current reference may not be representative of the population; potential implications of an inadequate reference on downstream analyses should be investigated.

## Supplementary Information

Supplementary Legends.

Supplementary Information 2.

Supplementary Information 3.

Supplementary Information 4.

Supplementary Information 5.

Supplementary Information 6.

## Data Availability

Full list of sequence identifiers, and the corresponding acknowledgements for the sequences used in this work are provided in Supplementary files 2 and 3.
