# Supplementary material for: Emergence of novel SARS-CoV-2 variants in the Netherlands
Source: Sci Rep. 2021 Mar 23;11:6625. doi: 10.1038/s41598-021-85363-7 (PMC7988010; doi:10.1038/s41598-021-85363-7)
Supplement: Supplementary file 1 — Supplementary Legends. [file 41598_2021_85363_MOESM1_ESM.docx]

Supplementary file 1:

Document contains Supplementary Text: Annotation of mutations further elucidate conserved regions and show a general preference of non-silent changes in the genome, and Supplementary Figures: Figure S3 to Figure S7.

Supplementary file 2:

Tab-separated file (tsv) which contains the acknowledgement table for the sequence records retrieved from the GISAID EpiFlu database.

Supplementary file 3:

Tab-separated file (tsv) which contains the list of sequence records retrieved from the NCBI and NGDC databases.

Supplementary file 4:

Phylogenetic tree constructed for the SARS-CoV-2 genomes collected in the Netherlands, annotated with GISAID clade assignments, collection dates and within-Netherlands location.

Supplementary file 5:

Global phylogenetic tree constructed for all SARS-CoV-2 genomes, annotated with GISAID clade assignments, collection dates and location.
