# Supplementary material for: Emergence of novel SARS-CoV-2 variants in the Netherlands
Source: Sci Rep. 2021 Mar 23;11:6625. doi: 10.1038/s41598-021-85363-7 (PMC7988010; doi:10.1038/s41598-021-85363-7)
Supplement: Supplementary file 3 — Supplementary Information 3. [file 41598_2021_85363_MOESM3_ESM.pdf]

Emergence of Novel SARS-CoV-2 Variants in the Netherlands - Supplementary File 2
Aysun Urhan1, Thomas Abeel1,2,*
1 Delft Bioinformatics Lab, Delft University of Technology Van Mourik Broekmanweg 6, 2628 XE Delft, The Netherlands
2 Infectious Disease and Microbiome Program, Broad Institute of MIT and Harvard, 415 Main Street, Cambridge, MA 02142, USA
* Corresponding author: t.abeel@tudelft.nl

We gratefully acknowledge the authors, originating and submitting laboratories of the sequences from GISAID's EpiFlu™ Database on which this research is based. The list is detailed below.
All submitters of data may be contacted directly via www.gisaid.org
Accession ID Virus name Location Collection date Originating lab Submitting lab Authors
EPI_ISL_406531 hCoV-19/Guangdong/20SF174/2020 Asia / China / Guangdong / Zhuhai 2020-01-22 Guangdong Provincial Center for Diseases Contorl and Prevention; Guangdong Provinical Public Health Guangdong Provincial Center for Disease Control and Prevention Min Kang, Jie Wu, Jing Lu, Tao Liu, Baisheng Li, Shujiang Mei, Feng Ruan, Lifeng Lin, Changwen Ke, Haojie Zhong, Yingtao Zhang, Lirong Zou, Xuguang Chen, Qi Zhu, Jianpeng Xiao, Jianxiang Geng, Zhe Liu, Jianxiong Hu, Weilin Zeng, Xing Li, Yuhuang Liao, Xiujuan Tang, Songjian Xiao, Ying Wang, Yingchao Song, Xue Zhuang, Lijun Liang, Guanhao He, Huihong Deng, Tie Song, Jianfeng He, Wenjun Ma
EPI_ISL_406533 hCoV-19/Guangzhou/20SF206/2020 Asia / China / Guangdong / Guangzhou 2020-01-22 Guangdong Provincial Center for Diseases Contorl and Prevention; Guangdong Provinical Public Health Guangdong Provincial Center for Diseases Control and Prevention Min Kang, Jie Wu, Jing Lu, Tao Liu, Baisheng Li, Shujiang Mei, Feng Ruan, Lifeng Lin, Changwen Ke, Haojie Zhong, Yingtao Zhang, Lirong Zou, Xuguang Chen, Qi Zhu, Jianpeng Xiao, Jianxiang Geng, Zhe Liu, Jianxiong Hu, Weilin Zeng, Xing Li, Yuhuang Liao, Xiujuan Tang, Songjian Xiao, Ying Wang, Yingchao Song, Xue Zhuang, Lijun Liang, Guanhao He, Huihong Deng, Tie Song, Jianfeng He, Wenjun Ma
EPI_ISL_406534 hCoV-19/Foshan/20SF207/2020 Asia / China / Guangdong / Foshan 2020-01-22 Guangdong Provincial Center for Diseases Control and Prevention; Guangdong Provincial Public Health Guangdong Provincial Center for Diseases Control and Prevention Min Kang, Jie Wu, Jing Lu, Tao Liu, Baisheng Li, Shujiang Mei, Feng Ruan, Lifeng Lin, Changwen Ke, Haojie Zhong, Yingtao Zhang, Lirong Zou, Xuguang Chen, Qi Zhu, Jianpeng Xiao, Jianxiang Geng, Zhe Liu, Jianxiong Hu, Weilin Zeng, Xing Li, Yuhuang Liao, Xiujuan Tang, Songjian Xiao, Ying Wang, Yingchao Song, Xue Zhuang, Lijun Liang, Guanhao He, Huihong Deng, Tie Song, Jianfeng He, Wenjun Ma
EPI_ISL_406535 hCoV-19/Foshan/20SF210/2020 Asia / China / Guangdong / Foshan 2020-01-22 Guangdong Provincial Center for Diseases Control and Prevention; Guangdong Provincial Public Health Guangdong Provincial Center for Diseases Control and Prevention Min Kang, Jie Wu, Jing Lu, Tao Liu, Baisheng Li, Shujiang Mei, Feng Ruan, Lifeng Lin, Changwen Ke, Haojie Zhong, Yingtao Zhang, Lirong Zou, Xuguang Chen, Qi Zhu, Jianpeng Xiao, Jianxiang Geng, Zhe Liu, Jianxiong Hu, Weilin Zeng, Xing Li, Yuhuang Liao, Xiujuan Tang, Songjian Xiao, Ying Wang, Yingchao Song, Xue Zhuang, Lijun Liang, Guanhao He, Huihong Deng, Tie Song, Jianfeng He, Wenjun Ma
EPI_ISL_406536 hCoV-19/Foshan/20SF211/2020 Asia / China / Guangdong / Foshan 2020-01-22 Guangdong Provincial Center for Diseases Control and Prevention; Guangdong Provincial Public Health Guangdong Provincial Center for Diseases Control and Prevention Min Kang, Jie Wu, Jing Lu, Tao Liu, Baisheng Li, Shujiang Mei, Feng Ruan, Lifeng Lin, Changwen Ke, Haojie Zhong, Yingtao Zhang, Lirong Zou, Xuguang Chen, Qi Zhu, Jianpeng Xiao, Jianxiang Geng, Zhe Liu, Jianxiong Hu, Weilin Zeng, Xing Li, Yuhuang Liao, Xiujuan Tang, Songjian Xiao, Ying Wang, Yingchao Song, Xue Zhuang, Lijun Liang, Guanhao He, Huihong Deng, Tie Song, Jianfeng He, Wenjun Ma
EPI_ISL_406538 hCoV-19/Guangdong/20SF201/2020 Asia / China / Guangdong 2020-01-23 Guangdong Provincial Center for Diseases Control and Prevention;Guangdong Provincial Institute of Public Health Guangdong Provincial Center for Diseases Control and Prevention Min Kang, Jie Wu, Jing Lu, Tao Liu, Baisheng Li, Shujiang Mei, Feng Ruan, Lifeng Lin, Changwen Ke, Haojie Zhong, Yingtao Zhang, Lirong Zou, Xuguang Chen, Qi Zhu, Jianpeng Xiao, Jianxiang Geng, Zhe Liu, Jianxiong Hu, Weilin Zeng, Xing Li, Yuhuang Liao, Xiujuan Tang, Songjian Xiao, Ying Wang, Yingchao Song, Xue Zhuang, Lijun Liang, Guanhao He, Huihong Deng, Tie Song, Jianfeng He, Wenjun Ma
EPI_ISL_413513 hCoV-19/South Korea/KUMC03/2020 Asia / South Korea 2020-02-27 Division of Infectious Diseases, Department of Internal Medicine, Korea University College of Medicine Department of Microbiology, Institute for Viral Diseases, College of Medicine, Korea University Changmin Kang, Joon-Yong Bae, Jungmin Lee, Jin Gu Yoon, Heedo Park, Juyoung Cho, Jeonghun Kim, Gee Eun Lee, Cui Chunguang, Kyeong-ryeol Shin, Ji Yun Noh, Joon Young Song, Hee Jin Cheong, Woo Joo Kim, Jin Il Kim, Man-Seong Park
EPI_ISL_413514 hCoV-19/South Korea/KUMC04/2020 Asia / South Korea 2020-02-27 Department of Microbiology, Institute for Viral Diseases, College of Medicine, Korea University Department of Microbiology, Institute for Viral Diseases, College of Medicine, Korea University Changmin Kang, Joon-Yong Bae, Jungmin Lee, Jin Gu Yoon, Heedo Park, Juyoung Cho, Jeonghun Kim, Gee Eun Lee, Cui Chunguang, Kyeong-ryeol Shin, Ji Yun Noh, Joon Young Song, Hee Jin Cheong, Woo Joo Kim, Jin Il Kim, Man-Seong Park
EPI_ISL_413515 hCoV-19/South Korea/KUMC05/2020 Asia / South Korea 2020-02-27 Division of Infectious Diseases, Department of Internal Medicine, Korea University College of Medicine Department of Microbiology, Institute for Viral Diseases, College of Medicine, Korea University Changmin Kang, Joon-Yong Bae, Jungmin Lee, Jin Gu Yoon, Heedo Park, Juyoung Cho, Jeonghun Kim, Gee Eun Lee, Cui Chunguang, Kyeong-ryeol Shin, Ji Yun Noh, Joon Young Song, Hee Jin Cheong, Woo Joo Kim, Jin Il Kim, Man-Seong Park

EPI_ISL_413516 hCoV-19/South Korea/KUMC06/2020 Asia / South Korea 2020-02-27 Department of Microbiology, Institute for Viral Diseases, College of Medicine, Korea University Department of Microbiology, Institute for Viral Diseases, College of Medicine, Korea University Changmin Kang, Joon-Yong Bae, Jungmin Lee, Jin Gu Yoon, Heedo Park, Juyoung Cho, Jeonghun Kim, Gee Eun Lee, Cui Chunguang, Kyeong-ryeol Shin, Ji Yun Noh, Joon Young Song, Hee Jin Cheong, Woo Joo Kim, Jin Il Kim, Man-Seong Park
EPI_ISL_413518 hCoV-19/Beijing/105/2020 Asia / China / Beijing 2020-01-26 unknown Infectious Disease Control Center Li,J., Li,L., Li,Z., Qiu,S., Song,H., Li,P. and Li,P.
EPI_ISL_413519 hCoV-19/Beijing/231/2020 Asia / China / Beijing 2020-01-28 unknown Infectious Disease Control Center Li,J., Li,L., Li,Z., Qiu,S., Song,H., Li,P. and Li,P.
EPI_ISL_413520 hCoV-19/Beijing/233/2020 Asia / China / Beijing 2020-01-28 unknown Infectious Disease Control Center Li,J., Li,L., Li,Z., Qiu,S., Song,H., Li,P. and Li,P.
EPI_ISL_413521 hCoV-19/Beijing/235/2020 Asia / China / Beijing 2020-01-28 unknown Infectious Disease Control Center Li,J., Li,L., Li,Z., Qiu,S., Song,H., Li,P. and Li,P.
EPI_ISL_413522 hCoV-19/India/1-27/2020 Asia / India / Kerala 2020-01-27 Indian Council of Medical Research - National Institute of Virology National Influenza Center, Indian Council of Medical Research - National Institute of Virology Potdar V, Yadav PD, Choudhary ML, Shete-Aich A
EPI_ISL_413523 hCoV-19/India/1-31/2020 Asia / India / Kerala 2020-01-31 Indian Council of Medical Research-National Institute of Virology National Influenza Center, Indian Council of Medical Research-National Institute of Virology Potdar V, Yadav PD, Choudhary ML, Shete-Aich A
EPI_ISL_413555 hCoV-19/Wales/PHW1/2020 Europe / United Kingdom / Wales 2020-02-27 Wales Specialist Virology Centre Public Health Wales Microbiology Cardiff Catherine Moore, Cen Sabu, Joanne Watkins, Sally Corden, Tom Connor
EPI_ISL_413556 hCoV-19/Wales/PHW2/2020 Europe / United Kingdom / Wales 2020-03-04 Wales Specialist Virology Centre Public Health Wales Microbiology Cardiff Catherine Moore, Tim Jones, Joanne Watkins, Sally Corden, Tom Connor
EPI_ISL_413557 hCoV-19/USA/CA-CDPH-UC1/2020 North America / USA / California / Sonoma County 2020-02-28 California Department of Public Health Chiu Laboratory, University of California, San Francisco Xianding Deng, Scot Federman, Chao-Yang Pan, Hugo Guevara,Wei Gu, Debra A. Wadford, and Charles Y. Chiu
EPI_ISL_413558 hCoV-19/USA/CA-CDPH-UC2/2020 North America / USA / California / Solano County 2020-02-27 California Department of Public Health Chiu Laboratory, University of California, San Francisco Xianding Deng, Scot Federman, Chao-Yang Pan, Hugo Guevara,Wei Gu, Debra A. Wadford, and Charles Y. Chiu
EPI_ISL_413559 hCoV-19/USA/CA-CDPH-UC3/2020 North America / USA / California / Solano County 2020-02-27 California Department of Public Health Chiu Laboratory, University of California, San Francisco Xianding Deng, Scot Federman, Chao-Yang Pan, Hugo Guevara,Wei Gu, Debra A. Wadford, and Charles Y. Chiu
EPI_ISL_413560 hCoV-19/USA/WA-S3/2020 North America / USA / Washington 2020-02-28 Seattle Flu Study Seattle Flu Study Chu et al
EPI_ISL_413561 hCoV-19/USA/CA-CDPH-UC4/2020 North America / USA / California / Solano County 2020-02-27 California Department of Public Health Chiu Laboratory, University of California, San Francisco Xianding Deng, Scot Federman, Chao-Yang Pan, Hugo Guevara,Wei Gu, Debra A. Wadford, and Charles Y. Chiu
EPI_ISL_413562 hCoV-19/USA/WA11-UW7/2020 North America / USA / Washington 2020-03-02 UW Virology Lab UW Virology Lab Pavitra Roychoudhury, Hong Xie, Keith Jerome, Alexander Greninger
EPI_ISL_413563 hCoV-19/USA/WA12-UW8/2020 North America / USA / Washington 2020-03-03 UW Virology Lab UW Virology Lab Pavitra Roychoudhury, Hong Xie, Keith Jerome, Alexander Greninger
EPI_ISL_413564 hCoV-19/Netherlands/Andel_1365066/2020 Europe / Netherlands / Andel 2020-03-01 MHC West-Brabant Erasmus Medical Center David Nieuwenhuijse, Bas Oude Munnink, Reina Sikkema, Claudia Schapendonk, Irina Chestakova, Anne van der Linden, Mark Pronk, Pascal Lexmond, Corien Swaan, Manon Haverkate, Madelief Mollers, Mart Stein, Sandra Kengne Kamga Mobou, Jeroen van Kampen, Jolanda Voermans, Aura Timen, Corine GeurtsvanKessel, Annemiek van der Eijk, Richard Molenkamp, Marion Koopmans, on behalf of the Dutch national COVID-19 response team.
EPI_ISL_413566 hCoV-19/Netherlands/Blaricum_1364780/2020 Europe / Netherlands / Blaricum 2020-03-02 MHC Gooi & Vechtstreek Erasmus Medical Center David Nieuwenhuijse, Bas Oude Munnink, Reina Sikkema, Claudia Schapendonk, Irina Chestakova, Anne van der Linden, Mark Pronk, Pascal Lexmond, Corien Swaan, Manon Haverkate, Madelief Mollers, Mart Stein, Sandra Kengne Kamga Mobou, Jeroen van Kampen, Jolanda Voermans, Aura Timen, Corine GeurtsvanKessel, Annemiek van der Eijk, Richard Molenkamp, Marion Koopmans, on behalf of the Dutch national COVID-19 response team.
EPI_ISL_413568 hCoV-19/Netherlands/Dalen_1363624/2020 Europe / Netherlands / Dalen 2020-03-01 MHC Drente Erasmus Medical Center David Nieuwenhuijse, Bas Oude Munnink, Reina Sikkema, Claudia Schapendonk, Irina Chestakova, Anne van der Linden, Mark Pronk, Pascal Lexmond, Corien Swaan, Manon Haverkate, Madelief Mollers, Mart Stein, Sandra Kengne Kamga Mobou, Jeroen van Kampen, Jolanda Voermans, Aura Timen, Corine GeurtsvanKessel, Annemiek van der Eijk, Richard Molenkamp, Marion Koopmans, on behalf of the Dutch national COVID-19 response team.
EPI_ISL_413571 hCoV-19/Netherlands/Eindhoven_1363782/2020 Europe / Netherlands / Eindhoven 2020-03-02 MHC Brabant Zuidoost Erasmus Medical Center David Nieuwenhuijse, Bas Oude Munnink, Reina Sikkema, Claudia Schapendonk, Irina Chestakova, Anne van der Linden, Mark Pronk, Pascal Lexmond, Corien Swaan, Manon Haverkate, Madelief Mollers, Mart Stein, Sandra Kengne Kamga Mobou, Jeroen van Kampen, Jolanda Voermans, Aura Timen, Corine GeurtsvanKessel, Annemiek van der Eijk, Richard Molenkamp, Marion Koopmans, on behalf of the Dutch national COVID-19 response team.
EPI_ISL_413572 hCoV-19/Netherlands/Haarlem_1363688/2020 Europe / Netherlands / Haarlem 2020-03-01 MHC Kennemerland Erasmus Medical Center David Nieuwenhuijse, Bas Oude Munnink, Reina Sikkema, Claudia Schapendonk, Irina Chestakova, Anne van der Linden, Mark Pronk, Pascal Lexmond, Corien Swaan, Manon Haverkate,

Madelief Mollers, Mart Stein, Sandra Kengne Kamga Mobou, Jeroen van Kampen, Jolanda Voermans, Aura Timen, Corine GeurtsvanKessel, Annemiek van der Eijk, Richard Molenkamp, Marion Koopmans, on behalf of the Dutch national COVID-19 response team.
EPI_ISL_413573 hCoV-19/Netherlands/Hardinxveld_Giessendam_1364806/2020 Europe / Netherlands / Hardinxveld Giessendam 2020-03-02 Dienst Gezondheid & Jeugd Zuid-Holland Zuid Erasmus Medical Center David Nieuwenhuijse, Bas Oude Munnink, Reina Sikkema, Claudia Schapendonk, Irina Chestakova, Anne van der Linden, Mark Pronk, Pascal Lexmond, Corien Swaan, Manon Haverkate, Madelief Mollers, Mart Stein, Sandra Kengne Kamga Mobou, Jeroen van Kampen, Jolanda Voermans, Aura Timen, Corine GeurtsvanKessel, Annemiek van der Eijk, Richard Molenkamp, Marion Koopmans, on behalf of the Dutch national COVID-19 response team.
EPI_ISL_413574 hCoV-19/Netherlands/Helmond_1363548/2020 Europe / Netherlands / Helmond 2020-02-29 MHC West-Brabant Erasmus Medical Center David Nieuwenhuijse, Bas Oude Munnink, Reina Sikkema, Claudia Schapendonk, Irina Chestakova, Anne van der Linden, Mark Pronk, Pascal Lexmond, Corien Swaan, Manon Haverkate, Madelief Mollers, Mart Stein, Sandra Kengne Kamga Mobou, Jeroen van Kampen, Jolanda Voermans, Aura Timen, Corine GeurtsvanKessel, Annemiek van der Eijk, Richard Molenkamp, Marion Koopmans, on behalf of the Dutch national COVID-19 response team.
EPI_ISL_413577 hCoV-19/Netherlands/Naarden_1364774/2020 Europe / Netherlands / Naarden 2020-03-02 MHC Gooi & Vechtstreek Erasmus Medical Center David Nieuwenhuijse, Bas Oude Munnink, Reina Sikkema, Claudia Schapendonk, Irina Chestakova, Anne van der Linden, Mark Pronk, Pascal Lexmond, Corien Swaan, Manon Haverkate, Madelief Mollers, Mart Stein, Sandra Kengne Kamga Mobou, Jeroen van Kampen, Jolanda Voermans, Aura Timen, Corine GeurtsvanKessel, Annemiek van der Eijk, Richard Molenkamp, Marion Koopmans, on behalf of the Dutch national COVID-19 response team.
EPI_ISL_413579 hCoV-19/Netherlands/Nootdorp_1364222/2020 Europe / Netherlands / Nootdorp 2020-03-03 MHC Haaglanden Erasmus Medical Center David Nieuwenhuijse, Bas Oude Munnink, Reina Sikkema, Claudia Schapendonk, Irina Chestakova, Anne van der Linden, Mark Pronk, Pascal Lexmond, Corien Swaan, Manon Haverkate, Madelief Mollers, Mart Stein, Sandra Kengne Kamga Mobou, Jeroen van Kampen, Jolanda Voermans, Aura Timen, Corine GeurtsvanKessel, Annemiek van der Eijk, Richard Molenkamp, Marion Koopmans, on behalf of the Dutch national COVID-19 response team.
EPI_ISL_413580 hCoV-19/Netherlands/Oisterwijk_1364072/2020 Europe / Netherlands / Oisterwijk 2020-03-02 MHC Hart voor Brabant Erasmus Medical Center David Nieuwenhuijse, Bas Oude Munnink, Reina Sikkema, Claudia Schapendonk, Irina Chestakova, Anne van der Linden, Mark Pronk, Pascal Lexmond, Corien Swaan, Manon Haverkate, Madelief Mollers, Mart Stein, Sandra Kengne Kamga Mobou, Jeroen van Kampen, Jolanda Voermans, Aura Timen, Corine GeurtsvanKessel, Annemiek van der Eijk, Richard Molenkamp, Marion Koopmans, on behalf of the Dutch national COVID-19 response team.
EPI_ISL_413582 hCoV-19/Netherlands/Rotterdam_1363790/2020 Europe / Netherlands / Rotterdam 2020-03-01 ErasmusMC Erasmus Medical Center David Nieuwenhuijse, Bas Oude Munnink, Reina Sikkema, Claudia Schapendonk, Irina Chestakova, Anne van der Linden, Mark Pronk, Pascal Lexmond, Corien Swaan, Manon Haverkate, Madelief Mollers, Mart Stein, Sandra Kengne Kamga Mobou, Jeroen van Kampen, Jolanda Voermans, Aura Timen, Corine GeurtsvanKessel, Annemiek van der Eijk, Richard Molenkamp, Marion Koopmans, on behalf of the Dutch national COVID-19 response team.
EPI_ISL_413583 hCoV-19/Netherlands/Rotterdam_1364040/2020 Europe / Netherlands / Rotterdam 2020-03-02 MHC Rotterdam-Rijnmond Erasmus Medical Center David Nieuwenhuijse, Bas Oude Munnink, Reina Sikkema, Claudia Schapendonk, Irina Chestakova, Anne van der Linden, Mark Pronk, Pascal Lexmond, Corien Swaan, Manon Haverkate, Madelief Mollers, Mart Stein, Sandra Kengne Kamga Mobou, Jeroen van Kampen, Jolanda Voermans, Aura Timen, Corine GeurtsvanKessel, Annemiek van der Eijk, Richard Molenkamp, Marion Koopmans, on behalf of the Dutch national COVID-19 response team.
EPI_ISL_413584 hCoV-19/Netherlands/Rotterdam_1364740/2020 Europe / Netherlands / Rotterdam 2020-03-03 unknown Erasmus Medical Center David Nieuwenhuijse, Bas Oude Munnink, Reina Sikkema, Claudia Schapendonk, Irina Chestakova, Anne van der Linden, Mark Pronk, Pascal Lexmond, Corien Swaan, Manon Haverkate, Madelief Mollers, Mart Stein, Sandra Kengne Kamga Mobou, Jeroen van Kampen, Jolanda Voermans, Aura Timen, Corine GeurtsvanKessel, Annemiek van der Eijk, Richard Molenkamp, Marion Koopmans, on behalf of the Dutch national COVID-19 response team.
EPI_ISL_413587 hCoV-19/Netherlands/Tilburg_1364286/2020 Europe / Netherlands / Tilburg 2020-03-03 Foundation Elisabeth-Tweesteden Ziekenhuis Erasmus Medical Center David Nieuwenhuijse, Bas Oude Munnink, Reina Sikkema, Claudia Schapendonk, Irina Chestakova, Anne van der Linden, Mark Pronk, Pascal Lexmond, Corien Swaan, Manon Haverkate, Madelief Mollers, Mart Stein, Sandra Kengne Kamga Mobou, Jeroen van Kampen, Jolanda Voermans, Aura Timen, Corine GeurtsvanKessel, Annemiek van der Eijk, Richard Molenkamp, Marion Koopmans, on behalf of the Dutch national COVID-19 response team.
EPI_ISL_413588 hCoV-19/Netherlands/Utrecht_1363564/2020 Europe / Netherlands / Utrecht 2020-03-01 MHC Utrecht Erasmus Medical Center David Nieuwenhuijse, Bas Oude Munnink, Reina Sikkema, Claudia Schapendonk, Irina Chestakova, Anne van der Linden, Mark Pronk, Pascal Lexmond, Corien Swaan, Manon Haverkate, Madelief Mollers, Mart Stein, Sandra Kengne Kamga Mobou, Jeroen van Kampen, Jolanda Voermans, Aura Timen, Corine GeurtsvanKessel, Annemiek van der Eijk, Richard Molenkamp, Marion Koopmans, on behalf of the Dutch national COVID-19 response team.
EPI_ISL_413589 hCoV-19/Netherlands/Utrecht_1363628/2020 Europe / Netherlands / Utrecht 2020-03-01 MHC Utrecht Erasmus Medical Center David Nieuwenhuijse, Bas Oude Munnink, Reina Sikkema, Claudia Schapendonk, Irina Chestakova, Anne van der Linden, Mark Pronk, Pascal Lexmond, Corien Swaan, Manon Haverkate, Madelief Mollers, Mart Stein, Sandra Kengne Kamga Mobou, Jeroen van Kampen, Jolanda Voermans, Aura Timen, Corine GeurtsvanKessel, Annemiek van der Eijk, Richard Molenkamp, Marion Koopmans, on behalf of the Dutch national COVID-19 response team.
EPI_ISL_413590 hCoV-19/Netherlands/Utrecht_1364066/2020 Europe / Netherlands / Utrecht 2020-03-02

MHC Utrecht	Erasmus Medical Center	David Nieuwenhuijse, Bas Oude Munnink, Reina Sikkema, Claudia Schapendonk, Irina Chestakova, Anne van der Linden, Mark Pronk, Pascal Lexmond, Corien Swaan, Manon Haverkate, Madelief Mollers, Mart Stein, Sandra Kengne Kamga Mobou, Jeroen van Kampen, Jolanda Voermans, Aura Timen, Corine GeurtsvanKessel, Annemiek van der Eijk, Richard Molenkamp, Marion Koopmans, on behalf of the Dutch national COVID-19 response team.
EPI_ISL_413591	hCoV-19/Netherlands/Zeewolde_1365080/2020	Europe / Netherlands / Zeewolde 2020-03-02	MHC Flevoland	Erasmus Medical Center	David Nieuwenhuijse, Bas Oude Munnink, Reina Sikkema, Claudia Schapendonk, Irina Chestakova, Anne van der Linden, Mark Pronk, Pascal Lexmond, Corien Swaan, Manon Haverkate, Madelief Mollers, Mart Stein, Sandra Kengne Kamga Mobou, Jeroen van Kampen, Jolanda Voermans, Aura Timen, Corine GeurtsvanKessel, Annemiek van der Eijk, Richard Molenkamp, Marion Koopmans, on behalf of the Dutch national COVID-19 response team.
EPI_ISL_413592	hCoV-19/Taiwan/NTU03/2020	Asia / Taiwan / Taipei	2020-03-02	Department of Laboratory Medicine, National Taiwan University Hospital	Microbial Genomics Core Lab, National Taiwan University Centers of Genomic and Precision Medicine	Shiou-Hwei Yeh, You-Yu Lin, Ya-Yun Lai, Chiao-Ling Li, Shan-Chwen Chang, Pei-Jer Chen, Sui-Yuan Chang
EPI_ISL_413593	hCoV-19/Luxembourg/Lux1/2020	Europe / Luxembourg	2020-02-29	Laboratoire National de Santé	Erasmus Medical Center	David Nieuwenhuijse, Bas Oude Munnink, Reina Sikkema, Claudia Schapendonk, Irina Chestakova, Anne van der Linden, Mark Pronk, Pascal Lexmond, T. Abdelrahman, G. Fournier, J. Mossong, T. Nguyen, Jeroen van Kampen, Jolanda Voermans, Corine GeurtsvanKessel, Annemiek van der Eijk, Richard Molenkamp, Marion Koopmans, on behalf of the Dutch national COVID-19 response team.
EPI_ISL_413594	hCoV-19/Australia/NSW08/2020	Oceania / Australia / NSW / Sydney	2020-02-28	Centre for Infectious Diseases and Microbiology Laboratory Services	NSW Health Pathology - Institute of Clinical Pathology and Medical Research; Westmead Hospital; University of Sydney	Rockett R, Eden J-S, Lam C, Gray K, Timms, V, Gall, M, Alicia, A, Carter I, Rahman H, Holmes EC, , O'Sullivan MV, Sintchenko V, Chen SC, Maddocks S, Kok J and Dwyer DE for the 2019-nCoV Study Group*
EPI_ISL_413595	hCoV-19/Australia/NSW09/2020	Oceania / Australia / NSW / Sydney	2020-02-28	Centre for Infectious Diseases and Microbiology Laboratory Services	NSW Health Pathology - Institute of Clinical Pathology and Medical Research; Westmead Hospital; University of Sydney	Rockett R, Eden J-S, Lam C, Gray K, Timms, V, Gall, M, Carter I, Rahman H, Holmes EC, O'Sullivan MV, Sintchenko V, Chen SC, Maddocks S, Kok J and Dwyer DE for the 2019-nCoV Study Group*
EPI_ISL_413596	hCoV-19/Australia/NSW10/2020	Oceania / Australia / NSW / Sydney	2020-02-28	Centre for Infectious Diseases and Microbiology - Public Health	NSW Health Pathology - Institute of Clinical Pathology and Medical Research; Westmead Hospital; University of Sydney	Rockett R, Eden J-S, Lam C, Gray K, Timms, V, Gall, M, Carter I, Rahman H, Holmes EC, O'Sullivan MV, Sintchenko V, Chen SC, Maddocks S, Kok J and Dwyer DE for the 2019-nCoV Study Group*
EPI_ISL_413597	hCoV-19/Australia/NSW11/2020	Oceania / Australia / NSW / Sydney	2020-03-02	Centre for Infectious Diseases and Microbiology- Public Health	NSW Health Pathology - Institute of Clinical Pathology and Medical Research; Westmead Hospital; University of Sydney	Lam C, Eden J-S, Rockett R, Gray K, Timms, V, Gall, M, Carter I, Rahman H, Holmes EC, O'Sullivan MV, Sintchenko V, Chen SC, Maddocks S, Kok J and Dwyer DE for the 2019-nCoV Study Group*
EPI_ISL_413598	hCoV-19/Australia/NSW12/2020	Oceania / Australia / NSW / Sydney	2020-03-04	Centre for Infectious Diseases and Microbiology - Public Health	NSW Health Pathology - Institute of Clinical Pathology and Medical Research; Westmead Hospital; University of Sydney	Gray K, Eden J-S, Lam C, Rockett R, Timms, V, Gall, M, Carter I, Rahman H, Holmes EC, O'Sullivan MV, Sintchenko V, Chen SC, Maddocks S, Kok J and Dwyer DE for the 2019-nCoV Study Group*
EPI_ISL_413599	hCoV-19/Australia/NSW13/2020	Oceania / Australia / NSW / Sydney	2020-03-04	Centre for Infectious Diseases and Microbiology - Public Health	NSW Health Pathology - Institute of Clinical Pathology and Medical Research; Westmead Hospital; University of Sydney	Timms, V, Eden J-S, Lam C, Gray K, Rockett R, Gall, M, Carter I, Rahman H, Holmes EC, O'Sullivan MV, Sintchenko V, Chen SC, Maddocks S, Kok J and Dwyer DE for the 2019-nCoV Study Group*
EPI_ISL_413600	hCoV-19/Australia/NSW14/2020	Oceania / Australia / NSW / Sydney	2020-03-03	Centre for Infectious Diseases and Microbiology - Public Health	NSW Health Pathology - Institute of Clinical Pathology and Medical Research; Westmead Hospital; University of Sydney	Gall, M, Eden J-S, Lam C, Gray K, Timms, V, Rockett R, Carter I, Rahman H, Holmes EC, O'Sullivan MV, Sintchenko V, Chen SC, Maddocks S, Kok J and Dwyer DE for the 2019-nCoV Study Group*
EPI_ISL_413601	hCoV-19/USA/WA13-UW9/2020	North America / USA / Washington	2020-03-02	UW Virology Lab	UW Virology Lab	Pavitra Roychoudhury, Hong Xie, Keith Jerome, Alexander Greninger
EPI_ISL_413711	hCoV-19/China/WF0014/2020	Asia / China	2020-02 Weifang Center for Disease Control and Prevention	Weifang Center for Disease Control and Prevention & BGI-Shenzhen	Qing Nie, Xingguang Li, Erik M Volz, Han Fu, Haowei Wang, Xiaoyue Xi, Wei Chen, Dehui Liu, Yingying Chen, Mengmeng Tian, Wei Tan, Junjie Zai, Wanying Sun, Jiandong Li, Junhua Li
EPI_ISL_417091	hCoV-19/USA/WA-S38/2020 North America / USA / Washington / Snohomish County	2020-03-04	Washington State Department of Health	Seattle Flu Study	Chu etl al
EPI_ISL_417092	hCoV-19/USA/WA-S39/2020 North America / USA / Washington / Snohomish County	2020-03-04	Washington State Department of Health	Seattle Flu Study	Chu etl al
EPI_ISL_417093	hCoV-19/USA/WA-S40/2020 North America / USA / Washington / Snohomish County	2020-02-28	Washington State Department of Health	Seattle Flu Study	Chu etl al
EPI_ISL_417095	hCoV-19/USA/WA-S42/2020 North America / USA / Washington / King County	2020-02-28	Washington State Department of Health	Seattle Flu Study	Chu etl al
EPI_ISL_417096	hCoV-19/USA/WA-S43/2020 North America / USA / Washington	2020-02-27	Washington

State Department of Health	Seattle Flu Study	Chu etl al
EPI_ISL_417097	hCoV-19/USA/WA-S44/2020 North America / USA / Washington	2020-02-28	Washington State Department of Health	Seattle Flu Study	Chu etl al
EPI_ISL_417098	hCoV-19/USA/WA-S45/2020 North America / USA / Washington	2020-02-29	Washington State Department of Health	Seattle Flu Study	Chu etl al
EPI_ISL_417099	hCoV-19/USA/WA-S46/2020 North America / USA / Washington	2020-02-29	Washington State Department of Health	Seattle Flu Study	Chu etl al
EPI_ISL_417100	hCoV-19/USA/WA-S47/2020 North America / USA / Washington / Snohomish County	2020-02-29	Washington State Department of Health	Seattle Flu Study	Chu etl al
EPI_ISL_417101	hCoV-19/USA/WA-S48/2020 North America / USA / Washington / Snohomish County	2020-02-29	Washington State Department of Health	Seattle Flu Study	Chu etl al
EPI_ISL_417102	hCoV-19/USA/WA-S49/2020 North America / USA / Washington / King County	2020-03-04	Washington State Department of Health	Seattle Flu Study	Chu etl al
EPI_ISL_417103	hCoV-19/USA/WA-S50/2020 North America / USA / Washington / King County	2020-03-05	Washington State Department of Health	Seattle Flu Study	Chu etl al
EPI_ISL_417104	hCoV-19/USA/WA-S51/2020 North America / USA / Washington	2020-03-03	Washington State Department of Health	Seattle Flu Study	Chu etl al
EPI_ISL_417105	hCoV-19/USA/WA-S52/2020 North America / USA / Washington / Snohomish County	2020-03-03	Washington State Department of Health	Seattle Flu Study	Chu etl al
EPI_ISL_417106	hCoV-19/USA/WA-S53/2020 North America / USA / Washington / Snohomish County	2020-03-03	Washington State Department of Health	Seattle Flu Study	Chu etl al
EPI_ISL_417107	hCoV-19/USA/WA-S54/2020 North America / USA / Washington / King County	2020-03-05	Washington State Department of Health	Seattle Flu Study	Chu etl al
EPI_ISL_417108	hCoV-19/USA/WA-S55/2020 North America / USA / Washington / King County	2020-02-29	Washington State Department of Health	Seattle Flu Study	Chu etl al
EPI_ISL_417109	hCoV-19/USA/WA-S56/2020 North America / USA / Washington / King County	2020-03-05	Washington State Department of Health	Seattle Flu Study	Chu etl al
EPI_ISL_417110	hCoV-19/USA/WA-S57/2020 North America / USA / Washington	2020-03-03	Washington State Department of Health	Seattle Flu Study	Chu etl al
EPI_ISL_417111	hCoV-19/USA/WA-S58/2020 North America / USA / Washington / Snohomish County	2020-03-05	Washington State Department of Health	Seattle Flu Study	Chu etl al
EPI_ISL_417112	hCoV-19/USA/WA-S59/2020 North America / USA / Washington / King County	2020-03-02	Washington State Department of Health	Seattle Flu Study	Chu etl al
EPI_ISL_417114	hCoV-19/USA/WA-S61/2020 North America / USA / Washington / King County	2020-03-05	Washington State Department of Health	Seattle Flu Study	Chu etl al
EPI_ISL_417115	hCoV-19/USA/WA-S62/2020 North America / USA / Washington / Snohomish County	2020-03-03	Washington State Department of Health	Seattle Flu Study	Chu etl al
EPI_ISL_417116	hCoV-19/USA/WA-S63/2020 North America / USA / Washington / King County	2020-03-04	Washington State Department of Health	Seattle Flu Study	Chu etl al
EPI_ISL_417117	hCoV-19/USA/WA-S64/2020 North America / USA / Washington / King County	2020-03-03	Washington State Department of Health	Seattle Flu Study	Chu etl al
EPI_ISL_417118	hCoV-19/USA/WA-S65/2020 North America / USA / Washington / King County	2020-03-03	Washington State Department of Health	Seattle Flu Study	Chu etl al
EPI_ISL_417119	hCoV-19/USA/WA-S66/2020 North America / USA / Washington / Snohomish County	2020-03-06	Washington State Department of Health	Seattle Flu Study	Chu etl al
EPI_ISL_417120	hCoV-19/USA/WA-S67/2020 North America / USA / Washington / King County	2020-03-04	Washington State Department of Health	Seattle Flu Study	Chu etl al
EPI_ISL_417121	hCoV-19/USA/WA-S68/2020 North America / USA / Washington / King County	2020-03-04	Washington State Department of Health	Seattle Flu Study	Chu etl al
EPI_ISL_417122	hCoV-19/USA/WA-S69/2020 North America / USA / Washington / King County	2020-03-05	Washington State Department of Health	Seattle Flu Study	Chu etl al
EPI_ISL_417123	hCoV-19/USA/WA-S70/2020 North America / USA / Washington / King County	2020-03-05	Washington State Department of Health	Seattle Flu Study	Chu etl al
EPI_ISL_417124	hCoV-19/USA/WA-S71/2020 North America / USA / Washington / King County	2020-03-05	Washington State Department of Health	Seattle Flu Study	Chu etl al
EPI_ISL_417125	hCoV-19/USA/WA-S72/2020 North America / USA / Washington / King County	2020-03-06	Washington State Department of Health	Seattle Flu Study	Chu etl al
EPI_ISL_417126	hCoV-19/USA/WA-S73/2020 North America / USA / Washington	2020-03-05	Washington State Department of Health	Seattle Flu Study	Chu etl al
EPI_ISL_417127	hCoV-19/USA/WA-S74/2020 North America / USA / Washington / King County	2020-03-05	Washington State Department of Health	Seattle Flu Study	Chu etl al
EPI_ISL_417128	hCoV-19/USA/WA-S75/2020 North America / USA / Washington / King County	2020-03-05	Washington State Department of Health	Seattle Flu Study	Chu etl al
EPI_ISL_417129	hCoV-19/USA/WA-S76/2020 North America / USA / Washington / King County	2020-03-05	Washington State Department of Health	Seattle Flu Study	Chu etl al
EPI_ISL_417130	hCoV-19/USA/WA-S77/2020 North America / USA / Washington / King County	2020-03-05	Washington State Department of Health	Seattle Flu Study	Chu etl al
EPI_ISL_417132	hCoV-19/USA/WA-S79/2020 North America / USA / Washington / King County	2020-03-05	Washington State Department of Health	Seattle Flu Study	Chu etl al
EPI_ISL_417133	hCoV-19/USA/WA-S80/2020 North America / USA / Washington / King County	2020-03-05

Washington State Department of Health	Seattle Flu Study	Chu etl al
EPI_ISL_417134	hCoV-19/USA/WA-S81/2020	North America / USA / Washington / King County	2020-02-26
Washington State Department of Health	Seattle Flu Study	Chu etl al
EPI_ISL_417135	hCoV-19/USA/WA-S82/2020	North America / USA / Washington / King County	2020-02-22
Washington State Department of Health	Seattle Flu Study	Chu etl al
EPI_ISL_417136	hCoV-19/USA/WA-S83/2020	North America / USA / Washington / King County	2020-03-05
Washington State Department of Health	Seattle Flu Study	Chu etl al
EPI_ISL_417137	hCoV-19/USA/WA-S84/2020	North America / USA / Washington / King County	2020-02-21
Washington State Department of Health	Seattle Flu Study	Chu etl al
EPI_ISL_417139	hCoV-19/USA/WA-S86/2020	North America / USA / Washington / King County	2020-03-01
Washington State Department of Health	Seattle Flu Study	Chu etl al
EPI_ISL_417140	hCoV-19/USA/WA-S87/2020	North America / USA / Washington / King County	2020-03-01
Washington State Department of Health	Seattle Flu Study	Chu etl al
EPI_ISL_417141	hCoV-19/USA/WA-S88/2020	North America / USA / Washington / King County	2020-03-01
Washington State Department of Health	Seattle Flu Study	Chu etl al
EPI_ISL_417142	hCoV-19/USA/WA-S89/2020	North America / USA / Washington / Umatilla County	2020-02-29
Washington State Department of Health	Seattle Flu Study	Chu etl al
EPI_ISL_417143	hCoV-19/USA/WA-S90/2020	North America / USA / Washington / Umatilla County	2020-02-29
Washington State Department of Health	Seattle Flu Study	Chu etl al
EPI_ISL_417144	hCoV-19/USA/WA-S91/2020	North America / USA / Washington / Snohomish County	2020-03-02
Washington State Department of Health	Seattle Flu Study	Chu etl al
EPI_ISL_417145	hCoV-19/USA/WA-S92/2020	North America / USA / Washington / King County	2020-02-29
Washington State Department of Health	Seattle Flu Study	Chu etl al
EPI_ISL_417146	hCoV-19/USA/WA-S93/2020	North America / USA / Washington / King County	2020-02-29
Washington State Department of Health	Seattle Flu Study	Chu etl al
EPI_ISL_417147	hCoV-19/USA/WA-S94/2020	North America / USA / Washington / Snohomish County	2020-02-28
Washington State Department of Health	Seattle Flu Study	Chu etl al
EPI_ISL_417148	hCoV-19/USA/WA-S95/2020	North America / USA / Washington / King County	2020-02-28
Washington State Department of Health	Seattle Flu Study	Chu etl al
EPI_ISL_417149	hCoV-19/USA/WA-S96/2020	North America / USA / Washington / King County	2020-02-28
Washington State Department of Health	Seattle Flu Study	Chu etl al
EPI_ISL_417150	hCoV-19/USA/WA-S97/2020	North America / USA / Washington / King County	2020-02-28
Washington State Department of Health	Seattle Flu Study	Chu etl al
EPI_ISL_417151	hCoV-19/USA/WA-S98/2020	North America / USA / Washington / King County	2020-02-29
Washington State Department of Health	Seattle Flu Study	Chu etl al
EPI_ISL_417152	hCoV-19/USA/WA-S99/2020	North America / USA / Washington / King County	2020-02-28
Washington State Department of Health	Seattle Flu Study	Chu etl al
EPI_ISL_417153	hCoV-19/USA/WA-S100/2020	North America / USA / Washington / King County	2020-02-29
Washington State Department of Health	Seattle Flu Study	Chu etl al
EPI_ISL_417154	hCoV-19/USA/WA-S101/2020	North America / USA / Washington / King County	2020-02-28
Washington State Department of Health	Seattle Flu Study	Chu etl al
EPI_ISL_417155	hCoV-19/USA/WA-S102/2020	North America / USA / Washington / King County	2020-02-28
Washington State Department of Health	Seattle Flu Study	Chu etl al
EPI_ISL_417156	hCoV-19/USA/WA-S103/2020	North America / USA / Washington / King County	2020-02-28
Washington State Department of Health	Seattle Flu Study	Chu etl al
EPI_ISL_417157	hCoV-19/USA/WA-S104/2020	North America / USA / Washington / Snohomish County	2020-03-05	Washington State Department of Health	Seattle Flu Study	Chu etl al
EPI_ISL_417158	hCoV-19/USA/WA-S105/2020	North America / USA / Washington / King County	2020-02-28
Washington State Department of Health	Seattle Flu Study	Chu etl al
EPI_ISL_417159	hCoV-19/USA/WA-S106/2020	North America / USA / Washington / King County	2020-02-29
Washington State Department of Health	Seattle Flu Study	Chu etl al
EPI_ISL_417160	hCoV-19/USA/WA-S107/2020	North America / USA / Washington / King County	2020-02-29
Washington State Department of Health	Seattle Flu Study	Chu etl al
EPI_ISL_417161	hCoV-19/USA/WA-S108/2020	North America / USA / Washington / King County	2020-02-29
Washington State Department of Health	Seattle Flu Study	Chu etl al
EPI_ISL_417162	hCoV-19/USA/WA-S109/2020	North America / USA / Washington / Snohomish County	2020-03-01	Washington State Department of Health	Seattle Flu Study	Chu etl al
EPI_ISL_417163	hCoV-19/USA/WA-S110/2020	North America / USA / Washington / King County	2020-03-05
Seattle Flu Study	Seattle Flu Study	Chu etl al
EPI_ISL_417164	hCoV-19/USA/WA-S111/2020	North America / USA / Washington / King County	2020-03-07
Seattle Flu Study	Seattle Flu Study	Chu etl al
EPI_ISL_417165	hCoV-19/USA/WA-S112/2020	North America / USA / Washington / King County	2020-03-08
Seattle Flu Study	Seattle Flu Study	Chu etl al
EPI_ISL_417167	hCoV-19/USA/WA-S114/2020	North America / USA / Washington / Snohomish County	2020-03-05	Washington State Department of Health	Seattle Flu Study	Chu etl al
EPI_ISL_417168	hCoV-19/USA/WA-S115/2020	North America / USA / Washington / King County	2020-02-29
Washington State Department of Health	Seattle Flu Study	Chu etl al
EPI_ISL_417169	hCoV-19/USA/WA-S116/2020	North America / USA / Washington	2020-03-02
Washington State Department of Health	Seattle Flu Study	Chu etl al
EPI_ISL_417170	hCoV-19/USA/WA-S117/2020	North America / USA / Washington	2020-03-02

Washington State Department of Health   Seattle Flu Study   Chu etl al
EPI_ISL_417171 hCoV-19/USA/WA-S118/2020   North America / USA / Washington / King County 2020-03-01
Washington State Department of Health   Seattle Flu Study   Chu etl al
EPI_ISL_417172 hCoV-19/USA/WA-S119/2020   North America / USA / Washington / King County 2020-02-29
Washington State Department of Health   Seattle Flu Study   Chu etl al
EPI_ISL_417175 hCoV-19/USA/WA-S122/2020   North America / USA / Washington / Grant County 2020-03-02
Washington State Department of Health   Seattle Flu Study   Chu etl al
EPI_ISL_417176 hCoV-19/Hong Kong/HKPU1_2101/2020   Asia / Hong Kong   2020-01-21   Department of Pathology, Princess Margaret Hospital   Department of Health Technology and Informatics, Faculty of Health and Social Science, The Hong Kong Polytechnic University   Kenneth Siu-Sing LEUNG, Timothy Ting-Leung NG, Alan Ka-Lun WU, Miranda Chong-Yee YAU, Hiu-Yin LAO, Ming-Pan CHOI, Kingsley King-Gee TAM, Lam-Kwong LEE, Barry Kin-Chung WONG, Alex Yat-Man HO, Kam-Tong Yip, Kwok-Cheung LUNG, Raymond Wai-To LIU, Eugene Yuk-Keung TSO, Wai-Shing LEUNG, Man-Chun CHAN, Yuk-Yung NG, Kit-Man SIN, Kitty Sau-Chun FUNG, Sandy Ka-Yee CHAU, Wing-Kin TO, Tak-Lun Que, David Ho-Keung SHUM, Shea Ping YIP, Wing Cheong YAM, Gilman Kit-Hang SIU
EPI_ISL_417178 hCoV-19/Hong Kong/HKPU6_2101/2020   Asia / Hong Kong   2020-01-25   Department of Pathology, Princess Margaret Hospital   Department of Health Technology and Informatics, Faculty of Health and Social Science, The Hong Kong Polytechnic University   Kenneth Siu-Sing LEUNG, Timothy Ting-Leung NG, Alan Ka-Lun WU, Miranda Chong-Yee YAU, Hiu-Yin LAO, Ming-Pan CHOI, Kingsley King-Gee TAM, Lam-Kwong LEE, Barry Kin-Chung WONG, Alex Yat-Man HO, Kam-Tong Yip, Kwok-Cheung LUNG, Raymond Wai-To LIU, Eugene Yuk-Keung TSO, Wai-Shing LEUNG, Man-Chun CHAN, Yuk-Yung NG, Kit-Man SIN, Kitty Sau-Chun FUNG, Sandy Ka-Yee CHAU, Wing-Kin TO, Tak-Lun Que, David Ho-Keung SHUM, Shea Ping YIP, Wing Cheong YAM, Gilman Kit-Hang SIU
EPI_ISL_417181 hCoV-19/Hong Kong/HKPU19_0402/2020   Asia / Hong Kong   2020-02-05   Department of Pathology, United Christian Hospital   Department of Health Technology and Informatics, Faculty of Health and Social Science, The Hong Kong Polytechnic University   Kenneth Siu-Sing LEUNG, Timothy Ting-Leung NG, Alan Ka-Lun WU, Miranda Chong-Yee YAU, Hiu-Yin LAO, Ming-Pan CHOI, Kingsley King-Gee TAM, Lam-Kwong LEE, Barry Kin-Chung WONG, Alex Yat-Man HO, Kam-Tong Yip, Kwok-Cheung LUNG, Raymond Wai-To LIU, Eugene Yuk-Keung TSO, Wai-Shing LEUNG, Man-Chun CHAN, Yuk-Yung NG, Kit-Man SIN, Kitty Sau-Chun FUNG, Sandy Ka-Yee CHAU, Wing-Kin TO, Tak-Lun Que, David Ho-Keung SHUM, Shea Ping YIP, Wing Cheong YAM, Gilman Kit-Hang SIU
EPI_ISL_417185 hCoV-19/Hong Kong/HKPU28_3001/2020   Asia / Hong Kong   2020-02-09   Department of Pathology, United Christian Hospital   Department of Health Technology and Informatics, Faculty of Health and Social Science, The Hong Kong Polytechnic University   Kenneth Siu-Sing LEUNG, Timothy Ting-Leung NG, Alan Ka-Lun WU, Miranda Chong-Yee YAU, Hiu-Yin LAO, Ming-Pan CHOI, Kingsley King-Gee TAM, Lam-Kwong LEE, Barry Kin-Chung WONG, Alex Yat-Man HO, Kam-Tong Yip, Kwok-Cheung LUNG, Raymond Wai-To LIU, Eugene Yuk-Keung TSO, Wai-Shing LEUNG, Man-Chun CHAN, Yuk-Yung NG, Kit-Man SIN, Kitty Sau-Chun FUNG, Sandy Ka-Yee CHAU, Wing-Kin TO, Tak-Lun Que, David Ho-Keung SHUM, Shea Ping YIP, Wing Cheong YAM, Gilman Kit-Hang SIU
EPI_ISL_417186 hCoV-19/South Africa/R03006/2020   Africa / South Africa / KZN   2020-03-07
National Institute for Communicable Diseases of the National Health Laboratory Service National Institute for Communicable Diseases of the National Health Laboratory Service Allam M, Kwenda S, van Heusden P, Khumalo Z, Mohale T, Subramoney K, von Gottberg, A, Ismail A, Bhiman JN
EPI_ISL_417187 hCoV-19/Hong Kong/HKPU29_0102/2020   Asia / Hong Kong   2020-02-08   Department of Clinical Pathology, Pamela Youde Nethersole Eastern Hospital   Department of Health Technology and Informatics, Faculty of Health and Social Science, The Hong Kong Polytechnic University Kenneth Siu-Sing LEUNG, Timothy Ting-Leung NG, Alan Ka-Lun WU, Miranda Chong-Yee YAU, Hiu-Yin LAO, Ming-Pan CHOI, Kingsley King-Gee TAM, Lam-Kwong LEE, Barry Kin-Chung WONG, Alex Yat-Man HO, Kam-Tong Yip, Kwok-Cheung LUNG, Raymond Wai-To LIU, Eugene Yuk-Keung TSO, Wai-Shing LEUNG, Man-Chun CHAN, Yuk-Yung NG, Kit-Man SIN, Kitty Sau-Chun FUNG, Sandy Ka-Yee CHAU, Wing-Kin TO, Tak-Lun Que, David Ho-Keung SHUM, Shea Ping YIP, Wing Cheong YAM, Gilman Kit-Hang SIU
EPI_ISL_417188 hCoV-19/Hong Kong/HKPU30_2901/2020   Asia / Hong Kong   2020-02-08   Department of Clinical Pathology, Pamela Youde Nethersole Eastern Hospital   Department of Health Technology and Informatics, Faculty of Health and Social Science, The Hong Kong Polytechnic University Kenneth Siu-Sing LEUNG, Timothy Ting-Leung NG, Alan Ka-Lun WU, Miranda Chong-Yee YAU, Hiu-Yin LAO, Ming-Pan CHOI, Kingsley King-Gee TAM, Lam-Kwong LEE, Barry Kin-Chung WONG, Alex Yat-Man HO, Kam-Tong Yip, Kwok-Cheung LUNG, Raymond Wai-To LIU, Eugene Yuk-Keung TSO, Wai-Shing LEUNG, Man-Chun CHAN, Yuk-Yung NG, Kit-Man SIN, Kitty Sau-Chun FUNG, Sandy Ka-Yee CHAU, Wing-Kin TO, Tak-Lun Que, David Ho-Keung SHUM, Shea Ping YIP, Wing Cheong YAM, Gilman Kit-Hang SIU
EPI_ISL_417191 hCoV-19/USA/MN5-MDH5/2020   North America / USA / Minnesota 2020-03-10   Minnesota Department of Health, Public Health Laboratory Minnesota Department of Health, Public Health Laboratory
Matt Plumb, Jake Garfin and Xiong Wang
EPI_ISL_417192 hCoV-19/USA/MN25-MDH25/2020   North America / USA / Minnesota 2020-03-12   Minnesota Department of Health, Public Health Laboratory Minnesota Department of Health, Public Health Laboratory
Matt Plumb, Jake Garfin and Xiong Wang
EPI_ISL_417193 hCoV-19/Hong Kong/HKPU32_0402/2020   Asia / Hong Kong   2020-02-09   Department of Clinical Pathology, Pamela Youde Nethersole Eastern Hospital   Department of Health Technology and Informatics, Faculty of Health and Social Science, The Hong Kong Polytechnic University Kenneth Siu-Sing LEUNG, Timothy Ting-Leung NG, Alan Ka-Lun WU, Miranda Chong-Yee YAU, Hiu-Yin LAO, Ming-Pan CHOI, Kingsley King-Gee TAM, Lam-Kwong LEE, Barry Kin-Chung WONG, Alex Yat-Man HO, Kam-Tong Yip, Kwok-Cheung LUNG, Raymond Wai-To LIU, Eugene Yuk-Keung TSO, Wai-Shing LEUNG, Man-Chun CHAN, Yuk-Yung NG, Kit-Man SIN, Kitty Sau-Chun FUNG, Sandy Ka-Yee CHAU, Wing-Kin TO, Tak-Lun Que, David Ho-Keung SHUM, Shea Ping YIP, Wing Cheong YAM, Gilman Kit-Hang SIU
EPI_ISL_417194 hCoV-19/USA/MN26-MDH26/2020   North America / USA / Minnesota 2020-03-12   Minnesota Department of Health, Public Health Laboratory Minnesota Department of Health, Public Health Laboratory
Matt Plumb, Jake Garfin and Xiong Wang
EPI_ISL_417196 hCoV-19/USA/MN29-MDH29/2020   North America / USA / Minnesota 2020-03-11   Minnesota

Department of Health, Public Health Laboratory Minnesota Department of Health, Public Health Laboratory Matt Plumb, Jake Garfin and Xiong Wang
EPI_ISL_417197 hCoV-19/Hong Kong/HKPU34_3001/2020 Asia / Hong Kong 2020-02-09 Department of Clinical Pathology, Pamela Youde Nethersole Eastern Hospital Department of Health Technology and Informatics, Faculty of Health and Social Science, The Hong Kong Polytechnic University Kenneth Siu-Sing LEUNG, Timothy Ting-Leung NG, Alan Ka-Lun WU, Miranda Chong-Yee YAU, Hiu-Yin LAO, Ming-Pan CHOI, Kingsley King-Gee TAM, Lam-Kwong LEE, Barry Kin-Chung WONG, Alex Yat-Man HO, Kam-Tong Yip, Kwok-Cheung LUNG, Raymond Wai-To LIU, Eugene Yuk-Keung TSO, Wai-Shing LEUNG, Man-Chun CHAN, Yuk-Yung NG, Kit-Man SIN, Kitty Sau-Chun FUNG, Sandy Ka-Yee CHAU, Wing-Kin TO, Tak-Lun Que, David Ho-Keung SHUM, Shea Ping YIP, Wing Cheong YAM, Gilman Kit-Hang SIU
EPI_ISL_417250 hCoV-19/England/20108004702/2020 Europe / United Kingdom / England 2020-03-02 Respiratory Virus Unit, Microbiology Services Colindale, Public Health England Respiratory Virus Unit, Microbiology Services Colindale, Public Health England Monica Galiano, Shahjahan Miah, Angie Lackenby, Omolola Akinbami, Tiina Talts, Leena Bhaw, Richard Myers, Steven Platt, Kirstin Edwards, Jonathan Hubb, Joanna Ellis, Maria Zambon
EPI_ISL_417252 hCoV-19/England/20108006003/2020 Europe / United Kingdom / England 2020-03-05 Respiratory Virus Unit, Microbiology Services Colindale, Public Health England Respiratory Virus Unit, Microbiology Services Colindale, Public Health England Monica Galiano, Shahjahan Miah, Angie Lackenby, Omolola Akinbami, Tiina Talts, Leena Bhaw, Richard Myers, Steven Platt, Kirstin Edwards, Jonathan Hubb, Joanna Ellis, Maria Zambon
EPI_ISL_417254 hCoV-19/England/20108006802/2020 Europe / United Kingdom / England 2020-03-04 Respiratory Virus Unit, Microbiology Services Colindale, Public Health England Respiratory Virus Unit, Microbiology Services Colindale, Public Health England Monica Galiano, Shahjahan Miah, Angie Lackenby, Omolola Akinbami, Tiina Talts, Leena Bhaw, Richard Myers, Steven Platt, Kirstin Edwards, Jonathan Hubb, Joanna Ellis, Maria Zambon
EPI_ISL_417255 hCoV-19/England/20108007002/2020 Europe / United Kingdom / England 2020-03-04 Respiratory Virus Unit, Microbiology Services Colindale, Public Health England Respiratory Virus Unit, Microbiology Services Colindale, Public Health England Monica Galiano, Shahjahan Miah, Angie Lackenby, Omolola Akinbami, Tiina Talts, Leena Bhaw, Richard Myers, Steven Platt, Kirstin Edwards, Jonathan Hubb, Joanna Ellis, Maria Zambon
EPI_ISL_417256 hCoV-19/England/20108007302/2020 Europe / United Kingdom / England 2020-03-03 Respiratory Virus Unit, Microbiology Services Colindale, Public Health England Respiratory Virus Unit, Microbiology Services Colindale, Public Health England Monica Galiano, Shahjahan Miah, Angie Lackenby, Omolola Akinbami, Tiina Talts, Leena Bhaw, Richard Myers, Steven Platt, Kirstin Edwards, Jonathan Hubb, Joanna Ellis, Maria Zambon
EPI_ISL_417258 hCoV-19/England/20108034006/2020 Europe / United Kingdom / England 2020-03-06 Respiratory Virus Unit, Microbiology Services Colindale, Public Health England Respiratory Virus Unit, Microbiology Services Colindale, Public Health England Monica Galiano, Shahjahan Miah, Angie Lackenby, Omolola Akinbami, Tiina Talts, Leena Bhaw, Richard Myers, Steven Platt, Kirstin Edwards, Jonathan Hubb, Joanna Ellis, Maria Zambon
EPI_ISL_417260 hCoV-19/England/20109035906/2020 Europe / United Kingdom / England 2020-03-05 Respiratory Virus Unit, Microbiology Services Colindale, Public Health England Respiratory Virus Unit, Microbiology Services Colindale, Public Health England Monica Galiano, Shahjahan Miah, Angie Lackenby, Omolola Akinbami, Tiina Talts, Leena Bhaw, Richard Myers, Steven Platt, Kirstin Edwards, Jonathan Hubb, Joanna Ellis, Maria Zambon
EPI_ISL_417262 hCoV-19/England/20109039306/2020 Europe / United Kingdom / England 2020-03-06 Respiratory Virus Unit, Microbiology Services Colindale, Public Health England Respiratory Virus Unit, Microbiology Services Colindale, Public Health England Monica Galiano, Shahjahan Miah, Angie Lackenby, Omolola Akinbami, Tiina Talts, Leena Bhaw, Richard Myers, Steven Platt, Kirstin Edwards, Jonathan Hubb, Joanna Ellis, Maria Zambon
EPI_ISL_417263 hCoV-19/England/20109050106/2020 Europe / United Kingdom / England 2020-03-06 Respiratory Virus Unit, Microbiology Services Colindale, Public Health England Respiratory Virus Unit, Microbiology Services Colindale, Public Health England Monica Galiano, Shahjahan Miah, Angie Lackenby, Omolola Akinbami, Tiina Talts, Leena Bhaw, Richard Myers, Steven Platt, Kirstin Edwards, Jonathan Hubb, Joanna Ellis, Maria Zambon
EPI_ISL_417264 hCoV-19/England/20109050306/2020 Europe / United Kingdom / England 2020-03-04 Respiratory Virus Unit, Microbiology Services Colindale, Public Health England Respiratory Virus Unit, Microbiology Services Colindale, Public Health England Monica Galiano, Shahjahan Miah, Angie Lackenby, Omolola Akinbami, Tiina Talts, Leena Bhaw, Richard Myers, Steven Platt, Kirstin Edwards, Jonathan Hubb, Joanna Ellis, Maria Zambon
EPI_ISL_417265 hCoV-19/England/20109050406/2020 Europe / United Kingdom / England 2020-03-08 Respiratory Virus Unit, Microbiology Services Colindale, Public Health England Respiratory Virus Unit, Microbiology Services Colindale, Public Health England Monica Galiano, Shahjahan Miah, Angie Lackenby, Omolola Akinbami, Tiina Talts, Leena Bhaw, Richard Myers, Steven Platt, Kirstin Edwards, Jonathan Hubb, Joanna Ellis, Maria Zambon
EPI_ISL_417266 hCoV-19/England/20109050506/2020 Europe / United Kingdom / England 2020-03-05 Respiratory Virus Unit, Microbiology Services Colindale, Public Health England Respiratory Virus Unit, Microbiology Services Colindale, Public Health England Monica Galiano, Shahjahan Miah, Angie Lackenby, Omolola Akinbami, Tiina Talts, Leena Bhaw, Richard Myers, Steven Platt, Kirstin Edwards, Jonathan Hubb, Joanna Ellis, Maria Zambon
EPI_ISL_417267 hCoV-19/England/20109050606/2020 Europe / United Kingdom / England 2020-03-05

Respiratory Virus Unit, Microbiology Services Colindale, Public Health England Respiratory Virus Unit, Microbiology Services Colindale, Public Health England Monica Galiano, Shahjahan Miah, Angie Lackenby, Omolola Akinbami, Tiina Talts, Leena Bhaw, Richard Myers, Steven Platt, Kirstin Edwards, Jonathan Hubb, Joanna Ellis, Maria Zambon
EPI_ISL_417268 hCoV-19/England/20109050706/2020 Europe / United Kingdom / England 2020-03-05 Respiratory Virus Unit, Microbiology Services Colindale, Public Health England Respiratory Virus Unit, Microbiology Services Colindale, Public Health England Monica Galiano, Shahjahan Miah, Angie Lackenby, Omolola Akinbami, Tiina Talts, Leena Bhaw, Richard Myers, Steven Platt, Kirstin Edwards, Jonathan Hubb, Joanna Ellis, Maria Zambon
EPI_ISL_417269 hCoV-19/England/20109050806/2020 Europe / United Kingdom / England 2020-03-05 Respiratory Virus Unit, Microbiology Services Colindale, Public Health England Respiratory Virus Unit, Microbiology Services Colindale, Public Health England Monica Galiano, Shahjahan Miah, Angie Lackenby, Omolola Akinbami, Tiina Talts, Leena Bhaw, Richard Myers, Steven Platt, Kirstin Edwards, Jonathan Hubb, Joanna Ellis, Maria Zambon
EPI_ISL_417270 hCoV-19/England/20109051806/2020 Europe / United Kingdom / England 2020-03-06 Respiratory Virus Unit, Microbiology Services Colindale, Public Health England Respiratory Virus Unit, Microbiology Services Colindale, Public Health England Monica Galiano, Shahjahan Miah, Angie Lackenby, Omolola Akinbami, Tiina Talts, Leena Bhaw, Richard Myers, Steven Platt, Kirstin Edwards, Jonathan Hubb, Joanna Ellis, Maria Zambon
EPI_ISL_417272 hCoV-19/England/20109052006/2020 Europe / United Kingdom / England 2020-03-06 Respiratory Virus Unit, Microbiology Services Colindale, Public Health England Respiratory Virus Unit, Microbiology Services Colindale, Public Health England Monica Galiano, Shahjahan Miah, Angie Lackenby, Omolola Akinbami, Tiina Talts, Leena Bhaw, Richard Myers, Steven Platt, Kirstin Edwards, Jonathan Hubb, Joanna Ellis, Maria Zambon
EPI_ISL_417276 hCoV-19/England/20109052506/2020 Europe / United Kingdom / England 2020-03-07 Respiratory Virus Unit, Microbiology Services Colindale, Public Health England Respiratory Virus Unit, Microbiology Services Colindale, Public Health England Monica Galiano, Shahjahan Miah, Angie Lackenby, Omolola Akinbami, Tiina Talts, Leena Bhaw, Richard Myers, Steven Platt, Kirstin Edwards, Jonathan Hubb, Joanna Ellis, Maria Zambon
EPI_ISL_417278 hCoV-19/England/20109053406/2020 Europe / United Kingdom / England 2020-03-07 Respiratory Virus Unit, Microbiology Services Colindale, Public Health England Respiratory Virus Unit, Microbiology Services Colindale, Public Health England Monica Galiano, Shahjahan Miah, Angie Lackenby, Omolola Akinbami, Tiina Talts, Leena Bhaw, Richard Myers, Steven Platt, Kirstin Edwards, Jonathan Hubb, Joanna Ellis, Maria Zambon
EPI_ISL_417279 hCoV-19/England/20109053606/2020 Europe / United Kingdom / England 2020-03-07 Respiratory Virus Unit, Microbiology Services Colindale, Public Health England Respiratory Virus Unit, Microbiology Services Colindale, Public Health England Monica Galiano, Shahjahan Miah, Angie Lackenby, Omolola Akinbami, Tiina Talts, Leena Bhaw, Richard Myers, Steven Platt, Kirstin Edwards, Jonathan Hubb, Joanna Ellis, Maria Zambon
EPI_ISL_417280 hCoV-19/England/20109054806/2020 Europe / United Kingdom / England 2020-03-07 Respiratory Virus Unit, Microbiology Services Colindale, Public Health England Respiratory Virus Unit, Microbiology Services Colindale, Public Health England Monica Galiano, Shahjahan Miah, Angie Lackenby, Omolola Akinbami, Tiina Talts, Leena Bhaw, Richard Myers, Steven Platt, Kirstin Edwards, Jonathan Hubb, Joanna Ellis, Maria Zambon
EPI_ISL_417282 hCoV-19/England/20109056906/2020 Europe / United Kingdom / England 2020-03-05 Respiratory Virus Unit, Microbiology Services Colindale, Public Health England Respiratory Virus Unit, Microbiology Services Colindale, Public Health England Monica Galiano, Shahjahan Miah, Angie Lackenby, Omolola Akinbami, Tiina Talts, Leena Bhaw, Richard Myers, Steven Platt, Kirstin Edwards, Jonathan Hubb, Joanna Ellis, Maria Zambon
EPI_ISL_417283 hCoV-19/England/20109058906/2020 Europe / United Kingdom / England 2020-03-06 Respiratory Virus Unit, Microbiology Services Colindale, Public Health England Respiratory Virus Unit, Microbiology Services Colindale, Public Health England Monica Galiano, Shahjahan Miah, Angie Lackenby, Omolola Akinbami, Tiina Talts, Leena Bhaw, Richard Myers, Steven Platt, Kirstin Edwards, Jonathan Hubb, Joanna Ellis, Maria Zambon
EPI_ISL_417285 hCoV-19/England/20109093606/2020 Europe / United Kingdom / England 2020-03-06 Respiratory Virus Unit, Microbiology Services Colindale, Public Health England Respiratory Virus Unit, Microbiology Services Colindale, Public Health England Monica Galiano, Shahjahan Miah, Angie Lackenby, Omolola Akinbami, Tiina Talts, Leena Bhaw, Richard Myers, Steven Platt, Kirstin Edwards, Jonathan Hubb, Joanna Ellis, Maria Zambon
EPI_ISL_417286 hCoV-19/England/20109093706/2020 Europe / United Kingdom / England 2020-03-05 Respiratory Virus Unit, Microbiology Services Colindale, Public Health England Respiratory Virus Unit, Microbiology Services Colindale, Public Health England Monica Galiano, Shahjahan Miah, Angie Lackenby, Omolola Akinbami, Tiina Talts, Leena Bhaw, Richard Myers, Steven Platt, Kirstin Edwards, Jonathan Hubb, Joanna Ellis, Maria Zambon
EPI_ISL_417287 hCoV-19/England/20109093806/2020 Europe / United Kingdom / England 2020-03-06 Respiratory Virus Unit, Microbiology Services Colindale, Public Health England Respiratory Virus Unit, Microbiology Services Colindale, Public Health England Monica Galiano, Shahjahan Miah, Angie Lackenby, Omolola Akinbami, Tiina Talts, Leena Bhaw, Richard Myers, Steven Platt, Kirstin Edwards, Jonathan Hubb, Joanna Ellis, Maria Zambon
EPI_ISL_417289 hCoV-19/England/20109094006/2020 Europe / United Kingdom / England 2020-03-06

Respiratory Virus Unit, Microbiology Services Colindale, Public Health England Respiratory Virus Unit, Microbiology Services Colindale, Public Health England Monica Galiano, Shahjahan Miah, Angie Lackenby, Omolola Akinbami, Tiina Talts, Leena Bhaw, Richard Myers, Steven Platt, Kirstin Edwards, Jonathan Hubb, Joanna Ellis, Maria Zambon
EPI_ISL_417290 hCoV-19/England/20109094106/2020 Europe / United Kingdom / England 2020-03-06 Respiratory Virus Unit, Microbiology Services Colindale, Public Health England Respiratory Virus Unit, Microbiology Services Colindale, Public Health England Monica Galiano, Shahjahan Miah, Angie Lackenby, Omolola Akinbami, Tiina Talts, Leena Bhaw, Richard Myers, Steven Platt, Kirstin Edwards, Jonathan Hubb, Joanna Ellis, Maria Zambon
EPI_ISL_417291 hCoV-19/England/20109094206/2020 Europe / United Kingdom / England 2020-03-08 Respiratory Virus Unit, Microbiology Services Colindale, Public Health England Respiratory Virus Unit, Microbiology Services Colindale, Public Health England Monica Galiano, Shahjahan Miah, Angie Lackenby, Omolola Akinbami, Tiina Talts, Leena Bhaw, Richard Myers, Steven Platt, Kirstin Edwards, Jonathan Hubb, Joanna Ellis, Maria Zambon
EPI_ISL_417292 hCoV-19/England/20109094506/2020 Europe / United Kingdom / England 2020-03-08 Respiratory Virus Unit, Microbiology Services Colindale, Public Health England Respiratory Virus Unit, Microbiology Services Colindale, Public Health England Monica Galiano, Shahjahan Miah, Angie Lackenby, Omolola Akinbami, Tiina Talts, Leena Bhaw, Richard Myers, Steven Platt, Kirstin Edwards, Jonathan Hubb, Joanna Ellis, Maria Zambon
EPI_ISL_417293 hCoV-19/England/20109097506/2020 Europe / United Kingdom / England 2020-03-08 Respiratory Virus Unit, Microbiology Services Colindale, Public Health England Respiratory Virus Unit, Microbiology Services Colindale, Public Health England Monica Galiano, Shahjahan Miah, Angie Lackenby, Omolola Akinbami, Tiina Talts, Leena Bhaw, Richard Myers, Steven Platt, Kirstin Edwards, Jonathan Hubb, Joanna Ellis, Maria Zambon
EPI_ISL_417295 hCoV-19/England/20109098806/2020 Europe / United Kingdom / England 2020-03-08 Respiratory Virus Unit, Microbiology Services Colindale, Public Health England Respiratory Virus Unit, Microbiology Services Colindale, Public Health England Monica Galiano, Shahjahan Miah, Angie Lackenby, Omolola Akinbami, Tiina Talts, Leena Bhaw, Richard Myers, Steven Platt, Kirstin Edwards, Jonathan Hubb, Joanna Ellis, Maria Zambon
EPI_ISL_417297 hCoV-19/England/20109099006/2020 Europe / United Kingdom / England 2020-03-06 Respiratory Virus Unit, Microbiology Services Colindale, Public Health England Respiratory Virus Unit, Microbiology Services Colindale, Public Health England Monica Galiano, Shahjahan Miah, Angie Lackenby, Omolola Akinbami, Tiina Talts, Leena Bhaw, Richard Myers, Steven Platt, Kirstin Edwards, Jonathan Hubb, Joanna Ellis, Maria Zambon
EPI_ISL_417298 hCoV-19/England/20109099106/2020 Europe / United Kingdom / England 2020-03-08 Respiratory Virus Unit, Microbiology Services Colindale, Public Health England Respiratory Virus Unit, Microbiology Services Colindale, Public Health England Monica Galiano, Shahjahan Miah, Angie Lackenby, Omolola Akinbami, Tiina Talts, Leena Bhaw, Richard Myers, Steven Platt, Kirstin Edwards, Jonathan Hubb, Joanna Ellis, Maria Zambon
EPI_ISL_417299 hCoV-19/England/20109099206/2020 Europe / United Kingdom / England 2020-03-08 Respiratory Virus Unit, Microbiology Services Colindale, Public Health England Respiratory Virus Unit, Microbiology Services Colindale, Public Health England Monica Galiano, Shahjahan Miah, Angie Lackenby, Omolola Akinbami, Tiina Talts, Leena Bhaw, Richard Myers, Steven Platt, Kirstin Edwards, Jonathan Hubb, Joanna Ellis, Maria Zambon
EPI_ISL_418400 hCoV-19/Finland/13M83/2020 Europe / Finland 2020-03-13 Department of Virology and Immunology, University of Helsinki and Helsinki University Hospital, Huslab Finland Department of Virology, Faculty of Medicine, University of Helsinki, Helsinki, Finland Teemu Smura, Hannimari Kallio-Kokko, Olli Vapalahti
EPI_ISL_418401 hCoV-19/Finland/14M12/2020 Europe / Finland 2020-03-14 Department of Virology and Immunology, University of Helsinki and Helsinki University Hospital, Huslab Finland Department of Virology, Faculty of Medicine, University of Helsinki, Helsinki, Finland Teemu Smura, Hannimari Kallio-Kokko, Olli Vapalahti
EPI_ISL_418402 hCoV-19/Finland/14M13/2020 Europe / Finland 2020-03-14 Department of Virology and Immunology, University of Helsinki and Helsinki University Hospital, Huslab Finland Department of Virology, Faculty of Medicine, University of Helsinki, Helsinki, Finland Teemu Smura, Hannimari Kallio-Kokko, Olli Vapalahti
EPI_ISL_418403 hCoV-19/Finland/14M14/2020 Europe / Finland 2020-03-14 Department of Virology and Immunology, University of Helsinki and Helsinki University Hospital, Huslab Finland Department of Virology, Faculty of Medicine, University of Helsinki, Helsinki, Finland Teemu Smura, Hannimari Kallio-Kokko, Olli Vapalahti
EPI_ISL_418404 hCoV-19/Finland/14M16/2020 Europe / Finland 2020-03-14 Department of Virology and Immunology, University of Helsinki and Helsinki University Hospital, Huslab Finland Department of Virology, Faculty of Medicine, University of Helsinki, Helsinki, Finland Teemu Smura, Hannimari Kallio-Kokko, Olli Vapalahti
EPI_ISL_418405 hCoV-19/Finland/14M20/2020 Europe / Finland 2020-03-14 Department of Virology and Immunology, University of Helsinki and Helsinki University Hospital, Huslab Finland Department of Virology, Faculty of Medicine, University of Helsinki, Helsinki, Finland Teemu Smura, Hannimari Kallio-Kokko, Olli Vapalahti
EPI_ISL_418406 hCoV-19/Finland/14M26/2020 Europe / Finland 2020-03-14 Department of Virology and Immunology, University of Helsinki and Helsinki University Hospital, Huslab Finland Department of Virology,

Faculty of Medicine, University of Helsinki, Helsinki, Finland Teemu Smura, Hannimari Kallio-Kokko, Olli Vapalahti
EPI_ISL_418408 hCoV-19/Finland/14M32/2020 Europe / Finland / Helsinki 2020-03-14 Department of Virology and Immunology, University of Helsinki and Helsinki University Hospital, Huslab Finland Department of Virology, Faculty of Medicine, University of Helsinki, Helsinki, Finland Teemu Smura, Hannimari Kallio-Kokko, Olli Vapalahti
EPI_ISL_418409 hCoV-19/Finland/14M74/2020 Europe / Finland / Helsinki 2020-03-14 Department of Virology and Immunology, University of Helsinki and Helsinki University Hospital, Huslab Finland Department of Virology, Faculty of Medicine, University of Helsinki, Helsinki, Finland Teemu Smura, Hannimari Kallio-Kokko, Olli Vapalahti
EPI_ISL_418410 hCoV-19/Finland/14M77/2020 Europe / Finland / Helsinki 2020-03-14 Department of Virology and Immunology, University of Helsinki and Helsinki University Hospital, Huslab Finland Department of Virology, Faculty of Medicine, University of Helsinki, Helsinki, Finland Teemu Smura, Hannimari Kallio-Kokko, Olli Vapalahti
EPI_ISL_418411 hCoV-19/Finland/14M82/2020 Europe / Finland / Helsinki 2020-03-14 Department of Virology and Immunology, University of Helsinki and Helsinki University Hospital, Huslab Finland Department of Virology, Faculty of Medicine, University of Helsinki, Helsinki, Finland Teemu Smura, Hannimari Kallio-Kokko, Olli Vapalahti
EPI_ISL_418412 hCoV-19/France/ARA094100/2020 Europe / France / ARA / Privas 2020-03-15 Centre Hospitalier des Vals d'Ardeche CNR Virus des Infections Respiratoires - France SUD Antonin Bal, Gregory Destras, Gwendolyne Burfin, Solenne Brun, Carine Moustaud, Raphaelle Lamy, Alexandre Gaymard, Maude Bouscambert-Duchamp, Florence Morfin-Sherpa, Martine Valette, Bruno Lina, Laurence Josset
EPI_ISL_418413 hCoV-19/France/ARA09428/2020 Europe / France / ARA / Macon 2020-03-15 Centre Hospitalier de Macon CNR Virus des Infections Respiratoires - France SUD Antonin Bal, Gregory Destras, Gwendolyne Burfin, Solenne Brun, Carine Moustaud, Raphaelle Lamy, Alexandre Gaymard, Maude Bouscambert-Duchamp, Florence Morfin-Sherpa, Martine Valette, Bruno Lina, Laurence Josset
EPI_ISL_418414 hCoV-19/France/ARA09434/2020 Europe / France / ARA / Valence 2020-03-15 Centre Hospitalier de Valence CNR Virus des Infections Respiratoires - France SUD Antonin Bal, Gregory Destras, Gwendolyne Burfin, Solenne Brun, Carine Moustaud, Raphaelle Lamy, Alexandre Gaymard, Maude Bouscambert-Duchamp, Florence Morfin-Sherpa, Martine Valette, Bruno Lina, Laurence Josset
EPI_ISL_418416 hCoV-19/France/ARA09588/2020 Europe / France / ARA / Venissieux 2020-03-16 GH Les Portes du Sud CNR Virus des Infections Respiratoires - France SUD Antonin Bal, Gregory Destras, Gwendolyne Burfin, Solenne Brun, Carine Moustaud, Raphaelle Lamy, Alexandre Gaymard, Maude Bouscambert-Duchamp, Florence Morfin-Sherpa, Martine Valette, Bruno Lina, Laurence Josset
EPI_ISL_418417 hCoV-19/France/ARA09686/2020 Europe / France / ARA / Valence 2020-03-16 Centre Hospitalier de Valence CNR Virus des Infections Respiratoires - France SUD Antonin Bal, Gregory Destras, Gwendolyne Burfin, Solenne Brun, Carine Moustaud, Raphaelle Lamy, Alexandre Gaymard, Maude Bouscambert-Duchamp, Florence Morfin-Sherpa, Martine Valette, Bruno Lina, Laurence Josset
EPI_ISL_418418 hCoV-19/France/ARA10163/2020 Europe / France / ARA / Lyon 2020-03-16 Centre Hospitalier Saint Joseph Saint Luc CNR Virus des Infections Respiratoires - France SUD Antonin Bal, Gregory Destras, Gwendolyne Burfin, Solenne Brun, Carine Moustaud, Raphaelle Lamy, Alexandre Gaymard, Maude Bouscambert-Duchamp, Florence Morfin-Sherpa, Martine Valette, Bruno Lina, Laurence Josset
EPI_ISL_418419 hCoV-19/France/ARA10165/2020 Europe / France / ARA / Lyon 2020-03-16 Centre Hospitalier Saint Joseph Saint Luc CNR Virus des Infections Respiratoires - France SUD Antonin Bal, Gregory Destras, Gwendolyne Burfin, Solenne Brun, Carine Moustaud, Raphaelle Lamy, Alexandre Gaymard, Maude Bouscambert-Duchamp, Florence Morfin-Sherpa, Martine Valette, Bruno Lina, Laurence Josset
EPI_ISL_418420 hCoV-19/France/ARA10170/2020 Europe / France / ARA / Lyon 2020-03-17 Institut des Agents Infectieux (IAI), Hospices Civils de Lyon CNR Virus des Infections Respiratoires - France SUD Antonin Bal, Gregory Destras, Gwendolyne Burfin, Solenne Brun, Carine Moustaud, Raphaelle Lamy, Alexandre Gaymard, Maude Bouscambert-Duchamp, Florence Morfin-Sherpa, Martine Valette, Bruno Lina, Laurence Josset
EPI_ISL_418422 hCoV-19/France/ARA10184/2020 Europe / France / ARA / Lyon 2020-03-17 Institut des Agents Infectieux (IAI), Hospices Civils de Lyon CNR Virus des Infections Respiratoires - France SUD Antonin Bal, Gregory Destras, Gwendolyne Burfin, Solenne Brun, Carine Moustaud, Raphaelle Lamy, Alexandre Gaymard, Maude Bouscambert-Duchamp, Florence Morfin-Sherpa, Martine Valette, Bruno Lina, Laurence Josset
EPI_ISL_418423 hCoV-19/France/ARA10188/2020 Europe / France / ARA / Lyon 2020-03-17 Institut des Agents Infectieux (IAI), Hospices Civils de Lyon CNR Virus des Infections Respiratoires - France SUD Antonin Bal, Gregory Destras, Gwendolyne Burfin, Solenne Brun, Carine Moustaud, Raphaelle Lamy, Alexandre Gaymard, Maude Bouscambert-Duchamp, Florence Morfin-Sherpa, Martine Valette, Bruno Lina, Laurence Josset
EPI_ISL_418424 hCoV-19/France/ARA10189/2020 Europe / France / ARA / Lyon 2020-03-17 Institut des Agents Infectieux (IAI), Hospices Civils de Lyon CNR Virus des Infections Respiratoires - France SUD Antonin Bal, Gregory Destras, Gwendolyne Burfin, Solenne Brun, Carine Moustaud, Raphaelle Lamy, Alexandre Gaymard, Maude Bouscambert-Duchamp, Florence Morfin-Sherpa, Martine Valette, Bruno Lina, Laurence Josset
EPI_ISL_418425 hCoV-19/France/ARA10192/2020 Europe / France / ARA / Lyon 2020-03-17 Institut des Agents Infectieux (IAI), Hospices Civils de Lyon CNR Virus des Infections Respiratoires - France SUD Antonin Bal, Gregory Destras, Gwendolyne Burfin, Solenne Brun, Carine Moustaud, Raphaelle Lamy, Alexandre Gaymard, Maude Bouscambert-Duchamp, Florence Morfin-Sherpa, Martine Valette, Bruno Lina, Laurence Josset
EPI_ISL_418426 hCoV-19/France/ARA10251/2020 Europe / France / ARA / Bourg-en-Bresse 2020-03-17 Centre Hospitalier de Bourg en Bresse CNR Virus des Infections Respiratoires - France SUD Antonin Bal, Gregory Destras, Gwendolyne Burfin, Solenne Brun, Carine Moustaud, Raphaelle Lamy, Alexandre Gaymard, Maude Bouscambert-Duchamp, Florence Morfin-Sherpa, Martine Valette, Bruno Lina, Laurence Josset

EPI_ISL_418427 hCoV-19/France/ARA10257/2020 Europe / France / ARA / Saint-Priest 2020-03-17 Hopital Privé de l'Est Lyonnais CNR Virus des Infections Respiratoires - France SUD Antonin Bal, Gregory Destras, Gwendolyne Burfin, Solenne Brun, Carine Moustaud, Raphaelle Lamy, Alexandre Gaymard, Maude Bouscambert-Duchamp, Florence Morfin-Sherpa, Martine Valette, Bruno Lina, Laurence Josset
EPI_ISL_418428 hCoV-19/France/ARA10282/2020 Europe / France / ARA / Vienne 2020-03-17 Centre Hospitalier Lucien Hussel CNR Virus des Infections Respiratoires - France SUD Antonin Bal, Gregory Destras, Gwendolyne Burfin, Solenne Brun, Carine Moustaud, Raphaelle Lamy, Alexandre Gaymard, Maude Bouscambert-Duchamp, Florence Morfin-Sherpa, Martine Valette, Bruno Lina, Laurence Josset
EPI_ISL_418429 hCoV-19/France/ARA10876/2020 Europe / France / ARA / Lyon 2020-03-18 Institut des Agents Infectieux (IAI), Hospices Civils de Lyon CNR Virus des Infections Respiratoires - France SUD Antonin Bal, Gregory Destras, Gwendolyne Burfin, Solenne Brun, Carine Moustaud, Raphaelle Lamy, Alexandre Gaymard, Maude Bouscambert-Duchamp, Florence Morfin-Sherpa, Martine Valette, Bruno Lina, Laurence Josset
EPI_ISL_418430 hCoV-19/France/ARA10910/2020 Europe / France / ARA / Lyon 2020-03-18 Institut des Agents Infectieux (IAI), Hospices Civils de Lyon CNR Virus des Infections Respiratoires - France SUD Antonin Bal, Gregory Destras, Gwendolyne Burfin, Solenne Brun, Carine Moustaud, Raphaelle Lamy, Alexandre Gaymard, Maude Bouscambert-Duchamp, Florence Morfin-Sherpa, Martine Valette, Bruno Lina, Laurence Josset
EPI_ISL_418431 hCoV-19/France/ARA10968/2020 Europe / France / ARA / Lyon 2020-03-18 Institut des Agents Infectieux (IAI), Hospices Civils de Lyon CNR Virus des Infections Respiratoires - France SUD Antonin Bal, Gregory Destras, Gwendolyne Burfin, Solenne Brun, Carine Moustaud, Raphaelle Lamy, Alexandre Gaymard, Maude Bouscambert-Duchamp, Florence Morfin-Sherpa, Martine Valette, Bruno Lina, Laurence Josset
EPI_ISL_418432 hCoV-19/France/ARA11036/2020 Europe / France / ARA / Lyon 2020-03-18 Institut des Agents Infectieux (IAI), Hospices Civils de Lyon CNR Virus des Infections Respiratoires - France SUD Antonin Bal, Gregory Destras, Gwendolyne Burfin, Solenne Brun, Carine Moustaud, Raphaelle Lamy, Alexandre Gaymard, Maude Bouscambert-Duchamp, Florence Morfin-Sherpa, Martine Valette, Bruno Lina, Laurence Josset
EPI_ISL_418441 hCoV-19/Hangzhou/HZ48/2020 Asia / China / Hangzhou 2020-01-21 Hangzhou Center for Disease Control and Prevention Inspection Center of Hangzhou Center for Disease Control and Prevention Yu hua, Wang haoqiu, Li jun, Yu xinfeng, Pan jingcao
EPI_ISL_418442 hCoV-19/Hangzhou/HZ49/2020 Asia / China / Hangzhou 2020-01-21 Hangzhou Center for Disease Control and Prevention Inspection Center of Hangzhou Center for Disease Control and Prevention Yu hua, Wang haoqiu, Li jun, Yu xinfeng, Pan jingcao
EPI_ISL_418502 hCoV-19/Hangzhou/HZ60/2020 Asia / China / Hangzhou 2020-01-22 Hangzhou Center for Disease Control and Prevention Inspection Center of Hangzhou Center for Disease Control and Prevention Yu hua, Wang haoqiu, Li jun, Yu xinfeng, Pan jingcao
EPI_ISL_418503 hCoV-19/Hangzhou/HZ62/2020 Asia / China / Hangzhou 2020-01-22 Hangzhou Center for Disease Control and Prevention Inspection Center of Hangzhou Center for Disease Control and Prevention Yu hua, Wang haoqiu, Li jun, Yu xinfeng, Pan jingcao
EPI_ISL_418504 hCoV-19/Hangzhou/HZ79/2020 Asia / China / Hangzhou 2020-01-21 Hangzhou Center for Disease Control and Prevention Inspection Center of Hangzhou Center for Disease Control and Prevention Yu hua, Wang haoqiu, Li jun, Yu xinfeng, Pan jingcao
EPI_ISL_418506 hCoV-19/Hangzhou/HZ90/2020 Asia / China / Hangzhou 2020-01-21 Hangzhou Center for Disease Control and Prevention Inspection Center of Hanghzou Center for Disease Control and Prevention Yu hua, Wang haoqiu, Li jun, Yu xinfeng, Pan jingcao
EPI_ISL_418507 hCoV-19/Hangzhou/HZ91/2020 Asia/China/Zhejiang/Hangzhou 2020-01-21 Hangzhou Center for Disease Control and Prevention Inspection Center of Hangzhou Center for Disease Control and Prevention Yu hua, Wang haoqiu, Li jun, Yu xinfeng, Pan jingcao
EPI_ISL_418508 hCoV-19/Hangzhou/HZ162/2020 Asia / China / Zhejiang / Hangzhou 2020-01-23 Hangzhou Center for Disease Control and Prevention Inspection Center of Hangzhou Center for Disease Control and Prevention Yu hua, Wang haoqiu, Li jun, Yu xinfeng, Pan jingcao
EPI_ISL_418509 hCoV-19/Hangzhou/HZ178/2020 Asia / China / Zhejiang / Hangzhou 2020-01-23 Hangzhou Center for Disease Control and Prevention Inspection Center of Hangzhou Center for Disease Control and Prevention Yu hua, Wang haoqiu, Li jun, Yu xinfeng, Pan jingcao
EPI_ISL_418510 hCoV-19/Hangzhou/HZ185/2020 Asia / China / Hangzhou 2020-01-23 Hangzhou Center for Disease Control and Prevention Insepction Center of Hangzhou Center for Disease Control and Prevention Yu hua, Wang haoqiu, Li jun, Yu xinfeng, Pan jingcao
EPI_ISL_418511 hCoV-19/Hangzhou/HZ477/2020 Asia / China / Zhejiang / Hangzhou 2020-01-24 Hangzhou Center for Disease Control and Prevention Inspection Center of Hangzhou Center for Disease Control and Prevention Yu hua, Wang haoqiu, Li jun, Yu xinfeng, Pan jingcao
EPI_ISL_418512 hCoV-19/Hangzhou/HZ481/2020 Asia / China / Zhejiang / Hangzhou 2020-01-25 Hangzhou Center for Disease Control and Prevention Inspection Center of Hangzhou Center for Disease Control and Prevention Yu hua, Wang haoqiu, Li jun, Yu xinfeng, Pan jingcao
EPI_ISL_418513 hCoV-19/Hangzhou/HZ551/2020 Asia / China / Hangzhou 2020-01-25 Hangzhou Center for Disease Control and Prevention Inspection Center of Hangzhou Center for Disease Control and Prevention Yu hua, Wang haoqiu, Li jun, Yu xinfeng, Pan jingcao
EPI_ISL_418514 hCoV-19/Hangzhou/HZ576/2020 Asia / China / Zhejiang / Hangzhou 2020-01-25 Hangzhou Center for Disease Control and Prevention Inspection Center of Hangzhou Center for Disease Control and Prevention Yu hua, Wang haoqiu, Li jun, Yu xinfeng, Pan jingcao
EPI_ISL_418515 hCoV-19/Hangzhou/HZ638/2020 Asia / China / Hangzhou 2020-01-25 Hangzhou Center for Disease Control and Prevention Inspection Center of Hangzhou Center for Disease Control and Prevention Yu hua, Wang haoqiu, Li jun, Yu xinfeng, Pan jingcao
EPI_ISL_418516 hCoV-19/Ireland/21023/2020 Europe / Ireland / Tipperary 2020-03-06 UCD National

Virus Reference Laboratory	UCD National Virus Reference Laboratory	Michael Carr, Gabriel Gonzalez, Jonathan Dean, Suzie Coughlan, Alison Murphy, Kevin Byrne, Ken Wolfe, Jeff Connell, Brendan Loftus, Cillian F De Gascun
EPI_ISL_418580	hCoV-19/Ireland/Dublin-22361/2020	Europe / Ireland / Cork	2020-03-08	UCD National Virus Reference Laboratory	UCD National Virus Reference Laboratory	Michael Carr, Gabriel Gonzalez, Jonathan Dean, Suzie Coughlan, Alison Murphy, Kevin Byrne, Ken Wolfe, Jeff Connell, Brendan Loftus, Cillian F De Gascun
EPI_ISL_418581	hCoV-19/Ireland/Dublin-22428/2020	Europe / Ireland / Dublin	2020-03-08	UCD National Virus Reference Laboratory	UCD National Virus Reference Laboratory	Michael Carr, Gabriel Gonzalez, Jonathan Dean, Suzie Coughlan, Alison Murphy, Kevin Byrne, Ken Wolfe, Jeff Connell, Brendan Loftus, Cillian F De Gascun
EPI_ISL_418582	hCoV-19/Ireland/22901/2020	Europe / Ireland / Louth	2020-03-10	UCD National Virus Reference Laboratory	UCD National Virus Reference Laboratory	Michael Carr, Gabriel Gonzalez, Jonathan Dean, Suzie Coughlan, Alison Murphy, Kevin Byrne, Ken Wolfe, Jeff Connell, Brendan Loftus, Cillian F De Gascun
EPI_ISL_418583	hCoV-19/Ireland/24042/2020	Europe / Ireland / Dublin	2020-03-10	UCD National Virus Reference Laboratory	UCD National Virus Reference Laboratory	Michael Carr, Gabriel Gonzalez, Jonathan Dean, Suzie Coughlan, Alison Murphy, Kevin Byrne, Ken Wolfe, Jeff Connell, Brendan Loftus, Cillian F De Gascun
EPI_ISL_418584	hCoV-19/Ireland/24052/2020	Europe / Ireland / Wicklow	2020-03-10	UCD National Virus Reference Laboratory	UCD National Virus Reference Laboratory	Michael Carr, Gabriel Gonzalez, Jonathan Dean, Suzie Coughlan, Alison Murphy, Kevin Byrne, Ken Wolfe, Jeff Connell, Brendan Loftus, Cillian F De Gascun
EPI_ISL_419700	hCoV-19/USA/NY-NYUMC39/2020	North America / USA / New York / Manhattan	2020-03-18	NYU Langone Health	Departments of Pathology and Medicine, New York University School of Medicine	Maria Aguero-Rosenfeld, Margaret Black, John Cadley, Paolo Cotzia, John Chen, Dacia Dimartino, Xiaojun Feng, Adriana Heguy, Megan Hogan, Emily Huang, George Jour, Christian Marier, Matthew T. Maurano, Mark J. Mulligan, Peter Meyn, Jared Pinnell, Sitharam Ramaswami, Amy Rapkiewicz, Marie Samanovic-Golden, Antonio Serrano, Guomiao Shen, Matija Snuderl, Nick Vulpescu, Gael Westby, Paul Zappile, Yutong Zhang
EPI_ISL_419702	hCoV-19/USA/NY-NYUMC41/2020	North America / USA / New York / Brooklyn	2020-03-18	NYU Langone Health	Departments of Pathology and Medicine, New York University School of Medicine	Maria Aguero-Rosenfeld, Margaret Black, John Cadley, Paolo Cotzia, John Chen, Dacia Dimartino, Xiaojun Feng, Adriana Heguy, Megan Hogan, Emily Huang, George Jour, Christian Marier, Matthew T. Maurano, Mark J. Mulligan, Peter Meyn, Jared Pinnell, Sitharam Ramaswami, Amy Rapkiewicz, Marie Samanovic-Golden, Antonio Serrano, Guomiao Shen, Matija Snuderl, Nick Vulpescu, Gael Westby, Paul Zappile, Yutong Zhang
EPI_ISL_419703	hCoV-19/USA/NY-NYUMC42/2020	North America / USA / New York / Manhattan	2020-03-18	NYU Langone Health	Departments of Pathology and Medicine, New York University School of Medicine	Maria Aguero-Rosenfeld, Margaret Black, John Cadley, Paolo Cotzia, John Chen, Dacia Dimartino, Xiaojun Feng, Adriana Heguy, Megan Hogan, Emily Huang, George Jour, Christian Marier, Matthew T. Maurano, Mark J. Mulligan, Peter Meyn, Jared Pinnell, Sitharam Ramaswami, Amy Rapkiewicz, Marie Samanovic-Golden, Antonio Serrano, Guomiao Shen, Matija Snuderl, Nick Vulpescu, Gael Westby, Paul Zappile, Yutong Zhang
EPI_ISL_419704	hCoV-19/USA/NY-NYUMC43/2020	North America / USA / New York / Manhattan	2020-03-18	NYU Langone Health	Departments of Pathology and Medicine, New York University School of Medicine	Maria Aguero-Rosenfeld, Margaret Black, John Cadley, Paolo Cotzia, John Chen, Dacia Dimartino, Xiaojun Feng, Adriana Heguy, Megan Hogan, Emily Huang, George Jour, Christian Marier, Matthew T. Maurano, Mark J. Mulligan, Peter Meyn, Jared Pinnell, Sitharam Ramaswami, Amy Rapkiewicz, Marie Samanovic-Golden, Antonio Serrano, Guomiao Shen, Matija Snuderl, Nick Vulpescu, Gael Westby, Paul Zappile, Yutong Zhang
EPI_ISL_419705	hCoV-19/USA/NY-NYUMC44/2020	North America / USA / New York / Manhattan	2020-03-18	NYU Langone Health	Departments of Pathology and Medicine, New York University School of Medicine	Maria Aguero-Rosenfeld, Margaret Black, John Cadley, Paolo Cotzia, John Chen, Dacia Dimartino, Xiaojun Feng, Adriana Heguy, Megan Hogan, Emily Huang, George Jour, Christian Marier, Matthew T. Maurano, Mark J. Mulligan, Peter Meyn, Jared Pinnell, Sitharam Ramaswami, Amy Rapkiewicz, Marie Samanovic-Golden, Antonio Serrano, Guomiao Shen, Matija Snuderl, Nick Vulpescu, Gael Westby, Paul Zappile, Yutong Zhang
EPI_ISL_419706	hCoV-19/USA/VA-DCLS-0012/2020	North America / USA / Virginia	2020-03-10	Virginia DCLS	Virginia DCLS	Virginia DCLS
EPI_ISL_419707	hCoV-19/Spain/Cataluna201397/2020	Europe / Spain / Catalonia	2020	HOSPITAL CLINIC	Instituto de Salud Carlos III	Iglesias-Caballero, M. Molinero Calamita, M. González-Esguevillas, M. Camarero S. Pozo F. Casas I. Jiménez, P. Jiménez, M. Zaballos, A. Monzón, S. Varona, S. Juliá, M. Cuesta, I. Marcos, M.A
EPI_ISL_419708	hCoV-19/USA/VA-DCLS-0014/2020	North America / USA / Virginia	2020-03-12	Virginia DCLS	Virginia DCLS	Virginia DCLS
EPI_ISL_419709	hCoV-19/Spain/PaisVasco201602/2020	Europe / Spain / BasqueCountry	2020-03-04	HOSPITAL TXAGORRITXU	Instituto de Salud Carlos III	Iglesias-Caballero, M. Molinero Calamita, M. González-Esguevillas, M. Camarero S. Pozo F. Casas I. Jiménez, P. Jiménez, M. Zaballos, A. Monzón, S. Varona, S. Juliá, M. Cuesta, I. Gómez, C.
EPI_ISL_419710	hCoV-19/USA/VA-DCLS-0016/2020	North America / USA / Virginia	2020-03-12	Virginia DCLS	Virginia DCLS	Virginia DCLS
EPI_ISL_419711	hCoV-19/USA/VA-DCLS-0017/2020	North America / USA / Virginia	2020-03-11	Virginia DCLS	Virginia DCLS	Virginia DCLS
EPI_ISL_419712	hCoV-19/USA/VA-DCLS-0020/2020	North America / USA / Virginia	2020-03-10	Virginia DCLS	Virginia DCLS	Virginia DCLS

EPI_ISL_419713 hCoV-19/USA/VA-DCLS-0021/2020 North America / USA / Virginia 2020-03-11 Virginia DCLS
Virginia DCLS Virginia DCLS
EPI_ISL_419714 hCoV-19/Australia/VIC108/2020 Oceania / Australia / Victoria 2020-03-18 Microbiological
Diagnostic Unit Public Health Laboratory Microbiological Diagnostic Unit Public Health Laboratory
Seemann T., Schultz M., Sait, M., Sherry, N.
EPI_ISL_419715 hCoV-19/Australia/VIC111/2020 Oceania / Australia / Victoria 2020-03-18 Microbiological
Diagnostic Unit Public Health Laboratory Microbiological Diagnostic Unit Public Health Laboratory
Seemann T., Schultz M., Sait, M., Sherry, N.
EPI_ISL_419716 hCoV-19/Australia/VIC110/2020 Oceania / Australia / Victoria 2020-03-18 Microbiological
Diagnostic Unit Public Health Laboratory Microbiological Diagnostic Unit Public Health Laboratory
Seemann T., Schultz M., Sait, M., Sherry, N.
EPI_ISL_419717 hCoV-19/Australia/VIC115/2020 Oceania / Australia / Victoria 2020-03-19 Microbiological
Diagnostic Unit Public Health Laboratory Microbiological Diagnostic Unit Public Health Laboratory
Seemann T., Schultz M., Sait, M., Sherry, N.
EPI_ISL_419718 hCoV-19/Australia/VIC109/2020 Oceania / Australia / Victoria 2020-03-18 Microbiological
Diagnostic Unit Public Health Laboratory Microbiological Diagnostic Unit Public Health Laboratory
Seemann T., Schultz M., Sait, M., Sherry, N.
EPI_ISL_419719 hCoV-19/Australia/VIC118/2020 Oceania / Australia / Victoria 2020-03-20 Microbiological
Diagnostic Unit Public Health Laboratory Microbiological Diagnostic Unit Public Health Laboratory
Seemann T., Schultz M., Sait, M., Sherry, N.
EPI_ISL_419720 hCoV-19/Australia/VIC107/2020 Oceania / Australia / Victoria 2020-03-17 Microbiological
Diagnostic Unit Public Health Laboratory Microbiological Diagnostic Unit Public Health Laboratory
Seemann T., Schultz M., Sait, M., Sherry, N.
EPI_ISL_419721 hCoV-19/Australia/VIC116/2020 Oceania / Australia / Victoria 2020-03-19 Microbiological
Diagnostic Unit Public Health Laboratory Microbiological Diagnostic Unit Public Health Laboratory
Seemann T., Schultz M., Sait, M., Sherry, N.
EPI_ISL_419722 hCoV-19/Australia/VIC120/2020 Oceania / Australia / Victoria 2020-03-19 Microbiological
Diagnostic Unit Public Health Laboratory Microbiological Diagnostic Unit Public Health Laboratory
Seemann T., Schultz M., Sait, M., Sherry, N.
EPI_ISL_419723 hCoV-19/Australia/VIC121/2020 Oceania / Australia / Victoria 2020-03-18 Microbiological
Diagnostic Unit Public Health Laboratory Microbiological Diagnostic Unit Public Health Laboratory
Seemann T., Schultz M., Sait, M., Sherry, N.
EPI_ISL_419725 hCoV-19/Australia/VIC114/2020 Oceania / Australia / Victoria 2020-03-18 Microbiological
Diagnostic Unit Public Health Laboratory Microbiological Diagnostic Unit Public Health Laboratory
Seemann T., Schultz M., Sait, M., Sherry, N.
EPI_ISL_419726 hCoV-19/Australia/VIC119/2020 Oceania / Australia / Victoria 2020-03-19 Microbiological
Diagnostic Unit Public Health Laboratory Microbiological Diagnostic Unit Public Health Laboratory
Seemann T., Schultz M., Sait, M., Sherry, N.
EPI_ISL_419727 hCoV-19/Australia/VIC117/2020 Oceania / Australia / Victoria 2020-03-19 Microbiological
Diagnostic Unit Public Health Laboratory Microbiological Diagnostic Unit Public Health Laboratory
Seemann T., Schultz M., Sait, M., Sherry, N.
EPI_ISL_419728 hCoV-19/Australia/VIC126/2020 Oceania / Australia / Victoria 2020-03-20 Microbiological
Diagnostic Unit Public Health Laboratory Microbiological Diagnostic Unit Public Health Laboratory
Seemann T., Schultz M., Sait, M., Sherry, N.
EPI_ISL_419729 hCoV-19/Australia/VIC127/2020 Oceania / Australia / Victoria 2020-03-20 Microbiological
Diagnostic Unit Public Health Laboratory Microbiological Diagnostic Unit Public Health Laboratory
Seemann T., Schultz M., Sait, M., Sherry, N.
EPI_ISL_419730 hCoV-19/Australia/VIC128/2020 Oceania / Australia / Victoria 2020-03-20 Microbiological
Diagnostic Unit Public Health Laboratory Microbiological Diagnostic Unit Public Health Laboratory
Seemann T., Schultz M., Sait, M., Sherry, N.
EPI_ISL_419731 hCoV-19/Australia/VIC123/2020 Oceania / Australia / Victoria 2020-03-20 Microbiological
Diagnostic Unit Public Health Laboratory Microbiological Diagnostic Unit Public Health Laboratory
Seemann T., Schultz M., Sait, M., Sherry, N.
EPI_ISL_419732 hCoV-19/Australia/VIC129/2020 Oceania / Australia / Victoria 2020-03-21 Microbiological
Diagnostic Unit Public Health Laboratory Microbiological Diagnostic Unit Public Health Laboratory
Seemann T., Schultz M., Sait, M., Sherry, N.
EPI_ISL_419733 hCoV-19/Australia/VIC13/2020 Oceania / Australia / Victoria 2020-01-31 Victorian
Infectious Diseases Reference Laboratory (VIDRL) Victorian Infectious Diseases Reference Laboratory and
Microbiological Diagnostic Unit Public Health Laboratory, Doherty Institute Caly L., Seemann T., Sait, M.,
Schultz M., Druce J., Sherry, N.
EPI_ISL_419736 hCoV-19/Australia/VIC17/2020 Oceania / Australia / Victoria 2020-03-07 Victorian
Infectious Diseases Reference Laboratory (VIDRL) Victorian Infectious Diseases Reference Laboratory and
Microbiological Diagnostic Unit Public Health Laboratory, Doherty Institute Caly L., Seemann T., Sait, M.,
Schultz M., Druce J., Sherry, N.
EPI_ISL_419737 hCoV-19/Australia/VIC18/2020 Oceania / Australia / Victoria 2020-03-07 Victorian
Infectious Diseases Reference Laboratory (VIDRL) Victorian Infectious Diseases Reference Laboratory and
Microbiological Diagnostic Unit Public Health Laboratory, Doherty Institute Caly L., Seemann T., Sait, M.,
Schultz M., Druce J., Sherry, N.
EPI_ISL_419739 hCoV-19/Australia/VIC24/2020 Oceania / Australia / Victoria 2020-03-09 Victorian
Infectious Diseases Reference Laboratory (VIDRL) Victorian Infectious Diseases Reference Laboratory and

Microbiological Diagnostic Unit Public Health Laboratory, Doherty Institute	Caly L., Seemann T., Sait, M., Schultz M., Druce J., Sherry, N.
EPI_ISL_419740	hCoV-19/Australia/VIC21/2020	Oceania / Australia / Victoria	2020-03-09	Victorian Infectious Diseases Reference Laboratory (VIDRL)	Victorian Infectious Diseases Reference Laboratory and Microbiological Diagnostic Unit Public Health Laboratory, Doherty Institute	Caly L., Seemann T., Sait, M., Schultz M., Druce J., Sherry, N.
EPI_ISL_419741	hCoV-19/Australia/VIC20/2020	Oceania / Australia / Victoria	2020-03-08	Victorian Infectious Diseases Reference Laboratory (VIDRL)	Victorian Infectious Diseases Reference Laboratory and Microbiological Diagnostic Unit Public Health Laboratory, Doherty Institute	Caly L., Seemann T., Sait, M., Schultz M., Druce J., Sherry, N.
EPI_ISL_419742	hCoV-19/Australia/VIC22/2020	Oceania / Australia / Victoria	2020-03-09	Victorian Infectious Diseases Reference Laboratory (VIDRL)	Victorian Infectious Diseases Reference Laboratory and Microbiological Diagnostic Unit Public Health Laboratory, Doherty Institute	Caly L., Seemann T., Sait, M., Schultz M., Druce J., Sherry, N.
EPI_ISL_419743	hCoV-19/Australia/VIC23/2020	Oceania / Australia / Victoria	2020-03-09	Victorian Infectious Diseases Reference Laboratory (VIDRL)	Victorian Infectious Diseases Reference Laboratory and Microbiological Diagnostic Unit Public Health Laboratory, Doherty Institute	Caly L., Seemann T., Sait, M., Schultz M., Druce J., Sherry, N.
EPI_ISL_419744	hCoV-19/Australia/VIC25/2020	Oceania / Australia / Victoria	2020-03-10	Victorian Infectious Diseases Reference Laboratory (VIDRL)	Victorian Infectious Diseases Reference Laboratory and Microbiological Diagnostic Unit Public Health Laboratory, Doherty Institute	Caly L., Seemann T., Sait, M., Schultz M., Druce J., Sherry, N.
EPI_ISL_419745	hCoV-19/Australia/VIC26/2020	Oceania / Australia / Victoria	2020-03-10	Victorian Infectious Diseases Reference Laboratory (VIDRL)	Victorian Infectious Diseases Reference Laboratory and Microbiological Diagnostic Unit Public Health Laboratory, Doherty Institute	Caly L., Seemann T., Sait, M., Schultz M., Druce J., Sherry, N.
EPI_ISL_419746	hCoV-19/Australia/VIC27/2020	Oceania / Australia / Victoria	2020-03-10	Victorian Infectious Diseases Reference Laboratory (VIDRL)	Victorian Infectious Diseases Reference Laboratory and Microbiological Diagnostic Unit Public Health Laboratory, Doherty Institute	Caly L., Seemann T., Sait, M., Schultz M., Druce J., Sherry, N.
EPI_ISL_419747	hCoV-19/Australia/VIC28/2020	Oceania / Australia / Victoria	2020-03-10	Victorian Infectious Diseases Reference Laboratory (VIDRL)	Victorian Infectious Diseases Reference Laboratory and Microbiological Diagnostic Unit Public Health Laboratory, Doherty Institute	Caly L., Seemann T., Sait, M., Schultz M., Druce J., Sherry, N.
EPI_ISL_419748	hCoV-19/Australia/VIC29/2020	Oceania / Australia / Victoria	2020-03-10	Victorian Infectious Diseases Reference Laboratory (VIDRL)	Victorian Infectious Diseases Reference Laboratory and Microbiological Diagnostic Unit Public Health Laboratory, Doherty Institute	Caly L., Seemann T., Sait, M., Schultz M., Druce J., Sherry, N.
EPI_ISL_419749	hCoV-19/Australia/VIC30/2020	Oceania / Australia / Victoria	2020-03-10	Victorian Infectious Diseases Reference Laboratory (VIDRL)	Victorian Infectious Diseases Reference Laboratory and Microbiological Diagnostic Unit Public Health Laboratory, Doherty Institute	Caly L., Seemann T., Sait, M., Schultz M., Druce J., Sherry, N.
EPI_ISL_419750	hCoV-19/Australia/VIC31/2020	Oceania / Australia / Victoria	2020-03-10	Victorian Infectious Diseases Reference Laboratory (VIDRL)	Victorian Infectious Diseases Reference Laboratory and Microbiological Diagnostic Unit Public Health Laboratory, Doherty Institute	Caly L., Seemann T., Sait, M., Schultz M., Druce J., Sherry, N.
EPI_ISL_419751	hCoV-19/Australia/VIC32/2020	Oceania / Australia / Victoria	2020-03-10	Victorian Infectious Diseases Reference Laboratory (VIDRL)	Victorian Infectious Diseases Reference Laboratory and Microbiological Diagnostic Unit Public Health Laboratory, Doherty Institute	Caly L., Seemann T., Sait, M., Schultz M., Druce J., Sherry, N.
EPI_ISL_419752	hCoV-19/Australia/VIC33/2020	Oceania / Australia / Victoria	2020-03-10	Victorian Infectious Diseases Reference Laboratory (VIDRL)	Victorian Infectious Diseases Reference Laboratory and Microbiological Diagnostic Unit Public Health Laboratory, Doherty Institute	Caly L., Seemann T., Sait, M., Schultz M., Druce J., Sherry, N.
EPI_ISL_419753	hCoV-19/Australia/VIC34/2020	Oceania / Australia / Victoria	2020-03-10	Victorian Infectious Diseases Reference Laboratory (VIDRL)	Victorian Infectious Diseases Reference Laboratory and Microbiological Diagnostic Unit Public Health Laboratory, Doherty Institute	Caly L., Seemann T., Sait, M., Schultz M., Druce J., Sherry, N.
EPI_ISL_419754	hCoV-19/Australia/VIC37/2020	Oceania / Australia / Victoria	2020-03-11	Victorian Infectious Diseases Reference Laboratory (VIDRL)	Victorian Infectious Diseases Reference Laboratory and Microbiological Diagnostic Unit Public Health Laboratory, Doherty Institute	Caly L., Seemann T., Sait, M., Schultz M., Druce J., Sherry, N.
EPI_ISL_419755	hCoV-19/Australia/VIC35/2020	Oceania / Australia / Victoria	2020-03-10	Victorian Infectious Diseases Reference Laboratory (VIDRL)	Victorian Infectious Diseases Reference Laboratory and Microbiological Diagnostic Unit Public Health Laboratory, Doherty Institute	Caly L., Seemann T., Sait, M., Schultz M., Druce J., Sherry, N.
EPI_ISL_419756	hCoV-19/Australia/VIC38/2020	Oceania / Australia / Victoria	2020-03-11	Victorian Infectious Diseases Reference Laboratory (VIDRL)	Victorian Infectious Diseases Reference Laboratory and Microbiological Diagnostic Unit Public Health Laboratory, Doherty Institute	Caly L., Seemann T., Sait, M., Schultz M., Druce J., Sherry, N.

EPI_ISL_419757 hCoV-19/Australia/VIC39/2020 Oceania / Australia / Victoria 2020-03-11 Victorian Infectious Diseases Reference Laboratory (VIDRL) Victorian Infectious Diseases Reference Laboratory and Microbiological Diagnostic Unit Public Health Laboratory, Doherty Institute Caly L., Seemann T., Sait, M., Schultz M., Druce J., Sherry, N.
EPI_ISL_419758 hCoV-19/Australia/VIC40/2020 Oceania / Australia / Victoria 2020-03-11 Victorian Infectious Diseases Reference Laboratory (VIDRL) Victorian Infectious Diseases Reference Laboratory and Microbiological Diagnostic Unit Public Health Laboratory, Doherty Institute Caly L., Seemann T., Sait, M., Schultz M., Druce J., Sherry, N.
EPI_ISL_419759 hCoV-19/Australia/VIC47/2020 Oceania / Australia / Victoria 2020-03-12 Victorian Infectious Diseases Reference Laboratory (VIDRL) Victorian Infectious Diseases Reference Laboratory and Microbiological Diagnostic Unit Public Health Laboratory, Doherty Institute Caly L., Seemann T., Sait, M., Schultz M., Druce J., Sherry, N.
EPI_ISL_419760 hCoV-19/Australia/VIC41/2020 Oceania / Australia / Victoria 2020-03-11 Victorian Infectious Diseases Reference Laboratory (VIDRL) Victorian Infectious Diseases Reference Laboratory and Microbiological Diagnostic Unit Public Health Laboratory, Doherty Institute Caly L., Seemann T., Sait, M., Schultz M., Druce J., Sherry, N.
EPI_ISL_419761 hCoV-19/Australia/VIC42/2020 Oceania / Australia / Victoria 2020-03-11 Victorian Infectious Diseases Reference Laboratory (VIDRL) Victorian Infectious Diseases Reference Laboratory and Microbiological Diagnostic Unit Public Health Laboratory, Doherty Institute Caly L., Seemann T., Sait, M., Schultz M., Druce J., Sherry, N.
EPI_ISL_419762 hCoV-19/Australia/VIC48/2020 Oceania / Australia / Victoria 2020-03-12 Victorian Infectious Diseases Reference Laboratory (VIDRL) Victorian Infectious Diseases Reference Laboratory and Microbiological Diagnostic Unit Public Health Laboratory, Doherty Institute Caly L., Seemann T., Sait, M., Schultz M., Druce J., Sherry, N.
EPI_ISL_419763 hCoV-19/Australia/VIC43/2020 Oceania / Australia / Victoria 2020-03-11 Victorian Infectious Diseases Reference Laboratory (VIDRL) Victorian Infectious Diseases Reference Laboratory and Microbiological Diagnostic Unit Public Health Laboratory, Doherty Institute Caly L., Seemann T., Sait, M., Schultz M., Druce J., Sherry, N.
EPI_ISL_419764 hCoV-19/Australia/VIC49/2020 Oceania / Australia / Victoria 2020-03-12 Victorian Infectious Diseases Reference Laboratory (VIDRL) Victorian Infectious Diseases Reference Laboratory and Microbiological Diagnostic Unit Public Health Laboratory, Doherty Institute Caly L., Seemann T., Sait, M., Schultz M., Druce J., Sherry, N.
EPI_ISL_419765 hCoV-19/Australia/VIC36/2020 Oceania / Australia / Victoria 2020-03-10 Victorian Infectious Diseases Reference Laboratory (VIDRL) Victorian Infectious Diseases Reference Laboratory and Microbiological Diagnostic Unit Public Health Laboratory, Doherty Institute Caly L., Seemann T., Sait, M., Schultz M., Druce J., Sherry, N.
EPI_ISL_419766 hCoV-19/Australia/VIC50/2020 Oceania / Australia / Victoria 2020-03-12 Victorian Infectious Diseases Reference Laboratory (VIDRL) Victorian Infectious Diseases Reference Laboratory and Microbiological Diagnostic Unit Public Health Laboratory, Doherty Institute Caly L., Seemann T., Sait, M., Schultz M., Druce J., Sherry, N.
EPI_ISL_419767 hCoV-19/Australia/VIC44/2020 Oceania / Australia / Victoria 2020-03-11 Victorian Infectious Diseases Reference Laboratory (VIDRL) Victorian Infectious Diseases Reference Laboratory and Microbiological Diagnostic Unit Public Health Laboratory, Doherty Institute Caly L., Seemann T., Sait, M., Schultz M., Druce J., Sherry, N.
EPI_ISL_419768 hCoV-19/Australia/VIC52/2020 Oceania / Australia / Victoria 2020-03-12 Victorian Infectious Diseases Reference Laboratory (VIDRL) Victorian Infectious Diseases Reference Laboratory and Microbiological Diagnostic Unit Public Health Laboratory, Doherty Institute Caly L., Seemann T., Sait, M., Schultz M., Druce J., Sherry, N.
EPI_ISL_419769 hCoV-19/Australia/VIC53/2020 Oceania / Australia / Victoria 2020-03-12 Victorian Infectious Diseases Reference Laboratory (VIDRL) Victorian Infectious Diseases Reference Laboratory and Microbiological Diagnostic Unit Public Health Laboratory, Doherty Institute Caly L., Seemann T., Sait, M., Schultz M., Druce J., Sherry, N.
EPI_ISL_419770 hCoV-19/Australia/VIC54/2020 Oceania / Australia / Victoria 2020-03-12 Victorian Infectious Diseases Reference Laboratory (VIDRL) Victorian Infectious Diseases Reference Laboratory and Microbiological Diagnostic Unit Public Health Laboratory, Doherty Institute Caly L., Seemann T., Sait, M., Schultz M., Druce J., Sherry, N.
EPI_ISL_419771 hCoV-19/Australia/VIC59/2020 Oceania / Australia / Victoria 2020-03-13 Victorian Infectious Diseases Reference Laboratory (VIDRL) Victorian Infectious Diseases Reference Laboratory and Microbiological Diagnostic Unit Public Health Laboratory, Doherty Institute Caly L., Seemann T., Sait, M., Schultz M., Druce J., Sherry, N.
EPI_ISL_419772 hCoV-19/Australia/VIC60/2020 Oceania / Australia / Victoria 2020-03-13 Victorian Infectious Diseases Reference Laboratory (VIDRL) Victorian Infectious Diseases Reference Laboratory and Microbiological Diagnostic Unit Public Health Laboratory, Doherty Institute Caly L., Seemann T., Sait, M., Schultz M., Druce J., Sherry, N.
EPI_ISL_419773 hCoV-19/Australia/VIC61/2020 Oceania / Australia / Victoria 2020-03-13 Victorian Infectious Diseases Reference Laboratory (VIDRL) Victorian Infectious Diseases Reference Laboratory and Microbiological Diagnostic Unit Public Health Laboratory, Doherty Institute Caly L., Seemann T., Sait, M., Schultz M., Druce J., Sherry, N.
EPI_ISL_419774 hCoV-19/Australia/VIC62/2020 Oceania / Australia / Victoria 2020-03-13 Victorian Infectious Diseases Reference Laboratory (VIDRL) Victorian Infectious Diseases Reference Laboratory and

Microbiological Diagnostic Unit Public Health Laboratory, Doherty Institute	Caly L., Seemann T., Sait, M., Schultz M., Druce J., Sherry, N.
EPI_ISL_419775	hCoV-19/Australia/VIC63/2020	Oceania / Australia / Victoria	2020-03-13	Victorian Infectious Diseases Reference Laboratory (VIDRL)	Victorian Infectious Diseases Reference Laboratory and Microbiological Diagnostic Unit Public Health Laboratory, Doherty Institute	Caly L., Seemann T., Sait, M., Schultz M., Druce J., Sherry, N.
EPI_ISL_419776	hCoV-19/Australia/VIC55/2020	Oceania / Australia / Victoria	2020-03-12	Victorian Infectious Diseases Reference Laboratory (VIDRL)	Victorian Infectious Diseases Reference Laboratory and Microbiological Diagnostic Unit Public Health Laboratory, Doherty Institute	Caly L., Seemann T., Sait, M., Schultz M., Druce J., Sherry, N.
EPI_ISL_419777	hCoV-19/Australia/VIC56/2020	Oceania / Australia / Victoria	2020-03-12	Victorian Infectious Diseases Reference Laboratory (VIDRL)	Victorian Infectious Diseases Reference Laboratory and Microbiological Diagnostic Unit Public Health Laboratory, Doherty Institute	Caly L., Seemann T., Sait, M., Schultz M., Druce J., Sherry, N.
EPI_ISL_419778	hCoV-19/Australia/VIC64/2020	Oceania / Australia / Victoria	2020-03-13	Victorian Infectious Diseases Reference Laboratory (VIDRL)	Victorian Infectious Diseases Reference Laboratory and Microbiological Diagnostic Unit Public Health Laboratory, Doherty Institute	Caly L., Seemann T., Sait, M., Schultz M., Druce J., Sherry, N.
EPI_ISL_419779	hCoV-19/Australia/VIC57/2020	Oceania / Australia / Victoria	2020-03-12	Victorian Infectious Diseases Reference Laboratory (VIDRL)	Victorian Infectious Diseases Reference Laboratory and Microbiological Diagnostic Unit Public Health Laboratory, Doherty Institute	Caly L., Seemann T., Sait, M., Schultz M., Druce J., Sherry, N.
EPI_ISL_419780	hCoV-19/Australia/VIC66/2020	Oceania / Australia / Victoria	2020-03-13	Victorian Infectious Diseases Reference Laboratory (VIDRL)	Victorian Infectious Diseases Reference Laboratory and Microbiological Diagnostic Unit Public Health Laboratory, Doherty Institute	Caly L., Seemann T., Sait, M., Schultz M., Druce J., Sherry, N.
EPI_ISL_419781	hCoV-19/Australia/VIC67/2020	Oceania / Australia / Victoria	2020-03-13	Victorian Infectious Diseases Reference Laboratory (VIDRL)	Victorian Infectious Diseases Reference Laboratory and Microbiological Diagnostic Unit Public Health Laboratory, Doherty Institute	Caly L., Seemann T., Sait, M., Schultz M., Druce J., Sherry, N.
EPI_ISL_419782	hCoV-19/Australia/VIC68/2020	Oceania / Australia / Victoria	2020-03-13	Victorian Infectious Diseases Reference Laboratory (VIDRL)	Victorian Infectious Diseases Reference Laboratory and Microbiological Diagnostic Unit Public Health Laboratory, Doherty Institute	Caly L., Seemann T., Sait, M., Schultz M., Druce J., Sherry, N.
EPI_ISL_419783	hCoV-19/Australia/VIC69/2020	Oceania / Australia / Victoria	2020-03-13	Victorian Infectious Diseases Reference Laboratory (VIDRL)	Victorian Infectious Diseases Reference Laboratory and Microbiological Diagnostic Unit Public Health Laboratory, Doherty Institute	Caly L., Seemann T., Sait, M., Schultz M., Druce J., Sherry, N.
EPI_ISL_419784	hCoV-19/Australia/VIC58/2020	Oceania / Australia / Victoria	2020-03-12	Victorian Infectious Diseases Reference Laboratory (VIDRL)	Victorian Infectious Diseases Reference Laboratory and Microbiological Diagnostic Unit Public Health Laboratory, Doherty Institute	Caly L., Seemann T., Sait, M., Schultz M., Druce J., Sherry, N.
EPI_ISL_419785	hCoV-19/Australia/VIC73/2020	Oceania / Australia / Victoria	2020-03-14	Victorian Infectious Diseases Reference Laboratory (VIDRL)	Victorian Infectious Diseases Reference Laboratory and Microbiological Diagnostic Unit Public Health Laboratory, Doherty Institute	Caly L., Seemann T., Sait, M., Schultz M., Druce J., Sherry, N.
EPI_ISL_419786	hCoV-19/Australia/VIC70/2020	Oceania / Australia / Victoria	2020-03-13	Victorian Infectious Diseases Reference Laboratory (VIDRL)	Victorian Infectious Diseases Reference Laboratory and Microbiological Diagnostic Unit Public Health Laboratory, Doherty Institute	Caly L., Seemann T., Sait, M., Schultz M., Druce J., Sherry, N.
EPI_ISL_419787	hCoV-19/Australia/VIC75/2020	Oceania / Australia / Victoria	2020-03-14	Victorian Infectious Diseases Reference Laboratory (VIDRL)	Victorian Infectious Diseases Reference Laboratory and Microbiological Diagnostic Unit Public Health Laboratory, Doherty Institute	Caly L., Seemann T., Sait, M., Schultz M., Druce J., Sherry, N.
EPI_ISL_419788	hCoV-19/Australia/VIC76/2020	Oceania / Australia / Victoria	2020-03-14	Victorian Infectious Diseases Reference Laboratory (VIDRL)	Victorian Infectious Diseases Reference Laboratory and Microbiological Diagnostic Unit Public Health Laboratory, Doherty Institute	Caly L., Seemann T., Sait, M., Schultz M., Druce J., Sherry, N.
EPI_ISL_419789	hCoV-19/Australia/VIC77/2020	Oceania / Australia / Victoria	2020-03-14	Victorian Infectious Diseases Reference Laboratory (VIDRL)	Victorian Infectious Diseases Reference Laboratory and Microbiological Diagnostic Unit Public Health Laboratory, Doherty Institute	Caly L., Seemann T., Sait, M., Schultz M., Druce J., Sherry, N.
EPI_ISL_419790	hCoV-19/Australia/VIC78/2020	Oceania / Australia / Victoria	2020-03-14	Victorian Infectious Diseases Reference Laboratory (VIDRL)	Victorian Infectious Diseases Reference Laboratory and Microbiological Diagnostic Unit Public Health Laboratory, Doherty Institute	Caly L., Seemann T., Sait, M., Schultz M., Druce J., Sherry, N.
EPI_ISL_419791	hCoV-19/Australia/VIC79/2020	Oceania / Australia / Victoria	2020-03-14	Victorian Infectious Diseases Reference Laboratory (VIDRL)	Victorian Infectious Diseases Reference Laboratory and Microbiological Diagnostic Unit Public Health Laboratory, Doherty Institute	Caly L., Seemann T., Sait, M., Schultz M., Druce J., Sherry, N.

EPI_ISL_419792 hCoV-19/Australia/VIC80/2020 Oceania / Australia / Victoria 2020-03-14 Victorian Infectious Diseases Reference Laboratory (VIDRL) Victorian Infectious Diseases Reference Laboratory and Microbiological Diagnostic Unit Public Health Laboratory, Doherty Institute Caly L., Seemann T., Sait, M., Schultz M., Druce J., Sherry, N.
EPI_ISL_419793 hCoV-19/Australia/VIC81/2020 Oceania / Australia / Victoria 2020-03-14 Victorian Infectious Diseases Reference Laboratory (VIDRL) Victorian Infectious Diseases Reference Laboratory and Microbiological Diagnostic Unit Public Health Laboratory, Doherty Institute Caly L., Seemann T., Sait, M., Schultz M., Druce J., Sherry, N.
EPI_ISL_419794 hCoV-19/Australia/VIC82/2020 Oceania / Australia / Victoria 2020-03-14 Victorian Infectious Diseases Reference Laboratory (VIDRL) Victorian Infectious Diseases Reference Laboratory and Microbiological Diagnostic Unit Public Health Laboratory, Doherty Institute Caly L., Seemann T., Sait, M., Schultz M., Druce J., Sherry, N.
EPI_ISL_419795 hCoV-19/Australia/VIC85/2020 Oceania / Australia / Victoria 2020-03-15 Victorian Infectious Diseases Reference Laboratory (VIDRL) Victorian Infectious Diseases Reference Laboratory and Microbiological Diagnostic Unit Public Health Laboratory, Doherty Institute Caly L., Seemann T., Sait, M., Schultz M., Druce J., Sherry, N.
EPI_ISL_419796 hCoV-19/Australia/VIC86/2020 Oceania / Australia / Victoria 2020-03-15 Victorian Infectious Diseases Reference Laboratory (VIDRL) Victorian Infectious Diseases Reference Laboratory and Microbiological Diagnostic Unit Public Health Laboratory, Doherty Institute Caly L., Seemann T., Sait, M., Schultz M., Druce J., Sherry, N.
EPI_ISL_419797 hCoV-19/Australia/VIC87/2020 Oceania / Australia / Victoria 2020-03-15 Victorian Infectious Diseases Reference Laboratory (VIDRL) Victorian Infectious Diseases Reference Laboratory and Microbiological Diagnostic Unit Public Health Laboratory, Doherty Institute Caly L., Seemann T., Sait, M., Schultz M., Druce J., Sherry, N.
EPI_ISL_419798 hCoV-19/Australia/VIC88/2020 Oceania / Australia / Victoria 2020-03-15 Victorian Infectious Diseases Reference Laboratory (VIDRL) Victorian Infectious Diseases Reference Laboratory and Microbiological Diagnostic Unit Public Health Laboratory, Doherty Institute Caly L., Seemann T., Sait, M., Schultz M., Druce J., Sherry, N.
EPI_ISL_419829 hCoV-19/Australia/VIC136/2020 Oceania / Australia / Victoria 2020-03-22 Microbiological Diagnostic Unit Public Health Laboratory Microbiological Diagnostic Unit Public Health Laboratory Seemann T., Schultz M., Sait, M., Sherry, N.
EPI_ISL_419830 hCoV-19/Australia/VIC137/2020 Oceania / Australia / Victoria 2020-03-22 Microbiological Diagnostic Unit Public Health Laboratory Microbiological Diagnostic Unit Public Health Laboratory Seemann T., Schultz M., Sait, M., Sherry, N.
EPI_ISL_419831 hCoV-19/Australia/NT01/2020 Oceania / Australia / Northern Territory 2020-02-21 Royal Darwin Hospital Victorian Infectious Diseases Reference Laboratory and Microbiological Diagnostic Unit Public Health Laboratory, Doherty Institute Meumann, E., Seemann T., Sait, M., Schultz M., Caly L., Druce J.
EPI_ISL_419832 hCoV-19/Australia/NT02/2020 Oceania / Australia / Northern Territory 2020-02-21 Royal Darwin Hospital Victorian Infectious Diseases Reference Laboratory and Microbiological Diagnostic Unit Public Health Laboratory, Doherty Institute Meumann, E., Seemann T., Sait, M., Schultz M., Caly L., Druce J.
EPI_ISL_419834 hCoV-19/Australia/VIC138/2020 Oceania / Australia / Victoria 2020-02-23 Victorian Infectious Diseases Reference Laboratory (VIDRL) Victorian Infectious Diseases Reference Laboratory and Microbiological Diagnostic Unit Public Health Laboratory, Doherty Institute Caly L., Seemann T., Sait, M., Schultz M., Druce J., Sherry, N.
EPI_ISL_419835 hCoV-19/Australia/NT05/2020 Oceania / Australia / Northern Territory 2020-02-24 Royal Darwin Hospital Victorian Infectious Diseases Reference Laboratory and Microbiological Diagnostic Unit Public Health Laboratory, Doherty Institute Meumann, E., Seemann T., Sait, M., Schultz M., Caly L., Druce J.
EPI_ISL_419836 hCoV-19/Australia/VIC139/2020 Oceania / Australia / Victoria 2020-03-15 Victorian Infectious Diseases Reference Laboratory (VIDRL) Victorian Infectious Diseases Reference Laboratory and Microbiological Diagnostic Unit Public Health Laboratory, Doherty Institute Caly L., Seemann T., Sait, M., Schultz M., Druce J., Sherry, N.
EPI_ISL_419837 hCoV-19/Australia/VIC140/2020 Oceania / Australia / Victoria 2020-03-16 Victorian Infectious Diseases Reference Laboratory (VIDRL) Victorian Infectious Diseases Reference Laboratory and Microbiological Diagnostic Unit Public Health Laboratory, Doherty Institute Caly L., Seemann T., Sait, M., Schultz M., Druce J., Sherry, N.
EPI_ISL_419838 hCoV-19/Australia/VIC141/2020 Oceania / Australia / Victoria 2020-03-16 Victorian Infectious Diseases Reference Laboratory (VIDRL) Victorian Infectious Diseases Reference Laboratory and Microbiological Diagnostic Unit Public Health Laboratory, Doherty Institute Caly L., Seemann T., Sait, M., Schultz M., Druce J., Sherry, N.
EPI_ISL_419839 hCoV-19/Australia/VIC142/2020 Oceania / Australia / Victoria 2020-03-16 Victorian Infectious Diseases Reference Laboratory (VIDRL) Victorian Infectious Diseases Reference Laboratory and Microbiological Diagnostic Unit Public Health Laboratory, Doherty Institute Caly L., Seemann T., Sait, M., Schultz M., Druce J., Sherry, N.
EPI_ISL_419840 hCoV-19/Australia/VIC143/2020 Oceania / Australia / Victoria 2020-03-16 Victorian Infectious Diseases Reference Laboratory (VIDRL) Victorian Infectious Diseases Reference Laboratory and Microbiological Diagnostic Unit Public Health Laboratory, Doherty Institute Caly L., Seemann T., Sait, M., Schultz M., Druce J., Sherry, N.

EPI_ISL_419841 hCoV-19/Australia/VIC144/2020 Oceania / Australia / Victoria 2020-03-17 Victorian Infectious Diseases Reference Laboratory (VIDRL) Victorian Infectious Diseases Reference Laboratory and Microbiological Diagnostic Unit Public Health Laboratory, Doherty Institute Caly L., Seemann T., Sait, M., Schultz M., Druce J., Sherry, N.
EPI_ISL_419842 hCoV-19/Australia/VIC145/2020 Oceania / Australia / Victoria 2020-03-17 Victorian Infectious Diseases Reference Laboratory (VIDRL) Victorian Infectious Diseases Reference Laboratory and Microbiological Diagnostic Unit Public Health Laboratory, Doherty Institute Caly L., Seemann T., Sait, M., Schultz M., Druce J., Sherry, N.
EPI_ISL_419843 hCoV-19/Australia/VIC146/2020 Oceania / Australia / Victoria 2020-03-17 Victorian Infectious Diseases Reference Laboratory (VIDRL) Victorian Infectious Diseases Reference Laboratory and Microbiological Diagnostic Unit Public Health Laboratory, Doherty Institute Caly L., Seemann T., Sait, M., Schultz M., Druce J., Sherry, N.
EPI_ISL_419844 hCoV-19/Australia/VIC147/2020 Oceania / Australia / Victoria 2020-03-17 Victorian Infectious Diseases Reference Laboratory (VIDRL) Victorian Infectious Diseases Reference Laboratory and Microbiological Diagnostic Unit Public Health Laboratory, Doherty Institute Caly L., Seemann T., Sait, M., Schultz M., Druce J., Sherry, N.
EPI_ISL_419845 hCoV-19/Australia/VIC148/2020 Oceania / Australia / Victoria 2020-03-17 Victorian Infectious Diseases Reference Laboratory (VIDRL) Victorian Infectious Diseases Reference Laboratory and Microbiological Diagnostic Unit Public Health Laboratory, Doherty Institute Caly L., Seemann T., Sait, M., Schultz M., Druce J., Sherry, N.
EPI_ISL_419846 hCoV-19/Australia/VIC149/2020 Oceania / Australia / Victoria 2020-03-17 Victorian Infectious Diseases Reference Laboratory (VIDRL) Victorian Infectious Diseases Reference Laboratory and Microbiological Diagnostic Unit Public Health Laboratory, Doherty Institute Caly L., Seemann T., Sait, M., Schultz M., Druce J., Sherry, N.
EPI_ISL_419847 hCoV-19/Australia/VIC150/2020 Oceania / Australia / Victoria 2020-03-17 Victorian Infectious Diseases Reference Laboratory (VIDRL) Victorian Infectious Diseases Reference Laboratory and Microbiological Diagnostic Unit Public Health Laboratory, Doherty Institute Caly L., Seemann T., Sait, M., Schultz M., Druce J., Sherry, N.
EPI_ISL_419848 hCoV-19/Australia/VIC151/2020 Oceania / Australia / Victoria 2020-03-17 Victorian Infectious Diseases Reference Laboratory (VIDRL) Victorian Infectious Diseases Reference Laboratory and Microbiological Diagnostic Unit Public Health Laboratory, Doherty Institute Caly L., Seemann T., Sait, M., Schultz M., Druce J., Sherry, N.
EPI_ISL_419849 hCoV-19/Australia/VIC152/2020 Oceania / Australia / Victoria 2020-03-17 Victorian Infectious Diseases Reference Laboratory (VIDRL) Victorian Infectious Diseases Reference Laboratory and Microbiological Diagnostic Unit Public Health Laboratory, Doherty Institute Caly L., Seemann T., Sait, M., Schultz M., Druce J., Sherry, N.
EPI_ISL_420714 hCoV-19/England/20139055204/2020 Europe / United Kingdom / England 2020-03-28 Respiratory Virus Unit, Microbiology Services Colindale, Public Health England Respiratory Virus Unit, Microbiology Services Colindale, Public Health England Monica Galiano, Shahjahan Miah, Angie Lackenby, Omolola Akinbami, Tiina Talts, Leena Bhaw, Richard Myers, Steven Platt, Kirstin Edwards, Jonathan Hubb, Joanna Ellis, Maria Zambon
EPI_ISL_420715 hCoV-19/England/20139055304/2020 Europe / United Kingdom / England 2020-03-29 Respiratory Virus Unit, Microbiology Services Colindale, Public Health England Respiratory Virus Unit, Microbiology Services Colindale, Public Health England Monica Galiano, Shahjahan Miah, Angie Lackenby, Omolola Akinbami, Tiina Talts, Leena Bhaw, Richard Myers, Steven Platt, Kirstin Edwards, Jonathan Hubb, Joanna Ellis, Maria Zambon
EPI_ISL_420716 hCoV-19/England/20139056904/2020 Europe / United Kingdom / England 2020-03-26 Respiratory Virus Unit, Microbiology Services Colindale, Public Health England Respiratory Virus Unit, Microbiology Services Colindale, Public Health England Monica Galiano, Shahjahan Miah, Angie Lackenby, Omolola Akinbami, Tiina Talts, Leena Bhaw, Richard Myers, Steven Platt, Kirstin Edwards, Jonathan Hubb, Joanna Ellis, Maria Zambon
EPI_ISL_420717 hCoV-19/England/20139057004/2020 Europe / United Kingdom / England 2020-03-26 Respiratory Virus Unit, Microbiology Services Colindale, Public Health England Respiratory Virus Unit, Microbiology Services Colindale, Public Health England Monica Galiano, Shahjahan Miah, Angie Lackenby, Omolola Akinbami, Tiina Talts, Leena Bhaw, Richard Myers, Steven Platt, Kirstin Edwards, Jonathan Hubb, Joanna Ellis, Maria Zambon
EPI_ISL_420718 hCoV-19/England/20139057204/2020 Europe / United Kingdom / England 2020-03-27 Respiratory Virus Unit, Microbiology Services Colindale, Public Health England Respiratory Virus Unit, Microbiology Services Colindale, Public Health England Monica Galiano, Shahjahan Miah, Angie Lackenby, Omolola Akinbami, Tiina Talts, Leena Bhaw, Richard Myers, Steven Platt, Kirstin Edwards, Jonathan Hubb, Joanna Ellis, Maria Zambon
EPI_ISL_420719 hCoV-19/England/20139057404/2020 Europe / United Kingdom / England 2020-03-27 Respiratory Virus Unit, Microbiology Services Colindale, Public Health England Respiratory Virus Unit, Microbiology Services Colindale, Public Health England Monica Galiano, Shahjahan Miah, Angie Lackenby, Omolola Akinbami, Tiina Talts, Leena Bhaw, Richard Myers, Steven Platt, Kirstin Edwards, Jonathan Hubb, Joanna Ellis, Maria Zambon
EPI_ISL_420720 hCoV-19/England/20139057504/2020 Europe / United Kingdom / England 2020-03-28 Respiratory Virus Unit, Microbiology Services Colindale, Public Health England Respiratory Virus Unit, Microbiology Services Colindale, Public Health England Monica Galiano, Shahjahan Miah, Angie Lackenby, Omolola Akinbami, Tiina Talts, Leena Bhaw, Richard Myers, Steven Platt, Kirstin Edwards, Jonathan Hubb, Joanna Ellis,

Maria Zambon
EPI_ISL_420721 hCoV-19/England/20139057604/2020 Europe / United Kingdom / England 2020-03-28 Respiratory Virus Unit, Microbiology Services Colindale, Public Health England Respiratory Virus Unit, Microbiology Services Colindale, Public Health England Monica Galiano, Shahjahan Miah, Angie Lackenby, Omolola Akinbami, Tiina Talts, Leena Bhaw, Richard Myers, Steven Platt, Kirstin Edwards, Jonathan Hubb, Joanna Ellis, Maria Zambon
EPI_ISL_420722 hCoV-19/England/20139057704/2020 Europe / United Kingdom / England 2020-03-28 Respiratory Virus Unit, Microbiology Services Colindale, Public Health England Respiratory Virus Unit, Microbiology Services Colindale, Public Health England Monica Galiano, Shahjahan Miah, Angie Lackenby, Omolola Akinbami, Tiina Talts, Leena Bhaw, Richard Myers, Steven Platt, Kirstin Edwards, Jonathan Hubb, Joanna Ellis, Maria Zambon
EPI_ISL_420723 hCoV-19/England/20139058104/2020 Europe / United Kingdom / England 2020-03-28 Respiratory Virus Unit, Microbiology Services Colindale, Public Health England Respiratory Virus Unit, Microbiology Services Colindale, Public Health England Monica Galiano, Shahjahan Miah, Angie Lackenby, Omolola Akinbami, Tiina Talts, Leena Bhaw, Richard Myers, Steven Platt, Kirstin Edwards, Jonathan Hubb, Joanna Ellis, Maria Zambon
EPI_ISL_420724 hCoV-19/England/20139058304/2020 Europe / United Kingdom / England 2020-03-27 Respiratory Virus Unit, Microbiology Services Colindale, Public Health England Respiratory Virus Unit, Microbiology Services Colindale, Public Health England Monica Galiano, Shahjahan Miah, Angie Lackenby, Omolola Akinbami, Tiina Talts, Leena Bhaw, Richard Myers, Steven Platt, Kirstin Edwards, Jonathan Hubb, Joanna Ellis, Maria Zambon
EPI_ISL_420727 hCoV-19/England/20139059004/2020 Europe / United Kingdom / England 2020-03-28 Respiratory Virus Unit, Microbiology Services Colindale, Public Health England Respiratory Virus Unit, Microbiology Services Colindale, Public Health England Monica Galiano, Shahjahan Miah, Angie Lackenby, Omolola Akinbami, Tiina Talts, Leena Bhaw, Richard Myers, Steven Platt, Kirstin Edwards, Jonathan Hubb, Joanna Ellis, Maria Zambon
EPI_ISL_420728 hCoV-19/England/20139059504/2020 Europe / United Kingdom / England 2020-03-28 Respiratory Virus Unit, Microbiology Services Colindale, Public Health England Respiratory Virus Unit, Microbiology Services Colindale, Public Health England Monica Galiano, Shahjahan Miah, Angie Lackenby, Omolola Akinbami, Tiina Talts, Leena Bhaw, Richard Myers, Steven Platt, Kirstin Edwards, Jonathan Hubb, Joanna Ellis, Maria Zambon
EPI_ISL_420729 hCoV-19/England/20139059804/2020 Europe / United Kingdom / England 2020-03-28 Respiratory Virus Unit, Microbiology Services Colindale, Public Health England Respiratory Virus Unit, Microbiology Services Colindale, Public Health England Monica Galiano, Shahjahan Miah, Angie Lackenby, Omolola Akinbami, Tiina Talts, Leena Bhaw, Richard Myers, Steven Platt, Kirstin Edwards, Jonathan Hubb, Joanna Ellis, Maria Zambon
EPI_ISL_420730 hCoV-19/England/20139059904/2020 Europe / United Kingdom / England 2020-03-27 Respiratory Virus Unit, Microbiology Services Colindale, Public Health England Respiratory Virus Unit, Microbiology Services Colindale, Public Health England Monica Galiano, Shahjahan Miah, Angie Lackenby, Omolola Akinbami, Tiina Talts, Leena Bhaw, Richard Myers, Steven Platt, Kirstin Edwards, Jonathan Hubb, Joanna Ellis, Maria Zambon
EPI_ISL_420731 hCoV-19/England/20139060004/2020 Europe / United Kingdom / England 2020-03-28 Respiratory Virus Unit, Microbiology Services Colindale, Public Health England Respiratory Virus Unit, Microbiology Services Colindale, Public Health England Monica Galiano, Shahjahan Miah, Angie Lackenby, Omolola Akinbami, Tiina Talts, Leena Bhaw, Richard Myers, Steven Platt, Kirstin Edwards, Jonathan Hubb, Joanna Ellis, Maria Zambon
EPI_ISL_420732 hCoV-19/England/20139060404/2020 Europe / United Kingdom / England 2020-03-28 Respiratory Virus Unit, Microbiology Services Colindale, Public Health England Respiratory Virus Unit, Microbiology Services Colindale, Public Health England Monica Galiano, Shahjahan Miah, Angie Lackenby, Omolola Akinbami, Tiina Talts, Leena Bhaw, Richard Myers, Steven Platt, Kirstin Edwards, Jonathan Hubb, Joanna Ellis, Maria Zambon
EPI_ISL_420733 hCoV-19/England/20139060504/2020 Europe / United Kingdom / England 2020-03-28 Respiratory Virus Unit, Microbiology Services Colindale, Public Health England Respiratory Virus Unit, Microbiology Services Colindale, Public Health England Monica Galiano, Shahjahan Miah, Angie Lackenby, Omolola Akinbami, Tiina Talts, Leena Bhaw, Richard Myers, Steven Platt, Kirstin Edwards, Jonathan Hubb, Joanna Ellis, Maria Zambon
EPI_ISL_420734 hCoV-19/England/20139060804/2020 Europe / United Kingdom / England 2020-03-27 Respiratory Virus Unit, Microbiology Services Colindale, Public Health England Respiratory Virus Unit, Microbiology Services Colindale, Public Health England Monica Galiano, Shahjahan Miah, Angie Lackenby, Omolola Akinbami, Tiina Talts, Leena Bhaw, Richard Myers, Steven Platt, Kirstin Edwards, Jonathan Hubb, Joanna Ellis, Maria Zambon
EPI_ISL_420735 hCoV-19/England/20139061104/2020 Europe / United Kingdom / England 2020-03-27 Respiratory Virus Unit, Microbiology Services Colindale, Public Health England Respiratory Virus Unit, Microbiology Services Colindale, Public Health England Monica Galiano, Shahjahan Miah, Angie Lackenby, Omolola Akinbami, Tiina Talts, Leena Bhaw, Richard Myers, Steven Platt, Kirstin Edwards, Jonathan Hubb, Joanna Ellis, Maria Zambon
EPI_ISL_420741 hCoV-19/England/20139062504/2020 Europe / United Kingdom / England 2020-03-27 Respiratory Virus Unit, Microbiology Services Colindale, Public Health England Respiratory Virus Unit, Microbiology Services Colindale, Public Health England Monica Galiano, Shahjahan Miah, Angie Lackenby, Omolola Akinbami, Tiina Talts, Leena Bhaw, Richard Myers, Steven Platt, Kirstin Edwards, Jonathan Hubb, Joanna Ellis,

Maria Zambon
EPI_ISL_420742 hCoV-19/England/20139062704/2020   Europe / United Kingdom / England   2020-03-28
Respiratory Virus Unit, Microbiology Services Colindale, Public Health England Respiratory Virus Unit, Microbiology Services Colindale, Public Health England Monica Galiano, Shahjahan Miah, Angie Lackenby, Omolola Akinbami, Tiina Talts, Leena Bhaw, Richard Myers, Steven Platt, Kirstin Edwards, Jonathan Hubb, Joanna Ellis, Maria Zambon
EPI_ISL_420743 hCoV-19/England/20139062804/2020   Europe / United Kingdom / England   2020-03-27
Respiratory Virus Unit, Microbiology Services Colindale, Public Health England Respiratory Virus Unit, Microbiology Services Colindale, Public Health England Monica Galiano, Shahjahan Miah, Angie Lackenby, Omolola Akinbami, Tiina Talts, Leena Bhaw, Richard Myers, Steven Platt, Kirstin Edwards, Jonathan Hubb, Joanna Ellis, Maria Zambon
EPI_ISL_420744 hCoV-19/England/20139063204/2020   Europe / United Kingdom / England   2020-03-28
Respiratory Virus Unit, Microbiology Services Colindale, Public Health England Respiratory Virus Unit, Microbiology Services Colindale, Public Health England Monica Galiano, Shahjahan Miah, Angie Lackenby, Omolola Akinbami, Tiina Talts, Leena Bhaw, Richard Myers, Steven Platt, Kirstin Edwards, Jonathan Hubb, Joanna Ellis, Maria Zambon
EPI_ISL_420745 hCoV-19/England/20139063504/2020   Europe / United Kingdom / England   2020-03-28
Respiratory Virus Unit, Microbiology Services Colindale, Public Health England Respiratory Virus Unit, Microbiology Services Colindale, Public Health England Monica Galiano, Shahjahan Miah, Angie Lackenby, Omolola Akinbami, Tiina Talts, Leena Bhaw, Richard Myers, Steven Platt, Kirstin Edwards, Jonathan Hubb, Joanna Ellis, Maria Zambon
EPI_ISL_420746 hCoV-19/England/20139063604/2020   Europe / United Kingdom / England   2020-03-25
Respiratory Virus Unit, Microbiology Services Colindale, Public Health England Respiratory Virus Unit, Microbiology Services Colindale, Public Health England Monica Galiano, Shahjahan Miah, Angie Lackenby, Omolola Akinbami, Tiina Talts, Leena Bhaw, Richard Myers, Steven Platt, Kirstin Edwards, Jonathan Hubb, Joanna Ellis, Maria Zambon
EPI_ISL_420747 hCoV-19/England/20139063704/2020   Europe / United Kingdom / England   2020-03-28
Respiratory Virus Unit, Microbiology Services Colindale, Public Health England Respiratory Virus Unit, Microbiology Services Colindale, Public Health England Monica Galiano, Shahjahan Miah, Angie Lackenby, Omolola Akinbami, Tiina Talts, Leena Bhaw, Richard Myers, Steven Platt, Kirstin Edwards, Jonathan Hubb, Joanna Ellis, Maria Zambon
EPI_ISL_420748 hCoV-19/England/20139063804/2020   Europe / United Kingdom / England   2020-03-28
Respiratory Virus Unit, Microbiology Services Colindale, Public Health England Respiratory Virus Unit, Microbiology Services Colindale, Public Health England Monica Galiano, Shahjahan Miah, Angie Lackenby, Omolola Akinbami, Tiina Talts, Leena Bhaw, Richard Myers, Steven Platt, Kirstin Edwards, Jonathan Hubb, Joanna Ellis, Maria Zambon
EPI_ISL_420749 hCoV-19/England/20140037504/2020   Europe / United Kingdom / England   2020-03-25
Respiratory Virus Unit, Microbiology Services Colindale, Public Health England Respiratory Virus Unit, Microbiology Services Colindale, Public Health England Monica Galiano, Shahjahan Miah, Angie Lackenby, Omolola Akinbami, Tiina Talts, Leena Bhaw, Richard Myers, Steven Platt, Kirstin Edwards, Jonathan Hubb, Joanna Ellis, Maria Zambon
EPI_ISL_420750 hCoV-19/England/20140038604/2020   Europe / United Kingdom / England   2020-03-23
Respiratory Virus Unit, Microbiology Services Colindale, Public Health England Respiratory Virus Unit, Microbiology Services Colindale, Public Health England Monica Galiano, Shahjahan Miah, Angie Lackenby, Omolola Akinbami, Tiina Talts, Leena Bhaw, Richard Myers, Steven Platt, Kirstin Edwards, Jonathan Hubb, Joanna Ellis, Maria Zambon
EPI_ISL_420751 hCoV-19/England/20140038704/2020   Europe / United Kingdom / England   2020-03-30
Respiratory Virus Unit, Microbiology Services Colindale, Public Health England Respiratory Virus Unit, Microbiology Services Colindale, Public Health England Monica Galiano, Shahjahan Miah, Angie Lackenby, Omolola Akinbami, Tiina Talts, Leena Bhaw, Richard Myers, Steven Platt, Kirstin Edwards, Jonathan Hubb, Joanna Ellis, Maria Zambon
EPI_ISL_420752 hCoV-19/England/20140039004/2020   Europe / United Kingdom / England   2020-03-26
Respiratory Virus Unit, Microbiology Services Colindale, Public Health England Respiratory Virus Unit, Microbiology Services Colindale, Public Health England Monica Galiano, Shahjahan Miah, Angie Lackenby, Omolola Akinbami, Tiina Talts, Leena Bhaw, Richard Myers, Steven Platt, Kirstin Edwards, Jonathan Hubb, Joanna Ellis, Maria Zambon
EPI_ISL_420753 hCoV-19/England/20140039104/2020   Europe / United Kingdom / England   2020-03-25
Respiratory Virus Unit, Microbiology Services Colindale, Public Health England Respiratory Virus Unit, Microbiology Services Colindale, Public Health England Monica Galiano, Shahjahan Miah, Angie Lackenby, Omolola Akinbami, Tiina Talts, Leena Bhaw, Richard Myers, Steven Platt, Kirstin Edwards, Jonathan Hubb, Joanna Ellis, Maria Zambon
EPI_ISL_420755 hCoV-19/England/20140039304/2020   Europe / United Kingdom / England   2020-03-27
Respiratory Virus Unit, Microbiology Services Colindale, Public Health England Respiratory Virus Unit, Microbiology Services Colindale, Public Health England Monica Galiano, Shahjahan Miah, Angie Lackenby, Omolola Akinbami, Tiina Talts, Leena Bhaw, Richard Myers, Steven Platt, Kirstin Edwards, Jonathan Hubb, Joanna Ellis, Maria Zambon
EPI_ISL_420756 hCoV-19/England/20140039404/2020   Europe / United Kingdom / England   2020-03-27
Respiratory Virus Unit, Microbiology Services Colindale, Public Health England Respiratory Virus Unit, Microbiology Services Colindale, Public Health England Monica Galiano, Shahjahan Miah, Angie Lackenby, Omolola Akinbami, Tiina Talts, Leena Bhaw, Richard Myers, Steven Platt, Kirstin Edwards, Jonathan Hubb, Joanna Ellis,

Maria Zambon
EPI_ISL_420758 hCoV-19/England/20140039604/2020 Europe / United Kingdom / England 2020-03-27 Respiratory Virus Unit, Microbiology Services Colindale, Public Health England Respiratory Virus Unit, Microbiology Services Colindale, Public Health England Monica Galiano, Shahjahan Miah, Angie Lackenby, Omolola Akinbami, Tiina Talts, Leena Bhaw, Richard Myers, Steven Platt, Kirstin Edwards, Jonathan Hubb, Joanna Ellis, Maria Zambon
EPI_ISL_420761 hCoV-19/England/20140048704/2020 Europe / United Kingdom / England 2020-03-25 Respiratory Virus Unit, Microbiology Services Colindale, Public Health England Respiratory Virus Unit, Microbiology Services Colindale, Public Health England Monica Galiano, Shahjahan Miah, Angie Lackenby, Omolola Akinbami, Tiina Talts, Leena Bhaw, Richard Myers, Steven Platt, Kirstin Edwards, Jonathan Hubb, Joanna Ellis, Maria Zambon
EPI_ISL_420765 hCoV-19/England/20142022504/2020 Europe / United Kingdom / England 2020-03-27 Respiratory Virus Unit, Microbiology Services Colindale, Public Health England Respiratory Virus Unit, Microbiology Services Colindale, Public Health England Monica Galiano, Shahjahan Miah, Angie Lackenby, Omolola Akinbami, Tiina Talts, Leena Bhaw, Richard Myers, Steven Platt, Kirstin Edwards, Jonathan Hubb, Joanna Ellis, Maria Zambon
EPI_ISL_420766 hCoV-19/England/20142060404/2020 Europe / United Kingdom / England 2020-03-27 Respiratory Virus Unit, Microbiology Services Colindale, Public Health England Respiratory Virus Unit, Microbiology Services Colindale, Public Health England Monica Galiano, Shahjahan Miah, Angie Lackenby, Omolola Akinbami, Tiina Talts, Leena Bhaw, Richard Myers, Steven Platt, Kirstin Edwards, Jonathan Hubb, Joanna Ellis, Maria Zambon
EPI_ISL_420767 hCoV-19/England/20142060804/2020 Europe / United Kingdom / England 2020-03-30 Respiratory Virus Unit, Microbiology Services Colindale, Public Health England Respiratory Virus Unit, Microbiology Services Colindale, Public Health England Monica Galiano, Shahjahan Miah, Angie Lackenby, Omolola Akinbami, Tiina Talts, Leena Bhaw, Richard Myers, Steven Platt, Kirstin Edwards, Jonathan Hubb, Joanna Ellis, Maria Zambon
EPI_ISL_420772 hCoV-19/England/20142061804/2020 Europe / United Kingdom / England 2020-03-30 Respiratory Virus Unit, Microbiology Services Colindale, Public Health England Respiratory Virus Unit, Microbiology Services Colindale, Public Health England Monica Galiano, Shahjahan Miah, Angie Lackenby, Omolola Akinbami, Tiina Talts, Leena Bhaw, Richard Myers, Steven Platt, Kirstin Edwards, Jonathan Hubb, Joanna Ellis, Maria Zambon
EPI_ISL_420773 hCoV-19/England/20142062204/2020 Europe / United Kingdom / England 2020-03-30 Respiratory Virus Unit, Microbiology Services Colindale, Public Health England Respiratory Virus Unit, Microbiology Services Colindale, Public Health England Monica Galiano, Shahjahan Miah, Angie Lackenby, Omolola Akinbami, Tiina Talts, Leena Bhaw, Richard Myers, Steven Platt, Kirstin Edwards, Jonathan Hubb, Joanna Ellis, Maria Zambon
EPI_ISL_420774 hCoV-19/England/20142062604/2020 Europe / United Kingdom / England 2020-03-28 Respiratory Virus Unit, Microbiology Services Colindale, Public Health England Respiratory Virus Unit, Microbiology Services Colindale, Public Health England Monica Galiano, Shahjahan Miah, Angie Lackenby, Omolola Akinbami, Tiina Talts, Leena Bhaw, Richard Myers, Steven Platt, Kirstin Edwards, Jonathan Hubb, Joanna Ellis, Maria Zambon
EPI_ISL_420775 hCoV-19/England/20142062904/2020 Europe / United Kingdom / England 2020-03-30 Respiratory Virus Unit, Microbiology Services Colindale, Public Health England Respiratory Virus Unit, Microbiology Services Colindale, Public Health England Monica Galiano, Shahjahan Miah, Angie Lackenby, Omolola Akinbami, Tiina Talts, Leena Bhaw, Richard Myers, Steven Platt, Kirstin Edwards, Jonathan Hubb, Joanna Ellis, Maria Zambon
EPI_ISL_420784 hCoV-19/USA/AZ_4811/2020 North America / USA / Arizona 2020-03-02 AZ Department of Health Services Pathogen Discovery, Respiratory Viruses Branch, Division of Viral Diseases, Centers for Disease Control and Prevention Krista Queen, Yan Li, Ying Tao, Jing Zhang, Anne Uehara, Clinton R. Paden, Haibin Wang, Rachel Marine, Mary S. Keckler, Alison S. Laufer Halpin, Jasmine Padilla, Justin Lee, Christopher A. Elkins, Suxiang Tong
EPI_ISL_420785 hCoV-19/USA/FL_6318/2020 North America / USA / Florida 2020-03-02 FL Bureau of Health Laboratories Tampa Pathogen Discovery, Respiratory Viruses Branch, Division of Viral Diseases, Centers for Disease Control and Prevention Krista Queen, Yan Li, Ying Tao, Jing Zhang, Anne Uehara, Clinton R. Paden, Haibin Wang, Rachel Marine, Mary S. Keckler, Alison S. Laufer Halpin, Jasmine Padilla, Justin Lee, Christopher A. Elkins, Suxiang Tong
EPI_ISL_420786 hCoV-19/USA/GA_1320/2020 North America / USA / Georgia 2020-03-03 GA Department of Public Health Pathogen Discovery, Respiratory Viruses Branch, Division of Viral Diseases, Centers for Disease Control and Prevention Krista Queen, Yan Li, Ying Tao, Jing Zhang, Anne Uehara, Clinton R. Paden, Haibin Wang, Rachel Marine, Mary S. Keckler, Alison S. Laufer Halpin, Jasmine Padilla, Justin Lee, Christopher A. Elkins, Suxiang Tong
EPI_ISL_420787 hCoV-19/USA/GA_1299/2020 North America / USA / Georgia 2020-03-03 GA Department of Public Health Pathogen Discovery, Respiratory Viruses Branch, Division of Viral Diseases, Centers for Disease Control and Prevention Krista Queen, Yan Li, Ying Tao, Jing Zhang, Anne Uehara, Clinton R. Paden, Haibin Wang, Rachel Marine, Mary S. Keckler, Alison S. Laufer Halpin, Jasmine Padilla, Justin Lee, Christopher A. Elkins, Suxiang Tong
EPI_ISL_420788 hCoV-19/USA/GA_1445/2020 North America / USA / Georgia 2020-03-04 GA Department of Public Health Pathogen Discovery, Respiratory Viruses Branch, Division of Viral Diseases, Centers for Disease Control and Prevention Krista Queen, Yan Li, Ying Tao, Jing Zhang, Anne Uehara, Clinton R. Paden, Haibin Wang, Rachel Marine, Mary S. Keckler, Alison S. Laufer Halpin, Jasmine Padilla, Justin Lee, Christopher

A. Elkins, Suxiang Tong
EPI_ISL_420789 hCoV-19/USA/IL_1375/2020 North America / USA / Illinois 2020-03-01 Illinois Department of Public Health Chicago Laboratory Pathogen Discovery, Respiratory Viruses Branch, Division of Viral Diseases, Centers for Disease Control and Prevention Krista Queen, Yan Li, Ying Tao, Jing Zhang, Anne Uehara, Clinton R. Paden, Haibin Wang, Rachel Marine, Mary S. Keckler, Alison S. Laufer Halpin, Jasmine Padilla, Justin Lee, Christopher A. Elkins, Suxiang Tong
EPI_ISL_420790 hCoV-19/USA/IL_1293/2020 North America / USA / Illinois 2020-03-01 Illinois Department of Public Health Chicago Laboratory Pathogen Discovery, Respiratory Viruses Branch, Division of Viral Diseases, Centers for Disease Control and Prevention Krista Queen, Yan Li, Ying Tao, Jing Zhang, Anne Uehara, Clinton R. Paden, Haibin Wang, Rachel Marine, Mary S. Keckler, Alison S. Laufer Halpin, Jasmine Padilla, Justin Lee, Christopher A. Elkins, Suxiang Tong
EPI_ISL_420791 hCoV-19/USA/NH_0004/2020 North America / USA / New Hampshire 2020-02-29 NH Department of Health and Human Services Public Health Labs Pathogen Discovery, Respiratory Viruses Branch, Division of Viral Diseases, Centers for Disease Control and Prevention Krista Queen, Yan Li, Ying Tao, Jing Zhang, Anne Uehara, Clinton R. Paden, Haibin Wang, Rachel Marine, Mary S. Keckler, Alison S. Laufer Halpin, Jasmine Padilla, Justin Lee, Christopher A. Elkins, Suxiang Tong
EPI_ISL_420792 hCoV-19/USA/NH_0008/2020 North America / USA / New Hampshire 2020-03-02 NH Department of Health and Human Services Public Health Labs Pathogen Discovery, Respiratory Viruses Branch, Division of Viral Diseases, Centers for Disease Control and Prevention Krista Queen, Yan Li, Ying Tao, Jing Zhang, Anne Uehara, Clinton R. Paden, Haibin Wang, Rachel Marine, Mary S. Keckler, Alison S. Laufer Halpin, Jasmine Padilla, Justin Lee, Christopher A. Elkins, Suxiang Tong
EPI_ISL_420793 hCoV-19/USA/NY_2929/2020 North America / USA / New York 2020-03-02 NYC Department of Health and Mental Hygiene Pathogen Discovery, Respiratory Viruses Branch, Division of Viral Diseases, Centers for Disease Control and Prevention Krista Queen, Yan Li, Ying Tao, Jing Zhang, Anne Uehara, Clinton R. Paden, Haibin Wang, Rachel Marine, Mary S. Keckler, Alison S. Laufer Halpin, Jasmine Padilla, Justin Lee, Christopher A. Elkins, Suxiang Tong
EPI_ISL_420794 hCoV-19/USA/OR_5430/2020 North America / USA / Oregon 2020-03-01 Oregon State Public Health- Virology section Pathogen Discovery, Respiratory Viruses Branch, Division of Viral Diseases, Centers for Disease Control and Prevention Krista Queen, Yan Li, Ying Tao, Jing Zhang, Anne Uehara, Clinton R. Paden, Haibin Wang, Rachel Marine, Mary S. Keckler, Alison S. Laufer Halpin, Jasmine Padilla, Justin Lee, Christopher A. Elkins, Suxiang Tong
EPI_ISL_420795 hCoV-19/USA/RI_0556/2020 North America / USA / Rhode Island 2020-03-01 RI State Health Laboratory Pathogen Discovery, Respiratory Viruses Branch, Division of Viral Diseases, Centers for Disease Control and Prevention Krista Queen, Yan Li, Ying Tao, Jing Zhang, Anne Uehara, Clinton R. Paden, Haibin Wang, Rachel Marine, Mary S. Keckler, Alison S. Laufer Halpin, Jasmine Padilla, Justin Lee, Christopher A. Elkins, Suxiang Tong
EPI_ISL_420796 hCoV-19/USA/TX_2039/2020 North America / USA / Texas 2020-02-29 Texas DSHS Lab Services Pathogen Discovery, Respiratory Viruses Branch, Division of Viral Diseases, Centers for Disease Control and Prevention Krista Queen, Yan Li, Ying Tao, Jing Zhang, Anne Uehara, Clinton R. Paden, Haibin Wang, Rachel Marine, Mary S. Keckler, Alison S. Laufer Halpin, Jasmine Padilla, Justin Lee, Christopher A. Elkins, Suxiang Tong
EPI_ISL_420797 hCoV-19/USA/TX_2817/2020 North America / USA / Texas 2020-03-01 Texas DSHS Lab Services Pathogen Discovery, Respiratory Viruses Branch, Division of Viral Diseases, Centers for Disease Control and Prevention Krista Queen, Yan Li, Ying Tao, Jing Zhang, Anne Uehara, Clinton R. Paden, Haibin Wang, Rachel Marine, Mary S. Keckler, Alison S. Laufer Halpin, Jasmine Padilla, Justin Lee, Christopher A. Elkins, Suxiang Tong
EPI_ISL_420798 hCoV-19/USA/TX_2967/2020 North America / USA / Texas 2020-03-01 Texas DSHS Lab Services Pathogen Discovery, Respiratory Viruses Branch, Division of Viral Diseases, Centers for Disease Control and Prevention Krista Queen, Yan Li, Ying Tao, Jing Zhang, Anne Uehara, Clinton R. Paden, Haibin Wang, Rachel Marine, Mary S. Keckler, Alison S. Laufer Halpin, Jasmine Padilla, Justin Lee, Christopher A. Elkins, Suxiang Tong
EPI_ISL_420799 hCoV-19/Korea/BA-ACH_2604/2020 Asia / Korea 2020-02-27 Brian D. Allgood Army Community Hospital Pathogen Discovery, Respiratory Viruses Branch, Division of Viral Diseases, Centers for Disease Control and Prevention Krista Queen, Yan Li, Ying Tao, Jing Zhang, Anne Uehara, Clinton R. Paden, Haibin Wang, Rachel Marine, Mary S. Keckler, Alison S. Laufer Halpin, Jasmine Padilla, Justin Lee, Christopher A. Elkins, Suxiang Tong
EPI_ISL_420822 hCoV-19/USA/UT-0349/2020 North America / USA / Utah 2020-03-25 Utah Public Health Laboratory Utah Public Health Laboratory Erin Young, Kelly Oakeson
EPI_ISL_420899 hCoV-19/Germany/BAV-V2010492/2020 Europe / Germany / Munich 2020-03-11 Max von Pettenkofer Institute, Virology, National Reference Center for Retroviruses, LMU Munich Laboratory for Functional Genome Analysis, Dept. Genomics, Gene Center of the LMU Munich Max Muenchhoff, Stefan Krebs, Alexander Graf, Ashok Varadharajan, Oliver Keppler, Helmut Blum
EPI_ISL_423091 hCoV-19/England/20134016802/2020 Europe / United Kingdom / England 2020-03-24 Respiratory Virus Unit, Microbiology Services Colindale, Public Health England Respiratory Virus Unit, Microbiology Services Colindale, Public Health England Monica Galiano, Shahjahan Miah, Angie Lackenby, Omolola Akinbami, Tiina Talts, Leena Bhaw, Richard Myers, Steven Platt, Kirstin Edwards, Jonathan Hubb, Joanna Ellis, Maria Zambon
EPI_ISL_423092 hCoV-19/England/20134016902/2020 Europe / United Kingdom / England 2020-03-24 Respiratory Virus Unit, Microbiology Services Colindale, Public Health England Respiratory Virus Unit, Microbiology Services Colindale, Public Health England Monica Galiano, Shahjahan Miah, Angie Lackenby, Omolola

Akinbami, Tiina Talts, Leena Bhaw, Richard Myers, Steven Platt, Kirstin Edwards, Jonathan Hubb, Joanna Ellis, Maria Zambon
EPI_ISL_423094 hCoV-19/England/20134017404/2020 Europe / United Kingdom / England 2020-03-24 Respiratory Virus Unit, Microbiology Services Colindale, Public Health England Respiratory Virus Unit, Microbiology Services Colindale, Public Health England Monica Galiano, Shahjahan Miah, Angie Lackenby, Omolola Akinbami, Tiina Talts, Leena Bhaw, Richard Myers, Steven Platt, Kirstin Edwards, Jonathan Hubb, Joanna Ellis, Maria Zambon
EPI_ISL_423095 hCoV-19/England/20134017504/2020 Europe / United Kingdom / England 2020-03-24 Respiratory Virus Unit, Microbiology Services Colindale, Public Health England Respiratory Virus Unit, Microbiology Services Colindale, Public Health England Monica Galiano, Shahjahan Miah, Angie Lackenby, Omolola Akinbami, Tiina Talts, Leena Bhaw, Richard Myers, Steven Platt, Kirstin Edwards, Jonathan Hubb, Joanna Ellis, Maria Zambon
EPI_ISL_423096 hCoV-19/England/20134017904/2020 Europe / United Kingdom / England 2020-03-23 Respiratory Virus Unit, Microbiology Services Colindale, Public Health England Respiratory Virus Unit, Microbiology Services Colindale, Public Health England Monica Galiano, Shahjahan Miah, Angie Lackenby, Omolola Akinbami, Tiina Talts, Leena Bhaw, Richard Myers, Steven Platt, Kirstin Edwards, Jonathan Hubb, Joanna Ellis, Maria Zambon
EPI_ISL_423097 hCoV-19/England/20134018004/2020 Europe / United Kingdom / England 2020-03-23 Respiratory Virus Unit, Microbiology Services Colindale, Public Health England Respiratory Virus Unit, Microbiology Services Colindale, Public Health England Monica Galiano, Shahjahan Miah, Angie Lackenby, Omolola Akinbami, Tiina Talts, Leena Bhaw, Richard Myers, Steven Platt, Kirstin Edwards, Jonathan Hubb, Joanna Ellis, Maria Zambon
EPI_ISL_423098 hCoV-19/England/20134018402/2020 Europe / United Kingdom / England 2020-03-23 Respiratory Virus Unit, Microbiology Services Colindale, Public Health England Respiratory Virus Unit, Microbiology Services Colindale, Public Health England Monica Galiano, Shahjahan Miah, Angie Lackenby, Omolola Akinbami, Tiina Talts, Leena Bhaw, Richard Myers, Steven Platt, Kirstin Edwards, Jonathan Hubb, Joanna Ellis, Maria Zambon
EPI_ISL_423099 hCoV-19/England/20134018602/2020 Europe / United Kingdom / England 2020-03-23 Respiratory Virus Unit, Microbiology Services Colindale, Public Health England Respiratory Virus Unit, Microbiology Services Colindale, Public Health England Monica Galiano, Shahjahan Miah, Angie Lackenby, Omolola Akinbami, Tiina Talts, Leena Bhaw, Richard Myers, Steven Platt, Kirstin Edwards, Jonathan Hubb, Joanna Ellis, Maria Zambon
EPI_ISL_424200 hCoV-19/USA/WA-UW-1723/2020 North America / USA / Washington 2020-03-20 UW Virology Lab UW Virology Lab Pavitra Roychoudhury, Hong Xie, Keith Jerome, Alexander Greninger
EPI_ISL_424202 hCoV-19/USA/WA-UW-1651/2020 North America / USA / Washington 2020-03-20 UW Virology Lab UW Virology Lab Pavitra Roychoudhury, Hong Xie, Keith Jerome, Alexander Greninger
EPI_ISL_424203 hCoV-19/USA/WA-UW-1652/2020 North America / USA / Washington 2020-03-20 UW Virology Lab UW Virology Lab Pavitra Roychoudhury, Hong Xie, Keith Jerome, Alexander Greninger
EPI_ISL_424204 hCoV-19/USA/WA-UW-1698/2020 North America / USA / Washington 2020-03-20 UW Virology Lab UW Virology Lab Pavitra Roychoudhury, Hong Xie, Keith Jerome, Alexander Greninger
EPI_ISL_424205 hCoV-19/USA/WA-UW-1697/2020 North America / USA / Washington 2020-03-20 UW Virology Lab UW Virology Lab Pavitra Roychoudhury, Hong Xie, Keith Jerome, Alexander Greninger
EPI_ISL_424207 hCoV-19/USA/WA-UW-1716/2020 North America / USA / Washington 2020-03-20 UW Virology Lab UW Virology Lab Pavitra Roychoudhury, Hong Xie, Keith Jerome, Alexander Greninger
EPI_ISL_424208 hCoV-19/USA/WA-UW-1712/2020 North America / USA / Washington 2020-03-20 UW Virology Lab UW Virology Lab Pavitra Roychoudhury, Hong Xie, Keith Jerome, Alexander Greninger
EPI_ISL_424209 hCoV-19/USA/WA-UW-1765/2020 North America / USA / Washington 2020-03-20 UW Virology Lab UW Virology Lab Pavitra Roychoudhury, Hong Xie, Keith Jerome, Alexander Greninger
EPI_ISL_424210 hCoV-19/USA/WA-UW-1718/2020 North America / USA / Washington 2020-03-20 UW Virology Lab UW Virology Lab Pavitra Roychoudhury, Hong Xie, Keith Jerome, Alexander Greninger
EPI_ISL_424211 hCoV-19/USA/WA-UW-1689/2020 North America / USA / Washington 2020-03-19 UW Virology Lab UW Virology Lab Pavitra Roychoudhury, Hong Xie, Keith Jerome, Alexander Greninger
EPI_ISL_424212 hCoV-19/USA/WA-UW-1715/2020 North America / USA / Washington 2020-03-19 UW Virology Lab UW Virology Lab Pavitra Roychoudhury, Hong Xie, Keith Jerome, Alexander Greninger
EPI_ISL_424213 hCoV-19/USA/WA-UW-1670/2020 North America / USA / Washington 2020-03-20 UW Virology Lab UW Virology Lab Pavitra Roychoudhury, Hong Xie, Keith Jerome, Alexander Greninger
EPI_ISL_424214 hCoV-19/USA/WA-UW-1677/2020 North America / USA / Washington 2020-03-20 UW Virology Lab UW Virology Lab Pavitra Roychoudhury, Hong Xie, Keith Jerome, Alexander Greninger
EPI_ISL_424215 hCoV-19/USA/WA-UW-1680/2020 North America / USA / Washington 2020-03-20 UW Virology Lab UW Virology Lab Pavitra Roychoudhury, Hong Xie, Keith Jerome, Alexander Greninger
EPI_ISL_424216 hCoV-19/USA/WA-UW-1690/2020 North America / USA / Washington 2020-03-19 UW Virology Lab UW Virology Lab Pavitra Roychoudhury, Hong Xie, Keith Jerome, Alexander Greninger
EPI_ISL_424218 hCoV-19/USA/WA-UW-1682/2020 North America / USA / Washington 2020-03-20 UW Virology Lab UW Virology Lab Pavitra Roychoudhury, Hong Xie, Keith Jerome, Alexander Greninger
EPI_ISL_424219 hCoV-19/USA/WA-UW-1695/2020 North America / USA / Washington 2020-03-20 UW Virology Lab UW Virology Lab Pavitra Roychoudhury, Hong Xie, Keith Jerome, Alexander Greninger
EPI_ISL_424220 hCoV-19/USA/WA-UW-1729/2020 North America / USA / Washington 2020-03-21 UW Virology Lab UW Virology Lab Pavitra Roychoudhury, Hong Xie, Keith Jerome, Alexander Greninger
EPI_ISL_424221 hCoV-19/USA/WA-UW-1748/2020 North America / USA / Washington 2020-03-21 UW Virology Lab UW Virology Lab Pavitra Roychoudhury, Hong Xie, Keith Jerome, Alexander Greninger

EPI_ISL_424222 hCoV-19/USA/WA-UW-1700/2020 North America / USA / Washington 2020-03-20 UW
Virology Lab UW Virology Lab Pavitra Roychoudhury, Hong Xie, Keith Jerome, Alexander Greninger
EPI_ISL_424223 hCoV-19/USA/WA-UW-1744/2020 North America / USA / Washington 2020-03-21 UW
Virology Lab UW Virology Lab Pavitra Roychoudhury, Hong Xie, Keith Jerome, Alexander Greninger
EPI_ISL_424224 hCoV-19/USA/WA-UW-1745/2020 North America / USA / Washington 2020-03-21 UW
Virology Lab UW Virology Lab Pavitra Roychoudhury, Hong Xie, Keith Jerome, Alexander Greninger
EPI_ISL_424225 hCoV-19/USA/WA-UW-1707/2020 North America / USA / Washington 2020-03-21 UW
Virology Lab UW Virology Lab Pavitra Roychoudhury, Hong Xie, Keith Jerome, Alexander Greninger
EPI_ISL_424226 hCoV-19/USA/WA-UW-1705/2020 North America / USA / Washington 2020-03-21 UW
Virology Lab UW Virology Lab Pavitra Roychoudhury, Hong Xie, Keith Jerome, Alexander Greninger
EPI_ISL_424227 hCoV-19/USA/WA-UW-1706/2020 North America / USA / Washington 2020-03-21 UW
Virology Lab UW Virology Lab Pavitra Roychoudhury, Hong Xie, Keith Jerome, Alexander Greninger
EPI_ISL_424228 hCoV-19/USA/WA-UW-1702/2020 North America / USA / Washington 2020-03-21 UW
Virology Lab UW Virology Lab Pavitra Roychoudhury, Hong Xie, Keith Jerome, Alexander Greninger
EPI_ISL_424229 hCoV-19/USA/WA-UW-1724/2020 North America / USA / Washington 2020-03-21 UW
Virology Lab UW Virology Lab Pavitra Roychoudhury, Hong Xie, Keith Jerome, Alexander Greninger
EPI_ISL_424230 hCoV-19/USA/WA-UW-1709/2020 North America / USA / Washington 2020-03-21 UW
Virology Lab UW Virology Lab Pavitra Roychoudhury, Hong Xie, Keith Jerome, Alexander Greninger
EPI_ISL_424231 hCoV-19/USA/WA-UW-1731/2020 North America / USA / Washington 2020-03-21 UW
Virology Lab UW Virology Lab Pavitra Roychoudhury, Hong Xie, Keith Jerome, Alexander Greninger
EPI_ISL_424232 hCoV-19/USA/WA-UW-1708/2020 North America / USA / Washington 2020-03-21 UW
Virology Lab UW Virology Lab Pavitra Roychoudhury, Hong Xie, Keith Jerome, Alexander Greninger
EPI_ISL_424233 hCoV-19/USA/WA-UW-1772/2020 North America / USA / Washington 2020-03-21 UW
Virology Lab UW Virology Lab Pavitra Roychoudhury, Hong Xie, Keith Jerome, Alexander Greninger
EPI_ISL_424234 hCoV-19/USA/WA-UW-1722/2020 North America / USA / Washington 2020-03-21 UW
Virology Lab UW Virology Lab Pavitra Roychoudhury, Hong Xie, Keith Jerome, Alexander Greninger
EPI_ISL_424235 hCoV-19/USA/WA-UW-1733/2020 North America / USA / Washington 2020-03-21 UW
Virology Lab UW Virology Lab Pavitra Roychoudhury, Hong Xie, Keith Jerome, Alexander Greninger
EPI_ISL_424236 hCoV-19/USA/WA-UW-1732/2020 North America / USA / Washington 2020-03-21 UW
Virology Lab UW Virology Lab Pavitra Roychoudhury, Hong Xie, Keith Jerome, Alexander Greninger
EPI_ISL_424237 hCoV-19/USA/WA-UW-1739/2020 North America / USA / Washington 2020-03-21 UW
Virology Lab UW Virology Lab Pavitra Roychoudhury, Hong Xie, Keith Jerome, Alexander Greninger
EPI_ISL_424238 hCoV-19/USA/WA-UW-1741/2020 North America / USA / Washington 2020-03-21 UW
Virology Lab UW Virology Lab Pavitra Roychoudhury, Hong Xie, Keith Jerome, Alexander Greninger
EPI_ISL_424239 hCoV-19/USA/WA-UW-1750/2020 North America / USA / Washington 2020-03-21 UW
Virology Lab UW Virology Lab Pavitra Roychoudhury, Hong Xie, Keith Jerome, Alexander Greninger
EPI_ISL_424240 hCoV-19/USA/WA-UW-1730/2020 North America / USA / Washington 2020-03-21 UW
Virology Lab UW Virology Lab Pavitra Roychoudhury, Hong Xie, Keith Jerome, Alexander Greninger
EPI_ISL_424241 hCoV-19/USA/WA-UW-1735/2020 North America / USA / Washington 2020-03-21 UW
Virology Lab UW Virology Lab Pavitra Roychoudhury, Hong Xie, Keith Jerome, Alexander Greninger
EPI_ISL_424242 hCoV-19/USA/WA-UW-1753/2020 North America / USA / Washington 2020-03-21 UW
Virology Lab UW Virology Lab Pavitra Roychoudhury, Hong Xie, Keith Jerome, Alexander Greninger
EPI_ISL_424243 hCoV-19/USA/WA-UW-1756/2020 North America / USA / Washington 2020-03-21 UW
Virology Lab UW Virology Lab Pavitra Roychoudhury, Hong Xie, Keith Jerome, Alexander Greninger
EPI_ISL_424244 hCoV-19/USA/WA-UW-1770/2020 North America / USA / Washington 2020-03-22 UW
Virology Lab UW Virology Lab Pavitra Roychoudhury, Hong Xie, Keith Jerome, Alexander Greninger
EPI_ISL_424245 hCoV-19/USA/WA-UW-1762/2020 North America / USA / Washington 2020-03-21 UW
Virology Lab UW Virology Lab Pavitra Roychoudhury, Hong Xie, Keith Jerome, Alexander Greninger
EPI_ISL_424246 hCoV-19/USA/WA-UW-1758/2020 North America / USA / Washington 2020-03-21 UW
Virology Lab UW Virology Lab Pavitra Roychoudhury, Hong Xie, Keith Jerome, Alexander Greninger
EPI_ISL_424247 hCoV-19/USA/WA-UW-1760/2020 North America / USA / Washington 2020-03-21 UW
Virology Lab UW Virology Lab Pavitra Roychoudhury, Hong Xie, Keith Jerome, Alexander Greninger
EPI_ISL_424248 hCoV-19/USA/UN-UW-1773/2020 North America / USA 2020-03 UW Virology Lab UW Virology Lab
Pavitra Roychoudhury, Hong Xie, Keith Jerome, Alexander Greninger
EPI_ISL_424249 hCoV-19/USA/UN-UW-1791/2020 North America / USA 2020-03-20 UW Virology Lab UW
Virology Lab Pavitra Roychoudhury, Hong Xie, Keith Jerome, Alexander Greninger
EPI_ISL_424250 hCoV-19/USA/WA-UW-1785/2020 North America / USA / Washington 2020-03-19 UW
Virology Lab UW Virology Lab Pavitra Roychoudhury, Hong Xie, Keith Jerome, Alexander Greninger
EPI_ISL_424251 hCoV-19/USA/WA-UW-1801/2020 North America / USA / Washington 2020-03-23 UW
Virology Lab UW Virology Lab Pavitra Roychoudhury, Hong Xie, Keith Jerome, Alexander Greninger
EPI_ISL_424252 hCoV-19/USA/WA-UW-1840/2020 North America / USA / Washington 2020-03-23 UW
Virology Lab UW Virology Lab Pavitra Roychoudhury, Hong Xie, Keith Jerome, Alexander Greninger
EPI_ISL_424253 hCoV-19/USA/WA-UW-1792/2020 North America / USA / Washington 2020-03-21 UW
Virology Lab UW Virology Lab Pavitra Roychoudhury, Hong Xie, Keith Jerome, Alexander Greninger
EPI_ISL_424254 hCoV-19/USA/WA-UW-1796/2020 North America / USA / Washington 2020-03-21 UW
Virology Lab UW Virology Lab Pavitra Roychoudhury, Hong Xie, Keith Jerome, Alexander Greninger
EPI_ISL_424255 hCoV-19/USA/UN-UW-1832/2020 North America / USA 2020-03-16 UW Virology Lab UW
Virology Lab Pavitra Roychoudhury, Hong Xie, Keith Jerome, Alexander Greninger
EPI_ISL_424256 hCoV-19/USA/WA-UW-1774/2020 North America / USA / Washington 2020-03-22 UW
Virology Lab UW Virology Lab Pavitra Roychoudhury, Hong Xie, Keith Jerome, Alexander Greninger

EPI_ISL_424257 hCoV-19/USA/WA-UW-1775/2020 North America / USA / Washington 2020-03-22 UW
Virology Lab UW Virology Lab Pavitra Roychoudhury, Hong Xie, Keith Jerome, Alexander Greninger
EPI_ISL_424258 hCoV-19/USA/WA-UW-1778/2020 North America / USA / Washington 2020-03-22 UW
Virology Lab UW Virology Lab Pavitra Roychoudhury, Hong Xie, Keith Jerome, Alexander Greninger
EPI_ISL_424259 hCoV-19/USA/WA-UW-1797/2020 North America / USA / Washington 2020-03-22 UW
Virology Lab UW Virology Lab Pavitra Roychoudhury, Hong Xie, Keith Jerome, Alexander Greninger
EPI_ISL_424260 hCoV-19/USA/WA-UW-1779/2020 North America / USA / Washington 2020-03-22 UW
Virology Lab UW Virology Lab Pavitra Roychoudhury, Hong Xie, Keith Jerome, Alexander Greninger
EPI_ISL_424261 hCoV-19/USA/WA-UW-1784/2020 North America / USA / Washington 2020-03-21 UW
Virology Lab UW Virology Lab Pavitra Roychoudhury, Hong Xie, Keith Jerome, Alexander Greninger
EPI_ISL_424262 hCoV-19/USA/WA-UW-1782/2020 North America / USA / Washington 2020-03-22 UW
Virology Lab UW Virology Lab Pavitra Roychoudhury, Hong Xie, Keith Jerome, Alexander Greninger
EPI_ISL_424263 hCoV-19/USA/WA-UW-1799/2020 North America / USA / Washington 2020-03-22 UW
Virology Lab UW Virology Lab Pavitra Roychoudhury, Hong Xie, Keith Jerome, Alexander Greninger
EPI_ISL_424264 hCoV-19/USA/WA-UW-1805/2020 North America / USA / Washington 2020-03-22 UW
Virology Lab UW Virology Lab Pavitra Roychoudhury, Hong Xie, Keith Jerome, Alexander Greninger
EPI_ISL_424266 hCoV-19/USA/UN-UW-1818/2020 North America / USA 2020-03-22 UW Virology Lab UW
Virology Lab Pavitra Roychoudhury, Hong Xie, Keith Jerome, Alexander Greninger
EPI_ISL_424267 hCoV-19/USA/UN-UW-1817/2020 North America / USA 2020-03-22 UW Virology Lab UW
Virology Lab Pavitra Roychoudhury, Hong Xie, Keith Jerome, Alexander Greninger
EPI_ISL_424268 hCoV-19/USA/UN-UW-1816/2020 North America / USA 2020-03-22 UW Virology Lab UW
Virology Lab Pavitra Roychoudhury, Hong Xie, Keith Jerome, Alexander Greninger
EPI_ISL_424269 hCoV-19/USA/WA-UW-1828/2020 North America / USA / Washington 2020-03-22 UW
Virology Lab UW Virology Lab Pavitra Roychoudhury, Hong Xie, Keith Jerome, Alexander Greninger
EPI_ISL_424270 hCoV-19/USA/WA-UW-1831/2020 North America / USA / Washington 2020-03-22 UW
Virology Lab UW Virology Lab Pavitra Roychoudhury, Hong Xie, Keith Jerome, Alexander Greninger
EPI_ISL_424271 hCoV-19/USA/WA-UW-1827/2020 North America / USA / Washington 2020-03-22 UW
Virology Lab UW Virology Lab Pavitra Roychoudhury, Hong Xie, Keith Jerome, Alexander Greninger
EPI_ISL_424273 hCoV-19/USA/WA-UW-1826/2020 North America / USA / Washington 2020-03-22 UW
Virology Lab UW Virology Lab Pavitra Roychoudhury, Hong Xie, Keith Jerome, Alexander Greninger
EPI_ISL_424274 hCoV-19/USA/WA-UW-1824/2020 North America / USA / Washington 2020-03-22 UW
Virology Lab UW Virology Lab Pavitra Roychoudhury, Hong Xie, Keith Jerome, Alexander Greninger
EPI_ISL_424275 hCoV-19/USA/WA-UW-1821/2020 North America / USA / Washington 2020-03-22 UW
Virology Lab UW Virology Lab Pavitra Roychoudhury, Hong Xie, Keith Jerome, Alexander Greninger
EPI_ISL_424276 hCoV-19/USA/WA-UW-1822/2020 North America / USA / Washington 2020-03-22 UW
Virology Lab UW Virology Lab Pavitra Roychoudhury, Hong Xie, Keith Jerome, Alexander Greninger
EPI_ISL_424278 hCoV-19/USA/WA-UW-1866/2020 North America / USA / Washington 2020-03-23 UW
Virology Lab UW Virology Lab Pavitra Roychoudhury, Hong Xie, Keith Jerome, Alexander Greninger
EPI_ISL_424279 hCoV-19/USA/WA-UW-1850/2020 North America / USA / Washington 2020-03-23 UW
Virology Lab UW Virology Lab Pavitra Roychoudhury, Hong Xie, Keith Jerome, Alexander Greninger
EPI_ISL_424280 hCoV-19/USA/WA-UW-1868/2020 North America / USA / Washington 2020-03-23 UW
Virology Lab UW Virology Lab Pavitra Roychoudhury, Hong Xie, Keith Jerome, Alexander Greninger
EPI_ISL_424281 hCoV-19/USA/WA-UW-1864/2020 North America / USA / Washington 2020-03-23 UW
Virology Lab UW Virology Lab Pavitra Roychoudhury, Hong Xie, Keith Jerome, Alexander Greninger
EPI_ISL_424282 hCoV-19/USA/WA-UW-1835/2020 North America / USA / Washington 2020-03-23 UW
Virology Lab UW Virology Lab Pavitra Roychoudhury, Hong Xie, Keith Jerome, Alexander Greninger
EPI_ISL_424284 hCoV-19/USA/WA-UW-1863/2020 North America / USA / Washington 2020-03-23 UW
Virology Lab UW Virology Lab Pavitra Roychoudhury, Hong Xie, Keith Jerome, Alexander Greninger
EPI_ISL_424285 hCoV-19/USA/OR-UW-1851/2020 North America / USA / Oregon 2020-03-21 UW Virology Lab
UW Virology Lab Pavitra Roychoudhury, Hong Xie, Keith Jerome, Alexander Greninger
EPI_ISL_424286 hCoV-19/USA/OR-UW-1849/2020 North America / USA / Oregon 2020-03-20 UW Virology Lab
UW Virology Lab Pavitra Roychoudhury, Hong Xie, Keith Jerome, Alexander Greninger
EPI_ISL_424287 hCoV-19/USA/WA-UW-1843/2020 North America / USA / Washington 2020-03-23 UW
Virology Lab UW Virology Lab Pavitra Roychoudhury, Hong Xie, Keith Jerome, Alexander Greninger
EPI_ISL_424288 hCoV-19/USA/WA-UW-1846/2020 North America / USA / Washington 2020-03-22 UW
Virology Lab UW Virology Lab Pavitra Roychoudhury, Hong Xie, Keith Jerome, Alexander Greninger
EPI_ISL_424289 hCoV-19/USA/WA-UW-1860/2020 North America / USA / Washington 2020-03-23 UW
Virology Lab UW Virology Lab Pavitra Roychoudhury, Hong Xie, Keith Jerome, Alexander Greninger
EPI_ISL_424290 hCoV-19/USA/WA-UW-1853/2020 North America / USA / Washington 2020-03-23 UW
Virology Lab UW Virology Lab Pavitra Roychoudhury, Hong Xie, Keith Jerome, Alexander Greninger
EPI_ISL_424291 hCoV-19/USA/WA-UW-1854/2020 North America / USA / Washington 2020-03-23 UW
Virology Lab UW Virology Lab Pavitra Roychoudhury, Hong Xie, Keith Jerome, Alexander Greninger
EPI_ISL_424292 hCoV-19/USA/WA-UW-1855/2020 North America / USA / Washington 2020-03-23 UW
Virology Lab UW Virology Lab Pavitra Roychoudhury, Hong Xie, Keith Jerome, Alexander Greninger
EPI_ISL_424293 hCoV-19/USA/WA-UW-1870/2020 North America / USA / Washington 2020-03-23 UW
Virology Lab UW Virology Lab Pavitra Roychoudhury, Hong Xie, Keith Jerome, Alexander Greninger
EPI_ISL_424294 hCoV-19/USA/WA-UW-1872/2020 North America / USA / Washington 2020-03-23 UW
Virology Lab UW Virology Lab Pavitra Roychoudhury, Hong Xie, Keith Jerome, Alexander Greninger
EPI_ISL_424295 hCoV-19/USA/WA-UW-1905/2020 North America / USA / Washington 2020-03-23 UW
Virology Lab UW Virology Lab Pavitra Roychoudhury, Hong Xie, Keith Jerome, Alexander Greninger

EPI_ISL_424296 hCoV-19/USA/WA-UW-1919/2020 North America / USA / Washington 2020-03-23 UW Virology Lab UW Virology Lab Pavitra Roychoudhury, Hong Xie, Keith Jerome, Alexander Greninger
EPI_ISL_424297 hCoV-19/USA/WA-UW-1913/2020 North America / USA / Washington 2020-03-23 UW Virology Lab UW Virology Lab Pavitra Roychoudhury, Hong Xie, Keith Jerome, Alexander Greninger
EPI_ISL_424298 hCoV-19/USA/WA-UW-1920/2020 North America / USA / Washington 2020-03-23 UW Virology Lab UW Virology Lab Pavitra Roychoudhury, Hong Xie, Keith Jerome, Alexander Greninger
EPI_ISL_424299 hCoV-19/USA/WA-UW-1904/2020 North America / USA / Washington 2020-03-23 UW Virology Lab UW Virology Lab Pavitra Roychoudhury, Hong Xie, Keith Jerome, Alexander Greninger
EPI_ISL_425501 hCoV-19/England/NOTT-10E12C/2020 Europe / United Kingdom / England 2020-03-15 Queens Medical Centre, Clinical Microbiology Department / DeepSeq Nottingham COVID-19 Genomics UK (COG-UK) Consortium Gemma Clark, Wendy Smith, Manjinder Khakh, Hannah Howson-Wells, Jonathan Ball, Patrick McClure, Joseph Chappell, Theocharis Tsoleridis, Nadine Holmes, Matthew Carlisle, Christopher Moore, Fei Sang, Johnny Debebe, Victoria Wright, Matthew Loose
EPI_ISL_425502 hCoV-19/England/NOTT-10E14A/2020 Europe / United Kingdom / England 2020-03-17 Queens Medical Centre, Clinical Microbiology Department / DeepSeq Nottingham COVID-19 Genomics UK (COG-UK) Consortium Gemma Clark, Wendy Smith, Manjinder Khakh, Hannah Howson-Wells, Jonathan Ball, Patrick McClure, Joseph Chappell, Theocharis Tsoleridis, Nadine Holmes, Matthew Carlisle, Christopher Moore, Fei Sang, Johnny Debebe, Victoria Wright, Matthew Loose
EPI_ISL_425504 hCoV-19/England/NOTT-10E186/2020 Europe / United Kingdom / England 2020-03-18 Queens Medical Centre, Clinical Microbiology Department / DeepSeq Nottingham COVID-19 Genomics UK (COG-UK) Consortium Gemma Clark, Wendy Smith, Manjinder Khakh, Hannah Howson-Wells, Jonathan Ball, Patrick McClure, Joseph Chappell, Theocharis Tsoleridis, Nadine Holmes, Matthew Carlisle, Christopher Moore, Fei Sang, Johnny Debebe, Victoria Wright, Matthew Loose
EPI_ISL_425505 hCoV-19/England/NOTT-10E195/2020 Europe / United Kingdom / England 2020-03-18 Queens Medical Centre, Clinical Microbiology Department / DeepSeq Nottingham COVID-19 Genomics UK (COG-UK) Consortium Gemma Clark, Wendy Smith, Manjinder Khakh, Hannah Howson-Wells, Jonathan Ball, Patrick McClure, Joseph Chappell, Theocharis Tsoleridis, Nadine Holmes, Matthew Carlisle, Christopher Moore, Fei Sang, Johnny Debebe, Victoria Wright, Matthew Loose
EPI_ISL_425510 hCoV-19/England/NOTT-10E1E0/2020 Europe / United Kingdom / England 2020-03-19 Queens Medical Centre, Clinical Microbiology Department / DeepSeq Nottingham COVID-19 Genomics UK (COG-UK) Consortium Gemma Clark, Wendy Smith, Manjinder Khakh, Hannah Howson-Wells, Jonathan Ball, Patrick McClure, Joseph Chappell, Theocharis Tsoleridis, Nadine Holmes, Matthew Carlisle, Christopher Moore, Fei Sang, Johnny Debebe, Victoria Wright, Matthew Loose
EPI_ISL_425513 hCoV-19/England/NOTT-10E21A/2020 Europe / United Kingdom / England 2020-03-20 Queens Medical Centre, Clinical Microbiology Department / DeepSeq Nottingham COVID-19 Genomics UK (COG-UK) Consortium Gemma Clark, Wendy Smith, Manjinder Khakh, Hannah Howson-Wells, Jonathan Ball, Patrick McClure, Joseph Chappell, Theocharis Tsoleridis, Nadine Holmes, Matthew Carlisle, Christopher Moore, Fei Sang, Johnny Debebe, Victoria Wright, Matthew Loose
EPI_ISL_425514 hCoV-19/England/NOTT-10E229/2020 Europe / United Kingdom / England 2020-03-20 Queens Medical Centre, Clinical Microbiology Department / DeepSeq Nottingham COVID-19 Genomics UK (COG-UK) Consortium Gemma Clark, Wendy Smith, Manjinder Khakh, Hannah Howson-Wells, Jonathan Ball, Patrick McClure, Joseph Chappell, Theocharis Tsoleridis, Nadine Holmes, Matthew Carlisle, Christopher Moore, Fei Sang, Johnny Debebe, Victoria Wright, Matthew Loose
EPI_ISL_425515 hCoV-19/England/NOTT-10E238/2020 Europe / United Kingdom / England 2020-03-20 Queens Medical Centre, Clinical Microbiology Department / DeepSeq Nottingham COVID-19 Genomics UK (COG-UK) Consortium Gemma Clark, Wendy Smith, Manjinder Khakh, Hannah Howson-Wells, Jonathan Ball, Patrick McClure, Joseph Chappell, Theocharis Tsoleridis, Nadine Holmes, Matthew Carlisle, Christopher Moore, Fei Sang, Johnny Debebe, Victoria Wright, Matthew Loose
EPI_ISL_425517 hCoV-19/England/NOTT-10E256/2020 Europe / United Kingdom / England 2020-03-20 Queens Medical Centre, Clinical Microbiology Department / DeepSeq Nottingham COVID-19 Genomics UK (COG-UK) Consortium Gemma Clark, Wendy Smith, Manjinder Khakh, Hannah Howson-Wells, Jonathan Ball, Patrick McClure, Joseph Chappell, Theocharis Tsoleridis, Nadine Holmes, Matthew Carlisle, Christopher Moore, Fei Sang, Johnny Debebe, Victoria Wright, Matthew Loose
EPI_ISL_425518 hCoV-19/England/NOTT-10E265/2020 Europe / United Kingdom / England 2020-03-20 Queens Medical Centre, Clinical Microbiology Department / DeepSeq Nottingham COVID-19 Genomics UK (COG-UK) Consortium Gemma Clark, Wendy Smith, Manjinder Khakh, Hannah Howson-Wells, Jonathan Ball, Patrick McClure, Joseph Chappell, Theocharis Tsoleridis, Nadine Holmes, Matthew Carlisle, Christopher Moore, Fei Sang, Johnny Debebe, Victoria Wright, Matthew Loose
EPI_ISL_425519 hCoV-19/England/NOTT-10E283/2020 Europe / United Kingdom / England 2020-03-20 Queens Medical Centre, Clinical Microbiology Department / DeepSeq Nottingham COVID-19 Genomics UK (COG-UK) Consortium Gemma Clark, Wendy Smith, Manjinder Khakh, Hannah Howson-Wells, Jonathan Ball, Patrick McClure, Joseph Chappell, Theocharis Tsoleridis, Nadine Holmes, Matthew Carlisle, Christopher Moore, Fei Sang, Johnny Debebe, Victoria Wright, Matthew Loose
EPI_ISL_425522 hCoV-19/England/NOTT-10E2B0/2020 Europe / United Kingdom / England 2020-03-20 Queens Medical Centre, Clinical Microbiology Department / DeepSeq Nottingham COVID-19 Genomics UK (COG-UK) Consortium Gemma Clark, Wendy Smith, Manjinder Khakh, Hannah Howson-Wells, Jonathan Ball, Patrick McClure, Joseph Chappell, Theocharis Tsoleridis, Nadine Holmes, Matthew Carlisle, Christopher Moore, Fei Sang, Johnny Debebe, Victoria Wright, Matthew Loose
EPI_ISL_425523 hCoV-19/England/NOTT-10E2CF/2020 Europe / United Kingdom / England 2020-03-20 Queens Medical Centre, Clinical Microbiology Department / DeepSeq Nottingham COVID-19 Genomics UK (COG-UK)

Consortium Gemma Clark, Wendy Smith, Manjinder Khakh, Hannah Howson-Wells, Jonathan Ball, Patrick McClure, Joseph Chappell, Theocharis Tsoleridis, Nadine Holmes, Matthew Carlisle, Christopher Moore, Fei Sang, Johnny Debebe, Victoria Wright, Matthew Loose
EPI_ISL_425525 hCoV-19/England/NOTT-10E2ED/2020 Europe / United Kingdom / England 2020-03-21 Queens Medical Centre, Clinical Microbiology Department / DeepSeq Nottingham COVID-19 Genomics UK (COG-UK) Consortium Gemma Clark, Wendy Smith, Manjinder Khakh, Hannah Howson-Wells, Jonathan Ball, Patrick McClure, Joseph Chappell, Theocharis Tsoleridis, Nadine Holmes, Matthew Carlisle, Christopher Moore, Fei Sang, Johnny Debebe, Victoria Wright, Matthew Loose
EPI_ISL_425526 hCoV-19/England/NOTT-10E2FC/2020 Europe / United Kingdom / England 2020-03-21 Queens Medical Centre, Clinical Microbiology Department / DeepSeq Nottingham COVID-19 Genomics UK (COG-UK) Consortium Gemma Clark, Wendy Smith, Manjinder Khakh, Hannah Howson-Wells, Jonathan Ball, Patrick McClure, Joseph Chappell, Theocharis Tsoleridis, Nadine Holmes, Matthew Carlisle, Christopher Moore, Fei Sang, Johnny Debebe, Victoria Wright, Matthew Loose
EPI_ISL_425527 hCoV-19/England/NOTT-10E308/2020 Europe / United Kingdom / England 2020-03-19 Queens Medical Centre, Clinical Microbiology Department / DeepSeq Nottingham COVID-19 Genomics UK (COG-UK) Consortium Gemma Clark, Wendy Smith, Manjinder Khakh, Hannah Howson-Wells, Jonathan Ball, Patrick McClure, Joseph Chappell, Theocharis Tsoleridis, Nadine Holmes, Matthew Carlisle, Christopher Moore, Fei Sang, Johnny Debebe, Victoria Wright, Matthew Loose
EPI_ISL_425530 hCoV-19/England/NOTT-10E335/2020 Europe / United Kingdom / England 2020-03-21 Queens Medical Centre, Clinical Microbiology Department / DeepSeq Nottingham COVID-19 Genomics UK (COG-UK) Consortium Gemma Clark, Wendy Smith, Manjinder Khakh, Hannah Howson-Wells, Jonathan Ball, Patrick McClure, Joseph Chappell, Theocharis Tsoleridis, Nadine Holmes, Matthew Carlisle, Christopher Moore, Fei Sang, Johnny Debebe, Victoria Wright, Matthew Loose
EPI_ISL_425532 hCoV-19/England/NOTT-10E362/2020 Europe / United Kingdom / England 2020-03-21 Queens Medical Centre, Clinical Microbiology Department / DeepSeq Nottingham COVID-19 Genomics UK (COG-UK) Consortium Gemma Clark, Wendy Smith, Manjinder Khakh, Hannah Howson-Wells, Jonathan Ball, Patrick McClure, Joseph Chappell, Theocharis Tsoleridis, Nadine Holmes, Matthew Carlisle, Christopher Moore, Fei Sang, Johnny Debebe, Victoria Wright, Matthew Loose
EPI_ISL_425533 hCoV-19/England/NOTT-10E371/2020 Europe / United Kingdom / England 2020-03-21 Queens Medical Centre, Clinical Microbiology Department / DeepSeq Nottingham COVID-19 Genomics UK (COG-UK) Consortium Gemma Clark, Wendy Smith, Manjinder Khakh, Hannah Howson-Wells, Jonathan Ball, Patrick McClure, Joseph Chappell, Theocharis Tsoleridis, Nadine Holmes, Matthew Carlisle, Christopher Moore, Fei Sang, Johnny Debebe, Victoria Wright, Matthew Loose
EPI_ISL_425535 hCoV-19/England/NOTT-10E39F/2020 Europe / United Kingdom / England 2020-03-21 Queens Medical Centre, Clinical Microbiology Department / DeepSeq Nottingham COVID-19 Genomics UK (COG-UK) Consortium Gemma Clark, Wendy Smith, Manjinder Khakh, Hannah Howson-Wells, Jonathan Ball, Patrick McClure, Joseph Chappell, Theocharis Tsoleridis, Nadine Holmes, Matthew Carlisle, Christopher Moore, Fei Sang, Johnny Debebe, Victoria Wright, Matthew Loose
EPI_ISL_425536 hCoV-19/England/NOTT-10E3AE/2020 Europe / United Kingdom / England 2020-03-21 Queens Medical Centre, Clinical Microbiology Department / DeepSeq Nottingham COVID-19 Genomics UK (COG-UK) Consortium Gemma Clark, Wendy Smith, Manjinder Khakh, Hannah Howson-Wells, Jonathan Ball, Patrick McClure, Joseph Chappell, Theocharis Tsoleridis, Nadine Holmes, Matthew Carlisle, Christopher Moore, Fei Sang, Johnny Debebe, Victoria Wright, Matthew Loose
EPI_ISL_425537 hCoV-19/England/NOTT-10E3BD/2020 Europe / United Kingdom / England 2020-03-21 Queens Medical Centre, Clinical Microbiology Department / DeepSeq Nottingham COVID-19 Genomics UK (COG-UK) Consortium Gemma Clark, Wendy Smith, Manjinder Khakh, Hannah Howson-Wells, Jonathan Ball, Patrick McClure, Joseph Chappell, Theocharis Tsoleridis, Nadine Holmes, Matthew Carlisle, Christopher Moore, Fei Sang, Johnny Debebe, Victoria Wright, Matthew Loose
EPI_ISL_425548 hCoV-19/England/NOTT-10E46F/2020 Europe / United Kingdom / England 2020-03-22 Queens Medical Centre, Clinical Microbiology Department / DeepSeq Nottingham COVID-19 Genomics UK (COG-UK) Consortium Gemma Clark, Wendy Smith, Manjinder Khakh, Hannah Howson-Wells, Jonathan Ball, Patrick McClure, Joseph Chappell, Theocharis Tsoleridis, Nadine Holmes, Matthew Carlisle, Christopher Moore, Fei Sang, Johnny Debebe, Victoria Wright, Matthew Loose
EPI_ISL_425549 hCoV-19/England/NOTT-10E47E/2020 Europe / United Kingdom / England 2020-03-22 Queens Medical Centre, Clinical Microbiology Department / DeepSeq Nottingham COVID-19 Genomics UK (COG-UK) Consortium Gemma Clark, Wendy Smith, Manjinder Khakh, Hannah Howson-Wells, Jonathan Ball, Patrick McClure, Joseph Chappell, Theocharis Tsoleridis, Nadine Holmes, Matthew Carlisle, Christopher Moore, Fei Sang, Johnny Debebe, Victoria Wright, Matthew Loose
EPI_ISL_425551 hCoV-19/England/NOTT-10E49C/2020 Europe / United Kingdom / England 2020-03-22 Queens Medical Centre, Clinical Microbiology Department / DeepSeq Nottingham COVID-19 Genomics UK (COG-UK) Consortium Gemma Clark, Wendy Smith, Manjinder Khakh, Hannah Howson-Wells, Jonathan Ball, Patrick McClure, Joseph Chappell, Theocharis Tsoleridis, Nadine Holmes, Matthew Carlisle, Christopher Moore, Fei Sang, Johnny Debebe, Victoria Wright, Matthew Loose
EPI_ISL_425554 hCoV-19/England/NOTT-10E4C9/2020 Europe / United Kingdom / England 2020-03-22 Queens Medical Centre, Clinical Microbiology Department / DeepSeq Nottingham COVID-19 Genomics UK (COG-UK) Consortium Gemma Clark, Wendy Smith, Manjinder Khakh, Hannah Howson-Wells, Jonathan Ball, Patrick McClure, Joseph Chappell, Theocharis Tsoleridis, Nadine Holmes, Matthew Carlisle, Christopher Moore, Fei Sang, Johnny Debebe, Victoria Wright, Matthew Loose
EPI_ISL_425561 hCoV-19/England/NOTT-10E55D/2020 Europe / United Kingdom / England 2020-03-25 Queens Medical Centre, Clinical Microbiology Department / DeepSeq Nottingham COVID-19 Genomics UK (COG-UK)

Consortium	Gemma Clark, Wendy Smith, Manjinder Khakh, Hannah Howson-Wells, Jonathan Ball, Patrick McClure, Joseph Chappell, Theocharis Tsoleridis, Nadine Holmes, Matthew Carlisle, Christopher Moore, Fei Sang, Johnny Debebe, Victoria Wright, Matthew Loose
EPI_ISL_425562	hCoV-19/England/NOTT-10E57B/2020	Europe / United Kingdom / England	2020-03-25	Queens Medical Centre, Clinical Microbiology Department / DeepSeq Nottingham	COVID-19 Genomics UK (COG-UK) Consortium	Gemma Clark, Wendy Smith, Manjinder Khakh, Hannah Howson-Wells, Jonathan Ball, Patrick McClure, Joseph Chappell, Theocharis Tsoleridis, Nadine Holmes, Matthew Carlisle, Christopher Moore, Fei Sang, Johnny Debebe, Victoria Wright, Matthew Loose
EPI_ISL_425563	hCoV-19/England/NOTT-10E58A/2020	Europe / United Kingdom / England	2020-03-26	Queens Medical Centre, Clinical Microbiology Department / DeepSeq Nottingham	COVID-19 Genomics UK (COG-UK) Consortium	Gemma Clark, Wendy Smith, Manjinder Khakh, Hannah Howson-Wells, Jonathan Ball, Patrick McClure, Joseph Chappell, Theocharis Tsoleridis, Nadine Holmes, Matthew Carlisle, Christopher Moore, Fei Sang, Johnny Debebe, Victoria Wright, Matthew Loose
EPI_ISL_425565	hCoV-19/England/NOTT-10E5A8/2020	Europe / United Kingdom / England	2020-03-26	Queens Medical Centre, Clinical Microbiology Department / DeepSeq Nottingham	COVID-19 Genomics UK (COG-UK) Consortium	Gemma Clark, Wendy Smith, Manjinder Khakh, Hannah Howson-Wells, Jonathan Ball, Patrick McClure, Joseph Chappell, Theocharis Tsoleridis, Nadine Holmes, Matthew Carlisle, Christopher Moore, Fei Sang, Johnny Debebe, Victoria Wright, Matthew Loose
EPI_ISL_425570	hCoV-19/England/NOTT-10E5F3/2020	Europe / United Kingdom / England	2020-03-23	Queens Medical Centre, Clinical Microbiology Department / DeepSeq Nottingham	COVID-19 Genomics UK (COG-UK) Consortium	Gemma Clark, Wendy Smith, Manjinder Khakh, Hannah Howson-Wells, Jonathan Ball, Patrick McClure, Joseph Chappell, Theocharis Tsoleridis, Nadine Holmes, Matthew Carlisle, Christopher Moore, Fei Sang, Johnny Debebe, Victoria Wright, Matthew Loose
EPI_ISL_425571	hCoV-19/England/NOTT-10E61E/2020	Europe / United Kingdom / England	2020-03-23	Queens Medical Centre, Clinical Microbiology Department / DeepSeq Nottingham	COVID-19 Genomics UK (COG-UK) Consortium	Gemma Clark, Wendy Smith, Manjinder Khakh, Hannah Howson-Wells, Jonathan Ball, Patrick McClure, Joseph Chappell, Theocharis Tsoleridis, Nadine Holmes, Matthew Carlisle, Christopher Moore, Fei Sang, Johnny Debebe, Victoria Wright, Matthew Loose
EPI_ISL_425573	hCoV-19/England/NOTT-10E64B/2020	Europe / United Kingdom / England	2020-03-23	Queens Medical Centre, Clinical Microbiology Department / DeepSeq Nottingham	COVID-19 Genomics UK (COG-UK) Consortium	Gemma Clark, Wendy Smith, Manjinder Khakh, Hannah Howson-Wells, Jonathan Ball, Patrick McClure, Joseph Chappell, Theocharis Tsoleridis, Nadine Holmes, Matthew Carlisle, Christopher Moore, Fei Sang, Johnny Debebe, Victoria Wright, Matthew Loose
EPI_ISL_425575	hCoV-19/England/NOTT-10E669/2020	Europe / United Kingdom / England	2020-03-24	Queens Medical Centre, Clinical Microbiology Department / DeepSeq Nottingham	COVID-19 Genomics UK (COG-UK) Consortium	Gemma Clark, Wendy Smith, Manjinder Khakh, Hannah Howson-Wells, Jonathan Ball, Patrick McClure, Joseph Chappell, Theocharis Tsoleridis, Nadine Holmes, Matthew Carlisle, Christopher Moore, Fei Sang, Johnny Debebe, Victoria Wright, Matthew Loose
EPI_ISL_425576	hCoV-19/England/NOTT-10E678/2020	Europe / United Kingdom / England	2020-03-24	Queens Medical Centre, Clinical Microbiology Department / DeepSeq Nottingham	COVID-19 Genomics UK (COG-UK) Consortium	Gemma Clark, Wendy Smith, Manjinder Khakh, Hannah Howson-Wells, Jonathan Ball, Patrick McClure, Joseph Chappell, Theocharis Tsoleridis, Nadine Holmes, Matthew Carlisle, Christopher Moore, Fei Sang, Johnny Debebe, Victoria Wright, Matthew Loose
EPI_ISL_425581	hCoV-19/England/NOTT-10E6C3/2020	Europe / United Kingdom / England	2020-03-24	Queens Medical Centre, Clinical Microbiology Department / DeepSeq Nottingham	COVID-19 Genomics UK (COG-UK) Consortium	Gemma Clark, Wendy Smith, Manjinder Khakh, Hannah Howson-Wells, Jonathan Ball, Patrick McClure, Joseph Chappell, Theocharis Tsoleridis, Nadine Holmes, Matthew Carlisle, Christopher Moore, Fei Sang, Johnny Debebe, Victoria Wright, Matthew Loose
EPI_ISL_425584	hCoV-19/England/NOTT-10E71B/2020	Europe / United Kingdom / England	2020-03-24	Queens Medical Centre, Clinical Microbiology Department / DeepSeq Nottingham	COVID-19 Genomics UK (COG-UK) Consortium	Gemma Clark, Wendy Smith, Manjinder Khakh, Hannah Howson-Wells, Jonathan Ball, Patrick McClure, Joseph Chappell, Theocharis Tsoleridis, Nadine Holmes, Matthew Carlisle, Christopher Moore, Fei Sang, Johnny Debebe, Victoria Wright, Matthew Loose
EPI_ISL_425586	hCoV-19/England/NOTT-10E739/2020	Europe / United Kingdom / England	2020-03-24	Queens Medical Centre, Clinical Microbiology Department / DeepSeq Nottingham	COVID-19 Genomics UK (COG-UK) Consortium	Gemma Clark, Wendy Smith, Manjinder Khakh, Hannah Howson-Wells, Jonathan Ball, Patrick McClure, Joseph Chappell, Theocharis Tsoleridis, Nadine Holmes, Matthew Carlisle, Christopher Moore, Fei Sang, Johnny Debebe, Victoria Wright, Matthew Loose
EPI_ISL_425587	hCoV-19/England/NOTT-10E748/2020	Europe / United Kingdom / England	2020-03-24	Queens Medical Centre, Clinical Microbiology Department / DeepSeq Nottingham	COVID-19 Genomics UK (COG-UK) Consortium	Gemma Clark, Wendy Smith, Manjinder Khakh, Hannah Howson-Wells, Jonathan Ball, Patrick McClure, Joseph Chappell, Theocharis Tsoleridis, Nadine Holmes, Matthew Carlisle, Christopher Moore, Fei Sang, Johnny Debebe, Victoria Wright, Matthew Loose
EPI_ISL_425593	hCoV-19/England/NOTT-10E7A2/2020	Europe / United Kingdom / England	2020-03-25	Queens Medical Centre, Clinical Microbiology Department / DeepSeq Nottingham	COVID-19 Genomics UK (COG-UK) Consortium	Gemma Clark, Wendy Smith, Manjinder Khakh, Hannah Howson-Wells, Jonathan Ball, Patrick McClure, Joseph Chappell, Theocharis Tsoleridis, Nadine Holmes, Matthew Carlisle, Christopher Moore, Fei Sang, Johnny Debebe, Victoria Wright, Matthew Loose
EPI_ISL_425700	hCoV-19/Scotland/CVR161/2020	Europe / United Kingdom / Scotland	2020-03-17	West of Scotland Specialist Virology Centre, NHSGGC / MRC-University of Glasgow Centre for Virus Research	COVID-

19 Genomics UK (COG-UK) Consortium	Ana da Silva Filipe, Kathy Smollett, Stephen Carmichael, Natasha Johnson, Daniel Mair, Lily Tong, Jenna Nichols; Sarah McDonald; Richard Orton, Joseph Hughes, Sreenu Vattipally, David L Robertson; Kathy Li, Natasha Jesudason, Rajiv Shah, James Shepherd, Antonia Ho, Emma Thomson; Alasdair MacLean, Rory Gunson.
EPI_ISL_425701	hCoV-19/Scotland/CVR162/2020	Europe / United Kingdom / Scotland	2020-03-17	West of Scotland Specialist Virology Centre, NHSGGC / MRC-University of Glasgow Centre for Virus Research	COVID-19 Genomics UK (COG-UK) Consortium	Ana da Silva Filipe, Kathy Smollett, Stephen Carmichael, Natasha Johnson, Daniel Mair, Lily Tong, Jenna Nichols; Sarah McDonald; Richard Orton, Joseph Hughes, Sreenu Vattipally, David L Robertson; Kathy Li, Natasha Jesudason, Rajiv Shah, James Shepherd, Antonia Ho, Emma Thomson; Alasdair MacLean, Rory Gunson.
EPI_ISL_426800	hCoV-19/Australia/VIC534/2020	Oceania / Australia / Victoria	2020-03-25	Victorian Infectious Diseases Reference Laboratory (VIDRL)	Microbiological Diagnostic Unit Public Health Laboratory and Victorian Infectious Diseases Reference Laboratory, Doherty Institute	Caly L., Seemann T., Sait, M., Schultz M., Druce J., Sherry, N.
EPI_ISL_426801	hCoV-19/Australia/VIC535/2020	Oceania / Australia / Victoria	2020-03-25	Victorian Infectious Diseases Reference Laboratory (VIDRL)	Microbiological Diagnostic Unit Public Health Laboratory and Victorian Infectious Diseases Reference Laboratory, Doherty Institute	Caly L., Seemann T., Sait, M., Schultz M., Druce J., Sherry, N.
EPI_ISL_426802	hCoV-19/Australia/VIC536/2020	Oceania / Australia / Victoria	2020-03-24	Victorian Infectious Diseases Reference Laboratory (VIDRL)	Microbiological Diagnostic Unit Public Health Laboratory and Victorian Infectious Diseases Reference Laboratory, Doherty Institute	Caly L., Seemann T., Sait, M., Schultz M., Druce J., Sherry, N.
EPI_ISL_426803	hCoV-19/Australia/VIC537/2020	Oceania / Australia / Victoria	2020-03-24	Victorian Infectious Diseases Reference Laboratory (VIDRL)	Microbiological Diagnostic Unit Public Health Laboratory and Victorian Infectious Diseases Reference Laboratory, Doherty Institute	Caly L., Seemann T., Sait, M., Schultz M., Druce J., Sherry, N.
EPI_ISL_426804	hCoV-19/Australia/VIC538/2020	Oceania / Australia / Victoria	2020-03-23	Victorian Infectious Diseases Reference Laboratory (VIDRL)	Microbiological Diagnostic Unit Public Health Laboratory and Victorian Infectious Diseases Reference Laboratory, Doherty Institute	Caly L., Seemann T., Sait, M., Schultz M., Druce J., Sherry, N.
EPI_ISL_426805	hCoV-19/Australia/VIC540/2020	Oceania / Australia / Victoria	2020-03-24	Victorian Infectious Diseases Reference Laboratory (VIDRL)	Microbiological Diagnostic Unit Public Health Laboratory and Victorian Infectious Diseases Reference Laboratory, Doherty Institute	Caly L., Seemann T., Sait, M., Schultz M., Druce J., Sherry, N.
EPI_ISL_426806	hCoV-19/Australia/VIC541/2020	Oceania / Australia / Victoria	2020-03-24	Victorian Infectious Diseases Reference Laboratory (VIDRL)	Microbiological Diagnostic Unit Public Health Laboratory and Victorian Infectious Diseases Reference Laboratory, Doherty Institute	Caly L., Seemann T., Sait, M., Schultz M., Druce J., Sherry, N.
EPI_ISL_426807	hCoV-19/Australia/VIC544/2020	Oceania / Australia / Victoria	2020-03-23	Victorian Infectious Diseases Reference Laboratory (VIDRL)	Microbiological Diagnostic Unit Public Health Laboratory and Victorian Infectious Diseases Reference Laboratory, Doherty Institute	Caly L., Seemann T., Sait, M., Schultz M., Druce J., Sherry, N.
EPI_ISL_426809	hCoV-19/Australia/VIC546/2020	Oceania / Australia / Victoria	2020-03-24	Victorian Infectious Diseases Reference Laboratory (VIDRL)	Microbiological Diagnostic Unit Public Health Laboratory and Victorian Infectious Diseases Reference Laboratory, Doherty Institute	Caly L., Seemann T., Sait, M., Schultz M., Druce J., Sherry, N.
EPI_ISL_426810	hCoV-19/Australia/VIC547/2020	Oceania / Australia / Victoria	2020-03-25	Victorian Infectious Diseases Reference Laboratory (VIDRL)	Microbiological Diagnostic Unit Public Health Laboratory and Victorian Infectious Diseases Reference Laboratory, Doherty Institute	Caly L., Seemann T., Sait, M., Schultz M., Druce J., Sherry, N.
EPI_ISL_426811	hCoV-19/Australia/VIC548/2020	Oceania / Australia / Victoria	2020-03-25	Victorian Infectious Diseases Reference Laboratory (VIDRL)	Microbiological Diagnostic Unit Public Health Laboratory and Victorian Infectious Diseases Reference Laboratory, Doherty Institute	Caly L., Seemann T., Sait, M., Schultz M., Druce J., Sherry, N.
EPI_ISL_426812	hCoV-19/Australia/VIC549/2020	Oceania / Australia / Victoria	2020-03-23	Victorian Infectious Diseases Reference Laboratory (VIDRL)	Microbiological Diagnostic Unit Public Health Laboratory and Victorian Infectious Diseases Reference Laboratory, Doherty Institute	Caly L., Seemann T., Sait, M., Schultz M., Druce J., Sherry, N.
EPI_ISL_426813	hCoV-19/Australia/VIC551/2020	Oceania / Australia / Victoria	2020-03-25	Victorian Infectious Diseases Reference Laboratory (VIDRL)	Microbiological Diagnostic Unit Public Health Laboratory and Victorian Infectious Diseases Reference Laboratory, Doherty Institute	Caly L., Seemann T., Sait, M., Schultz M., Druce J., Sherry, N.
EPI_ISL_426816	hCoV-19/Australia/VIC555/2020	Oceania / Australia / Victoria	2020-03-25	Victorian Infectious Diseases Reference Laboratory (VIDRL)	Microbiological Diagnostic Unit Public Health Laboratory and Victorian Infectious Diseases Reference Laboratory, Doherty Institute	Caly L., Seemann T., Sait, M., Schultz M., Druce J., Sherry, N.
EPI_ISL_426817	hCoV-19/Australia/VIC556/2020	Oceania / Australia / Victoria	2020-03-25	Victorian Infectious Diseases Reference Laboratory (VIDRL)	Microbiological Diagnostic Unit Public Health Laboratory and Victorian Infectious Diseases Reference Laboratory, Doherty Institute	Caly L., Seemann T., Sait, M., Schultz M., Druce J., Sherry, N.

EPI_ISL_426819 hCoV-19/Australia/VIC558/2020 Oceania / Australia / Victoria 2020-03-25 Victorian Infectious Diseases Reference Laboratory (VIDRL) Microbiological Diagnostic Unit Public Health Laboratory and Victorian Infectious Diseases Reference Laboratory, Doherty Institute Caly L., Seemann T., Sait, M., Schultz M., Druce J., Sherry, N.
EPI_ISL_426821 hCoV-19/Australia/VIC560/2020 Oceania / Australia / Victoria 2020-03-25 Victorian Infectious Diseases Reference Laboratory (VIDRL) Microbiological Diagnostic Unit Public Health Laboratory and Victorian Infectious Diseases Reference Laboratory, Doherty Institute Caly L., Seemann T., Sait, M., Schultz M., Druce J., Sherry, N.
EPI_ISL_426822 hCoV-19/Australia/VIC561/2020 Oceania / Australia / Victoria 2020-03-25 Victorian Infectious Diseases Reference Laboratory (VIDRL) Microbiological Diagnostic Unit Public Health Laboratory and Victorian Infectious Diseases Reference Laboratory, Doherty Institute Caly L., Seemann T., Sait, M., Schultz M., Druce J., Sherry, N.
EPI_ISL_426824 hCoV-19/Australia/VIC563/2020 Oceania / Australia / Victoria 2020-03-24 Victorian Infectious Diseases Reference Laboratory (VIDRL) Microbiological Diagnostic Unit Public Health Laboratory and Victorian Infectious Diseases Reference Laboratory, Doherty Institute Caly L., Seemann T., Sait, M., Schultz M., Druce J., Sherry, N.
EPI_ISL_426825 hCoV-19/Australia/VIC565/2020 Oceania / Australia / Victoria 2020-03-25 Victorian Infectious Diseases Reference Laboratory (VIDRL) Microbiological Diagnostic Unit Public Health Laboratory and Victorian Infectious Diseases Reference Laboratory, Doherty Institute Caly L., Seemann T., Sait, M., Schultz M., Druce J., Sherry, N.
EPI_ISL_426826 hCoV-19/Australia/VIC566/2020 Oceania / Australia / Victoria 2020-03-25 Victorian Infectious Diseases Reference Laboratory (VIDRL) Microbiological Diagnostic Unit Public Health Laboratory and Victorian Infectious Diseases Reference Laboratory, Doherty Institute Caly L., Seemann T., Sait, M., Schultz M., Druce J., Sherry, N.
EPI_ISL_426827 hCoV-19/Australia/VIC567/2020 Oceania / Australia / Victoria 2020-03-25 Victorian Infectious Diseases Reference Laboratory (VIDRL) Microbiological Diagnostic Unit Public Health Laboratory and Victorian Infectious Diseases Reference Laboratory, Doherty Institute Caly L., Seemann T., Sait, M., Schultz M., Druce J., Sherry, N.
EPI_ISL_426828 hCoV-19/Australia/VIC568/2020 Oceania / Australia / Victoria 2020-03-25 Victorian Infectious Diseases Reference Laboratory (VIDRL) Microbiological Diagnostic Unit Public Health Laboratory and Victorian Infectious Diseases Reference Laboratory, Doherty Institute Caly L., Seemann T., Sait, M., Schultz M., Druce J., Sherry, N.
EPI_ISL_426831 hCoV-19/Australia/VIC571/2020 Oceania / Australia / Victoria 2020-03-25 Victorian Infectious Diseases Reference Laboratory (VIDRL) Microbiological Diagnostic Unit Public Health Laboratory and Victorian Infectious Diseases Reference Laboratory, Doherty Institute Caly L., Seemann T., Sait, M., Schultz M., Druce J., Sherry, N.
EPI_ISL_426832 hCoV-19/Australia/VIC572/2020 Oceania / Australia / Victoria 2020-03-25 Victorian Infectious Diseases Reference Laboratory (VIDRL) Microbiological Diagnostic Unit Public Health Laboratory and Victorian Infectious Diseases Reference Laboratory, Doherty Institute Caly L., Seemann T., Sait, M., Schultz M., Druce J., Sherry, N.
EPI_ISL_426833 hCoV-19/Australia/VIC574/2020 Oceania / Australia / Victoria 2020-03-26 Victorian Infectious Diseases Reference Laboratory (VIDRL) Microbiological Diagnostic Unit Public Health Laboratory and Victorian Infectious Diseases Reference Laboratory, Doherty Institute Caly L., Seemann T., Sait, M., Schultz M., Druce J., Sherry, N.
EPI_ISL_426835 hCoV-19/Australia/VIC576/2020 Oceania / Australia / Victoria 2020-03-26 Victorian Infectious Diseases Reference Laboratory (VIDRL) Microbiological Diagnostic Unit Public Health Laboratory and Victorian Infectious Diseases Reference Laboratory, Doherty Institute Caly L., Seemann T., Sait, M., Schultz M., Druce J., Sherry, N.
EPI_ISL_426836 hCoV-19/Australia/VIC578/2020 Oceania / Australia / Victoria 2020-03-26 Victorian Infectious Diseases Reference Laboratory (VIDRL) Microbiological Diagnostic Unit Public Health Laboratory and Victorian Infectious Diseases Reference Laboratory, Doherty Institute Caly L., Seemann T., Sait, M., Schultz M., Druce J., Sherry, N.
EPI_ISL_426839 hCoV-19/Australia/VIC582/2020 Oceania / Australia / Victoria 2020-03-25 Victorian Infectious Diseases Reference Laboratory (VIDRL) Microbiological Diagnostic Unit Public Health Laboratory and Victorian Infectious Diseases Reference Laboratory, Doherty Institute Caly L., Seemann T., Sait, M., Schultz M., Druce J., Sherry, N.
EPI_ISL_426840 hCoV-19/Australia/VIC583/2020 Oceania / Australia / Victoria 2020-03-25 Victorian Infectious Diseases Reference Laboratory (VIDRL) Microbiological Diagnostic Unit Public Health Laboratory and Victorian Infectious Diseases Reference Laboratory, Doherty Institute Caly L., Seemann T., Sait, M., Schultz M., Druce J., Sherry, N.
EPI_ISL_426841 hCoV-19/Australia/VIC584/2020 Oceania / Australia / Victoria 2020-03-25 Victorian Infectious Diseases Reference Laboratory (VIDRL) Microbiological Diagnostic Unit Public Health Laboratory and Victorian Infectious Diseases Reference Laboratory, Doherty Institute Caly L., Seemann T., Sait, M., Schultz M., Druce J., Sherry, N.
EPI_ISL_426842 hCoV-19/Australia/VIC585/2020 Oceania / Australia / Victoria 2020-03-25 Victorian Infectious Diseases Reference Laboratory (VIDRL) Microbiological Diagnostic Unit Public Health Laboratory and Victorian Infectious Diseases Reference Laboratory, Doherty Institute Caly L., Seemann T., Sait, M., Schultz M., Druce J., Sherry, N.
EPI_ISL_426844 hCoV-19/Australia/VIC587/2020 Oceania / Australia / Victoria 2020-03-25 Victorian Infectious Diseases Reference Laboratory (VIDRL) Microbiological Diagnostic Unit Public Health

Laboratory and Victorian Infectious Diseases Reference Laboratory, Doherty Institute Caly L., Seemann T., Sait, M., Schultz M., Druce J., Sherry, N.
EPI_ISL_426846 hCoV-19/Australia/VIC589/2020 Oceania / Australia / Victoria 2020-03-22 Victorian Infectious Diseases Reference Laboratory (VIDRL) Microbiological Diagnostic Unit Public Health Laboratory and Victorian Infectious Diseases Reference Laboratory, Doherty Institute Caly L., Seemann T., Sait, M., Schultz M., Druce J., Sherry, N.
EPI_ISL_426847 hCoV-19/Australia/VIC590/2020 Oceania / Australia / Victoria 2020-03-22 Victorian Infectious Diseases Reference Laboratory (VIDRL) Microbiological Diagnostic Unit Public Health Laboratory and Victorian Infectious Diseases Reference Laboratory, Doherty Institute Caly L., Seemann T., Sait, M., Schultz M., Druce J., Sherry, N.
EPI_ISL_426848 hCoV-19/Australia/VIC591/2020 Oceania / Australia / Victoria 2020-03-22 Victorian Infectious Diseases Reference Laboratory (VIDRL) Microbiological Diagnostic Unit Public Health Laboratory and Victorian Infectious Diseases Reference Laboratory, Doherty Institute Caly L., Seemann T., Sait, M., Schultz M., Druce J., Sherry, N.
EPI_ISL_426850 hCoV-19/Australia/VIC593/2020 Oceania / Australia / Victoria 2020-03-23 Victorian Infectious Diseases Reference Laboratory (VIDRL) Microbiological Diagnostic Unit Public Health Laboratory and Victorian Infectious Diseases Reference Laboratory, Doherty Institute Caly L., Seemann T., Sait, M., Schultz M., Druce J., Sherry, N.
EPI_ISL_426851 hCoV-19/Australia/VIC594/2020 Oceania / Australia / Victoria 2020-03-25 Victorian Infectious Diseases Reference Laboratory (VIDRL) Microbiological Diagnostic Unit Public Health Laboratory and Victorian Infectious Diseases Reference Laboratory, Doherty Institute Caly L., Seemann T., Sait, M., Schultz M., Druce J., Sherry, N.
EPI_ISL_426852 hCoV-19/Australia/VIC595/2020 Oceania / Australia / Victoria 2020-03-25 Victorian Infectious Diseases Reference Laboratory (VIDRL) Microbiological Diagnostic Unit Public Health Laboratory and Victorian Infectious Diseases Reference Laboratory, Doherty Institute Caly L., Seemann T., Sait, M., Schultz M., Druce J., Sherry, N.
EPI_ISL_426853 hCoV-19/Australia/VIC596/2020 Oceania / Australia / Victoria 2020-03-25 Victorian Infectious Diseases Reference Laboratory (VIDRL) Microbiological Diagnostic Unit Public Health Laboratory and Victorian Infectious Diseases Reference Laboratory, Doherty Institute Caly L., Seemann T., Sait, M., Schultz M., Druce J., Sherry, N.
EPI_ISL_426854 hCoV-19/Australia/VIC597/2020 Oceania / Australia / Victoria 2020-03-25 Victorian Infectious Diseases Reference Laboratory (VIDRL) Microbiological Diagnostic Unit Public Health Laboratory and Victorian Infectious Diseases Reference Laboratory, Doherty Institute Caly L., Seemann T., Sait, M., Schultz M., Druce J., Sherry, N.
EPI_ISL_426855 hCoV-19/Australia/VIC598/2020 Oceania / Australia / Victoria 2020-03-25 Victorian Infectious Diseases Reference Laboratory (VIDRL) Microbiological Diagnostic Unit Public Health Laboratory and Victorian Infectious Diseases Reference Laboratory, Doherty Institute Caly L., Seemann T., Sait, M., Schultz M., Druce J., Sherry, N.
EPI_ISL_426856 hCoV-19/Australia/VIC599/2020 Oceania / Australia / Victoria 2020-03-25 Victorian Infectious Diseases Reference Laboratory (VIDRL) Microbiological Diagnostic Unit Public Health Laboratory and Victorian Infectious Diseases Reference Laboratory, Doherty Institute Caly L., Seemann T., Sait, M., Schultz M., Druce J., Sherry, N.
EPI_ISL_426858 hCoV-19/Australia/VIC603/2020 Oceania / Australia / Victoria 2020-03-26 Victorian Infectious Diseases Reference Laboratory (VIDRL) Microbiological Diagnostic Unit Public Health Laboratory and Victorian Infectious Diseases Reference Laboratory, Doherty Institute Caly L., Seemann T., Sait, M., Schultz M., Druce J., Sherry, N.
EPI_ISL_426860 hCoV-19/Australia/VIC605/2020 Oceania / Australia / Victoria 2020-03-27 Victorian Infectious Diseases Reference Laboratory (VIDRL) Microbiological Diagnostic Unit Public Health Laboratory and Victorian Infectious Diseases Reference Laboratory, Doherty Institute Caly L., Seemann T., Sait, M., Schultz M., Druce J., Sherry, N.
EPI_ISL_426861 hCoV-19/Australia/VIC606/2020 Oceania / Australia / Victoria 2020-03-27 Victorian Infectious Diseases Reference Laboratory (VIDRL) Microbiological Diagnostic Unit Public Health Laboratory and Victorian Infectious Diseases Reference Laboratory, Doherty Institute Caly L., Seemann T., Sait, M., Schultz M., Druce J., Sherry, N.
EPI_ISL_426862 hCoV-19/Australia/VIC607/2020 Oceania / Australia / Victoria 2020-03-27 Victorian Infectious Diseases Reference Laboratory (VIDRL) Microbiological Diagnostic Unit Public Health Laboratory and Victorian Infectious Diseases Reference Laboratory, Doherty Institute Caly L., Seemann T., Sait, M., Schultz M., Druce J., Sherry, N.
EPI_ISL_426864 hCoV-19/Australia/VIC610/2020 Oceania / Australia / Victoria 2020-03-26 Victorian Infectious Diseases Reference Laboratory (VIDRL) Microbiological Diagnostic Unit Public Health Laboratory and Victorian Infectious Diseases Reference Laboratory, Doherty Institute Caly L., Seemann T., Sait, M., Schultz M., Druce J., Sherry, N.
EPI_ISL_426865 hCoV-19/Australia/VIC611/2020 Oceania / Australia / Victoria 2020-03-27 Victorian Infectious Diseases Reference Laboratory (VIDRL) Microbiological Diagnostic Unit Public Health Laboratory and Victorian Infectious Diseases Reference Laboratory, Doherty Institute Caly L., Seemann T., Sait, M., Schultz M., Druce J., Sherry, N.
EPI_ISL_426866 hCoV-19/Australia/VIC612/2020 Oceania / Australia / Victoria 2020-03-26 Victorian Infectious Diseases Reference Laboratory (VIDRL) Microbiological Diagnostic Unit Public Health Laboratory and Victorian Infectious Diseases Reference Laboratory, Doherty Institute Caly L., Seemann T., Sait, M., Schultz M., Druce J., Sherry, N.

EPI_ISL_426870 hCoV-19/Australia/VIC615/2020 Oceania / Australia / Victoria 2020-03-28 Victorian Infectious Diseases Reference Laboratory (VIDRL) Microbiological Diagnostic Unit Public Health Laboratory and Victorian Infectious Diseases Reference Laboratory, Doherty Institute Caly L., Seemann T., Sait, M., Schultz M., Druce J., Sherry, N.
EPI_ISL_426872 hCoV-19/Australia/VIC617/2020 Oceania / Australia / Victoria 2020-03-26 Victorian Infectious Diseases Reference Laboratory (VIDRL) Microbiological Diagnostic Unit Public Health Laboratory and Victorian Infectious Diseases Reference Laboratory, Doherty Institute Caly L., Seemann T., Sait, M., Schultz M., Druce J., Sherry, N.
EPI_ISL_426873 hCoV-19/Australia/VIC629/2020 Oceania / Australia / Victoria 2020-03-25 Victorian Infectious Diseases Reference Laboratory (VIDRL) Microbiological Diagnostic Unit Public Health Laboratory and Victorian Infectious Diseases Reference Laboratory, Doherty Institute Caly L., Seemann T., Sait, M., Schultz M., Druce J., Sherry, N.
EPI_ISL_426874 hCoV-19/Australia/VIC630/2020 Oceania / Australia / Victoria 2020-03-26 Victorian Infectious Diseases Reference Laboratory (VIDRL) Microbiological Diagnostic Unit Public Health Laboratory and Victorian Infectious Diseases Reference Laboratory, Doherty Institute Caly L., Seemann T., Sait, M., Schultz M., Druce J., Sherry, N.
EPI_ISL_426875 hCoV-19/Australia/VIC633/2020 Oceania / Australia / Victoria 2020-03-27 Victorian Infectious Diseases Reference Laboratory (VIDRL) Microbiological Diagnostic Unit Public Health Laboratory and Victorian Infectious Diseases Reference Laboratory, Doherty Institute Caly L., Seemann T., Sait, M., Schultz M., Druce J., Sherry, N.
EPI_ISL_426876 hCoV-19/Australia/VIC635/2020 Oceania / Australia / Victoria 2020-03-28 Victorian Infectious Diseases Reference Laboratory (VIDRL) Microbiological Diagnostic Unit Public Health Laboratory and Victorian Infectious Diseases Reference Laboratory, Doherty Institute Caly L., Seemann T., Sait, M., Schultz M., Druce J., Sherry, N.
EPI_ISL_426877 hCoV-19/Australia/VIC636/2020 Oceania / Australia / Victoria 2020-03-28 Victorian Infectious Diseases Reference Laboratory (VIDRL) Microbiological Diagnostic Unit Public Health Laboratory and Victorian Infectious Diseases Reference Laboratory, Doherty Institute Caly L., Seemann T., Sait, M., Schultz M., Druce J., Sherry, N.
EPI_ISL_426878 hCoV-19/Australia/VIC701/2020 Oceania / Australia / Victoria 2020-03-21 Victorian Infectious Diseases Reference Laboratory (VIDRL) Microbiological Diagnostic Unit Public Health Laboratory and Victorian Infectious Diseases Reference Laboratory, Doherty Institute Caly L., Seemann T., Sait, M., Schultz M., Druce J., Sherry, N.
EPI_ISL_426881 hCoV-19/Australia/VIC912/2020 Oceania / Australia / Victoria 2020-03-24 Victorian Infectious Diseases Reference Laboratory (VIDRL) Microbiological Diagnostic Unit Public Health Laboratory and Victorian Infectious Diseases Reference Laboratory, Doherty Institute Caly L., Seemann T., Sait, M., Schultz M., Druce J., Sherry, N.
EPI_ISL_426882 hCoV-19/Australia/VIC913/2020 Oceania / Australia / Victoria 2020-03-24 Victorian Infectious Diseases Reference Laboratory (VIDRL) Microbiological Diagnostic Unit Public Health Laboratory and Victorian Infectious Diseases Reference Laboratory, Doherty Institute Caly L., Seemann T., Sait, M., Schultz M., Druce J., Sherry, N.
EPI_ISL_426883 hCoV-19/Czech Republic/IAB_1/2020 Europe / Czech Republic 2020-03-27 Motol University Hospital Institute of Applied Biotechnologies a.s. Petr Brož, Jan Geryk, Petr Klempt, Martin Kašný, Adam Novotný, Kateřina Kvapilová, Pavel Dřevínek, Petr Kvapil, Milan Macek
EPI_ISL_426884 hCoV-19/Czech Republic/IAB_4/2020 Europe / Czech Republic 2020-03-27 Motol University Hospital Institute of Applied Biotechnologies a.s. Petr Brož, Jan Geryk, Petr Klempt, Martin Kašný, Adam Novotný, Kateřina Kvapilová, Pavel Dřevínek, Petr Kvapil, Milan Macek
EPI_ISL_426885 hCoV-19/Czech Republic/IAB_8/2020 Europe / Czech Republic 2020-03-27 Motol University Hospital Institute of Applied Biotechnologies a.s. Petr Brož, Jan Geryk, Petr Klempt, Martin Kašný, Adam Novotný, Kateřina Kvapilová, Pavel Dřevínek, Petr Kvapil, Milan Macek
EPI_ISL_426886 hCoV-19/Czech Republic/IAB_9/2020 Europe / Czech Republic 2020-03-28 Motol University Hospital Institute of Applied Biotechnologies a.s. Petr Brož, Jan Geryk, Petr Klempt, Martin Kašný, Adam Novotný, Kateřina Kvapilová, Pavel Dřevínek, Petr Kvapil, Milan Macek
EPI_ISL_426887 hCoV-19/Czech Republic/IAB_10/2020 Europe / Czech Republic 2020-03-31 Motol University Hospital Institute of Applied Biotechnologies a.s. Petr Brož, Jan Geryk, Petr Klempt, Martin Kašný, Adam Novotný, Kateřina Kvapilová, Pavel Dřevínek, Petr Kvapil, Milan Macek
EPI_ISL_426888 hCoV-19/Czech Republic/IAB_12/2020 Europe / Czech Republic 2020-03-31 Motol University Hospital Institute of Applied Biotechnologies a.s. Petr Brož, Jan Geryk, Petr Klempt, Martin Kašný, Adam Novotný, Kateřina Kvapilová, Pavel Dřevínek, Petr Kvapil, Milan Macek
EPI_ISL_426889 hCoV-19/Czech Republic/IAB_14/2020 Europe / Czech Republic 2020-04-02 Motol University Hospital Institute of Applied Biotechnologies a.s. Petr Brož, Jan Geryk, Petr Klempt, Martin Kašný, Adam Novotný, Kateřina Kvapilová, Pavel Dřevínek, Petr Kvapil, Milan Macek
EPI_ISL_426890 hCoV-19/Czech Republic/IAB_15/2020 Europe / Czech Republic 2020-03-26 Motol University Hospital Institute of Applied Biotechnologies a.s. Petr Brož, Jan Geryk, Petr Klempt, Martin Kašný, Adam Novotný, Kateřina Kvapilová, Pavel Dřevínek, Petr Kvapil, Milan Macek
EPI_ISL_426891 hCoV-19/Czech Republic/IAB_16/2020 Europe / Czech Republic 2020-03-26 Motol University Hospital Institute of Applied Biotechnologies a.s. Petr Brož, Jan Geryk, Petr Klempt, Martin Kašný, Adam Novotný, Kateřina Kvapilová, Pavel Dřevínek, Petr Kvapil, Milan Macek
EPI_ISL_426892 hCoV-19/Czech Republic/IAB_17/2020 Europe / Czech Republic 2020-03-27 Motol University Hospital Institute of Applied Biotechnologies a.s. Petr Brož, Jan Geryk, Petr Klempt, Martin Kašný, Adam Novotný, Kateřina Kvapilová, Pavel Dřevínek, Petr Kvapil, Milan Macek

EPI_ISL_426893 hCoV-19/Czech Republic/IAB_18/2020 Europe / Czech Republic 2020-03-27 Motol University Hospital Institute of Applied Biotechnologies a.s. Petr Brož, Jan Geryk, Petr Klempt, Martin Kašný, Adam Novotný, Kateřina Kvapilová, Pavel Dřevínek, Petr Kvapil, Milan Macek
EPI_ISL_426894 hCoV-19/Czech Republic/IAB_20/2020 Europe / Czech Republic 2020-03-27 Motol University Hospital Institute of Applied Biotechnologies a.s. Petr Brož, Jan Geryk, Petr Klempt, Martin Kašný, Adam Novotný, Kateřina Kvapilová, Pavel Dřevínek, Petr Kvapil, Milan Macek
EPI_ISL_426895 hCoV-19/Czech Republic/IAB_21/2020 Europe / Czech Republic 2020-03-27 Motol University Hospital Institute of Applied Biotechnologies a.s. Petr Brož, Jan Geryk, Petr Klempt, Martin Kašný, Adam Novotný, Kateřina Kvapilová, Pavel Dřevínek, Petr Kvapil, Milan Macek
EPI_ISL_426896 hCoV-19/Czech Republic/IAB_22/2020 Europe / Czech Republic 2020-03-28 Motol University Hospital Institute of Applied Biotechnologies a.s. Petr Brož, Jan Geryk, Petr Klempt, Martin Kašný, Adam Novotný, Kateřina Kvapilová, Pavel Dřevínek, Petr Kvapil, Milan Macek
EPI_ISL_426897 hCoV-19/Czech Republic/IAB_23/2020 Europe / Czech Republic 2020-03-29 Motol University Hospital Institute of Applied Biotechnologies a.s. Petr Brož, Jan Geryk, Petr Klempt, Martin Kašný, Adam Novotný, Kateřina Kvapilová, Pavel Dřevínek, Petr Kvapil, Milan Macek
EPI_ISL_426898 hCoV-19/Australia/NT10/2020 Oceania / Australia / Northern territory 2020-03-26 Royal Darwin Hospital Pathology Microbiological Diagnostic Unit Public Health Laboratory and Victorian Infectious Diseases Reference Laboratory, Doherty Institute Meumann, E., Caly L., Seemann T., Sait, M., Schultz M., Druce J., Sherry, N.
EPI_ISL_426899 hCoV-19/Australia/NT11/2020 Oceania / Australia / Northern territory 2020-03-27 Royal Darwin Hospital Pathology Microbiological Diagnostic Unit Public Health Laboratory and Victorian Infectious Diseases Reference Laboratory, Doherty Institute Meumann, E., Caly L., Seemann T., Sait, M., Schultz M., Druce J., Sherry, N.
EPI_ISL_426900 hCoV-19/Australia/NT12/2020 Oceania / Australia / Northern territory 2020-03-25 Royal Darwin Hospital Pathology Microbiological Diagnostic Unit Public Health Laboratory and Victorian Infectious Diseases Reference Laboratory, Doherty Institute Meumann, E., Caly L., Seemann T., Sait, M., Schultz M., Druce J., Sherry, N.
EPI_ISL_426901 hCoV-19/Australia/NT13/2020 Oceania / Australia / Northern territory 2020-03-25 Royal Darwin Hospital Pathology Microbiological Diagnostic Unit Public Health Laboratory and Victorian Infectious Diseases Reference Laboratory, Doherty Institute Meumann, E., Caly L., Seemann T., Sait, M., Schultz M., Druce J., Sherry, N.
EPI_ISL_426904 hCoV-19/Australia/NT17/2020 Oceania / Australia / Northern territory 2020-03-24 Royal Darwin Hospital Pathology Microbiological Diagnostic Unit Public Health Laboratory and Victorian Infectious Diseases Reference Laboratory, Doherty Institute Meumann, E., Caly L., Seemann T., Sait, M., Schultz M., Druce J., Sherry, N.
EPI_ISL_426906 hCoV-19/Australia/VIC473/2020 Oceania / Australia / Victoria 2020-03-28 Microbiological Diagnostic Unit Public Health Laboratory Microbiological Diagnostic Unit Public Health Laboratory Seemann T., Schultz M., Sait, M., Sherry, N.
EPI_ISL_426907 hCoV-19/Australia/VIC475/2020 Oceania / Australia / Victoria 2020-03-28 Microbiological Diagnostic Unit Public Health Laboratory Microbiological Diagnostic Unit Public Health Laboratory Seemann T., Schultz M., Sait, M., Sherry, N.
EPI_ISL_426910 hCoV-19/Australia/VIC478/2020 Oceania / Australia / Victoria 2020-03-28 Microbiological Diagnostic Unit Public Health Laboratory Microbiological Diagnostic Unit Public Health Laboratory Seemann T., Schultz M., Sait, M., Sherry, N.
EPI_ISL_429074 hCoV-19/Guangzhou/GZMU0003/2020 Asia / China / Guangzhou 2020-02-07 The First Affiliated Hospital of Guangzhou Medical University BGI-shenzhen & The First Affiliated Hospital of Guangzhou Medical University Yanqun Wang, Daxi Wang, Lu Zhang, Wanying Sun, Zhaoyong Zhang et al.
EPI_ISL_429075 hCoV-19/Guangzhou/GZMU0008/2020 Asia / China / Guangzhou 2020-02-07 The First Affiliated Hospital of Guangzhou Medical University BGI-shenzhen & The First Affiliated Hospital of Guangzhou Medical University Yanqun Wang, Daxi Wang, Lu Zhang, Wanying Sun, Zhaoyong Zhang et al.
EPI_ISL_429076 hCoV-19/Guangzhou/GZMU0009/2020 Asia / China / Guangzhou 2020-02-07 The First Affiliated Hospital of Guangzhou Medical University BGI-shenzhen & The First Affiliated Hospital of Guangzhou Medical University
EPI_ISL_429077 hCoV-19/Guangzhou/GZMU0010/2020 Asia / China / Guangzhou 2020-02-07 The First Affiliated Hospital of Guangzhou Medical University BGI-shenzhen & The First Affiliated Hospital of Guangzhou Medical University Yanqun Wang, Daxi Wang, Lu Zhang, Wanying Sun, Zhaoyong Zhang et al.
EPI_ISL_429078 hCoV-19/Guangzhou/GZMU0013/2020 Asia / China / Guangzhou 2020-02-07 The First Affiliated Hospital of Guangzhou Medical University BGI-shenzhen & The First Affiliated Hospital of Guangzhou Medical University
EPI_ISL_429079 hCoV-19/Guangzhou/GZMU0017/2020 Asia / China / Guangzhou 2020-02-05 The First Affiliated Hospital of Guangzhou Medical University BGI-shenzhen & The First Affiliated Hospital of Guangzhou Medical University
EPI_ISL_429080 hCoV-19/Guangzhou/GZMU0019/2020 Asia / China / Guangzhou 2020-02-05 The First Affiliated Hospital of Guangzhou Medical University BGI-shenzhen & The First Affiliated Hospital of Guangzhou Medical University
EPI_ISL_429081 hCoV-19/Guangzhou/GZMU0020/2020 Asia / China / Guangzhou 2020-02-05 The First Affiliated Hospital of Guangzhou Medical University BGI-shenzhen & The First Affiliated Hospital of Guangzhou Medical University
EPI_ISL_429082 hCoV-19/Guangzhou/GZMU0023/2020 Asia / China / Guangzhou 2020-02-05 The First Affiliated Hospital of Guangzhou Medical University BGI-shenzhen & The First Affiliated Hospital of

Guangzhou Medical University   Yanqun Wang, Daxi Wang, Lu Zhang, Wanying Sun, Zhaoyong Zhang et al.
EPI_ISL_429083 hCoV-19/Guangzhou/GZMU0024/2020 Asia / China / Guangzhou   2020-02-05   The First Affiliated Hospital of Guangzhou Medical University   BGI-shenzhen & The First Affiliated Hospital of Guangzhou Medical University   Yanqun Wang, Daxi Wang, Lu Zhang, Wanying Sun, Zhaoyong Zhang et al.
EPI_ISL_429084 hCoV-19/Guangzhou/GZMU0025/2020 Asia / China / Guangzhou   2020-02-05   The First Affiliated Hospital of Guangzhou Medical University   BGI-shenzhen & The First Affiliated Hospital of Guangzhou Medical University
EPI_ISL_429085 hCoV-19/Guangzhou/GZMU0033/2020 Asia / China / Guangzhou   2020-02-01   The First Affiliated Hospital of Guangzhou Medical University   BGI-shenzhen & The First Affiliated Hospital of Guangzhou Medical University   Yanqun Wang, Daxi Wang, Lu Zhang, Wanying Sun, Zhaoyong Zhang et al.
EPI_ISL_429086 hCoV-19/Guangzhou/GZMU0034/2020 Asia / China / Guangzhou   2020-02-01   The First Affiliated Hospital of Guangzhou Medical University   BGI-shenzhen & The First Affiliated Hospital of Guangzhou Medical University
EPI_ISL_429088 hCoV-19/Guangzhou/GZMU0035/2020 Asia / China / Guangzhou   2020-02-01   The First Affiliated Hospital of Guangzhou Medical University   BGI-shenzhen & The First Affiliated Hospital of Guangzhou Medical University   Yanqun Wang, Daxi Wang, Lu Zhang, Wanying Sun, Zhaoyong Zhang et al.
EPI_ISL_429089 hCoV-19/Guangzhou/GZMU0037/2020 Asia / China / Guangzhou   2020-02-08   The First Affiliated Hospital of Guangzhou Medical University   BGI-shenzhen & The First Affiliated Hospital of Guangzhou Medical University   Yanqun Wang, Daxi Wang, Lu Zhang, Wanying Sun, Zhaoyong Zhang et al.
EPI_ISL_429090 hCoV-19/Guangzhou/GZMU0039/2020 Asia / China / Guangzhou   2020-02-08   The First Affiliated Hospital of Guangzhou Medical University   BGI-shenzhen & The First Affiliated Hospital of Guangzhou Medical University   Yanqun Wang, Daxi Wang, Lu Zhang, Wanying Sun, Zhaoyong Zhang et al.
EPI_ISL_429091 hCoV-19/Guangzhou/GZMU0041/2020 Asia / China / Guangzhou   2020-02-08   The First Affiliated Hospital of Guangzhou Medical University   BGI-shenzhen & The First Affiliated Hospital of Guangzhou Medical University   Yanqun Wang, Daxi Wang, Lu Zhang, Wanying Sun, Zhaoyong Zhang et al.
EPI_ISL_429092 hCoV-19/Guangzhou/GZMU0045/2020 Asia / China / Guangzhou   2020-02-08   The First Affiliated Hospital of Guangzhou Medical University   BGI-shenzhen & The First Affiliated Hospital of Guangzhou Medical University   Yanqun Wang, Daxi Wang, Lu Zhang, Wanying Sun, Zhaoyong Zhang et al.
EPI_ISL_429093 hCoV-19/Guangzhou/GZMU0046/2020 Asia / China / Guangzhou   2020-02-08   The First Affiliated Hospital of Guangzhou Medical University   BGI-shenzhen & The First Affiliated Hospital of Guangzhou Medical University   Yanqun Wang, Daxi Wang, Lu Zhang, Wanying Sun, Zhaoyong Zhang et al.
EPI_ISL_429094 hCoV-19/Guangzhou/GZMU0049/2020 Asia / China / Guangzhou   2020-02-08   The First Affiliated Hospital of Guangzhou Medical University   BGI-shenzhen & The First Affiliated Hospital of Guangzhou Medical University
EPI_ISL_429095 hCoV-19/Guangzhou/GZMU0050/2020 Asia / China / Guangzhou   2020-02-08   The First Affiliated Hospital of Guangzhou Medical University   BGI-shenzhen & The First Affiliated Hospital of Guangzhou Medical University
EPI_ISL_429096 hCoV-19/Guangzhou/GZMU0051/2020 Asia / China / Guangzhou   2020-01-29   The First Affiliated Hospital of Guangzhou Medical University   BGI-shenzhen & The First Affiliated Hospital of Guangzhou Medical University   Yanqun Wang, Daxi Wang, Lu Zhang, Wanying Sun, Zhaoyong Zhang et al.
EPI_ISL_429097 hCoV-19/Guangzhou/GZMU0052/2020 Asia / China / Guangzhou   2020-01-29   The First Affiliated Hospital of Guangzhou Medical University   BGI-shenzhen & The First Affiliated Hospital of Guangzhou Medical University   Yanqun Wang, Daxi Wang, Lu Zhang, Wanying Sun, Zhaoyong Zhang et al.
EPI_ISL_429098 hCoV-19/Guangzhou/GZMU0054/2020 Asia / China / Guangzhou   2020-01-29   The First Affiliated Hospital of Guangzhou Medical University   BGI-shenzhen & The First Affiliated Hospital of Guangzhou Medical University   Yanqun Wang, Daxi Wang, Lu Zhang, Wanying Sun, Zhaoyong Zhang et al.
EPI_ISL_429100 hCoV-19/Guangzhou/GZMU0057/2020 Asia / China / Guangzhou   2020-01-27   The First Affiliated Hospital of Guangzhou Medical University   BGI-shenzhen & The First Affiliated Hospital of Guangzhou Medical University
EPI_ISL_429101 hCoV-19/Guangzhou/GZMU0058/2020 Asia / China / Guangzhou   2020-01-27   The First Affiliated Hospital of Guangzhou Medical University   BGI-shenzhen & The First Affiliated Hospital of Guangzhou Medical University
EPI_ISL_429102 hCoV-19/Guangzhou/GZMU0059/2020 Asia / China / Guangzhou   2020-01-27   The First Affiliated Hospital of Guangzhou Medical University   BGI-shenzhen & The First Affiliated Hospital of Guangzhou Medical University
EPI_ISL_429103 hCoV-19/Guangzhou/GZMU0060/2020 Asia / China / Guangzhou   2020-02-09   The First Affiliated Hospital of Guangzhou Medical University   BGI-shenzhen & The First Affiliated Hospital of Guangzhou Medical University
EPI_ISL_429104 hCoV-19/Guangzhou/GZMU0036/2020 Asia / China / Guangzhou   2020-02-01   The First Affiliated Hospital of Guangzhou Medical University   BGI-shenzhen & The First Affiliated Hospital of Guangzhou Medical University
EPI_ISL_429105 hCoV-19/Guangzhou/GZMU0055/2020 Asia / China / Guangzhou   2020-01-29   The First Affiliated Hospital of Guangzhou Medical University   BGI-shenzhen & The First Affiliated Hospital of Guangzhou Medical University
EPI_ISL_429115 hCoV-19/Sweden/20-08142/2020   Europe / Sweden / Halland   2020-03-11   Klinisk mikrobiologi och vardhygien Halmstad   The Public Health Agency of Sweden   Arne Kotz, Olov Svartstrom, Maria Lind Karlberg, Anna-Malin Linde, Oskar Karlsson Lindsjo, Anna Risberg, Shaman Muradrasoli, Karin Tegmark-Wisell
EPI_ISL_429116 hCoV-19/Sweden/20-08143/2020   Europe / Sweden / Halland   2020-03-15   Klinisk mikrobiologi och vardhygien Halmstad   The Public Health Agency of Sweden   Arne Kotz, Olov Svartstrom,

Maria Lind Karlberg, Anna-Malin Linde, Oskar Karlsson Lindsjo, Anna Risberg, Shaman Muradrasoli, Karin Tegmark-Wisell
EPI_ISL_429117 hCoV-19/Sweden/20-08145/2020 Europe / Sweden / Halland 2020-03-11 Klinisk mikrobiologi och vardhygien Halmstad The Public Health Agency of Sweden Arne Kotz, Olov Svartstrom, Maria Lind Karlberg, Anna-Malin Linde, Oskar Karlsson Lindsjo, Anna Risberg, Shaman Muradrasoli, Karin Tegmark-Wisell
EPI_ISL_429118 hCoV-19/Sweden/20-08146/2020 Europe / Sweden / Halland 2020-03-11 Klinisk mikrobiologi och vardhygien Halmstad The Public Health Agency of Sweden Arne Kotz, Olov Svartstrom, Maria Lind Karlberg, Anna-Malin Linde, Oskar Karlsson Lindsjo, Anna Risberg, Shaman Muradrasoli, Karin Tegmark-Wisell
EPI_ISL_429119 hCoV-19/Sweden/20-08148/2020 Europe / Sweden / Halland 2020-03-11 Klinisk mikrobiologi och vardhygien Halmstad The Public Health Agency of Sweden Arne Kotz, Olov Svartstrom, Maria Lind Karlberg, Anna-Malin Linde, Oskar Karlsson Lindsjo, Anna Risberg, Shaman Muradrasoli, Karin Tegmark-Wisell
EPI_ISL_429120 hCoV-19/Sweden/20-08149/2020 Europe / Sweden / Halland 2020-03-11 Klinisk mikrobiologi och vardhygien Halmstad The Public Health Agency of Sweden Arne Kotz, Olov Svartstrom, Maria Lind Karlberg, Anna-Malin Linde, Oskar Karlsson Lindsjo, Anna Risberg, Shaman Muradrasoli, Karin Tegmark-Wisell
EPI_ISL_429121 hCoV-19/Sweden/20-08151/2020 Europe / Sweden / Halland 2020-03-11 Klinisk mikrobiologi och vardhygien Halmstad The Public Health Agency of Sweden Arne Kotz, Olov Svartstrom, Maria Lind Karlberg, Anna-Malin Linde, Oskar Karlsson Lindsjo, Anna Risberg, Shaman Muradrasoli, Karin Tegmark-Wisell
EPI_ISL_429122 hCoV-19/Sweden/20-08155/2020 Europe / Sweden / Halland 2020-03-11 Klinisk mikrobiologi och vardhygien Halmstad The Public Health Agency of Sweden Arne Kotz, Olov Svartstrom, Maria Lind Karlberg, Anna-Malin Linde, Oskar Karlsson Lindsjo, Anna Risberg, Shaman Muradrasoli, Karin Tegmark-Wisell
EPI_ISL_429123 hCoV-19/Sweden/20-08169/2020 Europe / Sweden / Halland 2020-03-12 Klinisk mikrobiologi och vardhygien Halmstad The Public Health Agency of Sweden Arne Kotz, Olov Svartstrom, Maria Lind Karlberg, Anna-Malin Linde, Oskar Karlsson Lindsjo, Anna Risberg, Shaman Muradrasoli, Karin Tegmark-Wisell
EPI_ISL_429124 hCoV-19/Sweden/20-08170/2020 Europe / Sweden / Halland 2020-03-12 Klinisk mikrobiologi och vardhygien Halmstad The Public Health Agency of Sweden Arne Kotz, Olov Svartstrom, Maria Lind Karlberg, Anna-Malin Linde, Oskar Karlsson Lindsjo, Anna Risberg, Shaman Muradrasoli, Karin Tegmark-Wisell
EPI_ISL_429125 hCoV-19/Sweden/20-08173/2020 Europe / Sweden / Halland 2020-03-12 Klinisk mikrobiologi och vardhygien Halmstad The Public Health Agency of Sweden Arne Kotz, Olov Svartstrom, Maria Lind Karlberg, Anna-Malin Linde, Oskar Karlsson Lindsjo, Anna Risberg, Shaman Muradrasoli, Karin Tegmark-Wisell
EPI_ISL_429126 hCoV-19/Sweden/20-08320/2020 Europe / Sweden / Sormland 2020-03-21 Unilabs Skovde The Public Health Agency of Sweden Tobias Kollberg, Helena Enroth, Olov Svartstrom, Maria Lind Karlberg, Anna-Malin Linde, Oskar Karlsson Lindsjo, Anna Risberg, Shaman Muradrasoli, Karin Tegmark-Wisell
EPI_ISL_429127 hCoV-19/Sweden/20-08332/2020 Europe / Sweden / Sormland 2020-03-20 Unilabs Skovde The Public Health Agency of Sweden Tobias Kollberg, Helena Enroth, Olov Svartstrom, Maria Lind Karlberg, Anna-Malin Linde, Oskar Karlsson Lindsjo, Anna Risberg, Shaman Muradrasoli, Karin Tegmark-Wisell
EPI_ISL_429129 hCoV-19/Sweden/20-08681/2020 Europe / Sweden / Norrbotten 2020-03-27 Laboratoriemedicin The Public Health Agency of Sweden Olov Svartstrom, Maria Lind Karlberg, Anna-Malin Linde, Oskar Karlsson Lindsjo, Anna Risberg, Shaman Muradrasoli, Karin Tegmark-Wisell
EPI_ISL_429130 hCoV-19/Sweden/20-08683/2020 Europe / Sweden / Norrbotten 2020-03-23 Laboratoriemedicin The Public Health Agency of Sweden Olov Svartstrom, Maria Lind Karlberg, Anna-Malin Linde, Oskar Karlsson Lindsjo, Anna Risberg, Shaman Muradrasoli, Karin Tegmark-Wisell
EPI_ISL_429131 hCoV-19/Sweden/20-08684/2020 Europe / Sweden / Norrbotten 2020-03-27 Laboratoriemedicin The Public Health Agency of Sweden Olov Svartstrom, Maria Lind Karlberg, Anna-Malin Linde, Oskar Karlsson Lindsjo, Anna Risberg, Shaman Muradrasoli, Karin Tegmark-Wisell
EPI_ISL_429132 hCoV-19/Sweden/20-08685/2020 Europe / Sweden / Norrbotten 2020-03-27 Laboratoriemedicin The Public Health Agency of Sweden Olov Svartstrom, Maria Lind Karlberg, Anna-Malin Linde, Oskar Karlsson Lindsjo, Anna Risberg, Shaman Muradrasoli, Karin Tegmark-Wisell
EPI_ISL_429133 hCoV-19/Sweden/20-08690/2020 Europe / Sweden / Norrbotten 2020-03-26 Laboratoriemedicin The Public Health Agency of Sweden Olov Svartstrom, Maria Lind Karlberg, Anna-Malin Linde, Oskar Karlsson Lindsjo, Anna Risberg, Shaman Muradrasoli, Karin Tegmark-Wisell
EPI_ISL_429134 hCoV-19/Sweden/20-08693/2020 Europe / Sweden / Norrbotten 2020-03-24 Laboratoriemedicin The Public Health Agency of Sweden Olov Svartstrom, Maria Lind Karlberg, Anna-Malin Linde, Oskar Karlsson Lindsjo, Anna Risberg, Shaman Muradrasoli, Karin Tegmark-Wisell
EPI_ISL_429135 hCoV-19/Sweden/20-08719/2020 Europe / Sweden / Norrbotten 2020-04-02 Laboratoriemedicin The Public Health Agency of Sweden Olov Svartstrom, Maria Lind Karlberg, Anna-Malin Linde, Oskar Karlsson Lindsjo, Anna Risberg, Shaman Muradrasoli, Karin Tegmark-Wisell
EPI_ISL_429136 hCoV-19/Sweden/20-50048/2020 Europe / Sweden / Varmland 2020-03-04 Klinisk mikrobiologi Orebro The Public Health Agency of Sweden Martin Sundqvist, Olov Svartstrom, Maria Lind Karlberg, Anna-Malin Linde, Oskar Karlsson Lindsjo, Anna Risberg, Shaman Muradrasoli, Karin Tegmark-Wisell
EPI_ISL_429137 hCoV-19/Sweden/20-50076/2020 Europe / Sweden / Orebro lan 2020-03-04 Klinisk mikrobiologi Orebro The Public Health Agency of Sweden Martin Sundqvist, Olov Svartstrom, Maria Lind

Karlberg, Anna-Malin Linde, Oskar Karlsson Lindsjo, Anna Risberg, Shaman Muradrasoli, Karin Tegmark-Wisell
EPI_ISL_429138 hCoV-19/Sweden/20-50087/2020 Europe / Sweden / Varmland 2020-03-05 Klinisk mikrobiologi Orebro The Public Health Agency of Sweden Martin Sundqvist, Olov Svartstrom, Maria Lind Karlberg, Anna-Malin Linde, Oskar Karlsson Lindsjo, Anna Risberg, Shaman Muradrasoli, Karin Tegmark-Wisell
EPI_ISL_429139 hCoV-19/Sweden/20-50123/2020 Europe / Sweden / Varmland 2020-03-06 Klinisk mikrobiologi Orebro The Public Health Agency of Sweden Martin Sundqvist, Olov Svartstrom, Maria Lind Karlberg, Anna-Malin Linde, Oskar Karlsson Lindsjo, Anna Risberg, Shaman Muradrasoli, Karin Tegmark-Wisell
EPI_ISL_429140 hCoV-19/Sweden/20-50125/2020 Europe / Sweden / Varmland 2020-03-06 Klinisk mikrobiologi Orebro The Public Health Agency of Sweden Martin Sundqvist, Olov Svartstrom, Maria Lind Karlberg, Anna-Malin Linde, Oskar Karlsson Lindsjo, Anna Risberg, Shaman Muradrasoli, Karin Tegmark-Wisell
EPI_ISL_429141 hCoV-19/Sweden/20-50130/2020 Europe / Sweden / Varmland 2020-03-06 Klinisk mikrobiologi Orebro The Public Health Agency of Sweden Martin Sundqvist, Olov Svartstrom, Maria Lind Karlberg, Anna-Malin Linde, Oskar Karlsson Lindsjo, Anna Risberg, Shaman Muradrasoli, Karin Tegmark-Wisell
EPI_ISL_429142 hCoV-19/Sweden/20-50131/2020 Europe / Sweden / Varmland 2020-03-06 Klinisk mikrobiologi Orebro The Public Health Agency of Sweden Martin Sundqvist, Olov Svartstrom, Maria Lind Karlberg, Anna-Malin Linde, Oskar Karlsson Lindsjo, Anna Risberg, Shaman Muradrasoli, Karin Tegmark-Wisell
EPI_ISL_429143 hCoV-19/Sweden/20-50132/2020 Europe / Sweden / Varmland 2020-03-06 Klinisk mikrobiologi Orebro The Public Health Agency of Sweden Martin Sundqvist, Olov Svartstrom, Maria Lind Karlberg, Anna-Malin Linde, Oskar Karlsson Lindsjo, Anna Risberg, Shaman Muradrasoli, Karin Tegmark-Wisell
EPI_ISL_429144 hCoV-19/Sweden/20-50133/2020 Europe / Sweden / Varmland 2020-03-06 Klinisk mikrobiologi Orebro The Public Health Agency of Sweden Martin Sundqvist, Olov Svartstrom, Maria Lind Karlberg, Anna-Malin Linde, Oskar Karlsson Lindsjo, Anna Risberg, Shaman Muradrasoli, Karin Tegmark-Wisell
EPI_ISL_429145 hCoV-19/Sweden/20-50170/2020 Europe / Sweden / Varmland 2020-03-08 Klinisk mikrobiologi Orebro The Public Health Agency of Sweden Martin Sundqvist, Olov Svartstrom, Maria Lind Karlberg, Anna-Malin Linde, Oskar Karlsson Lindsjo, Anna Risberg, Shaman Muradrasoli, Karin Tegmark-Wisell
EPI_ISL_429146 hCoV-19/Sweden/20-50171/2020 Europe / Sweden / Varmland 2020-03-08 Klinisk mikrobiologi Orebro The Public Health Agency of Sweden Martin Sundqvist, Olov Svartstrom, Maria Lind Karlberg, Anna-Malin Linde, Oskar Karlsson Lindsjo, Anna Risberg, Shaman Muradrasoli, Karin Tegmark-Wisell
EPI_ISL_429147 hCoV-19/Sweden/20-50172/2020 Europe / Sweden / Varmland 2020-03-08 Klinisk mikrobiologi Orebro The Public Health Agency of Sweden Martin Sundqvist, Olov Svartstrom, Maria Lind Karlberg, Anna-Malin Linde, Oskar Karlsson Lindsjo, Anna Risberg, Shaman Muradrasoli, Karin Tegmark-Wisell
EPI_ISL_429148 hCoV-19/Sweden/20-50173/2020 Europe / Sweden / Varmland 2020-03-08 Klinisk mikrobiologi Orebro The Public Health Agency of Sweden Martin Sundqvist, Olov Svartstrom, Maria Lind Karlberg, Anna-Malin Linde, Oskar Karlsson Lindsjo, Anna Risberg, Shaman Muradrasoli, Karin Tegmark-Wisell
EPI_ISL_429150 hCoV-19/Sweden/20-50175/2020 Europe / Sweden / Varmland 2020-03-08 Klinisk mikrobiologi Orebro The Public Health Agency of Sweden Martin Sundqvist, Olov Svartstrom, Maria Lind Karlberg, Anna-Malin Linde, Oskar Karlsson Lindsjo, Anna Risberg, Shaman Muradrasoli, Karin Tegmark-Wisell
EPI_ISL_429151 hCoV-19/Sweden/20-50176/2020 Europe / Sweden / Varmland 2020-03-08 Klinisk mikrobiologi Orebro The Public Health Agency of Sweden Martin Sundqvist, Olov Svartstrom, Maria Lind Karlberg, Anna-Malin Linde, Oskar Karlsson Lindsjo, Anna Risberg, Shaman Muradrasoli, Karin Tegmark-Wisell
EPI_ISL_429152 hCoV-19/Sweden/20-50179/2020 Europe / Sweden / Orebro lan 2020-03-08 Klinisk mikrobiologi Orebro The Public Health Agency of Sweden Martin Sundqvist, Olov Svartstrom, Maria Lind Karlberg, Anna-Malin Linde, Oskar Karlsson Lindsjo, Anna Risberg, Shaman Muradrasoli, Karin Tegmark-Wisell
EPI_ISL_429153 hCoV-19/Sweden/20-50180/2020 Europe / Sweden / Varmland 2020-03-08 Klinisk mikrobiologi Orebro The Public Health Agency of Sweden Martin Sundqvist, Olov Svartstrom, Maria Lind Karlberg, Anna-Malin Linde, Oskar Karlsson Lindsjo, Anna Risberg, Shaman Muradrasoli, Karin Tegmark-Wisell
EPI_ISL_429154 hCoV-19/Sweden/20-50181/2020 Europe / Sweden / Varmland 2020-03-08 Klinisk mikrobiologi Orebro The Public Health Agency of Sweden Martin Sundqvist, Olov Svartstrom, Maria Lind Karlberg, Anna-Malin Linde, Oskar Karlsson Lindsjo, Anna Risberg, Shaman Muradrasoli, Karin Tegmark-Wisell
EPI_ISL_429155 hCoV-19/Sweden/20-50230/2020 Europe / Sweden / Orebro lan 2020-03-09 Klinisk mikrobiologi Orebro The Public Health Agency of Sweden Martin Sundqvist, Olov Svartstrom, Maria Lind Karlberg, Anna-Malin Linde, Oskar Karlsson Lindsjo, Anna Risberg, Shaman Muradrasoli, Karin Tegmark-Wisell
EPI_ISL_429156 hCoV-19/Sweden/20-50232/2020 Europe / Sweden / Varmland 2020-03-09 Klinisk mikrobiologi Orebro The Public Health Agency of Sweden Martin Sundqvist, Olov Svartstrom, Maria Lind Karlberg, Anna-Malin Linde, Oskar Karlsson Lindsjo, Anna Risberg, Shaman Muradrasoli, Karin Tegmark-Wisell
EPI_ISL_429157 hCoV-19/Sweden/20-50234/2020 Europe / Sweden / Orebro lan 2020-03-10 Klinisk mikrobiologi Orebro The Public Health Agency of Sweden Martin Sundqvist, Olov Svartstrom, Maria Lind Karlberg, Anna-Malin Linde, Oskar Karlsson Lindsjo, Anna Risberg, Shaman Muradrasoli, Karin Tegmark-Wisell
EPI_ISL_429158 hCoV-19/Sweden/20-50235/2020 Europe / Sweden / Varmland 2020-03-10 Klinisk mikrobiologi Orebro The Public Health Agency of Sweden Martin Sundqvist, Olov Svartstrom, Maria Lind Karlberg, Anna-Malin Linde, Oskar Karlsson Lindsjo, Anna Risberg, Shaman Muradrasoli, Karin Tegmark-Wisell
EPI_ISL_429160 hCoV-19/Sweden/20-50312/2020 Europe / Sweden / Ostergotland 2020-03-12 Klinisk mikrobiologi Orebro The Public Health Agency of Sweden Martin Sundqvist, Olov Svartstrom, Maria Lind Karlberg, Anna-Malin Linde, Oskar Karlsson Lindsjo, Anna Risberg, Shaman Muradrasoli, Karin Tegmark-Wisell
EPI_ISL_429161 hCoV-19/Sweden/20-50315/2020 Europe / Sweden / Stockholm 2020-03-12 The Public Health Agency of Sweden The Public Health Agency of Sweden Olov Svartstrom, Maria Lind Karlberg, Anna-Malin Linde, Oskar Karlsson Lindsjo, Anna Risberg, Shaman Muradrasoli, Karin Tegmark-Wisell
EPI_ISL_429162 hCoV-19/Sweden/20-50357/2020 Europe / Sweden / Stockholm 2020-03-15 The Public Health Agency of Sweden The Public Health Agency of Sweden Olov Svartstrom, Maria Lind Karlberg, Anna-Malin Linde, Oskar Karlsson Lindsjo, Anna Risberg, Shaman Muradrasoli, Karin Tegmark-Wisell

EPI_ISL_429163 hCoV-19/Sweden/20-50361/2020 Europe / Sweden / Gavleborgs lan 2020-03-16 The Public Health Agency of Sweden The Public Health Agency of Sweden Olov Svartstrom, Maria Lind Karlberg, Anna-Malin Linde, Oskar Karlsson Lindsjo, Anna Risberg, Shaman Muradrasoli, Karin Tegmark-Wisell
EPI_ISL_429164 hCoV-19/Thailand/Bangkok-0025/2020 Asia / Thailand / Bangkok 2020-03-13 Ramathibodi Hospital COVID-19 Network Investigations (CONI) Alliance Elizabeth Batty, Wasun Chantratita, Thanat Chookajorn, Stefan Fernandez, Angkana Huang, Poramate Jiaranai, Anthony R. Jones, Khajohn Joonsalak, Chonticha Klungtong, Theerarat Kochakarn, Namfon Kotanan, Krittikorn Kumpornsin, Wudtichai Manasatienkij, Bhakbhoom Panthan, Ekawat Pasomsub, Kingkan Rakmanee, Insee Sensorn, Janjira Thaipadungpanit, Arporn Wangwiwatsin,Treewat Watthanachockchai
EPI_ISL_429165 hCoV-19/Thailand/Bangkok-0026/2020 Asia / Thailand / Bangkok 2020-03-13 Ramathibodi Hospital COVID-19 Network Investigations (CONI) Alliance Elizabeth Batty, Wasun Chantratita, Thanat Chookajorn, Stefan Fernandez, Angkana Huang, Poramate Jiaranai, Anthony R. Jones, Khajohn Joonsalak, Chonticha Klungtong, Theerarat Kochakarn, Namfon Kotanan, Krittikorn Kumpornsin, Wudtichai Manasatienkij, Bhakbhoom Panthan, Ekawat Pasomsub, Kingkan Rakmanee, Insee Sensorn, Janjira Thaipadungpanit, Arporn Wangwiwatsin,Treewat Watthanachockchai
EPI_ISL_429166 hCoV-19/Thailand/Bangkok-0028/2020 Asia / Thailand / Bangkok 2020-03-13 Ramathibodi Hospital COVID-19 Network Investigations (CONI) Alliance Elizabeth Batty, Wasun Chantratita, Thanat Chookajorn, Stefan Fernandez, Angkana Huang, Poramate Jiaranai, Anthony R. Jones, Khajohn Joonsalak, Chonticha Klungtong, Theerarat Kochakarn, Namfon Kotanan, Krittikorn Kumpornsin, Wudtichai Manasatienkij, Bhakbhoom Panthan, Ekawat Pasomsub, Kingkan Rakmanee, Insee Sensorn, Janjira Thaipadungpanit, Arporn Wangwiwatsin,Treewat Watthanachockchai
EPI_ISL_429167 hCoV-19/Thailand/Bangkok-0029/2020 Asia / Thailand / Bangkok 2020-03-13 Ramathibodi Hospital COVID-19 Network Investigations (CONI) Alliance Elizabeth Batty, Wasun Chantratita, Thanat Chookajorn, Stefan Fernandez, Angkana Huang, Poramate Jiaranai, Anthony R. Jones, Khajohn Joonsalak, Chonticha Klungtong, Theerarat Kochakarn, Namfon Kotanan, Krittikorn Kumpornsin, Wudtichai Manasatienkij, Bhakbhoom Panthan, Ekawat Pasomsub, Kingkan Rakmanee, Insee Sensorn, Janjira Thaipadungpanit, Arporn Wangwiwatsin,Treewat Watthanachockchai
EPI_ISL_429169 hCoV-19/Thailand/Bangkok-0033/2020 Asia / Thailand / Bangkok 2020-03-17 Ramathibodi Hospital COVID-19 Network Investigations (CONI) Alliance Elizabeth Batty, Wasun Chantratita, Thanat Chookajorn, Stefan Fernandez, Angkana Huang, Poramate Jiaranai, Anthony R. Jones, Khajohn Joonsalak, Chonticha Klungtong, Theerarat Kochakarn, Namfon Kotanan, Krittikorn Kumpornsin, Wudtichai Manasatienkij, Bhakbhoom Panthan, Ekawat Pasomsub, Kingkan Rakmanee, Insee Sensorn, Janjira Thaipadungpanit, Arporn Wangwiwatsin,Treewat Watthanachockchai
EPI_ISL_429170 hCoV-19/Thailand/Bangkok-0034/2020 Asia / Thailand / Bangkok 2020-03-17 Ramathibodi Hospital COVID-19 Network Investigations (CONI) Alliance Elizabeth Batty, Wasun Chantratita, Thanat Chookajorn, Stefan Fernandez, Angkana Huang, Poramate Jiaranai, Anthony R. Jones, Khajohn Joonsalak, Chonticha Klungtong, Theerarat Kochakarn, Namfon Kotanan, Krittikorn Kumpornsin, Wudtichai Manasatienkij, Bhakbhoom Panthan, Ekawat Pasomsub, Kingkan Rakmanee, Insee Sensorn, Janjira Thaipadungpanit, Arporn Wangwiwatsin,Treewat Watthanachockchai
EPI_ISL_429171 hCoV-19/Thailand/Bangkok-0035/2020 Asia / Thailand / Bangkok 2020-03-18 Ramathibodi Hospital COVID-19 Network Investigations (CONI) Alliance Elizabeth Batty, Wasun Chantratita, Thanat Chookajorn, Stefan Fernandez, Angkana Huang, Poramate Jiaranai, Anthony R. Jones, Khajohn Joonsalak, Chonticha Klungtong, Theerarat Kochakarn, Namfon Kotanan, Krittikorn Kumpornsin, Wudtichai Manasatienkij, Bhakbhoom Panthan, Ekawat Pasomsub, Kingkan Rakmanee, Insee Sensorn, Janjira Thaipadungpanit, Arporn Wangwiwatsin,Treewat Watthanachockchai
EPI_ISL_429172 hCoV-19/Thailand/Bangkok-0036/2020 Asia / Thailand / Bangkok 2020-03-21 Ramathibodi Hospital COVID-19 Network Investigations (CONI) Alliance Elizabeth Batty, Wasun Chantratita, Thanat Chookajorn, Stefan Fernandez, Angkana Huang, Poramate Jiaranai, Anthony R. Jones, Khajohn Joonsalak, Chonticha Klungtong, Theerarat Kochakarn, Namfon Kotanan, Krittikorn Kumpornsin, Wudtichai Manasatienkij, Bhakbhoom Panthan, Ekawat Pasomsub, Kingkan Rakmanee, Insee Sensorn, Janjira Thaipadungpanit, Arporn Wangwiwatsin,Treewat Watthanachockchai
EPI_ISL_429173 hCoV-19/Thailand/Bangkok-0037/2020 Asia / Thailand / Bangkok 2020-03-21 Ramathibodi Hospital COVID-19 Network Investigations (CONI) Alliance Elizabeth Batty, Wasun Chantratita, Thanat Chookajorn, Stefan Fernandez, Angkana Huang, Poramate Jiaranai, Anthony R. Jones, Khajohn Joonsalak, Chonticha Klungtong, Theerarat Kochakarn, Namfon Kotanan, Krittikorn Kumpornsin, Wudtichai Manasatienkij, Bhakbhoom Panthan, Ekawat Pasomsub, Kingkan Rakmanee, Insee Sensorn, Janjira Thaipadungpanit, Arporn Wangwiwatsin,Treewat Watthanachockchai
EPI_ISL_429174 hCoV-19/Thailand/Bangkok-0038/2020 Asia / Thailand / Bangkok 2020-03-25 Ramathibodi Hospital COVID-19 Network Investigations (CONI) Alliance Elizabeth Batty, Wasun Chantratita, Thanat Chookajorn, Stefan Fernandez, Angkana Huang, Poramate Jiaranai, Anthony R. Jones, Khajohn Joonsalak, Chonticha Klungtong, Theerarat Kochakarn, Namfon Kotanan, Krittikorn Kumpornsin, Wudtichai Manasatienkij, Bhakbhoom Panthan, Ekawat Pasomsub, Kingkan Rakmanee, Insee Sensorn, Janjira Thaipadungpanit, Arporn Wangwiwatsin,Treewat Watthanachockchai
EPI_ISL_429175 hCoV-19/Thailand/Bangkok-0039/2020 Asia / Thailand / Bangkok 2020-03-28 Ramathibodi Hospital COVID-19 Network Investigations (CONI) Alliance Elizabeth Batty, Wasun Chantratita, Thanat Chookajorn, Stefan Fernandez, Angkana Huang, Poramate Jiaranai, Anthony R. Jones, Khajohn Joonsalak, Chonticha Klungtong, Theerarat Kochakarn, Namfon Kotanan, Krittikorn Kumpornsin, Wudtichai Manasatienkij, Bhakbhoom Panthan, Ekawat Pasomsub, Kingkan Rakmanee, Insee Sensorn, Janjira Thaipadungpanit, Arporn Wangwiwatsin,Treewat Watthanachockchai
EPI_ISL_429178 hCoV-19/Thailand/Bangkok-0042/2020 Asia / Thailand / Bangkok 2020-03-28

Ramathibodi Hospital COVID-19 Network Investigations (CONI) Alliance Elizabeth Batty, Wasun Chantratita, Thanat Chookajorn, Stefan Fernandez, Angkana Huang, Poramate Jiaranai, Anthony R. Jones, Khajohn Joonsalak, Chonticha Klungtong, Theerarat Kochakarn, Namfon Kotanan, Krittikorn Kumpornsin, Wudtichai Manasatienkij, Bhakbhoom Panthan, Ekawat Pasomsub, Kingkan Rakmanee, Insee Sensorn, Janjira Thaipadungpanit, Arporn Wangwiwatsin,Treewat Watthanachockchai
EPI_ISL_429179 hCoV-19/Thailand/Bangkok-0043/2020 Asia / Thailand / Bangkok 2020-03-28 Ramathibodi Hospital COVID-19 Network Investigations (CONI) Alliance Elizabeth Batty, Wasun Chantratita, Thanat Chookajorn, Stefan Fernandez, Angkana Huang, Poramate Jiaranai, Anthony R. Jones, Khajohn Joonsalak, Chonticha Klungtong, Theerarat Kochakarn, Namfon Kotanan, Krittikorn Kumpornsin, Wudtichai Manasatienkij, Bhakbhoom Panthan, Ekawat Pasomsub, Kingkan Rakmanee, Insee Sensorn, Janjira Thaipadungpanit, Arporn Wangwiwatsin,Treewat Watthanachockchai
EPI_ISL_429181 hCoV-19/Thailand/Bangkok-0045/2020 Asia / Thailand / Bangkok 2020-03-28 Ramathibodi Hospital COVID-19 Network Investigations (CONI) Alliance Elizabeth Batty, Wasun Chantratita, Thanat Chookajorn, Stefan Fernandez, Angkana Huang, Poramate Jiaranai, Anthony R. Jones, Khajohn Joonsalak, Chonticha Klungtong, Theerarat Kochakarn, Namfon Kotanan, Krittikorn Kumpornsin, Wudtichai Manasatienkij, Bhakbhoom Panthan, Ekawat Pasomsub, Kingkan Rakmanee, Insee Sensorn, Janjira Thaipadungpanit, Arporn Wangwiwatsin,Treewat Watthanachockchai
EPI_ISL_429182 hCoV-19/Thailand/Bangkok-0046/2020 Asia / Thailand / Bangkok 2020-03-28 Ramathibodi Hospital COVID-19 Network Investigations (CONI) Alliance Elizabeth Batty, Wasun Chantratita, Thanat Chookajorn, Stefan Fernandez, Angkana Huang, Poramate Jiaranai, Anthony R. Jones, Khajohn Joonsalak, Chonticha Klungtong, Theerarat Kochakarn, Namfon Kotanan, Krittikorn Kumpornsin, Wudtichai Manasatienkij, Bhakbhoom Panthan, Ekawat Pasomsub, Kingkan Rakmanee, Insee Sensorn, Janjira Thaipadungpanit, Arporn Wangwiwatsin,Treewat Watthanachockchai
EPI_ISL_429183 hCoV-19/Thailand/Bangkok-0047/2020 Asia / Thailand / Bangkok 2020-03-28 Ramathibodi Hospital COVID-19 Network Investigations (CONI) Alliance Elizabeth Batty, Wasun Chantratita, Thanat Chookajorn, Stefan Fernandez, Angkana Huang, Poramate Jiaranai, Anthony R. Jones, Khajohn Joonsalak, Chonticha Klungtong, Theerarat Kochakarn, Namfon Kotanan, Krittikorn Kumpornsin, Wudtichai Manasatienkij, Bhakbhoom Panthan, Ekawat Pasomsub, Kingkan Rakmanee, Insee Sensorn, Janjira Thaipadungpanit, Arporn Wangwiwatsin,Treewat Watthanachockchai
EPI_ISL_429184 hCoV-19/Thailand/Bangkok-0048/2020 Asia / Thailand / Bangkok 2020-03-28 Ramathibodi Hospital COVID-19 Network Investigations (CONI) Alliance Elizabeth Batty, Wasun Chantratita, Thanat Chookajorn, Stefan Fernandez, Angkana Huang, Poramate Jiaranai, Anthony R. Jones, Khajohn Joonsalak, Chonticha Klungtong, Theerarat Kochakarn, Namfon Kotanan, Krittikorn Kumpornsin, Wudtichai Manasatienkij, Bhakbhoom Panthan, Ekawat Pasomsub, Kingkan Rakmanee, Insee Sensorn, Janjira Thaipadungpanit, Arporn Wangwiwatsin,Treewat Watthanachockchai
EPI_ISL_429231 hCoV-19/Italy/TE6571/2020 Europe / Italy / Abruzzo 2020-03-24 Ospedale Civile Giuseppe Mazzini "Istituto Zooprofilattico Sperimentale dell'Abruzzo e Molise ""G. Caporale""" Lorusso A, Marcacci M, Di Domenico M, Ancora M, Curini V, Mangone I, Rinaldi A, Di Pasquale A, Camma C, Puglia I, Savini G
EPI_ISL_429232 hCoV-19/Italy/TE6572/2020 Europe / Italy / Abruzzo 2020-03-24 Ospedale Civile Giuseppe Mazzini "Istituto Zooprofilattico Sperimentale dell'Abruzzo e Molise ""G. Caporale""" Lorusso A, Marcacci M, Di Domenico M, Ancora M, Curini V, Mangone I, Rinaldi A, Di Pasquale A, Camma C, Puglia I, Savini G
EPI_ISL_429235 hCoV-19/Italy/TE6580/2020 Europe / Italy / Abruzzo 2020-03-24 Ospedale Civile Giuseppe Mazzini "Istituto Zooprofilattico Sperimentale dell'Abruzzo e Molise ""G. Caporale""" Lorusso A, Marcacci M, Di Domenico M, Ancora M, Curini V, Mangone I, Rinaldi A, Di Pasquale A, Camma C, Puglia I, Savini G
EPI_ISL_429236 hCoV-19/Italy/TE7859/2020 Europe / Italy / Abruzzo 2020-03-28 Ospedale Civile S. Liberatore di Atri "Istituto Zooprofilattico Sperimentale dell'Abruzzo e Molise ""G. Caporale""" Lorusso A, Marcacci M, Di Domenico M, Ancora M, Curini V, Mangone I, Rinaldi A, Di Pasquale A, Camma C, Puglia I, Savini G
EPI_ISL_429239 hCoV-19/Yunnan/0306-466/2020 Asia / China / Yunnan 2020-03-06 Department of Clinical Laboratory, the First People's Hospital of Yunnan Province Department of Clinical Laboratory, the First People's Hospital of Yunnan Province Yi Sun,Ziqin Dian,Ya Xu,Guiqian Zhang,Xin Fan,Yu Zhang
EPI_ISL_429255 hCoV-19/DRC/1131/2020 Africa / Democratic Republic of the Congo / Kinshasa 2020-04-05 Viral Respiratory Lab, National Institute for Biomedical Research (INRB) Pathogen Sequencing Lab, National Institute for Biomedical Research (INRB) Placide Mbala-Kingebeni, Edith Nkwembe, Eddy Kinganda-Lusamaki, Amuri Aziza, Catherine Pratt, Matthias Pauthner, Josh Quick, Allison Black, James Hadfield, Trevor Bedford, Ian Goodfellow, Nick Loman, Kristian Andersen, Michael Wiley, Steve Ahuka-Mundeke, Jean-Jacques Muyembe Tamfum
EPI_ISL_429256 hCoV-19/Spain/Vircell001/2020 Europe / Spain / Madrid 2020-03-12 Health Sciences Technology Park, Avicena, 8, 18016 Granada. Spain Sequencing and Bioinformatics Service FISABIO-Public Health Joaquín Mendoza, Almudena Rojas, Pablo Mendoza
EPI_ISL_429262 hCoV-19/Denmark/ALAB-HH01/2020 Europe / Denmark / Unknown 2020-03-10 Department of Clinical Microbiology, Copenhagen University Hospital, Hvidovre, Kettegaard Alle 30, 2650 Hvidovre. Albertsen lab, Department of Chemistry and Bioscience, Aalborg University, Denmark Rasmus Kirkegaard
EPI_ISL_429263 hCoV-19/Denmark/ALAB-HH02/2020 Europe / Denmark / Unknown 2020-03-10 Department of Clinical Microbiology, Copenhagen University Hospital, Hvidovre, Kettegaard Alle 30, 2650 Hvidovre. Albertsen lab, Department of Chemistry and Bioscience, Aalborg University, Denmark Rasmus Kirkegaard
EPI_ISL_429264 hCoV-19/Denmark/ALAB-HH03/2020 Europe / Denmark / Unknown 2020-03-10 Department of

Clinical Microbiology, Copenhagen University Hospital, Hvidovre, Kettegaard Alle 30, 2650 Hvidovre.
Albertsen lab, Department of Chemistry and Bioscience, Aalborg University, Denmark Rasmus Kirkegaard
EPI_ISL_429265 hCoV-19/Denmark/ALAB-HH04/2020 Europe / Denmark / Unknown 2020-03-10 Department of Clinical Microbiology, Copenhagen University Hospital, Hvidovre, Kettegaard Alle 30, 2650 Hvidovre.
Albertsen lab, Department of Chemistry and Bioscience, Aalborg University, Denmark Rasmus Kirkegaard
EPI_ISL_429266 hCoV-19/Denmark/ALAB-HH05/2020 Europe / Denmark / Unknown 2020-03-10 Department of Clinical Microbiology, Copenhagen University Hospital, Hvidovre, Kettegaard Alle 30, 2650 Hvidovre.
Albertsen lab, Department of Chemistry and Bioscience, Aalborg University, Denmark Rasmus Kirkegaard
EPI_ISL_429267 hCoV-19/Denmark/ALAB-HH06/2020 Europe / Denmark / Unknown 2020-03-10 Department of Clinical Microbiology, Copenhagen University Hospital, Hvidovre, Kettegaard Alle 30, 2650 Hvidovre.
Albertsen lab, Department of Chemistry and Bioscience, Aalborg University, Denmark Rasmus Kirkegaard
EPI_ISL_429268 hCoV-19/Denmark/ALAB-HH07/2020 Europe / Denmark / Unknown 2020-03-10 Department of Clinical Microbiology, Copenhagen University Hospital, Hvidovre, Kettegaard Alle 30, 2650 Hvidovre.
Albertsen lab, Department of Chemistry and Bioscience, Aalborg University, Denmark Rasmus Kirkegaard
EPI_ISL_429269 hCoV-19/Denmark/ALAB-HH08/2020 Europe / Denmark / Unknown 2020-03-10 Department of Clinical Microbiology, Copenhagen University Hospital, Hvidovre, Kettegaard Alle 30, 2650 Hvidovre.
Albertsen lab, Department of Chemistry and Bioscience, Aalborg University, Denmark Rasmus Kirkegaard
EPI_ISL_429270 hCoV-19/Denmark/ALAB-HH11/2020 Europe / Denmark / Unknown 2020-03-10 Department of Clinical Microbiology, Copenhagen University Hospital, Hvidovre, Kettegaard Alle 30, 2650 Hvidovre.
Albertsen lab, Department of Chemistry and Bioscience, Aalborg University, Denmark Rasmus Kirkegaard
EPI_ISL_429271 hCoV-19/Denmark/ALAB-HH13/2020 Europe / Denmark / Unknown 2020-03-10 Department of Clinical Microbiology, Copenhagen University Hospital, Hvidovre, Kettegaard Alle 30, 2650 Hvidovre.
Albertsen lab, Department of Chemistry and Bioscience, Aalborg University, Denmark Rasmus Kirkegaard
EPI_ISL_429272 hCoV-19/Denmark/ALAB-HH14/2020 Europe / Denmark / Unknown 2020-03-10 Department of Clinical Microbiology, Copenhagen University Hospital, Hvidovre, Kettegaard Alle 30, 2650 Hvidovre.
Albertsen lab, Department of Chemistry and Bioscience, Aalborg University, Denmark Rasmus Kirkegaard
EPI_ISL_429273 hCoV-19/Denmark/ALAB-HH15/2020 Europe / Denmark / Unknown 2020-03-10 Department of Clinical Microbiology, Copenhagen University Hospital, Hvidovre, Kettegaard Alle 30, 2650 Hvidovre.
Albertsen lab, Department of Chemistry and Bioscience, Aalborg University, Denmark Rasmus Kirkegaard
EPI_ISL_429274 hCoV-19/Denmark/ALAB-HH16/2020 Europe / Denmark / Unknown 2020-03-10 Department of Clinical Microbiology, Copenhagen University Hospital, Hvidovre, Kettegaard Alle 30, 2650 Hvidovre.
Albertsen lab, Department of Chemistry and Bioscience, Aalborg University, Denmark Rasmus Kirkegaard
EPI_ISL_429275 hCoV-19/Denmark/ALAB-HH17/2020 Europe / Denmark / Unknown 2020-03-10 Department of Clinical Microbiology, Copenhagen University Hospital, Hvidovre, Kettegaard Alle 30, 2650 Hvidovre.
Albertsen lab, Department of Chemistry and Bioscience, Aalborg University, Denmark Rasmus Kirkegaard
EPI_ISL_429276 hCoV-19/Denmark/ALAB-HH18/2020 Europe / Denmark / Unknown 2020-03-10 Department of Clinical Microbiology, Copenhagen University Hospital, Hvidovre, Kettegaard Alle 30, 2650 Hvidovre.
Albertsen lab, Department of Chemistry and Bioscience, Aalborg University, Denmark Rasmus Kirkegaard
EPI_ISL_429277 hCoV-19/Denmark/ALAB-HH19/2020 Europe / Denmark / Unknown 2020-03-10 Department of Clinical Microbiology, Copenhagen University Hospital, Hvidovre, Kettegaard Alle 30, 2650 Hvidovre.
Albertsen lab, Department of Chemistry and Bioscience, Aalborg University, Denmark Rasmus Kirkegaard
EPI_ISL_429278 hCoV-19/Denmark/ALAB-HH20/2020 Europe / Denmark / Unknown 2020-03-10 Department of Clinical Microbiology, Copenhagen University Hospital, Hvidovre, Kettegaard Alle 30, 2650 Hvidovre.
Albertsen lab, Department of Chemistry and Bioscience, Aalborg University, Denmark Rasmus Kirkegaard
EPI_ISL_429279 hCoV-19/Denmark/ALAB-HH21/2020 Europe / Denmark / Unknown 2020-03-10 Department of Clinical Microbiology, Copenhagen University Hospital, Hvidovre, Kettegaard Alle 30, 2650 Hvidovre.
Albertsen lab, Department of Chemistry and Bioscience, Aalborg University, Denmark Rasmus Kirkegaard
EPI_ISL_429280 hCoV-19/Denmark/ALAB-HH22/2020 Europe / Denmark / Unknown 2020-03-10 Department of Clinical Microbiology, Copenhagen University Hospital, Hvidovre, Kettegaard Alle 30, 2650 Hvidovre.
Albertsen lab, Department of Chemistry and Bioscience, Aalborg University, Denmark Rasmus Kirkegaard
EPI_ISL_429281 hCoV-19/Denmark/ALAB-HH23/2020 Europe / Denmark / Unknown 2020-03-10 Department of Clinical Microbiology, Copenhagen University Hospital, Hvidovre, Kettegaard Alle 30, 2650 Hvidovre.
Albertsen lab, Department of Chemistry and Bioscience, Aalborg University, Denmark Rasmus Kirkegaard
EPI_ISL_429282 hCoV-19/Denmark/ALAB-HH24/2020 Europe / Denmark / Unknown 2020-03-11 Department of Clinical Microbiology, Copenhagen University Hospital, Hvidovre, Kettegaard Alle 30, 2650 Hvidovre.
Albertsen lab, Department of Chemistry and Bioscience, Aalborg University, Denmark Rasmus Kirkegaard
EPI_ISL_429283 hCoV-19/Denmark/ALAB-HH25/2020 Europe / Denmark / Unknown 2020-03-11 Department of Clinical Microbiology, Copenhagen University Hospital, Hvidovre, Kettegaard Alle 30, 2650 Hvidovre.
Albertsen lab, Department of Chemistry and Bioscience, Aalborg University, Denmark Rasmus Kirkegaard
EPI_ISL_429284 hCoV-19/Denmark/ALAB-HH26/2020 Europe / Denmark / Unknown 2020-03-11 Department of Clinical Microbiology, Copenhagen University Hospital, Hvidovre, Kettegaard Alle 30, 2650 Hvidovre.
Albertsen lab, Department of Chemistry and Bioscience, Aalborg University, Denmark Rasmus Kirkegaard
EPI_ISL_429285 hCoV-19/Denmark/ALAB-HH27/2020 Europe / Denmark / Unknown 2020-03-11 Department of Clinical Microbiology, Copenhagen University Hospital, Hvidovre, Kettegaard Alle 30, 2650 Hvidovre.
Albertsen lab, Department of Chemistry and Bioscience, Aalborg University, Denmark Rasmus Kirkegaard
EPI_ISL_429287 hCoV-19/Denmark/ALAB-HH29/2020 Europe / Denmark / Unknown 2020-03-11 Department of Clinical Microbiology, Copenhagen University Hospital, Hvidovre, Kettegaard Alle 30, 2650 Hvidovre.
Albertsen lab, Department of Chemistry and Bioscience, Aalborg University, Denmark Rasmus Kirkegaard
EPI_ISL_429289 hCoV-19/Denmark/ALAB-HH31/2020 Europe / Denmark / Unknown 2020-03-12 Department of Clinical Microbiology, Copenhagen University Hospital, Hvidovre, Kettegaard Alle 30, 2650 Hvidovre.

Albertsen lab, Department of Chemistry and Bioscience, Aalborg University, Denmark	Rasmus Kirkegaard
EPI_ISL_429293	hCoV-19/Denmark/ALAB-HH37/2020	Europe / Denmark / Unknown	2020-03-13	Department of Clinical Microbiology, Copenhagen University Hospital, Hvidovre, Kettegaard Alle 30, 2650 Hvidovre.	Albertsen lab, Department of Chemistry and Bioscience, Aalborg University, Denmark	Rasmus Kirkegaard
EPI_ISL_429295	hCoV-19/Denmark/ALAB-HH39/2020	Europe / Denmark / Unknown	2020-03-13	Department of Clinical Microbiology, Copenhagen University Hospital, Hvidovre, Kettegaard Alle 30, 2650 Hvidovre.	Albertsen lab, Department of Chemistry and Bioscience, Aalborg University, Denmark	Rasmus Kirkegaard
EPI_ISL_429299	hCoV-19/Denmark/ALAB-HH43/2020	Europe / Denmark / Unknown	2020-03-14	Department of Clinical Microbiology, Copenhagen University Hospital, Hvidovre, Kettegaard Alle 30, 2650 Hvidovre.	Albertsen lab, Department of Chemistry and Bioscience, Aalborg University, Denmark	Rasmus Kirkegaard
EPI_ISL_430059	hCoV-19/USA/UT-0513/2020	North America / USA / Utah	2020-04-03	Utah Public Health Laboratory	Utah Public Health Laboratory	Erin Young, Kelly Oakeson
EPI_ISL_430061	hCoV-19/USA/UT-0519/2020	North America / USA / Utah	2020-04-03	Utah Public Health Laboratory	Utah Public Health Laboratory	Erin Young, Kelly Oakeson
EPI_ISL_430064	hCoV-19/Australia/VIC-CBA4/2020	Oceania / Australia / Victoria	2020-03-07	Geelong Centre for Emerging Infectious Diseases	Geelong Centre for Emerging Infectious Diseases	Chamings A., Bhatta T.R., Alexandersen S.
EPI_ISL_430065	hCoV-19/Australia/VIC-CBA5/2020	Oceania / Australia / Victoria	2020-04-08	Geelong Centre for Emerging Infectious Diseases	Geelong Centre for Emerging Infectious Diseases	Chamings A., Bhatta T.R., Alexandersen S.
EPI_ISL_430067	hCoV-19/Russia/StPetersburg-RII4705S/2020	Europe / Russia / St.Petersburg	2020-04-07	WHO National Influenza Centre Russian Federation	WHO National Influenza Centre Russian Federation	Andrey Komissarov, Artem Fadeev, Mariia Sergeeva, Anna Ivanova, Daria Danilenko
EPI_ISL_430068	hCoV-19/Russia/StPetersburg-RII4712V/2020	Europe / Russia / St.Petersburg	2020-04-07	WHO National Influenza Centre Russian Federation	WHO National Influenza Centre Russian Federation	Andrey Komissarov, Artem Fadeev, Mariia Sergeeva, Anna Ivanova, Daria Danilenko
EPI_ISL_430069	hCoV-19/Russia/StPetersburg-RII4780S/2020	Europe / Russia / St.Petersburg	2020-04-07	WHO National Influenza Centre Russian Federation	WHO National Influenza Centre Russian Federation	Andrey Komissarov, Artem Fadeev, Mariia Sergeeva, Anna Ivanova, Daria Danilenko
EPI_ISL_430070	hCoV-19/Russia/StPetersburg-RII4917S/2020	Europe / Russia / St.Petersburg	2020-04-07	WHO National Influenza Centre Russian Federation	WHO National Influenza Centre Russian Federation	Andrey Komissarov, Artem Fadeev, Mariia Sergeeva, Anna Ivanova, Daria Danilenko
EPI_ISL_430071	hCoV-19/Russia/StPetersburg-RII4936S/2020	Europe / Russia / St.Petersburg	2020-04-08	WHO National Influenza Centre Russian Federation	WHO National Influenza Centre Russian Federation	Andrey Komissarov, Artem Fadeev, Mariia Sergeeva, Anna Ivanova, Daria Danilenko
EPI_ISL_430072	hCoV-19/Russia/StPetersburg-RII4937S/2020	Europe / Russia / St.Petersburg	2020-04-08	WHO National Influenza Centre Russian Federation	WHO National Influenza Centre Russian Federation	Andrey Komissarov, Artem Fadeev, Mariia Sergeeva, Anna Ivanova, Daria Danilenko
EPI_ISL_430073	hCoV-19/Russia/StPetersburg-RII4938S/2020	Europe / Russia / St.Petersburg	2020-04-08	WHO National Influenza Centre Russian Federation	WHO National Influenza Centre Russian Federation	Andrey Komissarov, Artem Fadeev, Mariia Sergeeva, Anna Ivanova, Daria Danilenko
EPI_ISL_430074	hCoV-19/Russia/StPetersburg-RII4944S/2020	Europe / Russia / St.Petersburg	2020-04-08	WHO National Influenza Centre Russian Federation	WHO National Influenza Centre Russian Federation	Andrey Komissarov, Artem Fadeev, Mariia Sergeeva, Anna Ivanova, Daria Danilenko
EPI_ISL_430075	hCoV-19/Russia/StPetersburg-RII4949S/2020	Europe / Russia / St.Petersburg	2020-04-09	WHO National Influenza Centre Russian Federation	WHO National Influenza Centre Russian Federation	Andrey Komissarov, Artem Fadeev, Mariia Sergeeva, Anna Ivanova, Daria Danilenko
EPI_ISL_430076	hCoV-19/Russia/StPetersburg-RII4969S/2020	Europe / Russia / St.Petersburg	2020-04-10	WHO National Influenza Centre Russian Federation	WHO National Influenza Centre Russian Federation	Andrey Komissarov, Artem Fadeev, Mariia Sergeeva, Anna Ivanova, Daria Danilenko
EPI_ISL_430077	hCoV-19/Russia/StPetersburg-RII4970S/2020	Europe / Russia / St.Petersburg	2020-04-10	WHO National Influenza Centre Russian Federation	WHO National Influenza Centre Russian Federation	Andrey Komissarov, Artem Fadeev, Mariia Sergeeva, Anna Ivanova, Daria Danilenko
EPI_ISL_430078	hCoV-19/Russia/StPetersburg-RII4972S/2020	Europe / Russia / St.Petersburg	2020-04-10	WHO National Influenza Centre Russian Federation	WHO National Influenza Centre Russian Federation	Andrey Komissarov, Artem Fadeev, Mariia Sergeeva, Anna Ivanova, Daria Danilenko
EPI_ISL_430079	hCoV-19/Russia/StPetersburg-RII4980S/2020	Europe / Russia / St.Petersburg	2020-04-10	WHO National Influenza Centre Russian Federation	WHO National Influenza Centre Russian Federation	Andrey Komissarov, Artem Fadeev, Mariia Sergeeva, Anna Ivanova, Daria Danilenko
EPI_ISL_430080	hCoV-19/Russia/StPetersburg-RII4983S/2020	Europe / Russia / St.Petersburg	2020-04-10	WHO National Influenza Centre Russian Federation	WHO National Influenza Centre Russian Federation	Andrey Komissarov, Artem Fadeev, Mariia Sergeeva, Anna Ivanova, Daria Danilenko
EPI_ISL_430081	hCoV-19/Russia/StPetersburg-RII4984S/2020	Europe / Russia / St.Petersburg	2020-04-10	WHO National Influenza Centre Russian Federation	WHO National Influenza Centre Russian Federation	Andrey Komissarov, Artem Fadeev, Mariia Sergeeva, Anna Ivanova, Daria Danilenko
EPI_ISL_430082	hCoV-19/Russia/StPetersburg-RII4985S/2020	Europe / Russia / St.Petersburg	2020-04-10	WHO National Influenza Centre Russian Federation	WHO National Influenza Centre Russian Federation	Andrey Komissarov, Artem Fadeev, Mariia Sergeeva, Anna Ivanova, Daria Danilenko
EPI_ISL_430083	hCoV-19/Russia/StPetersburg-RII4988S/2020	Europe / Russia / St.Petersburg	2020-04-10	WHO National Influenza Centre Russian Federation	WHO National Influenza Centre Russian Federation

Andrey Komissarov, Artem Fadeev, Mariia Sergeeva, Anna Ivanova, Daria Danilenko
EPI_ISL_430084 hCoV-19/Russia/StPetersburg-RII5017V/2020 Europe / Russia / St.Petersburg 2020-04-09
WHO National Influenza Centre Russian Federation WHO National Influenza Centre Russian Federation
Andrey Komissarov, Artem Fadeev, Mariia Sergeeva, Anna Ivanova, Daria Danilenko
EPI_ISL_430085 hCoV-19/Russia/StPetersburg-RII5029S/2020 Europe / Russia / St.Petersburg 2020-04-10
WHO National Influenza Centre Russian Federation WHO National Influenza Centre Russian Federation
Andrey Komissarov, Artem Fadeev, Mariia Sergeeva, Anna Ivanova, Daria Danilenko
EPI_ISL_430086 hCoV-19/Russia/StPetersburg-RII5033S/2020 Europe / Russia / St.Petersburg 2020-04-10
WHO National Influenza Centre Russian Federation WHO National Influenza Centre Russian Federation
Andrey Komissarov, Artem Fadeev, Mariia Sergeeva, Anna Ivanova, Daria Danilenko
EPI_ISL_430087 hCoV-19/Russia/StPetersburg-RII5047S/2020 Europe / Russia / St.Petersburg 2020-04-10
WHO National Influenza Centre Russian Federation WHO National Influenza Centre Russian Federation
Andrey Komissarov, Artem Fadeev, Mariia Sergeeva, Anna Ivanova, Daria Danilenko
EPI_ISL_430088 hCoV-19/Russia/StPetersburg-RII5169S/2020 Europe / Russia / St.Petersburg 2020-04-09
WHO National Influenza Centre Russian Federation WHO National Influenza Centre Russian Federation
Andrey Komissarov, Artem Fadeev, Mariia Sergeeva, Anna Ivanova, Daria Danilenko
EPI_ISL_430089 hCoV-19/Russia/StPetersburg-RII5170S/2020 Europe / Russia / St.Petersburg 2020-04-09
WHO National Influenza Centre Russian Federation WHO National Influenza Centre Russian Federation
Andrey Komissarov, Artem Fadeev, Mariia Sergeeva, Anna Ivanova, Daria Danilenko
EPI_ISL_430090 hCoV-19/Russia/StPetersburg-RII5244S/2020 Europe / Russia / St.Petersburg 2020-04-10
WHO National Influenza Centre Russian Federation WHO National Influenza Centre Russian Federation
Andrey Komissarov, Artem Fadeev, Mariia Sergeeva, Anna Ivanova, Daria Danilenko
EPI_ISL_430091 hCoV-19/Russia/StPetersburg-RII5247S/2020 Europe / Russia / St.Petersburg 2020-04-10
WHO National Influenza Centre Russian Federation WHO National Influenza Centre Russian Federation
Andrey Komissarov, Artem Fadeev, Mariia Sergeeva, Anna Ivanova, Daria Danilenko
EPI_ISL_430092 hCoV-19/Russia/StPetersburg-RII5633S/2020 Europe / Russia / St.Petersburg 2020-04-14
WHO National Influenza Centre Russian Federation WHO National Influenza Centre Russian Federation
Andrey Komissarov, Artem Fadeev, Mariia Sergeeva, Anna Ivanova, Daria Danilenko
EPI_ISL_430093 hCoV-19/Russia/StPetersburg-RII5634S/2020 Europe / Russia / St.Petersburg 2020-04-14
WHO National Influenza Centre Russian Federation WHO National Influenza Centre Russian Federation
Andrey Komissarov, Artem Fadeev, Mariia Sergeeva, Anna Ivanova, Daria Danilenko
EPI_ISL_430094 hCoV-19/Russia/StPetersburg-RII5635S/2020 Europe / Russia / St.Petersburg 2020-04-14
WHO National Influenza Centre Russian Federation WHO National Influenza Centre Russian Federation
Andrey Komissarov, Artem Fadeev, Mariia Sergeeva, Anna Ivanova, Daria Danilenko
EPI_ISL_430095 hCoV-19/Russia/StPetersburg-RII5642S/2020 Europe / Russia / St.Petersburg 2020-04-14
WHO National Influenza Centre Russian Federation WHO National Influenza Centre Russian Federation
Andrey Komissarov, Artem Fadeev, Mariia Sergeeva, Anna Ivanova, Daria Danilenko
EPI_ISL_430096 hCoV-19/Russia/StPetersburg-RII5643S/2020 Europe / Russia / St.Petersburg 2020-04-14
WHO National Influenza Centre Russian Federation WHO National Influenza Centre Russian Federation
Andrey Komissarov, Artem Fadeev, Mariia Sergeeva, Anna Ivanova, Daria Danilenko
EPI_ISL_430097 hCoV-19/Russia/StPetersburg-RII5644S/2020 Europe / Russia / St.Petersburg 2020-04-14
WHO National Influenza Centre Russian Federation WHO National Influenza Centre Russian Federation
Andrey Komissarov, Artem Fadeev, Mariia Sergeeva, Anna Ivanova, Daria Danilenko
EPI_ISL_430098 hCoV-19/Russia/StPetersburg-RII5654S/2020 Europe / Russia / St.Petersburg 2020-04-14
WHO National Influenza Centre Russian Federation WHO National Influenza Centre Russian Federation
Andrey Komissarov, Artem Fadeev, Mariia Sergeeva, Anna Ivanova, Daria Danilenko
EPI_ISL_430099 hCoV-19/Russia/StPetersburg-RII5942S/2020 Europe / Russia / St.Petersburg 2020-04-13
WHO National Influenza Centre Russian Federation WHO National Influenza Centre Russian Federation
Andrey Komissarov, Artem Fadeev, Mariia Sergeeva, Anna Ivanova, Daria Danilenko
EPI_ISL_430100 hCoV-19/Russia/StPetersburg-RII6027S/2020 Europe / Russia / St.Petersburg 2020-04-14
WHO National Influenza Centre Russian Federation WHO National Influenza Centre Russian Federation
Andrey Komissarov, Artem Fadeev, Mariia Sergeeva, Anna Ivanova, Daria Danilenko
EPI_ISL_430101 hCoV-19/Russia/StPetersburg-RII6031S/2020 Europe / Russia / St.Petersburg 2020-04-14
WHO National Influenza Centre Russian Federation WHO National Influenza Centre Russian Federation
Andrey Komissarov, Artem Fadeev, Mariia Sergeeva, Anna Ivanova, Daria Danilenko
EPI_ISL_430102 hCoV-19/Russia/StPetersburg-RII6039S/2020 Europe / Russia / St.Petersburg 2020-04-14
WHO National Influenza Centre Russian Federation WHO National Influenza Centre Russian Federation
Andrey Komissarov, Artem Fadeev, Mariia Sergeeva, Anna Ivanova, Daria Danilenko
EPI_ISL_430103 hCoV-19/Russia/StPetersburg-RII6043S/2020 Europe / Russia / St.Petersburg 2020-04-14
WHO National Influenza Centre Russian Federation WHO National Influenza Centre Russian Federation
Andrey Komissarov, Artem Fadeev, Mariia Sergeeva, Anna Ivanova, Daria Danilenko
EPI_ISL_430104 hCoV-19/Russia/StPetersburg-RII6053S/2020 Europe / Russia / St.Petersburg 2020-04-15
WHO National Influenza Centre Russian Federation WHO National Influenza Centre Russian Federation
Andrey Komissarov, Artem Fadeev, Mariia Sergeeva, Anna Ivanova, Daria Danilenko
EPI_ISL_430105 hCoV-19/Russia/StPetersburg-RII6055S/2020 Europe / Russia / St.Petersburg 2020-04-15
WHO National Influenza Centre Russian Federation WHO National Influenza Centre Russian Federation
Andrey Komissarov, Artem Fadeev, Mariia Sergeeva, Anna Ivanova, Daria Danilenko
EPI_ISL_430106 hCoV-19/Russia/StPetersburg-RII6056S/2020 Europe / Russia / St.Petersburg 2020-04-15
WHO National Influenza Centre Russian Federation WHO National Influenza Centre Russian Federation
Andrey Komissarov, Artem Fadeev, Mariia Sergeeva, Anna Ivanova, Daria Danilenko

EPI_ISL_430107 hCoV-19/Russia/StPetersburg-RII6060S/2020   Europe / Russia / St.Petersburg 2020-04-15
WHO National Influenza Centre Russian Federation   WHO National Influenza Centre Russian Federation
Andrey Komissarov, Artem Fadeev, Mariia Sergeeva, Anna Ivanova, Daria Danilenko
EPI_ISL_430108 hCoV-19/Russia/StPetersburg-RII6061S/2020   Europe / Russia / St.Petersburg 2020-04-15
WHO National Influenza Centre Russian Federation   WHO National Influenza Centre Russian Federation
Andrey Komissarov, Artem Fadeev, Mariia Sergeeva, Anna Ivanova, Daria Danilenko
EPI_ISL_430109 hCoV-19/Russia/StPetersburg-RII6063S/2020   Europe / Russia / St.Petersburg 2020-04-15
WHO National Influenza Centre Russian Federation   WHO National Influenza Centre Russian Federation
Andrey Komissarov, Artem Fadeev, Mariia Sergeeva, Anna Ivanova, Daria Danilenko
EPI_ISL_430110 hCoV-19/Russia/StPetersburg-RII6065S/2020   Europe / Russia / St.Petersburg 2020-04-15
WHO National Influenza Centre Russian Federation   WHO National Influenza Centre Russian Federation
Andrey Komissarov, Artem Fadeev, Mariia Sergeeva, Anna Ivanova, Daria Danilenko
EPI_ISL_430111 hCoV-19/Russia/StPetersburg-RII6086S/2020   Europe / Russia / St.Petersburg 2020-04-15
WHO National Influenza Centre Russian Federation   WHO National Influenza Centre Russian Federation
Andrey Komissarov, Artem Fadeev, Mariia Sergeeva, Anna Ivanova, Daria Danilenko
EPI_ISL_430112 hCoV-19/Russia/Ulan-Ude-RII4562V/2020   Europe / Russia / Buryat Republic / Ulan-Ude   2020-03-25   WHO National Influenza Centre Russian Federation   WHO National Influenza Centre Russian Federation   Andrey Komissarov, Artem Fadeev, Mariia Sergeeva, Anna Ivanova, Daria Danilenko
EPI_ISL_430113 hCoV-19/USA/WA-S259/2020   North America / USA / Washington / King County 2020-03-04
Seattle Flu Study   Seattle Flu Study   Chu et al
EPI_ISL_430114 hCoV-19/USA/WA-S260/2020   North America / USA / Washington / King County 2020-03-12
Seattle Flu Study   Seattle Flu Study   Chu et al
EPI_ISL_430115 hCoV-19/USA/WA-S261/2020   North America / USA / Washington   2020-03-30   Seattle Flu Study   Seattle Flu Study   Chu et al
EPI_ISL_430116 hCoV-19/USA/WA-S262/2020   North America / USA / Washington   2020-03-16   Seattle Flu Study   Seattle Flu Study   Chu et al
EPI_ISL_430117 hCoV-19/USA/WA-S263/2020   North America / USA / Washington / King County 2020-03-05
Seattle Flu Study   Seattle Flu Study   Chu et al
EPI_ISL_430118 hCoV-19/USA/WA-S264/2020   North America / USA / Washington / King County 2020-03-03
Seattle Flu Study   Seattle Flu Study   Chu et al
EPI_ISL_430119 hCoV-19/USA/WA-S265/2020   North America / USA / Washington / Snohomish County   2020-03-05   Seattle Flu Study   Seattle Flu Study   Chu et al
EPI_ISL_430121 hCoV-19/USA/WA-S267/2020   North America / USA / Washington / King County 2020-03-31
Seattle Flu Study   Seattle Flu Study   Chu et al
EPI_ISL_430122 hCoV-19/USA/WA-S268/2020   North America / USA / Washington / King County 2020-03-05
Seattle Flu Study   Seattle Flu Study   Chu et al
EPI_ISL_430123 hCoV-19/USA/WA-S269/2020   North America / USA / Washington / King County 2020-03-10
Seattle Flu Study   Seattle Flu Study   Chu et al
EPI_ISL_430124 hCoV-19/USA/WA-S270/2020   North America / USA / Washington / King County 2020-03-06
Seattle Flu Study   Seattle Flu Study   Chu et al
EPI_ISL_430125 hCoV-19/USA/WA-S271/2020   North America / USA / Washington / King County 2020-03-31
Seattle Flu Study   Seattle Flu Study   Chu et al
EPI_ISL_430126 hCoV-19/USA/WA-S272/2020   North America / USA / Washington / King County 2020-02-26
Seattle Flu Study   Seattle Flu Study   Chu et al
EPI_ISL_430127 hCoV-19/USA/WA-S273/2020   North America / USA / Washington / King County 2020-03-12
Seattle Flu Study   Seattle Flu Study   Chu et al
EPI_ISL_430128 hCoV-19/USA/WA-S274/2020   North America / USA / Washington / King County 2020-03-08
Seattle Flu Study   Seattle Flu Study   Chu et al
EPI_ISL_430129 hCoV-19/USA/WA-S275/2020   North America / USA / Washington / King County 2020-03-06
Seattle Flu Study   Seattle Flu Study   Chu et al
EPI_ISL_430130 hCoV-19/USA/WA-S276/2020   North America / USA / Washington   2020-04-01   Seattle Flu Study   Seattle Flu Study   Chu et al
EPI_ISL_430131 hCoV-19/USA/WA-S277/2020   North America / USA / Washington / King County 2020-02-22
Seattle Flu Study   Seattle Flu Study   Chu et al
EPI_ISL_430132 hCoV-19/USA/WA-S278/2020   North America / USA / Washington   2020-03-17   Seattle Flu Study   Seattle Flu Study   Chu et al
EPI_ISL_430133 hCoV-19/USA/WA-S279/2020   North America / USA / Washington / King County 2020-03-04
Seattle Flu Study   Seattle Flu Study   Chu et al
EPI_ISL_430134 hCoV-19/USA/WA-S280/2020   North America / USA / Washington / King County 2020-03-09
Seattle Flu Study   Seattle Flu Study   Chu et al
EPI_ISL_430135 hCoV-19/USA/WA-S281/2020   North America / USA / Washington / King County 2020-02-29
Seattle Flu Study   Seattle Flu Study   Chu et al
EPI_ISL_430136 hCoV-19/USA/WA-S282/2020   North America / USA / Washington / King County 2020-03-10
Seattle Flu Study   Seattle Flu Study   Chu et al
EPI_ISL_430137 hCoV-19/USA/WA-S283/2020   North America / USA / Washington / King County 2020-03-21
Seattle Flu Study   Seattle Flu Study   Chu et al
EPI_ISL_430140 hCoV-19/USA/WA-S286/2020   North America / USA / Washington / Pierce County   2020-03-15   Seattle Flu Study   Seattle Flu Study   Chu et al
EPI_ISL_430141 hCoV-19/USA/WA-S287/2020   North America / USA / Washington / King County 2020-03-06
Seattle Flu Study   Seattle Flu Study   Chu et al

EPI_ISL_430142 hCoV-19/USA/WA-S288/2020 North America / USA / Washington / King County 2020-03-06 Seattle Flu Study Seattle Flu Study Chu et al
EPI_ISL_430143 hCoV-19/USA/WA-S289/2020 North America / USA / Washington / King County 2020-03-23 Seattle Flu Study Seattle Flu Study Chu et al
EPI_ISL_430146 hCoV-19/USA/WA-S292/2020 North America / USA / Washington / King County 2020-03-05 Seattle Flu Study Seattle Flu Study Chu et al
EPI_ISL_430147 hCoV-19/USA/WA-S293/2020 North America / USA / Washington 2020-04-01 Seattle Flu Study Seattle Flu Study Chu et al
EPI_ISL_430148 hCoV-19/USA/WA-S294/2020 North America / USA / Washington / King County 2020-03-23 Seattle Flu Study Seattle Flu Study Chu et al
EPI_ISL_430149 hCoV-19/USA/WA-S295/2020 North America / USA / Washington / King County 2020-03-31 Seattle Flu Study Seattle Flu Study Chu et al
EPI_ISL_430150 hCoV-19/USA/WA-S296/2020 North America / USA / Washington / King County 2020-03-13 Seattle Flu Study Seattle Flu Study Chu et al
EPI_ISL_430151 hCoV-19/USA/WA-S297/2020 North America / USA / Washington / King County 2020-03-13 Seattle Flu Study Seattle Flu Study Chu et al
EPI_ISL_430152 hCoV-19/USA/WA-S298/2020 North America / USA / Washington / King County 2020-03-05 Seattle Flu Study Seattle Flu Study Chu et al
EPI_ISL_430153 hCoV-19/USA/WA-S299/2020 North America / USA / Washington / King County 2020-03-31 Seattle Flu Study Seattle Flu Study Chu et al
EPI_ISL_430154 hCoV-19/USA/WA-S300/2020 North America / USA / Washington / King County 2020-03-25 Seattle Flu Study Seattle Flu Study Chu et al
EPI_ISL_430155 hCoV-19/USA/WA-S301/2020 North America / USA / Washington / Pierce County 2020-03-14 Seattle Flu Study Seattle Flu Study Chu et al
EPI_ISL_430160 hCoV-19/USA/WA-S123/2020 North America / USA / Washington 2020-02-29 Washington State Department of Health Seattle Flu Study Chu et al
EPI_ISL_430161 hCoV-19/USA/WA-S124/2020 North America / USA / Washington / King County 2020-03-13 Washington State Department of Health Seattle Flu Study Chu et al
EPI_ISL_430162 hCoV-19/USA/WA-S125/2020 North America / USA / Washington / King County 2020-03-13 Washington State Department of Health Seattle Flu Study Chu et al
EPI_ISL_430163 hCoV-19/USA/WA-S126/2020 North America / USA / Washington / King County 2020-03-13 Washington State Department of Health Seattle Flu Study Chu et al
EPI_ISL_430164 hCoV-19/USA/WA-S127/2020 North America / USA / Washington / King County 2020-03-13 Washington State Department of Health Seattle Flu Study Chu et al
EPI_ISL_430165 hCoV-19/USA/WA-S128/2020 North America / USA / Washington / King County 2020-03-13 Washington State Department of Health Seattle Flu Study Chu et al
EPI_ISL_430166 hCoV-19/USA/WA-S129/2020 North America / USA / Washington / King County 2020-03-13 Washington State Department of Health Seattle Flu Study Chu et al
EPI_ISL_430167 hCoV-19/USA/WA-S130/2020 North America / USA / Washington / King County 2020-03-13 Washington State Department of Health Seattle Flu Study Chu et al
EPI_ISL_430168 hCoV-19/USA/WA-S131/2020 North America / USA / Washington / King County 2020-03-13 Washington State Department of Health Seattle Flu Study Chu et al
EPI_ISL_430169 hCoV-19/USA/WA-S132/2020 North America / USA / Washington / King County 2020-03-13 Washington State Department of Health Seattle Flu Study Chu et al
EPI_ISL_430170 hCoV-19/USA/WA-S133/2020 North America / USA / Washington / King County 2020-03-13 Washington State Department of Health Seattle Flu Study Chu et al
EPI_ISL_430171 hCoV-19/USA/WA-S134/2020 North America / USA / Washington / King County 2020-03-13 Washington State Department of Health Seattle Flu Study Chu et al
EPI_ISL_430172 hCoV-19/USA/WA-S135/2020 North America / USA / Washington / King County 2020-03-13 Washington State Department of Health Seattle Flu Study Chu et al
EPI_ISL_430173 hCoV-19/USA/WA-S136/2020 North America / USA / Washington / King County 2020-03-13 Washington State Department of Health Seattle Flu Study Chu et al
EPI_ISL_430174 hCoV-19/USA/WA-S137/2020 North America / USA / Washington / King County 2020-03-13 Washington State Department of Health Seattle Flu Study Chu et al
EPI_ISL_430175 hCoV-19/USA/WA-S138/2020 North America / USA / Washington / Snohomish County 2020-03-12 Washington State Department of Health Seattle Flu Study Chu et al
EPI_ISL_430176 hCoV-19/USA/WA-S139/2020 North America / USA / Washington / King County 2020-03-12 Washington State Department of Health Seattle Flu Study Chu et al
EPI_ISL_430177 hCoV-19/USA/WA-S140/2020 North America / USA / Washington / King County 2020-03-12 Washington State Department of Health Seattle Flu Study Chu et al
EPI_ISL_430178 hCoV-19/USA/WA-S141/2020 North America / USA / Washington / King County 2020-03-13 Washington State Department of Health Seattle Flu Study Chu et al
EPI_ISL_430212 hCoV-19/USA/WA-S174/2020 North America / USA / Washington / King County 2020-03-19 Washington State Department of Health Seattle Flu Study Chu et al
EPI_ISL_430213 hCoV-19/USA/WA-S175/2020 North America / USA / Washington / King County 2020-03-19 Washington State Department of Health Seattle Flu Study Chu et al
EPI_ISL_430214 hCoV-19/USA/WA-S176/2020 North America / USA / Washington / King County 2020-03-19 Washington State Department of Health Seattle Flu Study Chu et al
EPI_ISL_430215 hCoV-19/USA/WA-S177/2020 North America / USA / Washington / King County 2020-03-19 Washington State Department of Health Seattle Flu Study Chu et al

EPI_ISL_430216 hCoV-19/USA/WA-S178/2020   North America / USA / Washington / King County 2020-03-20
Washington State Department of Health   Seattle Flu Study   Chu et al
EPI_ISL_430217 hCoV-19/USA/WA-S179/2020   North America / USA / Washington / Whatcom County   2020-03-20   Washington State Department of Health   Seattle Flu Study   Chu et al
EPI_ISL_430219 hCoV-19/USA/WA-S181/2020   North America / USA / Washington   2020-03-19
Washington State Department of Health   Seattle Flu Study   Chu et al
EPI_ISL_430220 hCoV-19/USA/WA-S182/2020   North America / USA / Washington / Whatcom County   2020-03-20   Washington State Department of Health   Seattle Flu Study   Chu et al
EPI_ISL_430221 hCoV-19/USA/WA-S183/2020   North America / USA / Washington / King County 2020-03-19
Washington State Department of Health   Seattle Flu Study   Chu et al
EPI_ISL_430222 hCoV-19/USA/WA-S184/2020   North America / USA / Washington / King County 2020-03-19
Washington State Department of Health   Seattle Flu Study   Chu et al
EPI_ISL_430223 hCoV-19/USA/WA-S185/2020   North America / USA / Washington   2020-03-19
Washington State Department of Health   Seattle Flu Study   Chu et al
EPI_ISL_430224 hCoV-19/USA/WA-S186/2020   North America / USA / Washington / Whatcom County   2020-03-20   Washington State Department of Health   Seattle Flu Study   Chu et al
EPI_ISL_430225 hCoV-19/USA/WA-S187/2020   North America / USA / Washington / King County 2020-03-20
Washington State Department of Health   Seattle Flu Study   Chu et al
EPI_ISL_430226 hCoV-19/USA/WA-S188/2020   North America / USA / Washington / Whatcom County   2020-03-20   Washington State Department of Health   Seattle Flu Study   Chu et al
EPI_ISL_430227 hCoV-19/USA/WA-S189/2020   North America / USA / Washington / Whatcom County   2020-03-20   Washington State Department of Health   Seattle Flu Study   Chu et al
EPI_ISL_430228 hCoV-19/USA/WA-S190/2020   North America / USA / Washington / King County 2020-03-23
Washington State Department of Health   Seattle Flu Study   Chu et al
EPI_ISL_430229 hCoV-19/USA/WA-S191/2020   North America / USA / Washington / Whatcom County   2020-03-20   Washington State Department of Health   Seattle Flu Study   Chu et al
EPI_ISL_430230 hCoV-19/USA/WA-S192/2020   North America / USA / Washington / Whatcom County   2020-03-20   Washington State Department of Health   Seattle Flu Study   Chu et al
EPI_ISL_430231 hCoV-19/USA/WA-S193/2020   North America / USA / Washington / Grant County 2020-03-19
Washington State Department of Health   Seattle Flu Study   Chu et al
EPI_ISL_430232 hCoV-19/USA/WA-S194/2020   North America / USA / Washington / King County 2020-03-20
Washington State Department of Health   Seattle Flu Study   Chu et al
EPI_ISL_430233 hCoV-19/USA/WA-S195/2020   North America / USA / Washington / Whatcom County   2020-03-20   Washington State Department of Health   Seattle Flu Study   Chu et al
EPI_ISL_430234 hCoV-19/USA/WA-S196/2020   North America / USA / Washington / King County 2020-03-19
Washington State Department of Health   Seattle Flu Study   Chu et al
EPI_ISL_430235 hCoV-19/USA/WA-S197/2020   North America / USA / Washington / Whatcom County   2020-03-20   Washington State Department of Health   Seattle Flu Study   Chu et al
EPI_ISL_430236 hCoV-19/USA/WA-S198/2020   North America / USA / Washington / Whatcom County   2020-03-20   Washington State Department of Health   Seattle Flu Study   Chu et al
EPI_ISL_430237 hCoV-19/USA/WA-S199/2020   North America / USA / Washington / Whatcom County   2020-03-20   Washington State Department of Health   Seattle Flu Study   Chu et al
EPI_ISL_430238 hCoV-19/USA/WA-S200/2020   North America / USA / Washington / Yakima County   2020-03-20   Washington State Department of Health   Seattle Flu Study   Chu et al
EPI_ISL_430239 hCoV-19/USA/WA-S201/2020   North America / USA / Washington / King County 2020-03-20
Washington State Department of Health   Seattle Flu Study   Chu et al
EPI_ISL_430240 hCoV-19/USA/WA-S202/2020   North America / USA / Washington / Whatcom County   2020-03-20   Washington State Department of Health   Seattle Flu Study   Chu et al
EPI_ISL_430241 hCoV-19/USA/WA-S203/2020   North America / USA / Washington / Whatcom County   2020-03-20   Washington State Department of Health   Seattle Flu Study   Chu et al
EPI_ISL_430242 hCoV-19/USA/WA-S204/2020   North America / USA / Washington / Whatcom County   2020-03-22   Washington State Department of Health   Seattle Flu Study   Chu et al
EPI_ISL_430243 hCoV-19/USA/WA-S205/2020   North America / USA / Washington / Whatcom County   2020-03-20   Washington State Department of Health   Seattle Flu Study   Chu et al
EPI_ISL_430244 hCoV-19/USA/WA-S206/2020   North America / USA / Washington / Whatcom County   2020-03-20   Washington State Department of Health   Seattle Flu Study   Chu et al
EPI_ISL_430245 hCoV-19/USA/WA-S207/2020   North America / USA / Washington   2020-03-20
Washington State Department of Health   Seattle Flu Study   Chu et al
EPI_ISL_430246 hCoV-19/USA/WA-S208/2020   North America / USA / Washington / Whatcom County   2020-03-23   Washington State Department of Health   Seattle Flu Study   Chu et al
EPI_ISL_430247 hCoV-19/USA/WA-S209/2020   North America / USA / Washington / Whatcom County   2020-03-23   Washington State Department of Health   Seattle Flu Study   Chu et al
EPI_ISL_430248 hCoV-19/USA/WA-S210/2020   North America / USA / Washington / King County 2020-03-19
Washington State Department of Health   Seattle Flu Study   Chu et al
EPI_ISL_430249 hCoV-19/USA/WA-S211/2020   North America / USA / Washington / King County 2020-03-20
Washington State Department of Health   Seattle Flu Study   Chu et al
EPI_ISL_430250 hCoV-19/USA/WA-S212/2020   North America / USA / Washington   2020-03-19
Washington State Department of Health   Seattle Flu Study   Chu et al
EPI_ISL_430252 hCoV-19/USA/WA-S214/2020   North America / USA / Washington / Unknown County   2020-03-20   Washington State Department of Health   Seattle Flu Study   Chu et al

EPI_ISL_430253 hCoV-19/USA/WA-S215/2020 North America / USA / Washington 2020-03-20
Washington State Department of Health Seattle Flu Study Chu et al
EPI_ISL_430254 hCoV-19/USA/WA-S216/2020 North America / USA / Washington / Whatcom County 2020-
03-20 Washington State Department of Health Seattle Flu Study Chu et al
EPI_ISL_430255 hCoV-19/USA/WA-S217/2020 North America / USA / Washington / Whatcom County 2020-
03-20 Washington State Department of Health Seattle Flu Study Chu et al
EPI_ISL_430256 hCoV-19/USA/WA-S218/2020 North America / USA / Washington / Whatcom County 2020-
03-23 Washington State Department of Health Seattle Flu Study Chu et al
EPI_ISL_430257 hCoV-19/USA/WA-S219/2020 North America / USA / Washington / Pierce County 2020-
03-20 Washington State Department of Health Seattle Flu Study Chu et al
EPI_ISL_430258 hCoV-19/USA/WA-S220/2020 North America / USA / Washington / Whatcom County 2020-
03-20 Washington State Department of Health Seattle Flu Study Chu et al
EPI_ISL_430259 hCoV-19/USA/WA-S221/2020 North America / USA / Washington / King County 2020-03-19
Washington State Department of Health Seattle Flu Study Chu et al
EPI_ISL_430261 hCoV-19/USA/WA-S223/2020 North America / USA / Washington / Whatcom County 2020-
03-20 Washington State Department of Health Seattle Flu Study Chu et al
EPI_ISL_430262 hCoV-19/USA/WA-S224/2020 North America / USA / Washington / King County 2020-03-19
Washington State Department of Health Seattle Flu Study Chu et al
EPI_ISL_430263 hCoV-19/USA/WA-S225/2020 North America / USA / Washington / Whatcom County 2020-
03-20 Washington State Department of Health Seattle Flu Study Chu et al
EPI_ISL_430264 hCoV-19/USA/WA-S226/2020 North America / USA / Washington / Whatcom County 2020-
03-21 Washington State Department of Health Seattle Flu Study Chu et al
EPI_ISL_430265 hCoV-19/USA/WA-S227/2020 North America / USA / Washington 2020-03-20
Washington State Department of Health Seattle Flu Study Chu et al
EPI_ISL_430266 hCoV-19/USA/WA-S228/2020 North America / USA / Washington / Whatcom County 2020-
03-20 Washington State Department of Health Seattle Flu Study Chu et al
EPI_ISL_430267 hCoV-19/USA/WA-S229/2020 North America / USA / Washington / King County 2020-03-19
Washington State Department of Health Seattle Flu Study Chu et al
EPI_ISL_430268 hCoV-19/USA/WA-S230/2020 North America / USA / Washington / King County 2020-03-19
Washington State Department of Health Seattle Flu Study Chu et al
EPI_ISL_430269 hCoV-19/USA/WA-S231/2020 North America / USA / Washington / Whatcom County 2020-
03-20 Washington State Department of Health Seattle Flu Study Chu et al
EPI_ISL_430270 hCoV-19/USA/WA-S232/2020 North America / USA / Washington / Clark County 2020-03-08
Washington State Department of Health Seattle Flu Study Chu et al
EPI_ISL_430271 hCoV-19/USA/WA-S233/2020 North America / USA / Washington / Snohomish County 2020-
03-03 Washington State Department of Health Seattle Flu Study Chu et al
EPI_ISL_430272 hCoV-19/USA/WA-S234/2020 North America / USA / Washington / King County 2020-02-28
Washington State Department of Health Seattle Flu Study Chu et al
EPI_ISL_430273 hCoV-19/USA/WA-S235/2020 North America / USA / Washington / King County 2020-02-29
Washington State Department of Health Seattle Flu Study Chu et al
EPI_ISL_430274 hCoV-19/USA/WA-S236/2020 North America / USA / Washington / King County 2020-03-01
Washington State Department of Health Seattle Flu Study Chu et al
EPI_ISL_430275 hCoV-19/USA/WA-S237/2020 North America / USA / Washington 2020-03-06
Washington State Department of Health Seattle Flu Study Chu et al
EPI_ISL_430276 hCoV-19/USA/WA-S238/2020 North America / USA / Washington / King County 2020-03-02
Washington State Department of Health Seattle Flu Study Chu et al
EPI_ISL_430277 hCoV-19/USA/WA-S239/2020 North America / USA / Washington / King County 2020-03-07
Washington State Department of Health Seattle Flu Study Chu et al
EPI_ISL_430278 hCoV-19/USA/WA-S240/2020 North America / USA / Washington / King County 2020-03-06
Washington State Department of Health Seattle Flu Study Chu et al
EPI_ISL_430279 hCoV-19/USA/WA-S241/2020 North America / USA / Washington / King County 2020-03-04
Washington State Department of Health Seattle Flu Study Chu et al
EPI_ISL_430280 hCoV-19/USA/WA-S242/2020 North America / USA / Washington / King County 2020-03-01
Washington State Department of Health Seattle Flu Study Chu et al
EPI_ISL_430281 hCoV-19/USA/WA-S243/2020 North America / USA / Washington / King County 2020-02-29
Washington State Department of Health Seattle Flu Study Chu et al
EPI_ISL_430282 hCoV-19/USA/WA-S244/2020 North America / USA / Washington / Thurston County 2020-
03-11 Washington State Department of Health Seattle Flu Study Chu et al
EPI_ISL_430283 hCoV-19/USA/WA-S245/2020 North America / USA / Washington / Snohomish County 2020-
03-02 Washington State Department of Health Seattle Flu Study Chu et al
EPI_ISL_430285 hCoV-19/USA/WA-S247/2020 North America / USA / Washington / King County 2020-03-01
Washington State Department of Health Seattle Flu Study Chu et al
EPI_ISL_430286 hCoV-19/USA/WA-S248/2020 North America / USA / Washington / King County 2020-03-10
Washington State Department of Health Seattle Flu Study Chu et al
EPI_ISL_430287 hCoV-19/USA/WA-S249/2020 North America / USA / Washington / King County 2020-03-01
Washington State Department of Health Seattle Flu Study Chu et al
EPI_ISL_430288 hCoV-19/USA/WA-S250/2020 North America / USA / Washington / Snohomish County 2020-
03-03 Washington State Department of Health Seattle Flu Study Chu et al
EPI_ISL_430289 hCoV-19/USA/WA-S251/2020 North America / USA / Washington / Skagit County 2020-
03-11 Washington State Department of Health Seattle Flu Study Chu et al

EPI_ISL_430290 hCoV-19/USA/WA-S252/2020 North America / USA / Washington / King County 2020-03-02 Washington State Department of Health Seattle Flu Study Chu et al
EPI_ISL_430291 hCoV-19/USA/WA-S253/2020 North America / USA / Washington / Snohomish County 2020-03-07 Washington State Department of Health Seattle Flu Study Chu et al
EPI_ISL_430292 hCoV-19/USA/WA-S254/2020 North America / USA / Washington / Snohomish County 2020-02-24 Washington State Department of Health Seattle Flu Study Chu et al
EPI_ISL_430293 hCoV-19/USA/WA-S255/2020 North America / USA / Washington / Clark County 2020-03-08 Washington State Department of Health Seattle Flu Study Chu et al
EPI_ISL_430294 hCoV-19/USA/WA-S256/2020 North America / USA / Washington / King County 2020-03-02 Washington State Department of Health Seattle Flu Study Chu et al
EPI_ISL_430295 hCoV-19/USA/WA-S257/2020 North America / USA / Washington / King County 2020-02-25 Washington State Department of Health Seattle Flu Study Chu et al
EPI_ISL_430297 hCoV-19/South Africa/R02827/2020 Africa / South Africa / GP 2020-03-06 National Institute for Communicable Diseases of the National Health Laboratory Service National Institute for Communicable Diseases of the National Health Laboratory Service Allam M, Kwenda S, van Heusden P, Khumalo Z, Mohale T, Subramoney K, von Gottberg, A, Ismail A, Bhiman JN
EPI_ISL_450500 hCoV-19/Wuhan/OS52/2020 Asia / China / Hubei / Wuhan 2020-01-10 unknown CAS Key Laboratory of Special Pathogens and Biosafety and Center for Emerging Infectious Diseases Si,H., Zhu,Y., Lin,H., Xie,S., Shi,Z., Zhou,P.
EPI_ISL_450501 hCoV-19/Wuhan/YB012611/2020 Asia / China / Hubei / Wuhan 2020-01-26 unknown CAS Key Laboratory of Special Pathogens and Biosafety and Center for Emerging Infectious Diseases Si,H., Zhu,Y., Lin,H., Xie,S., Shi,Z., Zhou,P.
EPI_ISL_450502 hCoV-19/Wuhan/YB012605/2020 Asia / China / Hubei / Wuhan 2020-01-26 unknown CAS Key Laboratory of Special Pathogens and Biosafety and Center for Emerging Infectious Diseases Si,H., Zhu,Y., Lin,H., Xie,S., Shi,Z., Zhou,P.
EPI_ISL_450503 hCoV-19/Wuhan/YB012602/2020 Asia / China / Hubei / Wuhan 2020-01-26 unknown CAS Key Laboratory of Special Pathogens and Biosafety and Center for Emerging Infectious Diseases Si,H., Zhu,Y., Lin,H., Xie,S., Shi,Z., Zhou,P.
EPI_ISL_450504 hCoV-19/Wuhan/YB012506/2020 Asia / China / Hubei / Wuhan 2020-01-25 unknown CAS Key Laboratory of Special Pathogens and Biosafety and Center for Emerging Infectious Diseases Si,H., Zhu,Y., Lin,H., Xie,S., Shi,Z., Zhou,P.
EPI_ISL_450506 hCoV-19/Brazil/SP02cc/2020 South America / Brazil 2020-03-18 unknown Clinical Laboratory Malta,F., Amgarten,D., de Oliveira,D.B.L., Araujo,D.B., Machado,R.R.G., Santana,R.A.F., Mangueira,C.L.P., Durigon,E.L. and Pinho,J.R.R.
EPI_ISL_403928 hCoV-19/Wuhan/IPBCAMS-WH-05/2020 Asia / China / Hubei / Wuhan 2020-01-01 Institute of Pathogen Biology, Chinese Academy of Medical Sciences & Peking Union Medical College Institute of Pathogen Biology, Chinese Academy of Medical Sciences & Peking Union Medical College Lili Ren, Jianwei Wang, Qi Jin, Zichun Xiang, Zhiqiang Wu, Chao Wu, Yiwei Liu
EPI_ISL_403929 hCoV-19/Wuhan/IPBCAMS-WH-04/2019 Asia / China / Hubei / Wuhan 2019-12-30 Institute of Pathogen Biology, Chinese Academy of Medical Sciences & Peking Union Medical College Institute of Pathogen Biology, Chinese Academy of Medical Sciences & Peking Union Medical College Lili Ren, Jianwei Wang, Qi Jin, Zichun Xiang, Zhiqiang Wu, Chao Wu, Yiwei Liu
EPI_ISL_403930 hCoV-19/Wuhan/IPBCAMS-WH-03/2019 Asia / China / Hubei / Wuhan 2019-12-30 Institute of Pathogen Biology, Chinese Academy of Medical Sciences & Peking Union Medical College Institute of Pathogen Biology, Chinese Academy of Medical Sciences & Peking Union Medical College Lili Ren, Jianwei Wang, Qi Jin, Zichun Xiang, Zhiqiang Wu, Chao Wu, Yiwei Liu
EPI_ISL_403931 hCoV-19/Wuhan/IPBCAMS-WH-02/2019 Asia / China / Hubei / Wuhan 2019-12-30 Institute of Pathogen Biology, Chinese Academy of Medical Sciences & Peking Union Medical College Institute of Pathogen Biology, Chinese Academy of Medical Sciences & Peking Union Medical College Lili Ren, Jianwei Wang, Qi Jin, Zichun Xiang, Zhiqiang Wu, Chao Wu, Yiwei Liu
EPI_ISL_403932 hCoV-19/Guangdong/20SF012/2020 Asia / China / Guangdong / Shenzhen 2020-01-14 Guangdong Provincial Center for Diseases Control and Prevention; Guangdong Provincial Public Health Department of Microbiology, Guangdong Provincial Center for Diseases Control and Prevention Min Kang, Jie Wu, Jing Lu, Tao Liu, Baisheng Li, Shujiang Mei, Feng Ruan, Lifeng Lin, Changwen Ke, Haojie Zhong, Yingtao Zhang, Lirong Zou, Xuguang Chen, Qi Zhu, Jianpeng Xiao, Jianxiang Geng, Zhe Liu, Jianxiong Hu, Weilin Zeng, Xing Li, Yuhuang Liao, Xiujuan Tang, Songjian Xiao, Ying Wang, Yingchao Song, Xue Zhuang, Lijun Liang, Guanhao He, Huihong Deng, Tie Song, Jianfeng He, Wenjun Ma
EPI_ISL_403933 hCoV-19/Guangdong/20SF013/2020 Asia / China / Guangdong / Shenzhen 2020-01-15 Guangdong Provincial Center for Diseases Control and Prevention; Guangdong Provincial Public Health Department of Microbiology, Guangdong Provincial Center for Diseases Control and Prevention Min Kang, Jie Wu, Jing Lu, Tao Liu, Baisheng Li, Shujiang Mei, Feng Ruan, Lifeng Lin, Changwen Ke, Haojie Zhong, Yingtao Zhang, Lirong Zou, Xuguang Chen, Qi Zhu, Jianpeng Xiao, Jianxiang Geng, Zhe Liu, Jianxiong Hu, Weilin Zeng, Xing Li, Yuhuang Liao, Xiujuan Tang, Songjian Xiao, Ying Wang, Yingchao Song, Xue Zhuang, Lijun Liang, Guanhao He, Huihong Deng, Tie Song, Jianfeng He, Wenjun Ma
EPI_ISL_403934 hCoV-19/Guangdong/20SF014/2020 Asia / China / Guangdong / Shenzhen 2020-01-15 Guangdong Provincial Center for Diseases Control and Prevention; Guangdong Provincial Public Health Department of Microbiology, Guangdong Provincial Center for Diseases Control and Prevention Min Kang, Jie Wu, Jing Lu, Tao Liu, Baisheng Li, Shujiang Mei, Feng Ruan, Lifeng Lin, Changwen Ke, Haojie Zhong, Yingtao Zhang, Lirong Zou, Xuguang Chen, Qi Zhu, Jianpeng Xiao, Jianxiang Geng, Zhe Liu, Jianxiong Hu, Weilin Zeng, Xing Li, Yuhuang Liao, Xiujuan Tang, Songjian Xiao, Ying Wang, Yingchao Song, Xue Zhuang, Lijun Liang, Guanhao

He, Huihong Deng, Tie Song, Jianfeng He, Wenjun Ma
EPI_ISL_403935 hCoV-19/Guangdong/20SF025/2020 Asia / China / Guangdong / Shenzhen 2020-01-15 Guangdong Provincial Center for Diseases Control and Prevention; Guangdong Provincial Public Health Department of Microbiology, Guangdong Provincial Center for Diseases Control and Prevention Min Kang, Jie Wu, Jing Lu, Tao Liu, Baisheng Li, Shujiang Mei, Feng Ruan, Lifeng Lin, Changwen Ke, Haojie Zhong, Yingtao Zhang, Lirong Zou, Xuguang Chen, Qi Zhu, Jianpeng Xiao, Jianxiang Geng, Zhe Liu, Jianxiong Hu, Weilin Zeng, Xing Li, Yuhuang Liao, Xiujuan Tang, Songjian Xiao, Ying Wang, Yingchao Song, Xue Zhuang, Lijun Liang, Guanhao He, Huihong Deng, Tie Song, Jianfeng He, Wenjun Ma
EPI_ISL_403936 hCoV-19/Guangdong/20SF028/2020 Asia / China / Guangdong / Zhuhai 2020-01-17 Guangdong Provincial Center for Diseases Control and Prevention; Guangdong Provincial Public Health Department of Microbiology, Guangdong Provincial Center for Diseases Control and Prevention Min Kang, Jie Wu, Jing Lu, Tao Liu, Baisheng Li, Shujiang Mei, Feng Ruan, Lifeng Lin, Changwen Ke, Haojie Zhong, Yingtao Zhang, Lirong Zou, Xuguang Chen, Qi Zhu, Jianpeng Xiao, Jianxiang Geng, Zhe Liu, Jianxiong Hu, Weilin Zeng, Xing Li, Yuhuang Liao, Xiujuan Tang, Songjian Xiao, Ying Wang, Yingchao Song, Xue Zhuang, Lijun Liang, Guanhao He, Huihong Deng, Tie Song, Jianfeng He, Wenjun Ma
EPI_ISL_403937 hCoV-19/Guangdong/20SF040/2020 Asia / China / Guangdong / Zhuhai 2020-01-18 Guangdong Provincial Center for Diseases Control and Prevention; Guangdong Provincial Public Health Department of Microbiology, Guangdong Provincial Center for Diseases Control and Prevention Min Kang, Jie Wu, Jing Lu, Tao Liu, Baisheng Li, Shujiang Mei, Feng Ruan, Lifeng Lin, Changwen Ke, Haojie Zhong, Yingtao Zhang, Lirong Zou, Xuguang Chen, Qi Zhu, Jianpeng Xiao, Jianxiang Geng, Zhe Liu, Jianxiong Hu, Weilin Zeng, Xing Li, Yuhuang Liao, Xiujuan Tang, Songjian Xiao, Ying Wang, Yingchao Song, Xue Zhuang, Lijun Liang, Guanhao He, Huihong Deng, Tie Song, Jianfeng He, Wenjun Ma
EPI_ISL_403962 hCoV-19/Thailand/61/2020 Asia / Thailand / Nonthaburi 2020-01-08 Bamrasnaradura Hospital 1. Department of Medical Sciences, Ministry of Public Health, Thailand 2. Thai Red Cross Emerging Infectious Diseases - Health Science Centre 3. Department of Disease Control, Ministry of Public Health, Thailand Pilailuk,Okada; Siripaporn,Phuygun; Thanutsapa,Thanadachakul; Supaporn,Wacharapluesadee; Sittiporn,Parnmen; Warawan,Wongboot; Sunthareeya,Waicharoen; Rome,Buathong; Malinee,Chittaganpitch; Nanthawan,Mekha
EPI_ISL_403963 hCoV-19/Thailand/74/2020 Asia / Thailand / Nonthaburi 2020-01-13 Bamrasnaradura Hospital 1. Department of Medical Sciences, Ministry of Public Health, Thailand 2. Thai Red Cross Emerging Infectious Diseases - Health Science Centre 3. Department of Disease Control, Ministry of Public Health, Thailand Pilailuk,Okada; Siripaporn,Phuygun; Thanutsapa,Thanadachakul; Supaporn,Wacharapluesadee; Sittiporn,Parnmen; Warawan,Wongboot; Sunthareeya,Waicharoen; Rome,Buathong; Malinee,Chittaganpitch; Nanthawan,Mekha
EPI_ISL_406223 hCoV-19/USA/AZ1/2020 North America / USA / Arizona / Phoenix 2020-01-22 Arizona Department of Health Services Pathogen Discovery, Respiratory Viruses Branch, Division of Viral Diseases, Centers for Disease Control and Prevention Ying Tao, Clinton R. Paden, Krista Queen, Anna Uehara, Yan Li, Jing Zhang, Xiaoyan Lu, Brian Lynch, Senthil Kumar K. Sakthivel, Brett L. Whitaker, Shifaq Kamili, Lijuan Wang, Janna' R. Murray, Susan I. Gerber, Stephen Lindstrom, Suxiang Tong
EPI_ISL_406592 hCoV-19/Shenzhen/SZTH-001/2020 Asia / China / Guangdong / Shenzhen 2020-01-13 Shenzhen Third People's Hospital Shenzhen Key Laboratory of Pathogen and Immunity, National Clinical Research Center for Infectious Disease,Shenzhen Third People's Hospital Yang Yang, Chenguang Shen, Li Xing, Zhixiang Xu, Haixia Zheng, Yingxia Liu
EPI_ISL_406593 hCoV-19/Shenzhen/SZTH-002/2020 Asia / China / Guangdong / Shenzhen 2020-01-13 Shenzhen Key Laboratory of Pathogen and Immunity, National Clinical Research Center for Infectious Disease, Shenzhen Third People's Hospital Shenzhen Key Laboratory of Pathogen and Immunity, National Clinical Research Center for Infectious Disease, Shenzhen Third People's Hospital Yang Yang, Chenguang Shen, Li Xing, Zhixiang Xu, Haixia Zheng, Yingxia Liu
EPI_ISL_406594 hCoV-19/Shenzhen/SZTH-003/2020 Asia / China / Guangdong / Shenzhen 2020-01-16 Shenzhen Key Laboratory of Pathogen and Immunity, National Clinical Research Center for Infectious Disease, Shenzhen Third People's Hospital Shenzhen Key Laboratory of Pathogen and Immunity, National Clinical Research Center for Infectious Disease, Shenzhen Third People's Hospital Yang Yang, Chenguang Shen, Li Xing, Zhixiang Xu, Haixia Zheng, Yingxia Liu
EPI_ISL_406595 hCoV-19/Shenzhen/SZTH-004/2020 Asia / China / Guangdong / Shenzhen 2020-01-16 Shenzhen Key Laboratory of Pathogen and Immunity, National Clinical Research Center for Infectious Disease, Shenzhen Third People's Hospital Shenzhen Key Laboratory of Pathogen and Immunity, National Clinical Research Center for Infectious Disease, Shenzhen Third People's Hospital Yang Yang, Chenguang Shen, Li Xing, Zhixiang Xu, Haixia Zheng, Yingxia Liu
EPI_ISL_406596 hCoV-19/France/IDF0372/2020 Europe / France / Ile-de-France / Paris 2020-01-23 Department of Infectious and Tropical Diseases, Bichat Claude Bernard Hospital, Paris National Reference Center for Viruses of Respiratory Infections, Institut Pasteur, Paris Mélanie Albert, Marion Barbet, Sylvie Behillil, Méline Bizard, Angela Brisebarre, Flora Donati, Vincent Enouf, Maud Vanpeene, Sylvie van der Werf, Yazdan Yazdanpanah, Xavier Lescure.
EPI_ISL_406597 hCoV-19/France/IDF0373/2020 Europe / France / Ile-de-France / Paris 2020-01-23 Department of Infectious and Tropical Diseases, Bichat Claude Bernard Hospital, Paris National Reference Center for Viruses of Respiratory Infections, Institut Pasteur, Paris Mélanie Albert, Marion Barbet, Sylvie Behillil, Méline Bizard, Angela Brisebarre, Flora Donati, Vincent Enouf, Maud Vanpeene, Sylvie van der Werf, Yazdan Yazdanpanah, Xavier Lescure.
EPI_ISL_407893 hCoV-19/Australia/NSW01/2020 Oceania / Australia / New South Wales / Sydney 2020-01-24 Centre for Infectious Diseases and Microbiology Laboratory Services NSW Health Pathology - Institute of

Clinical Pathology and Medical Research; Westmead Hospital; University of Sydney Eden J-S, Carter I, Rahman H, Holmes EC, Rockett R, O'Sullivan MV, Sintchenko V, Chen SC, Maddocks S, Kok J and Dwyer DE for the 2019-nCoV Study Group
EPI_ISL_407894 hCoV-19/Australia/QLD01/2020 Oceania / Australia / Queensland / Gold Coast 2020-01-28 Pathology Queensland Public Health Virology Laboratory Ben Huang, Alyssa Pyke, Amanda De Jong, Andrew Van Den Hurk, Carmel Taylor, David Warrilow, Doris Genge, Elisabeth Gamez, Glen Hewitson, Ian Maxwell Mackay, Inga Sultana, Jamie McMahon, Jean Barcelon, Judy Northill, Mitchell Finger, Natalie Simpson, Neelima Nair, Peter Burtonclay, Peter Moore, Sarah Wheatley, Sean Moody, Sonja Hall-Mendelin, Timothy Gardam, and Frederick Moore.
EPI_ISL_407896 hCoV-19/Australia/QLD02/2020 Oceania / Australia / Queensland / Gold Coast 2020-01-30 Pathology Queensland Public Health Virology Laboratory Ben Huang, Alyssa Pyke, Amanda De Jong, Andrew Van Den Hurk, Carmel Taylor, David Warrilow, Doris Genge, Elisabeth Gamez, Glen Hewitson, Ian Maxwell Mackay, Inga Sultana, Jamie McMahon, Jean Barcelon, Judy Northill, Mitchell Finger, Natalie Simpson, Neelima Nair, Peter Burtonclay, Peter Moore, Sarah Wheatley, Sean Moody, Sonja Hall-Mendelin, Timothy Gardam, and Frederick Moore.
EPI_ISL_411060 hCoV-19/Fujian/8/2020 Asia / China / Fujian 2020-01-21 Fujian Center for Disease Control and Prevention Fujian Center for Disease Control and Prevention Chen Wei, Zhang Yanhua, He Wenxiang, Weng Yuwei
EPI_ISL_411066 hCoV-19/Fujian/13/2020 Asia / China / Fujian 2020-01-22 Fujian Center for Disease Control and Prevention Fujian Center for Disease Control and Prevention Chen Wei, Zhang Yanhua, He Wenxiang, Weng Yuwei
EPI_ISL_412026 hCoV-19/Hefei/2/2020 Asia / China / Anhui / Hefei 2020-02-23 Second Hospital of Anhui Medical University Second Hospital of Anhui Medical University Changtai Wang, Zhongping Liua, Zixiang Chen, Xin Huang, Mengyuan Xua, Tengfei He, Mengji Lu, Zhenhua Zhang
EPI_ISL_412028 hCoV-19/Hong Kong/VM20001061-2/2020 Asia / Hong Kong 2020-01-22 Hong Kong Department of Health School of Public Health, The University of Hon g Kong Dominic N.C. Tsang, Daniel K.W. Chu, Leo L.M. Poon, Malik Peiris
EPI_ISL_412029 hCoV-19/Hong Kong/VB20024950-2/2020 Asia / Hong Kong 2020-01-30 Hong Kong Department of Health The University of Hong Kong Dominic N.C. Tsang, Daniel K.W. Chu, Leo L.M. Poon, Malik Peiris
EPI_ISL_412030 hCoV-19/Hong Kong/VB20026565-2/2020 Asia / Hong Kong 2020-02-01 Hong Kong Department of Health School of Public Health, The University of Hon g Kong Dominic N.C. Tsang, Daniel K.W. Chu, Leo L.M. Poon, Malik Peiris
EPI_ISL_413602 hCoV-19/Finland/FIN03032020A/2020 Europe / Finland / Helsinki 2020-03-03 Department of Virology and Immunology, University of Helsinki and Helsinki University Hospital, Huslab Finland Department of Virology, Faculty of Medicine, University of Helsinki, Helsinki, Finland Teemu Smura, Hannimari Kallio-Kokko, Olli Vapalahti
EPI_ISL_413603 hCoV-19/Finland/FIN03032020B/2020 Europe / Finland / Helsinki 2020-03-03 Department of Virology and Immunology, University of Helsinki and Helsinki University Hospital, Huslab Finland Department of Virology, Faculty of Medicine, University of Helsinki, Helsinki, Finland Teemu Smura, Hannimari Kallio-Kokko, Olli Vapalahti
EPI_ISL_413604 hCoV-19/Finland/FIN03032020C/2020 Europe / Finland / Helsinki 2020-03-03 Department of Virology and Immunology, University of Helsinki and Helsinki University Hospital, Huslab Finland Department of Virology, Faculty of Medicine, University of Helsinki, Helsinki, Finland Teemu Smura, Hannimari Kallio-Kokko, Olli Vapalahti
EPI_ISL_413606 hCoV-19/USA/CruiseA-1/2020 North America / USA 2020-02-17 unknown Pathogen Discovery, Respiratory Viruses Branch, Division of Viral Diseases, Centers for Dieases Control and Prevention Anna Uehara, Ying Tao, Clinton R. Paden, Krista Queen, Jing Zhang, Yan Li, Mary S. Keckler, Alison S Laufer Halpin, Haibin Wang, Jasmine Padilla, Justin Lee, Christopher A. Elkins, Susan I. Gerber, Suxiang Tong
EPI_ISL_413607 hCoV-19/USA/CruiseA-2/2020 North America / USA 2020-02-18 unknown Pathogen Discovery, Respiratory Viruses Branch, Division of Viral Diseases, Centers for Dieases Control and Prevention Anna Uehara, Ying Tao, Clinton R. Paden, Krista Queen, Jing Zhang, Yan Li, Mary S. Keckler, Alison S Laufer Halpin, Haibin Wang, Jasmine Padilla, Justin Lee, Christopher A. Elkins, Susan I. Gerber, Suxiang Tong
EPI_ISL_413608 hCoV-19/USA/CruiseA-3/2020 North America / USA 2020-02-18 unknown Pathogen Discovery, Respiratory Viruses Branch, Division of Viral Diseases, Centers for Dieases Control and Prevention Anna Uehara, Ying Tao, Clinton R. Paden, Krista Queen, Jing Zhang, Yan Li, Mary S. Keckler, Alison S Laufer Halpin, Haibin Wang, Jasmine Padilla, Justin Lee, Christopher A. Elkins, Susan I. Gerber, Suxiang Tong
EPI_ISL_413609 hCoV-19/USA/CruiseA-4/2020 North America / USA 2020-02-21 unknown Pathogen Discovery, Respiratory Viruses Branch, Division of Viral Diseases, Centers for Dieases Control and Prevention Anna Uehara, Ying Tao, Clinton R. Paden, Krista Queen, Jing Zhang, Yan Li, Mary S. Keckler, Alison S Laufer Halpin, Haibin Wang, Jasmine Padilla, Justin Lee, Christopher A. Elkins, Susan I. Gerber, Suxiang Tong
EPI_ISL_413610 hCoV-19/USA/CruiseA-5/2020 North America / USA 2020-02-21 unknown Pathogen Discovery, Respiratory Viruses Branch, Division of Viral Diseases, Centers for Dieases Control and Prevention Anna Uehara, Ying Tao, Clinton R. Paden, Krista Queen, Jing Zhang, Yan Li, Mary S. Keckler, Alison S Laufer Halpin, Haibin Wang, Jasmine Padilla, Justin Lee, Christopher A. Elkins, Susan I. Gerber, Suxiang Tong
EPI_ISL_413611 hCoV-19/USA/CruiseA-6/2020 North America / USA 2020-02-21 unknown Pathogen Discovery, Respiratory Viruses Branch, Division of Viral Diseases, Centers for Dieases Control and Prevention Anna Uehara, Ying Tao, Clinton R. Paden, Krista Queen, Jing Zhang, Yan Li, Mary S. Keckler, Alison S Laufer Halpin, Haibin Wang, Jasmine Padilla, Justin Lee, Christopher A. Elkins, Susan I. Gerber, Suxiang Tong
EPI_ISL_413612 hCoV-19/USA/CruiseA-7/2020 North America / USA 2020-02-17 unknown Pathogen

Discovery, Respiratory Viruses Branch, Division of Viral Diseases, Centers for Dieases Control and Prevention Ying Tao, Clinton R. Paden, Krista Queen, Anna Uehara, Jing Zhang, Yan Li, Haibin Wang, Shifaq Kamili, Xiaoyan Lu, Brian Lynch, Senthil Kumar K. Sakthivel, Brett L. Whitaker, Lijuan Wang, Janna' R. Murray, Jasmine Padilla, Justin Lee, Susan I. Gerber, Stephen Lindstrom, Suxiang Tong
EPI_ISL_413613 hCoV-19/USA/CruiseA-8/2020 North America / USA 2020-02-17 unknown Pathogen Discovery, Respiratory Viruses Branch, Division of Viral Diseases, Centers for Dieases Control and Prevention Ying Tao, Clinton R. Paden, Krista Queen, Anna Uehara, Jing Zhang, Yan Li, Haibin Wang, Shifaq Kamili, Xiaoyan Lu, Brian Lynch, Senthil Kumar K. Sakthivel, Brett L. Whitaker, Lijuan Wang, Janna' R. Murray, Jasmine Padilla, Justin Lee, Susan I. Gerber, Stephen Lindstrom, Suxiang Tong
EPI_ISL_413614 hCoV-19/USA/CruiseA-9/2020 North America / USA 2020-02-17 unknown Pathogen Discovery, Respiratory Viruses Branch, Division of Viral Diseases, Centers for Dieases Control and Prevention Ying Tao, Clinton R. Paden, Krista Queen, Anna Uehara, Jing Zhang, Yan Li, Haibin Wang, Shifaq Kamili, Xiaoyan Lu, Brian Lynch, Senthil Kumar K. Sakthivel, Brett L. Whitaker, Lijuan Wang, Janna' R. Murray, Jasmine Padilla, Justin Lee, Susan I. Gerber, Stephen Lindstrom, Suxiang Tong
EPI_ISL_413615 hCoV-19/USA/CruiseA-10/2020 North America / USA 2020-02-17 unknown Pathogen Discovery, Respiratory Viruses Branch, Division of Viral Diseases, Centers for Dieases Control and Prevention Ying Tao, Clinton R. Paden, Krista Queen, Anna Uehara, Jing Zhang, Yan Li, Haibin Wang, Shifaq Kamili, Xiaoyan Lu, Brian Lynch, Senthil Kumar K. Sakthivel, Brett L. Whitaker, Lijuan Wang, Janna' R. Murray, Jasmine Padilla, Justin Lee, Susan I. Gerber, Stephen Lindstrom, Suxiang Tong
EPI_ISL_413616 hCoV-19/USA/CruiseA-11/2020 North America / USA 2020-02-17 unknown Pathogen Discovery, Respiratory Viruses Branch, Division of Viral Diseases, Centers for Dieases Control and Prevention Ying Tao, Clinton R. Paden, Krista Queen, Anna Uehara, Jing Zhang, Yan Li, Haibin Wang, Shifaq Kamili, Xiaoyan Lu, Brian Lynch, Senthil Kumar K. Sakthivel, Brett L. Whitaker, Lijuan Wang, Janna' R. Murray, Jasmine Padilla, Justin Lee, Susan I. Gerber, Stephen Lindstrom, Suxiang Tong
EPI_ISL_413617 hCoV-19/USA/CruiseA-12/2020 North America / USA 2020-02-20 unknown Pathogen Discovery, Respiratory Viruses Branch, Division of Viral Diseases, Centers for Dieases Control and Prevention Ying Tao, Clinton R. Paden, Krista Queen, Anna Uehara, Jing Zhang, Yan Li, Haibin Wang, Shifaq Kamili, Xiaoyan Lu, Brian Lynch, Senthil Kumar K. Sakthivel, Brett L. Whitaker, Lijuan Wang, Janna' R. Murray, Jasmine Padilla, Justin Lee, Susan I. Gerber, Stephen Lindstrom, Suxiang Tong
EPI_ISL_413618 hCoV-19/USA/CruiseA-13/2020 North America / USA 2020-02-20 unknown Pathogen Discovery, Respiratory Viruses Branch, Division of Viral Diseases, Centers for Dieases Control and Prevention Clinton R. Paden, Ying Tao, Krista Queen, Anna Uehara, Jing Zhang, Yan Li, Haibin Wang, Shifaq Kamili, Xiaoyan Lu, Brian Lynch, Senthil Kumar K. Sakthivel, Brett L. Whitaker, Lijuan Wang, Janna' R. Murray, Jasmine Padilla, Justin Lee, Susan I. Gerber, Stephen Lindstrom, Suxiang Tong
EPI_ISL_413619 hCoV-19/USA/CruiseA-14/2020 North America / USA 2020-02-25 unknown Pathogen Discovery, Respiratory Viruses Branch, Division of Viral Diseases, Centers for Dieases Control and Prevention Clinton R. Paden, Ying Tao, Krista Queen, Anna Uehara, Jing Zhang, Yan Li, Haibin Wang, Shifaq Kamili, Xiaoyan Lu, Brian Lynch, Senthil Kumar K. Sakthivel, Brett L. Whitaker, Lijuan Wang, Janna' R. Murray, Jasmine Padilla, Justin Lee, Susan I. Gerber, Stephen Lindstrom, Suxiang Tong
EPI_ISL_413620 hCoV-19/USA/CruiseA-15/2020 North America / USA 2020-02-18 unknown Pathogen Discovery, Respiratory Viruses Branch, Division of Viral Diseases, Centers for Dieases Control and Prevention Clinton R. Paden, Ying Tao, Krista Queen, Anna Uehara, Jing Zhang, Yan Li, Haibin Wang, Shifaq Kamili, Xiaoyan Lu, Brian Lynch, Senthil Kumar K. Sakthivel, Brett L. Whitaker, Lijuan Wang, Janna' R. Murray, Jasmine Padilla, Justin Lee, Susan I. Gerber, Stephen Lindstrom, Suxiang Tong
EPI_ISL_413621 hCoV-19/USA/CruiseA-16/2020 North America / USA 2020-02-18 unknown Pathogen Discovery, Respiratory Viruses Branch, Division of Viral Diseases, Centers for Dieases Control and Prevention Clinton R. Paden, Ying Tao, Krista Queen, Anna Uehara, Jing Zhang, Yan Li, Haibin Wang, Shifaq Kamili, Xiaoyan Lu, Brian Lynch, Senthil Kumar K. Sakthivel, Brett L. Whitaker, Lijuan Wang, Janna' R. Murray, Jasmine Padilla, Justin Lee, Susan I. Gerber, Stephen Lindstrom, Suxiang Tong
EPI_ISL_413622 hCoV-19/USA/CruiseA-17/2020 North America / USA 2020-02-24 unknown Pathogen Discovery, Respiratory Viruses Branch, Division of Viral Diseases, Centers for Dieases Control and Prevention Clinton R. Paden, Ying Tao, Krista Queen, Anna Uehara, Jing Zhang, Yan Li, Haibin Wang, Shifaq Kamili, Xiaoyan Lu, Brian Lynch, Senthil Kumar K. Sakthivel, Brett L. Whitaker, Lijuan Wang, Janna' R. Murray, Jasmine Padilla, Justin Lee, Susan I. Gerber, Stephen Lindstrom, Suxiang Tong
EPI_ISL_413623 hCoV-19/USA/CruiseA-18/2020 North America / USA 2020-02-24 unknown Pathogen Discovery, Respiratory Viruses Branch, Division of Viral Diseases, Centers for Dieases Control and Prevention Clinton R. Paden, Ying Tao, Krista Queen, Anna Uehara, Jing Zhang, Yan Li, Haibin Wang, Shifaq Kamili, Xiaoyan Lu, Brian Lynch, Senthil Kumar K. Sakthivel, Brett L. Whitaker, Lijuan Wang, Janna' R. Murray, Jasmine Padilla, Justin Lee, Susan I. Gerber, Stephen Lindstrom, Suxiang Tong
EPI_ISL_413647 hCoV-19/Portugal/CV62/2020 Europe / Portugal 2020-03-01 Centro Hospital do Porto, E.P.E. - H. Geral de Santo Antonio Instituto Nacional de Saude (INSA) Raquel Guiomar, Inês Costa, Pedro Pechirra, Joana Mendonça, Luís Vieira, Helena Ramos, Joana Isidro, Vítor Borges, João Paulo Gomes
EPI_ISL_413648 hCoV-19/Portugal/CV63/2020 Europe / Portugal 2020-03-01 Centro Hospitalar e Universitário de Sao Joao, Porto Instituto Nacional de Saude (INSA) Raquel Guiomar, Inês Costa, Pedro Pechirra, Joana Mendonça, Luís Vieira, João Tiago Guimarães, Joana Isidro, Vítor Borges, João Paulo Gomes
EPI_ISL_413649 hCoV-19/USA/WA14-UW10/2020 North America / USA / Washington 2020-03-05 UW Virology Lab UW Virology Lab Pavitra Roychoudhury, Hong Xie, Keith Jerome, Alexander Greninger
EPI_ISL_413650 hCoV-19/USA/WA15-UW11/2020 North America / USA / Washington 2020-03-05 UW Virology Lab UW Virology Lab Pavitra Roychoudhury, Hong Xie, Keith Jerome, Alexander Greninger
EPI_ISL_413651 hCoV-19/USA/WA16-UW12/2020 North America / USA / Washington 2020-03-05 UW

Virology Lab	UW Virology Lab Pavitra Roychoudhury, Hong Xie, Keith Jerome, Alexander Greninger
EPI_ISL_413652	hCoV-19/USA/WA17-UW13/2020	North America / USA / Washington	2020-03-05	UW Virology Lab	UW Virology Lab Pavitra Roychoudhury, Hong Xie, Keith Jerome, Alexander Greninger
EPI_ISL_413653	hCoV-19/USA/WA18-UW14/2020	North America / USA / Washington	2020-03-05	UW Virology Lab	UW Virology Lab Pavitra Roychoudhury, Hong Xie, Keith Jerome, Alexander Greninger
EPI_ISL_413691	hCoV-19/China/WF0001/2020	Asia / China	2020-01 Weifang Center for Disease Control and Prevention	Weifang Center for Disease Control and Prevention & BGI-Shenzhen	Qing Nie, Xingguang Li, Erik M Volz, Han Fu, Haowei Wang, Xiaoyue Xi, Wei Chen, Dehui Liu, Yingying Chen, Mengmeng Tian, Wei Tan, Junjie Zai, Wanying Sun, Jiandong Li, Junhua Li
EPI_ISL_413692	hCoV-19/China/WF0002/2020	Asia / China	2020-01 Weifang Center for Disease Control and Prevention	Weifang Center for Disease Control and Prevention & BGI-Shenzhen	Qing Nie, Xingguang Li, Erik M Volz, Han Fu, Haowei Wang, Xiaoyue Xi, Wei Chen, Dehui Liu, Yingying Chen, Mengmeng Tian, Wei Tan, Junjie Zai, Wanying Sun, Jiandong Li, Junhua Li
EPI_ISL_413693	hCoV-19/China/WF0003/2020	Asia / China	2020-01 Weifang Center for Disease Control and Prevention	Weifang Center for Disease Control and Prevention & BGI-Shenzhen	Qing Nie, Xingguang Li, Erik M Volz, Han Fu, Haowei Wang, Xiaoyue Xi, Wei Chen, Dehui Liu, Yingying Chen, Mengmeng Tian, Wei Tan, Junjie Zai, Wanying Sun, Jiandong Li, Junhua Li
EPI_ISL_413694	hCoV-19/China/WF0004/2020	Asia / China	2020-01 Weifang Center for Disease Control and Prevention	Weifang Center for Disease Control and Prevention & BGI-Shenzhen	Qing Nie, Xingguang Li, Erik M Volz, Han Fu, Haowei Wang, Xiaoyue Xi, Wei Chen, Dehui Liu, Yingying Chen, Mengmeng Tian, Wei Tan, Junjie Zai, Wanying Sun, Jiandong Li, Junhua Li
EPI_ISL_413697	hCoV-19/China/WF0012/2020	Asia / China	2020-02 Weifang Center for Disease Control and Prevention	Weifang Center for Disease Control and Prevention & BGI-Shenzhen	Qing Nie, Xingguang Li, Erik M Volz, Han Fu, Haowei Wang, Xiaoyue Xi, Wei Chen, Dehui Liu, Yingying Chen, Mengmeng Tian, Wei Tan, Junjie Zai, Wanying Sun, Jiandong Li, Junhua Li
EPI_ISL_414600	hCoV-19/France/GE1583/2020	Europe / France / Grand-Est / Strasbourg	2020-02-26	Laboratoire de Virologie Institut de Virologie - INSERM U 1109 Hôpitaux Universitaires de Strasbourg	National Reference Center for Viruses of Respiratory Infections, Institut Pasteur, Paris	Mélnie Albert, Marion Barbet, Sylvie Behillil, Méline Bizard, Angela Brisebarre, Flora Donati Vincent Enouf, Maud Vanpeene, Sylvie van der Werf, Samira Fafi-Kremer
EPI_ISL_414620	hCoV-19/USA/WA-UW33/2020	North America / USA	2020-03-08	UW Virology Lab UW Virology Lab	Pavitra Roychoudhury, Hong Xie, Keith Jerome, Alexander Greninger
EPI_ISL_414625	hCoV-19/France/PL1643/2020	Europe / France / Pays de la Loire / Nantes	2020-02-26	Centre Hospitalier Régional Universitaire de Nantes Laboratoire de Virologie	National Reference Center for Viruses of Respiratory Infections, Institut Pasteur, Paris	Mélnie Albert, Marion Barbet, Sylvie Behillil, Méline Bizard, Angela Brisebarre, Flora Donati Vincent Enouf, Maud Vanpeene, Sylvie van der Werf, Marianne Coste-Burel
EPI_ISL_414626	hCoV-19/France/HF1684/2020	Europe / France / Hauts de France / Crépy en Valois	2020-02-29	unknown National Reference Center for Viruses of Respiratory Infections, Institut Pasteur, Paris	Mélnie Albert, Marion Barbet, Sylvie Behillil, Méline Bizard, Angela Brisebarre, Flora Donati Vincent Enouf, Maud Vanpeene, Sylvie van der Werf
EPI_ISL_414627	hCoV-19/France/HF1795/2020	Europe / France / Hauts de France / Compiègne	2020-03-02	Centre Hospitalier Compiègne Laboratoire de Biologie	National Reference Center for Viruses of Respiratory Infections, Institut Pasteur, Paris	Mélnie Albert, Marion Barbet, Sylvie Behillil, Méline Bizard, Angela Brisebarre, Flora Donati Vincent Enouf, Maud Vanpeene, Sylvie van der Werf, Raulin Olivia
EPI_ISL_414628	hCoV-19/France/HF1805/2020	Europe / France / Hauts de France / Compiègne	2020-03-02	Centre Hospitalier Compiègne Laboratoire de Biologie	National Reference Center for Viruses of Respiratory Infections, Institut Pasteur, Paris	Mélnie Albert, Marion Barbet, Sylvie Behillil, Méline Bizard, Angela Brisebarre, Flora Donati Vincent Enouf, Maud Vanpeene, Sylvie van der Werf, Raulin Olivia
EPI_ISL_414629	hCoV-19/France/HF1870/2020	Europe / France / Hauts de France / Compiègne	2020-03-03	Centre Hospitalier Compiègne Laboratoire de Biologie	National Reference Center for Viruses of Respiratory Infections, Institut Pasteur, Paris	Mélnie Albert, Marion Barbet, Sylvie Behillil, Méline Bizard, Angela Brisebarre, Flora Donati Vincent Enouf, Maud Vanpeene, Sylvie van der Werf, Raulin Olivia
EPI_ISL_414630	hCoV-19/France/HF1871/2020	Europe / France / Hauts de France / Compiègne	2020-03-03	Centre Hospitalier Compiègne Laboratoire de Biologie	National Reference Center for Viruses of Respiratory Infections, Institut Pasteur, Paris	Mélnie Albert, Marion Barbet, Sylvie Behillil, Méline Bizard, Angela Brisebarre, Flora Donati Vincent Enouf, Maud Vanpeene, Sylvie van der Werf, Raulin Olivia
EPI_ISL_414631	hCoV-19/France/GE1973/2020	Europe / France / Grand-Est / Reims	2020-03-04	Hôpital Robert Debré Laboratoire de Virologie	National Reference Center for Viruses of Respiratory Infections, Institut Pasteur, Paris Mélnie Albert, Marion Barbet, Sylvie Behillil, Méline Bizard, Angela Brisebarre, Flora Donati Vincent Enouf, Maud Vanpeene, Sylvie van der Werf, Laurent Andreoletti
EPI_ISL_414632	hCoV-19/France/GE1977/2020	Europe / France / Grand-Est / Reims	2020-03-04	Hôpital Robert Debré Laboratoire de Virologie	National Reference Center for Viruses of Respiratory Infections, Institut Pasteur, Paris Mélnie Albert, Marion Barbet, Sylvie Behillil, Méline Bizard, Angela Brisebarre, Flora Donati Vincent Enouf, Maud Vanpeene, Sylvie van der Werf, Laurent Andreoletti
EPI_ISL_414633	hCoV-19/France/IDF1980/2020	Europe / France / Ile-de-France / Pontoise	2020-03-04	Centre Hospitalier René Dubois Laboratoire de Microbiologie - Bât A	National Reference Center for Viruses of Respiratory Infections, Institut Pasteur, Paris	Mélnie Albert, Marion Barbet, Sylvie Behillil, Méline Bizard, Angela Brisebarre, Flora Donati Vincent Enouf, Maud Vanpeene, Sylvie van der Werf, Pascale Martres
EPI_ISL_414634	hCoV-19/France/HF1986/2020	Europe / France / Hauts de France / Compiègne	2020-03-04

Centre Hospitalier Compiègne Laboratoire de Biologie	National Reference Center for Viruses of Respiratory Infections, Institut Pasteur, Paris	Mélnie Albert, Marion Barbet, Sylvie Behillil, Méline Bizard, Angela Brisebarre, Flora Donati Vincent Enouf, Maud Vanpeene, Sylvie van der Werf, Raulin Olivia
EPI_ISL_414635	hCoV-19/France/HF1988/2020	Europe / France / Hauts de France / Compiègne	2020-03-04	Centre Hospitalier Compiègne Laboratoire de Biologie	National Reference Center for Viruses of Respiratory Infections, Institut Pasteur, Paris	Mélnie Albert, Marion Barbet, Sylvie Behillil, Méline Bizard, Angela Brisebarre, Flora Donati Vincent Enouf, Maud Vanpeene, Sylvie van der Werf, Raulin Olivia
EPI_ISL_414663	hCoV-19/Guangzhou/GZMU0016/2020	Asia / China / Guangdong / Guangzhou	2020-02-25	State Key Laboratory of Respiratory Disease, National Clinical Research Center for Respiratory Disease, Guangzhou Institute of Respiratory Health, the First Affiliated Hospital of Guangzhou Medical University	The First Affiliated Hospital of Guangzhou Medical University & BGI-Shenzhen	Zhao et al
EPI_ISL_414686	hCoV-19/Guangzhou/GZMU0030/2020	Asia / China / Guangdong / Guangzhou	2020-02-27	State Key Laboratory of Respiratory Disease, National Clinical Research Center for Respiratory Disease, Guangzhou Institute of Respiratory Health, the First Affiliated Hospital of Guangzhou Medical University	The First Affiliated Hospital of Guangzhou Medical University & BGI-Shenzhen	Zhao et al
EPI_ISL_414687	hCoV-19/Guangzhou/GZMU0031/2020	Asia / China / Guangdong / Guangzhou	2020-02-25	State Key Laboratory of Respiratory Disease, National Clinical Research Center for Respiratory Disease, Guangzhou Institute of Respiratory Health, the First Affiliated Hospital of Guangzhou Medical University	the First Affiliated Hospital of Guangzhou Medical University & BGI-Shenzhen	Zhao et al
EPI_ISL_414689	hCoV-19/Guangzhou/GZMU0044/2020	Asia / China / Guangdong / Guangzhou	2020-02-25	State Key Laboratory of Respiratory Disease, National Clinical Research Center for Respiratory Disease, Guangzhou Institute of Respiratory Health, the First Affiliated Hospital of Guangzhou Medical University	The First Affiliated Hospital of Guangzhou Medical University & BGI-Shenzhen	Zhao et al
EPI_ISL_414690	hCoV-19/Guangzhou/GZMU0047/2020	Asia / China / Guangdong / Guangzhou	2020-02-25	State Key Laboratory of Respiratory Disease, National Clinical Research Center for Respiratory Disease, Guangzhou Institute of Respiratory Health, the First Affiliated Hospital of Guangzhou Medical University	The First Affiliated Hospital of Guangzhou Medical University & BGI-Shenzhen	Zhao et al
EPI_ISL_414691	hCoV-19/Guangzhou/GZMU0048/2020	Asia / China / Guangdong / Guangzhou	2020-02-25	State Key Laboratory of Respiratory Disease, National Clinical Research Center for Respiratory Disease, Guangzhou Institute of Respiratory Health, the First Affiliated Hospital of Guangzhou Medical University	The First Affiliated Hospital of Guangzhou Medical University & BGI-Shenzhen	Zhao et al
EPI_ISL_414692	hCoV-19/Guangzhou/GZMU0014/2020	Asia / China / Guangdong / Guangzhou	2020-02-25	State Key Laboratory of Respiratory Disease, National Clinical Research Center for Respiratory Disease, Guangzhou Institute of Respiratory Health, the First Affiliated Hospital of Guangzhou Medical University	The First Affiliated Hospital of Guangzhou Medical University & BGI-Shenzhen	Zhao et al
EPI_ISL_414936	hCoV-19/Shandong/LY003-2/2020	Asia / China / Shandong	2020-01-23	Shandong Provincial Center for Disease Control and Prevention	Beijing Institute of Microbiology and Epidemiology	Xiao-Lin Jiang, Xiao-Li Zhang, Xiang-Na Zhao, Cun-Bao Li, Jie Lei, Zeng-Qiang Kou, Wen-Kui Sun, Yang Hang, Feng Gao, Sheng-Xiang Ji, Can-Fang Lin, Bo Pang, Ming-Xiao Yao, Guo-Lin Wang, Lin Yao, Li-Jun Duan, Xiao Wei, Dian-Ming Kang, Mai-Juan Ma
EPI_ISL_414937	hCoV-19/Shandong/LY004-2/2020	Asia / China / Shandong	2020-01-26	Shandong Provincial Center for Disease Control and Prevention	Beijing Institute of Microbiology and Epidemiology	Xiao-Lin Jiang, Xiao-Li Zhang, Xiang-Na Zhao, Cun-Bao Li, Jie Lei, Zeng-Qiang Kou, Wen-Kui Sun, Yang Hang, Feng Gao, Sheng-Xiang Ji, Can-Fang Lin, Bo Pang, Ming-Xiao Yao, Guo-Lin Wang, Lin Yao, Li-Jun Duan, Xiao Wei, Dian-Ming Kang, Mai-Juan Ma
EPI_ISL_414938	hCoV-19/Shandong/LY005-2/2020	Asia / China / Shandong	2020-01-24	Shandong Provincial Center for Disease Control and Prevention	Beijing Institute of Microbiology and Epidemiology	Xiao-Lin Jiang, Xiao-Li Zhang, Xiang-Na Zhao, Cun-Bao Li, Jie Lei, Zeng-Qiang Kou, Wen-Kui Sun, Yang Hang, Feng Gao, Sheng-Xiang Ji, Can-Fang Lin, Bo Pang, Ming-Xiao Yao, Guo-Lin Wang, Lin Yao, Li-Jun Duan, Xiao Wei, Dian-Ming Kang, Mai-Juan Ma
EPI_ISL_414940	hCoV-19/Shandong/LY007-2/2020	Asia / China / Shandong	2020-01-25	Shandong Provincial Center for Disease Control and Prevention	Beijing Institute of Microbiology and Epidemiology	Xiao-Lin Jiang, Xiao-Li Zhang, Xiang-Na Zhao, Cun-Bao Li, Jie Lei, Zeng-Qiang Kou, Wen-Kui Sun, Yang Hang, Feng Gao, Sheng-Xiang Ji, Can-Fang Lin, Bo Pang, Ming-Xiao Yao, Guo-Lin Wang, Lin Yao, Li-Jun Duan, Xiao Wei, Dian-Ming Kang, Mai-Juan Ma
EPI_ISL_414941	hCoV-19/Shandong/LY008-2/2020	Asia / China / Shandong	2020-01-30	Shandong Provincial Center for Disease Control and Prevention	Beijing Institute of Microbiology and Epidemiology	Xiao-Lin Jiang, Xiao-Li Zhang, Xiang-Na Zhao, Cun-Bao Li, Jie Lei, Zeng-Qiang Kou, Wen-Kui Sun, Yang Hang, Feng Gao, Sheng-Xiang Ji, Can-Fang Lin, Bo Pang, Ming-Xiao Yao, Guo-Lin Wang, Lin Yao, Li-Jun Duan, Xiao Wei, Dian-Ming Kang, Mai-Juan Ma
EPI_ISL_415920	hCoV-19/Wales/PHW32/2020	Europe / United Kingdom / Wales	2020-03-12	Wales Specialist Virology Centre	Public Health Wales Microbiology Cardiff	Catherine Moore, Joanne Watkins, Sally Corden, Tom Connor
EPI_ISL_417200	hCoV-19/USA/WI-UW-06/2020	North America / USA / Wisconsin / Cottage Grove	2020-03-21	University of Wisconsin-Madison AIDS Vaccine Research Laboratories	University of Wisconsin-Madison AIDS Vaccine Research Laboratories	Gage Moreno, Katarina Braun, et al. AIDS Vaccine Research Laboratories
EPI_ISL_417201	hCoV-19/USA/WI-UW-07/2020	North America / USA / Wisconsin / Madison	2020-03-21	University of Wisconsin-Madison AIDS Vaccine Research Laboratories	University of Wisconsin-Madison AIDS Vaccine Research Laboratories	Gage Moreno, Katarina Braun, et al. AIDS Vaccine Research Laboratories
EPI_ISL_417202	hCoV-19/USA/WI-UW-08/2020	North America / USA / Wisconsin / Stoughton	2020-03-21

University of Wisconsin-Madison AIDS Vaccine Research Laboratories	University of Wisconsin-Madison AIDS Vaccine Research Laboratories	Gage Moreno, Katarina Braun, et al. AIDS Vaccine Research Laboratories
EPI_ISL_417203	hCoV-19/USA/WI-UW-09/2020	North America / USA / Wisconsin / Madison	2020-03-21	University of Wisconsin-Madison AIDS Vaccine Research Laboratories	University of Wisconsin-Madison AIDS Vaccine Research Laboratories	Gage Moreno, Katarina Braun, et al. AIDS Vaccine Research Laboratories
EPI_ISL_417204	hCoV-19/USA/WI-UW-10/2020	North America / USA / Wisconsin / Cambridge	2020-03-21	University of Wisconsin-Madison AIDS Vaccine Research Laboratories	University of Wisconsin-Madison AIDS Vaccine Research Laboratories	Gage Moreno, Katarina Braun, et al. AIDS Vaccine Research Laboratories
EPI_ISL_417211	hCoV-19/NewZealand/CoV001/2020	Oceania / New Zealand / Otago	2020-03-11	Dunedin Hospital	University of Otago	M.E. Quiñones-Mateu, B. Lawley, J. Grant, R. Harfoot, J. Ussher
EPI_ISL_417213	hCoV-19/England/20102068502/2020	Europe / United Kingdom / England	2020-03-01	Respiratory Virus Unit, Microbiology Services Colindale, Public Health England	Respiratory Virus Unit, Microbiology Services Colindale, Public Health England	Monica Galiano, Shahjahan Miah, Angie Lackenby, Omolola Akinbami, Tiina Talts, Leena Bhaw, Richard Myers, Steven Platt, Kirstin Edwards, Jonathan Hubb, Joanna Ellis, Maria Zambon
EPI_ISL_417217	hCoV-19/England/20102098802/2020	Europe / United Kingdom / England	2020-03-01	Respiratory Virus Unit, Microbiology Services Colindale, Public Health England	Respiratory Virus Unit, Microbiology Services Colindale, Public Health England	Monica Galiano, Shahjahan Miah, Angie Lackenby, Omolola Akinbami, Tiina Talts, Leena Bhaw, Richard Myers, Steven Platt, Kirstin Edwards, Jonathan Hubb, Joanna Ellis, Maria Zambon
EPI_ISL_417220	hCoV-19/England/20104002606/2020	Europe / United Kingdom / England	2020-03-03	Respiratory Virus Unit, Microbiology Services Colindale, Public Health England	Respiratory Virus Unit, Microbiology Services Colindale, Public Health England	Monica Galiano, Shahjahan Miah, Angie Lackenby, Omolola Akinbami, Tiina Talts, Leena Bhaw, Richard Myers, Steven Platt, Kirstin Edwards, Jonathan Hubb, Joanna Ellis, Maria Zambon
EPI_ISL_417222	hCoV-19/England/20104003002/2020	Europe / United Kingdom / England	2020-03-04	Respiratory Virus Unit, Microbiology Services Colindale, Public Health England	Respiratory Virus Unit, Microbiology Services Colindale, Public Health England	Monica Galiano, Shahjahan Miah, Angie Lackenby, Omolola Akinbami, Tiina Talts, Leena Bhaw, Richard Myers, Steven Platt, Kirstin Edwards, Jonathan Hubb, Joanna Ellis, Maria Zambon
EPI_ISL_417226	hCoV-19/England/20104004902/2020	Europe / United Kingdom / England	2020-03-02	Respiratory Virus Unit, Microbiology Services Colindale, Public Health England	Respiratory Virus Unit, Microbiology Services Colindale, Public Health England	Monica Galiano, Shahjahan Miah, Angie Lackenby, Omolola Akinbami, Tiina Talts, Leena Bhaw, Richard Myers, Steven Platt, Kirstin Edwards, Jonathan Hubb, Joanna Ellis, Maria Zambon
EPI_ISL_417227	hCoV-19/England/20104007503/2020	Europe / United Kingdom / England	2020-03-03	Respiratory Virus Unit, Microbiology Services Colindale, Public Health England	Respiratory Virus Unit, Microbiology Services Colindale, Public Health England	Monica Galiano, Shahjahan Miah, Angie Lackenby, Omolola Akinbami, Tiina Talts, Leena Bhaw, Richard Myers, Steven Platt, Kirstin Edwards, Jonathan Hubb, Joanna Ellis, Maria Zambon
EPI_ISL_417228	hCoV-19/England/20104008402/2020	Europe / United Kingdom / England	2020-03-03	Respiratory Virus Unit, Microbiology Services Colindale, Public Health England	Respiratory Virus Unit, Microbiology Services Colindale, Public Health England	Monica Galiano, Shahjahan Miah, Angie Lackenby, Omolola Akinbami, Tiina Talts, Leena Bhaw, Richard Myers, Steven Platt, Kirstin Edwards, Jonathan Hubb, Joanna Ellis, Maria Zambon
EPI_ISL_417230	hCoV-19/England/20104008702/2020	Europe / United Kingdom / England	2020-03-02	Respiratory Virus Unit, Microbiology Services Colindale, Public Health England	Respiratory Virus Unit, Microbiology Services Colindale, Public Health England	Monica Galiano, Shahjahan Miah, Angie Lackenby, Omolola Akinbami, Tiina Talts, Leena Bhaw, Richard Myers, Steven Platt, Kirstin Edwards, Jonathan Hubb, Joanna Ellis, Maria Zambon
EPI_ISL_417231	hCoV-19/England/20104008802/2020	Europe / United Kingdom / England	2020-03-03	Respiratory Virus Unit, Microbiology Services Colindale, Public Health England	Respiratory Virus Unit, Microbiology Services Colindale, Public Health England	Monica Galiano, Shahjahan Miah, Angie Lackenby, Omolola Akinbami, Tiina Talts, Leena Bhaw, Richard Myers, Steven Platt, Kirstin Edwards, Jonathan Hubb, Joanna Ellis, Maria Zambon
EPI_ISL_417232	hCoV-19/England/20104008902/2020	Europe / United Kingdom / England	2020-03-04	Respiratory Virus Unit, Microbiology Services Colindale, Public Health England	Respiratory Virus Unit, Microbiology Services Colindale, Public Health England	Monica Galiano, Shahjahan Miah, Angie Lackenby, Omolola Akinbami, Tiina Talts, Leena Bhaw, Richard Myers, Steven Platt, Kirstin Edwards, Jonathan Hubb, Joanna Ellis, Maria Zambon
EPI_ISL_417233	hCoV-19/England/20104009002/2020	Europe / United Kingdom / England	2020-03-02	Respiratory Virus Unit, Microbiology Services Colindale, Public Health England	Respiratory Virus Unit, Microbiology Services Colindale, Public Health England	Monica Galiano, Shahjahan Miah, Angie Lackenby, Omolola Akinbami, Tiina Talts, Leena Bhaw, Richard Myers, Steven Platt, Kirstin Edwards, Jonathan Hubb, Joanna Ellis, Maria Zambon
EPI_ISL_417234	hCoV-19/England/20104009102/2020	Europe / United Kingdom / England	2020-03-02	Respiratory Virus Unit, Microbiology Services Colindale, Public Health England	Respiratory Virus Unit, Microbiology Services Colindale, Public Health England	Monica Galiano, Shahjahan Miah, Angie Lackenby, Omolola Akinbami, Tiina Talts, Leena Bhaw, Richard Myers, Steven Platt, Kirstin Edwards, Jonathan Hubb, Joanna Ellis, Maria Zambon

EPI_ISL_417235 hCoV-19/England/20104013703/2020 Europe / United Kingdom / England 2020-03-02 Respiratory Virus Unit, Microbiology Services Colindale, Public Health England Respiratory Virus Unit, Microbiology Services Colindale, Public Health England Monica Galiano, Shahjahan Miah, Angie Lackenby, Omolola Akinbami, Tiina Talts, Leena Bhaw, Richard Myers, Steven Platt, Kirstin Edwards, Jonathan Hubb, Joanna Ellis, Maria Zambon
EPI_ISL_417236 hCoV-19/England/20104015302/2020 Europe / United Kingdom / England 2020-03-01 Respiratory Virus Unit, Microbiology Services Colindale, Public Health England Respiratory Virus Unit, Microbiology Services Colindale, Public Health England Monica Galiano, Shahjahan Miah, Angie Lackenby, Omolola Akinbami, Tiina Talts, Leena Bhaw, Richard Myers, Steven Platt, Kirstin Edwards, Jonathan Hubb, Joanna Ellis, Maria Zambon
EPI_ISL_417238 hCoV-19/England/20104035803/2020 Europe / United Kingdom / England 2020-03-03 Respiratory Virus Unit, Microbiology Services Colindale, Public Health England Respiratory Virus Unit, Microbiology Services Colindale, Public Health England Monica Galiano, Shahjahan Miah, Angie Lackenby, Omolola Akinbami, Tiina Talts, Leena Bhaw, Richard Myers, Steven Platt, Kirstin Edwards, Jonathan Hubb, Joanna Ellis, Maria Zambon
EPI_ISL_417239 hCoV-19/England/20106003303/2020 Europe / United Kingdom / England 2020-03-03 Respiratory Virus Unit, Microbiology Services Colindale, Public Health England Respiratory Virus Unit, Microbiology Services Colindale, Public Health England Monica Galiano, Shahjahan Miah, Angie Lackenby, Omolola Akinbami, Tiina Talts, Leena Bhaw, Richard Myers, Steven Platt, Kirstin Edwards, Jonathan Hubb, Joanna Ellis, Maria Zambon
EPI_ISL_417240 hCoV-19/England/20106004803/2020 Europe / United Kingdom / England 2020-03-03 Respiratory Virus Unit, Microbiology Services Colindale, Public Health England Respiratory Virus Unit, Microbiology Services Colindale, Public Health England Monica Galiano, Shahjahan Miah, Angie Lackenby, Omolola Akinbami, Tiina Talts, Leena Bhaw, Richard Myers, Steven Platt, Kirstin Edwards, Jonathan Hubb, Joanna Ellis, Maria Zambon
EPI_ISL_417244 hCoV-19/England/20106005403/2020 Europe / United Kingdom / England 2020-03-03 Respiratory Virus Unit, Microbiology Services Colindale, Public Health England Respiratory Virus Unit, Microbiology Services Colindale, Public Health England Monica Galiano, Shahjahan Miah, Angie Lackenby, Omolola Akinbami, Tiina Talts, Leena Bhaw, Richard Myers, Steven Platt, Kirstin Edwards, Jonathan Hubb, Joanna Ellis, Maria Zambon
EPI_ISL_417246 hCoV-19/England/20106145903/2020 Europe / United Kingdom / England 2020-03-03 Respiratory Virus Unit, Microbiology Services Colindale, Public Health England Respiratory Virus Unit, Microbiology Services Colindale, Public Health England Monica Galiano, Shahjahan Miah, Angie Lackenby, Omolola Akinbami, Tiina Talts, Leena Bhaw, Richard Myers, Steven Platt, Kirstin Edwards, Jonathan Hubb, Joanna Ellis, Maria Zambon
EPI_ISL_417248 hCoV-19/England/20108003302/2020 Europe / United Kingdom / England 2020-03-03 Respiratory Virus Unit, Microbiology Services Colindale, Public Health England Respiratory Virus Unit, Microbiology Services Colindale, Public Health England Monica Galiano, Shahjahan Miah, Angie Lackenby, Omolola Akinbami, Tiina Talts, Leena Bhaw, Richard Myers, Steven Platt, Kirstin Edwards, Jonathan Hubb, Joanna Ellis, Maria Zambon
EPI_ISL_418182 hCoV-19/Spain/Madrid_H8_37/2020 Europe / Spain / Madrid 2020-03-12 Hospital Universitario 12 de Octubre Hospital Universitario La Paz Elias Dahdouh, Sara González, Fernando Lázaro, Esther Viedma, Natalia Stella, Julio García, Juan Carlos Galán, Rafael Cantón, Mª Dolores Folgueira, Rafael Delgado, Jesús Mingorance
EPI_ISL_418183 hCoV-19/Hungary/mbl2/2020 Europe / Hungary / Baranya 2020-03-17 Virological Research Group, Szentágothai Research Centre Bioinformatics Research Group, Szentágothai Research Centre Péter Urbán, Endre Gábor Tóth, Gábor Kemenesi, Róbert Herczeg, Attila Gyenesei, Ferenc Jakab
EPI_ISL_418184 hCoV-19/USA/WI-GMF-00018/2020 North America / USA / Wisconsin / La Crosse County 2020-03-18 Gundersen Molecular Diagnostics Laboratory Kabara Cancer Research Institute Craig S. Richmond & Paraic A. Kenny
EPI_ISL_418186 hCoV-19/USA/WI-GMF-00227/2020 North America / USA / Wisconsin / La Crosse County 2020-03-23 Gundersen Molecular Diagnostic Laboratory Kabara Cancer Research Institute Craig S. Richmond & Paraic A. Kenny
EPI_ISL_418187 hCoV-19/USA/WI-GMF-00228/2020 North America / USA / Wisconsin / La Crosse County 2020-03-23 Gundersen Molecular Diagnostics Laboratory Kabara Cancer Research Institute Craig S. Richmond & Paraic A. Kenny
EPI_ISL_418188 hCoV-19/USA/WI-GMF-00232/2020 North America / USA / Wisconsin / Monroe County 2020-03-23 Gundersen Molecular Diagnostics Laboratory Kabara Cancer Research Institute Craig S. Richmond & Paraic A. Kenny
EPI_ISL_418192 hCoV-19/USA/NY-NYUMC7/2020 North America / USA / New York / Manhattan 2020-03-16 NYU Langone Health Department of Pathology and Medicine, New York University School of Medicine Margaret Black, John Cadley, Paolo Cotzia, John Chen, Dacia Dimartino, Xiaojun Feng, Adriana Heguy, Megan Hogan, Emily Huang, George Jour, Christian Marier, Matthew T. Maurano, Mark J. Mulligan, Peter Meyn, Jared Pinnell, Amy Rapkiewicz, Marie Samanovic-Golden, Antonio Serrano, Guomiao Shen, Matija Snuderl, Nick Vulpescu, Gael Westby, Paul Zappile
EPI_ISL_418193 hCoV-19/USA/NY-NYUMC8/2020 North America / USA / New York / Manhattan 2020-03-17 NYU Langone Health Department of Pathology and Medicine, New York University School of Medicine Margaret Black, John Cadley, Paolo Cotzia, John Chen, Dacia Dimartino, Xiaojun Feng, Adriana Heguy, Megan Hogan, Emily Huang, George Jour, Christian Marier, Matthew T. Maurano, Mark J. Mulligan, Peter Meyn, Jared Pinnell, Amy Rapkiewicz, Marie Samanovic-Golden, Antonio Serrano, Guomiao Shen, Matija Snuderl, Nick Vulpescu,

Gael Westby, Paul Zappile
EPI_ISL_418196 hCoV-19/USA/NY-NYUMC11/2020 North America / USA / New York / Manhattan 2020-03-14 NYU Langone Health Department of Pathology and Medicine, New York University School of Medicine Margaret Black, John Cadley, Paolo Cotzia, John Chen, Dacia Dimartino, Xiaojun Feng, Adriana Heguy, Megan Hogan, Emily Huang, George Jour, Christian Marier, Matthew T. Maurano, Mark J. Mulligan, Peter Meyn, Jared Pinnell, Amy Rapkiewicz, Marie Samanovic-Golden, Antonio Serrano, Guomiao Shen, Matija Snuderl, Nick Vulpescu, Gael Westby, Paul Zappile
EPI_ISL_418197 hCoV-19/USA/NY-NYUMC12/2020 North America / USA / New York / Manhattan 2020-03-15 NYU Langone Health Department of Pathology and Medicine, New York University School of Medicine Margaret Black, John Cadley, Paolo Cotzia, John Chen, Dacia Dimartino, Xiaojun Feng, Adriana Heguy, Megan Hogan, Emily Huang, George Jour, Christian Marier, Matthew T. Maurano, Mark J. Mulligan, Peter Meyn, Jared Pinnell, Amy Rapkiewicz, Marie Samanovic-Golden, Antonio Serrano, Guomiao Shen, Matija Snuderl, Nick Vulpescu, Gael Westby, Paul Zappile
EPI_ISL_418198 hCoV-19/USA/NY-NYUMC13/2020 North America / USA / New York / Manhattan 2020-03-17 NYU Langone Health Department of Pathology and Medicine, New York University School of Medicine Margaret Black, John Cadley, Paolo Cotzia, John Chen, Dacia Dimartino, Xiaojun Feng, Adriana Heguy, Megan Hogan, Emily Huang, George Jour, Christian Marier, Matthew T. Maurano, Mark J. Mulligan, Peter Meyn, Jared Pinnell, Amy Rapkiewicz, Marie Samanovic-Golden, Antonio Serrano, Guomiao Shen, Matija Snuderl, Nick Vulpescu, Gael Westby, Paul Zappile
EPI_ISL_418199 hCoV-19/USA/NY-NYUMC14/2020 North America / USA / New York / Manhattan 2020-03-17 NYU Langone Health Department of Pathology and Medicine, New York University School of Medicine Margaret Black, John Cadley, Paolo Cotzia, John Chen, Dacia Dimartino, Xiaojun Feng, Adriana Heguy, Megan Hogan, Emily Huang, George Jour, Christian Marier, Matthew T. Maurano, Mark J. Mulligan, Peter Meyn, Jared Pinnell, Amy Rapkiewicz, Marie Samanovic-Golden, Antonio Serrano, Guomiao Shen, Matija Snuderl, Nick Vulpescu, Gael Westby, Paul Zappile
EPI_ISL_418200 hCoV-19/USA/NY-NYUMC15/2020 North America / USA / New York / Manhattan 2020-03-17 NYU Langone Health Department of Pathology and Medicine, New York University School of Medicine Margaret Black, John Cadley, Paolo Cotzia, John Chen, Dacia Dimartino, Xiaojun Feng, Adriana Heguy, Megan Hogan, Emily Huang, George Jour, Christian Marier, Matthew T. Maurano, Mark J. Mulligan, Peter Meyn, Jared Pinnell, Amy Rapkiewicz, Marie Samanovic-Golden, Antonio Serrano, Guomiao Shen, Matija Snuderl, Nick Vulpescu, Gael Westby, Paul Zappile
EPI_ISL_418203 hCoV-19/USA/NY-NYUMC18/2020 North America / USA / New York / Manhattan 2020-03-17 NYU Langone Health Department of Pathology and Medicine, New York University School of Medicine Margaret Black, John Cadley, Paolo Cotzia, John Chen, Dacia Dimartino, Xiaojun Feng, Adriana Heguy, Megan Hogan, Emily Huang, George Jour, Christian Marier, Matthew T. Maurano, Mark J. Mulligan, Peter Meyn, Jared Pinnell, Amy Rapkiewicz, Marie Samanovic-Golden, Antonio Serrano, Guomiao Shen, Matija Snuderl, Nick Vulpescu, Gael Westby, Paul Zappile
EPI_ISL_418204 hCoV-19/USA/NY-NYUMC19/2020 North America / USA / New York / Manhattan 2020-03-17 NYU Langone Health Department of Pathology and Medicine, New York University School of Medicine Margaret Black, John Cadley, Paolo Cotzia, John Chen, Dacia Dimartino, Xiaojun Feng, Adriana Heguy, Megan Hogan, Emily Huang, George Jour, Christian Marier, Matthew T. Maurano, Mark J. Mulligan, Peter Meyn, Jared Pinnell, Amy Rapkiewicz, Marie Samanovic-Golden, Antonio Serrano, Guomiao Shen, Matija Snuderl, Nick Vulpescu, Gael Westby, Paul Zappile
EPI_ISL_418206 hCoV-19/Senegal/003/2020 Africa / Senegal / Dakar 2020-02-28 Institut Pasteur Dakar Institut Pasteur de Dakar Ndongo Dia, Ousmane Faye, Amadou Alpha Sall
EPI_ISL_418207 hCoV-19/Senegal/016/2020 Africa / Senegal / Dakar 2020-03-02 Institut Pasteur Dakar Institut Pasteur de Dakar Ndongo Dia, Ousmane Faye, Amadou Alpha Sall
EPI_ISL_418208 hCoV-19/Senegal/020/2020 Africa / Senegal / Dakar 2020-03-04 Institut Pasteur Dakar Institut Pasteur de Dakar Ndongo Dia, Ousmane Faye, Amadou Alpha Sall
EPI_ISL_418209 hCoV-19/Senegal/026/2020 Africa / Senegal / Dakar 2020-03-03 Institut Pasteur Dakar Institut Pasteur de Dakar Ndongo Dia, Ousmane Faye, Amadou Alpha Sall
EPI_ISL_418210 hCoV-19/Senegal/073/2020 Africa / Senegal / Touba 2020-03-10 Institut Pasteur Dakar Institut Pasteur de Dakar Ndongo Dia, Ousmane Faye, Amadou Alpha Sall
EPI_ISL_418211 hCoV-19/Senegal/082/2020 Africa / Senegal / Touba 2020-03-11 Institut Pasteur Dakar Institut Pasteur de Dakar Ndongo Dia, Ousmane Faye, Amadou Alpha Sall
EPI_ISL_418212 hCoV-19/Senegal/087/2020 Africa / Senegal / Touba 2020-03-11 Institut Pasteur Dakar Institut Pasteur de Dakar Ndongo Dia, Ousmane Faye, Amadou Alpha sall
EPI_ISL_418213 hCoV-19/Senegal/094/2020 Africa / Senegal / Touba 2020-03-12 Institut Pasteur Dakar Institut Pasteur de Dakar Ndongo Dia, Ousmane Faye, Amadou Alpha Sall
EPI_ISL_418216 hCoV-19/Senegal/136/2020 Africa / Senegal / Dakar 2020-03-13 Institut Pasteur Dakar Institut Pasteur de Dakar Ndongo Dia, Ousmane Faye, Amadou Alpha Sall
EPI_ISL_418217 hCoV-19/Senegal/139/2020 Africa / Senegal / Mbour 2020-03-13 Institut Pasteur Dakar Institut Pasteur de Dakar Ndongo Dia, Ousmane Faye, Amadou Alpha Sall
EPI_ISL_418218 hCoV-19/France/HF1465/2020 Europe / France / Hauts de France / Compiègne 2020-02-21 Centre Hospitalier Compiègne Laboratoire de Biologie National Reference Center for Viruses of Respiratory Infections, Institut Pasteur, Paris Mélanie Albert, Marion Barbet, Sylvie Behillil, Méline Bizard, Angela Brisebarre, Flora Donati, Fabiana Gambaro, Etienne Simon-Lorière, Vincent Enouf, Maud Vanpeene, Sylvie van der Werf, Raulin Olivia
EPI_ISL_418219 hCoV-19/France/B1623/2020 Europe / France / Bretagne / Brest 2020-02-26 CHU - Hôpital Cavale Blanche - Labo. de Virologie National Reference Center for Viruses of Respiratory

Infections, Institut Pasteur, Paris Mélanie Albert, Marion Barbet, Sylvie Behillil, Méline Bizard, Angela Brisebarre, Flora Donati, Fabiana Gambaro, Etienne Simon-Lorière, Vincent Enouf, Maud Vanpeene, Sylvie van der Werf, Léa Pilorge
EPI_ISL_418220 hCoV-19/France/HF1645/2020 Europe / France / Hauts de France / Compiègne 2020-02-28 Centre Hospitalier Compiègne Laboratoire de Biologie National Reference Center for Viruses of Respiratory Infections, Institut Pasteur, Paris Mélanie Albert, Marion Barbet, Sylvie Behillil, Méline Bizard, Angela Brisebarre, Flora Donati, Fabiana Gambaro, Etienne Simon-Lorière, Vincent Enouf, Maud Vanpeene, Sylvie van der Werf, Raulin Olivia
EPI_ISL_418222 hCoV-19/France/CVL2000/2020 Europe / France / Centre-Val de Loire / Tours 2020-03-04 CHRU Bretonneau - Serv. Bacterio-Virol. National Reference Center for Viruses of Respiratory Infections, Institut Pasteur, Paris Mélanie Albert, Marion Barbet, Sylvie Behillil, Méline Bizard, Angela Brisebarre, Flora Donati, Fabiana Gambaro, Etienne Simon-Lorière, Vincent Enouf, Maud Vanpeene, Sylvie van der Werf, Julien Marlet
EPI_ISL_418223 hCoV-19/France/HF2060/2020 Europe / France / Hauts de France / Compiègne 2020-03-05 Centre Hospitalier Compiègne Laboratoire de Biologie National Reference Center for Viruses of Respiratory Infections, Institut Pasteur, Paris Mélanie Albert, Marion Barbet, Sylvie Behillil, Méline Bizard, Angela Brisebarre, Flora Donati, Fabiana Gambaro, Etienne Simon-Lorière, Vincent Enouf, Maud Vanpeene, Sylvie van der Werf, Raulin Olivia
EPI_ISL_418224 hCoV-19/France/HF2150/2020 Europe / France / Hauts de France / Compiègne 2020-03-08 Centre Hospitalier Compiègne Laboratoire de Biologie National Reference Center for Viruses of Respiratory Infections, Institut Pasteur, Paris Mélanie Albert, Marion Barbet, Sylvie Behillil, Méline Bizard, Angela Brisebarre, Flora Donati, Fabiana Gambaro, Etienne Simon-Lorière, Vincent Enouf, Maud Vanpeene, Sylvie van der Werf, Raulin Olivia
EPI_ISL_418225 hCoV-19/France/HF2155/2020 Europe / France / Hauts de France / Compiègne 2020-03-08 Centre Hospitalier Compiègne Laboratoire de Biologie National Reference Center for Viruses of Respiratory Infections, Institut Pasteur, Paris Mélanie Albert, Marion Barbet, Sylvie Behillil, Méline Bizard, Angela Brisebarre, Flora Donati, Fabiana Gambaro, Etienne Simon-Lorière, Vincent Enouf, Maud Vanpeene, Sylvie van der Werf, Raulin Olivia
EPI_ISL_418226 hCoV-19/France/HF2381/2020 Europe / France / Hauts de France / Crouy en Thelle 2020-03-09 EHPAD - Résidences les Cèdres National Reference Center for Viruses of Respiratory Infections, Institut Pasteur, Paris Mélanie Albert, Marion Barbet, Sylvie Behillil, Méline Bizard, Angela Brisebarre, Flora Donati, Etienne Simon-Lorière, Vincent Enouf, Maud Vanpeene, Sylvie van der Werf
EPI_ISL_418227 hCoV-19/France/HF2393/2020 Europe / France / Hauts de France / Compiègne 2020-03-12 Centre Hospitalier Compiègne Laboratoire de Biologie National Reference Center for Viruses of Respiratory Infections, Institut Pasteur, Paris Mélanie Albert, Marion Barbet, Sylvie Behillil, Méline Bizard, Angela Brisebarre, Flora Donati, Etienne Simon-Lorière, Vincent Enouf, Maud Vanpeene, Sylvie van der Werf, Raulin Olivia
EPI_ISL_418228 hCoV-19/France/HF2405/2020 Europe / France / Hauts de France / Compiègne 2020-03-12 Centre Hospitalier Compiègne Laboratoire de Biologie National Reference Center for Viruses of Respiratory Infections, Institut Pasteur, Paris Mélanie Albert, Marion Barbet, Sylvie Behillil, Méline Bizard, Angela Brisebarre, Flora Donati, Etienne Simon-Lorière, Vincent Enouf, Maud Vanpeene, Sylvie van der Werf, Raulin Olivia
EPI_ISL_418229 hCoV-19/France/IDF2410/2020 Europe / France / Ile de France / Levallois-Perret 2020-03-12 Hopital franco britannique - Laboratoire National Reference Center for Viruses of Respiratory Infections, Institut Pasteur, Paris Mélanie Albert, Marion Barbet, Sylvie Behillil, Méline Bizard, Angela Brisebarre, Flora Donati, Etienne Simon-Lorière, Vincent Enouf, Maud Vanpeene, Sylvie van der Werf, Marianne Asso Bonnet
EPI_ISL_418230 hCoV-19/France/IDF2420/2020 Europe / France / Ile de France / Paris 2020-03-13 Clinique AVERAY LA BROUSTE, Med. Polyvalente National Reference Center for Viruses of Respiratory Infections, Institut Pasteur, Paris Mélanie Albert, Marion Barbet, Sylvie Behillil, Méline Bizard, Angela Brisebarre, Flora Donati, Etienne Simon-Lorière, Vincent Enouf, Maud Vanpeene, Sylvie van der Werf, Elsa Ngwem
EPI_ISL_418231 hCoV-19/France/HF2496/2020 Europe / France / Hauts de France / Compiègne 2020-03-15 Centre Hospitalier Compiègne Laboratoire de Biologie National Reference Center for Viruses of Respiratory Infections, Institut Pasteur, Paris Mélanie Albert, Marion Barbet, Sylvie Behillil, Méline Bizard, Angela Brisebarre, Flora Donati, Etienne Simon-Lorière, Vincent Enouf, Maud Vanpeene, Sylvie van der Werf, Raulin Olivia
EPI_ISL_418232 hCoV-19/France/IDF2532/2020 Europe / France / Ile de France / Meudon la Forêt 2020-03-15 Service des Urgences National Reference Center for Viruses of Respiratory Infections, Institut Pasteur, Paris Mélanie Albert, Marion Barbet, Sylvie Behillil, Méline Bizard, Angela Brisebarre, Flora Donati, Etienne Simon-Lorière, Vincent Enouf, Maud Vanpeene, Sylvie van der Werf, Boubkeur
EPI_ISL_418233 hCoV-19/France/IDF2533/2020 Europe / France / Ile de France / Meudon la Forêt 2020-03-15 Service des Urgences National Reference Center for Viruses of Respiratory Infections, Institut Pasteur, Paris Mélanie Albert, Marion Barbet, Sylvie Behillil, Méline Bizard, Angela Brisebarre, Flora Donati, Etienne Simon-Lorière, Vincent Enouf, Maud Vanpeene, Sylvie van der Werf, Boubkeur
EPI_ISL_418234 hCoV-19/France/IDF2534/2020 Europe / France / Ile de France / Longjumeau 2020-03-14 LABM GH nord Essonne National Reference Center for Viruses of Respiratory Infections, Institut Pasteur, Paris Mélanie Albert, Marion Barbet, Sylvie Behillil, Méline Bizard, Angela Brisebarre, Flora Donati, Etienne Simon-Lorière, Vincent Enouf, Maud Vanpeene, Sylvie van der Werf, Christine Lambert
EPI_ISL_418235 hCoV-19/France/IDF2561/2020 Europe / France / Ile de France / Vanves 2020-03-16 Cabinet médical National Reference Center for Viruses of Respiratory Infections, Institut Pasteur, Paris

Mélanie Albert, Marion Barbet, Sylvie Behillil, Méline Bizard, Angela Brisebarre, Flora Donati, Etienne Simon-Lorière, Vincent Enouf, Maud Vanpeene, Sylvie van der Werf
EPI_ISL_418236 hCoV-19/France/HF2586/2020 Europe / France / Hauts de France / Compiègne 2020-03-16 Centre Hospitalier Compiègne Laboratoire de Biologie National Reference Center for Viruses of Respiratory Infections, Institut Pasteur, Paris Mélanie Albert, Marion Barbet, Sylvie Behillil, Méline Bizard, Angela Brisebarre, Flora Donati, Etienne Simon-Lorière, Vincent Enouf, Maud Vanpeene, Sylvie van der Werf, Raulin Olivia
EPI_ISL_418237 hCoV-19/France/HF2595/2020 Europe / France / Hauts de France / Compiègne 2020-03-16 Centre Hospitalier Compiègne Laboratoire de Biologie National Reference Center for Viruses of Respiratory Infections, Institut Pasteur, Paris Mélanie Albert, Marion Barbet, Sylvie Behillil, Méline Bizard, Angela Brisebarre, Flora Donati, Etienne Simon-Lorière, Vincent Enouf, Maud Vanpeene, Sylvie van der Werf, Raulin Olivia
EPI_ISL_418238 hCoV-19/France/HF2597/2020 Europe / France / Hauts de France / Compiègne 2020-03-16 Centre Hospitalier Compiègne Laboratoire de Biologie National Reference Center for Viruses of Respiratory Infections, Institut Pasteur, Paris Mélanie Albert, Marion Barbet, Sylvie Behillil, Méline Bizard, Angela Brisebarre, Flora Donati, Etienne Simon-Lorière, Vincent Enouf, Maud Vanpeene, Sylvie van der Werf, Raulin Olivia
EPI_ISL_418239 hCoV-19/France/HF2601/2020 Europe / France / Hauts de France / Compiègne 2020-03-16 Centre Hospitalier Compiègne Laboratoire de Biologie National Reference Center for Viruses of Respiratory Infections, Institut Pasteur, Paris Mélanie Albert, Marion Barbet, Sylvie Behillil, Méline Bizard, Angela Brisebarre, Flora Donati, Etienne Simon-Lorière, Vincent Enouf, Maud Vanpeene, Sylvie van der Werf, Raulin Olivia
EPI_ISL_418240 hCoV-19/France/IDF2684/2020 Europe / France / Ile de France / Longjumeau 2020-03-16 LABM GH nord Essonne National Reference Center for Viruses of Respiratory Infections, Institut Pasteur, Paris Mélanie Albert, Marion Barbet, Sylvie Behillil, Méline Bizard, Angela Brisebarre, Flora Donati, Etienne Simon-Lorière, Vincent Enouf, Maud Vanpeene, Sylvie van der Werf, Christine Lambert
EPI_ISL_418241 hCoV-19/Algeria/G0638_2264/2020 Africa / Algeria / Boufarik 2020-03-02 NIC Viral Respiratory Unit - Institut Pasteur of Algeria National Reference Center for Viruses of Respiratory Infections, Institut Pasteur, Paris Mélanie Albert, Marion Barbet, Sylvie Behillil, Méline Bizard, Angela Brisebarre, Flora Donati, Etienne Simon-Lorière, Vincent Enouf, Maud Vanpeene, Sylvie van der Werf, Fawzi Derrar
EPI_ISL_418242 hCoV-19/Algeria/G0640_2265/2020 Africa / Algeria / Blida 2020-03-08 NIC Viral Respiratory Unit - Institut Pasteur of Algeria National Reference Center for Viruses of Respiratory Infections, Institut Pasteur, Paris Mélanie Albert, Marion Barbet, Sylvie Behillil, Méline Bizard, Angela Brisebarre, Flora Donati, Etienne Simon-Lorière, Vincent Enouf, Maud Vanpeene, Sylvie van der Werf, Fawzi Derrar
EPI_ISL_418243 hCoV-19/Spain/Andalucia201272/2020 Europe / Spain / Andalusia 2020-02-28 HOSPITAL UNIVERSITARIO VIRGEN DE LAS NIEVES Instituto de Salud Carlos III Iglesias-Caballero, M. Molinero Calamita, M. González-Esguevillas, M. Camarero, S. Pozo, F. Casas, I. Jiménez, P. Jiménez, M. Zaballos, A. Monzón, S. Varona, S. Juliá, M. Cuesta, I. Sanbonmatsu S.
EPI_ISL_418244 hCoV-19/Spain/Andalucia201373/2020 Europe / Spain / Andalusia 2020-03-02 HOSPITAL UNIVERSITARIO VIRGEN DE LAS NIEVES Instituto de Salud Carlos III Iglesias-Caballero, M. Molinero Calamita, M. González-Esguevillas, M. Camarero, S. Pozo, F. Casas, I. Jiménez, P. Jiménez, M. Zaballos, A. Monzón, S. Varona, S. Juliá, M. Cuesta, I. Sanbonmatsu S.
EPI_ISL_418245 hCoV-19/Spain/CastillaLaMancha201328/2020 Europe / Spain / Castilla La Mancha 2020-03-01 Hospital General y Universitario de Guadalajara Instituto de Salud Carlos III Iglesias-Caballero, M. Molinero Calamita, M. González-Esguevillas, M. Camarero, S. Pozo, F. Casas, I. Jiménez, P. Jiménez, M. Zaballos, A. Monzón, S. Varona, S. Juliá, M. Cuesta, I. Gonzalez-Praetorius A.
EPI_ISL_418246 hCoV-19/Spain/CastillaLaMancha201329/2020 Europe / Spain / Castilla-La Mancha 2020-03-01 Hospital General y Universitario de Guadalajara Instituto de Salud Carlos III Iglesias-Caballero, M. Molinero Calamita, M. González-Esguevillas, M. Camarero, S. Pozo, F. Casas, I. Jiménez, P. Jiménez, M. Zaballos, A. Monzón, S. Varona, S. Juliá, M. Cuesta, I. Gonzalez-Praetorius A.
EPI_ISL_418247 hCoV-19/Spain/CastillayLeon201061/2020 Europe / Spain / Castilla y Leon 2020-02-26 HOSPITAL GENERAL DE SEGOVIA Instituto de Salud Carlos III Iglesias-Caballero, M. Molinero Calamita, M. González-Esguevillas, M. Camarero, S. Pozo, F. Casas, I. Jiménez, P. Jiménez, M. Zaballos, A. Monzón, S. Varona, S. Juliá, M. Cuesta, I. Hernando-Real S.
EPI_ISL_418248 hCoV-19/Spain/CastillayLeon201323/2020 Europe / Spain / Castilla y Leon 2020-03-01 COMPLEJO ASISTENCIAL UNIVERSITARIO DE BURGOS Instituto de Salud Carlos III Iglesias-Caballero, M. Molinero Calamita, M. González-Esguevillas, M. Camarero, S. Pozo, F. Casas, I. Jiménez, P. Jiménez, M. Zaballos, A. Monzón, S. Varona, S. Juliá, M. Cuesta, I. Megias-Lobon G.
EPI_ISL_418249 hCoV-19/Spain/CastillayLeon201372/2020 Europe / Spain / Castilla y Leon 2020-03-03 COMPLEJO ASISTENCIAL UNIVERSITARIO DE BURGOS Instituto de Salud Carlos III Iglesias-Caballero, M. Molinero Calamita, M. González-Esguevillas, M. Camarero, S. Pozo, F. Casas, I. Jiménez, P. Jiménez, M. Zaballos, A. Monzón, S. Varona, S. Juliá, M. Cuesta, I. Megias-Lobon G.
EPI_ISL_418250 hCoV-19/Spain/Cataluna201396/2020 Europe / Spain / Catalonia 2020 HOSPITAL CLINIC Instituto de Salud Carlos III Iglesias-Caballero, M. Molinero Calamita, M. González-Esguevillas, M. Camarero, S. Pozo, F. Casas, I. Jiménez, P. Jiménez, M. Zaballos, A. Monzón, S. Varona, S. Juliá, M. Cuesta, I. Marcos M.A
EPI_ISL_418251 hCoV-19/Spain/Madrid201105/2020 Europe / Spain / Madrid 2020-02-25 HOSPITAL UNIVERSITARIO LA PAZ Instituto de Salud Carlos III Iglesias-Caballero, M. Molinero Calamita, M. González-Esguevillas, M.

Camarero, S. Pozo, F. Casas, I. Jiménez, P. Jiménez, M. Zaballos, A. Monzón, S. Varona, S. Juliá, M. Cuesta, I. Romero P.
EPI_ISL_418252 hCoV-19/Spain/Madrid201449/2020 Europe / Spain / Madrid 2020-03-04 FUNDACION JIMENEZ DIAZ Instituto de Salud Carlos III Iglesias-Caballero, M. Molinero Calamita, M. González-Esguevillas, M. Camarero, S. Pozo, F. Casas, I. Jiménez, P. Jiménez, M. Zaballos, A. Monzón, S. Varona, S. Juliá, M. Cuesta, I. Fernández Roblas, R.
EPI_ISL_418253 hCoV-19/Spain/PaisVasco201382/2020 Europe / Spain / Basque Country 2020-03-02 HOSPITAL TXAGORRITXU Instituto de Salud Carlos III Iglesias-Caballero, M. Molinero Calamita, M. González-Esguevillas, M. Camarero, S. Pozo, F. Casas, I. Jiménez, P. Jiménez, M. Zaballos, A. Monzón, S. Varona, S. Juliá, M. Cuesta, I. Gomez-Gonzalez C.
EPI_ISL_418255 hCoV-19/Italy/TE4925/2020 Europe / Italy / Abruzzo 2020-03-14 "Presidio Ospedaliero ""S. Spirito"" - PESCARA" "Istituto Zooprofilattico Sperimentale dell'Abruzzo e Molise ""G. Caporale""" Lorusso A, Marcacci M, Cammà C, Monaco F, Puglia I, Di Pasquale A, Rinaldi A, Mangone I, Savini G
EPI_ISL_418256 hCoV-19/Italy/TE4880/2020 Europe / Italy / Abruzzo 2020-03-14 Ospedale “San Liberatore” di Atri "Istituto Zooprofilattico Sperimentale dell'Abruzzo e Molise ""G. Caporale""" Lorusso A, Marcacci M, Di Domenico M, Puglia I, Curini V, Ancora M, Di Pasquale A, Rinaldi A, Mangone I, Cammà C, Savini G.
EPI_ISL_418257 hCoV-19/Italy/TE5056/2020 Europe / Italy / Abruzzo 2020-03-17 Ospedale Civile Giuseppe Mazzini, Teramo "Istituto Zooprofilattico Sperimentale dell'Abruzzo e Molise ""G.Caporale""" Lorusso A, Marcacci M, Di Domenico M, Puglia I, Curini V, Ancora M, Di Pasquale A, Rinaldi A, Mangone I, Cammà C, Savini G.
EPI_ISL_418258 hCoV-19/Italy/TE4953/2020 Europe / Italy / Abruzzo 2020-03-14 "Presidio ospedaliero ""Santo Spirito""" "Istituto Zooprofilattico Sperimentale dell'Abruzzo e Molise ""G. Caporale""" Lorusso A, Marcacci M, Di Domenico M, Puglia I, Curini V, Ancora M, Di Pasquale A, Rinaldi A, Mangone I, Cammà C, Savini G.
EPI_ISL_418259 hCoV-19/Italy/TE4959/2020 Europe / Italy / Abruzzo 2020-03-14 "Presidio ospedaliero ""Santo Spirito""" "Istituto Zooprofilattico Sperimentale dell'Abruzzo e Molise ""G. Caporale""" Lorusso A, Marcacci M, Di Domenico M, Puglia I, Curini V, Ancora M, Di Pasquale A, Rinaldi A, Mangone I, Cammà C, Savini G.
EPI_ISL_418260 hCoV-19/Italy/TE4836/2020 Europe / Italy / Abruzzo 2020-03-16 Ospedale Civile Giuseppe Mazzini "Istituto Zooprofilattico Sperimentale dell'Abruzzo e Molise ""G. Caporale""" Lorusso A, Marcacci M, Di Domenico M, Puglia I, Curini V, Ancora M, Di Pasquale A, Rinaldi A, Mangone I, Cammà C, Savini G.
EPI_ISL_418263 hCoV-19/Greece/10/2020 Europe / Greece / Athens 2020-03-18 Laboratory of Microbiology, Department of Medicine, National and Kapodistrian University of Athens, Greece Laboratory of Biology, Department of Medicine, Democritus University of Thrace, Greece Maria Bampali, Elisavet Gatzidou, Nikolaos Dovrolis, Stavroula Veletza, Nikolaos Spanakis, Ioannis Karakasiliotis
EPI_ISL_418264 hCoV-19/Greece/12/2020 Europe / Greece / Athens 2020-03-18 Laboratory of Microbiology, Department of Medicine, National and Kapodistrian University of Athens, Greece Laboratory of Biology, Department of Medicine, Democritus University of Thrace, Greece Maria Bampali, Elisavet Gatzidou, Nikolaos Dovrolis, Stavroula Veletza, Nikolaos Spanakis, Ioannis Karakasiliotis
EPI_ISL_418265 hCoV-19/Greece/16/2020 Europe / Greece / Athens 2020-03-18 Laboratory of Microbiology, Department of Medicine, National and Kapodistrian University of Athens, Greece Laboratory of Biology, Department of Medicine, Democritus University of Thrace, Greece Maria Bampali, Elisavet Gatzidou, Nikolaos Dovrolis, Stavroula Veletza, Nikolaos Spanakis, Ioannis Karakasiliotis
EPI_ISL_418267 hCoV-19/Vietnam/19-02S/2020 Asia / Vietnam / Ho Chi Minh City 2020-01-22 unknown Microbiology and Immunology department Nguyen,H.T., Cao,T.M., Pham,H.T.T., Vu,N.P.H., Dao,M.H., Huynh,L.T.K., Nguyen,L.T., Nguyen,N.T., Nguyen,T.T.N., Nguyen,A.H., Luong,Q.C., Nguyen,T.V., Tran,K.C., Pham,Q.D., Tran,T., Hoang,C.Q., Nguyen,T.T., Le,H.Q., Phung,T.M., Vo,T.N.A., Nguyen,S.N., Pham,D.T., Nguyen,T.V. and Phan,L.T.
EPI_ISL_418269 hCoV-19/Vietnam/19-01S/2020 Asia / Vietnam / Ho Chi Minh City 2020-01-22 unknown Microbiology and Immunology department Cao,T.M., Nguyen,H.T., Pham,H.T.T., Vu,N.P.H., Dao,M.H., Huynh,L.T.K., Nguyen,L.T., Nguyen,N.T., Nguyen,T.T.N., Nguyen,A.H., Luong,Q.C., Nguyen,T.V., Tran,K.C., Pham,Q.D., Tran,T., Hoang,C.Q., Nguyen,T.T., Le,H.Q., Phung,T.M., Vo,T.N.A., Nguyen,S.N., Pham,D.T., Phan,L.T. and Nguyen,T.V.
EPI_ISL_418270 hCoV-19/Belgium/JL-03044/2020 Europe / Belgium / Antwerp 2020-03-04 KU Leuven, Clinical and Epidemiological Virology KU Leuven, Clinical and Epidemiological Virology Tony Wawina, Joan Marti-Carreras, Bert Vanmechelen, Piet Maes
EPI_ISL_418294 hCoV-19/England/SHEF-BFDFA/2020 Europe / United Kingdom / England / South Yorkshire 2020-03-18 Virology Department, Sheffield Teaching Hospitals NHS Foundation Trust Department of Infection, Immunity and Cardiovascular Disease, The Florey Institute, The Medical School, University of Sheffield Thushan de Silva, Matthew Parker, Adri Angyal, Rebecca Brown, Rachel Tucker, Paul Parsons, Danielle Groves, Alex Keeley, Dave Partridge, Matthew Wyles, Benjamin Lindsey, Mehmet Yavuz, Mohammad Raza, Cariad Evans
EPI_ISL_418295 hCoV-19/England/SHEF-BFE06/2020 Europe / United Kingdom / England / South Yorkshire 2020-03-17 Virology Department, Sheffield Teaching Hospitals NHS Foundation Trust Department of Infection, Immunity and Cardiovascular Disease, The Florey Institute, The Medical School, University of Sheffield Thushan de Silva, Matthew Parker, Adri Angyal, Rebecca Brown, Rachel Tucker, Paul Parsons, Danielle Groves, Alex Keeley, Dave Partridge, Matthew Wyles, Benjamin Lindsey, Mehmet Yavuz, Mohammad Raza, Cariad Evans
EPI_ISL_418548 hCoV-19/Ireland/21145/2020 Europe / Ireland / Dublin 2020-03-06 UCD National Virus Reference Laboratory UCD National Virus Reference Laboratory Michael Carr, Gabriel Gonzalez, Jonathan Dean, Suzie Coughlan, Alison Murphy, Kevin Byrne, Ken Wolfe, Jeff Connell, Brendan Loftus, Cillian F

De Gascun
EPI_ISL_419508 hCoV-19/Wales/PHWC-24D87/2020 Europe / United Kingdom / Wales 2020-03-20 Wales Specialist Virology Centre Public Health Wales Microbiology Cardiff Catherine Moore, Joanne Watkins, Sally Corden, Sara Rey, Matt Bull, Tom Connor
EPI_ISL_419541 hCoV-19/Germany/NRW-24/2020 Europe / Germany / North Rhine Westphalia / Duesseldorf 2020-03-14 Center of Medical Microbiology, Virology, and Hospital Hygiene, University of Duesseldorf Center of Medical Microbiology, Virology, and Hospital Hygiene, University of Duesseldorf Ortwin Adams, Marcel Andree, Alexander Dilthey, Torsten Feldt, Sandra Hauka, Torsten Houwaart, Björn-Erik Jensen, Detlef Kindgen-Milles, Malte Kohns Vasconcelos, Klaus Pfeffer, Tina Senff, Daniel Strelow, Jörg Timm, Andreas Walker, Tobias Wienemann
EPI_ISL_419542 hCoV-19/Germany/NRW-25/2020 Europe / Germany / North Rhine Westphalia / Duesseldorf 2020-03-15 Center of Medical Microbiology, Virology, and Hospital Hygiene, University of Duesseldorf Center of Medical Microbiology, Virology, and Hospital Hygiene, University of Duesseldorf Ortwin Adams, Marcel Andree, Alexander Dilthey, Torsten Feldt, Sandra Hauka, Torsten Houwaart, Björn-Erik Jensen, Detlef Kindgen-Milles, Malte Kohns Vasconcelos, Klaus Pfeffer, Tina Senff, Daniel Strelow, Jörg Timm, Andreas Walker, Tobias Wienemann
EPI_ISL_419543 hCoV-19/Germany/NRW-26/2020 Europe / Germany / North Rhine Westphalia / Duesseldorf 2020-03-15 Center of Medical Microbiology, Virology, and Hospital Hygiene, University of Duesseldorf Center of Medical Microbiology, Virology, and Hospital Hygiene, University of Duesseldorf Ortwin Adams, Marcel Andree, Alexander Dilthey, Torsten Feldt, Sandra Hauka, Torsten Houwaart, Björn-Erik Jensen, Detlef Kindgen-Milles, Malte Kohns Vasconcelos, Klaus Pfeffer, Tina Senff, Daniel Strelow, Jörg Timm, Andreas Walker, Tobias Wienemann
EPI_ISL_419544 hCoV-19/Germany/NRW-27/2020 Europe / Germany / North Rhine Westphalia / Duesseldorf 2020-03-15 Center of Medical Microbiology, Virology, and Hospital Hygiene, University of Duesseldorf Center of Medical Microbiology, Virology, and Hospital Hygiene, University of Duesseldorf Ortwin Adams, Marcel Andree, Alexander Dilthey, Torsten Feldt, Sandra Hauka, Torsten Houwaart, Björn-Erik Jensen, Detlef Kindgen-Milles, Malte Kohns Vasconcelos, Klaus Pfeffer, Tina Senff, Daniel Strelow, Jörg Timm, Andreas Walker, Tobias Wienemann
EPI_ISL_419545 hCoV-19/Germany/NRW-28/2020 Europe / Germany / North Rhine Westphalia / Duesseldorf 2020-03-15 Center of Medical Microbiology, Virology, and Hospital Hygiene, University of Duesseldorf Center of Medical Microbiology, Virology, and Hospital Hygiene, University of Duesseldorf Ortwin Adams, Marcel Andree, Alexander Dilthey, Torsten Feldt, Sandra Hauka, Torsten Houwaart, Björn-Erik Jensen, Detlef Kindgen-Milles, Malte Kohns Vasconcelos, Klaus Pfeffer, Tina Senff, Daniel Strelow, Jörg Timm, Andreas Walker, Tobias Wienemann
EPI_ISL_419546 hCoV-19/Germany/NRW-29/2020 Europe / Germany / North Rhine Westphalia / Duesseldorf 2020-03-15 Center of Medical Microbiology, Virology, and Hospital Hygiene, University of Duesseldorf Center of Medical Microbiology, Virology, and Hospital Hygiene, University of Duesseldorf Ortwin Adams, Marcel Andree, Alexander Dilthey, Torsten Feldt, Sandra Hauka, Torsten Houwaart, Björn-Erik Jensen, Detlef Kindgen-Milles, Malte Kohns Vasconcelos, Klaus Pfeffer, Tina Senff, Daniel Strelow, Jörg Timm, Andreas Walker, Tobias Wienemann
EPI_ISL_419548 hCoV-19/Germany/NRW-31/2020 Europe / Germany / North Rhine Westphalia / Duesseldorf 2020-03-15 Center of Medical Microbiology, Virology, and Hospital Hygiene, University of Duesseldorf Center of Medical Microbiology, Virology, and Hospital Hygiene, University of Duesseldorf Ortwin Adams, Marcel Andree, Alexander Dilthey, Torsten Feldt, Sandra Hauka, Torsten Houwaart, Björn-Erik Jensen, Detlef Kindgen-Milles, Malte Kohns Vasconcelos, Klaus Pfeffer, Tina Senff, Daniel Strelow, Jörg Timm, Andreas Walker, Tobias Wienemann
EPI_ISL_419549 hCoV-19/Germany/NRW-32/2020 Europe / Germany / North Rhine Westphalia / Duesseldorf 2020-03-15 Center of Medical Microbiology, Virology, and Hospital Hygiene, University of Duesseldorf Center of Medical Microbiology, Virology, and Hospital Hygiene, University of Duesseldorf Ortwin Adams, Marcel Andree, Alexander Dilthey, Torsten Feldt, Sandra Hauka, Torsten Houwaart, Björn-Erik Jensen, Detlef Kindgen-Milles, Malte Kohns Vasconcelos, Klaus Pfeffer, Tina Senff, Daniel Strelow, Jörg Timm, Andreas Walker, Tobias Wienemann
EPI_ISL_419550 hCoV-19/Germany/NRW-33/2020 Europe / Germany / North Rhine Westphalia / Duesseldorf 2020-03-16 Center of Medical Microbiology, Virology, and Hospital Hygiene, University of Duesseldorf Center of Medical Microbiology, Virology, and Hospital Hygiene, University of Duesseldorf Ortwin Adams, Marcel Andree, Alexander Dilthey, Torsten Feldt, Sandra Hauka, Torsten Houwaart, Björn-Erik Jensen, Detlef Kindgen-Milles, Malte Kohns Vasconcelos, Klaus Pfeffer, Tina Senff, Daniel Strelow, Jörg Timm, Andreas Walker, Tobias Wienemann
EPI_ISL_419551 hCoV-19/Germany/NRW-34/2020 Europe / Germany / North Rhine Westphalia / Duesseldorf 2020-03-16 Center of Medical Microbiology, Virology, and Hospital Hygiene, University of Duesseldorf Center of Medical Microbiology, Virology, and Hospital Hygiene, University of Duesseldorf Ortwin Adams, Marcel Andree, Alexander Dilthey, Torsten Feldt, Sandra Hauka, Torsten Houwaart, Björn-Erik Jensen, Detlef Kindgen-Milles, Malte Kohns Vasconcelos, Klaus Pfeffer, Tina Senff, Daniel Strelow, Jörg Timm, Andreas Walker, Tobias Wienemann
EPI_ISL_419552 hCoV-19/Germany/NRW-35/2020 Europe / Germany / North Rhine Westphalia / Duesseldorf 2020-03-16 Center of Medical Microbiology, Virology, and Hospital Hygiene, University of Duesseldorf Center of Medical Microbiology, Virology, and Hospital Hygiene, University of Duesseldorf Ortwin Adams, Marcel Andree, Alexander Dilthey, Torsten Feldt, Sandra Hauka, Torsten Houwaart, Björn-Erik Jensen, Detlef Kindgen-Milles, Malte Kohns Vasconcelos, Klaus Pfeffer, Tina Senff, Daniel Strelow, Jörg Timm, Andreas Walker, Tobias Wienemann

EPI_ISL_419553 hCoV-19/USA/RI_0520/2020 North America / USA / Rhode Island 2020-02-28 RI State Health Laboratories Pathogen Discovery, Respiratory Viruses Branch, Division of Viral Diseases, Centers for Disease Control and Prevention Ying Tao, Jing Zhang, Krista Queen, Anna Uehara, Clinton R. Paden, Yan Li, Haibin Wang, Jasmine Padilla, Justin Lee, Suxiang Tong
EPI_ISL_419554 hCoV-19/USA/CA_2602/2020 North America / USA / California 2020-02-26 California Department of Public Health Pathogen Discovery, Respiratory Viruses Branch, Division of Viral Diseases, Centers for Disease Control and Prevention Ying Tao, Jing Zhang, Krista Queen, Anna Uehara, Clinton R. Paden, Yan Li, Haibin Wang, Jasmine Padilla, Justin Lee, Suxiang Tong
EPI_ISL_419555 hCoV-19/USA/WA_5030/2020 North America / USA / Washington 2020-02-27 WA State Department of Health Pathogen Discovery, Respiratory Viruses Branch, Division of Viral Diseases, Centers for Disease Control and Prevention Ying Tao, Jing Zhang, Krista Queen, Anna Uehara, Clinton R. Paden, Yan Li, Haibin Wang, Jasmine Padilla, Justin Lee, Suxiang Tong
EPI_ISL_419556 hCoV-19/USA/GA_2741/2020 North America / USA / Georgia 2020-02-29 GA Department of Public Health Laboratory Pathogen Discovery, Respiratory Viruses Branch, Division of Viral Diseases, Centers for Disease Control and Prevention Ying Tao, Jing Zhang, Krista Queen, Anna Uehara, Clinton R. Paden, Yan Li, Haibin Wang, Jasmine Padilla, Justin Lee, Suxiang Tong
EPI_ISL_419557 hCoV-19/USA/GA_2742/2020 North America / USA / Georgia 2020-02-29 GA Department of Public Health Laboratory Pathogen Discovery, Respiratory Viruses Branch, Division of Viral Diseases, Centers for Disease Control and Prevention Ying Tao, Jing Zhang, Krista Queen, Anna Uehara, Clinton R. Paden, Yan Li, Haibin Wang, Jasmine Padilla, Justin Lee, Suxiang Tong
EPI_ISL_419558 hCoV-19/USA/OR_2656/2020 North America / USA / Oregon 2020-02-27 OR State PHL-Virology/Immunology Section Pathogen Discovery, Respiratory Viruses Branch, Division of Viral Diseases, Centers for Disease Control and Prevention Ying Tao, Jing Zhang, Krista Queen, Anna Uehara, Clinton R. Paden, Yan Li, Haibin Wang, Jasmine Padilla, Justin Lee, Suxiang Tong
EPI_ISL_419559 hCoV-19/USA/FL_5125/2020 North America / USA / Florida 2020-02-28 FL Bureau of Public Health Laboratories-Tampa Pathogen Discovery, Respiratory Viruses Branch, Division of Viral Diseases, Centers for Disease Control and Prevention Anna Uehara, Ying Tao, Jing Zhang, Krista Queen, Clinton R. Paden, Yan Li, Haibin Wang, Jasmine Padilla, Justin Lee, Suxiang Tong
EPI_ISL_419560 hCoV-19/USA/FL_5091/2020 North America / USA / Florida 2020-02-28 FL Bureau of Public Health Laboratories-Tampa Pathogen Discovery, Respiratory Viruses Branch, Division of Viral Diseases, Centers for Disease Control and Prevention Anna Uehara, Ying Tao, Jing Zhang, Krista Queen, Clinton R. Paden, Yan Li, Haibin Wang, Jasmine Padilla, Justin Lee, Suxiang Tong
EPI_ISL_419562 hCoV-19/Luxembourg/LNS0000001/2020 Europe / Luxembourg 2020-02-29 Laboratoire National de Santé, Microbiology, Virology Laboratoire National de Santé, Microbiology, Epidemiology and Microbial Genomics Anke Wienecke-Baldacchino, Ardashel Latsuzbaia, Jessica Tapp, Catherine Ragimbeau, Guillaume Fournier, Tamir Abdelrahman, Trung Nguyen Nguyen, Joel Mossong
EPI_ISL_419563 hCoV-19/Luxembourg/LNS0156959/2020 Europe / Luxembourg 2020-03-12 Laboratoire National de Santé, Microbiology, Virology Laboratoire National de Santé, Microbiology, Epidemiology and Microbial Genomics Anke Wienecke-Baldacchino, Ardashel Latsuzbaia, Jessica Tapp, Catherine Ragimbeau, Guillaume Fournier, Tamir Abdelrahman, Trung Nguyen Nguyen, Joel Mossong
EPI_ISL_419564 hCoV-19/Luxembourg/LNS0366116/2020 Europe / Luxembourg 2020-03-15 Laboratoire National de Santé, Microbiology, Virology Laboratoire National de Santé, Microbiology, Epidemiology and Microbial Genomics Anke Wienecke-Baldacchino, Ardashel Latsuzbaia, Jessica Tapp, Catherine Ragimbeau, Guillaume Fournier, Tamir Abdelrahman, Trung Nguyen Nguyen, Joel Mossong
EPI_ISL_419566 hCoV-19/Luxembourg/LNS0641910/2020 Europe / Luxembourg 2020-03-05 Laboratoire National de Santé, Microbiology, Virology Laboratoire National de Santé, Microbiology, Epidemiology and Microbial Genomics Anke Wienecke-Baldacchino, Ardashel Latsuzbaia, Jessica Tapp, Catherine Ragimbeau, Guillaume Fournier, Tamir Abdelrahman, Trung Nguyen Nguyen, Joel Mossong
EPI_ISL_419568 hCoV-19/Luxembourg/LNS0756270/2020 Europe / Luxembourg 2020-03-14 Laboratoire National de Santé, Microbiology, Virology Laboratoire National de Santé, Microbiology, Epidemiology and Microbial Genomics Anke Wienecke-Baldacchino, Ardashel Latsuzbaia, Jessica Tapp, Catherine Ragimbeau, Guillaume Fournier, Tamir Abdelrahman, Trung Nguyen Nguyen, Joel Mossong
EPI_ISL_419569 hCoV-19/Luxembourg/LNS0945359/2020 Europe / Luxembourg 2020-03-07 Laboratoire National de Santé, Microbiology, Virology Laboratoire National de Santé, Microbiology, Epidemiology and Microbial Genomics Anke Wienecke-Baldacchino, Ardashel Latsuzbaia, Jessica Tapp, Catherine Ragimbeau, Guillaume Fournier, Tamir Abdelrahman, Trung Nguyen Nguyen, Joel Mossong
EPI_ISL_419570 hCoV-19/Luxembourg/LNS1234709/2020 Europe / Luxembourg 2020-03-12 Laboratoire National de Santé, Microbiology, Virology Laboratoire National de Santé, Microbiology, Epidemiology and Microbial Genomics Anke Wienecke-Baldacchino, Ardashel Latsuzbaia, Jessica Tapp, Catherine Ragimbeau, Guillaume Fournier, Tamir Abdelrahman, Trung Nguyen Nguyen, Joel Mossong
EPI_ISL_419573 hCoV-19/Luxembourg/LNS1874423/2020 Europe / Luxembourg 2020-03-11 Laboratoire National de Santé, Microbiology, Virology Laboratoire National de Santé, Microbiology, Epidemiology and Microbial Genomics Anke Wienecke-Baldacchino, Ardashel Latsuzbaia, Jessica Tapp, Catherine Ragimbeau, Guillaume Fournier, Tamir Abdelrahman, Trung Nguyen Nguyen, Joel Mossong
EPI_ISL_419578 hCoV-19/Luxembourg/LNS2614631/2020 Europe / Luxembourg 2020-03-08 Laboratoire National de Santé, Microbiology, Virology Laboratoire National de Santé, Microbiology, Epidemiology and Microbial Genomics Anke Wienecke-Baldacchino, Ardashel Latsuzbaia, Jessica Tapp, Catherine Ragimbeau, Guillaume Fournier, Tamir Abdelrahman, Trung Nguyen Nguyen, Joel Mossong
EPI_ISL_419579 hCoV-19/Luxembourg/LNS2886370/2020 Europe / Luxembourg 2020-03-14 Laboratoire National de Santé, Microbiology, Virology Laboratoire National de Santé, Microbiology, Epidemiology and

Microbial Genomics	Anke Wienecke-Baldacchino, Ardashel Latsuzbaia, Jessica Tapp, Catherine Ragimbeau, Guillaume Fournier, Tamir Abdelrahman, Trung Nguyen Nguyen, Joel Mossong
EPI_ISL_419580	hCoV-19/Luxembourg/LNS2907333/2020	Europe / Luxembourg	2020-03-12	Laboratoire National de Santé, Microbiology, Virology	Laboratoire National de Santé, Microbiology, Epidemiology and Microbial Genomics	Anke Wienecke-Baldacchino, Ardashel Latsuzbaia, Jessica Tapp, Catherine Ragimbeau, Guillaume Fournier, Tamir Abdelrahman, Trung Nguyen Nguyen, Joel Mossong
EPI_ISL_419582	hCoV-19/Luxembourg/LNS3156434/2020	Europe / Luxembourg	2020-03-13	Laboratoire National de Santé, Microbiology, Virology	Laboratoire National de Santé, Microbiology, Epidemiology and Microbial Genomics	Anke Wienecke-Baldacchino, Ardashel Latsuzbaia, Jessica Tapp, Catherine Ragimbeau, Guillaume Fournier, Tamir Abdelrahman, Trung Nguyen Nguyen, Joel Mossong
EPI_ISL_419583	hCoV-19/Luxembourg/LNS3588186/2020	Europe / Luxembourg	2020-03-15	Laboratoire National de Santé, Microbiology, Virology	Laboratoire National de Santé, Microbiology, Epidemiology and Microbial Genomics	Anke Wienecke-Baldacchino, Ardashel Latsuzbaia, Jessica Tapp, Catherine Ragimbeau, Guillaume Fournier, Tamir Abdelrahman, Trung Nguyen Nguyen, Joel Mossong
EPI_ISL_419584	hCoV-19/Luxembourg/LNS3711853/2020	Europe / Luxembourg	2020-03-13	Laboratoire National de Santé, Microbiology, Virology	Laboratoire National de Santé, Microbiology, Epidemiology and Microbial Genomics	Anke Wienecke-Baldacchino, Ardashel Latsuzbaia, Jessica Tapp, Catherine Ragimbeau, Guillaume Fournier, Tamir Abdelrahman, Trung Nguyen Nguyen, Joel Mossong
EPI_ISL_419585	hCoV-19/Luxembourg/LNS3879580/2020	Europe / Luxembourg	2020-03-14	Laboratoire National de Santé, Microbiology, Virology	Laboratoire National de Santé, Microbiology, Epidemiology and Microbial Genomics	Anke Wienecke-Baldacchino, Ardashel Latsuzbaia, Jessica Tapp, Catherine Ragimbeau, Guillaume Fournier, Tamir Abdelrahman, Trung Nguyen Nguyen, Joel Mossong
EPI_ISL_419586	hCoV-19/Luxembourg/LNS4134806/2020	Europe / Luxembourg	2020-03-14	Laboratoire National de Santé, Microbiology, Virology	Laboratoire National de Santé, Microbiology, Epidemiology and Microbial Genomics	Anke Wienecke-Baldacchino, Ardashel Latsuzbaia, Jessica Tapp, Catherine Ragimbeau, Guillaume Fournier, Tamir Abdelrahman, Trung Nguyen Nguyen, Joel Mossong
EPI_ISL_419587	hCoV-19/Luxembourg/LNS4691488/2020	Europe / Luxembourg	2020-03-11	Laboratoire National de Santé, Microbiology, Virology	Laboratoire National de Santé, Microbiology, Epidemiology and Microbial Genomics	Anke Wienecke-Baldacchino, Ardashel Latsuzbaia, Jessica Tapp, Catherine Ragimbeau, Guillaume Fournier, Tamir Abdelrahman, Trung Nguyen Nguyen, Joel Mossong
EPI_ISL_419588	hCoV-19/Luxembourg/LNS4836560/2020	Europe / Luxembourg	2020-03-16	Laboratoire National de Santé, Microbiology, Virology	Laboratoire National de Santé, Microbiology, Epidemiology and Microbial Genomics	Anke Wienecke-Baldacchino, Ardashel Latsuzbaia, Jessica Tapp, Catherine Ragimbeau, Guillaume Fournier, Tamir Abdelrahman, Trung Nguyen Nguyen, Joel Mossong
EPI_ISL_419589	hCoV-19/Luxembourg/LNS4845603/2020	Europe / Luxembourg	2020-03-15	Laboratoire National de Santé, Microbiology, Virology	Laboratoire National de Santé, Microbiology, Epidemiology and Microbial Genomics	Anke Wienecke-Baldacchino, Ardashel Latsuzbaia, Jessica Tapp, Catherine Ragimbeau, Guillaume Fournier, Tamir Abdelrahman, Trung Nguyen Nguyen, Joel Mossong
EPI_ISL_419590	hCoV-19/Luxembourg/LNS5032431/2020	Europe / Luxembourg	2020-03-11	Laboratoire National de Santé, Microbiology, Virology	Laboratoire National de Santé, Microbiology, Epidemiology and Microbial Genomics	Anke Wienecke-Baldacchino, Ardashel Latsuzbaia, Jessica Tapp, Catherine Ragimbeau, Guillaume Fournier, Tamir Abdelrahman, Trung Nguyen Nguyen, Joel Mossong
EPI_ISL_419591	hCoV-19/Luxembourg/LNS5228153/2020	Europe / Luxembourg	2020-03-14	Laboratoire National de Santé, Microbiology, Virology	Laboratoire National de Santé, Microbiology, Epidemiology and Microbial Genomics	Anke Wienecke-Baldacchino, Ardashel Latsuzbaia, Jessica Tapp, Catherine Ragimbeau, Guillaume Fournier, Tamir Abdelrahman, Trung Nguyen Nguyen, Joel Mossong
EPI_ISL_419592	hCoV-19/Luxembourg/LNS5440244/2020	Europe / Luxembourg	2020-03-15	Laboratoire National de Santé, Microbiology, Virology	Laboratoire National de Santé, Microbiology, Epidemiology and Microbial Genomics	Anke Wienecke-Baldacchino, Ardashel Latsuzbaia, Jessica Tapp, Catherine Ragimbeau, Guillaume Fournier, Tamir Abdelrahman, Trung Nguyen Nguyen, Joel Mossong
EPI_ISL_419593	hCoV-19/Luxembourg/LNS6603085/2020	Europe / Luxembourg	2020-03-16	Laboratoire National de Santé, Microbiology, Virology	Laboratoire National de Santé, Microbiology, Epidemiology and Microbial Genomics	Anke Wienecke-Baldacchino, Ardashel Latsuzbaia, Jessica Tapp, Catherine Ragimbeau, Guillaume Fournier, Tamir Abdelrahman, Trung Nguyen Nguyen, Joel Mossong
EPI_ISL_419594	hCoV-19/Luxembourg/LNS7537751/2020	Europe / Luxembourg	2020-03-16	Laboratoire National de Santé, Microbiology, Virology	Laboratoire National de Santé, Microbiology, Epidemiology and Microbial Genomics	Anke Wienecke-Baldacchino, Ardashel Latsuzbaia, Jessica Tapp, Catherine Ragimbeau, Guillaume Fournier, Tamir Abdelrahman, Trung Nguyen Nguyen, Joel Mossong
EPI_ISL_419595	hCoV-19/Luxembourg/LNS7753431/2020	Europe / Luxembourg	2020-03-12	Laboratoire National de Santé, Microbiology, Virology	Laboratoire National de Santé, Microbiology, Epidemiology and Microbial Genomics	Anke Wienecke-Baldacchino, Ardashel Latsuzbaia, Jessica Tapp, Catherine Ragimbeau, Guillaume Fournier, Tamir Abdelrahman, Trung Nguyen Nguyen, Joel Mossong
EPI_ISL_419596	hCoV-19/Luxembourg/LNS7866283/2020	Europe / Luxembourg	2020-03-12	Laboratoire National de Santé, Microbiology, Virology	Laboratoire National de Santé, Microbiology, Epidemiology and Microbial Genomics	Anke Wienecke-Baldacchino, Ardashel Latsuzbaia, Jessica Tapp, Catherine Ragimbeau, Guillaume Fournier, Tamir Abdelrahman, Trung Nguyen Nguyen, Joel Mossong
EPI_ISL_419597	hCoV-19/Luxembourg/LNS7928872/2020	Europe / Luxembourg	2020-03-15	Laboratoire National de Santé, Microbiology, Virology	Laboratoire National de Santé, Microbiology, Epidemiology and Microbial Genomics	Anke Wienecke-Baldacchino, Ardashel Latsuzbaia, Jessica Tapp, Catherine Ragimbeau, Guillaume Fournier, Tamir Abdelrahman, Trung Nguyen Nguyen, Joel Mossong

EPI_ISL_419598 hCoV-19/Luxembourg/LNS8188502/2020 Europe / Luxembourg 2020-03-14 Laboratoire National de Santé, Microbiology, Virology Laboratoire National de Santé, Microbiology, Epidemiology and Microbial Genomics Anke Wienecke-Baldacchino, Ardashel Latsuzbaia, Jessica Tapp, Catherine Ragimbeau, Guillaume Fournier, Tamir Abdelrahman, Trung Nguyen Nguyen, Joel Mossong
EPI_ISL_419599 hCoV-19/Luxembourg/LNS8639502/2020 Europe / Luxembourg 2020-03-16 Laboratoire National de Santé, Microbiology, Virology Laboratoire National de Santé, Microbiology, Epidemiology and Microbial Genomics Anke Wienecke-Baldacchino, Ardashel Latsuzbaia, Jessica Tapp, Catherine Ragimbeau, Guillaume Fournier, Tamir Abdelrahman, Trung Nguyen Nguyen, Joel Mossong
EPI_ISL_419800 hCoV-19/Australia/VIC89/2020 Oceania / Australia / Victoria 2020-03-15 Victorian Infectious Diseases Reference Laboratory (VIDRL) Victorian Infectious Diseases Reference Laboratory and Microbiological Diagnostic Unit Public Health Laboratory, Doherty Institute Caly L., Seemann T., Sait, M., Schultz M., Druce J., Sherry, N.
EPI_ISL_419801 hCoV-19/Australia/VIC90/2020 Oceania / Australia / Victoria 2020-03-15 Victorian Infectious Diseases Reference Laboratory (VIDRL) Victorian Infectious Diseases Reference Laboratory and Microbiological Diagnostic Unit Public Health Laboratory, Doherty Institute Caly L., Seemann T., Sait, M., Schultz M., Druce J., Sherry, N.
EPI_ISL_419802 hCoV-19/Australia/VIC45/2020 Oceania / Australia / Victoria 2020-03-11 Victorian Infectious Diseases Reference Laboratory (VIDRL) Victorian Infectious Diseases Reference Laboratory and Microbiological Diagnostic Unit Public Health Laboratory, Doherty Institute Caly L., Seemann T., Sait, M., Schultz M., Druce J., Sherry, N.
EPI_ISL_419803 hCoV-19/Australia/VIC84/2020 Oceania / Australia / Victoria 2020-03-14 Victorian Infectious Diseases Reference Laboratory (VIDRL) Victorian Infectious Diseases Reference Laboratory and Microbiological Diagnostic Unit Public Health Laboratory, Doherty Institute Caly L., Seemann T., Sait, M., Schultz M., Druce J., Sherry, N.
EPI_ISL_419804 hCoV-19/Australia/VIC91/2020 Oceania / Australia / Victoria 2020-03-15 Victorian Infectious Diseases Reference Laboratory (VIDRL) Victorian Infectious Diseases Reference Laboratory and Microbiological Diagnostic Unit Public Health Laboratory, Doherty Institute Caly L., Seemann T., Sait, M., Schultz M., Druce J., Sherry, N.
EPI_ISL_419805 hCoV-19/Australia/VIC94/2020 Oceania / Australia / Victoria 2020-03-16 Victorian Infectious Diseases Reference Laboratory (VIDRL) Victorian Infectious Diseases Reference Laboratory and Microbiological Diagnostic Unit Public Health Laboratory, Doherty Institute Caly L., Seemann T., Sait, M., Schultz M., Druce J., Sherry, N.
EPI_ISL_419806 hCoV-19/Australia/VIC95/2020 Oceania / Australia / Victoria 2020-03-16 Victorian Infectious Diseases Reference Laboratory (VIDRL) Victorian Infectious Diseases Reference Laboratory and Microbiological Diagnostic Unit Public Health Laboratory, Doherty Institute Caly L., Seemann T., Sait, M., Schultz M., Druce J., Sherry, N.
EPI_ISL_419807 hCoV-19/Australia/VIC96/2020 Oceania / Australia / Victoria 2020-03-16 Victorian Infectious Diseases Reference Laboratory (VIDRL) Victorian Infectious Diseases Reference Laboratory and Microbiological Diagnostic Unit Public Health Laboratory, Doherty Institute Caly L., Seemann T., Sait, M., Schultz M., Druce J., Sherry, N.
EPI_ISL_419808 hCoV-19/Australia/VIC97/2020 Oceania / Australia / Victoria 2020-03-16 Victorian Infectious Diseases Reference Laboratory (VIDRL) Victorian Infectious Diseases Reference Laboratory and Microbiological Diagnostic Unit Public Health Laboratory, Doherty Institute Caly L., Seemann T., Sait, M., Schultz M., Druce J., Sherry, N.
EPI_ISL_419809 hCoV-19/Australia/VIC92/2020 Oceania / Australia / Victoria 2020-03-15 Victorian Infectious Diseases Reference Laboratory (VIDRL) Victorian Infectious Diseases Reference Laboratory and Microbiological Diagnostic Unit Public Health Laboratory, Doherty Institute Caly L., Seemann T., Sait, M., Schultz M., Druce J., Sherry, N.
EPI_ISL_419810 hCoV-19/Australia/VIC98/2020 Oceania / Australia / Victoria 2020-03-16 Victorian Infectious Diseases Reference Laboratory (VIDRL) Victorian Infectious Diseases Reference Laboratory and Microbiological Diagnostic Unit Public Health Laboratory, Doherty Institute Caly L., Seemann T., Sait, M., Schultz M., Druce J., Sherry, N.
EPI_ISL_419811 hCoV-19/Australia/VIC99/2020 Oceania / Australia / Victoria 2020-03-16 Victorian Infectious Diseases Reference Laboratory (VIDRL) Victorian Infectious Diseases Reference Laboratory and Microbiological Diagnostic Unit Public Health Laboratory, Doherty Institute Caly L., Seemann T., Sait, M., Schultz M., Druce J., Sherry, N.
EPI_ISL_419812 hCoV-19/Australia/VIC100/2020 Oceania / Australia / Victoria 2020-03-16 Victorian Infectious Diseases Reference Laboratory (VIDRL) Victorian Infectious Diseases Reference Laboratory and Microbiological Diagnostic Unit Public Health Laboratory, Doherty Institute Caly L., Seemann T., Sait, M., Schultz M., Druce J., Sherry, N.
EPI_ISL_419813 hCoV-19/Australia/VIC101/2020 Oceania / Australia / Victoria 2020-03-16 Victorian Infectious Diseases Reference Laboratory (VIDRL) Victorian Infectious Diseases Reference Laboratory and Microbiological Diagnostic Unit Public Health Laboratory, Doherty Institute Caly L., Seemann T., Sait, M., Schultz M., Druce J., Sherry, N.
EPI_ISL_419814 hCoV-19/Australia/VIC103/2020 Oceania / Australia / Victoria 2020-03-17 Victorian Infectious Diseases Reference Laboratory (VIDRL) Victorian Infectious Diseases Reference Laboratory and Microbiological Diagnostic Unit Public Health Laboratory, Doherty Institute Caly L., Seemann T., Sait, M., Schultz M., Druce J., Sherry, N.
EPI_ISL_419816 hCoV-19/Australia/VIC46/2020 Oceania / Australia / Victoria 2020-03-11 Victorian Infectious Diseases Reference Laboratory (VIDRL) Victorian Infectious Diseases Reference Laboratory and

Microbiological Diagnostic Unit Public Health Laboratory, Doherty Institute Caly L., Seemann T., Sait, M., Schultz M., Druce J., Sherry, N.
EPI_ISL_419817 hCoV-19/Australia/VIC102/2020 Oceania / Australia / Victoria 2020-03-16 Victorian Infectious Diseases Reference Laboratory (VIDRL) Victorian Infectious Diseases Reference Laboratory and Microbiological Diagnostic Unit Public Health Laboratory, Doherty Institute Caly L., Seemann T., Sait, M., Schultz M., Druce J., Sherry, N.
EPI_ISL_419818 hCoV-19/Australia/VIC104/2020 Oceania / Australia / Victoria 2020-03-17 Victorian Infectious Diseases Reference Laboratory (VIDRL) Victorian Infectious Diseases Reference Laboratory and Microbiological Diagnostic Unit Public Health Laboratory, Doherty Institute Caly L., Seemann T., Sait, M., Schultz M., Druce J., Sherry, N.
EPI_ISL_419819 hCoV-19/Australia/VIC105/2020 Oceania / Australia / Victoria 2020-03-17 Victorian Infectious Diseases Reference Laboratory (VIDRL) Victorian Infectious Diseases Reference Laboratory and Microbiological Diagnostic Unit Public Health Laboratory, Doherty Institute Caly L., Seemann T., Sait, M., Schultz M., Druce J., Sherry, N.
EPI_ISL_419820 hCoV-19/Australia/VIC106/2020 Oceania / Australia / Victoria 2020-03-17 Victorian Infectious Diseases Reference Laboratory (VIDRL) Victorian Infectious Diseases Reference Laboratory and Microbiological Diagnostic Unit Public Health Laboratory, Doherty Institute Caly L., Seemann T., Sait, M., Schultz M., Druce J., Sherry, N.
EPI_ISL_419821 hCoV-19/Australia/VIC112/2020 Oceania / Australia / Victoria 2020-03-18 Victorian Infectious Diseases Reference Laboratory (VIDRL) Victorian Infectious Diseases Reference Laboratory and Microbiological Diagnostic Unit Public Health Laboratory, Doherty Institute Caly L., Seemann T., Sait, M., Schultz M., Druce J., Sherry, N.
EPI_ISL_419822 hCoV-19/Australia/VIC113/2020 Oceania / Australia / Victoria 2020-03-18 Victorian Infectious Diseases Reference Laboratory (VIDRL) Victorian Infectious Diseases Reference Laboratory and Microbiological Diagnostic Unit Public Health Laboratory, Doherty Institute Caly L., Seemann T., Sait, M., Schultz M., Druce J., Sherry, N.
EPI_ISL_419823 hCoV-19/Australia/VIC130/2020 Oceania / Australia / Victoria 2020-03-21 Microbiological Diagnostic Unit Public Health Laboratory Microbiological Diagnostic Unit Public Health Laboratory Seemann T., Schultz M., Sait, M., Sherry, N.
EPI_ISL_419824 hCoV-19/Australia/VIC131/2020 Oceania / Australia / Victoria 2020-03-21 Microbiological Diagnostic Unit Public Health Laboratory Microbiological Diagnostic Unit Public Health Laboratory Seemann T., Schultz M., Sait, M., Sherry, N.
EPI_ISL_419825 hCoV-19/Australia/VIC132/2020 Oceania / Australia / Victoria 2020-03-22 Microbiological Diagnostic Unit Public Health Laboratory Microbiological Diagnostic Unit Public Health Laboratory Seemann T., Schultz M., Sait, M., Sherry, N.
EPI_ISL_419826 hCoV-19/Australia/VIC51/2020 Oceania / Australia / Victoria 2020-03-12 Victorian Infectious Diseases Reference Laboratory (VIDRL) Victorian Infectious Diseases Reference Laboratory and Microbiological Diagnostic Unit Public Health Laboratory, Doherty Institute Caly L., Seemann T., Sait, M., Schultz M., Druce J., Sherry, N.
EPI_ISL_419827 hCoV-19/Australia/VIC134/2020 Oceania / Australia / Victoria 2020-03-22 Microbiological Diagnostic Unit Public Health Laboratory Microbiological Diagnostic Unit Public Health Laboratory Seemann T., Schultz M., Sait, M., Sherry, N.
EPI_ISL_419828 hCoV-19/Australia/VIC135/2020 Oceania / Australia / Victoria 2020-03-22 Microbiological Diagnostic Unit Public Health Laboratory Microbiological Diagnostic Unit Public Health Laboratory Seemann T., Schultz M., Sait, M., Sherry, N.
EPI_ISL_419850 hCoV-19/Australia/VIC153/2020 Oceania / Australia / Victoria 2020-03-17 Victorian Infectious Diseases Reference Laboratory (VIDRL) Victorian Infectious Diseases Reference Laboratory and Microbiological Diagnostic Unit Public Health Laboratory, Doherty Institute Caly L., Seemann T., Sait, M., Schultz M., Druce J., Sherry, N.
EPI_ISL_419851 hCoV-19/Australia/VIC154/2020 Oceania / Australia / Victoria 2020-03-17 Victorian Infectious Diseases Reference Laboratory (VIDRL) Victorian Infectious Diseases Reference Laboratory and Microbiological Diagnostic Unit Public Health Laboratory, Doherty Institute Caly L., Seemann T., Sait, M., Schultz M., Druce J., Sherry, N.
EPI_ISL_419852 hCoV-19/Australia/VIC155/2020 Oceania / Australia / Victoria 2020-03-17 Victorian Infectious Diseases Reference Laboratory (VIDRL) Victorian Infectious Diseases Reference Laboratory and Microbiological Diagnostic Unit Public Health Laboratory, Doherty Institute Caly L., Seemann T., Sait, M., Schultz M., Druce J., Sherry, N.
EPI_ISL_419853 hCoV-19/Australia/VIC156/2020 Oceania / Australia / Victoria 2020-03-17 Victorian Infectious Diseases Reference Laboratory (VIDRL) Victorian Infectious Diseases Reference Laboratory and Microbiological Diagnostic Unit Public Health Laboratory, Doherty Institute Caly L., Seemann T., Sait, M., Schultz M., Druce J., Sherry, N.
EPI_ISL_419854 hCoV-19/Australia/VIC157/2020 Oceania / Australia / Victoria 2020-03-17 Victorian Infectious Diseases Reference Laboratory (VIDRL) Victorian Infectious Diseases Reference Laboratory and Microbiological Diagnostic Unit Public Health Laboratory, Doherty Institute Caly L., Seemann T., Sait, M., Schultz M., Druce J., Sherry, N.
EPI_ISL_419855 hCoV-19/Australia/VIC158/2020 Oceania / Australia / Victoria 2020-03-18 Victorian Infectious Diseases Reference Laboratory (VIDRL) Victorian Infectious Diseases Reference Laboratory and Microbiological Diagnostic Unit Public Health Laboratory, Doherty Institute Caly L., Seemann T., Sait, M., Schultz M., Druce J., Sherry, N.
EPI_ISL_419856 hCoV-19/Australia/VIC159/2020 Oceania / Australia / Victoria 2020-03-18 Victorian

Infectious Diseases Reference Laboratory (VIDRL)	Victorian Infectious Diseases Reference Laboratory and Microbiological Diagnostic Unit Public Health Laboratory, Doherty Institute	Caly L., Seemann T., Sait, M., Schultz M., Druce J., Sherry, N.
EPI_ISL_419857	hCoV-19/Australia/VIC160/2020	Oceania / Australia / Victoria	2020-03-18	Victorian Infectious Diseases Reference Laboratory (VIDRL)	Victorian Infectious Diseases Reference Laboratory and Microbiological Diagnostic Unit Public Health Laboratory, Doherty Institute	Caly L., Seemann T., Sait, M., Schultz M., Druce J., Sherry, N.
EPI_ISL_419859	hCoV-19/Australia/VIC162/2020	Oceania / Australia / Victoria	2020-03-18	Victorian Infectious Diseases Reference Laboratory (VIDRL)	Victorian Infectious Diseases Reference Laboratory and Microbiological Diagnostic Unit Public Health Laboratory, Doherty Institute	Caly L., Seemann T., Sait, M., Schultz M., Druce J., Sherry, N.
EPI_ISL_419860	hCoV-19/Australia/VIC163/2020	Oceania / Australia / Victoria	2020-03-18	Victorian Infectious Diseases Reference Laboratory (VIDRL)	Victorian Infectious Diseases Reference Laboratory and Microbiological Diagnostic Unit Public Health Laboratory, Doherty Institute	Caly L., Seemann T., Sait, M., Schultz M., Druce J., Sherry, N.
EPI_ISL_419861	hCoV-19/Australia/VIC164/2020	Oceania / Australia / Victoria	2020-03-18	Victorian Infectious Diseases Reference Laboratory (VIDRL)	Victorian Infectious Diseases Reference Laboratory and Microbiological Diagnostic Unit Public Health Laboratory, Doherty Institute	Caly L., Seemann T., Sait, M., Schultz M., Druce J., Sherry, N.
EPI_ISL_419862	hCoV-19/Australia/VIC165/2020	Oceania / Australia / Victoria	2020-03-18	Victorian Infectious Diseases Reference Laboratory (VIDRL)	Victorian Infectious Diseases Reference Laboratory and Microbiological Diagnostic Unit Public Health Laboratory, Doherty Institute	Caly L., Seemann T., Sait, M., Schultz M., Druce J., Sherry, N.
EPI_ISL_419863	hCoV-19/Australia/VIC166/2020	Oceania / Australia / Victoria	2020-03-18	Victorian Infectious Diseases Reference Laboratory (VIDRL)	Victorian Infectious Diseases Reference Laboratory and Microbiological Diagnostic Unit Public Health Laboratory, Doherty Institute	Caly L., Seemann T., Sait, M., Schultz M., Druce J., Sherry, N.
EPI_ISL_419864	hCoV-19/Australia/VIC167/2020	Oceania / Australia / Victoria	2020-03-18	Victorian Infectious Diseases Reference Laboratory (VIDRL)	Victorian Infectious Diseases Reference Laboratory and Microbiological Diagnostic Unit Public Health Laboratory, Doherty Institute	Caly L., Seemann T., Sait, M., Schultz M., Druce J., Sherry, N.
EPI_ISL_419865	hCoV-19/Australia/VIC168/2020	Oceania / Australia / Victoria	2020-03-18	Victorian Infectious Diseases Reference Laboratory (VIDRL)	Victorian Infectious Diseases Reference Laboratory and Microbiological Diagnostic Unit Public Health Laboratory, Doherty Institute	Caly L., Seemann T., Sait, M., Schultz M., Druce J., Sherry, N.
EPI_ISL_419866	hCoV-19/Australia/VIC169/2020	Oceania / Australia / Victoria	2020-03-18	Victorian Infectious Diseases Reference Laboratory (VIDRL)	Victorian Infectious Diseases Reference Laboratory and Microbiological Diagnostic Unit Public Health Laboratory, Doherty Institute	Caly L., Seemann T., Sait, M., Schultz M., Druce J., Sherry, N.
EPI_ISL_419867	hCoV-19/Australia/VIC170/2020	Oceania / Australia / Victoria	2020-03-18	Victorian Infectious Diseases Reference Laboratory (VIDRL)	Victorian Infectious Diseases Reference Laboratory and Microbiological Diagnostic Unit Public Health Laboratory, Doherty Institute	Caly L., Seemann T., Sait, M., Schultz M., Druce J., Sherry, N.
EPI_ISL_419868	hCoV-19/Australia/VIC171/2020	Oceania / Australia / Victoria	2020-03-18	Victorian Infectious Diseases Reference Laboratory (VIDRL)	Victorian Infectious Diseases Reference Laboratory and Microbiological Diagnostic Unit Public Health Laboratory, Doherty Institute	Caly L., Seemann T., Sait, M., Schultz M., Druce J., Sherry, N.
EPI_ISL_419869	hCoV-19/Australia/VIC172/2020	Oceania / Australia / Victoria	2020-03-18	Victorian Infectious Diseases Reference Laboratory (VIDRL)	Victorian Infectious Diseases Reference Laboratory and Microbiological Diagnostic Unit Public Health Laboratory, Doherty Institute	Caly L., Seemann T., Sait, M., Schultz M., Druce J., Sherry, N.
EPI_ISL_419870	hCoV-19/Australia/VIC173/2020	Oceania / Australia / Victoria	2020-03-18	Victorian Infectious Diseases Reference Laboratory (VIDRL)	Victorian Infectious Diseases Reference Laboratory and Microbiological Diagnostic Unit Public Health Laboratory, Doherty Institute	Caly L., Seemann T., Sait, M., Schultz M., Druce J., Sherry, N.
EPI_ISL_419871	hCoV-19/Australia/VIC174/2020	Oceania / Australia / Victoria	2020-03-18	Victorian Infectious Diseases Reference Laboratory (VIDRL)	Victorian Infectious Diseases Reference Laboratory and Microbiological Diagnostic Unit Public Health Laboratory, Doherty Institute	Caly L., Seemann T., Sait, M., Schultz M., Druce J., Sherry, N.
EPI_ISL_419872	hCoV-19/Australia/VIC175/2020	Oceania / Australia / Victoria	2020-03-18	Victorian Infectious Diseases Reference Laboratory (VIDRL)	Victorian Infectious Diseases Reference Laboratory and Microbiological Diagnostic Unit Public Health Laboratory, Doherty Institute	Caly L., Seemann T., Sait, M., Schultz M., Druce J., Sherry, N.
EPI_ISL_419873	hCoV-19/Australia/VIC176/2020	Oceania / Australia / Victoria	2020-03-18	Victorian Infectious Diseases Reference Laboratory (VIDRL)	Victorian Infectious Diseases Reference Laboratory and Microbiological Diagnostic Unit Public Health Laboratory, Doherty Institute	Caly L., Seemann T., Sait, M., Schultz M., Druce J., Sherry, N.
EPI_ISL_419874	hCoV-19/Australia/VIC177/2020	Oceania / Australia / Victoria	2020-03-18	Victorian Infectious Diseases Reference Laboratory (VIDRL)	Victorian Infectious Diseases Reference Laboratory and Microbiological Diagnostic Unit Public Health Laboratory, Doherty Institute	Caly L., Seemann T., Sait, M.,

Schultz M., Druce J., Sherry, N.
EPI_ISL_419875 hCoV-19/Australia/VIC178/2020 Oceania / Australia / Victoria 2020-03-18 Victorian Infectious Diseases Reference Laboratory (VIDRL) Victorian Infectious Diseases Reference Laboratory and Microbiological Diagnostic Unit Public Health Laboratory, Doherty Institute Caly L., Seemann T., Sait, M., Schultz M., Druce J., Sherry, N.
EPI_ISL_419876 hCoV-19/Australia/VIC179/2020 Oceania / Australia / Victoria 2020-03-18 Victorian Infectious Diseases Reference Laboratory (VIDRL) Victorian Infectious Diseases Reference Laboratory and Microbiological Diagnostic Unit Public Health Laboratory, Doherty Institute Caly L., Seemann T., Sait, M., Schultz M., Druce J., Sherry, N.
EPI_ISL_419877 hCoV-19/Australia/VIC180/2020 Oceania / Australia / Victoria 2020-03-19 Victorian Infectious Diseases Reference Laboratory (VIDRL) Victorian Infectious Diseases Reference Laboratory and Microbiological Diagnostic Unit Public Health Laboratory, Doherty Institute Caly L., Seemann T., Sait, M., Schultz M., Druce J., Sherry, N.
EPI_ISL_419878 hCoV-19/Australia/VIC181/2020 Oceania / Australia / Victoria 2020-03-19 Victorian Infectious Diseases Reference Laboratory (VIDRL) Victorian Infectious Diseases Reference Laboratory and Microbiological Diagnostic Unit Public Health Laboratory, Doherty Institute Caly L., Seemann T., Sait, M., Schultz M., Druce J., Sherry, N.
EPI_ISL_419879 hCoV-19/Australia/VIC183/2020 Oceania / Australia / Victoria 2020-03-19 Victorian Infectious Diseases Reference Laboratory (VIDRL) Victorian Infectious Diseases Reference Laboratory and Microbiological Diagnostic Unit Public Health Laboratory, Doherty Institute Caly L., Seemann T., Sait, M., Schultz M., Druce J., Sherry, N.
EPI_ISL_419880 hCoV-19/Australia/VIC184/2020 Oceania / Australia / Victoria 2020-03-19 Victorian Infectious Diseases Reference Laboratory (VIDRL) Victorian Infectious Diseases Reference Laboratory and Microbiological Diagnostic Unit Public Health Laboratory, Doherty Institute Caly L., Seemann T., Sait, M., Schultz M., Druce J., Sherry, N.
EPI_ISL_419881 hCoV-19/Australia/VIC185/2020 Oceania / Australia / Victoria 2020-03-19 Victorian Infectious Diseases Reference Laboratory (VIDRL) Victorian Infectious Diseases Reference Laboratory and Microbiological Diagnostic Unit Public Health Laboratory, Doherty Institute Caly L., Seemann T., Sait, M., Schultz M., Druce J., Sherry, N.
EPI_ISL_419882 hCoV-19/Australia/VIC186/2020 Oceania / Australia / Victoria 2020-03-19 Victorian Infectious Diseases Reference Laboratory (VIDRL) Victorian Infectious Diseases Reference Laboratory and Microbiological Diagnostic Unit Public Health Laboratory, Doherty Institute Caly L., Seemann T., Sait, M., Schultz M., Druce J., Sherry, N.
EPI_ISL_419883 hCoV-19/Australia/VIC187/2020 Oceania / Australia / Victoria 2020-03-19 Victorian Infectious Diseases Reference Laboratory (VIDRL) Victorian Infectious Diseases Reference Laboratory and Microbiological Diagnostic Unit Public Health Laboratory, Doherty Institute Caly L., Seemann T., Sait, M., Schultz M., Druce J., Sherry, N.
EPI_ISL_419884 hCoV-19/Australia/VIC188/2020 Oceania / Australia / Victoria 2020-03-19 Victorian Infectious Diseases Reference Laboratory (VIDRL) Victorian Infectious Diseases Reference Laboratory and Microbiological Diagnostic Unit Public Health Laboratory, Doherty Institute Caly L., Seemann T., Sait, M., Schultz M., Druce J., Sherry, N.
EPI_ISL_419885 hCoV-19/Australia/VIC189/2020 Oceania / Australia / Victoria 2020-03-19 Victorian Infectious Diseases Reference Laboratory (VIDRL) Victorian Infectious Diseases Reference Laboratory and Microbiological Diagnostic Unit Public Health Laboratory, Doherty Institute Caly L., Seemann T., Sait, M., Schultz M., Druce J., Sherry, N.
EPI_ISL_419886 hCoV-19/Australia/VIC190/2020 Oceania / Australia / Victoria 2020-03-19 Victorian Infectious Diseases Reference Laboratory (VIDRL) Victorian Infectious Diseases Reference Laboratory and Microbiological Diagnostic Unit Public Health Laboratory, Doherty Institute Caly L., Seemann T., Sait, M., Schultz M., Druce J., Sherry, N.
EPI_ISL_419887 hCoV-19/Australia/VIC191/2020 Oceania / Australia / Victoria 2020-03-19 Victorian Infectious Diseases Reference Laboratory (VIDRL) Victorian Infectious Diseases Reference Laboratory and Microbiological Diagnostic Unit Public Health Laboratory, Doherty Institute Caly L., Seemann T., Sait, M., Schultz M., Druce J., Sherry, N.
EPI_ISL_419888 hCoV-19/Australia/VIC192/2020 Oceania / Australia / Victoria 2020-03-19 Victorian Infectious Diseases Reference Laboratory (VIDRL) Victorian Infectious Diseases Reference Laboratory and Microbiological Diagnostic Unit Public Health Laboratory, Doherty Institute Caly L., Seemann T., Sait, M., Schultz M., Druce J., Sherry, N.
EPI_ISL_419889 hCoV-19/Australia/VIC193/2020 Oceania / Australia / Victoria 2020-03-19 Victorian Infectious Diseases Reference Laboratory (VIDRL) Victorian Infectious Diseases Reference Laboratory and Microbiological Diagnostic Unit Public Health Laboratory, Doherty Institute Caly L., Seemann T., Sait, M., Schultz M., Druce J., Sherry, N.
EPI_ISL_419890 hCoV-19/Australia/VIC194/2020 Oceania / Australia / Victoria 2020-03-19 Victorian Infectious Diseases Reference Laboratory (VIDRL) Victorian Infectious Diseases Reference Laboratory and Microbiological Diagnostic Unit Public Health Laboratory, Doherty Institute Caly L., Seemann T., Sait, M., Schultz M., Druce J., Sherry, N.
EPI_ISL_419891 hCoV-19/Australia/VIC195/2020 Oceania / Australia / Victoria 2020-03-19 Victorian Infectious Diseases Reference Laboratory (VIDRL) Victorian Infectious Diseases Reference Laboratory and Microbiological Diagnostic Unit Public Health Laboratory, Doherty Institute Caly L., Seemann T., Sait, M., Schultz M., Druce J., Sherry, N.
EPI_ISL_419892 hCoV-19/Australia/VIC196/2020 Oceania / Australia / Victoria 2020-03-19 Victorian

Infectious Diseases Reference Laboratory (VIDRL)	Victorian Infectious Diseases Reference Laboratory and Microbiological Diagnostic Unit Public Health Laboratory, Doherty Institute	Caly L., Seemann T., Sait, M., Schultz M., Druce J., Sherry, N.
EPI_ISL_419893	hCoV-19/Australia/VIC197/2020	Oceania / Australia / Victoria	2020-03-19	Victorian Infectious Diseases Reference Laboratory (VIDRL)	Victorian Infectious Diseases Reference Laboratory and Microbiological Diagnostic Unit Public Health Laboratory, Doherty Institute	Caly L., Seemann T., Sait, M., Schultz M., Druce J., Sherry, N.
EPI_ISL_419894	hCoV-19/Australia/VIC198/2020	Oceania / Australia / Victoria	2020-03-19	Victorian Infectious Diseases Reference Laboratory (VIDRL)	Victorian Infectious Diseases Reference Laboratory and Microbiological Diagnostic Unit Public Health Laboratory, Doherty Institute	Caly L., Seemann T., Sait, M., Schultz M., Druce J., Sherry, N.
EPI_ISL_419895	hCoV-19/Australia/VIC199/2020	Oceania / Australia / Victoria	2020-03-19	Victorian Infectious Diseases Reference Laboratory (VIDRL)	Victorian Infectious Diseases Reference Laboratory and Microbiological Diagnostic Unit Public Health Laboratory, Doherty Institute	Caly L., Seemann T., Sait, M., Schultz M., Druce J., Sherry, N.
EPI_ISL_419896	hCoV-19/Australia/VIC201/2020	Oceania / Australia / Victoria	2020-03-19	Victorian Infectious Diseases Reference Laboratory (VIDRL)	Victorian Infectious Diseases Reference Laboratory and Microbiological Diagnostic Unit Public Health Laboratory, Doherty Institute	Caly L., Seemann T., Sait, M., Schultz M., Druce J., Sherry, N.
EPI_ISL_419897	hCoV-19/Australia/VIC202/2020	Oceania / Australia / Victoria	2020-03-19	Victorian Infectious Diseases Reference Laboratory (VIDRL)	Victorian Infectious Diseases Reference Laboratory and Microbiological Diagnostic Unit Public Health Laboratory, Doherty Institute	Caly L., Seemann T., Sait, M., Schultz M., Druce J., Sherry, N.
EPI_ISL_419898	hCoV-19/Australia/VIC203/2020	Oceania / Australia / Victoria	2020-03-19	Victorian Infectious Diseases Reference Laboratory (VIDRL)	Victorian Infectious Diseases Reference Laboratory and Microbiological Diagnostic Unit Public Health Laboratory, Doherty Institute	Caly L., Seemann T., Sait, M., Schultz M., Druce J., Sherry, N.
EPI_ISL_419899	hCoV-19/Australia/VIC204/2020	Oceania / Australia / Victoria	2020-03-19	Victorian Infectious Diseases Reference Laboratory (VIDRL)	Victorian Infectious Diseases Reference Laboratory and Microbiological Diagnostic Unit Public Health Laboratory, Doherty Institute	Caly L., Seemann T., Sait, M., Schultz M., Druce J., Sherry, N.
EPI_ISL_420508	hCoV-19/England/20132072802/2020	Europe / United Kingdom / England	2020-03-23	Respiratory Virus Unit, Microbiology Services Colindale, Public Health England	Respiratory Virus Unit, Microbiology Services Colindale, Public Health England	Monica Galiano, Shahjahan Miah, Angie Lackenby, Omolola Akinbami, Tiina Talts, Leena Bhaw, Richard Myers, Steven Platt, Kirstin Edwards, Jonathan Hubb, Joanna Ellis, Maria Zambon
EPI_ISL_420520	hCoV-19/England/20136015502/2020	Europe / United Kingdom / England	2020-03-24	Respiratory Virus Unit, Microbiology Services Colindale, Public Health England	Respiratory Virus Unit, Microbiology Services Colindale, Public Health England	Monica Galiano, Shahjahan Miah, Angie Lackenby, Omolola Akinbami, Tiina Talts, Leena Bhaw, Richard Myers, Steven Platt, Kirstin Edwards, Jonathan Hubb, Joanna Ellis, Maria Zambon
EPI_ISL_420521	hCoV-19/England/20138012802/2020	Europe / United Kingdom / England	2020-03-25	Respiratory Virus Unit, Microbiology Services Colindale, Public Health England	Respiratory Virus Unit, Microbiology Services Colindale, Public Health England	Monica Galiano, Shahjahan Miah, Angie Lackenby, Omolola Akinbami, Tiina Talts, Leena Bhaw, Richard Myers, Steven Platt, Kirstin Edwards, Jonathan Hubb, Joanna Ellis, Maria Zambon
EPI_ISL_420522	hCoV-19/England/20138014402/2020	Europe / United Kingdom / England	2020-03-25	Respiratory Virus Unit, Microbiology Services Colindale, Public Health England	Respiratory Virus Unit, Microbiology Services Colindale, Public Health England	Monica Galiano, Shahjahan Miah, Angie Lackenby, Omolola Akinbami, Tiina Talts, Leena Bhaw, Richard Myers, Steven Platt, Kirstin Edwards, Jonathan Hubb, Joanna Ellis, Maria Zambon
EPI_ISL_420524	hCoV-19/England/20139018302/2020	Europe / United Kingdom / England	2020-03-26	Respiratory Virus Unit, Microbiology Services Colindale, Public Health England	Respiratory Virus Unit, Microbiology Services Colindale, Public Health England	Monica Galiano, Shahjahan Miah, Angie Lackenby, Omolola Akinbami, Tiina Talts, Leena Bhaw, Richard Myers, Steven Platt, Kirstin Edwards, Jonathan Hubb, Joanna Ellis, Maria Zambon
EPI_ISL_420531	hCoV-19/Australia/WA01/2020	Oceania / Australia	2020-03-14	Department of Microbiology, PathWest QEII Medical Centre	Department of Microbiology, PathWest QEII Medical Centre	Chisha Sikazwe, Jurissa Lang, Avram Levy, David Speers and David Smith
EPI_ISL_420532	hCoV-19/Australia/WA02/2020	Oceania / Australia / Western Australia / Perth	2020-03-13	Department of Microbiology, PathWest QEII Medical Centre	Department of Microbiology, PathWest QEII Medical Centre	Chisha Sikazwe, Jurissa Lang, Avram Levy, David Speers and David Smith
EPI_ISL_420533	hCoV-19/Australia/WA03/2020	Oceania / Australia / Western Australia / Perth	2020-03-14	Department of Microbiology, PathWest QEII Medical Centre	Department of Microbiology, PathWest QEII Medical Centre	Chisha Sikazwe, Jurissa Lang, Avram Levy, David Speers and David Smith
EPI_ISL_420534	hCoV-19/Australia/WA06/2020	Oceania / Australia / Western Australia / Perth	2020-03-04	Department of Microbiology, PathWest QEII Medical Centre	Department of Microbiology, PathWest QEII Medical Centre	Chisha Sikazwe, Jurissa Lang, Avram Levy, David Speers and David Smith
EPI_ISL_420536	hCoV-19/Australia/WA04/2020	Oceania / Australia / Western Australia / Perth	2020-03-15	Department of Microbiology, PathWest QEII Medical Centre	Department of Microbiology, PathWest QEII

Medical Centre		Chisha Sikazwe, Jurissa Lang, Avram Levy, David Speers and David Smith
EPI_ISL_420537	hCoV-19/Australia/WA07/2020	Oceania / Australia / Western Australia / Perth 2020-03-13	Department of Microbiology, PathWest QEII Medical Centre	Department of Microbiology, PathWest QEII Medical Centre		Chisha Sikazwe, Jurissa Lang, Avram Levy, David Speers and David Smith
EPI_ISL_420538	hCoV-19/Australia/WA09/2020	Oceania / Australia / Western Australia / Perth 2020-03-14	Department of Microbiology, PathWest QEII Medical Centre	Department of Microbiology, PathWest QEII Medical Centre		Chisha Sikazwe, Jurissa Lang, Avram Levy, David Speers and David Smith
EPI_ISL_420539	hCoV-19/Australia/WA10/2020	Oceania / Australia / Western Australia / Perth 2020-03-14	Department of Microbiology, PathWest QEII Medical Centre	Department of Microbiology, PathWest QEII Medical Centre		Chisha Sikazwe, Jurissa Lang, Avram Levy, David Speers and David Smith
EPI_ISL_420540	hCoV-19/Estonia/ChVir2148/2020	Europe / Estonia	2020-03 SYNLAB Eesti OÜ Charité Universitätsmedizin Berlin, Institute of Virology	Victor M Corman, Jörn Beheim-Schwarzbach, Barbara Mühlemann, Talitha Veith, Julia Schneider, Paul Naaber, Terry Jones, Christian Drosten
EPI_ISL_420541	hCoV-19/Slovenia/808/2020	Europe / Slovenia	2020-03-05	Institute of Microbiology and Immunology, Faculty of Medicine, University of Ljubljana	Institute of Microbiology and Immunology, Faculty of Medicine, University of Ljubljana	Tomaž Mark Zorec, Samo Zakotnik, Lucijan Skubic, Miša Korva, Tatjana Avšič - Županc, Mario Poljak
EPI_ISL_420543	hCoV-19/India/763/2020	Asia / India	2020-03-03	National Influenza Center, Indian Council of Medical Research - National Institute of Virology	Indian Council of Medical Research-National Institute of Virology, Microbial Containment Complex	Pragya D. Yadav. Savita Patil, Varsha Potdar, Prasad Sarkale, Dimpal A. Nyayanit, Gajanan Sapkal, Anita M. Shete, Atanu Basu, Lalit Dar, M Choudhary, Amita Jain, Bharati Malhotra, Pranita Gawande, Sarah Cherian, Priya Abraham
EPI_ISL_420544	hCoV-19/India/2020763/2020	Asia / India	2020	Indian Council of Medical Research-National Institute of Virology, Microbial Containment Complex	Indian Council of Medical Research-National Institute of Virology, Microbial Containment Complex	Pragya D. Yadav. Savita Patil, Varsha Potdar, Prasad Sarkale, Dimpal A. Nyayanit, Gajanan Sapkal, Anita M. Shete, Atanu Basu, Lalit Dar, M Choudhary, Amita Jain, Bharati Malhotra, Pranita Gawande, Sarah Cherian, Priya Abraham
EPI_ISL_420545	hCoV-19/India/770/2020	Asia / India	2020-03-03	National Influenza Center, Indian Council of Medical Research - National Institute of Virology	Indian Council of Medical Research-National Institute of Virology, Microbial Containment Complex	Pragya D. Yadav. Savita Patil, Varsha Potdar, Prasad Sarkale, Dimpal A. Nyayanit, Gajanan Sapkal, Anita M. Shete, Atanu Basu, Lalit Dar, M Choudhary, Amita Jain, Bharati Malhotra, Pranita Gawande, Sarah Cherian, Priya Abraham
EPI_ISL_420546	hCoV-19/India/2020770/2020	Asia / India	2020	Indian Council of Medical Research-National Institute of Virology, Microbial Containment Complex	Indian Council of Medical Research-National Institute of Virology, Microbial Containment Complex	Pragya D. Yadav. Savita Patil, Varsha Potdar, Prasad Sarkale, Dimpal A. Nyayanit, Gajanan Sapkal, Anita M. Shete, Atanu Basu, Lalit Dar, M Choudhary, Amita Jain, Bharati Malhotra, Pranita Gawande, Sarah Cherian, Priya Abraham
EPI_ISL_420547	hCoV-19/India/772/2020	Asia / India	2020-03-03	National Influenza Center, Indian Council of Medical Research - National Institute of Virology	Indian Council of Medical Research-National Institute of Virology, Microbial Containment Complex	Pragya D. Yadav. Savita Patil, Varsha Potdar, Prasad Sarkale, Dimpal A. Nyayanit, Gajanan Sapkal, Anita M. Shete, Atanu Basu, Lalit Dar, M Choudhary, Amita Jain, Bharati Malhotra, Pranita Gawande, Sarah Cherian, Priya Abraham
EPI_ISL_420548	hCoV-19/India/2020772/2020	Asia / India	2020	Indian Council of Medical Research-National Institute of Virology, Microbial Containment Complex	Indian Council of Medical Research-National Institute of Virology, Microbial Containment Complex	Pragya D. Yadav. Savita Patil, Varsha Potdar, Prasad Sarkale, Dimpal A. Nyayanit, Gajanan Sapkal, Anita M. Shete, Atanu Basu, Lalit Dar, M Choudhary, Amita Jain, Bharati Malhotra, Pranita Gawande, Sarah Cherian, Priya Abraham
EPI_ISL_420549	hCoV-19/India/773/2020	Asia / India	2020-03-03	National Influenza Center, Indian Council of Medical Research - National Institute of Virology	Indian Council of Medical Research-National Institute of Virology, Microbial Containment Complex	Pragya D. Yadav. Savita Patil, Varsha Potdar, Prasad Sarkale, Dimpal A. Nyayanit, Gajanan Sapkal, Anita M. Shete, Atanu Basu, Lalit Dar, M Choudhary, Amita Jain, Bharati Malhotra, Pranita Gawande, Sarah Cherian, Priya Abraham
EPI_ISL_420550	hCoV-19/India/2020773/2020	Asia / India	2020	Indian Council of Medical Research-National Institute of Virology, Microbial Containment Complex	Indian Council of Medical Research-National Institute of Virology, Microbial Containment Complex	Pragya D. Yadav. Savita Patil, Varsha Potdar, Prasad Sarkale, Dimpal A. Nyayanit, Gajanan Sapkal, Anita M. Shete, Atanu Basu, Lalit Dar, M Choudhary, Amita Jain, Bharati Malhotra, Pranita Gawande, Sarah Cherian, Priya Abraham
EPI_ISL_420551	hCoV-19/India/777/2020	Asia / India	2020-03-03	National Influenza Center, Indian Council of Medical Research - National Institute of Virology	Indian Council of Medical Research-National Institute of Virology, Microbial Containment Complex	Pragya D. Yadav. Savita Patil, Varsha Potdar, Prasad Sarkale, Dimpal A. Nyayanit, Gajanan Sapkal, Anita M. Shete, Atanu Basu, Lalit Dar, M Choudhary, Amita Jain, Bharati Malhotra, Pranita Gawande, Sarah Cherian, Priya Abraham
EPI_ISL_420552	hCoV-19/India/2020777/2020	Asia / India	2020	Indian Council of Medical Research-National Institute of Virology, Microbial Containment Complex	Indian Council of Medical Research-National Institute of Virology, Microbial Containment Complex	Pragya D. Yadav. Savita Patil, Varsha Potdar, Prasad Sarkale, Dimpal A. Nyayanit, Gajanan Sapkal, Anita M. Shete, Atanu Basu, Lalit Dar, M Choudhary, Amita Jain, Bharati Malhotra, Pranita Gawande, Sarah Cherian, Priya Abraham
EPI_ISL_420553	hCoV-19/India/781/2020	Asia / India	2020-03-03	National Influenza Center, Indian Council of Medical Research - National Institute of Virology	Indian Council of Medical Research-National Institute of Virology, Microbial Containment Complex	Pragya D. Yadav. Savita Patil, Varsha Potdar, Prasad

Sarkale, Dimpal A. Nyayanit, Gajanan Sapkal, Anita M. Shete, Atanu Basu, Lalit Dar, M Choudhary, Amita Jain, Bharati Malhotra, Pranita Gawande, Sarah Cherian, Priya Abraham
EPI_ISL_420554 hCoV-19/India/2020781/2020 Asia / India 2020 Indian Council of Medical Research-National Institute of Virology, Microbial Containment Complex Indian Council of Medical Research-National Institute of Virology, Microbial Containment Complex Pragya D. Yadav. Savita Patil, Varsha Potdar, Prasad Sarkale, Dimpal A. Nyayanit, Gajanan Sapkal, Anita M. Shete, Atanu Basu, Lalit Dar, M Choudhary, Amita Jain, Bharati Malhotra, Pranita Gawande, Sarah Cherian, Priya Abraham
EPI_ISL_420555 hCoV-19/India/c32/2020 Asia / India 2020-03-03 National Influenza Center, Indian Council of Medical Research - National Institute of Virology Indian Council of Medical Research-National Institute of Virology, Microbial Containment Complex Pragya D. Yadav. Savita Patil, Varsha Potdar, Prasad Sarkale, Dimpal A. Nyayanit, Gajanan Sapkal, Anita M. Shete, Atanu Basu, Lalit Dar, M Choudhary, Amita Jain, Bharati Malhotra, Pranita Gawande, Sarah Cherian, Priya Abraham
EPI_ISL_420556 hCoV-19/India/2020c32/2020 Asia / India 2020 Indian Council of Medical Research-National Institute of Virology, Microbial Containment Complex Indian Council of Medical Research-National Institute of Virology, Microbial Containment Complex Pragya D. Yadav. Savita Patil, Varsha Potdar, Prasad Sarkale, Dimpal A. Nyayanit, Gajanan Sapkal, Anita M. Shete, Atanu Basu, Lalit Dar, M Choudhary, Amita Jain, Bharati Malhotra, Pranita Gawande, Sarah Cherian, Priya Abraham
EPI_ISL_420563 hCoV-19/Italy/TE5166/2020 Europe / Italy / Teramo 2020-03-18 Ospedale Civile Giuseppe Mazzini "Istituto Zooprofilattico Sperimentale dell'Abruzzo e Molise ""G. Caporale""" Lorusso A, Marcacci M, Di Domenico M, Ancora M, Curini V, Mangone I, Rinaldi A, Di Pasquale A, Cammà C, Puglia I, Savini G
EPI_ISL_420564 hCoV-19/Italy/TE5472/2020 Europe / Italy / Castel di Sangro 2020-03-19 Ospedale Civile Castel Di Sangro Istituto Zooprofilattico Sperimentale dell’Abruzzo e Molise “G. Caporale” Lorusso A, Marcacci M, Di Domenico M, Ancora M, Curini V, Mangone I, Rinaldi A, Di Pasquale A, Cammà C, Puglia I, Savini G
EPI_ISL_420565 hCoV-19/Italy/TE5476/2020 Europe / Italy / Abruzzo / Teramo 2020-03-19 Ospedale Civile Giuseppe Mazzini Istituto Zooprofilattico Sperimentale dell’Abruzzo e Molise “G. Caporale” Lorusso A, Marcacci M, Di Domenico M, Ancora M, Curini V, Mangone I, Rinaldi A, Di Pasquale A, Cammà C, Puglia I, Savini G
EPI_ISL_420566 hCoV-19/Italy/TE5512/2020 Europe / Italy / Abruzzo 2020-03-19 Ospedale Regionale San Salvatore Istituto Zooprofilattico Sperimentale dell’Abruzzo e Molise “G. Caporale” Lorusso A, Marcacci M, Di Domenico M, Ancora M, Curini V, Mangone I, Rinaldi A, Di Pasquale A, Cammà C, Puglia I, Savini G
EPI_ISL_420567 hCoV-19/Italy/TE5780/2020 Europe / Italy / Abruzzo / L'Aquila 2020-03-21 Ospedale Regionale San Salvatore Istituto Zooprofilattico Sperimentale dell’Abruzzo e Molise “G. Caporale” Lorusso A, Marcacci M, Di Domenico M, Ancora M, Curini V, Mangone I, Rinaldi A, Di Pasquale A, Cammà C, Puglia I, Savini G
EPI_ISL_420568 hCoV-19/Italy/6193/2020 Europe / Italy / Abruzzo 2020-03-23 Ospedale Civile Giuseppe Mazzini Istituto Zooprofilattico Sperimentale dell’Abruzzo e Molise “G. Caporale” Lorusso A, Marcacci M, Di Domenico M, Ancora M, Curini V, Mangone I, Rinaldi A, Di Pasquale A, Cammà C, Puglia I, Savini G
EPI_ISL_420569 hCoV-19/Italy/TE6195/2020 Europe / Italy / Abruzzo 2020-03-23 Ospedale Civile Giuseppe Mazzini Istituto Zooprofilattico Sperimentale dell’Abruzzo e Molise “G. Caporale” Lorusso A, Marcacci M, Di Domenico M, Ancora M, Curini V, Mangone I, Rinaldi A, Di Pasquale A, Cammà C, Puglia I, Savini G
EPI_ISL_420570 hCoV-19/USA/NY-NYUMC55/2020 North America / USA / New York / Nassau County 2020-03-17 NYU Langone Health Departments of Pathology and Medicine, New York University School of Medicine Maria Aguero-Rosenfeld, Brendan Belovarac, Margaret Black, Ludovic Boytard, John Cadley, Paolo Cotzia, John Chen, Dacia Dimartino, Xiaojun Feng, Tatyana Gindin, Adriana Heguy, Megan Hogan, Emily Huang, George Jour, Andrew Lytle, Christian Marier, Matthew T. Maurano, Mark J. Mulligan, Peter Meyn, Iman Osman, Jared Pinnell, Sitharam Ramaswami, Amy Rapkiewicz, Marie Samanovic-Golden, Antonio Serrano, Guomiao Shen, Matija Snuderl, Theodore Vougiouklakis, Nick Vulpescu, Gael Westby, Paul Zappile, Yutong Zhang
EPI_ISL_420571 hCoV-19/USA/NY-NYUMC56/2020 North America / USA / New York / Nassau County 2020-03-18 NYU Langone Health Departments of Pathology and Medicine, New York University School of Medicine Maria Aguero-Rosenfeld, Brendan Belovarac, Margaret Black, Ludovic Boytard, John Cadley, Paolo Cotzia, John Chen, Dacia Dimartino, Xiaojun Feng, Tatyana Gindin, Adriana Heguy, Megan Hogan, Emily Huang, George Jour, Andrew Lytle, Christian Marier, Matthew T. Maurano, Mark J. Mulligan, Peter Meyn, Iman Osman, Jared Pinnell, Sitharam Ramaswami, Amy Rapkiewicz, Marie Samanovic-Golden, Antonio Serrano, Guomiao Shen, Matija Snuderl, Theodore Vougiouklakis, Nick Vulpescu, Gael Westby, Paul Zappile, Yutong Zhang
EPI_ISL_420572 hCoV-19/USA/NY-NYUMC57/2020 North America / USA / New York / Nassau County 2020-03-17 NYU Langone Health Departments of Pathology and Medicine, New York University School of Medicine Maria Aguero-Rosenfeld, Brendan Belovarac, Margaret Black, Ludovic Boytard, John Cadley, Paolo Cotzia, John Chen, Dacia Dimartino, Xiaojun Feng, Tatyana Gindin, Adriana Heguy, Megan Hogan, Emily Huang, George Jour, Andrew Lytle, Christian Marier, Matthew T. Maurano, Mark J. Mulligan, Peter Meyn, Iman Osman, Jared Pinnell, Sitharam Ramaswami, Amy Rapkiewicz, Marie Samanovic-Golden, Antonio Serrano, Guomiao Shen, Matija Snuderl, Theodore Vougiouklakis, Nick Vulpescu, Gael Westby, Paul Zappile, Yutong Zhang
EPI_ISL_420573 hCoV-19/USA/NY-NYUMC58/2020 North America / USA / New York / Manhattan 2020-03-18 NYU Langone Health Departments of Pathology and Medicine, New York University School of Medicine Maria Aguero-Rosenfeld, Brendan Belovarac, Margaret Black, Ludovic Boytard, John Cadley, Paolo Cotzia, John Chen, Dacia Dimartino, Xiaojun Feng, Tatyana Gindin, Adriana Heguy, Megan Hogan, Emily Huang, George Jour, Andrew

Lytle, Christian Marier, Matthew T. Maurano, Mark J. Mulligan, Peter Meyn, Iman Osman, Jared Pinnell, Sitharam Ramaswami, Amy Rapkiewicz, Marie Samanovic-Golden, Antonio Serrano, Guomiao Shen, Matija Snuderl, Theodore Vougiouklakis, Nick Vulpescu, Gael Westby, Paul Zappile, Yutong Zhang
EPI_ISL_420575 hCoV-19/USA/NY-NYUMC60/2020 North America / USA / New York / Nassau County 2020-03-18 NYU Langone Health Departments of Pathology and Medicine, New York University School of Medicine Maria Aguero-Rosenfeld, Brendan Belovarac, Margaret Black, Ludovic Boytard, John Cadley, Paolo Cotzia, John Chen, Dacia Dimartino, Xiaojun Feng, Tatyana Gindin, Adriana Heguy, Megan Hogan, Emily Huang, George Jour, Andrew Lytle, Christian Marier, Matthew T. Maurano, Mark J. Mulligan, Peter Meyn, Iman Osman, Jared Pinnell, Sitharam Ramaswami, Amy Rapkiewicz, Marie Samanovic-Golden, Antonio Serrano, Guomiao Shen, Matija Snuderl, Theodore Vougiouklakis, Nick Vulpescu, Gael Westby, Paul Zappile, Yutong Zhang
EPI_ISL_420576 hCoV-19/USA/NY-NYUMC61/2020 North America / USA / New York 2020-03-18 NYU Langone Health Departments of Pathology and Medicine, New York University School of Medicine Maria Aguero-Rosenfeld, Brendan Belovarac, Margaret Black, Ludovic Boytard, John Cadley, Paolo Cotzia, John Chen, Dacia Dimartino, Xiaojun Feng, Tatyana Gindin, Adriana Heguy, Megan Hogan, Emily Huang, George Jour, Andrew Lytle, Christian Marier, Matthew T. Maurano, Mark J. Mulligan, Peter Meyn, Iman Osman, Jared Pinnell, Sitharam Ramaswami, Amy Rapkiewicz, Marie Samanovic-Golden, Antonio Serrano, Guomiao Shen, Matija Snuderl, Theodore Vougiouklakis, Nick Vulpescu, Gael Westby, Paul Zappile, Yutong Zhang
EPI_ISL_420577 hCoV-19/USA/NY-NYUMC62/2020 North America / USA / New York / Manhattan 2020-03-18 NYU Langone Health Departments of Pathology and Medicine, New York University School of Medicine Maria Aguero-Rosenfeld, Brendan Belovarac, Margaret Black, Ludovic Boytard, John Cadley, Paolo Cotzia, John Chen, Dacia Dimartino, Xiaojun Feng, Tatyana Gindin, Adriana Heguy, Megan Hogan, Emily Huang, George Jour, Andrew Lytle, Christian Marier, Matthew T. Maurano, Mark J. Mulligan, Peter Meyn, Iman Osman, Jared Pinnell, Sitharam Ramaswami, Amy Rapkiewicz, Marie Samanovic-Golden, Antonio Serrano, Guomiao Shen, Matija Snuderl, Theodore Vougiouklakis, Nick Vulpescu, Gael Westby, Paul Zappile, Yutong Zhang
EPI_ISL_420578 hCoV-19/USA/NY-NYUMC63/2020 North America / USA / New York / Manhattan 2020-03-18 NYU Langone Health Departments of Pathology and Medicine, New York University School of Medicine Maria Aguero-Rosenfeld, Brendan Belovarac, Margaret Black, Ludovic Boytard, John Cadley, Paolo Cotzia, John Chen, Dacia Dimartino, Xiaojun Feng, Tatyana Gindin, Adriana Heguy, Megan Hogan, Emily Huang, George Jour, Andrew Lytle, Christian Marier, Matthew T. Maurano, Mark J. Mulligan, Peter Meyn, Iman Osman, Jared Pinnell, Sitharam Ramaswami, Amy Rapkiewicz, Marie Samanovic-Golden, Antonio Serrano, Guomiao Shen, Matija Snuderl, Theodore Vougiouklakis, Nick Vulpescu, Gael Westby, Paul Zappile, Yutong Zhang
EPI_ISL_420579 hCoV-19/USA/NY-NYUMC64/2020 North America / USA / New York / Manhattan 2020-03-18 NYU Langone Health Departments of Pathology and Medicine, New York University School of Medicine Maria Aguero-Rosenfeld, Brendan Belovarac, Margaret Black, Ludovic Boytard, John Cadley, Paolo Cotzia, John Chen, Dacia Dimartino, Xiaojun Feng, Tatyana Gindin, Adriana Heguy, Megan Hogan, Emily Huang, George Jour, Andrew Lytle, Christian Marier, Matthew T. Maurano, Mark J. Mulligan, Peter Meyn, Iman Osman, Jared Pinnell, Sitharam Ramaswami, Amy Rapkiewicz, Marie Samanovic-Golden, Antonio Serrano, Guomiao Shen, Matija Snuderl, Theodore Vougiouklakis, Nick Vulpescu, Gael Westby, Paul Zappile, Yutong Zhang
EPI_ISL_420580 hCoV-19/USA/NY-NYUMC65/2020 North America / USA / New York / Manhattan 2020-03-18 NYU Langone Health Departments of Pathology and Medicine, New York University School of Medicine Maria Aguero-Rosenfeld, Brendan Belovarac, Margaret Black, Ludovic Boytard, John Cadley, Paolo Cotzia, John Chen, Dacia Dimartino, Xiaojun Feng, Tatyana Gindin, Adriana Heguy, Megan Hogan, Emily Huang, George Jour, Andrew Lytle, Christian Marier, Matthew T. Maurano, Mark J. Mulligan, Peter Meyn, Iman Osman, Jared Pinnell, Sitharam Ramaswami, Amy Rapkiewicz, Marie Samanovic-Golden, Antonio Serrano, Guomiao Shen, Matija Snuderl, Theodore Vougiouklakis, Nick Vulpescu, Gael Westby, Paul Zappile, Yutong Zhang
EPI_ISL_420581 hCoV-19/USA/NY-NYUMC66/2020 North America / USA / New York / Manhattan 2020-03-18 NYU Langone Health Departments of Pathology and Medicine, New York University School of Medicine Maria Aguero-Rosenfeld, Brendan Belovarac, Margaret Black, Ludovic Boytard, John Cadley, Paolo Cotzia, John Chen, Dacia Dimartino, Xiaojun Feng, Tatyana Gindin, Adriana Heguy, Megan Hogan, Emily Huang, George Jour, Andrew Lytle, Christian Marier, Matthew T. Maurano, Mark J. Mulligan, Peter Meyn, Iman Osman, Jared Pinnell, Sitharam Ramaswami, Amy Rapkiewicz, Marie Samanovic-Golden, Antonio Serrano, Guomiao Shen, Matija Snuderl, Theodore Vougiouklakis, Nick Vulpescu, Gael Westby, Paul Zappile, Yutong Zhang
EPI_ISL_420583 hCoV-19/Italy/TE6222/2020 Europe / Italy / Abruzzo 2020-03-23 Ospedale Civile Giuseppe Mazzini Istituto Zooprofilattico Sperimentale dell'Abruzzo e Molise "G. Caporale" Lorusso A, Marcacci M, Di Domenico M, Ancora M, Curini V, Mangone I, Rinaldi A, Di Pasquale A, Cammà C, Puglia I, Savini G
EPI_ISL_420585 hCoV-19/USA/NY-NYUMC69/2020 North America / USA / New York / Manhattan 2020-03-18 NYU Langone Health Departments of Pathology and Medicine, New York University School of Medicine Maria Aguero-Rosenfeld, Brendan Belovarac, Margaret Black, Ludovic Boytard, John Cadley, Paolo Cotzia, John Chen, Dacia Dimartino, Xiaojun Feng, Tatyana Gindin, Adriana Heguy, Megan Hogan, Emily Huang, George Jour, Andrew Lytle, Christian Marier, Matthew T. Maurano, Mark J. Mulligan, Peter Meyn, Iman Osman, Jared Pinnell, Sitharam Ramaswami, Amy Rapkiewicz, Marie Samanovic-Golden, Antonio Serrano, Guomiao Shen, Matija Snuderl, Theodore Vougiouklakis, Nick Vulpescu, Gael Westby, Paul Zappile, Yutong Zhang
EPI_ISL_420586 hCoV-19/USA/NY-NYUMC70/2020 North America / USA / New York / Brooklyn 2020-03-19 NYU Langone Health Departments of Pathology and Medicine, New York University School of Medicine Maria Aguero-Rosenfeld, Brendan Belovarac, Margaret Black, Ludovic Boytard, John Cadley, Paolo Cotzia, John Chen, Dacia Dimartino, Xiaojun Feng, Tatyana Gindin, Adriana Heguy, Megan Hogan, Emily Huang, George Jour, Andrew Lytle, Christian Marier, Matthew T. Maurano, Mark J. Mulligan, Peter Meyn, Iman Osman, Jared Pinnell, Sitharam Ramaswami, Amy Rapkiewicz, Marie Samanovic-Golden, Antonio Serrano, Guomiao Shen, Matija Snuderl, Theodore Vougiouklakis, Nick Vulpescu, Gael Westby, Paul Zappile, Yutong Zhang

EPI_ISL_420587 hCoV-19/USA/NY-NYUMC71/2020 North America / USA / New York / Manhattan 2020-03-18 NYU Langone Health Departments of Pathology and Medicine, New York University School of Medicine Maria Aguero-Rosenfeld, Brendan Belovarac, Margaret Black, Ludovic Boytard, John Cadley, Paolo Cotzia, John Chen, Dacia Dimartino, Xiaojun Feng, Tatyana Gindin, Adriana Heguy, Megan Hogan, Emily Huang, George Jour, Andrew Lytle, Christian Marier, Matthew T. Maurano, Mark J. Mulligan, Peter Meyn, Iman Osman, Jared Pinnell, Sitharam Ramaswami, Amy Rapkiewicz, Marie Samanovic-Golden, Antonio Serrano, Guomiao Shen, Matija Snuderl, Theodore Vougiouklakis, Nick Vulpescu, Gael Westby, Paul Zappile, Yutong Zhang
EPI_ISL_420588 hCoV-19/USA/NY-NYUMC72/2020 North America / USA / New York / Manhattan 2020-03-18 NYU Langone Health Departments of Pathology and Medicine, New York University School of Medicine Maria Aguero-Rosenfeld, Brendan Belovarac, Margaret Black, Ludovic Boytard, John Cadley, Paolo Cotzia, John Chen, Dacia Dimartino, Xiaojun Feng, Tatyana Gindin, Adriana Heguy, Megan Hogan, Emily Huang, George Jour, Andrew Lytle, Christian Marier, Matthew T. Maurano, Mark J. Mulligan, Peter Meyn, Iman Osman, Jared Pinnell, Sitharam Ramaswami, Amy Rapkiewicz, Marie Samanovic-Golden, Antonio Serrano, Guomiao Shen, Matija Snuderl, Theodore Vougiouklakis, Nick Vulpescu, Gael Westby, Paul Zappile, Yutong Zhang
EPI_ISL_420589 hCoV-19/USA/NY-NYUMC73/2020 North America / USA / New York / Manhattan 2020-03-18 NYU Langone Health Departments of Pathology and Medicine, New York University School of Medicine Maria Aguero-Rosenfeld, Brendan Belovarac, Margaret Black, Ludovic Boytard, John Cadley, Paolo Cotzia, John Chen, Dacia Dimartino, Xiaojun Feng, Tatyana Gindin, Adriana Heguy, Megan Hogan, Emily Huang, George Jour, Andrew Lytle, Christian Marier, Matthew T. Maurano, Mark J. Mulligan, Peter Meyn, Iman Osman, Jared Pinnell, Sitharam Ramaswami, Amy Rapkiewicz, Marie Samanovic-Golden, Antonio Serrano, Guomiao Shen, Matija Snuderl, Theodore Vougiouklakis, Nick Vulpescu, Gael Westby, Paul Zappile, Yutong Zhang
EPI_ISL_420590 hCoV-19/USA/NY-NYUMC74/2020 North America / USA / New York / Nassau County 2020-03-18 NYU Langone Health Departments of Pathology and Medicine, New York University School of Medicine Maria Aguero-Rosenfeld, Brendan Belovarac, Margaret Black, Ludovic Boytard, John Cadley, Paolo Cotzia, John Chen, Dacia Dimartino, Xiaojun Feng, Tatyana Gindin, Adriana Heguy, Megan Hogan, Emily Huang, George Jour, Andrew Lytle, Christian Marier, Matthew T. Maurano, Mark J. Mulligan, Peter Meyn, Iman Osman, Jared Pinnell, Sitharam Ramaswami, Amy Rapkiewicz, Marie Samanovic-Golden, Antonio Serrano, Guomiao Shen, Matija Snuderl, Theodore Vougiouklakis, Nick Vulpescu, Gael Westby, Paul Zappile, Yutong Zhang
EPI_ISL_420591 hCoV-19/USA/NY-NYUMC75/2020 North America / USA / New York / Manhattan 2020-03-18 NYU Langone Health Departments of Pathology and Medicine, New York University School of Medicine Maria Aguero-Rosenfeld, Brendan Belovarac, Margaret Black, Ludovic Boytard, John Cadley, Paolo Cotzia, John Chen, Dacia Dimartino, Xiaojun Feng, Tatyana Gindin, Adriana Heguy, Megan Hogan, Emily Huang, George Jour, Andrew Lytle, Christian Marier, Matthew T. Maurano, Mark J. Mulligan, Peter Meyn, Iman Osman, Jared Pinnell, Sitharam Ramaswami, Amy Rapkiewicz, Marie Samanovic-Golden, Antonio Serrano, Guomiao Shen, Matija Snuderl, Theodore Vougiouklakis, Nick Vulpescu, Gael Westby, Paul Zappile, Yutong Zhang
EPI_ISL_420598 hCoV-19/Argentina/C3013/2020 South America / Argentina 2020-03-22 Servicio Virosis Respiratorias-Departamento Virología-INEI Instituto Nacional Enfermedades Infecciosas C.G.Malbran Baumeister E., Avaro M., Benedetti E., Russo M., Dattero ME, Pontoriero A., Cisterna D., Molina V., Perandones C., Tuduri E., Lorenzo F., Poklepovich T., Campos J.
EPI_ISL_420599 hCoV-19/Argentina/C1374/2020 South America / Argentina 2020-03-18 Servicio Virosis Respiratorias-Departamento Virología-INEI Instituto Nacional Enfermedades Infecciosas C.G.Malbran Baumeister E., Avaro M., Benedetti E., Russo M., Dattero ME, Pontoriero A., Cisterna D., Molina V., Perandones C., Tuduri E., Lorenzo F., Poklepovich T., Campos J.
EPI_ISL_420800 hCoV-19/Korea/BA-ACH_2718/2020 Asia / Korea 2020-02-29 Brian D. Allgood Army Community Hospital Pathogen Discovery, Respiratory Viruses Branch, Division of Viral Diseases, Centers for Disease Control and Prevention Krista Queen, Yan Li, Ying Tao, Jing Zhang, Anne Uehara, Clinton R. Paden, Haibin Wang, Rachel Marine, Mary S. Keckler, Alison S. Laufer Halpin, Jasmine Padilla, Justin Lee, Christopher A. Elkins, Suxiang Tong
EPI_ISL_420801 hCoV-19/Korea/BA-ACH_2719/2020 Asia / Korea 2020-02-29 Brian D. Allgood Army Community Hospital Pathogen Discovery, Respiratory Viruses Branch, Division of Viral Diseases, Centers for Disease Control and Prevention Krista Queen, Yan Li, Ying Tao, Jing Zhang, Anne Uehara, Clinton R. Paden, Haibin Wang, Rachel Marine, Mary S. Keckler, Alison S. Laufer Halpin, Jasmine Padilla, Justin Lee, Christopher A. Elkins, Suxiang Tong
EPI_ISL_420803 hCoV-19/USA/UT-089/2020 North America / USA / Utah 2020-03-12 Utah Public Health Laboratory Utah Public Health Laboratory Erin Young, Kelly Oakeson
EPI_ISL_420810 hCoV-19/USA/UT-0290/2020 North America / USA / Utah 2020-03-27 Utah Public Health Laboratory Utah Public Health Laboratory Erin Young, Kelly Oakeson
EPI_ISL_420812 hCoV-19/USA/UT-0297/2020 North America / USA / Utah 2020-03-27 Utah Public Health Laboratory Utah Public Health Laboratory Erin Young, Kelly Oakeson
EPI_ISL_420814 hCoV-19/USA/UT-0301/2020 North America / USA / Utah 2020-03-27 Utah Public Health Laboratory Utah Public Health Laboratory Erin Young, Kelly Oakeson
EPI_ISL_420838 hCoV-19/DRC/253/2020 Africa / Democratic Republic of the Congo / Kinshasa 2020-03-22 Viral Respiratory Lab, National Institute for Biomedical Research (INRB) Pathogen Sequencing Lab, National Institute for Biomedical Research (INRB) Placide Mbala-Kingebeni, Edith Nkwembe, Eddy Kinganda-Lusamaki, Amuri Aziza, Catherine Pratt, Matthias Pauthner, Josh Quick, Allison Black, James Hadfield, Trevor Bedford, Ian Goodfellow, Nick Loman, Kristian Andersen, Michael Wiley, Steve Ahuka-Mundeke, Jean-Jacques Muyembe Tamfum
EPI_ISL_420841 hCoV-19/DRC/300/2020 Africa / Democratic Republic of the Congo / Sud Kivu 2020-03-22 Viral Respiratory Lab, National Institute for Biomedical Research (INRB) Pathogen Sequencing Lab, National Institute for Biomedical Research (INRB) Placide Mbala-Kingebeni, Edith Nkwembe, Eddy Kinganda-

Lusamaki, Amuri Aziza, Catherine Pratt, Matthias Pauthner, Josh Quick, Allison Black, James Hadfield, Trevor Bedford, Ian Goodfellow, Nick Loman, Kristian Andersen, Michael Wiley, Steve Ahuka-Mundeke, Jean-Jacques Muyembe Tamfum
EPI_ISL_420844 hCoV-19/DRC/355/2020 Africa / Democratic Republic of the Congo / Kinshasa 2020-03 Viral Respiratory Lab, National Institute for Biomedical Research (INRB) Pathogen Sequencing Lab, National Institute for Biomedical Research (INRB) Placide Mbala-Kingebeni, Edith Nkwembe, Eddy Kinganda-Lusamaki, Amuri Aziza, Catherine Pratt, Matthias Pauthner, Josh Quick, Allison Black, James Hadfield, Trevor Bedford, Ian Goodfellow, Nick Loman, Kristian Andersen, Michael Wiley, Steve Ahuka-Mundeke, Jean-Jacques Muyembe Tamfum
EPI_ISL_420845 hCoV-19/DRC/397/2020 Africa / Democratic Republic of the Congo / Kinshasa 2020-03-26 Viral Respiratory Lab, National Institute for Biomedical Research (INRB) Pathogen Sequencing Lab, National Institute for Biomedical Research (INRB) Placide Mbala-Kingebeni, Edith Nkwembe, Eddy Kinganda-Lusamaki, Amuri Aziza, Catherine Pratt, Matthias Pauthner, Josh Quick, Allison Black, James Hadfield, Trevor Bedford, Ian Goodfellow, Nick Loman, Kristian Andersen, Michael Wiley, Steve Ahuka-Mundeke, Jean-Jacques Muyembe Tamfum
EPI_ISL_420846 hCoV-19/DRC/400/2020 Africa / Democratic Republic of the Congo / Kinshasa 2020-03 Viral Respiratory Lab, National Institute for Biomedical Research (INRB) Pathogen Sequencing Lab, National Institute for Biomedical Research (INRB) Placide Mbala-Kingebeni, Edith Nkwembe, Eddy Kinganda-Lusamaki, Amuri Aziza, Catherine Pratt, Matthias Pauthner, Josh Quick, Allison Black, James Hadfield, Trevor Bedford, Ian Goodfellow, Nick Loman, Kristian Andersen, Michael Wiley, Steve Ahuka-Mundeke, Jean-Jacques Muyembe Tamfum
EPI_ISL_420847 hCoV-19/DRC/431/2020 Africa / Democratic Republic of the Congo 2020-03-26 Viral Respiratory Lab, National Institute for Biomedical Research (INRB) Pathogen Sequencing Lab, National Institute for Biomedical Research (INRB) Placide Mbala-Kingebeni, Edith Nkwembe, Eddy Kinganda-Lusamaki, Amuri Aziza, Catherine Pratt, Matthias Pauthner, Josh Quick, Allison Black, James Hadfield, Trevor Bedford, Ian Goodfellow, Nick Loman, Kristian Andersen, Michael Wiley, Steve Ahuka-Mundeke, Jean-Jacques Muyembe Tamfum
EPI_ISL_420849 hCoV-19/DRC/523/2020 Africa / Democratic Republic of the Congo / Kinshasa 2020-03-28 Viral Respiratory Lab, National Institute for Biomedical Research (INRB) Pathogen Sequencing Lab, National Institute for Biomedical Research (INRB) Placide Mbala-Kingebeni, Edith Nkwembe, Eddy Kinganda-Lusamaki, Amuri Aziza, Catherine Pratt, Matthias Pauthner, Josh Quick, Allison Black, James Hadfield, Trevor Bedford, Ian Goodfellow, Nick Loman, Kristian Andersen, Michael Wiley, Steve Ahuka-Mundeke, Jean-Jacques Muyembe Tamfum
EPI_ISL_420851 hCoV-19/DRC/376/2020 Africa / Democratic Republic of the Congo / Kinshasa 2020-03-25 Viral Respiratory Lab, National Institute for Biomedical Research (INRB) Pathogen Sequencing Lab, National Institute for Biomedical Research (INRB) Placide Mbala-Kingebeni, Edith Nkwembe, Eddy Kinganda-Lusamaki, Amuri Aziza, Catherine Pratt, Matthias Pauthner, Josh Quick, Allison Black, James Hadfield, Trevor Bedford, Ian Goodfellow, Nick Loman, Kristian Andersen, Michael Wiley, Steve Ahuka-Mundeke, Jean-Jacques Muyembe Tamfum
EPI_ISL_420852 hCoV-19/DRC/396/2020 Africa / Democratic Republic of the Congo / Kinshasa 2020-03-26 Viral Respiratory Lab, National Institute for Biomedical Research (INRB) Pathogen Sequencing Lab, National Institute for Biomedical Research (INRB) Placide Mbala-Kingebeni, Edith Nkwembe, Eddy Kinganda-Lusamaki, Amuri Aziza, Catherine Pratt, Matthias Pauthner, Josh Quick, Allison Black, James Hadfield, Trevor Bedford, Ian Goodfellow, Nick Loman, Kristian Andersen, Michael Wiley, Steve Ahuka-Mundeke, Jean-Jacques Muyembe Tamfum
EPI_ISL_420854 hCoV-19/DRC/521/2020 Africa / Democratic Republic of the Congo / Kinshasa 2020-03-25 Viral Respiratory Lab, National Institute for Biomedical Research (INRB) Pathogen Sequencing Lab, National Institute for Biomedical Research (INRB) Placide Mbala-Kingebeni, Edith Nkwembe, Eddy Kinganda-Lusamaki, Amuri Aziza, Catherine Pratt, Matthias Pauthner, Josh Quick, Allison Black, James Hadfield, Trevor Bedford, Ian Goodfellow, Nick Loman, Kristian Andersen, Michael Wiley, Steve Ahuka-Mundeke, Jean-Jacques Muyembe Tamfum
EPI_ISL_420855 hCoV-19/Australia/VIC-CBA1/2020 Oceania / Australia / Victoria 2020-03-23 Geelong Centre for Emerging Infectious Diseases Geelong Centre for Emerging Infectious Diseases Chamings,A., Raj Bhatta T., Alexandersen S.
EPI_ISL_420876 hCoV-19/Australia/VIC-CBA2/2020 Oceania / Australia / Victoria 2020-03-24 Geelong Centre for Emerging Infectious Diseases Geelong Centre for Emerging Infectious Diseases Chamings,A., Raj Bhatta T., Alexandersen S.
EPI_ISL_420877 hCoV-19/Australia/VIC-CBA3/2020 Oceania / Australia / Victoria 2020-03-28 Geelong Centre for Emerging Infectious Diseases Geelong Centre for Emerging Infectious Diseases Chamings,A., Raj Bhatta T., Alexandersen S.
EPI_ISL_420878 hCoV-19/Australia/QLDID929/2020 Oceania / Australia / Queensland / Brisbane 2020-03-24 Mater Pathology Public Health Virology Laboratory Bixing Huang, Alyssa Pyke, Amanda De Jong, Andrew Van Den Hurk, Carmel Taylor, David Warrilow, Doris Genge, Elisabeth Gamez, Glen Hewitson, Ian Maxwell Mackay, Inga Sultana, Jamie McMahon, Jean Barcelon, Judy Northill, Mitchell Finger, Natalie Simpson, Neelima Nair, Peter Burtonclay, Peter Moore, Sarah Wheatley, Sean Moody, Sonja Hall-Mendelin, Timothy Gardam, and Frederick Moore
EPI_ISL_420879 hCoV-19/Australia/QLDID931/2020 Oceania / Australia / Queensland / Brisbane 2020-03-25 Mater Pathology Public Health Virology Laboratory Bixing Huang, Alyssa Pyke, Amanda De Jong, Andrew Van Den Hurk, Carmel Taylor, David Warrilow, Doris Genge, Elisabeth Gamez, Glen Hewitson, Ian Maxwell Mackay, Inga Sultana, Jamie McMahon, Jean Barcelon, Judy Northill, Mitchell Finger, Natalie Simpson, Neelima Nair, Peter Burtonclay, Peter Moore, Sarah Wheatley, Sean Moody, Sonja Hall-Mendelin, Timothy Gardam, and Frederick Moore
EPI_ISL_420889 hCoV-19/Japan/Hu_DP_Kng_19-031/2020 Asia / Japan 2020-02-14 unknown Takayuki Hishiki Kanagawa Prefectural Institute of Public Health Hishiki,T., Suzuki,R., Sakuragi,J., Usui,K., Tanaka,Y., Kawai,J., Kogo,Y., Matsuki,Y., An,T., Hayashizaki,Y. and Takasaki,T.

EPI_ISL_420890 hCoV-19/Japan/Hu_Kng_19-437/2020   Asia / Japan   2020-03-09   unknown Takayuki Hishiki Kanagawa Prefectural Institute of Public Health Hishiki,T., Suzuki,R., Sakuragi,J., Usui,K., Tanaka,Y., Kawai,J., Kogo,Y., Matsuki,Y., An,T., Hayashizaki,Y. and Takasaki,T.
EPI_ISL_421800 hCoV-19/England/201380036/2020 Europe / United Kingdom / England   2020-03-26   Respiratory Virus Unit, Microbiology Services Colindale, Public Health England Respiratory Virus Unit, Microbiology Services Colindale, Public Health England Monica Galiano, Shahjahan Miah, Angie Lackenby, Omolola Akinbami, Tiina Talts, Leena Bhaw, Richard Myers, Steven Platt, Kirstin Edwards, Jonathan Hubb, Joanna Ellis, Maria Zambon
EPI_ISL_421801 hCoV-19/England/201380037/2020 Europe / United Kingdom / England   2020-03-26   Respiratory Virus Unit, Microbiology Services Colindale, Public Health England Respiratory Virus Unit, Microbiology Services Colindale, Public Health England Monica Galiano, Shahjahan Miah, Angie Lackenby, Omolola Akinbami, Tiina Talts, Leena Bhaw, Richard Myers, Steven Platt, Kirstin Edwards, Jonathan Hubb, Joanna Ellis, Maria Zambon
EPI_ISL_421802 hCoV-19/England/201380040/2020 Europe / United Kingdom / England   2020-03-27   Respiratory Virus Unit, Microbiology Services Colindale, Public Health England Respiratory Virus Unit, Microbiology Services Colindale, Public Health England Monica Galiano, Shahjahan Miah, Angie Lackenby, Omolola Akinbami, Tiina Talts, Leena Bhaw, Richard Myers, Steven Platt, Kirstin Edwards, Jonathan Hubb, Joanna Ellis, Maria Zambon
EPI_ISL_421803 hCoV-19/England/201380041/2020 Europe / United Kingdom / England   2020-03-26   Respiratory Virus Unit, Microbiology Services Colindale, Public Health England Respiratory Virus Unit, Microbiology Services Colindale, Public Health England Monica Galiano, Shahjahan Miah, Angie Lackenby, Omolola Akinbami, Tiina Talts, Leena Bhaw, Richard Myers, Steven Platt, Kirstin Edwards, Jonathan Hubb, Joanna Ellis, Maria Zambon
EPI_ISL_421804 hCoV-19/England/201380042/2020 Europe / United Kingdom / England   2020-03-25   Respiratory Virus Unit, Microbiology Services Colindale, Public Health England Respiratory Virus Unit, Microbiology Services Colindale, Public Health England Monica Galiano, Shahjahan Miah, Angie Lackenby, Omolola Akinbami, Tiina Talts, Leena Bhaw, Richard Myers, Steven Platt, Kirstin Edwards, Jonathan Hubb, Joanna Ellis, Maria Zambon
EPI_ISL_421805 hCoV-19/England/201380044/2020 Europe / United Kingdom / England   2020-03-26   Respiratory Virus Unit, Microbiology Services Colindale, Public Health England Respiratory Virus Unit, Microbiology Services Colindale, Public Health England Monica Galiano, Shahjahan Miah, Angie Lackenby, Omolola Akinbami, Tiina Talts, Leena Bhaw, Richard Myers, Steven Platt, Kirstin Edwards, Jonathan Hubb, Joanna Ellis, Maria Zambon
EPI_ISL_421806 hCoV-19/England/201380049/2020 Europe / United Kingdom / England   2020-03-25   Respiratory Virus Unit, Microbiology Services Colindale, Public Health England Respiratory Virus Unit, Microbiology Services Colindale, Public Health England Monica Galiano, Shahjahan Miah, Angie Lackenby, Omolola Akinbami, Tiina Talts, Leena Bhaw, Richard Myers, Steven Platt, Kirstin Edwards, Jonathan Hubb, Joanna Ellis, Maria Zambon
EPI_ISL_421807 hCoV-19/England/201380050/2020 Europe / United Kingdom / England   2020-03-25   Respiratory Virus Unit, Microbiology Services Colindale, Public Health England Respiratory Virus Unit, Microbiology Services Colindale, Public Health England Monica Galiano, Shahjahan Miah, Angie Lackenby, Omolola Akinbami, Tiina Talts, Leena Bhaw, Richard Myers, Steven Platt, Kirstin Edwards, Jonathan Hubb, Joanna Ellis, Maria Zambon
EPI_ISL_421809 hCoV-19/England/201380054/2020 Europe / United Kingdom / England   2020-03-25   Respiratory Virus Unit, Microbiology Services Colindale, Public Health England Respiratory Virus Unit, Microbiology Services Colindale, Public Health England Monica Galiano, Shahjahan Miah, Angie Lackenby, Omolola Akinbami, Tiina Talts, Leena Bhaw, Richard Myers, Steven Platt, Kirstin Edwards, Jonathan Hubb, Joanna Ellis, Maria Zambon
EPI_ISL_421810 hCoV-19/England/201380055/2020 Europe / United Kingdom / England   2020-03-25   Respiratory Virus Unit, Microbiology Services Colindale, Public Health England Respiratory Virus Unit, Microbiology Services Colindale, Public Health England Monica Galiano, Shahjahan Miah, Angie Lackenby, Omolola Akinbami, Tiina Talts, Leena Bhaw, Richard Myers, Steven Platt, Kirstin Edwards, Jonathan Hubb, Joanna Ellis, Maria Zambon
EPI_ISL_421811 hCoV-19/England/201380056/2020 Europe / United Kingdom / England   2020-03-25   Respiratory Virus Unit, Microbiology Services Colindale, Public Health England Respiratory Virus Unit, Microbiology Services Colindale, Public Health England Monica Galiano, Shahjahan Miah, Angie Lackenby, Omolola Akinbami, Tiina Talts, Leena Bhaw, Richard Myers, Steven Platt, Kirstin Edwards, Jonathan Hubb, Joanna Ellis, Maria Zambon
EPI_ISL_421813 hCoV-19/England/201380061/2020 Europe / United Kingdom / England   2020-03-25   Respiratory Virus Unit, Microbiology Services Colindale, Public Health England Respiratory Virus Unit, Microbiology Services Colindale, Public Health England Monica Galiano, Shahjahan Miah, Angie Lackenby, Omolola Akinbami, Tiina Talts, Leena Bhaw, Richard Myers, Steven Platt, Kirstin Edwards, Jonathan Hubb, Joanna Ellis, Maria Zambon
EPI_ISL_421815 hCoV-19/England/201380063/2020 Europe / United Kingdom / England   2020-03-25   Respiratory Virus Unit, Microbiology Services Colindale, Public Health England Respiratory Virus Unit, Microbiology Services Colindale, Public Health England Monica Galiano, Shahjahan Miah, Angie Lackenby, Omolola Akinbami, Tiina Talts, Leena Bhaw, Richard Myers, Steven Platt, Kirstin Edwards, Jonathan Hubb, Joanna Ellis, Maria Zambon
EPI_ISL_421816 hCoV-19/England/201380064/2020 Europe / United Kingdom / England   2020-03-25   Respiratory Virus Unit, Microbiology Services Colindale, Public Health England Respiratory Virus Unit,

Microbiology Services Colindale, Public Health England Monica Galiano, Shahjahan Miah, Angie Lackenby, Omolola Akinbami, Tiina Talts, Leena Bhaw, Richard Myers, Steven Platt, Kirstin Edwards, Jonathan Hubb, Joanna Ellis, Maria Zambon
EPI_ISL_421817 hCoV-19/England/201380069/2020 Europe / United Kingdom / England 2020-03-25 Respiratory Virus Unit, Microbiology Services Colindale, Public Health England Respiratory Virus Unit, Microbiology Services Colindale, Public Health England Monica Galiano, Shahjahan Miah, Angie Lackenby, Omolola Akinbami, Tiina Talts, Leena Bhaw, Richard Myers, Steven Platt, Kirstin Edwards, Jonathan Hubb, Joanna Ellis, Maria Zambon
EPI_ISL_421818 hCoV-19/England/201380070/2020 Europe / United Kingdom / England 2020-03-25 Respiratory Virus Unit, Microbiology Services Colindale, Public Health England Respiratory Virus Unit, Microbiology Services Colindale, Public Health England Monica Galiano, Shahjahan Miah, Angie Lackenby, Omolola Akinbami, Tiina Talts, Leena Bhaw, Richard Myers, Steven Platt, Kirstin Edwards, Jonathan Hubb, Joanna Ellis, Maria Zambon
EPI_ISL_421822 hCoV-19/England/201380217/2020 Europe / United Kingdom / England 2020-03-25 Respiratory Virus Unit, Microbiology Services Colindale, Public Health England Respiratory Virus Unit, Microbiology Services Colindale, Public Health England Monica Galiano, Shahjahan Miah, Angie Lackenby, Omolola Akinbami, Tiina Talts, Leena Bhaw, Richard Myers, Steven Platt, Kirstin Edwards, Jonathan Hubb, Joanna Ellis, Maria Zambon
EPI_ISL_421823 hCoV-19/England/201380221/2020 Europe / United Kingdom / England 2020-03-25 Respiratory Virus Unit, Microbiology Services Colindale, Public Health England Respiratory Virus Unit, Microbiology Services Colindale, Public Health England Monica Galiano, Shahjahan Miah, Angie Lackenby, Omolola Akinbami, Tiina Talts, Leena Bhaw, Richard Myers, Steven Platt, Kirstin Edwards, Jonathan Hubb, Joanna Ellis, Maria Zambon
EPI_ISL_421824 hCoV-19/England/201380228/2020 Europe / United Kingdom / England 2020-03-25 Respiratory Virus Unit, Microbiology Services Colindale, Public Health England Respiratory Virus Unit, Microbiology Services Colindale, Public Health England Monica Galiano, Shahjahan Miah, Angie Lackenby, Omolola Akinbami, Tiina Talts, Leena Bhaw, Richard Myers, Steven Platt, Kirstin Edwards, Jonathan Hubb, Joanna Ellis, Maria Zambon
EPI_ISL_421825 hCoV-19/England/201380229/2020 Europe / United Kingdom / England 2020-03-25 Respiratory Virus Unit, Microbiology Services Colindale, Public Health England Respiratory Virus Unit, Microbiology Services Colindale, Public Health England Monica Galiano, Shahjahan Miah, Angie Lackenby, Omolola Akinbami, Tiina Talts, Leena Bhaw, Richard Myers, Steven Platt, Kirstin Edwards, Jonathan Hubb, Joanna Ellis, Maria Zambon
EPI_ISL_421826 hCoV-19/England/201380276/2020 Europe / United Kingdom / England 2020-03-23 Respiratory Virus Unit, Microbiology Services Colindale, Public Health England Respiratory Virus Unit, Microbiology Services Colindale, Public Health England Monica Galiano, Shahjahan Miah, Angie Lackenby, Omolola Akinbami, Tiina Talts, Leena Bhaw, Richard Myers, Steven Platt, Kirstin Edwards, Jonathan Hubb, Joanna Ellis, Maria Zambon
EPI_ISL_421827 hCoV-19/England/201380277/2020 Europe / United Kingdom / England 2020-03-24 Respiratory Virus Unit, Microbiology Services Colindale, Public Health England Respiratory Virus Unit, Microbiology Services Colindale, Public Health England Monica Galiano, Shahjahan Miah, Angie Lackenby, Omolola Akinbami, Tiina Talts, Leena Bhaw, Richard Myers, Steven Platt, Kirstin Edwards, Jonathan Hubb, Joanna Ellis, Maria Zambon
EPI_ISL_421828 hCoV-19/England/201380280/2020 Europe / United Kingdom / England 2020-03-23 Respiratory Virus Unit, Microbiology Services Colindale, Public Health England Respiratory Virus Unit, Microbiology Services Colindale, Public Health England Monica Galiano, Shahjahan Miah, Angie Lackenby, Omolola Akinbami, Tiina Talts, Leena Bhaw, Richard Myers, Steven Platt, Kirstin Edwards, Jonathan Hubb, Joanna Ellis, Maria Zambon
EPI_ISL_421829 hCoV-19/England/201380281/2020 Europe / United Kingdom / England 2020-03-25 Respiratory Virus Unit, Microbiology Services Colindale, Public Health England Respiratory Virus Unit, Microbiology Services Colindale, Public Health England Monica Galiano, Shahjahan Miah, Angie Lackenby, Omolola Akinbami, Tiina Talts, Leena Bhaw, Richard Myers, Steven Platt, Kirstin Edwards, Jonathan Hubb, Joanna Ellis, Maria Zambon
EPI_ISL_421830 hCoV-19/England/201380282/2020 Europe / United Kingdom / England 2020-03-25 Respiratory Virus Unit, Microbiology Services Colindale, Public Health England Respiratory Virus Unit, Microbiology Services Colindale, Public Health England Monica Galiano, Shahjahan Miah, Angie Lackenby, Omolola Akinbami, Tiina Talts, Leena Bhaw, Richard Myers, Steven Platt, Kirstin Edwards, Jonathan Hubb, Joanna Ellis, Maria Zambon
EPI_ISL_421831 hCoV-19/England/201380283/2020 Europe / United Kingdom / England 2020-03-23 Respiratory Virus Unit, Microbiology Services Colindale, Public Health England Respiratory Virus Unit, Microbiology Services Colindale, Public Health England Monica Galiano, Shahjahan Miah, Angie Lackenby, Omolola Akinbami, Tiina Talts, Leena Bhaw, Richard Myers, Steven Platt, Kirstin Edwards, Jonathan Hubb, Joanna Ellis, Maria Zambon
EPI_ISL_421834 hCoV-19/England/20138082004/2020 Europe / United Kingdom / England 2020-03-26 Respiratory Virus Unit, Microbiology Services Colindale, Public Health England Respiratory Virus Unit, Microbiology Services Colindale, Public Health England Monica Galiano, Shahjahan Miah, Angie Lackenby, Omolola Akinbami, Tiina Talts, Leena Bhaw, Richard Myers, Steven Platt, Kirstin Edwards, Jonathan Hubb, Joanna Ellis, Maria Zambon
EPI_ISL_421836 hCoV-19/England/20138082704/2020 Europe / United Kingdom / England 2020-03-26 Respiratory Virus Unit, Microbiology Services Colindale, Public Health England Respiratory Virus Unit,

Microbiology Services Colindale, Public Health England	Monica Galiano, Shahjahan Miah, Angie Lackenby, Omolola Akinbami, Tiina Talts, Leena Bhaw, Richard Myers, Steven Platt, Kirstin Edwards, Jonathan Hubb, Joanna Ellis, Maria Zambon
EPI_ISL_421837	hCoV-19/England/20138082804/2020	Europe / United Kingdom / England	2020-03-27	Respiratory Virus Unit, Microbiology Services Colindale, Public Health England	Respiratory Virus Unit, Microbiology Services Colindale, Public Health England	Monica Galiano, Shahjahan Miah, Angie Lackenby, Omolola Akinbami, Tiina Talts, Leena Bhaw, Richard Myers, Steven Platt, Kirstin Edwards, Jonathan Hubb, Joanna Ellis, Maria Zambon
EPI_ISL_421839	hCoV-19/England/20138083204/2020	Europe / United Kingdom / England	2020-03-27	Respiratory Virus Unit, Microbiology Services Colindale, Public Health England	Respiratory Virus Unit, Microbiology Services Colindale, Public Health England	Monica Galiano, Shahjahan Miah, Angie Lackenby, Omolola Akinbami, Tiina Talts, Leena Bhaw, Richard Myers, Steven Platt, Kirstin Edwards, Jonathan Hubb, Joanna Ellis, Maria Zambon
EPI_ISL_421842	hCoV-19/England/20138086904/2020	Europe / United Kingdom / England	2020-03-23	Respiratory Virus Unit, Microbiology Services Colindale, Public Health England	Respiratory Virus Unit, Microbiology Services Colindale, Public Health England	Monica Galiano, Shahjahan Miah, Angie Lackenby, Omolola Akinbami, Tiina Talts, Leena Bhaw, Richard Myers, Steven Platt, Kirstin Edwards, Jonathan Hubb, Joanna Ellis, Maria Zambon
EPI_ISL_421845	hCoV-19/England/20138088004/2020	Europe / United Kingdom / England	2020-03-24	Respiratory Virus Unit, Microbiology Services Colindale, Public Health England	Respiratory Virus Unit, Microbiology Services Colindale, Public Health England	Monica Galiano, Shahjahan Miah, Angie Lackenby, Omolola Akinbami, Tiina Talts, Leena Bhaw, Richard Myers, Steven Platt, Kirstin Edwards, Jonathan Hubb, Joanna Ellis, Maria Zambon
EPI_ISL_421847	hCoV-19/England/20138088204/2020	Europe / United Kingdom / England	2020-03-26	Respiratory Virus Unit, Microbiology Services Colindale, Public Health England	Respiratory Virus Unit, Microbiology Services Colindale, Public Health England	Monica Galiano, Shahjahan Miah, Angie Lackenby, Omolola Akinbami, Tiina Talts, Leena Bhaw, Richard Myers, Steven Platt, Kirstin Edwards, Jonathan Hubb, Joanna Ellis, Maria Zambon
EPI_ISL_421862	hCoV-19/England/20139008204/2020	Europe / United Kingdom / England	2020-03-26	Respiratory Virus Unit, Microbiology Services Colindale, Public Health England	Respiratory Virus Unit, Microbiology Services Colindale, Public Health England	Monica Galiano, Shahjahan Miah, Angie Lackenby, Omolola Akinbami, Tiina Talts, Leena Bhaw, Richard Myers, Steven Platt, Kirstin Edwards, Jonathan Hubb, Joanna Ellis, Maria Zambon
EPI_ISL_421863	hCoV-19/England/20139008304/2020	Europe / United Kingdom / England	2020-03-26	Respiratory Virus Unit, Microbiology Services Colindale, Public Health England	Respiratory Virus Unit, Microbiology Services Colindale, Public Health England	Monica Galiano, Shahjahan Miah, Angie Lackenby, Omolola Akinbami, Tiina Talts, Leena Bhaw, Richard Myers, Steven Platt, Kirstin Edwards, Jonathan Hubb, Joanna Ellis, Maria Zambon
EPI_ISL_421864	hCoV-19/England/20139008404/2020	Europe / United Kingdom / England	2020-03-27	Respiratory Virus Unit, Microbiology Services Colindale, Public Health England	Respiratory Virus Unit, Microbiology Services Colindale, Public Health England	Monica Galiano, Shahjahan Miah, Angie Lackenby, Omolola Akinbami, Tiina Talts, Leena Bhaw, Richard Myers, Steven Platt, Kirstin Edwards, Jonathan Hubb, Joanna Ellis, Maria Zambon
EPI_ISL_421865	hCoV-19/England/20139008704/2020	Europe / United Kingdom / England	2020-03-27	Respiratory Virus Unit, Microbiology Services Colindale, Public Health England	Respiratory Virus Unit, Microbiology Services Colindale, Public Health England	Monica Galiano, Shahjahan Miah, Angie Lackenby, Omolola Akinbami, Tiina Talts, Leena Bhaw, Richard Myers, Steven Platt, Kirstin Edwards, Jonathan Hubb, Joanna Ellis, Maria Zambon
EPI_ISL_421866	hCoV-19/England/20139009004/2020	Europe / United Kingdom / England	2020-03-27	Respiratory Virus Unit, Microbiology Services Colindale, Public Health England	Respiratory Virus Unit, Microbiology Services Colindale, Public Health England	Monica Galiano, Shahjahan Miah, Angie Lackenby, Omolola Akinbami, Tiina Talts, Leena Bhaw, Richard Myers, Steven Platt, Kirstin Edwards, Jonathan Hubb, Joanna Ellis, Maria Zambon
EPI_ISL_421867	hCoV-19/England/20139009204/2020	Europe / United Kingdom / England	2020-03-27	Respiratory Virus Unit, Microbiology Services Colindale, Public Health England	Respiratory Virus Unit, Microbiology Services Colindale, Public Health England	Monica Galiano, Shahjahan Miah, Angie Lackenby, Omolola Akinbami, Tiina Talts, Leena Bhaw, Richard Myers, Steven Platt, Kirstin Edwards, Jonathan Hubb, Joanna Ellis, Maria Zambon
EPI_ISL_421868	hCoV-19/England/20139009504/2020	Europe / United Kingdom / England	2020-03-27	Respiratory Virus Unit, Microbiology Services Colindale, Public Health England	Respiratory Virus Unit, Microbiology Services Colindale, Public Health England	Monica Galiano, Shahjahan Miah, Angie Lackenby, Omolola Akinbami, Tiina Talts, Leena Bhaw, Richard Myers, Steven Platt, Kirstin Edwards, Jonathan Hubb, Joanna Ellis, Maria Zambon
EPI_ISL_421869	hCoV-19/England/20139009604/2020	Europe / United Kingdom / England	2020-03-27	Respiratory Virus Unit, Microbiology Services Colindale, Public Health England	Respiratory Virus Unit, Microbiology Services Colindale, Public Health England	Monica Galiano, Shahjahan Miah, Angie Lackenby, Omolola Akinbami, Tiina Talts, Leena Bhaw, Richard Myers, Steven Platt, Kirstin Edwards, Jonathan Hubb, Joanna Ellis, Maria Zambon
EPI_ISL_421870	hCoV-19/England/20139009704/2020	Europe / United Kingdom / England	2020-03-27	Respiratory Virus Unit, Microbiology Services Colindale, Public Health England	Respiratory Virus Unit,

Microbiology Services Colindale, Public Health England Monica Galiano, Shahjahan Miah, Angie Lackenby, Omolola Akinbami, Tiina Talts, Leena Bhaw, Richard Myers, Steven Platt, Kirstin Edwards, Jonathan Hubb, Joanna Ellis, Maria Zambon
EPI_ISL_421875 hCoV-19/England/20139011304/2020 Europe / United Kingdom / England 2020-03-26 Respiratory Virus Unit, Microbiology Services Colindale, Public Health England Respiratory Virus Unit, Microbiology Services Colindale, Public Health England Monica Galiano, Shahjahan Miah, Angie Lackenby, Omolola Akinbami, Tiina Talts, Leena Bhaw, Richard Myers, Steven Platt, Kirstin Edwards, Jonathan Hubb, Joanna Ellis, Maria Zambon
EPI_ISL_421877 hCoV-19/England/20139014404/2020 Europe / United Kingdom / England 2020-03-27 Respiratory Virus Unit, Microbiology Services Colindale, Public Health England Respiratory Virus Unit, Microbiology Services Colindale, Public Health England Monica Galiano, Shahjahan Miah, Angie Lackenby, Omolola Akinbami, Tiina Talts, Leena Bhaw, Richard Myers, Steven Platt, Kirstin Edwards, Jonathan Hubb, Joanna Ellis, Maria Zambon
EPI_ISL_421879 hCoV-19/England/20139020204/2020 Europe / United Kingdom / England 2020-03-27 Respiratory Virus Unit, Microbiology Services Colindale, Public Health England Respiratory Virus Unit, Microbiology Services Colindale, Public Health England Monica Galiano, Shahjahan Miah, Angie Lackenby, Omolola Akinbami, Tiina Talts, Leena Bhaw, Richard Myers, Steven Platt, Kirstin Edwards, Jonathan Hubb, Joanna Ellis, Maria Zambon
EPI_ISL_421880 hCoV-19/England/20139021104/2020 Europe / United Kingdom / England 2020-03-26 Respiratory Virus Unit, Microbiology Services Colindale, Public Health England Respiratory Virus Unit, Microbiology Services Colindale, Public Health England Monica Galiano, Shahjahan Miah, Angie Lackenby, Omolola Akinbami, Tiina Talts, Leena Bhaw, Richard Myers, Steven Platt, Kirstin Edwards, Jonathan Hubb, Joanna Ellis, Maria Zambon
EPI_ISL_421882 hCoV-19/England/20139021604/2020 Europe / United Kingdom / England 2020-03-27 Respiratory Virus Unit, Microbiology Services Colindale, Public Health England Respiratory Virus Unit, Microbiology Services Colindale, Public Health England Monica Galiano, Shahjahan Miah, Angie Lackenby, Omolola Akinbami, Tiina Talts, Leena Bhaw, Richard Myers, Steven Platt, Kirstin Edwards, Jonathan Hubb, Joanna Ellis, Maria Zambon
EPI_ISL_421883 hCoV-19/England/20139022004/2020 Europe / United Kingdom / England 2020-03-27 Respiratory Virus Unit, Microbiology Services Colindale, Public Health England Respiratory Virus Unit, Microbiology Services Colindale, Public Health England Monica Galiano, Shahjahan Miah, Angie Lackenby, Omolola Akinbami, Tiina Talts, Leena Bhaw, Richard Myers, Steven Platt, Kirstin Edwards, Jonathan Hubb, Joanna Ellis, Maria Zambon
EPI_ISL_421885 hCoV-19/England/20139022504/2020 Europe / United Kingdom / England 2020-03-27 Respiratory Virus Unit, Microbiology Services Colindale, Public Health England Respiratory Virus Unit, Microbiology Services Colindale, Public Health England Monica Galiano, Shahjahan Miah, Angie Lackenby, Omolola Akinbami, Tiina Talts, Leena Bhaw, Richard Myers, Steven Platt, Kirstin Edwards, Jonathan Hubb, Joanna Ellis, Maria Zambon
EPI_ISL_421886 hCoV-19/England/20139022704/2020 Europe / United Kingdom / England 2020-03-27 Respiratory Virus Unit, Microbiology Services Colindale, Public Health England Respiratory Virus Unit, Microbiology Services Colindale, Public Health England Monica Galiano, Shahjahan Miah, Angie Lackenby, Omolola Akinbami, Tiina Talts, Leena Bhaw, Richard Myers, Steven Platt, Kirstin Edwards, Jonathan Hubb, Joanna Ellis, Maria Zambon
EPI_ISL_421887 hCoV-19/England/20139023504/2020 Europe / United Kingdom / England 2020-03-27 Respiratory Virus Unit, Microbiology Services Colindale, Public Health England Respiratory Virus Unit, Microbiology Services Colindale, Public Health England Monica Galiano, Shahjahan Miah, Angie Lackenby, Omolola Akinbami, Tiina Talts, Leena Bhaw, Richard Myers, Steven Platt, Kirstin Edwards, Jonathan Hubb, Joanna Ellis, Maria Zambon
EPI_ISL_421888 hCoV-19/England/20139023604/2020 Europe / United Kingdom / England 2020-03-27 Respiratory Virus Unit, Microbiology Services Colindale, Public Health England Respiratory Virus Unit, Microbiology Services Colindale, Public Health England Monica Galiano, Shahjahan Miah, Angie Lackenby, Omolola Akinbami, Tiina Talts, Leena Bhaw, Richard Myers, Steven Platt, Kirstin Edwards, Jonathan Hubb, Joanna Ellis, Maria Zambon
EPI_ISL_421890 hCoV-19/England/20139024004/2020 Europe / United Kingdom / England 2020-03-27 Respiratory Virus Unit, Microbiology Services Colindale, Public Health England Respiratory Virus Unit, Microbiology Services Colindale, Public Health England Monica Galiano, Shahjahan Miah, Angie Lackenby, Omolola Akinbami, Tiina Talts, Leena Bhaw, Richard Myers, Steven Platt, Kirstin Edwards, Jonathan Hubb, Joanna Ellis, Maria Zambon
EPI_ISL_421891 hCoV-19/England/20139024204/2020 Europe / United Kingdom / England 2020-03-27 Respiratory Virus Unit, Microbiology Services Colindale, Public Health England Respiratory Virus Unit, Microbiology Services Colindale, Public Health England Monica Galiano, Shahjahan Miah, Angie Lackenby, Omolola Akinbami, Tiina Talts, Leena Bhaw, Richard Myers, Steven Platt, Kirstin Edwards, Jonathan Hubb, Joanna Ellis, Maria Zambon
EPI_ISL_421894 hCoV-19/England/20139024804/2020 Europe / United Kingdom / England 2020-03-27 Respiratory Virus Unit, Microbiology Services Colindale, Public Health England Respiratory Virus Unit, Microbiology Services Colindale, Public Health England Monica Galiano, Shahjahan Miah, Angie Lackenby, Omolola Akinbami, Tiina Talts, Leena Bhaw, Richard Myers, Steven Platt, Kirstin Edwards, Jonathan Hubb, Joanna Ellis, Maria Zambon
EPI_ISL_421895 hCoV-19/England/20139024904/2020 Europe / United Kingdom / England 2020-03-27 Respiratory Virus Unit, Microbiology Services Colindale, Public Health England Respiratory Virus Unit,

Microbiology Services Colindale, Public Health England Monica Galiano, Shahjahan Miah, Angie Lackenby, Omolola Akinbami, Tiina Talts, Leena Bhaw, Richard Myers, Steven Platt, Kirstin Edwards, Jonathan Hubb, Joanna Ellis, Maria Zambon
EPI_ISL_421899 hCoV-19/England/20139026904/2020 Europe / United Kingdom / England 2020-03-26 Respiratory Virus Unit, Microbiology Services Colindale, Public Health England Respiratory Virus Unit, Microbiology Services Colindale, Public Health England Monica Galiano, Shahjahan Miah, Angie Lackenby, Omolola Akinbami, Tiina Talts, Leena Bhaw, Richard Myers, Steven Platt, Kirstin Edwards, Jonathan Hubb, Joanna Ellis, Maria Zambon
EPI_ISL_423000 hCoV-19/USA/WA-UW-1420/2020 North America / USA / Washington 2020-03-18 UW Virology Lab UW Virology Lab Pavitra Roychoudhury, Hong Xie, Keith Jerome, Alexander Greninger
EPI_ISL_423001 hCoV-19/USA/WA-UW-1567/2020 North America / USA / Washington 2020-03-19 UW Virology Lab UW Virology Lab Pavitra Roychoudhury, Hong Xie, Keith Jerome, Alexander Greninger
EPI_ISL_423002 hCoV-19/USA/WA-UW-1593/2020 North America / USA / Washington 2020-03-19 UW Virology Lab UW Virology Lab Pavitra Roychoudhury, Hong Xie, Keith Jerome, Alexander Greninger
EPI_ISL_423003 hCoV-19/USA/WA-UW-1583/2020 North America / USA / Washington 2020-03-19 UW Virology Lab UW Virology Lab Pavitra Roychoudhury, Hong Xie, Keith Jerome, Alexander Greninger
EPI_ISL_423004 hCoV-19/USA/WA-UW-1581/2020 North America / USA / Washington 2020-03-19 UW Virology Lab UW Virology Lab Pavitra Roychoudhury, Hong Xie, Keith Jerome, Alexander Greninger
EPI_ISL_423005 hCoV-19/USA/WA-UW-1603/2020 North America / USA / Washington 2020-03-19 UW Virology Lab UW Virology Lab Pavitra Roychoudhury, Hong Xie, Keith Jerome, Alexander Greninger
EPI_ISL_423006 hCoV-19/USA/WA-UW-1611/2020 North America / USA / Washington 2020-03-19 UW Virology Lab UW Virology Lab Pavitra Roychoudhury, Hong Xie, Keith Jerome, Alexander Greninger
EPI_ISL_423007 hCoV-19/USA/WA-UW-1448/2020 North America / USA / Washington 2020-03-19 UW Virology Lab UW Virology Lab Pavitra Roychoudhury, Hong Xie, Keith Jerome, Alexander Greninger
EPI_ISL_423008 hCoV-19/USA/WA-UW-1426/2020 North America / USA / Washington 2020-03-18 UW Virology Lab UW Virology Lab Pavitra Roychoudhury, Hong Xie, Keith Jerome, Alexander Greninger
EPI_ISL_423010 hCoV-19/USA/WA-UW-1446/2020 North America / USA / Washington 2020-03-18 UW Virology Lab UW Virology Lab Pavitra Roychoudhury, Hong Xie, Keith Jerome, Alexander Greninger
EPI_ISL_423011 hCoV-19/USA/WA-UW-1561/2020 North America / USA / Washington 2020-03-19 UW Virology Lab UW Virology Lab Pavitra Roychoudhury, Hong Xie, Keith Jerome, Alexander Greninger
EPI_ISL_423012 hCoV-19/USA/WA-UW-1588/2020 North America / USA / Washington 2020-03-19 UW Virology Lab UW Virology Lab Pavitra Roychoudhury, Hong Xie, Keith Jerome, Alexander Greninger
EPI_ISL_423013 hCoV-19/USA/WA-UW-1586/2020 North America / USA / Washington 2020-03-19 UW Virology Lab UW Virology Lab Pavitra Roychoudhury, Hong Xie, Keith Jerome, Alexander Greninger
EPI_ISL_423014 hCoV-19/USA/WA-UW-1587/2020 North America / USA / Washington 2020-03-19 UW Virology Lab UW Virology Lab Pavitra Roychoudhury, Hong Xie, Keith Jerome, Alexander Greninger
EPI_ISL_423015 hCoV-19/USA/WA-UW-1552/2020 North America / USA / Washington 2020-03-19 UW Virology Lab UW Virology Lab Pavitra Roychoudhury, Hong Xie, Keith Jerome, Alexander Greninger
EPI_ISL_423016 hCoV-19/USA/WA-UW-1578/2020 North America / USA / Washington 2020-03-19 UW Virology Lab UW Virology Lab Pavitra Roychoudhury, Hong Xie, Keith Jerome, Alexander Greninger
EPI_ISL_423017 hCoV-19/USA/WA-UW-1591/2020 North America / USA / Washington 2020-03-19 UW Virology Lab UW Virology Lab Pavitra Roychoudhury, Hong Xie, Keith Jerome, Alexander Greninger
EPI_ISL_423018 hCoV-19/USA/WA-UW-1576/2020 North America / USA / Washington 2020-03-19 UW Virology Lab UW Virology Lab Pavitra Roychoudhury, Hong Xie, Keith Jerome, Alexander Greninger
EPI_ISL_423019 hCoV-19/USA/WA-UW-1574/2020 North America / USA / Washington 2020-03-19 UW Virology Lab UW Virology Lab Pavitra Roychoudhury, Hong Xie, Keith Jerome, Alexander Greninger
EPI_ISL_423020 hCoV-19/USA/WA-UW-1570/2020 North America / USA / Washington 2020-03-19 UW Virology Lab UW Virology Lab Pavitra Roychoudhury, Hong Xie, Keith Jerome, Alexander Greninger
EPI_ISL_423021 hCoV-19/USA/WA-UW-1569/2020 North America / USA / Washington 2020-03-19 UW Virology Lab UW Virology Lab Pavitra Roychoudhury, Hong Xie, Keith Jerome, Alexander Greninger
EPI_ISL_423022 hCoV-19/USA/WA-UW-1562/2020 North America / USA / Washington 2020-03-19 UW Virology Lab UW Virology Lab Pavitra Roychoudhury, Hong Xie, Keith Jerome, Alexander Greninger
EPI_ISL_423023 hCoV-19/USA/UN-UW-1449/2020 North America / USA 2020-03 UW Virology Lab UW Virology Lab Pavitra Roychoudhury, Hong Xie, Keith Jerome, Alexander Greninger
EPI_ISL_423024 hCoV-19/USA/WA-UW-1435/2020 North America / USA / Washington 2020-03-17 UW Virology Lab UW Virology Lab Pavitra Roychoudhury, Hong Xie, Keith Jerome, Alexander Greninger
EPI_ISL_423025 hCoV-19/USA/WA-UW-1441/2020 North America / USA / Washington 2020-03-18 UW Virology Lab UW Virology Lab Pavitra Roychoudhury, Hong Xie, Keith Jerome, Alexander Greninger
EPI_ISL_423026 hCoV-19/USA/WA-UW-1440/2020 North America / USA / Washington 2020-03-18 UW Virology Lab UW Virology Lab Pavitra Roychoudhury, Hong Xie, Keith Jerome, Alexander Greninger
EPI_ISL_423027 hCoV-19/USA/WA-UW-1473/2020 North America / USA / Washington 2020-03-18 UW Virology Lab UW Virology Lab Pavitra Roychoudhury, Hong Xie, Keith Jerome, Alexander Greninger
EPI_ISL_423028 hCoV-19/USA/WA-UW-1470/2020 North America / USA / Washington 2020-03-18 UW Virology Lab UW Virology Lab Pavitra Roychoudhury, Hong Xie, Keith Jerome, Alexander Greninger
EPI_ISL_423029 hCoV-19/USA/WA-UW-1471/2020 North America / USA / Washington 2020-03-18 UW Virology Lab UW Virology Lab Pavitra Roychoudhury, Hong Xie, Keith Jerome, Alexander Greninger
EPI_ISL_423030 hCoV-19/USA/WA-UW-1433/2020 North America / USA / Washington 2020-03-18 UW Virology Lab UW Virology Lab Pavitra Roychoudhury, Hong Xie, Keith Jerome, Alexander Greninger
EPI_ISL_423031 hCoV-19/USA/WA-UW-1425/2020 North America / USA / Washington 2020-03-18 UW Virology Lab UW Virology Lab Pavitra Roychoudhury, Hong Xie, Keith Jerome, Alexander Greninger

EPI_ISL_423032 hCoV-19/USA/WA-UW-1608/2020 North America / USA / Washington 2020-03-18 UW Virology Lab UW Virology Lab Pavitra Roychoudhury, Hong Xie, Keith Jerome, Alexander Greninger
EPI_ISL_423033 hCoV-19/USA/WA-UW-1599/2020 North America / USA / Washington 2020-03-18 UW Virology Lab UW Virology Lab Pavitra Roychoudhury, Hong Xie, Keith Jerome, Alexander Greninger
EPI_ISL_423039 hCoV-19/Thailand/Bangkok-0017/2020 Asia / Thailand / Bangkok 2020-03-21 Ramathibodi Hospital COVID-19 Network Investigations (CONI) Alliance Elizabeth Batty, Wasun Chantratita, Thanat Chookajorn, Stefan Fernandez, Angkana Huang, Anthony R. Jones, Khajohn Joonsalak, Chonticha Klungtong, Theerarat Kochakarn, Namfon Kotanan, Krittikorn Kumpornsin, Wudtichai Manasatienkij, Bhakbhoom Panthan, Ekawat Pasomsub, Insee Sensorn, Arporn Wangwiwatsin
EPI_ISL_423040 hCoV-19/Thailand/Bangkok-0019/2020 Asia / Thailand / Bangkok 2020-03-20 Ramathibodi Hospital COVID-19 Network Investigations (CONI) Alliance Elizabeth Batty, Wasun Chantratita, Thanat Chookajorn, Stefan Fernandez, Angkana Huang, Anthony R. Jones, Khajohn Joonsalak, Chonticha Klungtong, Theerarat Kochakarn, Namfon Kotanan, Krittikorn Kumpornsin, Wudtichai Manasatienkij, Bhakbhoom Panthan, Ekawat Pasomsub, Insee Sensorn, Arporn Wangwiwatsin
EPI_ISL_423041 hCoV-19/Thailand/Bangkok-0020/2020 Asia / Thailand / Bangkok 2020-03-21 Ramathibodi Hospital COVID-19 Network Investigations (CONI) Alliance Elizabeth Batty, Wasun Chantratita, Thanat Chookajorn, Stefan Fernandez, Angkana Huang, Anthony R. Jones, Khajohn Joonsalak, Chonticha Klungtong, Theerarat Kochakarn, Namfon Kotanan, Krittikorn Kumpornsin, Wudtichai Manasatienkij, Bhakbhoom Panthan, Ekawat Pasomsub, Insee Sensorn, Arporn Wangwiwatsin
EPI_ISL_423042 hCoV-19/Thailand/Bangkok-0021/2020 Asia / Thailand / Bangkok 2020-03-24 Ramathibodi Hospital COVID-19 Network Investigations (CONI) Alliance Elizabeth Batty, Wasun Chantratita, Thanat Chookajorn, Stefan Fernandez, Angkana Huang, Anthony R. Jones, Khajohn Joonsalak, Chonticha Klungtong, Theerarat Kochakarn, Namfon Kotanan, Krittikorn Kumpornsin, Wudtichai Manasatienkij, Bhakbhoom Panthan, Ekawat Pasomsub, Insee Sensorn, Arporn Wangwiwatsin
EPI_ISL_423043 hCoV-19/Thailand/Bangkok-0022/2020 Asia / Thailand / Bangkok 2020-03-18 Ramathibodi Hospital COVID-19 Network Investigations (CONI) Alliance Elizabeth Batty, Wasun Chantratita, Thanat Chookajorn, Stefan Fernandez, Angkana Huang, Anthony R. Jones, Khajohn Joonsalak, Chonticha Klungtong, Theerarat Kochakarn, Namfon Kotanan, Krittikorn Kumpornsin, Wudtichai Manasatienkij, Bhakbhoom Panthan, Ekawat Pasomsub, Insee Sensorn, Arporn Wangwiwatsin
EPI_ISL_423044 hCoV-19/England/20134000704/2020 Europe / United Kingdom / England 2020-03-25 Respiratory Virus Unit, Microbiology Services Colindale, Public Health England Respiratory Virus Unit, Microbiology Services Colindale, Public Health England Monica Galiano, Shahjahan Miah, Angie Lackenby, Omolola Akinbami, Tiina Talts, Leena Bhaw, Richard Myers, Steven Platt, Kirstin Edwards, Jonathan Hubb, Joanna Ellis, Maria Zambon
EPI_ISL_423045 hCoV-19/England/20134000804/2020 Europe / United Kingdom / England 2020-03-24 Respiratory Virus Unit, Microbiology Services Colindale, Public Health England Respiratory Virus Unit, Microbiology Services Colindale, Public Health England Monica Galiano, Shahjahan Miah, Angie Lackenby, Omolola Akinbami, Tiina Talts, Leena Bhaw, Richard Myers, Steven Platt, Kirstin Edwards, Jonathan Hubb, Joanna Ellis, Maria Zambon
EPI_ISL_423047 hCoV-19/England/20134001104/2020 Europe / United Kingdom / England 2020-03-24 Respiratory Virus Unit, Microbiology Services Colindale, Public Health England Respiratory Virus Unit, Microbiology Services Colindale, Public Health England Monica Galiano, Shahjahan Miah, Angie Lackenby, Omolola Akinbami, Tiina Talts, Leena Bhaw, Richard Myers, Steven Platt, Kirstin Edwards, Jonathan Hubb, Joanna Ellis, Maria Zambon
EPI_ISL_423052 hCoV-19/England/20134001904/2020 Europe / United Kingdom / England 2020-03-19 Respiratory Virus Unit, Microbiology Services Colindale, Public Health England Respiratory Virus Unit, Microbiology Services Colindale, Public Health England Monica Galiano, Shahjahan Miah, Angie Lackenby, Omolola Akinbami, Tiina Talts, Leena Bhaw, Richard Myers, Steven Platt, Kirstin Edwards, Jonathan Hubb, Joanna Ellis, Maria Zambon
EPI_ISL_423053 hCoV-19/England/20134002104/2020 Europe / United Kingdom / England 2020-03-18 Respiratory Virus Unit, Microbiology Services Colindale, Public Health England Respiratory Virus Unit, Microbiology Services Colindale, Public Health England Monica Galiano, Shahjahan Miah, Angie Lackenby, Omolola Akinbami, Tiina Talts, Leena Bhaw, Richard Myers, Steven Platt, Kirstin Edwards, Jonathan Hubb, Joanna Ellis, Maria Zambon
EPI_ISL_423055 hCoV-19/England/20134003004/2020 Europe / United Kingdom / England 2020-03-19 Respiratory Virus Unit, Microbiology Services Colindale, Public Health England Respiratory Virus Unit, Microbiology Services Colindale, Public Health England Monica Galiano, Shahjahan Miah, Angie Lackenby, Omolola Akinbami, Tiina Talts, Leena Bhaw, Richard Myers, Steven Platt, Kirstin Edwards, Jonathan Hubb, Joanna Ellis, Maria Zambon
EPI_ISL_423057 hCoV-19/England/20134003204/2020 Europe / United Kingdom / England 2020-03-23 Respiratory Virus Unit, Microbiology Services Colindale, Public Health England Respiratory Virus Unit, Microbiology Services Colindale, Public Health England Monica Galiano, Shahjahan Miah, Angie Lackenby, Omolola Akinbami, Tiina Talts, Leena Bhaw, Richard Myers, Steven Platt, Kirstin Edwards, Jonathan Hubb, Joanna Ellis, Maria Zambon
EPI_ISL_423059 hCoV-19/England/20134003404/2020 Europe / United Kingdom / England 2020-03-24 Respiratory Virus Unit, Microbiology Services Colindale, Public Health England Respiratory Virus Unit, Microbiology Services Colindale, Public Health England Monica Galiano, Shahjahan Miah, Angie Lackenby, Omolola Akinbami, Tiina Talts, Leena Bhaw, Richard Myers, Steven Platt, Kirstin Edwards, Jonathan Hubb, Joanna Ellis, Maria Zambon
EPI_ISL_423063 hCoV-19/England/20134004704/2020 Europe / United Kingdom / England 2020-03-24

Respiratory Virus Unit, Microbiology Services Colindale, Public Health England Respiratory Virus Unit, Microbiology Services Colindale, Public Health England Monica Galiano, Shahjahan Miah, Angie Lackenby, Omolola Akinbami, Tiina Talts, Leena Bhaw, Richard Myers, Steven Platt, Kirstin Edwards, Jonathan Hubb, Joanna Ellis, Maria Zambon
EPI_ISL_423065 hCoV-19/England/20134004904/2020 Europe / United Kingdom / England 2020-03-24 Respiratory Virus Unit, Microbiology Services Colindale, Public Health England Respiratory Virus Unit, Microbiology Services Colindale, Public Health England Monica Galiano, Shahjahan Miah, Angie Lackenby, Omolola Akinbami, Tiina Talts, Leena Bhaw, Richard Myers, Steven Platt, Kirstin Edwards, Jonathan Hubb, Joanna Ellis, Maria Zambon
EPI_ISL_423066 hCoV-19/England/20134005004/2020 Europe / United Kingdom / England 2020-03-24 Respiratory Virus Unit, Microbiology Services Colindale, Public Health England Respiratory Virus Unit, Microbiology Services Colindale, Public Health England Monica Galiano, Shahjahan Miah, Angie Lackenby, Omolola Akinbami, Tiina Talts, Leena Bhaw, Richard Myers, Steven Platt, Kirstin Edwards, Jonathan Hubb, Joanna Ellis, Maria Zambon
EPI_ISL_423067 hCoV-19/England/20134005104/2020 Europe / United Kingdom / England 2020-03-24 Respiratory Virus Unit, Microbiology Services Colindale, Public Health England Respiratory Virus Unit, Microbiology Services Colindale, Public Health England Monica Galiano, Shahjahan Miah, Angie Lackenby, Omolola Akinbami, Tiina Talts, Leena Bhaw, Richard Myers, Steven Platt, Kirstin Edwards, Jonathan Hubb, Joanna Ellis, Maria Zambon
EPI_ISL_423068 hCoV-19/England/20134005304/2020 Europe / United Kingdom / England 2020-03-23 Respiratory Virus Unit, Microbiology Services Colindale, Public Health England Respiratory Virus Unit, Microbiology Services Colindale, Public Health England Monica Galiano, Shahjahan Miah, Angie Lackenby, Omolola Akinbami, Tiina Talts, Leena Bhaw, Richard Myers, Steven Platt, Kirstin Edwards, Jonathan Hubb, Joanna Ellis, Maria Zambon
EPI_ISL_423069 hCoV-19/England/20134006404/2020 Europe / United Kingdom / England 2020-03-24 Respiratory Virus Unit, Microbiology Services Colindale, Public Health England Respiratory Virus Unit, Microbiology Services Colindale, Public Health England Monica Galiano, Shahjahan Miah, Angie Lackenby, Omolola Akinbami, Tiina Talts, Leena Bhaw, Richard Myers, Steven Platt, Kirstin Edwards, Jonathan Hubb, Joanna Ellis, Maria Zambon
EPI_ISL_423070 hCoV-19/England/20134006504/2020 Europe / United Kingdom / England 2020-03-24 Respiratory Virus Unit, Microbiology Services Colindale, Public Health England Respiratory Virus Unit, Microbiology Services Colindale, Public Health England Monica Galiano, Shahjahan Miah, Angie Lackenby, Omolola Akinbami, Tiina Talts, Leena Bhaw, Richard Myers, Steven Platt, Kirstin Edwards, Jonathan Hubb, Joanna Ellis, Maria Zambon
EPI_ISL_423071 hCoV-19/England/20134006604/2020 Europe / United Kingdom / England 2020-03-23 Respiratory Virus Unit, Microbiology Services Colindale, Public Health England Respiratory Virus Unit, Microbiology Services Colindale, Public Health England Monica Galiano, Shahjahan Miah, Angie Lackenby, Omolola Akinbami, Tiina Talts, Leena Bhaw, Richard Myers, Steven Platt, Kirstin Edwards, Jonathan Hubb, Joanna Ellis, Maria Zambon
EPI_ISL_423072 hCoV-19/England/20134006704/2020 Europe / United Kingdom / England 2020-03-24 Respiratory Virus Unit, Microbiology Services Colindale, Public Health England Respiratory Virus Unit, Microbiology Services Colindale, Public Health England Monica Galiano, Shahjahan Miah, Angie Lackenby, Omolola Akinbami, Tiina Talts, Leena Bhaw, Richard Myers, Steven Platt, Kirstin Edwards, Jonathan Hubb, Joanna Ellis, Maria Zambon
EPI_ISL_423073 hCoV-19/England/20134007004/2020 Europe / United Kingdom / England 2020-03-24 Respiratory Virus Unit, Microbiology Services Colindale, Public Health England Respiratory Virus Unit, Microbiology Services Colindale, Public Health England Monica Galiano, Shahjahan Miah, Angie Lackenby, Omolola Akinbami, Tiina Talts, Leena Bhaw, Richard Myers, Steven Platt, Kirstin Edwards, Jonathan Hubb, Joanna Ellis, Maria Zambon
EPI_ISL_423074 hCoV-19/England/20134007104/2020 Europe / United Kingdom / England 2020-03-24 Respiratory Virus Unit, Microbiology Services Colindale, Public Health England Respiratory Virus Unit, Microbiology Services Colindale, Public Health England Monica Galiano, Shahjahan Miah, Angie Lackenby, Omolola Akinbami, Tiina Talts, Leena Bhaw, Richard Myers, Steven Platt, Kirstin Edwards, Jonathan Hubb, Joanna Ellis, Maria Zambon
EPI_ISL_423075 hCoV-19/England/20134007204/2020 Europe / United Kingdom / England 2020-03-23 Respiratory Virus Unit, Microbiology Services Colindale, Public Health England Respiratory Virus Unit, Microbiology Services Colindale, Public Health England Monica Galiano, Shahjahan Miah, Angie Lackenby, Omolola Akinbami, Tiina Talts, Leena Bhaw, Richard Myers, Steven Platt, Kirstin Edwards, Jonathan Hubb, Joanna Ellis, Maria Zambon
EPI_ISL_423076 hCoV-19/England/20134009504/2020 Europe / United Kingdom / England 2020-03-24 Respiratory Virus Unit, Microbiology Services Colindale, Public Health England Respiratory Virus Unit, Microbiology Services Colindale, Public Health England Monica Galiano, Shahjahan Miah, Angie Lackenby, Omolola Akinbami, Tiina Talts, Leena Bhaw, Richard Myers, Steven Platt, Kirstin Edwards, Jonathan Hubb, Joanna Ellis, Maria Zambon
EPI_ISL_423078 hCoV-19/England/20134010904/2020 Europe / United Kingdom / England 2020-03-25 Respiratory Virus Unit, Microbiology Services Colindale, Public Health England Respiratory Virus Unit, Microbiology Services Colindale, Public Health England Monica Galiano, Shahjahan Miah, Angie Lackenby, Omolola Akinbami, Tiina Talts, Leena Bhaw, Richard Myers, Steven Platt, Kirstin Edwards, Jonathan Hubb, Joanna Ellis, Maria Zambon
EPI_ISL_423081 hCoV-19/England/201340120/2020 Europe / United Kingdom / England 2020-03-24

Respiratory Virus Unit, Microbiology Services Colindale, Public Health England Respiratory Virus Unit, Microbiology Services Colindale, Public Health England Monica Galiano, Shahjahan Miah, Angie Lackenby, Omolola Akinbami, Tiina Talts, Leena Bhaw, Richard Myers, Steven Platt, Kirstin Edwards, Jonathan Hubb, Joanna Ellis, Maria Zambon
EPI_ISL_423084 hCoV-19/England/201340127/2020 Europe / United Kingdom / England 2020-03-24 Respiratory Virus Unit, Microbiology Services Colindale, Public Health England Respiratory Virus Unit, Microbiology Services Colindale, Public Health England Monica Galiano, Shahjahan Miah, Angie Lackenby, Omolola Akinbami, Tiina Talts, Leena Bhaw, Richard Myers, Steven Platt, Kirstin Edwards, Jonathan Hubb, Joanna Ellis, Maria Zambon
EPI_ISL_423085 hCoV-19/England/20134015504/2020 Europe / United Kingdom / England 2020-03-25 Respiratory Virus Unit, Microbiology Services Colindale, Public Health England Respiratory Virus Unit, Microbiology Services Colindale, Public Health England Monica Galiano, Shahjahan Miah, Angie Lackenby, Omolola Akinbami, Tiina Talts, Leena Bhaw, Richard Myers, Steven Platt, Kirstin Edwards, Jonathan Hubb, Joanna Ellis, Maria Zambon
EPI_ISL_423086 hCoV-19/England/20134015704/2020 Europe / United Kingdom / England 2020-03-24 Respiratory Virus Unit, Microbiology Services Colindale, Public Health England Respiratory Virus Unit, Microbiology Services Colindale, Public Health England Monica Galiano, Shahjahan Miah, Angie Lackenby, Omolola Akinbami, Tiina Talts, Leena Bhaw, Richard Myers, Steven Platt, Kirstin Edwards, Jonathan Hubb, Joanna Ellis, Maria Zambon
EPI_ISL_423087 hCoV-19/England/20134015902/2020 Europe / United Kingdom / England 2020-03-23 Respiratory Virus Unit, Microbiology Services Colindale, Public Health England Respiratory Virus Unit, Microbiology Services Colindale, Public Health England Monica Galiano, Shahjahan Miah, Angie Lackenby, Omolola Akinbami, Tiina Talts, Leena Bhaw, Richard Myers, Steven Platt, Kirstin Edwards, Jonathan Hubb, Joanna Ellis, Maria Zambon
EPI_ISL_424001 hCoV-19/England/20129016104/2020 Europe / United Kingdom / England 2020-03-19 Respiratory Virus Unit, Microbiology Services Colindale, Public Health England Respiratory Virus Unit, Microbiology Services Colindale, Public Health England Monica Galiano, Shahjahan Miah, Angie Lackenby, Omolola Akinbami, Tiina Talts, Leena Bhaw, Richard Myers, Steven Platt, Kirstin Edwards, Jonathan Hubb, Joanna Ellis, Maria Zambon
EPI_ISL_424003 hCoV-19/England/20129017104/2020 Europe / United Kingdom / England 2020-03-20 Respiratory Virus Unit, Microbiology Services Colindale, Public Health England Respiratory Virus Unit, Microbiology Services Colindale, Public Health England Monica Galiano, Shahjahan Miah, Angie Lackenby, Omolola Akinbami, Tiina Talts, Leena Bhaw, Richard Myers, Steven Platt, Kirstin Edwards, Jonathan Hubb, Joanna Ellis, Maria Zambon
EPI_ISL_424006 hCoV-19/England/20129018604/2020 Europe / United Kingdom / England 2020-03-20 Respiratory Virus Unit, Microbiology Services Colindale, Public Health England Respiratory Virus Unit, Microbiology Services Colindale, Public Health England Monica Galiano, Shahjahan Miah, Angie Lackenby, Omolola Akinbami, Tiina Talts, Leena Bhaw, Richard Myers, Steven Platt, Kirstin Edwards, Jonathan Hubb, Joanna Ellis, Maria Zambon
EPI_ISL_424007 hCoV-19/England/20129018904/2020 Europe / United Kingdom / England 2020-03-20 Respiratory Virus Unit, Microbiology Services Colindale, Public Health England Respiratory Virus Unit, Microbiology Services Colindale, Public Health England Monica Galiano, Shahjahan Miah, Angie Lackenby, Omolola Akinbami, Tiina Talts, Leena Bhaw, Richard Myers, Steven Platt, Kirstin Edwards, Jonathan Hubb, Joanna Ellis, Maria Zambon
EPI_ISL_424009 hCoV-19/England/20129020004/2020 Europe / United Kingdom / England 2020-03-20 Respiratory Virus Unit, Microbiology Services Colindale, Public Health England Respiratory Virus Unit, Microbiology Services Colindale, Public Health England Monica Galiano, Shahjahan Miah, Angie Lackenby, Omolola Akinbami, Tiina Talts, Leena Bhaw, Richard Myers, Steven Platt, Kirstin Edwards, Jonathan Hubb, Joanna Ellis, Maria Zambon
EPI_ISL_424010 hCoV-19/England/20129020304/2020 Europe / United Kingdom / England 2020-03-20 Respiratory Virus Unit, Microbiology Services Colindale, Public Health England Respiratory Virus Unit, Microbiology Services Colindale, Public Health England Monica Galiano, Shahjahan Miah, Angie Lackenby, Omolola Akinbami, Tiina Talts, Leena Bhaw, Richard Myers, Steven Platt, Kirstin Edwards, Jonathan Hubb, Joanna Ellis, Maria Zambon
EPI_ISL_424012 hCoV-19/England/20129020604/2020 Europe / United Kingdom / England 2020-03-20 Respiratory Virus Unit, Microbiology Services Colindale, Public Health England Respiratory Virus Unit, Microbiology Services Colindale, Public Health England Monica Galiano, Shahjahan Miah, Angie Lackenby, Omolola Akinbami, Tiina Talts, Leena Bhaw, Richard Myers, Steven Platt, Kirstin Edwards, Jonathan Hubb, Joanna Ellis, Maria Zambon
EPI_ISL_424015 hCoV-19/England/20129023104/2020 Europe / United Kingdom / England 2020-03-19 Respiratory Virus Unit, Microbiology Services Colindale, Public Health England Respiratory Virus Unit, Microbiology Services Colindale, Public Health England Monica Galiano, Shahjahan Miah, Angie Lackenby, Omolola Akinbami, Tiina Talts, Leena Bhaw, Richard Myers, Steven Platt, Kirstin Edwards, Jonathan Hubb, Joanna Ellis, Maria Zambon
EPI_ISL_424017 hCoV-19/England/20129025404/2020 Europe / United Kingdom / England 2020-03-12 Respiratory Virus Unit, Microbiology Services Colindale, Public Health England Respiratory Virus Unit, Microbiology Services Colindale, Public Health England Monica Galiano, Shahjahan Miah, Angie Lackenby, Omolola Akinbami, Tiina Talts, Leena Bhaw, Richard Myers, Steven Platt, Kirstin Edwards, Jonathan Hubb, Joanna Ellis, Maria Zambon
EPI_ISL_424019 hCoV-19/England/20129031804/2020 Europe / United Kingdom / England 2020-03-19

Respiratory Virus Unit, Microbiology Services Colindale, Public Health England Respiratory Virus Unit, Microbiology Services Colindale, Public Health England Monica Galiano, Shahjahan Miah, Angie Lackenby, Omolola Akinbami, Tiina Talts, Leena Bhaw, Richard Myers, Steven Platt, Kirstin Edwards, Jonathan Hubb, Joanna Ellis, Maria Zambon
EPI_ISL_424021 hCoV-19/England/20129032404/2020 Europe / United Kingdom / England 2020-03-20 Respiratory Virus Unit, Microbiology Services Colindale, Public Health England Respiratory Virus Unit, Microbiology Services Colindale, Public Health England Monica Galiano, Shahjahan Miah, Angie Lackenby, Omolola Akinbami, Tiina Talts, Leena Bhaw, Richard Myers, Steven Platt, Kirstin Edwards, Jonathan Hubb, Joanna Ellis, Maria Zambon
EPI_ISL_424024 hCoV-19/England/20129033804/2020 Europe / United Kingdom / England 2020-03-20 Respiratory Virus Unit, Microbiology Services Colindale, Public Health England Respiratory Virus Unit, Microbiology Services Colindale, Public Health England Monica Galiano, Shahjahan Miah, Angie Lackenby, Omolola Akinbami, Tiina Talts, Leena Bhaw, Richard Myers, Steven Platt, Kirstin Edwards, Jonathan Hubb, Joanna Ellis, Maria Zambon
EPI_ISL_424025 hCoV-19/England/20129033904/2020 Europe / United Kingdom / England 2020-03-20 Respiratory Virus Unit, Microbiology Services Colindale, Public Health England Respiratory Virus Unit, Microbiology Services Colindale, Public Health England Monica Galiano, Shahjahan Miah, Angie Lackenby, Omolola Akinbami, Tiina Talts, Leena Bhaw, Richard Myers, Steven Platt, Kirstin Edwards, Jonathan Hubb, Joanna Ellis, Maria Zambon
EPI_ISL_424028 hCoV-19/England/20129035304/2020 Europe / United Kingdom / England 2020-03-21 Respiratory Virus Unit, Microbiology Services Colindale, Public Health England Respiratory Virus Unit, Microbiology Services Colindale, Public Health England Monica Galiano, Shahjahan Miah, Angie Lackenby, Omolola Akinbami, Tiina Talts, Leena Bhaw, Richard Myers, Steven Platt, Kirstin Edwards, Jonathan Hubb, Joanna Ellis, Maria Zambon
EPI_ISL_424029 hCoV-19/England/20129035404/2020 Europe / United Kingdom / England 2020-03-20 Respiratory Virus Unit, Microbiology Services Colindale, Public Health England Respiratory Virus Unit, Microbiology Services Colindale, Public Health England Monica Galiano, Shahjahan Miah, Angie Lackenby, Omolola Akinbami, Tiina Talts, Leena Bhaw, Richard Myers, Steven Platt, Kirstin Edwards, Jonathan Hubb, Joanna Ellis, Maria Zambon
EPI_ISL_424031 hCoV-19/England/20129035804/2020 Europe / United Kingdom / England 2020-03-20 Respiratory Virus Unit, Microbiology Services Colindale, Public Health England Respiratory Virus Unit, Microbiology Services Colindale, Public Health England Monica Galiano, Shahjahan Miah, Angie Lackenby, Omolola Akinbami, Tiina Talts, Leena Bhaw, Richard Myers, Steven Platt, Kirstin Edwards, Jonathan Hubb, Joanna Ellis, Maria Zambon
EPI_ISL_424032 hCoV-19/England/20129036004/2020 Europe / United Kingdom / England 2020-03-20 Respiratory Virus Unit, Microbiology Services Colindale, Public Health England Respiratory Virus Unit, Microbiology Services Colindale, Public Health England Monica Galiano, Shahjahan Miah, Angie Lackenby, Omolola Akinbami, Tiina Talts, Leena Bhaw, Richard Myers, Steven Platt, Kirstin Edwards, Jonathan Hubb, Joanna Ellis, Maria Zambon
EPI_ISL_424034 hCoV-19/England/20129036404/2020 Europe / United Kingdom / England 2020-03-20 Respiratory Virus Unit, Microbiology Services Colindale, Public Health England Respiratory Virus Unit, Microbiology Services Colindale, Public Health England Monica Galiano, Shahjahan Miah, Angie Lackenby, Omolola Akinbami, Tiina Talts, Leena Bhaw, Richard Myers, Steven Platt, Kirstin Edwards, Jonathan Hubb, Joanna Ellis, Maria Zambon
EPI_ISL_424035 hCoV-19/England/20129036504/2020 Europe / United Kingdom / England 2020-03-20 Respiratory Virus Unit, Microbiology Services Colindale, Public Health England Respiratory Virus Unit, Microbiology Services Colindale, Public Health England Monica Galiano, Shahjahan Miah, Angie Lackenby, Omolola Akinbami, Tiina Talts, Leena Bhaw, Richard Myers, Steven Platt, Kirstin Edwards, Jonathan Hubb, Joanna Ellis, Maria Zambon
EPI_ISL_424037 hCoV-19/England/20129037204/2020 Europe / United Kingdom / England 2020-03-20 Respiratory Virus Unit, Microbiology Services Colindale, Public Health England Respiratory Virus Unit, Microbiology Services Colindale, Public Health England Monica Galiano, Shahjahan Miah, Angie Lackenby, Omolola Akinbami, Tiina Talts, Leena Bhaw, Richard Myers, Steven Platt, Kirstin Edwards, Jonathan Hubb, Joanna Ellis, Maria Zambon
EPI_ISL_424038 hCoV-19/England/20129037304/2020 Europe / United Kingdom / England 2020-03-20 Respiratory Virus Unit, Microbiology Services Colindale, Public Health England Respiratory Virus Unit, Microbiology Services Colindale, Public Health England Monica Galiano, Shahjahan Miah, Angie Lackenby, Omolola Akinbami, Tiina Talts, Leena Bhaw, Richard Myers, Steven Platt, Kirstin Edwards, Jonathan Hubb, Joanna Ellis, Maria Zambon
EPI_ISL_424052 hCoV-19/England/20129056804/2020 Europe / United Kingdom / England 2020-03-21 Respiratory Virus Unit, Microbiology Services Colindale, Public Health England Respiratory Virus Unit, Microbiology Services Colindale, Public Health England Monica Galiano, Shahjahan Miah, Angie Lackenby, Omolola Akinbami, Tiina Talts, Leena Bhaw, Richard Myers, Steven Platt, Kirstin Edwards, Jonathan Hubb, Joanna Ellis, Maria Zambon
EPI_ISL_424054 hCoV-19/England/20129059204/2020 Europe / United Kingdom / England 2020-03-09 Respiratory Virus Unit, Microbiology Services Colindale, Public Health England Respiratory Virus Unit, Microbiology Services Colindale, Public Health England Monica Galiano, Shahjahan Miah, Angie Lackenby, Omolola Akinbami, Tiina Talts, Leena Bhaw, Richard Myers, Steven Platt, Kirstin Edwards, Jonathan Hubb, Joanna Ellis, Maria Zambon
EPI_ISL_424062 hCoV-19/England/20129063804/2020 Europe / United Kingdom / England 2020-03-11

Respiratory Virus Unit, Microbiology Services Colindale, Public Health England Respiratory Virus Unit, Microbiology Services Colindale, Public Health England Monica Galiano, Shahjahan Miah, Angie Lackenby, Omolola Akinbami, Tiina Talts, Leena Bhaw, Richard Myers, Steven Platt, Kirstin Edwards, Jonathan Hubb, Joanna Ellis, Maria Zambon
EPI_ISL_424076 hCoV-19/England/20129085704/2020 Europe / United Kingdom / England 2020-03-21 Respiratory Virus Unit, Microbiology Services Colindale, Public Health England Respiratory Virus Unit, Microbiology Services Colindale, Public Health England Monica Galiano, Shahjahan Miah, Angie Lackenby, Omolola Akinbami, Tiina Talts, Leena Bhaw, Richard Myers, Steven Platt, Kirstin Edwards, Jonathan Hubb, Joanna Ellis, Maria Zambon
EPI_ISL_424078 hCoV-19/England/20129089504/2020 Europe / United Kingdom / England 2020-03-11 Respiratory Virus Unit, Microbiology Services Colindale, Public Health England Respiratory Virus Unit, Microbiology Services Colindale, Public Health England Monica Galiano, Shahjahan Miah, Angie Lackenby, Omolola Akinbami, Tiina Talts, Leena Bhaw, Richard Myers, Steven Platt, Kirstin Edwards, Jonathan Hubb, Joanna Ellis, Maria Zambon
EPI_ISL_424082 hCoV-19/England/20129096604/2020 Europe / United Kingdom / England 2020-03-11 Respiratory Virus Unit, Microbiology Services Colindale, Public Health England Respiratory Virus Unit, Microbiology Services Colindale, Public Health England Monica Galiano, Shahjahan Miah, Angie Lackenby, Omolola Akinbami, Tiina Talts, Leena Bhaw, Richard Myers, Steven Platt, Kirstin Edwards, Jonathan Hubb, Joanna Ellis, Maria Zambon
EPI_ISL_424084 hCoV-19/England/20129096804/2020 Europe / United Kingdom / England 2020-03-11 Respiratory Virus Unit, Microbiology Services Colindale, Public Health England Respiratory Virus Unit, Microbiology Services Colindale, Public Health England Monica Galiano, Shahjahan Miah, Angie Lackenby, Omolola Akinbami, Tiina Talts, Leena Bhaw, Richard Myers, Steven Platt, Kirstin Edwards, Jonathan Hubb, Joanna Ellis, Maria Zambon
EPI_ISL_424300 hCoV-19/USA/WA-UW-1879/2020 North America / USA / Washington 2020-03-21 UW Virology Lab UW Virology Lab Pavitra Roychoudhury, Hong Xie, Keith Jerome, Alexander Greninger
EPI_ISL_424301 hCoV-19/USA/WA-UW-1948/2020 North America / USA / Washington 2020-03-24 UW Virology Lab UW Virology Lab Pavitra Roychoudhury, Hong Xie, Keith Jerome, Alexander Greninger
EPI_ISL_424302 hCoV-19/USA/WA-UW-1878/2020 North America / USA / Washington 2020-03-24 UW Virology Lab UW Virology Lab Pavitra Roychoudhury, Hong Xie, Keith Jerome, Alexander Greninger
EPI_ISL_424304 hCoV-19/USA/WA-UW-1930/2020 North America / USA / Washington 2020-03-24 UW Virology Lab UW Virology Lab Pavitra Roychoudhury, Hong Xie, Keith Jerome, Alexander Greninger
EPI_ISL_424305 hCoV-19/USA/WA-UW-1877/2020 North America / USA / Washington 2020-03-24 UW Virology Lab UW Virology Lab Pavitra Roychoudhury, Hong Xie, Keith Jerome, Alexander Greninger
EPI_ISL_424306 hCoV-19/USA/WA-UW-1874/2020 North America / USA / Washington 2020-03-23 UW Virology Lab UW Virology Lab Pavitra Roychoudhury, Hong Xie, Keith Jerome, Alexander Greninger
EPI_ISL_424307 hCoV-19/USA/OR-UW-1885/2020 North America / USA / Oregon 2020-03-23 UW Virology Lab UW Virology Lab Pavitra Roychoudhury, Hong Xie, Keith Jerome, Alexander Greninger
EPI_ISL_424308 hCoV-19/USA/OR-UW-1887/2020 North America / USA / Oregon 2020-03-22 UW Virology Lab UW Virology Lab Pavitra Roychoudhury, Hong Xie, Keith Jerome, Alexander Greninger
EPI_ISL_424309 hCoV-19/USA/WA-UW-1881/2020 North America / USA / Washington 2020-03-24 UW Virology Lab UW Virology Lab Pavitra Roychoudhury, Hong Xie, Keith Jerome, Alexander Greninger
EPI_ISL_424310 hCoV-19/USA/WA-UW-1888/2020 North America / USA / Washington 2020-03-23 UW Virology Lab UW Virology Lab Pavitra Roychoudhury, Hong Xie, Keith Jerome, Alexander Greninger
EPI_ISL_424311 hCoV-19/USA/WA-UW-1880/2020 North America / USA / Washington 2020-03-24 UW Virology Lab UW Virology Lab Pavitra Roychoudhury, Hong Xie, Keith Jerome, Alexander Greninger
EPI_ISL_424312 hCoV-19/USA/WA-UW-1955/2020 North America / USA / Washington 2020-03-24 UW Virology Lab UW Virology Lab Pavitra Roychoudhury, Hong Xie, Keith Jerome, Alexander Greninger
EPI_ISL_424313 hCoV-19/USA/WA-UW-1956/2020 North America / USA / Washington 2020-03-24 UW Virology Lab UW Virology Lab Pavitra Roychoudhury, Hong Xie, Keith Jerome, Alexander Greninger
EPI_ISL_424314 hCoV-19/USA/WA-UW-1921/2020 North America / USA / Washington 2020-03-24 UW Virology Lab UW Virology Lab Pavitra Roychoudhury, Hong Xie, Keith Jerome, Alexander Greninger
EPI_ISL_424315 hCoV-19/USA/ID-UW-1957/2020 North America / USA / Idaho 2020-03-20 UW Virology Lab UW Virology Lab Pavitra Roychoudhury, Hong Xie, Keith Jerome, Alexander Greninger
EPI_ISL_424316 hCoV-19/USA/WA-UW-1890/2020 North America / USA / Washington 2020-03-24 UW Virology Lab UW Virology Lab Pavitra Roychoudhury, Hong Xie, Keith Jerome, Alexander Greninger
EPI_ISL_424317 hCoV-19/USA/WA-UW-1928/2020 North America / USA / Washington 2020-03-24 UW Virology Lab UW Virology Lab Pavitra Roychoudhury, Hong Xie, Keith Jerome, Alexander Greninger
EPI_ISL_424318 hCoV-19/USA/ID-UW-1938/2020 North America / USA / Idaho 2020-03-22 UW Virology Lab UW Virology Lab Pavitra Roychoudhury, Hong Xie, Keith Jerome, Alexander Greninger
EPI_ISL_424319 hCoV-19/USA/ID-UW-1935/2020 North America / USA / Idaho 2020-03-22 UW Virology Lab UW Virology Lab Pavitra Roychoudhury, Hong Xie, Keith Jerome, Alexander Greninger
EPI_ISL_424321 hCoV-19/USA/ID-UW-1925/2020 North America / USA / Idaho 2020-03-20 UW Virology Lab UW Virology Lab Pavitra Roychoudhury, Hong Xie, Keith Jerome, Alexander Greninger
EPI_ISL_424322 hCoV-19/USA/ID-UW-1934/2020 North America / USA / Idaho 2020-03-20 UW Virology Lab UW Virology Lab Pavitra Roychoudhury, Hong Xie, Keith Jerome, Alexander Greninger
EPI_ISL_424323 hCoV-19/USA/WA-UW-1944/2020 North America / USA / Washington 2020-03-24 UW Virology Lab UW Virology Lab Pavitra Roychoudhury, Hong Xie, Keith Jerome, Alexander Greninger
EPI_ISL_424324 hCoV-19/USA/WA-UW-1965/2020 North America / USA / Washington 2020-03-24 UW Virology Lab UW Virology Lab Pavitra Roychoudhury, Hong Xie, Keith Jerome, Alexander Greninger

EPI_ISL_424325 hCoV-19/USA/WA-UW-1954/2020 North America / USA / Washington 2020-03-24 UW Virology Lab UW Virology Lab Pavitra Roychoudhury, Hong Xie, Keith Jerome, Alexander Greninger
EPI_ISL_424326 hCoV-19/USA/WA-UW-1968/2020 North America / USA / Washington 2020-03-20 UW Virology Lab UW Virology Lab Pavitra Roychoudhury, Hong Xie, Keith Jerome, Alexander Greninger
EPI_ISL_424327 hCoV-19/USA/WA-UW-1947/2020 North America / USA / Washington 2020-03-23 UW Virology Lab UW Virology Lab Pavitra Roychoudhury, Hong Xie, Keith Jerome, Alexander Greninger
EPI_ISL_424329 hCoV-19/USA/WA-UW-1984/2020 North America / USA / Washington 2020-03-24 UW Virology Lab UW Virology Lab Pavitra Roychoudhury, Hong Xie, Keith Jerome, Alexander Greninger
EPI_ISL_424330 hCoV-19/USA/WA-UW-1865/2020 North America / USA / Washington 2020-03-23 UW Virology Lab UW Virology Lab Pavitra Roychoudhury, Hong Xie, Keith Jerome, Alexander Greninger
EPI_ISL_424331 hCoV-19/USA/WA-UW-1907/2020 North America / USA / Washington 2020-03-22 UW Virology Lab UW Virology Lab Pavitra Roychoudhury, Hong Xie, Keith Jerome, Alexander Greninger
EPI_ISL_424332 hCoV-19/USA/WA-UW-1917/2020 North America / USA / Washington 2020-03-22 UW Virology Lab UW Virology Lab Pavitra Roychoudhury, Hong Xie, Keith Jerome, Alexander Greninger
EPI_ISL_424333 hCoV-19/USA/WA-UW-2006/2020 North America / USA / Washington 2020-03-24 UW Virology Lab UW Virology Lab Pavitra Roychoudhury, Hong Xie, Keith Jerome, Alexander Greninger
EPI_ISL_424334 hCoV-19/USA/WA-UW-1994/2020 North America / USA / Washington 2020-03-24 UW Virology Lab UW Virology Lab Pavitra Roychoudhury, Hong Xie, Keith Jerome, Alexander Greninger
EPI_ISL_424335 hCoV-19/USA/WA-UW-1998/2020 North America / USA / Washington 2020-03-24 UW Virology Lab UW Virology Lab Pavitra Roychoudhury, Hong Xie, Keith Jerome, Alexander Greninger
EPI_ISL_424336 hCoV-19/USA/WA-UW-1988/2020 North America / USA / Washington 2020-03-24 UW Virology Lab UW Virology Lab Pavitra Roychoudhury, Hong Xie, Keith Jerome, Alexander Greninger
EPI_ISL_424337 hCoV-19/USA/WA-UW-1977/2020 North America / USA / Washington 2020-03-24 UW Virology Lab UW Virology Lab Pavitra Roychoudhury, Hong Xie, Keith Jerome, Alexander Greninger
EPI_ISL_424338 hCoV-19/USA/WA-UW-1986/2020 North America / USA / Washington 2020-03-24 UW Virology Lab UW Virology Lab Pavitra Roychoudhury, Hong Xie, Keith Jerome, Alexander Greninger
EPI_ISL_424339 hCoV-19/USA/WA-UW-2016/2020 North America / USA / Washington 2020-03-24 UW Virology Lab UW Virology Lab Pavitra Roychoudhury, Hong Xie, Keith Jerome, Alexander Greninger
EPI_ISL_424340 hCoV-19/USA/WA-UW-2009/2020 North America / USA / Washington 2020-03-24 UW Virology Lab UW Virology Lab Pavitra Roychoudhury, Hong Xie, Keith Jerome, Alexander Greninger
EPI_ISL_424341 hCoV-19/USA/WA-UW-2008/2020 North America / USA / Washington 2020-03-24 UW Virology Lab UW Virology Lab Pavitra Roychoudhury, Hong Xie, Keith Jerome, Alexander Greninger
EPI_ISL_424342 hCoV-19/Italy/INMI8/2020 Europe / Italy / Rome 2020-03-07 INMI Lazzaro Spallanzani IRCCS Laboratory of Virology, INMI Lazzaro Spallanzani IRCCS Concetta Castilletti, Barbara Bartolini, Martina Rueca, Cesare Ernesto Maria Gruber, Francesco Messina, Fabrizio Carletti, Eleonora Lalle, Licia Bordi, Giulia Matusali, Francesca Colavita, Maria Rosaria Capobianchi, Francesco Vairo, Giuseppe Ippolito, Antonino Di Caro
EPI_ISL_424343 hCoV-19/Italy/INMI9/2020 Europe / Italy / Rome 2020-03-23 INMI Lazzaro Spallanzani IRCCS Laboratory of Virology, INMI Lazzaro Spallanzani IRCCS Fabrizio Carletti, Barbara Bartolini, Martina Rueca, Cesare Ernesto Maria Gruber, Francesco Messina, Eleonora Lalle, Licia Bordi, Giulia Matusali, Francesca Colavita, Maria Rosaria Capobianchi, Concetta Castilletti, Francesco Vairo, Giuseppe Ippolito, Antonino Di Caro
EPI_ISL_424344 hCoV-19/Italy/INMI10/2020 Europe / Italy / Rome 2020-03-04 INMI Lazzaro Spallanzani IRCCS Laboratory of Virology, INMI Lazzaro Spallanzani IRCCS Eleonora Lalle, Barbara Bartolini, Martina Rueca, Cesare Ernesto Maria Gruber, Francesco Messina, Fabrizio Carletti, Licia Bordi, Giulia Matusali, Francesca Colavita, Maria Rosaria Capobianchi, Concetta Castilletti, Francesco Vairo, Giuseppe Ippolito, Antonino Di Caro
EPI_ISL_424345 hCoV-19/Mexico/CDMX-INER_01/2020 North America / Mexico / Mexico City 2020-03-12 Instituto Nacional de Enfermedades Respiratorias Instituto Nacional de Enfermedades Respiratorias Joel Armando Vázquez Pérez, Celia Boukadida, Santiago Avila Ríos, Mario Mújica Sánchez, José Arturo Martínez Orozco, Eduardo Becerril Vargas, Jorge Salas Hernández, Irma López Martínez, Lucia Hernández Rivas, Gisela Barrera Badillo, Edgar Mendieta Condado, Fabiola Garcés Ayala, Adnan Araiza Rodríguez, José Ernesto Ramírez González, Victor Hugo Borja Aburto, Concepción Grajales Muñiz, Cesar Raúl González Bonilla, Carolina González Torres, Francisco Javier Gaytán Cervantes, José Esteban Muñoz Medina, Guillermo M. Ruiz-Palacios, Pilar Ramos Cervantes, Violeta Ibarra Gonzalez, Fernando Ledesma Barrientos, Luis Alberto García Andrade, Alfredo Ponce de León Garduño, Blanca Taboada, Alejandro Sánchez, Pavel Isa, Ricardo Grande, Gloria Vázquez, Francisco Pulido, Carlos F. Arias.
EPI_ISL_424346 hCoV-19/USA/AK-PHL037/2020 North America / USA / Alaska / Southeast 2020-03-21 Alaska State Virology Laboratory Alaska State Virology Laboratory Chen, J.
EPI_ISL_424347 hCoV-19/USA/AK-PHL077/2020 North America / USA / Alaska / Interior 2020-03-29 Alaska State Virology Laboratory Alaska State Virology Laboratory Chen, J.
EPI_ISL_424352 hCoV-19/Fuyang/FY002/2020 Asia / China / Anhui / Fuyang 2020-03-10 unknown Clinical Laboratory Ge,B.
EPI_ISL_424353 hCoV-19/USA/NC_200173/2020 North America / USA / North Carolina 2020-03 Dirk Dittmer Dirk Dittmer Bailey,A.G., Caro-Vegas,C., Thompson,C., Dittmer,D., Eason,A.B., Juarez,A., Landis,J.T., McNamara,R.P., Miller,M.B., Moorad,R., Pluta,L.J., Seltzer,T.A., Villamor,F. and Vahrson,W.
EPI_ISL_424354 hCoV-19/USA/NC_200189/2020 North America / USA / North Carolina 2020-03 Dirk Dittmer Dirk Dittmer Bailey,A.G., Caro-Vegas,C.P., Dittmer,D., Eason,A.B., Juarez,A., Landis,J.T., McNamara,R.P., Miller,M.B., Moorad,R., Pluta,L.J., Seltzer,T.A., Thompson,C., Vahrson,W. and Villamor,F.
EPI_ISL_424355 hCoV-19/Beijing/Wuhan_IME-BJ01/2020 Asia / China / Beijing 2020-01-24 unknown Beijing

Institute of Microbiology and Epidemiology	Fan,H., Qin,E., Wu,Y., Guo,Y., Zhang,X., Yong,Y., Hou,J., Xu,Z., Mu,J., Teng,Y., Mi,Z., Yang,R., Song,Y., Li,B. and Cui,Y.
EPI_ISL_424356	hCoV-19/Beijing/Wuhan_IME-BJ02/2020	Asia / China / Beijing	2020-01-25	unknown Beijing Institute of Microbiology and Epidemiology	Fan,H., Qin,E., Wu,Y., Guo,Y., Zhang,X., Yong,Y., Hou,J., Xu,Z., Mu,J., Teng,Y., Mi,Z., Yang,R., Song,Y., Li,B. and Cui,Y.
EPI_ISL_424357	hCoV-19/Beijing/Wuhan_IME-BJ03/2020	Asia / China / Beijing	2020-01-28	unknown Beijing Institute of Microbiology and Epidemiology	Fan,H., Qin,E., Wu,Y., Guo,Y., Zhang,X., Yong,Y., Hou,J., Xu,Z., Mu,J., Teng,Y., Mi,Z., Yang,R., Song,Y., Li,B. and Cui,Y.
EPI_ISL_424358	hCoV-19/Beijing/Wuhan_IME-BJ04/2020	Asia / China / Beijing	2020-01-28	unknown Beijing Institute of Microbiology and Epidemiology	Fan,H., Qin,E., Wu,Y., Guo,Y., Zhang,X., Yong,Y., Hou,J., Xu,Z., Mu,J., Teng,Y., Mi,Z., Yang,R., Song,Y., Li,B. and Cui,Y.
EPI_ISL_424359	hCoV-19/Beijing/Wuhan_IME-BJ05/2020	Asia / China / Beijing	2020-01-27	unknown Beijing Institute of Microbiology and Epidemiology	Fan,H., Qin,E., Wu,Y., Guo,Y., Zhang,X., Yong,Y., Hou,J., Xu,Z., Mu,J., Teng,Y., Mi,Z., Yang,R., Song,Y., Li,B. and Cui,Y.
EPI_ISL_424360	hCoV-19/Beijing/Wuhan_IME-BJ07/2020	Asia / China / Beijing	2020-01-29	unknown Beijing Institute of Microbiology and Epidemiology	Fan,H., Qin,E., Wu,Y., Guo,Y., Zhang,X., Yong,Y., Hou,J., Xu,Z., Mu,J., Teng,Y., Mi,Z., Yang,R., Song,Y., Li,B. and Cui,Y.
EPI_ISL_424361	hCoV-19/India/1063/2020 Asia / India	2020-03-10	National Influenza Center, Indian Council of Medical Research - National Institute of Virology	Indian Council of Medical Research-National Institute of Virology, Microbial Containment Complex	Pragya D. Yadav, Varsha Potdar, Savita Patil, Dimpal A. Nyayanit, Triparna Majumdar, Manohar. L. Chaudhary, Gururaj Deshpande, Padinjaremattathil Thankappan Ullas, Anita Shete-Aich, Hitesh Dighe, Sreelekshmy Mohandas, Gajanan Sapkal, Atanu Basu, Amita Jain, Bharti Malhotra, Deepika Chaudhary, Sarah Cherian, Priya Abraham
EPI_ISL_424362	hCoV-19/India/1135/2020 Asia / India	2020-03-10	National Influenza Center, Indian Council of Medical Research - National Institute of Virology	Indian Council of Medical Research-National Institute of Virology, Microbial Containment Complex	Pragya D. Yadav, Varsha Potdar, Savita Patil, Dimpal A. Nyayanit, Triparna Majumdar, Manohar. L. Chaudhary, Gururaj Deshpande, Padinjaremattathil Thankappan Ullas, Anita Shete-Aich, Hitesh Dighe, Sreelekshmy Mohandas, Gajanan Sapkal, Atanu Basu, Amita Jain, Bharti Malhotra, Deepika Chaudhary, Sarah Cherian, Priya Abraham
EPI_ISL_424363	hCoV-19/India/1652/2020 Asia / India	2020-03-12	National Influenza Center, Indian Council of Medical Research - National Institute of Virology	Indian Council of Medical Research-National Institute of Virology, Microbial Containment Complex	Pragya D. Yadav, Varsha Potdar, Savita Patil, Dimpal A. Nyayanit, Triparna Majumdar, Manohar. L. Chaudhary, Gururaj Deshpande, Padinjaremattathil Thankappan Ullas, Anita Shete-Aich, Hitesh Dighe, Sreelekshmy Mohandas, Gajanan Sapkal, Atanu Basu, Amita Jain, Bharti Malhotra, Deepika Chaudhary, Sarah Cherian, Priya Abraham
EPI_ISL_424364	hCoV-19/India/3118/2020 Asia / India	2020-03-17	National Influenza Center, Indian Council of Medical Research - National Institute of Virology	Indian Council of Medical Research-National Institute of Virology, Microbial Containment Complex	Pragya D. Yadav, Varsha Potdar, Savita Patil, Dimpal A. Nyayanit, Triparna Majumdar, Manohar. L. Chaudhary, Gururaj Deshpande, Padinjaremattathil Thankappan Ullas, Anita Shete-Aich, Hitesh Dighe, Sreelekshmy Mohandas, Gajanan Sapkal, Atanu Basu, Amita Jain, Bharti Malhotra, Deepika Chaudhary, Sarah Cherian, Priya Abraham
EPI_ISL_424365	hCoV-19/India/3239/2020 Asia / India	2020-03-17	National Influenza Center, Indian Council of Medical Research - National Institute of Virology	Indian Council of Medical Research-National Institute of Virology, Microbial Containment Complex	Pragya D. Yadav, Varsha Potdar, Savita Patil, Dimpal A. Nyayanit, Triparna Majumdar, Manohar. L. Chaudhary, Gururaj Deshpande, Padinjaremattathil Thankappan Ullas, Anita Shete-Aich, Hitesh Dighe, Sreelekshmy Mohandas, Gajanan Sapkal, Atanu Basu, Amita Jain, Bharti Malhotra, Deepika Chaudhary, Sarah Cherian, Priya Abraham
EPI_ISL_424366	hCoV-19/Turkey/ERAGEM-001/2020	Europe / Turkey / Kayseri	2020-03-17	Vaccine Research, Development and Application Center, Erciyes University	Gen Era Diagnostics Inc.	Shaikh Terkis Islam Pavel, Hazel Yetiskin, Gunsu Aydin, Can Holyavkin, Muhammet Ali Uygut, Zehra B Dursun, İlhami Celik, Alper Iseri, Aykut Ozdarendeli
EPI_ISL_424367	hCoV-19/Iceland/343/2020	Europe / Iceland / Reykjavik	2020-03 The National University Hospital of Iceland	deCODE genetics Daniel F Gudbjartsson; Agnar Helgason; Hakon Jonsson; Olafur T Magnusson; Pall Melsted; Gudmundur L Norddahl; Jona Saemundsdottir; Asgeir Sigurdsson; Patrick Sulem; Arna B Agustsdottir; Berglind Eiriksdottir; Run Fridriksdottir; Elisabet E Gardarsdottir; Gudmundur Georgsson; Olafia S Gretarsdottir; Kjartan R Gudmundsson; Thora R Gunnarsdottir; Arnaldur Gylfason; Hilma Holm; Brynjar O Jensson; Aslaug Jonasdottir; Kamilla S Josefsdottir; Thordur Kristjansson; Droplaug N Magnusdottir; Louise le Roux; Gudrun Sigmundsdottir; Gardar Sveinbjornsson; Kristin E Sveinsdottir; Maney Sveinsdottir; Emil A Thorarensen; Bjarni Thorbjornsson; Gisli Masson; Ingileif Jonsdottir; Alma Moller; Thorolfur Gudnason; Karl G Kristinsson; Unnur Thorsteinsdottir; Kari Stefansson
EPI_ISL_424368	hCoV-19/Iceland/344/2020	Europe / Iceland / Reykjavik	2020-03 The National University Hospital of Iceland	deCODE genetics Daniel F Gudbjartsson; Agnar Helgason; Hakon Jonsson; Olafur T Magnusson; Pall Melsted; Gudmundur L Norddahl; Jona Saemundsdottir; Asgeir Sigurdsson; Patrick Sulem; Arna B Agustsdottir; Berglind Eiriksdottir; Run Fridriksdottir; Elisabet E Gardarsdottir; Gudmundur Georgsson; Olafia S Gretarsdottir; Kjartan R Gudmundsson; Thora R Gunnarsdottir; Arnaldur Gylfason; Hilma Holm; Brynjar O Jensson; Aslaug Jonasdottir; Kamilla S Josefsdottir; Thordur Kristjansson; Droplaug N Magnusdottir; Louise le Roux; Gudrun Sigmundsdottir; Gardar Sveinbjornsson; Kristin E Sveinsdottir; Maney Sveinsdottir; Emil A Thorarensen; Bjarni Thorbjornsson; Gisli Masson; Ingileif Jonsdottir; Alma Moller; Thorolfur Gudnason; Karl G Kristinsson; Unnur Thorsteinsdottir; Kari Stefansson
EPI_ISL_424369	hCoV-19/Iceland/345/2020	Europe / Iceland / Reykjavik	2020-03 The National University

Hospital of Iceland deCODE genetics Daniel F Gudbjartsson; Agnar Helgason; Hakon Jonsson; Olafur T Magnusson; Pall Melsted; Gudmundur L Norddahl; Jona Saemundsdottir; Asgeir Sigurdsson; Patrick Sulem; Arna B Agustsdottir; Berglind Eiriksdottir; Run Fridriksdottir; Elisabet E Gardarsdottir; Gudmundur Georgsson; Olafia S Gretarsdottir; Kjartan R Gudmundsson; Thora R Gunnarsdottir; Arnaldur Gylfason; Hilma Holm; Brynjar O Jensson; Aslaug Jonasdottir; Kamilla S Josefsdottir; Thordur Kristjansson; Droplaug N Magnusdottir; Louise le Roux; Gudrun Sigmundsdottir; Gardar Sveinbjornsson; Kristin E Sveinsdottir; Maney Sveinsdottir; Emil A Thorarensen; Bjarni Thorbjornsson; Gisli Masson; Ingileif Jonsdottir; Alma Moller; Thorolfur Gudnason; Karl G Kristinsson; Unnur Thorsteinsdottir; Kari Stefansson
EPI_ISL_424370 hCoV-19/Iceland/346/2020 Europe / Iceland / Reykjavik 2020-03 The National University Hospital of Iceland deCODE genetics Daniel F Gudbjartsson; Agnar Helgason; Hakon Jonsson; Olafur T Magnusson; Pall Melsted; Gudmundur L Norddahl; Jona Saemundsdottir; Asgeir Sigurdsson; Patrick Sulem; Arna B Agustsdottir; Berglind Eiriksdottir; Run Fridriksdottir; Elisabet E Gardarsdottir; Gudmundur Georgsson; Olafia S Gretarsdottir; Kjartan R Gudmundsson; Thora R Gunnarsdottir; Arnaldur Gylfason; Hilma Holm; Brynjar O Jensson; Aslaug Jonasdottir; Kamilla S Josefsdottir; Thordur Kristjansson; Droplaug N Magnusdottir; Louise le Roux; Gudrun Sigmundsdottir; Gardar Sveinbjornsson; Kristin E Sveinsdottir; Maney Sveinsdottir; Emil A Thorarensen; Bjarni Thorbjornsson; Gisli Masson; Ingileif Jonsdottir; Alma Moller; Thorolfur Gudnason; Karl G Kristinsson; Unnur Thorsteinsdottir; Kari Stefansson
EPI_ISL_424371 hCoV-19/Iceland/347/2020 Europe / Iceland / Reykjavik 2020-03 The National University Hospital of Iceland deCODE genetics Daniel F Gudbjartsson; Agnar Helgason; Hakon Jonsson; Olafur T Magnusson; Pall Melsted; Gudmundur L Norddahl; Jona Saemundsdottir; Asgeir Sigurdsson; Patrick Sulem; Arna B Agustsdottir; Berglind Eiriksdottir; Run Fridriksdottir; Elisabet E Gardarsdottir; Gudmundur Georgsson; Olafia S Gretarsdottir; Kjartan R Gudmundsson; Thora R Gunnarsdottir; Arnaldur Gylfason; Hilma Holm; Brynjar O Jensson; Aslaug Jonasdottir; Kamilla S Josefsdottir; Thordur Kristjansson; Droplaug N Magnusdottir; Louise le Roux; Gudrun Sigmundsdottir; Gardar Sveinbjornsson; Kristin E Sveinsdottir; Maney Sveinsdottir; Emil A Thorarensen; Bjarni Thorbjornsson; Gisli Masson; Ingileif Jonsdottir; Alma Moller; Thorolfur Gudnason; Karl G Kristinsson; Unnur Thorsteinsdottir; Kari Stefansson
EPI_ISL_424377 hCoV-19/Iceland/353/2020 Europe / Iceland / Reykjavik 2020-03-18 deCODE genetics deCODE genetics Daniel F Gudbjartsson; Agnar Helgason; Hakon Jonsson; Olafur T Magnusson; Pall Melsted; Gudmundur L Norddahl; Jona Saemundsdottir; Asgeir Sigurdsson; Patrick Sulem; Arna B Agustsdottir; Berglind Eiriksdottir; Run Fridriksdottir; Elisabet E Gardarsdottir; Gudmundur Georgsson; Olafia S Gretarsdottir; Kjartan R Gudmundsson; Thora R Gunnarsdottir; Arnaldur Gylfason; Hilma Holm; Brynjar O Jensson; Aslaug Jonasdottir; Kamilla S Josefsdottir; Thordur Kristjansson; Droplaug N Magnusdottir; Louise le Roux; Gudrun Sigmundsdottir; Gardar Sveinbjornsson; Kristin E Sveinsdottir; Maney Sveinsdottir; Emil A Thorarensen; Bjarni Thorbjornsson; Gisli Masson; Ingileif Jonsdottir; Alma Moller; Thorolfur Gudnason; Karl G Kristinsson; Unnur Thorsteinsdottir; Kari Stefansson
EPI_ISL_424379 hCoV-19/Iceland/355/2020 Europe / Iceland / Reykjavik 2020-03-19 The National University Hospital of Iceland deCODE genetics Daniel F Gudbjartsson; Agnar Helgason; Hakon Jonsson; Olafur T Magnusson; Pall Melsted; Gudmundur L Norddahl; Jona Saemundsdottir; Asgeir Sigurdsson; Patrick Sulem; Arna B Agustsdottir; Berglind Eiriksdottir; Run Fridriksdottir; Elisabet E Gardarsdottir; Gudmundur Georgsson; Olafia S Gretarsdottir; Kjartan R Gudmundsson; Thora R Gunnarsdottir; Arnaldur Gylfason; Hilma Holm; Brynjar O Jensson; Aslaug Jonasdottir; Kamilla S Josefsdottir; Thordur Kristjansson; Droplaug N Magnusdottir; Louise le Roux; Gudrun Sigmundsdottir; Gardar Sveinbjornsson; Kristin E Sveinsdottir; Maney Sveinsdottir; Emil A Thorarensen; Bjarni Thorbjornsson; Gisli Masson; Ingileif Jonsdottir; Alma Moller; Thorolfur Gudnason; Karl G Kristinsson; Unnur Thorsteinsdottir; Kari Stefansson
EPI_ISL_424380 hCoV-19/Iceland/356/2020 Europe / Iceland / Reykjavik 2020-03-19 The National University Hospital of Iceland deCODE genetics Daniel F Gudbjartsson; Agnar Helgason; Hakon Jonsson; Olafur T Magnusson; Pall Melsted; Gudmundur L Norddahl; Jona Saemundsdottir; Asgeir Sigurdsson; Patrick Sulem; Arna B Agustsdottir; Berglind Eiriksdottir; Run Fridriksdottir; Elisabet E Gardarsdottir; Gudmundur Georgsson; Olafia S Gretarsdottir; Kjartan R Gudmundsson; Thora R Gunnarsdottir; Arnaldur Gylfason; Hilma Holm; Brynjar O Jensson; Aslaug Jonasdottir; Kamilla S Josefsdottir; Thordur Kristjansson; Droplaug N Magnusdottir; Louise le Roux; Gudrun Sigmundsdottir; Gardar Sveinbjornsson; Kristin E Sveinsdottir; Maney Sveinsdottir; Emil A Thorarensen; Bjarni Thorbjornsson; Gisli Masson; Ingileif Jonsdottir; Alma Moller; Thorolfur Gudnason; Karl G Kristinsson; Unnur Thorsteinsdottir; Kari Stefansson
EPI_ISL_424381 hCoV-19/Iceland/357/2020 Europe / Iceland / Reykjavik 2020-03-19 The National University Hospital of Iceland deCODE genetics Daniel F Gudbjartsson; Agnar Helgason; Hakon Jonsson; Olafur T Magnusson; Pall Melsted; Gudmundur L Norddahl; Jona Saemundsdottir; Asgeir Sigurdsson; Patrick Sulem; Arna B Agustsdottir; Berglind Eiriksdottir; Run Fridriksdottir; Elisabet E Gardarsdottir; Gudmundur Georgsson; Olafia S Gretarsdottir; Kjartan R Gudmundsson; Thora R Gunnarsdottir; Arnaldur Gylfason; Hilma Holm; Brynjar O Jensson; Aslaug Jonasdottir; Kamilla S Josefsdottir; Thordur Kristjansson; Droplaug N Magnusdottir; Louise le Roux; Gudrun Sigmundsdottir; Gardar Sveinbjornsson; Kristin E Sveinsdottir; Maney Sveinsdottir; Emil A Thorarensen; Bjarni Thorbjornsson; Gisli Masson; Ingileif Jonsdottir; Alma Moller; Thorolfur Gudnason; Karl G Kristinsson; Unnur Thorsteinsdottir; Kari Stefansson
EPI_ISL_424382 hCoV-19/Iceland/358/2020 Europe / Iceland / Reykjavik 2020-03-19 The National University Hospital of Iceland deCODE genetics Daniel F Gudbjartsson; Agnar Helgason; Hakon Jonsson; Olafur T Magnusson; Pall Melsted; Gudmundur L Norddahl; Jona Saemundsdottir; Asgeir Sigurdsson; Patrick Sulem; Arna B Agustsdottir; Berglind Eiriksdottir; Run Fridriksdottir; Elisabet E Gardarsdottir; Gudmundur Georgsson; Olafia S Gretarsdottir; Kjartan R Gudmundsson; Thora R Gunnarsdottir; Arnaldur Gylfason; Hilma Holm; Brynjar O Jensson; Aslaug Jonasdottir; Kamilla S Josefsdottir; Thordur Kristjansson; Droplaug N Magnusdottir; Louise le Roux; Gudrun Sigmundsdottir; Gardar Sveinbjornsson; Kristin E Sveinsdottir; Maney Sveinsdottir; Emil A Thorarensen; Bjarni Thorbjornsson; Gisli Masson; Ingileif Jonsdottir; Alma Moller; Thorolfur Gudnason; Karl G

Kristinsson; Unnur Thorsteinsdottir; Kari Stefansson
EPI_ISL_424383 hCoV-19/Iceland/359/2020 Europe / Iceland / Reykjavik 2020-03-19 The National University Hospital of Iceland deCODE genetics Daniel F Gudbjartsson; Agnar Helgason; Hakon Jonsson; Olafur T Magnusson; Pall Melsted; Gudmundur L Norddahl; Jona Saemundsdottir; Asgeir Sigurdsson; Patrick Sulem; Arna B Agustsdottir; Berglind Eiriksdottir; Run Fridriksdottir; Elisabet E Gardarsdottir; Gudmundur Georgsson; Olafia S Gretarsdottir; Kjartan R Gudmundsson; Thora R Gunnarsdottir; Arnaldur Gylfason; Hilma Holm; Brynjar O Jensson; Aslaug Jonasdottir; Kamilla S Josefsdottir; Thordur Kristjansson; Droplaug N Magnusdottir; Louise le Roux; Gudrun Sigmundsdottir; Gardar Sveinbjornsson; Kristin E Sveinsdottir; Maney Sveinsdottir; Emil A Thorarensen; Bjarni Thorbjornsson; Gisli Masson; Ingileif Jonsdottir; Alma Moller; Thorolfur Gudnason; Karl G Kristinsson; Unnur Thorsteinsdottir; Kari Stefansson
EPI_ISL_424384 hCoV-19/Iceland/360/2020 Europe / Iceland / Reykjavik 2020-03-19 The National University Hospital of Iceland deCODE genetics Daniel F Gudbjartsson; Agnar Helgason; Hakon Jonsson; Olafur T Magnusson; Pall Melsted; Gudmundur L Norddahl; Jona Saemundsdottir; Asgeir Sigurdsson; Patrick Sulem; Arna B Agustsdottir; Berglind Eiriksdottir; Run Fridriksdottir; Elisabet E Gardarsdottir; Gudmundur Georgsson; Olafia S Gretarsdottir; Kjartan R Gudmundsson; Thora R Gunnarsdottir; Arnaldur Gylfason; Hilma Holm; Brynjar O Jensson; Aslaug Jonasdottir; Kamilla S Josefsdottir; Thordur Kristjansson; Droplaug N Magnusdottir; Louise le Roux; Gudrun Sigmundsdottir; Gardar Sveinbjornsson; Kristin E Sveinsdottir; Maney Sveinsdottir; Emil A Thorarensen; Bjarni Thorbjornsson; Gisli Masson; Ingileif Jonsdottir; Alma Moller; Thorolfur Gudnason; Karl G Kristinsson; Unnur Thorsteinsdottir; Kari Stefansson
EPI_ISL_424385 hCoV-19/Iceland/361/2020 Europe / Iceland / Reykjavik 2020-03-19 The National University Hospital of Iceland deCODE genetics Daniel F Gudbjartsson; Agnar Helgason; Hakon Jonsson; Olafur T Magnusson; Pall Melsted; Gudmundur L Norddahl; Jona Saemundsdottir; Asgeir Sigurdsson; Patrick Sulem; Arna B Agustsdottir; Berglind Eiriksdottir; Run Fridriksdottir; Elisabet E Gardarsdottir; Gudmundur Georgsson; Olafia S Gretarsdottir; Kjartan R Gudmundsson; Thora R Gunnarsdottir; Arnaldur Gylfason; Hilma Holm; Brynjar O Jensson; Aslaug Jonasdottir; Kamilla S Josefsdottir; Thordur Kristjansson; Droplaug N Magnusdottir; Louise le Roux; Gudrun Sigmundsdottir; Gardar Sveinbjornsson; Kristin E Sveinsdottir; Maney Sveinsdottir; Emil A Thorarensen; Bjarni Thorbjornsson; Gisli Masson; Ingileif Jonsdottir; Alma Moller; Thorolfur Gudnason; Karl G Kristinsson; Unnur Thorsteinsdottir; Kari Stefansson
EPI_ISL_424388 hCoV-19/Iceland/364/2020 Europe / Iceland / Reykjavik 2020-03-19 The National University Hospital of Iceland deCODE genetics Daniel F Gudbjartsson; Agnar Helgason; Hakon Jonsson; Olafur T Magnusson; Pall Melsted; Gudmundur L Norddahl; Jona Saemundsdottir; Asgeir Sigurdsson; Patrick Sulem; Arna B Agustsdottir; Berglind Eiriksdottir; Run Fridriksdottir; Elisabet E Gardarsdottir; Gudmundur Georgsson; Olafia S Gretarsdottir; Kjartan R Gudmundsson; Thora R Gunnarsdottir; Arnaldur Gylfason; Hilma Holm; Brynjar O Jensson; Aslaug Jonasdottir; Kamilla S Josefsdottir; Thordur Kristjansson; Droplaug N Magnusdottir; Louise le Roux; Gudrun Sigmundsdottir; Gardar Sveinbjornsson; Kristin E Sveinsdottir; Maney Sveinsdottir; Emil A Thorarensen; Bjarni Thorbjornsson; Gisli Masson; Ingileif Jonsdottir; Alma Moller; Thorolfur Gudnason; Karl G Kristinsson; Unnur Thorsteinsdottir; Kari Stefansson
EPI_ISL_424389 hCoV-19/Iceland/365/2020 Europe / Iceland / Reykjavik 2020-03-19 The National University Hospital of Iceland deCODE genetics Daniel F Gudbjartsson; Agnar Helgason; Hakon Jonsson; Olafur T Magnusson; Pall Melsted; Gudmundur L Norddahl; Jona Saemundsdottir; Asgeir Sigurdsson; Patrick Sulem; Arna B Agustsdottir; Berglind Eiriksdottir; Run Fridriksdottir; Elisabet E Gardarsdottir; Gudmundur Georgsson; Olafia S Gretarsdottir; Kjartan R Gudmundsson; Thora R Gunnarsdottir; Arnaldur Gylfason; Hilma Holm; Brynjar O Jensson; Aslaug Jonasdottir; Kamilla S Josefsdottir; Thordur Kristjansson; Droplaug N Magnusdottir; Louise le Roux; Gudrun Sigmundsdottir; Gardar Sveinbjornsson; Kristin E Sveinsdottir; Maney Sveinsdottir; Emil A Thorarensen; Bjarni Thorbjornsson; Gisli Masson; Ingileif Jonsdottir; Alma Moller; Thorolfur Gudnason; Karl G Kristinsson; Unnur Thorsteinsdottir; Kari Stefansson
EPI_ISL_424390 hCoV-19/Iceland/366/2020 Europe / Iceland / Reykjavik 2020-03-19 The National University Hospital of Iceland deCODE genetics Daniel F Gudbjartsson; Agnar Helgason; Hakon Jonsson; Olafur T Magnusson; Pall Melsted; Gudmundur L Norddahl; Jona Saemundsdottir; Asgeir Sigurdsson; Patrick Sulem; Arna B Agustsdottir; Berglind Eiriksdottir; Run Fridriksdottir; Elisabet E Gardarsdottir; Gudmundur Georgsson; Olafia S Gretarsdottir; Kjartan R Gudmundsson; Thora R Gunnarsdottir; Arnaldur Gylfason; Hilma Holm; Brynjar O Jensson; Aslaug Jonasdottir; Kamilla S Josefsdottir; Thordur Kristjansson; Droplaug N Magnusdottir; Louise le Roux; Gudrun Sigmundsdottir; Gardar Sveinbjornsson; Kristin E Sveinsdottir; Maney Sveinsdottir; Emil A Thorarensen; Bjarni Thorbjornsson; Gisli Masson; Ingileif Jonsdottir; Alma Moller; Thorolfur Gudnason; Karl G Kristinsson; Unnur Thorsteinsdottir; Kari Stefansson
EPI_ISL_424391 hCoV-19/Iceland/367/2020 Europe / Iceland / Reykjavik 2020-03-19 The National University Hospital of Iceland deCODE genetics Daniel F Gudbjartsson; Agnar Helgason; Hakon Jonsson; Olafur T Magnusson; Pall Melsted; Gudmundur L Norddahl; Jona Saemundsdottir; Asgeir Sigurdsson; Patrick Sulem; Arna B Agustsdottir; Berglind Eiriksdottir; Run Fridriksdottir; Elisabet E Gardarsdottir; Gudmundur Georgsson; Olafia S Gretarsdottir; Kjartan R Gudmundsson; Thora R Gunnarsdottir; Arnaldur Gylfason; Hilma Holm; Brynjar O Jensson; Aslaug Jonasdottir; Kamilla S Josefsdottir; Thordur Kristjansson; Droplaug N Magnusdottir; Louise le Roux; Gudrun Sigmundsdottir; Gardar Sveinbjornsson; Kristin E Sveinsdottir; Maney Sveinsdottir; Emil A Thorarensen; Bjarni Thorbjornsson; Gisli Masson; Ingileif Jonsdottir; Alma Moller; Thorolfur Gudnason; Karl G Kristinsson; Unnur Thorsteinsdottir; Kari Stefansson
EPI_ISL_424392 hCoV-19/Iceland/368/2020 Europe / Iceland / Reykjavik 2020-03-19 The National University Hospital of Iceland deCODE genetics Daniel F Gudbjartsson; Agnar Helgason; Hakon Jonsson; Olafur T Magnusson; Pall Melsted; Gudmundur L Norddahl; Jona Saemundsdottir; Asgeir Sigurdsson; Patrick Sulem; Arna B Agustsdottir; Berglind Eiriksdottir; Run Fridriksdottir; Elisabet E Gardarsdottir; Gudmundur Georgsson; Olafia S Gretarsdottir; Kjartan R Gudmundsson; Thora R Gunnarsdottir; Arnaldur Gylfason; Hilma Holm; Brynjar O Jensson; Aslaug Jonasdottir; Kamilla S Josefsdottir; Thordur Kristjansson; Droplaug N Magnusdottir; Louise le

Roux; Gudrun Sigmundsdottir; Gardar Sveinbjornsson; Kristin E Sveinsdottir; Maney Sveinsdottir; Emil A Thorarensen; Bjarni Thorbjornsson; Gisli Masson; Ingileif Jonsdottir; Alma Moller; Thorolfur Gudnason; Karl G Kristinsson; Unnur Thorsteinsdottir; Kari Stefansson
EPI_ISL_424393 hCoV-19/Iceland/369/2020 Europe / Iceland / Reykjavik 2020-03-19 The National University Hospital of Iceland deCODE genetics Daniel F Gudbjartsson; Agnar Helgason; Hakon Jonsson; Olafur T Magnusson; Pall Melsted; Gudmundur L Norddahl; Jona Saemundsdottir; Asgeir Sigurdsson; Patrick Sulem; Arna B Agustsdottir; Berglind Eiriksdottir; Run Fridriksdottir; Elisabet E Gardarsdottir; Gudmundur Georgsson; Olafia S Gretarsdottir; Kjartan R Gudmundsson; Thora R Gunnarsdottir; Arnaldur Gylfason; Hilma Holm; Brynjar O Jensson; Aslaug Jonasdottir; Kamilla S Josefsdottir; Thordur Kristjansson; Droplaug N Magnusdottir; Louise le Roux; Gudrun Sigmundsdottir; Gardar Sveinbjornsson; Kristin E Sveinsdottir; Maney Sveinsdottir; Emil A Thorarensen; Bjarni Thorbjornsson; Gisli Masson; Ingileif Jonsdottir; Alma Moller; Thorolfur Gudnason; Karl G Kristinsson; Unnur Thorsteinsdottir; Kari Stefansson
EPI_ISL_424394 hCoV-19/Iceland/370/2020 Europe / Iceland / Reykjavik 2020-03-19 The National University Hospital of Iceland deCODE genetics Daniel F Gudbjartsson; Agnar Helgason; Hakon Jonsson; Olafur T Magnusson; Pall Melsted; Gudmundur L Norddahl; Jona Saemundsdottir; Asgeir Sigurdsson; Patrick Sulem; Arna B Agustsdottir; Berglind Eiriksdottir; Run Fridriksdottir; Elisabet E Gardarsdottir; Gudmundur Georgsson; Olafia S Gretarsdottir; Kjartan R Gudmundsson; Thora R Gunnarsdottir; Arnaldur Gylfason; Hilma Holm; Brynjar O Jensson; Aslaug Jonasdottir; Kamilla S Josefsdottir; Thordur Kristjansson; Droplaug N Magnusdottir; Louise le Roux; Gudrun Sigmundsdottir; Gardar Sveinbjornsson; Kristin E Sveinsdottir; Maney Sveinsdottir; Emil A Thorarensen; Bjarni Thorbjornsson; Gisli Masson; Ingileif Jonsdottir; Alma Moller; Thorolfur Gudnason; Karl G Kristinsson; Unnur Thorsteinsdottir; Kari Stefansson
EPI_ISL_424396 hCoV-19/Iceland/372/2020 Europe / Iceland / Reykjavik 2020-03-19 The National University Hospital of Iceland deCODE genetics Daniel F Gudbjartsson; Agnar Helgason; Hakon Jonsson; Olafur T Magnusson; Pall Melsted; Gudmundur L Norddahl; Jona Saemundsdottir; Asgeir Sigurdsson; Patrick Sulem; Arna B Agustsdottir; Berglind Eiriksdottir; Run Fridriksdottir; Elisabet E Gardarsdottir; Gudmundur Georgsson; Olafia S Gretarsdottir; Kjartan R Gudmundsson; Thora R Gunnarsdottir; Arnaldur Gylfason; Hilma Holm; Brynjar O Jensson; Aslaug Jonasdottir; Kamilla S Josefsdottir; Thordur Kristjansson; Droplaug N Magnusdottir; Louise le Roux; Gudrun Sigmundsdottir; Gardar Sveinbjornsson; Kristin E Sveinsdottir; Maney Sveinsdottir; Emil A Thorarensen; Bjarni Thorbjornsson; Gisli Masson; Ingileif Jonsdottir; Alma Moller; Thorolfur Gudnason; Karl G Kristinsson; Unnur Thorsteinsdottir; Kari Stefansson
EPI_ISL_424398 hCoV-19/Iceland/374/2020 Europe / Iceland / Reykjavik 2020-03-19 The National University Hospital of Iceland deCODE genetics Daniel F Gudbjartsson; Agnar Helgason; Hakon Jonsson; Olafur T Magnusson; Pall Melsted; Gudmundur L Norddahl; Jona Saemundsdottir; Asgeir Sigurdsson; Patrick Sulem; Arna B Agustsdottir; Berglind Eiriksdottir; Run Fridriksdottir; Elisabet E Gardarsdottir; Gudmundur Georgsson; Olafia S Gretarsdottir; Kjartan R Gudmundsson; Thora R Gunnarsdottir; Arnaldur Gylfason; Hilma Holm; Brynjar O Jensson; Aslaug Jonasdottir; Kamilla S Josefsdottir; Thordur Kristjansson; Droplaug N Magnusdottir; Louise le Roux; Gudrun Sigmundsdottir; Gardar Sveinbjornsson; Kristin E Sveinsdottir; Maney Sveinsdottir; Emil A Thorarensen; Bjarni Thorbjornsson; Gisli Masson; Ingileif Jonsdottir; Alma Moller; Thorolfur Gudnason; Karl G Kristinsson; Unnur Thorsteinsdottir; Kari Stefansson
EPI_ISL_424399 hCoV-19/Iceland/375/2020 Europe / Iceland / Reykjavik 2020-03-19 The National University Hospital of Iceland deCODE genetics Daniel F Gudbjartsson; Agnar Helgason; Hakon Jonsson; Olafur T Magnusson; Pall Melsted; Gudmundur L Norddahl; Jona Saemundsdottir; Asgeir Sigurdsson; Patrick Sulem; Arna B Agustsdottir; Berglind Eiriksdottir; Run Fridriksdottir; Elisabet E Gardarsdottir; Gudmundur Georgsson; Olafia S Gretarsdottir; Kjartan R Gudmundsson; Thora R Gunnarsdottir; Arnaldur Gylfason; Hilma Holm; Brynjar O Jensson; Aslaug Jonasdottir; Kamilla S Josefsdottir; Thordur Kristjansson; Droplaug N Magnusdottir; Louise le Roux; Gudrun Sigmundsdottir; Gardar Sveinbjornsson; Kristin E Sveinsdottir; Maney Sveinsdottir; Emil A Thorarensen; Bjarni Thorbjornsson; Gisli Masson; Ingileif Jonsdottir; Alma Moller; Thorolfur Gudnason; Karl G Kristinsson; Unnur Thorsteinsdottir; Kari Stefansson
EPI_ISL_425308 hCoV-19/England/CAMB-74580/2020 Europe / United Kingdom / England 2020-03-28 Department of Pathology, University of Cambridge COVID-19 Genomics UK (COG-UK) Consortium Luke W Meredith, M. Estee Torok , Myra Hosmillo, William L. Hamilton, Martin D. Curran, Theresa Feltwell, Anna Yakovleva, Charlotte J. Houldcroft, Aminu S. Jahun, Sarah L. Caddy, Ian Goodfellow
EPI_ISL_425309 hCoV-19/England/CAMB-7459F/2020 Europe / United Kingdom / England 2020-03-30 Department of Pathology, University of Cambridge COVID-19 Genomics UK (COG-UK) Consortium Luke W Meredith, M. Estee Torok , Myra Hosmillo, William L. Hamilton, Martin D. Curran, Theresa Feltwell, Anna Yakovleva, Charlotte J. Houldcroft, Aminu S. Jahun, Sarah L. Caddy, Ian Goodfellow
EPI_ISL_425310 hCoV-19/England/CAMB-745AE/2020 Europe / United Kingdom / England 2020-03-27 Department of Pathology, University of Cambridge COVID-19 Genomics UK (COG-UK) Consortium Luke W Meredith, M. Estee Torok , Myra Hosmillo, William L. Hamilton, Martin D. Curran, Theresa Feltwell, Anna Yakovleva, Charlotte J. Houldcroft, Aminu S. Jahun, Sarah L. Caddy, Ian Goodfellow
EPI_ISL_425311 hCoV-19/England/CAMB-745BD/2020 Europe / United Kingdom / England 2020-03-27 Department of Pathology, University of Cambridge COVID-19 Genomics UK (COG-UK) Consortium Luke W Meredith, M. Estee Torok , Myra Hosmillo, William L. Hamilton, Martin D. Curran, Theresa Feltwell, Anna Yakovleva, Charlotte J. Houldcroft, Aminu S. Jahun, Sarah L. Caddy, Ian Goodfellow
EPI_ISL_425312 hCoV-19/England/CAMB-745CC/2020 Europe / United Kingdom / England 2020-03-29 Department of Pathology, University of Cambridge COVID-19 Genomics UK (COG-UK) Consortium Luke W Meredith, M. Estee Torok , Myra Hosmillo, William L. Hamilton, Martin D. Curran, Theresa Feltwell, Anna Yakovleva, Charlotte J. Houldcroft, Aminu S. Jahun, Sarah L. Caddy, Ian Goodfellow
EPI_ISL_425313 hCoV-19/England/CAMB-745DB/2020 Europe / United Kingdom / England 2020-03-28 Department of Pathology, University of Cambridge COVID-19 Genomics UK (COG-UK) Consortium Luke W

Meredith, M. Estee Torok , Myra Hosmillo, William L. Hamilton, Martin D. Curran, Theresa Feltwell, Anna Yakovleva, Charlotte J. Houldcroft, Aminu S. Jahun, Sarah L. Caddy, Ian Goodfellow
EPI_ISL_425314 hCoV-19/England/CAMB-745EA/2020 Europe / United Kingdom / England 2020-03-26
Department of Pathology, University of Cambridge COVID-19 Genomics UK (COG-UK) Consortium Luke W Meredith, M. Estee Torok , Myra Hosmillo, William L. Hamilton, Martin D. Curran, Theresa Feltwell, Anna Yakovleva, Charlotte J. Houldcroft, Aminu S. Jahun, Sarah L. Caddy, Ian Goodfellow
EPI_ISL_425315 hCoV-19/England/CAMB-74605/2020 Europe / United Kingdom / England 2020-03-24
Department of Pathology, University of Cambridge COVID-19 Genomics UK (COG-UK) Consortium Luke W Meredith, M. Estee Torok , Myra Hosmillo, William L. Hamilton, Martin D. Curran, Theresa Feltwell, Anna Yakovleva, Charlotte J. Houldcroft, Aminu S. Jahun, Sarah L. Caddy, Ian Goodfellow
EPI_ISL_425316 hCoV-19/England/CAMB-74614/2020 Europe / United Kingdom / England 2020-03-22
Department of Pathology, University of Cambridge COVID-19 Genomics UK (COG-UK) Consortium Luke W Meredith, M. Estee Torok , Myra Hosmillo, William L. Hamilton, Martin D. Curran, Theresa Feltwell, Anna Yakovleva, Charlotte J. Houldcroft, Aminu S. Jahun, Sarah L. Caddy, Ian Goodfellow
EPI_ISL_425317 hCoV-19/England/CAMB-74623/2020 Europe / United Kingdom / England 2020-03-22
Department of Pathology, University of Cambridge COVID-19 Genomics UK (COG-UK) Consortium Luke W Meredith, M. Estee Torok , Myra Hosmillo, William L. Hamilton, Martin D. Curran, Theresa Feltwell, Anna Yakovleva, Charlotte J. Houldcroft, Aminu S. Jahun, Sarah L. Caddy, Ian Goodfellow
EPI_ISL_425318 hCoV-19/England/CAMB-74641/2020 Europe / United Kingdom / England 2020-03-20
Department of Pathology, University of Cambridge COVID-19 Genomics UK (COG-UK) Consortium Luke W Meredith, M. Estee Torok , Myra Hosmillo, William L. Hamilton, Martin D. Curran, Theresa Feltwell, Anna Yakovleva, Charlotte J. Houldcroft, Aminu S. Jahun, Sarah L. Caddy, Ian Goodfellow
EPI_ISL_425319 hCoV-19/England/CAMB-74650/2020 Europe / United Kingdom / England 2020-03-28
Department of Pathology, University of Cambridge COVID-19 Genomics UK (COG-UK) Consortium Luke W Meredith, M. Estee Torok , Myra Hosmillo, William L. Hamilton, Martin D. Curran, Theresa Feltwell, Anna Yakovleva, Charlotte J. Houldcroft, Aminu S. Jahun, Sarah L. Caddy, Ian Goodfellow
EPI_ISL_425323 hCoV-19/England/CAMB-746AB/2020 Europe / United Kingdom / England 2020-03-29
Department of Pathology, University of Cambridge COVID-19 Genomics UK (COG-UK) Consortium Luke W Meredith, M. Estee Torok , Myra Hosmillo, William L. Hamilton, Martin D. Curran, Theresa Feltwell, Anna Yakovleva, Charlotte J. Houldcroft, Aminu S. Jahun, Sarah L. Caddy, Ian Goodfellow
EPI_ISL_425324 hCoV-19/England/CAMB-746BA/2020 Europe / United Kingdom / England 2020-03-29
Department of Pathology, University of Cambridge COVID-19 Genomics UK (COG-UK) Consortium Luke W Meredith, M. Estee Torok , Myra Hosmillo, William L. Hamilton, Martin D. Curran, Theresa Feltwell, Anna Yakovleva, Charlotte J. Houldcroft, Aminu S. Jahun, Sarah L. Caddy, Ian Goodfellow
EPI_ISL_425325 hCoV-19/England/CAMB-746D8/2020 Europe / United Kingdom / England 2020-03-29
Department of Pathology, University of Cambridge COVID-19 Genomics UK (COG-UK) Consortium Luke W Meredith, M. Estee Torok , Myra Hosmillo, William L. Hamilton, Martin D. Curran, Theresa Feltwell, Anna Yakovleva, Charlotte J. Houldcroft, Aminu S. Jahun, Sarah L. Caddy, Ian Goodfellow
EPI_ISL_425326 hCoV-19/England/CAMB-74702/2020 Europe / United Kingdom / England 2020-03-29
Department of Pathology, University of Cambridge COVID-19 Genomics UK (COG-UK) Consortium Luke W Meredith, M. Estee Torok , Myra Hosmillo, William L. Hamilton, Martin D. Curran, Theresa Feltwell, Anna Yakovleva, Charlotte J. Houldcroft, Aminu S. Jahun, Sarah L. Caddy, Ian Goodfellow
EPI_ISL_425327 hCoV-19/England/CAMB-74711/2020 Europe / United Kingdom / England 2020-03-29
Department of Pathology, University of Cambridge COVID-19 Genomics UK (COG-UK) Consortium Luke W Meredith, M. Estee Torok , Myra Hosmillo, William L. Hamilton, Martin D. Curran, Theresa Feltwell, Anna Yakovleva, Charlotte J. Houldcroft, Aminu S. Jahun, Sarah L. Caddy, Ian Goodfellow
EPI_ISL_425331 hCoV-19/England/CAMB-7475D/2020 Europe / United Kingdom / England 2020-03-29
Department of Pathology, University of Cambridge COVID-19 Genomics UK (COG-UK) Consortium Luke W Meredith, M. Estee Torok , Myra Hosmillo, William L. Hamilton, Martin D. Curran, Theresa Feltwell, Anna Yakovleva, Charlotte J. Houldcroft, Aminu S. Jahun, Sarah L. Caddy, Ian Goodfellow
EPI_ISL_425332 hCoV-19/England/CAMB-7476C/2020 Europe / United Kingdom / England 2020-03-29
Department of Pathology, University of Cambridge COVID-19 Genomics UK (COG-UK) Consortium Luke W Meredith, M. Estee Torok , Myra Hosmillo, William L. Hamilton, Martin D. Curran, Theresa Feltwell, Anna Yakovleva, Charlotte J. Houldcroft, Aminu S. Jahun, Sarah L. Caddy, Ian Goodfellow
EPI_ISL_425333 hCoV-19/England/CAMB-7477B/2020 Europe / United Kingdom / England 2020-03-29
Department of Pathology, University of Cambridge COVID-19 Genomics UK (COG-UK) Consortium Luke W Meredith, M. Estee Torok , Myra Hosmillo, William L. Hamilton, Martin D. Curran, Theresa Feltwell, Anna Yakovleva, Charlotte J. Houldcroft, Aminu S. Jahun, Sarah L. Caddy, Ian Goodfellow
EPI_ISL_425335 hCoV-19/England/CAMB-748E1/2020 Europe / United Kingdom / England 2020-03-28
Department of Pathology, University of Cambridge COVID-19 Genomics UK (COG-UK) Consortium Luke W Meredith, M. Estee Torok , Myra Hosmillo, William L. Hamilton, Martin D. Curran, Theresa Feltwell, Anna Yakovleva, Charlotte J. Houldcroft, Aminu S. Jahun, Sarah L. Caddy, Ian Goodfellow
EPI_ISL_425338 hCoV-19/England/CAMB-7491B/2020 Europe / United Kingdom / England 2020-03-28
Department of Pathology, University of Cambridge COVID-19 Genomics UK (COG-UK) Consortium Luke W Meredith, M. Estee Torok , Myra Hosmillo, William L. Hamilton, Martin D. Curran, Theresa Feltwell, Anna Yakovleva, Charlotte J. Houldcroft, Aminu S. Jahun, Sarah L. Caddy, Ian Goodfellow
EPI_ISL_425348 hCoV-19/England/CAMB-74B42/2020 Europe / United Kingdom / England 2020-03-18
Department of Pathology, University of Cambridge COVID-19 Genomics UK (COG-UK) Consortium Luke W Meredith, M. Estee Torok , Myra Hosmillo, William L. Hamilton, Martin D. Curran, Theresa Feltwell, Anna Yakovleva, Charlotte J. Houldcroft, Aminu S. Jahun, Sarah L. Caddy, Ian Goodfellow

EPI_ISL_425359 hCoV-19/England/CAMB-74C30/2020 Europe / United Kingdom / England 2020-03-17 Department of Pathology, University of Cambridge COVID-19 Genomics UK (COG-UK) Consortium Luke W Meredith, M. Estee Torok , Myra Hosmillo, William L. Hamilton, Martin D. Curran, Theresa Feltwell, Anna Yakovleva, Charlotte J. Houldcroft, Aminu S. Jahun, Sarah L. Caddy, Ian Goodfellow
EPI_ISL_425361 hCoV-19/England/CAMB-74C5E/2020 Europe / United Kingdom / England 2020-03-19 Department of Pathology, University of Cambridge COVID-19 Genomics UK (COG-UK) Consortium Luke W Meredith, M. Estee Torok , Myra Hosmillo, William L. Hamilton, Martin D. Curran, Theresa Feltwell, Anna Yakovleva, Charlotte J. Houldcroft, Aminu S. Jahun, Sarah L. Caddy, Ian Goodfellow
EPI_ISL_425362 hCoV-19/England/CAMB-74C8B/2020 Europe / United Kingdom / England 2020-03-17 Department of Pathology, University of Cambridge COVID-19 Genomics UK (COG-UK) Consortium Luke W Meredith, M. Estee Torok , Myra Hosmillo, William L. Hamilton, Martin D. Curran, Theresa Feltwell, Anna Yakovleva, Charlotte J. Houldcroft, Aminu S. Jahun, Sarah L. Caddy, Ian Goodfellow
EPI_ISL_425371 hCoV-19/England/CAMB-74D4C/2020 Europe / United Kingdom / England 2020-03-19 Department of Pathology, University of Cambridge COVID-19 Genomics UK (COG-UK) Consortium Luke W Meredith, M. Estee Torok , Myra Hosmillo, William L. Hamilton, Martin D. Curran, Theresa Feltwell, Anna Yakovleva, Charlotte J. Houldcroft, Aminu S. Jahun, Sarah L. Caddy, Ian Goodfellow
EPI_ISL_425373 hCoV-19/England/CAMB-74D5B/2020 Europe / United Kingdom / England 2020-03-19 Department of Pathology, University of Cambridge COVID-19 Genomics UK (COG-UK) Consortium Luke W Meredith, M. Estee Torok , Myra Hosmillo, William L. Hamilton, Martin D. Curran, Theresa Feltwell, Anna Yakovleva, Charlotte J. Houldcroft, Aminu S. Jahun, Sarah L. Caddy, Ian Goodfellow
EPI_ISL_425374 hCoV-19/England/CAMB-74D6A/2020 Europe / United Kingdom / England 2020-03-19 Department of Pathology, University of Cambridge COVID-19 Genomics UK (COG-UK) Consortium Luke W Meredith, M. Estee Torok , Myra Hosmillo, William L. Hamilton, Martin D. Curran, Theresa Feltwell, Anna Yakovleva, Charlotte J. Houldcroft, Aminu S. Jahun, Sarah L. Caddy, Ian Goodfellow
EPI_ISL_425382 hCoV-19/England/CAMB-74E85/2020 Europe / United Kingdom / England 2020-03-18 Department of Pathology, University of Cambridge COVID-19 Genomics UK (COG-UK) Consortium Luke W Meredith, M. Estee Torok , Myra Hosmillo, William L. Hamilton, Martin D. Curran, Theresa Feltwell, Anna Yakovleva, Charlotte J. Houldcroft, Aminu S. Jahun, Sarah L. Caddy, Ian Goodfellow
EPI_ISL_425611 hCoV-19/England/NOTT-10E8FA/2020 Europe / United Kingdom / England 2020-03-29 Queens Medical Centre, Clinical Microbiology Department / DeepSeq Nottingham COVID-19 Genomics UK (COG-UK) Consortium Gemma Clark, Wendy Smith, Manjinder Khakh, Hannah Howson-Wells, Jonathan Ball, Patrick McClure, Joseph Chappell, Theocharis Tsoleridis, Nadine Holmes, Matthew Carlisle, Christopher Moore, Fei Sang, Johnny Debebe, Victoria Wright, Matthew Loose
EPI_ISL_425614 hCoV-19/England/NOTT-10E933/2020 Europe / United Kingdom / England 2020-03-29 Queens Medical Centre, Clinical Microbiology Department / DeepSeq Nottingham COVID-19 Genomics UK (COG-UK) Consortium Gemma Clark, Wendy Smith, Manjinder Khakh, Hannah Howson-Wells, Jonathan Ball, Patrick McClure, Joseph Chappell, Theocharis Tsoleridis, Nadine Holmes, Matthew Carlisle, Christopher Moore, Fei Sang, Johnny Debebe, Victoria Wright, Matthew Loose
EPI_ISL_425621 hCoV-19/England/NOTT-10E9BB/2020 Europe / United Kingdom / England 2020-03-29 Queens Medical Centre, Clinical Microbiology Department / DeepSeq Nottingham COVID-19 Genomics UK (COG-UK) Consortium Gemma Clark, Wendy Smith, Manjinder Khakh, Hannah Howson-Wells, Jonathan Ball, Patrick McClure, Joseph Chappell, Theocharis Tsoleridis, Nadine Holmes, Matthew Carlisle, Christopher Moore, Fei Sang, Johnny Debebe, Victoria Wright, Matthew Loose
EPI_ISL_425628 hCoV-19/England/NOTT-10EA30/2020 Europe / United Kingdom / England 2020-03-30 Queens Medical Centre, Clinical Microbiology Department / DeepSeq Nottingham COVID-19 Genomics UK (COG-UK) Consortium Gemma Clark, Wendy Smith, Manjinder Khakh, Hannah Howson-Wells, Jonathan Ball, Patrick McClure, Joseph Chappell, Theocharis Tsoleridis, Nadine Holmes, Matthew Carlisle, Christopher Moore, Fei Sang, Johnny Debebe, Victoria Wright, Matthew Loose
EPI_ISL_425630 hCoV-19/England/NOTT-10EA5E/2020 Europe / United Kingdom / England 2020-03-29 Queens Medical Centre, Clinical Microbiology Department / DeepSeq Nottingham COVID-19 Genomics UK (COG-UK) Consortium Gemma Clark, Wendy Smith, Manjinder Khakh, Hannah Howson-Wells, Jonathan Ball, Patrick McClure, Joseph Chappell, Theocharis Tsoleridis, Nadine Holmes, Matthew Carlisle, Christopher Moore, Fei Sang, Johnny Debebe, Victoria Wright, Matthew Loose
EPI_ISL_425631 hCoV-19/England/NOTT-10EA6D/2020 Europe / United Kingdom / England 2020-03-29 Queens Medical Centre, Clinical Microbiology Department / DeepSeq Nottingham COVID-19 Genomics UK (COG-UK) Consortium Gemma Clark, Wendy Smith, Manjinder Khakh, Hannah Howson-Wells, Jonathan Ball, Patrick McClure, Joseph Chappell, Theocharis Tsoleridis, Nadine Holmes, Matthew Carlisle, Christopher Moore, Fei Sang, Johnny Debebe, Victoria Wright, Matthew Loose
EPI_ISL_425632 hCoV-19/England/NOTT-10EA7C/2020 Europe / United Kingdom / England 2020-03-30 Queens Medical Centre, Clinical Microbiology Department / DeepSeq Nottingham COVID-19 Genomics UK (COG-UK) Consortium Gemma Clark, Wendy Smith, Manjinder Khakh, Hannah Howson-Wells, Jonathan Ball, Patrick McClure, Joseph Chappell, Theocharis Tsoleridis, Nadine Holmes, Matthew Carlisle, Christopher Moore, Fei Sang, Johnny Debebe, Victoria Wright, Matthew Loose
EPI_ISL_425633 hCoV-19/England/NOTT-10EA8B/2020 Europe / United Kingdom / England 2020-03-29 Queens Medical Centre, Clinical Microbiology Department / DeepSeq Nottingham COVID-19 Genomics UK (COG-UK) Consortium Gemma Clark, Wendy Smith, Manjinder Khakh, Hannah Howson-Wells, Jonathan Ball, Patrick McClure, Joseph Chappell, Theocharis Tsoleridis, Nadine Holmes, Matthew Carlisle, Christopher Moore, Fei Sang, Johnny Debebe, Victoria Wright, Matthew Loose
EPI_ISL_425635 hCoV-19/England/NOTT-10EAA9/2020 Europe / United Kingdom / England 2020-03-30 Queens Medical Centre, Clinical Microbiology Department / DeepSeq Nottingham COVID-19 Genomics UK (COG-UK)

Consortium	Gemma Clark, Wendy Smith, Manjinder Khakh, Hannah Howson-Wells, Jonathan Ball, Patrick McClure, Joseph Chappell, Theocharis Tsoleridis, Nadine Holmes, Matthew Carlisle, Christopher Moore, Fei Sang, Johnny Debebe, Victoria Wright, Matthew Loose
EPI_ISL_425638	hCoV-19/England/NOTT-10EAD6/2020	Europe / United Kingdom / England	2020-03-30	Queens Medical Centre, Clinical Microbiology Department / DeepSeq Nottingham	COVID-19 Genomics UK (COG-UK) Consortium	Gemma Clark, Wendy Smith, Manjinder Khakh, Hannah Howson-Wells, Jonathan Ball, Patrick McClure, Joseph Chappell, Theocharis Tsoleridis, Nadine Holmes, Matthew Carlisle, Christopher Moore, Fei Sang, Johnny Debebe, Victoria Wright, Matthew Loose
EPI_ISL_425639	hCoV-19/England/NOTT-10EAE5/2020	Europe / United Kingdom / England	2020-03-30	Queens Medical Centre, Clinical Microbiology Department / DeepSeq Nottingham	COVID-19 Genomics UK (COG-UK) Consortium	Gemma Clark, Wendy Smith, Manjinder Khakh, Hannah Howson-Wells, Jonathan Ball, Patrick McClure, Joseph Chappell, Theocharis Tsoleridis, Nadine Holmes, Matthew Carlisle, Christopher Moore, Fei Sang, Johnny Debebe, Victoria Wright, Matthew Loose
EPI_ISL_425640	hCoV-19/England/NOTT-10EAF4/2020	Europe / United Kingdom / England	2020-03-30	Queens Medical Centre, Clinical Microbiology Department / DeepSeq Nottingham	COVID-19 Genomics UK (COG-UK) Consortium	Gemma Clark, Wendy Smith, Manjinder Khakh, Hannah Howson-Wells, Jonathan Ball, Patrick McClure, Joseph Chappell, Theocharis Tsoleridis, Nadine Holmes, Matthew Carlisle, Christopher Moore, Fei Sang, Johnny Debebe, Victoria Wright, Matthew Loose
EPI_ISL_425642	hCoV-19/England/NOTT-10EB2E/2020	Europe / United Kingdom / England	2020-03-30	Queens Medical Centre, Clinical Microbiology Department / DeepSeq Nottingham	COVID-19 Genomics UK (COG-UK) Consortium	Gemma Clark, Wendy Smith, Manjinder Khakh, Hannah Howson-Wells, Jonathan Ball, Patrick McClure, Joseph Chappell, Theocharis Tsoleridis, Nadine Holmes, Matthew Carlisle, Christopher Moore, Fei Sang, Johnny Debebe, Victoria Wright, Matthew Loose
EPI_ISL_425644	hCoV-19/England/NOTT-10EB6A/2020	Europe / United Kingdom / England	2020-03-17	Queens Medical Centre, Clinical Microbiology Department / DeepSeq Nottingham	COVID-19 Genomics UK (COG-UK) Consortium	Gemma Clark, Wendy Smith, Manjinder Khakh, Hannah Howson-Wells, Jonathan Ball, Patrick McClure, Joseph Chappell, Theocharis Tsoleridis, Nadine Holmes, Matthew Carlisle, Christopher Moore, Fei Sang, Johnny Debebe, Victoria Wright, Matthew Loose
EPI_ISL_425645	hCoV-19/England/NOTT-10EB79/2020	Europe / United Kingdom / England	2020-03-18	Queens Medical Centre, Clinical Microbiology Department / DeepSeq Nottingham	COVID-19 Genomics UK (COG-UK) Consortium	Gemma Clark, Wendy Smith, Manjinder Khakh, Hannah Howson-Wells, Jonathan Ball, Patrick McClure, Joseph Chappell, Theocharis Tsoleridis, Nadine Holmes, Matthew Carlisle, Christopher Moore, Fei Sang, Johnny Debebe, Victoria Wright, Matthew Loose
EPI_ISL_425647	hCoV-19/Scotland/CVR100/2020	Europe / United Kingdom / Scotland	2020-03-14	West of Scotland Specialist Virology Centre, NHSGGC / MRC-University of Glasgow Centre for Virus Research	COVID-19 Genomics UK (COG-UK) Consortium	Ana da Silva Filipe, Kathy Smollett, Stephen Carmichael, Natasha Johnson, Daniel Mair, Lily Tong, Jenna Nichols; Sarah McDonald; Richard Orton, Joseph Hughes, Sreenu Vattipally, David L Robertson; Kathy Li, Natasha Jesudason, Rajiv Shah, James Shepherd, Antonia Ho, Emma Thomson; Alasdair MacLean, Rory Gunson.
EPI_ISL_425648	hCoV-19/Scotland/CVR101/2020	Europe / United Kingdom / Scotland	2020-03-14	West of Scotland Specialist Virology Centre, NHSGGC / MRC-University of Glasgow Centre for Virus Research	COVID-19 Genomics UK (COG-UK) Consortium	Ana da Silva Filipe, Kathy Smollett, Stephen Carmichael, Natasha Johnson, Daniel Mair, Lily Tong, Jenna Nichols; Sarah McDonald; Richard Orton, Joseph Hughes, Sreenu Vattipally, David L Robertson; Kathy Li, Natasha Jesudason, Rajiv Shah, James Shepherd, Antonia Ho, Emma Thomson; Alasdair MacLean, Rory Gunson.
EPI_ISL_425650	hCoV-19/Scotland/CVR103/2020	Europe / United Kingdom / Scotland	2020-03-13	West of Scotland Specialist Virology Centre, NHSGGC / MRC-University of Glasgow Centre for Virus Research	COVID-19 Genomics UK (COG-UK) Consortium	Ana da Silva Filipe, Kathy Smollett, Stephen Carmichael, Natasha Johnson, Daniel Mair, Lily Tong, Jenna Nichols; Sarah McDonald; Richard Orton, Joseph Hughes, Sreenu Vattipally, David L Robertson; Kathy Li, Natasha Jesudason, Rajiv Shah, James Shepherd, Antonia Ho, Emma Thomson; Alasdair MacLean, Rory Gunson.
EPI_ISL_425651	hCoV-19/Scotland/CVR104/2020	Europe / United Kingdom / Scotland	2020-03-13	West of Scotland Specialist Virology Centre, NHSGGC / MRC-University of Glasgow Centre for Virus Research	COVID-19 Genomics UK (COG-UK) Consortium	Ana da Silva Filipe, Kathy Smollett, Stephen Carmichael, Natasha Johnson, Daniel Mair, Lily Tong, Jenna Nichols; Sarah McDonald; Richard Orton, Joseph Hughes, Sreenu Vattipally, David L Robertson; Kathy Li, Natasha Jesudason, Rajiv Shah, James Shepherd, Antonia Ho, Emma Thomson; Alasdair MacLean, Rory Gunson.
EPI_ISL_425652	hCoV-19/Scotland/CVR106/2020	Europe / United Kingdom / Scotland	2020-03-13	West of Scotland Specialist Virology Centre, NHSGGC / MRC-University of Glasgow Centre for Virus Research	COVID-19 Genomics UK (COG-UK) Consortium	Ana da Silva Filipe, Kathy Smollett, Stephen Carmichael, Natasha Johnson, Daniel Mair, Lily Tong, Jenna Nichols; Sarah McDonald; Richard Orton, Joseph Hughes, Sreenu Vattipally, David L Robertson; Kathy Li, Natasha Jesudason, Rajiv Shah, James Shepherd, Antonia Ho, Emma Thomson; Alasdair MacLean, Rory Gunson.
EPI_ISL_425653	hCoV-19/Scotland/CVR107/2020	Europe / United Kingdom / Scotland	2020-03-13	West of Scotland Specialist Virology Centre, NHSGGC / MRC-University of Glasgow Centre for Virus Research	COVID-19 Genomics UK (COG-UK) Consortium	Ana da Silva Filipe, Kathy Smollett, Stephen Carmichael, Natasha Johnson, Daniel Mair, Lily Tong, Jenna Nichols; Sarah McDonald; Richard Orton, Joseph Hughes, Sreenu Vattipally, David L Robertson; Kathy Li, Natasha Jesudason, Rajiv Shah, James Shepherd, Antonia Ho, Emma Thomson; Alasdair MacLean, Rory Gunson.
EPI_ISL_425654	hCoV-19/Scotland/CVR108/2020	Europe / United Kingdom / Scotland	2020-03-13	West of

Scotland Specialist Virology Centre, NHSGGC / MRC-University of Glasgow Centre for Virus Research COVID-19 Genomics UK (COG-UK) Consortium Ana da Silva Filipe, Kathy Smollett, Stephen Carmichael, Natasha Johnson, Daniel Mair, Lily Tong, Jenna Nichols; Sarah McDonald; Richard Orton, Joseph Hughes, Sreenu Vattipally, David L Robertson; Kathy Li, Natasha Jesudason, Rajiv Shah, James Shepherd, Antonia Ho, Emma Thomson; Alasdair MacLean, Rory Gunson.
EPI_ISL_425656 hCoV-19/Scotland/CVR110/2020 Europe / United Kingdom / Scotland 2020-03-14 West of Scotland Specialist Virology Centre, NHSGGC / MRC-University of Glasgow Centre for Virus Research COVID-19 Genomics UK (COG-UK) Consortium Ana da Silva Filipe, Kathy Smollett, Stephen Carmichael, Natasha Johnson, Daniel Mair, Lily Tong, Jenna Nichols; Sarah McDonald; Richard Orton, Joseph Hughes, Sreenu Vattipally, David L Robertson; Kathy Li, Natasha Jesudason, Rajiv Shah, James Shepherd, Antonia Ho, Emma Thomson; Alasdair MacLean, Rory Gunson.
EPI_ISL_425657 hCoV-19/Scotland/CVR112/2020 Europe / United Kingdom / Scotland 2020-03-15 West of Scotland Specialist Virology Centre, NHSGGC / MRC-University of Glasgow Centre for Virus Research COVID-19 Genomics UK (COG-UK) Consortium Ana da Silva Filipe, Kathy Smollett, Stephen Carmichael, Natasha Johnson, Daniel Mair, Lily Tong, Jenna Nichols; Sarah McDonald; Richard Orton, Joseph Hughes, Sreenu Vattipally, David L Robertson; Kathy Li, Natasha Jesudason, Rajiv Shah, James Shepherd, Antonia Ho, Emma Thomson; Alasdair MacLean, Rory Gunson.
EPI_ISL_425658 hCoV-19/Scotland/CVR113/2020 Europe / United Kingdom / Scotland 2020-03-15 West of Scotland Specialist Virology Centre, NHSGGC / MRC-University of Glasgow Centre for Virus Research COVID-19 Genomics UK (COG-UK) Consortium Ana da Silva Filipe, Kathy Smollett, Stephen Carmichael, Natasha Johnson, Daniel Mair, Lily Tong, Jenna Nichols; Sarah McDonald; Richard Orton, Joseph Hughes, Sreenu Vattipally, David L Robertson; Kathy Li, Natasha Jesudason, Rajiv Shah, James Shepherd, Antonia Ho, Emma Thomson; Alasdair MacLean, Rory Gunson.
EPI_ISL_425659 hCoV-19/Scotland/CVR114/2020 Europe / United Kingdom / Scotland 2020-03-13 West of Scotland Specialist Virology Centre, NHSGGC / MRC-University of Glasgow Centre for Virus Research COVID-19 Genomics UK (COG-UK) Consortium Ana da Silva Filipe, Kathy Smollett, Stephen Carmichael, Natasha Johnson, Daniel Mair, Lily Tong, Jenna Nichols; Sarah McDonald; Richard Orton, Joseph Hughes, Sreenu Vattipally, David L Robertson; Kathy Li, Natasha Jesudason, Rajiv Shah, James Shepherd, Antonia Ho, Emma Thomson; Alasdair MacLean, Rory Gunson.
EPI_ISL_425660 hCoV-19/Scotland/CVR115/2020 Europe / United Kingdom / Scotland 2020-03-13 West of Scotland Specialist Virology Centre, NHSGGC / MRC-University of Glasgow Centre for Virus Research COVID-19 Genomics UK (COG-UK) Consortium Ana da Silva Filipe, Kathy Smollett, Stephen Carmichael, Natasha Johnson, Daniel Mair, Lily Tong, Jenna Nichols; Sarah McDonald; Richard Orton, Joseph Hughes, Sreenu Vattipally, David L Robertson; Kathy Li, Natasha Jesudason, Rajiv Shah, James Shepherd, Antonia Ho, Emma Thomson; Alasdair MacLean, Rory Gunson.
EPI_ISL_425662 hCoV-19/Scotland/CVR118/2020 Europe / United Kingdom / Scotland 2020-03-12 West of Scotland Specialist Virology Centre, NHSGGC / MRC-University of Glasgow Centre for Virus Research COVID-19 Genomics UK (COG-UK) Consortium Ana da Silva Filipe, Kathy Smollett, Stephen Carmichael, Natasha Johnson, Daniel Mair, Lily Tong, Jenna Nichols; Sarah McDonald; Richard Orton, Joseph Hughes, Sreenu Vattipally, David L Robertson; Kathy Li, Natasha Jesudason, Rajiv Shah, James Shepherd, Antonia Ho, Emma Thomson; Alasdair MacLean, Rory Gunson.
EPI_ISL_425663 hCoV-19/Scotland/CVR12/2020 Europe / United Kingdom / Scotland 2020-03-10 West of Scotland Specialist Virology Centre, NHSGGC / MRC-University of Glasgow Centre for Virus Research COVID-19 Genomics UK (COG-UK) Consortium Ana da Silva Filipe, Kathy Smollett, Stephen Carmichael, Natasha Johnson, Daniel Mair, Lily Tong, Jenna Nichols; Sarah McDonald; Richard Orton, Joseph Hughes, Sreenu Vattipally, David L Robertson; Kathy Li, Natasha Jesudason, Rajiv Shah, James Shepherd, Antonia Ho, Emma Thomson; Alasdair MacLean, Rory Gunson.
EPI_ISL_425668 hCoV-19/Scotland/CVR125/2020 Europe / United Kingdom / Scotland 2020-03-14 West of Scotland Specialist Virology Centre, NHSGGC / MRC-University of Glasgow Centre for Virus Research COVID-19 Genomics UK (COG-UK) Consortium Ana da Silva Filipe, Kathy Smollett, Stephen Carmichael, Natasha Johnson, Daniel Mair, Lily Tong, Jenna Nichols; Sarah McDonald; Richard Orton, Joseph Hughes, Sreenu Vattipally, David L Robertson; Kathy Li, Natasha Jesudason, Rajiv Shah, James Shepherd, Antonia Ho, Emma Thomson; Alasdair MacLean, Rory Gunson.
EPI_ISL_425669 hCoV-19/Scotland/CVR126/2020 Europe / United Kingdom / Scotland 2020-03-15 West of Scotland Specialist Virology Centre, NHSGGC / MRC-University of Glasgow Centre for Virus Research COVID-19 Genomics UK (COG-UK) Consortium Ana da Silva Filipe, Kathy Smollett, Stephen Carmichael, Natasha Johnson, Daniel Mair, Lily Tong, Jenna Nichols; Sarah McDonald; Richard Orton, Joseph Hughes, Sreenu Vattipally, David L Robertson; Kathy Li, Natasha Jesudason, Rajiv Shah, James Shepherd, Antonia Ho, Emma Thomson; Alasdair MacLean, Rory Gunson.
EPI_ISL_425670 hCoV-19/Scotland/CVR128/2020 Europe / United Kingdom / Scotland 2020-03-13 West of Scotland Specialist Virology Centre, NHSGGC / MRC-University of Glasgow Centre for Virus Research COVID-19 Genomics UK (COG-UK) Consortium Ana da Silva Filipe, Kathy Smollett, Stephen Carmichael, Natasha Johnson, Daniel Mair, Lily Tong, Jenna Nichols; Sarah McDonald; Richard Orton, Joseph Hughes, Sreenu Vattipally, David L Robertson; Kathy Li, Natasha Jesudason, Rajiv Shah, James Shepherd, Antonia Ho, Emma Thomson; Alasdair MacLean, Rory Gunson.
EPI_ISL_425671 hCoV-19/Scotland/CVR129/2020 Europe / United Kingdom / Scotland 2020-03-13 West of Scotland Specialist Virology Centre, NHSGGC / MRC-University of Glasgow Centre for Virus Research COVID-19 Genomics UK (COG-UK) Consortium Ana da Silva Filipe, Kathy Smollett, Stephen Carmichael, Natasha Johnson, Daniel Mair, Lily Tong, Jenna Nichols; Sarah McDonald; Richard Orton, Joseph Hughes, Sreenu Vattipally, David L Robertson; Kathy Li, Natasha Jesudason, Rajiv Shah, James Shepherd, Antonia Ho, Emma

Thomson; Alasdair MacLean, Rory Gunson.
EPI_ISL_425672 hCoV-19/Scotland/CVR13/2020 Europe / United Kingdom / Scotland 2020-03-11 West of Scotland Specialist Virology Centre, NHSGGC / MRC-University of Glasgow Centre for Virus Research COVID-19 Genomics UK (COG-UK) Consortium Ana da Silva Filipe, Kathy Smollett, Stephen Carmichael, Natasha Johnson, Daniel Mair, Lily Tong, Jenna Nichols; Sarah McDonald; Richard Orton, Joseph Hughes, Sreenu Vattipally, David L Robertson; Kathy Li, Natasha Jesudason, Rajiv Shah, James Shepherd, Antonia Ho, Emma Thomson; Alasdair MacLean, Rory Gunson.
EPI_ISL_425674 hCoV-19/Scotland/CVR131/2020 Europe / United Kingdom / Scotland 2020-03-12 West of Scotland Specialist Virology Centre, NHSGGC / MRC-University of Glasgow Centre for Virus Research COVID-19 Genomics UK (COG-UK) Consortium Ana da Silva Filipe, Kathy Smollett, Stephen Carmichael, Natasha Johnson, Daniel Mair, Lily Tong, Jenna Nichols; Sarah McDonald; Richard Orton, Joseph Hughes, Sreenu Vattipally, David L Robertson; Kathy Li, Natasha Jesudason, Rajiv Shah, James Shepherd, Antonia Ho, Emma Thomson; Alasdair MacLean, Rory Gunson.
EPI_ISL_425675 hCoV-19/Scotland/CVR132/2020 Europe / United Kingdom / Scotland 2020-03-16 West of Scotland Specialist Virology Centre, NHSGGC / MRC-University of Glasgow Centre for Virus Research COVID-19 Genomics UK (COG-UK) Consortium Ana da Silva Filipe, Kathy Smollett, Stephen Carmichael, Natasha Johnson, Daniel Mair, Lily Tong, Jenna Nichols; Sarah McDonald; Richard Orton, Joseph Hughes, Sreenu Vattipally, David L Robertson; Kathy Li, Natasha Jesudason, Rajiv Shah, James Shepherd, Antonia Ho, Emma Thomson; Alasdair MacLean, Rory Gunson.
EPI_ISL_425676 hCoV-19/Scotland/CVR133/2020 Europe / United Kingdom / Scotland 2020-03-16 West of Scotland Specialist Virology Centre, NHSGGC / MRC-University of Glasgow Centre for Virus Research COVID-19 Genomics UK (COG-UK) Consortium Ana da Silva Filipe, Kathy Smollett, Stephen Carmichael, Natasha Johnson, Daniel Mair, Lily Tong, Jenna Nichols; Sarah McDonald; Richard Orton, Joseph Hughes, Sreenu Vattipally, David L Robertson; Kathy Li, Natasha Jesudason, Rajiv Shah, James Shepherd, Antonia Ho, Emma Thomson; Alasdair MacLean, Rory Gunson.
EPI_ISL_425677 hCoV-19/Scotland/CVR134/2020 Europe / United Kingdom / Scotland 2020-03-16 West of Scotland Specialist Virology Centre, NHSGGC / MRC-University of Glasgow Centre for Virus Research COVID-19 Genomics UK (COG-UK) Consortium Ana da Silva Filipe, Kathy Smollett, Stephen Carmichael, Natasha Johnson, Daniel Mair, Lily Tong, Jenna Nichols; Sarah McDonald; Richard Orton, Joseph Hughes, Sreenu Vattipally, David L Robertson; Kathy Li, Natasha Jesudason, Rajiv Shah, James Shepherd, Antonia Ho, Emma Thomson; Alasdair MacLean, Rory Gunson.
EPI_ISL_425678 hCoV-19/Scotland/CVR135/2020 Europe / United Kingdom / Scotland 2020-03-16 West of Scotland Specialist Virology Centre, NHSGGC / MRC-University of Glasgow Centre for Virus Research COVID-19 Genomics UK (COG-UK) Consortium Ana da Silva Filipe, Kathy Smollett, Stephen Carmichael, Natasha Johnson, Daniel Mair, Lily Tong, Jenna Nichols; Sarah McDonald; Richard Orton, Joseph Hughes, Sreenu Vattipally, David L Robertson; Kathy Li, Natasha Jesudason, Rajiv Shah, James Shepherd, Antonia Ho, Emma Thomson; Alasdair MacLean, Rory Gunson.
EPI_ISL_425679 hCoV-19/Scotland/CVR136/2020 Europe / United Kingdom / Scotland 2020-03-16 West of Scotland Specialist Virology Centre, NHSGGC / MRC-University of Glasgow Centre for Virus Research COVID-19 Genomics UK (COG-UK) Consortium Ana da Silva Filipe, Kathy Smollett, Stephen Carmichael, Natasha Johnson, Daniel Mair, Lily Tong, Jenna Nichols; Sarah McDonald; Richard Orton, Joseph Hughes, Sreenu Vattipally, David L Robertson; Kathy Li, Natasha Jesudason, Rajiv Shah, James Shepherd, Antonia Ho, Emma Thomson; Alasdair MacLean, Rory Gunson.
EPI_ISL_425682 hCoV-19/Scotland/CVR139/2020 Europe / United Kingdom / Scotland 2020-03-16 West of Scotland Specialist Virology Centre, NHSGGC / MRC-University of Glasgow Centre for Virus Research COVID-19 Genomics UK (COG-UK) Consortium Ana da Silva Filipe, Kathy Smollett, Stephen Carmichael, Natasha Johnson, Daniel Mair, Lily Tong, Jenna Nichols; Sarah McDonald; Richard Orton, Joseph Hughes, Sreenu Vattipally, David L Robertson; Kathy Li, Natasha Jesudason, Rajiv Shah, James Shepherd, Antonia Ho, Emma Thomson; Alasdair MacLean, Rory Gunson.
EPI_ISL_425684 hCoV-19/Scotland/CVR140/2020 Europe / United Kingdom / Scotland 2020-03-16 West of Scotland Specialist Virology Centre, NHSGGC / MRC-University of Glasgow Centre for Virus Research COVID-19 Genomics UK (COG-UK) Consortium Ana da Silva Filipe, Kathy Smollett, Stephen Carmichael, Natasha Johnson, Daniel Mair, Lily Tong, Jenna Nichols; Sarah McDonald; Richard Orton, Joseph Hughes, Sreenu Vattipally, David L Robertson; Kathy Li, Natasha Jesudason, Rajiv Shah, James Shepherd, Antonia Ho, Emma Thomson; Alasdair MacLean, Rory Gunson.
EPI_ISL_425685 hCoV-19/Scotland/CVR141/2020 Europe / United Kingdom / Scotland 2020-03-16 West of Scotland Specialist Virology Centre, NHSGGC / MRC-University of Glasgow Centre for Virus Research COVID-19 Genomics UK (COG-UK) Consortium Ana da Silva Filipe, Kathy Smollett, Stephen Carmichael, Natasha Johnson, Daniel Mair, Lily Tong, Jenna Nichols; Sarah McDonald; Richard Orton, Joseph Hughes, Sreenu Vattipally, David L Robertson; Kathy Li, Natasha Jesudason, Rajiv Shah, James Shepherd, Antonia Ho, Emma Thomson; Alasdair MacLean, Rory Gunson.
EPI_ISL_425687 hCoV-19/Scotland/CVR143/2020 Europe / United Kingdom / Scotland 2020-03-17 West of Scotland Specialist Virology Centre, NHSGGC / MRC-University of Glasgow Centre for Virus Research COVID-19 Genomics UK (COG-UK) Consortium Ana da Silva Filipe, Kathy Smollett, Stephen Carmichael, Natasha Johnson, Daniel Mair, Lily Tong, Jenna Nichols; Sarah McDonald; Richard Orton, Joseph Hughes, Sreenu Vattipally, David L Robertson; Kathy Li, Natasha Jesudason, Rajiv Shah, James Shepherd, Antonia Ho, Emma Thomson; Alasdair MacLean, Rory Gunson.
EPI_ISL_425688 hCoV-19/Scotland/CVR144/2020 Europe / United Kingdom / Scotland 2020-03-17 West of Scotland Specialist Virology Centre, NHSGGC / MRC-University of Glasgow Centre for Virus Research COVID-19 Genomics UK (COG-UK) Consortium Ana da Silva Filipe, Kathy Smollett, Stephen Carmichael, Natasha

Johnson, Daniel Mair, Lily Tong, Jenna Nichols; Sarah McDonald; Richard Orton, Joseph Hughes, Sreenu Vattipally, David L Robertson; Kathy Li, Natasha Jesudason, Rajiv Shah, James Shepherd, Antonia Ho, Emma Thomson; Alasdair MacLean, Rory Gunson.
EPI_ISL_425692 hCoV-19/Scotland/CVR15/2020 Europe / United Kingdom / Scotland 2020-03-12 West of Scotland Specialist Virology Centre, NHSGGC / MRC-University of Glasgow Centre for Virus Research COVID-19 Genomics UK (COG-UK) Consortium Ana da Silva Filipe, Kathy Smollett, Stephen Carmichael, Natasha Johnson, Daniel Mair, Lily Tong, Jenna Nichols; Sarah McDonald; Richard Orton, Joseph Hughes, Sreenu Vattipally, David L Robertson; Kathy Li, Natasha Jesudason, Rajiv Shah, James Shepherd, Antonia Ho, Emma Thomson; Alasdair MacLean, Rory Gunson.
EPI_ISL_425693 hCoV-19/Scotland/CVR150/2020 Europe / United Kingdom / Scotland 2020-03-16 West of Scotland Specialist Virology Centre, NHSGGC / MRC-University of Glasgow Centre for Virus Research COVID-19 Genomics UK (COG-UK) Consortium Ana da Silva Filipe, Kathy Smollett, Stephen Carmichael, Natasha Johnson, Daniel Mair, Lily Tong, Jenna Nichols; Sarah McDonald; Richard Orton, Joseph Hughes, Sreenu Vattipally, David L Robertson; Kathy Li, Natasha Jesudason, Rajiv Shah, James Shepherd, Antonia Ho, Emma Thomson; Alasdair MacLean, Rory Gunson.
EPI_ISL_425695 hCoV-19/Scotland/CVR152/2020 Europe / United Kingdom / Scotland 2020-03-16 West of Scotland Specialist Virology Centre, NHSGGC / MRC-University of Glasgow Centre for Virus Research COVID-19 Genomics UK (COG-UK) Consortium Ana da Silva Filipe, Kathy Smollett, Stephen Carmichael, Natasha Johnson, Daniel Mair, Lily Tong, Jenna Nichols; Sarah McDonald; Richard Orton, Joseph Hughes, Sreenu Vattipally, David L Robertson; Kathy Li, Natasha Jesudason, Rajiv Shah, James Shepherd, Antonia Ho, Emma Thomson; Alasdair MacLean, Rory Gunson.
EPI_ISL_425696 hCoV-19/Scotland/CVR153/2020 Europe / United Kingdom / Scotland 2020-03-17 West of Scotland Specialist Virology Centre, NHSGGC / MRC-University of Glasgow Centre for Virus Research COVID-19 Genomics UK (COG-UK) Consortium Ana da Silva Filipe, Kathy Smollett, Stephen Carmichael, Natasha Johnson, Daniel Mair, Lily Tong, Jenna Nichols; Sarah McDonald; Richard Orton, Joseph Hughes, Sreenu Vattipally, David L Robertson; Kathy Li, Natasha Jesudason, Rajiv Shah, James Shepherd, Antonia Ho, Emma Thomson; Alasdair MacLean, Rory Gunson.
EPI_ISL_425697 hCoV-19/Scotland/CVR155/2020 Europe / United Kingdom / Scotland 2020-03-17 West of Scotland Specialist Virology Centre, NHSGGC / MRC-University of Glasgow Centre for Virus Research COVID-19 Genomics UK (COG-UK) Consortium Ana da Silva Filipe, Kathy Smollett, Stephen Carmichael, Natasha Johnson, Daniel Mair, Lily Tong, Jenna Nichols; Sarah McDonald; Richard Orton, Joseph Hughes, Sreenu Vattipally, David L Robertson; Kathy Li, Natasha Jesudason, Rajiv Shah, James Shepherd, Antonia Ho, Emma Thomson; Alasdair MacLean, Rory Gunson.
EPI_ISL_425698 hCoV-19/Scotland/CVR159/2020 Europe / United Kingdom / Scotland 2020-03-17 West of Scotland Specialist Virology Centre, NHSGGC / MRC-University of Glasgow Centre for Virus Research COVID-19 Genomics UK (COG-UK) Consortium Ana da Silva Filipe, Kathy Smollett, Stephen Carmichael, Natasha Johnson, Daniel Mair, Lily Tong, Jenna Nichols; Sarah McDonald; Richard Orton, Joseph Hughes, Sreenu Vattipally, David L Robertson; Kathy Li, Natasha Jesudason, Rajiv Shah, James Shepherd, Antonia Ho, Emma Thomson; Alasdair MacLean, Rory Gunson.
EPI_ISL_425699 hCoV-19/Scotland/CVR16/2020 Europe / United Kingdom / Scotland 2020-03-12 West of Scotland Specialist Virology Centre, NHSGGC / MRC-University of Glasgow Centre for Virus Research COVID-19 Genomics UK (COG-UK) Consortium Ana da Silva Filipe, Kathy Smollett, Stephen Carmichael, Natasha Johnson, Daniel Mair, Lily Tong, Jenna Nichols; Sarah McDonald; Richard Orton, Joseph Hughes, Sreenu Vattipally, David L Robertson; Kathy Li, Natasha Jesudason, Rajiv Shah, James Shepherd, Antonia Ho, Emma Thomson; Alasdair MacLean, Rory Gunson.
EPI_ISL_426617 hCoV-19/USA/NY-NYUMC162/2020 North America / USA / New York / Suffolk county 2020-04-01 NYU Langone Health Departments of Pathology and Medicine, New York University School of Medicine Maria Aguero-Rosenfeld, Brendan Belovarac, Margaret Black, Ludovic Boytard, John Cadley, Paolo Cotzia, John Chen, Dacia Dimartino, Xiaojun Feng, Tatyana Gindin, Emily Guzman, Adriana Heguy, Megan Hogan, Emily Huang, George Jour, Andrew Lytle, Christian Marier, Matthew T. Maurano, Mark J. Mulligan, Peter Meyn, Iman Osman, Jared Pinnell, Vanessa Raabe, Sitharam Ramaswami, Amy Rapkiewicz, Marie Samanovic-Golden, Antonio Serrano, Guomiao Shen, Matija Snuderl, Theodore Vougiouklakis, Nick Vulpescu, Gael Westby, Paul Zappile, Yutong Zhang
EPI_ISL_426618 hCoV-19/USA/NY-NYUMC163/2020 North America / USA / New York / Nassau county 2020-04-01 NYU Langone Health Departments of Pathology and Medicine, New York University School of Medicine Maria Aguero-Rosenfeld, Brendan Belovarac, Margaret Black, Ludovic Boytard, John Cadley, Paolo Cotzia, John Chen, Dacia Dimartino, Xiaojun Feng, Tatyana Gindin, Emily Guzman, Adriana Heguy, Megan Hogan, Emily Huang, George Jour, Andrew Lytle, Christian Marier, Matthew T. Maurano, Mark J. Mulligan, Peter Meyn, Iman Osman, Jared Pinnell, Vanessa Raabe, Sitharam Ramaswami, Amy Rapkiewicz, Marie Samanovic-Golden, Antonio Serrano, Guomiao Shen, Matija Snuderl, Theodore Vougiouklakis, Nick Vulpescu, Gael Westby, Paul Zappile, Yutong Zhang
EPI_ISL_426619 hCoV-19/USA/NY-NYUMC164/2020 North America / USA / New York / Nassau county 2020-04-01 NYU Langone Health Departments of Pathology and Medicine, New York University School of Medicine Maria Aguero-Rosenfeld, Brendan Belovarac, Margaret Black, Ludovic Boytard, John Cadley, Paolo Cotzia, John Chen, Dacia Dimartino, Xiaojun Feng, Tatyana Gindin, Emily Guzman, Adriana Heguy, Megan Hogan, Emily Huang, George Jour, Andrew Lytle, Christian Marier, Matthew T. Maurano, Mark J. Mulligan, Peter Meyn, Iman Osman, Jared Pinnell, Vanessa Raabe, Sitharam Ramaswami, Amy Rapkiewicz, Marie Samanovic-Golden, Antonio Serrano, Guomiao Shen, Matija Snuderl, Theodore Vougiouklakis, Nick Vulpescu, Gael Westby, Paul Zappile, Yutong Zhang
EPI_ISL_426620 hCoV-19/USA/NY-NYUMC165/2020 North America / USA / New York / Brooklyn 2020-04-01 NYU Langone Health Departments of Pathology and Medicine, New York University School of Medicine Maria Aguero-Rosenfeld, Brendan Belovarac, Margaret Black, Ludovic Boytard, John Cadley, Paolo Cotzia, John Chen, Dacia Dimartino, Xiaojun Feng, Tatyana Gindin, Emily Guzman, Adriana Heguy, Megan Hogan, Emily Huang, George

Jour, Andrew Lytle, Christian Marier, Matthew T. Maurano, Mark J. Mulligan, Peter Meyn, Iman Osman, Jared Pinnell, Vanessa Raabe, Sitharam Ramaswami, Amy Rapkiewicz, Marie Samanovic-Golden, Antonio Serrano, Guomiao Shen, Matija Snuderl, Theodore Vougiouklakis, Nick Vulpescu, Gael Westby, Paul Zappile, Yutong Zhang
EPI_ISL_426621 hCoV-19/USA/NY-NYUMC166/2020 North America / USA / New York / Brooklyn 2020-04-01 NYU Langone Health Departments of Pathology and Medicine, New York University School of Medicine Maria Aguero-Rosenfeld, Brendan Belovarac, Margaret Black, Ludovic Boytard, John Cadley, Paolo Cotzia, John Chen, Dacia Dimartino, Xiaojun Feng, Tatyana Gindin, Emily Guzman, Adriana Heguy, Megan Hogan, Emily Huang, George Jour, Andrew Lytle, Christian Marier, Matthew T. Maurano, Mark J. Mulligan, Peter Meyn, Iman Osman, Jared Pinnell, Vanessa Raabe, Sitharam Ramaswami, Amy Rapkiewicz, Marie Samanovic-Golden, Antonio Serrano, Guomiao Shen, Matija Snuderl, Theodore Vougiouklakis, Nick Vulpescu, Gael Westby, Paul Zappile, Yutong Zhang
EPI_ISL_426622 hCoV-19/USA/NY-NYUMC167/2020 North America / USA / New York / Manhattan 2020-04-01 NYU Langone Health Departments of Pathology and Medicine, New York University School of Medicine Maria Aguero-Rosenfeld, Brendan Belovarac, Margaret Black, Ludovic Boytard, John Cadley, Paolo Cotzia, John Chen, Dacia Dimartino, Xiaojun Feng, Tatyana Gindin, Emily Guzman, Adriana Heguy, Megan Hogan, Emily Huang, George Jour, Andrew Lytle, Christian Marier, Matthew T. Maurano, Mark J. Mulligan, Peter Meyn, Iman Osman, Jared Pinnell, Vanessa Raabe, Sitharam Ramaswami, Amy Rapkiewicz, Marie Samanovic-Golden, Antonio Serrano, Guomiao Shen, Matija Snuderl, Theodore Vougiouklakis, Nick Vulpescu, Gael Westby, Paul Zappile, Yutong Zhang
EPI_ISL_426623 hCoV-19/USA/NY-NYUMC168/2020 North America / USA / New York / Manhattan 2020-04-01 NYU Langone Health Departments of Pathology and Medicine, New York University School of Medicine Maria Aguero-Rosenfeld, Brendan Belovarac, Margaret Black, Ludovic Boytard, John Cadley, Paolo Cotzia, John Chen, Dacia Dimartino, Xiaojun Feng, Tatyana Gindin, Emily Guzman, Adriana Heguy, Megan Hogan, Emily Huang, George Jour, Andrew Lytle, Christian Marier, Matthew T. Maurano, Mark J. Mulligan, Peter Meyn, Iman Osman, Jared Pinnell, Vanessa Raabe, Sitharam Ramaswami, Amy Rapkiewicz, Marie Samanovic-Golden, Antonio Serrano, Guomiao Shen, Matija Snuderl, Theodore Vougiouklakis, Nick Vulpescu, Gael Westby, Paul Zappile, Yutong Zhang
EPI_ISL_426624 hCoV-19/USA/NY-NYUMC169/2020 North America / USA / New York / Brooklyn 2020-04-01 NYU Langone Health Departments of Pathology and Medicine, New York University School of Medicine Maria Aguero-Rosenfeld, Brendan Belovarac, Margaret Black, Ludovic Boytard, John Cadley, Paolo Cotzia, John Chen, Dacia Dimartino, Xiaojun Feng, Tatyana Gindin, Emily Guzman, Adriana Heguy, Megan Hogan, Emily Huang, George Jour, Andrew Lytle, Christian Marier, Matthew T. Maurano, Mark J. Mulligan, Peter Meyn, Iman Osman, Jared Pinnell, Vanessa Raabe, Sitharam Ramaswami, Amy Rapkiewicz, Marie Samanovic-Golden, Antonio Serrano, Guomiao Shen, Matija Snuderl, Theodore Vougiouklakis, Nick Vulpescu, Gael Westby, Paul Zappile, Yutong Zhang
EPI_ISL_426625 hCoV-19/USA/NY-NYUMC170/2020 North America / USA / New York / Brooklyn 2020-04-01 NYU Langone Health Departments of Pathology and Medicine, New York University School of Medicine Maria Aguero-Rosenfeld, Brendan Belovarac, Margaret Black, Ludovic Boytard, John Cadley, Paolo Cotzia, John Chen, Dacia Dimartino, Xiaojun Feng, Tatyana Gindin, Emily Guzman, Adriana Heguy, Megan Hogan, Emily Huang, George Jour, Andrew Lytle, Christian Marier, Matthew T. Maurano, Mark J. Mulligan, Peter Meyn, Iman Osman, Jared Pinnell, Vanessa Raabe, Sitharam Ramaswami, Amy Rapkiewicz, Marie Samanovic-Golden, Antonio Serrano, Guomiao Shen, Matija Snuderl, Theodore Vougiouklakis, Nick Vulpescu, Gael Westby, Paul Zappile, Yutong Zhang
EPI_ISL_426626 hCoV-19/USA/NY-NYUMC171/2020 North America / USA / New York / Brooklyn 2020-04-01 NYU Langone Health Departments of Pathology and Medicine, New York University School of Medicine Maria Aguero-Rosenfeld, Brendan Belovarac, Margaret Black, Ludovic Boytard, John Cadley, Paolo Cotzia, John Chen, Dacia Dimartino, Xiaojun Feng, Tatyana Gindin, Emily Guzman, Adriana Heguy, Megan Hogan, Emily Huang, George Jour, Andrew Lytle, Christian Marier, Matthew T. Maurano, Mark J. Mulligan, Peter Meyn, Iman Osman, Jared Pinnell, Vanessa Raabe, Sitharam Ramaswami, Amy Rapkiewicz, Marie Samanovic-Golden, Antonio Serrano, Guomiao Shen, Matija Snuderl, Theodore Vougiouklakis, Nick Vulpescu, Gael Westby, Paul Zappile, Yutong Zhang
EPI_ISL_426627 hCoV-19/USA/LA-BIE-096/2020 North America / USA / Louisiana 2020-04-06 Ochsner Health BioInfoExperts, LLC Amy Feehan, David Nolan, Rebecca Rose, Susanna Lamers, Sissy Cross, Julia-Garcia-Diaz, Tong Yang, Luke Caruso, David Moraga Amador, Wayra Navia, Lydia Von Borstel, Xiao Hui Zhou
EPI_ISL_426628 hCoV-19/USA/LA-BIE-097/2020 North America / USA / Louisiana 2020-04-06 Ochsner Health BioInfoExperts, LLC Amy Feehan, David Nolan, Rebecca Rose, Susanna Lamers, Sissy Cross, Julia-Garcia-Diaz, Tong Yang, Luke Caruso, David Moraga Amador, Wayra Navia, Lydia Von Borstel, Xiao Hui Zhou
EPI_ISL_426629 hCoV-19/Taiwan/TSGH-01/2020 Asia / Taiwan / Taipei 2020-02-07 TSGH-CP molecular lab TSGH-CP molecular lab Cherng-Lih Perng, Ming-Jr Jian, Chih-Kai Chang, Jung-Chung Lin, Kuo-Ming Yeh, Chien-Wen Chen, Sheng-Kang Chiu, Hsing-Yi Chung, Shih-Hung Tsai, Kuo-Sheng Hung, Feng-Yee Chang, Hung-Sheng Shang
EPI_ISL_426637 hCoV-19/Australia/VIC327/2020 Oceania / Australia / Victoria 2020-03-17 Victorian Infectious Diseases Reference Laboratory (VIDRL) Microbiological Diagnostic Unit Public Health Laboratory and Victorian Infectious Diseases Reference Laboratory, Doherty Institute Caly L., Seemann T., Sait, M., Schultz M., Druce J., Sherry, N.
EPI_ISL_426638 hCoV-19/Australia/VIC328/2020 Oceania / Australia / Victoria 2020-03-17 Victorian Infectious Diseases Reference Laboratory (VIDRL) Microbiological Diagnostic Unit Public Health Laboratory and Victorian Infectious Diseases Reference Laboratory, Doherty Institute Caly L., Seemann T., Sait, M., Schultz M., Druce J., Sherry, N.
EPI_ISL_426639 hCoV-19/Australia/VIC329/2020 Oceania / Australia / Victoria 2020-03-17 Victorian Infectious Diseases Reference Laboratory (VIDRL) Microbiological Diagnostic Unit Public Health Laboratory and Victorian Infectious Diseases Reference Laboratory, Doherty Institute Caly L., Seemann T., Sait, M., Schultz M., Druce J., Sherry, N.
EPI_ISL_426640 hCoV-19/Australia/VIC330/2020 Oceania / Australia / Victoria 2020-03-18 Victorian Infectious Diseases Reference Laboratory (VIDRL) Microbiological Diagnostic Unit Public Health Laboratory and Victorian Infectious Diseases Reference Laboratory, Doherty Institute Caly L., Seemann T., Sait, M., Schultz M., Druce J., Sherry, N.

EPI_ISL_426641 hCoV-19/Australia/VIC332/2020 Oceania / Australia / Victoria 2020-03-18 Victorian Infectious Diseases Reference Laboratory (VIDRL) Microbiological Diagnostic Unit Public Health Laboratory and Victorian Infectious Diseases Reference Laboratory, Doherty Institute Caly L., Seemann T., Sait, M., Schultz M., Druce J., Sherry, N.
EPI_ISL_426642 hCoV-19/Australia/VIC333/2020 Oceania / Australia / Victoria 2020-03-18 Victorian Infectious Diseases Reference Laboratory (VIDRL) Microbiological Diagnostic Unit Public Health Laboratory and Victorian Infectious Diseases Reference Laboratory, Doherty Institute Caly L., Seemann T., Sait, M., Schultz M., Druce J., Sherry, N.
EPI_ISL_426643 hCoV-19/Australia/VIC334/2020 Oceania / Australia / Victoria 2020-03-19 Victorian Infectious Diseases Reference Laboratory (VIDRL) Microbiological Diagnostic Unit Public Health Laboratory and Victorian Infectious Diseases Reference Laboratory, Doherty Institute Caly L., Seemann T., Sait, M., Schultz M., Druce J., Sherry, N.
EPI_ISL_426644 hCoV-19/Australia/VIC335/2020 Oceania / Australia / Victoria 2020-03-20 Victorian Infectious Diseases Reference Laboratory (VIDRL) Microbiological Diagnostic Unit Public Health Laboratory and Victorian Infectious Diseases Reference Laboratory, Doherty Institute Caly L., Seemann T., Sait, M., Schultz M., Druce J., Sherry, N.
EPI_ISL_426645 hCoV-19/Australia/VIC336/2020 Oceania / Australia / Victoria 2020-03-20 Victorian Infectious Diseases Reference Laboratory (VIDRL) Microbiological Diagnostic Unit Public Health Laboratory and Victorian Infectious Diseases Reference Laboratory, Doherty Institute Caly L., Seemann T., Sait, M., Schultz M., Druce J., Sherry, N.
EPI_ISL_426646 hCoV-19/Australia/VIC337/2020 Oceania / Australia / Victoria 2020-03-20 Victorian Infectious Diseases Reference Laboratory (VIDRL) Microbiological Diagnostic Unit Public Health Laboratory and Victorian Infectious Diseases Reference Laboratory, Doherty Institute Caly L., Seemann T., Sait, M., Schultz M., Druce J., Sherry, N.
EPI_ISL_426647 hCoV-19/Australia/VIC338/2020 Oceania / Australia / Victoria 2020-03-20 Victorian Infectious Diseases Reference Laboratory (VIDRL) Microbiological Diagnostic Unit Public Health Laboratory and Victorian Infectious Diseases Reference Laboratory, Doherty Institute Caly L., Seemann T., Sait, M., Schultz M., Druce J., Sherry, N.
EPI_ISL_426648 hCoV-19/Australia/VIC339/2020 Oceania / Australia / Victoria 2020-03-20 Victorian Infectious Diseases Reference Laboratory (VIDRL) Microbiological Diagnostic Unit Public Health Laboratory and Victorian Infectious Diseases Reference Laboratory, Doherty Institute Caly L., Seemann T., Sait, M., Schultz M., Druce J., Sherry, N.
EPI_ISL_426649 hCoV-19/Australia/VIC340/2020 Oceania / Australia / Victoria 2020-03-20 Victorian Infectious Diseases Reference Laboratory (VIDRL) Microbiological Diagnostic Unit Public Health Laboratory and Victorian Infectious Diseases Reference Laboratory, Doherty Institute Caly L., Seemann T., Sait, M., Schultz M., Druce J., Sherry, N.
EPI_ISL_426650 hCoV-19/Australia/VIC341/2020 Oceania / Australia / Victoria 2020-03-20 Victorian Infectious Diseases Reference Laboratory (VIDRL) Microbiological Diagnostic Unit Public Health Laboratory and Victorian Infectious Diseases Reference Laboratory, Doherty Institute Caly L., Seemann T., Sait, M., Schultz M., Druce J., Sherry, N.
EPI_ISL_426651 hCoV-19/Australia/VIC342/2020 Oceania / Australia / Victoria 2020-03-20 Victorian Infectious Diseases Reference Laboratory (VIDRL) Microbiological Diagnostic Unit Public Health Laboratory and Victorian Infectious Diseases Reference Laboratory, Doherty Institute Caly L., Seemann T., Sait, M., Schultz M., Druce J., Sherry, N.
EPI_ISL_426652 hCoV-19/Australia/VIC343/2020 Oceania / Australia / Victoria 2020-03-20 Victorian Infectious Diseases Reference Laboratory (VIDRL) Microbiological Diagnostic Unit Public Health Laboratory and Victorian Infectious Diseases Reference Laboratory, Doherty Institute Caly L., Seemann T., Sait, M., Schultz M., Druce J., Sherry, N.
EPI_ISL_426653 hCoV-19/Australia/VIC344/2020 Oceania / Australia / Victoria 2020-03-20 Victorian Infectious Diseases Reference Laboratory (VIDRL) Microbiological Diagnostic Unit Public Health Laboratory and Victorian Infectious Diseases Reference Laboratory, Doherty Institute Caly L., Seemann T., Sait, M., Schultz M., Druce J., Sherry, N.
EPI_ISL_426654 hCoV-19/Australia/VIC345/2020 Oceania / Australia / Victoria 2020-03-20 Victorian Infectious Diseases Reference Laboratory (VIDRL) Microbiological Diagnostic Unit Public Health Laboratory and Victorian Infectious Diseases Reference Laboratory, Doherty Institute Caly L., Seemann T., Sait, M., Schultz M., Druce J., Sherry, N.
EPI_ISL_426655 hCoV-19/Australia/VIC346/2020 Oceania / Australia / Victoria 2020-03-20 Victorian Infectious Diseases Reference Laboratory (VIDRL) Microbiological Diagnostic Unit Public Health Laboratory and Victorian Infectious Diseases Reference Laboratory, Doherty Institute Caly L., Seemann T., Sait, M., Schultz M., Druce J., Sherry, N.
EPI_ISL_426656 hCoV-19/Australia/VIC347/2020 Oceania / Australia / Victoria 2020-03-20 Victorian Infectious Diseases Reference Laboratory (VIDRL) Microbiological Diagnostic Unit Public Health Laboratory and Victorian Infectious Diseases Reference Laboratory, Doherty Institute Caly L., Seemann T., Sait, M., Schultz M., Druce J., Sherry, N.
EPI_ISL_426657 hCoV-19/Australia/VIC348/2020 Oceania / Australia / Victoria 2020-03-20 Victorian Infectious Diseases Reference Laboratory (VIDRL) Microbiological Diagnostic Unit Public Health Laboratory and Victorian Infectious Diseases Reference Laboratory, Doherty Institute Caly L., Seemann T., Sait, M., Schultz M., Druce J., Sherry, N.
EPI_ISL_426658 hCoV-19/Australia/VIC350/2020 Oceania / Australia / Victoria 2020-03-20 Victorian Infectious Diseases Reference Laboratory (VIDRL) Microbiological Diagnostic Unit Public Health

Laboratory and Victorian Infectious Diseases Reference Laboratory, Doherty Institute Caly L., Seemann T., Sait, M., Schultz M., Druce J., Sherry, N.
EPI_ISL_426659 hCoV-19/Australia/VIC351/2020 Oceania / Australia / Victoria 2020-03-20 Victorian Infectious Diseases Reference Laboratory (VIDRL) Microbiological Diagnostic Unit Public Health Laboratory and Victorian Infectious Diseases Reference Laboratory, Doherty Institute Caly L., Seemann T., Sait, M., Schultz M., Druce J., Sherry, N.
EPI_ISL_426661 hCoV-19/Australia/VIC353/2020 Oceania / Australia / Victoria 2020-03-21 Victorian Infectious Diseases Reference Laboratory (VIDRL) Microbiological Diagnostic Unit Public Health Laboratory and Victorian Infectious Diseases Reference Laboratory, Doherty Institute Caly L., Seemann T., Sait, M., Schultz M., Druce J., Sherry, N.
EPI_ISL_426662 hCoV-19/Australia/VIC354/2020 Oceania / Australia / Victoria 2020-03-21 Victorian Infectious Diseases Reference Laboratory (VIDRL) Microbiological Diagnostic Unit Public Health Laboratory and Victorian Infectious Diseases Reference Laboratory, Doherty Institute Caly L., Seemann T., Sait, M., Schultz M., Druce J., Sherry, N.
EPI_ISL_426663 hCoV-19/Australia/VIC355/2020 Oceania / Australia / Victoria 2020-03-21 Victorian Infectious Diseases Reference Laboratory (VIDRL) Microbiological Diagnostic Unit Public Health Laboratory and Victorian Infectious Diseases Reference Laboratory, Doherty Institute Caly L., Seemann T., Sait, M., Schultz M., Druce J., Sherry, N.
EPI_ISL_426664 hCoV-19/Australia/VIC356/2020 Oceania / Australia / Victoria 2020-03-21 Victorian Infectious Diseases Reference Laboratory (VIDRL) Microbiological Diagnostic Unit Public Health Laboratory and Victorian Infectious Diseases Reference Laboratory, Doherty Institute Caly L., Seemann T., Sait, M., Schultz M., Druce J., Sherry, N.
EPI_ISL_426665 hCoV-19/Australia/VIC357/2020 Oceania / Australia / Victoria 2020-03-21 Victorian Infectious Diseases Reference Laboratory (VIDRL) Microbiological Diagnostic Unit Public Health Laboratory and Victorian Infectious Diseases Reference Laboratory, Doherty Institute Caly L., Seemann T., Sait, M., Schultz M., Druce J., Sherry, N.
EPI_ISL_426666 hCoV-19/Australia/VIC358/2020 Oceania / Australia / Victoria 2020-03-21 Victorian Infectious Diseases Reference Laboratory (VIDRL) Microbiological Diagnostic Unit Public Health Laboratory and Victorian Infectious Diseases Reference Laboratory, Doherty Institute Caly L., Seemann T., Sait, M., Schultz M., Druce J., Sherry, N.
EPI_ISL_426667 hCoV-19/Australia/VIC359/2020 Oceania / Australia / Victoria 2020-03-22 Victorian Infectious Diseases Reference Laboratory (VIDRL) Microbiological Diagnostic Unit Public Health Laboratory and Victorian Infectious Diseases Reference Laboratory, Doherty Institute Caly L., Seemann T., Sait, M., Schultz M., Druce J., Sherry, N.
EPI_ISL_426669 hCoV-19/Australia/VIC361/2020 Oceania / Australia / Victoria 2020-03-22 Victorian Infectious Diseases Reference Laboratory (VIDRL) Microbiological Diagnostic Unit Public Health Laboratory and Victorian Infectious Diseases Reference Laboratory, Doherty Institute Caly L., Seemann T., Sait, M., Schultz M., Druce J., Sherry, N.
EPI_ISL_426670 hCoV-19/Australia/VIC362/2020 Oceania / Australia / Victoria 2020-03-22 Victorian Infectious Diseases Reference Laboratory (VIDRL) Microbiological Diagnostic Unit Public Health Laboratory and Victorian Infectious Diseases Reference Laboratory, Doherty Institute Caly L., Seemann T., Sait, M., Schultz M., Druce J., Sherry, N.
EPI_ISL_426671 hCoV-19/Australia/VIC363/2020 Oceania / Australia / Victoria 2020-03-22 Victorian Infectious Diseases Reference Laboratory (VIDRL) Microbiological Diagnostic Unit Public Health Laboratory and Victorian Infectious Diseases Reference Laboratory, Doherty Institute Caly L., Seemann T., Sait, M., Schultz M., Druce J., Sherry, N.
EPI_ISL_426672 hCoV-19/Australia/VIC364/2020 Oceania / Australia / Victoria 2020-03-22 Victorian Infectious Diseases Reference Laboratory (VIDRL) Microbiological Diagnostic Unit Public Health Laboratory and Victorian Infectious Diseases Reference Laboratory, Doherty Institute Caly L., Seemann T., Sait, M., Schultz M., Druce J., Sherry, N.
EPI_ISL_426673 hCoV-19/Australia/VIC365/2020 Oceania / Australia / Victoria 2020-03-22 Victorian Infectious Diseases Reference Laboratory (VIDRL) Microbiological Diagnostic Unit Public Health Laboratory and Victorian Infectious Diseases Reference Laboratory, Doherty Institute Caly L., Seemann T., Sait, M., Schultz M., Druce J., Sherry, N.
EPI_ISL_426674 hCoV-19/Australia/VIC366/2020 Oceania / Australia / Victoria 2020-03-22 Victorian Infectious Diseases Reference Laboratory (VIDRL) Microbiological Diagnostic Unit Public Health Laboratory and Victorian Infectious Diseases Reference Laboratory, Doherty Institute Caly L., Seemann T., Sait, M., Schultz M., Druce J., Sherry, N.
EPI_ISL_426675 hCoV-19/Australia/VIC367/2020 Oceania / Australia / Victoria 2020-03-22 Victorian Infectious Diseases Reference Laboratory (VIDRL) Microbiological Diagnostic Unit Public Health Laboratory and Victorian Infectious Diseases Reference Laboratory, Doherty Institute Caly L., Seemann T., Sait, M., Schultz M., Druce J., Sherry, N.
EPI_ISL_426676 hCoV-19/Australia/VIC368/2020 Oceania / Australia / Victoria 2020-03-22 Victorian Infectious Diseases Reference Laboratory (VIDRL) Microbiological Diagnostic Unit Public Health Laboratory and Victorian Infectious Diseases Reference Laboratory, Doherty Institute Caly L., Seemann T., Sait, M., Schultz M., Druce J., Sherry, N.
EPI_ISL_426677 hCoV-19/Australia/VIC369/2020 Oceania / Australia / Victoria 2020-03-22 Victorian Infectious Diseases Reference Laboratory (VIDRL) Microbiological Diagnostic Unit Public Health Laboratory and Victorian Infectious Diseases Reference Laboratory, Doherty Institute Caly L., Seemann T., Sait, M., Schultz M., Druce J., Sherry, N.

EPI_ISL_426679 hCoV-19/Australia/VIC372/2020 Oceania / Australia / Victoria 2020-03-22 Victorian Infectious Diseases Reference Laboratory (VIDRL) Microbiological Diagnostic Unit Public Health Laboratory and Victorian Infectious Diseases Reference Laboratory, Doherty Institute Caly L., Seemann T., Sait, M., Schultz M., Druce J., Sherry, N.
EPI_ISL_426680 hCoV-19/Australia/VIC373/2020 Oceania / Australia / Victoria 2020-03-22 Victorian Infectious Diseases Reference Laboratory (VIDRL) Microbiological Diagnostic Unit Public Health Laboratory and Victorian Infectious Diseases Reference Laboratory, Doherty Institute Caly L., Seemann T., Sait, M., Schultz M., Druce J., Sherry, N.
EPI_ISL_426682 hCoV-19/Australia/VIC375/2020 Oceania / Australia / Victoria 2020-03-22 Victorian Infectious Diseases Reference Laboratory (VIDRL) Microbiological Diagnostic Unit Public Health Laboratory and Victorian Infectious Diseases Reference Laboratory, Doherty Institute Caly L., Seemann T., Sait, M., Schultz M., Druce J., Sherry, N.
EPI_ISL_426683 hCoV-19/Australia/VIC376/2020 Oceania / Australia / Victoria 2020-03-22 Victorian Infectious Diseases Reference Laboratory (VIDRL) Microbiological Diagnostic Unit Public Health Laboratory and Victorian Infectious Diseases Reference Laboratory, Doherty Institute Caly L., Seemann T., Sait, M., Schultz M., Druce J., Sherry, N.
EPI_ISL_426684 hCoV-19/Australia/VIC377/2020 Oceania / Australia / Victoria 2020-03-22 Victorian Infectious Diseases Reference Laboratory (VIDRL) Microbiological Diagnostic Unit Public Health Laboratory and Victorian Infectious Diseases Reference Laboratory, Doherty Institute Caly L., Seemann T., Sait, M., Schultz M., Druce J., Sherry, N.
EPI_ISL_426685 hCoV-19/Australia/VIC378/2020 Oceania / Australia / Victoria 2020-03-22 Victorian Infectious Diseases Reference Laboratory (VIDRL) Microbiological Diagnostic Unit Public Health Laboratory and Victorian Infectious Diseases Reference Laboratory, Doherty Institute Caly L., Seemann T., Sait, M., Schultz M., Druce J., Sherry, N.
EPI_ISL_426686 hCoV-19/Australia/VIC379/2020 Oceania / Australia / Victoria 2020-03-22 Victorian Infectious Diseases Reference Laboratory (VIDRL) Microbiological Diagnostic Unit Public Health Laboratory and Victorian Infectious Diseases Reference Laboratory, Doherty Institute Caly L., Seemann T., Sait, M., Schultz M., Druce J., Sherry, N.
EPI_ISL_426687 hCoV-19/Australia/VIC380/2020 Oceania / Australia / Victoria 2020-03-23 Victorian Infectious Diseases Reference Laboratory (VIDRL) Microbiological Diagnostic Unit Public Health Laboratory and Victorian Infectious Diseases Reference Laboratory, Doherty Institute Caly L., Seemann T., Sait, M., Schultz M., Druce J., Sherry, N.
EPI_ISL_426688 hCoV-19/Australia/VIC381/2020 Oceania / Australia / Victoria 2020-03-23 Victorian Infectious Diseases Reference Laboratory (VIDRL) Microbiological Diagnostic Unit Public Health Laboratory and Victorian Infectious Diseases Reference Laboratory, Doherty Institute Caly L., Seemann T., Sait, M., Schultz M., Druce J., Sherry, N.
EPI_ISL_426689 hCoV-19/Australia/VIC382/2020 Oceania / Australia / Victoria 2020-03-23 Victorian Infectious Diseases Reference Laboratory (VIDRL) Microbiological Diagnostic Unit Public Health Laboratory and Victorian Infectious Diseases Reference Laboratory, Doherty Institute Caly L., Seemann T., Sait, M., Schultz M., Druce J., Sherry, N.
EPI_ISL_426690 hCoV-19/Australia/VIC383/2020 Oceania / Australia / Victoria 2020-03-23 Victorian Infectious Diseases Reference Laboratory (VIDRL) Microbiological Diagnostic Unit Public Health Laboratory and Victorian Infectious Diseases Reference Laboratory, Doherty Institute Caly L., Seemann T., Sait, M., Schultz M., Druce J., Sherry, N.
EPI_ISL_426691 hCoV-19/Australia/VIC384/2020 Oceania / Australia / Victoria 2020-03-23 Victorian Infectious Diseases Reference Laboratory (VIDRL) Microbiological Diagnostic Unit Public Health Laboratory and Victorian Infectious Diseases Reference Laboratory, Doherty Institute Caly L., Seemann T., Sait, M., Schultz M., Druce J., Sherry, N.
EPI_ISL_426692 hCoV-19/Australia/VIC385/2020 Oceania / Australia / Victoria 2020-03-23 Victorian Infectious Diseases Reference Laboratory (VIDRL) Microbiological Diagnostic Unit Public Health Laboratory and Victorian Infectious Diseases Reference Laboratory, Doherty Institute Caly L., Seemann T., Sait, M., Schultz M., Druce J., Sherry, N.
EPI_ISL_426693 hCoV-19/Australia/VIC386/2020 Oceania / Australia / Victoria 2020-03-23 Victorian Infectious Diseases Reference Laboratory (VIDRL) Microbiological Diagnostic Unit Public Health Laboratory and Victorian Infectious Diseases Reference Laboratory, Doherty Institute Caly L., Seemann T., Sait, M., Schultz M., Druce J., Sherry, N.
EPI_ISL_426694 hCoV-19/Australia/VIC387/2020 Oceania / Australia / Victoria 2020-03-23 Victorian Infectious Diseases Reference Laboratory (VIDRL) Microbiological Diagnostic Unit Public Health Laboratory and Victorian Infectious Diseases Reference Laboratory, Doherty Institute Caly L., Seemann T., Sait, M., Schultz M., Druce J., Sherry, N.
EPI_ISL_426696 hCoV-19/Australia/VIC389/2020 Oceania / Australia / Victoria 2020-03-23 Victorian Infectious Diseases Reference Laboratory (VIDRL) Microbiological Diagnostic Unit Public Health Laboratory and Victorian Infectious Diseases Reference Laboratory, Doherty Institute Caly L., Seemann T., Sait, M., Schultz M., Druce J., Sherry, N.
EPI_ISL_426697 hCoV-19/Australia/VIC394/2020 Oceania / Australia / Victoria 2020-03-24 Victorian Infectious Diseases Reference Laboratory (VIDRL) Microbiological Diagnostic Unit Public Health Laboratory and Victorian Infectious Diseases Reference Laboratory, Doherty Institute Caly L., Seemann T., Sait, M., Schultz M., Druce J., Sherry, N.
EPI_ISL_426698 hCoV-19/Australia/VIC395/2020 Oceania / Australia / Victoria 2020-03-24 Victorian Infectious Diseases Reference Laboratory (VIDRL) Microbiological Diagnostic Unit Public Health

Laboratory and Victorian Infectious Diseases Reference Laboratory, Doherty Institute Caly L., Seemann T., Sait, M., Schultz M., Druce J., Sherry, N.
EPI_ISL_426914 hCoV-19/Australia/VIC483/2020 Oceania / Australia / Victoria 2020-03-29 Microbiological Diagnostic Unit Public Health Laboratory Microbiological Diagnostic Unit Public Health Laboratory Seemann T., Schultz M., Sait, M., Sherry, N.
EPI_ISL_426917 hCoV-19/Australia/VIC486/2020 Oceania / Australia / Victoria 2020-03-29 Microbiological Diagnostic Unit Public Health Laboratory Microbiological Diagnostic Unit Public Health Laboratory Seemann T., Schultz M., Sait, M., Sherry, N.
EPI_ISL_426918 hCoV-19/Australia/VIC487/2020 Oceania / Australia / Victoria 2020-03-29 Microbiological Diagnostic Unit Public Health Laboratory Microbiological Diagnostic Unit Public Health Laboratory Seemann T., Schultz M., Sait, M., Sherry, N.
EPI_ISL_426919 hCoV-19/Australia/VIC488/2020 Oceania / Australia / Victoria 2020-03-29 Microbiological Diagnostic Unit Public Health Laboratory Microbiological Diagnostic Unit Public Health Laboratory Seemann T., Schultz M., Sait, M., Sherry, N.
EPI_ISL_426920 hCoV-19/Australia/VIC489/2020 Oceania / Australia / Victoria 2020-03-29 Microbiological Diagnostic Unit Public Health Laboratory Microbiological Diagnostic Unit Public Health Laboratory Seemann T., Schultz M., Sait, M., Sherry, N.
EPI_ISL_426921 hCoV-19/Australia/VIC490/2020 Oceania / Australia / Victoria 2020-03-30 Microbiological Diagnostic Unit Public Health Laboratory Microbiological Diagnostic Unit Public Health Laboratory Seemann T., Schultz M., Sait, M., Sherry, N.
EPI_ISL_426922 hCoV-19/Australia/VIC491/2020 Oceania / Australia / Victoria 2020-03-30 Microbiological Diagnostic Unit Public Health Laboratory Microbiological Diagnostic Unit Public Health Laboratory Seemann T., Schultz M., Sait, M., Sherry, N.
EPI_ISL_426923 hCoV-19/Australia/VIC619/2020 Oceania / Australia / Victoria 2020-03-28 Victorian Infectious Diseases Reference Laboratory (VIDRL) Microbiological Diagnostic Unit Public Health Laboratory and Victorian Infectious Diseases Reference Laboratory, Doherty Institute Caly L., Seemann T., Sait, M., Schultz M., Druce J., Sherry, N.
EPI_ISL_426924 hCoV-19/Australia/VIC620/2020 Oceania / Australia / Victoria 2020-03-28 Victorian Infectious Diseases Reference Laboratory (VIDRL) Microbiological Diagnostic Unit Public Health Laboratory and Victorian Infectious Diseases Reference Laboratory, Doherty Institute Caly L., Seemann T., Sait, M., Schultz M., Druce J., Sherry, N.
EPI_ISL_426926 hCoV-19/Australia/VIC622/2020 Oceania / Australia / Victoria 2020-03-29 Victorian Infectious Diseases Reference Laboratory (VIDRL) Microbiological Diagnostic Unit Public Health Laboratory and Victorian Infectious Diseases Reference Laboratory, Doherty Institute Caly L., Seemann T., Sait, M., Schultz M., Druce J., Sherry, N.
EPI_ISL_426927 hCoV-19/Australia/VIC623/2020 Oceania / Australia / Victoria 2020-03-28 Victorian Infectious Diseases Reference Laboratory (VIDRL) Microbiological Diagnostic Unit Public Health Laboratory and Victorian Infectious Diseases Reference Laboratory, Doherty Institute Caly L., Seemann T., Sait, M., Schultz M., Druce J., Sherry, N.
EPI_ISL_426929 hCoV-19/Australia/VIC625/2020 Oceania / Australia / Victoria 2020-03-28 Victorian Infectious Diseases Reference Laboratory (VIDRL) Microbiological Diagnostic Unit Public Health Laboratory and Victorian Infectious Diseases Reference Laboratory, Doherty Institute Caly L., Seemann T., Sait, M., Schultz M., Druce J., Sherry, N.
EPI_ISL_426930 hCoV-19/Australia/VIC626/2020 Oceania / Australia / Victoria 2020-03-28 Victorian Infectious Diseases Reference Laboratory (VIDRL) Microbiological Diagnostic Unit Public Health Laboratory and Victorian Infectious Diseases Reference Laboratory, Doherty Institute Caly L., Seemann T., Sait, M., Schultz M., Druce J., Sherry, N.
EPI_ISL_426931 hCoV-19/Australia/VIC627/2020 Oceania / Australia / Victoria 2020-03-29 Victorian Infectious Diseases Reference Laboratory (VIDRL) Microbiological Diagnostic Unit Public Health Laboratory and Victorian Infectious Diseases Reference Laboratory, Doherty Institute Caly L., Seemann T., Sait, M., Schultz M., Druce J., Sherry, N.
EPI_ISL_426932 hCoV-19/Australia/VIC628/2020 Oceania / Australia / Victoria 2020-03-29 Victorian Infectious Diseases Reference Laboratory (VIDRL) Microbiological Diagnostic Unit Public Health Laboratory and Victorian Infectious Diseases Reference Laboratory, Doherty Institute Caly L., Seemann T., Sait, M., Schultz M., Druce J., Sherry, N.
EPI_ISL_426933 hCoV-19/Australia/VIC637/2020 Oceania / Australia / Victoria 2020-03-28 Victorian Infectious Diseases Reference Laboratory (VIDRL) Microbiological Diagnostic Unit Public Health Laboratory and Victorian Infectious Diseases Reference Laboratory, Doherty Institute Caly L., Seemann T., Sait, M., Schultz M., Druce J., Sherry, N.
EPI_ISL_426934 hCoV-19/Australia/VIC638/2020 Oceania / Australia / Victoria 2020-03-29 Victorian Infectious Diseases Reference Laboratory (VIDRL) Microbiological Diagnostic Unit Public Health Laboratory and Victorian Infectious Diseases Reference Laboratory, Doherty Institute Caly L., Seemann T., Sait, M., Schultz M., Druce J., Sherry, N.
EPI_ISL_426935 hCoV-19/Australia/VIC639/2020 Oceania / Australia / Victoria 2020-03-29 Victorian Infectious Diseases Reference Laboratory (VIDRL) Microbiological Diagnostic Unit Public Health Laboratory and Victorian Infectious Diseases Reference Laboratory, Doherty Institute Caly L., Seemann T., Sait, M., Schultz M., Druce J., Sherry, N.
EPI_ISL_426940 hCoV-19/Australia/VIC644/2020 Oceania / Australia / Victoria 2020-03-28 Victorian Infectious Diseases Reference Laboratory (VIDRL) Microbiological Diagnostic Unit Public Health Laboratory and Victorian Infectious Diseases Reference Laboratory, Doherty Institute Caly L., Seemann T.,

Sait, M., Schultz M., Druce J., Sherry, N.
EPI_ISL_426941 hCoV-19/Australia/VIC645/2020 Oceania / Australia / Victoria 2020-03-28 Victorian Infectious Diseases Reference Laboratory (VIDRL) Microbiological Diagnostic Unit Public Health Laboratory and Victorian Infectious Diseases Reference Laboratory, Doherty Institute Caly L., Seemann T., Sait, M., Schultz M., Druce J., Sherry, N.
EPI_ISL_426942 hCoV-19/Australia/VIC646/2020 Oceania / Australia / Victoria 2020-03-24 Victorian Infectious Diseases Reference Laboratory (VIDRL) Microbiological Diagnostic Unit Public Health Laboratory and Victorian Infectious Diseases Reference Laboratory, Doherty Institute Caly L., Seemann T., Sait, M., Schultz M., Druce J., Sherry, N.
EPI_ISL_426943 hCoV-19/Australia/VIC647/2020 Oceania / Australia / Victoria 2020-03-28 Victorian Infectious Diseases Reference Laboratory (VIDRL) Microbiological Diagnostic Unit Public Health Laboratory and Victorian Infectious Diseases Reference Laboratory, Doherty Institute Caly L., Seemann T., Sait, M., Schultz M., Druce J., Sherry, N.
EPI_ISL_426947 hCoV-19/Australia/VIC651/2020 Oceania / Australia / Victoria 2020-03-28 Victorian Infectious Diseases Reference Laboratory (VIDRL) Microbiological Diagnostic Unit Public Health Laboratory and Victorian Infectious Diseases Reference Laboratory, Doherty Institute Caly L., Seemann T., Sait, M., Schultz M., Druce J., Sherry, N.
EPI_ISL_426948 hCoV-19/Australia/VIC652/2020 Oceania / Australia / Victoria 2020-03-28 Victorian Infectious Diseases Reference Laboratory (VIDRL) Microbiological Diagnostic Unit Public Health Laboratory and Victorian Infectious Diseases Reference Laboratory, Doherty Institute Caly L., Seemann T., Sait, M., Schultz M., Druce J., Sherry, N.
EPI_ISL_426951 hCoV-19/Australia/VIC655/2020 Oceania / Australia / Victoria 2020-03-28 Victorian Infectious Diseases Reference Laboratory (VIDRL) Microbiological Diagnostic Unit Public Health Laboratory and Victorian Infectious Diseases Reference Laboratory, Doherty Institute Caly L., Seemann T., Sait, M., Schultz M., Druce J., Sherry, N.
EPI_ISL_426952 hCoV-19/Australia/VIC656/2020 Oceania / Australia / Victoria 2020-03-28 Victorian Infectious Diseases Reference Laboratory (VIDRL) Microbiological Diagnostic Unit Public Health Laboratory and Victorian Infectious Diseases Reference Laboratory, Doherty Institute Caly L., Seemann T., Sait, M., Schultz M., Druce J., Sherry, N.
EPI_ISL_426953 hCoV-19/Australia/VIC657/2020 Oceania / Australia / Victoria 2020-03-28 Victorian Infectious Diseases Reference Laboratory (VIDRL) Microbiological Diagnostic Unit Public Health Laboratory and Victorian Infectious Diseases Reference Laboratory, Doherty Institute Caly L., Seemann T., Sait, M., Schultz M., Druce J., Sherry, N.
EPI_ISL_426954 hCoV-19/Australia/VIC658/2020 Oceania / Australia / Victoria 2020-03-28 Victorian Infectious Diseases Reference Laboratory (VIDRL) Microbiological Diagnostic Unit Public Health Laboratory and Victorian Infectious Diseases Reference Laboratory, Doherty Institute Caly L., Seemann T., Sait, M., Schultz M., Druce J., Sherry, N.
EPI_ISL_426955 hCoV-19/Australia/VIC659/2020 Oceania / Australia / Victoria 2020-03-27 Victorian Infectious Diseases Reference Laboratory (VIDRL) Microbiological Diagnostic Unit Public Health Laboratory and Victorian Infectious Diseases Reference Laboratory, Doherty Institute Caly L., Seemann T., Sait, M., Schultz M., Druce J., Sherry, N.
EPI_ISL_426956 hCoV-19/Australia/VIC660/2020 Oceania / Australia / Victoria 2020-03-28 Victorian Infectious Diseases Reference Laboratory (VIDRL) Microbiological Diagnostic Unit Public Health Laboratory and Victorian Infectious Diseases Reference Laboratory, Doherty Institute Caly L., Seemann T., Sait, M., Schultz M., Druce J., Sherry, N.
EPI_ISL_426957 hCoV-19/Australia/VIC661/2020 Oceania / Australia / Victoria 2020-03-27 Victorian Infectious Diseases Reference Laboratory (VIDRL) Microbiological Diagnostic Unit Public Health Laboratory and Victorian Infectious Diseases Reference Laboratory, Doherty Institute Caly L., Seemann T., Sait, M., Schultz M., Druce J., Sherry, N.
EPI_ISL_426958 hCoV-19/Australia/VIC662/2020 Oceania / Australia / Victoria 2020-03-27 Victorian Infectious Diseases Reference Laboratory (VIDRL) Microbiological Diagnostic Unit Public Health Laboratory and Victorian Infectious Diseases Reference Laboratory, Doherty Institute Caly L., Seemann T., Sait, M., Schultz M., Druce J., Sherry, N.
EPI_ISL_426959 hCoV-19/Australia/VIC663/2020 Oceania / Australia / Victoria 2020-03-28 Victorian Infectious Diseases Reference Laboratory (VIDRL) Microbiological Diagnostic Unit Public Health Laboratory and Victorian Infectious Diseases Reference Laboratory, Doherty Institute Caly L., Seemann T., Sait, M., Schultz M., Druce J., Sherry, N.
EPI_ISL_426960 hCoV-19/Australia/VIC664/2020 Oceania / Australia / Victoria 2020-03-27 Victorian Infectious Diseases Reference Laboratory (VIDRL) Microbiological Diagnostic Unit Public Health Laboratory and Victorian Infectious Diseases Reference Laboratory, Doherty Institute Caly L., Seemann T., Sait, M., Schultz M., Druce J., Sherry, N.
EPI_ISL_426961 hCoV-19/Australia/VIC665/2020 Oceania / Australia / Victoria 2020-03-27 Victorian Infectious Diseases Reference Laboratory (VIDRL) Microbiological Diagnostic Unit Public Health Laboratory and Victorian Infectious Diseases Reference Laboratory, Doherty Institute Caly L., Seemann T., Sait, M., Schultz M., Druce J., Sherry, N.
EPI_ISL_426962 hCoV-19/Australia/VIC666/2020 Oceania / Australia / Victoria 2020-03-27 Victorian Infectious Diseases Reference Laboratory (VIDRL) Microbiological Diagnostic Unit Public Health Laboratory and Victorian Infectious Diseases Reference Laboratory, Doherty Institute Caly L., Seemann T., Sait, M., Schultz M., Druce J., Sherry, N.
EPI_ISL_426963 hCoV-19/Australia/VIC667/2020 Oceania / Australia / Victoria 2020-03-27 Victorian

Infectious Diseases Reference Laboratory (VIDRL)	Microbiological Diagnostic Unit Public Health Laboratory and Victorian Infectious Diseases Reference Laboratory, Doherty Institute	Caly L., Seemann T., Sait, M., Schultz M., Druce J., Sherry, N.
EPI_ISL_426964	hCoV-19/Australia/VIC668/2020	Oceania / Australia / Victoria	2020-03-27	Victorian Infectious Diseases Reference Laboratory (VIDRL)	Microbiological Diagnostic Unit Public Health Laboratory and Victorian Infectious Diseases Reference Laboratory, Doherty Institute	Caly L., Seemann T., Sait, M., Schultz M., Druce J., Sherry, N.
EPI_ISL_426968	hCoV-19/Australia/VIC672/2020	Oceania / Australia / Victoria	2020-03-27	Victorian Infectious Diseases Reference Laboratory (VIDRL)	Microbiological Diagnostic Unit Public Health Laboratory and Victorian Infectious Diseases Reference Laboratory, Doherty Institute	Caly L., Seemann T., Sait, M., Schultz M., Druce J., Sherry, N.
EPI_ISL_426970	hCoV-19/Australia/VIC674/2020	Oceania / Australia / Victoria	2020-03-27	Victorian Infectious Diseases Reference Laboratory (VIDRL)	Microbiological Diagnostic Unit Public Health Laboratory and Victorian Infectious Diseases Reference Laboratory, Doherty Institute	Caly L., Seemann T., Sait, M., Schultz M., Druce J., Sherry, N.
EPI_ISL_426971	hCoV-19/Australia/VIC676/2020	Oceania / Australia / Victoria	2020-03-27	Victorian Infectious Diseases Reference Laboratory (VIDRL)	Microbiological Diagnostic Unit Public Health Laboratory and Victorian Infectious Diseases Reference Laboratory, Doherty Institute	Caly L., Seemann T., Sait, M., Schultz M., Druce J., Sherry, N.
EPI_ISL_426972	hCoV-19/Australia/VIC677/2020	Oceania / Australia / Victoria	2020-03-27	Victorian Infectious Diseases Reference Laboratory (VIDRL)	Microbiological Diagnostic Unit Public Health Laboratory and Victorian Infectious Diseases Reference Laboratory, Doherty Institute	Caly L., Seemann T., Sait, M., Schultz M., Druce J., Sherry, N.
EPI_ISL_426973	hCoV-19/Australia/VIC678/2020	Oceania / Australia / Victoria	2020-03-27	Victorian Infectious Diseases Reference Laboratory (VIDRL)	Microbiological Diagnostic Unit Public Health Laboratory and Victorian Infectious Diseases Reference Laboratory, Doherty Institute	Caly L., Seemann T., Sait, M., Schultz M., Druce J., Sherry, N.
EPI_ISL_426974	hCoV-19/Australia/VIC679/2020	Oceania / Australia / Victoria	2020-03-27	Victorian Infectious Diseases Reference Laboratory (VIDRL)	Microbiological Diagnostic Unit Public Health Laboratory and Victorian Infectious Diseases Reference Laboratory, Doherty Institute	Caly L., Seemann T., Sait, M., Schultz M., Druce J., Sherry, N.
EPI_ISL_426975	hCoV-19/Australia/VIC680/2020	Oceania / Australia / Victoria	2020-03-27	Victorian Infectious Diseases Reference Laboratory (VIDRL)	Microbiological Diagnostic Unit Public Health Laboratory and Victorian Infectious Diseases Reference Laboratory, Doherty Institute	Caly L., Seemann T., Sait, M., Schultz M., Druce J., Sherry, N.
EPI_ISL_426976	hCoV-19/Australia/VIC681/2020	Oceania / Australia / Victoria	2020-03-27	Victorian Infectious Diseases Reference Laboratory (VIDRL)	Microbiological Diagnostic Unit Public Health Laboratory and Victorian Infectious Diseases Reference Laboratory, Doherty Institute	Caly L., Seemann T., Sait, M., Schultz M., Druce J., Sherry, N.
EPI_ISL_426977	hCoV-19/Australia/VIC682/2020	Oceania / Australia / Victoria	2020-03-29	Victorian Infectious Diseases Reference Laboratory (VIDRL)	Microbiological Diagnostic Unit Public Health Laboratory and Victorian Infectious Diseases Reference Laboratory, Doherty Institute	Caly L., Seemann T., Sait, M., Schultz M., Druce J., Sherry, N.
EPI_ISL_426978	hCoV-19/Australia/VIC683/2020	Oceania / Australia / Victoria	2020-03-29	Victorian Infectious Diseases Reference Laboratory (VIDRL)	Microbiological Diagnostic Unit Public Health Laboratory and Victorian Infectious Diseases Reference Laboratory, Doherty Institute	Caly L., Seemann T., Sait, M., Schultz M., Druce J., Sherry, N.
EPI_ISL_426980	hCoV-19/Australia/VIC685/2020	Oceania / Australia / Victoria	2020-03-28	Victorian Infectious Diseases Reference Laboratory (VIDRL)	Microbiological Diagnostic Unit Public Health Laboratory and Victorian Infectious Diseases Reference Laboratory, Doherty Institute	Caly L., Seemann T., Sait, M., Schultz M., Druce J., Sherry, N.
EPI_ISL_426981	hCoV-19/Australia/VIC687/2020	Oceania / Australia / Victoria	2020-03-29	Victorian Infectious Diseases Reference Laboratory (VIDRL)	Microbiological Diagnostic Unit Public Health Laboratory and Victorian Infectious Diseases Reference Laboratory, Doherty Institute	Caly L., Seemann T., Sait, M., Schultz M., Druce J., Sherry, N.
EPI_ISL_426986	hCoV-19/Australia/VIC694/2020	Oceania / Australia / Victoria	2020-03-28	Victorian Infectious Diseases Reference Laboratory (VIDRL)	Microbiological Diagnostic Unit Public Health Laboratory and Victorian Infectious Diseases Reference Laboratory, Doherty Institute	Caly L., Seemann T., Sait, M., Schultz M., Druce J., Sherry, N.
EPI_ISL_426988	hCoV-19/Australia/VIC696/2020	Oceania / Australia / Victoria	2020-03-28	Victorian Infectious Diseases Reference Laboratory (VIDRL)	Microbiological Diagnostic Unit Public Health Laboratory and Victorian Infectious Diseases Reference Laboratory, Doherty Institute	Caly L., Seemann T., Sait, M., Schultz M., Druce J., Sherry, N.
EPI_ISL_426989	hCoV-19/Australia/VIC697/2020	Oceania / Australia / Victoria	2020-03-28	Victorian Infectious Diseases Reference Laboratory (VIDRL)	Microbiological Diagnostic Unit Public Health Laboratory and Victorian Infectious Diseases Reference Laboratory, Doherty Institute	Caly L., Seemann T., Sait, M., Schultz M., Druce J., Sherry, N.
EPI_ISL_426993	hCoV-19/Australia/VIC706/2020	Oceania / Australia / Victoria	2020-03-30	Victorian Infectious Diseases Reference Laboratory (VIDRL)	Microbiological Diagnostic Unit Public Health Laboratory and Victorian Infectious Diseases Reference Laboratory, Doherty Institute	Caly L., Seemann T.,

Sait, M., Schultz M., Druce J., Sherry, N.
EPI_ISL_426994 hCoV-19/Australia/VIC707/2020 Oceania / Australia / Victoria 2020-03-29 Victorian Infectious Diseases Reference Laboratory (VIDRL) Microbiological Diagnostic Unit Public Health Laboratory and Victorian Infectious Diseases Reference Laboratory, Doherty Institute Caly L., Seemann T., Sait, M., Schultz M., Druce J., Sherry, N.
EPI_ISL_426995 hCoV-19/Australia/VIC708/2020 Oceania / Australia / Victoria 2020-03-29 Victorian Infectious Diseases Reference Laboratory (VIDRL) Microbiological Diagnostic Unit Public Health Laboratory and Victorian Infectious Diseases Reference Laboratory, Doherty Institute Caly L., Seemann T., Sait, M., Schultz M., Druce J., Sherry, N.
EPI_ISL_426997 hCoV-19/Australia/VIC710/2020 Oceania / Australia / Victoria 2020-03-29 Victorian Infectious Diseases Reference Laboratory (VIDRL) Microbiological Diagnostic Unit Public Health Laboratory and Victorian Infectious Diseases Reference Laboratory, Doherty Institute Caly L., Seemann T., Sait, M., Schultz M., Druce J., Sherry, N.
EPI_ISL_426999 hCoV-19/Australia/VIC712/2020 Oceania / Australia / Victoria 2020-03-29 Victorian Infectious Diseases Reference Laboratory (VIDRL) Microbiological Diagnostic Unit Public Health Laboratory and Victorian Infectious Diseases Reference Laboratory, Doherty Institute Caly L., Seemann T., Sait, M., Schultz M., Druce J., Sherry, N.
EPI_ISL_429196 hCoV-19/Switzerland/GE3144/2020 Europe / Switzerland 2020-03-18 University Hospitals of Geneva Laboratory of Virology University Hospitals of Geneva Laboratory of Virology Laubscher F.
EPI_ISL_429197 hCoV-19/Switzerland/GE0199-2/2020 Europe / Switzerland 2020-03-21 University Hospitals of Geneva Laboratory of Virology University Hospitals of Geneva Laboratory of Virology Laubscher F.
EPI_ISL_429199 hCoV-19/Switzerland/GE0636/2020 Europe / Switzerland 2020-03-22 University Hospitals of Geneva Laboratory of Virology University Hospitals of Geneva Laboratory of Virology Laubscher F.
EPI_ISL_429200 hCoV-19/Switzerland/GE5737/2020 Europe / Switzerland 2020-03-19 University Hospitals of Geneva Laboratory of Virology University Hospitals of Geneva Laboratory of Virology Laubscher F.
EPI_ISL_429201 hCoV-19/Switzerland/GE2297/2020 Europe / Switzerland 2020-03-29 University Hospitals of Geneva Laboratory of Virology University Hospitals of Geneva Laboratory of Virology Laubscher F.
EPI_ISL_429203 hCoV-19/Switzerland/GE1736/2020 Europe / Switzerland 2020-03-21 University Hospitals of Geneva Laboratory of Virology University Hospitals of Geneva Laboratory of Virology Laubscher F.
EPI_ISL_429204 hCoV-19/Switzerland/GE7617/2020 Europe / Switzerland 2020-04-03 University Hospitals of Geneva Laboratory of Virology University Hospitals of Geneva Laboratory of Virology Laubscher F.
EPI_ISL_429206 hCoV-19/Switzerland/GE5007/2020 Europe / Switzerland 2020-04-07 University Hospitals of Geneva Laboratory of Virology University Hospitals of Geneva Laboratory of Virology Laubscher F.
EPI_ISL_429207 hCoV-19/Switzerland/GE1184/2020 Europe / Switzerland 2020-04-03 University Hospitals of Geneva Laboratory of Virology University Hospitals of Geneva Laboratory of Virology Laubscher F.
EPI_ISL_429208 hCoV-19/Switzerland/GE2164/2020 Europe / Switzerland 2020-03-17 University Hospitals of Geneva Laboratory of Virology University Hospitals of Geneva Laboratory of Virology Laubscher F.
EPI_ISL_429209 hCoV-19/Switzerland/GE3650/2020 Europe / Switzerland 2020-03-16 University Hospitals of Geneva Laboratory of Virology University Hospitals of Geneva Laboratory of Virology Laubscher F.
EPI_ISL_429210 hCoV-19/Switzerland/GE8147/2020 Europe / Switzerland 2020-03-16 University Hospitals of Geneva Laboratory of Virology University Hospitals of Geneva Laboratory of Virology Laubscher F.
EPI_ISL_429211 hCoV-19/Switzerland/GE5708/2020 Europe / Switzerland 2020-03-24 University Hospitals of Geneva Laboratory of Virology University Hospitals of Geneva Laboratory of Virology Laubscher F.
EPI_ISL_429212 hCoV-19/Switzerland/GE0056/2020 Europe / Switzerland 2020-03-24 University Hospitals of Geneva Laboratory of Virology University Hospitals of Geneva Laboratory of Virology Laubscher F.
EPI_ISL_429213 hCoV-19/Switzerland/GE8086/2020 Europe / Switzerland 2020-03-16 University Hospitals of Geneva Laboratory of Virology University Hospitals of Geneva Laboratory of Virology Laubscher F.
EPI_ISL_429214 hCoV-19/Switzerland/GE2759/2020 Europe / Switzerland 2020-03-23 University Hospitals of Geneva Laboratory of Virology University Hospitals of Geneva Laboratory of Virology Laubscher F.
EPI_ISL_429215 hCoV-19/Switzerland/GE4807/2020 Europe / Switzerland 2020-03-23 University Hospitals of Geneva Laboratory of Virology University Hospitals of Geneva Laboratory of Virology Laubscher F.
EPI_ISL_429217 hCoV-19/Switzerland/GE6414/2020 Europe / Switzerland 2020-04-06 University Hospitals of Geneva Laboratory of Virology University Hospitals of Geneva Laboratory of Virology Laubscher F.
EPI_ISL_429218 hCoV-19/Switzerland/GE0304/2020 Europe / Switzerland 2020-04-02 University Hospitals of Geneva Laboratory of Virology University Hospitals of Geneva Laboratory of Virology Laubscher F.
EPI_ISL_429219 hCoV-19/Switzerland/GE2453/2020 Europe / Switzerland 2020-04-03 University Hospitals of Geneva Laboratory of Virology University Hospitals of Geneva Laboratory of Virology Laubscher F.
EPI_ISL_429220 hCoV-19/Switzerland/GE6065/2020 Europe / Switzerland 2020-04-06 University Hospitals of Geneva Laboratory of Virology University Hospitals of Geneva Laboratory of Virology Laubscher F.
EPI_ISL_429221 hCoV-19/Switzerland/GE6099/2020 Europe / Switzerland 2020-04-06 University Hospitals of Geneva Laboratory of Virology University Hospitals of Geneva Laboratory of Virology Laubscher F.
EPI_ISL_429222 hCoV-19/Switzerland/GE3655/2020 Europe / Switzerland 2020-04-07 University Hospitals of Geneva Laboratory of Virology University Hospitals of Geneva Laboratory of Virology Laubscher F.
EPI_ISL_429223 hCoV-19/Switzerland/GE4644/2020 Europe / Switzerland 2020-04-07 University Hospitals of Geneva Laboratory of Virology University Hospitals of Geneva Laboratory of Virology Laubscher F.
EPI_ISL_429226 hCoV-19/Italy/TE5551/2020 Europe / Italy / Abruzzo 2020-03-17 Presidio Ospedaliero Santo Spirito "Istituto Zooprofilattico Sperimentale dell'Abruzzo e Molise ""G. Caporale""" Lorusso A, Marcacci M, Di Domenico M, Ancora M, Curini V, Mangone I, Rinaldi A, Di Pasquale A, Camma C, Puglia I, Savini G

EPI_ISL_429227 hCoV-19/Italy/TE5553/2020 Europe / Italy / Abruzzo 2020-03-17 Presidio Ospedaliero Santo Spirito "Istituto Zooprofilattico Sperimentale dell'Abruzzo e Molise ""G. Caporale""" Lorusso A, Marcacci M, Di Domenico M, Ancora M, Curini V, Mangone I, Rinaldi A, Di Pasquale A, Camma C, Puglia I, Savini G
EPI_ISL_429228 hCoV-19/Italy/TE5674/2020 Europe / Italy / Abruzzo 2020-03-20 Ospedale Civile Giuseppe Mazzini "Istituto Zooprofilattico Sperimentale dell'Abruzzo e Molise ""G. Caporale""" Lorusso A, Marcacci M, Di Domenico M, Ancora M, Curini V, Mangone I, Rinaldi A, Di Pasquale A, Camma C, Puglia I, Savini G
EPI_ISL_430179 hCoV-19/USA/WA-S142/2020 North America / USA / Washington / King County 2020-03-13 Washington State Department of Health Seattle Flu Study Chu et al
EPI_ISL_430180 hCoV-19/USA/WA-S143/2020 North America / USA / Washington / King County 2020-03-15 Washington State Department of Health Seattle Flu Study Chu et al
EPI_ISL_430181 hCoV-19/USA/WA-S144/2020 North America / USA / Washington / King County 2020-03-15 Washington State Department of Health Seattle Flu Study Chu et al
EPI_ISL_430182 hCoV-19/USA/WA-S145/2020 North America / USA / Washington / King County 2020-03-15 Washington State Department of Health Seattle Flu Study Chu et al
EPI_ISL_430183 hCoV-19/USA/WA-S146/2020 North America / USA / Washington / King County 2020-03-14 Washington State Department of Health Seattle Flu Study Chu et al
EPI_ISL_430184 hCoV-19/USA/WA-S147/2020 North America / USA / Washington / King County 2020-03-14 Washington State Department of Health Seattle Flu Study Chu et al
EPI_ISL_430185 hCoV-19/USA/WA-S148/2020 North America / USA / Washington / King County 2020-03-13 Washington State Department of Health Seattle Flu Study Chu et al
EPI_ISL_430186 hCoV-19/USA/WA-S149/2020 North America / USA / Washington / King County 2020-03-14 Washington State Department of Health Seattle Flu Study Chu et al
EPI_ISL_430187 hCoV-19/USA/WA-S150/2020 North America / USA / Washington / King County 2020-03-14 Washington State Department of Health Seattle Flu Study Chu et al
EPI_ISL_430188 hCoV-19/USA/WA-S151/2020 North America / USA / Washington / Snohomish County 2020-03-16 Washington State Department of Health Seattle Flu Study Chu et al
EPI_ISL_430189 hCoV-19/USA/WA-S152/2020 North America / USA / Washington / King County 2020-03-16 Washington State Department of Health Seattle Flu Study Chu et al
EPI_ISL_430190 hCoV-19/USA/WA-S153/2020 North America / USA / Washington / King County 2020-03-16 Washington State Department of Health Seattle Flu Study Chu et al
EPI_ISL_430191 hCoV-19/USA/WA-S154/2020 North America / USA / Washington / King County 2020-03-16 Washington State Department of Health Seattle Flu Study Chu et al
EPI_ISL_430193 hCoV-19/USA/WA-S156/2020 North America / USA / Washington / King County 2020-03-16 Washington State Department of Health Seattle Flu Study Chu et al
EPI_ISL_430196 hCoV-19/USA/WA-S158/2020 North America / USA / Washington / Snohomish County 2020-03-16 Washington State Department of Health Seattle Flu Study Chu et al
EPI_ISL_430197 hCoV-19/USA/WA-S159/2020 North America / USA / Washington / Snohomish County 2020-03-16 Washington State Department of Health Seattle Flu Study Chu et al
EPI_ISL_430198 hCoV-19/USA/WA-S160/2020 North America / USA / Washington / King County 2020-03-17 Washington State Department of Health Seattle Flu Study Chu et al
EPI_ISL_430199 hCoV-19/USA/WA-S161/2020 North America / USA / Washington / King County 2020-03-17 Washington State Department of Health Seattle Flu Study Chu et al
EPI_ISL_430200 hCoV-19/USA/WA-S162/2020 North America / USA / Washington 2020-03-04 Washington State Department of Health Seattle Flu Study Chu et al
EPI_ISL_430201 hCoV-19/USA/WA-S163/2020 North America / USA / Washington 2020-03-04 Washington State Department of Health Seattle Flu Study Chu et al
EPI_ISL_430202 hCoV-19/USA/WA-S164/2020 North America / USA / Washington 2020-03-02 Washington State Department of Health Seattle Flu Study Chu et al
EPI_ISL_430204 hCoV-19/USA/WA-S166/2020 North America / USA / Washington / King County 2020-03-18 Washington State Department of Health Seattle Flu Study Chu et al
EPI_ISL_430205 hCoV-19/USA/WA-S167/2020 North America / USA / Washington / Whatcom County 2020-03-18 Washington State Department of Health Seattle Flu Study Chu et al
EPI_ISL_430206 hCoV-19/USA/WA-S168/2020 North America / USA / Washington / Whatcom County 2020-03-18 Washington State Department of Health Seattle Flu Study Chu et al
EPI_ISL_430207 hCoV-19/USA/WA-S169/2020 North America / USA / Washington / Whatcom County 2020-03-18 Washington State Department of Health Seattle Flu Study Chu et al
EPI_ISL_430208 hCoV-19/USA/WA-S170/2020 North America / USA / Washington / Yakima County 2020-03-17 Washington State Department of Health Seattle Flu Study Chu et al
EPI_ISL_430209 hCoV-19/USA/WA-S171/2020 North America / USA / Washington 2020-03-19 Washington State Department of Health Seattle Flu Study Chu et al
EPI_ISL_430210 hCoV-19/USA/WA-S172/2020 North America / USA / Washington / King County 2020-03-19 Washington State Department of Health Seattle Flu Study Chu et al
EPI_ISL_430211 hCoV-19/USA/WA-S173/2020 North America / USA / Washington 2020-03-19 Washington State Department of Health Seattle Flu Study Chu et al
EPI_ISL_404227 hCoV-19/Zhejiang/WZ-01/2020 Asia / China / Zhejiang 2020-01-16 Zhejiang Provincial Center for Disease Control and Prevention Department of Microbiology, Zhejiang Provincial Center for Disease Control and Prevention Yin Chen, Yanjun Zhang, Haiyan Mao, Junhang Pan, Xiuyu Lou, Yiyu Lu, Juying Yan, Hanping Zhu, Jian Gao, Yan Feng, Yi Sun, Hao Yan, Zhen Li, Yisheng Sun, Liming Gong, Qiong Ge, Wen Shi,

Xinying Wang, Wenwu Yao, Zhangnv Yang, Fang Xu, Chen Chen, Enfu Chen, Zhen Wang, Zhiping Chen, Jianmin Jiang, Chonggao Hu
EPI_ISL_404228 hCoV-19/Zhejiang/WZ-02/2020 Asia / China / Zhejiang 2020-01-17 Zhejiang Provincial Center for Disease Control and Prevention Department of Microbiology, Zhejiang Provincial Center for Disease Control and Prevention Yanjun Zhang, Yin Chen, Haiyan Mao, Junhang Pan, Xiuyu Lou, Yiyu Lu, Juying Yan, Hanping Zhu, Jian Gao, Yan Feng, Yi Sun, Hao Yan, Zhen Li, Yisheng Sun, Liming Gong, Qiong Ge, Wen Shi, Xinying Wang, Wenwu Yao, Zhangnv Yang, Fang Xu, Chen Chen, Enfu Chen, Zhen Wang, Zhiping Chen, Jianmin Jiang, Chonggao Hu
EPI_ISL_404253 hCoV-19/USA/IL1/2020 North America / USA / Illinois / Chicago 2020-01-21 IL Department of Public Health Chicago Laboratory Pathogen Discovery, Respiratory Viruses Branch, Division of Viral Diseases, Centers for Dieases Control and Prevention Ying Tao, Krista Queen, Clinton R. Paden, Jing Zhang, Yan Li, Anna Uehara, Xiaoyan Lu, Brian Lynch, Senthil Kumar K. Sakthivel, Brett L. Whitaker, Shifaq Kamili, Lijuan Wang, Janna' R. Murray, Susan I. Gerber, Stephen Lindstrom, Suxiang Tong
EPI_ISL_406800 hCoV-19/Wuhan/WH03/2020 Asia / China / Hubei / Wuhan 2020-01-01 General Hospital of Central Theater Command of People's Liberation Army of China BGI & Institute of Microbiology, Chinese Academy of Sciences & Shandong First Medical University & Shandong Academy of Medical Sciences & General Hospital of Central Theater Command of People's Liberation Army of China Weijun Chen, Yuhai Bi, Weifeng Shi and Zhenhong Hu
EPI_ISL_406801 hCoV-19/Wuhan/WH04/2020 Asia / China / Hubei / Wuhan 2020-01-05 General Hospital of Central Theater Command of People's Liberation Army of China BGI & Institute of Microbiology, Chinese Academy of Sciences & Shandong First Medical University & Shandong Academy of Medical Sciences & General Hospital of Central Theater Command of People's Liberation Army of China Weijun Chen, Yuhai Bi, Weifeng Shi and Zhenhong Hu
EPI_ISL_408976 hCoV-19/Australia/NSW02/2020 Oceania / Australia / New South Wales / Sydney 2020-01-22 Centre for Infectious Diseases and Microbiology Laboratory Services NSW Health Pathology - Institute of Clinical Pathology and Medical Research; Westmead Hospital; University of Sydney Rockett R, Sadsad R, Eden J-S, Carter I, Rahman H, Holmes EC, O’Sullivan MV, Sintchenko V, Chen SC, Maddocks S, Kok J and Dwyer DE for the 2019-nCoV Study Group*
EPI_ISL_408977 hCoV-19/Australia/NSW03/2020 Oceania / Australia / New South Wales / Sydney 2020-01-25 Serology, Virology and OTDS Laboratories (SAViD), NSW Health Pathology Randwick NSW Health Pathology - Institute of Clinical Pathology and Medical Research; Centre for Infectious Diseases and Microbiology Laboratory Services; Westmead Hospital; University of Sydney Eden J-S, Carter I, Rahman H, Rawlinson W, Holmes EC, Rockett R, O’Sullivan MV, Sintchenko V, Chen SC, Maddocks S, Kok J and Dwyer DE for the 2019-nCoV Study Group*
EPI_ISL_412116 hCoV-19/England/09c/2020 Europe / United Kingdom / England 2020-02-09 Respiratory Virus Unit, Microbiology Services Colindale, Public Health England Respiratory Virus Unit, Microbiology Services Colindale, Public Health England Monica Galiano, Shahjahan Miah, Angie Lackenby, Omolola Akinbami, Tiina Talts, Leena Bhaw, Richard Myers, Steven Platt, Kirstin Edwards, Jonathan Hubb, Joanna Ellis, Maria Zambon
EPI_ISL_413455 hCoV-19/USA/WA4-UW2/2020 North America / USA / Washington 2020-02-28 Washington State Public Health Lab University of Washington Virology Lab Pavitra Roychoudhury, Arun Nalla, Hong Xie, Keith Jerome, Alexander Greninger
EPI_ISL_413456 hCoV-19/USA/WA-S2/2020 North America / USA / Washington / King County 2020-02-20 Seattle Flu Study Seattle Flu Study Chu et al
EPI_ISL_413457 hCoV-19/USA/WA6-UW3/2020 North America / USA / Washington 2020-02-29 Washington State Public Health Lab UW Virology Lab Pavitra Roychoudhury, Arun Nalla, Hong Xie, Keith Jerome, Alexander Greninger
EPI_ISL_413458 hCoV-19/USA/WA7-UW4/2020 North America / USA / Washington 2020-03-01 Washington State Public Health Lab UW Virology Lab Pavitra Roychoudhury, Arun Nalla, Hong Xie, Keith Jerome, Alexander Greninger
EPI_ISL_413459 hCoV-19/Japan/TK-20-31-3/2020 Asia / Japan / Tokyo 2020-02-20 Department of Pathology, Toshima Hospital Pathogen Genomics Center, National Institute of Infectious Diseases Tsuyoshi Sekizuka, Kentaro Itokawa, Takuya Adachi, Masahiro Sano, Jun Yamazaki, Ippei Miyamoto, Haruka Nishioka, Ja-Mun Chong, Noriko Nakajima, Yuko Sato, Minoru Tobiume, Harutaka Katano, Tadaki Suzuki, Makoto Kuroda
EPI_ISL_413486 hCoV-19/USA/WA8-UW5/2020 North America / USA / Washington 2020-03-01 Valley Medical Center University of Washington Virology Lab Pavitra Roychoudhury, Arun Nalla, Hong Xie, Keith Jerome, Alexander Greninger
EPI_ISL_413487 hCoV-19/USA/WA9-UW6/2020 North America / USA / Washington 2020-03-01 Harborview Medical Center University of Washington Virology Lab Pavitra Roychoudhury, Arun Nalla, Hong Xie, Keith Jerome, Alexander Greninger
EPI_ISL_413488 hCoV-19/Germany/NRW-01/2020 Europe / Germany / North Rhine Westphalia / Heinsberg District 2020-02-28 Center of Medical Microbiology, Virology, and Hospital Hygiene, University of Duesseldorf Center of Medical Microbiology, Virology, and Hospital Hygiene, University of Duesseldorf Ortwin Adams, Marcel Andree, Alexander Dilthey, Torsten Feldt, Sandra Hauka, Torsten Houwaart, Björn-Erik Jensen, Detlef Kindgen-Milles, Malte Kohns Vasconcelos, Klaus Pfeffer, Tina Senff, Daniel Strelow, Jörg Timm, Andreas Walker, Tobias Wienemann
EPI_ISL_413489 hCoV-19/Italy/UniSR1/2020 Europe / Italy / Lombardy / Milan 2020-03-03 Laboratorio di Microbiologia e Virologia, Università Vita-Salute San Raffaele, Milano Laboratorio di Microbiologia e Virologia, Università Vita-Salute San Raffaele, Milano R.A Diotti, E. Criscuolo, M. Castelli,

V. Caputo, R. Ferrarese, M. Sampaolo, E. Boeri, I. Negri, V. Amato, G. Lo Raso, C. Di Resta, R. Burioni, M. Clementi, N. Mancini & N. Clementi
EPI_ISL_413490 hCoV-19/New Zealand/01/2020 Oceania / New Zealand / Auckland 2020-02-27 Auckland Hospital Institute of Environmental Science and Research (ESR) Matt Storey, Xiaoyun Ren, Gary McAuliffe, Sally Roberts, Matthew Blakiston, Erasmus Smit, Lauren Jelly, Joep de Ligt
EPI_ISL_414616 hCoV-19/USA/WA-UW29/2020 North America / USA / Washington / Kirkland 2020-03-08 UW Virology Lab UW Virology Lab Pavitra Roychoudhury, Hong Xie, Keith Jerome, Alexander Greninger
EPI_ISL_414617 hCoV-19/USA/WA-UW30/2020 North America / USA 2020-03-08 UW Virology Lab UW Virology Lab Pavitra Roychoudhury, Hong Xie, Keith Jerome, Alexander Greninger
EPI_ISL_414618 hCoV-19/USA/WA-UW31/2020 North America / USA 2020-03-08 UW Virology Lab UW Virology Lab Pavitra Roychoudhury, Hong Xie, Keith Jerome, Alexander Greninger
EPI_ISL_414619 hCoV-19/USA/WA-UW32/2020 North America / USA 2020-03-07 UW Virology Lab UW Virology Lab Pavitra Roychoudhury, Hong Xie, Keith Jerome, Alexander Greninger
EPI_ISL_414621 hCoV-19/USA/WA-UW34/2020 North America / USA / Washington 2020-03-07 UW Virology Lab UW Virology Lab Pavitra Roychoudhury, Hong Xie, Keith Jerome, Alexander Greninger
EPI_ISL_414622 hCoV-19/USA/WA-UW35/2020 North America / USA / Washington / Seattle 2020-03-08 UW Virology Lab UW Virology Lab Pavitra Roychoudhury, Hong Xie, Keith Jerome, Alexander Greninger
EPI_ISL_414623 hCoV-19/France/GE1583/2020 Europe / France / Grand-Est / Strasbourg 2020-02-25 Laboratoire de Virologie Institut de Virologie - INSERM U 1109 Hôpitaux Universitaires de Strasbourg National Reference Center for Viruses of Respiratory Infections, Institut Pasteur, Paris Mélnie Albert, Marion Barbet, Sylvie Behillil, Méline Bizard, Angela Brisebarre, Flora Donati Vincent Enouf, Maud Vanpeene, Sylvie van der Werf, Samira Fafi-Kremer
EPI_ISL_414624 hCoV-19/France/N1620/2020 Europe / France / Normandie / Rouen 2020-02-26 Centre Hositalier Universitaire de Rouen Laboratoire de Virologie National Reference Center for Viruses of Respiratory Infections, Institut Pasteur, Paris Mélnie Albert, Marion Barbet, Sylvie Behillil, Méline Bizard, Angela Brisebarre, Flora Donati Vincent Enouf, Maud Vanpeene, Sylvie van der Werf, Jean-Christophe Plantier
EPI_ISL_414636 hCoV-19/France/HF1989/2020 Europe / France / Hauts de France / Compiègne 2020-03-04 Centre Hospitalier Compiègne Laboratoire de Biologie National Reference Center for Viruses of Respiratory Infections, Institut Pasteur, Paris Mélnie Albert, Marion Barbet, Sylvie Behillil, Méline Bizard, Angela Brisebarre, Flora Donati Vincent Enouf, Maud Vanpeene, Sylvie van der Werf, Raulin Olivia
EPI_ISL_414637 hCoV-19/France/HF1993/2020 Europe / France / Hauts de France / Compiègne 2020-03-04 Centre Hospitalier Compiègne Laboratoire de Biologie National Reference Center for Viruses of Respiratory Infections, Institut Pasteur, Paris Mélnie Albert, Marion Barbet, Sylvie Behillil, Méline Bizard, Angela Brisebarre, Flora Donati Vincent Enouf, Maud Vanpeene, Sylvie van der Werf, Raulin Olivia
EPI_ISL_414638 hCoV-19/France/HF1995/2020 Europe / France / Hauts de France / Compiègne 2020-03-04 Centre Hospitalier Compiègne Laboratoire de Biologie National Reference Center for Viruses of Respiratory Infections, Institut Pasteur, Paris Mélnie Albert, Marion Barbet, Sylvie Behillil, Méline Bizard, Angela Brisebarre, Flora Donati Vincent Enouf, Maud Vanpeene, Sylvie van der Werf, Raulin Olivia
EPI_ISL_414641 hCoV-19/Finland/FIN-313/2020 Europe / Finland 2020-03-05 Department of Virology and Immunology, University of Helsinki and Helsinki University Hospital, Huslab Finland Department of Virology, Faculty of Medicine, University of Helsinki, Helsinki, Finland Teemu Smura, Hannimari Kallio-Kokko, Olli Vapalahti
EPI_ISL_414642 hCoV-19/Finland/FIN-455/2020 Europe / Finland 2020-03-08 Department of Virology and Immunology, University of Helsinki and Helsinki University Hospital, Huslab Finland Department of Virology, Faculty of Medicine, University of Helsinki, Helsinki, Finland Teemu Smura, Hannimari Kallio-Kokko, Olli Vapalahti
EPI_ISL_414643 hCoV-19/Finland/FIN-508/2020 Europe / Finland 2020-03-07 Department of Virology and Immunology, University of Helsinki and Helsinki University Hospital, Huslab Finland Department of Virology, Faculty of Medicine, University of Helsinki, Helsinki, Finland Teemu Smura, Hannimari Kallio-Kokko, Olli Vapalahti
EPI_ISL_414646 hCoV-19/Finland/FIN-266/2020 Europe / Finland 2020-03-04 Department of Virology and Immunology, University of Helsinki and Helsinki University Hospital, Huslab Finland Department of Virology, Faculty of Medicine, University of Helsinki, Helsinki, Finland Teemu Smura, Hannimari Kallio-Kokko, Olli Vapalahti
EPI_ISL_414648 hCoV-19/USA/CA-PC101P/2020 North America / USA / California / San Diego County 2020-03-11 Andersen Lab, The Scripps Research Institute Andersen Lab, The Scripps Research Institute Mark Zeller, Catie Anderson, Emily Spender, Sarah Topol, Raphaelle Klitting, Refugio Robles-Sikisaka, Karthik Gangavarapu, Laura Nicholson, Kristian Andersen
EPI_ISL_417004 hCoV-19/Belgium/ULG-3000/2020 Europe / Belgium / Liège 2020-03-05 Department of Clinical Microbiology GIGA Medical Genomics Durkin Keith, Artesi Maria, Bontems Sébastien, Boreux Raphaël, Meex Cécile, Melin Pierrette, Hayette Marie-Pierre, Bours Vincent.
EPI_ISL_417006 hCoV-19/Belgium/ULG-3163/2020 Europe / Belgium / Liège 2020-03-05 Department of Clinical Microbiology GIGA Medical Genomics Durkin Keith, Artesi Maria, Bontems Sébastien, Boreux Raphaël, Meex Cécile, Melin Pierrette, Hayette Marie-Pierre, Bours Vincent.
EPI_ISL_417007 hCoV-19/Spain/Galicia201663/2020 Europe / Spain / Galicia 2020-03-07 HOSPITAL SANTA MARIA NAI Instituto de Salud Carlos III Iglesias-Caballero, M. Molinero Calamita, M. González-Esguevillas, M. Camarero S. Pozo F. Casas I. Jiménez, P. Jiménez, M. Zaballos, A. Monzón, S. Varona, S. Juliá, M. Cuesta, I. García Costa, J.
EPI_ISL_417008 hCoV-19/Belgium/ULG-3662/2020 Europe / Belgium / Liège 2020-03-07 Department of Clinical Microbiology GIGA Medical Genomics Durkin Keith, Artesi Maria, Bontems Sébastien, Boreux Raphaël,

Meex Cécile, Melin Pierrette, Hayette Marie-Pierre, Bours Vincent.
EPI_ISL_417009 hCoV-19/Belgium/ULG-3665/2020 Europe / Belgium / Liège 2020-03-07 Department of Clinical Microbiology GIGA Medical Genomics Durkin Keith, Artesi Maria, Bontems Sébastien, Boreux Raphaël, Meex Cécile, Melin Pierrette, Hayette Marie-Pierre, Bours Vincent.
EPI_ISL_417010 hCoV-19/Spain/Madrid201442/2020 Europe / Spain / Madrid 2020-03-04 FUNDACION JIMENEZ DIAZ Instituto de Salud Carlos III Iglesias-Caballero, M. Molinero Calamita, M. González-Esguevillas, M. Camarero, S. Pozo, F. Casas, I. Jiménez, P. Jiménez, M. Zaballos, A. Monzón, S. Varona, S. Juliá, M. Cuesta, I. Fernández Roblas, R.
EPI_ISL_417012 hCoV-19/Belgium/ULG-3843/2020 Europe / Belgium / Liège 2020-03-08 Department of Clinical Microbiology GIGA Medical Genomics Durkin Keith, Artesi Maria, Bontems Sébastien, Boreux Raphaël, Meex Cécile, Melin Pierrette, Hayette Marie-Pierre, Bours Vincent.
EPI_ISL_417013 hCoV-19/Belgium/ULG-4163/2020 Europe / Belgium / Liège 2020-03-09 Department of Clinical Microbiology GIGA Medical Genomics Durkin Keith, Artesi Maria, Bontems Sébastien, Boreux Raphaël, Meex Cécile, Melin Pierrette, Hayette Marie-Pierre, Bours Vincent.
EPI_ISL_417014 hCoV-19/Belgium/ULG-6216/2020 Europe / Belgium / Liège 2020-03-13 Department of Clinical Microbiology GIGA Medical Genomics Durkin Keith, Artesi Maria, Bontems Sébastien, Boreux Raphaël, Meex Cécile, Melin Pierrette, Hayette Marie-Pierre, Bours Vincent.
EPI_ISL_417015 hCoV-19/Belgium/ULG-6457/2020 Europe / Belgium / Liège 2020-03-13 Department of Clinical Microbiology GIGA Medical Genomics Durkin Keith, Artesi Maria, Bontems Sébastien, Boreux Raphaël, Meex Cécile, Melin Pierrette, Hayette Marie-Pierre, Bours Vincent.
EPI_ISL_417016 hCoV-19/Belgium/ULG-6503/2020 Europe / Belgium / Liège 2020-03-13 Department of Clinical Microbiology GIGA Medical Genomics Durkin Keith, Artesi Maria, Bontems Sébastien, Boreux Raphaël, Meex Cécile, Melin Pierrette, Hayette Marie-Pierre, Bours Vincent.
EPI_ISL_417017 hCoV-19/Belgium/ULG-6638/2020 Europe / Belgium / Liège 2020-03-14 Department of Clinical Microbiology GIGA Medical Genomics Durkin Keith, Artesi Maria, Bontems Sébastien, Boreux Raphaël, Meex Cécile, Melin Pierrette, Hayette Marie-Pierre, Bours Vincent.
EPI_ISL_417018 hCoV-19/Belgium/ULG-6670/2020 Europe / Belgium / Liège 2020-03-14 Department of Clinical Microbiology GIGA Medical Genomics Durkin Keith, Artesi Maria, Bontems Sébastien, Boreux Raphaël, Meex Cécile, Melin Pierrette, Hayette Marie-Pierre, Bours Vincent.
EPI_ISL_417019 hCoV-19/Belgium/ULG-6754/2020 Europe / Belgium / Liege 2020-03-14 Department of Clinical Microbiology GIGA Medical Genomics Durkin Keith, Artesi Maria, Bontems Sébastien, Boreux Raphaël, Meex Cécile, Melin Pierrette, Hayette Marie-Pierre, Bours Vincent.
EPI_ISL_417020 hCoV-19/Belgium/ULG-6939/2020 Europe / Belgium / Liège 2020-03-15 Department of Clinical Microbiology GIGA Medical Genomics Durkin Keith, Artesi Maria, Bontems Sébastien, Boreux Raphaël, Meex Cécile, Melin Pierrette, Hayette Marie-Pierre, Bours Vincent.
EPI_ISL_417021 hCoV-19/Belgium/ULG-6942/2020 Europe / Belgium / Liège 2020-03-15 Department of Clinical Microbiology GIGA Medical Genomics Durkin Keith, Artesi Maria, Bontems Sébastien, Boreux Raphaël, Meex Cécile, Melin Pierrette, Hayette Marie-Pierre, Bours Vincent.
EPI_ISL_417022 hCoV-19/Belgium/ULG-6948/2020 Europe / Belgium / Liège 2020-03-15 Department of Clinical Microbiology GIGA Medical Genomics Durkin Keith, Artesi Maria, Bontems Sébastien, Boreux Raphaël, Meex Cécile, Melin Pierrette, Hayette Marie-Pierre, Bours Vincent.
EPI_ISL_417023 hCoV-19/Belgium/ULG-6950/2020 Europe / Belgium / Liège 2020-03-15 Department of Clinical Microbiology GIGA Medical Genomics Durkin Keith, Artesi Maria, Bontems Sébastien, Boreux Raphaël, Meex Cécile, Melin Pierrette, Hayette Marie-Pierre, Bours Vincent.
EPI_ISL_417025 hCoV-19/Belgium/ULG-7019/2020 Europe / Belgium / Liège 2020-03-15 Department of Clinical Microbiology GIGA Medical Genomics Durkin Keith, Artesi Maria, Bontems Sébastien, Boreux Raphaël, Meex Cécile, Melin Pierrette, Hayette Marie-Pierre, Bours Vincent.
EPI_ISL_417026 hCoV-19/USA/UT-08/2020 North America / USA / Utah 2020-03-20 Utah Public Health Laboratory Utah Public Health Laboratory Erin Young, Kelly Oakeson
EPI_ISL_417028 hCoV-19/USA/UT-020/2020 North America / USA / Utah 2020-03-20 Utah Public Health Laboratory Utah Public Health Laboratory Erin Young, Kelly Oakeson
EPI_ISL_417030 hCoV-19/Australia/NSW04/2020 Oceania / Australia / New South Wales / Sydney 2020-01-24 Centre for Infectious Diseases and Microbiology Laboratory Services NSW Health Pathology - Institute of Clinical Pathology and Medical Research; Westmead Hospital; University of Sydney Eden J-S, Rockett R, Carter I, Rahman H, Holmes EC, O'Sullivan MV, Sintchenko V, Chen SC, Maddocks S, Kok J and Dwyer DE for the 2019-nCoV Study Group*
EPI_ISL_417031 hCoV-19/Australia/QLDID919/2020 Oceania / Australia / Queensland / Gold Coast 2020-03-11 Pathology Queensland Public Health Virology Laboratory Bixing Huang, Alyssa Pyke, Amanda De Jong, Andrew Van Den Hurk, Carmel Taylor, David Warrilow, Doris Genge, Elisabeth Gamez, Glen Hewitson, Ian Maxwell Mackay, Inga Sultana, Jamie McMahon, Jean Barcelon, Judy Northill, Mitchell Finger, Natalie Simpson, Neelima Nair, Peter Burtonclay, Peter Moore, Sarah Wheatley, Sean Moody, Sonja Hall-Mendelin, Timothy Gardam, and Frederick Moore
EPI_ISL_417032 hCoV-19/Australia/QLDID920/2020 Oceania / Australia / Queensland / Rockhampton 2020-03-11 Rockhampton Base Hospital Public Health Virology Laboratory Bixing Huang, Alyssa Pyke, Amanda De Jong, Andrew Van Den Hurk, Carmel Taylor, David Warrilow, Doris Genge, Elisabeth Gamez, Glen Hewitson, Ian Maxwell Mackay, Inga Sultana, Jamie McMahon, Jean Barcelon, Judy Northill, Mitchell Finger, Natalie Simpson, Neelima Nair, Peter Burtonclay, Peter Moore, Sarah Wheatley, Sean Moody, Sonja Hall-Mendelin, Timothy Gardam, and Frederick Moore
EPI_ISL_417033 hCoV-19/Australia/QLDID921/2020 Oceania / Australia / Queensland / Brisbane 2020-03-11 Sullivan Nicolaides Pathology Public Health Virology Laboratory Bixing Huang, Alyssa Pyke, Amanda De

Jong, Andrew Van Den Hurk, Carmel Taylor, David Warrilow, Doris Genge, Elisabeth Gamez, Glen Hewitson, Ian Maxwell Mackay, Inga Sultana, Jamie McMahon, Jean Barcelon, Judy Northill, Mitchell Finger, Natalie Simpson, Neelima Nair, Peter Burtonclay, Peter Moore, Sarah Wheatley, Sean Moody, Sonja Hall-Mendelin, Timothy Gardam, and Frederick Moore
EPI_ISL_417034 hCoV-19/Brazil/AMBR-02/2020 South America / Brazil / Amazonas State / Manaus 2020-03-16 Laboratorio de Ecologia de Doencas Transmissiveis na Amazonia, Instituto Leonidas e Maria Deane - Fiocruz Amazonia Laboratorio de Ecologia de Doencas Transmissiveis na Amazonia, Instituto Leonidas e Maria Deane - Fiocruz Amazonia Valdinete Nascimento, André Corado, Fernanda Nascimento, Ágatha Costa, Debora Duarte, Luciana Gonçalves, Michele Jesus, Sérgio Luz, Felipe Naveca
EPI_ISL_417064 hCoV-19/Hong Kong/VB20017970-2/2020 Asia / Hong Kong 2020-01-21 Prince of Wales Hospital Hong Kong Department of Health Alan K.L. Tsang, Peter C.W. Yip, Edman T.K. Lam, Rickjason C.W. Chan, Dominic N.C. Tsang
EPI_ISL_417065 hCoV-19/USA/WA-S12/2020 North America / USA / Washington 2020-03-03 Washington State Department of Health Seattle Flu Study Chu etl al
EPI_ISL_417066 hCoV-19/USA/WA-S13/2020 North America / USA / Washington / King County 2020-03-03 Washington State Department of Health Seattle Flu Study Chu etl al
EPI_ISL_417068 hCoV-19/USA/WA-S15/2020 North America / USA / Washington / King County 2020-03-02 Washington State Department of Health Seattle Flu Study Chu etl al
EPI_ISL_417069 hCoV-19/USA/WA-S16/2020 North America / USA / Washington 2020-03-03 Washington State Department of Health Seattle Flu Study Chu etl al
EPI_ISL_417070 hCoV-19/USA/WA-S17/2020 North America / USA / Washington / King County 2020-03-03 Washington State Department of Health Seattle Flu Study Chu etl al
EPI_ISL_417071 hCoV-19/USA/WA-S18/2020 North America / USA / Washington / King County 2020-03-03 Washington State Department of Health Seattle Flu Study Chu etl al
EPI_ISL_417072 hCoV-19/USA/WA-S19/2020 North America / USA / Washington / King County 2020-03-02 Washington State Department of Health Seattle Flu Study Chu etl al
EPI_ISL_417073 hCoV-19/USA/WA-S20/2020 North America / USA / Washington 2020-03-02 Washington State Department of Health Seattle Flu Study Chu etl al
EPI_ISL_417074 hCoV-19/USA/WA-S21/2020 North America / USA / Washington / King County 2020-03-02 Washington State Department of Health Seattle Flu Study Chu etl al
EPI_ISL_417075 hCoV-19/USA/WA-S22/2020 North America / USA / Washington / Snohomish County 2020-03-02 Washington State Department of Health Seattle Flu Study Chu etl al
EPI_ISL_417076 hCoV-19/USA/WA-S23/2020 North America / USA / Washington / Snohomish County 2020-03-02 Washington State Department of Health Seattle Flu Study Chu etl al
EPI_ISL_417077 hCoV-19/USA/WA-S24/2020 North America / USA / Washington / Snohomish County 2020-03-02 Washington State Department of Health Seattle Flu Study Chu etl al
EPI_ISL_417079 hCoV-19/USA/WA-S26/2020 North America / USA / Washington / Snohomish County 2020-03-02 Washington State Department of Health Seattle Flu Study Chu etl al
EPI_ISL_417081 hCoV-19/USA/WA-S28/2020 North America / USA / Washington / Snohomish County 2020-03-02 Washington State Department of Health Seattle Flu Study Chu etl al
EPI_ISL_417082 hCoV-19/USA/WA-S29/2020 North America / USA / Washington / Grant County 2020-03-02 Washington State Department of Health Seattle Flu Study Chu etl al
EPI_ISL_417085 hCoV-19/USA/WA-S32/2020 North America / USA / Washington / Snohomish County 2020-03-02 Washington State Department of Health Seattle Flu Study Chu etl al
EPI_ISL_417086 hCoV-19/USA/WA-S33/2020 North America / USA / Washington / Clark County 2020-03-01 Washington State Department of Health Seattle Flu Study Chu etl al
EPI_ISL_417087 hCoV-19/USA/WA-S34/2020 North America / USA / Washington 2020-03-02 Washington State Department of Health Seattle Flu Study Chu etl al
EPI_ISL_417088 hCoV-19/USA/WA-S35/2020 North America / USA / Washington 2020-03-02 Washington State Department of Health Seattle Flu Study Chu etl al
EPI_ISL_417089 hCoV-19/USA/WA-S36/2020 North America / USA / Washington 2020-03-02 Washington State Department of Health Seattle Flu Study Chu etl al
EPI_ISL_417500 hCoV-19/USA/MN56-MDH56/2020 North America / USA / Minnesota 2020-03-14 Minnesota Department of Health, Public Health Laboratory Minnesota Department of Health, Public Health Laboratory Matt Plumb, Jake Garfin and Xiong Wang
EPI_ISL_417501 hCoV-19/USA/MN57-MDH57/2020 North America / USA / Minnesota 2020-03-14 Minnesota Department of Health, Public Health Laboratory Minnesota Department of Health, Public Health Laboratory Matt Plumb, Jake Garfin and Xiong Wang
EPI_ISL_417502 hCoV-19/USA/MN58-MDH58/2020 North America / USA / Minnesota 2020-03-14 Minnesota Department of Health, Public Health Laboratory Minnesota Department of Health, Public Health Laboratory Matt Plumb, Jake Garfin and Xiong Wang
EPI_ISL_417503 hCoV-19/USA/MN59-MDH59/2020 North America / USA / Minnesota 2020-03-14 Minnesota Department of Health, Public Health Laboratory Minnesota Department of Health, Public Health Laboratory Matt Plumb, Jake Garfin and Xiong Wang
EPI_ISL_417504 hCoV-19/USA/WI-UW-15/2020 North America / USA / Wisconsin 2020-03-16 University of Wisconsin-Madison AIDS Vaccine Research Laboratories University of Wisconsin-Madison AIDS Vaccine Research Laboratories Gage Moreno, Katarina Braun, et al. AIDS Vaccine Research Laboratories
EPI_ISL_417505 hCoV-19/USA/WI-UW-11/2020 North America / USA / Wisconsin 2020-03-15 University of Wisconsin-Madison AIDS Vaccine Research Laboratories University of Wisconsin-Madison AIDS Vaccine Research Laboratories Gage Moreno, Katarina Braun, et al. AIDS Vaccine Research Laboratories

EPI_ISL_417506 hCoV-19/USA/WI-UW-12/2020 North America / USA / Wisconsin 2020-03-16 University of Wisconsin-Madison AIDS Vaccine Research Laboratories University of Wisconsin-Madison AIDS Vaccine Research Laboratories Gage Moreno, Katarina Braun, et al. AIDS Vaccine Research Laboratories
EPI_ISL_417529 hCoV-19/Luxembourg/LNS5731562/2020 Europe / Luxembourg 2020-03-18 Laboratoire Nationale de Santé, Microbiology, Virology Laboratoire Nationale de Santé, Microbiology, Epidemiology and Microbial Genomics Anke Wienecke-Baldacchino, Ardashel Latsuzbaia, Jessica Tapp, Catherine Ragimbeau, Guillaume Fournier, Tamir Abdelrahman, Trung Nguyen Nguyen, Joel Mossong
EPI_ISL_417530 hCoV-19/Luxembourg/LNS2128808/2020 Europe / Luxembourg 2020-03-18 Laboratoire Nationale de Santé, Microbiology, Virology Laboratoire Nationale de Santé, Microbiology, Epidemiology and Microbial Genomics Anke Wienecke-Baldacchino, Ardashel Latsuzbaia, Jessica Tapp, Catherine Ragimbeau, Guillaume Fournier, Tamir Abdelrahman, Trung Nguyen Nguyen, Joel Mossong
EPI_ISL_417531 hCoV-19/Luxembourg/LNS2013896/2020 Europe / Luxembourg 2020-03-18 Laboratoire Nationale de Santé, Microbiology, Virology Laboratoire Nationale de Santé, Microbiology, Epidemiology and Microbial Genomics Anke Wienecke-Baldacchino, Ardashel Latsuzbaia, Jessica Tapp, Catherine Ragimbeau, Guillaume Fournier, Tamir Abdelrahman, Trung Nguyen Nguyen, Joel Mossong
EPI_ISL_417532 hCoV-19/Luxembourg/LNS8489624/2020 Europe / Luxembourg 2020-03-18 Laboratoire Nationale de Santé, Microbiology, Virology Laboratoire Nationale de Santé, Microbiology, Epidemiology and Microbial Genomics Anke Wienecke-Baldacchino, Ardashel Latsuzbaia, Jessica Tapp, Catherine Ragimbeau, Guillaume Fournier, Tamir Abdelrahman, Trung Nguyen Nguyen, Joel Mossong
EPI_ISL_417533 hCoV-19/Luxembourg/LNS6282845/2020 Europe / Luxembourg 2020-03-18 Laboratoire Nationale de Santé, Microbiology, Virology Laboratoire Nationale de Santé, Microbiology, Epidemiology and Microbial Genomics Anke Wienecke-Baldacchino, Ardashel Latsuzbaia, Jessica Tapp, Catherine Ragimbeau, Guillaume Fournier, Tamir Abdelrahman, Trung Nguyen Nguyen, Joel Mossong
EPI_ISL_417534 hCoV-19/Luxembourg/LNS0158952/2020 Europe / Luxembourg 2020-03-18 Laboratoire Nationale de Santé, Microbiology, Virology Laboratoire Nationale de Santé, Microbiology, Epidemiology and Microbial Genomics Anke Wienecke-Baldacchino, Ardashel Latsuzbaia, Jessica Tapp, Catherine Ragimbeau, Guillaume Fournier, Tamir Abdelrahman, Trung Nguyen Nguyen, Joel Mossong
EPI_ISL_417535 hCoV-19/Iceland/1/2020 Europe / Iceland / Reykjavik 2020-03-13 deCODE genetics deCODE genetics Daniel F Gudbjartsson; Agnar Helgason; Hakon Jonsson; Olafur T Magnusson; Pall Melsted; Gudmundur L Norddahl; Jona Saemundsdottir; Asgeir Sigurdsson; Patrick Sulem; Arna B Agustsdottir; Berglind Eiriksdottir; Run Fridriksdottir; Elisabet E Gardarsdottir; Gudmundur Georgsson; Olafia S Gretarsdottir; Kjartan R Gudmundsson; Thora R Gunnarsdottir; Arnaldur Gylfason; Hilma Holm; Brynjar O Jensson; Aslaug Jonasdottir; Kamilla S Josefsdottir; Thordur Kristjansson; Droplaug N Magnusdottir; Louise le Roux; Gudrun Sigmundsdottir; Gardar Sveinbjornsson; Kristin E Sveinsdottir; Maney Sveinsdottir; Emil A Thorarensen; Bjarni Thorbjornsson; Gisli Masson; Ingileif Jonsdottir; Alma Moller; Thorolfur Gudnason; Karl G Kristinsson; Unnur Thorsteinsdottir; Kari Stefansson
EPI_ISL_417536 hCoV-19/Iceland/10/2020 Europe / Iceland / Reykjavik 2020-03-16 deCODE genetics deCODE genetics Daniel F Gudbjartsson; Agnar Helgason; Hakon Jonsson; Olafur T Magnusson; Pall Melsted; Gudmundur L Norddahl; Jona Saemundsdottir; Asgeir Sigurdsson; Patrick Sulem; Arna B Agustsdottir; Berglind Eiriksdottir; Run Fridriksdottir; Elisabet E Gardarsdottir; Gudmundur Georgsson; Olafia S Gretarsdottir; Kjartan R Gudmundsson; Thora R Gunnarsdottir; Arnaldur Gylfason; Hilma Holm; Brynjar O Jensson; Aslaug Jonasdottir; Kamilla S Josefsdottir; Thordur Kristjansson; Droplaug N Magnusdottir; Louise le Roux; Gudrun Sigmundsdottir; Gardar Sveinbjornsson; Kristin E Sveinsdottir; Maney Sveinsdottir; Emil A Thorarensen; Bjarni Thorbjornsson; Gisli Masson; Ingileif Jonsdottir; Alma Moller; Thorolfur Gudnason; Karl G Kristinsson; Unnur Thorsteinsdottir; Kari Stefansson
EPI_ISL_417537 hCoV-19/Iceland/100/2020 Europe / Iceland / Reykjavik 2020-03-16 deCODE genetics deCODE genetics Daniel F Gudbjartsson; Agnar Helgason; Hakon Jonsson; Olafur T Magnusson; Pall Melsted; Gudmundur L Norddahl; Jona Saemundsdottir; Asgeir Sigurdsson; Patrick Sulem; Arna B Agustsdottir; Berglind Eiriksdottir; Run Fridriksdottir; Elisabet E Gardarsdottir; Gudmundur Georgsson; Olafia S Gretarsdottir; Kjartan R Gudmundsson; Thora R Gunnarsdottir; Arnaldur Gylfason; Hilma Holm; Brynjar O Jensson; Aslaug Jonasdottir; Kamilla S Josefsdottir; Thordur Kristjansson; Droplaug N Magnusdottir; Louise le Roux; Gudrun Sigmundsdottir; Gardar Sveinbjornsson; Kristin E Sveinsdottir; Maney Sveinsdottir; Emil A Thorarensen; Bjarni Thorbjornsson; Gisli Masson; Ingileif Jonsdottir; Alma Moller; Thorolfur Gudnason; Karl G Kristinsson; Unnur Thorsteinsdottir; Kari Stefansson
EPI_ISL_417538 hCoV-19/Iceland/104/2020 Europe / Iceland / Reykjavik 2020-03-17 deCODE genetics deCODE genetics Daniel F Gudbjartsson; Agnar Helgason; Hakon Jonsson; Olafur T Magnusson; Pall Melsted; Gudmundur L Norddahl; Jona Saemundsdottir; Asgeir Sigurdsson; Patrick Sulem; Arna B Agustsdottir; Berglind Eiriksdottir; Run Fridriksdottir; Elisabet E Gardarsdottir; Gudmundur Georgsson; Olafia S Gretarsdottir; Kjartan R Gudmundsson; Thora R Gunnarsdottir; Arnaldur Gylfason; Hilma Holm; Brynjar O Jensson; Aslaug Jonasdottir; Kamilla S Josefsdottir; Thordur Kristjansson; Droplaug N Magnusdottir; Louise le Roux; Gudrun Sigmundsdottir; Gardar Sveinbjornsson; Kristin E Sveinsdottir; Maney Sveinsdottir; Emil A Thorarensen; Bjarni Thorbjornsson; Gisli Masson; Ingileif Jonsdottir; Alma Moller; Thorolfur Gudnason; Karl G Kristinsson; Unnur Thorsteinsdottir; Kari Stefansson
EPI_ISL_417539 hCoV-19/Iceland/11/2020 Europe / Iceland / Reykjavik 2020-03-16 deCODE genetics deCODE genetics Daniel F Gudbjartsson; Agnar Helgason; Hakon Jonsson; Olafur T Magnusson; Pall Melsted; Gudmundur L Norddahl; Jona Saemundsdottir; Asgeir Sigurdsson; Patrick Sulem; Arna B Agustsdottir; Berglind Eiriksdottir; Run Fridriksdottir; Elisabet E Gardarsdottir; Gudmundur Georgsson; Olafia S Gretarsdottir; Kjartan R Gudmundsson; Thora R Gunnarsdottir; Arnaldur Gylfason; Hilma Holm; Brynjar O Jensson; Aslaug Jonasdottir; Kamilla S Josefsdottir; Thordur Kristjansson; Droplaug N Magnusdottir; Louise le Roux; Gudrun Sigmundsdottir; Gardar Sveinbjornsson; Kristin E Sveinsdottir; Maney Sveinsdottir; Emil A Thorarensen; Bjarni

Thorbjornsson; Gisli Masson; Ingileif Jonsdottir; Alma Moller; Thorolfur Gudnason; Karl G Kristinsson; Unnur Thorsteinsdottir; Kari Stefansson
EPI_ISL_417540 hCoV-19/Iceland/115/2020 Europe / Iceland / Reykjavik 2020-03-17 deCODE genetics deCODE genetics Daniel F Gudbjartsson; Agnar Helgason; Hakon Jonsson; Olafur T Magnusson; Pall Melsted; Gudmundur L Norddahl; Jona Saemundsdottir; Asgeir Sigurdsson; Patrick Sulem; Arna B Agustsdottir; Berglind Eiriksdottir; Run Fridriksdottir; Elisabet E Gardarsdottir; Gudmundur Georgsson; Olafia S Gretarsdottir; Kjartan R Gudmundsson; Thora R Gunnarsdottir; Arnaldur Gylfason; Hilma Holm; Brynjar O Jensson; Aslaug Jonasdottir; Kamilla S Josefsdottir; Thordur Kristjansson; Droplaug N Magnusdottir; Louise le Roux; Gudrun Sigmundsdottir; Gardar Sveinbjornsson; Kristin E Sveinsdottir; Maney Sveinsdottir; Emil A Thorarensen; Bjarni Thorbjornsson; Gisli Masson; Ingileif Jonsdottir; Alma Moller; Thorolfur Gudnason; Karl G Kristinsson; Unnur Thorsteinsdottir; Kari Stefansson
EPI_ISL_417541 hCoV-19/Iceland/116/2020 Europe / Iceland / Reykjavik 2020-03-17 deCODE genetics deCODE genetics Daniel F Gudbjartsson; Agnar Helgason; Hakon Jonsson; Olafur T Magnusson; Pall Melsted; Gudmundur L Norddahl; Jona Saemundsdottir; Asgeir Sigurdsson; Patrick Sulem; Arna B Agustsdottir; Berglind Eiriksdottir; Run Fridriksdottir; Elisabet E Gardarsdottir; Gudmundur Georgsson; Olafia S Gretarsdottir; Kjartan R Gudmundsson; Thora R Gunnarsdottir; Arnaldur Gylfason; Hilma Holm; Brynjar O Jensson; Aslaug Jonasdottir; Kamilla S Josefsdottir; Thordur Kristjansson; Droplaug N Magnusdottir; Louise le Roux; Gudrun Sigmundsdottir; Gardar Sveinbjornsson; Kristin E Sveinsdottir; Maney Sveinsdottir; Emil A Thorarensen; Bjarni Thorbjornsson; Gisli Masson; Ingileif Jonsdottir; Alma Moller; Thorolfur Gudnason; Karl G Kristinsson; Unnur Thorsteinsdottir; Kari Stefansson
EPI_ISL_417542 hCoV-19/Iceland/117/2020 Europe / Iceland / Reykjavik 2020-03-17 deCODE genetics deCODE genetics Daniel F Gudbjartsson; Agnar Helgason; Hakon Jonsson; Olafur T Magnusson; Pall Melsted; Gudmundur L Norddahl; Jona Saemundsdottir; Asgeir Sigurdsson; Patrick Sulem; Arna B Agustsdottir; Berglind Eiriksdottir; Run Fridriksdottir; Elisabet E Gardarsdottir; Gudmundur Georgsson; Olafia S Gretarsdottir; Kjartan R Gudmundsson; Thora R Gunnarsdottir; Arnaldur Gylfason; Hilma Holm; Brynjar O Jensson; Aslaug Jonasdottir; Kamilla S Josefsdottir; Thordur Kristjansson; Droplaug N Magnusdottir; Louise le Roux; Gudrun Sigmundsdottir; Gardar Sveinbjornsson; Kristin E Sveinsdottir; Maney Sveinsdottir; Emil A Thorarensen; Bjarni Thorbjornsson; Gisli Masson; Ingileif Jonsdottir; Alma Moller; Thorolfur Gudnason; Karl G Kristinsson; Unnur Thorsteinsdottir; Kari Stefansson
EPI_ISL_417543 hCoV-19/Iceland/118/2020 Europe / Iceland / Reykjavik 2020-03-17 deCODE genetics deCODE genetics Daniel F Gudbjartsson; Agnar Helgason; Hakon Jonsson; Olafur T Magnusson; Pall Melsted; Gudmundur L Norddahl; Jona Saemundsdottir; Asgeir Sigurdsson; Patrick Sulem; Arna B Agustsdottir; Berglind Eiriksdottir; Run Fridriksdottir; Elisabet E Gardarsdottir; Gudmundur Georgsson; Olafia S Gretarsdottir; Kjartan R Gudmundsson; Thora R Gunnarsdottir; Arnaldur Gylfason; Hilma Holm; Brynjar O Jensson; Aslaug Jonasdottir; Kamilla S Josefsdottir; Thordur Kristjansson; Droplaug N Magnusdottir; Louise le Roux; Gudrun Sigmundsdottir; Gardar Sveinbjornsson; Kristin E Sveinsdottir; Maney Sveinsdottir; Emil A Thorarensen; Bjarni Thorbjornsson; Gisli Masson; Ingileif Jonsdottir; Alma Moller; Thorolfur Gudnason; Karl G Kristinsson; Unnur Thorsteinsdottir; Kari Stefansson
EPI_ISL_417544 hCoV-19/Iceland/119/2020 Europe / Iceland / Reykjavik 2020-03-17 deCODE genetics deCODE genetics Daniel F Gudbjartsson; Agnar Helgason; Hakon Jonsson; Olafur T Magnusson; Pall Melsted; Gudmundur L Norddahl; Jona Saemundsdottir; Asgeir Sigurdsson; Patrick Sulem; Arna B Agustsdottir; Berglind Eiriksdottir; Run Fridriksdottir; Elisabet E Gardarsdottir; Gudmundur Georgsson; Olafia S Gretarsdottir; Kjartan R Gudmundsson; Thora R Gunnarsdottir; Arnaldur Gylfason; Hilma Holm; Brynjar O Jensson; Aslaug Jonasdottir; Kamilla S Josefsdottir; Thordur Kristjansson; Droplaug N Magnusdottir; Louise le Roux; Gudrun Sigmundsdottir; Gardar Sveinbjornsson; Kristin E Sveinsdottir; Maney Sveinsdottir; Emil A Thorarensen; Bjarni Thorbjornsson; Gisli Masson; Ingileif Jonsdottir; Alma Moller; Thorolfur Gudnason; Karl G Kristinsson; Unnur Thorsteinsdottir; Kari Stefansson
EPI_ISL_417545 hCoV-19/Iceland/120/2020 Europe / Iceland / Reykjavik 2020-03-17 deCODE genetics deCODE genetics Daniel F Gudbjartsson; Agnar Helgason; Hakon Jonsson; Olafur T Magnusson; Pall Melsted; Gudmundur L Norddahl; Jona Saemundsdottir; Asgeir Sigurdsson; Patrick Sulem; Arna B Agustsdottir; Berglind Eiriksdottir; Run Fridriksdottir; Elisabet E Gardarsdottir; Gudmundur Georgsson; Olafia S Gretarsdottir; Kjartan R Gudmundsson; Thora R Gunnarsdottir; Arnaldur Gylfason; Hilma Holm; Brynjar O Jensson; Aslaug Jonasdottir; Kamilla S Josefsdottir; Thordur Kristjansson; Droplaug N Magnusdottir; Louise le Roux; Gudrun Sigmundsdottir; Gardar Sveinbjornsson; Kristin E Sveinsdottir; Maney Sveinsdottir; Emil A Thorarensen; Bjarni Thorbjornsson; Gisli Masson; Ingileif Jonsdottir; Alma Moller; Thorolfur Gudnason; Karl G Kristinsson; Unnur Thorsteinsdottir; Kari Stefansson
EPI_ISL_417548 hCoV-19/Iceland/170/2020 Europe / Iceland / Reykjavik 2020-03-17 deCODE genetics deCODE genetics Daniel F Gudbjartsson; Agnar Helgason; Hakon Jonsson; Olafur T Magnusson; Pall Melsted; Gudmundur L Norddahl; Jona Saemundsdottir; Asgeir Sigurdsson; Patrick Sulem; Arna B Agustsdottir; Berglind Eiriksdottir; Run Fridriksdottir; Elisabet E Gardarsdottir; Gudmundur Georgsson; Olafia S Gretarsdottir; Kjartan R Gudmundsson; Thora R Gunnarsdottir; Arnaldur Gylfason; Hilma Holm; Brynjar O Jensson; Aslaug Jonasdottir; Kamilla S Josefsdottir; Thordur Kristjansson; Droplaug N Magnusdottir; Louise le Roux; Gudrun Sigmundsdottir; Gardar Sveinbjornsson; Kristin E Sveinsdottir; Maney Sveinsdottir; Emil A Thorarensen; Bjarni Thorbjornsson; Gisli Masson; Ingileif Jonsdottir; Alma Moller; Thorolfur Gudnason; Karl G Kristinsson; Unnur Thorsteinsdottir; Kari Stefansson
EPI_ISL_417549 hCoV-19/Iceland/171/2020 Europe / Iceland / Reykjavik 2020-03-14 deCODE genetics deCODE genetics Daniel F Gudbjartsson; Agnar Helgason; Hakon Jonsson; Olafur T Magnusson; Pall Melsted; Gudmundur L Norddahl; Jona Saemundsdottir; Asgeir Sigurdsson; Patrick Sulem; Arna B Agustsdottir; Berglind Eiriksdottir; Run Fridriksdottir; Elisabet E Gardarsdottir; Gudmundur Georgsson; Olafia S Gretarsdottir; Kjartan R Gudmundsson; Thora R Gunnarsdottir; Arnaldur Gylfason; Hilma Holm; Brynjar O Jensson; Aslaug

Jonasdottir; Kamilla S Josefsdottir; Thordur Kristjansson; Droplaug N Magnusdottir; Louise le Roux; Gudrun Sigmundsdottir; Gardar Sveinbjornsson; Kristin E Sveinsdottir; Maney Sveinsdottir; Emil A Thorarensen; Bjarni Thorbjornsson; Gisli Masson; Ingileif Jonsdottir; Alma Moller; Thorolfur Gudnason; Karl G Kristinsson; Unnur Thorsteinsdottir; Kari Stefansson
EPI_ISL_417550 hCoV-19/Iceland/184/2020 Europe / Iceland / Reykjavik 2020-03-16 The National University Hospital of Iceland deCODE genetics Daniel F Gudbjartsson; Agnar Helgason; Hakon Jonsson; Olafur T Magnusson; Pall Melsted; Gudmundur L Norddahl; Jona Saemundsdottir; Asgeir Sigurdsson; Patrick Sulem; Arna B Agustsdottir; Berglind Eiriksdottir; Run Fridriksdottir; Elisabet E Gardarsdottir; Gudmundur Georgsson; Olafia S Gretarsdottir; Kjartan R Gudmundsson; Thora R Gunnarsdottir; Arnaldur Gylfason; Hilma Holm; Brynjar O Jensson; Aslaug Jonasdottir; Kamilla S Josefsdottir; Thordur Kristjansson; Droplaug N Magnusdottir; Louise le Roux; Gudrun Sigmundsdottir; Gardar Sveinbjornsson; Kristin E Sveinsdottir; Maney Sveinsdottir; Emil A Thorarensen; Bjarni Thorbjornsson; Gisli Masson; Ingileif Jonsdottir; Alma Moller; Thorolfur Gudnason; Karl G Kristinsson; Unnur Thorsteinsdottir; Kari Stefansson
EPI_ISL_417551 hCoV-19/Iceland/187/2020 Europe / Iceland / Reykjavik 2020-03-16 The National University Hospital of Iceland deCODE genetics Daniel F Gudbjartsson; Agnar Helgason; Hakon Jonsson; Olafur T Magnusson; Pall Melsted; Gudmundur L Norddahl; Jona Saemundsdottir; Asgeir Sigurdsson; Patrick Sulem; Arna B Agustsdottir; Berglind Eiriksdottir; Run Fridriksdottir; Elisabet E Gardarsdottir; Gudmundur Georgsson; Olafia S Gretarsdottir; Kjartan R Gudmundsson; Thora R Gunnarsdottir; Arnaldur Gylfason; Hilma Holm; Brynjar O Jensson; Aslaug Jonasdottir; Kamilla S Josefsdottir; Thordur Kristjansson; Droplaug N Magnusdottir; Louise le Roux; Gudrun Sigmundsdottir; Gardar Sveinbjornsson; Kristin E Sveinsdottir; Maney Sveinsdottir; Emil A Thorarensen; Bjarni Thorbjornsson; Gisli Masson; Ingileif Jonsdottir; Alma Moller; Thorolfur Gudnason; Karl G Kristinsson; Unnur Thorsteinsdottir; Kari Stefansson
EPI_ISL_417552 hCoV-19/Iceland/2/2020 Europe / Iceland / Reykjavik 2020-03-16 deCODE genetics deCODE genetics Daniel F Gudbjartsson; Agnar Helgason; Hakon Jonsson; Olafur T Magnusson; Pall Melsted; Gudmundur L Norddahl; Jona Saemundsdottir; Asgeir Sigurdsson; Patrick Sulem; Arna B Agustsdottir; Berglind Eiriksdottir; Run Fridriksdottir; Elisabet E Gardarsdottir; Gudmundur Georgsson; Olafia S Gretarsdottir; Kjartan R Gudmundsson; Thora R Gunnarsdottir; Arnaldur Gylfason; Hilma Holm; Brynjar O Jensson; Aslaug Jonasdottir; Kamilla S Josefsdottir; Thordur Kristjansson; Droplaug N Magnusdottir; Louise le Roux; Gudrun Sigmundsdottir; Gardar Sveinbjornsson; Kristin E Sveinsdottir; Maney Sveinsdottir; Emil A Thorarensen; Bjarni Thorbjornsson; Gisli Masson; Ingileif Jonsdottir; Alma Moller; Thorolfur Gudnason; Karl G Kristinsson; Unnur Thorsteinsdottir; Kari Stefansson
EPI_ISL_417553 hCoV-19/Iceland/220/2020 Europe / Iceland / Reykjavik 2020-03-16 The National University Hospital of Iceland deCODE genetics Daniel F Gudbjartsson; Agnar Helgason; Hakon Jonsson; Olafur T Magnusson; Pall Melsted; Gudmundur L Norddahl; Jona Saemundsdottir; Asgeir Sigurdsson; Patrick Sulem; Arna B Agustsdottir; Berglind Eiriksdottir; Run Fridriksdottir; Elisabet E Gardarsdottir; Gudmundur Georgsson; Olafia S Gretarsdottir; Kjartan R Gudmundsson; Thora R Gunnarsdottir; Arnaldur Gylfason; Hilma Holm; Brynjar O Jensson; Aslaug Jonasdottir; Kamilla S Josefsdottir; Thordur Kristjansson; Droplaug N Magnusdottir; Louise le Roux; Gudrun Sigmundsdottir; Gardar Sveinbjornsson; Kristin E Sveinsdottir; Maney Sveinsdottir; Emil A Thorarensen; Bjarni Thorbjornsson; Gisli Masson; Ingileif Jonsdottir; Alma Moller; Thorolfur Gudnason; Karl G Kristinsson; Unnur Thorsteinsdottir; Kari Stefansson
EPI_ISL_417555 hCoV-19/Iceland/227/2020 Europe / Iceland / Reykjavik 2020-03-17 The National University Hospital of Iceland deCODE genetics Daniel F Gudbjartsson; Agnar Helgason; Hakon Jonsson; Olafur T Magnusson; Pall Melsted; Gudmundur L Norddahl; Jona Saemundsdottir; Asgeir Sigurdsson; Patrick Sulem; Arna B Agustsdottir; Berglind Eiriksdottir; Run Fridriksdottir; Elisabet E Gardarsdottir; Gudmundur Georgsson; Olafia S Gretarsdottir; Kjartan R Gudmundsson; Thora R Gunnarsdottir; Arnaldur Gylfason; Hilma Holm; Brynjar O Jensson; Aslaug Jonasdottir; Kamilla S Josefsdottir; Thordur Kristjansson; Droplaug N Magnusdottir; Louise le Roux; Gudrun Sigmundsdottir; Gardar Sveinbjornsson; Kristin E Sveinsdottir; Maney Sveinsdottir; Emil A Thorarensen; Bjarni Thorbjornsson; Gisli Masson; Ingileif Jonsdottir; Alma Moller; Thorolfur Gudnason; Karl G Kristinsson; Unnur Thorsteinsdottir; Kari Stefansson
EPI_ISL_417556 hCoV-19/Iceland/228/2020 Europe / Iceland / Reykjavik 2020-03-17 The National University Hospital of Iceland deCODE genetics Daniel F Gudbjartsson; Agnar Helgason; Hakon Jonsson; Olafur T Magnusson; Pall Melsted; Gudmundur L Norddahl; Jona Saemundsdottir; Asgeir Sigurdsson; Patrick Sulem; Arna B Agustsdottir; Berglind Eiriksdottir; Run Fridriksdottir; Elisabet E Gardarsdottir; Gudmundur Georgsson; Olafia S Gretarsdottir; Kjartan R Gudmundsson; Thora R Gunnarsdottir; Arnaldur Gylfason; Hilma Holm; Brynjar O Jensson; Aslaug Jonasdottir; Kamilla S Josefsdottir; Thordur Kristjansson; Droplaug N Magnusdottir; Louise le Roux; Gudrun Sigmundsdottir; Gardar Sveinbjornsson; Kristin E Sveinsdottir; Maney Sveinsdottir; Emil A Thorarensen; Bjarni Thorbjornsson; Gisli Masson; Ingileif Jonsdottir; Alma Moller; Thorolfur Gudnason; Karl G Kristinsson; Unnur Thorsteinsdottir; Kari Stefansson
EPI_ISL_417557 hCoV-19/Iceland/229/2020 Europe / Iceland / Reykjavik 2020-03-17 The National University Hospital of Iceland deCODE genetics Daniel F Gudbjartsson; Agnar Helgason; Hakon Jonsson; Olafur T Magnusson; Pall Melsted; Gudmundur L Norddahl; Jona Saemundsdottir; Asgeir Sigurdsson; Patrick Sulem; Arna B Agustsdottir; Berglind Eiriksdottir; Run Fridriksdottir; Elisabet E Gardarsdottir; Gudmundur Georgsson; Olafia S Gretarsdottir; Kjartan R Gudmundsson; Thora R Gunnarsdottir; Arnaldur Gylfason; Hilma Holm; Brynjar O Jensson; Aslaug Jonasdottir; Kamilla S Josefsdottir; Thordur Kristjansson; Droplaug N Magnusdottir; Louise le Roux; Gudrun Sigmundsdottir; Gardar Sveinbjornsson; Kristin E Sveinsdottir; Maney Sveinsdottir; Emil A Thorarensen; Bjarni Thorbjornsson; Gisli Masson; Ingileif Jonsdottir; Alma Moller; Thorolfur Gudnason; Karl G Kristinsson; Unnur Thorsteinsdottir; Kari Stefansson
EPI_ISL_417558 hCoV-19/Iceland/230/2020 Europe / Iceland / Reykjavik 2020-03-17 The National University Hospital of Iceland deCODE genetics Daniel F Gudbjartsson; Agnar Helgason; Hakon Jonsson; Olafur T Magnusson; Pall Melsted; Gudmundur L Norddahl; Jona Saemundsdottir; Asgeir Sigurdsson; Patrick Sulem; Arna B

Agustsdottir; Berglind Eiriksdottir; Run Fridriksdottir; Elisabet E Gardarsdottir; Gudmundur Georgsson; Olafia S Gretarsdottir; Kjartan R Gudmundsson; Thora R Gunnarsdottir; Arnaldur Gylfason; Hilma Holm; Brynjar O Jensson; Aslaug Jonasdottir; Kamilla S Josefsdottir; Thordur Kristjansson; Droplaug N Magnusdottir; Louise le Roux; Gudrun Sigmundsdottir; Gardar Sveinbjornsson; Kristin E Sveinsdottir; Maney Sveinsdottir; Emil A Thorarensen; Bjarni Thorbjornsson; Gisli Masson; Ingileif Jonsdottir; Alma Moller; Thorolfur Gudnason; Karl G Kristinsson; Unnur Thorsteinsdottir; Kari Stefansson
EPI_ISL_417560 hCoV-19/Iceland/232/2020 Europe / Iceland / Reykjavik 2020-03-17 The National University Hospital of Iceland deCODE genetics Daniel F Gudbjartsson; Agnar Helgason; Hakon Jonsson; Olafur T Magnusson; Pall Melsted; Gudmundur L Norddahl; Jona Saemundsdottir; Asgeir Sigurdsson; Patrick Sulem; Arna B Agustsdottir; Berglind Eiriksdottir; Run Fridriksdottir; Elisabet E Gardarsdottir; Gudmundur Georgsson; Olafia S Gretarsdottir; Kjartan R Gudmundsson; Thora R Gunnarsdottir; Arnaldur Gylfason; Hilma Holm; Brynjar O Jensson; Aslaug Jonasdottir; Kamilla S Josefsdottir; Thordur Kristjansson; Droplaug N Magnusdottir; Louise le Roux; Gudrun Sigmundsdottir; Gardar Sveinbjornsson; Kristin E Sveinsdottir; Maney Sveinsdottir; Emil A Thorarensen; Bjarni Thorbjornsson; Gisli Masson; Ingileif Jonsdottir; Alma Moller; Thorolfur Gudnason; Karl G Kristinsson; Unnur Thorsteinsdottir; Kari Stefansson
EPI_ISL_417561 hCoV-19/Iceland/233/2020 Europe / Iceland / Reykjavik 2020-03-17 The National University Hospital of Iceland deCODE genetics Daniel F Gudbjartsson; Agnar Helgason; Hakon Jonsson; Olafur T Magnusson; Pall Melsted; Gudmundur L Norddahl; Jona Saemundsdottir; Asgeir Sigurdsson; Patrick Sulem; Arna B Agustsdottir; Berglind Eiriksdottir; Run Fridriksdottir; Elisabet E Gardarsdottir; Gudmundur Georgsson; Olafia S Gretarsdottir; Kjartan R Gudmundsson; Thora R Gunnarsdottir; Arnaldur Gylfason; Hilma Holm; Brynjar O Jensson; Aslaug Jonasdottir; Kamilla S Josefsdottir; Thordur Kristjansson; Droplaug N Magnusdottir; Louise le Roux; Gudrun Sigmundsdottir; Gardar Sveinbjornsson; Kristin E Sveinsdottir; Maney Sveinsdottir; Emil A Thorarensen; Bjarni Thorbjornsson; Gisli Masson; Ingileif Jonsdottir; Alma Moller; Thorolfur Gudnason; Karl G Kristinsson; Unnur Thorsteinsdottir; Kari Stefansson
EPI_ISL_417562 hCoV-19/Iceland/234/2020 Europe / Iceland / Reykjavik 2020-03-17 The National University Hospital of Iceland deCODE genetics Daniel F Gudbjartsson; Agnar Helgason; Hakon Jonsson; Olafur T Magnusson; Pall Melsted; Gudmundur L Norddahl; Jona Saemundsdottir; Asgeir Sigurdsson; Patrick Sulem; Arna B Agustsdottir; Berglind Eiriksdottir; Run Fridriksdottir; Elisabet E Gardarsdottir; Gudmundur Georgsson; Olafia S Gretarsdottir; Kjartan R Gudmundsson; Thora R Gunnarsdottir; Arnaldur Gylfason; Hilma Holm; Brynjar O Jensson; Aslaug Jonasdottir; Kamilla S Josefsdottir; Thordur Kristjansson; Droplaug N Magnusdottir; Louise le Roux; Gudrun Sigmundsdottir; Gardar Sveinbjornsson; Kristin E Sveinsdottir; Maney Sveinsdottir; Emil A Thorarensen; Bjarni Thorbjornsson; Gisli Masson; Ingileif Jonsdottir; Alma Moller; Thorolfur Gudnason; Karl G Kristinsson; Unnur Thorsteinsdottir; Kari Stefansson
EPI_ISL_417563 hCoV-19/Iceland/235/2020 Europe / Iceland / Reykjavik 2020-03-16 The National University Hospital of Iceland deCODE genetics Daniel F Gudbjartsson; Agnar Helgason; Hakon Jonsson; Olafur T Magnusson; Pall Melsted; Gudmundur L Norddahl; Jona Saemundsdottir; Asgeir Sigurdsson; Patrick Sulem; Arna B Agustsdottir; Berglind Eiriksdottir; Run Fridriksdottir; Elisabet E Gardarsdottir; Gudmundur Georgsson; Olafia S Gretarsdottir; Kjartan R Gudmundsson; Thora R Gunnarsdottir; Arnaldur Gylfason; Hilma Holm; Brynjar O Jensson; Aslaug Jonasdottir; Kamilla S Josefsdottir; Thordur Kristjansson; Droplaug N Magnusdottir; Louise le Roux; Gudrun Sigmundsdottir; Gardar Sveinbjornsson; Kristin E Sveinsdottir; Maney Sveinsdottir; Emil A Thorarensen; Bjarni Thorbjornsson; Gisli Masson; Ingileif Jonsdottir; Alma Moller; Thorolfur Gudnason; Karl G Kristinsson; Unnur Thorsteinsdottir; Kari Stefansson
EPI_ISL_417564 hCoV-19/Iceland/236/2020 Europe / Iceland / Reykjavik 2020-03-17 The National University Hospital of Iceland deCODE genetics Daniel F Gudbjartsson; Agnar Helgason; Hakon Jonsson; Olafur T Magnusson; Pall Melsted; Gudmundur L Norddahl; Jona Saemundsdottir; Asgeir Sigurdsson; Patrick Sulem; Arna B Agustsdottir; Berglind Eiriksdottir; Run Fridriksdottir; Elisabet E Gardarsdottir; Gudmundur Georgsson; Olafia S Gretarsdottir; Kjartan R Gudmundsson; Thora R Gunnarsdottir; Arnaldur Gylfason; Hilma Holm; Brynjar O Jensson; Aslaug Jonasdottir; Kamilla S Josefsdottir; Thordur Kristjansson; Droplaug N Magnusdottir; Louise le Roux; Gudrun Sigmundsdottir; Gardar Sveinbjornsson; Kristin E Sveinsdottir; Maney Sveinsdottir; Emil A Thorarensen; Bjarni Thorbjornsson; Gisli Masson; Ingileif Jonsdottir; Alma Moller; Thorolfur Gudnason; Karl G Kristinsson; Unnur Thorsteinsdottir; Kari Stefansson
EPI_ISL_417565 hCoV-19/Iceland/237/2020 Europe / Iceland / Reykjavik 2020-03-17 The National University Hospital of Iceland deCODE genetics Daniel F Gudbjartsson; Agnar Helgason; Hakon Jonsson; Olafur T Magnusson; Pall Melsted; Gudmundur L Norddahl; Jona Saemundsdottir; Asgeir Sigurdsson; Patrick Sulem; Arna B Agustsdottir; Berglind Eiriksdottir; Run Fridriksdottir; Elisabet E Gardarsdottir; Gudmundur Georgsson; Olafia S Gretarsdottir; Kjartan R Gudmundsson; Thora R Gunnarsdottir; Arnaldur Gylfason; Hilma Holm; Brynjar O Jensson; Aslaug Jonasdottir; Kamilla S Josefsdottir; Thordur Kristjansson; Droplaug N Magnusdottir; Louise le Roux; Gudrun Sigmundsdottir; Gardar Sveinbjornsson; Kristin E Sveinsdottir; Maney Sveinsdottir; Emil A Thorarensen; Bjarni Thorbjornsson; Gisli Masson; Ingileif Jonsdottir; Alma Moller; Thorolfur Gudnason; Karl G Kristinsson; Unnur Thorsteinsdottir; Kari Stefansson
EPI_ISL_417566 hCoV-19/Iceland/238/2020 Europe / Iceland / Reykjavik 2020-03-17 The National University Hospital of Iceland deCODE genetics Daniel F Gudbjartsson; Agnar Helgason; Hakon Jonsson; Olafur T Magnusson; Pall Melsted; Gudmundur L Norddahl; Jona Saemundsdottir; Asgeir Sigurdsson; Patrick Sulem; Arna B Agustsdottir; Berglind Eiriksdottir; Run Fridriksdottir; Elisabet E Gardarsdottir; Gudmundur Georgsson; Olafia S Gretarsdottir; Kjartan R Gudmundsson; Thora R Gunnarsdottir; Arnaldur Gylfason; Hilma Holm; Brynjar O Jensson; Aslaug Jonasdottir; Kamilla S Josefsdottir; Thordur Kristjansson; Droplaug N Magnusdottir; Louise le Roux; Gudrun Sigmundsdottir; Gardar Sveinbjornsson; Kristin E Sveinsdottir; Maney Sveinsdottir; Emil A Thorarensen; Bjarni Thorbjornsson; Gisli Masson; Ingileif Jonsdottir; Alma Moller; Thorolfur Gudnason; Karl G Kristinsson; Unnur Thorsteinsdottir; Kari Stefansson
EPI_ISL_417567 hCoV-19/Iceland/239/2020 Europe / Iceland / Reykjavik 2020-03-17 The National

University Hospital of Iceland deCODE genetics Daniel F Gudbjartsson; Agnar Helgason; Hakon Jonsson; Olafur T Magnusson; Pall Melsted; Gudmundur L Norddahl; Jona Saemundsdottir; Asgeir Sigurdsson; Patrick Sulem; Arna B Agustsdottir; Berglind Eiriksdottir; Run Fridriksdottir; Elisabet E Gardarsdottir; Gudmundur Georgsson; Olafia S Gretarsdottir; Kjartan R Gudmundsson; Thora R Gunnarsdottir; Arnaldur Gylfason; Hilma Holm; Brynjar O Jensson; Aslaug Jonasdottir; Kamilla S Josefsdottir; Thordur Kristjansson; Droplaug N Magnusdottir; Louise le Roux; Gudrun Sigmundsdottir; Gardar Sveinbjornsson; Kristin E Sveinsdottir; Maney Sveinsdottir; Emil A Thorarensen; Bjarni Thorbjornsson; Gisli Masson; Ingileif Jonsdottir; Alma Moller; Thorolfur Gudnason; Karl G Kristinsson; Unnur Thorsteinsdottir; Kari Stefansson
EPI_ISL_417568 hCoV-19/Iceland/240/2020 Europe / Iceland / Reykjavik 2020-03-17 The National University Hospital of Iceland deCODE genetics Daniel F Gudbjartsson; Agnar Helgason; Hakon Jonsson; Olafur T Magnusson; Pall Melsted; Gudmundur L Norddahl; Jona Saemundsdottir; Asgeir Sigurdsson; Patrick Sulem; Arna B Agustsdottir; Berglind Eiriksdottir; Run Fridriksdottir; Elisabet E Gardarsdottir; Gudmundur Georgsson; Olafia S Gretarsdottir; Kjartan R Gudmundsson; Thora R Gunnarsdottir; Arnaldur Gylfason; Hilma Holm; Brynjar O Jensson; Aslaug Jonasdottir; Kamilla S Josefsdottir; Thordur Kristjansson; Droplaug N Magnusdottir; Louise le Roux; Gudrun Sigmundsdottir; Gardar Sveinbjornsson; Kristin E Sveinsdottir; Maney Sveinsdottir; Emil A Thorarensen; Bjarni Thorbjornsson; Gisli Masson; Ingileif Jonsdottir; Alma Moller; Thorolfur Gudnason; Karl G Kristinsson; Unnur Thorsteinsdottir; Kari Stefansson
EPI_ISL_417569 hCoV-19/Iceland/241/2020 Europe / Iceland / Reykjavik 2020-03-17 The National University Hospital of Iceland deCODE genetics Daniel F Gudbjartsson; Agnar Helgason; Hakon Jonsson; Olafur T Magnusson; Pall Melsted; Gudmundur L Norddahl; Jona Saemundsdottir; Asgeir Sigurdsson; Patrick Sulem; Arna B Agustsdottir; Berglind Eiriksdottir; Run Fridriksdottir; Elisabet E Gardarsdottir; Gudmundur Georgsson; Olafia S Gretarsdottir; Kjartan R Gudmundsson; Thora R Gunnarsdottir; Arnaldur Gylfason; Hilma Holm; Brynjar O Jensson; Aslaug Jonasdottir; Kamilla S Josefsdottir; Thordur Kristjansson; Droplaug N Magnusdottir; Louise le Roux; Gudrun Sigmundsdottir; Gardar Sveinbjornsson; Kristin E Sveinsdottir; Maney Sveinsdottir; Emil A Thorarensen; Bjarni Thorbjornsson; Gisli Masson; Ingileif Jonsdottir; Alma Moller; Thorolfur Gudnason; Karl G Kristinsson; Unnur Thorsteinsdottir; Kari Stefansson
EPI_ISL_417570 hCoV-19/Iceland/242/2020 Europe / Iceland / Reykjavik 2020-03-17 The National University Hospital of Iceland deCODE genetics Daniel F Gudbjartsson; Agnar Helgason; Hakon Jonsson; Olafur T Magnusson; Pall Melsted; Gudmundur L Norddahl; Jona Saemundsdottir; Asgeir Sigurdsson; Patrick Sulem; Arna B Agustsdottir; Berglind Eiriksdottir; Run Fridriksdottir; Elisabet E Gardarsdottir; Gudmundur Georgsson; Olafia S Gretarsdottir; Kjartan R Gudmundsson; Thora R Gunnarsdottir; Arnaldur Gylfason; Hilma Holm; Brynjar O Jensson; Aslaug Jonasdottir; Kamilla S Josefsdottir; Thordur Kristjansson; Droplaug N Magnusdottir; Louise le Roux; Gudrun Sigmundsdottir; Gardar Sveinbjornsson; Kristin E Sveinsdottir; Maney Sveinsdottir; Emil A Thorarensen; Bjarni Thorbjornsson; Gisli Masson; Ingileif Jonsdottir; Alma Moller; Thorolfur Gudnason; Karl G Kristinsson; Unnur Thorsteinsdottir; Kari Stefansson
EPI_ISL_417571 hCoV-19/Iceland/243/2020 Europe / Iceland / Reykjavik 2020-03-17 The National University Hospital of Iceland deCODE genetics Daniel F Gudbjartsson; Agnar Helgason; Hakon Jonsson; Olafur T Magnusson; Pall Melsted; Gudmundur L Norddahl; Jona Saemundsdottir; Asgeir Sigurdsson; Patrick Sulem; Arna B Agustsdottir; Berglind Eiriksdottir; Run Fridriksdottir; Elisabet E Gardarsdottir; Gudmundur Georgsson; Olafia S Gretarsdottir; Kjartan R Gudmundsson; Thora R Gunnarsdottir; Arnaldur Gylfason; Hilma Holm; Brynjar O Jensson; Aslaug Jonasdottir; Kamilla S Josefsdottir; Thordur Kristjansson; Droplaug N Magnusdottir; Louise le Roux; Gudrun Sigmundsdottir; Gardar Sveinbjornsson; Kristin E Sveinsdottir; Maney Sveinsdottir; Emil A Thorarensen; Bjarni Thorbjornsson; Gisli Masson; Ingileif Jonsdottir; Alma Moller; Thorolfur Gudnason; Karl G Kristinsson; Unnur Thorsteinsdottir; Kari Stefansson
EPI_ISL_417572 hCoV-19/Iceland/244/2020 Europe / Iceland / Reykjavik 2020-03-17 The National University Hospital of Iceland deCODE genetics Daniel F Gudbjartsson; Agnar Helgason; Hakon Jonsson; Olafur T Magnusson; Pall Melsted; Gudmundur L Norddahl; Jona Saemundsdottir; Asgeir Sigurdsson; Patrick Sulem; Arna B Agustsdottir; Berglind Eiriksdottir; Run Fridriksdottir; Elisabet E Gardarsdottir; Gudmundur Georgsson; Olafia S Gretarsdottir; Kjartan R Gudmundsson; Thora R Gunnarsdottir; Arnaldur Gylfason; Hilma Holm; Brynjar O Jensson; Aslaug Jonasdottir; Kamilla S Josefsdottir; Thordur Kristjansson; Droplaug N Magnusdottir; Louise le Roux; Gudrun Sigmundsdottir; Gardar Sveinbjornsson; Kristin E Sveinsdottir; Maney Sveinsdottir; Emil A Thorarensen; Bjarni Thorbjornsson; Gisli Masson; Ingileif Jonsdottir; Alma Moller; Thorolfur Gudnason; Karl G Kristinsson; Unnur Thorsteinsdottir; Kari Stefansson
EPI_ISL_417573 hCoV-19/Iceland/245/2020 Europe / Iceland / Reykjavik 2020-03-16 The National University Hospital of Iceland deCODE genetics Daniel F Gudbjartsson; Agnar Helgason; Hakon Jonsson; Olafur T Magnusson; Pall Melsted; Gudmundur L Norddahl; Jona Saemundsdottir; Asgeir Sigurdsson; Patrick Sulem; Arna B Agustsdottir; Berglind Eiriksdottir; Run Fridriksdottir; Elisabet E Gardarsdottir; Gudmundur Georgsson; Olafia S Gretarsdottir; Kjartan R Gudmundsson; Thora R Gunnarsdottir; Arnaldur Gylfason; Hilma Holm; Brynjar O Jensson; Aslaug Jonasdottir; Kamilla S Josefsdottir; Thordur Kristjansson; Droplaug N Magnusdottir; Louise le Roux; Gudrun Sigmundsdottir; Gardar Sveinbjornsson; Kristin E Sveinsdottir; Maney Sveinsdottir; Emil A Thorarensen; Bjarni Thorbjornsson; Gisli Masson; Ingileif Jonsdottir; Alma Moller; Thorolfur Gudnason; Karl G Kristinsson; Unnur Thorsteinsdottir; Kari Stefansson
EPI_ISL_417574 hCoV-19/Iceland/246/2020 Europe / Iceland / Reykjavik 2020-03-17 The National University Hospital of Iceland deCODE genetics Daniel F Gudbjartsson; Agnar Helgason; Hakon Jonsson; Olafur T Magnusson; Pall Melsted; Gudmundur L Norddahl; Jona Saemundsdottir; Asgeir Sigurdsson; Patrick Sulem; Arna B Agustsdottir; Berglind Eiriksdottir; Run Fridriksdottir; Elisabet E Gardarsdottir; Gudmundur Georgsson; Olafia S Gretarsdottir; Kjartan R Gudmundsson; Thora R Gunnarsdottir; Arnaldur Gylfason; Hilma Holm; Brynjar O Jensson; Aslaug Jonasdottir; Kamilla S Josefsdottir; Thordur Kristjansson; Droplaug N Magnusdottir; Louise le Roux; Gudrun Sigmundsdottir; Gardar Sveinbjornsson; Kristin E Sveinsdottir; Maney Sveinsdottir; Emil A Thorarensen; Bjarni Thorbjornsson; Gisli Masson; Ingileif Jonsdottir; Alma Moller; Thorolfur Gudnason; Karl G

Kristinsson; Unnur Thorsteinsdottir; Kari Stefansson
EPI_ISL_417575 hCoV-19/Iceland/247/2020 Europe / Iceland / Reykjavik 2020-03-17 The National University Hospital of Iceland deCODE genetics Daniel F Gudbjartsson; Agnar Helgason; Hakon Jonsson; Olafur T Magnusson; Pall Melsted; Gudmundur L Norddahl; Jona Saemundsdottir; Asgeir Sigurdsson; Patrick Sulem; Arna B Agustsdottir; Berglind Eiriksdottir; Run Fridriksdottir; Elisabet E Gardarsdottir; Gudmundur Georgsson; Olafia S Gretarsdottir; Kjartan R Gudmundsson; Thora R Gunnarsdottir; Arnaldur Gylfason; Hilma Holm; Brynjar O Jensson; Aslaug Jonasdottir; Kamilla S Josefsdottir; Thordur Kristjansson; Droplaug N Magnusdottir; Louise le Roux; Gudrun Sigmundsdottir; Gardar Sveinbjornsson; Kristin E Sveinsdottir; Maney Sveinsdottir; Emil A Thorarensen; Bjarni Thorbjornsson; Gisli Masson; Ingileif Jonsdottir; Alma Moller; Thorolfur Gudnason; Karl G Kristinsson; Unnur Thorsteinsdottir; Kari Stefansson
EPI_ISL_417576 hCoV-19/Iceland/248/2020 Europe / Iceland / Reykjavik 2020-03-17 The National University Hospital of Iceland deCODE genetics Daniel F Gudbjartsson; Agnar Helgason; Hakon Jonsson; Olafur T Magnusson; Pall Melsted; Gudmundur L Norddahl; Jona Saemundsdottir; Asgeir Sigurdsson; Patrick Sulem; Arna B Agustsdottir; Berglind Eiriksdottir; Run Fridriksdottir; Elisabet E Gardarsdottir; Gudmundur Georgsson; Olafia S Gretarsdottir; Kjartan R Gudmundsson; Thora R Gunnarsdottir; Arnaldur Gylfason; Hilma Holm; Brynjar O Jensson; Aslaug Jonasdottir; Kamilla S Josefsdottir; Thordur Kristjansson; Droplaug N Magnusdottir; Louise le Roux; Gudrun Sigmundsdottir; Gardar Sveinbjornsson; Kristin E Sveinsdottir; Maney Sveinsdottir; Emil A Thorarensen; Bjarni Thorbjornsson; Gisli Masson; Ingileif Jonsdottir; Alma Moller; Thorolfur Gudnason; Karl G Kristinsson; Unnur Thorsteinsdottir; Kari Stefansson
EPI_ISL_417577 hCoV-19/Iceland/249/2020 Europe / Iceland / Reykjavik 2020-03-17 The National University Hospital of Iceland deCODE genetics Daniel F Gudbjartsson; Agnar Helgason; Hakon Jonsson; Olafur T Magnusson; Pall Melsted; Gudmundur L Norddahl; Jona Saemundsdottir; Asgeir Sigurdsson; Patrick Sulem; Arna B Agustsdottir; Berglind Eiriksdottir; Run Fridriksdottir; Elisabet E Gardarsdottir; Gudmundur Georgsson; Olafia S Gretarsdottir; Kjartan R Gudmundsson; Thora R Gunnarsdottir; Arnaldur Gylfason; Hilma Holm; Brynjar O Jensson; Aslaug Jonasdottir; Kamilla S Josefsdottir; Thordur Kristjansson; Droplaug N Magnusdottir; Louise le Roux; Gudrun Sigmundsdottir; Gardar Sveinbjornsson; Kristin E Sveinsdottir; Maney Sveinsdottir; Emil A Thorarensen; Bjarni Thorbjornsson; Gisli Masson; Ingileif Jonsdottir; Alma Moller; Thorolfur Gudnason; Karl G Kristinsson; Unnur Thorsteinsdottir; Kari Stefansson
EPI_ISL_417580 hCoV-19/Iceland/252/2020 Europe / Iceland / Reykjavik 2020-03-17 The National University Hospital of Iceland deCODE genetics Daniel F Gudbjartsson; Agnar Helgason; Hakon Jonsson; Olafur T Magnusson; Pall Melsted; Gudmundur L Norddahl; Jona Saemundsdottir; Asgeir Sigurdsson; Patrick Sulem; Arna B Agustsdottir; Berglind Eiriksdottir; Run Fridriksdottir; Elisabet E Gardarsdottir; Gudmundur Georgsson; Olafia S Gretarsdottir; Kjartan R Gudmundsson; Thora R Gunnarsdottir; Arnaldur Gylfason; Hilma Holm; Brynjar O Jensson; Aslaug Jonasdottir; Kamilla S Josefsdottir; Thordur Kristjansson; Droplaug N Magnusdottir; Louise le Roux; Gudrun Sigmundsdottir; Gardar Sveinbjornsson; Kristin E Sveinsdottir; Maney Sveinsdottir; Emil A Thorarensen; Bjarni Thorbjornsson; Gisli Masson; Ingileif Jonsdottir; Alma Moller; Thorolfur Gudnason; Karl G Kristinsson; Unnur Thorsteinsdottir; Kari Stefansson
EPI_ISL_417581 hCoV-19/Iceland/253/2020 Europe / Iceland / Reykjavik 2020-03-17 The National University Hospital of Iceland deCODE genetics Daniel F Gudbjartsson; Agnar Helgason; Hakon Jonsson; Olafur T Magnusson; Pall Melsted; Gudmundur L Norddahl; Jona Saemundsdottir; Asgeir Sigurdsson; Patrick Sulem; Arna B Agustsdottir; Berglind Eiriksdottir; Run Fridriksdottir; Elisabet E Gardarsdottir; Gudmundur Georgsson; Olafia S Gretarsdottir; Kjartan R Gudmundsson; Thora R Gunnarsdottir; Arnaldur Gylfason; Hilma Holm; Brynjar O Jensson; Aslaug Jonasdottir; Kamilla S Josefsdottir; Thordur Kristjansson; Droplaug N Magnusdottir; Louise le Roux; Gudrun Sigmundsdottir; Gardar Sveinbjornsson; Kristin E Sveinsdottir; Maney Sveinsdottir; Emil A Thorarensen; Bjarni Thorbjornsson; Gisli Masson; Ingileif Jonsdottir; Alma Moller; Thorolfur Gudnason; Karl G Kristinsson; Unnur Thorsteinsdottir; Kari Stefansson
EPI_ISL_417582 hCoV-19/Iceland/254/2020 Europe / Iceland / Reykjavik 2020-03-17 The National University Hospital of Iceland deCODE genetics Daniel F Gudbjartsson; Agnar Helgason; Hakon Jonsson; Olafur T Magnusson; Pall Melsted; Gudmundur L Norddahl; Jona Saemundsdottir; Asgeir Sigurdsson; Patrick Sulem; Arna B Agustsdottir; Berglind Eiriksdottir; Run Fridriksdottir; Elisabet E Gardarsdottir; Gudmundur Georgsson; Olafia S Gretarsdottir; Kjartan R Gudmundsson; Thora R Gunnarsdottir; Arnaldur Gylfason; Hilma Holm; Brynjar O Jensson; Aslaug Jonasdottir; Kamilla S Josefsdottir; Thordur Kristjansson; Droplaug N Magnusdottir; Louise le Roux; Gudrun Sigmundsdottir; Gardar Sveinbjornsson; Kristin E Sveinsdottir; Maney Sveinsdottir; Emil A Thorarensen; Bjarni Thorbjornsson; Gisli Masson; Ingileif Jonsdottir; Alma Moller; Thorolfur Gudnason; Karl G Kristinsson; Unnur Thorsteinsdottir; Kari Stefansson
EPI_ISL_417583 hCoV-19/Iceland/255/2020 Europe / Iceland / Reykjavik 2020-03-17 The National University Hospital of Iceland deCODE genetics Daniel F Gudbjartsson; Agnar Helgason; Hakon Jonsson; Olafur T Magnusson; Pall Melsted; Gudmundur L Norddahl; Jona Saemundsdottir; Asgeir Sigurdsson; Patrick Sulem; Arna B Agustsdottir; Berglind Eiriksdottir; Run Fridriksdottir; Elisabet E Gardarsdottir; Gudmundur Georgsson; Olafia S Gretarsdottir; Kjartan R Gudmundsson; Thora R Gunnarsdottir; Arnaldur Gylfason; Hilma Holm; Brynjar O Jensson; Aslaug Jonasdottir; Kamilla S Josefsdottir; Thordur Kristjansson; Droplaug N Magnusdottir; Louise le Roux; Gudrun Sigmundsdottir; Gardar Sveinbjornsson; Kristin E Sveinsdottir; Maney Sveinsdottir; Emil A Thorarensen; Bjarni Thorbjornsson; Gisli Masson; Ingileif Jonsdottir; Alma Moller; Thorolfur Gudnason; Karl G Kristinsson; Unnur Thorsteinsdottir; Kari Stefansson
EPI_ISL_417584 hCoV-19/Iceland/256/2020 Europe / Iceland / Reykjavik 2020-03-17 The National University Hospital of Iceland deCODE genetics Daniel F Gudbjartsson; Agnar Helgason; Hakon Jonsson; Olafur T Magnusson; Pall Melsted; Gudmundur L Norddahl; Jona Saemundsdottir; Asgeir Sigurdsson; Patrick Sulem; Arna B Agustsdottir; Berglind Eiriksdottir; Run Fridriksdottir; Elisabet E Gardarsdottir; Gudmundur Georgsson; Olafia S Gretarsdottir; Kjartan R Gudmundsson; Thora R Gunnarsdottir; Arnaldur Gylfason; Hilma Holm; Brynjar O Jensson; Aslaug Jonasdottir; Kamilla S Josefsdottir; Thordur Kristjansson; Droplaug N Magnusdottir; Louise le

Roux; Gudrun Sigmundsdottir; Gardar Sveinbjornsson; Kristin E Sveinsdottir; Maney Sveinsdottir; Emil A Thorarensen; Bjarni Thorbjornsson; Gisli Masson; Ingileif Jonsdottir; Alma Moller; Thorolfur Gudnason; Karl G Kristinsson; Unnur Thorsteinsdottir; Kari Stefansson
EPI_ISL_417585 hCoV-19/Iceland/257/2020 Europe / Iceland / Reykjavik 2020-03-17 The National University Hospital of Iceland deCODE genetics Daniel F Gudbjartsson; Agnar Helgason; Hakon Jonsson; Olafur T Magnusson; Pall Melsted; Gudmundur L Norddahl; Jona Saemundsdottir; Asgeir Sigurdsson; Patrick Sulem; Arna B Agustsdottir; Berglind Eiriksdottir; Run Fridriksdottir; Elisabet E Gardarsdottir; Gudmundur Georgsson; Olafia S Gretarsdottir; Kjartan R Gudmundsson; Thora R Gunnarsdottir; Arnaldur Gylfason; Hilma Holm; Brynjar O Jensson; Aslaug Jonasdottir; Kamilla S Josefsdottir; Thordur Kristjansson; Droplaug N Magnusdottir; Louise le Roux; Gudrun Sigmundsdottir; Gardar Sveinbjornsson; Kristin E Sveinsdottir; Maney Sveinsdottir; Emil A Thorarensen; Bjarni Thorbjornsson; Gisli Masson; Ingileif Jonsdottir; Alma Moller; Thorolfur Gudnason; Karl G Kristinsson; Unnur Thorsteinsdottir; Kari Stefansson
EPI_ISL_417586 hCoV-19/Iceland/258/2020 Europe / Iceland / Reykjavik 2020-03-17 The National University Hospital of Iceland deCODE genetics Daniel F Gudbjartsson; Agnar Helgason; Hakon Jonsson; Olafur T Magnusson; Pall Melsted; Gudmundur L Norddahl; Jona Saemundsdottir; Asgeir Sigurdsson; Patrick Sulem; Arna B Agustsdottir; Berglind Eiriksdottir; Run Fridriksdottir; Elisabet E Gardarsdottir; Gudmundur Georgsson; Olafia S Gretarsdottir; Kjartan R Gudmundsson; Thora R Gunnarsdottir; Arnaldur Gylfason; Hilma Holm; Brynjar O Jensson; Aslaug Jonasdottir; Kamilla S Josefsdottir; Thordur Kristjansson; Droplaug N Magnusdottir; Louise le Roux; Gudrun Sigmundsdottir; Gardar Sveinbjornsson; Kristin E Sveinsdottir; Maney Sveinsdottir; Emil A Thorarensen; Bjarni Thorbjornsson; Gisli Masson; Ingileif Jonsdottir; Alma Moller; Thorolfur Gudnason; Karl G Kristinsson; Unnur Thorsteinsdottir; Kari Stefansson
EPI_ISL_417587 hCoV-19/Iceland/259/2020 Europe / Iceland / Reykjavik 2020-03-17 The National University Hospital of Iceland deCODE genetics Daniel F Gudbjartsson; Agnar Helgason; Hakon Jonsson; Olafur T Magnusson; Pall Melsted; Gudmundur L Norddahl; Jona Saemundsdottir; Asgeir Sigurdsson; Patrick Sulem; Arna B Agustsdottir; Berglind Eiriksdottir; Run Fridriksdottir; Elisabet E Gardarsdottir; Gudmundur Georgsson; Olafia S Gretarsdottir; Kjartan R Gudmundsson; Thora R Gunnarsdottir; Arnaldur Gylfason; Hilma Holm; Brynjar O Jensson; Aslaug Jonasdottir; Kamilla S Josefsdottir; Thordur Kristjansson; Droplaug N Magnusdottir; Louise le Roux; Gudrun Sigmundsdottir; Gardar Sveinbjornsson; Kristin E Sveinsdottir; Maney Sveinsdottir; Emil A Thorarensen; Bjarni Thorbjornsson; Gisli Masson; Ingileif Jonsdottir; Alma Moller; Thorolfur Gudnason; Karl G Kristinsson; Unnur Thorsteinsdottir; Kari Stefansson
EPI_ISL_417588 hCoV-19/Iceland/260/2020 Europe / Iceland / Reykjavik 2020-03-17 The National University Hospital of Iceland deCODE genetics Daniel F Gudbjartsson; Agnar Helgason; Hakon Jonsson; Olafur T Magnusson; Pall Melsted; Gudmundur L Norddahl; Jona Saemundsdottir; Asgeir Sigurdsson; Patrick Sulem; Arna B Agustsdottir; Berglind Eiriksdottir; Run Fridriksdottir; Elisabet E Gardarsdottir; Gudmundur Georgsson; Olafia S Gretarsdottir; Kjartan R Gudmundsson; Thora R Gunnarsdottir; Arnaldur Gylfason; Hilma Holm; Brynjar O Jensson; Aslaug Jonasdottir; Kamilla S Josefsdottir; Thordur Kristjansson; Droplaug N Magnusdottir; Louise le Roux; Gudrun Sigmundsdottir; Gardar Sveinbjornsson; Kristin E Sveinsdottir; Maney Sveinsdottir; Emil A Thorarensen; Bjarni Thorbjornsson; Gisli Masson; Ingileif Jonsdottir; Alma Moller; Thorolfur Gudnason; Karl G Kristinsson; Unnur Thorsteinsdottir; Kari Stefansson
EPI_ISL_417589 hCoV-19/Iceland/261/2020 Europe / Iceland / Reykjavik 2020-03-17 The National University Hospital of Iceland deCODE genetics Daniel F Gudbjartsson; Agnar Helgason; Hakon Jonsson; Olafur T Magnusson; Pall Melsted; Gudmundur L Norddahl; Jona Saemundsdottir; Asgeir Sigurdsson; Patrick Sulem; Arna B Agustsdottir; Berglind Eiriksdottir; Run Fridriksdottir; Elisabet E Gardarsdottir; Gudmundur Georgsson; Olafia S Gretarsdottir; Kjartan R Gudmundsson; Thora R Gunnarsdottir; Arnaldur Gylfason; Hilma Holm; Brynjar O Jensson; Aslaug Jonasdottir; Kamilla S Josefsdottir; Thordur Kristjansson; Droplaug N Magnusdottir; Louise le Roux; Gudrun Sigmundsdottir; Gardar Sveinbjornsson; Kristin E Sveinsdottir; Maney Sveinsdottir; Emil A Thorarensen; Bjarni Thorbjornsson; Gisli Masson; Ingileif Jonsdottir; Alma Moller; Thorolfur Gudnason; Karl G Kristinsson; Unnur Thorsteinsdottir; Kari Stefansson
EPI_ISL_417590 hCoV-19/Iceland/262/2020 Europe / Iceland / Reykjavik 2020-03-18 deCODE genetics deCODE genetics Daniel F Gudbjartsson; Agnar Helgason; Hakon Jonsson; Olafur T Magnusson; Pall Melsted; Gudmundur L Norddahl; Jona Saemundsdottir; Asgeir Sigurdsson; Patrick Sulem; Arna B Agustsdottir; Berglind Eiriksdottir; Run Fridriksdottir; Elisabet E Gardarsdottir; Gudmundur Georgsson; Olafia S Gretarsdottir; Kjartan R Gudmundsson; Thora R Gunnarsdottir; Arnaldur Gylfason; Hilma Holm; Brynjar O Jensson; Aslaug Jonasdottir; Kamilla S Josefsdottir; Thordur Kristjansson; Droplaug N Magnusdottir; Louise le Roux; Gudrun Sigmundsdottir; Gardar Sveinbjornsson; Kristin E Sveinsdottir; Maney Sveinsdottir; Emil A Thorarensen; Bjarni Thorbjornsson; Gisli Masson; Ingileif Jonsdottir; Alma Moller; Thorolfur Gudnason; Karl G Kristinsson; Unnur Thorsteinsdottir; Kari Stefansson
EPI_ISL_417591 hCoV-19/Iceland/263/2020 Europe / Iceland / Reykjavik 2020-03-17 The National University Hospital of Iceland deCODE genetics Daniel F Gudbjartsson; Agnar Helgason; Hakon Jonsson; Olafur T Magnusson; Pall Melsted; Gudmundur L Norddahl; Jona Saemundsdottir; Asgeir Sigurdsson; Patrick Sulem; Arna B Agustsdottir; Berglind Eiriksdottir; Run Fridriksdottir; Elisabet E Gardarsdottir; Gudmundur Georgsson; Olafia S Gretarsdottir; Kjartan R Gudmundsson; Thora R Gunnarsdottir; Arnaldur Gylfason; Hilma Holm; Brynjar O Jensson; Aslaug Jonasdottir; Kamilla S Josefsdottir; Thordur Kristjansson; Droplaug N Magnusdottir; Louise le Roux; Gudrun Sigmundsdottir; Gardar Sveinbjornsson; Kristin E Sveinsdottir; Maney Sveinsdottir; Emil A Thorarensen; Bjarni Thorbjornsson; Gisli Masson; Ingileif Jonsdottir; Alma Moller; Thorolfur Gudnason; Karl G Kristinsson; Unnur Thorsteinsdottir; Kari Stefansson
EPI_ISL_417592 hCoV-19/Iceland/264/2020 Europe / Iceland / Reykjavik 2020-03-17 The National University Hospital of Iceland deCODE genetics Daniel F Gudbjartsson; Agnar Helgason; Hakon Jonsson; Olafur T Magnusson; Pall Melsted; Gudmundur L Norddahl; Jona Saemundsdottir; Asgeir Sigurdsson; Patrick Sulem; Arna B Agustsdottir; Berglind Eiriksdottir; Run Fridriksdottir; Elisabet E Gardarsdottir; Gudmundur Georgsson; Olafia

S Gretarsdottir; Kjartan R Gudmundsson; Thora R Gunnarsdottir; Arnaldur Gylfason; Hilma Holm; Brynjar O Jensson; Aslaug Jonasdottir; Kamilla S Josefsdottir; Thordur Kristjansson; Droplaug N Magnusdottir; Louise le Roux; Gudrun Sigmundsdottir; Gardar Sveinbjornsson; Kristin E Sveinsdottir; Maney Sveinsdottir; Emil A Thorarensen; Bjarni Thorbjornsson; Gisli Masson; Ingileif Jonsdottir; Alma Moller; Thorolfur Gudnason; Karl G Kristinsson; Unnur Thorsteinsdottir; Kari Stefansson
EPI_ISL_417595 hCoV-19/Iceland/267/2020 Europe / Iceland / Reykjavik 2020-03-17 The National University Hospital of Iceland deCODE genetics Daniel F Gudbjartsson; Agnar Helgason; Hakon Jonsson; Olafur T Magnusson; Pall Melsted; Gudmundur L Norddahl; Jona Saemundsdottir; Asgeir Sigurdsson; Patrick Sulem; Arna B Agustsdottir; Berglind Eiriksdottir; Run Fridriksdottir; Elisabet E Gardarsdottir; Gudmundur Georgsson; Olafia S Gretarsdottir; Kjartan R Gudmundsson; Thora R Gunnarsdottir; Arnaldur Gylfason; Hilma Holm; Brynjar O Jensson; Aslaug Jonasdottir; Kamilla S Josefsdottir; Thordur Kristjansson; Droplaug N Magnusdottir; Louise le Roux; Gudrun Sigmundsdottir; Gardar Sveinbjornsson; Kristin E Sveinsdottir; Maney Sveinsdottir; Emil A Thorarensen; Bjarni Thorbjornsson; Gisli Masson; Ingileif Jonsdottir; Alma Moller; Thorolfur Gudnason; Karl G Kristinsson; Unnur Thorsteinsdottir; Kari Stefansson
EPI_ISL_417597 hCoV-19/Iceland/269/2020 Europe / Iceland / Reykjavik 2020-03-17 The National University Hospital of Iceland deCODE genetics Daniel F Gudbjartsson; Agnar Helgason; Hakon Jonsson; Olafur T Magnusson; Pall Melsted; Gudmundur L Norddahl; Jona Saemundsdottir; Asgeir Sigurdsson; Patrick Sulem; Arna B Agustsdottir; Berglind Eiriksdottir; Run Fridriksdottir; Elisabet E Gardarsdottir; Gudmundur Georgsson; Olafia S Gretarsdottir; Kjartan R Gudmundsson; Thora R Gunnarsdottir; Arnaldur Gylfason; Hilma Holm; Brynjar O Jensson; Aslaug Jonasdottir; Kamilla S Josefsdottir; Thordur Kristjansson; Droplaug N Magnusdottir; Louise le Roux; Gudrun Sigmundsdottir; Gardar Sveinbjornsson; Kristin E Sveinsdottir; Maney Sveinsdottir; Emil A Thorarensen; Bjarni Thorbjornsson; Gisli Masson; Ingileif Jonsdottir; Alma Moller; Thorolfur Gudnason; Karl G Kristinsson; Unnur Thorsteinsdottir; Kari Stefansson
EPI_ISL_417598 hCoV-19/Iceland/270/2020 Europe / Iceland / Reykjavik 2020-03-17 The National University Hospital of Iceland deCODE genetics Daniel F Gudbjartsson; Agnar Helgason; Hakon Jonsson; Olafur T Magnusson; Pall Melsted; Gudmundur L Norddahl; Jona Saemundsdottir; Asgeir Sigurdsson; Patrick Sulem; Arna B Agustsdottir; Berglind Eiriksdottir; Run Fridriksdottir; Elisabet E Gardarsdottir; Gudmundur Georgsson; Olafia S Gretarsdottir; Kjartan R Gudmundsson; Thora R Gunnarsdottir; Arnaldur Gylfason; Hilma Holm; Brynjar O Jensson; Aslaug Jonasdottir; Kamilla S Josefsdottir; Thordur Kristjansson; Droplaug N Magnusdottir; Louise le Roux; Gudrun Sigmundsdottir; Gardar Sveinbjornsson; Kristin E Sveinsdottir; Maney Sveinsdottir; Emil A Thorarensen; Bjarni Thorbjornsson; Gisli Masson; Ingileif Jonsdottir; Alma Moller; Thorolfur Gudnason; Karl G Kristinsson; Unnur Thorsteinsdottir; Kari Stefansson
EPI_ISL_418302 hCoV-19/England/SHEF-BFE9D/2020 Europe / United Kingdom / England / South Yorkshire 2020-03-24 Virology Department, Sheffield Teaching Hospitals NHS Foundation Trust Department of Infection, Immunity and Cardiovascular Disease, The Florey Institute, The Medical School, University of Sheffield Thushan de Silva, Matthew Parker, Adri Angyal, Rebecca Brown, Rachel Tucker, Paul Parsons, Danielle Groves, Alex Keeley, Dave Partridge, Matthew Wyles, Benjamin Lindsey, Mehmet Yavuz, Mohammad Raza, Cariad Evans
EPI_ISL_418303 hCoV-19/England/SHEF-BFEAC/2020 Europe / United Kingdom / England / Derbyshire 2020-03-25 Virology Department, Sheffield Teaching Hospitals NHS Foundation Trust Department of Infection, Immunity and Cardiovascular Disease, The Florey Institute, The Medical School, University of Sheffield Thushan de Silva, Matthew Parker, Adri Angyal, Rebecca Brown, Rachel Tucker, Paul Parsons, Danielle Groves, Alex Keeley, Dave Partridge, Matthew Wyles, Benjamin Lindsey, Mehmet Yavuz, Mohammad Raza, Cariad Evans
EPI_ISL_418304 hCoV-19/England/SHEF-BFEBB/2020 Europe / United Kingdom / England / Derbyshire 2020-03-24 Virology Department, Sheffield Teaching Hospitals NHS Foundation Trust Department of Infection, Immunity and Cardiovascular Disease, The Florey Institute, The Medical School, University of Sheffield Thushan de Silva, Matthew Parker, Adri Angyal, Rebecca Brown, Rachel Tucker, Paul Parsons, Danielle Groves, Alex Keeley, Dave Partridge, Matthew Wyles, Benjamin Lindsey, Mehmet Yavuz, Mohammad Raza, Cariad Evans
EPI_ISL_418305 hCoV-19/England/SHEF-BFED9/2020 Europe / United Kingdom / England / South Yorkshire 2020-03-25 Virology Department, Sheffield Teaching Hospitals NHS Foundation Trust Department of Infection, Immunity and Cardiovascular Disease, The Florey Institute, The Medical School, University of Sheffield Thushan de Silva, Matthew Parker, Adri Angyal, Rebecca Brown, Rachel Tucker, Paul Parsons, Danielle Groves, Alex Keeley, Dave Partridge, Matthew Wyles, Benjamin Lindsey, Mehmet Yavuz, Mohammad Raza, Cariad Evans
EPI_ISL_418306 hCoV-19/England/SHEF-BFEE8/2020 Europe / United Kingdom / England / South Yorkshire 2020-03-25 Virology Department, Sheffield Teaching Hospitals NHS Foundation Trust Department of Infection, Immunity and Cardiovascular Disease, The Florey Institute, The Medical School, University of Sheffield Thushan de Silva, Matthew Parker, Adri Angyal, Rebecca Brown, Rachel Tucker, Paul Parsons, Danielle Groves, Alex Keeley, Dave Partridge, Matthew Wyles, Benjamin Lindsey, Mehmet Yavuz, Mohammad Raza, Cariad Evans
EPI_ISL_418307 hCoV-19/England/SHEF-BFF03/2020 Europe / United Kingdom / England / Derbyshire 2020-03-24 Virology Department, Sheffield Teaching Hospitals NHS Foundation Trust Department of Infection, Immunity and Cardiovascular Disease, The Florey Institute, The Medical School, University of Sheffield Thushan de Silva, Matthew Parker, Adri Angyal, Rebecca Brown, Rachel Tucker, Paul Parsons, Danielle Groves, Alex Keeley, Dave Partridge, Matthew Wyles, Benjamin Lindsey, Mehmet Yavuz, Mohammad Raza, Cariad Evans
EPI_ISL_418313 hCoV-19/England/SHEF-BFF6D/2020 Europe / United Kingdom / England / Derbyshire 2020-03-24 Virology Department, Sheffield Teaching Hospitals NHS Foundation Trust Department of Infection, Immunity and Cardiovascular Disease, The Florey Institute, The Medical School, University of Sheffield Thushan de Silva, Matthew Parker, Adri Angyal, Rebecca Brown, Rachel Tucker, Paul Parsons, Danielle Groves, Alex Keeley, Dave Partridge, Matthew Wyles, Benjamin Lindsey, Mehmet Yavuz, Mohammad Raza, Cariad Evans
EPI_ISL_418317 hCoV-19/England/SHEF-BFFA9/2020 Europe / United Kingdom / England / South Yorkshire 2020-03-23 Virology Department, Sheffield Teaching Hospitals NHS Foundation Trust Department of Infection, Immunity and Cardiovascular Disease, The Florey Institute, The Medical School, University of Sheffield Thushan

de Silva, Matthew Parker, Adri Angyal, Rebecca Brown, Rachel Tucker, Paul Parsons, Danielle Groves, Alex Keeley, Dave Partridge, Matthew Wyles, Benjamin Lindsey, Mehmet Yavuz, Mohammad Raza, Cariad Evans
EPI_ISL_418318 hCoV-19/England/SHEF-BFFB8/2020 Europe / United Kingdom / England / South Yorkshire 2020-03-25 Virology Department, Sheffield Teaching Hospitals NHS Foundation Trust Department of Infection, Immunity and Cardiovascular Disease, The Florey Institute, The Medical School, University of Sheffield Thushan de Silva, Matthew Parker, Adri Angyal, Rebecca Brown, Rachel Tucker, Paul Parsons, Danielle Groves, Alex Keeley, Dave Partridge, Matthew Wyles, Benjamin Lindsey, Mehmet Yavuz, Mohammad Raza, Cariad Evans
EPI_ISL_418320 hCoV-19/England/SHEF-BFFD6/2020 Europe / United Kingdom / England / South Yorkshire 2020-03-25 Virology Department, Sheffield Teaching Hospitals NHS Foundation Trust Department of Infection, Immunity and Cardiovascular Disease, The Florey Institute, The Medical School, University of Sheffield Thushan de Silva, Matthew Parker, Adri Angyal, Rebecca Brown, Rachel Tucker, Paul Parsons, Danielle Groves, Alex Keeley, Dave Partridge, Matthew Wyles, Benjamin Lindsey, Mehmet Yavuz, Mohammad Raza, Cariad Evans
EPI_ISL_418322 hCoV-19/Canada/ON_PHL3917/2020 North America / Canada / Ontario 2020-03-08 Public Health Ontario Laboratories Public Health Ontario Laboratories Alireza Eshaghi, Samir N Patel, Jonathan B Gubbay, Vanessa G Allen, Christine Frantz, Aimin Li, Sandeep Nagra
EPI_ISL_418323 hCoV-19/Canada/ON_PHL3919/2020 North America / Canada / Ontario 2020-03-14 Public Health Ontario Laboratories Public Health Ontario Laboratories Alireza Eshaghi, Samir N Patel, Jonathan B Gubbay, Vanessa G Allen, Christine Frantz, Aimin Li, Sandeep Nagra
EPI_ISL_418324 hCoV-19/Canada/ON_PHL3318/2020 North America / Canada / Ontario 2020-03-11 Public Health Ontario Laboratories Public Health Ontario Laboratories Alireza Eshaghi, Samir N Patel, Jonathan B Gubbay, Vanessa G Allen, Christine Frantz, Aimin Li, Sandeep Nagra
EPI_ISL_418325 hCoV-19/Canada/ON_PHL5472/2020 North America / Canada / Ontario 2020-02-20 Public Health Ontario Laboratories Public Health Ontario Laboratories Alireza Eshaghi, Samir N Patel, Jonathan B Gubbay, Vanessa G Allen, Christine Frantz, Aimin Li, Sandeep Nagra
EPI_ISL_418326 hCoV-19/Canada/ON_PHL7972/2020 North America / Canada / Ontario 2020-03-12 Public Health Ontario Laboratories Public Health Ontario Laboratories Alireza Eshaghi, Samir N Patel, Jonathan B Gubbay, Vanessa G Allen, Christine Frantz, Aimin Li, Sandeep Nagra
EPI_ISL_418327 hCoV-19/Canada/ON_PHL4181/2020 North America / Canada / Ontario 2020-01-25 Public Health Ontario Laboratories Public Health Ontario Laboratories Alireza Eshaghi, Samir N Patel, Jonathan B Gubbay, Vanessa G Allen, Christine Frantz, Aimin Li, Sandeep Nagra
EPI_ISL_418328 hCoV-19/Canada/ON_PHL3670/2020 North America / Canada / Ontario 2020-03-20 Public Health Ontario Laboratories Public Health Ontario Laboratories Alireza Eshaghi, Samir N Patel, Jonathan B Gubbay, Vanessa G Allen, Christine Frantz, Aimin Li, Sandeep Nagra
EPI_ISL_418329 hCoV-19/Canada/ON_PHL5694/2020 North America / Canada / Ontario 2020-03-12 Public Health Ontario Laboratories Public Health Ontario Laboratories Alireza Eshaghi, Samir N Patel, Jonathan B Gubbay, Vanessa G Allen, Christine Frantz, Aimin Li, Sandeep Nagra
EPI_ISL_418330 hCoV-19/Canada/ON_PHL8580/2020 North America / Canada / Ontario 2020-03-05 Public Health Ontario Laboratories Public Health Ontario Laboratories Alireza Eshaghi, Samir N Patel, Jonathan B Gubbay, Vanessa G Allen, Christine Frantz, Aimin Li, Sandeep Nagra
EPI_ISL_418331 hCoV-19/Canada/ON_PHL6883/2020 North America / Canada / Ontario 2020-03-08 Public Health Ontario Laboratories Public Health Ontario Laboratories Alireza Eshaghi, Samir N Patel, Jonathan B Gubbay, Vanessa G Allen, Christine Frantz, Aimin Li, Sandeep Nagra
EPI_ISL_418332 hCoV-19/Canada/ON_PHL0052/2020 North America / Canada / Ontario 2020-03-11 Public Health Ontario Laboratories Public Health Ontario Laboratories Alireza Eshaghi, Samir N Patel, Jonathan B Gubbay, Vanessa G Allen, Christine Frantz, Aimin Li, Sandeep Nagra
EPI_ISL_418333 hCoV-19/Canada/ON_PHL3501/2020 North America / Canada / Ontario 2020-03-10 Public Health Ontario Laboratories Public Health Ontario Laboratories Alireza Eshaghi, Samir N Patel, Jonathan B Gubbay, Vanessa G Allen, Christine Frantz, Aimin Li, Sandeep Nagra
EPI_ISL_418334 hCoV-19/Canada/ON_PHL4069/2020 North America / Canada / Ontario 2020-03-07 Public Health Ontario Laboratories Public Health Ontario Laboratories Alireza Eshaghi, Samir N Patel, Jonathan B Gubbay, Vanessa G Allen, Christine Frantz, Aimin Li, Sandeep Nagra
EPI_ISL_418335 hCoV-19/Canada/ON_PHL4464/2020 North America / Canada / Ontario 2020-03-14 Public Health Ontario Laboratories Public Health Ontario Laboratories Alireza Eshaghi, Samir N Patel, Jonathan B Gubbay, Vanessa G Allen, Christine Frantz, Aimin Li, Sandeep Nagra
EPI_ISL_418336 hCoV-19/Canada/ON_PHL5705/2020 North America / Canada / Ontario 2020-03-12 Public Health Ontario Laboratories Public Health Ontario Laboratories Alireza Eshaghi, Samir N Patel, Jonathan B Gubbay, Vanessa G Allen, Christine Frantz, Aimin Li, Sandeep Nagra
EPI_ISL_418337 hCoV-19/Canada/ON_PHL3802/2020 North America / Canada / Ontario 2020-03-12 Public Health Ontario Laboratories Public Health Ontario Laboratories Alireza Eshaghi, Samir N Patel, Jonathan B Gubbay, Vanessa G Allen, Christine Frantz, Aimin Li, Sandeep Nagra
EPI_ISL_418338 hCoV-19/Canada/ON_PHL5710/2020 North America / Canada / Ontario 2020-03-12 Public Health Ontario Laboratories Public Health Ontario Laboratories Alireza Eshaghi, Samir N Patel, Jonathan B Gubbay, Vanessa G Allen, Christine Frantz, Aimin Li, Sandeep Nagra
EPI_ISL_418339 hCoV-19/Canada/ON_PHL3877/2020 North America / Canada / Ontario 2020-03-09 Public Health Ontario Laboratories Public Health Ontario Laboratories Alireza Eshaghi, Samir N Patel, Jonathan B Gubbay, Vanessa G Allen, Christine Frantz, Aimin Li, Sandeep Nagra
EPI_ISL_418340 hCoV-19/Canada/ON_PHL1083/2020 North America / Canada / Ontario 2020-03-09 Public Health Ontario Laboratories Public Health Ontario Laboratories Alireza Eshaghi, Samir N Patel, Jonathan B Gubbay, Vanessa G Allen, Christine Frantz, Aimin Li, Sandeep Nagra
EPI_ISL_418341 hCoV-19/Canada/ON_PHL0142/2020 North America / Canada / Ontario 2020-03-12 Public

Health Ontario Laboratories	Public Health Ontario Laboratories	Alireza Eshaghi, Samir N Patel, Jonathan B Gubbay, Vanessa G Allen, Christine Frantz, Aimin Li, Sandeep Nagra
EPI_ISL_418342	hCoV-19/Canada/ON_PHL0178/2020	North America / Canada / Ontario	2020-03-11	Public Health Ontario Laboratories	Public Health Ontario Laboratories	Alireza Eshaghi, Samir N Patel, Jonathan B Gubbay, Vanessa G Allen, Christine Frantz, Aimin Li, Sandeep Nagra
EPI_ISL_418343	hCoV-19/Canada/ON_PHL6884/2020	North America / Canada / Ontario	2020-03-10	Public Health Ontario Laboratories	Public Health Ontario Laboratories	Alireza Eshaghi, Samir N Patel, Jonathan B Gubbay, Vanessa G Allen, Christine Frantz, Aimin Li, Sandeep Nagra
EPI_ISL_418344	hCoV-19/Canada/ON_PHL2259/2020	North America / Canada / Ontario	2020-02-29	Public Health Ontario Laboratories	Public Health Ontario Laboratories	Alireza Eshaghi, Samir N Patel, Jonathan B Gubbay, Vanessa G Allen, Christine Frantz, Aimin Li, Sandeep Nagra
EPI_ISL_418345	hCoV-19/Canada/ON_PHL8751/2020	North America / Canada / Ontario	2020-02-29	Public Health Ontario Laboratories	Public Health Ontario Laboratories	Alireza Eshaghi, Samir N Patel, Jonathan B Gubbay, Vanessa G Allen, Christine Frantz, Aimin Li, Sandeep Nagra
EPI_ISL_418346	hCoV-19/Canada/ON_PHL0743/2020	North America / Canada / Ontario	2020-03-07	Public Health Ontario Laboratories	Public Health Ontario Laboratories	Alireza Eshaghi, Samir N Patel, Jonathan B Gubbay, Vanessa G Allen, Christine Frantz, Aimin Li, Sandeep Nagra
EPI_ISL_418347	hCoV-19/Canada/ON_PHL3741/2020	North America / Canada / Ontario	2020-03-11	Public Health Ontario Laboratories	Public Health Ontario Laboratories	Alireza Eshaghi, Samir N Patel, Jonathan B Gubbay, Vanessa G Allen, Christine Frantz, Aimin Li, Sandeep Nagra
EPI_ISL_418348	hCoV-19/Canada/ON_PHL3680/2020	North America / Canada / Ontario	2020-03-08	Public Health Ontario Laboratories	Public Health Ontario Laboratories	Alireza Eshaghi, Samir N Patel, Jonathan B Gubbay, Vanessa G Allen, Christine Frantz, Aimin Li, Sandeep Nagra
EPI_ISL_418349	hCoV-19/Canada/ON_PHL3650/2020	North America / Canada / Ontario	2020-03-07	Public Health Ontario Laboratories	Public Health Ontario Laboratories	Alireza Eshaghi, Samir N Patel, Jonathan B Gubbay, Vanessa G Allen, Christine Frantz, Aimin Li, Sandeep Nagra
EPI_ISL_418350	hCoV-19/Canada/ON_PHL3380/2020	North America / Canada / Ontario	2020-03-20	Public Health Ontario Laboratories	Public Health Ontario Laboratories	Alireza Eshaghi, Samir N Patel, Jonathan B Gubbay, Vanessa G Allen, Christine Frantz, Aimin Li, Sandeep Nagra
EPI_ISL_418351	hCoV-19/Canada/ON_PHL3575/2020	North America / Canada / Ontario	2020-03-12	Public Health Ontario Laboratories	Public Health Ontario Laboratories	Alireza Eshaghi, Samir N Patel, Jonathan B Gubbay, Vanessa G Allen, Christine Frantz, Aimin Li, Sandeep Nagra
EPI_ISL_418352	hCoV-19/Canada/ON_PHL5672/2020	North America / Canada / Ontario	2020-03-11	Public Health Ontario Laboratories	Public Health Ontario Laboratories	Alireza Eshaghi, Samir N Patel, Jonathan B Gubbay, Vanessa G Allen, Christine Frantz, Aimin Li, Sandeep Nagra
EPI_ISL_418353	hCoV-19/Canada/ON_PHL0539/2020	North America / Canada / Ontario	2020-03-09	Public Health Ontario Laboratories	Public Health Ontario Laboratories	Alireza Eshaghi, Samir N Patel, Jonathan B Gubbay, Vanessa G Allen, Christine Frantz, Aimin Li, Sandeep Nagra
EPI_ISL_418354	hCoV-19/Canada/ON_PHL4088/2020	North America / Canada / Ontario	2020-03-15	Public Health Ontario Laboratories	Public Health Ontario Laboratories	Alireza Eshaghi, Samir N Patel, Jonathan B Gubbay, Vanessa G Allen, Christine Frantz, Aimin Li, Sandeep Nagra
EPI_ISL_418355	hCoV-19/Canada/ON_PHLU8150/2020	North America / Canada / Ontario	2020-03-08	Public Health Ontario Laboratories	Public Health Ontario Laboratories	Alireza Eshaghi, Samir N Patel, Jonathan B Gubbay, Vanessa G Allen, Christine Frantz, Aimin Li, Sandeep Nagra
EPI_ISL_418356	hCoV-19/Canada/ON_PHL6980/2020	North America / Canada / Ontario	2020-03-12	Public Health Ontario Laboratories	Public Health Ontario Laboratories	Alireza Eshaghi, Samir N Patel, Jonathan B Gubbay, Vanessa G Allen, Christine Frantz, Aimin Li, Sandeep Nagra
EPI_ISL_418357	hCoV-19/Canada/ON_PHLH6415/2020	North America / Canada / Ontario	2020-03-12	Public Health Ontario Laboratories	Public Health Ontario Laboratories	Alireza Eshaghi, Samir N Patel, Jonathan B Gubbay, Vanessa G Allen, Christine Frantz, Aimin Li, Sandeep Nagra
EPI_ISL_418358	hCoV-19/Canada/ON_PHL5756/2020	North America / Canada / Ontario	2020-03-12	Public Health Ontario Laboratories	Public Health Ontario Laboratories	Alireza Eshaghi, Samir N Patel, Jonathan B Gubbay, Vanessa G Allen, Christine Frantz, Aimin Li, Sandeep Nagra
EPI_ISL_418359	hCoV-19/Canada/ON_PHL5757/2020	North America / Canada / Ontario	2020-03-12	Public Health Ontario Laboratories	Public Health Ontario Laboratories	Alireza Eshaghi, Samir N Patel, Jonathan B Gubbay, Vanessa G Allen, Christine Frantz, Aimin Li, Sandeep Nagra
EPI_ISL_418360	hCoV-19/Canada/ON_PHL3692/2020	North America / Canada / Ontario	2020-03-11	Public Health Ontario Laboratories	Public Health Ontario Laboratories	Alireza Eshaghi, Samir N Patel, Jonathan B Gubbay, Vanessa G Allen, Christine Frantz, Aimin Li, Sandeep Nagra
EPI_ISL_418361	hCoV-19/Canada/ON_PHL1095/2020	North America / Canada / Ontario	2020-03-12	Public Health Ontario Laboratories	Public Health Ontario Laboratories	Alireza Eshaghi, Samir N Patel, Jonathan B Gubbay, Vanessa G Allen, Christine Frantz, Aimin Li, Sandeep Nagra
EPI_ISL_418362	hCoV-19/Canada/ON_PHL3536/2020	North America / Canada / Ontario	2020-03-10	Public Health Ontario Laboratories	Public Health Ontario Laboratories	Alireza Eshaghi, Samir N Patel, Jonathan B Gubbay, Vanessa G Allen, Christine Frantz, Aimin Li, Sandeep Nagra
EPI_ISL_418363	hCoV-19/Canada/ON_PHL3695/2020	North America / Canada / Ontario	2020-03-11	Public Health Ontario Laboratories	Public Health Ontario Laboratories	Alireza Eshaghi, Samir N Patel, Jonathan B Gubbay, Vanessa G Allen, Christine Frantz, Aimin Li, Sandeep Nagra
EPI_ISL_418364	hCoV-19/Canada/ON_PHL6922/2020	North America / Canada / Ontario	2020-03-13	Public Health Ontario Laboratories	Public Health Ontario Laboratories	Alireza Eshaghi, Samir N Patel,

Jonathan B Gubbay, Vanessa G Allen, Christine Frantz, Aimin Li, Sandeep Nagra
EPI_ISL_418365 hCoV-19/Canada/ON_PHL0977/2020 North America / Canada / Ontario 2020-03-10 Public Health Ontario Laboratories Public Health Ontario Laboratories Alireza Eshaghi, Samir N Patel, Jonathan B Gubbay, Vanessa G Allen, Christine Frantz, Aimin Li, Sandeep Nagra
EPI_ISL_418366 hCoV-19/Canada/ON_PHL3350/2020 North America / Canada / Ontario 2020-03-05 Public Health Ontario Laboratories Public Health Ontario Laboratories Alireza Eshaghi, Samir N Patel, Jonathan B Gubbay, Vanessa G Allen, Christine Frantz, Aimin Li, Sandeep Nagra
EPI_ISL_418367 hCoV-19/Canada/ON_PHL7513/2020 North America / Canada / Ontario 2020-03-12 Public Health Ontario Laboratories Public Health Ontario Laboratories Alireza Eshaghi, Samir N Patel, Jonathan B Gubbay, Vanessa G Allen, Christine Frantz, Aimin Li, Sandeep Nagra
EPI_ISL_418368 hCoV-19/Canada/ON_PHL3458/2020 North America / Canada / Ontario 2020-03-12 Public Health Ontario Laboratories Public Health Ontario Laboratories Alireza Eshaghi, Samir N Patel, Jonathan B Gubbay, Vanessa G Allen, Christine Frantz, Aimin Li, Sandeep Nagra
EPI_ISL_418369 hCoV-19/Canada/ON_PHL8539/2020 North America / Canada / Ontario 2020-03-10 Public Health Ontario Laboratories Public Health Ontario Laboratories Alireza Eshaghi, Samir N Patel, Jonathan B Gubbay, Vanessa G Allen, Christine Frantz, Aimin Li, Sandeep Nagra
EPI_ISL_418370 hCoV-19/Canada/ON_PHL0654/2020 North America / Canada / Ontario 2020-03-11 Public Health Ontario Laboratories Public Health Ontario Laboratories Alireza Eshaghi, Samir N Patel, Jonathan B Gubbay, Vanessa G Allen, Christine Frantz, Aimin Li, Sandeep Nagra
EPI_ISL_418371 hCoV-19/Canada/ON_PHL2653/2020 North America / Canada / Ontario 2020-03-07 Public Health Ontario Laboratories Public Health Ontario Laboratories Alireza Eshaghi, Samir N Patel, Jonathan B Gubbay, Vanessa G Allen, Christine Frantz, Aimin Li, Sandeep Nagra
EPI_ISL_418372 hCoV-19/Canada/ON_PHL8458/2020 North America / Canada / Ontario 2020-03-12 Public Health Ontario Laboratories Public Health Ontario Laboratories Alireza Eshaghi, Samir N Patel, Jonathan B Gubbay, Vanessa G Allen, Christine Frantz, Aimin Li, Sandeep Nagra
EPI_ISL_418373 hCoV-19/Canada/ON_PHL7590/2020 North America / Canada / Ontario 2020-03-14 Public Health Ontario Laboratories Public Health Ontario Laboratories Alireza Eshaghi, Samir N Patel, Jonathan B Gubbay, Vanessa G Allen, Christine Frantz, Aimin Li, Sandeep Nagra
EPI_ISL_418374 hCoV-19/Canada/ON_PHL4232/2020 North America / Canada / Ontario 2020-03-11 Public Health Ontario Laboratories Public Health Ontario Laboratories Alireza Eshaghi, Samir N Patel, Jonathan B Gubbay, Vanessa G Allen, Christine Frantz, Aimin Li, Sandeep Nagra
EPI_ISL_418375 hCoV-19/Canada/ON_PHL0141/2020 North America / Canada / Ontario 2020-03-12 Public Health Ontario Laboratories Public Health Ontario Laboratories Alireza Eshaghi, Samir N Patel, Jonathan B Gubbay, Vanessa G Allen, Christine Frantz, Aimin Li, Sandeep Nagra
EPI_ISL_418376 hCoV-19/Canada/ON_PHL3459/2020 North America / Canada / Ontario 2020-03-12 Public Health Ontario Laboratories Public Health Ontario Laboratories Alireza Eshaghi, Samir N Patel, Jonathan B Gubbay, Vanessa G Allen, Christine Frantz, Aimin Li, Sandeep Nagra
EPI_ISL_418377 hCoV-19/Canada/ON_PHL1898/2020 North America / Canada / Ontario 2020-03-13 Public Health Ontario Laboratories Public Health Ontario Laboratories Alireza Eshaghi, Samir N Patel, Jonathan B Gubbay, Vanessa G Allen, Christine Frantz, Aimin Li, Sandeep Nagra
EPI_ISL_418378 hCoV-19/Canada/ON_PHL0976/2020 North America / Canada / Ontario 2020-03-13 Public Health Ontario Laboratories Public Health Ontario Laboratories Alireza Eshaghi, Samir N Patel, Jonathan B Gubbay, Vanessa G Allen, Christine Frantz, Aimin Li, Sandeep Nagra
EPI_ISL_418379 hCoV-19/Canada/ON_PHL7512/2020 North America / Canada / Ontario 2020-03-13 Public Health Ontario Laboratories Public Health Ontario Laboratories Alireza Eshaghi, Samir N Patel, Jonathan B Gubbay, Vanessa G Allen, Christine Frantz, Aimin Li, Sandeep Nagra
EPI_ISL_418380 hCoV-19/Canada/ON_PHL3476/2020 North America / Canada / Ontario 2020-03-13 Public Health Ontario Laboratories Public Health Ontario Laboratories Alireza Eshaghi, Samir N Patel, Jonathan B Gubbay, Vanessa G Allen, Christine Frantz, Aimin Li, Sandeep Nagra
EPI_ISL_418381 hCoV-19/Canada/ON_PHL2223/2020 North America / Canada / Ontario 2020-03-19 Public Health Ontario Laboratories Public Health Ontario Laboratories Alireza Eshaghi, Samir N Patel, Jonathan B Gubbay, Vanessa G Allen, Christine Frantz, Aimin Li, Sandeep Nagra
EPI_ISL_418382 hCoV-19/Canada/ON_PHL5930/2020 North America / Canada / Ontario 2020-02-28 Public Health Ontario Laboratories Public Health Ontario Laboratories Alireza Eshaghi, Samir N Patel, Jonathan B Gubbay, Vanessa G Allen, Christine Frantz, Aimin Li, Sandeep Nagra
EPI_ISL_418383 hCoV-19/Canada/ON_PHL2273/2020 North America / Canada / Ontario 2020-02-29 Public Health Ontario Laboratories Public Health Ontario Laboratories Alireza Eshaghi, Samir N Patel, Jonathan B Gubbay, Vanessa G Allen, Christine Frantz, Aimin Li, Sandeep Nagra
EPI_ISL_418385 hCoV-19/Finland/13M19/2020 Europe / Finland 2020-03-13 Department of Virology and Immunology, University of Helsinki and Helsinki University Hospital, Huslab Finland Department of Virology, Faculty of Medicine, University of Helsinki, Helsinki, Finland Teemu Smura, Hannimari Kallio-Kokko, Olli Vapalahti
EPI_ISL_418386 hCoV-19/Finland/13M26/2020 Europe / Finland 2020-03-13 Department of Virology and Immunology, University of Helsinki and Helsinki University Hospital, Huslab Finland Department of Virology, Faculty of Medicine, University of Helsinki, Helsinki, Finland Teemu Smura, Hannimari Kallio-Kokko, Olli Vapalahti
EPI_ISL_418387 hCoV-19/Finland/13M27/2020 Europe / Finland 2020-03-13 Department of Virology and Immunology, University of Helsinki and Helsinki University Hospital, Huslab Finland Department of Virology, Faculty of Medicine, University of Helsinki, Helsinki, Finland Teemu Smura, Hannimari Kallio-Kokko, Olli Vapalahti

EPI_ISL_418388 hCoV-19/Finland/13M29/2020 Europe / Finland 2020-03-13 Department of Virology and Immunology, University of Helsinki and Helsinki University Hospital, Huslab Finland Department of Virology, Faculty of Medicine, University of Helsinki, Helsinki, Finland Teemu Smura, Hannimari Kallio-Kokko, Olli Vapalahti
EPI_ISL_418389 hCoV-19/Finland/13M3/2020 Europe / Finland 2020-03-13 Department of Virology and Immunology, University of Helsinki and Helsinki University Hospital, Huslab Finland Department of Virology, Faculty of Medicine, University of Helsinki, Helsinki, Finland Teemu Smura, Hannimari Kallio-Kokko, Olli Vapalahti
EPI_ISL_418390 hCoV-19/Finland/13M33/2020 Europe / Finland 2020-03-13 Department of Virology and Immunology, University of Helsinki and Helsinki University Hospital, Huslab Finland Department of Virology, Faculty of Medicine, University of Helsinki, Helsinki, Finland Teemu Smura, Hannimari Kallio-Kokko, Olli Vapalahti
EPI_ISL_418391 hCoV-19/Finland/13M57/2020 Europe / Finland 2020-03-13 Department of Virology and Immunology, University of Helsinki and Helsinki University Hospital, Huslab Finland Department of Virology, Faculty of Medicine, University of Helsinki, Helsinki, Finland Teemu Smura, Hannimari Kallio-Kokko, Olli Vapalahti
EPI_ISL_418392 hCoV-19/Finland/13M58/2020 Europe / Finland 2020-03-13 Department of Virology and Immunology, University of Helsinki and Helsinki University Hospital, Huslab Finland Department of Virology, Faculty of Medicine, University of Helsinki, Helsinki, Finland Teemu Smura, Hannimari Kallio-Kokko, Olli Vapalahti
EPI_ISL_418393 hCoV-19/Finland/13M60/2020 Europe / Finland 2020-03-13 Department of Virology and Immunology, University of Helsinki and Helsinki University Hospital, Huslab Finland Department of Virology, Faculty of Medicine, University of Helsinki, Helsinki, Finland Teemu Smura, Hannimari Kallio-Kokko, Olli Vapalahti
EPI_ISL_418394 hCoV-19/Finland/13M64/2020 Europe / Finland 2020-03-13 Department of Virology and Immunology, University of Helsinki and Helsinki University Hospital, Huslab Finland Department of Virology, Faculty of Medicine, University of Helsinki, Helsinki, Finland Teemu Smura, Hannimari Kallio-Kokko, Olli Vapalahti
EPI_ISL_418395 hCoV-19/Finland/13M65/2020 Europe / Finland 2020-03-13 Department of Virology and Immunology, University of Helsinki and Helsinki University Hospital, Huslab Finland Department of Virology, Faculty of Medicine, University of Helsinki, Helsinki, Finland Teemu Smura, Hannimari Kallio-Kokko, Olli Vapalahti
EPI_ISL_418396 hCoV-19/Finland/13M69/2020 Europe / Finland 2020-03-13 Department of Virology and Immunology, University of Helsinki and Helsinki University Hospital, Huslab Finland Department of Virology, Faculty of Medicine, University of Helsinki, Helsinki, Finland Teemu Smura, Hannimari Kallio-Kokko, Olli Vapalahti
EPI_ISL_418397 hCoV-19/Finland/13M77/2020 Europe / Finland 2020-03-13 Department of Virology and Immunology, University of Helsinki and Helsinki University Hospital, Huslab Finland Department of Virology, Faculty of Medicine, University of Helsinki, Helsinki, Finland Teemu Smura, Hannimari Kallio-Kokko, Olli Vapalahti
EPI_ISL_418399 hCoV-19/Finland/13M82/2020 Europe / Finland 2020-03-13 Department of Virology and Immunology, University of Helsinki and Helsinki University Hospital, Huslab Finland Department of Virology, Faculty of Medicine, University of Helsinki, Helsinki, Finland Teemu Smura, Hannimari Kallio-Kokko, Olli Vapalahti
EPI_ISL_418800 hCoV-19/Belgium/CS-031052/2020 Europe / Belgium / Gierle 2020-03-10 KU Leuven, Clinical and Epidemiological Virology KU Leuven, Clinical and Epidemiological Virology Bert Vanmechelen, Joan Marti-Carreras, Tony Wawina, Piet Maes
EPI_ISL_418801 hCoV-19/Australia/QLDID923/2020 Oceania / Australia / Queensland / Brisbane 2020-03-14 Mater Pathology Public Health Virology Laboratory Bixing Huang, Alyssa Pyke, Amanda De Jong, Andrew Van Den Hurk, Carmel Taylor, David Warrilow, Doris Genge, Elisabeth Gamez, Glen Hewitson, Ian Maxwell Mackay, Inga Sultana, Jamie McMahon, Jean Barcelon, Judy Northill, Mitchell Finger, Natalie Simpson, Neelima Nair, Peter Burtonclay, Peter Moore, Sarah Wheatley, Sean Moody, Sonja Hall-Mendelin, Timothy Gardam, and Frederick Moore
EPI_ISL_418802 hCoV-19/Australia/QLDID924/2020 Oceania / Australia / Queensland / Gold Coast 2020-03-18 Pathology Queensland Public Health Virology Laboratory Bixing Huang, Alyssa Pyke, Amanda De Jong, Andrew Van Den Hurk, Carmel Taylor, David Warrilow, Doris Genge, Elisabeth Gamez, Glen Hewitson, Ian Maxwell Mackay, Inga Sultana, Jamie McMahon, Jean Barcelon, Judy Northill, Mitchell Finger, Natalie Simpson, Neelima Nair, Peter Burtonclay, Peter Moore, Sarah Wheatley, Sean Moody, Sonja Hall-Mendelin, Timothy Gardam, and Frederick Moore
EPI_ISL_418803 hCoV-19/Australia/QLDID925/2020 Oceania / Australia / Queensland / Sunshine Coast 2020-03-18 Pathology Queensland Public Health Virology Laboratory Bixing Huang, Alyssa Pyke, Amanda De Jong, Andrew Van Den Hurk, Carmel Taylor, David Warrilow, Doris Genge, Elisabeth Gamez, Glen Hewitson, Ian Maxwell Mackay, Inga Sultana, Jamie McMahon, Jean Barcelon, Judy Northill, Mitchell Finger, Natalie Simpson, Neelima Nair, Peter Burtonclay, Peter Moore, Sarah Wheatley, Sean Moody, Sonja Hall-Mendelin, Timothy Gardam, and Frederick Moore
EPI_ISL_418804 hCoV-19/Australia/QLDID926/2020 Oceania / Australia / Queensland / Sunshine Coast 2020-03-18 Pathology Queensland Public Health Virology Laboratory Bixing Huang, Alyssa Pyke, Amanda De Jong, Andrew Van Den Hurk, Carmel Taylor, David Warrilow, Doris Genge, Elisabeth Gamez, Glen Hewitson, Ian Maxwell Mackay, Inga Sultana, Jamie McMahon, Jean Barcelon, Judy Northill, Mitchell Finger, Natalie Simpson, Neelima Nair, Peter Burtonclay, Peter Moore, Sarah Wheatley, Sean Moody, Sonja Hall-Mendelin, Timothy Gardam, and Frederick Moore

EPI_ISL_418805 hCoV-19/Belgium/VPE-030650/2020 Europe / Belgium / Deinze 2020-03-06 KU Leuven, Clinical and Epidemiological Virology KU Leuven, Clinical and Epidemiological Virology Bert Vanmechelen, Joan Marti-Carreras, Tony Wawina, Piet Maes
EPI_ISL_418806 hCoV-19/Belgium/BG-030551/2020 Europe / Belgium / Beersel 2020-03-05 KU Leuven, Clinical and Epidemiological Virology KU Leuven, Clinical and Epidemiological Virology Bert Vanmechelen, Joan Marti-Carreras, Tony Wawina, Piet Maes
EPI_ISL_418807 hCoV-19/Australia/QLDID927/2020 Oceania / Australia / Queensland / Brisbane 2020-03-19 Pathology Queensland Public Health Virology Laboratory Bixing Huang, Alyssa Pyke, Amanda De Jong, Andrew Van Den Hurk, Carmel Taylor, David Warrilow, Doris Genge, Elisabeth Gamez, Glen Hewitson, Ian Maxwell Mackay, Inga Sultana, Jamie McMahon, Jean Barcelon, Judy Northill, Mitchell Finger, Natalie Simpson, Neelima Nair, Peter Burtonclay, Peter Moore, Sarah Wheatley, Sean Moody, Sonja Hall-Mendelin, Timothy Gardam, and Frederick Moore
EPI_ISL_418808 hCoV-19/Australia/QLDID928/2020 Oceania / Australia / Queensland / Brisbane 2020-03-19 Pathology Queensland Public Health Virology Laboratory Bixing Huang, Alyssa Pyke, Amanda De Jong, Andrew Van Den Hurk, Carmel Taylor, David Warrilow, Doris Genge, Elisabeth Gamez, Glen Hewitson, Ian Maxwell Mackay, Inga Sultana, Jamie McMahon, Jean Barcelon, Judy Northill, Mitchell Finger, Natalie Simpson, Neelima Nair, Peter Burtonclay, Peter Moore, Sarah Wheatley, Sean Moody, Sonja Hall-Mendelin, Timothy Gardam, and Frederick Moore
EPI_ISL_418809 hCoV-19/Japan/UT-NCGM02/2020 Asia / Japan / Tokyo 2020-02-01 University of Wisconsin - Madison: Influenza Research Institute University of Wisconsin Madison, AIDS Vaccine Research Laboratories Katarina Braun, Gage Moreno, Peter Halfmann, et al.
EPI_ISL_418811 hCoV-19/Canada/NB_6/2020 North America / Canada / New Brunswick 2020-03-12 Dr. Georges-L.-Dumont University Hospital Centre National Microbiology Laboratory Anna Majer, Shari Tyson, Grace Seo, Philip Mabon, Natalie Knox, Morag Graham, Richard Garceau, Guillaume Desnoyers, Nathalie Bastien, Yan Li, Matthew Gilmour, Timothy Booth
EPI_ISL_418812 hCoV-19/Canada/MB_8/2020 North America / Canada / Manitoba 2020-03-12 Cadham Provincial Laboratory National Microbiology Laboratory Anna Majer, Shari Tyson, Grace Seo, Philip Mabon, Natalie Knox, Morag Graham, Paul Van Caeseele, Jared Bullard, David Alexander, Kerry Dust, Nathalie Bastien, Yan Li, Matthew Gilmour, Timothy Booth
EPI_ISL_418813 hCoV-19/Canada/MB_10/2020 North America / Canada / Manitoba 2020-03-13 Cadham Provincial Laboratory National Microbiology Laboratory Anna Majer, Shari Tyson, Grace Seo, Philip Mabon, Natalie Knox, Morag Graham, Paul Van Caeseele, Jared Bullard, David Alexander, Kerry Dust, Nathalie Bastien, Yan Li, Matthew Gilmour, Timothy Booth
EPI_ISL_418814 hCoV-19/Canada/NS_13/2020 North America / Canada / Nova Scotia 2020-03-13 Queen Elizabeth II Health Science Centre National Microbiology Laboratory Anna Majer, Shari Tyson, Grace Seo, Philip Mabon, Natalie Knox, Morag Graham, Todd Hatchette, Jason LeBlanc, Nathalie Bastien, Yan Li, Matthew Gilmour, Timothy Booth
EPI_ISL_418815 hCoV-19/Hong Kong/HKPU36-0702/2020 Asia / Hong Kong 2020-02-09 Department of Clinical Pathology, Pamela Youde Nethersole Eastern Hospital Department of Health Technology and Informatics, Faculty of Health and Social Science, The Hong Kong Polytechnic University Kenneth Siu-Sing LEUNG, Timothy Ting-Leung NG, Alan Ka-Lun WU, Miranda Chong-Yee YAU, Hiu-Yin LAO, Ming-Pan CHOI, Kingsley King-Gee TAM, Lam-Kwong LEE, Barry Kin-Chung WONG, Alex Yat-Man HO, Kam-Tong YIP, Kwok-Cheung LUNG, Raymond Wai-To LIU, Eugene Yuk-Keung TSO, Wai-Shing LEUNG, Man-Chun CHAN, Yuk-Yung NG, Kit-Man SIN, Kitty Sau-Chun FUNG, Sandy Ka-Yee CHAU, Wing-Kin TO, Tak-Lun QUE, David Ho-Keung SHUM, Shea Ping YIP, Wing Cheong YAM, Gilman Kit-Hang SIU
EPI_ISL_418819 hCoV-19/Canada/BC_3808524/2020 North America / Canada / British Columbia 2020-03-09 BCCDC Public Health Laboratory BCCDC Public Health Laboratory Harrigan, Prystajecky, Krajden, Lee, Kamelian, Lapointe, Choi, Hoang, Sekirov, Levett, Tyson, Snutch, Loman, Quick, Li, Gilmour
EPI_ISL_418820 hCoV-19/Canada/BC_3842755/2020 North America / Canada / British Columbia 2020-03-09 BCCDC Public Health Laboratory BCCDC Public Health Laboratory Harrigan, Prystajecky, Krajden, Lee, Kamelian, Lapointe, Choi, Hoang, Sekirov, Levett, Tyson, Snutch, Loman, Quick, Li, Gilmour
EPI_ISL_418821 hCoV-19/Canada/BC_3968175/2020 North America / Canada / British Columbia 2020-03-10 BCCDC Public Health Laboratory BCCDC Public Health Laboratory Harrigan, Prystajecky, Krajden, Lee, Kamelian, Lapointe, Choi, Hoang, Sekirov, Levett, Tyson, Snutch, Loman, Quick, Li, Gilmour
EPI_ISL_418822 hCoV-19/Canada/BC_3972884/2020 North America / Canada / British Columbia 2020-03-10 BCCDC Public Health Laboratory BCCDC Public Health Laboratory Harrigan, Prystajecky, Krajden, Lee, Kamelian, Lapointe, Choi, Hoang, Sekirov, Levett, Tyson, Snutch, Loman, Quick, Li, Gilmour
EPI_ISL_418823 hCoV-19/Canada/BC_3989992/2020 North America / Canada / British Columbia 2020-03-09 BCCDC Public Health Laboratory BCCDC Public Health Laboratory Harrigan, Prystajecky, Krajden, Lee, Kamelian, Lapointe, Choi, Hoang, Sekirov, Levett, Tyson, Snutch, Loman, Quick, Li, Gilmour
EPI_ISL_418824 hCoV-19/Canada/BC_4078583/2020 North America / Canada / British Columbia 2020-03-03 BCCDC Public Health Laboratory BCCDC Public Health Laboratory Harrigan, Prystajecky, Krajden, Lee, Kamelian, Lapointe, Choi, Hoang, Sekirov, Levett, Tyson, Snutch, Loman, Quick, Li, Gilmour
EPI_ISL_418825 hCoV-19/Canada/BC_4118226/2020 North America / Canada / British Columbia 2020-03-10 BCCDC Public Health Laboratory BCCDC Public Health Laboratory Harrigan, Prystajecky, Krajden, Lee, Kamelian, Lapointe, Choi, Hoang, Sekirov, Levett, Tyson, Snutch, Loman, Quick, Li, Gilmour
EPI_ISL_418826 hCoV-19/Canada/BC_4122951/2020 North America / Canada / British Columbia 2020-03-10 BCCDC Public Health Laboratory BCCDC Public Health Laboratory Harrigan, Prystajecky, Krajden, Lee, Kamelian, Lapointe, Choi, Hoang, Sekirov, Levett, Tyson, Snutch, Loman, Quick, Li, Gilmour
EPI_ISL_418829 hCoV-19/Canada/BC_4540462/2020 North America / Canada / British Columbia 2020-03-10 BCCDC Public Health Laboratory BCCDC Public Health Laboratory Harrigan, Prystajecky, Krajden, Lee, Kamelian,

Lapointe, Choi, Hoang, Sekirov, Levett, Tyson, Snutch, Loman, Quick, Li, Gilmour
EPI_ISL_418830 hCoV-19/Canada/BC_4799711/2020 North America / Canada / British Columbia 2020-03-09 BCCDC Public Health Laboratory BCCDC Public Health Laboratory Harrigan, Prystajecky, Krajden, Lee, Kamelian, Lapointe, Choi, Hoang, Sekirov, Levett, Tyson, Snutch, Loman, Quick, Li, Gilmour
EPI_ISL_418831 hCoV-19/Canada/BC_5275718/2020 North America / Canada / British Columbia 2020-03-11 BCCDC Public Health Laboratory BCCDC Public Health Laboratory Harrigan, Prystajecky, Krajden, Lee, Kamelian, Lapointe, Choi, Hoang, Sekirov, Levett, Tyson, Snutch, Loman, Quick, Li, Gilmour
EPI_ISL_418832 hCoV-19/Canada/BC_5282984/2020 North America / Canada / British Columbia 2020-03-11 BCCDC Public Health Laboratory BCCDC Public Health Laboratory Harrigan, Prystajecky, Krajden, Lee, Kamelian, Lapointe, Choi, Hoang, Sekirov, Levett, Tyson, Snutch, Loman, Quick, Li, Gilmour
EPI_ISL_418833 hCoV-19/Canada/BC_5306970/2020 North America / Canada / British Columbia 2020-03-11 BCCDC Public Health Laboratory BCCDC Public Health Laboratory Harrigan, Prystajecky, Krajden, Lee, Kamelian, Lapointe, Choi, Hoang, Sekirov, Levett, Tyson, Snutch, Loman, Quick, Li, Gilmour
EPI_ISL_418834 hCoV-19/Canada/BC_5522039/2020 North America / Canada / British Columbia 2020-03-11 BCCDC Public Health Laboratory BCCDC Public Health Laboratory Harrigan, Prystajecky, Krajden, Lee, Kamelian, Lapointe, Choi, Hoang, Sekirov, Levett, Tyson, Snutch, Loman, Quick, Li, Gilmour
EPI_ISL_418835 hCoV-19/Canada/BC_5979789/2020 North America / Canada / British Columbia 2020-03-11 BCCDC Public Health Laboratory BCCDC Public Health Laboratory Harrigan, Prystajecky, Krajden, Lee, Kamelian, Lapointe, Choi, Hoang, Sekirov, Levett, Tyson, Snutch, Loman, Quick, Li, Gilmour
EPI_ISL_418836 hCoV-19/Canada/BC_6004567/2020 North America / Canada / British Columbia 2020-03-10 BCCDC Public Health Laboratory BCCDC Public Health Laboratory Harrigan, Prystajecky, Krajden, Lee, Kamelian, Lapointe, Choi, Hoang, Sekirov, Levett, Tyson, Snutch, Loman, Quick, Li, Gilmour
EPI_ISL_418837 hCoV-19/Canada/BC_6129127/2020 North America / Canada / British Columbia 2020-03-10 BCCDC Public Health Laboratory BCCDC Public Health Laboratory Harrigan, Prystajecky, Krajden, Lee, Kamelian, Lapointe, Choi, Hoang, Sekirov, Levett, Tyson, Snutch, Loman, Quick, Li, Gilmour
EPI_ISL_418860 hCoV-19/Spain/VH000001133/2020 Europe / Spain / Barcelona 2020-03-15 Hospital Universitari Vall d'Hebron (HUVH) - Vall d'Hebron Research Institute (VHIR) Hospital Universitari Vall d'Hebron (HUVH) - Vall d'Hebron Research Institute (VHIR) Cristina Andrés, Dàmir Garcia-Cehic, Maria Piñana, Mercedes Guerrero-Murillo, Ariadna Rando, Tomàs Pumarola, Maria Gema Codina, Andrés Antón, Josep Quer
EPI_ISL_418861 hCoV-19/Spain/VH198152683/2020 Europe / Spain / Barcelona 2020-03-24 Hospital Universitari Vall d'Hebron (HUVH) - Vall d'Hebron Research Institute (VHIR) Hospital Universitari Vall d'Hebron (HUVH) - Vall d'Hebron Research Institute (VHIR) Cristina Andrés, Dàmir Garcia-Cehic, Maria Piñana, Mercedes Guerrero-Murillo, Ariadna Rando, Tomàs Pumarola, Maria Gema Codina, Andrés Antón, Josep Quer
EPI_ISL_418863 hCoV-19/Belgium/VS-030542/2020 Europe / Belgium / Komen 2020-03-05 KU Leuven, Clinical and Epidemiological Virology KU Leuven, Clinical and Epidemiological Virology Bert Vanmechelen, Joan Marti-Carreras, Tony Wawina, Piet Maes
EPI_ISL_418864 hCoV-19/USA/VA-DCLS-0001/2020 North America / USA / Virginia 2020-03-17 Virginia DCLS Virginia DCLS Virginia DCLS
EPI_ISL_418866 hCoV-19/USA/WA-UW298/2020 North America / USA / Washington 2020-03-13 UW Virology Lab UW Virology Lab Pavitra Roychoudhury, Hong Xie, Keith Jerome, Alexander Greninger
EPI_ISL_418867 hCoV-19/USA/WA-UW299/2020 North America / USA / Washington 2020-03-13 UW Virology Lab UW Virology Lab Pavitra Roychoudhury, Hong Xie, Keith Jerome, Alexander Greninger
EPI_ISL_418868 hCoV-19/USA/WA-UW300/2020 North America / USA / Washington 2020-03-13 UW Virology Lab UW Virology Lab Pavitra Roychoudhury, Hong Xie, Keith Jerome, Alexander Greninger
EPI_ISL_418869 hCoV-19/USA/WA-UW301/2020 North America / USA / Washington 2020-03-16 UW Virology Lab UW Virology Lab Pavitra Roychoudhury, Hong Xie, Keith Jerome, Alexander Greninger
EPI_ISL_418870 hCoV-19/USA/WA-UW302/2020 North America / USA / Washington 2020-03-16 UW Virology Lab UW Virology Lab Pavitra Roychoudhury, Hong Xie, Keith Jerome, Alexander Greninger
EPI_ISL_418871 hCoV-19/USA/CT-UW303/2020 North America / USA / Connecticut 2020-03-14 UW Virology Lab UW Virology Lab Pavitra Roychoudhury, Hong Xie, Keith Jerome, Alexander Greninger
EPI_ISL_418872 hCoV-19/USA/WA-UW304/2020 North America / USA / Washington 2020-03-16 UW Virology Lab UW Virology Lab Pavitra Roychoudhury, Hong Xie, Keith Jerome, Alexander Greninger
EPI_ISL_418873 hCoV-19/USA/WA-UW305/2020 North America / USA / Washington 2020-03-16 UW Virology Lab UW Virology Lab Pavitra Roychoudhury, Hong Xie, Keith Jerome, Alexander Greninger
EPI_ISL_418875 hCoV-19/USA/WA-UW307/2020 North America / USA / Washington 2020-03-16 UW Virology Lab UW Virology Lab Pavitra Roychoudhury, Hong Xie, Keith Jerome, Alexander Greninger
EPI_ISL_418876 hCoV-19/USA/WA-UW308/2020 North America / USA / Washington 2020-03-16 UW Virology Lab UW Virology Lab Pavitra Roychoudhury, Hong Xie, Keith Jerome, Alexander Greninger
EPI_ISL_418877 hCoV-19/USA/WA-UW309/2020 North America / USA / Washington 2020-03-24 UW Virology Lab UW Virology Lab Pavitra Roychoudhury, Hong Xie, Keith Jerome, Alexander Greninger
EPI_ISL_418878 hCoV-19/USA/WA-UW310/2020 North America / USA / Washington 2020-03-14 UW Virology Lab UW Virology Lab Pavitra Roychoudhury, Hong Xie, Keith Jerome, Alexander Greninger
EPI_ISL_418880 hCoV-19/USA/WA-UW313/2020 North America / USA / Washington 2020-03-14 UW Virology Lab UW Virology Lab Pavitra Roychoudhury, Hong Xie, Keith Jerome, Alexander Greninger
EPI_ISL_418881 hCoV-19/USA/WA-UW314/2020 North America / USA / Washington 2020-03-14 UW Virology Lab UW Virology Lab Pavitra Roychoudhury, Hong Xie, Keith Jerome, Alexander Greninger
EPI_ISL_418882 hCoV-19/USA/WA-UW315/2020 North America / USA / Washington 2020-03-13 UW Virology Lab UW Virology Lab Pavitra Roychoudhury, Hong Xie, Keith Jerome, Alexander Greninger
EPI_ISL_418883 hCoV-19/USA/WA-UW316/2020 North America / USA / Washington 2020-03-14 UW Virology Lab UW Virology Lab Pavitra Roychoudhury, Hong Xie, Keith Jerome, Alexander Greninger

EPI_ISL_418884 hCoV-19/USA/WA-UW317/2020 North America / USA / Washington 2020-03-15 UW Virology Lab UW Virology Lab Pavitra Roychoudhury, Hong Xie, Keith Jerome, Alexander Greninger
EPI_ISL_418886 hCoV-19/USA/WA-UW319/2020 North America / USA / Washington 2020-03-15 UW Virology Lab UW Virology Lab Pavitra Roychoudhury, Hong Xie, Keith Jerome, Alexander Greninger
EPI_ISL_418887 hCoV-19/USA/WA-UW320/2020 North America / USA / Washington 2020-03-14 UW Virology Lab UW Virology Lab Pavitra Roychoudhury, Hong Xie, Keith Jerome, Alexander Greninger
EPI_ISL_418888 hCoV-19/USA/WA-UW321/2020 North America / USA / Washington 2020-03-15 UW Virology Lab UW Virology Lab Pavitra Roychoudhury, Hong Xie, Keith Jerome, Alexander Greninger
EPI_ISL_418889 hCoV-19/USA/WA-UW322/2020 North America / USA / Washington 2020-03-16 UW Virology Lab UW Virology Lab Pavitra Roychoudhury, Hong Xie, Keith Jerome, Alexander Greninger
EPI_ISL_418890 hCoV-19/USA/WA-UW323/2020 North America / USA / Washington 2020-03-16 UW Virology Lab UW Virology Lab Pavitra Roychoudhury, Hong Xie, Keith Jerome, Alexander Greninger
EPI_ISL_418891 hCoV-19/USA/WA-UW324/2020 North America / USA / Washington 2020-03-24 UW Virology Lab UW Virology Lab Pavitra Roychoudhury, Hong Xie, Keith Jerome, Alexander Greninger
EPI_ISL_418892 hCoV-19/USA/WA-UW325/2020 North America / USA / Washington 2020-03-16 UW Virology Lab UW Virology Lab Pavitra Roychoudhury, Hong Xie, Keith Jerome, Alexander Greninger
EPI_ISL_418893 hCoV-19/USA/MN-UW326/2020 North America / USA / Minnesota 2020-03-13 UW Virology Lab UW Virology Lab Pavitra Roychoudhury, Hong Xie, Keith Jerome, Alexander Greninger
EPI_ISL_418895 hCoV-19/USA/CT-UW328/2020 North America / USA / Connecticut 2020-03-14 UW Virology Lab UW Virology Lab Pavitra Roychoudhury, Hong Xie, Keith Jerome, Alexander Greninger
EPI_ISL_418897 hCoV-19/USA/WA-UW330/2020 North America / USA / Washington 2020-03-16 UW Virology Lab UW Virology Lab Pavitra Roychoudhury, Hong Xie, Keith Jerome, Alexander Greninger
EPI_ISL_418898 hCoV-19/USA/CT-UW331/2020 North America / USA / Connecticut 2020-03-14 UW Virology Lab UW Virology Lab Pavitra Roychoudhury, Hong Xie, Keith Jerome, Alexander Greninger
EPI_ISL_418899 hCoV-19/USA/WA-UW332/2020 North America / USA / Washington 2020-03-16 UW Virology Lab UW Virology Lab Pavitra Roychoudhury, Hong Xie, Keith Jerome, Alexander Greninger
EPI_ISL_419600 hCoV-19/Luxembourg/LNS9080444/2020 Europe / Luxembourg 2020-03-13 Laboratoire National de Santé, Microbiology, Virology Laboratoire National de Santé, Microbiology, Epidemiology and Microbial Genomics Anke Wienecke-Baldacchino, Ardashel Latsuzbaia, Jessica Tapp, Catherine Ragimbeau, Guillaume Fournier, Tamir Abdelrahman, Trung Nguyen Nguyen, Joel Mossong
EPI_ISL_419601 hCoV-19/Luxembourg/LNS9086704/2020 Europe / Luxembourg 2020-03-15 Laboratoire National de Santé, Microbiology, Virology Laboratoire National de Santé, Microbiology, Epidemiology and Microbial Genomics Anke Wienecke-Baldacchino, Ardashel Latsuzbaia, Jessica Tapp, Catherine Ragimbeau, Guillaume Fournier, Tamir Abdelrahman, Trung Nguyen Nguyen, Joel Mossong
EPI_ISL_419602 hCoV-19/Luxembourg/LNS9324837/2020 Europe / Luxembourg 2020-03-12 Laboratoire National de Santé, Microbiology, Virology Laboratoire National de Santé, Microbiology, Epidemiology and Microbial Genomics Anke Wienecke-Baldacchino, Ardashel Latsuzbaia, Jessica Tapp, Catherine Ragimbeau, Guillaume Fournier, Tamir Abdelrahman, Trung Nguyen Nguyen, Joel Mossong
EPI_ISL_419603 hCoV-19/Luxembourg/LNS9512434/2020 Europe / Luxembourg 2020-03-16 Laboratoire National de Santé, Microbiology, Virology Laboratoire National de Santé, Microbiology, Epidemiology and Microbial Genomics Anke Wienecke-Baldacchino, Ardashel Latsuzbaia, Jessica Tapp, Catherine Ragimbeau, Guillaume Fournier, Tamir Abdelrahman, Trung Nguyen Nguyen, Joel Mossong
EPI_ISL_419604 hCoV-19/Luxembourg/LNS9627078/2020 Europe / Luxembourg 2020-03-15 Laboratoire National de Santé, Microbiology, Virology Laboratoire National de Santé, Microbiology, Epidemiology and Microbial Genomics Anke Wienecke-Baldacchino, Ardashel Latsuzbaia, Jessica Tapp, Catherine Ragimbeau, Guillaume Fournier, Tamir Abdelrahman, Trung Nguyen Nguyen, Joel Mossong
EPI_ISL_419606 hCoV-19/Luxembourg/LNS9862689/2020 Europe / Luxembourg 2020-03-11 Laboratoire National de Santé, Microbiology, Virology Laboratoire National de Santé, Microbiology, Epidemiology and Microbial Genomics Anke Wienecke-Baldacchino, Ardashel Latsuzbaia, Jessica Tapp, Catherine Ragimbeau, Guillaume Fournier, Tamir Abdelrahman, Trung Nguyen Nguyen, Joel Mossong
EPI_ISL_419607 hCoV-19/Luxembourg/LNS9982497/2020 Europe / Luxembourg 2020-03-14 Laboratoire National de Santé, Microbiology, Virology Laboratoire National de Santé, Microbiology, Epidemiology and Microbial Genomics Anke Wienecke-Baldacchino, Ardashel Latsuzbaia, Jessica Tapp, Catherine Ragimbeau, Guillaume Fournier, Tamir Abdelrahman, Trung Nguyen Nguyen, Joel Mossong
EPI_ISL_419651 hCoV-19/USA/WI-GMF-00466/2020 North America / USA / Minnesota / Winona County 2020-03-29 Gundersen Molecular Diagnostics Laboratory Kabara Cancer Research Institute Craig S. Richmond & Paraic A. Kenny
EPI_ISL_419652 hCoV-19/USA/WI-GMF-00281/2020 North America / USA / Wisconsin / La Crosse County 2020-03-25 Gundersen Molecular Diagnostics Laboratory Kabara Cancer Research Institute Craig S. Richmond & Paraic A. Kenny
EPI_ISL_419654 hCoV-19/Austria/CeMM0001/2020 Europe / Austria 2020-03-03 Center for Virology, Medical University of Vienna Bergthaler laboratory, CeMM Research Center for Molecular Medicine of the Austrian Academy of Sciences Alexandra Popa, Benedikt Agerer, Henrique Colaco, Lukas Endler, Jakob-Wendelin Genger, Alexander Lercher, Mark Smyth, Thomas Penz, Michael Schuster, Judith Aberle, Stephan Aberle, Elisabeth Puchhammer-Stöckl, Christoph Bock, Andreas Bergthaler
EPI_ISL_419655 hCoV-19/Austria/CeMM0002/2020 Europe / Austria / Vienna 2020-02-26 Center for Virology, Medical University of Vienna Bergthaler laboratory, CeMM Research Center for Molecular Medicine of the Austrian Academy of Sciences Alexandra Popa, Benedikt Agerer, Henrique Colaco, Lukas Endler, Jakob-Wendelin Genger, Alexander Lercher, Mark Smyth, Thomas Penz, Michael Schuster, Judith Aberle, Stephan Aberle, Elisabeth Puchhammer-Stöckl, Christoph Bock, Andreas Bergthaler

EPI_ISL_419656 hCoV-19/Austria/CeMM0003/2020 Europe / Austria 2020-02-26 Center for Virology, Medical University of Vienna Bergthaler laboratory, CeMM Research Center for Molecular Medicine of the Austrian Academy of Sciences Alexandra Popa, Benedikt Agerer, Henrique Colaco, Lukas Endler, Jakob-Wendelin Genger, Alexander Lercher, Mark Smyth, Thomas Penz, Michael Schuster, Judith Aberle, Stephan Aberle, Elisabeth Puchhammer-Stöckl, Christoph Bock, Andreas Bergthaler
EPI_ISL_419658 hCoV-19/Austria/CeMM0005/2020 Europe / Austria 2020-03-06 Center for Virology, Medical University of Vienna Bergthaler laboratory, CeMM Research Center for Molecular Medicine of the Austrian Academy of Sciences Alexandra Popa, Benedikt Agerer, Henrique Colaco, Lukas Endler, Jakob-Wendelin Genger, Alexander Lercher, Mark Smyth, Thomas Penz, Michael Schuster, Judith Aberle, Stephan Aberle, Elisabeth Puchhammer-Stöckl, Christoph Bock, Andreas Bergthaler
EPI_ISL_419659 hCoV-19/Austria/CeMM0006/2020 Europe / Austria 2020-03-10 Center for Virology, Medical University of Vienna Bergthaler laboratory, CeMM Research Center for Molecular Medicine of the Austrian Academy of Sciences Alexandra Popa, Benedikt Agerer, Henrique Colaco, Lukas Endler, Jakob-Wendelin Genger, Alexander Lercher, Mark Smyth, Thomas Penz, Michael Schuster, Judith Aberle, Stephan Aberle, Elisabeth Puchhammer-Stöckl, Christoph Bock, Andreas Bergthaler
EPI_ISL_419660 hCoV-19/Austria/CeMM0007/2020 Europe / Austria 2020-03-13 Center for Virology, Medical University of Vienna Bergthaler laboratory, CeMM Research Center for Molecular Medicine of the Austrian Academy of Sciences Alexandra Popa, Benedikt Agerer, Henrique Colaco, Lukas Endler, Jakob-Wendelin Genger, Alexander Lercher, Mark Smyth, Thomas Penz, Michael Schuster, Judith Aberle, Stephan Aberle, Elisabeth Puchhammer-Stöckl, Christoph Bock, Andreas Bergthaler
EPI_ISL_419661 hCoV-19/Austria/CeMM0008/2020 Europe / Austria 2020-03-10 Center for Virology, Medical University of Vienna Bergthaler laboratory, CeMM Research Center for Molecular Medicine of the Austrian Academy of Sciences Alexandra Popa, Benedikt Agerer, Henrique Colaco, Lukas Endler, Jakob-Wendelin Genger, Alexander Lercher, Mark Smyth, Thomas Penz, Michael Schuster, Judith Aberle, Stephan Aberle, Elisabeth Puchhammer-Stöckl, Christoph Bock, Andreas Bergthaler
EPI_ISL_419662 hCoV-19/Austria/CeMM0009/2020 Europe / Austria 2020-03-14 Center for Virology, Medical University of Vienna Bergthaler laboratory, CeMM Research Center for Molecular Medicine of the Austrian Academy of Sciences Alexandra Popa, Benedikt Agerer, Henrique Colaco, Lukas Endler, Jakob-Wendelin Genger, Alexander Lercher, Mark Smyth, Thomas Penz, Michael Schuster, Judith Aberle, Stephan Aberle, Elisabeth Puchhammer-Stöckl, Christoph Bock, Andreas Bergthaler
EPI_ISL_419664 hCoV-19/Austria/CeMM0011/2020 Europe / Austria 2020-03-23 Center for Virology, Medical University of Vienna Bergthaler laboratory, CeMM Research Center for Molecular Medicine of the Austrian Academy of Sciences Alexandra Popa, Benedikt Agerer, Henrique Colaco, Lukas Endler, Jakob-Wendelin Genger, Alexander Lercher, Mark Smyth, Thomas Penz, Michael Schuster, Judith Aberle, Stephan Aberle, Elisabeth Puchhammer-Stöckl, Christoph Bock, Andreas Bergthaler
EPI_ISL_419665 hCoV-19/Austria/CeMM0012/2020 Europe / Austria 2020-03-10 Center for Virology, Medical University of Vienna Bergthaler laboratory, CeMM Research Center for Molecular Medicine of the Austrian Academy of Sciences Alexandra Popa, Benedikt Agerer, Henrique Colaco, Lukas Endler, Jakob-Wendelin Genger, Alexander Lercher, Mark Smyth, Thomas Penz, Michael Schuster, Judith Aberle, Stephan Aberle, Elisabeth Puchhammer-Stöckl, Christoph Bock, Andreas Bergthaler
EPI_ISL_419666 hCoV-19/Austria/CeMM0013/2020 Europe / Austria 2020-03-11 Center for Virology, Medical University of Vienna Bergthaler laboratory, CeMM Research Center for Molecular Medicine of the Austrian Academy of Sciences Alexandra Popa, Benedikt Agerer, Henrique Colaco, Lukas Endler, Jakob-Wendelin Genger, Alexander Lercher, Mark Smyth, Thomas Penz, Michael Schuster, Judith Aberle, Stephan Aberle, Elisabeth Puchhammer-Stöckl, Christoph Bock, Andreas Bergthaler
EPI_ISL_419667 hCoV-19/Austria/CeMM0014/2020 Europe / Austria 2020-03-13 Center for Virology, Medical University of Vienna Bergthaler laboratory, CeMM Research Center for Molecular Medicine of the Austrian Academy of Sciences Alexandra Popa, Benedikt Agerer, Henrique Colaco, Lukas Endler, Jakob-Wendelin Genger, Alexander Lercher, Mark Smyth, Thomas Penz, Michael Schuster, Judith Aberle, Stephan Aberle, Elisabeth Puchhammer-Stöckl, Christoph Bock, Andreas Bergthaler
EPI_ISL_419669 hCoV-19/Austria/CeMM0016/2020 Europe / Austria 2020-03-16 Center for Virology, Medical University of Vienna Bergthaler laboratory, CeMM Research Center for Molecular Medicine of the Austrian Academy of Sciences Alexandra Popa, Benedikt Agerer, Henrique Colaco, Lukas Endler, Jakob-Wendelin Genger, Alexander Lercher, Mark Smyth, Thomas Penz, Michael Schuster, Judith Aberle, Stephan Aberle, Elisabeth Puchhammer-Stöckl, Christoph Bock, Andreas Bergthaler
EPI_ISL_419670 hCoV-19/Austria/CeMM0017/2020 Europe / Austria 2020-03-16 Center for Virology, Medical University of Vienna Bergthaler laboratory, CeMM Research Center for Molecular Medicine of the Austrian Academy of Sciences Alexandra Popa, Benedikt Agerer, Henrique Colaco, Lukas Endler, Jakob-Wendelin Genger, Alexander Lercher, Mark Smyth, Thomas Penz, Michael Schuster, Judith Aberle, Stephan Aberle, Elisabeth Puchhammer-Stöckl, Christoph Bock, Andreas Bergthaler
EPI_ISL_419671 hCoV-19/Austria/CeMM0018/2020 Europe / Austria 2020-03-19 Center for Virology, Medical University of Vienna Bergthaler laboratory, CeMM Research Center for Molecular Medicine of the Austrian Academy of Sciences Alexandra Popa, Benedikt Agerer, Henrique Colaco, Lukas Endler, Jakob-Wendelin Genger, Alexander Lercher, Mark Smyth, Thomas Penz, Michael Schuster, Judith Aberle, Stephan Aberle, Elisabeth Puchhammer-Stöckl, Christoph Bock, Andreas Bergthaler
EPI_ISL_419672 hCoV-19/Austria/CeMM0019/2020 Europe / Austria 2020-03-19 Center for Virology, Medical University of Vienna Bergthaler laboratory, CeMM Research Center for Molecular Medicine of the Austrian Academy of Sciences Alexandra Popa, Benedikt Agerer, Henrique Colaco, Lukas Endler, Jakob-Wendelin Genger, Alexander Lercher, Mark Smyth, Thomas Penz, Michael Schuster, Judith Aberle, Stephan Aberle, Elisabeth Puchhammer-Stöckl, Christoph Bock, Andreas Bergthaler

EPI_ISL_419673 hCoV-19/Austria/CeMM0020/2020 Europe / Austria 2020-03-22 Center for Virology, Medical University of Vienna Bergthaler laboratory, CeMM Research Center for Molecular Medicine of the Austrian Academy of Sciences Alexandra Popa, Benedikt Agerer, Henrique Colaco, Lukas Endler, Jakob-Wendelin Genger, Alexander Lercher, Mark Smyth, Thomas Penz, Michael Schuster, Judith Aberle, Stephan Aberle, Elisabeth Puchhammer-Stöckl, Christoph Bock, Andreas Bergthaler
EPI_ISL_419674 hCoV-19/Austria/CeMM0021/2020 Europe / Austria 2020-03-24 Center for Virology, Medical University of Vienna Bergthaler laboratory, CeMM Research Center for Molecular Medicine of the Austrian Academy of Sciences Alexandra Popa, Benedikt Agerer, Henrique Colaco, Lukas Endler, Jakob-Wendelin Genger, Alexander Lercher, Mark Smyth, Thomas Penz, Michael Schuster, Judith Aberle, Stephan Aberle, Elisabeth Puchhammer-Stöckl, Christoph Bock, Andreas Bergthaler
EPI_ISL_419675 hCoV-19/Spain/Valencia11/2020 Europe / Spain / Comunitat Valenciana / Valencia 2020-03-20 Servicio de Microbiología. Consorcio Hospital General Universitario de Valencia Sequencing and Bioinformatics Service and Molecular Epidemiology Research Group. FISABIO-Public Health Maria Alma Bracho, Maria Dolores Ocete, Giuseppe D'Auria, Griselda De Marco, Neris Garcia-Gonzalez, Concepcion Gimeno, Fernando Gonzalez-Candelas
EPI_ISL_419676 hCoV-19/Spain/Valencia12/2020 Europe / Spain / Comunitat Valenciana / Valencia 2020-03-09 Servicio de Microbiología. Consorcio Hospital General Universitario de Valencia Sequencing and Bioinformatics Service and Molecular Epidemiology Research Group. FISABIO-Public Health Maria Dolores Ocete, Giuseppe D'Auria, Griselda De Marco, Neris Garcia-Gonzalez, Maria Alma Bracho, Concepcion Gimeno, Fernando Gonzalez-Candelas
EPI_ISL_419677 hCoV-19/Spain/Valencia13/2020 Europe / Spain / Comunitat Valenciana / Valencia 2020-03-09 Servicio de Microbiología. Consorcio Hospital General Universitario de Valencia Sequencing and Bioinformatics Service and Molecular Epidemiology Research Group. FISABIO-Public Health Giuseppe D'Auria, Griselda De Marco, Neris Garcia-Gonzalez, Maria Alma Bracho, Maria Dolores Ocete, Concepcion Gimeno, Fernando Gonzalez-Candelas
EPI_ISL_419678 hCoV-19/Spain/Valencia14/2020 Europe / Spain / Comunitat Valenciana / Valencia 2020-03-09 Servicio de Microbiología. Consorcio Hospital General Universitario de Valencia Sequencing and Bioinformatics Service and Molecular Epidemiology Research Group. FISABIO-Public Health Griselda De Marco, Neris Garcia-Gonzalez, Maria Alma Bracho, Maria Dolores Ocete, Giuseppe D'Auria, Concepcion Gimeno, Fernando Gonzalez-Candelas
EPI_ISL_419679 hCoV-19/Spain/Valencia15/2020 Europe / Spain / Comunitat Valenciana / Valencia 2020-03-02 Servicio de Microbiología. Consorcio Hospital General Universitario de Valencia Sequencing and Bioinformatics Service and Molecular Epidemiology Research Group. FISABIO-Public Health Neris Garcia-Gonzalez, Maria Alma Bracho, Maria Dolores Ocete, Giuseppe D'Auria, Griselda De Marco, Concepcion Gimeno, Fernando Gonzalez-Candelas
EPI_ISL_419680 hCoV-19/Spain/Valencia16/2020 Europe / Spain / Comunitat Valenciana / Valencia 2020-03-10 Servicio de Microbiología. Consorcio Hospital General Universitario de Valencia Sequencing and Bioinformatics Service and Molecular Epidemiology Research Group. FISABIO-Public Health Maria Alma Bracho, Maria Dolores Ocete, Giuseppe D'Auria, Griselda De Marco, Neris Garcia-Gonzalez, Concepcion Gimeno, Fernando Gonzalez-Candelas
EPI_ISL_419681 hCoV-19/Spain/Valencia17/2020 Europe / Spain / Comunitat Valenciana / Valencia 2020-03-10 Servicio de Microbiología. Consorcio Hospital General Universitario de Valencia Sequencing and Bioinformatics Service and Molecular Epidemiology Research Group. FISABIO-Public Health Maria Dolores Ocete, Giuseppe D'Auria, Griselda De Marco, Neris Garcia-Gonzalez, Maria Alma Bracho, Concepcion Gimeno, Fernando Gonzalez-Candelas
EPI_ISL_419682 hCoV-19/Spain/Valencia18/2020 Europe / Spain / Comunitat Valenciana / Valencia 2020-03-10 Servicio de Microbiología. Consorcio Hospital General Universitario de Valencia Sequencing and Bioinformatics Service and Molecular Epidemiology Research Group. FISABIO-Public Health Giuseppe D'Auria, Griselda De Marco, Neris Garcia-Gonzalez, Maria Alma Bracho, Maria Dolores Ocete, Concepcion Gimeno, Fernando Gonzalez-Candelas
EPI_ISL_419683 hCoV-19/Spain/Valencia19/2020 Europe / Spain / Comunitat Valenciana / Valencia 2020-03-08 Servicio de Microbiología. Consorcio Hospital General Universitario de Valencia Sequencing and Bioinformatics Service and Molecular Epidemiology Research Group. FISABIO-Public Health Griselda De Marco, Neris Garcia-Gonzalez, Maria Alma Bracho, Maria Dolores Ocete, Giuseppe D'Auria, Concepcion Gimeno, Fernando Gonzalez-Candelas
EPI_ISL_419691 hCoV-19/Latvia/ChVir2025/2020 Europe / Latvia / Riga 2020-03 E. Gulbja Laboratorija Charité Universitätsmedizin Berlin, Institute of Virology Victor M Corman, Julia Schneider, Barbara Mühlemann, Talitha Veith, Jörn Beheim-Schwarzbach, Terry Jones, Dr. Didzis Gavars, Mikus Gavars, Dmitrijs Perminovs, Christian Drosten
EPI_ISL_419692 hCoV-19/Belarus/ChVir2072/2020 Europe / Belarus 2020-03 The Republican Research and Practical Center for Epidemiology and Microbiology Charité Universitätsmedizin Berlin, Institute of Virology Victor M Corman, Julia Schneider, Barbara Mühlemann, Talitha Veith, Jörn Beheim-Schwarzbach, Terry Jones, Natallia Shmialiova, Natallia Sivets, Christian Drosten
EPI_ISL_419693 hCoV-19/Belarus/ChVir2073/2020 Europe / Belarus 2020-03 The Republican Research and Practical Center for Epidemiology and Microbiology Charité Universitätsmedizin Berlin, Institute of Virology Victor M Corman, Julia Schneider, Barbara Mühlemann, Talitha Veith, Jörn Beheim-Schwarzbach, Terry Jones, Natallia Shmialiova, Natallia Sivets, Christian Drosten
EPI_ISL_419696 hCoV-19/USA/NY-NYUMC35/2020 North America / USA / New York / Manhattan 2020-03-18 NYU Langone Health Departments of Pathology and Medicine, New York University School of Medicine Maria Aguero-Rosenfeld, Margaret Black, John Cadley, Paolo Cotzia, John Chen, Dacia Dimartino, Xiaojun Feng, Adriana

Heguy, Megan Hogan, Emily Huang, George Jour, Christian Marier, Matthew T. Maurano, Mark J. Mulligan, Peter Meyn, Jared Pinnell, Sitharam Ramaswami, Amy Rapkiewicz, Marie Samanovic-Golden, Antonio Serrano, Guomiao Shen, Matija Snuderl, Nick Vulpescu, Gael Westby, Paul Zappile, Yutong Zhang
EPI_ISL_419697 hCoV-19/USA/NY-NYUMC36/2020 North America / USA / New York / Manhattan 2020-03-18 NYU Langone Health Departments of Pathology and Medicine, New York University School of Medicine Maria Aguero-Rosenfeld, Margaret Black, John Cadley, Paolo Cotzia, John Chen, Dacia Dimartino, Xiaojun Feng, Adriana Heguy, Megan Hogan, Emily Huang, George Jour, Christian Marier, Matthew T. Maurano, Mark J. Mulligan, Peter Meyn, Jared Pinnell, Sitharam Ramaswami, Amy Rapkiewicz, Marie Samanovic-Golden, Antonio Serrano, Guomiao Shen, Matija Snuderl, Nick Vulpescu, Gael Westby, Paul Zappile, Yutong Zhang
EPI_ISL_419698 hCoV-19/USA/NY-NYUMC37/2020 North America / USA / New York / Manhattan 2020-03-18 NYU Langone Health Departments of Pathology and Medicine, New York University School of Medicine Maria Aguero-Rosenfeld, Margaret Black, John Cadley, Paolo Cotzia, John Chen, Dacia Dimartino, Xiaojun Feng, Adriana Heguy, Megan Hogan, Emily Huang, George Jour, Christian Marier, Matthew T. Maurano, Mark J. Mulligan, Peter Meyn, Jared Pinnell, Sitharam Ramaswami, Amy Rapkiewicz, Marie Samanovic-Golden, Antonio Serrano, Guomiao Shen, Matija Snuderl, Nick Vulpescu, Gael Westby, Paul Zappile, Yutong Zhang
EPI_ISL_420600 hCoV-19/Argentina/C121/2020 South America / Argentina 2020-03-07 Servicio Virosis Respiratorias-Departamento Virología-INEI Instituto Nacional Enfermedades Infecciosas C.G.Malbran Baumeister E., Avaro M., Benedetti E., Russo M., Dattero ME, Pontoriero A., Cisterna D., Molina V., Perandones C., Tuduri E., Lorenzo F., Poklepovich T., Campos J.
EPI_ISL_420604 hCoV-19/France/ARA12371/2020 Europe / France / ARA / Lyon 2020-03-23 Institut des Agents Infectieux (IAI), Hospices Civils de Lyon CNR Virus des Infections Respiratoires - France SUD Antonin Bal, Gregory Destras, Gwendolyne Burfin, Solenne Brun, Carine Moustaud, Raphaelle Lamy, Alexandre Gaymard, Maude Bouscambert-Duchamp, Florence Morfin-Sherpa, Martine Valette, Bruno Lina, Laurence Josset
EPI_ISL_420605 hCoV-19/France/ARA12384/2020 Europe / France / ARA / Lyon 2020-03-22 Institut des Agents Infectieux (IAI), Hospices Civils de Lyon CNR Virus des Infections Respiratoires - France SUD Antonin Bal, Gregory Destras, Gwendolyne Burfin, Solenne Brun, Carine Moustaud, Raphaelle Lamy, Alexandre Gaymard, Maude Bouscambert-Duchamp, Florence Morfin-Sherpa, Martine Valette, Bruno Lina, Laurence Josset
EPI_ISL_420606 hCoV-19/France/ARA12388/2020 Europe / France / ARA / Lyon 2020-03-22 Institut des Agents Infectieux (IAI), Hospices Civils de Lyon CNR Virus des Infections Respiratoires - France SUD Antonin Bal, Gregory Destras, Gwendolyne Burfin, Solenne Brun, Carine Moustaud, Raphaelle Lamy, Alexandre Gaymard, Maude Bouscambert-Duchamp, Florence Morfin-Sherpa, Martine Valette, Bruno Lina, Laurence Josset
EPI_ISL_420607 hCoV-19/France/ARA12485/2020 Europe / France / ARA / Lyon 2020-03-23 Institut des Agents Infectieux (IAI), Hospices Civils de Lyon CNR Virus des Infections Respiratoires - France SUD Antonin Bal, Gregory Destras, Gwendolyne Burfin, Solenne Brun, Carine Moustaud, Raphaelle Lamy, Alexandre Gaymard, Maude Bouscambert-Duchamp, Florence Morfin-Sherpa, Martine Valette, Bruno Lina, Laurence Josset
EPI_ISL_420608 hCoV-19/France/ARA12499/2020 Europe / France / ARA / Lyon 2020-03-23 Institut des Agents Infectieux (IAI), Hospices Civils de Lyon CNR Virus des Infections Respiratoires - France SUD Antonin Bal, Gregory Destras, Gwendolyne Burfin, Solenne Brun, Carine Moustaud, Raphaelle Lamy, Alexandre Gaymard, Maude Bouscambert-Duchamp, Florence Morfin-Sherpa, Martine Valette, Bruno Lina, Laurence Josset
EPI_ISL_420609 hCoV-19/France/ARA12524/2020 Europe / France / ARA / Lyon 2020-03-23 Institut des Agents Infectieux (IAI), Hospices Civils de Lyon CNR Virus des Infections Respiratoires - France SUD Antonin Bal, Gregory Destras, Gwendolyne Burfin, Solenne Brun, Carine Moustaud, Raphaelle Lamy, Alexandre Gaymard, Maude Bouscambert-Duchamp, Florence Morfin-Sherpa, Martine Valette, Bruno Lina, Laurence Josset
EPI_ISL_420610 hCoV-19/France/ARA12558/2020 Europe / France / ARA / Lyon 2020-03-23 Institut des Agents Infectieux (IAI), Hospices Civils de Lyon CNR Virus des Infections Respiratoires - France SUD Antonin Bal, Gregory Destras, Gwendolyne Burfin, Solenne Brun, Carine Moustaud, Raphaelle Lamy, Alexandre Gaymard, Maude Bouscambert-Duchamp, Florence Morfin-Sherpa, Martine Valette, Bruno Lina, Laurence Josset
EPI_ISL_420611 hCoV-19/France/ARA12576/2020 Europe / France / ARA / Lyon 2020-03-23 Institut des Agents Infectieux (IAI), Hospices Civils de Lyon CNR Virus des Infections Respiratoires - France SUD Antonin Bal, Gregory Destras, Gwendolyne Burfin, Solenne Brun, Carine Moustaud, Raphaelle Lamy, Alexandre Gaymard, Maude Bouscambert-Duchamp, Florence Morfin-Sherpa, Martine Valette, Bruno Lina, Laurence Josset
EPI_ISL_420613 hCoV-19/France/ARA12630/2020 Europe / France / ARA / Macon 2020-03-23 Centre Hospitalier de Macon CNR Virus des Infections Respiratoires - France SUD Antonin Bal, Gregory Destras, Gwendolyne Burfin, Solenne Brun, Carine Moustaud, Raphaelle Lamy, Alexandre Gaymard, Maude Bouscambert-Duchamp, Florence Morfin-Sherpa, Martine Valette, Bruno Lina, Laurence Josset
EPI_ISL_420614 hCoV-19/France/ARA12632/2020 Europe / France / ARA / Macon 2020-03-23 Centre Hospitalier de Macon CNR Virus des Infections Respiratoires - France SUD Antonin Bal, Gregory Destras, Gwendolyne Burfin, Solenne Brun, Carine Moustaud, Raphaelle Lamy, Alexandre Gaymard, Maude Bouscambert-Duchamp, Florence Morfin-Sherpa, Martine Valette, Bruno Lina, Laurence Josset
EPI_ISL_420615 hCoV-19/France/ARA12759/2020 Europe / France / ARA / Lyon 2020-03-23 Institut des Agents Infectieux (IAI), Hospices Civils de Lyon CNR Virus des Infections Respiratoires - France SUD Antonin Bal, Gregory Destras, Gwendolyne Burfin, Solenne Brun, Carine Moustaud, Raphaelle Lamy, Alexandre Gaymard, Maude Bouscambert-Duchamp, Florence Morfin-Sherpa, Martine Valette, Bruno Lina, Laurence Josset
EPI_ISL_420616 hCoV-19/France/ARA1284/2020 Europe / France / ARA / Lyon 2020-03-23 Institut des Agents Infectieux (IAI), Hospices Civils de Lyon CNR Virus des Infections Respiratoires - France SUD Antonin Bal, Gregory Destras, Gwendolyne Burfin, Solenne Brun, Carine Moustaud, Raphaelle Lamy, Alexandre Gaymard, Maude Bouscambert-Duchamp, Florence Morfin-Sherpa, Martine Valette, Bruno Lina, Laurence Josset
EPI_ISL_420617 hCoV-19/France/ARA12877/2020 Europe / France / ARA / Lyon 2020-03-23 Centre Hospitalier Saint Joseph Saint Luc CNR Virus des Infections Respiratoires - France SUD Antonin Bal, Gregory Destras, Gwendolyne Burfin, Solenne Brun, Carine Moustaud, Raphaelle Lamy, Alexandre Gaymard, Maude

Bouscambert-Duchamp, Florence Morfin-Sherpa, Martine Valette, Bruno Lina, Laurence Josset
EPI_ISL_420618 hCoV-19/France/ARA12915/2020 Europe / France / ARA / Lyon 2020-03-23 Institut des Agents Infectieux (IAI), Hospices Civils de Lyon CNR Virus des Infections Respiratoires - France SUD Antonin Bal, Gregory Destras, Gwendolyne Burfin, Solenne Brun, Carine Moustaud, Raphaelle Lamy, Alexandre Gaymard, Maude Bouscambert-Duchamp, Florence Morfin-Sherpa, Martine Valette, Bruno Lina, Laurence Josset
EPI_ISL_420619 hCoV-19/France/ARA12973/2020 Europe / France / ARA / Lyon 2020-03-23 Institut des Agents Infectieux (IAI), Hospices Civils de Lyon CNR Virus des Infections Respiratoires - France SUD Antonin Bal, Gregory Destras, Gwendolyne Burfin, Solenne Brun, Carine Moustaud, Raphaelle Lamy, Alexandre Gaymard, Maude Bouscambert-Duchamp, Florence Morfin-Sherpa, Martine Valette, Bruno Lina, Laurence Josset
EPI_ISL_420620 hCoV-19/France/ARA12996/2020 Europe / France / ARA / Bourg-en-Bresse 2020-03-23 Centre Hospitalier de Bourg en Bresse CNR Virus des Infections Respiratoires - France SUD Antonin Bal, Gregory Destras, Gwendolyne Burfin, Solenne Brun, Carine Moustaud, Raphaelle Lamy, Alexandre Gaymard, Maude Bouscambert-Duchamp, Florence Morfin-Sherpa, Martine Valette, Bruno Lina, Laurence Josset
EPI_ISL_420621 hCoV-19/France/ARA1307/2020 Europe / France / ARA / Lyon 2020-03-24 Institut des Agents Infectieux (IAI), Hospices Civils de Lyon CNR Virus des Infections Respiratoires - France SUD Antonin Bal, Gregory Destras, Gwendolyne Burfin, Solenne Brun, Carine Moustaud, Raphaelle Lamy, Alexandre Gaymard, Maude Bouscambert-Duchamp, Florence Morfin-Sherpa, Martine Valette, Bruno Lina, Laurence Josset
EPI_ISL_420623 hCoV-19/France/ARA13095/2020 Europe / France / ARA / Lyon 2020-03-24 Institut des Agents Infectieux (IAI), Hospices Civils de Lyon CNR Virus des Infections Respiratoires - France SUD Antonin Bal, Gregory Destras, Gwendolyne Burfin, Solenne Brun, Carine Moustaud, Raphaelle Lamy, Alexandre Gaymard, Maude Bouscambert-Duchamp, Florence Morfin-Sherpa, Martine Valette, Bruno Lina, Laurence Josset
EPI_ISL_420624 hCoV-19/France/ARA13160/2020 Europe / France / ARA / Lyon 2020-03-24 Institut des Agents Infectieux (IAI), Hospices Civils de Lyon CNR Virus des Infections Respiratoires - France SUD Antonin Bal, Gregory Destras, Gwendolyne Burfin, Solenne Brun, Carine Moustaud, Raphaelle Lamy, Alexandre Gaymard, Maude Bouscambert-Duchamp, Florence Morfin-Sherpa, Martine Valette, Bruno Lina, Laurence Josset
EPI_ISL_420625 hCoV-19/France/ARA1322/2020 Europe / France / ARA / Lyon 2020-03-24 Institut des Agents Infectieux (IAI), Hospices Civils de Lyon CNR Virus des Infections Respiratoires - France SUD Antonin Bal, Gregory Destras, Gwendolyne Burfin, Solenne Brun, Carine Moustaud, Raphaelle Lamy, Alexandre Gaymard, Maude Bouscambert-Duchamp, Florence Morfin-Sherpa, Martine Valette, Bruno Lina, Laurence Josset
EPI_ISL_420628 hCoV-19/USA/VA-DCLS-0024/2020 North America / USA / Virginia 2020-03-13 Virginia DCLS Virginia DCLS Virginia DCLS
EPI_ISL_420629 hCoV-19/USA/VA-DCLS-0034/2020 North America / USA / Virginia 2020-03-20 Virginia DCLS Virginia DCLS Virginia DCLS
EPI_ISL_420630 hCoV-19/USA/VA-DCLS-0038/2020 North America / USA / Virginia 2020-03-23 Virginia DCLS Virginia DCLS Virginia DCLS
EPI_ISL_420633 hCoV-19/England/20130055502/2020 Europe / United Kingdom / England 2020-03-19 Respiratory Virus Unit, Microbiology Services Colindale, Public Health England Respiratory Virus Unit, Microbiology Services Colindale, Public Health England Monica Galiano, Shahjahan Miah, Angie Lackenby, Omolola Akinbami, Tiina Talts, Leena Bhaw, Richard Myers, Steven Platt, Kirstin Edwards, Jonathan Hubb, Joanna Ellis, Maria Zambon
EPI_ISL_420636 hCoV-19/England/20136085404/2020 Europe / United Kingdom / England 2020-03-24 Respiratory Virus Unit, Microbiology Services Colindale, Public Health England Respiratory Virus Unit, Microbiology Services Colindale, Public Health England Monica Galiano, Shahjahan Miah, Angie Lackenby, Omolola Akinbami, Tiina Talts, Leena Bhaw, Richard Myers, Steven Platt, Kirstin Edwards, Jonathan Hubb, Joanna Ellis, Maria Zambon
EPI_ISL_420640 hCoV-19/England/20136088602/2020 Europe / United Kingdom / England 2020-03-23 Respiratory Virus Unit, Microbiology Services Colindale, Public Health England Respiratory Virus Unit, Microbiology Services Colindale, Public Health England Monica Galiano, Shahjahan Miah, Angie Lackenby, Omolola Akinbami, Tiina Talts, Leena Bhaw, Richard Myers, Steven Platt, Kirstin Edwards, Jonathan Hubb, Joanna Ellis, Maria Zambon
EPI_ISL_420642 hCoV-19/England/20136089402/2020 Europe / United Kingdom / England 2020-03-23 Respiratory Virus Unit, Microbiology Services Colindale, Public Health England Respiratory Virus Unit, Microbiology Services Colindale, Public Health England Monica Galiano, Shahjahan Miah, Angie Lackenby, Omolola Akinbami, Tiina Talts, Leena Bhaw, Richard Myers, Steven Platt, Kirstin Edwards, Jonathan Hubb, Joanna Ellis, Maria Zambon
EPI_ISL_420643 hCoV-19/England/20136094704/2020 Europe / United Kingdom / England 2020-03-26 Respiratory Virus Unit, Microbiology Services Colindale, Public Health England Respiratory Virus Unit, Microbiology Services Colindale, Public Health England Monica Galiano, Shahjahan Miah, Angie Lackenby, Omolola Akinbami, Tiina Talts, Leena Bhaw, Richard Myers, Steven Platt, Kirstin Edwards, Jonathan Hubb, Joanna Ellis, Maria Zambon
EPI_ISL_420648 hCoV-19/England/20136096504/2020 Europe / United Kingdom / England 2020-03-23 Respiratory Virus Unit, Microbiology Services Colindale, Public Health England Respiratory Virus Unit, Microbiology Services Colindale, Public Health England Monica Galiano, Shahjahan Miah, Angie Lackenby, Omolola Akinbami, Tiina Talts, Leena Bhaw, Richard Myers, Steven Platt, Kirstin Edwards, Jonathan Hubb, Joanna Ellis, Maria Zambon
EPI_ISL_420652 hCoV-19/England/20136097204/2020 Europe / United Kingdom / England 2020-03-23 Respiratory Virus Unit, Microbiology Services Colindale, Public Health England Respiratory Virus Unit, Microbiology Services Colindale, Public Health England Monica Galiano, Shahjahan Miah, Angie Lackenby, Omolola Akinbami, Tiina Talts, Leena Bhaw, Richard Myers, Steven Platt, Kirstin Edwards, Jonathan Hubb, Joanna Ellis, Maria Zambon

EPI_ISL_420661 hCoV-19/England/20139013406/2020   Europe / United Kingdom / England   2020-03-27
Respiratory Virus Unit, Microbiology Services Colindale, Public Health England Respiratory Virus Unit, Microbiology Services Colindale, Public Health England Monica Galiano, Shahjahan Miah, Angie Lackenby, Omolola Akinbami, Tiina Talts, Leena Bhaw, Richard Myers, Steven Platt, Kirstin Edwards, Jonathan Hubb, Joanna Ellis, Maria Zambon
EPI_ISL_420670 hCoV-19/England/20139037504/2020   Europe / United Kingdom / England   2020-03-27
Respiratory Virus Unit, Microbiology Services Colindale, Public Health England Respiratory Virus Unit, Microbiology Services Colindale, Public Health England Monica Galiano, Shahjahan Miah, Angie Lackenby, Omolola Akinbami, Tiina Talts, Leena Bhaw, Richard Myers, Steven Platt, Kirstin Edwards, Jonathan Hubb, Joanna Ellis, Maria Zambon
EPI_ISL_420683 hCoV-19/England/20139044504/2020   Europe / United Kingdom / England   2020-03-28
Respiratory Virus Unit, Microbiology Services Colindale, Public Health England Respiratory Virus Unit, Microbiology Services Colindale, Public Health England Monica Galiano, Shahjahan Miah, Angie Lackenby, Omolola Akinbami, Tiina Talts, Leena Bhaw, Richard Myers, Steven Platt, Kirstin Edwards, Jonathan Hubb, Joanna Ellis, Maria Zambon
EPI_ISL_420686 hCoV-19/England/20139045804/2020   Europe / United Kingdom / England   2020-03-28
Respiratory Virus Unit, Microbiology Services Colindale, Public Health England Respiratory Virus Unit, Microbiology Services Colindale, Public Health England Monica Galiano, Shahjahan Miah, Angie Lackenby, Omolola Akinbami, Tiina Talts, Leena Bhaw, Richard Myers, Steven Platt, Kirstin Edwards, Jonathan Hubb, Joanna Ellis, Maria Zambon
EPI_ISL_420687 hCoV-19/England/20139045904/2020   Europe / United Kingdom / England   2020-03-28
Respiratory Virus Unit, Microbiology Services Colindale, Public Health England Respiratory Virus Unit, Microbiology Services Colindale, Public Health England Monica Galiano, Shahjahan Miah, Angie Lackenby, Omolola Akinbami, Tiina Talts, Leena Bhaw, Richard Myers, Steven Platt, Kirstin Edwards, Jonathan Hubb, Joanna Ellis, Maria Zambon
EPI_ISL_420688 hCoV-19/England/20139046504/2020   Europe / United Kingdom / England   2020-03-28
Respiratory Virus Unit, Microbiology Services Colindale, Public Health England Respiratory Virus Unit, Microbiology Services Colindale, Public Health England Monica Galiano, Shahjahan Miah, Angie Lackenby, Omolola Akinbami, Tiina Talts, Leena Bhaw, Richard Myers, Steven Platt, Kirstin Edwards, Jonathan Hubb, Joanna Ellis, Maria Zambon
EPI_ISL_420689 hCoV-19/England/20139046604/2020   Europe / United Kingdom / England   2020-03-28
Respiratory Virus Unit, Microbiology Services Colindale, Public Health England Respiratory Virus Unit, Microbiology Services Colindale, Public Health England Monica Galiano, Shahjahan Miah, Angie Lackenby, Omolola Akinbami, Tiina Talts, Leena Bhaw, Richard Myers, Steven Platt, Kirstin Edwards, Jonathan Hubb, Joanna Ellis, Maria Zambon
EPI_ISL_420690 hCoV-19/England/20139046704/2020   Europe / United Kingdom / England   2020-03-28
Respiratory Virus Unit, Microbiology Services Colindale, Public Health England Respiratory Virus Unit, Microbiology Services Colindale, Public Health England Monica Galiano, Shahjahan Miah, Angie Lackenby, Omolola Akinbami, Tiina Talts, Leena Bhaw, Richard Myers, Steven Platt, Kirstin Edwards, Jonathan Hubb, Joanna Ellis, Maria Zambon
EPI_ISL_420692 hCoV-19/England/20139047104/2020   Europe / United Kingdom / England   2020-03-29
Respiratory Virus Unit, Microbiology Services Colindale, Public Health England Respiratory Virus Unit, Microbiology Services Colindale, Public Health England Monica Galiano, Shahjahan Miah, Angie Lackenby, Omolola Akinbami, Tiina Talts, Leena Bhaw, Richard Myers, Steven Platt, Kirstin Edwards, Jonathan Hubb, Joanna Ellis, Maria Zambon
EPI_ISL_420693 hCoV-19/England/20139047404/2020   Europe / United Kingdom / England   2020-03-28
Respiratory Virus Unit, Microbiology Services Colindale, Public Health England Respiratory Virus Unit, Microbiology Services Colindale, Public Health England Monica Galiano, Shahjahan Miah, Angie Lackenby, Omolola Akinbami, Tiina Talts, Leena Bhaw, Richard Myers, Steven Platt, Kirstin Edwards, Jonathan Hubb, Joanna Ellis, Maria Zambon
EPI_ISL_420694 hCoV-19/England/20139047504/2020   Europe / United Kingdom / England   2020-03-28
Respiratory Virus Unit, Microbiology Services Colindale, Public Health England Respiratory Virus Unit, Microbiology Services Colindale, Public Health England Monica Galiano, Shahjahan Miah, Angie Lackenby, Omolola Akinbami, Tiina Talts, Leena Bhaw, Richard Myers, Steven Platt, Kirstin Edwards, Jonathan Hubb, Joanna Ellis, Maria Zambon
EPI_ISL_420697 hCoV-19/England/20139047804/2020   Europe / United Kingdom / England   2020-03-29
Respiratory Virus Unit, Microbiology Services Colindale, Public Health England Respiratory Virus Unit, Microbiology Services Colindale, Public Health England Monica Galiano, Shahjahan Miah, Angie Lackenby, Omolola Akinbami, Tiina Talts, Leena Bhaw, Richard Myers, Steven Platt, Kirstin Edwards, Jonathan Hubb, Joanna Ellis, Maria Zambon
EPI_ISL_420698 hCoV-19/England/20139048804/2020   Europe / United Kingdom / England   2020-03-28
Respiratory Virus Unit, Microbiology Services Colindale, Public Health England Respiratory Virus Unit, Microbiology Services Colindale, Public Health England Monica Galiano, Shahjahan Miah, Angie Lackenby, Omolola Akinbami, Tiina Talts, Leena Bhaw, Richard Myers, Steven Platt, Kirstin Edwards, Jonathan Hubb, Joanna Ellis, Maria Zambon
EPI_ISL_421900 hCoV-19/England/20139027004/2020   Europe / United Kingdom / England   2020-03-26
Respiratory Virus Unit, Microbiology Services Colindale, Public Health England Respiratory Virus Unit, Microbiology Services Colindale, Public Health England Monica Galiano, Shahjahan Miah, Angie Lackenby, Omolola Akinbami, Tiina Talts, Leena Bhaw, Richard Myers, Steven Platt, Kirstin Edwards, Jonathan Hubb, Joanna Ellis, Maria Zambon

EPI_ISL_421903 hCoV-19/England/20139027304/2020   Europe / United Kingdom / England   2020-03-26
Respiratory Virus Unit, Microbiology Services Colindale, Public Health England Respiratory Virus Unit, Microbiology Services Colindale, Public Health England Monica Galiano, Shahjahan Miah, Angie Lackenby, Omolola Akinbami, Tiina Talts, Leena Bhaw, Richard Myers, Steven Platt, Kirstin Edwards, Jonathan Hubb, Joanna Ellis, Maria Zambon
EPI_ISL_421904 hCoV-19/England/20139027404/2020   Europe / United Kingdom / England   2020-03-26
Respiratory Virus Unit, Microbiology Services Colindale, Public Health England Respiratory Virus Unit, Microbiology Services Colindale, Public Health England Monica Galiano, Shahjahan Miah, Angie Lackenby, Omolola Akinbami, Tiina Talts, Leena Bhaw, Richard Myers, Steven Platt, Kirstin Edwards, Jonathan Hubb, Joanna Ellis, Maria Zambon
EPI_ISL_421907 hCoV-19/England/20139027904/2020   Europe / United Kingdom / England   2020-03-26
Respiratory Virus Unit, Microbiology Services Colindale, Public Health England Respiratory Virus Unit, Microbiology Services Colindale, Public Health England Monica Galiano, Shahjahan Miah, Angie Lackenby, Omolola Akinbami, Tiina Talts, Leena Bhaw, Richard Myers, Steven Platt, Kirstin Edwards, Jonathan Hubb, Joanna Ellis, Maria Zambon
EPI_ISL_421909 hCoV-19/England/20139029104/2020   Europe / United Kingdom / England   2020-03-26
Respiratory Virus Unit, Microbiology Services Colindale, Public Health England Respiratory Virus Unit, Microbiology Services Colindale, Public Health England Monica Galiano, Shahjahan Miah, Angie Lackenby, Omolola Akinbami, Tiina Talts, Leena Bhaw, Richard Myers, Steven Platt, Kirstin Edwards, Jonathan Hubb, Joanna Ellis, Maria Zambon
EPI_ISL_421910 hCoV-19/England/20139029304/2020   Europe / United Kingdom / England   2020-03-26
Respiratory Virus Unit, Microbiology Services Colindale, Public Health England Respiratory Virus Unit, Microbiology Services Colindale, Public Health England Monica Galiano, Shahjahan Miah, Angie Lackenby, Omolola Akinbami, Tiina Talts, Leena Bhaw, Richard Myers, Steven Platt, Kirstin Edwards, Jonathan Hubb, Joanna Ellis, Maria Zambon
EPI_ISL_421911 hCoV-19/England/20139029404/2020   Europe / United Kingdom / England   2020-03-26
Respiratory Virus Unit, Microbiology Services Colindale, Public Health England Respiratory Virus Unit, Microbiology Services Colindale, Public Health England Monica Galiano, Shahjahan Miah, Angie Lackenby, Omolola Akinbami, Tiina Talts, Leena Bhaw, Richard Myers, Steven Platt, Kirstin Edwards, Jonathan Hubb, Joanna Ellis, Maria Zambon
EPI_ISL_421912 hCoV-19/England/20139030404/2020   Europe / United Kingdom / England   2020-03-26
Respiratory Virus Unit, Microbiology Services Colindale, Public Health England Respiratory Virus Unit, Microbiology Services Colindale, Public Health England Monica Galiano, Shahjahan Miah, Angie Lackenby, Omolola Akinbami, Tiina Talts, Leena Bhaw, Richard Myers, Steven Platt, Kirstin Edwards, Jonathan Hubb, Joanna Ellis, Maria Zambon
EPI_ISL_421914 hCoV-19/England/20139031404/2020   Europe / United Kingdom / England   2020-03-27
Respiratory Virus Unit, Microbiology Services Colindale, Public Health England Respiratory Virus Unit, Microbiology Services Colindale, Public Health England Monica Galiano, Shahjahan Miah, Angie Lackenby, Omolola Akinbami, Tiina Talts, Leena Bhaw, Richard Myers, Steven Platt, Kirstin Edwards, Jonathan Hubb, Joanna Ellis, Maria Zambon
EPI_ISL_421915 hCoV-19/England/20139031504/2020   Europe / United Kingdom / England   2020-03-27
Respiratory Virus Unit, Microbiology Services Colindale, Public Health England Respiratory Virus Unit, Microbiology Services Colindale, Public Health England Monica Galiano, Shahjahan Miah, Angie Lackenby, Omolola Akinbami, Tiina Talts, Leena Bhaw, Richard Myers, Steven Platt, Kirstin Edwards, Jonathan Hubb, Joanna Ellis, Maria Zambon
EPI_ISL_421916 hCoV-19/England/20139031804/2020   Europe / United Kingdom / England   2020-03-27
Respiratory Virus Unit, Microbiology Services Colindale, Public Health England Respiratory Virus Unit, Microbiology Services Colindale, Public Health England Monica Galiano, Shahjahan Miah, Angie Lackenby, Omolola Akinbami, Tiina Talts, Leena Bhaw, Richard Myers, Steven Platt, Kirstin Edwards, Jonathan Hubb, Joanna Ellis, Maria Zambon
EPI_ISL_421918 hCoV-19/England/20139032404/2020   Europe / United Kingdom / England   2020-03-27
Respiratory Virus Unit, Microbiology Services Colindale, Public Health England Respiratory Virus Unit, Microbiology Services Colindale, Public Health England Monica Galiano, Shahjahan Miah, Angie Lackenby, Omolola Akinbami, Tiina Talts, Leena Bhaw, Richard Myers, Steven Platt, Kirstin Edwards, Jonathan Hubb, Joanna Ellis, Maria Zambon
EPI_ISL_421919 hCoV-19/England/20139033004/2020   Europe / United Kingdom / England   2020-03-27
Respiratory Virus Unit, Microbiology Services Colindale, Public Health England Respiratory Virus Unit, Microbiology Services Colindale, Public Health England Monica Galiano, Shahjahan Miah, Angie Lackenby, Omolola Akinbami, Tiina Talts, Leena Bhaw, Richard Myers, Steven Platt, Kirstin Edwards, Jonathan Hubb, Joanna Ellis, Maria Zambon
EPI_ISL_421920 hCoV-19/England/20139033304/2020   Europe / United Kingdom / England   2020-03-27
Respiratory Virus Unit, Microbiology Services Colindale, Public Health England Respiratory Virus Unit, Microbiology Services Colindale, Public Health England Monica Galiano, Shahjahan Miah, Angie Lackenby, Omolola Akinbami, Tiina Talts, Leena Bhaw, Richard Myers, Steven Platt, Kirstin Edwards, Jonathan Hubb, Joanna Ellis, Maria Zambon
EPI_ISL_421921 hCoV-19/England/20139033404/2020   Europe / United Kingdom / England   2020-03-27
Respiratory Virus Unit, Microbiology Services Colindale, Public Health England Respiratory Virus Unit, Microbiology Services Colindale, Public Health England Monica Galiano, Shahjahan Miah, Angie Lackenby, Omolola Akinbami, Tiina Talts, Leena Bhaw, Richard Myers, Steven Platt, Kirstin Edwards, Jonathan Hubb, Joanna Ellis, Maria Zambon

EPI_ISL_421923 hCoV-19/England/20139036504/2020   Europe / United Kingdom / England   2020-03-27
Respiratory Virus Unit, Microbiology Services Colindale, Public Health England Respiratory Virus Unit, Microbiology Services Colindale, Public Health England Monica Galiano, Shahjahan Miah, Angie Lackenby, Omolola Akinbami, Tiina Talts, Leena Bhaw, Richard Myers, Steven Platt, Kirstin Edwards, Jonathan Hubb, Joanna Ellis, Maria Zambon
EPI_ISL_421932 hCoV-19/England/20144013504/2020   Europe / United Kingdom / England   2020-03-31
Respiratory Virus Unit, Microbiology Services Colindale, Public Health England Respiratory Virus Unit, Microbiology Services Colindale, Public Health England Monica Galiano, Shahjahan Miah, Angie Lackenby, Omolola Akinbami, Tiina Talts, Leena Bhaw, Richard Myers, Steven Platt, Kirstin Edwards, Jonathan Hubb, Joanna Ellis, Maria Zambon
EPI_ISL_421935 hCoV-19/England/20144014604/2020   Europe / United Kingdom / England   2020-03-31
Respiratory Virus Unit, Microbiology Services Colindale, Public Health England Respiratory Virus Unit, Microbiology Services Colindale, Public Health England Monica Galiano, Shahjahan Miah, Angie Lackenby, Omolola Akinbami, Tiina Talts, Leena Bhaw, Richard Myers, Steven Platt, Kirstin Edwards, Jonathan Hubb, Joanna Ellis, Maria Zambon
EPI_ISL_421936 hCoV-19/England/20144014704/2020   Europe / United Kingdom / England   2020-03-31
Respiratory Virus Unit, Microbiology Services Colindale, Public Health England Respiratory Virus Unit, Microbiology Services Colindale, Public Health England Monica Galiano, Shahjahan Miah, Angie Lackenby, Omolola Akinbami, Tiina Talts, Leena Bhaw, Richard Myers, Steven Platt, Kirstin Edwards, Jonathan Hubb, Joanna Ellis, Maria Zambon
EPI_ISL_421937 hCoV-19/England/20144016604/2020   Europe / United Kingdom / England   2020-03-30
Respiratory Virus Unit, Microbiology Services Colindale, Public Health England Respiratory Virus Unit, Microbiology Services Colindale, Public Health England Monica Galiano, Shahjahan Miah, Angie Lackenby, Omolola Akinbami, Tiina Talts, Leena Bhaw, Richard Myers, Steven Platt, Kirstin Edwards, Jonathan Hubb, Joanna Ellis, Maria Zambon
EPI_ISL_421938 hCoV-19/England/20144018204/2020   Europe / United Kingdom / England   2020-03-30
Respiratory Virus Unit, Microbiology Services Colindale, Public Health England Respiratory Virus Unit, Microbiology Services Colindale, Public Health England Monica Galiano, Shahjahan Miah, Angie Lackenby, Omolola Akinbami, Tiina Talts, Leena Bhaw, Richard Myers, Steven Platt, Kirstin Edwards, Jonathan Hubb, Joanna Ellis, Maria Zambon
EPI_ISL_421939 hCoV-19/England/20144018704/2020   Europe / United Kingdom / England   2020-04-01
Respiratory Virus Unit, Microbiology Services Colindale, Public Health England Respiratory Virus Unit, Microbiology Services Colindale, Public Health England Monica Galiano, Shahjahan Miah, Angie Lackenby, Omolola Akinbami, Tiina Talts, Leena Bhaw, Richard Myers, Steven Platt, Kirstin Edwards, Jonathan Hubb, Joanna Ellis, Maria Zambon
EPI_ISL_421940 hCoV-19/England/20144019304/2020   Europe / United Kingdom / England   2020-03-30
Respiratory Virus Unit, Microbiology Services Colindale, Public Health England Respiratory Virus Unit, Microbiology Services Colindale, Public Health England Monica Galiano, Shahjahan Miah, Angie Lackenby, Omolola Akinbami, Tiina Talts, Leena Bhaw, Richard Myers, Steven Platt, Kirstin Edwards, Jonathan Hubb, Joanna Ellis, Maria Zambon
EPI_ISL_421942 hCoV-19/England/20144020004/2020   Europe / United Kingdom / England   2020-03-31
Respiratory Virus Unit, Microbiology Services Colindale, Public Health England Respiratory Virus Unit, Microbiology Services Colindale, Public Health England Monica Galiano, Shahjahan Miah, Angie Lackenby, Omolola Akinbami, Tiina Talts, Leena Bhaw, Richard Myers, Steven Platt, Kirstin Edwards, Jonathan Hubb, Joanna Ellis, Maria Zambon
EPI_ISL_421945 hCoV-19/England/20144035904/2020   Europe / United Kingdom / England   2020-03-31
Respiratory Virus Unit, Microbiology Services Colindale, Public Health England Respiratory Virus Unit, Microbiology Services Colindale, Public Health England Monica Galiano, Shahjahan Miah, Angie Lackenby, Omolola Akinbami, Tiina Talts, Leena Bhaw, Richard Myers, Steven Platt, Kirstin Edwards, Jonathan Hubb, Joanna Ellis, Maria Zambon
EPI_ISL_421946 hCoV-19/England/20144037304/2020   Europe / United Kingdom / England   2020-03-31
Respiratory Virus Unit, Microbiology Services Colindale, Public Health England Respiratory Virus Unit, Microbiology Services Colindale, Public Health England Monica Galiano, Shahjahan Miah, Angie Lackenby, Omolola Akinbami, Tiina Talts, Leena Bhaw, Richard Myers, Steven Platt, Kirstin Edwards, Jonathan Hubb, Joanna Ellis, Maria Zambon
EPI_ISL_421947 hCoV-19/England/20144038204/2020   Europe / United Kingdom / England   2020-03-31
Respiratory Virus Unit, Microbiology Services Colindale, Public Health England Respiratory Virus Unit, Microbiology Services Colindale, Public Health England Monica Galiano, Shahjahan Miah, Angie Lackenby, Omolola Akinbami, Tiina Talts, Leena Bhaw, Richard Myers, Steven Platt, Kirstin Edwards, Jonathan Hubb, Joanna Ellis, Maria Zambon
EPI_ISL_421948 hCoV-19/England/20144049404/2020   Europe / United Kingdom / England   2020-03-31
Respiratory Virus Unit, Microbiology Services Colindale, Public Health England Respiratory Virus Unit, Microbiology Services Colindale, Public Health England Monica Galiano, Shahjahan Miah, Angie Lackenby, Omolola Akinbami, Tiina Talts, Leena Bhaw, Richard Myers, Steven Platt, Kirstin Edwards, Jonathan Hubb, Joanna Ellis, Maria Zambon
EPI_ISL_421951 hCoV-19/England/20144049704/2020   Europe / United Kingdom / England   2020-03-31
Respiratory Virus Unit, Microbiology Services Colindale, Public Health England Respiratory Virus Unit, Microbiology Services Colindale, Public Health England Monica Galiano, Shahjahan Miah, Angie Lackenby, Omolola Akinbami, Tiina Talts, Leena Bhaw, Richard Myers, Steven Platt, Kirstin Edwards, Jonathan Hubb, Joanna Ellis, Maria Zambon

EPI_ISL_421956 hCoV-19/England/20144065604/2020 Europe / United Kingdom / England 2020-03-31 Respiratory Virus Unit, Microbiology Services Colindale, Public Health England Respiratory Virus Unit, Microbiology Services Colindale, Public Health England Monica Galiano, Shahjahan Miah, Angie Lackenby, Omolola Akinbami, Tiina Talts, Leena Bhaw, Richard Myers, Steven Platt, Kirstin Edwards, Jonathan Hubb, Joanna Ellis, Maria Zambon
EPI_ISL_421957 hCoV-19/England/20144067504/2020 Europe / United Kingdom / England 2020-03-20 Respiratory Virus Unit, Microbiology Services Colindale, Public Health England Respiratory Virus Unit, Microbiology Services Colindale, Public Health England Monica Galiano, Shahjahan Miah, Angie Lackenby, Omolola Akinbami, Tiina Talts, Leena Bhaw, Richard Myers, Steven Platt, Kirstin Edwards, Jonathan Hubb, Joanna Ellis, Maria Zambon
EPI_ISL_421958 hCoV-19/England/20144071004/2020 Europe / United Kingdom / England 2020-04-01 Respiratory Virus Unit, Microbiology Services Colindale, Public Health England Respiratory Virus Unit, Microbiology Services Colindale, Public Health England Monica Galiano, Shahjahan Miah, Angie Lackenby, Omolola Akinbami, Tiina Talts, Leena Bhaw, Richard Myers, Steven Platt, Kirstin Edwards, Jonathan Hubb, Joanna Ellis, Maria Zambon
EPI_ISL_421962 hCoV-19/England/20144072804/2020 Europe / United Kingdom / England 2020-04-01 Respiratory Virus Unit, Microbiology Services Colindale, Public Health England Respiratory Virus Unit, Microbiology Services Colindale, Public Health England Monica Galiano, Shahjahan Miah, Angie Lackenby, Omolola Akinbami, Tiina Talts, Leena Bhaw, Richard Myers, Steven Platt, Kirstin Edwards, Jonathan Hubb, Joanna Ellis, Maria Zambon
EPI_ISL_421963 hCoV-19/England/20144074804/2020 Europe / United Kingdom / England 2020-04-01 Respiratory Virus Unit, Microbiology Services Colindale, Public Health England Respiratory Virus Unit, Microbiology Services Colindale, Public Health England Monica Galiano, Shahjahan Miah, Angie Lackenby, Omolola Akinbami, Tiina Talts, Leena Bhaw, Richard Myers, Steven Platt, Kirstin Edwards, Jonathan Hubb, Joanna Ellis, Maria Zambon
EPI_ISL_421964 hCoV-19/England/20144076304/2020 Europe / United Kingdom / England 2020-04-01 Respiratory Virus Unit, Microbiology Services Colindale, Public Health England Respiratory Virus Unit, Microbiology Services Colindale, Public Health England Monica Galiano, Shahjahan Miah, Angie Lackenby, Omolola Akinbami, Tiina Talts, Leena Bhaw, Richard Myers, Steven Platt, Kirstin Edwards, Jonathan Hubb, Joanna Ellis, Maria Zambon
EPI_ISL_421982 hCoV-19/England/20146019704/2020 Europe / United Kingdom / England 2020-04-01 Respiratory Virus Unit, Microbiology Services Colindale, Public Health England Respiratory Virus Unit, Microbiology Services Colindale, Public Health England Monica Galiano, Shahjahan Miah, Angie Lackenby, Omolola Akinbami, Tiina Talts, Leena Bhaw, Richard Myers, Steven Platt, Kirstin Edwards, Jonathan Hubb, Joanna Ellis, Maria Zambon
EPI_ISL_422039 hCoV-19/Wales/PHWC-25779/2020 Europe / United Kingdom / Wales 2020-03-24 Wales Specialist Virology Centre Public Health Wales Microbiology Cardiff Catherine Moore, Johnathan Evans, Malorie Perry, Simon Cottrell, Alec Birchley, Alexander Adams, Amy Gaskin, Bree Gatica-Wilcox, Jason Coombes, Lauren Gilbert, Lee Graham, Nicole Pacchiarini, Sara Kumziene-Summerhayes, Sarah Taylor, Sophie Jones, Sara Rey, Matthew Bull, Joanne Watkins, Sally Corden, Tom Connor
EPI_ISL_422040 hCoV-19/Wales/PHWC-26D1C/2020 Europe / United Kingdom / Wales 2020-03-30 Wales Specialist Virology Centre Public Health Wales Microbiology Cardiff Catherine Moore, Johnathan Evans, Malorie Perry, Simon Cottrell, Alec Birchley, Alexander Adams, Amy Gaskin, Bree Gatica-Wilcox, Jason Coombes, Lauren Gilbert, Lee Graham, Nicole Pacchiarini, Sara Kumziene-Summerhayes, Sarah Taylor, Sophie Jones, Sara Rey, Matthew Bull, Joanne Watkins, Sally Corden, Tom Connor
EPI_ISL_422041 hCoV-19/Wales/PHWC-25C3E/2020 Europe / United Kingdom / Wales 2020-03-25 Wales Specialist Virology Centre Public Health Wales Microbiology Cardiff Catherine Moore, Johnathan Evans, Malorie Perry, Simon Cottrell, Alec Birchley, Alexander Adams, Amy Gaskin, Bree Gatica-Wilcox, Jason Coombes, Lauren Gilbert, Lee Graham, Nicole Pacchiarini, Sara Kumziene-Summerhayes, Sarah Taylor, Sophie Jones, Sara Rey, Matthew Bull, Joanne Watkins, Sally Corden, Tom Connor
EPI_ISL_422042 hCoV-19/Wales/PHWC-264BD/2020 Europe / United Kingdom / Wales 2020-03-28 Wales Specialist Virology Centre Public Health Wales Microbiology Cardiff Catherine Moore, Johnathan Evans, Malorie Perry, Simon Cottrell, Alec Birchley, Alexander Adams, Amy Gaskin, Bree Gatica-Wilcox, Jason Coombes, Lauren Gilbert, Lee Graham, Nicole Pacchiarini, Sara Kumziene-Summerhayes, Sarah Taylor, Sophie Jones, Sara Rey, Matthew Bull, Joanne Watkins, Sally Corden, Tom Connor
EPI_ISL_422043 hCoV-19/Wales/PHWC-269DC/2020 Europe / United Kingdom / Wales 2020-03-29 Wales Specialist Virology Centre Public Health Wales Microbiology Cardiff Catherine Moore, Johnathan Evans, Malorie Perry, Simon Cottrell, Alec Birchley, Alexander Adams, Amy Gaskin, Bree Gatica-Wilcox, Jason Coombes, Lauren Gilbert, Lee Graham, Nicole Pacchiarini, Sara Kumziene-Summerhayes, Sarah Taylor, Sophie Jones, Sara Rey, Matthew Bull, Joanne Watkins, Sally Corden, Tom Connor
EPI_ISL_422044 hCoV-19/Wales/PHWC-26E19/2020 Europe / United Kingdom / Wales 2020-03-29 Wales Specialist Virology Centre Public Health Wales Microbiology Cardiff Catherine Moore, Johnathan Evans, Malorie Perry, Simon Cottrell, Alec Birchley, Alexander Adams, Amy Gaskin, Bree Gatica-Wilcox, Jason Coombes, Lauren Gilbert, Lee Graham, Nicole Pacchiarini, Sara Kumziene-Summerhayes, Sarah Taylor, Sophie Jones, Sara Rey, Matthew Bull, Joanne Watkins, Sally Corden, Tom Connor
EPI_ISL_422045 hCoV-19/Wales/PHWC-25AF8/2020 Europe / United Kingdom / Wales 2020-03-24 Wales Specialist Virology Centre Public Health Wales Microbiology Cardiff Catherine Moore, Johnathan Evans, Malorie Perry, Simon Cottrell, Alec Birchley, Alexander Adams, Amy Gaskin, Bree Gatica-Wilcox, Jason Coombes, Lauren Gilbert, Lee Graham, Nicole Pacchiarini, Sara Kumziene-Summerhayes, Sarah Taylor, Sophie Jones, Sara Rey, Matthew Bull, Joanne Watkins, Sally Corden, Tom Connor

EPI_ISL_422046 hCoV-19/Wales/PHWC-2666C/2020 Europe / United Kingdom / Wales 2020-03-27 Wales Specialist Virology Centre Public Health Wales Microbiology Cardiff Catherine Moore, Johnathan Evans, Malorie Perry, Simon Cottrell, Alec Birchley, Alexander Adams, Amy Gaskin, Bree Gatica-Wilcox, Jason Coombes, Lauren Gilbert, Lee Graham, Nicole Pacchiarini, Sara Kumziene-Summerhayes, Sarah Taylor, Sophie Jones, Sara Rey, Matthew Bull, Joanne Watkins, Sally Corden, Tom Connor
EPI_ISL_422047 hCoV-19/Wales/PHWC-26F34/2020 Europe / United Kingdom / Wales 2020-03-30 Wales Specialist Virology Centre Public Health Wales Microbiology Cardiff Catherine Moore, Johnathan Evans, Malorie Perry, Simon Cottrell, Alec Birchley, Alexander Adams, Amy Gaskin, Bree Gatica-Wilcox, Jason Coombes, Lauren Gilbert, Lee Graham, Nicole Pacchiarini, Sara Kumziene-Summerhayes, Sarah Taylor, Sophie Jones, Sara Rey, Matthew Bull, Joanne Watkins, Sally Corden, Tom Connor
EPI_ISL_422048 hCoV-19/Wales/PHWC-26918/2020 Europe / United Kingdom / Wales 2020-03-27 Wales Specialist Virology Centre Public Health Wales Microbiology Cardiff Catherine Moore, Johnathan Evans, Malorie Perry, Simon Cottrell, Alec Birchley, Alexander Adams, Amy Gaskin, Bree Gatica-Wilcox, Jason Coombes, Lauren Gilbert, Lee Graham, Nicole Pacchiarini, Sara Kumziene-Summerhayes, Sarah Taylor, Sophie Jones, Sara Rey, Matthew Bull, Joanne Watkins, Sally Corden, Tom Connor
EPI_ISL_422049 hCoV-19/Wales/PHWC-2575B/2020 Europe / United Kingdom / Wales 2020-03-24 Wales Specialist Virology Centre Public Health Wales Microbiology Cardiff Catherine Moore, Johnathan Evans, Malorie Perry, Simon Cottrell, Alec Birchley, Alexander Adams, Amy Gaskin, Bree Gatica-Wilcox, Jason Coombes, Lauren Gilbert, Lee Graham, Nicole Pacchiarini, Sara Kumziene-Summerhayes, Sarah Taylor, Sophie Jones, Sara Rey, Matthew Bull, Joanne Watkins, Sally Corden, Tom Connor
EPI_ISL_422050 hCoV-19/Wales/PHWC-26DEF/2020 Europe / United Kingdom / Wales 2020-03-30 Wales Specialist Virology Centre Public Health Wales Microbiology Cardiff Catherine Moore, Johnathan Evans, Malorie Perry, Simon Cottrell, Alec Birchley, Alexander Adams, Amy Gaskin, Bree Gatica-Wilcox, Jason Coombes, Lauren Gilbert, Lee Graham, Nicole Pacchiarini, Sara Kumziene-Summerhayes, Sarah Taylor, Sophie Jones, Sara Rey, Matthew Bull, Joanne Watkins, Sally Corden, Tom Connor
EPI_ISL_422051 hCoV-19/Wales/PHWC-26A51/2020 Europe / United Kingdom / Wales 2020-03-30 Wales Specialist Virology Centre Public Health Wales Microbiology Cardiff Catherine Moore, Johnathan Evans, Malorie Perry, Simon Cottrell, Alec Birchley, Alexander Adams, Amy Gaskin, Bree Gatica-Wilcox, Jason Coombes, Lauren Gilbert, Lee Graham, Nicole Pacchiarini, Sara Kumziene-Summerhayes, Sarah Taylor, Sophie Jones, Sara Rey, Matthew Bull, Joanne Watkins, Sally Corden, Tom Connor
EPI_ISL_422052 hCoV-19/Wales/PHWC-26884/2020 Europe / United Kingdom / Wales 2020-03-27 Wales Specialist Virology Centre Public Health Wales Microbiology Cardiff Catherine Moore, Johnathan Evans, Malorie Perry, Simon Cottrell, Alec Birchley, Alexander Adams, Amy Gaskin, Bree Gatica-Wilcox, Jason Coombes, Lauren Gilbert, Lee Graham, Nicole Pacchiarini, Sara Kumziene-Summerhayes, Sarah Taylor, Sophie Jones, Sara Rey, Matthew Bull, Joanne Watkins, Sally Corden, Tom Connor
EPI_ISL_422053 hCoV-19/Wales/PHWC-25ABC/2020 Europe / United Kingdom / Wales 2020-03-24 Wales Specialist Virology Centre Public Health Wales Microbiology Cardiff Catherine Moore, Johnathan Evans, Malorie Perry, Simon Cottrell, Alec Birchley, Alexander Adams, Amy Gaskin, Bree Gatica-Wilcox, Jason Coombes, Lauren Gilbert, Lee Graham, Nicole Pacchiarini, Sara Kumziene-Summerhayes, Sarah Taylor, Sophie Jones, Sara Rey, Matthew Bull, Joanne Watkins, Sally Corden, Tom Connor
EPI_ISL_422054 hCoV-19/Wales/PHWC-26F9E/2020 Europe / United Kingdom / Wales 2020-03-29 Wales Specialist Virology Centre Public Health Wales Microbiology Cardiff Catherine Moore, Johnathan Evans, Malorie Perry, Simon Cottrell, Alec Birchley, Alexander Adams, Amy Gaskin, Bree Gatica-Wilcox, Jason Coombes, Lauren Gilbert, Lee Graham, Nicole Pacchiarini, Sara Kumziene-Summerhayes, Sarah Taylor, Sophie Jones, Sara Rey, Matthew Bull, Joanne Watkins, Sally Corden, Tom Connor
EPI_ISL_422055 hCoV-19/Wales/PHWC-25E47/2020 Europe / United Kingdom / Wales 2020-03-25 Wales Specialist Virology Centre Public Health Wales Microbiology Cardiff Catherine Moore, Johnathan Evans, Malorie Perry, Simon Cottrell, Alec Birchley, Alexander Adams, Amy Gaskin, Bree Gatica-Wilcox, Jason Coombes, Lauren Gilbert, Lee Graham, Nicole Pacchiarini, Sara Kumziene-Summerhayes, Sarah Taylor, Sophie Jones, Sara Rey, Matthew Bull, Joanne Watkins, Sally Corden, Tom Connor
EPI_ISL_422056 hCoV-19/Wales/PHWC-26CC4/2020 Europe / United Kingdom / Wales 2020-03-28 Wales Specialist Virology Centre Public Health Wales Microbiology Cardiff Catherine Moore, Johnathan Evans, Malorie Perry, Simon Cottrell, Alec Birchley, Alexander Adams, Amy Gaskin, Bree Gatica-Wilcox, Jason Coombes, Lauren Gilbert, Lee Graham, Nicole Pacchiarini, Sara Kumziene-Summerhayes, Sarah Taylor, Sophie Jones, Sara Rey, Matthew Bull, Joanne Watkins, Sally Corden, Tom Connor
EPI_ISL_422057 hCoV-19/Wales/PHWC-268A2/2020 Europe / United Kingdom / Wales 2020-03-28 Wales Specialist Virology Centre Public Health Wales Microbiology Cardiff Catherine Moore, Johnathan Evans, Malorie Perry, Simon Cottrell, Alec Birchley, Alexander Adams, Amy Gaskin, Bree Gatica-Wilcox, Jason Coombes, Lauren Gilbert, Lee Graham, Nicole Pacchiarini, Sara Kumziene-Summerhayes, Sarah Taylor, Sophie Jones, Sara Rey, Matthew Bull, Joanne Watkins, Sally Corden, Tom Connor
EPI_ISL_422058 hCoV-19/Wales/PHWC-26E91/2020 Europe / United Kingdom / Wales 2020-03-30 Wales Specialist Virology Centre Public Health Wales Microbiology Cardiff Catherine Moore, Johnathan Evans, Malorie Perry, Simon Cottrell, Alec Birchley, Alexander Adams, Amy Gaskin, Bree Gatica-Wilcox, Jason Coombes, Lauren Gilbert, Lee Graham, Nicole Pacchiarini, Sara Kumziene-Summerhayes, Sarah Taylor, Sophie Jones, Sara Rey, Matthew Bull, Joanne Watkins, Sally Corden, Tom Connor
EPI_ISL_422059 hCoV-19/Wales/PHWC-26FE9/2020 Europe / United Kingdom / Wales 2020-03-30 Wales Specialist Virology Centre Public Health Wales Microbiology Cardiff Catherine Moore, Johnathan Evans, Malorie Perry, Simon Cottrell, Alec Birchley, Alexander Adams, Amy Gaskin, Bree Gatica-Wilcox, Jason Coombes, Lauren Gilbert, Lee Graham, Nicole Pacchiarini, Sara Kumziene-Summerhayes, Sarah Taylor, Sophie Jones, Sara Rey, Matthew Bull, Joanne Watkins, Sally Corden, Tom Connor

EPI_ISL_422060 hCoV-19/Wales/PHWC-25FEA/2020 Europe / United Kingdom / Wales 2020-03-25 Wales Specialist Virology Centre Public Health Wales Microbiology Cardiff Catherine Moore, Johnathan Evans, Malorie Perry, Simon Cottrell, Alec Birchley, Alexander Adams, Amy Gaskin, Bree Gatica-Wilcox, Jason Coombes, Lauren Gilbert, Lee Graham, Nicole Pacchiarini, Sara Kumziene-Summerhayes, Sarah Taylor, Sophie Jones, Sara Rey, Matthew Bull, Joanne Watkins, Sally Corden, Tom Connor
EPI_ISL_422061 hCoV-19/Wales/PHWC-26E82/2020 Europe / United Kingdom / Wales 2020-03-30 Wales Specialist Virology Centre Public Health Wales Microbiology Cardiff Catherine Moore, Johnathan Evans, Malorie Perry, Simon Cottrell, Alec Birchley, Alexander Adams, Amy Gaskin, Bree Gatica-Wilcox, Jason Coombes, Lauren Gilbert, Lee Graham, Nicole Pacchiarini, Sara Kumziene-Summerhayes, Sarah Taylor, Sophie Jones, Sara Rey, Matthew Bull, Joanne Watkins, Sally Corden, Tom Connor
EPI_ISL_422062 hCoV-19/Wales/PHWC-269AF/2020 Europe / United Kingdom / Wales 2020-03-30 Wales Specialist Virology Centre Public Health Wales Microbiology Cardiff Catherine Moore, Johnathan Evans, Malorie Perry, Simon Cottrell, Alec Birchley, Alexander Adams, Amy Gaskin, Bree Gatica-Wilcox, Jason Coombes, Lauren Gilbert, Lee Graham, Nicole Pacchiarini, Sara Kumziene-Summerhayes, Sarah Taylor, Sophie Jones, Sara Rey, Matthew Bull, Joanne Watkins, Sally Corden, Tom Connor
EPI_ISL_422063 hCoV-19/Wales/PHWC-25B6E/2020 Europe / United Kingdom / Wales 2020-03-25 Wales Specialist Virology Centre Public Health Wales Microbiology Cardiff Catherine Moore, Johnathan Evans, Malorie Perry, Simon Cottrell, Alec Birchley, Alexander Adams, Amy Gaskin, Bree Gatica-Wilcox, Jason Coombes, Lauren Gilbert, Lee Graham, Nicole Pacchiarini, Sara Kumziene-Summerhayes, Sarah Taylor, Sophie Jones, Sara Rey, Matthew Bull, Joanne Watkins, Sally Corden, Tom Connor
EPI_ISL_422064 hCoV-19/Wales/PHWC-25C01/2020 Europe / United Kingdom / Wales 2020-03-25 Wales Specialist Virology Centre Public Health Wales Microbiology Cardiff Catherine Moore, Johnathan Evans, Malorie Perry, Simon Cottrell, Alec Birchley, Alexander Adams, Amy Gaskin, Bree Gatica-Wilcox, Jason Coombes, Lauren Gilbert, Lee Graham, Nicole Pacchiarini, Sara Kumziene-Summerhayes, Sarah Taylor, Sophie Jones, Sara Rey, Matthew Bull, Joanne Watkins, Sally Corden, Tom Connor
EPI_ISL_422065 hCoV-19/Wales/PHWC-2566D/2020 Europe / United Kingdom / Wales 2020-03-24 Wales Specialist Virology Centre Public Health Wales Microbiology Cardiff Catherine Moore, Johnathan Evans, Malorie Perry, Simon Cottrell, Alec Birchley, Alexander Adams, Amy Gaskin, Bree Gatica-Wilcox, Jason Coombes, Lauren Gilbert, Lee Graham, Nicole Pacchiarini, Sara Kumziene-Summerhayes, Sarah Taylor, Sophie Jones, Sara Rey, Matthew Bull, Joanne Watkins, Sally Corden, Tom Connor
EPI_ISL_422066 hCoV-19/Wales/PHWC-2573D/2020 Europe / United Kingdom / Wales 2020-03-24 Wales Specialist Virology Centre Public Health Wales Microbiology Cardiff Catherine Moore, Johnathan Evans, Malorie Perry, Simon Cottrell, Alec Birchley, Alexander Adams, Amy Gaskin, Bree Gatica-Wilcox, Jason Coombes, Lauren Gilbert, Lee Graham, Nicole Pacchiarini, Sara Kumziene-Summerhayes, Sarah Taylor, Sophie Jones, Sara Rey, Matthew Bull, Joanne Watkins, Sally Corden, Tom Connor
EPI_ISL_422067 hCoV-19/Wales/PHWC-25973/2020 Europe / United Kingdom / Wales 2020-03-24 Wales Specialist Virology Centre Public Health Wales Microbiology Cardiff Catherine Moore, Johnathan Evans, Malorie Perry, Simon Cottrell, Alec Birchley, Alexander Adams, Amy Gaskin, Bree Gatica-Wilcox, Jason Coombes, Lauren Gilbert, Lee Graham, Nicole Pacchiarini, Sara Kumziene-Summerhayes, Sarah Taylor, Sophie Jones, Sara Rey, Matthew Bull, Joanne Watkins, Sally Corden, Tom Connor
EPI_ISL_422068 hCoV-19/Wales/PHWC-26EFB/2020 Europe / United Kingdom / Wales 2020-03-30 Wales Specialist Virology Centre Public Health Wales Microbiology Cardiff Catherine Moore, Johnathan Evans, Malorie Perry, Simon Cottrell, Alec Birchley, Alexander Adams, Amy Gaskin, Bree Gatica-Wilcox, Jason Coombes, Lauren Gilbert, Lee Graham, Nicole Pacchiarini, Sara Kumziene-Summerhayes, Sarah Taylor, Sophie Jones, Sara Rey, Matthew Bull, Joanne Watkins, Sally Corden, Tom Connor
EPI_ISL_422069 hCoV-19/Wales/PHWC-270A9/2020 Europe / United Kingdom / Wales 2020-03-29 Wales Specialist Virology Centre Public Health Wales Microbiology Cardiff Catherine Moore, Johnathan Evans, Malorie Perry, Simon Cottrell, Alec Birchley, Alexander Adams, Amy Gaskin, Bree Gatica-Wilcox, Jason Coombes, Lauren Gilbert, Lee Graham, Nicole Pacchiarini, Sara Kumziene-Summerhayes, Sarah Taylor, Sophie Jones, Sara Rey, Matthew Bull, Joanne Watkins, Sally Corden, Tom Connor
EPI_ISL_422070 hCoV-19/Wales/PHWC-26839/2020 Europe / United Kingdom / Wales 2020-03-27 Wales Specialist Virology Centre Public Health Wales Microbiology Cardiff Catherine Moore, Johnathan Evans, Malorie Perry, Simon Cottrell, Alec Birchley, Alexander Adams, Amy Gaskin, Bree Gatica-Wilcox, Jason Coombes, Lauren Gilbert, Lee Graham, Nicole Pacchiarini, Sara Kumziene-Summerhayes, Sarah Taylor, Sophie Jones, Sara Rey, Matthew Bull, Joanne Watkins, Sally Corden, Tom Connor
EPI_ISL_422071 hCoV-19/Wales/PHWC-257B5/2020 Europe / United Kingdom / Wales 2020-03-24 Wales Specialist Virology Centre Public Health Wales Microbiology Cardiff Catherine Moore, Johnathan Evans, Malorie Perry, Simon Cottrell, Alec Birchley, Alexander Adams, Amy Gaskin, Bree Gatica-Wilcox, Jason Coombes, Lauren Gilbert, Lee Graham, Nicole Pacchiarini, Sara Kumziene-Summerhayes, Sarah Taylor, Sophie Jones, Sara Rey, Matthew Bull, Joanne Watkins, Sally Corden, Tom Connor
EPI_ISL_422072 hCoV-19/Wales/PHWC-26F52/2020 Europe / United Kingdom / Wales 2020-03-28 Wales Specialist Virology Centre Public Health Wales Microbiology Cardiff Catherine Moore, Johnathan Evans, Malorie Perry, Simon Cottrell, Alec Birchley, Alexander Adams, Amy Gaskin, Bree Gatica-Wilcox, Jason Coombes, Lauren Gilbert, Lee Graham, Nicole Pacchiarini, Sara Kumziene-Summerhayes, Sarah Taylor, Sophie Jones, Sara Rey, Matthew Bull, Joanne Watkins, Sally Corden, Tom Connor
EPI_ISL_422073 hCoV-19/Wales/PHWC-260AA/2020 Europe / United Kingdom / Wales 2020-03-24 Wales Specialist Virology Centre Public Health Wales Microbiology Cardiff Catherine Moore, Johnathan Evans, Malorie Perry, Simon Cottrell, Alec Birchley, Alexander Adams, Amy Gaskin, Bree Gatica-Wilcox, Jason Coombes, Lauren Gilbert, Lee Graham, Nicole Pacchiarini, Sara Kumziene-Summerhayes, Sarah Taylor, Sophie Jones, Sara Rey, Matthew Bull, Joanne Watkins, Sally Corden, Tom Connor

EPI_ISL_422074 hCoV-19/Wales/PHWC-26954/2020 Europe / United Kingdom / Wales 2020-03-27 Wales Specialist Virology Centre Public Health Wales Microbiology Cardiff Catherine Moore, Johnathan Evans, Malorie Perry, Simon Cottrell, Alec Birchley, Alexander Adams, Amy Gaskin, Bree Gatica-Wilcox, Jason Coombes, Lauren Gilbert, Lee Graham, Nicole Pacchiarini, Sara Kumziene-Summerhayes, Sarah Taylor, Sophie Jones, Sara Rey, Matthew Bull, Joanne Watkins, Sally Corden, Tom Connor
EPI_ISL_422075 hCoV-19/Wales/PHWC-26E46/2020 Europe / United Kingdom / Wales 2020-03-29 Wales Specialist Virology Centre Public Health Wales Microbiology Cardiff Catherine Moore, Johnathan Evans, Malorie Perry, Simon Cottrell, Alec Birchley, Alexander Adams, Amy Gaskin, Bree Gatica-Wilcox, Jason Coombes, Lauren Gilbert, Lee Graham, Nicole Pacchiarini, Sara Kumziene-Summerhayes, Sarah Taylor, Sophie Jones, Sara Rey, Matthew Bull, Joanne Watkins, Sally Corden, Tom Connor
EPI_ISL_422076 hCoV-19/Wales/PHWC-26D3A/2020 Europe / United Kingdom / Wales 2020-03-30 Wales Specialist Virology Centre Public Health Wales Microbiology Cardiff Catherine Moore, Johnathan Evans, Malorie Perry, Simon Cottrell, Alec Birchley, Alexander Adams, Amy Gaskin, Bree Gatica-Wilcox, Jason Coombes, Lauren Gilbert, Lee Graham, Nicole Pacchiarini, Sara Kumziene-Summerhayes, Sarah Taylor, Sophie Jones, Sara Rey, Matthew Bull, Joanne Watkins, Sally Corden, Tom Connor
EPI_ISL_422077 hCoV-19/Wales/PHWC-26A42/2020 Europe / United Kingdom / Wales 2020-03-29 Wales Specialist Virology Centre Public Health Wales Microbiology Cardiff Catherine Moore, Johnathan Evans, Malorie Perry, Simon Cottrell, Alec Birchley, Alexander Adams, Amy Gaskin, Bree Gatica-Wilcox, Jason Coombes, Lauren Gilbert, Lee Graham, Nicole Pacchiarini, Sara Kumziene-Summerhayes, Sarah Taylor, Sophie Jones, Sara Rey, Matthew Bull, Joanne Watkins, Sally Corden, Tom Connor
EPI_ISL_422078 hCoV-19/Wales/PHWC-26013/2020 Europe / United Kingdom / Wales 2020-03-25 Wales Specialist Virology Centre Public Health Wales Microbiology Cardiff Catherine Moore, Johnathan Evans, Malorie Perry, Simon Cottrell, Alec Birchley, Alexander Adams, Amy Gaskin, Bree Gatica-Wilcox, Jason Coombes, Lauren Gilbert, Lee Graham, Nicole Pacchiarini, Sara Kumziene-Summerhayes, Sarah Taylor, Sophie Jones, Sara Rey, Matthew Bull, Joanne Watkins, Sally Corden, Tom Connor
EPI_ISL_422079 hCoV-19/Wales/PHWC-26E37/2020 Europe / United Kingdom / Wales 2020-03-29 Wales Specialist Virology Centre Public Health Wales Microbiology Cardiff Catherine Moore, Johnathan Evans, Malorie Perry, Simon Cottrell, Alec Birchley, Alexander Adams, Amy Gaskin, Bree Gatica-Wilcox, Jason Coombes, Lauren Gilbert, Lee Graham, Nicole Pacchiarini, Sara Kumziene-Summerhayes, Sarah Taylor, Sophie Jones, Sara Rey, Matthew Bull, Joanne Watkins, Sally Corden, Tom Connor
EPI_ISL_422080 hCoV-19/Wales/PHWC-26E55/2020 Europe / United Kingdom / Wales 2020-03-29 Wales Specialist Virology Centre Public Health Wales Microbiology Cardiff Catherine Moore, Johnathan Evans, Malorie Perry, Simon Cottrell, Alec Birchley, Alexander Adams, Amy Gaskin, Bree Gatica-Wilcox, Jason Coombes, Lauren Gilbert, Lee Graham, Nicole Pacchiarini, Sara Kumziene-Summerhayes, Sarah Taylor, Sophie Jones, Sara Rey, Matthew Bull, Joanne Watkins, Sally Corden, Tom Connor
EPI_ISL_422081 hCoV-19/Wales/PHWC-26EBF/2020 Europe / United Kingdom / Wales 2020-03-30 Wales Specialist Virology Centre Public Health Wales Microbiology Cardiff Catherine Moore, Johnathan Evans, Malorie Perry, Simon Cottrell, Alec Birchley, Alexander Adams, Amy Gaskin, Bree Gatica-Wilcox, Jason Coombes, Lauren Gilbert, Lee Graham, Nicole Pacchiarini, Sara Kumziene-Summerhayes, Sarah Taylor, Sophie Jones, Sara Rey, Matthew Bull, Joanne Watkins, Sally Corden, Tom Connor
EPI_ISL_422082 hCoV-19/Wales/PHWC-26BD6/2020 Europe / United Kingdom / Wales 2020-03-28 Wales Specialist Virology Centre Public Health Wales Microbiology Cardiff Catherine Moore, Johnathan Evans, Malorie Perry, Simon Cottrell, Alec Birchley, Alexander Adams, Amy Gaskin, Bree Gatica-Wilcox, Jason Coombes, Lauren Gilbert, Lee Graham, Nicole Pacchiarini, Sara Kumziene-Summerhayes, Sarah Taylor, Sophie Jones, Sara Rey, Matthew Bull, Joanne Watkins, Sally Corden, Tom Connor
EPI_ISL_422083 hCoV-19/Wales/PHWC-2624A/2020 Europe / United Kingdom / Wales 2020-03-27 Wales Specialist Virology Centre Public Health Wales Microbiology Cardiff Catherine Moore, Johnathan Evans, Malorie Perry, Simon Cottrell, Alec Birchley, Alexander Adams, Amy Gaskin, Bree Gatica-Wilcox, Jason Coombes, Lauren Gilbert, Lee Graham, Nicole Pacchiarini, Sara Kumziene-Summerhayes, Sarah Taylor, Sophie Jones, Sara Rey, Matthew Bull, Joanne Watkins, Sally Corden, Tom Connor
EPI_ISL_422084 hCoV-19/Wales/PHWC-26DB2/2020 Europe / United Kingdom / Wales 2020-03-30 Wales Specialist Virology Centre Public Health Wales Microbiology Cardiff Catherine Moore, Johnathan Evans, Malorie Perry, Simon Cottrell, Alec Birchley, Alexander Adams, Amy Gaskin, Bree Gatica-Wilcox, Jason Coombes, Lauren Gilbert, Lee Graham, Nicole Pacchiarini, Sara Kumziene-Summerhayes, Sarah Taylor, Sophie Jones, Sara Rey, Matthew Bull, Joanne Watkins, Sally Corden, Tom Connor
EPI_ISL_422085 hCoV-19/Wales/PHWC-25FAE/2020 Europe / United Kingdom / Wales 2020-03-25 Wales Specialist Virology Centre Public Health Wales Microbiology Cardiff Catherine Moore, Johnathan Evans, Malorie Perry, Simon Cottrell, Alec Birchley, Alexander Adams, Amy Gaskin, Bree Gatica-Wilcox, Jason Coombes, Lauren Gilbert, Lee Graham, Nicole Pacchiarini, Sara Kumziene-Summerhayes, Sarah Taylor, Sophie Jones, Sara Rey, Matthew Bull, Joanne Watkins, Sally Corden, Tom Connor
EPI_ISL_422086 hCoV-19/Wales/PHWC-2708B/2020 Europe / United Kingdom / Wales 2020-03-29 Wales Specialist Virology Centre Public Health Wales Microbiology Cardiff Catherine Moore, Johnathan Evans, Malorie Perry, Simon Cottrell, Alec Birchley, Alexander Adams, Amy Gaskin, Bree Gatica-Wilcox, Jason Coombes, Lauren Gilbert, Lee Graham, Nicole Pacchiarini, Sara Kumziene-Summerhayes, Sarah Taylor, Sophie Jones, Sara Rey, Matthew Bull, Joanne Watkins, Sally Corden, Tom Connor
EPI_ISL_422087 hCoV-19/Wales/PHWC-26CA6/2020 Europe / United Kingdom / Wales 2020-03-28 Wales Specialist Virology Centre Public Health Wales Microbiology Cardiff Catherine Moore, Johnathan Evans, Malorie Perry, Simon Cottrell, Alec Birchley, Alexander Adams, Amy Gaskin, Bree Gatica-Wilcox, Jason Coombes, Lauren Gilbert, Lee Graham, Nicole Pacchiarini, Sara Kumziene-Summerhayes, Sarah Taylor, Sophie Jones, Sara Rey, Matthew Bull, Joanne Watkins, Sally Corden, Tom Connor

EPI_ISL_422088 hCoV-19/Wales/PHWC-26DC1/2020 Europe / United Kingdom / Wales 2020-03-30 Wales Specialist Virology Centre Public Health Wales Microbiology Cardiff Catherine Moore, Johnathan Evans, Malorie Perry, Simon Cottrell, Alec Birchley, Alexander Adams, Amy Gaskin, Bree Gatica-Wilcox, Jason Coombes, Lauren Gilbert, Lee Graham, Nicole Pacchiarini, Sara Kumziene-Summerhayes, Sarah Taylor, Sophie Jones, Sara Rey, Matthew Bull, Joanne Watkins, Sally Corden, Tom Connor
EPI_ISL_422089 hCoV-19/Wales/PHWC-26DA3/2020 Europe / United Kingdom / Wales 2020-03-30 Wales Specialist Virology Centre Public Health Wales Microbiology Cardiff Catherine Moore, Johnathan Evans, Malorie Perry, Simon Cottrell, Alec Birchley, Alexander Adams, Amy Gaskin, Bree Gatica-Wilcox, Jason Coombes, Lauren Gilbert, Lee Graham, Nicole Pacchiarini, Sara Kumziene-Summerhayes, Sarah Taylor, Sophie Jones, Sara Rey, Matthew Bull, Joanne Watkins, Sally Corden, Tom Connor
EPI_ISL_422090 hCoV-19/Wales/PHWC-26ECE/2020 Europe / United Kingdom / Wales 2020-03-30 Wales Specialist Virology Centre Public Health Wales Microbiology Cardiff Catherine Moore, Johnathan Evans, Malorie Perry, Simon Cottrell, Alec Birchley, Alexander Adams, Amy Gaskin, Bree Gatica-Wilcox, Jason Coombes, Lauren Gilbert, Lee Graham, Nicole Pacchiarini, Sara Kumziene-Summerhayes, Sarah Taylor, Sophie Jones, Sara Rey, Matthew Bull, Joanne Watkins, Sally Corden, Tom Connor
EPI_ISL_422091 hCoV-19/Wales/PHWC-264DB/2020 Europe / United Kingdom / Wales 2020-03-28 Wales Specialist Virology Centre Public Health Wales Microbiology Cardiff Catherine Moore, Johnathan Evans, Malorie Perry, Simon Cottrell, Alec Birchley, Alexander Adams, Amy Gaskin, Bree Gatica-Wilcox, Jason Coombes, Lauren Gilbert, Lee Graham, Nicole Pacchiarini, Sara Kumziene-Summerhayes, Sarah Taylor, Sophie Jones, Sara Rey, Matthew Bull, Joanne Watkins, Sally Corden, Tom Connor
EPI_ISL_422092 hCoV-19/Wales/PHWC-26857/2020 Europe / United Kingdom / Wales 2020-03-27 Wales Specialist Virology Centre Public Health Wales Microbiology Cardiff Catherine Moore, Johnathan Evans, Malorie Perry, Simon Cottrell, Alec Birchley, Alexander Adams, Amy Gaskin, Bree Gatica-Wilcox, Jason Coombes, Lauren Gilbert, Lee Graham, Nicole Pacchiarini, Sara Kumziene-Summerhayes, Sarah Taylor, Sophie Jones, Sara Rey, Matthew Bull, Joanne Watkins, Sally Corden, Tom Connor
EPI_ISL_422093 hCoV-19/Wales/PHWC-2707C/2020 Europe / United Kingdom / Wales 2020-03-29 Wales Specialist Virology Centre Public Health Wales Microbiology Cardiff Catherine Moore, Johnathan Evans, Malorie Perry, Simon Cottrell, Alec Birchley, Alexander Adams, Amy Gaskin, Bree Gatica-Wilcox, Jason Coombes, Lauren Gilbert, Lee Graham, Nicole Pacchiarini, Sara Kumziene-Summerhayes, Sarah Taylor, Sophie Jones, Sara Rey, Matthew Bull, Joanne Watkins, Sally Corden, Tom Connor
EPI_ISL_422094 hCoV-19/Wales/PHWC-2617A/2020 Europe / United Kingdom / Wales 2020-03-25 Wales Specialist Virology Centre Public Health Wales Microbiology Cardiff Catherine Moore, Johnathan Evans, Malorie Perry, Simon Cottrell, Alec Birchley, Alexander Adams, Amy Gaskin, Bree Gatica-Wilcox, Jason Coombes, Lauren Gilbert, Lee Graham, Nicole Pacchiarini, Sara Kumziene-Summerhayes, Sarah Taylor, Sophie Jones, Sara Rey, Matthew Bull, Joanne Watkins, Sally Corden, Tom Connor
EPI_ISL_422095 hCoV-19/Wales/PHWC-2705E/2020 Europe / United Kingdom / Wales 2020-03-28 Wales Specialist Virology Centre Public Health Wales Microbiology Cardiff Catherine Moore, Johnathan Evans, Malorie Perry, Simon Cottrell, Alec Birchley, Alexander Adams, Amy Gaskin, Bree Gatica-Wilcox, Jason Coombes, Lauren Gilbert, Lee Graham, Nicole Pacchiarini, Sara Kumziene-Summerhayes, Sarah Taylor, Sophie Jones, Sara Rey, Matthew Bull, Joanne Watkins, Sally Corden, Tom Connor
EPI_ISL_422096 hCoV-19/Wales/PHWC-2675A/2020 Europe / United Kingdom / Wales 2020-03-28 Wales Specialist Virology Centre Public Health Wales Microbiology Cardiff Catherine Moore, Johnathan Evans, Malorie Perry, Simon Cottrell, Alec Birchley, Alexander Adams, Amy Gaskin, Bree Gatica-Wilcox, Jason Coombes, Lauren Gilbert, Lee Graham, Nicole Pacchiarini, Sara Kumziene-Summerhayes, Sarah Taylor, Sophie Jones, Sara Rey, Matthew Bull, Joanne Watkins, Sally Corden, Tom Connor
EPI_ISL_422097 hCoV-19/Wales/PHWC-26D0D/2020 Europe / United Kingdom / Wales 2020-03-30 Wales Specialist Virology Centre Public Health Wales Microbiology Cardiff Catherine Moore, Johnathan Evans, Malorie Perry, Simon Cottrell, Alec Birchley, Alexander Adams, Amy Gaskin, Bree Gatica-Wilcox, Jason Coombes, Lauren Gilbert, Lee Graham, Nicole Pacchiarini, Sara Kumziene-Summerhayes, Sarah Taylor, Sophie Jones, Sara Rey, Matthew Bull, Joanne Watkins, Sally Corden, Tom Connor
EPI_ISL_422098 hCoV-19/Wales/PHWC-26848/2020 Europe / United Kingdom / Wales 2020-03-27 Wales Specialist Virology Centre Public Health Wales Microbiology Cardiff Catherine Moore, Johnathan Evans, Malorie Perry, Simon Cottrell, Alec Birchley, Alexander Adams, Amy Gaskin, Bree Gatica-Wilcox, Jason Coombes, Lauren Gilbert, Lee Graham, Nicole Pacchiarini, Sara Kumziene-Summerhayes, Sarah Taylor, Sophie Jones, Sara Rey, Matthew Bull, Joanne Watkins, Sally Corden, Tom Connor
EPI_ISL_422099 hCoV-19/Wales/PHWC-27021/2020 Europe / United Kingdom / Wales 2020-03-28 Wales Specialist Virology Centre Public Health Wales Microbiology Cardiff Catherine Moore, Johnathan Evans, Malorie Perry, Simon Cottrell, Alec Birchley, Alexander Adams, Amy Gaskin, Bree Gatica-Wilcox, Jason Coombes, Lauren Gilbert, Lee Graham, Nicole Pacchiarini, Sara Kumziene-Summerhayes, Sarah Taylor, Sophie Jones, Sara Rey, Matthew Bull, Joanne Watkins, Sally Corden, Tom Connor
EPI_ISL_423371 hCoV-19/England/20140002204/2020 Europe / United Kingdom / England 2020-03-29 Respiratory Virus Unit, Microbiology Services Colindale, Public Health England Respiratory Virus Unit, Microbiology Services Colindale, Public Health England Monica Galiano, Shahjahan Miah, Angie Lackenby, Omolola Akinbami, Tiina Talts, Leena Bhaw, Richard Myers, Steven Platt, Kirstin Edwards, Jonathan Hubb, Joanna Ellis, Maria Zambon
EPI_ISL_423372 hCoV-19/England/20140002604/2020 Europe / United Kingdom / England 2020-03-28 Respiratory Virus Unit, Microbiology Services Colindale, Public Health England Respiratory Virus Unit, Microbiology Services Colindale, Public Health England Monica Galiano, Shahjahan Miah, Angie Lackenby, Omolola Akinbami, Tiina Talts, Leena Bhaw, Richard Myers, Steven Platt, Kirstin Edwards, Jonathan Hubb, Joanna Ellis, Maria Zambon

EPI_ISL_423373 hCoV-19/England/20140002804/2020   Europe / United Kingdom / England   2020-03-28
Respiratory Virus Unit, Microbiology Services Colindale, Public Health England Respiratory Virus Unit, Microbiology Services Colindale, Public Health England Monica Galiano, Shahjahan Miah, Angie Lackenby, Omolola Akinbami, Tiina Talts, Leena Bhaw, Richard Myers, Steven Platt, Kirstin Edwards, Jonathan Hubb, Joanna Ellis, Maria Zambon
EPI_ISL_423375 hCoV-19/England/20140003402/2020   Europe / United Kingdom / England   2020-03-28
Respiratory Virus Unit, Microbiology Services Colindale, Public Health England Respiratory Virus Unit, Microbiology Services Colindale, Public Health England Monica Galiano, Shahjahan Miah, Angie Lackenby, Omolola Akinbami, Tiina Talts, Leena Bhaw, Richard Myers, Steven Platt, Kirstin Edwards, Jonathan Hubb, Joanna Ellis, Maria Zambon
EPI_ISL_423376 hCoV-19/England/20140003404/2020   Europe / United Kingdom / England   2020-03-28
Respiratory Virus Unit, Microbiology Services Colindale, Public Health England Respiratory Virus Unit, Microbiology Services Colindale, Public Health England Monica Galiano, Shahjahan Miah, Angie Lackenby, Omolola Akinbami, Tiina Talts, Leena Bhaw, Richard Myers, Steven Platt, Kirstin Edwards, Jonathan Hubb, Joanna Ellis, Maria Zambon
EPI_ISL_423379 hCoV-19/England/20140004004/2020   Europe / United Kingdom / England   2020-03-28
Respiratory Virus Unit, Microbiology Services Colindale, Public Health England Respiratory Virus Unit, Microbiology Services Colindale, Public Health England Monica Galiano, Shahjahan Miah, Angie Lackenby, Omolola Akinbami, Tiina Talts, Leena Bhaw, Richard Myers, Steven Platt, Kirstin Edwards, Jonathan Hubb, Joanna Ellis, Maria Zambon
EPI_ISL_423380 hCoV-19/England/20140005304/2020   Europe / United Kingdom / England   2020-03-29
Respiratory Virus Unit, Microbiology Services Colindale, Public Health England Respiratory Virus Unit, Microbiology Services Colindale, Public Health England Monica Galiano, Shahjahan Miah, Angie Lackenby, Omolola Akinbami, Tiina Talts, Leena Bhaw, Richard Myers, Steven Platt, Kirstin Edwards, Jonathan Hubb, Joanna Ellis, Maria Zambon
EPI_ISL_423382 hCoV-19/England/20140007004/2020   Europe / United Kingdom / England   2020-03-29
Respiratory Virus Unit, Microbiology Services Colindale, Public Health England Respiratory Virus Unit, Microbiology Services Colindale, Public Health England Monica Galiano, Shahjahan Miah, Angie Lackenby, Omolola Akinbami, Tiina Talts, Leena Bhaw, Richard Myers, Steven Platt, Kirstin Edwards, Jonathan Hubb, Joanna Ellis, Maria Zambon
EPI_ISL_423384 hCoV-19/England/20140007604/2020   Europe / United Kingdom / England   2020-03-29
Respiratory Virus Unit, Microbiology Services Colindale, Public Health England Respiratory Virus Unit, Microbiology Services Colindale, Public Health England Monica Galiano, Shahjahan Miah, Angie Lackenby, Omolola Akinbami, Tiina Talts, Leena Bhaw, Richard Myers, Steven Platt, Kirstin Edwards, Jonathan Hubb, Joanna Ellis, Maria Zambon
EPI_ISL_423385 hCoV-19/England/20140009204/2020   Europe / United Kingdom / England   2020-03-28
Respiratory Virus Unit, Microbiology Services Colindale, Public Health England Respiratory Virus Unit, Microbiology Services Colindale, Public Health England Monica Galiano, Shahjahan Miah, Angie Lackenby, Omolola Akinbami, Tiina Talts, Leena Bhaw, Richard Myers, Steven Platt, Kirstin Edwards, Jonathan Hubb, Joanna Ellis, Maria Zambon
EPI_ISL_423387 hCoV-19/England/20140009404/2020   Europe / United Kingdom / England   2020-03-28
Respiratory Virus Unit, Microbiology Services Colindale, Public Health England Respiratory Virus Unit, Microbiology Services Colindale, Public Health England Monica Galiano, Shahjahan Miah, Angie Lackenby, Omolola Akinbami, Tiina Talts, Leena Bhaw, Richard Myers, Steven Platt, Kirstin Edwards, Jonathan Hubb, Joanna Ellis, Maria Zambon
EPI_ISL_423389 hCoV-19/England/20140060304/2020   Europe / United Kingdom / England   2020-03-30
Respiratory Virus Unit, Microbiology Services Colindale, Public Health England Respiratory Virus Unit, Microbiology Services Colindale, Public Health England Monica Galiano, Shahjahan Miah, Angie Lackenby, Omolola Akinbami, Tiina Talts, Leena Bhaw, Richard Myers, Steven Platt, Kirstin Edwards, Jonathan Hubb, Joanna Ellis, Maria Zambon
EPI_ISL_423393 hCoV-19/England/20140064004/2020   Europe / United Kingdom / England   2020-03-30
Respiratory Virus Unit, Microbiology Services Colindale, Public Health England Respiratory Virus Unit, Microbiology Services Colindale, Public Health England Monica Galiano, Shahjahan Miah, Angie Lackenby, Omolola Akinbami, Tiina Talts, Leena Bhaw, Richard Myers, Steven Platt, Kirstin Edwards, Jonathan Hubb, Joanna Ellis, Maria Zambon
EPI_ISL_423394 hCoV-19/England/20140064504/2020   Europe / United Kingdom / England   2020-03-30
Respiratory Virus Unit, Microbiology Services Colindale, Public Health England Respiratory Virus Unit, Microbiology Services Colindale, Public Health England Monica Galiano, Shahjahan Miah, Angie Lackenby, Omolola Akinbami, Tiina Talts, Leena Bhaw, Richard Myers, Steven Platt, Kirstin Edwards, Jonathan Hubb, Joanna Ellis, Maria Zambon
EPI_ISL_423395 hCoV-19/England/20140064704/2020   Europe / United Kingdom / England   2020-03-28
Respiratory Virus Unit, Microbiology Services Colindale, Public Health England Respiratory Virus Unit, Microbiology Services Colindale, Public Health England Monica Galiano, Shahjahan Miah, Angie Lackenby, Omolola Akinbami, Tiina Talts, Leena Bhaw, Richard Myers, Steven Platt, Kirstin Edwards, Jonathan Hubb, Joanna Ellis, Maria Zambon
EPI_ISL_423396 hCoV-19/England/20140064804/2020   Europe / United Kingdom / England   2020-03-28
Respiratory Virus Unit, Microbiology Services Colindale, Public Health England Respiratory Virus Unit, Microbiology Services Colindale, Public Health England Monica Galiano, Shahjahan Miah, Angie Lackenby, Omolola Akinbami, Tiina Talts, Leena Bhaw, Richard Myers, Steven Platt, Kirstin Edwards, Jonathan Hubb, Joanna Ellis, Maria Zambon

EPI_ISL_423398	hCoV-19/England/20140066004/2020	Europe / United Kingdom / England	2020-03-29	Respiratory Virus Unit, Microbiology Services Colindale, Public Health England	Respiratory Virus Unit, Microbiology Services Colindale, Public Health England	Monica Galiano, Shahjahan Miah, Angie Lackenby, Omolola Akinbami, Tiina Talts, Leena Bhaw, Richard Myers, Steven Platt, Kirstin Edwards, Jonathan Hubb, Joanna Ellis, Maria Zambon
EPI_ISL_423399	hCoV-19/England/20140066204/2020	Europe / United Kingdom / England	2020-03-29	Respiratory Virus Unit, Microbiology Services Colindale, Public Health England	Respiratory Virus Unit, Microbiology Services Colindale, Public Health England	Monica Galiano, Shahjahan Miah, Angie Lackenby, Omolola Akinbami, Tiina Talts, Leena Bhaw, Richard Myers, Steven Platt, Kirstin Edwards, Jonathan Hubb, Joanna Ellis, Maria Zambon
EPI_ISL_423400	hCoV-19/England/20140068404/2020	Europe / United Kingdom / England	2020-03-30	Respiratory Virus Unit, Microbiology Services Colindale, Public Health England	Respiratory Virus Unit, Microbiology Services Colindale, Public Health England	Monica Galiano, Shahjahan Miah, Angie Lackenby, Omolola Akinbami, Tiina Talts, Leena Bhaw, Richard Myers, Steven Platt, Kirstin Edwards, Jonathan Hubb, Joanna Ellis, Maria Zambon
EPI_ISL_423401	hCoV-19/England/20140068904/2020	Europe / United Kingdom / England	2020-03-29	Respiratory Virus Unit, Microbiology Services Colindale, Public Health England	Respiratory Virus Unit, Microbiology Services Colindale, Public Health England	Monica Galiano, Shahjahan Miah, Angie Lackenby, Omolola Akinbami, Tiina Talts, Leena Bhaw, Richard Myers, Steven Platt, Kirstin Edwards, Jonathan Hubb, Joanna Ellis, Maria Zambon
EPI_ISL_424101	hCoV-19/England/20129126204/2020	Europe / United Kingdom / England	2020-03-12	Respiratory Virus Unit, Microbiology Services Colindale, Public Health England	Respiratory Virus Unit, Microbiology Services Colindale, Public Health England	Monica Galiano, Shahjahan Miah, Angie Lackenby, Omolola Akinbami, Tiina Talts, Leena Bhaw, Richard Myers, Steven Platt, Kirstin Edwards, Jonathan Hubb, Joanna Ellis, Maria Zambon
EPI_ISL_424117	hCoV-19/England/20129148904/2020	Europe / United Kingdom / England	2020-03-12	Respiratory Virus Unit, Microbiology Services Colindale, Public Health England	Respiratory Virus Unit, Microbiology Services Colindale, Public Health England	Monica Galiano, Shahjahan Miah, Angie Lackenby, Omolola Akinbami, Tiina Talts, Leena Bhaw, Richard Myers, Steven Platt, Kirstin Edwards, Jonathan Hubb, Joanna Ellis, Maria Zambon
EPI_ISL_424119	hCoV-19/England/20129158804/2020	Europe / United Kingdom / England	2020-03-21	Respiratory Virus Unit, Microbiology Services Colindale, Public Health England	Respiratory Virus Unit, Microbiology Services Colindale, Public Health England	Monica Galiano, Shahjahan Miah, Angie Lackenby, Omolola Akinbami, Tiina Talts, Leena Bhaw, Richard Myers, Steven Platt, Kirstin Edwards, Jonathan Hubb, Joanna Ellis, Maria Zambon
EPI_ISL_424121	hCoV-19/England/20129160704/2020	Europe / United Kingdom / England	2020-03-21	Respiratory Virus Unit, Microbiology Services Colindale, Public Health England	Respiratory Virus Unit, Microbiology Services Colindale, Public Health England	Monica Galiano, Shahjahan Miah, Angie Lackenby, Omolola Akinbami, Tiina Talts, Leena Bhaw, Richard Myers, Steven Platt, Kirstin Edwards, Jonathan Hubb, Joanna Ellis, Maria Zambon
EPI_ISL_424126	hCoV-19/England/20129163604/2020	Europe / United Kingdom / England	2020-03-21	Respiratory Virus Unit, Microbiology Services Colindale, Public Health England	Respiratory Virus Unit, Microbiology Services Colindale, Public Health England	Monica Galiano, Shahjahan Miah, Angie Lackenby, Omolola Akinbami, Tiina Talts, Leena Bhaw, Richard Myers, Steven Platt, Kirstin Edwards, Jonathan Hubb, Joanna Ellis, Maria Zambon
EPI_ISL_424140	hCoV-19/England/20129171604/2020	Europe / United Kingdom / England	2020-03-21	Respiratory Virus Unit, Microbiology Services Colindale, Public Health England	Respiratory Virus Unit, Microbiology Services Colindale, Public Health England	Monica Galiano, Shahjahan Miah, Angie Lackenby, Omolola Akinbami, Tiina Talts, Leena Bhaw, Richard Myers, Steven Platt, Kirstin Edwards, Jonathan Hubb, Joanna Ellis, Maria Zambon
EPI_ISL_424152	hCoV-19/England/20129174804/2020	Europe / United Kingdom / England	2020-03-20	Respiratory Virus Unit, Microbiology Services Colindale, Public Health England	Respiratory Virus Unit, Microbiology Services Colindale, Public Health England	Monica Galiano, Shahjahan Miah, Angie Lackenby, Omolola Akinbami, Tiina Talts, Leena Bhaw, Richard Myers, Steven Platt, Kirstin Edwards, Jonathan Hubb, Joanna Ellis, Maria Zambon
EPI_ISL_424166	hCoV-19/USA/WA-UW-1620/2020	North America / USA / Washington	2020-03-19	UW Virology Lab	UW Virology Lab	Pavitra Roychoudhury, Hong Xie, Keith Jerome, Alexander Greninger
EPI_ISL_424167	hCoV-19/USA/WA-UW-1616/2020	North America / USA / Washington	2020-03-19	UW Virology Lab	UW Virology Lab	Pavitra Roychoudhury, Hong Xie, Keith Jerome, Alexander Greninger
EPI_ISL_424169	hCoV-19/USA/WA-UW-1538/2020	North America / USA / Washington	2020-03-19	UW Virology Lab	UW Virology Lab	Pavitra Roychoudhury, Hong Xie, Keith Jerome, Alexander Greninger
EPI_ISL_424170	hCoV-19/USA/WA-UW-1595/2020	North America / USA / Washington	2020-03-19	UW Virology Lab	UW Virology Lab	Pavitra Roychoudhury, Hong Xie, Keith Jerome, Alexander Greninger
EPI_ISL_424172	hCoV-19/USA/WA-UW-1575/2020	North America / USA / Washington	2020-03-19	UW Virology Lab	UW Virology Lab	Pavitra Roychoudhury, Hong Xie, Keith Jerome, Alexander Greninger
EPI_ISL_424173	hCoV-19/USA/IL-UW-1311/2020	North America / USA / Illinois	2020-03-14	UW Virology Lab	UW Virology Lab	Pavitra Roychoudhury, Hong Xie, Keith Jerome, Alexander Greninger
EPI_ISL_424174	hCoV-19/USA/CT-UW-1365/2020	North America / USA / Connecticut	2020-03-13	UW Virology Lab	UW Virology Lab	Pavitra Roychoudhury, Hong Xie, Keith Jerome, Alexander Greninger
EPI_ISL_424175	hCoV-19/USA/WA-UW-1362/2020	North America / USA / Washington	2020-03-17	UW

Virology Lab	UW Virology Lab Pavitra Roychoudhury, Hong Xie, Keith Jerome, Alexander Greninger
EPI_ISL_424176	hCoV-19/USA/WA-UW-1472/2020	North America / USA / Washington	2020-03-17	UW Virology Lab	UW Virology Lab Pavitra Roychoudhury, Hong Xie, Keith Jerome, Alexander Greninger
EPI_ISL_424178	hCoV-19/USA/WA-UW-1342/2020	North America / USA / Washington	2020-03-17	UW Virology Lab	UW Virology Lab Pavitra Roychoudhury, Hong Xie, Keith Jerome, Alexander Greninger
EPI_ISL_424179	hCoV-19/USA/WA-UW-1341/2020	North America / USA / Washington	2020-03-17	UW Virology Lab	UW Virology Lab Pavitra Roychoudhury, Hong Xie, Keith Jerome, Alexander Greninger
EPI_ISL_424180	hCoV-19/USA/WA-UW-1294/2020	North America / USA / Washington	2020-03-18	UW Virology Lab	UW Virology Lab Pavitra Roychoudhury, Hong Xie, Keith Jerome, Alexander Greninger
EPI_ISL_424181	hCoV-19/USA/UN-UW-1402/2020	North America / USA	2020-03-16	UW Virology Lab UW Virology Lab	Pavitra Roychoudhury, Hong Xie, Keith Jerome, Alexander Greninger
EPI_ISL_424182	hCoV-19/USA/WA-UW-1478/2020	North America / USA / Washington	2020-03-18	UW Virology Lab	UW Virology Lab Pavitra Roychoudhury, Hong Xie, Keith Jerome, Alexander Greninger
EPI_ISL_424183	hCoV-19/USA/WA-UW-1436/2020	North America / USA / Washington	2020-03-18	UW Virology Lab	UW Virology Lab Pavitra Roychoudhury, Hong Xie, Keith Jerome, Alexander Greninger
EPI_ISL_424184	hCoV-19/USA/WA-UW-1422/2020	North America / USA / Washington	2020-03-18	UW Virology Lab	UW Virology Lab Pavitra Roychoudhury, Hong Xie, Keith Jerome, Alexander Greninger
EPI_ISL_424186	hCoV-19/USA/WA-UW-1644/2020	North America / USA / Washington	2020-03-20	UW Virology Lab	UW Virology Lab Pavitra Roychoudhury, Hong Xie, Keith Jerome, Alexander Greninger
EPI_ISL_424187	hCoV-19/USA/WA-UW-1646/2020	North America / USA / Washington	2020-03-20	UW Virology Lab	UW Virology Lab Pavitra Roychoudhury, Hong Xie, Keith Jerome, Alexander Greninger
EPI_ISL_424188	hCoV-19/USA/WA-UW-1645/2020	North America / USA / Washington	2020-03-20	UW Virology Lab	UW Virology Lab Pavitra Roychoudhury, Hong Xie, Keith Jerome, Alexander Greninger
EPI_ISL_424189	hCoV-19/USA/UN-UW-Not found/2020	North America / USA	2020-03 UW Virology Lab UW Virology Lab	Pavitra Roychoudhury, Hong Xie, Keith Jerome, Alexander Greninger
EPI_ISL_424191	hCoV-19/USA/WA-UW-1683/2020	North America / USA / Washington	2020-03-20	UW Virology Lab	UW Virology Lab Pavitra Roychoudhury, Hong Xie, Keith Jerome, Alexander Greninger
EPI_ISL_424192	hCoV-19/USA/WA-UW-1688/2020	North America / USA / Washington	2020-03-20	UW Virology Lab	UW Virology Lab Pavitra Roychoudhury, Hong Xie, Keith Jerome, Alexander Greninger
EPI_ISL_424193	hCoV-19/USA/WA-UW-1728/2020	North America / USA / Washington	2020-03-21	UW Virology Lab	UW Virology Lab Pavitra Roychoudhury, Hong Xie, Keith Jerome, Alexander Greninger
EPI_ISL_424194	hCoV-19/USA/WA-UW-1661/2020	North America / USA / Washington	2020-03-20	UW Virology Lab	UW Virology Lab Pavitra Roychoudhury, Hong Xie, Keith Jerome, Alexander Greninger
EPI_ISL_424195	hCoV-19/USA/WA-UW-1726/2020	North America / USA / Washington	2020-03-21	UW Virology Lab	UW Virology Lab Pavitra Roychoudhury, Hong Xie, Keith Jerome, Alexander Greninger
EPI_ISL_424196	hCoV-19/USA/WA-UW-1666/2020	North America / USA / Washington	2020-03-20	UW Virology Lab	UW Virology Lab Pavitra Roychoudhury, Hong Xie, Keith Jerome, Alexander Greninger
EPI_ISL_424197	hCoV-19/USA/WA-UW-1640/2020	North America / USA / Washington	2020-03-20	UW Virology Lab	UW Virology Lab Pavitra Roychoudhury, Hong Xie, Keith Jerome, Alexander Greninger
EPI_ISL_424198	hCoV-19/USA/WA-UW-1656/2020	North America / USA / Washington	2020-03-20	UW Virology Lab	UW Virology Lab Pavitra Roychoudhury, Hong Xie, Keith Jerome, Alexander Greninger
EPI_ISL_424703	hCoV-19/Sweden/RV-FOI-1/2020	Europe / Sweden / Vasterbotten	2020-03-15	Klinisk mikrobiologi, Region Västerbotten	Unit for Biological Agents, Department for CBRN Defence and Security, Swedish Defence Research Agency	FOI Bioinformatics team
EPI_ISL_425300	hCoV-19/England/CAMB-74508/2020 Europe / United Kingdom / England	2020-03-28	Department of Pathology, University of Cambridge	COVID-19 Genomics UK (COG-UK) Consortium	Luke W Meredith, M. Estee Torok , Myra Hosmillo, William L. Hamilton, Martin D. Curran, Theresa Feltwell, Anna Yakovleva, Charlotte J. Houldcroft, Aminu S. Jahun, Sarah L. Caddy, Ian Goodfellow
EPI_ISL_425304	hCoV-19/England/CAMB-74544/2020 Europe / United Kingdom / England	2020-03-28	Department of Pathology, University of Cambridge	COVID-19 Genomics UK (COG-UK) Consortium	Luke W Meredith, M. Estee Torok , Myra Hosmillo, William L. Hamilton, Martin D. Curran, Theresa Feltwell, Anna Yakovleva, Charlotte J. Houldcroft, Aminu S. Jahun, Sarah L. Caddy, Ian Goodfellow
EPI_ISL_425305	hCoV-19/England/CAMB-74553/2020 Europe / United Kingdom / England	2020-03-28	Department of Pathology, University of Cambridge	COVID-19 Genomics UK (COG-UK) Consortium	Luke W Meredith, M. Estee Torok , Myra Hosmillo, William L. Hamilton, Martin D. Curran, Theresa Feltwell, Anna Yakovleva, Charlotte J. Houldcroft, Aminu S. Jahun, Sarah L. Caddy, Ian Goodfellow
EPI_ISL_425423	hCoV-19/England/CAMB-76D1D/2020 Europe / United Kingdom / England	2020-03-30	Department of Pathology, University of Cambridge	COVID-19 Genomics UK (COG-UK) Consortium	Luke W Meredith, M. Estee Torok , Myra Hosmillo, William L. Hamilton, Martin D. Curran, Theresa Feltwell, Anna Yakovleva, Charlotte J. Houldcroft, Aminu S. Jahun, Sarah L. Caddy, Ian Goodfellow
EPI_ISL_425424	hCoV-19/England/CAMB-76D3B/2020 Europe / United Kingdom / England	2020-03-31	Department of Pathology, University of Cambridge	COVID-19 Genomics UK (COG-UK) Consortium	Luke W Meredith, M. Estee Torok , Myra Hosmillo, William L. Hamilton, Martin D. Curran, Theresa Feltwell, Anna Yakovleva, Charlotte J. Houldcroft, Aminu S. Jahun, Sarah L. Caddy, Ian Goodfellow
EPI_ISL_425425	hCoV-19/England/CAMB-76D59/2020 Europe / United Kingdom / England	2020-03-30	Department of Pathology, University of Cambridge	COVID-19 Genomics UK (COG-UK) Consortium	Luke W Meredith, M. Estee Torok , Myra Hosmillo, William L. Hamilton, Martin D. Curran, Theresa Feltwell, Anna Yakovleva, Charlotte J. Houldcroft, Aminu S. Jahun, Sarah L. Caddy, Ian Goodfellow
EPI_ISL_425427	hCoV-19/England/CAMB-76D95/2020 Europe / United Kingdom / England	2020-03-31	Department of Pathology, University of Cambridge	COVID-19 Genomics UK (COG-UK) Consortium	Luke W

Meredith, M. Estee Torok , Myra Hosmillo, William L. Hamilton, Martin D. Curran, Theresa Feltwell, Anna Yakovleva, Charlotte J. Houldcroft, Aminu S. Jahun, Sarah L. Caddy, Ian Goodfellow
EPI_ISL_425428 hCoV-19/England/CAMB-76DB3/2020 Europe / United Kingdom / England 2020-03-31 Department of Pathology, University of Cambridge COVID-19 Genomics UK (COG-UK) Consortium Luke W Meredith, M. Estee Torok , Myra Hosmillo, William L. Hamilton, Martin D. Curran, Theresa Feltwell, Anna Yakovleva, Charlotte J. Houldcroft, Aminu S. Jahun, Sarah L. Caddy, Ian Goodfellow
EPI_ISL_425430 hCoV-19/England/CAMB-76DFF/2020 Europe / United Kingdom / England 2020-03-30 Department of Pathology, University of Cambridge COVID-19 Genomics UK (COG-UK) Consortium Luke W Meredith, M. Estee Torok , Myra Hosmillo, William L. Hamilton, Martin D. Curran, Theresa Feltwell, Anna Yakovleva, Charlotte J. Houldcroft, Aminu S. Jahun, Sarah L. Caddy, Ian Goodfellow
EPI_ISL_425431 hCoV-19/England/CAMB-76E1A/2020 Europe / United Kingdom / England 2020-03-30 Department of Pathology, University of Cambridge COVID-19 Genomics UK (COG-UK) Consortium Luke W Meredith, M. Estee Torok , Myra Hosmillo, William L. Hamilton, Martin D. Curran, Theresa Feltwell, Anna Yakovleva, Charlotte J. Houldcroft, Aminu S. Jahun, Sarah L. Caddy, Ian Goodfellow
EPI_ISL_425433 hCoV-19/England/CAMB-76E56/2020 Europe / United Kingdom / England 2020-03-30 Department of Pathology, University of Cambridge COVID-19 Genomics UK (COG-UK) Consortium Luke W Meredith, M. Estee Torok , Myra Hosmillo, William L. Hamilton, Martin D. Curran, Theresa Feltwell, Anna Yakovleva, Charlotte J. Houldcroft, Aminu S. Jahun, Sarah L. Caddy, Ian Goodfellow
EPI_ISL_425434 hCoV-19/England/CAMB-76E74/2020 Europe / United Kingdom / England 2020-03-31 Department of Pathology, University of Cambridge COVID-19 Genomics UK (COG-UK) Consortium Luke W Meredith, M. Estee Torok , Myra Hosmillo, William L. Hamilton, Martin D. Curran, Theresa Feltwell, Anna Yakovleva, Charlotte J. Houldcroft, Aminu S. Jahun, Sarah L. Caddy, Ian Goodfellow
EPI_ISL_425435 hCoV-19/England/CAMB-76E92/2020 Europe / United Kingdom / England 2020-04-01 Department of Pathology, University of Cambridge COVID-19 Genomics UK (COG-UK) Consortium Luke W Meredith, M. Estee Torok , Myra Hosmillo, William L. Hamilton, Martin D. Curran, Theresa Feltwell, Anna Yakovleva, Charlotte J. Houldcroft, Aminu S. Jahun, Sarah L. Caddy, Ian Goodfellow
EPI_ISL_425436 hCoV-19/England/CAMB-76EB0/2020 Europe / United Kingdom / England 2020-04-01 Department of Pathology, University of Cambridge COVID-19 Genomics UK (COG-UK) Consortium Luke W Meredith, M. Estee Torok , Myra Hosmillo, William L. Hamilton, Martin D. Curran, Theresa Feltwell, Anna Yakovleva, Charlotte J. Houldcroft, Aminu S. Jahun, Sarah L. Caddy, Ian Goodfellow
EPI_ISL_425437 hCoV-19/England/CAMB-76EDE/2020 Europe / United Kingdom / England 2020-03-31 Department of Pathology, University of Cambridge COVID-19 Genomics UK (COG-UK) Consortium Luke W Meredith, M. Estee Torok , Myra Hosmillo, William L. Hamilton, Martin D. Curran, Theresa Feltwell, Anna Yakovleva, Charlotte J. Houldcroft, Aminu S. Jahun, Sarah L. Caddy, Ian Goodfellow
EPI_ISL_425439 hCoV-19/England/CAMB-76F17/2020 Europe / United Kingdom / England 2020 Department of Pathology, University of Cambridge COVID-19 Genomics UK (COG-UK) Consortium Luke W Meredith, M. Estee Torok , Myra Hosmillo, William L. Hamilton, Martin D. Curran, Theresa Feltwell, Anna Yakovleva, Charlotte J. Houldcroft, Aminu S. Jahun, Sarah L. Caddy, Ian Goodfellow
EPI_ISL_425440 hCoV-19/England/CAMB-76F35/2020 Europe / United Kingdom / England 2020 Department of Pathology, University of Cambridge COVID-19 Genomics UK (COG-UK) Consortium Luke W Meredith, M. Estee Torok , Myra Hosmillo, William L. Hamilton, Martin D. Curran, Theresa Feltwell, Anna Yakovleva, Charlotte J. Houldcroft, Aminu S. Jahun, Sarah L. Caddy, Ian Goodfellow
EPI_ISL_425441 hCoV-19/England/CAMB-76F62/2020 Europe / United Kingdom / England 2020-03-31 Department of Pathology, University of Cambridge COVID-19 Genomics UK (COG-UK) Consortium Luke W Meredith, M. Estee Torok , Myra Hosmillo, William L. Hamilton, Martin D. Curran, Theresa Feltwell, Anna Yakovleva, Charlotte J. Houldcroft, Aminu S. Jahun, Sarah L. Caddy, Ian Goodfellow
EPI_ISL_425443 hCoV-19/England/CAMB-76F9F/2020 Europe / United Kingdom / England 2020-04-01 Department of Pathology, University of Cambridge COVID-19 Genomics UK (COG-UK) Consortium Luke W Meredith, M. Estee Torok , Myra Hosmillo, William L. Hamilton, Martin D. Curran, Theresa Feltwell, Anna Yakovleva, Charlotte J. Houldcroft, Aminu S. Jahun, Sarah L. Caddy, Ian Goodfellow
EPI_ISL_425446 hCoV-19/England/CAMB-76FCC/2020 Europe / United Kingdom / England 2020-04-01 Department of Pathology, University of Cambridge COVID-19 Genomics UK (COG-UK) Consortium Luke W Meredith, M. Estee Torok , Myra Hosmillo, William L. Hamilton, Martin D. Curran, Theresa Feltwell, Anna Yakovleva, Charlotte J. Houldcroft, Aminu S. Jahun, Sarah L. Caddy, Ian Goodfellow
EPI_ISL_425449 hCoV-19/England/CAMB-76FF9/2020 Europe / United Kingdom / England 2020-04-01 Department of Pathology, University of Cambridge COVID-19 Genomics UK (COG-UK) Consortium Luke W Meredith, M. Estee Torok , Myra Hosmillo, William L. Hamilton, Martin D. Curran, Theresa Feltwell, Anna Yakovleva, Charlotte J. Houldcroft, Aminu S. Jahun, Sarah L. Caddy, Ian Goodfellow
EPI_ISL_425450 hCoV-19/England/CAMB-77004/2020 Europe / United Kingdom / England 2020-03-31 Department of Pathology, University of Cambridge COVID-19 Genomics UK (COG-UK) Consortium Luke W Meredith, M. Estee Torok , Myra Hosmillo, William L. Hamilton, Martin D. Curran, Theresa Feltwell, Anna Yakovleva, Charlotte J. Houldcroft, Aminu S. Jahun, Sarah L. Caddy, Ian Goodfellow
EPI_ISL_425451 hCoV-19/England/CAMB-77013/2020 Europe / United Kingdom / England 2020-04-01 Department of Pathology, University of Cambridge COVID-19 Genomics UK (COG-UK) Consortium Luke W Meredith, M. Estee Torok , Myra Hosmillo, William L. Hamilton, Martin D. Curran, Theresa Feltwell, Anna Yakovleva, Charlotte J. Houldcroft, Aminu S. Jahun, Sarah L. Caddy, Ian Goodfellow
EPI_ISL_425452 hCoV-19/England/CAMB-77022/2020 Europe / United Kingdom / England 2020-03-31 Department of Pathology, University of Cambridge COVID-19 Genomics UK (COG-UK) Consortium Luke W Meredith, M. Estee Torok , Myra Hosmillo, William L. Hamilton, Martin D. Curran, Theresa Feltwell, Anna Yakovleva, Charlotte J. Houldcroft, Aminu S. Jahun, Sarah L. Caddy, Ian Goodfellow

EPI_ISL_425453 hCoV-19/England/CAMB-77031/2020 Europe / United Kingdom / England 2020-04-01 Department of Pathology, University of Cambridge COVID-19 Genomics UK (COG-UK) Consortium Luke W Meredith, M. Estee Torok , Myra Hosmillo, William L. Hamilton, Martin D. Curran, Theresa Feltwell, Anna Yakovleva, Charlotte J. Houldcroft, Aminu S. Jahun, Sarah L. Caddy, Ian Goodfellow
EPI_ISL_425454 hCoV-19/England/CAMB-77040/2020 Europe / United Kingdom / England 2020-03-31 Department of Pathology, University of Cambridge COVID-19 Genomics UK (COG-UK) Consortium Luke W Meredith, M. Estee Torok , Myra Hosmillo, William L. Hamilton, Martin D. Curran, Theresa Feltwell, Anna Yakovleva, Charlotte J. Houldcroft, Aminu S. Jahun, Sarah L. Caddy, Ian Goodfellow
EPI_ISL_425455 hCoV-19/England/CAMB-7705F/2020 Europe / United Kingdom / England 2020-03-31 Department of Pathology, University of Cambridge COVID-19 Genomics UK (COG-UK) Consortium Luke W Meredith, M. Estee Torok , Myra Hosmillo, William L. Hamilton, Martin D. Curran, Theresa Feltwell, Anna Yakovleva, Charlotte J. Houldcroft, Aminu S. Jahun, Sarah L. Caddy, Ian Goodfellow
EPI_ISL_425457 hCoV-19/England/CAMB-7707D/2020 Europe / United Kingdom / England 2020-04-01 Department of Pathology, University of Cambridge COVID-19 Genomics UK (COG-UK) Consortium Luke W Meredith, M. Estee Torok , Myra Hosmillo, William L. Hamilton, Martin D. Curran, Theresa Feltwell, Anna Yakovleva, Charlotte J. Houldcroft, Aminu S. Jahun, Sarah L. Caddy, Ian Goodfellow
EPI_ISL_425458 hCoV-19/England/CAMB-7708C/2020 Europe / United Kingdom / England 2020-03-31 Department of Pathology, University of Cambridge COVID-19 Genomics UK (COG-UK) Consortium Luke W Meredith, M. Estee Torok , Myra Hosmillo, William L. Hamilton, Martin D. Curran, Theresa Feltwell, Anna Yakovleva, Charlotte J. Houldcroft, Aminu S. Jahun, Sarah L. Caddy, Ian Goodfellow
EPI_ISL_425460 hCoV-19/England/CAMB-770B9/2020 Europe / United Kingdom / England 2020-03-31 Department of Pathology, University of Cambridge COVID-19 Genomics UK (COG-UK) Consortium Luke W Meredith, M. Estee Torok , Myra Hosmillo, William L. Hamilton, Martin D. Curran, Theresa Feltwell, Anna Yakovleva, Charlotte J. Houldcroft, Aminu S. Jahun, Sarah L. Caddy, Ian Goodfellow
EPI_ISL_425466 hCoV-19/England/NOTT-10DEEA/2020 Europe / United Kingdom / England 2020-03-11 Queens Medical Centre, Clinical Microbiology Department / DeepSeq Nottingham COVID-19 Genomics UK (COG-UK) Consortium Gemma Clark, Wendy Smith, Manjinder Khakh, Hannah Howson-Wells, Jonathan Ball, Patrick McClure, Joseph Chappell, Theocharis Tsoleridis, Nadine Holmes, Matthew Carlisle, Christopher Moore, Fei Sang, Johnny Debebe, Victoria Wright, Matthew Loose
EPI_ISL_425468 hCoV-19/England/NOTT-10DF05/2020 Europe / United Kingdom / England 2020-03-12 Queens Medical Centre, Clinical Microbiology Department / DeepSeq Nottingham COVID-19 Genomics UK (COG-UK) Consortium Gemma Clark, Wendy Smith, Manjinder Khakh, Hannah Howson-Wells, Jonathan Ball, Patrick McClure, Joseph Chappell, Theocharis Tsoleridis, Nadine Holmes, Matthew Carlisle, Christopher Moore, Fei Sang, Johnny Debebe, Victoria Wright, Matthew Loose
EPI_ISL_425470 hCoV-19/England/NOTT-10DF23/2020 Europe / United Kingdom / England 2020-03-12 Queens Medical Centre, Clinical Microbiology Department / DeepSeq Nottingham COVID-19 Genomics UK (COG-UK) Consortium Gemma Clark, Wendy Smith, Manjinder Khakh, Hannah Howson-Wells, Jonathan Ball, Patrick McClure, Joseph Chappell, Theocharis Tsoleridis, Nadine Holmes, Matthew Carlisle, Christopher Moore, Fei Sang, Johnny Debebe, Victoria Wright, Matthew Loose
EPI_ISL_425471 hCoV-19/England/NOTT-10DF32/2020 Europe / United Kingdom / England 2020-03-13 Queens Medical Centre, Clinical Microbiology Department / DeepSeq Nottingham COVID-19 Genomics UK (COG-UK) Consortium Gemma Clark, Wendy Smith, Manjinder Khakh, Hannah Howson-Wells, Jonathan Ball, Patrick McClure, Joseph Chappell, Theocharis Tsoleridis, Nadine Holmes, Matthew Carlisle, Christopher Moore, Fei Sang, Johnny Debebe, Victoria Wright, Matthew Loose
EPI_ISL_425473 hCoV-19/England/NOTT-10DF50/2020 Europe / United Kingdom / England 2020-03-14 Queens Medical Centre, Clinical Microbiology Department / DeepSeq Nottingham COVID-19 Genomics UK (COG-UK) Consortium Gemma Clark, Wendy Smith, Manjinder Khakh, Hannah Howson-Wells, Jonathan Ball, Patrick McClure, Joseph Chappell, Theocharis Tsoleridis, Nadine Holmes, Matthew Carlisle, Christopher Moore, Fei Sang, Johnny Debebe, Victoria Wright, Matthew Loose
EPI_ISL_425474 hCoV-19/England/NOTT-10DF6F/2020 Europe / United Kingdom / England 2020-03-14 Queens Medical Centre, Clinical Microbiology Department / DeepSeq Nottingham COVID-19 Genomics UK (COG-UK) Consortium Gemma Clark, Wendy Smith, Manjinder Khakh, Hannah Howson-Wells, Jonathan Ball, Patrick McClure, Joseph Chappell, Theocharis Tsoleridis, Nadine Holmes, Matthew Carlisle, Christopher Moore, Fei Sang, Johnny Debebe, Victoria Wright, Matthew Loose
EPI_ISL_425475 hCoV-19/England/NOTT-10DF7E/2020 Europe / United Kingdom / England 2020-03-14 Queens Medical Centre, Clinical Microbiology Department / DeepSeq Nottingham COVID-19 Genomics UK (COG-UK) Consortium Gemma Clark, Wendy Smith, Manjinder Khakh, Hannah Howson-Wells, Jonathan Ball, Patrick McClure, Joseph Chappell, Theocharis Tsoleridis, Nadine Holmes, Matthew Carlisle, Christopher Moore, Fei Sang, Johnny Debebe, Victoria Wright, Matthew Loose
EPI_ISL_425476 hCoV-19/England/NOTT-10DF9C/2020 Europe / United Kingdom / England 2020-03-14 Queens Medical Centre, Clinical Microbiology Department / DeepSeq Nottingham COVID-19 Genomics UK (COG-UK) Consortium Gemma Clark, Wendy Smith, Manjinder Khakh, Hannah Howson-Wells, Jonathan Ball, Patrick McClure, Joseph Chappell, Theocharis Tsoleridis, Nadine Holmes, Matthew Carlisle, Christopher Moore, Fei Sang, Johnny Debebe, Victoria Wright, Matthew Loose
EPI_ISL_425477 hCoV-19/England/NOTT-10DFAB/2020 Europe / United Kingdom / England 2020-03-14 Queens Medical Centre, Clinical Microbiology Department / DeepSeq Nottingham COVID-19 Genomics UK (COG-UK) Consortium Gemma Clark, Wendy Smith, Manjinder Khakh, Hannah Howson-Wells, Jonathan Ball, Patrick McClure, Joseph Chappell, Theocharis Tsoleridis, Nadine Holmes, Matthew Carlisle, Christopher Moore, Fei Sang, Johnny Debebe, Victoria Wright, Matthew Loose
EPI_ISL_425480 hCoV-19/England/NOTT-10DFD8/2020 Europe / United Kingdom / England 2020-03-16

Queens Medical Centre, Clinical Microbiology Department / DeepSeq Nottingham   COVID-19 Genomics UK (COG-UK) Consortium   Gemma Clark, Wendy Smith, Manjinder Khakh, Hannah Howson-Wells, Jonathan Ball, Patrick McClure, Joseph Chappell, Theocharis Tsoleridis, Nadine Holmes, Matthew Carlisle, Christopher Moore, Fei Sang, Johnny Debebe, Victoria Wright, Matthew Loose
EPI_ISL_425481 hCoV-19/England/NOTT-10DFE7/2020   Europe / United Kingdom / England   2020-03-16   Queens Medical Centre, Clinical Microbiology Department / DeepSeq Nottingham   COVID-19 Genomics UK (COG-UK) Consortium   Gemma Clark, Wendy Smith, Manjinder Khakh, Hannah Howson-Wells, Jonathan Ball, Patrick McClure, Joseph Chappell, Theocharis Tsoleridis, Nadine Holmes, Matthew Carlisle, Christopher Moore, Fei Sang, Johnny Debebe, Victoria Wright, Matthew Loose
EPI_ISL_425482 hCoV-19/England/NOTT-10DFF6/2020   Europe / United Kingdom / England   2020-03-16   Queens Medical Centre, Clinical Microbiology Department / DeepSeq Nottingham   COVID-19 Genomics UK (COG-UK) Consortium   Gemma Clark, Wendy Smith, Manjinder Khakh, Hannah Howson-Wells, Jonathan Ball, Patrick McClure, Joseph Chappell, Theocharis Tsoleridis, Nadine Holmes, Matthew Carlisle, Christopher Moore, Fei Sang, Johnny Debebe, Victoria Wright, Matthew Loose
EPI_ISL_425483 hCoV-19/England/NOTT-10E001/2020   Europe / United Kingdom / England   2020-03-16   Queens Medical Centre, Clinical Microbiology Department / DeepSeq Nottingham   COVID-19 Genomics UK (COG-UK) Consortium   Gemma Clark, Wendy Smith, Manjinder Khakh, Hannah Howson-Wells, Jonathan Ball, Patrick McClure, Joseph Chappell, Theocharis Tsoleridis, Nadine Holmes, Matthew Carlisle, Christopher Moore, Fei Sang, Johnny Debebe, Victoria Wright, Matthew Loose
EPI_ISL_425484 hCoV-19/England/NOTT-10E010/2020   Europe / United Kingdom / England   2020-03-17   Queens Medical Centre, Clinical Microbiology Department / DeepSeq Nottingham   COVID-19 Genomics UK (COG-UK) Consortium   Gemma Clark, Wendy Smith, Manjinder Khakh, Hannah Howson-Wells, Jonathan Ball, Patrick McClure, Joseph Chappell, Theocharis Tsoleridis, Nadine Holmes, Matthew Carlisle, Christopher Moore, Fei Sang, Johnny Debebe, Victoria Wright, Matthew Loose
EPI_ISL_425486 hCoV-19/England/NOTT-10E03E/2020   Europe / United Kingdom / England   2020-03-17   Queens Medical Centre, Clinical Microbiology Department / DeepSeq Nottingham   COVID-19 Genomics UK (COG-UK) Consortium   Gemma Clark, Wendy Smith, Manjinder Khakh, Hannah Howson-Wells, Jonathan Ball, Patrick McClure, Joseph Chappell, Theocharis Tsoleridis, Nadine Holmes, Matthew Carlisle, Christopher Moore, Fei Sang, Johnny Debebe, Victoria Wright, Matthew Loose
EPI_ISL_425487 hCoV-19/England/NOTT-10E04D/2020   Europe / United Kingdom / England   2020-03-17   Queens Medical Centre, Clinical Microbiology Department / DeepSeq Nottingham   COVID-19 Genomics UK (COG-UK) Consortium   Gemma Clark, Wendy Smith, Manjinder Khakh, Hannah Howson-Wells, Jonathan Ball, Patrick McClure, Joseph Chappell, Theocharis Tsoleridis, Nadine Holmes, Matthew Carlisle, Christopher Moore, Fei Sang, Johnny Debebe, Victoria Wright, Matthew Loose
EPI_ISL_425488 hCoV-19/England/NOTT-10E05C/2020   Europe / United Kingdom / England   2020-03-18   Queens Medical Centre, Clinical Microbiology Department / DeepSeq Nottingham   COVID-19 Genomics UK (COG-UK) Consortium   Gemma Clark, Wendy Smith, Manjinder Khakh, Hannah Howson-Wells, Jonathan Ball, Patrick McClure, Joseph Chappell, Theocharis Tsoleridis, Nadine Holmes, Matthew Carlisle, Christopher Moore, Fei Sang, Johnny Debebe, Victoria Wright, Matthew Loose
EPI_ISL_425489 hCoV-19/England/NOTT-10E06B/2020   Europe / United Kingdom / England   2020-03-18   Queens Medical Centre, Clinical Microbiology Department / DeepSeq Nottingham   COVID-19 Genomics UK (COG-UK) Consortium   Gemma Clark, Wendy Smith, Manjinder Khakh, Hannah Howson-Wells, Jonathan Ball, Patrick McClure, Joseph Chappell, Theocharis Tsoleridis, Nadine Holmes, Matthew Carlisle, Christopher Moore, Fei Sang, Johnny Debebe, Victoria Wright, Matthew Loose
EPI_ISL_425491 hCoV-19/England/NOTT-10E089/2020   Europe / United Kingdom / England   2020-03-18   Queens Medical Centre, Clinical Microbiology Department / DeepSeq Nottingham   COVID-19 Genomics UK (COG-UK) Consortium   Gemma Clark, Wendy Smith, Manjinder Khakh, Hannah Howson-Wells, Jonathan Ball, Patrick McClure, Joseph Chappell, Theocharis Tsoleridis, Nadine Holmes, Matthew Carlisle, Christopher Moore, Fei Sang, Johnny Debebe, Victoria Wright, Matthew Loose
EPI_ISL_425492 hCoV-19/England/NOTT-10E098/2020   Europe / United Kingdom / England   2020-03-18   Queens Medical Centre, Clinical Microbiology Department / DeepSeq Nottingham   COVID-19 Genomics UK (COG-UK) Consortium   Gemma Clark, Wendy Smith, Manjinder Khakh, Hannah Howson-Wells, Jonathan Ball, Patrick McClure, Joseph Chappell, Theocharis Tsoleridis, Nadine Holmes, Matthew Carlisle, Christopher Moore, Fei Sang, Johnny Debebe, Victoria Wright, Matthew Loose
EPI_ISL_425494 hCoV-19/England/NOTT-10E0B6/2020   Europe / United Kingdom / England   2020-03-18   Queens Medical Centre, Clinical Microbiology Department / DeepSeq Nottingham   COVID-19 Genomics UK (COG-UK) Consortium   Gemma Clark, Wendy Smith, Manjinder Khakh, Hannah Howson-Wells, Jonathan Ball, Patrick McClure, Joseph Chappell, Theocharis Tsoleridis, Nadine Holmes, Matthew Carlisle, Christopher Moore, Fei Sang, Johnny Debebe, Victoria Wright, Matthew Loose
EPI_ISL_425495 hCoV-19/England/NOTT-10E0C5/2020   Europe / United Kingdom / England   2020-03-18   Queens Medical Centre, Clinical Microbiology Department / DeepSeq Nottingham   COVID-19 Genomics UK (COG-UK) Consortium   Gemma Clark, Wendy Smith, Manjinder Khakh, Hannah Howson-Wells, Jonathan Ball, Patrick McClure, Joseph Chappell, Theocharis Tsoleridis, Nadine Holmes, Matthew Carlisle, Christopher Moore, Fei Sang, Johnny Debebe, Victoria Wright, Matthew Loose
EPI_ISL_425496 hCoV-19/England/NOTT-10E0D4/2020   Europe / United Kingdom / England   2020-03-19   Queens Medical Centre, Clinical Microbiology Department / DeepSeq Nottingham   COVID-19 Genomics UK (COG-UK) Consortium   Gemma Clark, Wendy Smith, Manjinder Khakh, Hannah Howson-Wells, Jonathan Ball, Patrick McClure, Joseph Chappell, Theocharis Tsoleridis, Nadine Holmes, Matthew Carlisle, Christopher Moore, Fei Sang, Johnny Debebe, Victoria Wright, Matthew Loose
EPI_ISL_425497 hCoV-19/England/NOTT-10E0E3/2020   Europe / United Kingdom / England   2020-03-19

Queens Medical Centre, Clinical Microbiology Department / DeepSeq Nottingham COVID-19 Genomics UK (COG-UK) Consortium Gemma Clark, Wendy Smith, Manjinder Khakh, Hannah Howson-Wells, Jonathan Ball, Patrick McClure, Joseph Chappell, Theocharis Tsoleridis, Nadine Holmes, Matthew Carlisle, Christopher Moore, Fei Sang, Johnny Debebe, Victoria Wright, Matthew Loose
EPI_ISL_425498 hCoV-19/England/NOTT-10E0F2/2020 Europe / United Kingdom / England 2020-03-19 Queens Medical Centre, Clinical Microbiology Department / DeepSeq Nottingham COVID-19 Genomics UK (COG-UK) Consortium Gemma Clark, Wendy Smith, Manjinder Khakh, Hannah Howson-Wells, Jonathan Ball, Patrick McClure, Joseph Chappell, Theocharis Tsoleridis, Nadine Holmes, Matthew Carlisle, Christopher Moore, Fei Sang, Johnny Debebe, Victoria Wright, Matthew Loose
EPI_ISL_426630 hCoV-19/Taiwan/TSGH-02/2020 Asia / Taiwan / New Taipei city 2020-03-17 TSGH-CP molecular lab TSGH-CP molecular lab Cherng-Lih Perng, Ming-Jr Jian, Chih-Kai Chang, Jung-Chung Lin, Kuo-Ming Yeh, Chien-Wen Chen, Sheng-Kang Chiu, Hsing-Yi Chung, Shih-Hung Tsai, Kuo-Sheng Hung, Feng-Yee Chang, Hung-Sheng Shang
EPI_ISL_426631 hCoV-19/Taiwan/TSGH-03/2020 Asia / Taiwan / New Taipei City 2020-03-17 TSGH-CP molecular lab TSGH-CP molecular lab Cherng-Lih Perng, Ming-Jr Jian, Chih-Kai Chang, Jung-Chung Lin, Kuo-Ming Yeh, Chien-Wen Chen, Sheng-Kang Chiu, Hsing-Yi Chung, Shih-Hung Tsai, Kuo-Sheng Hung, Tien-Yao Chang, Feng-Yee Chang, Hung-Sheng Shang
EPI_ISL_426632 hCoV-19/Taiwan/TSGH-04/2020 Asia / Taiwan / New Taipei City 2020-03-17 TSGH-CP molecular lab TSGH-CP molecular lab Cherng-Lih Perng, Ming-Jr Jian, Chih-Kai Chang, Jung-Chung Lin, Kuo-Ming Yeh, Chien-Wen Chen, Sheng-Kang Chiu, Hsing-Yi Chung, Shih-Hung Tsai, Kuo-Sheng Hung, Tien-Yao Chang, Feng-Yee Chang, Hung-Sheng Shang
EPI_ISL_426634 hCoV-19/Australia/NT08/2020 Oceania / Australia / Northern territory 2020-03-20 Royal Darwin Hospital Pathology Microbiological Diagnostic Unit Public Health Laboratory and Victorian Infectious Diseases Reference Laboratory, Doherty Institute Meumann, E., Caly L., Seemann T., Sait, M., Schultz M., Druce J., Sherry, N.
EPI_ISL_426635 hCoV-19/Australia/NT18/2020 Oceania / Australia / Northern territory 2020-03-23 Royal Darwin Hospital Pathology Microbiological Diagnostic Unit Public Health Laboratory and Victorian Infectious Diseases Reference Laboratory, Doherty Institute Meumann, E., Caly L., Seemann T., Sait, M., Schultz M., Druce J., Sherry, N.
EPI_ISL_426636 hCoV-19/Australia/NT19/2020 Oceania / Australia / Northern territory 2020-03-25 Royal Darwin Hospital Pathology Microbiological Diagnostic Unit Public Health Laboratory and Victorian Infectious Diseases Reference Laboratory, Doherty Institute Meumann, E., Caly L., Seemann T., Sait, M., Schultz M., Druce J., Sherry, N.
EPI_ISL_426700 hCoV-19/Australia/VIC397/2020 Oceania / Australia / Victoria 2020-03-24 Victorian Infectious Diseases Reference Laboratory (VIDRL) Microbiological Diagnostic Unit Public Health Laboratory and Victorian Infectious Diseases Reference Laboratory, Doherty Institute Caly L., Seemann T., Sait, M., Schultz M., Druce J., Sherry, N.
EPI_ISL_426701 hCoV-19/Australia/VIC398/2020 Oceania / Australia / Victoria 2020-03-24 Victorian Infectious Diseases Reference Laboratory (VIDRL) Microbiological Diagnostic Unit Public Health Laboratory and Victorian Infectious Diseases Reference Laboratory, Doherty Institute Caly L., Seemann T., Sait, M., Schultz M., Druce J., Sherry, N.
EPI_ISL_426702 hCoV-19/Australia/VIC399/2020 Oceania / Australia / Victoria 2020-03-24 Victorian Infectious Diseases Reference Laboratory (VIDRL) Microbiological Diagnostic Unit Public Health Laboratory and Victorian Infectious Diseases Reference Laboratory, Doherty Institute Caly L., Seemann T., Sait, M., Schultz M., Druce J., Sherry, N.
EPI_ISL_426703 hCoV-19/Australia/VIC400/2020 Oceania / Australia / Victoria 2020-03-24 Victorian Infectious Diseases Reference Laboratory (VIDRL) Microbiological Diagnostic Unit Public Health Laboratory and Victorian Infectious Diseases Reference Laboratory, Doherty Institute Caly L., Seemann T., Sait, M., Schultz M., Druce J., Sherry, N.
EPI_ISL_426705 hCoV-19/Australia/VIC402/2020 Oceania / Australia / Victoria 2020-03-24 Victorian Infectious Diseases Reference Laboratory (VIDRL) Microbiological Diagnostic Unit Public Health Laboratory and Victorian Infectious Diseases Reference Laboratory, Doherty Institute Caly L., Seemann T., Sait, M., Schultz M., Druce J., Sherry, N.
EPI_ISL_426706 hCoV-19/Australia/VIC403/2020 Oceania / Australia / Victoria 2020-03-22 Victorian Infectious Diseases Reference Laboratory (VIDRL) Microbiological Diagnostic Unit Public Health Laboratory and Victorian Infectious Diseases Reference Laboratory, Doherty Institute Caly L., Seemann T., Sait, M., Schultz M., Druce J., Sherry, N.
EPI_ISL_426707 hCoV-19/Australia/VIC404/2020 Oceania / Australia / Victoria 2020-03-24 Victorian Infectious Diseases Reference Laboratory (VIDRL) Microbiological Diagnostic Unit Public Health Laboratory and Victorian Infectious Diseases Reference Laboratory, Doherty Institute Caly L., Seemann T., Sait, M., Schultz M., Druce J., Sherry, N.
EPI_ISL_426709 hCoV-19/Australia/VIC407/2020 Oceania / Australia / Victoria 2020-03-25 Victorian Infectious Diseases Reference Laboratory (VIDRL) Microbiological Diagnostic Unit Public Health Laboratory and Victorian Infectious Diseases Reference Laboratory, Doherty Institute Caly L., Seemann T., Sait, M., Schultz M., Druce J., Sherry, N.
EPI_ISL_426711 hCoV-19/Australia/VIC409/2020 Oceania / Australia / Victoria 2020-03-26 Victorian Infectious Diseases Reference Laboratory (VIDRL) Microbiological Diagnostic Unit Public Health Laboratory and Victorian Infectious Diseases Reference Laboratory, Doherty Institute Caly L., Seemann T., Sait, M., Schultz M., Druce J., Sherry, N.
EPI_ISL_426714 hCoV-19/Australia/VIC414/2020 Oceania / Australia / Victoria 2020-03-26 Victorian

Infectious Diseases Reference Laboratory (VIDRL)	Microbiological Diagnostic Unit Public Health Laboratory and Victorian Infectious Diseases Reference Laboratory, Doherty Institute	Caly L., Seemann T., Sait, M., Schultz M., Druce J., Sherry, N.
EPI_ISL_426715	hCoV-19/Australia/VIC415/2020	Oceania / Australia / Victoria	2020-03-25	Victorian Infectious Diseases Reference Laboratory (VIDRL)	Microbiological Diagnostic Unit Public Health Laboratory and Victorian Infectious Diseases Reference Laboratory, Doherty Institute	Caly L., Seemann T., Sait, M., Schultz M., Druce J., Sherry, N.
EPI_ISL_426717	hCoV-19/Australia/VIC417/2020	Oceania / Australia / Victoria	2020-03-25	Victorian Infectious Diseases Reference Laboratory (VIDRL)	Microbiological Diagnostic Unit Public Health Laboratory and Victorian Infectious Diseases Reference Laboratory, Doherty Institute	Caly L., Seemann T., Sait, M., Schultz M., Druce J., Sherry, N.
EPI_ISL_426718	hCoV-19/Australia/VIC418/2020	Oceania / Australia / Victoria	2020-03-25	Victorian Infectious Diseases Reference Laboratory (VIDRL)	Microbiological Diagnostic Unit Public Health Laboratory and Victorian Infectious Diseases Reference Laboratory, Doherty Institute	Caly L., Seemann T., Sait, M., Schultz M., Druce J., Sherry, N.
EPI_ISL_426721	hCoV-19/Australia/VIC421/2020	Oceania / Australia / Victoria	2020-03-25	Victorian Infectious Diseases Reference Laboratory (VIDRL)	Microbiological Diagnostic Unit Public Health Laboratory and Victorian Infectious Diseases Reference Laboratory, Doherty Institute	Caly L., Seemann T., Sait, M., Schultz M., Druce J., Sherry, N.
EPI_ISL_426725	hCoV-19/Australia/VIC425/2020	Oceania / Australia / Victoria	2020-03-25	Victorian Infectious Diseases Reference Laboratory (VIDRL)	Microbiological Diagnostic Unit Public Health Laboratory and Victorian Infectious Diseases Reference Laboratory, Doherty Institute	Caly L., Seemann T., Sait, M., Schultz M., Druce J., Sherry, N.
EPI_ISL_426727	hCoV-19/Australia/VIC427/2020	Oceania / Australia / Victoria	2020-03-25	Victorian Infectious Diseases Reference Laboratory (VIDRL)	Microbiological Diagnostic Unit Public Health Laboratory and Victorian Infectious Diseases Reference Laboratory, Doherty Institute	Caly L., Seemann T., Sait, M., Schultz M., Druce J., Sherry, N.
EPI_ISL_426729	hCoV-19/Australia/VIC429/2020	Oceania / Australia / Victoria	2020-03-26	Victorian Infectious Diseases Reference Laboratory (VIDRL)	Microbiological Diagnostic Unit Public Health Laboratory and Victorian Infectious Diseases Reference Laboratory, Doherty Institute	Caly L., Seemann T., Sait, M., Schultz M., Druce J., Sherry, N.
EPI_ISL_426730	hCoV-19/Australia/VIC430/2020	Oceania / Australia / Victoria	2020-03-26	Victorian Infectious Diseases Reference Laboratory (VIDRL)	Microbiological Diagnostic Unit Public Health Laboratory and Victorian Infectious Diseases Reference Laboratory, Doherty Institute	Caly L., Seemann T., Sait, M., Schultz M., Druce J., Sherry, N.
EPI_ISL_426732	hCoV-19/Australia/VIC433/2020	Oceania / Australia / Victoria	2020-03-25	Victorian Infectious Diseases Reference Laboratory (VIDRL)	Microbiological Diagnostic Unit Public Health Laboratory and Victorian Infectious Diseases Reference Laboratory, Doherty Institute	Caly L., Seemann T., Sait, M., Schultz M., Druce J., Sherry, N.
EPI_ISL_426733	hCoV-19/Australia/VIC434/2020	Oceania / Australia / Victoria	2020-03-25	Victorian Infectious Diseases Reference Laboratory (VIDRL)	Microbiological Diagnostic Unit Public Health Laboratory and Victorian Infectious Diseases Reference Laboratory, Doherty Institute	Caly L., Seemann T., Sait, M., Schultz M., Druce J., Sherry, N.
EPI_ISL_426734	hCoV-19/Australia/VIC437/2020	Oceania / Australia / Victoria	2020-03-26	Victorian Infectious Diseases Reference Laboratory (VIDRL)	Microbiological Diagnostic Unit Public Health Laboratory and Victorian Infectious Diseases Reference Laboratory, Doherty Institute	Caly L., Seemann T., Sait, M., Schultz M., Druce J., Sherry, N.
EPI_ISL_426735	hCoV-19/Australia/VIC438/2020	Oceania / Australia / Victoria	2020-03-26	Victorian Infectious Diseases Reference Laboratory (VIDRL)	Microbiological Diagnostic Unit Public Health Laboratory and Victorian Infectious Diseases Reference Laboratory, Doherty Institute	Caly L., Seemann T., Sait, M., Schultz M., Druce J., Sherry, N.
EPI_ISL_426736	hCoV-19/Australia/VIC439/2020	Oceania / Australia / Victoria	2020-03-26	Victorian Infectious Diseases Reference Laboratory (VIDRL)	Microbiological Diagnostic Unit Public Health Laboratory and Victorian Infectious Diseases Reference Laboratory, Doherty Institute	Caly L., Seemann T., Sait, M., Schultz M., Druce J., Sherry, N.
EPI_ISL_426737	hCoV-19/Australia/VIC440/2020	Oceania / Australia / Victoria	2020-03-26	Victorian Infectious Diseases Reference Laboratory (VIDRL)	Microbiological Diagnostic Unit Public Health Laboratory and Victorian Infectious Diseases Reference Laboratory, Doherty Institute	Caly L., Seemann T., Sait, M., Schultz M., Druce J., Sherry, N.
EPI_ISL_426740	hCoV-19/Australia/VIC443/2020	Oceania / Australia / Victoria	2020-03-27	Victorian Infectious Diseases Reference Laboratory (VIDRL)	Microbiological Diagnostic Unit Public Health Laboratory and Victorian Infectious Diseases Reference Laboratory, Doherty Institute	Caly L., Seemann T., Sait, M., Schultz M., Druce J., Sherry, N.
EPI_ISL_426741	hCoV-19/Australia/VIC444/2020	Oceania / Australia / Victoria	2020-03-27	Victorian Infectious Diseases Reference Laboratory (VIDRL)	Microbiological Diagnostic Unit Public Health Laboratory and Victorian Infectious Diseases Reference Laboratory, Doherty Institute	Caly L., Seemann T., Sait, M., Schultz M., Druce J., Sherry, N.
EPI_ISL_426743	hCoV-19/Australia/VIC447/2020	Oceania / Australia / Victoria	2020-03-27	Victorian Infectious Diseases Reference Laboratory (VIDRL)	Microbiological Diagnostic Unit Public Health Laboratory and Victorian Infectious Diseases Reference Laboratory, Doherty Institute	Caly L., Seemann T.,

Sait, M., Schultz M., Druce J., Sherry, N.
EPI_ISL_426745 hCoV-19/Australia/VIC449/2020 Oceania / Australia / Victoria 2020-03-24 Victorian Infectious Diseases Reference Laboratory (VIDRL) Microbiological Diagnostic Unit Public Health Laboratory and Victorian Infectious Diseases Reference Laboratory, Doherty Institute Caly L., Seemann T., Sait, M., Schultz M., Druce J., Sherry, N.
EPI_ISL_426746 hCoV-19/Australia/VIC450/2020 Oceania / Australia / Victoria 2020-03-25 Victorian Infectious Diseases Reference Laboratory (VIDRL) Microbiological Diagnostic Unit Public Health Laboratory and Victorian Infectious Diseases Reference Laboratory, Doherty Institute Caly L., Seemann T., Sait, M., Schultz M., Druce J., Sherry, N.
EPI_ISL_426747 hCoV-19/Australia/VIC451/2020 Oceania / Australia / Victoria 2020-03-25 Victorian Infectious Diseases Reference Laboratory (VIDRL) Microbiological Diagnostic Unit Public Health Laboratory and Victorian Infectious Diseases Reference Laboratory, Doherty Institute Caly L., Seemann T., Sait, M., Schultz M., Druce J., Sherry, N.
EPI_ISL_426751 hCoV-19/Australia/VIC456/2020 Oceania / Australia / Victoria 2020-03-25 Victorian Infectious Diseases Reference Laboratory (VIDRL) Microbiological Diagnostic Unit Public Health Laboratory and Victorian Infectious Diseases Reference Laboratory, Doherty Institute Caly L., Seemann T., Sait, M., Schultz M., Druce J., Sherry, N.
EPI_ISL_426752 hCoV-19/Australia/VIC458/2020 Oceania / Australia / Victoria 2020-03-26 Victorian Infectious Diseases Reference Laboratory (VIDRL) Microbiological Diagnostic Unit Public Health Laboratory and Victorian Infectious Diseases Reference Laboratory, Doherty Institute Caly L., Seemann T., Sait, M., Schultz M., Druce J., Sherry, N.
EPI_ISL_426754 hCoV-19/Australia/VIC461/2020 Oceania / Australia / Victoria 2020-03-27 Victorian Infectious Diseases Reference Laboratory (VIDRL) Microbiological Diagnostic Unit Public Health Laboratory and Victorian Infectious Diseases Reference Laboratory, Doherty Institute Caly L., Seemann T., Sait, M., Schultz M., Druce J., Sherry, N.
EPI_ISL_426756 hCoV-19/Australia/VIC463/2020 Oceania / Australia / Victoria 2020-03-26 Victorian Infectious Diseases Reference Laboratory (VIDRL) Microbiological Diagnostic Unit Public Health Laboratory and Victorian Infectious Diseases Reference Laboratory, Doherty Institute Caly L., Seemann T., Sait, M., Schultz M., Druce J., Sherry, N.
EPI_ISL_426761 hCoV-19/Australia/VIC468/2020 Oceania / Australia / Victoria 2020-03-27 Victorian Infectious Diseases Reference Laboratory (VIDRL) Microbiological Diagnostic Unit Public Health Laboratory and Victorian Infectious Diseases Reference Laboratory, Doherty Institute Caly L., Seemann T., Sait, M., Schultz M., Druce J., Sherry, N.
EPI_ISL_426763 hCoV-19/Australia/VIC471/2020 Oceania / Australia / Victoria 2020-03-26 Victorian Infectious Diseases Reference Laboratory (VIDRL) Microbiological Diagnostic Unit Public Health Laboratory and Victorian Infectious Diseases Reference Laboratory, Doherty Institute Caly L., Seemann T., Sait, M., Schultz M., Druce J., Sherry, N.
EPI_ISL_426764 hCoV-19/Australia/VIC472/2020 Oceania / Australia / Victoria 2020-03-26 Victorian Infectious Diseases Reference Laboratory (VIDRL) Microbiological Diagnostic Unit Public Health Laboratory and Victorian Infectious Diseases Reference Laboratory, Doherty Institute Caly L., Seemann T., Sait, M., Schultz M., Druce J., Sherry, N.
EPI_ISL_426765 hCoV-19/Australia/VIC492/2020 Oceania / Australia / Victoria 2020-03-23 Victorian Infectious Diseases Reference Laboratory (VIDRL) Microbiological Diagnostic Unit Public Health Laboratory and Victorian Infectious Diseases Reference Laboratory, Doherty Institute Caly L., Seemann T., Sait, M., Schultz M., Druce J., Sherry, N.
EPI_ISL_426766 hCoV-19/Australia/VIC493/2020 Oceania / Australia / Victoria 2020-03-22 Victorian Infectious Diseases Reference Laboratory (VIDRL) Microbiological Diagnostic Unit Public Health Laboratory and Victorian Infectious Diseases Reference Laboratory, Doherty Institute Caly L., Seemann T., Sait, M., Schultz M., Druce J., Sherry, N.
EPI_ISL_426767 hCoV-19/Australia/VIC494/2020 Oceania / Australia / Victoria 2020-03-23 Victorian Infectious Diseases Reference Laboratory (VIDRL) Microbiological Diagnostic Unit Public Health Laboratory and Victorian Infectious Diseases Reference Laboratory, Doherty Institute Caly L., Seemann T., Sait, M., Schultz M., Druce J., Sherry, N.
EPI_ISL_426768 hCoV-19/Australia/VIC495/2020 Oceania / Australia / Victoria 2020-03-23 Victorian Infectious Diseases Reference Laboratory (VIDRL) Microbiological Diagnostic Unit Public Health Laboratory and Victorian Infectious Diseases Reference Laboratory, Doherty Institute Caly L., Seemann T., Sait, M., Schultz M., Druce J., Sherry, N.
EPI_ISL_426769 hCoV-19/Australia/VIC496/2020 Oceania / Australia / Victoria 2020-03-23 Victorian Infectious Diseases Reference Laboratory (VIDRL) Microbiological Diagnostic Unit Public Health Laboratory and Victorian Infectious Diseases Reference Laboratory, Doherty Institute Caly L., Seemann T., Sait, M., Schultz M., Druce J., Sherry, N.
EPI_ISL_426770 hCoV-19/Australia/VIC497/2020 Oceania / Australia / Victoria 2020-03-23 Victorian Infectious Diseases Reference Laboratory (VIDRL) Microbiological Diagnostic Unit Public Health Laboratory and Victorian Infectious Diseases Reference Laboratory, Doherty Institute Caly L., Seemann T., Sait, M., Schultz M., Druce J., Sherry, N.
EPI_ISL_426771 hCoV-19/Australia/VIC498/2020 Oceania / Australia / Victoria 2020-03-23 Victorian Infectious Diseases Reference Laboratory (VIDRL) Microbiological Diagnostic Unit Public Health Laboratory and Victorian Infectious Diseases Reference Laboratory, Doherty Institute Caly L., Seemann T., Sait, M., Schultz M., Druce J., Sherry, N.
EPI_ISL_426772 hCoV-19/Australia/VIC500/2020 Oceania / Australia / Victoria 2020-03-24 Victorian

Infectious Diseases Reference Laboratory (VIDRL)	Microbiological Diagnostic Unit Public Health Laboratory and Victorian Infectious Diseases Reference Laboratory, Doherty Institute	Caly L., Seemann T., Sait, M., Schultz M., Druce J., Sherry, N.
EPI_ISL_426774	hCoV-19/Australia/VIC503/2020	Oceania / Australia / Victoria	2020-03-23	Victorian Infectious Diseases Reference Laboratory (VIDRL)	Microbiological Diagnostic Unit Public Health Laboratory and Victorian Infectious Diseases Reference Laboratory, Doherty Institute	Caly L., Seemann T., Sait, M., Schultz M., Druce J., Sherry, N.
EPI_ISL_426775	hCoV-19/Australia/VIC504/2020	Oceania / Australia / Victoria	2020-03-23	Victorian Infectious Diseases Reference Laboratory (VIDRL)	Microbiological Diagnostic Unit Public Health Laboratory and Victorian Infectious Diseases Reference Laboratory, Doherty Institute	Caly L., Seemann T., Sait, M., Schultz M., Druce J., Sherry, N.
EPI_ISL_426777	hCoV-19/Australia/VIC506/2020	Oceania / Australia / Victoria	2020-03-23	Victorian Infectious Diseases Reference Laboratory (VIDRL)	Microbiological Diagnostic Unit Public Health Laboratory and Victorian Infectious Diseases Reference Laboratory, Doherty Institute	Caly L., Seemann T., Sait, M., Schultz M., Druce J., Sherry, N.
EPI_ISL_426779	hCoV-19/Australia/VIC509/2020	Oceania / Australia / Victoria	2020-03-22	Victorian Infectious Diseases Reference Laboratory (VIDRL)	Microbiological Diagnostic Unit Public Health Laboratory and Victorian Infectious Diseases Reference Laboratory, Doherty Institute	Caly L., Seemann T., Sait, M., Schultz M., Druce J., Sherry, N.
EPI_ISL_426781	hCoV-19/Australia/VIC511/2020	Oceania / Australia / Victoria	2020-03-23	Victorian Infectious Diseases Reference Laboratory (VIDRL)	Microbiological Diagnostic Unit Public Health Laboratory and Victorian Infectious Diseases Reference Laboratory, Doherty Institute	Caly L., Seemann T., Sait, M., Schultz M., Druce J., Sherry, N.
EPI_ISL_426786	hCoV-19/Australia/VIC516/2020	Oceania / Australia / Victoria	2020-03-23	Victorian Infectious Diseases Reference Laboratory (VIDRL)	Microbiological Diagnostic Unit Public Health Laboratory and Victorian Infectious Diseases Reference Laboratory, Doherty Institute	Caly L., Seemann T., Sait, M., Schultz M., Druce J., Sherry, N.
EPI_ISL_426788	hCoV-19/Australia/VIC518/2020	Oceania / Australia / Victoria	2020-03-23	Victorian Infectious Diseases Reference Laboratory (VIDRL)	Microbiological Diagnostic Unit Public Health Laboratory and Victorian Infectious Diseases Reference Laboratory, Doherty Institute	Caly L., Seemann T., Sait, M., Schultz M., Druce J., Sherry, N.
EPI_ISL_426789	hCoV-19/Australia/VIC520/2020	Oceania / Australia / Victoria	2020-03-23	Victorian Infectious Diseases Reference Laboratory (VIDRL)	Microbiological Diagnostic Unit Public Health Laboratory and Victorian Infectious Diseases Reference Laboratory, Doherty Institute	Caly L., Seemann T., Sait, M., Schultz M., Druce J., Sherry, N.
EPI_ISL_426790	hCoV-19/Australia/VIC522/2020	Oceania / Australia / Victoria	2020-03-23	Victorian Infectious Diseases Reference Laboratory (VIDRL)	Microbiological Diagnostic Unit Public Health Laboratory and Victorian Infectious Diseases Reference Laboratory, Doherty Institute	Caly L., Seemann T., Sait, M., Schultz M., Druce J., Sherry, N.
EPI_ISL_426792	hCoV-19/Australia/VIC525/2020	Oceania / Australia / Victoria	2020-03-24	Victorian Infectious Diseases Reference Laboratory (VIDRL)	Microbiological Diagnostic Unit Public Health Laboratory and Victorian Infectious Diseases Reference Laboratory, Doherty Institute	Caly L., Seemann T., Sait, M., Schultz M., Druce J., Sherry, N.
EPI_ISL_426793	hCoV-19/Australia/VIC527/2020	Oceania / Australia / Victoria	2020-03-24	Victorian Infectious Diseases Reference Laboratory (VIDRL)	Microbiological Diagnostic Unit Public Health Laboratory and Victorian Infectious Diseases Reference Laboratory, Doherty Institute	Caly L., Seemann T., Sait, M., Schultz M., Druce J., Sherry, N.
EPI_ISL_426796	hCoV-19/Australia/VIC530/2020	Oceania / Australia / Victoria	2020-03-23	Victorian Infectious Diseases Reference Laboratory (VIDRL)	Microbiological Diagnostic Unit Public Health Laboratory and Victorian Infectious Diseases Reference Laboratory, Doherty Institute	Caly L., Seemann T., Sait, M., Schultz M., Druce J., Sherry, N.
EPI_ISL_426797	hCoV-19/Australia/VIC531/2020	Oceania / Australia / Victoria	2020-03-24	Victorian Infectious Diseases Reference Laboratory (VIDRL)	Microbiological Diagnostic Unit Public Health Laboratory and Victorian Infectious Diseases Reference Laboratory, Doherty Institute	Caly L., Seemann T., Sait, M., Schultz M., Druce J., Sherry, N.
EPI_ISL_426798	hCoV-19/Australia/VIC532/2020	Oceania / Australia / Victoria	2020-03-23	Victorian Infectious Diseases Reference Laboratory (VIDRL)	Microbiological Diagnostic Unit Public Health Laboratory and Victorian Infectious Diseases Reference Laboratory, Doherty Institute	Caly L., Seemann T., Sait, M., Schultz M., Druce J., Sherry, N.
EPI_ISL_426799	hCoV-19/Australia/VIC533/2020	Oceania / Australia / Victoria	2020-03-24	Victorian Infectious Diseases Reference Laboratory (VIDRL)	Microbiological Diagnostic Unit Public Health Laboratory and Victorian Infectious Diseases Reference Laboratory, Doherty Institute	Caly L., Seemann T., Sait, M., Schultz M., Druce J., Sherry, N.
EPI_ISL_428201	hCoV-19/Sweden/RV-FOI-6/2020	Europe / Sweden / Västerbotten	2020-03-20	Klinisk mikrobiologi, Region Västerbotten	Unit for Biological Agents, Department for CBRN Defence and Security, Swedish Defence Research Agency	FOI Bioinformatics team
EPI_ISL_428229	hCoV-19/Taiwan/TSGH-12/2020	Asia / Taiwan / Taipei	2020-03-18	TSGH-CP molecular lab	TSGH-CP molecular lab	Cherng-Lih Perng, Ming-Jr Jian, Chih-Kai Chang, Jung-Chung Lin, Kuo-Ming Yeh, Chien-Wen Chen, Sheng-Kang Chiu, Hsing-Yi Chung, Shih-Hung Tsai, Kuo-Sheng Hung, Tien-Yao Chang, Feng-Yee Chang, Hung-Sheng Shang

EPI_ISL_428230 hCoV-19/Taiwan/TSGH-13/2020 Asia / Taiwan / New Taipei City 2020-03-17 TSGH-CP molecular lab TSGH-CP molecular lab Cherng-Lih Perng, Ming-Jr Jian, Chih-Kai Chang, Jung-Chung Lin, Kuo-Ming Yeh, Chien-Wen Chen, Sheng-Kang Chiu, Hsing-Yi Chung, Shih-Hung Tsai, Kuo-Sheng Hung, Tien-Yao Chang, Feng-Yee Chang, Hung-Sheng Shang
EPI_ISL_428231 hCoV-19/Taiwan/TSGH-14/2020 Asia / Taiwan / New Taipei City 2020-03-24 TSGH-CP molecular lab TSGH-CP molecular lab Cherng-Lih Perng, Ming-Jr JIAN, Chih-Kai Chang, Jung-Chung Lin, Kuo-Ming Yeh, Chien-Wen Chen, Sheng-Kang Chiu, Hsing-Yi Chung, Shih-Hung Tsai, Kuo-Sheng Hung, Tien-Yao Chang, Feng-Yee Chang, Hung-Sheng Shang
EPI_ISL_428252 hCoV-19/USA/WI-UW-156/2020 North America / USA / Wisconsin / Wauwatosa 2020-03-25 University of Wisconsin-Madison AIDS Vaccine Research Laboratories University of Wisconsin-Madison AIDS Vaccine Research Laboratories Gage Moreno, Katarina Braun, et al. AIDS Vaccine Research Laboratories
EPI_ISL_428253 hCoV-19/USA/WI-UW-157/2020 North America / USA / Wisconsin / Milwaukee 2020-03-15 University of Wisconsin-Madison AIDS Vaccine Research Laboratories University of Wisconsin-Madison AIDS Vaccine Research Laboratories Gage Moreno, Katarina Braun, et al. AIDS Vaccine Research Laboratories
EPI_ISL_428254 hCoV-19/USA/WI-UW-158/2020 North America / USA / Wisconsin / Oak Creek 2020-03-15 University of Wisconsin-Madison AIDS Vaccine Research Laboratories University of Wisconsin-Madison AIDS Vaccine Research Laboratories Gage Moreno, Katarina Braun, et al. AIDS Vaccine Research Laboratories
EPI_ISL_428255 hCoV-19/USA/WI-UW-159/2020 North America / USA / Wisconsin / Oak Creek 2020-03-15 University of Wisconsin-Madison AIDS Vaccine Research Laboratories University of Wisconsin-Madison AIDS Vaccine Research Laboratories Gage Moreno, Katarina Braun, et al. AIDS Vaccine Research Laboratories
EPI_ISL_428256 hCoV-19/USA/WI-UW-160/2020 North America / USA / Wisconsin / Oak Creek 2020-03-15 University of Wisconsin-Madison AIDS Vaccine Research Laboratories University of Wisconsin-Madison AIDS Vaccine Research Laboratories Gage Moreno, Katarina Braun, et al. AIDS Vaccine Research Laboratories
EPI_ISL_428257 hCoV-19/USA/WI-UW-161/2020 North America / USA / Wisconsin / Milwaukee 2020-03-29 University of Wisconsin-Madison AIDS Vaccine Research Laboratories University of Wisconsin-Madison AIDS Vaccine Research Laboratories Gage Moreno, Katarina Braun, et al. AIDS Vaccine Research Laboratories
EPI_ISL_428258 hCoV-19/USA/WI-UW-162/2020 North America / USA / Wisconsin / Milwaukee 2020-03-29 University of Wisconsin-Madison AIDS Vaccine Research Laboratories University of Wisconsin-Madison AIDS Vaccine Research Laboratories Gage Moreno, Katarina Braun, et al. AIDS Vaccine Research Laboratories
EPI_ISL_428259 hCoV-19/USA/WI-UW-163/2020 North America / USA / Wisconsin / Elm Grove 2020-03-16 University of Wisconsin-Madison AIDS Vaccine Research Laboratories University of Wisconsin-Madison AIDS Vaccine Research Laboratories Gage Moreno, Katarina Braun, et al. AIDS Vaccine Research Laboratories
EPI_ISL_428260 hCoV-19/USA/WI-UW-164/2020 North America / USA / Wisconsin / Pewaukee 2020-03-16 University of Wisconsin-Madison AIDS Vaccine Research Laboratories University of Wisconsin-Madison AIDS Vaccine Research Laboratories Gage Moreno, Katarina Braun, et al. AIDS Vaccine Research Laboratories
EPI_ISL_428261 hCoV-19/USA/WI-UW-165/2020 North America / USA / Wisconsin / Milwaukee 2020-03-17 University of Wisconsin-Madison AIDS Vaccine Research Laboratories University of Wisconsin-Madison AIDS Vaccine Research Laboratories Gage Moreno, Katarina Braun, et al. AIDS Vaccine Research Laboratories
EPI_ISL_428262 hCoV-19/USA/WI-UW-166/2020 North America / USA / Wisconsin / Greenfield 2020-03-17 University of Wisconsin-Madison AIDS Vaccine Research Laboratories University of Wisconsin-Madison AIDS Vaccine Research Laboratories Gage Moreno, Katarina Braun, et al. AIDS Vaccine Research Laboratories
EPI_ISL_428263 hCoV-19/USA/WI-UW-167/2020 North America / USA / Wisconsin / New Berlin 2020-03-18 University of Wisconsin-Madison AIDS Vaccine Research Laboratories University of Wisconsin-Madison AIDS Vaccine Research Laboratories Gage Moreno, Katarina Braun, et al. AIDS Vaccine Research Laboratories
EPI_ISL_428264 hCoV-19/USA/WI-UW-168/2020 North America / USA / Wisconsin / Milwaukee 2020-03-19 University of Wisconsin-Madison AIDS Vaccine Research Laboratories University of Wisconsin-Madison AIDS Vaccine Research Laboratories Gage Moreno, Katarina Braun, et al. AIDS Vaccine Research Laboratories
EPI_ISL_428265 hCoV-19/USA/WI-UW-169/2020 North America / USA / Wisconsin / Grafton 2020-03-19 University of Wisconsin-Madison AIDS Vaccine Research Laboratories University of Wisconsin-Madison AIDS Vaccine Research Laboratories Gage Moreno, Katarina Braun, et al. AIDS Vaccine Research Laboratories
EPI_ISL_428266 hCoV-19/USA/WI-UW-170/2020 North America / USA / Wisconsin / Milwaukee 2020-03-19 University of Wisconsin-Madison AIDS Vaccine Research Laboratories University of Wisconsin-Madison AIDS Vaccine Research Laboratories Gage Moreno, Katarina Braun, et al. AIDS Vaccine Research Laboratories
EPI_ISL_428267 hCoV-19/USA/WI-UW-171/2020 North America / USA / Wisconsin / Milwaukee 2020-03-19 University of Wisconsin-Madison AIDS Vaccine Research Laboratories University of Wisconsin-Madison AIDS Vaccine Research Laboratories Gage Moreno, Katarina Braun, et al. AIDS Vaccine Research Laboratories
EPI_ISL_428268 hCoV-19/USA/WI-UW-172/2020 North America / USA / Wisconsin / Milwaukee 2020-03-19 University of Wisconsin-Madison AIDS Vaccine Research Laboratories University of Wisconsin-Madison AIDS Vaccine Research Laboratories Gage Moreno, Katarina Braun, et al. AIDS Vaccine Research Laboratories
EPI_ISL_428269 hCoV-19/USA/WI-UW-173/2020 North America / USA / Wisconsin / Milwaukee 2020-03-20 University of Wisconsin-Madison AIDS Vaccine Research Laboratories University of Wisconsin-Madison AIDS Vaccine Research Laboratories Gage Moreno, Katarina Braun, et al. AIDS Vaccine Research Laboratories
EPI_ISL_428270 hCoV-19/USA/WI-UW-174/2020 North America / USA / Wisconsin / Grafton 2020-03-20 University of Wisconsin-Madison AIDS Vaccine Research Laboratories University of Wisconsin-Madison AIDS Vaccine Research Laboratories Gage Moreno, Katarina Braun, et al. AIDS Vaccine Research Laboratories
EPI_ISL_428271 hCoV-19/USA/WI-UW-175/2020 North America / USA / Wisconsin / Grafton 2020-03-20 University of Wisconsin-Madison AIDS Vaccine Research Laboratories University of Wisconsin-Madison AIDS Vaccine Research Laboratories Gage Moreno, Katarina Braun, et al. AIDS Vaccine Research Laboratories
EPI_ISL_428272 hCoV-19/USA/WI-UW-176/2020 North America / USA / Wisconsin / Milwaukee 2020-03-20 University of Wisconsin-Madison AIDS Vaccine Research Laboratories University of Wisconsin-Madison AIDS

Vaccine Research Laboratories	Gage Moreno, Katarina Braun, et al. AIDS Vaccine Research Laboratories
EPI_ISL_428273	hCoV-19/USA/WI-UW-177/2020	North America / USA / Wisconsin / Milwaukee	2020-03-20
University of Wisconsin-Madison AIDS Vaccine Research Laboratories	University of Wisconsin-Madison AIDS Vaccine Research Laboratories	Gage Moreno, Katarina Braun, et al. AIDS Vaccine Research Laboratories
EPI_ISL_428274	hCoV-19/USA/WI-UW-178/2020	North America / USA / Wisconsin / Milwaukee	2020-03-20
University of Wisconsin-Madison AIDS Vaccine Research Laboratories	University of Wisconsin-Madison AIDS Vaccine Research Laboratories	Gage Moreno, Katarina Braun, et al. AIDS Vaccine Research Laboratories
EPI_ISL_428275	hCoV-19/USA/WI-UW-179/2020	North America / USA / Wisconsin / Milwaukee	2020-03-21
University of Wisconsin-Madison AIDS Vaccine Research Laboratories	University of Wisconsin-Madison AIDS Vaccine Research Laboratories	Gage Moreno, Katarina Braun, et al. AIDS Vaccine Research Laboratories
EPI_ISL_428276	hCoV-19/USA/WI-UW-180/2020	North America / USA / Wisconsin / Grafton	2020-03-21
University of Wisconsin-Madison AIDS Vaccine Research Laboratories	University of Wisconsin-Madison AIDS Vaccine Research Laboratories	Gage Moreno, Katarina Braun, et al. AIDS Vaccine Research Laboratories
EPI_ISL_428277	hCoV-19/USA/WI-UW-181/2020	North America / USA / Wisconsin / Milwaukee	2020-03-22
University of Wisconsin-Madison AIDS Vaccine Research Laboratories	University of Wisconsin-Madison AIDS Vaccine Research Laboratories	Gage Moreno, Katarina Braun, et al. AIDS Vaccine Research Laboratories
EPI_ISL_428278	hCoV-19/USA/WI-UW-182/2020	North America / USA / Wisconsin / Milwaukee	2020-03-22
University of Wisconsin-Madison AIDS Vaccine Research Laboratories	University of Wisconsin-Madison AIDS Vaccine Research Laboratories	Gage Moreno, Katarina Braun, et al. AIDS Vaccine Research Laboratories
EPI_ISL_428279	hCoV-19/USA/WI-UW-183/2020	North America / USA / Wisconsin / Milwaukee	2020-03-22
University of Wisconsin-Madison AIDS Vaccine Research Laboratories	University of Wisconsin-Madison AIDS Vaccine Research Laboratories	Gage Moreno, Katarina Braun, et al. AIDS Vaccine Research Laboratories
EPI_ISL_428280	hCoV-19/USA/WI-UW-184/2020	North America / USA / Wisconsin / Milwaukee	2020-03-22
University of Wisconsin-Madison AIDS Vaccine Research Laboratories	University of Wisconsin-Madison AIDS Vaccine Research Laboratories	Gage Moreno, Katarina Braun, et al. AIDS Vaccine Research Laboratories
EPI_ISL_428281	hCoV-19/USA/WI-UW-185/2020	North America / USA / Wisconsin / Milwaukee	2020-03-22
University of Wisconsin-Madison AIDS Vaccine Research Laboratories	University of Wisconsin-Madison AIDS Vaccine Research Laboratories	Gage Moreno, Katarina Braun, et al. AIDS Vaccine Research Laboratories
EPI_ISL_428282	hCoV-19/USA/WI-UW-186/2020	North America / USA / Wisconsin / Milwaukee	2020-03-23
University of Wisconsin-Madison AIDS Vaccine Research Laboratories	University of Wisconsin-Madison AIDS Vaccine Research Laboratories	Gage Moreno, Katarina Braun, et al. AIDS Vaccine Research Laboratories
EPI_ISL_428283	hCoV-19/USA/WI-UW-187/2020	North America / USA / Wisconsin / Milwaukee	2020-03-23
University of Wisconsin-Madison AIDS Vaccine Research Laboratories	University of Wisconsin-Madison AIDS Vaccine Research Laboratories	Gage Moreno, Katarina Braun, et al. AIDS Vaccine Research Laboratories
EPI_ISL_428284	hCoV-19/USA/WI-UW-188/2020	North America / USA / Wisconsin / Milwaukee	2020-03-23
University of Wisconsin-Madison AIDS Vaccine Research Laboratories	University of Wisconsin-Madison AIDS Vaccine Research Laboratories	Gage Moreno, Katarina Braun, et al. AIDS Vaccine Research Laboratories
EPI_ISL_428285	hCoV-19/USA/WI-UW-189/2020	North America / USA / Wisconsin / Milwaukee	2020-03-23
University of Wisconsin-Madison AIDS Vaccine Research Laboratories	University of Wisconsin-Madison AIDS Vaccine Research Laboratories	Gage Moreno, Katarina Braun, et al. AIDS Vaccine Research Laboratories
EPI_ISL_428286	hCoV-19/USA/WI-UW-190/2020	North America / USA / Wisconsin / Wauwatosa	2020-03-23
University of Wisconsin-Madison AIDS Vaccine Research Laboratories	University of Wisconsin-Madison AIDS Vaccine Research Laboratories	Gage Moreno, Katarina Braun, et al. AIDS Vaccine Research Laboratories
EPI_ISL_428287	hCoV-19/USA/WI-UW-191/2020	North America / USA / Wisconsin / Milwaukee	2020-03-23
University of Wisconsin-Madison AIDS Vaccine Research Laboratories	University of Wisconsin-Madison AIDS Vaccine Research Laboratories	Gage Moreno, Katarina Braun, et al. AIDS Vaccine Research Laboratories
EPI_ISL_428288	hCoV-19/USA/WI-UW-192/2020	North America / USA / Wisconsin / Milwaukee	2020-03-23
University of Wisconsin-Madison AIDS Vaccine Research Laboratories	University of Wisconsin-Madison AIDS Vaccine Research Laboratories	Gage Moreno, Katarina Braun, et al. AIDS Vaccine Research Laboratories
EPI_ISL_428289	hCoV-19/USA/WI-UW-193/2020	North America / USA / Wisconsin / Jackson	2020-03-23
University of Wisconsin-Madison AIDS Vaccine Research Laboratories	University of Wisconsin-Madison AIDS Vaccine Research Laboratories	Gage Moreno, Katarina Braun, et al. AIDS Vaccine Research Laboratories
EPI_ISL_428290	hCoV-19/USA/WI-UW-194/2020	North America / USA / Wisconsin / Grafton	2020-03-24
University of Wisconsin-Madison AIDS Vaccine Research Laboratories	University of Wisconsin-Madison AIDS Vaccine Research Laboratories	Gage Moreno, Katarina Braun, et al. AIDS Vaccine Research Laboratories
EPI_ISL_428291	hCoV-19/USA/WI-UW-195/2020	North America / USA / Wisconsin / Milwaukee	2020-03-24
University of Wisconsin-Madison AIDS Vaccine Research Laboratories	University of Wisconsin-Madison AIDS Vaccine Research Laboratories	Gage Moreno, Katarina Braun, et al. AIDS Vaccine Research Laboratories
EPI_ISL_428292	hCoV-19/USA/WI-UW-196/2020	North America / USA / Wisconsin / Milwaukee	2020-03-21
University of Wisconsin-Madison AIDS Vaccine Research Laboratories	University of Wisconsin-Madison AIDS Vaccine Research Laboratories	Gage Moreno, Katarina Braun, et al. AIDS Vaccine Research Laboratories
EPI_ISL_428293	hCoV-19/USA/WI-UW-197/2020	North America / USA / Wisconsin / Greenfield	2020-03-24
University of Wisconsin-Madison AIDS Vaccine Research Laboratories	University of Wisconsin-Madison AIDS Vaccine Research Laboratories	Gage Moreno, Katarina Braun, et al. AIDS Vaccine Research Laboratories
EPI_ISL_428294	hCoV-19/USA/WI-UW-198/2020	North America / USA / Wisconsin / Brookfield	2020-03-24
University of Wisconsin-Madison AIDS Vaccine Research Laboratories	University of Wisconsin-Madison AIDS Vaccine Research Laboratories	Gage Moreno, Katarina Braun, et al. AIDS Vaccine Research Laboratories
EPI_ISL_428295	hCoV-19/USA/WI-UW-199/2020	North America / USA / Wisconsin / Brookfield	2020-03-24
University of Wisconsin-Madison AIDS Vaccine Research Laboratories	University of Wisconsin-Madison AIDS Vaccine Research Laboratories	Gage Moreno, Katarina Braun, et al. AIDS Vaccine Research Laboratories

EPI_ISL_428296 hCoV-19/USA/WI-UW-200/2020 North America / USA / Wisconsin / Milwaukee 2020-03-24 University of Wisconsin-Madison AIDS Vaccine Research Laboratories University of Wisconsin-Madison AIDS Vaccine Research Laboratories Gage Moreno, Katarina Braun, et al. AIDS Vaccine Research Laboratories
EPI_ISL_428297 hCoV-19/USA/WI-UW-201/2020 North America / USA / Wisconsin / Grafton 2020-03-25 University of Wisconsin-Madison AIDS Vaccine Research Laboratories University of Wisconsin-Madison AIDS Vaccine Research Laboratories Gage Moreno, Katarina Braun, et al. AIDS Vaccine Research Laboratories
EPI_ISL_428298 hCoV-19/USA/WI-UW-202/2020 North America / USA / Wisconsin / Grafton 2020-03-25 University of Wisconsin-Madison AIDS Vaccine Research Laboratories University of Wisconsin-Madison AIDS Vaccine Research Laboratories Gage Moreno, Katarina Braun, et al. AIDS Vaccine Research Laboratories
EPI_ISL_428299 hCoV-19/USA/WI-UW-203/2020 North America / USA / Wisconsin / Grafton 2020-03-25 University of Wisconsin-Madison AIDS Vaccine Research Laboratories University of Wisconsin-Madison AIDS Vaccine Research Laboratories Gage Moreno, Katarina Braun, et al. AIDS Vaccine Research Laboratories
EPI_ISL_429000 hCoV-19/USA/CA-CZB0103/2020 North America / USA / California / San Francisco 2020-03-28 UCSF Clinical Microbiology Laboratory Chan-Zuckerberg Biohub CZB Cliahub Consortium
EPI_ISL_429001 hCoV-19/USA/CA-CZB046/2020 North America / USA / California / San Francisco 2020-03-25 UCSF Clinical Microbiology Laboratory Chan-Zuckerberg Biohub CZB Cliahub Consortium
EPI_ISL_429002 hCoV-19/USA/CA-CZB098/2020 North America / USA / California / San Francisco 2020-03-28 UCSF Clinical Microbiology Laboratory Chan-Zuckerberg Biohub CZB Cliahub Consortium
EPI_ISL_429003 hCoV-19/USA/CA-CZB0136/2020 North America / USA / California / San Francisco 2020-04-08 UCSF Clinical Microbiology Laboratory Chan-Zuckerberg Biohub CZB Cliahub Consortium
EPI_ISL_429004 hCoV-19/USA/CA-CZB085/2020 North America / USA / California / San Francisco 2020-03-28 UCSF Clinical Microbiology Laboratory Chan-Zuckerberg Biohub CZB Cliahub Consortium
EPI_ISL_429005 hCoV-19/USA/CA-CZB079/2020 North America / USA / California / San Francisco 2020-03-28 UCSF Clinical Microbiology Laboratory Chan-Zuckerberg Biohub CZB Cliahub Consortium
EPI_ISL_429006 hCoV-19/USA/CA-CZB095/2020 North America / USA / California / San Francisco 2020-03-28 UCSF Clinical Microbiology Laboratory Chan-Zuckerberg Biohub CZB Cliahub Consortium
EPI_ISL_429007 hCoV-19/USA/CA-CZB0458/2020 North America / USA / California / San Francisco 2020-03-18 UCSF Clinical Microbiology Laboratory Chan-Zuckerberg Biohub CZB Cliahub Consortium
EPI_ISL_429008 hCoV-19/USA/CA-CZB091/2020 North America / USA / California / San Francisco 2020-03-28 UCSF Clinical Microbiology Laboratory Chan-Zuckerberg Biohub CZB Cliahub Consortium
EPI_ISL_429009 hCoV-19/USA/CA-CZB0122/2020 North America / USA / California / San Francisco 2020-04-08 UCSF Clinical Microbiology Laboratory Chan-Zuckerberg Biohub CZB Cliahub Consortium
EPI_ISL_429010 hCoV-19/USA/CA-CZB0108/2020 North America / USA / California / San Francisco 2020-03-28 UCSF Clinical Microbiology Laboratory Chan-Zuckerberg Biohub CZB Cliahub Consortium
EPI_ISL_429011 hCoV-19/USA/CA-CZB0147/2020 North America / USA / California / San Francisco 2020-04-08 UCSF Clinical Microbiology Laboratory Chan-Zuckerberg Biohub CZB Cliahub Consortium
EPI_ISL_429012 hCoV-19/USA/CA-CZB016/2020 North America / USA / California / San Francisco 2020-03-25 UCSF Clinical Microbiology Laboratory Chan-Zuckerberg Biohub CZB Cliahub Consortium
EPI_ISL_429013 hCoV-19/USA/CA-CZB080/2020 North America / USA / California / San Francisco 2020-03-28 UCSF Clinical Microbiology Laboratory Chan-Zuckerberg Biohub CZB Cliahub Consortium
EPI_ISL_429014 hCoV-19/USA/CA-CZB0142/2020 North America / USA / California / San Francisco 2020-04-08 UCSF Clinical Microbiology Laboratory Chan-Zuckerberg Biohub CZB Cliahub Consortium
EPI_ISL_429015 hCoV-19/USA/CA-CZB0100/2020 North America / USA / California / San Francisco 2020-03-28 UCSF Clinical Microbiology Laboratory Chan-Zuckerberg Biohub CZB Cliahub Consortium
EPI_ISL_429016 hCoV-19/USA/CA-CZB0709/2020 North America / USA / California / San Francisco 2020-03-23 UCSF Clinical Microbiology Laboratory Chan-Zuckerberg Biohub CZB Cliahub Consortium
EPI_ISL_429017 hCoV-19/USA/CA-CZB030/2020 North America / USA / California / San Francisco 2020-03-25 UCSF Clinical Microbiology Laboratory Chan-Zuckerberg Biohub CZB Cliahub Consortium
EPI_ISL_429018 hCoV-19/USA/CA-CZB0750/2020 North America / USA / California / San Francisco 2020-04-03 UCSF Clinical Microbiology Laboratory Chan-Zuckerberg Biohub CZB Cliahub Consortium
EPI_ISL_429019 hCoV-19/USA/CA-CZB078/2020 North America / USA / California / San Francisco 2020-03-28 UCSF Clinical Microbiology Laboratory Chan-Zuckerberg Biohub CZB Cliahub Consortium
EPI_ISL_429020 hCoV-19/USA/CA-CZB0721/2020 North America / USA / California / San Francisco 2020-03-31 UCSF Clinical Microbiology Laboratory Chan-Zuckerberg Biohub CZB Cliahub Consortium
EPI_ISL_429021 hCoV-19/USA/CA-CZB045/2020 North America / USA / California / San Francisco 2020-03-25 UCSF Clinical Microbiology Laboratory Chan-Zuckerberg Biohub CZB Cliahub Consortium
EPI_ISL_429022 hCoV-19/USA/CA-CZB0713/2020 North America / USA / California / San Francisco 2020-03-23 UCSF Clinical Microbiology Laboratory Chan-Zuckerberg Biohub CZB Cliahub Consortium
EPI_ISL_429023 hCoV-19/USA/CA-CZB0740/2020 North America / USA / California / San Francisco 2020-03-31 UCSF Clinical Microbiology Laboratory Chan-Zuckerberg Biohub CZB Cliahub Consortium
EPI_ISL_429024 hCoV-19/USA/CA-CZB0705/2020 North America / USA / California / San Francisco 2020-03-23 UCSF Clinical Microbiology Laboratory Chan-Zuckerberg Biohub CZB Cliahub Consortium
EPI_ISL_429025 hCoV-19/USA/CA-CZB0737/2020 North America / USA / California / San Francisco 2020-03-31 UCSF Clinical Microbiology Laboratory Chan-Zuckerberg Biohub CZB Cliahub Consortium
EPI_ISL_429026 hCoV-19/USA/CA-CZB0731/2020 North America / USA / California / San Francisco 2020-03-31 UCSF Clinical Microbiology Laboratory Chan-Zuckerberg Biohub CZB Cliahub Consortium
EPI_ISL_429027 hCoV-19/USA/CA-CZB0137/2020 North America / USA / California / San Francisco 2020-04-08 UCSF Clinical Microbiology Laboratory Chan-Zuckerberg Biohub CZB Cliahub Consortium
EPI_ISL_429028 hCoV-19/USA/CA-CZB0746/2020 North America / USA / California / San Francisco 2020-04-03 UCSF Clinical Microbiology Laboratory Chan-Zuckerberg Biohub CZB Cliahub Consortium

EPI_ISL_429029 hCoV-19/USA/CA-CZB067/2020 North America / USA / California / San Francisco 2020-03-28 UCSF Clinical Microbiology Laboratory Chan-Zuckerberg Biohub CZB Cliahub Consortium
EPI_ISL_429030 hCoV-19/USA/CA-CZB072/2020 North America / USA / California / San Francisco 2020-03-28 UCSF Clinical Microbiology Laboratory Chan-Zuckerberg Biohub CZB Cliahub Consortium
EPI_ISL_429031 hCoV-19/USA/CA-CZB0139/2020 North America / USA / California / San Francisco 2020-04-08 UCSF Clinical Microbiology Laboratory Chan-Zuckerberg Biohub CZB Cliahub Consortium
EPI_ISL_429032 hCoV-19/USA/CA-CZB082/2020 North America / USA / California / San Francisco 2020-03-28 UCSF Clinical Microbiology Laboratory Chan-Zuckerberg Biohub CZB Cliahub Consortium
EPI_ISL_429033 hCoV-19/USA/CA-CZB086/2020 North America / USA / California / San Francisco 2020-03-28 UCSF Clinical Microbiology Laboratory Chan-Zuckerberg Biohub CZB Cliahub Consortium
EPI_ISL_429034 hCoV-19/USA/CA-CZB0706/2020 North America / USA / California / San Francisco 2020-03-23 UCSF Clinical Microbiology Laboratory Chan-Zuckerberg Biohub CZB Cliahub Consortium
EPI_ISL_429035 hCoV-19/USA/CA-CZB042/2020 North America / USA / California / San Francisco 2020-03-25 UCSF Clinical Microbiology Laboratory Chan-Zuckerberg Biohub CZB Cliahub Consortium
EPI_ISL_429036 hCoV-19/USA/CA-CZB0107/2020 North America / USA / California / San Francisco 2020-03-28 UCSF Clinical Microbiology Laboratory Chan-Zuckerberg Biohub CZB Cliahub Consortium
EPI_ISL_429037 hCoV-19/USA/CA-CZB09/2020 North America / USA / California / San Francisco 2020-03-25 UCSF Clinical Microbiology Laboratory Chan-Zuckerberg Biohub CZB Cliahub Consortium
EPI_ISL_429038 hCoV-19/USA/CA-CZB0707/2020 North America / USA / California / San Francisco 2020-03-23 UCSF Clinical Microbiology Laboratory Chan-Zuckerberg Biohub CZB Cliahub Consortium
EPI_ISL_429039 hCoV-19/USA/CA-CZB036/2020 North America / USA / California / San Francisco 2020-03-25 UCSF Clinical Microbiology Laboratory Chan-Zuckerberg Biohub CZB Cliahub Consortium
EPI_ISL_429040 hCoV-19/USA/CA-CZB0701/2020 North America / USA / California / San Francisco 2020-03-23 UCSF Clinical Microbiology Laboratory Chan-Zuckerberg Biohub CZB Cliahub Consortium
EPI_ISL_429041 hCoV-19/USA/CA-CZB096/2020 North America / USA / California / San Francisco 2020-03-28 UCSF Clinical Microbiology Laboratory Chan-Zuckerberg Biohub CZB Cliahub Consortium
EPI_ISL_429042 hCoV-19/USA/CA-CZB0710/2020 North America / USA / California / San Francisco 2020-03-23 UCSF Clinical Microbiology Laboratory Chan-Zuckerberg Biohub CZB Cliahub Consortium
EPI_ISL_429043 hCoV-19/USA/CA-CZB0156/2020 North America / USA / California / San Francisco 2020-04-08 UCSF Clinical Microbiology Laboratory Chan-Zuckerberg Biohub CZB Cliahub Consortium
EPI_ISL_429044 hCoV-19/USA/CA-CZB0158/2020 North America / USA / California / San Francisco 2020-04-08 UCSF Clinical Microbiology Laboratory Chan-Zuckerberg Biohub CZB Cliahub Consortium
EPI_ISL_429045 hCoV-19/USA/CA-CZB039/2020 North America / USA / California / San Francisco 2020-03-25 UCSF Clinical Microbiology Laboratory Chan-Zuckerberg Biohub CZB Cliahub Consortium
EPI_ISL_429046 hCoV-19/USA/CA-CZB0125/2020 North America / USA / California / San Francisco 2020-04-08 UCSF Clinical Microbiology Laboratory Chan-Zuckerberg Biohub CZB Cliahub Consortium
EPI_ISL_429047 hCoV-19/USA/CA-CZB0154/2020 North America / USA / California / San Francisco 2020-04-08 UCSF Clinical Microbiology Laboratory Chan-Zuckerberg Biohub CZB Cliahub Consortium
EPI_ISL_429048 hCoV-19/USA/CA-CZB043/2020 North America / USA / California / San Francisco 2020-03-25 UCSF Clinical Microbiology Laboratory Chan-Zuckerberg Biohub CZB Cliahub Consortium
EPI_ISL_429049 hCoV-19/USA/CA-CZB090/2020 North America / USA / California / San Francisco 2020-03-28 UCSF Clinical Microbiology Laboratory Chan-Zuckerberg Biohub CZB Cliahub Consortium
EPI_ISL_429050 hCoV-19/USA/CA-CZB089/2020 North America / USA / California / San Francisco 2020-03-28 UCSF Clinical Microbiology Laboratory Chan-Zuckerberg Biohub CZB Cliahub Consortium
EPI_ISL_429051 hCoV-19/USA/CA-CZB0741/2020 North America / USA / California / San Francisco 2020-04-03 UCSF Clinical Microbiology Laboratory Chan-Zuckerberg Biohub CZB Cliahub Consortium
EPI_ISL_429052 hCoV-19/USA/CA-CZB0714/2020 North America / USA / California / San Francisco 2020-03-23 UCSF Clinical Microbiology Laboratory Chan-Zuckerberg Biohub CZB Cliahub Consortium
EPI_ISL_429053 hCoV-19/USA/CA-CZB0167/2020 North America / USA / California / San Francisco 2020-04-08 UCSF Clinical Microbiology Laboratory Chan-Zuckerberg Biohub CZB Cliahub Consortium
EPI_ISL_429054 hCoV-19/USA/CA-CZB0141/2020 North America / USA / California / San Francisco 2020-04-08 UCSF Clinical Microbiology Laboratory Chan-Zuckerberg Biohub CZB Cliahub Consortium
EPI_ISL_429055 hCoV-19/USA/CA-CZB0726/2020 North America / USA / California / San Francisco 2020-03-31 UCSF Clinical Microbiology Laboratory Chan-Zuckerberg Biohub CZB Cliahub Consortium
EPI_ISL_429056 hCoV-19/USA/CA-CZB0101/2020 North America / USA / California / San Francisco 2020-03-28 UCSF Clinical Microbiology Laboratory Chan-Zuckerberg Biohub CZB Cliahub Consortium
EPI_ISL_429057 hCoV-19/USA/CA-CZB076/2020 North America / USA / California / San Francisco 2020-03-28 UCSF Clinical Microbiology Laboratory Chan-Zuckerberg Biohub CZB Cliahub Consortium
EPI_ISL_429058 hCoV-19/USA/CA-CZB0114/2020 North America / USA / California / San Francisco 2020-03-28 UCSF Clinical Microbiology Laboratory Chan-Zuckerberg Biohub CZB Cliahub Consortium
EPI_ISL_429059 hCoV-19/USA/CA-CZB0110/2020 North America / USA / California / San Francisco 2020-03-28 UCSF Clinical Microbiology Laboratory Chan-Zuckerberg Biohub CZB Cliahub Consortium
EPI_ISL_429508 hCoV-19/Denmark/ALAB-SSI412A/2020 Europe / Denmark / Unknown 2020-03-23 Department of Virus and Microbiological Special Diagnostics, Statens Serum Institut, Copenhagen, Denmark, Artillerivej 5, 2300 Copenahgen S Albertsen lab, Department of Chemistry and Bioscience, Aalborg University, Denmark Rasmus Kirkegaard
EPI_ISL_429509 hCoV-19/Denmark/ALAB-SSI413/2020 Europe / Denmark / Unknown 2020-03-10 Department of Virus and Microbiological Special Diagnostics, Statens Serum Institut, Copenhagen, Denmark, Artillerivej 5, 2300 Copenahgen S Albertsen lab, Department of Chemistry and Bioscience, Aalborg University, Denmark Rasmus Kirkegaard

EPI_ISL_429510 hCoV-19/Denmark/ALAB-SSI413A/2020 Europe / Denmark / Unknown 2020-03-23
Department of Virus and Microbiological Special Diagnostics, Statens Serum Institut, Copenhagen, Denmark, Artillerivej 5, 2300 Copenahgen S Albertsen lab, Department of Chemistry and Bioscience, Aalborg University, Denmark Rasmus Kirkegaard
EPI_ISL_429511 hCoV-19/Denmark/ALAB-SSI414/2020 Europe / Denmark / Unknown 2020-03-10
Department of Virus and Microbiological Special Diagnostics, Statens Serum Institut, Copenhagen, Denmark, Artillerivej 5, 2300 Copenahgen S Albertsen lab, Department of Chemistry and Bioscience, Aalborg University, Denmark Rasmus Kirkegaard
EPI_ISL_429512 hCoV-19/Denmark/ALAB-SSI414A/2020 Europe / Denmark / Unknown 2020-03-23
Department of Virus and Microbiological Special Diagnostics, Statens Serum Institut, Copenhagen, Denmark, Artillerivej 5, 2300 Copenahgen S Albertsen lab, Department of Chemistry and Bioscience, Aalborg University, Denmark Rasmus Kirkegaard
EPI_ISL_429513 hCoV-19/Denmark/ALAB-SSI416/2020 Europe / Denmark / Unknown 2020-03-10
Department of Virus and Microbiological Special Diagnostics, Statens Serum Institut, Copenhagen, Denmark, Artillerivej 5, 2300 Copenahgen S Albertsen lab, Department of Chemistry and Bioscience, Aalborg University, Denmark Rasmus Kirkegaard
EPI_ISL_429514 hCoV-19/Denmark/ALAB-SSI417/2020 Europe / Denmark / Unknown 2020-03-10
Department of Virus and Microbiological Special Diagnostics, Statens Serum Institut, Copenhagen, Denmark, Artillerivej 5, 2300 Copenahgen S Albertsen lab, Department of Chemistry and Bioscience, Aalborg University, Denmark Rasmus Kirkegaard
EPI_ISL_429516 hCoV-19/Denmark/ALAB-SSI418/2020 Europe / Denmark / Unknown 2020-03-10
Department of Virus and Microbiological Special Diagnostics, Statens Serum Institut, Copenhagen, Denmark, Artillerivej 5, 2300 Copenahgen S Albertsen lab, Department of Chemistry and Bioscience, Aalborg University, Denmark Rasmus Kirkegaard
EPI_ISL_429517 hCoV-19/Denmark/ALAB-SSI419/2020 Europe / Denmark / Unknown 2020-03-11
Department of Virus and Microbiological Special Diagnostics, Statens Serum Institut, Copenhagen, Denmark, Artillerivej 5, 2300 Copenahgen S Albertsen lab, Department of Chemistry and Bioscience, Aalborg University, Denmark Rasmus Kirkegaard
EPI_ISL_429518 hCoV-19/Denmark/ALAB-SSI420/2020 Europe / Denmark / Unknown 2020-03-11
Department of Virus and Microbiological Special Diagnostics, Statens Serum Institut, Copenhagen, Denmark, Artillerivej 5, 2300 Copenahgen S Albertsen lab, Department of Chemistry and Bioscience, Aalborg University, Denmark Rasmus Kirkegaard
EPI_ISL_429519 hCoV-19/Denmark/ALAB-SSI420A/2020 Europe / Denmark / Unknown 2020-03-22
Department of Virus and Microbiological Special Diagnostics, Statens Serum Institut, Copenhagen, Denmark, Artillerivej 5, 2300 Copenahgen S Albertsen lab, Department of Chemistry and Bioscience, Aalborg University, Denmark Rasmus Kirkegaard
EPI_ISL_429520 hCoV-19/Denmark/ALAB-SSI423/2020 Europe / Denmark / Unknown 2020-03-11
Department of Virus and Microbiological Special Diagnostics, Statens Serum Institut, Copenhagen, Denmark, Artillerivej 5, 2300 Copenahgen S Albertsen lab, Department of Chemistry and Bioscience, Aalborg University, Denmark Rasmus Kirkegaard
EPI_ISL_429521 hCoV-19/Denmark/ALAB-SSI424/2020 Europe / Denmark / Unknown 2020-03-11
Department of Virus and Microbiological Special Diagnostics, Statens Serum Institut, Copenhagen, Denmark, Artillerivej 5, 2300 Copenahgen S Albertsen lab, Department of Chemistry and Bioscience, Aalborg University, Denmark Rasmus Kirkegaard
EPI_ISL_429524 hCoV-19/Denmark/ALAB-SSI425A/2020 Europe / Denmark / Unknown 2020-03-24
Department of Virus and Microbiological Special Diagnostics, Statens Serum Institut, Copenhagen, Denmark, Artillerivej 5, 2300 Copenahgen S Albertsen lab, Department of Chemistry and Bioscience, Aalborg University, Denmark Rasmus Kirkegaard
EPI_ISL_429525 hCoV-19/Denmark/ALAB-SSI426/2020 Europe / Denmark / Unknown 2020-03-12
Department of Virus and Microbiological Special Diagnostics, Statens Serum Institut, Copenhagen, Denmark, Artillerivej 5, 2300 Copenahgen S Albertsen lab, Department of Chemistry and Bioscience, Aalborg University, Denmark Rasmus Kirkegaard
EPI_ISL_429526 hCoV-19/Denmark/ALAB-SSI430/2020 Europe / Denmark / Unknown 2020-03-24
Department of Virus and Microbiological Special Diagnostics, Statens Serum Institut, Copenhagen, Denmark, Artillerivej 5, 2300 Copenahgen S Albertsen lab, Department of Chemistry and Bioscience, Aalborg University, Denmark Rasmus Kirkegaard
EPI_ISL_429527 hCoV-19/Denmark/ALAB-SSI431/2020 Europe / Denmark / Unknown 2020-03-24
Department of Virus and Microbiological Special Diagnostics, Statens Serum Institut, Copenhagen, Denmark, Artillerivej 5, 2300 Copenahgen S Albertsen lab, Department of Chemistry and Bioscience, Aalborg University, Denmark Rasmus Kirkegaard
EPI_ISL_429528 hCoV-19/Denmark/ALAB-SSI432/2020 Europe / Denmark / Unknown 2020-03-24
Department of Virus and Microbiological Special Diagnostics, Statens Serum Institut, Copenhagen, Denmark, Artillerivej 5, 2300 Copenahgen S Albertsen lab, Department of Chemistry and Bioscience, Aalborg University, Denmark Rasmus Kirkegaard
EPI_ISL_429529 hCoV-19/Denmark/ALAB-SSI434/2020 Europe / Denmark / Unknown 2020-03-24
Department of Virus and Microbiological Special Diagnostics, Statens Serum Institut, Copenhagen, Denmark, Artillerivej 5, 2300 Copenahgen S Albertsen lab, Department of Chemistry and Bioscience, Aalborg University, Denmark Rasmus Kirkegaard
EPI_ISL_429530 hCoV-19/Denmark/ALAB-SSI437/2020 Europe / Denmark / Unknown 2020-03-25
Department of Virus and Microbiological Special Diagnostics, Statens Serum Institut, Copenhagen, Denmark,

Artillerivej 5, 2300 Copenahgen S	Albertsen lab, Department of Chemistry and Bioscience, Aalborg University, Denmark	Rasmus Kirkegaard
EPI_ISL_429533	hCoV-19/Denmark/ALAB-SSI444/2020	Europe / Denmark / Unknown	2020-03-25	Department of Virus and Microbiological Special Diagnostics, Statens Serum Institut, Copenhagen, Denmark, Artillerivej 5, 2300 Copenahgen S	Albertsen lab, Department of Chemistry and Bioscience, Aalborg University, Denmark	Rasmus Kirkegaard
EPI_ISL_429534	hCoV-19/Denmark/ALAB-SSI446/2020	Europe / Denmark / Unknown	2020-03-25	Department of Virus and Microbiological Special Diagnostics, Statens Serum Institut, Copenhagen, Denmark, Artillerivej 5, 2300 Copenahgen S	Albertsen lab, Department of Chemistry and Bioscience, Aalborg University, Denmark	Rasmus Kirkegaard
EPI_ISL_429535	hCoV-19/Denmark/ALAB-SSI448/2020	Europe / Denmark / Unknown	2020-03-25	Department of Virus and Microbiological Special Diagnostics, Statens Serum Institut, Copenhagen, Denmark, Artillerivej 5, 2300 Copenahgen S	Albertsen lab, Department of Chemistry and Bioscience, Aalborg University, Denmark	Rasmus Kirkegaard
EPI_ISL_429536	hCoV-19/Denmark/ALAB-SSI449/2020	Europe / Denmark / Unknown	2020-03-25	Department of Virus and Microbiological Special Diagnostics, Statens Serum Institut, Copenhagen, Denmark, Artillerivej 5, 2300 Copenahgen S	Albertsen lab, Department of Chemistry and Bioscience, Aalborg University, Denmark	Rasmus Kirkegaard
EPI_ISL_429537	hCoV-19/Denmark/ALAB-SSI450/2020	Europe / Denmark / Unknown	2020-03-24	Department of Virus and Microbiological Special Diagnostics, Statens Serum Institut, Copenhagen, Denmark, Artillerivej 5, 2300 Copenahgen S	Albertsen lab, Department of Chemistry and Bioscience, Aalborg University, Denmark	Rasmus Kirkegaard
EPI_ISL_429538	hCoV-19/Denmark/ALAB-SSI451/2020	Europe / Denmark / Unknown	2020-03-25	Department of Virus and Microbiological Special Diagnostics, Statens Serum Institut, Copenhagen, Denmark, Artillerivej 5, 2300 Copenahgen S	Albertsen lab, Department of Chemistry and Bioscience, Aalborg University, Denmark	Rasmus Kirkegaard
EPI_ISL_429539	hCoV-19/Denmark/ALAB-SSI454/2020	Europe / Denmark / Unknown	2020-03-25	Department of Virus and Microbiological Special Diagnostics, Statens Serum Institut, Copenhagen, Denmark, Artillerivej 5, 2300 Copenahgen S	Albertsen lab, Department of Chemistry and Bioscience, Aalborg University, Denmark	Rasmus Kirkegaard
EPI_ISL_429540	hCoV-19/Denmark/ALAB-SSI456/2020	Europe / Denmark / Unknown	2020-03-25	Department of Virus and Microbiological Special Diagnostics, Statens Serum Institut, Copenhagen, Denmark, Artillerivej 5, 2300 Copenahgen S	Albertsen lab, Department of Chemistry and Bioscience, Aalborg University, Denmark	Rasmus Kirkegaard
EPI_ISL_429542	hCoV-19/Denmark/ALAB-SSI461/2020	Europe / Denmark / Unknown	2020-03-25	Department of Virus and Microbiological Special Diagnostics, Statens Serum Institut, Copenhagen, Denmark, Artillerivej 5, 2300 Copenahgen S	Albertsen lab, Department of Chemistry and Bioscience, Aalborg University, Denmark	Rasmus Kirkegaard
EPI_ISL_429543	hCoV-19/Denmark/ALAB-SSI462/2020	Europe / Denmark / Unknown	2020-03-25	Department of Virus and Microbiological Special Diagnostics, Statens Serum Institut, Copenhagen, Denmark, Artillerivej 5, 2300 Copenahgen S	Albertsen lab, Department of Chemistry and Bioscience, Aalborg University, Denmark	Rasmus Kirkegaard
EPI_ISL_429544	hCoV-19/Denmark/ALAB-SSI463/2020	Europe / Denmark / Unknown	2020-03-25	Department of Virus and Microbiological Special Diagnostics, Statens Serum Institut, Copenhagen, Denmark, Artillerivej 5, 2300 Copenahgen S	Albertsen lab, Department of Chemistry and Bioscience, Aalborg University, Denmark	Rasmus Kirkegaard
EPI_ISL_429545	hCoV-19/Denmark/ALAB-SSI464/2020	Europe / Denmark / Unknown	2020-03-25	Department of Virus and Microbiological Special Diagnostics, Statens Serum Institut, Copenhagen, Denmark, Artillerivej 5, 2300 Copenahgen S	Albertsen lab, Department of Chemistry and Bioscience, Aalborg University, Denmark	Rasmus Kirkegaard
EPI_ISL_429546	hCoV-19/Denmark/ALAB-SSI466/2020	Europe / Denmark / Unknown	2020-03-25	Department of Virus and Microbiological Special Diagnostics, Statens Serum Institut, Copenhagen, Denmark, Artillerivej 5, 2300 Copenahgen S	Albertsen lab, Department of Chemistry and Bioscience, Aalborg University, Denmark	Rasmus Kirkegaard
EPI_ISL_429547	hCoV-19/Denmark/ALAB-SSI467/2020	Europe / Denmark / Unknown	2020-03-25	Department of Virus and Microbiological Special Diagnostics, Statens Serum Institut, Copenhagen, Denmark, Artillerivej 5, 2300 Copenahgen S	Albertsen lab, Department of Chemistry and Bioscience, Aalborg University, Denmark	Rasmus Kirkegaard
EPI_ISL_429548	hCoV-19/Denmark/ALAB-SSI468/2020	Europe / Denmark / Unknown	2020-03-25	Department of Virus and Microbiological Special Diagnostics, Statens Serum Institut, Copenhagen, Denmark, Artillerivej 5, 2300 Copenahgen S	Albertsen lab, Department of Chemistry and Bioscience, Aalborg University, Denmark	Rasmus Kirkegaard
EPI_ISL_429549	hCoV-19/Denmark/ALAB-SSI469/2020	Europe / Denmark / Unknown	2020-03-25	Department of Virus and Microbiological Special Diagnostics, Statens Serum Institut, Copenhagen, Denmark, Artillerivej 5, 2300 Copenahgen S	Albertsen lab, Department of Chemistry and Bioscience, Aalborg University, Denmark	Rasmus Kirkegaard
EPI_ISL_429550	hCoV-19/Denmark/ALAB-SSI470/2020	Europe / Denmark / Unknown	2020-03-25	Department of Virus and Microbiological Special Diagnostics, Statens Serum Institut, Copenhagen, Denmark, Artillerivej 5, 2300 Copenahgen S	Albertsen lab, Department of Chemistry and Bioscience, Aalborg University, Denmark	Rasmus Kirkegaard

EPI_ISL_429551 hCoV-19/Denmark/ALAB-SSI471/2020 Europe / Denmark / Unknown 2020-03-25
Department of Virus and Microbiological Special Diagnostics, Statens Serum Institut, Copenhagen, Denmark, Artillerivej 5, 2300 Copenahgen S Albertsen lab, Department of Chemistry and Bioscience, Aalborg University, Denmark Rasmus Kirkegaard
EPI_ISL_429552 hCoV-19/Denmark/ALAB-SSI472/2020 Europe / Denmark / Unknown 2020-03-25
Department of Virus and Microbiological Special Diagnostics, Statens Serum Institut, Copenhagen, Denmark, Artillerivej 5, 2300 Copenahgen S Albertsen lab, Department of Chemistry and Bioscience, Aalborg University, Denmark Rasmus Kirkegaard
EPI_ISL_429553 hCoV-19/Denmark/ALAB-SSI473/2020 Europe / Denmark / Unknown 2020-03-25
Department of Virus and Microbiological Special Diagnostics, Statens Serum Institut, Copenhagen, Denmark, Artillerivej 5, 2300 Copenahgen S Albertsen lab, Department of Chemistry and Bioscience, Aalborg University, Denmark Rasmus Kirkegaard
EPI_ISL_429554 hCoV-19/Denmark/ALAB-SSI474/2020 Europe / Denmark / Unknown 2020-03-25
Department of Virus and Microbiological Special Diagnostics, Statens Serum Institut, Copenhagen, Denmark, Artillerivej 5, 2300 Copenahgen S Albertsen lab, Department of Chemistry and Bioscience, Aalborg University, Denmark Rasmus Kirkegaard
EPI_ISL_429555 hCoV-19/Denmark/ALAB-SSI475/2020 Europe / Denmark / Unknown 2020-03-25
Department of Virus and Microbiological Special Diagnostics, Statens Serum Institut, Copenhagen, Denmark, Artillerivej 5, 2300 Copenahgen S Albertsen lab, Department of Chemistry and Bioscience, Aalborg University, Denmark Rasmus Kirkegaard
EPI_ISL_429556 hCoV-19/Denmark/ALAB-SSI477/2020 Europe / Denmark / Unknown 2020-03-26
Department of Virus and Microbiological Special Diagnostics, Statens Serum Institut, Copenhagen, Denmark, Artillerivej 5, 2300 Copenahgen S Albertsen lab, Department of Chemistry and Bioscience, Aalborg University, Denmark Rasmus Kirkegaard
EPI_ISL_429557 hCoV-19/Denmark/ALAB-SSI478/2020 Europe / Denmark / Unknown 2020-03-26
Department of Virus and Microbiological Special Diagnostics, Statens Serum Institut, Copenhagen, Denmark, Artillerivej 5, 2300 Copenahgen S Albertsen lab, Department of Chemistry and Bioscience, Aalborg University, Denmark Rasmus Kirkegaard
EPI_ISL_429558 hCoV-19/Denmark/ALAB-SSI479/2020 Europe / Denmark / Unknown 2020-03-25
Department of Virus and Microbiological Special Diagnostics, Statens Serum Institut, Copenhagen, Denmark, Artillerivej 5, 2300 Copenahgen S Albertsen lab, Department of Chemistry and Bioscience, Aalborg University, Denmark Rasmus Kirkegaard
EPI_ISL_429559 hCoV-19/Denmark/ALAB-SSI480/2020 Europe / Denmark / Unknown 2020-03-25
Department of Virus and Microbiological Special Diagnostics, Statens Serum Institut, Copenhagen, Denmark, Artillerivej 5, 2300 Copenahgen S Albertsen lab, Department of Chemistry and Bioscience, Aalborg University, Denmark Rasmus Kirkegaard
EPI_ISL_429560 hCoV-19/Denmark/ALAB-SSI481/2020 Europe / Denmark / Unknown 2020-03-25
Department of Virus and Microbiological Special Diagnostics, Statens Serum Institut, Copenhagen, Denmark, Artillerivej 5, 2300 Copenahgen S Albertsen lab, Department of Chemistry and Bioscience, Aalborg University, Denmark Rasmus Kirkegaard
EPI_ISL_429561 hCoV-19/Denmark/ALAB-SSI482/2020 Europe / Denmark / Unknown 2020-03-25
Department of Virus and Microbiological Special Diagnostics, Statens Serum Institut, Copenhagen, Denmark, Artillerivej 5, 2300 Copenahgen S Albertsen lab, Department of Chemistry and Bioscience, Aalborg University, Denmark Rasmus Kirkegaard
EPI_ISL_429562 hCoV-19/Denmark/ALAB-SSI483/2020 Europe / Denmark / Unknown 2020-03-26
Department of Virus and Microbiological Special Diagnostics, Statens Serum Institut, Copenhagen, Denmark, Artillerivej 5, 2300 Copenahgen S Albertsen lab, Department of Chemistry and Bioscience, Aalborg University, Denmark Rasmus Kirkegaard
EPI_ISL_429563 hCoV-19/Denmark/ALAB-SSI484/2020 Europe / Denmark / Unknown 2020-03-23
Department of Virus and Microbiological Special Diagnostics, Statens Serum Institut, Copenhagen, Denmark, Artillerivej 5, 2300 Copenahgen S Albertsen lab, Department of Chemistry and Bioscience, Aalborg University, Denmark Rasmus Kirkegaard
EPI_ISL_429564 hCoV-19/Denmark/ALAB-SSI486/2020 Europe / Denmark / Unknown 2020-03-26
Department of Virus and Microbiological Special Diagnostics, Statens Serum Institut, Copenhagen, Denmark, Artillerivej 5, 2300 Copenahgen S Albertsen lab, Department of Chemistry and Bioscience, Aalborg University, Denmark Rasmus Kirkegaard
EPI_ISL_429565 hCoV-19/Denmark/ALAB-SSI488/2020 Europe / Denmark / Unknown 2020-03-24
Department of Virus and Microbiological Special Diagnostics, Statens Serum Institut, Copenhagen, Denmark, Artillerivej 5, 2300 Copenahgen S Albertsen lab, Department of Chemistry and Bioscience, Aalborg University, Denmark Rasmus Kirkegaard
EPI_ISL_429566 hCoV-19/Denmark/ALAB-SSI489/2020 Europe / Denmark / Unknown 2020-03-23
Department of Virus and Microbiological Special Diagnostics, Statens Serum Institut, Copenhagen, Denmark, Artillerivej 5, 2300 Copenahgen S Albertsen lab, Department of Chemistry and Bioscience, Aalborg University, Denmark Rasmus Kirkegaard
EPI_ISL_429567 hCoV-19/Denmark/ALAB-SSI490/2020 Europe / Denmark / Unknown 2020-03-21
Department of Virus and Microbiological Special Diagnostics, Statens Serum Institut, Copenhagen, Denmark, Artillerivej 5, 2300 Copenahgen S Albertsen lab, Department of Chemistry and Bioscience, Aalborg University, Denmark Rasmus Kirkegaard
EPI_ISL_429569 hCoV-19/Denmark/ALAB-SSI494/2020 Europe / Denmark / Unknown 2020-03-24
Department of Virus and Microbiological Special Diagnostics, Statens Serum Institut, Copenhagen, Denmark,

Artillerivej 5, 2300 Copenahgen S	Albertsen lab, Department of Chemistry and Bioscience, Aalborg University, Denmark	Rasmus Kirkegaard
EPI_ISL_429570	hCoV-19/Denmark/ALAB-SSI495/2020	Europe / Denmark / Unknown	2020-03-24	Department of Virus and Microbiological Special Diagnostics, Statens Serum Institut, Copenhagen, Denmark, Artillerivej 5, 2300 Copenahgen S	Albertsen lab, Department of Chemistry and Bioscience, Aalborg University, Denmark	Rasmus Kirkegaard
EPI_ISL_429571	hCoV-19/Denmark/ALAB-SSI497/2020	Europe / Denmark / Unknown	2020-03-26	Department of Virus and Microbiological Special Diagnostics, Statens Serum Institut, Copenhagen, Denmark, Artillerivej 5, 2300 Copenahgen S	Albertsen lab, Department of Chemistry and Bioscience, Aalborg University, Denmark	Rasmus Kirkegaard
EPI_ISL_429572	hCoV-19/Denmark/ALAB-SSI501/2020	Europe / Denmark / Unknown	2020-03-24	Department of Virus and Microbiological Special Diagnostics, Statens Serum Institut, Copenhagen, Denmark, Artillerivej 5, 2300 Copenahgen S	Albertsen lab, Department of Chemistry and Bioscience, Aalborg University, Denmark	Rasmus Kirkegaard
EPI_ISL_429573	hCoV-19/Denmark/ALAB-SSI502/2020	Europe / Denmark / Unknown	2020-03-23	Department of Virus and Microbiological Special Diagnostics, Statens Serum Institut, Copenhagen, Denmark, Artillerivej 5, 2300 Copenahgen S	Albertsen lab, Department of Chemistry and Bioscience, Aalborg University, Denmark	Rasmus Kirkegaard
EPI_ISL_429574	hCoV-19/Denmark/ALAB-SSI506/2020	Europe / Denmark / Unknown	2020-03-26	Department of Virus and Microbiological Special Diagnostics, Statens Serum Institut, Copenhagen, Denmark, Artillerivej 5, 2300 Copenahgen S	Albertsen lab, Department of Chemistry and Bioscience, Aalborg University, Denmark	Rasmus Kirkegaard
EPI_ISL_429575	hCoV-19/Denmark/ALAB-SSI507/2020	Europe / Denmark / Unknown	2020-03-25	Department of Virus and Microbiological Special Diagnostics, Statens Serum Institut, Copenhagen, Denmark, Artillerivej 5, 2300 Copenahgen S	Albertsen lab, Department of Chemistry and Bioscience, Aalborg University, Denmark	Rasmus Kirkegaard
EPI_ISL_429577	hCoV-19/Denmark/ALAB-SSI509/2020	Europe / Denmark / Unknown	2020-03-25	Department of Virus and Microbiological Special Diagnostics, Statens Serum Institut, Copenhagen, Denmark, Artillerivej 5, 2300 Copenahgen S	Albertsen lab, Department of Chemistry and Bioscience, Aalborg University, Denmark	Rasmus Kirkegaard
EPI_ISL_429578	hCoV-19/Denmark/ALAB-SSI510/2020	Europe / Denmark / Unknown	2020-03-25	Department of Virus and Microbiological Special Diagnostics, Statens Serum Institut, Copenhagen, Denmark, Artillerivej 5, 2300 Copenahgen S	Albertsen lab, Department of Chemistry and Bioscience, Aalborg University, Denmark	Rasmus Kirkegaard
EPI_ISL_429579	hCoV-19/Denmark/ALAB-SSI511/2020	Europe / Denmark / Unknown	2020-03-26	Department of Virus and Microbiological Special Diagnostics, Statens Serum Institut, Copenhagen, Denmark, Artillerivej 5, 2300 Copenahgen S	Albertsen lab, Department of Chemistry and Bioscience, Aalborg University, Denmark	Rasmus Kirkegaard
EPI_ISL_429580	hCoV-19/Denmark/ALAB-SSI512/2020	Europe / Denmark / Unknown	2020-03-25	Department of Virus and Microbiological Special Diagnostics, Statens Serum Institut, Copenhagen, Denmark, Artillerivej 5, 2300 Copenahgen S	Albertsen lab, Department of Chemistry and Bioscience, Aalborg University, Denmark	Rasmus Kirkegaard
EPI_ISL_429581	hCoV-19/Denmark/ALAB-SSI513/2020	Europe / Denmark / Unknown	2020-03-24	Department of Virus and Microbiological Special Diagnostics, Statens Serum Institut, Copenhagen, Denmark, Artillerivej 5, 2300 Copenahgen S	Albertsen lab, Department of Chemistry and Bioscience, Aalborg University, Denmark	Rasmus Kirkegaard
EPI_ISL_429582	hCoV-19/Denmark/ALAB-SSI514/2020	Europe / Denmark / Unknown	2020-03-16	Department of Virus and Microbiological Special Diagnostics, Statens Serum Institut, Copenhagen, Denmark, Artillerivej 5, 2300 Copenahgen S	Albertsen lab, Department of Chemistry and Bioscience, Aalborg University, Denmark	Rasmus Kirkegaard
EPI_ISL_429583	hCoV-19/Denmark/ALAB-SSI515/2020	Europe / Denmark / Unknown	2020-03-23	Department of Virus and Microbiological Special Diagnostics, Statens Serum Institut, Copenhagen, Denmark, Artillerivej 5, 2300 Copenahgen S	Albertsen lab, Department of Chemistry and Bioscience, Aalborg University, Denmark	Rasmus Kirkegaard
EPI_ISL_429584	hCoV-19/Denmark/ALAB-SSI518/2020	Europe / Denmark / Unknown	2020-03-26	Department of Virus and Microbiological Special Diagnostics, Statens Serum Institut, Copenhagen, Denmark, Artillerivej 5, 2300 Copenahgen S	Albertsen lab, Department of Chemistry and Bioscience, Aalborg University, Denmark	Rasmus Kirkegaard
EPI_ISL_429585	hCoV-19/Denmark/ALAB-SSI520/2020	Europe / Denmark / Unknown	2020-03-26	Department of Virus and Microbiological Special Diagnostics, Statens Serum Institut, Copenhagen, Denmark, Artillerivej 5, 2300 Copenahgen S	Albertsen lab, Department of Chemistry and Bioscience, Aalborg University, Denmark	Rasmus Kirkegaard
EPI_ISL_429586	hCoV-19/Denmark/ALAB-SSI521/2020	Europe / Denmark / Unknown	2020-03-26	Department of Virus and Microbiological Special Diagnostics, Statens Serum Institut, Copenhagen, Denmark, Artillerivej 5, 2300 Copenahgen S	Albertsen lab, Department of Chemistry and Bioscience, Aalborg University, Denmark	Rasmus Kirkegaard
EPI_ISL_429587	hCoV-19/Denmark/ALAB-SSI522/2020	Europe / Denmark / Unknown	2020-03-26	Department of Virus and Microbiological Special Diagnostics, Statens Serum Institut, Copenhagen, Denmark, Artillerivej 5, 2300 Copenahgen S	Albertsen lab, Department of Chemistry and Bioscience, Aalborg University, Denmark	Rasmus Kirkegaard

EPI_ISL_429588 hCoV-19/Denmark/ALAB-SSI523/2020   Europe / Denmark / Unknown   2020-03-26
Department of Virus and Microbiological Special Diagnostics, Statens Serum Institut, Copenhagen, Denmark, Artillerivej 5, 2300 Copenahgen S   Albertsen lab, Department of Chemistry and Bioscience, Aalborg University, Denmark   Rasmus Kirkegaard
EPI_ISL_429589 hCoV-19/Denmark/ALAB-SSI592/2020   Europe / Denmark / Unknown   2020-03-28
Department of Virus and Microbiological Special Diagnostics, Statens Serum Institut, Copenhagen, Denmark, Artillerivej 5, 2300 Copenahgen S   Albertsen lab, Department of Chemistry and Bioscience, Aalborg University, Denmark   Rasmus Kirkegaard
EPI_ISL_429590 hCoV-19/Denmark/ALAB-SSI595/2020   Europe / Denmark / Unknown   2020-03-28
Department of Virus and Microbiological Special Diagnostics, Statens Serum Institut, Copenhagen, Denmark, Artillerivej 5, 2300 Copenahgen S   Albertsen lab, Department of Chemistry and Bioscience, Aalborg University, Denmark   Rasmus Kirkegaard
EPI_ISL_429598 hCoV-19/USA/WA-UW-4546/2020   North America / USA / Washington   2020-03-31   UW Virology Lab   UW Virology Lab Pavitra Roychoudhury, Hong Xie, Keith Jerome, Alexander Greninger
EPI_ISL_430000 hCoV-19/Jordan/SR-042/2020   Asia / Jordan / Amman   2020-03-30   Biolab Diagnostic Laboratories   Andersen lab at Scripps Research   Issa Abu-Dayyeh, Ahmad Tibi, Lama Hussein, Lina Mohammad, Zein Naber, Amid Abdelnour with SEARCH Alliance San Diego
EPI_ISL_430001 hCoV-19/Jordan/SR-043/2020   Asia / Jordan / Amman   2020-03-17   Biolab Diagnostic Laboratories   Andersen lab at Scripps Research   Issa Abu-Dayyeh, Ahmad Tibi, Lama Hussein, Lina Mohammad, Zein Naber, Amid Abdelnour with SEARCH Alliance San Diego
EPI_ISL_430002 hCoV-19/Jordan/SR-044/2020   Asia / Jordan / Amman   2020-03-16   Biolab Diagnostic Laboratories   Andersen lab at Scripps Research   Issa Abu-Dayyeh, Ahmad Tibi, Lama Hussein, Lina Mohammad, Zein Naber, Amid Abdelnour with SEARCH Alliance San Diego
EPI_ISL_430003 hCoV-19/Jordan/SR-045/2020   Asia / Jordan / Amman   2020-03-16   Biolab Diagnostic Laboratories   Andersen lab at Scripps Research   Issa Abu-Dayyeh, Ahmad Tibi, Lama Hussein, Lina Mohammad, Zein Naber, Amid Abdelnour with SEARCH Alliance San Diego
EPI_ISL_430004 hCoV-19/Jordan/SR-046/2020   Asia / Jordan / Amman   2020-03-17   Biolab Diagnostic Laboratories   Andersen lab at Scripps Research   Issa Abu-Dayyeh, Ahmad Tibi, Lama Hussein, Lina Mohammad, Zein Naber, Amid Abdelnour with SEARCH Alliance San Diego
EPI_ISL_430006 hCoV-19/Jordan/SR-048/2020   Asia / Jordan / Amman   2020-03-23   Biolab Diagnostic Laboratories   Andersen lab at Scripps Research   Issa Abu-Dayyeh, Ahmad Tibi, Lama Hussein, Lina Mohammad, Zein Naber, Amid Abdelnour with SEARCH Alliance San Diego
EPI_ISL_430007 hCoV-19/Jordan/SR-049/2020   Asia / Jordan / Amman   2020-03-19   Biolab Diagnostic Laboratories   Andersen lab at Scripps Research   Issa Abu-Dayyeh, Ahmad Tibi, Lama Hussein, Lina Mohammad, Zein Naber, Amid Abdelnour with SEARCH Alliance San Diego
EPI_ISL_430009 hCoV-19/Jordan/SR-052/2020   Asia / Jordan / Irbid   2020-03-30   Biolab Diagnostic Laboratories   Andersen lab at Scripps Research   Issa Abu-Dayyeh, Ahmad Tibi, Lama Hussein, Lina Mohammad, Zein Naber, Amid Abdelnour with SEARCH Alliance San Diego
EPI_ISL_430011 hCoV-19/Jordan/SR-053/2020   Asia / Jordan / Amman   2020-03-19   Biolab Diagnostic Laboratories   Andersen lab at Scripps Research   Issa Abu-Dayyeh, Ahmad Tibi, Lama Hussein, Lina Mohammad, Zein Naber, Amid Abdelnour with SEARCH Alliance San Diego
EPI_ISL_430012 hCoV-19/Jordan/SR-054/2020   Asia / Jordan / Irbid   2020-03-30   Biolab Diagnostic Laboratories   Andersen lab at Scripps Research   Issa Abu-Dayyeh, Ahmad Tibi, Lama Hussein, Lina Mohammad, Zein Naber, Amid Abdelnour with SEARCH Alliance San Diego
EPI_ISL_430014 hCoV-19/Jordan/SR-056/2020   Asia / Jordan / Amman   2020-03-17   Biolab Diagnostic Laboratories   Andersen lab at Scripps Research   Issa Abu-Dayyeh, Ahmad Tibi, Lama Hussein, Lina Mohammad, Zein Naber, Amid Abdelnour with SEARCH Alliance San Diego
EPI_ISL_430015 hCoV-19/Jordan/SR-057/2020   Asia / Jordan / Amman   2020-03-24   Biolab Diagnostic Laboratories   Andersen lab at Scripps Research   Issa Abu-Dayyeh, Ahmad Tibi, Lama Hussein, Lina Mohammad, Zein Naber, Amid Abdelnour with SEARCH Alliance San Diego
EPI_ISL_430016 hCoV-19/USA/CA-SR016/2020   North America / USA / California / San Diego   2020-03-24
Andersen lab at Scripps Research   Andersen lab at Scripps Research   SEARCH Alliance San Diego
EPI_ISL_430039 hCoV-19/USA/UT-0370/2020   North America / USA / Utah   2020-03-30   Utah Public Health Laboratory   Utah Public Health Laboratory   Erin Young, Kelly Oakeson
EPI_ISL_430041 hCoV-19/USA/UT-0443/2020   North America / USA / Utah   2020-03-30   Utah Public Health Laboratory   Utah Public Health Laboratory   Erin Young, Kelly Oakeson
EPI_ISL_430043 hCoV-19/USA/UT-0445/2020   North America / USA / Utah   2020-03-30   Utah Public Health Laboratory   Utah Public Health Laboratory   Erin Young, Kelly Oakeson
EPI_ISL_430044 hCoV-19/USA/UT-0446/2020   North America / USA / Utah   2020-03-30   Utah Public Health Laboratory   Utah Public Health Laboratory   Erin Young, Kelly Oakeson
EPI_ISL_430045 hCoV-19/USA/UT-0447/2020   North America / USA / Utah   2020-03-31   Utah Public Health Laboratory   Utah Public Health Laboratory   Erin Young, Kelly Oakeson
EPI_ISL_430047 hCoV-19/USA/UT-0492/2020   North America / USA / Utah   2020-04-02   Utah Public Health Laboratory   Utah Public Health Laboratory   Erin Young, Kelly Oakeson
EPI_ISL_430050 hCoV-19/USA/UT-0500/2020   North America / USA / Utah   2020-03-31   Utah Public Health Laboratory   Utah Public Health Laboratory   Erin Young, Kelly Oakeson
EPI_ISL_430051 hCoV-19/USA/UT-0501/2020   North America / USA / Utah   2020-04-02   Utah Public Health Laboratory   Utah Public Health Laboratory   Erin Young, Kelly Oakeson
EPI_ISL_430053 hCoV-19/USA/UT-0503/2020   North America / USA / Utah   2020-04-02   Utah Public Health Laboratory   Utah Public Health Laboratory   Erin Young, Kelly Oakeson

EPI_ISL_430055 hCoV-19/USA/UT-0507/2020 North America / USA / Utah 2020-04-02 Utah Public Health Laboratory Utah Public Health Laboratory Erin Young, Kelly Oakeson
EPI_ISL_430057 hCoV-19/USA/UT-0510/2020 North America / USA / Utah 2020-04-03 Utah Public Health Laboratory Utah Public Health Laboratory Erin Young, Kelly Oakeson
EPI_ISL_430500 hCoV-19/Australia/VIC1210/2020 Oceania / Australia / Victoria 2020-04-01 Victorian Infectious Diseases Reference Laboratory (VIDRL) Microbiological Diagnostic Unit Public Health Laboratory and Victorian Infectious Diseases Reference Laboratory, The Peter Doherty Institute for Infection and Immunity Caly L., Seemann T., Sait, M., Schultz M., Druce J., Sherry, N.
EPI_ISL_430512 hCoV-19/Australia/VIC1235/2020 Oceania / Australia / Victoria 2020-04-02 Victorian Infectious Diseases Reference Laboratory (VIDRL) Microbiological Diagnostic Unit Public Health Laboratory and Victorian Infectious Diseases Reference Laboratory, The Peter Doherty Institute for Infection and Immunity Caly L., Seemann T., Sait, M., Schultz M., Druce J., Sherry, N.
EPI_ISL_430519 hCoV-19/Australia/VIC1222/2020 Oceania / Australia / Victoria 2020-04-01 Victorian Infectious Diseases Reference Laboratory (VIDRL) Microbiological Diagnostic Unit Public Health Laboratory and Victorian Infectious Diseases Reference Laboratory, The Peter Doherty Institute for Infection and Immunity Caly L., Seemann T., Sait, M., Schultz M., Druce J., Sherry, N.
EPI_ISL_430522 hCoV-19/Australia/VIC1224/2020 Oceania / Australia / Victoria 2020-04-01 Victorian Infectious Diseases Reference Laboratory (VIDRL) Microbiological Diagnostic Unit Public Health Laboratory and Victorian Infectious Diseases Reference Laboratory, The Peter Doherty Institute for Infection and Immunity Caly L., Seemann T., Sait, M., Schultz M., Druce J., Sherry, N.
EPI_ISL_430528 hCoV-19/Australia/VIC1192/2020 Oceania / Australia / Victoria 2020-03-30 Victorian Infectious Diseases Reference Laboratory (VIDRL) Microbiological Diagnostic Unit Public Health Laboratory and Victorian Infectious Diseases Reference Laboratory, The Peter Doherty Institute for Infection and Immunity Caly L., Seemann T., Sait, M., Schultz M., Druce J., Sherry, N.
EPI_ISL_430532 hCoV-19/Australia/VIC1025/2020 Oceania / Australia / Victoria 2020-04-02 Victorian Infectious Diseases Reference Laboratory (VIDRL) Microbiological Diagnostic Unit Public Health Laboratory and Victorian Infectious Diseases Reference Laboratory, The Peter Doherty Institute for Infection and Immunity Caly L., Seemann T., Sait, M., Schultz M., Druce J., Sherry, N.
EPI_ISL_430533 hCoV-19/Australia/VIC1037/2020 Oceania / Australia / Victoria 2020-04-03 Victorian Infectious Diseases Reference Laboratory (VIDRL) Microbiological Diagnostic Unit Public Health Laboratory and Victorian Infectious Diseases Reference Laboratory, The Peter Doherty Institute for Infection and Immunity Caly L., Seemann T., Sait, M., Schultz M., Druce J., Sherry, N.
EPI_ISL_430535 hCoV-19/Australia/VIC1039/2020 Oceania / Australia / Victoria 2020-04-03 Victorian Infectious Diseases Reference Laboratory (VIDRL) Microbiological Diagnostic Unit Public Health Laboratory and Victorian Infectious Diseases Reference Laboratory, The Peter Doherty Institute for Infection and Immunity Caly L., Seemann T., Sait, M., Schultz M., Druce J., Sherry, N.
EPI_ISL_430536 hCoV-19/Australia/VIC1041/2020 Oceania / Australia / Victoria 2020-04-03 Victorian Infectious Diseases Reference Laboratory (VIDRL) Microbiological Diagnostic Unit Public Health Laboratory and Victorian Infectious Diseases Reference Laboratory, The Peter Doherty Institute for Infection and Immunity Caly L., Seemann T., Sait, M., Schultz M., Druce J., Sherry, N.
EPI_ISL_430538 hCoV-19/Australia/VIC1042/2020 Oceania / Australia / Victoria 2020-04-03 Victorian Infectious Diseases Reference Laboratory (VIDRL) Microbiological Diagnostic Unit Public Health Laboratory and Victorian Infectious Diseases Reference Laboratory, The Peter Doherty Institute for Infection and Immunity Caly L., Seemann T., Sait, M., Schultz M., Druce J., Sherry, N.
EPI_ISL_430539 hCoV-19/Australia/VIC1043/2020 Oceania / Australia / Victoria 2020-04-03 Victorian Infectious Diseases Reference Laboratory (VIDRL) Microbiological Diagnostic Unit Public Health Laboratory and Victorian Infectious Diseases Reference Laboratory, The Peter Doherty Institute for Infection and Immunity Caly L., Seemann T., Sait, M., Schultz M., Druce J., Sherry, N.
EPI_ISL_430542 hCoV-19/Australia/VIC978/2020 Oceania / Australia / Victoria 2020-03-29 Victorian Infectious Diseases Reference Laboratory (VIDRL) Microbiological Diagnostic Unit Public Health Laboratory and Victorian Infectious Diseases Reference Laboratory, The Peter Doherty Institute for Infection and Immunity Caly L., Seemann T., Sait, M., Schultz M., Druce J., Sherry, N.
EPI_ISL_430543 hCoV-19/Australia/VIC982/2020 Oceania / Australia / Victoria 2020-03-30 Victorian Infectious Diseases Reference Laboratory (VIDRL) Microbiological Diagnostic Unit Public Health Laboratory and Victorian Infectious Diseases Reference Laboratory, The Peter Doherty Institute for Infection and Immunity Caly L., Seemann T., Sait, M., Schultz M., Druce J., Sherry, N.
EPI_ISL_430545 hCoV-19/Australia/VIC983/2020 Oceania / Australia / Victoria 2020-03-30 Victorian Infectious Diseases Reference Laboratory (VIDRL) Microbiological Diagnostic Unit Public Health Laboratory and Victorian Infectious Diseases Reference Laboratory, The Peter Doherty Institute for Infection and Immunity Caly L., Seemann T., Sait, M., Schultz M., Druce J., Sherry, N.
EPI_ISL_430547 hCoV-19/Australia/VIC1011/2020 Oceania / Australia / Victoria 2020-04-01 Victorian Infectious Diseases Reference Laboratory (VIDRL) Microbiological Diagnostic Unit Public Health Laboratory and Victorian Infectious Diseases Reference Laboratory, The Peter Doherty Institute for Infection and Immunity Caly L., Seemann T., Sait, M., Schultz M., Druce J., Sherry, N.
EPI_ISL_430550 hCoV-19/Australia/VIC1046/2020 Oceania / Australia / Victoria 2020-04-03 Victorian Infectious Diseases Reference Laboratory (VIDRL) Microbiological Diagnostic Unit Public Health Laboratory and Victorian Infectious Diseases Reference Laboratory, The Peter Doherty Institute for Infection and Immunity Caly L., Seemann T., Sait, M., Schultz M., Druce J., Sherry, N.
EPI_ISL_430551 hCoV-19/Australia/VIC989/2020 Oceania / Australia / Victoria 2020-03-31 Victorian Infectious Diseases Reference Laboratory (VIDRL) Microbiological Diagnostic Unit Public Health

Laboratory and Victorian Infectious Diseases Reference Laboratory, The Peter Doherty Institute for Infection and Immunity Caly L., Seemann T., Sait, M., Schultz M., Druce J., Sherry, N.
EPI_ISL_430552 hCoV-19/Australia/VIC984/2020 Oceania / Australia / Victoria 2020-03-30 Victorian Infectious Diseases Reference Laboratory (VIDRL) Microbiological Diagnostic Unit Public Health Laboratory and Victorian Infectious Diseases Reference Laboratory, The Peter Doherty Institute for Infection and Immunity Caly L., Seemann T., Sait, M., Schultz M., Druce J., Sherry, N.
EPI_ISL_430553 hCoV-19/Australia/VIC1013/2020 Oceania / Australia / Victoria 2020-04-01 Victorian Infectious Diseases Reference Laboratory (VIDRL) Microbiological Diagnostic Unit Public Health Laboratory and Victorian Infectious Diseases Reference Laboratory, The Peter Doherty Institute for Infection and Immunity Caly L., Seemann T., Sait, M., Schultz M., Druce J., Sherry, N.
EPI_ISL_430556 hCoV-19/Australia/VIC990/2020 Oceania / Australia / Victoria 2020-03-31 Victorian Infectious Diseases Reference Laboratory (VIDRL) Microbiological Diagnostic Unit Public Health Laboratory and Victorian Infectious Diseases Reference Laboratory, The Peter Doherty Institute for Infection and Immunity Caly L., Seemann T., Sait, M., Schultz M., Druce J., Sherry, N.
EPI_ISL_430559 hCoV-19/Australia/VIC1015/2020 Oceania / Australia / Victoria 2020-04-01 Victorian Infectious Diseases Reference Laboratory (VIDRL) Microbiological Diagnostic Unit Public Health Laboratory and Victorian Infectious Diseases Reference Laboratory, The Peter Doherty Institute for Infection and Immunity Caly L., Seemann T., Sait, M., Schultz M., Druce J., Sherry, N.
EPI_ISL_430567 hCoV-19/Australia/VIC1119/2020 Oceania / Australia / Victoria 2020-04-08 Victorian Infectious Diseases Reference Laboratory (VIDRL) Microbiological Diagnostic Unit Public Health Laboratory and Victorian Infectious Diseases Reference Laboratory, The Peter Doherty Institute for Infection and Immunity Caly L., Seemann T., Sait, M., Schultz M., Druce J., Sherry, N.
EPI_ISL_430569 hCoV-19/Australia/VIC1110/2020 Oceania / Australia / Victoria 2020-04-07 Victorian Infectious Diseases Reference Laboratory (VIDRL) Microbiological Diagnostic Unit Public Health Laboratory and Victorian Infectious Diseases Reference Laboratory, The Peter Doherty Institute for Infection and Immunity Caly L., Seemann T., Sait, M., Schultz M., Druce J., Sherry, N.
EPI_ISL_430573 hCoV-19/Australia/VIC1112/2020 Oceania / Australia / Victoria 2020-04-07 Victorian Infectious Diseases Reference Laboratory (VIDRL) Microbiological Diagnostic Unit Public Health Laboratory and Victorian Infectious Diseases Reference Laboratory, The Peter Doherty Institute for Infection and Immunity Caly L., Seemann T., Sait, M., Schultz M., Druce J., Sherry, N.
EPI_ISL_430574 hCoV-19/Australia/VIC1087/2020 Oceania / Australia / Victoria 2020-04-06 Victorian Infectious Diseases Reference Laboratory (VIDRL) Microbiological Diagnostic Unit Public Health Laboratory and Victorian Infectious Diseases Reference Laboratory, The Peter Doherty Institute for Infection and Immunity Caly L., Seemann T., Sait, M., Schultz M., Druce J., Sherry, N.
EPI_ISL_430576 hCoV-19/Australia/VIC1114/2020 Oceania / Australia / Victoria 2020-04-07 Victorian Infectious Diseases Reference Laboratory (VIDRL) Microbiological Diagnostic Unit Public Health Laboratory and Victorian Infectious Diseases Reference Laboratory, The Peter Doherty Institute for Infection and Immunity Caly L., Seemann T., Sait, M., Schultz M., Druce J., Sherry, N.
EPI_ISL_430577 hCoV-19/Australia/VIC1115/2020 Oceania / Australia / Victoria 2020-04-07 Victorian Infectious Diseases Reference Laboratory (VIDRL) Microbiological Diagnostic Unit Public Health Laboratory and Victorian Infectious Diseases Reference Laboratory, The Peter Doherty Institute for Infection and Immunity Caly L., Seemann T., Sait, M., Schultz M., Druce J., Sherry, N.
EPI_ISL_430578 hCoV-19/Australia/VIC1062/2020 Oceania / Australia / Victoria 2020-04-04 Victorian Infectious Diseases Reference Laboratory (VIDRL) Microbiological Diagnostic Unit Public Health Laboratory and Victorian Infectious Diseases Reference Laboratory, The Peter Doherty Institute for Infection and Immunity Caly L., Seemann T., Sait, M., Schultz M., Druce J., Sherry, N.
EPI_ISL_430579 hCoV-19/Australia/VIC1080/2020 Oceania / Australia / Victoria 2020-04-06 Victorian Infectious Diseases Reference Laboratory (VIDRL) Microbiological Diagnostic Unit Public Health Laboratory and Victorian Infectious Diseases Reference Laboratory, The Peter Doherty Institute for Infection and Immunity Caly L., Seemann T., Sait, M., Schultz M., Druce J., Sherry, N.
EPI_ISL_430580 hCoV-19/Australia/VIC1081/2020 Oceania / Australia / Victoria 2020-04-06 Victorian Infectious Diseases Reference Laboratory (VIDRL) Microbiological Diagnostic Unit Public Health Laboratory and Victorian Infectious Diseases Reference Laboratory, The Peter Doherty Institute for Infection and Immunity Caly L., Seemann T., Sait, M., Schultz M., Druce J., Sherry, N.
EPI_ISL_430581 hCoV-19/Australia/VIC1071/2020 Oceania / Australia / Victoria 2020-04-05 Victorian Infectious Diseases Reference Laboratory (VIDRL) Microbiological Diagnostic Unit Public Health Laboratory and Victorian Infectious Diseases Reference Laboratory, The Peter Doherty Institute for Infection and Immunity Caly L., Seemann T., Sait, M., Schultz M., Druce J., Sherry, N.
EPI_ISL_430583 hCoV-19/Australia/VIC1094/2020 Oceania / Australia / Victoria 2020-04-07 Victorian Infectious Diseases Reference Laboratory (VIDRL) Microbiological Diagnostic Unit Public Health Laboratory and Victorian Infectious Diseases Reference Laboratory, The Peter Doherty Institute for Infection and Immunity Caly L., Seemann T., Sait, M., Schultz M., Druce J., Sherry, N.
EPI_ISL_430584 hCoV-19/Australia/VIC1103/2020 Oceania / Australia / Victoria 2020-04-07 Victorian Infectious Diseases Reference Laboratory (VIDRL) Microbiological Diagnostic Unit Public Health Laboratory and Victorian Infectious Diseases Reference Laboratory, The Peter Doherty Institute for Infection and Immunity Caly L., Seemann T., Sait, M., Schultz M., Druce J., Sherry, N.
EPI_ISL_430590 hCoV-19/Australia/VIC1107/2020 Oceania / Australia / Victoria 2020-04-07 Victorian Infectious Diseases Reference Laboratory (VIDRL) Microbiological Diagnostic Unit Public Health Laboratory and Victorian Infectious Diseases Reference Laboratory, The Peter Doherty Institute for Infection and Immunity Caly L., Seemann T., Sait, M., Schultz M., Druce J., Sherry, N.

EPI_ISL_430596 hCoV-19/Australia/VIC1133/2020 Oceania / Australia / Victoria 2020-04-08 Victorian Infectious Diseases Reference Laboratory (VIDRL) Microbiological Diagnostic Unit Public Health Laboratory and Victorian Infectious Diseases Reference Laboratory, The Peter Doherty Institute for Infection and Immunity Caly L., Seemann T., Sait, M., Schultz M., Druce J., Sherry, N.
EPI_ISL_430597 hCoV-19/Australia/VIC1086/2020 Oceania / Australia / Victoria 2020-04-06 Victorian Infectious Diseases Reference Laboratory (VIDRL) Microbiological Diagnostic Unit Public Health Laboratory and Victorian Infectious Diseases Reference Laboratory, The Peter Doherty Institute for Infection and Immunity Caly L., Seemann T., Sait, M., Schultz M., Druce J., Sherry, N.
EPI_ISL_430598 hCoV-19/Australia/VIC1157/2020 Oceania / Australia / Victoria 2020-04-09 Victorian Infectious Diseases Reference Laboratory (VIDRL) Microbiological Diagnostic Unit Public Health Laboratory and Victorian Infectious Diseases Reference Laboratory, The Peter Doherty Institute for Infection and Immunity Caly L., Seemann T., Sait, M., Schultz M., Druce J., Sherry, N.
EPI_ISL_450404 hCoV-19/Hong Kong/VM20009579/2020 Asia / Hong Kong 2020-03-16 unknown School of Public Health Sit,T.H.S., Brackman,C.J., Sims,L.D., Tsang,D.N.C., Chu,D.K.W., Perera,R.A.P.M., Poon,L.L.M. and Peiris,M.
EPI_ISL_450444 hCoV-19/Beijing/IME-HZ01/2020 Asia / China / Beijing 2020-01-22 unknown Dept. OPA Zhang,X.A., Fan,H., Qi,R.Z., Zheng,W., Zheng,K., Gong,J.H., Fang,L.Q. and Liu,W.
EPI_ISL_450489 hCoV-19/Wuhan/YB012504/2020 Asia / China / Wuhan / Hubei 2020-01-25 unknown CAS Key Laboratory of Special Pathogens and Biosafety and Center for Emerging Infectious Diseases Si,H., Zhu,Y., Lin,H., Xie,S., Shi,Z. and Zhou,P.
EPI_ISL_406862 hCoV-19/Germany/BavPat1/2020 Europe / Germany / Bavaria / Munich 2020-01-28 Charité Universitätsmedizin Berlin, Institute of Virology; Institut für Mikrobiologie der Bundeswehr, Munich Charité Universitätsmedizin Berlin, Institute of Virology Victor M Corman, Julia Schneider, Talitha Veith, Barbara Mühlemann, Markus Antwerpen, Christian Drosten, Roman Wölfel
EPI_ISL_406970 hCoV-19/Hangzhou/HZ-1/2020 Asia / China / Zhejiang / Hangzhou 2020-01-20 Hangzhou Center for Disease and Control Microbiology Lab Hangzhou Center for Disease and Control Microbiology Lab Yu Hua, Wang Haoqiu, Li Jun, Yu Xinfeng
EPI_ISL_406973 hCoV-19/Singapore/1/2020 Asia / Singapore 2020-01-23 Singapore General Hospital National Public Health Laboratory Mak, TM; Octavia S; Chavatte JM; Zhou, ZY; Cui, L; Lin, RTP
EPI_ISL_409067 hCoV-19/USA/MA1/2020 North America / USA / Massachusetts 2020-01-29 Massachusetts Department of Public Health Pathogen Discovery, Respiratory Viruses Branch, Division of Viral Diseases, Centers for Dieases Control and Prevention Clinton R. Paden, Jing Zhang, Krista Queen, Yan Li, Ying Tao, Anna Uehara, Xiaoyan Lu, Brian Lynch, Senthil Kumar K. Sakthivel, Brett L. Whitaker, Shifaq Kamili, Lijuan Wang, Janna' R. Murray, Susan I. Gerber, Stephen Lindstrom, Suxiang Tong
EPI_ISL_410044 hCoV-19/USA/CA6/2020 North America / USA / California 2020-01-27 California Department of Public Health Pathogen Discovery, Respiratory Viruses Branch, Division of Viral Diseases, Centers for Dieases Control and Prevention Jing Zhang, Krista Queen, Yan Li, Ying Tao, Anna Uehara, Clinton R. Paden, Xiaoyan Lu, Brian Lynch, Senthil Kumar K. Sakthivel, Brett L. Whitaker, Shifaq Kamili, Lijuan Wang, Janna' R. Murray, Susan I. Gerber, Stephen Lindstrom, Suxiang Tong
EPI_ISL_410045 hCoV-19/USA/IL2/2020 North America / USA / Illinois 2020-01-28 IL Department of Public Health Chicago Laboratory Pathogen Discovery, Respiratory Viruses Branch, Division of Viral Diseases, Centers for Dieases Control and Prevention Yan Li, Jing Zhang, Krista Queen, Ying Tao, Anna Uehara, Clinton R. Paden, Xiaoyan Lu, Brian Lynch, Senthil Kumar K. Sakthivel, Brett L. Whitaker, Shifaq Kamili, Lijuan Wang, Janna' R. Murray, Susan I. Gerber, Stephen Lindstrom, Suxiang Tong
EPI_ISL_412862 hCoV-19/USA/CA9/2020 North America / USA / California / Solano 2020-02-23 California Department of Public Health Pathogen Discovery, Respiratory Viruses Branch, Division of Viral Diseases, Centers for Disease Control and Prevention Krista Queen, Anna Uehara, Jing Zhang, Yan Li, Ying Tao, Clinton R. Paden, Haibin Wang, Shifaq Kamili, Xiaoyan Lu, Brian Lynch, Senthil Kumar K. Sakthivel, Brett L. Whitaker, Lijuan Wang, Janna' R. Murray, Jasmine Padilla, Justin Lee, Susan I. Gerber, Stephen Lindstrom, Suxiang Tong
EPI_ISL_412869 hCoV-19/South Korea/KCDC05/2020 Asia / South Korea / Seoul 2020-01-30 Division of Viral Diseases, Center for Laboratory Control of Infectious Diseases, Korea Centers for Diseases Control and Prevention Division of Viral Diseases, Center for Laboratory Control of Infectious Diseases, Korea Centers for Diseases Control and Prevention Jeong-Min Kim, Yoon-Seok Chung, Namjoo Lee, Mi-Seon Kim, Sang Hee Woo, Hye-Jun Jo, Sehee Park, Heui Man Kim, Myung Guk Han
EPI_ISL_412870 hCoV-19/South Korea/KCDC06/2020 Asia / South Korea / Seoul 2020-01-30 Division of Viral Diseases, Center for Laboratory Control of Infectious Diseases, Korea Centers for Diseases Control and Prevention Division of Viral Diseases, Center for Laboratory Control of Infectious Diseases, Korea Centers for Diseases Control and Prevention Jeong-Min Kim, Yoon-Seok Chung, Namjoo Lee, Mi-Seon Kim, Sang Hee Woo, Hye-Jun Jo, Sehee Park, Heui Man Kim, Myung Guk Han
EPI_ISL_412871 hCoV-19/South Korea/KCDC07/2020 Asia / South Korea / Seoul 2020-01-31 Division of Viral Diseases, Center for Laboratory Control of Infectious Diseases, Korea Centers for Diseases Control and Prevention Division of Viral Diseases, Center for Laboratory Control of Infectious Diseases, Korea Centers for Diseases Control and Prevention Jeong-Min Kim, Yoon-Seok Chung, Namjoo Lee, Mi-Seon Kim, Sang Hee Woo, Hye-Jun Jo, Sehee Park, Heui Man Kim, Myung Guk Han
EPI_ISL_412872 hCoV-19/South Korea/KCDC12/2020 Asia / South Korea / Gyeonggi-do 2020-02-01 Division of Viral Diseases, Center for Laboratory Control of Infectious Diseases, Korea Centers for Diseases Control and Prevention Division of Viral Diseases, Center for Laboratory Control of Infectious Diseases, Korea Centers for Diseases Control and Prevention Jeong-Min Kim, Yoon-Seok Chung, Namjoo Lee, Mi-Seon Kim, Sang

Hee Woo, Hye-Jun Jo, Sehee Park, Heui Man Kim, Myung Guk Han
EPI_ISL_412873 hCoV-19/South Korea/KCDC24/2020 Asia / South Korea / Chungcheongnam-do 2020-02-06 Division of Viral Diseases, Center for Laboratory Control of Infectious Diseases, Korea Centers for Diseases Control and Prevention Division of Viral Diseases, Center for Laboratory Control of Infectious Diseases, Korea Centers for Diseases Control and Prevention Jeong-Min Kim, Yoon-Seok Chung, Namjoo Lee, Mi-Seon Kim, Sang Hee Woo, Hye-Jun Jo, Sehee Park, Heui Man Kim, Myung Guk Han
EPI_ISL_413996 hCoV-19/Switzerland/TI9486/2020 Europe / Switzerland / Tessin 2020-02-24 Laboratoire de Virologie, HUG Swiss National Reference Centre for Influenza LAUBSCHER Florian et al.
EPI_ISL_413997 hCoV-19/Switzerland/GE3895/2020 Europe / Switzerland / Geneva 2020-02-26 Laboratoire de Virologie, HUG Swiss National Reference Centre for Influenza LAUBSCHER Florian et al.
EPI_ISL_413999 hCoV-19/Switzerland/AG0361/2020 Europe / Switzerland / Argovie 2020-02-27 Laboratoire de Virologie, HUG Swiss National Reference Centre for Influenza LAUBSCHER Florian et al.
EPI_ISL_415041 hCoV-19/Wales/PHW04/2020 Europe / United Kingdom / Wales 2020-03-07 Wales Specialist Virology Centre Public Health Wales Microbiology Cardiff Catherine Moore, Joanne Watkins, Sally Corden, Tom Connor
EPI_ISL_416042 hCoV-19/Hangzhou/ZJU-02/2020 Asia / China / Hangzhou 2020-01-26 State Key Laboratory for Diagnosis and Treatment of Infectious Diseases, National Clinical Research Center for Infectious Diseases, First Affiliated Hospital, Zhejiang University School of Medicine, Hangzhou, China. 310003 State Key Laboratory for Diagnosis and Treatment of Infectious Diseases, National Clinical Research Center for Infectious Diseases, First Affiliated Hospital, Zhejiang University School of Medicine, Hangzhou, China. 310003 Hangping Yao, Nanping Wu, Chao Jiang, Xiangyun Lu, Linfang Cheng, Fumin Liu, Zhigang Wu, Haibo Wu, Changzhong Jin, Min Zheng, Lanjuan Li
EPI_ISL_416044 hCoV-19/Hangzhou/ZJU-03/2020 Asia / China / Hangzhou 2020-01-25 State Key Laboratory for Diagnosis and Treatment of Infectious Diseases, National Clinical Research Center for Infectious Diseases, First Affiliated Hospital, Zhejiang University School of Medicine, Hangzhou, China 310003 State Key Laboratory for Diagnosis and Treatment of Infectious Diseases, National Clinical Research Center for Infectious Diseases, First Affiliated Hospital, Zhejiang University School of Medicine, Hangzhou, China 310003 Hangping Yao, Nanping Wu, Chao Jiang, Xiangyun Lu, Linfang Cheng, Fumin Liu, Zhigang Wu, Haibo Wu, Changzhong Jin, Min Zheng, Lanjuan Li
EPI_ISL_416046 hCoV-19/Hangzhou/ZJU-04/2020 Asia / China / Hangzhou 2020-01-24 State Key Laboratory for Diagnosis and Treatment of Infectious Diseases, National Clinical Research Center for Infectious Diseases, First Affiliated Hospital, Zhejiang University School of Medicine, Hangzhou, China 310003 State Key Laboratory for Diagnosis and Treatment of Infectious Diseases, National Clinical Research Center for Infectious Diseases, First Affiliated Hospital, Zhejiang University School of Medicine, Hangzhou, China 310003 Hangping Yao, Nanping Wu, Chao Jiang, Xiangyun Lu, Linfang Cheng, Fumin Liu, Zhigang Wu, Haibo Wu, Changzhong Jin, Min Zheng, Lanjuan Li
EPI_ISL_416047 hCoV-19/Hangzhou/ZJU-06/2020 Asia / China / Hangzhou 2020-02-02 State Key Laboratory for Diagnosis and Treatment of Infectious Diseases, National Clinical Research Center for Infectious Diseases, First Affiliated Hospital, Zhejiang University School of Medicine, Hangzhou, China 310003 State Key Laboratory for Diagnosis and Treatment of Infectious Diseases, National Clinical Research Center for Infectious Diseases, First Affiliated Hospital, Zhejiang University School of Medicine, Hangzhou, China 310003 Hangping Yao, Nanping Wu, Chao Jiang, Xiangyun Lu, Linfang Cheng, Fumin Liu, Zhigang Wu, Haibo Wu, Changzhong Jin, Min Zheng, Lanjuan Li
EPI_ISL_416314 hCoV-19/Hong Kong/CUHK1/2020 Asia / Hong Kong 2020-02-07 Department of Microbiology, Faculty of Medicine, The Chinese University of Hong Kong, Hong Kong SAR, China Department of Microbiology, Faculty of Medicine, Chinese University of Hong Kong, Hong Kong SAR, China Zigui Chen, Paul KS Chan
EPI_ISL_416316 hCoV-19/Shanghai/SH0002/2020 Asia / China / Shanghai 2020-01-25 Shanghai Public Health Clinical Center, Shanghai Medical College, Fudan University National Research Center for Translational Medicine (Shanghai), Ruijin Hospital affiliated to Shanghai Jiao Tong University School of Medicine & Shanghai Public Health Clinical Center Shengyue Wang, Xiaonan Zhang, Gang Lu, Yun Tan, Yun Ling, Hongzhou Lu, Saijuan Chen
EPI_ISL_416317 hCoV-19/Shanghai/SH0003/2020 Asia / China / Shanghai 2020-01-25 Shanghai Public Health Clinical Center, Shanghai Medical College, Fudan University National Research Center for Translational Medicine (Shanghai), Ruijin Hospital affiliated to Shanghai Jiao Tong University School of Medicine & Shanghai Public Health Clinical Center Shengyue Wang, Xiaonan Zhang, Gang Lu, Yun Tan, Yun Ling, Hongzhou Lu, Saijuan Chen
EPI_ISL_416318 hCoV-19/Shanghai/SH0004/2020 Asia / China / Shanghai 2020-01-28 Shanghai Public Health Clinical Center, Shanghai Medical College, Fudan University National Research Center for Translational Medicine (Shanghai), Ruijin Hospital affiliated to Shanghai Jiao Tong University School of Medicine & Shanghai Public Health Clinical Center Shengyue Wang, Xiaonan Zhang, Gang Lu, Yun Tan, Yun Ling, Hongzhou Lu, Saijuan Chen
EPI_ISL_416319 hCoV-19/Shanghai/SH0005/2020 Asia / China / Shanghai 2020-01-28 Shanghai Public Health Clinical Center, Shanghai Medical College, Fudan University National Research Center for Translational Medicine (Shanghai), Ruijin Hospital affiliated to Shanghai Jiao Tong University School of Medicine & Shanghai Public Health Clinical Center Shengyue Wang, Xiaonan Zhang, Gang Lu, Yun Tan, Yun Ling, Hongzhou Lu, Saijuan Chen
EPI_ISL_416320 hCoV-19/Shanghai/SH0007/2020 Asia / China / Shanghai 2020-01-28 Shanghai Public Health Clinical Center, Shanghai Medical College, Fudan University National Research Center for Translational Medicine (Shanghai), Ruijin Hospital affiliated to Shanghai Jiao Tong University School of Medicine & Shanghai

Public Health Clinical Center   Shengyue Wang, Xiaonan Zhang, Gang Lu, Yun Tan, Yun Ling, Hongzhou Lu, Saijuan Chen
EPI_ISL_416321 hCoV-19/Shanghai/SH0008/2020   Asia / China / Shanghai 2020-01-28   Shanghai Public Health Clinical Center, Shanghai Medical College, Fudan University   National Research Center for Translational Medicine (Shanghai), Ruijin Hospital affiliated to Shanghai Jiao Tong University School of Medicine & Shanghai Public Health Clinical Center   Shengyue Wang, Xiaonan Zhang, Gang Lu, Yun Tan, Yun Ling, Hongzhou Lu, Saijuan Chen
EPI_ISL_416322 hCoV-19/Shanghai/SH0009/2020   Asia / China / Shanghai 2020-01-29   Shanghai Public Health Clinical Center, Shanghai Medical College, Fudan University   National Research Center for Translational Medicine (Shanghai), Ruijin Hospital affiliated to Shanghai Jiao Tong University School of Medicine & Shanghai Public Health Clinical Center   Shengyue Wang, Xiaonan Zhang, Gang Lu, Yun Tan, Yun Ling, Hongzhou Lu, Saijuan Chen
EPI_ISL_416323 hCoV-19/Shanghai/SH0010/2020   Asia / China / Shanghai 2020-01-29   Shanghai Public Health Clinical Center, Shanghai Medical College, Fudan University   National Research Center for Translational Medicine (Shanghai), Ruijin Hospital affiliated to Shanghai Jiao Tong University School of Medicine & Shanghai Public Health Clinical Center   Shengyue Wang, Xiaonan Zhang, Gang Lu, Yun Tan, Yun Ling, Hongzhou Lu, Saijuan Chen
EPI_ISL_416324 hCoV-19/Shanghai/SH0011/2020   Asia / China / Shanghai 2020-01-29   Shanghai Public Health Clinical Center, Shanghai Medical College, Fudan University   National Research Center for Translational Medicine (Shanghai), Ruijin Hospital affiliated to Shanghai Jiao Tong University School of Medicine & Shanghai Public Health Clinical Center   Shengyue Wang, Xiaonan Zhang, Gang Lu, Yun Tan, Yun Ling, Hongzhou Lu, Saijuan Chen
EPI_ISL_416325 hCoV-19/Shanghai/SH0012/2020   Asia / China / Shanghai 2020-02-02   Shanghai Public Health Clinical Center, Shanghai Medical College, Fudan University   National Research Center for Translational Medicine (Shanghai), Ruijin Hospital affiliated to Shanghai Jiao Tong University School of Medicine & Shanghai Public Health Clinical Center   Shengyue Wang, Xiaonan Zhang, Gang Lu, Yun Tan, Yun Ling, Hongzhou Lu, Saijuan Chen
EPI_ISL_416326 hCoV-19/Shanghai/SH0013/2020   Asia / China / Shanghai 2020-01-30   Shanghai Public Health Clinical Center, Shanghai Medical College, Fudan University   National Research Center for Translational Medicine (Shanghai), Ruijin Hospital affiliated to Shanghai Jiao Tong University School of Medicine & Shanghai Public Health Clinical Center   Shengyue Wang, Xiaonan Zhang, Gang Lu, Yun Tan, Yun Ling, Hongzhou Lu, Saijuan Chen
EPI_ISL_416329 hCoV-19/Shanghai/SH0020/2020   Asia / China / Shanghai 2020-02-01   Shanghai Public Health Clinical Center, Shanghai Medical College, Fudan University   National Research Center for Translational Medicine (Shanghai), Ruijin Hospital affiliated to Shanghai Jiao Tong University School of Medicine & Shanghai Public Health Clinical Center   Shengyue Wang, Xiaonan Zhang, Gang Lu, Yun Tan, Yun Ling, Hongzhou Lu, Saijuan Chen
EPI_ISL_416330 hCoV-19/Shanghai/SH0021/2020   Asia / China / Shanghai 2020-02-02   Shanghai Public Health Clinical Center, Shanghai Medical College, Fudan University   National Research Center for Translational Medicine (Shanghai), Ruijin Hospital affiliated to Shanghai Jiao Tong University School of Medicine & Shanghai Public Health Clinical Center   Shengyue Wang, Xiaonan Zhang, Gang Lu, Yun Tan, Yun Ling, Hongzhou Lu, Saijuan Chen
EPI_ISL_416331 hCoV-19/Shanghai/SH0022/2020   Asia / China / Shanghai 2020-01-30   Shanghai Public Health Clinical Center, Shanghai Medical College, Fudan University   National Research Center for Translational Medicine (Shanghai), Ruijin Hospital affiliated to Shanghai Jiao Tong University School of Medicine & Shanghai Public Health Clinical Center   Shengyue Wang, Xiaonan Zhang, Gang Lu, Yun Tan, Yun Ling, Hongzhou Lu, Saijuan Chen
EPI_ISL_416332 hCoV-19/Shanghai/SH0023/2020   Asia / China / Shanghai 2020-01-30   Shanghai Public Health Clinical Center, Shanghai Medical College, Fudan University   National Research Center for Translational Medicine (Shanghai), Ruijin Hospital affiliated to Shanghai Jiao Tong University School of Medicine & Shanghai Public Health Clinical Center   Shengyue Wang, Xiaonan Zhang, Gang Lu, Yun Tan, Yun Ling, Hongzhou Lu, Saijuan Chen
EPI_ISL_416333 hCoV-19/Shanghai/SH0024/2020   Asia / China / Shanghai 2020-01-30   Shanghai Public Health Clinical Center, Shanghai Medical College, Fudan University   National Research Center for Translational Medicine (Shanghai), Ruijin Hospital affiliated to Shanghai Jiao Tong University School of Medicine & Shanghai Public Health Clinical Center   Shengyue Wang, Xiaonan Zhang, Gang Lu, Yun Tan, Yun Ling, Hongzhou Lu, Saijuan Chen
EPI_ISL_416334 hCoV-19/Shanghai/SH0025/2020   Asia / China / Shanghai 2020-02-06   Shanghai Public Health Clinical Center, Shanghai Medical College, Fudan University   National Research Center for Translational Medicine (Shanghai), Ruijin Hospital affiliated to Shanghai Jiao Tong University School of Medicine & Shanghai Public Health Clinical Center   Shengyue Wang, Xiaonan Zhang, Gang Lu, Yun Tan, Yun Ling, Hongzhou Lu, Saijuan Chen
EPI_ISL_416335 hCoV-19/Shanghai/SH0026/2020   Asia / China / Shanghai 2020-02-02   Shanghai Public Health Clinical Center, Shanghai Medical College, Fudan University   National Research Center for Translational Medicine (Shanghai), Ruijin Hospital affiliated to Shanghai Jiao Tong University School of Medicine & Shanghai Public Health Clinical Center   Shengyue Wang, Xiaonan Zhang, Gang Lu, Yun Tan, Yun Ling, Hongzhou Lu, Saijuan Chen
EPI_ISL_416336 hCoV-19/Shanghai/SH0027/2020   Asia / China / Shanghai 2020-02-01   Shanghai Public Health Clinical Center, Shanghai Medical College, Fudan University   National Research Center for Translational Medicine (Shanghai), Ruijin Hospital affiliated to Shanghai Jiao Tong University School of Medicine & Shanghai

Public Health Clinical Center	Shengyue Wang, Xiaonan Zhang, Gang Lu, Yun Tan, Yun Ling, Hongzhou Lu, Saijuan Chen
EPI_ISL_416337	hCoV-19/Shanghai/SH0028/2020	Asia / China / Shanghai	2020-02-04	Shanghai Public Health Clinical Center, Shanghai Medical College, Fudan University	National Research Center for Translational Medicine (Shanghai), Ruijin Hospital affiliated to Shanghai Jiao Tong University School of Medicine & Shanghai Public Health Clinical Center	Shengyue Wang, Xiaonan Zhang, Gang Lu, Yun Tan, Yun Ling, Hongzhou Lu, Saijuan Chen
EPI_ISL_416338	hCoV-19/Shanghai/SH0029/2020	Asia / China / Shanghai	2020-02-01	Shanghai Public Health Clinical Center, Shanghai Medical College, Fudan University	National Research Center for Translational Medicine (Shanghai), Ruijin Hospital affiliated to Shanghai Jiao Tong University School of Medicine & Shanghai Public Health Clinical Center	Shengyue Wang, Xiaonan Zhang, Gang Lu, Yun Tan, Yun Ling, Hongzhou Lu, Saijuan Chen
EPI_ISL_416339	hCoV-19/Shanghai/SH0030/2020	Asia / China / Shanghai	2020-02-03	Shanghai Public Health Clinical Center, Shanghai Medical College, Fudan University	National Research Center for Translational Medicine (Shanghai), Ruijin Hospital affiliated to Shanghai Jiao Tong University School of Medicine & Shanghai Public Health Clinical Center	Shengyue Wang, Xiaonan Zhang, Gang Lu, Yun Tan, Yun Ling, Hongzhou Lu, Saijuan Chen
EPI_ISL_416340	hCoV-19/Shanghai/SH0031/2020	Asia / China / Shanghai	2020-02-02	Shanghai Public Health Clinical Center, Shanghai Medical College, Fudan University	National Research Center for Translational Medicine (Shanghai), Ruijin Hospital affiliated to Shanghai Jiao Tong University School of Medicine & Shanghai Public Health Clinical Center	Shengyue Wang, Xiaonan Zhang, Gang Lu, Yun Tan, Yun Ling, Hongzhou Lu, Saijuan Chen
EPI_ISL_416341	hCoV-19/Shanghai/SH0032/2020	Asia / China / Shanghai	2020-02-01	Shanghai Public Health Clinical Center, Shanghai Medical College, Fudan University	National Research Center for Translational Medicine (Shanghai), Ruijin Hospital affiliated to Shanghai Jiao Tong University School of Medicine & Shanghai Public Health Clinical Center	Shengyue Wang, Xiaonan Zhang, Gang Lu, Yun Tan, Yun Ling, Hongzhou Lu, Saijuan Chen
EPI_ISL_416342	hCoV-19/Shanghai/SH0033/2020	Asia / China / Shanghai	2020-02-04	Shanghai Public Health Clinical Center, Shanghai Medical College, Fudan University	National Research Center for Translational Medicine (Shanghai), Ruijin Hospital affiliated to Shanghai Jiao Tong University School of Medicine & Shanghai Public Health Clinical Center	Shengyue Wang, Xiaonan Zhang, Gang Lu, Yun Tan, Yun Ling, Hongzhou Lu, Saijuan Chen
EPI_ISL_416348	hCoV-19/Shanghai/SH0039/2020	Asia / China / Shanghai	2020-02-06	Shanghai Public Health Clinical Center, Shanghai Medical College, Fudan University	National Research Center for Translational Medicine (Shanghai), Ruijin Hospital affiliated to Shanghai Jiao Tong University School of Medicine & Shanghai Public Health Clinical Center	Shengyue Wang, Xiaonan Zhang, Gang Lu, Yun Tan, Yun Ling, Hongzhou Lu, Saijuan Chen
EPI_ISL_416349	hCoV-19/Shanghai/SH0040/2020	Asia / China / Shanghai	2020-01-30	Shanghai Public Health Clinical Center, Shanghai Medical College, Fudan University	National Research Center for Translational Medicine (Shanghai), Ruijin Hospital affiliated to Shanghai Jiao Tong University School of Medicine & Shanghai Public Health Clinical Center	Shengyue Wang, Xiaonan Zhang, Gang Lu, Yun Tan, Yun Ling, Hongzhou Lu, Saijuan Chen
EPI_ISL_416350	hCoV-19/Shanghai/SH0041/2020	Asia / China / Shanghai	2020-01-29	Shanghai Public Health Clinical Center, Shanghai Medical College, Fudan University	National Research Center for Translational Medicine (Shanghai), Ruijin Hospital affiliated to Shanghai Jiao Tong University School of Medicine & Shanghai Public Health Clinical Center	Shengyue Wang, Xiaonan Zhang, Gang Lu, Yun Tan, Yun Ling, Hongzhou Lu, Saijuan Chen
EPI_ISL_416352	hCoV-19/Shanghai/SH0043/2020	Asia / China / Shanghai	2020-01-30	Shanghai Public Health Clinical Center, Shanghai Medical College, Fudan University	National Research Center for Translational Medicine (Shanghai), Ruijin Hospital affiliated to Shanghai Jiao Tong University School of Medicine & Shanghai Public Health Clinical Center	Shengyue Wang, Xiaonan Zhang, Gang Lu, Yun Tan, Yun Ling, Hongzhou Lu, Saijuan Chen
EPI_ISL_416353	hCoV-19/Shanghai/SH0044/2020	Asia / China / Shanghai	2020-02-04	Shanghai Public Health Clinical Center, Shanghai Medical College, Fudan University	National Research Center for Translational Medicine (Shanghai), Ruijin Hospital affiliated to Shanghai Jiao Tong University School of Medicine & Shanghai Public Health Clinical Center	Shengyue Wang, Xiaonan Zhang, Gang Lu, Yun Tan, Yun Ling, Hongzhou Lu, Saijuan Chen
EPI_ISL_416354	hCoV-19/Shanghai/SH0045/2020	Asia / China / Shanghai	2020-02-04	Shanghai Public Health Clinical Center, Shanghai Medical College, Fudan University	National Research Center for Translational Medicine (Shanghai), Ruijin Hospital affiliated to Shanghai Jiao Tong University School of Medicine & Shanghai Public Health Clinical Center	Shengyue Wang, Xiaonan Zhang, Gang Lu, Yun Tan, Yun Ling, Hongzhou Lu, Saijuan Chen
EPI_ISL_416355	hCoV-19/Shanghai/SH0047/2020	Asia / China / Shanghai	2020-02-04	Shanghai Public Health Clinical Center, Shanghai Medical College, Fudan University	National Research Center for Translational Medicine (Shanghai), Ruijin Hospital affiliated to Shanghai Jiao Tong University School of Medicine & Shanghai Public Health Clinical Center	Shengyue Wang, Xiaonan Zhang, Gang Lu, Yun Tan, Yun Ling, Hongzhou Lu, Saijuan Chen
EPI_ISL_416358	hCoV-19/Shanghai/SH0050/2020	Asia / China / Shanghai	2020-02-05	Shanghai Public Health Clinical Center, Shanghai Medical College, Fudan University	National Research Center for Translational Medicine (Shanghai), Ruijin Hospital affiliated to Shanghai Jiao Tong University School of Medicine & Shanghai

Public Health Clinical Center   Shengyue Wang, Xiaonan Zhang, Gang Lu, Yun Tan, Yun Ling, Hongzhou Lu, Saijuan Chen
EPI_ISL_416359 hCoV-19/Shanghai/SH0051/2020   Asia / China / Shanghai 2020-02-06   Shanghai Public Health Clinical Center, Shanghai Medical College, Fudan University   National Research Center for Translational Medicine (Shanghai), Ruijin Hospital affiliated to Shanghai Jiao Tong University School of Medicine & Shanghai Public Health Clinical Center   Shengyue Wang, Xiaonan Zhang, Gang Lu, Yun Tan, Yun Ling, Hongzhou Lu, Saijuan Chen
EPI_ISL_416361 hCoV-19/Shanghai/SH0053/2020   Asia / China / Shanghai 2020-02-01   Shanghai Public Health Clinical Center, Shanghai Medical College, Fudan University   National Research Center for Translational Medicine (Shanghai), Ruijin Hospital affiliated to Shanghai Jiao Tong University School of Medicine & Shanghai Public Health Clinical Center   Shengyue Wang, Xiaonan Zhang, Gang Lu, Yun Tan, Yun Ling, Hongzhou Lu, Saijuan Chen
EPI_ISL_416362 hCoV-19/Shanghai/SH0054/2020   Asia / China / Shanghai 2020-02-02   Shanghai Public Health Clinical Center, Shanghai Medical College, Fudan University   National Research Center for Translational Medicine (Shanghai), Ruijin Hospital affiliated to Shanghai Jiao Tong University School of Medicine & Shanghai Public Health Clinical Center   Shengyue Wang, Xiaonan Zhang, Gang Lu, Yun Tan, Yun Ling, Hongzhou Lu, Saijuan Chen
EPI_ISL_416363 hCoV-19/Shanghai/SH0055/2020   Asia / China / Shanghai 2020-02-08   Shanghai Public Health Clinical Center, Shanghai Medical College, Fudan University   National Research Center for Translational Medicine (Shanghai), Ruijin Hospital affiliated to Shanghai Jiao Tong University School of Medicine & Shanghai Public Health Clinical Center   Shengyue Wang, Xiaonan Zhang, Gang Lu, Yun Tan, Yun Ling, Hongzhou Lu, Saijuan Chen
EPI_ISL_416364 hCoV-19/Shanghai/SH0056/2020   Asia / China / Shanghai 2020-02-08   Shanghai Public Health Clinical Center, Shanghai Medical College, Fudan University   National Research Center for Translational Medicine (Shanghai), Ruijin Hospital affiliated to Shanghai Jiao Tong University School of Medicine & Shanghai Public Health Clinical Center   Shengyue Wang, Xiaonan Zhang, Gang Lu, Yun Tan, Yun Ling, Hongzhou Lu, Saijuan Chen
EPI_ISL_416365 hCoV-19/Shanghai/SH0058/2020   Asia / China / Shanghai 2020-01-30   Shanghai Public Health Clinical Center, Shanghai Medical College, Fudan University   National Research Center for Translational Medicine (Shanghai), Ruijin Hospital affiliated to Shanghai Jiao Tong University School of Medicine & Shanghai Public Health Clinical Center   Shengyue Wang, Xiaonan Zhang, Gang Lu, Yun Tan, Yun Ling, Hongzhou Lu, Saijuan Chen
EPI_ISL_416366 hCoV-19/Shanghai/SH0059/2020   Asia / China / Shanghai 2020-01-30   Shanghai Public Health Clinical Center, Shanghai Medical College, Fudan University   National Research Center for Translational Medicine (Shanghai), Ruijin Hospital affiliated to Shanghai Jiao Tong University School of Medicine & Shanghai Public Health Clinical Center   Shengyue Wang, Xiaonan Zhang, Gang Lu, Yun Tan, Yun Ling, Hongzhou Lu, Saijuan Chen
EPI_ISL_416367 hCoV-19/Shanghai/SH0060/2020   Asia / China / Shanghai 2020-02-01   Shanghai Public Health Clinical Center, Shanghai Medical College, Fudan University   National Research Center for Translational Medicine (Shanghai), Ruijin Hospital affiliated to Shanghai Jiao Tong University School of Medicine & Shanghai Public Health Clinical Center   Shengyue Wang, Xiaonan Zhang, Gang Lu, Yun Tan, Yun Ling, Hongzhou Lu, Saijuan Chen
EPI_ISL_416368 hCoV-19/Shanghai/SH0065/2020   Asia / China / Shanghai 2020-02-05   Shanghai Public Health Clinical Center, Shanghai Medical College, Fudan University   National Research Center for Translational Medicine (Shanghai), Ruijin Hospital affiliated to Shanghai Jiao Tong University School of Medicine & Shanghai Public Health Clinical Center   Shengyue Wang, Xiaonan Zhang, Gang Lu, Yun Tan, Yun Ling, Hongzhou Lu, Saijuan Chen
EPI_ISL_416369 hCoV-19/Shanghai/SH0066/2020   Asia / China / Shanghai 2020-02-05   Shanghai Public Health Clinical Center, Shanghai Medical College, Fudan University   National Research Center for Translational Medicine (Shanghai), Ruijin Hospital affiliated to Shanghai Jiao Tong University School of Medicine & Shanghai Public Health Clinical Center   Shengyue Wang, Xiaonan Zhang, Gang Lu, Yun Tan, Yun Ling, Hongzhou Lu, Saijuan Chen
EPI_ISL_416370 hCoV-19/Shanghai/SH0067/2020   Asia / China / Shanghai 2020-02-01   Shanghai Public Health Clinical Center, Shanghai Medical College, Fudan University   National Research Center for Translational Medicine (Shanghai), Ruijin Hospital affiliated to Shanghai Jiao Tong University School of Medicine & Shanghai Public Health Clinical Center   Shengyue Wang, Xiaonan Zhang, Gang Lu, Yun Tan, Yun Ling, Hongzhou Lu, Saijuan Chen
EPI_ISL_416372 hCoV-19/Shanghai/SH0069/2020   Asia / China / Shanghai 2020-02-01   Shanghai Public Health Clinical Center, Shanghai Medical College, Fudan University   National Research Center for Translational Medicine (Shanghai), Ruijin Hospital affiliated to Shanghai Jiao Tong University School of Medicine & Shanghai Public Health Clinical Center   Shengyue Wang, Xiaonan Zhang, Gang Lu, Yun Tan, Yun Ling, Hongzhou Lu, Saijuan Chen
EPI_ISL_416373 hCoV-19/Shanghai/SH0070/2020   Asia / China / Shanghai 2020-02-07   Shanghai Public Health Clinical Center, Shanghai Medical College, Fudan University   National Research Center for Translational Medicine (Shanghai), Ruijin Hospital affiliated to Shanghai Jiao Tong University School of Medicine & Shanghai Public Health Clinical Center   Shengyue Wang, Xiaonan Zhang, Gang Lu, Yun Tan, Yun Ling, Hongzhou Lu, Saijuan Chen
EPI_ISL_416376 hCoV-19/Shanghai/SH0073/2020   Asia / China / Shanghai 2020-02-05   Shanghai Public Health Clinical Center, Shanghai Medical College, Fudan University   National Research Center for Translational Medicine (Shanghai), Ruijin Hospital affiliated to Shanghai Jiao Tong University School of Medicine & Shanghai

Public Health Clinical Center	Shengyue Wang, Xiaonan Zhang, Gang Lu, Yun Tan, Yun Ling, Hongzhou Lu, Saijuan Chen
EPI_ISL_416377	hCoV-19/Shanghai/SH0074/2020	Asia / China / Shanghai 2020-02-07	Shanghai Public Health Clinical Center, Shanghai Medical College, Fudan University	National Research Center for Translational Medicine (Shanghai), Ruijin Hospital affiliated to Shanghai Jiao Tong University School of Medicine & Shanghai Public Health Clinical Center	Shengyue Wang, Xiaonan Zhang, Gang Lu, Yun Tan, Yun Ling, Hongzhou Lu, Saijuan Chen
EPI_ISL_416378	hCoV-19/Shanghai/SH0075/2020	Asia / China / Shanghai 2020-01-30	Shanghai Public Health Clinical Center, Shanghai Medical College, Fudan University	National Research Center for Translational Medicine (Shanghai), Ruijin Hospital affiliated to Shanghai Jiao Tong University School of Medicine & Shanghai Public Health Clinical Center	Shengyue Wang, Xiaonan Zhang, Gang Lu, Yun Tan, Yun Ling, Hongzhou Lu, Saijuan Chen
EPI_ISL_416379	hCoV-19/Shanghai/SH0076/2020	Asia / China / Shanghai 2020-02-01	Shanghai Public Health Clinical Center, Shanghai Medical College, Fudan University	National Research Center for Translational Medicine (Shanghai), Ruijin Hospital affiliated to Shanghai Jiao Tong University School of Medicine & Shanghai Public Health Clinical Center	Shengyue Wang, Xiaonan Zhang, Gang Lu, Yun Tan, Yun Ling, Hongzhou Lu, Saijuan Chen
EPI_ISL_416380	hCoV-19/Shanghai/SH0077/2020	Asia / China / Shanghai 2020-02-09	Shanghai Public Health Clinical Center, Shanghai Medical College, Fudan University	National Research Center for Translational Medicine (Shanghai), Ruijin Hospital affiliated to Shanghai Jiao Tong University School of Medicine & Shanghai Public Health Clinical Center	Shengyue Wang, Xiaonan Zhang, Gang Lu, Yun Tan, Yun Ling, Hongzhou Lu, Saijuan Chen
EPI_ISL_416381	hCoV-19/Shanghai/SH0079/2020	Asia / China / Shanghai 2020-02-01	Shanghai Public Health Clinical Center, Shanghai Medical College, Fudan University	National Research Center for Translational Medicine (Shanghai), Ruijin Hospital affiliated to Shanghai Jiao Tong University School of Medicine & Shanghai Public Health Clinical Center	Shengyue Wang, Xiaonan Zhang, Gang Lu, Yun Tan, Yun Ling, Hongzhou Lu, Saijuan Chen
EPI_ISL_416382	hCoV-19/Shanghai/SH0080/2020	Asia / China / Shanghai 2020-02-08	Shanghai Public Health Clinical Center, Shanghai Medical College, Fudan University	National Research Center for Translational Medicine (Shanghai), Ruijin Hospital affiliated to Shanghai Jiao Tong University School of Medicine & Shanghai Public Health Clinical Center	Shengyue Wang, Xiaonan Zhang, Gang Lu, Yun Tan, Yun Ling, Hongzhou Lu, Saijuan Chen
EPI_ISL_416384	hCoV-19/Shanghai/SH0083/2020	Asia / China / Shanghai 2020-02-04	Shanghai Public Health Clinical Center, Shanghai Medical College, Fudan University	National Research Center for Translational Medicine (Shanghai), Ruijin Hospital affiliated to Shanghai Jiao Tong University School of Medicine & Shanghai Public Health Clinical Center	Shengyue Wang, Xiaonan Zhang, Gang Lu, Yun Tan, Yun Ling, Hongzhou Lu, Saijuan Chen
EPI_ISL_416387	hCoV-19/Shanghai/SH0088/2020	Asia / China / Shanghai 2020-02-01	Shanghai Public Health Clinical Center, Shanghai Medical College, Fudan University	National Research Center for Translational Medicine (Shanghai), Ruijin Hospital affiliated to Shanghai Jiao Tong University School of Medicine & Shanghai Public Health Clinical Center	Shengyue Wang, Xiaonan Zhang, Gang Lu, Yun Tan, Yun Ling, Hongzhou Lu, Saijuan Chen
EPI_ISL_416389	hCoV-19/Shanghai/SH0093/2020	Asia / China / Shanghai 2020-01-21	Shanghai Public Health Clinical Center, Shanghai Medical College, Fudan University	National Research Center for Translational Medicine (Shanghai), Ruijin Hospital affiliated to Shanghai Jiao Tong University School of Medicine & Shanghai Public Health Clinical Center	Shengyue Wang, Xiaonan Zhang, Gang Lu, Yun Tan, Yun Ling, Hongzhou Lu, Saijuan Chen
EPI_ISL_416390	hCoV-19/Shanghai/SH0094/2020	Asia / China / Shanghai 2020-01-31	Shanghai Public Health Clinical Center, Shanghai Medical College, Fudan University	National Research Center for Translational Medicine (Shanghai), Ruijin Hospital affiliated to Shanghai Jiao Tong University School of Medicine & Shanghai Public Health Clinical Center	Shengyue Wang, Xiaonan Zhang, Gang Lu, Yun Tan, Yun Ling, Hongzhou Lu, Saijuan Chen
EPI_ISL_416393	hCoV-19/Shanghai/SH0101/2020	Asia / China / Shanghai 2020-02-09	Shanghai Public Health Clinical Center, Shanghai Medical College, Fudan University	National Research Center for Translational Medicine (Shanghai), Ruijin Hospital affiliated to Shanghai Jiao Tong University School of Medicine & Shanghai Public Health Clinical Center	Shengyue Wang, Xiaonan Zhang, Gang Lu, Yun Tan, Yun Ling, Hongzhou Lu, Saijuan Chen
EPI_ISL_416394	hCoV-19/Shanghai/SH0102/2020	Asia / China / Shanghai 2020-02-14	Shanghai Public Health Clinical Center, Shanghai Medical College, Fudan University	National Research Center for Translational Medicine (Shanghai), Ruijin Hospital affiliated to Shanghai Jiao Tong University School of Medicine & Shanghai Public Health Clinical Center	Shengyue Wang, Xiaonan Zhang, Gang Lu, Yun Tan, Yun Ling, Hongzhou Lu, Saijuan Chen
EPI_ISL_416396	hCoV-19/Shanghai/SH0106/2020	Asia / China / Shanghai 2020-02-10	Shanghai Public Health Clinical Center, Shanghai Medical College, Fudan University	National Research Center for Translational Medicine (Shanghai), Ruijin Hospital affiliated to Shanghai Jiao Tong University School of Medicine & Shanghai Public Health Clinical Center	Shengyue Wang, Xiaonan Zhang, Gang Lu, Yun Tan, Yun Ling, Hongzhou Lu, Saijuan Chen
EPI_ISL_416397	hCoV-19/Shanghai/SH0107/2020	Asia / China / Shanghai 2020-02-02	Shanghai Public Health Clinical Center, Shanghai Medical College, Fudan University	National Research Center for Translational Medicine (Shanghai), Ruijin Hospital affiliated to Shanghai Jiao Tong University School of Medicine & Shanghai

Public Health Clinical Center	Shengyue Wang, Xiaonan Zhang, Gang Lu, Yun Tan, Yun Ling, Hongzhou Lu, Saijuan Chen
EPI_ISL_416398	hCoV-19/Shanghai/SH0109/2020	Asia / China / Shanghai	2020-02-02	Shanghai Public Health Clinical Center, Shanghai Medical College, Fudan University	National Research Center for Translational Medicine (Shanghai), Ruijin Hospital affiliated to Shanghai Jiao Tong University School of Medicine & Shanghai Public Health Clinical Center	Shengyue Wang, Xiaonan Zhang, Gang Lu, Yun Tan, Yun Ling, Hongzhou Lu, Saijuan Chen
EPI_ISL_416399	hCoV-19/Shanghai/SH0110/2020	Asia / China / Shanghai	2020-02-13	Shanghai Public Health Clinical Center, Shanghai Medical College, Fudan University	National Research Center for Translational Medicine (Shanghai), Ruijin Hospital affiliated to Shanghai Jiao Tong University School of Medicine & Shanghai Public Health Clinical Center	Shengyue Wang, Xiaonan Zhang, Gang Lu, Yun Tan, Yun Ling, Hongzhou Lu, Saijuan Chen
EPI_ISL_417370	hCoV-19/USA/WA-UW221/2020	North America / USA / Washington	2020-03-14	UW Virology Lab	UW Virology Lab	Pavitra Roychoudhury, Hong Xie, Keith Jerome, Alexander Greninger
EPI_ISL_417371	hCoV-19/USA/WA-UW222/2020	North America / USA / Washington	2020-03-13	UW Virology Lab	UW Virology Lab	Pavitra Roychoudhury, Hong Xie, Keith Jerome, Alexander Greninger
EPI_ISL_417372	hCoV-19/USA/CT-UW223/2020	North America / USA / Connecticut	2020-03-13	UW Virology Lab	UW Virology Lab	Pavitra Roychoudhury, Hong Xie, Keith Jerome, Alexander Greninger
EPI_ISL_417373	hCoV-19/USA/CT-UW224/2020	North America / USA / Connecticut	2020-03-13	UW Virology Lab	UW Virology Lab	Pavitra Roychoudhury, Hong Xie, Keith Jerome, Alexander Greninger
EPI_ISL_417374	hCoV-19/USA/WA-UW225/2020	North America / USA / Washington	2020-03-15	UW Virology Lab	UW Virology Lab	Pavitra Roychoudhury, Hong Xie, Keith Jerome, Alexander Greninger
EPI_ISL_417375	hCoV-19/USA/WA-UW226/2020	North America / USA / Washington	2020-03-15	UW Virology Lab	UW Virology Lab	Pavitra Roychoudhury, Hong Xie, Keith Jerome, Alexander Greninger
EPI_ISL_417376	hCoV-19/USA/WA-UW227/2020	North America / USA / Washington	2020-03-15	UW Virology Lab	UW Virology Lab	Pavitra Roychoudhury, Hong Xie, Keith Jerome, Alexander Greninger
EPI_ISL_417377	hCoV-19/USA/WA-UW228/2020	North America / USA / Washington	2020-03-15	UW Virology Lab	UW Virology Lab	Pavitra Roychoudhury, Hong Xie, Keith Jerome, Alexander Greninger
EPI_ISL_417378	hCoV-19/USA/WA-UW229/2020	North America / USA / Washington	2020-03-14	UW Virology Lab	UW Virology Lab	Pavitra Roychoudhury, Hong Xie, Keith Jerome, Alexander Greninger
EPI_ISL_417379	hCoV-19/USA/WA-UW230/2020	North America / USA / Washington	2020-03-15	UW Virology Lab	UW Virology Lab	Pavitra Roychoudhury, Hong Xie, Keith Jerome, Alexander Greninger
EPI_ISL_417380	hCoV-19/USA/WA-UW231/2020	North America / USA / Washington	2020-03-14	UW Virology Lab	UW Virology Lab	Pavitra Roychoudhury, Hong Xie, Keith Jerome, Alexander Greninger
EPI_ISL_417381	hCoV-19/USA/WA-UW232/2020	North America / USA / Washington	2020-03-14	UW Virology Lab	UW Virology Lab	Pavitra Roychoudhury, Hong Xie, Keith Jerome, Alexander Greninger
EPI_ISL_417382	hCoV-19/USA/WA-UW233/2020	North America / USA / Washington	2020-03-15	UW Virology Lab	UW Virology Lab	Pavitra Roychoudhury, Hong Xie, Keith Jerome, Alexander Greninger
EPI_ISL_417383	hCoV-19/Australia/NSW15/2020	Oceania / Australia / New South Wales / Sydney	2020-03-03	Centre for Infectious Diseases and Microbiology Public Health	NSW Health Pathology - Institute of Clinical Pathology and Medical Research; Westmead Hospital; University of Sydney	Rockett R, Eden J-S, Lam C, Gray K, Timms V, Gall M, Arnott A, Sadsad R, Carter I, Rahman H, Holmes EC, O’Sullivan MV, Sintchenko V, Chen SC, Maddocks S, Kok J and Dwyer DE for the 2019-nCoV Study Group
EPI_ISL_417384	hCoV-19/Australia/NSW18/2020	Oceania / Australia / New South Wales / Sydney	2020-03-05	Centre for Infectious Diseases and Microbiology Public Health	NSW Health Pathology - Institute of Clinical Pathology and Medical Research; Westmead Hospital; University of Sydney	Eden J-S, Lam C, Gray K, Timms V, Gall M, Arnott A, Sadsad R, Carter I, Rahman H, Holmes EC, O’Sullivan MV, Sintchenko V, Chen SC, Maddocks S, Kok J, Dwyer DE and Rockett R for the 2019-nCoV Study Group
EPI_ISL_417385	hCoV-19/Australia/NSW19/2020	Oceania / Australia / New South Wales / Sydney	2020-03-06	Centre for Infectious Diseases and Microbiology Public Health	NSW Health Pathology - Institute of Clinical Pathology and Medical Research; Westmead Hospital; University of Sydney	Lam C, Gray K, Timms V, Gall M, Arnott A, Sadsad R, Carter I, Rahman H, Holmes EC, O’Sullivan MV, Sintchenko V, Chen SC, Maddocks S, Kok J, Dwyer DE, Rockett R and Eden J-S for the 2019-nCoV Study Group
EPI_ISL_417386	hCoV-19/Australia/NSW21/2020	Oceania / Australia / New South Wales / Sydney	2020-03-04	Centre for Infectious Diseases and Microbiology Public Health	NSW Health Pathology - Institute of Clinical Pathology and Medical Research; Westmead Hospital; University of Sydney	Gray K, Timms V, Gall M, Arnott A, Sadsad R, Carter I, Rahman H, Holmes EC, O’Sullivan MV, Sintchenko V, Chen SC, Maddocks S, Kok J, Dwyer DE, Rockett R, Eden J-S and Lam C for the 2019-nCoV Study Group
EPI_ISL_417387	hCoV-19/Australia/NSW24/2020	Oceania / Australia / New South Wales / Sydney	2020-03-06	Centre for Infectious Diseases and Microbiology Public Health	NSW Health Pathology - Institute of Clinical Pathology and Medical Research; Westmead Hospital; University of Sydney	Timms V, Gall M, Arnott A, Sadsad R, Carter I, Rahman H, Holmes EC, O’Sullivan MV, Sintchenko V, Chen SC, Maddocks S, Kok J, Dwyer DE, Rockett R, Eden J-S, Lam C and Gray K for the 2019-nCoV Study Group
EPI_ISL_417388	hCoV-19/Australia/NSW25/2020	Oceania / Australia / New South Wales / Sydney	2020-03-05	Centre for Infectious Diseases and Microbiology Public Health	NSW Health Pathology - Institute of Clinical Pathology and Medical Research; Westmead Hospital; University of Sydney	Gall M, Arnott A, Sadsad R, Carter I, Rahman H, Holmes EC, O’Sullivan MV, Sintchenko V, Chen SC, Maddocks S, Kok J, Dwyer DE, Rockett R, Eden J-S, Lam C, Gray K and Timms V for the 2019-nCoV Study Group
EPI_ISL_417389	hCoV-19/Australia/NSW26/2020	Oceania / Australia / New South Wales / Sydney	2020-03-09	Centre for Infectious Diseases and Microbiology Public Health	NSW Health Pathology - Institute of Clinical

Pathology and Medical Research; Westmead Hospital; University of Sydney Arnott A, Sadsad R, Carter I, Rahman H, Holmes EC, O’Sullivan MV, Sintchenko V, Chen SC, Maddocks S, Kok J, Dwyer DE, Rockett R, Eden J-S, Lam C, Gray K, Timms V and Gall M for the 2019-nCoV Study Group
EPI_ISL_417507 hCoV-19/USA/WI-UW-23/2020 North America / USA / Wisconsin 2020-03-17 University of Wisconsin-Madison AIDS Vaccine Research Laboratories University of Wisconsin-Madison AIDS Vaccine Research Laboratories Gage Moreno, Katarina Braun, et al. AIDS Vaccine Research Laboratories
EPI_ISL_417508 hCoV-19/USA/WI-UW-21/2020 North America / USA / Wisconsin 2020-03-16 University of Wisconsin-Madison AIDS Vaccine Research Laboratories University of Wisconsin-Madison AIDS Vaccine Research Laboratories Gage Moreno, Katarina Braun, et al. AIDS Vaccine Research Laboratories
EPI_ISL_417509 hCoV-19/USA/WI-UW-16/2020 North America / USA / Wisconsin 2020-03-14 University of Wisconsin-Madison AIDS Vaccine Research Laboratories University of Wisconsin-Madison AIDS Vaccine Research Laboratories Gage Moreno, Katarina Braun, et al. AIDS Vaccine Research Laboratories
EPI_ISL_417510 hCoV-19/USA/WI-UW-19/2020 North America / USA / Wisconsin 2020-03-16 University of Wisconsin-Madison AIDS Vaccine Research Laboratories University of Wisconsin-Madison AIDS Vaccine Research Laboratories Gage Moreno, Katarina Braun, et al. AIDS Vaccine Research Laboratories
EPI_ISL_417512 hCoV-19/USA/WI-UW-24/2020 North America / USA / Wisconsin 2020-03-15 University of Wisconsin-Madison AIDS Vaccine Research Laboratories University of Wisconsin-Madison AIDS Vaccine Research Laboratories Gage Moreno, Katarina Braun, et al. AIDS Vaccine Research Laboratories
EPI_ISL_417513 hCoV-19/USA/WI-UW-14/2020 North America / USA / Wisconsin 2020-03-16 University of Wisconsin-Madison AIDS Vaccine Research Laboratories University of Wisconsin-Madison AIDS Vaccine Research Laboratories Gage Moreno, Katarina Braun, et al. AIDS Vaccine Research Laboratories
EPI_ISL_417514 hCoV-19/USA/WI-UW-22/2020 North America / USA / Wisconsin 2020-03-13 University of Wisconsin-Madison AIDS Vaccine Research Laboratories University of Wisconsin-Madison AIDS Vaccine Research Laboratories Gage Moreno, Katarina Braun, et al. AIDS Vaccine Research Laboratories
EPI_ISL_417515 hCoV-19/USA/WI-UW-18/2020 North America / USA / Wisconsin 2020-03-17 University of Wisconsin-Madison AIDS Vaccine Research Laboratories University of Wisconsin-Madison AIDS Vaccine Research Laboratories Gage Moreno, Katarina Braun, et al. AIDS Vaccine Research Laboratories
EPI_ISL_417516 hCoV-19/USA/WI-UW-13/2020 North America / USA / Wisconsin 2020-03-15 University of Wisconsin-Madison AIDS Vaccine Research Laboratories University of Wisconsin-Madison AIDS Vaccine Research Laboratories Gage Moreno, Katarina Braun, et al. AIDS Vaccine Research Laboratories
EPI_ISL_417517 hCoV-19/USA/WI-UW-17/2020 North America / USA / Wisconsin 2020-03-13 University of Wisconsin-Madison AIDS Vaccine Research Laboratories University of Wisconsin-Madison AIDS Vaccine Research Laboratories Gage Moreno, Katarina Braun, et al. AIDS Vaccine Research Laboratories
EPI_ISL_417519 hCoV-19/Taiwan/CGMH-CGU-06/2020 Asia / Taiwan / Taoyuan 2020-03-05 Laboratory Medicine Department of Laboratory Medicine, Lin-Kou Chang Gung Memorial Hospital, Taoyuan, Taiwan Kuo-Chien Tsao, Yu-Nong Gong, Shu-Li Yang, Yi-Chun Liu, Chung-Guei Huang, Po-Wei Huang, Mei-Jen Hsiao, Cheng-Ta Yang, Cheng-Hsun Chiu, Peng-Nien Huang, Kuo-Ming Lee, Guang-Wu Chen , Shin-Ru Shih
EPI_ISL_417520 hCoV-19/Taiwan/CGMH-CGU-07/2020 Asia / Taiwan / Taoyuan 2020-03-09 Laboratory Medicine Department of Laboratory Medicine, Lin-Kou Chang Gung Memorial Hospital, Taoyuan, Taiwan Kuo-Chien Tsao, Yu-Nong Gong, Shu-Li Yang, Yi-Chun Liu, Chung-Guei Huang, Po-Wei Huang, Mei-Jen Hsiao, Cheng-Ta Yang, Cheng-Hsun Chiu, Peng-Nien Huang, Kuo-Ming Lee, Guang-Wu Chen , Shin-Ru Shih
EPI_ISL_417521 hCoV-19/Taiwan/CGMH-CGU-08/2020 Asia / Taiwan / Taoyuan 2020-03-10 Laboratory Medicine Department of Laboratory Medicine, Lin-Kou Chang Gung Memorial Hospital, Taoyuan, Taiwan Kuo-Chien Tsao, Yu-Nong Gong, Shu-Li Yang, Yi-Chun Liu, Chung-Guei Huang, Po-Wei Huang, Mei-Jen Hsiao, Cheng-Ta Yang, Cheng-Hsun Chiu, Peng-Nien Huang, Kuo-Ming Lee, Guang-Wu Chen , Shin-Ru Shih
EPI_ISL_417522 hCoV-19/Taiwan/CGMH-CGU-09/2020 Asia / Taiwan / Taoyuan 2020-03-13 Laboratory Medicine Department of Laboratory Medicine, Lin-Kou Chang Gung Memorial Hospital, Taoyuan, Taiwan Kuo-Chien Tsao, Yu-Nong Gong, Shu-Li Yang, Yi-Chun Liu, Chung-Guei Huang, Po-Wei Huang, Mei-Jen Hsiao, Cheng-Ta Yang, Cheng-Hsun Chiu, Peng-Nien Huang, Kuo-Ming Lee, Guang-Wu Chen , Shin-Ru Shih
EPI_ISL_417523 hCoV-19/Taiwan/CGMH-CGU-10/2020 Asia / Taiwan / Taoyuan 2020-03-13 Laboratory Medicine Department of Laboratory Medicine, Lin-Kou Chang Gung Memorial Hospital, Taoyuan, Taiwan Kuo-Chien Tsao, Yu-Nong Gong, Shu-Li Yang, Yi-Chun Liu, Chung-Guei Huang, Po-Wei Huang, Mei-Jen Hsiao, Cheng-Ta Yang, Cheng-Hsun Chiu, Peng-Nien Huang, Kuo-Ming Lee, Guang-Wu Chen , Shin-Ru Shih
EPI_ISL_417524 hCoV-19/Taiwan/CGMH-CGU-11/2020 Asia / Taiwan / Taoyuan 2020-03-14 Laboratory Medicine Department of Laboratory Medicine, Lin-Kou Chang Gung Memorial Hospital, Taoyuan, Taiwan Kuo-Chien Tsao, Yu-Nong Gong, Shu-Li Yang, Yi-Chun Liu, Chung-Guei Huang, Po-Wei Huang, Mei-Jen Hsiao, Cheng-Ta Yang, Cheng-Hsun Chiu, Peng-Nien Huang, Kuo-Ming Lee, Guang-Wu Chen , Shin-Ru Shih
EPI_ISL_417525 hCoV-19/Taiwan/CGMH-CGU-12/2020 Asia / Taiwan / Taoyuan 2020-03-14 Laboratory Medicine Department of Laboratory Medicine, Lin-Kou Chang Gung Memorial Hospital, Taoyuan, Taiwan Kuo-Chien Tsao, Yu-Nong Gong, Shu-Li Yang, Yi-Chun Liu, Chung-Guei Huang, Po-Wei Huang, Mei-Jen Hsiao, Cheng-Ta Yang, Cheng-Hsun Chiu, Peng-Nien Huang, Kuo-Ming Lee, Guang-Wu Chen , Shin-Ru Shih
EPI_ISL_417526 hCoV-19/Luxembourg/LNS9371718/2020 Europe / Luxembourg 2020-03-18 Laboratoire Nationale de Santé, Microbiology, Virology Laboratoire Nationale de Santé, Microbiology, Epidemiology and Microbial Genomics Anke Wienecke-Baldacchino, Ardashel Latsuzbaia, Jessica Tapp, Catherine Ragimbeau, Guillaume Fournier, Tamir Abdelrahman, Trung Nguyen Nguyen, Joel Mossong
EPI_ISL_417527 hCoV-19/Luxembourg/LNS2848109/2020 Europe / Luxembourg 2020-03-18 Laboratoire Nationale de Santé, Microbiology, Virology Laboratoire Nationale de Santé, Microbiology, Epidemiology and Microbial Genomics Anke Wienecke-Baldacchino, Ardashel Latsuzbaia, Jessica Tapp, Catherine Ragimbeau, Guillaume Fournier, Tamir Abdelrahman, Trung Nguyen Nguyen, Joel Mossong
EPI_ISL_417528 hCoV-19/Luxembourg/LNS3694003/2020 Europe / Luxembourg 2020-03-18 Laboratoire

Nationale de Santé, Microbiology, Virology	Laboratoire Nationale de Santé, Microbiology, Epidemiology and Microbial Genomics	Anke Wienecke-Baldacchino, Ardashel Latsuzbaia, Jessica Tapp, Catherine Ragimbeau, Guillaume Fournier, Tamir Abdelrahman, Trung Nguyen Nguyen, Joel Mossong
EPI_ISL_417600	hCoV-19/Iceland/272/2020	Europe / Iceland / Reykjavik	2020-03-17	The National University Hospital of Iceland	deCODE genetics	Daniel F Gudbjartsson; Agnar Helgason; Hakon Jonsson; Olafur T Magnusson; Pall Melsted; Gudmundur L Norddahl; Jona Saemundsdottir; Asgeir Sigurdsson; Patrick Sulem; Arna B Agustsdottir; Berglind Eiriksdottir; Run Fridriksdottir; Elisabet E Gardarsdottir; Gudmundur Georgsson; Olafia S Gretarsdottir; Kjartan R Gudmundsson; Thora R Gunnarsdottir; Arnaldur Gylfason; Hilma Holm; Brynjar O Jensson; Aslaug Jonasdottir; Kamilla S Josefsdottir; Thordur Kristjansson; Droplaug N Magnusdottir; Louise le Roux; Gudrun Sigmundsdottir; Gardar Sveinbjornsson; Kristin E Sveinsdottir; Maney Sveinsdottir; Emil A Thorarensen; Bjarni Thorbjornsson; Gisli Masson; Ingileif Jonsdottir; Alma Moller; Thorolfur Gudnason; Karl G Kristinsson; Unnur Thorsteinsdottir; Kari Stefansson
EPI_ISL_417601	hCoV-19/Iceland/273/2020	Europe / Iceland / Reykjavik	2020-03-17	The National University Hospital of Iceland	deCODE genetics	Daniel F Gudbjartsson; Agnar Helgason; Hakon Jonsson; Olafur T Magnusson; Pall Melsted; Gudmundur L Norddahl; Jona Saemundsdottir; Asgeir Sigurdsson; Patrick Sulem; Arna B Agustsdottir; Berglind Eiriksdottir; Run Fridriksdottir; Elisabet E Gardarsdottir; Gudmundur Georgsson; Olafia S Gretarsdottir; Kjartan R Gudmundsson; Thora R Gunnarsdottir; Arnaldur Gylfason; Hilma Holm; Brynjar O Jensson; Aslaug Jonasdottir; Kamilla S Josefsdottir; Thordur Kristjansson; Droplaug N Magnusdottir; Louise le Roux; Gudrun Sigmundsdottir; Gardar Sveinbjornsson; Kristin E Sveinsdottir; Maney Sveinsdottir; Emil A Thorarensen; Bjarni Thorbjornsson; Gisli Masson; Ingileif Jonsdottir; Alma Moller; Thorolfur Gudnason; Karl G Kristinsson; Unnur Thorsteinsdottir; Kari Stefansson
EPI_ISL_417602	hCoV-19/Iceland/274/2020	Europe / Iceland / Reykjavik	2020-03-17	The National University Hospital of Iceland	deCODE genetics	Daniel F Gudbjartsson; Agnar Helgason; Hakon Jonsson; Olafur T Magnusson; Pall Melsted; Gudmundur L Norddahl; Jona Saemundsdottir; Asgeir Sigurdsson; Patrick Sulem; Arna B Agustsdottir; Berglind Eiriksdottir; Run Fridriksdottir; Elisabet E Gardarsdottir; Gudmundur Georgsson; Olafia S Gretarsdottir; Kjartan R Gudmundsson; Thora R Gunnarsdottir; Arnaldur Gylfason; Hilma Holm; Brynjar O Jensson; Aslaug Jonasdottir; Kamilla S Josefsdottir; Thordur Kristjansson; Droplaug N Magnusdottir; Louise le Roux; Gudrun Sigmundsdottir; Gardar Sveinbjornsson; Kristin E Sveinsdottir; Maney Sveinsdottir; Emil A Thorarensen; Bjarni Thorbjornsson; Gisli Masson; Ingileif Jonsdottir; Alma Moller; Thorolfur Gudnason; Karl G Kristinsson; Unnur Thorsteinsdottir; Kari Stefansson
EPI_ISL_417603	hCoV-19/Iceland/275/2020	Europe / Iceland / Reykjavik	2020-03-17	The National University Hospital of Iceland	deCODE genetics	Daniel F Gudbjartsson; Agnar Helgason; Hakon Jonsson; Olafur T Magnusson; Pall Melsted; Gudmundur L Norddahl; Jona Saemundsdottir; Asgeir Sigurdsson; Patrick Sulem; Arna B Agustsdottir; Berglind Eiriksdottir; Run Fridriksdottir; Elisabet E Gardarsdottir; Gudmundur Georgsson; Olafia S Gretarsdottir; Kjartan R Gudmundsson; Thora R Gunnarsdottir; Arnaldur Gylfason; Hilma Holm; Brynjar O Jensson; Aslaug Jonasdottir; Kamilla S Josefsdottir; Thordur Kristjansson; Droplaug N Magnusdottir; Louise le Roux; Gudrun Sigmundsdottir; Gardar Sveinbjornsson; Kristin E Sveinsdottir; Maney Sveinsdottir; Emil A Thorarensen; Bjarni Thorbjornsson; Gisli Masson; Ingileif Jonsdottir; Alma Moller; Thorolfur Gudnason; Karl G Kristinsson; Unnur Thorsteinsdottir; Kari Stefansson
EPI_ISL_417604	hCoV-19/Iceland/276/2020	Europe / Iceland / Reykjavik	2020-03-17	The National University Hospital of Iceland	deCODE genetics	Daniel F Gudbjartsson; Agnar Helgason; Hakon Jonsson; Olafur T Magnusson; Pall Melsted; Gudmundur L Norddahl; Jona Saemundsdottir; Asgeir Sigurdsson; Patrick Sulem; Arna B Agustsdottir; Berglind Eiriksdottir; Run Fridriksdottir; Elisabet E Gardarsdottir; Gudmundur Georgsson; Olafia S Gretarsdottir; Kjartan R Gudmundsson; Thora R Gunnarsdottir; Arnaldur Gylfason; Hilma Holm; Brynjar O Jensson; Aslaug Jonasdottir; Kamilla S Josefsdottir; Thordur Kristjansson; Droplaug N Magnusdottir; Louise le Roux; Gudrun Sigmundsdottir; Gardar Sveinbjornsson; Kristin E Sveinsdottir; Maney Sveinsdottir; Emil A Thorarensen; Bjarni Thorbjornsson; Gisli Masson; Ingileif Jonsdottir; Alma Moller; Thorolfur Gudnason; Karl G Kristinsson; Unnur Thorsteinsdottir; Kari Stefansson
EPI_ISL_417607	hCoV-19/Iceland/279/2020	Europe / Iceland / Reykjavik	2020-03-17	The National University Hospital of Iceland	deCODE genetics	Daniel F Gudbjartsson; Agnar Helgason; Hakon Jonsson; Olafur T Magnusson; Pall Melsted; Gudmundur L Norddahl; Jona Saemundsdottir; Asgeir Sigurdsson; Patrick Sulem; Arna B Agustsdottir; Berglind Eiriksdottir; Run Fridriksdottir; Elisabet E Gardarsdottir; Gudmundur Georgsson; Olafia S Gretarsdottir; Kjartan R Gudmundsson; Thora R Gunnarsdottir; Arnaldur Gylfason; Hilma Holm; Brynjar O Jensson; Aslaug Jonasdottir; Kamilla S Josefsdottir; Thordur Kristjansson; Droplaug N Magnusdottir; Louise le Roux; Gudrun Sigmundsdottir; Gardar Sveinbjornsson; Kristin E Sveinsdottir; Maney Sveinsdottir; Emil A Thorarensen; Bjarni Thorbjornsson; Gisli Masson; Ingileif Jonsdottir; Alma Moller; Thorolfur Gudnason; Karl G Kristinsson; Unnur Thorsteinsdottir; Kari Stefansson
EPI_ISL_417608	hCoV-19/Iceland/280/2020	Europe / Iceland / Reykjavik	2020-03-17	The National University Hospital of Iceland	deCODE genetics	Daniel F Gudbjartsson; Agnar Helgason; Hakon Jonsson; Olafur T Magnusson; Pall Melsted; Gudmundur L Norddahl; Jona Saemundsdottir; Asgeir Sigurdsson; Patrick Sulem; Arna B Agustsdottir; Berglind Eiriksdottir; Run Fridriksdottir; Elisabet E Gardarsdottir; Gudmundur Georgsson; Olafia S Gretarsdottir; Kjartan R Gudmundsson; Thora R Gunnarsdottir; Arnaldur Gylfason; Hilma Holm; Brynjar O Jensson; Aslaug Jonasdottir; Kamilla S Josefsdottir; Thordur Kristjansson; Droplaug N Magnusdottir; Louise le Roux; Gudrun Sigmundsdottir; Gardar Sveinbjornsson; Kristin E Sveinsdottir; Maney Sveinsdottir; Emil A Thorarensen; Bjarni Thorbjornsson; Gisli Masson; Ingileif Jonsdottir; Alma Moller; Thorolfur Gudnason; Karl G Kristinsson; Unnur Thorsteinsdottir; Kari Stefansson
EPI_ISL_417609	hCoV-19/Iceland/281/2020	Europe / Iceland / Reykjavik	2020-03-17	The National University Hospital of Iceland	deCODE genetics	Daniel F Gudbjartsson; Agnar Helgason; Hakon Jonsson; Olafur T Magnusson; Pall Melsted; Gudmundur L Norddahl; Jona Saemundsdottir; Asgeir Sigurdsson; Patrick Sulem; Arna B Agustsdottir; Berglind Eiriksdottir; Run Fridriksdottir; Elisabet E Gardarsdottir; Gudmundur Georgsson; Olafia

S Gretarsdottir; Kjartan R Gudmundsson; Thora R Gunnarsdottir; Arnaldur Gylfason; Hilma Holm; Brynjar O Jensson; Aslaug Jonasdottir; Kamilla S Josefsdottir; Thordur Kristjansson; Droplaug N Magnusdottir; Louise le Roux; Gudrun Sigmundsdottir; Gardar Sveinbjornsson; Kristin E Sveinsdottir; Maney Sveinsdottir; Emil A Thorarensen; Bjarni Thorbjornsson; Gisli Masson; Ingileif Jonsdottir; Alma Moller; Thorolfur Gudnason; Karl G Kristinsson; Unnur Thorsteinsdottir; Kari Stefansson
EPI_ISL_417610 hCoV-19/Iceland/282/2020 Europe / Iceland / Reykjavik 2020-03-17 The National University Hospital of Iceland deCODE genetics Daniel F Gudbjartsson; Agnar Helgason; Hakon Jonsson; Olafur T Magnusson; Pall Melsted; Gudmundur L Norddahl; Jona Saemundsdottir; Asgeir Sigurdsson; Patrick Sulem; Arna B Agustsdottir; Berglind Eiriksdottir; Run Fridriksdottir; Elisabet E Gardarsdottir; Gudmundur Georgsson; Olafia S Gretarsdottir; Kjartan R Gudmundsson; Thora R Gunnarsdottir; Arnaldur Gylfason; Hilma Holm; Brynjar O Jensson; Aslaug Jonasdottir; Kamilla S Josefsdottir; Thordur Kristjansson; Droplaug N Magnusdottir; Louise le Roux; Gudrun Sigmundsdottir; Gardar Sveinbjornsson; Kristin E Sveinsdottir; Maney Sveinsdottir; Emil A Thorarensen; Bjarni Thorbjornsson; Gisli Masson; Ingileif Jonsdottir; Alma Moller; Thorolfur Gudnason; Karl G Kristinsson; Unnur Thorsteinsdottir; Kari Stefansson
EPI_ISL_417611 hCoV-19/Iceland/283/2020 Europe / Iceland / Reykjavik 2020-03-17 The National University Hospital of Iceland deCODE genetics Daniel F Gudbjartsson; Agnar Helgason; Hakon Jonsson; Olafur T Magnusson; Pall Melsted; Gudmundur L Norddahl; Jona Saemundsdottir; Asgeir Sigurdsson; Patrick Sulem; Arna B Agustsdottir; Berglind Eiriksdottir; Run Fridriksdottir; Elisabet E Gardarsdottir; Gudmundur Georgsson; Olafia S Gretarsdottir; Kjartan R Gudmundsson; Thora R Gunnarsdottir; Arnaldur Gylfason; Hilma Holm; Brynjar O Jensson; Aslaug Jonasdottir; Kamilla S Josefsdottir; Thordur Kristjansson; Droplaug N Magnusdottir; Louise le Roux; Gudrun Sigmundsdottir; Gardar Sveinbjornsson; Kristin E Sveinsdottir; Maney Sveinsdottir; Emil A Thorarensen; Bjarni Thorbjornsson; Gisli Masson; Ingileif Jonsdottir; Alma Moller; Thorolfur Gudnason; Karl G Kristinsson; Unnur Thorsteinsdottir; Kari Stefansson
EPI_ISL_417612 hCoV-19/Iceland/284/2020 Europe / Iceland / Reykjavik 2020-03-17 The National University Hospital of Iceland deCODE genetics Daniel F Gudbjartsson; Agnar Helgason; Hakon Jonsson; Olafur T Magnusson; Pall Melsted; Gudmundur L Norddahl; Jona Saemundsdottir; Asgeir Sigurdsson; Patrick Sulem; Arna B Agustsdottir; Berglind Eiriksdottir; Run Fridriksdottir; Elisabet E Gardarsdottir; Gudmundur Georgsson; Olafia S Gretarsdottir; Kjartan R Gudmundsson; Thora R Gunnarsdottir; Arnaldur Gylfason; Hilma Holm; Brynjar O Jensson; Aslaug Jonasdottir; Kamilla S Josefsdottir; Thordur Kristjansson; Droplaug N Magnusdottir; Louise le Roux; Gudrun Sigmundsdottir; Gardar Sveinbjornsson; Kristin E Sveinsdottir; Maney Sveinsdottir; Emil A Thorarensen; Bjarni Thorbjornsson; Gisli Masson; Ingileif Jonsdottir; Alma Moller; Thorolfur Gudnason; Karl G Kristinsson; Unnur Thorsteinsdottir; Kari Stefansson
EPI_ISL_417613 hCoV-19/Iceland/285/2020 Europe / Iceland / Reykjavik 2020-03-17 The National University Hospital of Iceland deCODE genetics Daniel F Gudbjartsson; Agnar Helgason; Hakon Jonsson; Olafur T Magnusson; Pall Melsted; Gudmundur L Norddahl; Jona Saemundsdottir; Asgeir Sigurdsson; Patrick Sulem; Arna B Agustsdottir; Berglind Eiriksdottir; Run Fridriksdottir; Elisabet E Gardarsdottir; Gudmundur Georgsson; Olafia S Gretarsdottir; Kjartan R Gudmundsson; Thora R Gunnarsdottir; Arnaldur Gylfason; Hilma Holm; Brynjar O Jensson; Aslaug Jonasdottir; Kamilla S Josefsdottir; Thordur Kristjansson; Droplaug N Magnusdottir; Louise le Roux; Gudrun Sigmundsdottir; Gardar Sveinbjornsson; Kristin E Sveinsdottir; Maney Sveinsdottir; Emil A Thorarensen; Bjarni Thorbjornsson; Gisli Masson; Ingileif Jonsdottir; Alma Moller; Thorolfur Gudnason; Karl G Kristinsson; Unnur Thorsteinsdottir; Kari Stefansson
EPI_ISL_417614 hCoV-19/Iceland/286/2020 Europe / Iceland / Reykjavik 2020-03-18 The National University Hospital of Iceland deCODE genetics Daniel F Gudbjartsson; Agnar Helgason; Hakon Jonsson; Olafur T Magnusson; Pall Melsted; Gudmundur L Norddahl; Jona Saemundsdottir; Asgeir Sigurdsson; Patrick Sulem; Arna B Agustsdottir; Berglind Eiriksdottir; Run Fridriksdottir; Elisabet E Gardarsdottir; Gudmundur Georgsson; Olafia S Gretarsdottir; Kjartan R Gudmundsson; Thora R Gunnarsdottir; Arnaldur Gylfason; Hilma Holm; Brynjar O Jensson; Aslaug Jonasdottir; Kamilla S Josefsdottir; Thordur Kristjansson; Droplaug N Magnusdottir; Louise le Roux; Gudrun Sigmundsdottir; Gardar Sveinbjornsson; Kristin E Sveinsdottir; Maney Sveinsdottir; Emil A Thorarensen; Bjarni Thorbjornsson; Gisli Masson; Ingileif Jonsdottir; Alma Moller; Thorolfur Gudnason; Karl G Kristinsson; Unnur Thorsteinsdottir; Kari Stefansson
EPI_ISL_417615 hCoV-19/Iceland/287/2020 Europe / Iceland / Reykjavik 2020-03-18 The National University Hospital of Iceland deCODE genetics Daniel F Gudbjartsson; Agnar Helgason; Hakon Jonsson; Olafur T Magnusson; Pall Melsted; Gudmundur L Norddahl; Jona Saemundsdottir; Asgeir Sigurdsson; Patrick Sulem; Arna B Agustsdottir; Berglind Eiriksdottir; Run Fridriksdottir; Elisabet E Gardarsdottir; Gudmundur Georgsson; Olafia S Gretarsdottir; Kjartan R Gudmundsson; Thora R Gunnarsdottir; Arnaldur Gylfason; Hilma Holm; Brynjar O Jensson; Aslaug Jonasdottir; Kamilla S Josefsdottir; Thordur Kristjansson; Droplaug N Magnusdottir; Louise le Roux; Gudrun Sigmundsdottir; Gardar Sveinbjornsson; Kristin E Sveinsdottir; Maney Sveinsdottir; Emil A Thorarensen; Bjarni Thorbjornsson; Gisli Masson; Ingileif Jonsdottir; Alma Moller; Thorolfur Gudnason; Karl G Kristinsson; Unnur Thorsteinsdottir; Kari Stefansson
EPI_ISL_417616 hCoV-19/Iceland/288/2020 Europe / Iceland / Reykjavik 2020-03-18 The National University Hospital of Iceland deCODE genetics Daniel F Gudbjartsson; Agnar Helgason; Hakon Jonsson; Olafur T Magnusson; Pall Melsted; Gudmundur L Norddahl; Jona Saemundsdottir; Asgeir Sigurdsson; Patrick Sulem; Arna B Agustsdottir; Berglind Eiriksdottir; Run Fridriksdottir; Elisabet E Gardarsdottir; Gudmundur Georgsson; Olafia S Gretarsdottir; Kjartan R Gudmundsson; Thora R Gunnarsdottir; Arnaldur Gylfason; Hilma Holm; Brynjar O Jensson; Aslaug Jonasdottir; Kamilla S Josefsdottir; Thordur Kristjansson; Droplaug N Magnusdottir; Louise le Roux; Gudrun Sigmundsdottir; Gardar Sveinbjornsson; Kristin E Sveinsdottir; Maney Sveinsdottir; Emil A Thorarensen; Bjarni Thorbjornsson; Gisli Masson; Ingileif Jonsdottir; Alma Moller; Thorolfur Gudnason; Karl G Kristinsson; Unnur Thorsteinsdottir; Kari Stefansson
EPI_ISL_417618 hCoV-19/Iceland/29/2020 Europe / Iceland / Reykjavik 2020-03-16 deCODE genetics deCODE genetics Daniel F Gudbjartsson; Agnar Helgason; Hakon Jonsson; Olafur T Magnusson; Pall Melsted;

Gudmundur L Norddahl; Jona Saemundsdottir; Asgeir Sigurdsson; Patrick Sulem; Arna B Agustsdottir; Berglind Eiriksdottir; Run Fridriksdottir; Elisabet E Gardarsdottir; Gudmundur Georgsson; Olafia S Gretarsdottir; Kjartan R Gudmundsson; Thora R Gunnarsdottir; Arnaldur Gylfason; Hilma Holm; Brynjar O Jensson; Aslaug Jonasdottir; Kamilla S Josefsdottir; Thordur Kristjansson; Droplaug N Magnusdottir; Louise le Roux; Gudrun Sigmundsdottir; Gardar Sveinbjornsson; Kristin E Sveinsdottir; Maney Sveinsdottir; Emil A Thorarensen; Bjarni Thorbjornsson; Gisli Masson; Ingileif Jonsdottir; Alma Moller; Thorolfur Gudnason; Karl G Kristinsson; Unnur Thorsteinsdottir; Kari Stefansson
EPI_ISL_417619 hCoV-19/Iceland/290/2020 Europe / Iceland / Reykjavik 2020-03-18 The National University Hospital of Iceland deCODE genetics Daniel F Gudbjartsson; Agnar Helgason; Hakon Jonsson; Olafur T Magnusson; Pall Melsted; Gudmundur L Norddahl; Jona Saemundsdottir; Asgeir Sigurdsson; Patrick Sulem; Arna B Agustsdottir; Berglind Eiriksdottir; Run Fridriksdottir; Elisabet E Gardarsdottir; Gudmundur Georgsson; Olafia S Gretarsdottir; Kjartan R Gudmundsson; Thora R Gunnarsdottir; Arnaldur Gylfason; Hilma Holm; Brynjar O Jensson; Aslaug Jonasdottir; Kamilla S Josefsdottir; Thordur Kristjansson; Droplaug N Magnusdottir; Louise le Roux; Gudrun Sigmundsdottir; Gardar Sveinbjornsson; Kristin E Sveinsdottir; Maney Sveinsdottir; Emil A Thorarensen; Bjarni Thorbjornsson; Gisli Masson; Ingileif Jonsdottir; Alma Moller; Thorolfur Gudnason; Karl G Kristinsson; Unnur Thorsteinsdottir; Kari Stefansson
EPI_ISL_417620 hCoV-19/Iceland/291/2020 Europe / Iceland / Reykjavik 2020-03-18 The National University Hospital of Iceland deCODE genetics Daniel F Gudbjartsson; Agnar Helgason; Hakon Jonsson; Olafur T Magnusson; Pall Melsted; Gudmundur L Norddahl; Jona Saemundsdottir; Asgeir Sigurdsson; Patrick Sulem; Arna B Agustsdottir; Berglind Eiriksdottir; Run Fridriksdottir; Elisabet E Gardarsdottir; Gudmundur Georgsson; Olafia S Gretarsdottir; Kjartan R Gudmundsson; Thora R Gunnarsdottir; Arnaldur Gylfason; Hilma Holm; Brynjar O Jensson; Aslaug Jonasdottir; Kamilla S Josefsdottir; Thordur Kristjansson; Droplaug N Magnusdottir; Louise le Roux; Gudrun Sigmundsdottir; Gardar Sveinbjornsson; Kristin E Sveinsdottir; Maney Sveinsdottir; Emil A Thorarensen; Bjarni Thorbjornsson; Gisli Masson; Ingileif Jonsdottir; Alma Moller; Thorolfur Gudnason; Karl G Kristinsson; Unnur Thorsteinsdottir; Kari Stefansson
EPI_ISL_417621 hCoV-19/Iceland/292/2020 Europe / Iceland / Reykjavik 2020-03-18 The National University Hospital of Iceland deCODE genetics Daniel F Gudbjartsson; Agnar Helgason; Hakon Jonsson; Olafur T Magnusson; Pall Melsted; Gudmundur L Norddahl; Jona Saemundsdottir; Asgeir Sigurdsson; Patrick Sulem; Arna B Agustsdottir; Berglind Eiriksdottir; Run Fridriksdottir; Elisabet E Gardarsdottir; Gudmundur Georgsson; Olafia S Gretarsdottir; Kjartan R Gudmundsson; Thora R Gunnarsdottir; Arnaldur Gylfason; Hilma Holm; Brynjar O Jensson; Aslaug Jonasdottir; Kamilla S Josefsdottir; Thordur Kristjansson; Droplaug N Magnusdottir; Louise le Roux; Gudrun Sigmundsdottir; Gardar Sveinbjornsson; Kristin E Sveinsdottir; Maney Sveinsdottir; Emil A Thorarensen; Bjarni Thorbjornsson; Gisli Masson; Ingileif Jonsdottir; Alma Moller; Thorolfur Gudnason; Karl G Kristinsson; Unnur Thorsteinsdottir; Kari Stefansson
EPI_ISL_417622 hCoV-19/Iceland/293/2020 Europe / Iceland / Reykjavik 2020-03-18 The National University Hospital of Iceland deCODE genetics Daniel F Gudbjartsson; Agnar Helgason; Hakon Jonsson; Olafur T Magnusson; Pall Melsted; Gudmundur L Norddahl; Jona Saemundsdottir; Asgeir Sigurdsson; Patrick Sulem; Arna B Agustsdottir; Berglind Eiriksdottir; Run Fridriksdottir; Elisabet E Gardarsdottir; Gudmundur Georgsson; Olafia S Gretarsdottir; Kjartan R Gudmundsson; Thora R Gunnarsdottir; Arnaldur Gylfason; Hilma Holm; Brynjar O Jensson; Aslaug Jonasdottir; Kamilla S Josefsdottir; Thordur Kristjansson; Droplaug N Magnusdottir; Louise le Roux; Gudrun Sigmundsdottir; Gardar Sveinbjornsson; Kristin E Sveinsdottir; Maney Sveinsdottir; Emil A Thorarensen; Bjarni Thorbjornsson; Gisli Masson; Ingileif Jonsdottir; Alma Moller; Thorolfur Gudnason; Karl G Kristinsson; Unnur Thorsteinsdottir; Kari Stefansson
EPI_ISL_417623 hCoV-19/Iceland/294/2020 Europe / Iceland / Reykjavik 2020-03-18 The National University Hospital of Iceland deCODE genetics Daniel F Gudbjartsson; Agnar Helgason; Hakon Jonsson; Olafur T Magnusson; Pall Melsted; Gudmundur L Norddahl; Jona Saemundsdottir; Asgeir Sigurdsson; Patrick Sulem; Arna B Agustsdottir; Berglind Eiriksdottir; Run Fridriksdottir; Elisabet E Gardarsdottir; Gudmundur Georgsson; Olafia S Gretarsdottir; Kjartan R Gudmundsson; Thora R Gunnarsdottir; Arnaldur Gylfason; Hilma Holm; Brynjar O Jensson; Aslaug Jonasdottir; Kamilla S Josefsdottir; Thordur Kristjansson; Droplaug N Magnusdottir; Louise le Roux; Gudrun Sigmundsdottir; Gardar Sveinbjornsson; Kristin E Sveinsdottir; Maney Sveinsdottir; Emil A Thorarensen; Bjarni Thorbjornsson; Gisli Masson; Ingileif Jonsdottir; Alma Moller; Thorolfur Gudnason; Karl G Kristinsson; Unnur Thorsteinsdottir; Kari Stefansson
EPI_ISL_417625 hCoV-19/Iceland/296/2020 Europe / Iceland / Reykjavik 2020-03-18 The National University Hospital of Iceland deCODE genetics Daniel F Gudbjartsson; Agnar Helgason; Hakon Jonsson; Olafur T Magnusson; Pall Melsted; Gudmundur L Norddahl; Jona Saemundsdottir; Asgeir Sigurdsson; Patrick Sulem; Arna B Agustsdottir; Berglind Eiriksdottir; Run Fridriksdottir; Elisabet E Gardarsdottir; Gudmundur Georgsson; Olafia S Gretarsdottir; Kjartan R Gudmundsson; Thora R Gunnarsdottir; Arnaldur Gylfason; Hilma Holm; Brynjar O Jensson; Aslaug Jonasdottir; Kamilla S Josefsdottir; Thordur Kristjansson; Droplaug N Magnusdottir; Louise le Roux; Gudrun Sigmundsdottir; Gardar Sveinbjornsson; Kristin E Sveinsdottir; Maney Sveinsdottir; Emil A Thorarensen; Bjarni Thorbjornsson; Gisli Masson; Ingileif Jonsdottir; Alma Moller; Thorolfur Gudnason; Karl G Kristinsson; Unnur Thorsteinsdottir; Kari Stefansson
EPI_ISL_417626 hCoV-19/Iceland/297/2020 Europe / Iceland / Reykjavik 2020-03-18 The National University Hospital of Iceland deCODE genetics Daniel F Gudbjartsson; Agnar Helgason; Hakon Jonsson; Olafur T Magnusson; Pall Melsted; Gudmundur L Norddahl; Jona Saemundsdottir; Asgeir Sigurdsson; Patrick Sulem; Arna B Agustsdottir; Berglind Eiriksdottir; Run Fridriksdottir; Elisabet E Gardarsdottir; Gudmundur Georgsson; Olafia S Gretarsdottir; Kjartan R Gudmundsson; Thora R Gunnarsdottir; Arnaldur Gylfason; Hilma Holm; Brynjar O Jensson; Aslaug Jonasdottir; Kamilla S Josefsdottir; Thordur Kristjansson; Droplaug N Magnusdottir; Louise le Roux; Gudrun Sigmundsdottir; Gardar Sveinbjornsson; Kristin E Sveinsdottir; Maney Sveinsdottir; Emil A Thorarensen; Bjarni Thorbjornsson; Gisli Masson; Ingileif Jonsdottir; Alma Moller; Thorolfur Gudnason; Karl G Kristinsson; Unnur Thorsteinsdottir; Kari Stefansson

EPI_ISL_417627 hCoV-19/Iceland/298/2020 Europe / Iceland / Reykjavik 2020-03-18 The National University Hospital of Iceland deCODE genetics Daniel F Gudbjartsson; Agnar Helgason; Hakon Jonsson; Olafur T Magnusson; Pall Melsted; Gudmundur L Norddahl; Jona Saemundsdottir; Asgeir Sigurdsson; Patrick Sulem; Arna B Agustsdottir; Berglind Eiriksdottir; Run Fridriksdottir; Elisabet E Gardarsdottir; Gudmundur Georgsson; Olafia S Gretarsdottir; Kjartan R Gudmundsson; Thora R Gunnarsdottir; Arnaldur Gylfason; Hilma Holm; Brynjar O Jensson; Aslaug Jonasdottir; Kamilla S Josefsdottir; Thordur Kristjansson; Droplaug N Magnusdottir; Louise le Roux; Gudrun Sigmundsdottir; Gardar Sveinbjornsson; Kristin E Sveinsdottir; Maney Sveinsdottir; Emil A Thorarensen; Bjarni Thorbjornsson; Gisli Masson; Ingileif Jonsdottir; Alma Moller; Thorolfur Gudnason; Karl G Kristinsson; Unnur Thorsteinsdottir; Kari Stefansson
EPI_ISL_417628 hCoV-19/Iceland/299/2020 Europe / Iceland / Reykjavik 2020-03-18 The National University Hospital of Iceland deCODE genetics Daniel F Gudbjartsson; Agnar Helgason; Hakon Jonsson; Olafur T Magnusson; Pall Melsted; Gudmundur L Norddahl; Jona Saemundsdottir; Asgeir Sigurdsson; Patrick Sulem; Arna B Agustsdottir; Berglind Eiriksdottir; Run Fridriksdottir; Elisabet E Gardarsdottir; Gudmundur Georgsson; Olafia S Gretarsdottir; Kjartan R Gudmundsson; Thora R Gunnarsdottir; Arnaldur Gylfason; Hilma Holm; Brynjar O Jensson; Aslaug Jonasdottir; Kamilla S Josefsdottir; Thordur Kristjansson; Droplaug N Magnusdottir; Louise le Roux; Gudrun Sigmundsdottir; Gardar Sveinbjornsson; Kristin E Sveinsdottir; Maney Sveinsdottir; Emil A Thorarensen; Bjarni Thorbjornsson; Gisli Masson; Ingileif Jonsdottir; Alma Moller; Thorolfur Gudnason; Karl G Kristinsson; Unnur Thorsteinsdottir; Kari Stefansson
EPI_ISL_417629 hCoV-19/Iceland/300/2020 Europe / Iceland / Reykjavik 2020-03-18 The National University Hospital of Iceland deCODE genetics Daniel F Gudbjartsson; Agnar Helgason; Hakon Jonsson; Olafur T Magnusson; Pall Melsted; Gudmundur L Norddahl; Jona Saemundsdottir; Asgeir Sigurdsson; Patrick Sulem; Arna B Agustsdottir; Berglind Eiriksdottir; Run Fridriksdottir; Elisabet E Gardarsdottir; Gudmundur Georgsson; Olafia S Gretarsdottir; Kjartan R Gudmundsson; Thora R Gunnarsdottir; Arnaldur Gylfason; Hilma Holm; Brynjar O Jensson; Aslaug Jonasdottir; Kamilla S Josefsdottir; Thordur Kristjansson; Droplaug N Magnusdottir; Louise le Roux; Gudrun Sigmundsdottir; Gardar Sveinbjornsson; Kristin E Sveinsdottir; Maney Sveinsdottir; Emil A Thorarensen; Bjarni Thorbjornsson; Gisli Masson; Ingileif Jonsdottir; Alma Moller; Thorolfur Gudnason; Karl G Kristinsson; Unnur Thorsteinsdottir; Kari Stefansson
EPI_ISL_417630 hCoV-19/Iceland/301/2020 Europe / Iceland / Reykjavik 2020-03-18 The National University Hospital of Iceland deCODE genetics Daniel F Gudbjartsson; Agnar Helgason; Hakon Jonsson; Olafur T Magnusson; Pall Melsted; Gudmundur L Norddahl; Jona Saemundsdottir; Asgeir Sigurdsson; Patrick Sulem; Arna B Agustsdottir; Berglind Eiriksdottir; Run Fridriksdottir; Elisabet E Gardarsdottir; Gudmundur Georgsson; Olafia S Gretarsdottir; Kjartan R Gudmundsson; Thora R Gunnarsdottir; Arnaldur Gylfason; Hilma Holm; Brynjar O Jensson; Aslaug Jonasdottir; Kamilla S Josefsdottir; Thordur Kristjansson; Droplaug N Magnusdottir; Louise le Roux; Gudrun Sigmundsdottir; Gardar Sveinbjornsson; Kristin E Sveinsdottir; Maney Sveinsdottir; Emil A Thorarensen; Bjarni Thorbjornsson; Gisli Masson; Ingileif Jonsdottir; Alma Moller; Thorolfur Gudnason; Karl G Kristinsson; Unnur Thorsteinsdottir; Kari Stefansson
EPI_ISL_417632 hCoV-19/Iceland/303/2020 Europe / Iceland / Reykjavik 2020-03-18 The National University Hospital of Iceland deCODE genetics Daniel F Gudbjartsson; Agnar Helgason; Hakon Jonsson; Olafur T Magnusson; Pall Melsted; Gudmundur L Norddahl; Jona Saemundsdottir; Asgeir Sigurdsson; Patrick Sulem; Arna B Agustsdottir; Berglind Eiriksdottir; Run Fridriksdottir; Elisabet E Gardarsdottir; Gudmundur Georgsson; Olafia S Gretarsdottir; Kjartan R Gudmundsson; Thora R Gunnarsdottir; Arnaldur Gylfason; Hilma Holm; Brynjar O Jensson; Aslaug Jonasdottir; Kamilla S Josefsdottir; Thordur Kristjansson; Droplaug N Magnusdottir; Louise le Roux; Gudrun Sigmundsdottir; Gardar Sveinbjornsson; Kristin E Sveinsdottir; Maney Sveinsdottir; Emil A Thorarensen; Bjarni Thorbjornsson; Gisli Masson; Ingileif Jonsdottir; Alma Moller; Thorolfur Gudnason; Karl G Kristinsson; Unnur Thorsteinsdottir; Kari Stefansson
EPI_ISL_417633 hCoV-19/Iceland/304/2020 Europe / Iceland / Reykjavik 2020-03-17 The National University Hospital of Iceland deCODE genetics Daniel F Gudbjartsson; Agnar Helgason; Hakon Jonsson; Olafur T Magnusson; Pall Melsted; Gudmundur L Norddahl; Jona Saemundsdottir; Asgeir Sigurdsson; Patrick Sulem; Arna B Agustsdottir; Berglind Eiriksdottir; Run Fridriksdottir; Elisabet E Gardarsdottir; Gudmundur Georgsson; Olafia S Gretarsdottir; Kjartan R Gudmundsson; Thora R Gunnarsdottir; Arnaldur Gylfason; Hilma Holm; Brynjar O Jensson; Aslaug Jonasdottir; Kamilla S Josefsdottir; Thordur Kristjansson; Droplaug N Magnusdottir; Louise le Roux; Gudrun Sigmundsdottir; Gardar Sveinbjornsson; Kristin E Sveinsdottir; Maney Sveinsdottir; Emil A Thorarensen; Bjarni Thorbjornsson; Gisli Masson; Ingileif Jonsdottir; Alma Moller; Thorolfur Gudnason; Karl G Kristinsson; Unnur Thorsteinsdottir; Kari Stefansson
EPI_ISL_417634 hCoV-19/Iceland/305/2020 Europe / Iceland / Reykjavik 2020-03-18 The National University Hospital of Iceland deCODE genetics Daniel F Gudbjartsson; Agnar Helgason; Hakon Jonsson; Olafur T Magnusson; Pall Melsted; Gudmundur L Norddahl; Jona Saemundsdottir; Asgeir Sigurdsson; Patrick Sulem; Arna B Agustsdottir; Berglind Eiriksdottir; Run Fridriksdottir; Elisabet E Gardarsdottir; Gudmundur Georgsson; Olafia S Gretarsdottir; Kjartan R Gudmundsson; Thora R Gunnarsdottir; Arnaldur Gylfason; Hilma Holm; Brynjar O Jensson; Aslaug Jonasdottir; Kamilla S Josefsdottir; Thordur Kristjansson; Droplaug N Magnusdottir; Louise le Roux; Gudrun Sigmundsdottir; Gardar Sveinbjornsson; Kristin E Sveinsdottir; Maney Sveinsdottir; Emil A Thorarensen; Bjarni Thorbjornsson; Gisli Masson; Ingileif Jonsdottir; Alma Moller; Thorolfur Gudnason; Karl G Kristinsson; Unnur Thorsteinsdottir; Kari Stefansson
EPI_ISL_417635 hCoV-19/Iceland/306/2020 Europe / Iceland / Reykjavik 2020-03-18 The National University Hospital of Iceland deCODE genetics Daniel F Gudbjartsson; Agnar Helgason; Hakon Jonsson; Olafur T Magnusson; Pall Melsted; Gudmundur L Norddahl; Jona Saemundsdottir; Asgeir Sigurdsson; Patrick Sulem; Arna B Agustsdottir; Berglind Eiriksdottir; Run Fridriksdottir; Elisabet E Gardarsdottir; Gudmundur Georgsson; Olafia S Gretarsdottir; Kjartan R Gudmundsson; Thora R Gunnarsdottir; Arnaldur Gylfason; Hilma Holm; Brynjar O Jensson; Aslaug Jonasdottir; Kamilla S Josefsdottir; Thordur Kristjansson; Droplaug N Magnusdottir; Louise le Roux; Gudrun Sigmundsdottir; Gardar Sveinbjornsson; Kristin E Sveinsdottir; Maney Sveinsdottir; Emil A

Thorarensen; Bjarni Thorbjornsson; Gisli Masson; Ingileif Jonsdottir; Alma Moller; Thorolfur Gudnason; Karl G Kristinsson; Unnur Thorsteinsdottir; Kari Stefansson
EPI_ISL_417636 hCoV-19/Iceland/307/2020 Europe / Iceland / Reykjavik 2020-03-18 The National University Hospital of Iceland deCODE genetics Daniel F Gudbjartsson; Agnar Helgason; Hakon Jonsson; Olafur T Magnusson; Pall Melsted; Gudmundur L Norddahl; Jona Saemundsdottir; Asgeir Sigurdsson; Patrick Sulem; Arna B Agustsdottir; Berglind Eiriksdottir; Run Fridriksdottir; Elisabet E Gardarsdottir; Gudmundur Georgsson; Olafia S Gretarsdottir; Kjartan R Gudmundsson; Thora R Gunnarsdottir; Arnaldur Gylfason; Hilma Holm; Brynjar O Jensson; Aslaug Jonasdottir; Kamilla S Josefsdottir; Thordur Kristjansson; Droplaug N Magnusdottir; Louise le Roux; Gudrun Sigmundsdottir; Gardar Sveinbjornsson; Kristin E Sveinsdottir; Maney Sveinsdottir; Emil A Thorarensen; Bjarni Thorbjornsson; Gisli Masson; Ingileif Jonsdottir; Alma Moller; Thorolfur Gudnason; Karl G Kristinsson; Unnur Thorsteinsdottir; Kari Stefansson
EPI_ISL_417637 hCoV-19/Iceland/308/2020 Europe / Iceland / Reykjavik 2020-03-18 The National University Hospital of Iceland deCODE genetics Daniel F Gudbjartsson; Agnar Helgason; Hakon Jonsson; Olafur T Magnusson; Pall Melsted; Gudmundur L Norddahl; Jona Saemundsdottir; Asgeir Sigurdsson; Patrick Sulem; Arna B Agustsdottir; Berglind Eiriksdottir; Run Fridriksdottir; Elisabet E Gardarsdottir; Gudmundur Georgsson; Olafia S Gretarsdottir; Kjartan R Gudmundsson; Thora R Gunnarsdottir; Arnaldur Gylfason; Hilma Holm; Brynjar O Jensson; Aslaug Jonasdottir; Kamilla S Josefsdottir; Thordur Kristjansson; Droplaug N Magnusdottir; Louise le Roux; Gudrun Sigmundsdottir; Gardar Sveinbjornsson; Kristin E Sveinsdottir; Maney Sveinsdottir; Emil A Thorarensen; Bjarni Thorbjornsson; Gisli Masson; Ingileif Jonsdottir; Alma Moller; Thorolfur Gudnason; Karl G Kristinsson; Unnur Thorsteinsdottir; Kari Stefansson
EPI_ISL_417639 hCoV-19/Iceland/310/2020 Europe / Iceland / Reykjavik 2020-03-18 The National University Hospital of Iceland deCODE genetics Daniel F Gudbjartsson; Agnar Helgason; Hakon Jonsson; Olafur T Magnusson; Pall Melsted; Gudmundur L Norddahl; Jona Saemundsdottir; Asgeir Sigurdsson; Patrick Sulem; Arna B Agustsdottir; Berglind Eiriksdottir; Run Fridriksdottir; Elisabet E Gardarsdottir; Gudmundur Georgsson; Olafia S Gretarsdottir; Kjartan R Gudmundsson; Thora R Gunnarsdottir; Arnaldur Gylfason; Hilma Holm; Brynjar O Jensson; Aslaug Jonasdottir; Kamilla S Josefsdottir; Thordur Kristjansson; Droplaug N Magnusdottir; Louise le Roux; Gudrun Sigmundsdottir; Gardar Sveinbjornsson; Kristin E Sveinsdottir; Maney Sveinsdottir; Emil A Thorarensen; Bjarni Thorbjornsson; Gisli Masson; Ingileif Jonsdottir; Alma Moller; Thorolfur Gudnason; Karl G Kristinsson; Unnur Thorsteinsdottir; Kari Stefansson
EPI_ISL_417641 hCoV-19/Iceland/312/2020 Europe / Iceland / Reykjavik 2020-03-18 The National University Hospital of Iceland deCODE genetics Daniel F Gudbjartsson; Agnar Helgason; Hakon Jonsson; Olafur T Magnusson; Pall Melsted; Gudmundur L Norddahl; Jona Saemundsdottir; Asgeir Sigurdsson; Patrick Sulem; Arna B Agustsdottir; Berglind Eiriksdottir; Run Fridriksdottir; Elisabet E Gardarsdottir; Gudmundur Georgsson; Olafia S Gretarsdottir; Kjartan R Gudmundsson; Thora R Gunnarsdottir; Arnaldur Gylfason; Hilma Holm; Brynjar O Jensson; Aslaug Jonasdottir; Kamilla S Josefsdottir; Thordur Kristjansson; Droplaug N Magnusdottir; Louise le Roux; Gudrun Sigmundsdottir; Gardar Sveinbjornsson; Kristin E Sveinsdottir; Maney Sveinsdottir; Emil A Thorarensen; Bjarni Thorbjornsson; Gisli Masson; Ingileif Jonsdottir; Alma Moller; Thorolfur Gudnason; Karl G Kristinsson; Unnur Thorsteinsdottir; Kari Stefansson
EPI_ISL_417642 hCoV-19/Iceland/313/2020 Europe / Iceland / Reykjavik 2020-03-18 The National University Hospital of Iceland deCODE genetics Daniel F Gudbjartsson; Agnar Helgason; Hakon Jonsson; Olafur T Magnusson; Pall Melsted; Gudmundur L Norddahl; Jona Saemundsdottir; Asgeir Sigurdsson; Patrick Sulem; Arna B Agustsdottir; Berglind Eiriksdottir; Run Fridriksdottir; Elisabet E Gardarsdottir; Gudmundur Georgsson; Olafia S Gretarsdottir; Kjartan R Gudmundsson; Thora R Gunnarsdottir; Arnaldur Gylfason; Hilma Holm; Brynjar O Jensson; Aslaug Jonasdottir; Kamilla S Josefsdottir; Thordur Kristjansson; Droplaug N Magnusdottir; Louise le Roux; Gudrun Sigmundsdottir; Gardar Sveinbjornsson; Kristin E Sveinsdottir; Maney Sveinsdottir; Emil A Thorarensen; Bjarni Thorbjornsson; Gisli Masson; Ingileif Jonsdottir; Alma Moller; Thorolfur Gudnason; Karl G Kristinsson; Unnur Thorsteinsdottir; Kari Stefansson
EPI_ISL_417643 hCoV-19/Iceland/314/2020 Europe / Iceland / Reykjavik 2020-03-18 The National University Hospital of Iceland deCODE genetics Daniel F Gudbjartsson; Agnar Helgason; Hakon Jonsson; Olafur T Magnusson; Pall Melsted; Gudmundur L Norddahl; Jona Saemundsdottir; Asgeir Sigurdsson; Patrick Sulem; Arna B Agustsdottir; Berglind Eiriksdottir; Run Fridriksdottir; Elisabet E Gardarsdottir; Gudmundur Georgsson; Olafia S Gretarsdottir; Kjartan R Gudmundsson; Thora R Gunnarsdottir; Arnaldur Gylfason; Hilma Holm; Brynjar O Jensson; Aslaug Jonasdottir; Kamilla S Josefsdottir; Thordur Kristjansson; Droplaug N Magnusdottir; Louise le Roux; Gudrun Sigmundsdottir; Gardar Sveinbjornsson; Kristin E Sveinsdottir; Maney Sveinsdottir; Emil A Thorarensen; Bjarni Thorbjornsson; Gisli Masson; Ingileif Jonsdottir; Alma Moller; Thorolfur Gudnason; Karl G Kristinsson; Unnur Thorsteinsdottir; Kari Stefansson
EPI_ISL_417644 hCoV-19/Iceland/315/2020 Europe / Iceland / Reykjavik 2020-03-18 The National University Hospital of Iceland deCODE genetics Daniel F Gudbjartsson; Agnar Helgason; Hakon Jonsson; Olafur T Magnusson; Pall Melsted; Gudmundur L Norddahl; Jona Saemundsdottir; Asgeir Sigurdsson; Patrick Sulem; Arna B Agustsdottir; Berglind Eiriksdottir; Run Fridriksdottir; Elisabet E Gardarsdottir; Gudmundur Georgsson; Olafia S Gretarsdottir; Kjartan R Gudmundsson; Thora R Gunnarsdottir; Arnaldur Gylfason; Hilma Holm; Brynjar O Jensson; Aslaug Jonasdottir; Kamilla S Josefsdottir; Thordur Kristjansson; Droplaug N Magnusdottir; Louise le Roux; Gudrun Sigmundsdottir; Gardar Sveinbjornsson; Kristin E Sveinsdottir; Maney Sveinsdottir; Emil A Thorarensen; Bjarni Thorbjornsson; Gisli Masson; Ingileif Jonsdottir; Alma Moller; Thorolfur Gudnason; Karl G Kristinsson; Unnur Thorsteinsdottir; Kari Stefansson
EPI_ISL_417645 hCoV-19/Iceland/316/2020 Europe / Iceland / Reykjavik 2020-03-18 The National University Hospital of Iceland deCODE genetics Daniel F Gudbjartsson; Agnar Helgason; Hakon Jonsson; Olafur T Magnusson; Pall Melsted; Gudmundur L Norddahl; Jona Saemundsdottir; Asgeir Sigurdsson; Patrick Sulem; Arna B Agustsdottir; Berglind Eiriksdottir; Run Fridriksdottir; Elisabet E Gardarsdottir; Gudmundur Georgsson; Olafia S Gretarsdottir; Kjartan R Gudmundsson; Thora R Gunnarsdottir; Arnaldur Gylfason; Hilma Holm; Brynjar O

Jensson; Aslaug Jonasdottir; Kamilla S Josefsdottir; Thordur Kristjansson; Droplaug N Magnusdottir; Louise le Roux; Gudrun Sigmundsdottir; Gardar Sveinbjornsson; Kristin E Sveinsdottir; Maney Sveinsdottir; Emil A Thorarensen; Bjarni Thorbjornsson; Gisli Masson; Ingileif Jonsdottir; Alma Moller; Thorolfur Gudnason; Karl G Kristinsson; Unnur Thorsteinsdottir; Kari Stefansson
EPI_ISL_417646 hCoV-19/Iceland/317/2020 Europe / Iceland / Reykjavik 2020-03-18 The National University Hospital of Iceland deCODE genetics Daniel F Gudbjartsson; Agnar Helgason; Hakon Jonsson; Olafur T Magnusson; Pall Melsted; Gudmundur L Norddahl; Jona Saemundsdottir; Asgeir Sigurdsson; Patrick Sulem; Arna B Agustsdottir; Berglind Eiriksdottir; Run Fridriksdottir; Elisabet E Gardarsdottir; Gudmundur Georgsson; Olafia S Gretarsdottir; Kjartan R Gudmundsson; Thora R Gunnarsdottir; Arnaldur Gylfason; Hilma Holm; Brynjar O Jensson; Aslaug Jonasdottir; Kamilla S Josefsdottir; Thordur Kristjansson; Droplaug N Magnusdottir; Louise le Roux; Gudrun Sigmundsdottir; Gardar Sveinbjornsson; Kristin E Sveinsdottir; Maney Sveinsdottir; Emil A Thorarensen; Bjarni Thorbjornsson; Gisli Masson; Ingileif Jonsdottir; Alma Moller; Thorolfur Gudnason; Karl G Kristinsson; Unnur Thorsteinsdottir; Kari Stefansson
EPI_ISL_417647 hCoV-19/Iceland/318/2020 Europe / Iceland / Reykjavik 2020-03-18 The National University Hospital of Iceland deCODE genetics Daniel F Gudbjartsson; Agnar Helgason; Hakon Jonsson; Olafur T Magnusson; Pall Melsted; Gudmundur L Norddahl; Jona Saemundsdottir; Asgeir Sigurdsson; Patrick Sulem; Arna B Agustsdottir; Berglind Eiriksdottir; Run Fridriksdottir; Elisabet E Gardarsdottir; Gudmundur Georgsson; Olafia S Gretarsdottir; Kjartan R Gudmundsson; Thora R Gunnarsdottir; Arnaldur Gylfason; Hilma Holm; Brynjar O Jensson; Aslaug Jonasdottir; Kamilla S Josefsdottir; Thordur Kristjansson; Droplaug N Magnusdottir; Louise le Roux; Gudrun Sigmundsdottir; Gardar Sveinbjornsson; Kristin E Sveinsdottir; Maney Sveinsdottir; Emil A Thorarensen; Bjarni Thorbjornsson; Gisli Masson; Ingileif Jonsdottir; Alma Moller; Thorolfur Gudnason; Karl G Kristinsson; Unnur Thorsteinsdottir; Kari Stefansson
EPI_ISL_417649 hCoV-19/Iceland/320/2020 Europe / Iceland / Reykjavik 2020-03-18 The National University Hospital of Iceland deCODE genetics Daniel F Gudbjartsson; Agnar Helgason; Hakon Jonsson; Olafur T Magnusson; Pall Melsted; Gudmundur L Norddahl; Jona Saemundsdottir; Asgeir Sigurdsson; Patrick Sulem; Arna B Agustsdottir; Berglind Eiriksdottir; Run Fridriksdottir; Elisabet E Gardarsdottir; Gudmundur Georgsson; Olafia S Gretarsdottir; Kjartan R Gudmundsson; Thora R Gunnarsdottir; Arnaldur Gylfason; Hilma Holm; Brynjar O Jensson; Aslaug Jonasdottir; Kamilla S Josefsdottir; Thordur Kristjansson; Droplaug N Magnusdottir; Louise le Roux; Gudrun Sigmundsdottir; Gardar Sveinbjornsson; Kristin E Sveinsdottir; Maney Sveinsdottir; Emil A Thorarensen; Bjarni Thorbjornsson; Gisli Masson; Ingileif Jonsdottir; Alma Moller; Thorolfur Gudnason; Karl G Kristinsson; Unnur Thorsteinsdottir; Kari Stefansson
EPI_ISL_417650 hCoV-19/Iceland/321/2020 Europe / Iceland / Reykjavik 2020-03-18 The National University Hospital of Iceland deCODE genetics Daniel F Gudbjartsson; Agnar Helgason; Hakon Jonsson; Olafur T Magnusson; Pall Melsted; Gudmundur L Norddahl; Jona Saemundsdottir; Asgeir Sigurdsson; Patrick Sulem; Arna B Agustsdottir; Berglind Eiriksdottir; Run Fridriksdottir; Elisabet E Gardarsdottir; Gudmundur Georgsson; Olafia S Gretarsdottir; Kjartan R Gudmundsson; Thora R Gunnarsdottir; Arnaldur Gylfason; Hilma Holm; Brynjar O Jensson; Aslaug Jonasdottir; Kamilla S Josefsdottir; Thordur Kristjansson; Droplaug N Magnusdottir; Louise le Roux; Gudrun Sigmundsdottir; Gardar Sveinbjornsson; Kristin E Sveinsdottir; Maney Sveinsdottir; Emil A Thorarensen; Bjarni Thorbjornsson; Gisli Masson; Ingileif Jonsdottir; Alma Moller; Thorolfur Gudnason; Karl G Kristinsson; Unnur Thorsteinsdottir; Kari Stefansson
EPI_ISL_417651 hCoV-19/Iceland/322/2020 Europe / Iceland / Reykjavik 2020-03-18 The National University Hospital of Iceland deCODE genetics Daniel F Gudbjartsson; Agnar Helgason; Hakon Jonsson; Olafur T Magnusson; Pall Melsted; Gudmundur L Norddahl; Jona Saemundsdottir; Asgeir Sigurdsson; Patrick Sulem; Arna B Agustsdottir; Berglind Eiriksdottir; Run Fridriksdottir; Elisabet E Gardarsdottir; Gudmundur Georgsson; Olafia S Gretarsdottir; Kjartan R Gudmundsson; Thora R Gunnarsdottir; Arnaldur Gylfason; Hilma Holm; Brynjar O Jensson; Aslaug Jonasdottir; Kamilla S Josefsdottir; Thordur Kristjansson; Droplaug N Magnusdottir; Louise le Roux; Gudrun Sigmundsdottir; Gardar Sveinbjornsson; Kristin E Sveinsdottir; Maney Sveinsdottir; Emil A Thorarensen; Bjarni Thorbjornsson; Gisli Masson; Ingileif Jonsdottir; Alma Moller; Thorolfur Gudnason; Karl G Kristinsson; Unnur Thorsteinsdottir; Kari Stefansson
EPI_ISL_417652 hCoV-19/Iceland/323/2020 Europe / Iceland / Reykjavik 2020-03-18 The National University Hospital of Iceland deCODE genetics Daniel F Gudbjartsson; Agnar Helgason; Hakon Jonsson; Olafur T Magnusson; Pall Melsted; Gudmundur L Norddahl; Jona Saemundsdottir; Asgeir Sigurdsson; Patrick Sulem; Arna B Agustsdottir; Berglind Eiriksdottir; Run Fridriksdottir; Elisabet E Gardarsdottir; Gudmundur Georgsson; Olafia S Gretarsdottir; Kjartan R Gudmundsson; Thora R Gunnarsdottir; Arnaldur Gylfason; Hilma Holm; Brynjar O Jensson; Aslaug Jonasdottir; Kamilla S Josefsdottir; Thordur Kristjansson; Droplaug N Magnusdottir; Louise le Roux; Gudrun Sigmundsdottir; Gardar Sveinbjornsson; Kristin E Sveinsdottir; Maney Sveinsdottir; Emil A Thorarensen; Bjarni Thorbjornsson; Gisli Masson; Ingileif Jonsdottir; Alma Moller; Thorolfur Gudnason; Karl G Kristinsson; Unnur Thorsteinsdottir; Kari Stefansson
EPI_ISL_417653 hCoV-19/Iceland/324/2020 Europe / Iceland / Reykjavik 2020-03-18 The National University Hospital of Iceland deCODE genetics Daniel F Gudbjartsson; Agnar Helgason; Hakon Jonsson; Olafur T Magnusson; Pall Melsted; Gudmundur L Norddahl; Jona Saemundsdottir; Asgeir Sigurdsson; Patrick Sulem; Arna B Agustsdottir; Berglind Eiriksdottir; Run Fridriksdottir; Elisabet E Gardarsdottir; Gudmundur Georgsson; Olafia S Gretarsdottir; Kjartan R Gudmundsson; Thora R Gunnarsdottir; Arnaldur Gylfason; Hilma Holm; Brynjar O Jensson; Aslaug Jonasdottir; Kamilla S Josefsdottir; Thordur Kristjansson; Droplaug N Magnusdottir; Louise le Roux; Gudrun Sigmundsdottir; Gardar Sveinbjornsson; Kristin E Sveinsdottir; Maney Sveinsdottir; Emil A Thorarensen; Bjarni Thorbjornsson; Gisli Masson; Ingileif Jonsdottir; Alma Moller; Thorolfur Gudnason; Karl G Kristinsson; Unnur Thorsteinsdottir; Kari Stefansson
EPI_ISL_417654 hCoV-19/Iceland/325/2020 Europe / Iceland / Reykjavik 2020-03-18 The National University Hospital of Iceland deCODE genetics Daniel F Gudbjartsson; Agnar Helgason; Hakon Jonsson; Olafur T Magnusson; Pall Melsted; Gudmundur L Norddahl; Jona Saemundsdottir; Asgeir Sigurdsson; Patrick Sulem; Arna B

Agustsdottir; Berglind Eiriksdottir; Run Fridriksdottir; Elisabet E Gardarsdottir; Gudmundur Georgsson; Olafia S Gretarsdottir; Kjartan R Gudmundsson; Thora R Gunnarsdottir; Arnaldur Gylfason; Hilma Holm; Brynjar O Jensson; Aslaug Jonasdottir; Kamilla S Josefsdottir; Thordur Kristjansson; Droplaug N Magnusdottir; Louise le Roux; Gudrun Sigmundsdottir; Gardar Sveinbjornsson; Kristin E Sveinsdottir; Maney Sveinsdottir; Emil A Thorarensen; Bjarni Thorbjornsson; Gisli Masson; Ingileif Jonsdottir; Alma Moller; Thorolfur Gudnason; Karl G Kristinsson; Unnur Thorsteinsdottir; Kari Stefansson
EPI_ISL_417655 hCoV-19/Iceland/326/2020 Europe / Iceland / Reykjavik 2020-03-14 deCODE genetics deCODE genetics Daniel F Gudbjartsson; Agnar Helgason; Hakon Jonsson; Olafur T Magnusson; Pall Melsted; Gudmundur L Norddahl; Jona Saemundsdottir; Asgeir Sigurdsson; Patrick Sulem; Arna B Agustsdottir; Berglind Eiriksdottir; Run Fridriksdottir; Elisabet E Gardarsdottir; Gudmundur Georgsson; Olafia S Gretarsdottir; Kjartan R Gudmundsson; Thora R Gunnarsdottir; Arnaldur Gylfason; Hilma Holm; Brynjar O Jensson; Aslaug Jonasdottir; Kamilla S Josefsdottir; Thordur Kristjansson; Droplaug N Magnusdottir; Louise le Roux; Gudrun Sigmundsdottir; Gardar Sveinbjornsson; Kristin E Sveinsdottir; Maney Sveinsdottir; Emil A Thorarensen; Bjarni Thorbjornsson; Gisli Masson; Ingileif Jonsdottir; Alma Moller; Thorolfur Gudnason; Karl G Kristinsson; Unnur Thorsteinsdottir; Kari Stefansson
EPI_ISL_417659 hCoV-19/Iceland/330/2020 Europe / Iceland / Reykjavik 2020-03-14 deCODE genetics deCODE genetics Daniel F Gudbjartsson; Agnar Helgason; Hakon Jonsson; Olafur T Magnusson; Pall Melsted; Gudmundur L Norddahl; Jona Saemundsdottir; Asgeir Sigurdsson; Patrick Sulem; Arna B Agustsdottir; Berglind Eiriksdottir; Run Fridriksdottir; Elisabet E Gardarsdottir; Gudmundur Georgsson; Olafia S Gretarsdottir; Kjartan R Gudmundsson; Thora R Gunnarsdottir; Arnaldur Gylfason; Hilma Holm; Brynjar O Jensson; Aslaug Jonasdottir; Kamilla S Josefsdottir; Thordur Kristjansson; Droplaug N Magnusdottir; Louise le Roux; Gudrun Sigmundsdottir; Gardar Sveinbjornsson; Kristin E Sveinsdottir; Maney Sveinsdottir; Emil A Thorarensen; Bjarni Thorbjornsson; Gisli Masson; Ingileif Jonsdottir; Alma Moller; Thorolfur Gudnason; Karl G Kristinsson; Unnur Thorsteinsdottir; Kari Stefansson
EPI_ISL_417660 hCoV-19/Iceland/331/2020 Europe / Iceland / Reykjavik 2020-03-14 deCODE genetics deCODE genetics Daniel F Gudbjartsson; Agnar Helgason; Hakon Jonsson; Olafur T Magnusson; Pall Melsted; Gudmundur L Norddahl; Jona Saemundsdottir; Asgeir Sigurdsson; Patrick Sulem; Arna B Agustsdottir; Berglind Eiriksdottir; Run Fridriksdottir; Elisabet E Gardarsdottir; Gudmundur Georgsson; Olafia S Gretarsdottir; Kjartan R Gudmundsson; Thora R Gunnarsdottir; Arnaldur Gylfason; Hilma Holm; Brynjar O Jensson; Aslaug Jonasdottir; Kamilla S Josefsdottir; Thordur Kristjansson; Droplaug N Magnusdottir; Louise le Roux; Gudrun Sigmundsdottir; Gardar Sveinbjornsson; Kristin E Sveinsdottir; Maney Sveinsdottir; Emil A Thorarensen; Bjarni Thorbjornsson; Gisli Masson; Ingileif Jonsdottir; Alma Moller; Thorolfur Gudnason; Karl G Kristinsson; Unnur Thorsteinsdottir; Kari Stefansson
EPI_ISL_417662 hCoV-19/Iceland/333/2020 Europe / Iceland / Reykjavik 2020-03-14 deCODE genetics deCODE genetics Daniel F Gudbjartsson; Agnar Helgason; Hakon Jonsson; Olafur T Magnusson; Pall Melsted; Gudmundur L Norddahl; Jona Saemundsdottir; Asgeir Sigurdsson; Patrick Sulem; Arna B Agustsdottir; Berglind Eiriksdottir; Run Fridriksdottir; Elisabet E Gardarsdottir; Gudmundur Georgsson; Olafia S Gretarsdottir; Kjartan R Gudmundsson; Thora R Gunnarsdottir; Arnaldur Gylfason; Hilma Holm; Brynjar O Jensson; Aslaug Jonasdottir; Kamilla S Josefsdottir; Thordur Kristjansson; Droplaug N Magnusdottir; Louise le Roux; Gudrun Sigmundsdottir; Gardar Sveinbjornsson; Kristin E Sveinsdottir; Maney Sveinsdottir; Emil A Thorarensen; Bjarni Thorbjornsson; Gisli Masson; Ingileif Jonsdottir; Alma Moller; Thorolfur Gudnason; Karl G Kristinsson; Unnur Thorsteinsdottir; Kari Stefansson
EPI_ISL_417663 hCoV-19/Iceland/334/2020 Europe / Iceland / Reykjavik 2020-03-14 deCODE genetics deCODE genetics Daniel F Gudbjartsson; Agnar Helgason; Hakon Jonsson; Olafur T Magnusson; Pall Melsted; Gudmundur L Norddahl; Jona Saemundsdottir; Asgeir Sigurdsson; Patrick Sulem; Arna B Agustsdottir; Berglind Eiriksdottir; Run Fridriksdottir; Elisabet E Gardarsdottir; Gudmundur Georgsson; Olafia S Gretarsdottir; Kjartan R Gudmundsson; Thora R Gunnarsdottir; Arnaldur Gylfason; Hilma Holm; Brynjar O Jensson; Aslaug Jonasdottir; Kamilla S Josefsdottir; Thordur Kristjansson; Droplaug N Magnusdottir; Louise le Roux; Gudrun Sigmundsdottir; Gardar Sveinbjornsson; Kristin E Sveinsdottir; Maney Sveinsdottir; Emil A Thorarensen; Bjarni Thorbjornsson; Gisli Masson; Ingileif Jonsdottir; Alma Moller; Thorolfur Gudnason; Karl G Kristinsson; Unnur Thorsteinsdottir; Kari Stefansson
EPI_ISL_417664 hCoV-19/Iceland/335/2020 Europe / Iceland / Reykjavik 2020-03-13 deCODE genetics deCODE genetics Daniel F Gudbjartsson; Agnar Helgason; Hakon Jonsson; Olafur T Magnusson; Pall Melsted; Gudmundur L Norddahl; Jona Saemundsdottir; Asgeir Sigurdsson; Patrick Sulem; Arna B Agustsdottir; Berglind Eiriksdottir; Run Fridriksdottir; Elisabet E Gardarsdottir; Gudmundur Georgsson; Olafia S Gretarsdottir; Kjartan R Gudmundsson; Thora R Gunnarsdottir; Arnaldur Gylfason; Hilma Holm; Brynjar O Jensson; Aslaug Jonasdottir; Kamilla S Josefsdottir; Thordur Kristjansson; Droplaug N Magnusdottir; Louise le Roux; Gudrun Sigmundsdottir; Gardar Sveinbjornsson; Kristin E Sveinsdottir; Maney Sveinsdottir; Emil A Thorarensen; Bjarni Thorbjornsson; Gisli Masson; Ingileif Jonsdottir; Alma Moller; Thorolfur Gudnason; Karl G Kristinsson; Unnur Thorsteinsdottir; Kari Stefansson
EPI_ISL_417665 hCoV-19/Iceland/336/2020 Europe / Iceland / Reykjavik 2020-03-14 deCODE genetics deCODE genetics Daniel F Gudbjartsson; Agnar Helgason; Hakon Jonsson; Olafur T Magnusson; Pall Melsted; Gudmundur L Norddahl; Jona Saemundsdottir; Asgeir Sigurdsson; Patrick Sulem; Arna B Agustsdottir; Berglind Eiriksdottir; Run Fridriksdottir; Elisabet E Gardarsdottir; Gudmundur Georgsson; Olafia S Gretarsdottir; Kjartan R Gudmundsson; Thora R Gunnarsdottir; Arnaldur Gylfason; Hilma Holm; Brynjar O Jensson; Aslaug Jonasdottir; Kamilla S Josefsdottir; Thordur Kristjansson; Droplaug N Magnusdottir; Louise le Roux; Gudrun Sigmundsdottir; Gardar Sveinbjornsson; Kristin E Sveinsdottir; Maney Sveinsdottir; Emil A Thorarensen; Bjarni Thorbjornsson; Gisli Masson; Ingileif Jonsdottir; Alma Moller; Thorolfur Gudnason; Karl G Kristinsson; Unnur Thorsteinsdottir; Kari Stefansson
EPI_ISL_417666 hCoV-19/Iceland/337/2020 Europe / Iceland / Reykjavik 2020-03-15 deCODE genetics

deCODE genetics Daniel F Gudbjartsson; Agnar Helgason; Hakon Jonsson; Olafur T Magnusson; Pall Melsted; Gudmundur L Norddahl; Jona Saemundsdottir; Asgeir Sigurdsson; Patrick Sulem; Arna B Agustsdottir; Berglind Eiriksdottir; Run Fridriksdottir; Elisabet E Gardarsdottir; Gudmundur Georgsson; Olafia S Gretarsdottir; Kjartan R Gudmundsson; Thora R Gunnarsdottir; Arnaldur Gylfason; Hilma Holm; Brynjar O Jensson; Aslaug Jonasdottir; Kamilla S Josefsdottir; Thordur Kristjansson; Droplaug N Magnusdottir; Louise le Roux; Gudrun Sigmundsdottir; Gardar Sveinbjornsson; Kristin E Sveinsdottir; Maney Sveinsdottir; Emil A Thorarensen; Bjarni Thorbjornsson; Gisli Masson; Ingileif Jonsdottir; Alma Moller; Thorolfur Gudnason; Karl G Kristinsson; Unnur Thorsteinsdottir; Kari Stefansson
EPI_ISL_417667 hCoV-19/Iceland/338/2020 Europe / Iceland / Reykjavik 2020-03-15 deCODE genetics deCODE genetics Daniel F Gudbjartsson; Agnar Helgason; Hakon Jonsson; Olafur T Magnusson; Pall Melsted; Gudmundur L Norddahl; Jona Saemundsdottir; Asgeir Sigurdsson; Patrick Sulem; Arna B Agustsdottir; Berglind Eiriksdottir; Run Fridriksdottir; Elisabet E Gardarsdottir; Gudmundur Georgsson; Olafia S Gretarsdottir; Kjartan R Gudmundsson; Thora R Gunnarsdottir; Arnaldur Gylfason; Hilma Holm; Brynjar O Jensson; Aslaug Jonasdottir; Kamilla S Josefsdottir; Thordur Kristjansson; Droplaug N Magnusdottir; Louise le Roux; Gudrun Sigmundsdottir; Gardar Sveinbjornsson; Kristin E Sveinsdottir; Maney Sveinsdottir; Emil A Thorarensen; Bjarni Thorbjornsson; Gisli Masson; Ingileif Jonsdottir; Alma Moller; Thorolfur Gudnason; Karl G Kristinsson; Unnur Thorsteinsdottir; Kari Stefansson
EPI_ISL_417668 hCoV-19/Iceland/339/2020 Europe / Iceland / Reykjavik 2020-03-15 deCODE genetics deCODE genetics Daniel F Gudbjartsson; Agnar Helgason; Hakon Jonsson; Olafur T Magnusson; Pall Melsted; Gudmundur L Norddahl; Jona Saemundsdottir; Asgeir Sigurdsson; Patrick Sulem; Arna B Agustsdottir; Berglind Eiriksdottir; Run Fridriksdottir; Elisabet E Gardarsdottir; Gudmundur Georgsson; Olafia S Gretarsdottir; Kjartan R Gudmundsson; Thora R Gunnarsdottir; Arnaldur Gylfason; Hilma Holm; Brynjar O Jensson; Aslaug Jonasdottir; Kamilla S Josefsdottir; Thordur Kristjansson; Droplaug N Magnusdottir; Louise le Roux; Gudrun Sigmundsdottir; Gardar Sveinbjornsson; Kristin E Sveinsdottir; Maney Sveinsdottir; Emil A Thorarensen; Bjarni Thorbjornsson; Gisli Masson; Ingileif Jonsdottir; Alma Moller; Thorolfur Gudnason; Karl G Kristinsson; Unnur Thorsteinsdottir; Kari Stefansson
EPI_ISL_417670 hCoV-19/Iceland/341/2020 Europe / Iceland / Reykjavik 2020-03-15 deCODE genetics deCODE genetics Daniel F Gudbjartsson; Agnar Helgason; Hakon Jonsson; Olafur T Magnusson; Pall Melsted; Gudmundur L Norddahl; Jona Saemundsdottir; Asgeir Sigurdsson; Patrick Sulem; Arna B Agustsdottir; Berglind Eiriksdottir; Run Fridriksdottir; Elisabet E Gardarsdottir; Gudmundur Georgsson; Olafia S Gretarsdottir; Kjartan R Gudmundsson; Thora R Gunnarsdottir; Arnaldur Gylfason; Hilma Holm; Brynjar O Jensson; Aslaug Jonasdottir; Kamilla S Josefsdottir; Thordur Kristjansson; Droplaug N Magnusdottir; Louise le Roux; Gudrun Sigmundsdottir; Gardar Sveinbjornsson; Kristin E Sveinsdottir; Maney Sveinsdottir; Emil A Thorarensen; Bjarni Thorbjornsson; Gisli Masson; Ingileif Jonsdottir; Alma Moller; Thorolfur Gudnason; Karl G Kristinsson; Unnur Thorsteinsdottir; Kari Stefansson
EPI_ISL_417672 hCoV-19/Iceland/38/2020 Europe / Iceland / Reykjavik 2020-03-16 deCODE genetics deCODE genetics Daniel F Gudbjartsson; Agnar Helgason; Hakon Jonsson; Olafur T Magnusson; Pall Melsted; Gudmundur L Norddahl; Jona Saemundsdottir; Asgeir Sigurdsson; Patrick Sulem; Arna B Agustsdottir; Berglind Eiriksdottir; Run Fridriksdottir; Elisabet E Gardarsdottir; Gudmundur Georgsson; Olafia S Gretarsdottir; Kjartan R Gudmundsson; Thora R Gunnarsdottir; Arnaldur Gylfason; Hilma Holm; Brynjar O Jensson; Aslaug Jonasdottir; Kamilla S Josefsdottir; Thordur Kristjansson; Droplaug N Magnusdottir; Louise le Roux; Gudrun Sigmundsdottir; Gardar Sveinbjornsson; Kristin E Sveinsdottir; Maney Sveinsdottir; Emil A Thorarensen; Bjarni Thorbjornsson; Gisli Masson; Ingileif Jonsdottir; Alma Moller; Thorolfur Gudnason; Karl G Kristinsson; Unnur Thorsteinsdottir; Kari Stefansson
EPI_ISL_417676 hCoV-19/Iceland/98/2020 Europe / Iceland / Reykjavik 2020-03-16 deCODE genetics deCODE genetics Daniel F Gudbjartsson; Agnar Helgason; Hakon Jonsson; Olafur T Magnusson; Pall Melsted; Gudmundur L Norddahl; Jona Saemundsdottir; Asgeir Sigurdsson; Patrick Sulem; Arna B Agustsdottir; Berglind Eiriksdottir; Run Fridriksdottir; Elisabet E Gardarsdottir; Gudmundur Georgsson; Olafia S Gretarsdottir; Kjartan R Gudmundsson; Thora R Gunnarsdottir; Arnaldur Gylfason; Hilma Holm; Brynjar O Jensson; Aslaug Jonasdottir; Kamilla S Josefsdottir; Thordur Kristjansson; Droplaug N Magnusdottir; Louise le Roux; Gudrun Sigmundsdottir; Gardar Sveinbjornsson; Kristin E Sveinsdottir; Maney Sveinsdottir; Emil A Thorarensen; Bjarni Thorbjornsson; Gisli Masson; Ingileif Jonsdottir; Alma Moller; Thorolfur Gudnason; Karl G Kristinsson; Unnur Thorsteinsdottir; Kari Stefansson
EPI_ISL_417677 hCoV-19/Iceland/39/2020 Europe / Iceland / Reykjavik 2020-03-04 The National University Hospital of Iceland deCODE genetics Daniel F Gudbjartsson; Agnar Helgason; Hakon Jonsson; Olafur T Magnusson; Pall Melsted; Gudmundur L Norddahl; Jona Saemundsdottir; Asgeir Sigurdsson; Patrick Sulem; Arna B Agustsdottir; Berglind Eiriksdottir; Run Fridriksdottir; Elisabet E Gardarsdottir; Gudmundur Georgsson; Olafia S Gretarsdottir; Kjartan R Gudmundsson; Thora R Gunnarsdottir; Arnaldur Gylfason; Hilma Holm; Brynjar O Jensson; Aslaug Jonasdottir; Kamilla S Josefsdottir; Thordur Kristjansson; Droplaug N Magnusdottir; Louise le Roux; Gudrun Sigmundsdottir; Gardar Sveinbjornsson; Kristin E Sveinsdottir; Maney Sveinsdottir; Emil A Thorarensen; Bjarni Thorbjornsson; Gisli Masson; Ingileif Jonsdottir; Alma Moller; Thorolfur Gudnason; Karl G Kristinsson; Unnur Thorsteinsdottir; Kari Stefansson
EPI_ISL_417678 hCoV-19/Iceland/185/2020 Europe / Iceland / Reykjavik 2020-03-16 The National University Hospital of Iceland deCODE genetics Daniel F Gudbjartsson; Agnar Helgason; Hakon Jonsson; Olafur T Magnusson; Pall Melsted; Gudmundur L Norddahl; Jona Saemundsdottir; Asgeir Sigurdsson; Patrick Sulem; Arna B Agustsdottir; Berglind Eiriksdottir; Run Fridriksdottir; Elisabet E Gardarsdottir; Gudmundur Georgsson; Olafia S Gretarsdottir; Kjartan R Gudmundsson; Thora R Gunnarsdottir; Arnaldur Gylfason; Hilma Holm; Brynjar O Jensson; Aslaug Jonasdottir; Kamilla S Josefsdottir; Thordur Kristjansson; Droplaug N Magnusdottir; Louise le Roux; Gudrun Sigmundsdottir; Gardar Sveinbjornsson; Kristin E Sveinsdottir; Maney Sveinsdottir; Emil A Thorarensen; Bjarni Thorbjornsson; Gisli Masson; Ingileif Jonsdottir; Alma Moller; Thorolfur Gudnason; Karl G

Kristinsson; Unnur Thorsteinsdottir; Kari Stefansson
EPI_ISL_417679 hCoV-19/Iceland/79/2020 Europe / Iceland / Reykjavik 2020-03-09 The National University Hospital of Iceland deCODE genetics Daniel F Gudbjartsson; Agnar Helgason; Hakon Jonsson; Olafur T Magnusson; Pall Melsted; Gudmundur L Norddahl; Jona Saemundsdottir; Asgeir Sigurdsson; Patrick Sulem; Arna B Agustsdottir; Berglind Eiriksdottir; Run Fridriksdottir; Elisabet E Gardarsdottir; Gudmundur Georgsson; Olafia S Gretarsdottir; Kjartan R Gudmundsson; Thora R Gunnarsdottir; Arnaldur Gylfason; Hilma Holm; Brynjar O Jensson; Aslaug Jonasdottir; Kamilla S Josefsdottir; Thordur Kristjansson; Droplaug N Magnusdottir; Louise le Roux; Gudrun Sigmundsdottir; Gardar Sveinbjornsson; Kristin E Sveinsdottir; Maney Sveinsdottir; Emil A Thorarensen; Bjarni Thorbjornsson; Gisli Masson; Ingileif Jonsdottir; Alma Moller; Thorolfur Gudnason; Karl G Kristinsson; Unnur Thorsteinsdottir; Kari Stefansson
EPI_ISL_417680 hCoV-19/Iceland/177/2020 Europe / Iceland / Reykjavik 2020-03-15 The National University Hospital of Iceland deCODE genetics Daniel F Gudbjartsson; Agnar Helgason; Hakon Jonsson; Olafur T Magnusson; Pall Melsted; Gudmundur L Norddahl; Jona Saemundsdottir; Asgeir Sigurdsson; Patrick Sulem; Arna B Agustsdottir; Berglind Eiriksdottir; Run Fridriksdottir; Elisabet E Gardarsdottir; Gudmundur Georgsson; Olafia S Gretarsdottir; Kjartan R Gudmundsson; Thora R Gunnarsdottir; Arnaldur Gylfason; Hilma Holm; Brynjar O Jensson; Aslaug Jonasdottir; Kamilla S Josefsdottir; Thordur Kristjansson; Droplaug N Magnusdottir; Louise le Roux; Gudrun Sigmundsdottir; Gardar Sveinbjornsson; Kristin E Sveinsdottir; Maney Sveinsdottir; Emil A Thorarensen; Bjarni Thorbjornsson; Gisli Masson; Ingileif Jonsdottir; Alma Moller; Thorolfur Gudnason; Karl G Kristinsson; Unnur Thorsteinsdottir; Kari Stefansson
EPI_ISL_417681 hCoV-19/Iceland/37/2020 Europe / Iceland / Reykjavik 2020-03-04 The National University Hospital of Iceland deCODE genetics Daniel F Gudbjartsson; Agnar Helgason; Hakon Jonsson; Olafur T Magnusson; Pall Melsted; Gudmundur L Norddahl; Jona Saemundsdottir; Asgeir Sigurdsson; Patrick Sulem; Arna B Agustsdottir; Berglind Eiriksdottir; Run Fridriksdottir; Elisabet E Gardarsdottir; Gudmundur Georgsson; Olafia S Gretarsdottir; Kjartan R Gudmundsson; Thora R Gunnarsdottir; Arnaldur Gylfason; Hilma Holm; Brynjar O Jensson; Aslaug Jonasdottir; Kamilla S Josefsdottir; Thordur Kristjansson; Droplaug N Magnusdottir; Louise le Roux; Gudrun Sigmundsdottir; Gardar Sveinbjornsson; Kristin E Sveinsdottir; Maney Sveinsdottir; Emil A Thorarensen; Bjarni Thorbjornsson; Gisli Masson; Ingileif Jonsdottir; Alma Moller; Thorolfur Gudnason; Karl G Kristinsson; Unnur Thorsteinsdottir; Kari Stefansson
EPI_ISL_417682 hCoV-19/Iceland/101/2020 Europe / Iceland / Reykjavik 2020-03-10 The National University Hospital of Iceland deCODE genetics Daniel F Gudbjartsson; Agnar Helgason; Hakon Jonsson; Olafur T Magnusson; Pall Melsted; Gudmundur L Norddahl; Jona Saemundsdottir; Asgeir Sigurdsson; Patrick Sulem; Arna B Agustsdottir; Berglind Eiriksdottir; Run Fridriksdottir; Elisabet E Gardarsdottir; Gudmundur Georgsson; Olafia S Gretarsdottir; Kjartan R Gudmundsson; Thora R Gunnarsdottir; Arnaldur Gylfason; Hilma Holm; Brynjar O Jensson; Aslaug Jonasdottir; Kamilla S Josefsdottir; Thordur Kristjansson; Droplaug N Magnusdottir; Louise le Roux; Gudrun Sigmundsdottir; Gardar Sveinbjornsson; Kristin E Sveinsdottir; Maney Sveinsdottir; Emil A Thorarensen; Bjarni Thorbjornsson; Gisli Masson; Ingileif Jonsdottir; Alma Moller; Thorolfur Gudnason; Karl G Kristinsson; Unnur Thorsteinsdottir; Kari Stefansson
EPI_ISL_417683 hCoV-19/Iceland/47/2020 Europe / Iceland / Reykjavik 2020-03-05 The National University Hospital of Iceland deCODE genetics Daniel F Gudbjartsson; Agnar Helgason; Hakon Jonsson; Olafur T Magnusson; Pall Melsted; Gudmundur L Norddahl; Jona Saemundsdottir; Asgeir Sigurdsson; Patrick Sulem; Arna B Agustsdottir; Berglind Eiriksdottir; Run Fridriksdottir; Elisabet E Gardarsdottir; Gudmundur Georgsson; Olafia S Gretarsdottir; Kjartan R Gudmundsson; Thora R Gunnarsdottir; Arnaldur Gylfason; Hilma Holm; Brynjar O Jensson; Aslaug Jonasdottir; Kamilla S Josefsdottir; Thordur Kristjansson; Droplaug N Magnusdottir; Louise le Roux; Gudrun Sigmundsdottir; Gardar Sveinbjornsson; Kristin E Sveinsdottir; Maney Sveinsdottir; Emil A Thorarensen; Bjarni Thorbjornsson; Gisli Masson; Ingileif Jonsdottir; Alma Moller; Thorolfur Gudnason; Karl G Kristinsson; Unnur Thorsteinsdottir; Kari Stefansson
EPI_ISL_417684 hCoV-19/Iceland/54/2020 Europe / Iceland / Reykjavik 2020-03-06 The National University Hospital of Iceland deCODE genetics Daniel F Gudbjartsson; Agnar Helgason; Hakon Jonsson; Olafur T Magnusson; Pall Melsted; Gudmundur L Norddahl; Jona Saemundsdottir; Asgeir Sigurdsson; Patrick Sulem; Arna B Agustsdottir; Berglind Eiriksdottir; Run Fridriksdottir; Elisabet E Gardarsdottir; Gudmundur Georgsson; Olafia S Gretarsdottir; Kjartan R Gudmundsson; Thora R Gunnarsdottir; Arnaldur Gylfason; Hilma Holm; Brynjar O Jensson; Aslaug Jonasdottir; Kamilla S Josefsdottir; Thordur Kristjansson; Droplaug N Magnusdottir; Louise le Roux; Gudrun Sigmundsdottir; Gardar Sveinbjornsson; Kristin E Sveinsdottir; Maney Sveinsdottir; Emil A Thorarensen; Bjarni Thorbjornsson; Gisli Masson; Ingileif Jonsdottir; Alma Moller; Thorolfur Gudnason; Karl G Kristinsson; Unnur Thorsteinsdottir; Kari Stefansson
EPI_ISL_417685 hCoV-19/Iceland/215/2020 Europe / Iceland / Reykjavik 2020-03-16 The National University Hospital of Iceland deCODE genetics Daniel F Gudbjartsson; Agnar Helgason; Hakon Jonsson; Olafur T Magnusson; Pall Melsted; Gudmundur L Norddahl; Jona Saemundsdottir; Asgeir Sigurdsson; Patrick Sulem; Arna B Agustsdottir; Berglind Eiriksdottir; Run Fridriksdottir; Elisabet E Gardarsdottir; Gudmundur Georgsson; Olafia S Gretarsdottir; Kjartan R Gudmundsson; Thora R Gunnarsdottir; Arnaldur Gylfason; Hilma Holm; Brynjar O Jensson; Aslaug Jonasdottir; Kamilla S Josefsdottir; Thordur Kristjansson; Droplaug N Magnusdottir; Louise le Roux; Gudrun Sigmundsdottir; Gardar Sveinbjornsson; Kristin E Sveinsdottir; Maney Sveinsdottir; Emil A Thorarensen; Bjarni Thorbjornsson; Gisli Masson; Ingileif Jonsdottir; Alma Moller; Thorolfur Gudnason; Karl G Kristinsson; Unnur Thorsteinsdottir; Kari Stefansson
EPI_ISL_417688 hCoV-19/Iceland/12/2020 Europe / Iceland / Reykjavik 2020-03-01 The National University Hospital of Iceland deCODE genetics Daniel F Gudbjartsson; Agnar Helgason; Hakon Jonsson; Olafur T Magnusson; Pall Melsted; Gudmundur L Norddahl; Jona Saemundsdottir; Asgeir Sigurdsson; Patrick Sulem; Arna B Agustsdottir; Berglind Eiriksdottir; Run Fridriksdottir; Elisabet E Gardarsdottir; Gudmundur Georgsson; Olafia S Gretarsdottir; Kjartan R Gudmundsson; Thora R Gunnarsdottir; Arnaldur Gylfason; Hilma Holm; Brynjar O Jensson; Aslaug Jonasdottir; Kamilla S Josefsdottir; Thordur Kristjansson; Droplaug N Magnusdottir; Louise le

Roux; Gudrun Sigmundsdottir; Gardar Sveinbjornsson; Kristin E Sveinsdottir; Maney Sveinsdottir; Emil A Thorarensen; Bjarni Thorbjornsson; Gisli Masson; Ingileif Jonsdottir; Alma Moller; Thorolfur Gudnason; Karl G Kristinsson; Unnur Thorsteinsdottir; Kari Stefansson
EPI_ISL_417690 hCoV-19/Iceland/125/2020 Europe / Iceland / Reykjavik 2020-03-11 The National University Hospital of Iceland deCODE genetics Daniel F Gudbjartsson; Agnar Helgason; Hakon Jonsson; Olafur T Magnusson; Pall Melsted; Gudmundur L Norddahl; Jona Saemundsdottir; Asgeir Sigurdsson; Patrick Sulem; Arna B Agustsdottir; Berglind Eiriksdottir; Run Fridriksdottir; Elisabet E Gardarsdottir; Gudmundur Georgsson; Olafia S Gretarsdottir; Kjartan R Gudmundsson; Thora R Gunnarsdottir; Arnaldur Gylfason; Hilma Holm; Brynjar O Jensson; Aslaug Jonasdottir; Kamilla S Josefsdottir; Thordur Kristjansson; Droplaug N Magnusdottir; Louise le Roux; Gudrun Sigmundsdottir; Gardar Sveinbjornsson; Kristin E Sveinsdottir; Maney Sveinsdottir; Emil A Thorarensen; Bjarni Thorbjornsson; Gisli Masson; Ingileif Jonsdottir; Alma Moller; Thorolfur Gudnason; Karl G Kristinsson; Unnur Thorsteinsdottir; Kari Stefansson
EPI_ISL_417691 hCoV-19/Iceland/111/2020 Europe / Iceland / Reykjavik 2020-03-11 The National University Hospital of Iceland deCODE genetics Daniel F Gudbjartsson; Agnar Helgason; Hakon Jonsson; Olafur T Magnusson; Pall Melsted; Gudmundur L Norddahl; Jona Saemundsdottir; Asgeir Sigurdsson; Patrick Sulem; Arna B Agustsdottir; Berglind Eiriksdottir; Run Fridriksdottir; Elisabet E Gardarsdottir; Gudmundur Georgsson; Olafia S Gretarsdottir; Kjartan R Gudmundsson; Thora R Gunnarsdottir; Arnaldur Gylfason; Hilma Holm; Brynjar O Jensson; Aslaug Jonasdottir; Kamilla S Josefsdottir; Thordur Kristjansson; Droplaug N Magnusdottir; Louise le Roux; Gudrun Sigmundsdottir; Gardar Sveinbjornsson; Kristin E Sveinsdottir; Maney Sveinsdottir; Emil A Thorarensen; Bjarni Thorbjornsson; Gisli Masson; Ingileif Jonsdottir; Alma Moller; Thorolfur Gudnason; Karl G Kristinsson; Unnur Thorsteinsdottir; Kari Stefansson
EPI_ISL_417692 hCoV-19/Iceland/57/2020 Europe / Iceland / Reykjavik 2020-03-06 The National University Hospital of Iceland deCODE genetics Daniel F Gudbjartsson; Agnar Helgason; Hakon Jonsson; Olafur T Magnusson; Pall Melsted; Gudmundur L Norddahl; Jona Saemundsdottir; Asgeir Sigurdsson; Patrick Sulem; Arna B Agustsdottir; Berglind Eiriksdottir; Run Fridriksdottir; Elisabet E Gardarsdottir; Gudmundur Georgsson; Olafia S Gretarsdottir; Kjartan R Gudmundsson; Thora R Gunnarsdottir; Arnaldur Gylfason; Hilma Holm; Brynjar O Jensson; Aslaug Jonasdottir; Kamilla S Josefsdottir; Thordur Kristjansson; Droplaug N Magnusdottir; Louise le Roux; Gudrun Sigmundsdottir; Gardar Sveinbjornsson; Kristin E Sveinsdottir; Maney Sveinsdottir; Emil A Thorarensen; Bjarni Thorbjornsson; Gisli Masson; Ingileif Jonsdottir; Alma Moller; Thorolfur Gudnason; Karl G Kristinsson; Unnur Thorsteinsdottir; Kari Stefansson
EPI_ISL_417693 hCoV-19/Iceland/25/2020 Europe / Iceland / Reykjavik 2020-03-02 The National University Hospital of Iceland deCODE genetics Daniel F Gudbjartsson; Agnar Helgason; Hakon Jonsson; Olafur T Magnusson; Pall Melsted; Gudmundur L Norddahl; Jona Saemundsdottir; Asgeir Sigurdsson; Patrick Sulem; Arna B Agustsdottir; Berglind Eiriksdottir; Run Fridriksdottir; Elisabet E Gardarsdottir; Gudmundur Georgsson; Olafia S Gretarsdottir; Kjartan R Gudmundsson; Thora R Gunnarsdottir; Arnaldur Gylfason; Hilma Holm; Brynjar O Jensson; Aslaug Jonasdottir; Kamilla S Josefsdottir; Thordur Kristjansson; Droplaug N Magnusdottir; Louise le Roux; Gudrun Sigmundsdottir; Gardar Sveinbjornsson; Kristin E Sveinsdottir; Maney Sveinsdottir; Emil A Thorarensen; Bjarni Thorbjornsson; Gisli Masson; Ingileif Jonsdottir; Alma Moller; Thorolfur Gudnason; Karl G Kristinsson; Unnur Thorsteinsdottir; Kari Stefansson
EPI_ISL_417694 hCoV-19/Iceland/3/2020 Europe / Iceland / Reykjavik 2020-03-13 The National University Hospital of Iceland deCODE genetics Daniel F Gudbjartsson; Agnar Helgason; Hakon Jonsson; Olafur T Magnusson; Pall Melsted; Gudmundur L Norddahl; Jona Saemundsdottir; Asgeir Sigurdsson; Patrick Sulem; Arna B Agustsdottir; Berglind Eiriksdottir; Run Fridriksdottir; Elisabet E Gardarsdottir; Gudmundur Georgsson; Olafia S Gretarsdottir; Kjartan R Gudmundsson; Thora R Gunnarsdottir; Arnaldur Gylfason; Hilma Holm; Brynjar O Jensson; Aslaug Jonasdottir; Kamilla S Josefsdottir; Thordur Kristjansson; Droplaug N Magnusdottir; Louise le Roux; Gudrun Sigmundsdottir; Gardar Sveinbjornsson; Kristin E Sveinsdottir; Maney Sveinsdottir; Emil A Thorarensen; Bjarni Thorbjornsson; Gisli Masson; Ingileif Jonsdottir; Alma Moller; Thorolfur Gudnason; Karl G Kristinsson; Unnur Thorsteinsdottir; Kari Stefansson
EPI_ISL_417695 hCoV-19/Iceland/20/2020 Europe / Iceland / Reykjavik 2020-03-02 The National University Hospital of Iceland deCODE genetics Daniel F Gudbjartsson; Agnar Helgason; Hakon Jonsson; Olafur T Magnusson; Pall Melsted; Gudmundur L Norddahl; Jona Saemundsdottir; Asgeir Sigurdsson; Patrick Sulem; Arna B Agustsdottir; Berglind Eiriksdottir; Run Fridriksdottir; Elisabet E Gardarsdottir; Gudmundur Georgsson; Olafia S Gretarsdottir; Kjartan R Gudmundsson; Thora R Gunnarsdottir; Arnaldur Gylfason; Hilma Holm; Brynjar O Jensson; Aslaug Jonasdottir; Kamilla S Josefsdottir; Thordur Kristjansson; Droplaug N Magnusdottir; Louise le Roux; Gudrun Sigmundsdottir; Gardar Sveinbjornsson; Kristin E Sveinsdottir; Maney Sveinsdottir; Emil A Thorarensen; Bjarni Thorbjornsson; Gisli Masson; Ingileif Jonsdottir; Alma Moller; Thorolfur Gudnason; Karl G Kristinsson; Unnur Thorsteinsdottir; Kari Stefansson
EPI_ISL_417696 hCoV-19/Iceland/135/2020 Europe / Iceland / Reykjavik 2020-03-12 The National University Hospital of Iceland deCODE genetics Daniel F Gudbjartsson; Agnar Helgason; Hakon Jonsson; Olafur T Magnusson; Pall Melsted; Gudmundur L Norddahl; Jona Saemundsdottir; Asgeir Sigurdsson; Patrick Sulem; Arna B Agustsdottir; Berglind Eiriksdottir; Run Fridriksdottir; Elisabet E Gardarsdottir; Gudmundur Georgsson; Olafia S Gretarsdottir; Kjartan R Gudmundsson; Thora R Gunnarsdottir; Arnaldur Gylfason; Hilma Holm; Brynjar O Jensson; Aslaug Jonasdottir; Kamilla S Josefsdottir; Thordur Kristjansson; Droplaug N Magnusdottir; Louise le Roux; Gudrun Sigmundsdottir; Gardar Sveinbjornsson; Kristin E Sveinsdottir; Maney Sveinsdottir; Emil A Thorarensen; Bjarni Thorbjornsson; Gisli Masson; Ingileif Jonsdottir; Alma Moller; Thorolfur Gudnason; Karl G Kristinsson; Unnur Thorsteinsdottir; Kari Stefansson
EPI_ISL_417698 hCoV-19/Iceland/103/2020 Europe / Iceland / Reykjavik 2020-03-10 The National University Hospital of Iceland deCODE genetics Daniel F Gudbjartsson; Agnar Helgason; Hakon Jonsson; Olafur T Magnusson; Pall Melsted; Gudmundur L Norddahl; Jona Saemundsdottir; Asgeir Sigurdsson; Patrick Sulem; Arna B Agustsdottir; Berglind Eiriksdottir; Run Fridriksdottir; Elisabet E Gardarsdottir; Gudmundur Georgsson; Olafia

S Gretarsdottir; Kjartan R Gudmundsson; Thora R Gunnarsdottir; Arnaldur Gylfason; Hilma Holm; Brynjar O Jensson; Aslaug Jonasdottir; Kamilla S Josefsdottir; Thordur Kristjansson; Droplaug N Magnusdottir; Louise le Roux; Gudrun Sigmundsdottir; Gardar Sveinbjornsson; Kristin E Sveinsdottir; Maney Sveinsdottir; Emil A Thorarensen; Bjarni Thorbjornsson; Gisli Masson; Ingileif Jonsdottir; Alma Moller; Thorolfur Gudnason; Karl G Kristinsson; Unnur Thorsteinsdottir; Kari Stefansson
EPI_ISL_417699 hCoV-19/Iceland/206/2020 Europe / Iceland / Reykjavik 2020-03-16 The National University Hospital of Iceland deCODE genetics Daniel F Gudbjartsson; Agnar Helgason; Hakon Jonsson; Olafur T Magnusson; Pall Melsted; Gudmundur L Norddahl; Jona Saemundsdottir; Asgeir Sigurdsson; Patrick Sulem; Arna B Agustsdottir; Berglind Eiriksdottir; Run Fridriksdottir; Elisabet E Gardarsdottir; Gudmundur Georgsson; Olafia S Gretarsdottir; Kjartan R Gudmundsson; Thora R Gunnarsdottir; Arnaldur Gylfason; Hilma Holm; Brynjar O Jensson; Aslaug Jonasdottir; Kamilla S Josefsdottir; Thordur Kristjansson; Droplaug N Magnusdottir; Louise le Roux; Gudrun Sigmundsdottir; Gardar Sveinbjornsson; Kristin E Sveinsdottir; Maney Sveinsdottir; Emil A Thorarensen; Bjarni Thorbjornsson; Gisli Masson; Ingileif Jonsdottir; Alma Moller; Thorolfur Gudnason; Karl G Kristinsson; Unnur Thorsteinsdottir; Kari Stefansson
EPI_ISL_418816 hCoV-19/Canada/BC_0443574/2020 North America / Canada / British Columbia 2020-03-11 BCCDC Public Health Laboratory BCCDC Public Health Laboratory Harrigan, Prystajecky, Krajden, Lee, Kamelian, Lapointe, Choi, Hoang, Sekirov, Levett, Tyson, Snutch, Loman, Quick, Li, Gilmour
EPI_ISL_418817 hCoV-19/Canada/BC_0554880/2020 North America / Canada / British Columbia 2020-03-13 BCCDC Public Health Laboratory BCCDC Public Health Laboratory Harrigan, Prystajecky, Krajden, Lee, Kamelian, Lapointe, Choi, Hoang, Sekirov, Levett, Tyson, Snutch, Loman, Quick, Li, Gilmour
EPI_ISL_418818 hCoV-19/Canada/BC_1318414/2020 North America / Canada / British Columbia 2020-03-06 BCCDC Public Health Laboratory BCCDC Public Health Laboratory Harrigan, Prystajecky, Krajden, Lee, Kamelian, Lapointe, Choi, Hoang, Sekirov, Levett, Tyson, Snutch, Loman, Quick, Li, Gilmour
EPI_ISL_418838 hCoV-19/Canada/BC_6404889/2020 North America / Canada / British Columbia 2020-03-12 BCCDC Public Health Laboratory BCCDC Public Health Laboratory Harrigan, Prystajecky, Krajden, Lee, Kamelian, Lapointe, Choi, Hoang, Sekirov, Levett, Tyson, Snutch, Loman, Quick, Li, Gilmour
EPI_ISL_418839 hCoV-19/Canada/BC_6489864/2020 North America / Canada / British Columbia 2020-03-10 BCCDC Public Health Laboratory BCCDC Public Health Laboratory Harrigan, Prystajecky, Krajden, Lee, Kamelian, Lapointe, Choi, Hoang, Sekirov, Levett, Tyson, Snutch, Loman, Quick, Li, Gilmour
EPI_ISL_418840 hCoV-19/Canada/BC_6502001/2020 North America / Canada / British Columbia 2020-03-12 BCCDC Public Health Laboratory BCCDC Public Health Laboratory Harrigan, Prystajecky, Krajden, Lee, Kamelian, Lapointe, Choi, Hoang, Sekirov, Levett, Tyson, Snutch, Loman, Quick, Li, Gilmour
EPI_ISL_418841 hCoV-19/Canada/BC_6981299/2020 North America / Canada / British Columbia 2020-03-11 BCCDC Public Health Laboratory BCCDC Public Health Laboratory Harrigan, Prystajecky, Krajden, Lee, Kamelian, Lapointe, Choi, Hoang, Sekirov, Levett, Tyson, Snutch, Loman, Quick, Li, Gilmour
EPI_ISL_418842 hCoV-19/Canada/BC_6997898/2020 North America / Canada / British Columbia 2020-03-11 BCCDC Public Health Laboratory BCCDC Public Health Laboratory Harrigan, Prystajecky, Krajden, Lee, Kamelian, Lapointe, Choi, Hoang, Sekirov, Levett, Tyson, Snutch, Loman, Quick, Li, Gilmour
EPI_ISL_418843 hCoV-19/Canada/BC_7277381/2020 North America / Canada / British Columbia 2020-03-12 BCCDC Public Health Laboratory BCCDC Public Health Laboratory Harrigan, Prystajecky, Krajden, Lee, Kamelian, Lapointe, Choi, Hoang, Sekirov, Levett, Tyson, Snutch, Loman, Quick, Li, Gilmour
EPI_ISL_418845 hCoV-19/Canada/BC_7515663/2020 North America / Canada / British Columbia 2020-03-11 BCCDC Public Health Laboratory BCCDC Public Health Laboratory Harrigan, Prystajecky, Krajden, Lee, Kamelian, Lapointe, Choi, Hoang, Sekirov, Levett, Tyson, Snutch, Loman, Quick, Li, Gilmour
EPI_ISL_418846 hCoV-19/Canada/BC_7553799/2020 North America / Canada / British Columbia 2020-03-12 BCCDC Public Health Laboratory BCCDC Public Health Laboratory Harrigan, Prystajecky, Krajden, Lee, Kamelian, Lapointe, Choi, Hoang, Sekirov, Levett, Tyson, Snutch, Loman, Quick, Li, Gilmour
EPI_ISL_418847 hCoV-19/Canada/BC_8150537/2020 North America / Canada / British Columbia 2020-03-12 BCCDC Public Health Laboratory BCCDC Public Health Laboratory Harrigan, Prystajecky, Krajden, Lee, Kamelian, Lapointe, Choi, Hoang, Sekirov, Levett, Tyson, Snutch, Loman, Quick, Li, Gilmour
EPI_ISL_418848 hCoV-19/Canada/BC_8159203/2020 North America / Canada / British Columbia 2020-03-05 BCCDC Public Health Laboratory BCCDC Public Health Laboratory Harrigan, Prystajecky, Krajden, Lee, Kamelian, Lapointe, Choi, Hoang, Sekirov, Levett, Tyson, Snutch, Loman, Quick, Li, Gilmour
EPI_ISL_418849 hCoV-19/Canada/BC_8486790/2020 North America / Canada / British Columbia 2020-03-13 BCCDC Public Health Laboratory BCCDC Public Health Laboratory Harrigan, Prystajecky, Krajden, Lee, Kamelian, Lapointe, Choi, Hoang, Sekirov, Levett, Tyson, Snutch, Loman, Quick, Li, Gilmour
EPI_ISL_418851 hCoV-19/Canada/BC_8622445/2020 North America / Canada / British Columbia 2020-03-13 BCCDC Public Health Laboratory BCCDC Public Health Laboratory Harrigan, Prystajecky, Krajden, Lee, Kamelian, Lapointe, Choi, Hoang, Sekirov, Levett, Tyson, Snutch, Loman, Quick, Li, Gilmour
EPI_ISL_418852 hCoV-19/Canada/BC_8718874/2020 North America / Canada / British Columbia 2020-03-07 BCCDC Public Health Laboratory BCCDC Public Health Laboratory Harrigan, Prystajecky, Krajden, Lee, Kamelian, Lapointe, Choi, Hoang, Sekirov, Levett, Tyson, Snutch, Loman, Quick, Li, Gilmour
EPI_ISL_418853 hCoV-19/Canada/BC_8894200/2020 North America / Canada / British Columbia 2020-03-11 BCCDC Public Health Laboratory BCCDC Public Health Laboratory Harrigan, Prystajecky, Krajden, Lee, Kamelian, Lapointe, Choi, Hoang, Sekirov, Levett, Tyson, Snutch, Loman, Quick, Li, Gilmour
EPI_ISL_418854 hCoV-19/Canada/BC_8896915/2020 North America / Canada / British Columbia 2020-03-11 BCCDC Public Health Laboratory BCCDC Public Health Laboratory Harrigan, Prystajecky, Krajden, Lee, Kamelian, Lapointe, Choi, Hoang, Sekirov, Levett, Tyson, Snutch, Loman, Quick, Li, Gilmour
EPI_ISL_418855 hCoV-19/Canada/BC_8897642/2020 North America / Canada / British Columbia 2020-03-11 BCCDC Public Health Laboratory BCCDC Public Health Laboratory Harrigan, Prystajecky, Krajden, Lee, Kamelian,

Lapointe, Choi, Hoang, Sekirov, Levett, Tyson, Snutch, Loman, Quick, Li, Gilmour
EPI_ISL_418856 hCoV-19/Canada/BC_9345042/2020 North America / Canada / British Columbia 2020-03-13 BCCDC Public Health Laboratory BCCDC Public Health Laboratory Harrigan, Prystajecky, Krajden, Lee, Kamelian, Lapointe, Choi, Hoang, Sekirov, Levett, Tyson, Snutch, Loman, Quick, Li, Gilmour
EPI_ISL_418857 hCoV-19/Canada/BC_9345715/2020 North America / Canada / British Columbia 2020-03-13 BCCDC Public Health Laboratory BCCDC Public Health Laboratory Harrigan, Prystajecky, Krajden, Lee, Kamelian, Lapointe, Choi, Hoang, Sekirov, Levett, Tyson, Snutch, Loman, Quick, Li, Gilmour
EPI_ISL_418858 hCoV-19/Canada/BC_9446031/2020 North America / Canada / British Columbia 2020-03-13 BCCDC Public Health Laboratory BCCDC Public Health Laboratory Harrigan, Prystajecky, Krajden, Lee, Kamelian, Lapointe, Choi, Hoang, Sekirov, Levett, Tyson, Snutch, Loman, Quick, Li, Gilmour
EPI_ISL_418900 hCoV-19/USA/WA-UW333/2020 North America / USA / Washington 2020-03-16 UW Virology Lab UW Virology Lab Pavitra Roychoudhury, Hong Xie, Keith Jerome, Alexander Greninger
EPI_ISL_418901 hCoV-19/USA/MN-UW334/2020 North America / USA / Minnesota 2020-03-13 UW Virology Lab UW Virology Lab Pavitra Roychoudhury, Hong Xie, Keith Jerome, Alexander Greninger
EPI_ISL_418902 hCoV-19/USA/MN-UW335/2020 North America / USA / Minnesota 2020-03-13 UW Virology Lab UW Virology Lab Pavitra Roychoudhury, Hong Xie, Keith Jerome, Alexander Greninger
EPI_ISL_418903 hCoV-19/USA/WA-UW336/2020 North America / USA / Washington 2020-03-16 UW Virology Lab UW Virology Lab Pavitra Roychoudhury, Hong Xie, Keith Jerome, Alexander Greninger
EPI_ISL_418904 hCoV-19/USA/WA-UW337/2020 North America / USA / Washington 2020-03-16 UW Virology Lab UW Virology Lab Pavitra Roychoudhury, Hong Xie, Keith Jerome, Alexander Greninger
EPI_ISL_418905 hCoV-19/USA/WA-UW338/2020 North America / USA / Washington 2020-03-16 UW Virology Lab UW Virology Lab Pavitra Roychoudhury, Hong Xie, Keith Jerome, Alexander Greninger
EPI_ISL_418906 hCoV-19/USA/WA-UW339/2020 North America / USA / Washington 2020-03-16 UW Virology Lab UW Virology Lab Pavitra Roychoudhury, Hong Xie, Keith Jerome, Alexander Greninger
EPI_ISL_418907 hCoV-19/USA/WA-UW340/2020 North America / USA / Washington 2020-03-16 UW Virology Lab UW Virology Lab Pavitra Roychoudhury, Hong Xie, Keith Jerome, Alexander Greninger
EPI_ISL_418908 hCoV-19/USA/WA-UW341/2020 North America / USA / Washington 2020-03-16 UW Virology Lab UW Virology Lab Pavitra Roychoudhury, Hong Xie, Keith Jerome, Alexander Greninger
EPI_ISL_418909 hCoV-19/USA/WA-UW342/2020 North America / USA / Washington 2020-03-16 UW Virology Lab UW Virology Lab Pavitra Roychoudhury, Hong Xie, Keith Jerome, Alexander Greninger
EPI_ISL_418910 hCoV-19/USA/WA-UW343/2020 North America / USA / Washington 2020-03-16 UW Virology Lab UW Virology Lab Pavitra Roychoudhury, Hong Xie, Keith Jerome, Alexander Greninger
EPI_ISL_418911 hCoV-19/USA/WA-UW344/2020 North America / USA / Washington 2020-03-16 UW Virology Lab UW Virology Lab Pavitra Roychoudhury, Hong Xie, Keith Jerome, Alexander Greninger
EPI_ISL_418912 hCoV-19/USA/WA-UW345/2020 North America / USA / Washington 2020-03-16 UW Virology Lab UW Virology Lab Pavitra Roychoudhury, Hong Xie, Keith Jerome, Alexander Greninger
EPI_ISL_418913 hCoV-19/USA/WA-UW346/2020 North America / USA / Washington 2020-03-16 UW Virology Lab UW Virology Lab Pavitra Roychoudhury, Hong Xie, Keith Jerome, Alexander Greninger
EPI_ISL_418915 hCoV-19/USA/IL-UW348/2020 North America / USA / Illinois 2020-03-13 UW Virology Lab UW Virology Lab Pavitra Roychoudhury, Hong Xie, Keith Jerome, Alexander Greninger
EPI_ISL_418916 hCoV-19/USA/IL-UW349/2020 North America / USA / Illinois 2020-03-13 UW Virology Lab UW Virology Lab Pavitra Roychoudhury, Hong Xie, Keith Jerome, Alexander Greninger
EPI_ISL_418917 hCoV-19/USA/IL-UW350/2020 North America / USA / Illinois 2020-03-14 UW Virology Lab UW Virology Lab Pavitra Roychoudhury, Hong Xie, Keith Jerome, Alexander Greninger
EPI_ISL_418918 hCoV-19/USA/IL-UW351/2020 North America / USA / Illinois 2020-03-13 UW Virology Lab UW Virology Lab Pavitra Roychoudhury, Hong Xie, Keith Jerome, Alexander Greninger
EPI_ISL_418919 hCoV-19/USA/IL-UW352/2020 North America / USA / Illinois 2020-03-13 UW Virology Lab UW Virology Lab Pavitra Roychoudhury, Hong Xie, Keith Jerome, Alexander Greninger
EPI_ISL_418920 hCoV-19/USA/IL-UW353/2020 North America / USA / Illinois 2020-03-13 UW Virology Lab UW Virology Lab Pavitra Roychoudhury, Hong Xie, Keith Jerome, Alexander Greninger
EPI_ISL_418921 hCoV-19/USA/UN-UW354/2020 North America / USA 2020-03-16 UW Virology Lab UW Virology Lab Pavitra Roychoudhury, Hong Xie, Keith Jerome, Alexander Greninger
EPI_ISL_418922 hCoV-19/USA/WA-UW355/2020 North America / USA / Washington 2020-03-16 UW Virology Lab UW Virology Lab Pavitra Roychoudhury, Hong Xie, Keith Jerome, Alexander Greninger
EPI_ISL_418923 hCoV-19/USA/WA-UW356/2020 North America / USA / Washington 2020-03-16 UW Virology Lab UW Virology Lab Pavitra Roychoudhury, Hong Xie, Keith Jerome, Alexander Greninger
EPI_ISL_418924 hCoV-19/USA/WA-UW357/2020 North America / USA / Washington 2020-03-14 UW Virology Lab UW Virology Lab Pavitra Roychoudhury, Hong Xie, Keith Jerome, Alexander Greninger
EPI_ISL_418925 hCoV-19/USA/UN-UW358/2020 North America / USA 2020-03 UW Virology Lab UW Virology Lab Pavitra Roychoudhury, Hong Xie, Keith Jerome, Alexander Greninger
EPI_ISL_418926 hCoV-19/USA/WA-UW359/2020 North America / USA / Washington 2020-03-14 UW Virology Lab UW Virology Lab Pavitra Roychoudhury, Hong Xie, Keith Jerome, Alexander Greninger
EPI_ISL_418927 hCoV-19/USA/WA-UW360/2020 North America / USA / Washington 2020-03-16 UW Virology Lab UW Virology Lab Pavitra Roychoudhury, Hong Xie, Keith Jerome, Alexander Greninger
EPI_ISL_418928 hCoV-19/USA/WA-UW361/2020 North America / USA / Washington 2020-03-15 UW Virology Lab UW Virology Lab Pavitra Roychoudhury, Hong Xie, Keith Jerome, Alexander Greninger
EPI_ISL_418929 hCoV-19/USA/WA-UW362/2020 North America / USA / Washington 2020-03-17 UW Virology Lab UW Virology Lab Pavitra Roychoudhury, Hong Xie, Keith Jerome, Alexander Greninger
EPI_ISL_418930 hCoV-19/USA/WA-UW363/2020 North America / USA / Washington 2020-03-17 UW Virology Lab UW Virology Lab Pavitra Roychoudhury, Hong Xie, Keith Jerome, Alexander Greninger

EPI_ISL_418931 hCoV-19/USA/WA-UW364/2020 North America / USA / Washington 2020-03-17 UW Virology Lab UW Virology Lab Pavitra Roychoudhury, Hong Xie, Keith Jerome, Alexander Greninger
EPI_ISL_418932 hCoV-19/USA/WA-UW365/2020 North America / USA / Washington 2020-03-16 UW Virology Lab UW Virology Lab Pavitra Roychoudhury, Hong Xie, Keith Jerome, Alexander Greninger
EPI_ISL_418933 hCoV-19/USA/WA-UW366/2020 North America / USA / Washington 2020-03-17 UW Virology Lab UW Virology Lab Pavitra Roychoudhury, Hong Xie, Keith Jerome, Alexander Greninger
EPI_ISL_418934 hCoV-19/USA/WA-UW367/2020 North America / USA / Washington 2020-03-17 UW Virology Lab UW Virology Lab Pavitra Roychoudhury, Hong Xie, Keith Jerome, Alexander Greninger
EPI_ISL_418935 hCoV-19/USA/WA-UW368/2020 North America / USA / Washington 2020-03-16 UW Virology Lab UW Virology Lab Pavitra Roychoudhury, Hong Xie, Keith Jerome, Alexander Greninger
EPI_ISL_418936 hCoV-19/USA/WA-UW369/2020 North America / USA / Washington 2020-03-17 UW Virology Lab UW Virology Lab Pavitra Roychoudhury, Hong Xie, Keith Jerome, Alexander Greninger
EPI_ISL_418937 hCoV-19/USA/WA-UW370/2020 North America / USA / Washington 2020-03-17 UW Virology Lab UW Virology Lab Pavitra Roychoudhury, Hong Xie, Keith Jerome, Alexander Greninger
EPI_ISL_418938 hCoV-19/USA/WA-UW371/2020 North America / USA / Washington 2020-03-16 UW Virology Lab UW Virology Lab Pavitra Roychoudhury, Hong Xie, Keith Jerome, Alexander Greninger
EPI_ISL_418939 hCoV-19/USA/WA-UW372/2020 North America / USA / Washington 2020-03-17 UW Virology Lab UW Virology Lab Pavitra Roychoudhury, Hong Xie, Keith Jerome, Alexander Greninger
EPI_ISL_418940 hCoV-19/USA/WA-UW373/2020 North America / USA / Washington 2020-03-17 UW Virology Lab UW Virology Lab Pavitra Roychoudhury, Hong Xie, Keith Jerome, Alexander Greninger
EPI_ISL_418941 hCoV-19/USA/WA-UW374/2020 North America / USA 2020-03 UW Virology Lab UW Virology Lab Pavitra Roychoudhury, Hong Xie, Keith Jerome, Alexander Greninger
EPI_ISL_418942 hCoV-19/USA/WA-UW375/2020 North America / USA / Washington 2020-03-17 UW Virology Lab UW Virology Lab Pavitra Roychoudhury, Hong Xie, Keith Jerome, Alexander Greninger
EPI_ISL_418943 hCoV-19/USA/WA-UW376/2020 North America / USA / Washington 2020-03-17 UW Virology Lab UW Virology Lab Pavitra Roychoudhury, Hong Xie, Keith Jerome, Alexander Greninger
EPI_ISL_418944 hCoV-19/USA/WA-UW378/2020 North America / USA / Washington 2020-03-15 UW Virology Lab UW Virology Lab Pavitra Roychoudhury, Hong Xie, Keith Jerome, Alexander Greninger
EPI_ISL_418945 hCoV-19/USA/WA-UW379/2020 North America / USA / Washington 2020-03-15 UW Virology Lab UW Virology Lab Pavitra Roychoudhury, Hong Xie, Keith Jerome, Alexander Greninger
EPI_ISL_418948 hCoV-19/USA/WA-UW383/2020 North America / USA / Washington 2020-03-16 UW Virology Lab UW Virology Lab Pavitra Roychoudhury, Hong Xie, Keith Jerome, Alexander Greninger
EPI_ISL_418949 hCoV-19/USA/WA-UW384/2020 North America / USA / Washington 2020-03-16 UW Virology Lab UW Virology Lab Pavitra Roychoudhury, Hong Xie, Keith Jerome, Alexander Greninger
EPI_ISL_418950 hCoV-19/USA/WA-UW385/2020 North America / USA / Washington 2020-03-16 UW Virology Lab UW Virology Lab Pavitra Roychoudhury, Hong Xie, Keith Jerome, Alexander Greninger
EPI_ISL_418951 hCoV-19/USA/WA-UW386/2020 North America / USA / Washington 2020-03-16 UW Virology Lab UW Virology Lab Pavitra Roychoudhury, Hong Xie, Keith Jerome, Alexander Greninger
EPI_ISL_418952 hCoV-19/USA/WA-UW387/2020 North America / USA / Washington 2020-03-16 UW Virology Lab UW Virology Lab Pavitra Roychoudhury, Hong Xie, Keith Jerome, Alexander Greninger
EPI_ISL_418953 hCoV-19/USA/WA-UW388/2020 North America / USA / Washington 2020-03-16 UW Virology Lab UW Virology Lab Pavitra Roychoudhury, Hong Xie, Keith Jerome, Alexander Greninger
EPI_ISL_418954 hCoV-19/USA/WA-UW389/2020 North America / USA / Washington 2020-03-17 UW Virology Lab UW Virology Lab Pavitra Roychoudhury, Hong Xie, Keith Jerome, Alexander Greninger
EPI_ISL_418955 hCoV-19/USA/WA-UW390/2020 North America / USA / Washington 2020-03-15 UW Virology Lab UW Virology Lab Pavitra Roychoudhury, Hong Xie, Keith Jerome, Alexander Greninger
EPI_ISL_418956 hCoV-19/USA/VA-DCLS-0002/2020 North America / USA / Virginia 2020-03-17 Virginia DCLS Virginia DCLS Virginia DCLS
EPI_ISL_418957 hCoV-19/USA/VA-DCLS-0003/2020 North America / USA / Virginia 2020-03-17 Virginia DCLS Virginia DCLS Virginia DCLS
EPI_ISL_418958 hCoV-19/USA/VA-DCLS-0004/2020 North America / USA / Virginia 2020-03-17 Virginia DCLS Virginia DCLS Virginia DCLS
EPI_ISL_418962 hCoV-19/USA/UT-019/2020 North America / USA / Utah 2020-03-13 Utah Public Health Laboratory Utah Public Health Laboratory Erin Young, Kelly Oakeson
EPI_ISL_418963 hCoV-19/USA/UT-021/2020 North America / USA / Utah 2020-03-14 Utah Public Health Laboratory Utah Public Health Laboratory Erin Young, Kelly Oakeson
EPI_ISL_418964 hCoV-19/USA/UT-022/2020 North America / USA / Utah 2020-03-14 Utah Public Health Laboratory Utah Public Health Laboratory Erin Young, Kelly Oakeson
EPI_ISL_418965 hCoV-19/USA/UT-023/2020 North America / USA / Utah 2020-03-11 Utah Public Health Laboratory Utah Public Health Laboratory Erin Young, Kelly Oakeson
EPI_ISL_418967 hCoV-19/USA/UT-025/2020 North America / USA / Utah 2020-03-11 Utah Public Health Laboratory Utah Public Health Laboratory Erin Young, Kelly Oakeson
EPI_ISL_418972 hCoV-19/USA/NY-NYUMC26/2020 North America / USA / New York / Manhattan 2020-03-17 NYU Langone Health Department of Pathology and Medicine, New York University School of Medicine Maria Aguero-Rosenfeld, Margaret Black, John Cadley, Paolo Cotzia, John Chen, Dacia Dimartino, Xiaojun Feng, Adriana Heguy, Megan Hogan, Emily Huang, George Jour, Christian Marier, Matthew T. Maurano, Mark J. Mulligan, Peter Meyn, Jared Pinnell, Sitharam Ramaswami, Amy Rapkiewicz, Marie Samanovic-Golden, Antonio Serrano, Guomiao Shen, Matija Snuderl, Nick Vulpescu, Gael Westby, Paul Zappile, Yutong Zhang
EPI_ISL_418973 hCoV-19/USA/NY-NYUMC27/2020 North America / USA / New York / Manhattan 2020-03-17 NYU Langone Health Department of Pathology and Medicine, New York University School of Medicine Maria

Aguero-Rosenfeld, Margaret Black, John Cadley, Paolo Cotzia, John Chen, Dacia Dimartino, Xiaojun Feng, Adriana Heguy, Megan Hogan, Emily Huang, George Jour, Christian Marier, Matthew T. Maurano, Mark J. Mulligan, Peter Meyn, Jared Pinnell, Sitharam Ramaswami, Amy Rapkiewicz, Marie Samanovic-Golden, Antonio Serrano, Guomiao Shen, Matija Snuderl, Nick Vulpescu, Gael Westby, Paul Zappile, Yutong Zhang
EPI_ISL_418974 hCoV-19/USA/NY-NYUMC28/2020 North America / USA / New York / Manhattan 2020-03-17 NYU Langone Health Department of Pathology and Medicine, New York University School of Medicine Maria Aguero-Rosenfeld, Margaret Black, John Cadley, Paolo Cotzia, John Chen, Dacia Dimartino, Xiaojun Feng, Adriana Heguy, Megan Hogan, Emily Huang, George Jour, Christian Marier, Matthew T. Maurano, Mark J. Mulligan, Peter Meyn, Jared Pinnell, Sitharam Ramaswami, Amy Rapkiewicz, Marie Samanovic-Golden, Antonio Serrano, Guomiao Shen, Matija Snuderl, Nick Vulpescu, Gael Westby, Paul Zappile, Yutong Zhang
EPI_ISL_418975 hCoV-19/USA/NY-NYUMC29/2020 North America / USA / New York / Manhattan 2020-03-17 NYU Langone Health Department of Pathology and Medicine, New York University School of Medicine Maria Aguero-Rosenfeld, Margaret Black, John Cadley, Paolo Cotzia, John Chen, Dacia Dimartino, Xiaojun Feng, Adriana Heguy, Megan Hogan, Emily Huang, George Jour, Christian Marier, Matthew T. Maurano, Mark J. Mulligan, Peter Meyn, Jared Pinnell, Sitharam Ramaswami, Amy Rapkiewicz, Marie Samanovic-Golden, Antonio Serrano, Guomiao Shen, Matija Snuderl, Nick Vulpescu, Gael Westby, Paul Zappile, Yutong Zhang
EPI_ISL_418976 hCoV-19/USA/NY-NYUMC30/2020 North America / USA / New York / Manhattan 2020-03-17 NYU Langone Health Department of Pathology and Medicine, New York University School of Medicine Maria Aguero-Rosenfeld, Margaret Black, John Cadley, Paolo Cotzia, John Chen, Dacia Dimartino, Xiaojun Feng, Adriana Heguy, Megan Hogan, Emily Huang, George Jour, Christian Marier, Matthew T. Maurano, Mark J. Mulligan, Peter Meyn, Jared Pinnell, Sitharam Ramaswami, Amy Rapkiewicz, Marie Samanovic-Golden, Antonio Serrano, Guomiao Shen, Matija Snuderl, Nick Vulpescu, Gael Westby, Paul Zappile, Yutong Zhang
EPI_ISL_418977 hCoV-19/USA/NY-NYUMC31/2020 North America / USA / New York / Manhattan 2020-03-17 NYU Langone Health Department of Pathology and Medicine, New York University School of Medicine Maria Aguero-Rosenfeld, Margaret Black, John Cadley, Paolo Cotzia, John Chen, Dacia Dimartino, Xiaojun Feng, Adriana Heguy, Megan Hogan, Emily Huang, George Jour, Christian Marier, Matthew T. Maurano, Mark J. Mulligan, Peter Meyn, Jared Pinnell, Sitharam Ramaswami, Amy Rapkiewicz, Marie Samanovic-Golden, Antonio Serrano, Guomiao Shen, Matija Snuderl, Nick Vulpescu, Gael Westby, Paul Zappile, Yutong Zhang
EPI_ISL_418978 hCoV-19/USA/NY-NYUMC32/2020 North America / USA / New York / Manhattan 2020-03-17 NYU Langone Health Department of Pathology and Medicine, New York University School of Medicine Maria Aguero-Rosenfeld, Margaret Black, John Cadley, Paolo Cotzia, John Chen, Dacia Dimartino, Xiaojun Feng, Adriana Heguy, Megan Hogan, Emily Huang, George Jour, Christian Marier, Matthew T. Maurano, Mark J. Mulligan, Peter Meyn, Jared Pinnell, Sitharam Ramaswami, Amy Rapkiewicz, Marie Samanovic-Golden, Antonio Serrano, Guomiao Shen, Matija Snuderl, Nick Vulpescu, Gael Westby, Paul Zappile, Yutong Zhang
EPI_ISL_418979 hCoV-19/USA/NY-NYUMC33/2020 North America / USA / New York / Manhattan 2020-03-17 NYU Langone Health Department of Pathology and Medicine, New York University School of Medicine Maria Aguero-Rosenfeld, Margaret Black, John Cadley, Paolo Cotzia, John Chen, Dacia Dimartino, Xiaojun Feng, Adriana Heguy, Megan Hogan, Emily Huang, George Jour, Christian Marier, Matthew T. Maurano, Mark J. Mulligan, Peter Meyn, Jared Pinnell, Sitharam Ramaswami, Amy Rapkiewicz, Marie Samanovic-Golden, Antonio Serrano, Guomiao Shen, Matija Snuderl, Nick Vulpescu, Gael Westby, Paul Zappile, Yutong Zhang
EPI_ISL_418980 hCoV-19/USA/NY-NYUMC34/2020 North America / USA / New York / Manhattan 2020-03-17 NYU Langone Health Department of Pathology and Medicine, New York University School of Medicine Maria Aguero-Rosenfeld, Margaret Black, John Cadley, Paolo Cotzia, John Chen, Dacia Dimartino, Xiaojun Feng, Adriana Heguy, Megan Hogan, Emily Huang, George Jour, Christian Marier, Matthew T. Maurano, Mark J. Mulligan, Peter Meyn, Jared Pinnell, Sitharam Ramaswami, Amy Rapkiewicz, Marie Samanovic-Golden, Antonio Serrano, Guomiao Shen, Matija Snuderl, Nick Vulpescu, Gael Westby, Paul Zappile, Yutong Zhang
EPI_ISL_418981 hCoV-19/Belgium/VRAR-030643/2020 Europe / Belgium / Merelbeke 2020-03-06 KU Leuven, Clinical and Epidemiological Virology KU Leuven, Clinical and Epidemiological Virology Bert Vanmechelen, Joan Marti-Carreras, Tony Wawina, Piet Maes
EPI_ISL_418982 hCoV-19/Belgium/HAA-030953/2020 Europe / Belgium / Oevel 2020-03-09 KU Leuven, Clinical and Epidemiological Virology KU Leuven, Clinical and Epidemiological Virology Bert Vanmechelen, Joan Marti-Carreras, Tony Wawina, Piet Maes
EPI_ISL_418983 hCoV-19/Belgium/DWAEE-030954/2020 Europe / Belgium / Asse 2020-03-09 KU Leuven, Clinical and Epidemiological Virology KU Leuven, Clinical and Epidemiological Virology Bert Vanmechelen, Joan Marti-Carreras, Tony Wawina, Piet Maes
EPI_ISL_418984 hCoV-19/Belgium/BG-030955/2020 Europe / Belgium / Kaulile 2020-03-09 KU Leuven, Clinical and Epidemiological Virology KU Leuven, Clinical and Epidemiological Virology Bert Vanmechelen, Joan Marti-Carreras, Tony Wawina, Piet Maes
EPI_ISL_418985 hCoV-19/Belgium/LT-030956/2020 Europe / Belgium / Ellezelles 2020-03-09 KU Leuven, Clinical and Epidemiological Virology KU Leuven, Clinical and Epidemiological Virology Bert Vanmechelen, Joan Marti-Carreras, Tony Wawina, Piet Maes
EPI_ISL_418986 hCoV-19/Belgium/RS-030257/2020 Europe / Belgium / Hoegaarden 2020-03-02 KU Leuven, Clinical and Epidemiological Virology KU Leuven, Clinical and Epidemiological Virology Bert Vanmechelen, Joan Marti-Carreras, Tony Wawina, Piet Maes
EPI_ISL_418987 hCoV-19/Belgium/CG-030158/2020 Europe / Belgium / Eupen 2020-03-01 KU Leuven, Clinical and Epidemiological Virology KU Leuven, Clinical and Epidemiological Virology Bert Vanmechelen, Joan Marti-Carreras, Tony Wawina, Piet Maes
EPI_ISL_418988 hCoV-19/Belgium/JRH-030459/2020 Europe / Belgium / Zolder 2020-03-04 Institute information KU Leuven, Clinical and Epidemiological Virology Institute information KU Leuven, Clinical and Epidemiological Virology Bert Vanmechelen, Joan Marti-Carreras, Tony Wawina, Piet Maes

EPI_ISL_418989 hCoV-19/Belgium/RT-030460/2020 Europe / Belgium / Vorst 2020-03-04 KU Leuven, Clinical and Epidemiological Virology KU Leuven, Clinical and Epidemiological Virology Bert Vanmechelen, Joan Marti-Carreras, Tony Wawina, Piet Maes
EPI_ISL_418990 hCoV-19/Hangzhou/ZJU-010/2020 Asia / China / Hangzhou 2020-02-03 State Key Laboratory for Diagnosis and Treatment of Infectious Diseases, National Clinical Research Center for Infectious Diseases, First Affiliated Hospital, Zhejiang University School of Medicine, Hangzhou, China 310003 State Key Laboratory for Diagnosis and Treatment of Infectious Diseases, National Clinical Research Center for Infectious Diseases, First Affiliated Hospital, Zhejiang University School of Medicine, Hangzhou, China 310003 Hangping Yao, Nanping Wu, Chao Jiang, Xiangyun Lu, Linfang Cheng, Fumin Liu, Zhigang Wu, Haibo Wu, Changzhong Jin, Min Zheng, Lanjuan Li
EPI_ISL_418991 hCoV-19/Hangzhou/ZJU-011/2020 Asia / China / Hangzhou 2020-02-04 State Key Laboratory for Diagnosis and Treatment of Infectious Diseases, National Clinical Research Center for Infectious Diseases, First Affiliated Hospital, Zhejiang University School of Medicine, Hangzhou, China 310003 State Key Laboratory for Diagnosis and Treatment of Infectious Diseases, National Clinical Research Center for Infectious Diseases, First Affiliated Hospital, Zhejiang University School of Medicine, Hangzhou, China 310003 Hangping Yao, Nanping Wu, Chao Jiang, Xiangyun Lu, Linfang Cheng, Fumin Liu, Zhigang Wu, Haibo Wu, Changzhong Jin, Min Zheng, Lanjuan Li
EPI_ISL_418992 hCoV-19/Singapore/17/2020 Asia / Singapore 2020-02-10 National Public Health Laboratory, National Centre for Infectious Diseases National Public Health Laboratory, National Centre for Infectious Diseases Mak TM, Octavia S, Cui L, Lin RTP
EPI_ISL_418993 hCoV-19/Singapore/20/2020 Asia / Singapore 2020-01-29 National Public Health Laboratory, National Centre for Infectious Diseases National Public Health Laboratory, National Centre for Infectious Diseases Mak TM, Octavia S, Cui L, Lin RTP
EPI_ISL_418994 hCoV-19/Singapore/3Clin/2020 Asia / Singapore 2020-01-29 National Public Health Laboratory, National Centre for Infectious Diseases National Public Health Laboratory, National Centre for Infectious Diseases Mak TM, Octavia S, Cui L, Lin RTP
EPI_ISL_418995 hCoV-19/Singapore/12Clin/2020 Asia / Singapore 2020-02-28 National Public Health Laboratory, National Centre for Infectious Diseases National Public Health Laboratory, National Centre for Infectious Diseases Mak TM, Octavia S, Cui L, Lin RTP
EPI_ISL_418996 hCoV-19/Singapore/15/2020 Asia / Singapore 2020-01-27 National Public Health Laboratory, National Centre for Infectious Diseases National Public Health Laboratory, National Centre for Infectious Diseases Mak TM, Octavia S, Cui L, Lin RTP
EPI_ISL_418997 hCoV-19/Singapore/16/2020 Asia / Singapore 2020-02-06 National Public Health Laboratory, National Centre for Infectious Diseases National Public Health Laboratory, National Centre for Infectious Diseases Mak TM, Octavia S, Cui L, Lin RTP
EPI_ISL_418998 hCoV-19/Singapore/14Clin/2020 Asia / Singapore 2020-02-14 National Public Health Laboratory, National Centre for Infectious Diseases National Public Health Laboratory, National Centre for Infectious Diseases Mak TM, Octavia S, Cui L, Lin RTP
EPI_ISL_418999 hCoV-19/Singapore/18/2020 Asia / Singapore 2020-03-01 National Public Health Laboratory, National Centre for Infectious Diseases National Public Health Laboratory, National Centre for Infectious Diseases Mak TM, Octavia S, Cui L, Lin RTP
EPI_ISL_422001 hCoV-19/England/20148004904/2020 Europe / United Kingdom / England 2020-04-03 Respiratory Virus Unit, Microbiology Services Colindale, Public Health England Respiratory Virus Unit, Microbiology Services Colindale, Public Health England Monica Galiano, Shahjahan Miah, Angie Lackenby, Omolola Akinbami, Tiina Talts, Leena Bhaw, Richard Myers, Steven Platt, Kirstin Edwards, Jonathan Hubb, Joanna Ellis, Maria Zambon
EPI_ISL_422002 hCoV-19/England/20148006104/2020 Europe / United Kingdom / England 2020-03-30 Respiratory Virus Unit, Microbiology Services Colindale, Public Health England Respiratory Virus Unit, Microbiology Services Colindale, Public Health England Monica Galiano, Shahjahan Miah, Angie Lackenby, Omolola Akinbami, Tiina Talts, Leena Bhaw, Richard Myers, Steven Platt, Kirstin Edwards, Jonathan Hubb, Joanna Ellis, Maria Zambon
EPI_ISL_422009 hCoV-19/England/20148039404/2020 Europe / United Kingdom / England 2020-04-02 Respiratory Virus Unit, Microbiology Services Colindale, Public Health England Respiratory Virus Unit, Microbiology Services Colindale, Public Health England Monica Galiano, Shahjahan Miah, Angie Lackenby, Omolola Akinbami, Tiina Talts, Leena Bhaw, Richard Myers, Steven Platt, Kirstin Edwards, Jonathan Hubb, Joanna Ellis, Maria Zambon
EPI_ISL_422010 hCoV-19/England/20148041604/2020 Europe / United Kingdom / England 2020-04-02 Respiratory Virus Unit, Microbiology Services Colindale, Public Health England Respiratory Virus Unit, Microbiology Services Colindale, Public Health England Monica Galiano, Shahjahan Miah, Angie Lackenby, Omolola Akinbami, Tiina Talts, Leena Bhaw, Richard Myers, Steven Platt, Kirstin Edwards, Jonathan Hubb, Joanna Ellis, Maria Zambon
EPI_ISL_422016 hCoV-19/Wales/PHWC-26796/2020 Europe / United Kingdom / Wales 2020-03-28 Wales Specialist Virology Centre Public Health Wales Microbiology Cardiff Catherine Moore, Johnathan Evans, Malorie Perry, Simon Cottrell, Alec Birchley, Alexander Adams, Amy Gaskin, Bree Gatica-Wilcox, Jason Coombes, Lauren Gilbert, Lee Graham, Nicole Pacchiarini, Sara Kumziene-Summerhayes, Sarah Taylor, Sophie Jones, Sara Rey, Matthew Bull, Joanne Watkins, Sally Corden, Tom Connor
EPI_ISL_422017 hCoV-19/Wales/PHWC-2711F/2020 Europe / United Kingdom / Wales 2020-03-29 Wales Specialist Virology Centre Public Health Wales Microbiology Cardiff Catherine Moore, Johnathan Evans, Malorie Perry, Simon Cottrell, Alec Birchley, Alexander Adams, Amy Gaskin, Bree Gatica-Wilcox, Jason Coombes, Lauren Gilbert, Lee Graham, Nicole Pacchiarini, Sara Kumziene-Summerhayes, Sarah Taylor, Sophie Jones,

Sara Rey, Matthew Bull, Joanne Watkins, Sally Corden, Tom Connor
EPI_ISL_422018 hCoV-19/Wales/PHWC-2673C/2020 Europe / United Kingdom / Wales 2020-03-28 Wales Specialist Virology Centre Public Health Wales Microbiology Cardiff Catherine Moore, Johnathan Evans, Malorie Perry, Simon Cottrell, Alec Birchley, Alexander Adams, Amy Gaskin, Bree Gatica-Wilcox, Jason Coombes, Lauren Gilbert, Lee Graham, Nicole Pacchiarini, Sara Kumziene-Summerhayes, Sarah Taylor, Sophie Jones, Sara Rey, Matthew Bull, Joanne Watkins, Sally Corden, Tom Connor
EPI_ISL_422019 hCoV-19/Wales/PHWC-265E7/2020 Europe / United Kingdom / Wales 2020-03-27 Wales Specialist Virology Centre Public Health Wales Microbiology Cardiff Catherine Moore, Johnathan Evans, Malorie Perry, Simon Cottrell, Alec Birchley, Alexander Adams, Amy Gaskin, Bree Gatica-Wilcox, Jason Coombes, Lauren Gilbert, Lee Graham, Nicole Pacchiarini, Sara Kumziene-Summerhayes, Sarah Taylor, Sophie Jones, Sara Rey, Matthew Bull, Joanne Watkins, Sally Corden, Tom Connor
EPI_ISL_422020 hCoV-19/Wales/PHWC-2682A/2020 Europe / United Kingdom / Wales 2020-03-27 Wales Specialist Virology Centre Public Health Wales Microbiology Cardiff Catherine Moore, Johnathan Evans, Malorie Perry, Simon Cottrell, Alec Birchley, Alexander Adams, Amy Gaskin, Bree Gatica-Wilcox, Jason Coombes, Lauren Gilbert, Lee Graham, Nicole Pacchiarini, Sara Kumziene-Summerhayes, Sarah Taylor, Sophie Jones, Sara Rey, Matthew Bull, Joanne Watkins, Sally Corden, Tom Connor
EPI_ISL_422021 hCoV-19/Wales/PHWC-2665D/2020 Europe / United Kingdom / Wales 2020-03-27 Wales Specialist Virology Centre Public Health Wales Microbiology Cardiff Catherine Moore, Johnathan Evans, Malorie Perry, Simon Cottrell, Alec Birchley, Alexander Adams, Amy Gaskin, Bree Gatica-Wilcox, Jason Coombes, Lauren Gilbert, Lee Graham, Nicole Pacchiarini, Sara Kumziene-Summerhayes, Sarah Taylor, Sophie Jones, Sara Rey, Matthew Bull, Joanne Watkins, Sally Corden, Tom Connor
EPI_ISL_422022 hCoV-19/Wales/PHWC-26D49/2020 Europe / United Kingdom / Wales 2020-03-30 Wales Specialist Virology Centre Public Health Wales Microbiology Cardiff Catherine Moore, Johnathan Evans, Malorie Perry, Simon Cottrell, Alec Birchley, Alexander Adams, Amy Gaskin, Bree Gatica-Wilcox, Jason Coombes, Lauren Gilbert, Lee Graham, Nicole Pacchiarini, Sara Kumziene-Summerhayes, Sarah Taylor, Sophie Jones, Sara Rey, Matthew Bull, Joanne Watkins, Sally Corden, Tom Connor
EPI_ISL_422023 hCoV-19/Wales/PHWC-26981/2020 Europe / United Kingdom / Wales 2020-03-30 Wales Specialist Virology Centre Public Health Wales Microbiology Cardiff Catherine Moore, Johnathan Evans, Malorie Perry, Simon Cottrell, Alec Birchley, Alexander Adams, Amy Gaskin, Bree Gatica-Wilcox, Jason Coombes, Lauren Gilbert, Lee Graham, Nicole Pacchiarini, Sara Kumziene-Summerhayes, Sarah Taylor, Sophie Jones, Sara Rey, Matthew Bull, Joanne Watkins, Sally Corden, Tom Connor
EPI_ISL_422024 hCoV-19/Wales/PHWC-26B12/2020 Europe / United Kingdom / Wales 2020-03-29 Wales Specialist Virology Centre Public Health Wales Microbiology Cardiff Catherine Moore, Johnathan Evans, Malorie Perry, Simon Cottrell, Alec Birchley, Alexander Adams, Amy Gaskin, Bree Gatica-Wilcox, Jason Coombes, Lauren Gilbert, Lee Graham, Nicole Pacchiarini, Sara Kumziene-Summerhayes, Sarah Taylor, Sophie Jones, Sara Rey, Matthew Bull, Joanne Watkins, Sally Corden, Tom Connor
EPI_ISL_422025 hCoV-19/Wales/PHWC-263DE/2020 Europe / United Kingdom / Wales 2020-03-27 Wales Specialist Virology Centre Public Health Wales Microbiology Cardiff Catherine Moore, Johnathan Evans, Malorie Perry, Simon Cottrell, Alec Birchley, Alexander Adams, Amy Gaskin, Bree Gatica-Wilcox, Jason Coombes, Lauren Gilbert, Lee Graham, Nicole Pacchiarini, Sara Kumziene-Summerhayes, Sarah Taylor, Sophie Jones, Sara Rey, Matthew Bull, Joanne Watkins, Sally Corden, Tom Connor
EPI_ISL_422026 hCoV-19/Wales/PHWC-263FC/2020 Europe / United Kingdom / Wales 2020-03-27 Wales Specialist Virology Centre Public Health Wales Microbiology Cardiff Catherine Moore, Johnathan Evans, Malorie Perry, Simon Cottrell, Alec Birchley, Alexander Adams, Amy Gaskin, Bree Gatica-Wilcox, Jason Coombes, Lauren Gilbert, Lee Graham, Nicole Pacchiarini, Sara Kumziene-Summerhayes, Sarah Taylor, Sophie Jones, Sara Rey, Matthew Bull, Joanne Watkins, Sally Corden, Tom Connor
EPI_ISL_422027 hCoV-19/Wales/PHWC-2672D/2020 Europe / United Kingdom / Wales 2020-03-27 Wales Specialist Virology Centre Public Health Wales Microbiology Cardiff Catherine Moore, Johnathan Evans, Malorie Perry, Simon Cottrell, Alec Birchley, Alexander Adams, Amy Gaskin, Bree Gatica-Wilcox, Jason Coombes, Lauren Gilbert, Lee Graham, Nicole Pacchiarini, Sara Kumziene-Summerhayes, Sarah Taylor, Sophie Jones, Sara Rey, Matthew Bull, Joanne Watkins, Sally Corden, Tom Connor
EPI_ISL_422028 hCoV-19/Wales/PHWC-2631A/2020 Europe / United Kingdom / Wales 2020-03-27 Wales Specialist Virology Centre Public Health Wales Microbiology Cardiff Catherine Moore, Johnathan Evans, Malorie Perry, Simon Cottrell, Alec Birchley, Alexander Adams, Amy Gaskin, Bree Gatica-Wilcox, Jason Coombes, Lauren Gilbert, Lee Graham, Nicole Pacchiarini, Sara Kumziene-Summerhayes, Sarah Taylor, Sophie Jones, Sara Rey, Matthew Bull, Joanne Watkins, Sally Corden, Tom Connor
EPI_ISL_422029 hCoV-19/Wales/PHWC-25F44/2020 Europe / United Kingdom / Wales 2020-03-25 Wales Specialist Virology Centre Public Health Wales Microbiology Cardiff Catherine Moore, Johnathan Evans, Malorie Perry, Simon Cottrell, Alec Birchley, Alexander Adams, Amy Gaskin, Bree Gatica-Wilcox, Jason Coombes, Lauren Gilbert, Lee Graham, Nicole Pacchiarini, Sara Kumziene-Summerhayes, Sarah Taylor, Sophie Jones, Sara Rey, Matthew Bull, Joanne Watkins, Sally Corden, Tom Connor
EPI_ISL_422030 hCoV-19/Wales/PHWC-270B8/2020 Europe / United Kingdom / Wales 2020-03-29 Wales Specialist Virology Centre Public Health Wales Microbiology Cardiff Catherine Moore, Johnathan Evans, Malorie Perry, Simon Cottrell, Alec Birchley, Alexander Adams, Amy Gaskin, Bree Gatica-Wilcox, Jason Coombes, Lauren Gilbert, Lee Graham, Nicole Pacchiarini, Sara Kumziene-Summerhayes, Sarah Taylor, Sophie Jones, Sara Rey, Matthew Bull, Joanne Watkins, Sally Corden, Tom Connor
EPI_ISL_422031 hCoV-19/Wales/PHWC-26DD0/2020 Europe / United Kingdom / Wales 2020-03-30 Wales Specialist Virology Centre Public Health Wales Microbiology Cardiff Catherine Moore, Johnathan Evans, Malorie Perry, Simon Cottrell, Alec Birchley, Alexander Adams, Amy Gaskin, Bree Gatica-Wilcox, Jason Coombes, Lauren Gilbert, Lee Graham, Nicole Pacchiarini, Sara Kumziene-Summerhayes, Sarah Taylor, Sophie Jones,

Sara Rey, Matthew Bull, Joanne Watkins, Sally Corden, Tom Connor
EPI_ISL_422032 hCoV-19/Wales/PHWC-2649F/2020 Europe / United Kingdom / Wales 2020-03-27 Wales Specialist Virology Centre Public Health Wales Microbiology Cardiff Catherine Moore, Johnathan Evans, Malorie Perry, Simon Cottrell, Alec Birchley, Alexander Adams, Amy Gaskin, Bree Gatica-Wilcox, Jason Coombes, Lauren Gilbert, Lee Graham, Nicole Pacchiarini, Sara Kumziene-Summerhayes, Sarah Taylor, Sophie Jones, Sara Rey, Matthew Bull, Joanne Watkins, Sally Corden, Tom Connor
EPI_ISL_422033 hCoV-19/Wales/PHWC-2681B/2020 Europe / United Kingdom / Wales 2020-03-27 Wales Specialist Virology Centre Public Health Wales Microbiology Cardiff Catherine Moore, Johnathan Evans, Malorie Perry, Simon Cottrell, Alec Birchley, Alexander Adams, Amy Gaskin, Bree Gatica-Wilcox, Jason Coombes, Lauren Gilbert, Lee Graham, Nicole Pacchiarini, Sara Kumziene-Summerhayes, Sarah Taylor, Sophie Jones, Sara Rey, Matthew Bull, Joanne Watkins, Sally Corden, Tom Connor
EPI_ISL_422034 hCoV-19/Wales/PHWC-27012/2020 Europe / United Kingdom / Wales 2020-03-28 Wales Specialist Virology Centre Public Health Wales Microbiology Cardiff Catherine Moore, Johnathan Evans, Malorie Perry, Simon Cottrell, Alec Birchley, Alexander Adams, Amy Gaskin, Bree Gatica-Wilcox, Jason Coombes, Lauren Gilbert, Lee Graham, Nicole Pacchiarini, Sara Kumziene-Summerhayes, Sarah Taylor, Sophie Jones, Sara Rey, Matthew Bull, Joanne Watkins, Sally Corden, Tom Connor
EPI_ISL_422035 hCoV-19/Wales/PHWC-26D58/2020 Europe / United Kingdom / Wales 2020-03-30 Wales Specialist Virology Centre Public Health Wales Microbiology Cardiff Catherine Moore, Johnathan Evans, Malorie Perry, Simon Cottrell, Alec Birchley, Alexander Adams, Amy Gaskin, Bree Gatica-Wilcox, Jason Coombes, Lauren Gilbert, Lee Graham, Nicole Pacchiarini, Sara Kumziene-Summerhayes, Sarah Taylor, Sophie Jones, Sara Rey, Matthew Bull, Joanne Watkins, Sally Corden, Tom Connor
EPI_ISL_422036 hCoV-19/Wales/PHWC-26A15/2020 Europe / United Kingdom / Wales 2020-03-29 Wales Specialist Virology Centre Public Health Wales Microbiology Cardiff Catherine Moore, Johnathan Evans, Malorie Perry, Simon Cottrell, Alec Birchley, Alexander Adams, Amy Gaskin, Bree Gatica-Wilcox, Jason Coombes, Lauren Gilbert, Lee Graham, Nicole Pacchiarini, Sara Kumziene-Summerhayes, Sarah Taylor, Sophie Jones, Sara Rey, Matthew Bull, Joanne Watkins, Sally Corden, Tom Connor
EPI_ISL_422037 hCoV-19/Wales/PHWC-2663F/2020 Europe / United Kingdom / Wales 2020-03-27 Wales Specialist Virology Centre Public Health Wales Microbiology Cardiff Catherine Moore, Johnathan Evans, Malorie Perry, Simon Cottrell, Alec Birchley, Alexander Adams, Amy Gaskin, Bree Gatica-Wilcox, Jason Coombes, Lauren Gilbert, Lee Graham, Nicole Pacchiarini, Sara Kumziene-Summerhayes, Sarah Taylor, Sophie Jones, Sara Rey, Matthew Bull, Joanne Watkins, Sally Corden, Tom Connor
EPI_ISL_422038 hCoV-19/Wales/PHWC-2621D/2020 Europe / United Kingdom / Wales 2020-03-26 Wales Specialist Virology Centre Public Health Wales Microbiology Cardiff Catherine Moore, Johnathan Evans, Malorie Perry, Simon Cottrell, Alec Birchley, Alexander Adams, Amy Gaskin, Bree Gatica-Wilcox, Jason Coombes, Lauren Gilbert, Lee Graham, Nicole Pacchiarini, Sara Kumziene-Summerhayes, Sarah Taylor, Sophie Jones, Sara Rey, Matthew Bull, Joanne Watkins, Sally Corden, Tom Connor
EPI_ISL_422100 hCoV-19/Wales/PHWC-260B9/2020 Europe / United Kingdom / Wales 2020-03-25 Wales Specialist Virology Centre Public Health Wales Microbiology Cardiff Catherine Moore, Johnathan Evans, Malorie Perry, Simon Cottrell, Alec Birchley, Alexander Adams, Amy Gaskin, Bree Gatica-Wilcox, Jason Coombes, Lauren Gilbert, Lee Graham, Nicole Pacchiarini, Sara Kumziene-Summerhayes, Sarah Taylor, Sophie Jones, Sara Rey, Matthew Bull, Joanne Watkins, Sally Corden, Tom Connor
EPI_ISL_422102 hCoV-19/Wales/PHWC-26444/2020 Europe / United Kingdom / Wales 2020-03-27 Wales Specialist Virology Centre Public Health Wales Microbiology Cardiff Catherine Moore, Johnathan Evans, Malorie Perry, Simon Cottrell, Alec Birchley, Alexander Adams, Amy Gaskin, Bree Gatica-Wilcox, Jason Coombes, Lauren Gilbert, Lee Graham, Nicole Pacchiarini, Sara Kumziene-Summerhayes, Sarah Taylor, Sophie Jones, Sara Rey, Matthew Bull, Joanne Watkins, Sally Corden, Tom Connor
EPI_ISL_422103 hCoV-19/Wales/PHWC-26C00/2020 Europe / United Kingdom / Wales 2020-03-29 Wales Specialist Virology Centre Public Health Wales Microbiology Cardiff Catherine Moore, Johnathan Evans, Malorie Perry, Simon Cottrell, Alec Birchley, Alexander Adams, Amy Gaskin, Bree Gatica-Wilcox, Jason Coombes, Lauren Gilbert, Lee Graham, Nicole Pacchiarini, Sara Kumziene-Summerhayes, Sarah Taylor, Sophie Jones, Sara Rey, Matthew Bull, Joanne Watkins, Sally Corden, Tom Connor
EPI_ISL_422104 hCoV-19/Wales/PHWC-26022/2020 Europe / United Kingdom / Wales 2020-03-26 Wales Specialist Virology Centre Public Health Wales Microbiology Cardiff Catherine Moore, Johnathan Evans, Malorie Perry, Simon Cottrell, Alec Birchley, Alexander Adams, Amy Gaskin, Bree Gatica-Wilcox, Jason Coombes, Lauren Gilbert, Lee Graham, Nicole Pacchiarini, Sara Kumziene-Summerhayes, Sarah Taylor, Sophie Jones, Sara Rey, Matthew Bull, Joanne Watkins, Sally Corden, Tom Connor
EPI_ISL_422105 hCoV-19/Wales/PHWC-26F25/2020 Europe / United Kingdom / Wales 2020-03-31 Wales Specialist Virology Centre Public Health Wales Microbiology Cardiff Catherine Moore, Johnathan Evans, Malorie Perry, Simon Cottrell, Alec Birchley, Alexander Adams, Amy Gaskin, Bree Gatica-Wilcox, Jason Coombes, Lauren Gilbert, Lee Graham, Nicole Pacchiarini, Sara Kumziene-Summerhayes, Sarah Taylor, Sophie Jones, Sara Rey, Matthew Bull, Joanne Watkins, Sally Corden, Tom Connor
EPI_ISL_422106 hCoV-19/Wales/PHWC-260F5/2020 Europe / United Kingdom / Wales 2020-03-25 Wales Specialist Virology Centre Public Health Wales Microbiology Cardiff Catherine Moore, Johnathan Evans, Malorie Perry, Simon Cottrell, Alec Birchley, Alexander Adams, Amy Gaskin, Bree Gatica-Wilcox, Jason Coombes, Lauren Gilbert, Lee Graham, Nicole Pacchiarini, Sara Kumziene-Summerhayes, Sarah Taylor, Sophie Jones, Sara Rey, Matthew Bull, Joanne Watkins, Sally Corden, Tom Connor
EPI_ISL_422107 hCoV-19/Wales/PHWC-25FF9/2020 Europe / United Kingdom / Wales 2020-03-25 Wales Specialist Virology Centre Public Health Wales Microbiology Cardiff Catherine Moore, Johnathan Evans, Malorie Perry, Simon Cottrell, Alec Birchley, Alexander Adams, Amy Gaskin, Bree Gatica-Wilcox, Jason Coombes, Lauren Gilbert, Lee Graham, Nicole Pacchiarini, Sara Kumziene-Summerhayes, Sarah Taylor, Sophie Jones,

Sara Rey, Matthew Bull, Joanne Watkins, Sally Corden, Tom Connor
EPI_ISL_422108 hCoV-19/Wales/PHWC-2581C/2020 Europe / United Kingdom / Wales 2020-03-25 Wales Specialist Virology Centre Public Health Wales Microbiology Cardiff Catherine Moore, Johnathan Evans, Malorie Perry, Simon Cottrell, Alec Birchley, Alexander Adams, Amy Gaskin, Bree Gatica-Wilcox, Jason Coombes, Lauren Gilbert, Lee Graham, Nicole Pacchiarini, Sara Kumziene-Summerhayes, Sarah Taylor, Sophie Jones, Sara Rey, Matthew Bull, Joanne Watkins, Sally Corden, Tom Connor
EPI_ISL_422109 hCoV-19/Wales/PHWC-266F3/2020 Europe / United Kingdom / Wales 2020-03-27 Wales Specialist Virology Centre Public Health Wales Microbiology Cardiff Catherine Moore, Johnathan Evans, Malorie Perry, Simon Cottrell, Alec Birchley, Alexander Adams, Amy Gaskin, Bree Gatica-Wilcox, Jason Coombes, Lauren Gilbert, Lee Graham, Nicole Pacchiarini, Sara Kumziene-Summerhayes, Sarah Taylor, Sophie Jones, Sara Rey, Matthew Bull, Joanne Watkins, Sally Corden, Tom Connor
EPI_ISL_422110 hCoV-19/Wales/PHWC-260D7/2020 Europe / United Kingdom / Wales 2020-03-25 Wales Specialist Virology Centre Public Health Wales Microbiology Cardiff Catherine Moore, Johnathan Evans, Malorie Perry, Simon Cottrell, Alec Birchley, Alexander Adams, Amy Gaskin, Bree Gatica-Wilcox, Jason Coombes, Lauren Gilbert, Lee Graham, Nicole Pacchiarini, Sara Kumziene-Summerhayes, Sarah Taylor, Sophie Jones, Sara Rey, Matthew Bull, Joanne Watkins, Sally Corden, Tom Connor
EPI_ISL_422111 hCoV-19/Wales/PHWC-260C8/2020 Europe / United Kingdom / Wales 2020-03-26 Wales Specialist Virology Centre Public Health Wales Microbiology Cardiff Catherine Moore, Johnathan Evans, Malorie Perry, Simon Cottrell, Alec Birchley, Alexander Adams, Amy Gaskin, Bree Gatica-Wilcox, Jason Coombes, Lauren Gilbert, Lee Graham, Nicole Pacchiarini, Sara Kumziene-Summerhayes, Sarah Taylor, Sophie Jones, Sara Rey, Matthew Bull, Joanne Watkins, Sally Corden, Tom Connor
EPI_ISL_422112 hCoV-19/Wales/PHWC-26417/2020 Europe / United Kingdom / Wales 2020-03-27 Wales Specialist Virology Centre Public Health Wales Microbiology Cardiff Catherine Moore, Johnathan Evans, Malorie Perry, Simon Cottrell, Alec Birchley, Alexander Adams, Amy Gaskin, Bree Gatica-Wilcox, Jason Coombes, Lauren Gilbert, Lee Graham, Nicole Pacchiarini, Sara Kumziene-Summerhayes, Sarah Taylor, Sophie Jones, Sara Rey, Matthew Bull, Joanne Watkins, Sally Corden, Tom Connor
EPI_ISL_422113 hCoV-19/Wales/PHWC-26CB5/2020 Europe / United Kingdom / Wales 2020-03-28 Wales Specialist Virology Centre Public Health Wales Microbiology Cardiff Catherine Moore, Johnathan Evans, Malorie Perry, Simon Cottrell, Alec Birchley, Alexander Adams, Amy Gaskin, Bree Gatica-Wilcox, Jason Coombes, Lauren Gilbert, Lee Graham, Nicole Pacchiarini, Sara Kumziene-Summerhayes, Sarah Taylor, Sophie Jones, Sara Rey, Matthew Bull, Joanne Watkins, Sally Corden, Tom Connor
EPI_ISL_422114 hCoV-19/Wales/PHWC-25BC8/2020 Europe / United Kingdom / Wales 2020-03-25 Wales Specialist Virology Centre Public Health Wales Microbiology Cardiff Catherine Moore, Johnathan Evans, Malorie Perry, Simon Cottrell, Alec Birchley, Alexander Adams, Amy Gaskin, Bree Gatica-Wilcox, Jason Coombes, Lauren Gilbert, Lee Graham, Nicole Pacchiarini, Sara Kumziene-Summerhayes, Sarah Taylor, Sophie Jones, Sara Rey, Matthew Bull, Joanne Watkins, Sally Corden, Tom Connor
EPI_ISL_422115 hCoV-19/Wales/PHWC-26EDD/2020 Europe / United Kingdom / Wales 2020-03-30 Wales Specialist Virology Centre Public Health Wales Microbiology Cardiff Catherine Moore, Johnathan Evans, Malorie Perry, Simon Cottrell, Alec Birchley, Alexander Adams, Amy Gaskin, Bree Gatica-Wilcox, Jason Coombes, Lauren Gilbert, Lee Graham, Nicole Pacchiarini, Sara Kumziene-Summerhayes, Sarah Taylor, Sophie Jones, Sara Rey, Matthew Bull, Joanne Watkins, Sally Corden, Tom Connor
EPI_ISL_422116 hCoV-19/Wales/PHWC-25B04/2020 Europe / United Kingdom / Wales 2020-03-24 Wales Specialist Virology Centre Public Health Wales Microbiology Cardiff Catherine Moore, Johnathan Evans, Malorie Perry, Simon Cottrell, Alec Birchley, Alexander Adams, Amy Gaskin, Bree Gatica-Wilcox, Jason Coombes, Lauren Gilbert, Lee Graham, Nicole Pacchiarini, Sara Kumziene-Summerhayes, Sarah Taylor, Sophie Jones, Sara Rey, Matthew Bull, Joanne Watkins, Sally Corden, Tom Connor
EPI_ISL_422117 hCoV-19/Wales/PHWC-25F71/2020 Europe / United Kingdom / Wales 2020-03-25 Wales Specialist Virology Centre Public Health Wales Microbiology Cardiff Catherine Moore, Johnathan Evans, Malorie Perry, Simon Cottrell, Alec Birchley, Alexander Adams, Amy Gaskin, Bree Gatica-Wilcox, Jason Coombes, Lauren Gilbert, Lee Graham, Nicole Pacchiarini, Sara Kumziene-Summerhayes, Sarah Taylor, Sophie Jones, Sara Rey, Matthew Bull, Joanne Watkins, Sally Corden, Tom Connor
EPI_ISL_422118 hCoV-19/Wales/PHWC-25F9F/2020 Europe / United Kingdom / Wales 2020-03-25 Wales Specialist Virology Centre Public Health Wales Microbiology Cardiff Catherine Moore, Johnathan Evans, Malorie Perry, Simon Cottrell, Alec Birchley, Alexander Adams, Amy Gaskin, Bree Gatica-Wilcox, Jason Coombes, Lauren Gilbert, Lee Graham, Nicole Pacchiarini, Sara Kumziene-Summerhayes, Sarah Taylor, Sophie Jones, Sara Rey, Matthew Bull, Joanne Watkins, Sally Corden, Tom Connor
EPI_ISL_422119 hCoV-19/Wales/PHWC-26C5B/2020 Europe / United Kingdom / Wales 2020-03-27 Wales Specialist Virology Centre Public Health Wales Microbiology Cardiff Catherine Moore, Johnathan Evans, Malorie Perry, Simon Cottrell, Alec Birchley, Alexander Adams, Amy Gaskin, Bree Gatica-Wilcox, Jason Coombes, Lauren Gilbert, Lee Graham, Nicole Pacchiarini, Sara Kumziene-Summerhayes, Sarah Taylor, Sophie Jones, Sara Rey, Matthew Bull, Joanne Watkins, Sally Corden, Tom Connor
EPI_ISL_422120 hCoV-19/Wales/PHWC-26FBC/2020 Europe / United Kingdom / Wales 2020-03-30 Wales Specialist Virology Centre Public Health Wales Microbiology Cardiff Catherine Moore, Johnathan Evans, Malorie Perry, Simon Cottrell, Alec Birchley, Alexander Adams, Amy Gaskin, Bree Gatica-Wilcox, Jason Coombes, Lauren Gilbert, Lee Graham, Nicole Pacchiarini, Sara Kumziene-Summerhayes, Sarah Taylor, Sophie Jones, Sara Rey, Matthew Bull, Joanne Watkins, Sally Corden, Tom Connor
EPI_ISL_422121 hCoV-19/Wales/PHWC-26C97/2020 Europe / United Kingdom / Wales 2020-03-28 Wales Specialist Virology Centre Public Health Wales Microbiology Cardiff Catherine Moore, Johnathan Evans, Malorie Perry, Simon Cottrell, Alec Birchley, Alexander Adams, Amy Gaskin, Bree Gatica-Wilcox, Jason Coombes, Lauren Gilbert, Lee Graham, Nicole Pacchiarini, Sara Kumziene-Summerhayes, Sarah Taylor, Sophie Jones,

Sara Rey, Matthew Bull, Joanne Watkins, Sally Corden, Tom Connor
EPI_ISL_422122 hCoV-19/Wales/PHWC-26E73/2020 Europe / United Kingdom / Wales 2020-03-30 Wales Specialist Virology Centre Public Health Wales Microbiology Cardiff Catherine Moore, Johnathan Evans, Malorie Perry, Simon Cottrell, Alec Birchley, Alexander Adams, Amy Gaskin, Bree Gatica-Wilcox, Jason Coombes, Lauren Gilbert, Lee Graham, Nicole Pacchiarini, Sara Kumziene-Summerhayes, Sarah Taylor, Sophie Jones, Sara Rey, Matthew Bull, Joanne Watkins, Sally Corden, Tom Connor
EPI_ISL_422123 hCoV-19/Wales/PHWC-26FCB/2020 Europe / United Kingdom / Wales 2020-03-30 Wales Specialist Virology Centre Public Health Wales Microbiology Cardiff Catherine Moore, Johnathan Evans, Malorie Perry, Simon Cottrell, Alec Birchley, Alexander Adams, Amy Gaskin, Bree Gatica-Wilcox, Jason Coombes, Lauren Gilbert, Lee Graham, Nicole Pacchiarini, Sara Kumziene-Summerhayes, Sarah Taylor, Sophie Jones, Sara Rey, Matthew Bull, Joanne Watkins, Sally Corden, Tom Connor
EPI_ISL_422124 hCoV-19/Wales/PHWC-2715B/2020 Europe / United Kingdom / Wales 2020-03-29 Wales Specialist Virology Centre Public Health Wales Microbiology Cardiff Catherine Moore, Johnathan Evans, Malorie Perry, Simon Cottrell, Alec Birchley, Alexander Adams, Amy Gaskin, Bree Gatica-Wilcox, Jason Coombes, Lauren Gilbert, Lee Graham, Nicole Pacchiarini, Sara Kumziene-Summerhayes, Sarah Taylor, Sophie Jones, Sara Rey, Matthew Bull, Joanne Watkins, Sally Corden, Tom Connor
EPI_ISL_422125 hCoV-19/Wales/PHWC-27003/2020 Europe / United Kingdom / Wales 2020-03-28 Wales Specialist Virology Centre Public Health Wales Microbiology Cardiff Catherine Moore, Johnathan Evans, Malorie Perry, Simon Cottrell, Alec Birchley, Alexander Adams, Amy Gaskin, Bree Gatica-Wilcox, Jason Coombes, Lauren Gilbert, Lee Graham, Nicole Pacchiarini, Sara Kumziene-Summerhayes, Sarah Taylor, Sophie Jones, Sara Rey, Matthew Bull, Joanne Watkins, Sally Corden, Tom Connor
EPI_ISL_422126 hCoV-19/Wales/PHWC-267B4/2020 Europe / United Kingdom / Wales 2020-03-28 Wales Specialist Virology Centre Public Health Wales Microbiology Cardiff Catherine Moore, Johnathan Evans, Malorie Perry, Simon Cottrell, Alec Birchley, Alexander Adams, Amy Gaskin, Bree Gatica-Wilcox, Jason Coombes, Lauren Gilbert, Lee Graham, Nicole Pacchiarini, Sara Kumziene-Summerhayes, Sarah Taylor, Sophie Jones, Sara Rey, Matthew Bull, Joanne Watkins, Sally Corden, Tom Connor
EPI_ISL_422127 hCoV-19/Wales/PHWC-26699/2020 Europe / United Kingdom / Wales 2020-03-27 Wales Specialist Virology Centre Public Health Wales Microbiology Cardiff Catherine Moore, Johnathan Evans, Malorie Perry, Simon Cottrell, Alec Birchley, Alexander Adams, Amy Gaskin, Bree Gatica-Wilcox, Jason Coombes, Lauren Gilbert, Lee Graham, Nicole Pacchiarini, Sara Kumziene-Summerhayes, Sarah Taylor, Sophie Jones, Sara Rey, Matthew Bull, Joanne Watkins, Sally Corden, Tom Connor
EPI_ISL_422128 hCoV-19/Wales/PHWC-266A8/2020 Europe / United Kingdom / Wales 2020-03-27 Wales Specialist Virology Centre Public Health Wales Microbiology Cardiff Catherine Moore, Johnathan Evans, Malorie Perry, Simon Cottrell, Alec Birchley, Alexander Adams, Amy Gaskin, Bree Gatica-Wilcox, Jason Coombes, Lauren Gilbert, Lee Graham, Nicole Pacchiarini, Sara Kumziene-Summerhayes, Sarah Taylor, Sophie Jones, Sara Rey, Matthew Bull, Joanne Watkins, Sally Corden, Tom Connor
EPI_ISL_422129 hCoV-19/Wales/PHWC-267A5/2020 Europe / United Kingdom / Wales 2020-03-28 Wales Specialist Virology Centre Public Health Wales Microbiology Cardiff Catherine Moore, Johnathan Evans, Malorie Perry, Simon Cottrell, Alec Birchley, Alexander Adams, Amy Gaskin, Bree Gatica-Wilcox, Jason Coombes, Lauren Gilbert, Lee Graham, Nicole Pacchiarini, Sara Kumziene-Summerhayes, Sarah Taylor, Sophie Jones, Sara Rey, Matthew Bull, Joanne Watkins, Sally Corden, Tom Connor
EPI_ISL_422130 hCoV-19/Wales/PHWC-26A9D/2020 Europe / United Kingdom / Wales 2020-03-30 Wales Specialist Virology Centre Public Health Wales Microbiology Cardiff Catherine Moore, Johnathan Evans, Malorie Perry, Simon Cottrell, Alec Birchley, Alexander Adams, Amy Gaskin, Bree Gatica-Wilcox, Jason Coombes, Lauren Gilbert, Lee Graham, Nicole Pacchiarini, Sara Kumziene-Summerhayes, Sarah Taylor, Sophie Jones, Sara Rey, Matthew Bull, Joanne Watkins, Sally Corden, Tom Connor
EPI_ISL_422131 hCoV-19/Wales/PHWC-27030/2020 Europe / United Kingdom / Wales 2020-03-28 Wales Specialist Virology Centre Public Health Wales Microbiology Cardiff Catherine Moore, Johnathan Evans, Malorie Perry, Simon Cottrell, Alec Birchley, Alexander Adams, Amy Gaskin, Bree Gatica-Wilcox, Jason Coombes, Lauren Gilbert, Lee Graham, Nicole Pacchiarini, Sara Kumziene-Summerhayes, Sarah Taylor, Sophie Jones, Sara Rey, Matthew Bull, Joanne Watkins, Sally Corden, Tom Connor
EPI_ISL_422132 hCoV-19/Wales/PHWC-26F07/2020 Europe / United Kingdom / Wales 2020-03-30 Wales Specialist Virology Centre Public Health Wales Microbiology Cardiff Catherine Moore, Johnathan Evans, Malorie Perry, Simon Cottrell, Alec Birchley, Alexander Adams, Amy Gaskin, Bree Gatica-Wilcox, Jason Coombes, Lauren Gilbert, Lee Graham, Nicole Pacchiarini, Sara Kumziene-Summerhayes, Sarah Taylor, Sophie Jones, Sara Rey, Matthew Bull, Joanne Watkins, Sally Corden, Tom Connor
EPI_ISL_422133 hCoV-19/Wales/PHWC-270C7/2020 Europe / United Kingdom / Wales 2020-03-30 Wales Specialist Virology Centre Public Health Wales Microbiology Cardiff Catherine Moore, Johnathan Evans, Malorie Perry, Simon Cottrell, Alec Birchley, Alexander Adams, Amy Gaskin, Bree Gatica-Wilcox, Jason Coombes, Lauren Gilbert, Lee Graham, Nicole Pacchiarini, Sara Kumziene-Summerhayes, Sarah Taylor, Sophie Jones, Sara Rey, Matthew Bull, Joanne Watkins, Sally Corden, Tom Connor
EPI_ISL_422134 hCoV-19/Wales/PHWC-25DD1/2020 Europe / United Kingdom / Wales 2020-03-25 Wales Specialist Virology Centre Public Health Wales Microbiology Cardiff Catherine Moore, Johnathan Evans, Malorie Perry, Simon Cottrell, Alec Birchley, Alexander Adams, Amy Gaskin, Bree Gatica-Wilcox, Jason Coombes, Lauren Gilbert, Lee Graham, Nicole Pacchiarini, Sara Kumziene-Summerhayes, Sarah Taylor, Sophie Jones, Sara Rey, Matthew Bull, Joanne Watkins, Sally Corden, Tom Connor
EPI_ISL_422135 hCoV-19/Wales/PHWC-25E65/2020 Europe / United Kingdom / Wales 2020-03-25 Wales Specialist Virology Centre Public Health Wales Microbiology Cardiff Catherine Moore, Johnathan Evans, Malorie Perry, Simon Cottrell, Alec Birchley, Alexander Adams, Amy Gaskin, Bree Gatica-Wilcox, Jason Coombes, Lauren Gilbert, Lee Graham, Nicole Pacchiarini, Sara Kumziene-Summerhayes, Sarah Taylor, Sophie Jones,

Sara Rey, Matthew Bull, Joanne Watkins, Sally Corden, Tom Connor
EPI_ISL_422136 hCoV-19/Wales/PHWC-25EB0/2020 Europe / United Kingdom / Wales 2020-03-25 Wales Specialist Virology Centre Public Health Wales Microbiology Cardiff Catherine Moore, Johnathan Evans, Malorie Perry, Simon Cottrell, Alec Birchley, Alexander Adams, Amy Gaskin, Bree Gatica-Wilcox, Jason Coombes, Lauren Gilbert, Lee Graham, Nicole Pacchiarini, Sara Kumziene-Summerhayes, Sarah Taylor, Sophie Jones, Sara Rey, Matthew Bull, Joanne Watkins, Sally Corden, Tom Connor
EPI_ISL_422137 hCoV-19/Wales/PHWC-26EEC/2020 Europe / United Kingdom / Wales 2020-03-30 Wales Specialist Virology Centre Public Health Wales Microbiology Cardiff Catherine Moore, Johnathan Evans, Malorie Perry, Simon Cottrell, Alec Birchley, Alexander Adams, Amy Gaskin, Bree Gatica-Wilcox, Jason Coombes, Lauren Gilbert, Lee Graham, Nicole Pacchiarini, Sara Kumziene-Summerhayes, Sarah Taylor, Sophie Jones, Sara Rey, Matthew Bull, Joanne Watkins, Sally Corden, Tom Connor
EPI_ISL_422138 hCoV-19/Wales/PHWC-259A0/2020 Europe / United Kingdom / Wales 2020-03-24 Wales Specialist Virology Centre Public Health Wales Microbiology Cardiff Catherine Moore, Johnathan Evans, Malorie Perry, Simon Cottrell, Alec Birchley, Alexander Adams, Amy Gaskin, Bree Gatica-Wilcox, Jason Coombes, Lauren Gilbert, Lee Graham, Nicole Pacchiarini, Sara Kumziene-Summerhayes, Sarah Taylor, Sophie Jones, Sara Rey, Matthew Bull, Joanne Watkins, Sally Corden, Tom Connor
EPI_ISL_422139 hCoV-19/Wales/PHWC-25867/2020 Europe / United Kingdom / Wales 2020-03-25 Wales Specialist Virology Centre Public Health Wales Microbiology Cardiff Catherine Moore, Johnathan Evans, Malorie Perry, Simon Cottrell, Alec Birchley, Alexander Adams, Amy Gaskin, Bree Gatica-Wilcox, Jason Coombes, Lauren Gilbert, Lee Graham, Nicole Pacchiarini, Sara Kumziene-Summerhayes, Sarah Taylor, Sophie Jones, Sara Rey, Matthew Bull, Joanne Watkins, Sally Corden, Tom Connor
EPI_ISL_422140 hCoV-19/Wales/PHWC-26F8F/2020 Europe / United Kingdom / Wales 2020-03-29 Wales Specialist Virology Centre Public Health Wales Microbiology Cardiff Catherine Moore, Johnathan Evans, Malorie Perry, Simon Cottrell, Alec Birchley, Alexander Adams, Amy Gaskin, Bree Gatica-Wilcox, Jason Coombes, Lauren Gilbert, Lee Graham, Nicole Pacchiarini, Sara Kumziene-Summerhayes, Sarah Taylor, Sophie Jones, Sara Rey, Matthew Bull, Joanne Watkins, Sally Corden, Tom Connor
EPI_ISL_422141 hCoV-19/Wales/PHWC-26893/2020 Europe / United Kingdom / Wales 2020-03-27 Wales Specialist Virology Centre Public Health Wales Microbiology Cardiff Catherine Moore, Johnathan Evans, Malorie Perry, Simon Cottrell, Alec Birchley, Alexander Adams, Amy Gaskin, Bree Gatica-Wilcox, Jason Coombes, Lauren Gilbert, Lee Graham, Nicole Pacchiarini, Sara Kumziene-Summerhayes, Sarah Taylor, Sophie Jones, Sara Rey, Matthew Bull, Joanne Watkins, Sally Corden, Tom Connor
EPI_ISL_422142 hCoV-19/Wales/PHWC-2613E/2020 Europe / United Kingdom / Wales 2020-03-25 Wales Specialist Virology Centre Public Health Wales Microbiology Cardiff Catherine Moore, Johnathan Evans, Malorie Perry, Simon Cottrell, Alec Birchley, Alexander Adams, Amy Gaskin, Bree Gatica-Wilcox, Jason Coombes, Lauren Gilbert, Lee Graham, Nicole Pacchiarini, Sara Kumziene-Summerhayes, Sarah Taylor, Sophie Jones, Sara Rey, Matthew Bull, Joanne Watkins, Sally Corden, Tom Connor
EPI_ISL_422143 hCoV-19/Wales/PHWC-26E64/2020 Europe / United Kingdom / Wales 2020-03-29 Wales Specialist Virology Centre Public Health Wales Microbiology Cardiff Catherine Moore, Johnathan Evans, Malorie Perry, Simon Cottrell, Alec Birchley, Alexander Adams, Amy Gaskin, Bree Gatica-Wilcox, Jason Coombes, Lauren Gilbert, Lee Graham, Nicole Pacchiarini, Sara Kumziene-Summerhayes, Sarah Taylor, Sophie Jones, Sara Rey, Matthew Bull, Joanne Watkins, Sally Corden, Tom Connor
EPI_ISL_422144 hCoV-19/Wales/PHWC-26F43/2020 Europe / United Kingdom / Wales 2020-03-28 Wales Specialist Virology Centre Public Health Wales Microbiology Cardiff Catherine Moore, Johnathan Evans, Malorie Perry, Simon Cottrell, Alec Birchley, Alexander Adams, Amy Gaskin, Bree Gatica-Wilcox, Jason Coombes, Lauren Gilbert, Lee Graham, Nicole Pacchiarini, Sara Kumziene-Summerhayes, Sarah Taylor, Sophie Jones, Sara Rey, Matthew Bull, Joanne Watkins, Sally Corden, Tom Connor
EPI_ISL_422145 hCoV-19/Wales/PHWC-26EA0/2020 Europe / United Kingdom / Wales 2020-03-30 Wales Specialist Virology Centre Public Health Wales Microbiology Cardiff Catherine Moore, Johnathan Evans, Malorie Perry, Simon Cottrell, Alec Birchley, Alexander Adams, Amy Gaskin, Bree Gatica-Wilcox, Jason Coombes, Lauren Gilbert, Lee Graham, Nicole Pacchiarini, Sara Kumziene-Summerhayes, Sarah Taylor, Sophie Jones, Sara Rey, Matthew Bull, Joanne Watkins, Sally Corden, Tom Connor
EPI_ISL_422146 hCoV-19/Wales/PHWC-26D2B/2020 Europe / United Kingdom / Wales 2020-03-30 Wales Specialist Virology Centre Public Health Wales Microbiology Cardiff Catherine Moore, Johnathan Evans, Malorie Perry, Simon Cottrell, Alec Birchley, Alexander Adams, Amy Gaskin, Bree Gatica-Wilcox, Jason Coombes, Lauren Gilbert, Lee Graham, Nicole Pacchiarini, Sara Kumziene-Summerhayes, Sarah Taylor, Sophie Jones, Sara Rey, Matthew Bull, Joanne Watkins, Sally Corden, Tom Connor
EPI_ISL_422147 hCoV-19/Wales/PHWC-2706D/2020 Europe / United Kingdom / Wales 2020-03-28 Wales Specialist Virology Centre Public Health Wales Microbiology Cardiff Catherine Moore, Johnathan Evans, Malorie Perry, Simon Cottrell, Alec Birchley, Alexander Adams, Amy Gaskin, Bree Gatica-Wilcox, Jason Coombes, Lauren Gilbert, Lee Graham, Nicole Pacchiarini, Sara Kumziene-Summerhayes, Sarah Taylor, Sophie Jones, Sara Rey, Matthew Bull, Joanne Watkins, Sally Corden, Tom Connor
EPI_ISL_422148 hCoV-19/Wales/PHWC-26BC7/2020 Europe / United Kingdom / Wales 2020-03-27 Wales Specialist Virology Centre Public Health Wales Microbiology Cardiff Catherine Moore, Johnathan Evans, Malorie Perry, Simon Cottrell, Alec Birchley, Alexander Adams, Amy Gaskin, Bree Gatica-Wilcox, Jason Coombes, Lauren Gilbert, Lee Graham, Nicole Pacchiarini, Sara Kumziene-Summerhayes, Sarah Taylor, Sophie Jones, Sara Rey, Matthew Bull, Joanne Watkins, Sally Corden, Tom Connor
EPI_ISL_422149 hCoV-19/Wales/PHWC-26004/2020 Europe / United Kingdom / Wales 2020-03-25 Wales Specialist Virology Centre Public Health Wales Microbiology Cardiff Catherine Moore, Johnathan Evans, Malorie Perry, Simon Cottrell, Alec Birchley, Alexander Adams, Amy Gaskin, Bree Gatica-Wilcox, Jason Coombes, Lauren Gilbert, Lee Graham, Nicole Pacchiarini, Sara Kumziene-Summerhayes, Sarah Taylor, Sophie Jones,

Sara Rey, Matthew Bull, Joanne Watkins, Sally Corden, Tom Connor
EPI_ISL_422150 hCoV-19/Wales/PHWC-25E0B/2020 Europe / United Kingdom / Wales 2020-03-25 Wales Specialist Virology Centre Public Health Wales Microbiology Cardiff Catherine Moore, Johnathan Evans, Malorie Perry, Simon Cottrell, Alec Birchley, Alexander Adams, Amy Gaskin, Bree Gatica-Wilcox, Jason Coombes, Lauren Gilbert, Lee Graham, Nicole Pacchiarini, Sara Kumziene-Summerhayes, Sarah Taylor, Sophie Jones, Sara Rey, Matthew Bull, Joanne Watkins, Sally Corden, Tom Connor
EPI_ISL_422151 hCoV-19/Wales/PHWC-26C1F/2020 Europe / United Kingdom / Wales 2020-03-28 Wales Specialist Virology Centre Public Health Wales Microbiology Cardiff Catherine Moore, Johnathan Evans, Malorie Perry, Simon Cottrell, Alec Birchley, Alexander Adams, Amy Gaskin, Bree Gatica-Wilcox, Jason Coombes, Lauren Gilbert, Lee Graham, Nicole Pacchiarini, Sara Kumziene-Summerhayes, Sarah Taylor, Sophie Jones, Sara Rey, Matthew Bull, Joanne Watkins, Sally Corden, Tom Connor
EPI_ISL_422152 hCoV-19/Wales/PHWC-26FAD/2020 Europe / United Kingdom / Wales 2020-03-30 Wales Specialist Virology Centre Public Health Wales Microbiology Cardiff Catherine Moore, Johnathan Evans, Malorie Perry, Simon Cottrell, Alec Birchley, Alexander Adams, Amy Gaskin, Bree Gatica-Wilcox, Jason Coombes, Lauren Gilbert, Lee Graham, Nicole Pacchiarini, Sara Kumziene-Summerhayes, Sarah Taylor, Sophie Jones, Sara Rey, Matthew Bull, Joanne Watkins, Sally Corden, Tom Connor
EPI_ISL_422153 hCoV-19/Wales/PHWC-257C4/2020 Europe / United Kingdom / Wales 2020-03-24 Wales Specialist Virology Centre Public Health Wales Microbiology Cardiff Catherine Moore, Johnathan Evans, Malorie Perry, Simon Cottrell, Alec Birchley, Alexander Adams, Amy Gaskin, Bree Gatica-Wilcox, Jason Coombes, Lauren Gilbert, Lee Graham, Nicole Pacchiarini, Sara Kumziene-Summerhayes, Sarah Taylor, Sophie Jones, Sara Rey, Matthew Bull, Joanne Watkins, Sally Corden, Tom Connor
EPI_ISL_422154 hCoV-19/Wales/PHWC-25C10/2020 Europe / United Kingdom / Wales 2020-03-25 Wales Specialist Virology Centre Public Health Wales Microbiology Cardiff Catherine Moore, Johnathan Evans, Malorie Perry, Simon Cottrell, Alec Birchley, Alexander Adams, Amy Gaskin, Bree Gatica-Wilcox, Jason Coombes, Lauren Gilbert, Lee Graham, Nicole Pacchiarini, Sara Kumziene-Summerhayes, Sarah Taylor, Sophie Jones, Sara Rey, Matthew Bull, Joanne Watkins, Sally Corden, Tom Connor
EPI_ISL_422155 hCoV-19/Wales/PHWC-2713D/2020 Europe / United Kingdom / Wales 2020-03-29 Wales Specialist Virology Centre Public Health Wales Microbiology Cardiff Catherine Moore, Johnathan Evans, Malorie Perry, Simon Cottrell, Alec Birchley, Alexander Adams, Amy Gaskin, Bree Gatica-Wilcox, Jason Coombes, Lauren Gilbert, Lee Graham, Nicole Pacchiarini, Sara Kumziene-Summerhayes, Sarah Taylor, Sophie Jones, Sara Rey, Matthew Bull, Joanne Watkins, Sally Corden, Tom Connor
EPI_ISL_422156 hCoV-19/Wales/PHWC-26C88/2020 Europe / United Kingdom / Wales 2020-03-28 Wales Specialist Virology Centre Public Health Wales Microbiology Cardiff Catherine Moore, Johnathan Evans, Malorie Perry, Simon Cottrell, Alec Birchley, Alexander Adams, Amy Gaskin, Bree Gatica-Wilcox, Jason Coombes, Lauren Gilbert, Lee Graham, Nicole Pacchiarini, Sara Kumziene-Summerhayes, Sarah Taylor, Sophie Jones, Sara Rey, Matthew Bull, Joanne Watkins, Sally Corden, Tom Connor
EPI_ISL_422157 hCoV-19/Wales/PHWC-26787/2020 Europe / United Kingdom / Wales 2020-03-28 Wales Specialist Virology Centre Public Health Wales Microbiology Cardiff Catherine Moore, Johnathan Evans, Malorie Perry, Simon Cottrell, Alec Birchley, Alexander Adams, Amy Gaskin, Bree Gatica-Wilcox, Jason Coombes, Lauren Gilbert, Lee Graham, Nicole Pacchiarini, Sara Kumziene-Summerhayes, Sarah Taylor, Sophie Jones, Sara Rey, Matthew Bull, Joanne Watkins, Sally Corden, Tom Connor
EPI_ISL_422158 hCoV-19/Wales/PHWC-266D5/2020 Europe / United Kingdom / Wales 2020-03-27 Wales Specialist Virology Centre Public Health Wales Microbiology Cardiff Catherine Moore, Johnathan Evans, Malorie Perry, Simon Cottrell, Alec Birchley, Alexander Adams, Amy Gaskin, Bree Gatica-Wilcox, Jason Coombes, Lauren Gilbert, Lee Graham, Nicole Pacchiarini, Sara Kumziene-Summerhayes, Sarah Taylor, Sophie Jones, Sara Rey, Matthew Bull, Joanne Watkins, Sally Corden, Tom Connor
EPI_ISL_422159 hCoV-19/Wales/PHWC-26E28/2020 Europe / United Kingdom / Wales 2020-03-29 Wales Specialist Virology Centre Public Health Wales Microbiology Cardiff Catherine Moore, Johnathan Evans, Malorie Perry, Simon Cottrell, Alec Birchley, Alexander Adams, Amy Gaskin, Bree Gatica-Wilcox, Jason Coombes, Lauren Gilbert, Lee Graham, Nicole Pacchiarini, Sara Kumziene-Summerhayes, Sarah Taylor, Sophie Jones, Sara Rey, Matthew Bull, Joanne Watkins, Sally Corden, Tom Connor
EPI_ISL_422160 hCoV-19/Wales/PHWC-26D94/2020 Europe / United Kingdom / Wales 2020-03-30 Wales Specialist Virology Centre Public Health Wales Microbiology Cardiff Catherine Moore, Johnathan Evans, Malorie Perry, Simon Cottrell, Alec Birchley, Alexander Adams, Amy Gaskin, Bree Gatica-Wilcox, Jason Coombes, Lauren Gilbert, Lee Graham, Nicole Pacchiarini, Sara Kumziene-Summerhayes, Sarah Taylor, Sophie Jones, Sara Rey, Matthew Bull, Joanne Watkins, Sally Corden, Tom Connor
EPI_ISL_422161 hCoV-19/Wales/PHWC-26CE2/2020 Europe / United Kingdom / Wales 2020-03-29 Wales Specialist Virology Centre Public Health Wales Microbiology Cardiff Catherine Moore, Johnathan Evans, Malorie Perry, Simon Cottrell, Alec Birchley, Alexander Adams, Amy Gaskin, Bree Gatica-Wilcox, Jason Coombes, Lauren Gilbert, Lee Graham, Nicole Pacchiarini, Sara Kumziene-Summerhayes, Sarah Taylor, Sophie Jones, Sara Rey, Matthew Bull, Joanne Watkins, Sally Corden, Tom Connor
EPI_ISL_422162 hCoV-19/Wales/PHWC-26611/2020 Europe / United Kingdom / Wales 2020-03-27 Wales Specialist Virology Centre Public Health Wales Microbiology Cardiff Catherine Moore, Johnathan Evans, Malorie Perry, Simon Cottrell, Alec Birchley, Alexander Adams, Amy Gaskin, Bree Gatica-Wilcox, Jason Coombes, Lauren Gilbert, Lee Graham, Nicole Pacchiarini, Sara Kumziene-Summerhayes, Sarah Taylor, Sophie Jones, Sara Rey, Matthew Bull, Joanne Watkins, Sally Corden, Tom Connor
EPI_ISL_422163 hCoV-19/Wales/PHWC-26D85/2020 Europe / United Kingdom / Wales 2020-03-30 Wales Specialist Virology Centre Public Health Wales Microbiology Cardiff Catherine Moore, Johnathan Evans, Malorie Perry, Simon Cottrell, Alec Birchley, Alexander Adams, Amy Gaskin, Bree Gatica-Wilcox, Jason Coombes, Lauren Gilbert, Lee Graham, Nicole Pacchiarini, Sara Kumziene-Summerhayes, Sarah Taylor, Sophie Jones,

Sara Rey, Matthew Bull, Joanne Watkins, Sally Corden, Tom Connor
EPI_ISL_422164 hCoV-19/Wales/PHWC-26620/2020 Europe / United Kingdom / Wales 2020-03-26 Wales Specialist Virology Centre Public Health Wales Microbiology Cardiff Catherine Moore, Johnathan Evans, Malorie Perry, Simon Cottrell, Alec Birchley, Alexander Adams, Amy Gaskin, Bree Gatica-Wilcox, Jason Coombes, Lauren Gilbert, Lee Graham, Nicole Pacchiarini, Sara Kumziene-Summerhayes, Sarah Taylor, Sophie Jones, Sara Rey, Matthew Bull, Joanne Watkins, Sally Corden, Tom Connor
EPI_ISL_422165 hCoV-19/Wales/PHWC-26C3D/2020 Europe / United Kingdom / Wales 2020-03-30 Wales Specialist Virology Centre Public Health Wales Microbiology Cardiff Catherine Moore, Johnathan Evans, Malorie Perry, Simon Cottrell, Alec Birchley, Alexander Adams, Amy Gaskin, Bree Gatica-Wilcox, Jason Coombes, Lauren Gilbert, Lee Graham, Nicole Pacchiarini, Sara Kumziene-Summerhayes, Sarah Taylor, Sophie Jones, Sara Rey, Matthew Bull, Joanne Watkins, Sally Corden, Tom Connor
EPI_ISL_422166 hCoV-19/Wales/PHWC-26927/2020 Europe / United Kingdom / Wales 2020-03-28 Wales Specialist Virology Centre Public Health Wales Microbiology Cardiff Catherine Moore, Johnathan Evans, Malorie Perry, Simon Cottrell, Alec Birchley, Alexander Adams, Amy Gaskin, Bree Gatica-Wilcox, Jason Coombes, Lauren Gilbert, Lee Graham, Nicole Pacchiarini, Sara Kumziene-Summerhayes, Sarah Taylor, Sophie Jones, Sara Rey, Matthew Bull, Joanne Watkins, Sally Corden, Tom Connor
EPI_ISL_422167 hCoV-19/Wales/PHWC-266B7/2020 Europe / United Kingdom / Wales 2020-03-27 Wales Specialist Virology Centre Public Health Wales Microbiology Cardiff Catherine Moore, Johnathan Evans, Malorie Perry, Simon Cottrell, Alec Birchley, Alexander Adams, Amy Gaskin, Bree Gatica-Wilcox, Jason Coombes, Lauren Gilbert, Lee Graham, Nicole Pacchiarini, Sara Kumziene-Summerhayes, Sarah Taylor, Sophie Jones, Sara Rey, Matthew Bull, Joanne Watkins, Sally Corden, Tom Connor
EPI_ISL_422168 hCoV-19/Wales/PHWC-25F62/2020 Europe / United Kingdom / Wales 2020-03-25 Wales Specialist Virology Centre Public Health Wales Microbiology Cardiff Catherine Moore, Johnathan Evans, Malorie Perry, Simon Cottrell, Alec Birchley, Alexander Adams, Amy Gaskin, Bree Gatica-Wilcox, Jason Coombes, Lauren Gilbert, Lee Graham, Nicole Pacchiarini, Sara Kumziene-Summerhayes, Sarah Taylor, Sophie Jones, Sara Rey, Matthew Bull, Joanne Watkins, Sally Corden, Tom Connor
EPI_ISL_422169 hCoV-19/Wales/PHWC-25946/2020 Europe / United Kingdom / Wales 2020-03-25 Wales Specialist Virology Centre Public Health Wales Microbiology Cardiff Catherine Moore, Johnathan Evans, Malorie Perry, Simon Cottrell, Alec Birchley, Alexander Adams, Amy Gaskin, Bree Gatica-Wilcox, Jason Coombes, Lauren Gilbert, Lee Graham, Nicole Pacchiarini, Sara Kumziene-Summerhayes, Sarah Taylor, Sophie Jones, Sara Rey, Matthew Bull, Joanne Watkins, Sally Corden, Tom Connor
EPI_ISL_422170 hCoV-19/Wales/PHWC-26BA9/2020 Europe / United Kingdom / Wales 2020-03-27 Wales Specialist Virology Centre Public Health Wales Microbiology Cardiff Catherine Moore, Johnathan Evans, Malorie Perry, Simon Cottrell, Alec Birchley, Alexander Adams, Amy Gaskin, Bree Gatica-Wilcox, Jason Coombes, Lauren Gilbert, Lee Graham, Nicole Pacchiarini, Sara Kumziene-Summerhayes, Sarah Taylor, Sophie Jones, Sara Rey, Matthew Bull, Joanne Watkins, Sally Corden, Tom Connor
EPI_ISL_422171 hCoV-19/Wales/PHWC-26FDA/2020 Europe / United Kingdom / Wales 2020-03-30 Wales Specialist Virology Centre Public Health Wales Microbiology Cardiff Catherine Moore, Johnathan Evans, Malorie Perry, Simon Cottrell, Alec Birchley, Alexander Adams, Amy Gaskin, Bree Gatica-Wilcox, Jason Coombes, Lauren Gilbert, Lee Graham, Nicole Pacchiarini, Sara Kumziene-Summerhayes, Sarah Taylor, Sophie Jones, Sara Rey, Matthew Bull, Joanne Watkins, Sally Corden, Tom Connor
EPI_ISL_422172 hCoV-19/Wales/PHWC-25A52/2020 Europe / United Kingdom / Wales 2020-03-23 Wales Specialist Virology Centre Public Health Wales Microbiology Cardiff Catherine Moore, Johnathan Evans, Malorie Perry, Simon Cottrell, Alec Birchley, Alexander Adams, Amy Gaskin, Bree Gatica-Wilcox, Jason Coombes, Lauren Gilbert, Lee Graham, Nicole Pacchiarini, Sara Kumziene-Summerhayes, Sarah Taylor, Sophie Jones, Sara Rey, Matthew Bull, Joanne Watkins, Sally Corden, Tom Connor
EPI_ISL_422173 hCoV-19/Wales/PHWC-26C4C/2020 Europe / United Kingdom / Wales 2020-03-29 Wales Specialist Virology Centre Public Health Wales Microbiology Cardiff Catherine Moore, Johnathan Evans, Malorie Perry, Simon Cottrell, Alec Birchley, Alexander Adams, Amy Gaskin, Bree Gatica-Wilcox, Jason Coombes, Lauren Gilbert, Lee Graham, Nicole Pacchiarini, Sara Kumziene-Summerhayes, Sarah Taylor, Sophie Jones, Sara Rey, Matthew Bull, Joanne Watkins, Sally Corden, Tom Connor
EPI_ISL_422174 hCoV-19/Wales/PHWC-26F70/2020 Europe / United Kingdom / Wales 2020-03-29 Wales Specialist Virology Centre Public Health Wales Microbiology Cardiff Catherine Moore, Johnathan Evans, Malorie Perry, Simon Cottrell, Alec Birchley, Alexander Adams, Amy Gaskin, Bree Gatica-Wilcox, Jason Coombes, Lauren Gilbert, Lee Graham, Nicole Pacchiarini, Sara Kumziene-Summerhayes, Sarah Taylor, Sophie Jones, Sara Rey, Matthew Bull, Joanne Watkins, Sally Corden, Tom Connor
EPI_ISL_422175 hCoV-19/Wales/PHWC-257F1/2020 Europe / United Kingdom / Wales 2020-03-24 Wales Specialist Virology Centre Public Health Wales Microbiology Cardiff Catherine Moore, Johnathan Evans, Malorie Perry, Simon Cottrell, Alec Birchley, Alexander Adams, Amy Gaskin, Bree Gatica-Wilcox, Jason Coombes, Lauren Gilbert, Lee Graham, Nicole Pacchiarini, Sara Kumziene-Summerhayes, Sarah Taylor, Sophie Jones, Sara Rey, Matthew Bull, Joanne Watkins, Sally Corden, Tom Connor
EPI_ISL_422176 hCoV-19/Wales/PHWC-269CD/2020 Europe / United Kingdom / Wales 2020-03-30 Wales Specialist Virology Centre Public Health Wales Microbiology Cardiff Catherine Moore, Johnathan Evans, Malorie Perry, Simon Cottrell, Alec Birchley, Alexander Adams, Amy Gaskin, Bree Gatica-Wilcox, Jason Coombes, Lauren Gilbert, Lee Graham, Nicole Pacchiarini, Sara Kumziene-Summerhayes, Sarah Taylor, Sophie Jones, Sara Rey, Matthew Bull, Joanne Watkins, Sally Corden, Tom Connor
EPI_ISL_422177 hCoV-19/Wales/PHWC-2668A/2020 Europe / United Kingdom / Wales 2020-03-27 Wales Specialist Virology Centre Public Health Wales Microbiology Cardiff Catherine Moore, Johnathan Evans, Malorie Perry, Simon Cottrell, Alec Birchley, Alexander Adams, Amy Gaskin, Bree Gatica-Wilcox, Jason Coombes, Lauren Gilbert, Lee Graham, Nicole Pacchiarini, Sara Kumziene-Summerhayes, Sarah Taylor, Sophie Jones,

Sara Rey, Matthew Bull, Joanne Watkins, Sally Corden, Tom Connor
EPI_ISL_422178 hCoV-19/Wales/PHWC-26D67/2020 Europe / United Kingdom / Wales 2020-03-30 Wales Specialist Virology Centre Public Health Wales Microbiology Cardiff Catherine Moore, Johnathan Evans, Malorie Perry, Simon Cottrell, Alec Birchley, Alexander Adams, Amy Gaskin, Bree Gatica-Wilcox, Jason Coombes, Lauren Gilbert, Lee Graham, Nicole Pacchiarini, Sara Kumziene-Summerhayes, Sarah Taylor, Sophie Jones, Sara Rey, Matthew Bull, Joanne Watkins, Sally Corden, Tom Connor
EPI_ISL_422179 hCoV-19/Wales/PHWC-262A4/2020 Europe / United Kingdom / Wales 2020-03-27 Wales Specialist Virology Centre Public Health Wales Microbiology Cardiff Catherine Moore, Johnathan Evans, Malorie Perry, Simon Cottrell, Alec Birchley, Alexander Adams, Amy Gaskin, Bree Gatica-Wilcox, Jason Coombes, Lauren Gilbert, Lee Graham, Nicole Pacchiarini, Sara Kumziene-Summerhayes, Sarah Taylor, Sophie Jones, Sara Rey, Matthew Bull, Joanne Watkins, Sally Corden, Tom Connor
EPI_ISL_422180 hCoV-19/Wales/PHWC-25B7D/2020 Europe / United Kingdom / Wales 2020-03-25 Wales Specialist Virology Centre Public Health Wales Microbiology Cardiff Catherine Moore, Johnathan Evans, Malorie Perry, Simon Cottrell, Alec Birchley, Alexander Adams, Amy Gaskin, Bree Gatica-Wilcox, Jason Coombes, Lauren Gilbert, Lee Graham, Nicole Pacchiarini, Sara Kumziene-Summerhayes, Sarah Taylor, Sophie Jones, Sara Rey, Matthew Bull, Joanne Watkins, Sally Corden, Tom Connor
EPI_ISL_422181 hCoV-19/Wales/PHWC-261F2/2020 Europe / United Kingdom / Wales 2020-03-26 Wales Specialist Virology Centre Public Health Wales Microbiology Cardiff Catherine Moore, Johnathan Evans, Malorie Perry, Simon Cottrell, Alec Birchley, Alexander Adams, Amy Gaskin, Bree Gatica-Wilcox, Jason Coombes, Lauren Gilbert, Lee Graham, Nicole Pacchiarini, Sara Kumziene-Summerhayes, Sarah Taylor, Sophie Jones, Sara Rey, Matthew Bull, Joanne Watkins, Sally Corden, Tom Connor
EPI_ISL_422182 hCoV-19/Wales/PHWC-267D2/2020 Europe / United Kingdom / Wales 2020-03-25 Wales Specialist Virology Centre Public Health Wales Microbiology Cardiff Catherine Moore, Johnathan Evans, Malorie Perry, Simon Cottrell, Alec Birchley, Alexander Adams, Amy Gaskin, Bree Gatica-Wilcox, Jason Coombes, Lauren Gilbert, Lee Graham, Nicole Pacchiarini, Sara Kumziene-Summerhayes, Sarah Taylor, Sophie Jones, Sara Rey, Matthew Bull, Joanne Watkins, Sally Corden, Tom Connor
EPI_ISL_422183 hCoV-19/Wales/PHWC-25B5F/2020 Europe / United Kingdom / Wales 2020-03-24 Wales Specialist Virology Centre Public Health Wales Microbiology Cardiff Catherine Moore, Johnathan Evans, Malorie Perry, Simon Cottrell, Alec Birchley, Alexander Adams, Amy Gaskin, Bree Gatica-Wilcox, Jason Coombes, Lauren Gilbert, Lee Graham, Nicole Pacchiarini, Sara Kumziene-Summerhayes, Sarah Taylor, Sophie Jones, Sara Rey, Matthew Bull, Joanne Watkins, Sally Corden, Tom Connor
EPI_ISL_422184 hCoV-19/Wales/PHWC-26875/2020 Europe / United Kingdom / Wales 2020-03-27 Wales Specialist Virology Centre Public Health Wales Microbiology Cardiff Catherine Moore, Johnathan Evans, Malorie Perry, Simon Cottrell, Alec Birchley, Alexander Adams, Amy Gaskin, Bree Gatica-Wilcox, Jason Coombes, Lauren Gilbert, Lee Graham, Nicole Pacchiarini, Sara Kumziene-Summerhayes, Sarah Taylor, Sophie Jones, Sara Rey, Matthew Bull, Joanne Watkins, Sally Corden, Tom Connor
EPI_ISL_422185 hCoV-19/Wales/PHWC-2680C/2020 Europe / United Kingdom / Wales 2020-03-26 Wales Specialist Virology Centre Public Health Wales Microbiology Cardiff Catherine Moore, Johnathan Evans, Malorie Perry, Simon Cottrell, Alec Birchley, Alexander Adams, Amy Gaskin, Bree Gatica-Wilcox, Jason Coombes, Lauren Gilbert, Lee Graham, Nicole Pacchiarini, Sara Kumziene-Summerhayes, Sarah Taylor, Sophie Jones, Sara Rey, Matthew Bull, Joanne Watkins, Sally Corden, Tom Connor
EPI_ISL_422186 hCoV-19/Wales/PHWC-26B4F/2020 Europe / United Kingdom / Wales 2020-03-29 Wales Specialist Virology Centre Public Health Wales Microbiology Cardiff Catherine Moore, Johnathan Evans, Malorie Perry, Simon Cottrell, Alec Birchley, Alexander Adams, Amy Gaskin, Bree Gatica-Wilcox, Jason Coombes, Lauren Gilbert, Lee Graham, Nicole Pacchiarini, Sara Kumziene-Summerhayes, Sarah Taylor, Sophie Jones, Sara Rey, Matthew Bull, Joanne Watkins, Sally Corden, Tom Connor
EPI_ISL_422187 hCoV-19/Wales/PHWC-2709A/2020 Europe / United Kingdom / Wales 2020-03-29 Wales Specialist Virology Centre Public Health Wales Microbiology Cardiff Catherine Moore, Johnathan Evans, Malorie Perry, Simon Cottrell, Alec Birchley, Alexander Adams, Amy Gaskin, Bree Gatica-Wilcox, Jason Coombes, Lauren Gilbert, Lee Graham, Nicole Pacchiarini, Sara Kumziene-Summerhayes, Sarah Taylor, Sophie Jones, Sara Rey, Matthew Bull, Joanne Watkins, Sally Corden, Tom Connor
EPI_ISL_423321 hCoV-19/England/20138063804/2020 Europe / United Kingdom / England 2020-03-26 Respiratory Virus Unit, Microbiology Services Colindale, Public Health England Respiratory Virus Unit, Microbiology Services Colindale, Public Health England Monica Galiano, Shahjahan Miah, Angie Lackenby, Omolola Akinbami, Tiina Talts, Leena Bhaw, Richard Myers, Steven Platt, Kirstin Edwards, Jonathan Hubb, Joanna Ellis, Maria Zambon
EPI_ISL_423324 hCoV-19/England/20138078204/2020 Europe / United Kingdom / England 2020-03-26 Respiratory Virus Unit, Microbiology Services Colindale, Public Health England Respiratory Virus Unit, Microbiology Services Colindale, Public Health England Monica Galiano, Shahjahan Miah, Angie Lackenby, Omolola Akinbami, Tiina Talts, Leena Bhaw, Richard Myers, Steven Platt, Kirstin Edwards, Jonathan Hubb, Joanna Ellis, Maria Zambon
EPI_ISL_423328 hCoV-19/England/20138084904/2020 Europe / United Kingdom / England 2020-03-27 Respiratory Virus Unit, Microbiology Services Colindale, Public Health England Respiratory Virus Unit, Microbiology Services Colindale, Public Health England Monica Galiano, Shahjahan Miah, Angie Lackenby, Omolola Akinbami, Tiina Talts, Leena Bhaw, Richard Myers, Steven Platt, Kirstin Edwards, Jonathan Hubb, Joanna Ellis, Maria Zambon
EPI_ISL_423337 hCoV-19/England/20139002204/2020 Europe / United Kingdom / England 2020-03-27 Respiratory Virus Unit, Microbiology Services Colindale, Public Health England Respiratory Virus Unit, Microbiology Services Colindale, Public Health England Monica Galiano, Shahjahan Miah, Angie Lackenby, Omolola Akinbami, Tiina Talts, Leena Bhaw, Richard Myers, Steven Platt, Kirstin Edwards, Jonathan Hubb, Joanna Ellis,

Maria Zambon
EPI_ISL_423340 hCoV-19/England/20139015304/2020 Europe / United Kingdom / England 2020-03-27 Respiratory Virus Unit, Microbiology Services Colindale, Public Health England Respiratory Virus Unit, Microbiology Services Colindale, Public Health England Monica Galiano, Shahjahan Miah, Angie Lackenby, Omolola Akinbami, Tiina Talts, Leena Bhaw, Richard Myers, Steven Platt, Kirstin Edwards, Jonathan Hubb, Joanna Ellis, Maria Zambon
EPI_ISL_423346 hCoV-19/England/20139016704/2020 Europe / United Kingdom / England 2020-03-27 Respiratory Virus Unit, Microbiology Services Colindale, Public Health England Respiratory Virus Unit, Microbiology Services Colindale, Public Health England Monica Galiano, Shahjahan Miah, Angie Lackenby, Omolola Akinbami, Tiina Talts, Leena Bhaw, Richard Myers, Steven Platt, Kirstin Edwards, Jonathan Hubb, Joanna Ellis, Maria Zambon
EPI_ISL_423350 hCoV-19/England/20139060704/2020 Europe / United Kingdom / England 2020-03-28 Respiratory Virus Unit, Microbiology Services Colindale, Public Health England Respiratory Virus Unit, Microbiology Services Colindale, Public Health England Monica Galiano, Shahjahan Miah, Angie Lackenby, Omolola Akinbami, Tiina Talts, Leena Bhaw, Richard Myers, Steven Platt, Kirstin Edwards, Jonathan Hubb, Joanna Ellis, Maria Zambon
EPI_ISL_423351 hCoV-19/England/20139064404/2020 Europe / United Kingdom / England 2020-03-28 Respiratory Virus Unit, Microbiology Services Colindale, Public Health England Respiratory Virus Unit, Microbiology Services Colindale, Public Health England Monica Galiano, Shahjahan Miah, Angie Lackenby, Omolola Akinbami, Tiina Talts, Leena Bhaw, Richard Myers, Steven Platt, Kirstin Edwards, Jonathan Hubb, Joanna Ellis, Maria Zambon
EPI_ISL_423352 hCoV-19/England/20139064904/2020 Europe / United Kingdom / England 2020-03-29 Respiratory Virus Unit, Microbiology Services Colindale, Public Health England Respiratory Virus Unit, Microbiology Services Colindale, Public Health England Monica Galiano, Shahjahan Miah, Angie Lackenby, Omolola Akinbami, Tiina Talts, Leena Bhaw, Richard Myers, Steven Platt, Kirstin Edwards, Jonathan Hubb, Joanna Ellis, Maria Zambon
EPI_ISL_423353 hCoV-19/England/20139065204/2020 Europe / United Kingdom / England 2020-03-28 Respiratory Virus Unit, Microbiology Services Colindale, Public Health England Respiratory Virus Unit, Microbiology Services Colindale, Public Health England Monica Galiano, Shahjahan Miah, Angie Lackenby, Omolola Akinbami, Tiina Talts, Leena Bhaw, Richard Myers, Steven Platt, Kirstin Edwards, Jonathan Hubb, Joanna Ellis, Maria Zambon
EPI_ISL_423354 hCoV-19/England/20139065404/2020 Europe / United Kingdom / England 2020-03-29 Respiratory Virus Unit, Microbiology Services Colindale, Public Health England Respiratory Virus Unit, Microbiology Services Colindale, Public Health England Monica Galiano, Shahjahan Miah, Angie Lackenby, Omolola Akinbami, Tiina Talts, Leena Bhaw, Richard Myers, Steven Platt, Kirstin Edwards, Jonathan Hubb, Joanna Ellis, Maria Zambon
EPI_ISL_423355 hCoV-19/England/20139065604/2020 Europe / United Kingdom / England 2020-03-29 Respiratory Virus Unit, Microbiology Services Colindale, Public Health England Respiratory Virus Unit, Microbiology Services Colindale, Public Health England Monica Galiano, Shahjahan Miah, Angie Lackenby, Omolola Akinbami, Tiina Talts, Leena Bhaw, Richard Myers, Steven Platt, Kirstin Edwards, Jonathan Hubb, Joanna Ellis, Maria Zambon
EPI_ISL_423356 hCoV-19/England/20139067504/2020 Europe / United Kingdom / England 2020-03-28 Respiratory Virus Unit, Microbiology Services Colindale, Public Health England Respiratory Virus Unit, Microbiology Services Colindale, Public Health England Monica Galiano, Shahjahan Miah, Angie Lackenby, Omolola Akinbami, Tiina Talts, Leena Bhaw, Richard Myers, Steven Platt, Kirstin Edwards, Jonathan Hubb, Joanna Ellis, Maria Zambon
EPI_ISL_423357 hCoV-19/England/20139068504/2020 Europe / United Kingdom / England 2020-03-29 Respiratory Virus Unit, Microbiology Services Colindale, Public Health England Respiratory Virus Unit, Microbiology Services Colindale, Public Health England Monica Galiano, Shahjahan Miah, Angie Lackenby, Omolola Akinbami, Tiina Talts, Leena Bhaw, Richard Myers, Steven Platt, Kirstin Edwards, Jonathan Hubb, Joanna Ellis, Maria Zambon
EPI_ISL_423358 hCoV-19/England/20139068804/2020 Europe / United Kingdom / England 2020-03-28 Respiratory Virus Unit, Microbiology Services Colindale, Public Health England Respiratory Virus Unit, Microbiology Services Colindale, Public Health England Monica Galiano, Shahjahan Miah, Angie Lackenby, Omolola Akinbami, Tiina Talts, Leena Bhaw, Richard Myers, Steven Platt, Kirstin Edwards, Jonathan Hubb, Joanna Ellis, Maria Zambon
EPI_ISL_423359 hCoV-19/England/20139069104/2020 Europe / United Kingdom / England 2020-03-29 Respiratory Virus Unit, Microbiology Services Colindale, Public Health England Respiratory Virus Unit, Microbiology Services Colindale, Public Health England Monica Galiano, Shahjahan Miah, Angie Lackenby, Omolola Akinbami, Tiina Talts, Leena Bhaw, Richard Myers, Steven Platt, Kirstin Edwards, Jonathan Hubb, Joanna Ellis, Maria Zambon
EPI_ISL_423360 hCoV-19/England/20139069404/2020 Europe / United Kingdom / England 2020-03-29 Respiratory Virus Unit, Microbiology Services Colindale, Public Health England Respiratory Virus Unit, Microbiology Services Colindale, Public Health England Monica Galiano, Shahjahan Miah, Angie Lackenby, Omolola Akinbami, Tiina Talts, Leena Bhaw, Richard Myers, Steven Platt, Kirstin Edwards, Jonathan Hubb, Joanna Ellis, Maria Zambon
EPI_ISL_423361 hCoV-19/England/20139070204/2020 Europe / United Kingdom / England 2020-03-29 Respiratory Virus Unit, Microbiology Services Colindale, Public Health England Respiratory Virus Unit, Microbiology Services Colindale, Public Health England Monica Galiano, Shahjahan Miah, Angie Lackenby, Omolola Akinbami, Tiina Talts, Leena Bhaw, Richard Myers, Steven Platt, Kirstin Edwards, Jonathan Hubb, Joanna Ellis,

Maria Zambon
EPI_ISL_423362 hCoV-19/England/20139070504/2020 Europe / United Kingdom / England 2020-03-28 Respiratory Virus Unit, Microbiology Services Colindale, Public Health England Respiratory Virus Unit, Microbiology Services Colindale, Public Health England Monica Galiano, Shahjahan Miah, Angie Lackenby, Omolola Akinbami, Tiina Talts, Leena Bhaw, Richard Myers, Steven Platt, Kirstin Edwards, Jonathan Hubb, Joanna Ellis, Maria Zambon
EPI_ISL_423363 hCoV-19/England/20139070704/2020 Europe / United Kingdom / England 2020-03-29 Respiratory Virus Unit, Microbiology Services Colindale, Public Health England Respiratory Virus Unit, Microbiology Services Colindale, Public Health England Monica Galiano, Shahjahan Miah, Angie Lackenby, Omolola Akinbami, Tiina Talts, Leena Bhaw, Richard Myers, Steven Platt, Kirstin Edwards, Jonathan Hubb, Joanna Ellis, Maria Zambon
EPI_ISL_423364 hCoV-19/England/20139071704/2020 Europe / United Kingdom / England 2020-03-28 Respiratory Virus Unit, Microbiology Services Colindale, Public Health England Respiratory Virus Unit, Microbiology Services Colindale, Public Health England Monica Galiano, Shahjahan Miah, Angie Lackenby, Omolola Akinbami, Tiina Talts, Leena Bhaw, Richard Myers, Steven Platt, Kirstin Edwards, Jonathan Hubb, Joanna Ellis, Maria Zambon
EPI_ISL_423365 hCoV-19/England/20139072204/2020 Europe / United Kingdom / England 2020-03-29 Respiratory Virus Unit, Microbiology Services Colindale, Public Health England Respiratory Virus Unit, Microbiology Services Colindale, Public Health England Monica Galiano, Shahjahan Miah, Angie Lackenby, Omolola Akinbami, Tiina Talts, Leena Bhaw, Richard Myers, Steven Platt, Kirstin Edwards, Jonathan Hubb, Joanna Ellis, Maria Zambon
EPI_ISL_423366 hCoV-19/England/20140000304/2020 Europe / United Kingdom / England 2020-03-29 Respiratory Virus Unit, Microbiology Services Colindale, Public Health England Respiratory Virus Unit, Microbiology Services Colindale, Public Health England Monica Galiano, Shahjahan Miah, Angie Lackenby, Omolola Akinbami, Tiina Talts, Leena Bhaw, Richard Myers, Steven Platt, Kirstin Edwards, Jonathan Hubb, Joanna Ellis, Maria Zambon
EPI_ISL_423367 hCoV-19/England/20140000404/2020 Europe / United Kingdom / England 2020-03-29 Respiratory Virus Unit, Microbiology Services Colindale, Public Health England Respiratory Virus Unit, Microbiology Services Colindale, Public Health England Monica Galiano, Shahjahan Miah, Angie Lackenby, Omolola Akinbami, Tiina Talts, Leena Bhaw, Richard Myers, Steven Platt, Kirstin Edwards, Jonathan Hubb, Joanna Ellis, Maria Zambon
EPI_ISL_423368 hCoV-19/England/20140000604/2020 Europe / United Kingdom / England 2020-03-29 Respiratory Virus Unit, Microbiology Services Colindale, Public Health England Respiratory Virus Unit, Microbiology Services Colindale, Public Health England Monica Galiano, Shahjahan Miah, Angie Lackenby, Omolola Akinbami, Tiina Talts, Leena Bhaw, Richard Myers, Steven Platt, Kirstin Edwards, Jonathan Hubb, Joanna Ellis, Maria Zambon
EPI_ISL_423369 hCoV-19/England/20140000904/2020 Europe / United Kingdom / England 2020-03-29 Respiratory Virus Unit, Microbiology Services Colindale, Public Health England Respiratory Virus Unit, Microbiology Services Colindale, Public Health England Monica Galiano, Shahjahan Miah, Angie Lackenby, Omolola Akinbami, Tiina Talts, Leena Bhaw, Richard Myers, Steven Platt, Kirstin Edwards, Jonathan Hubb, Joanna Ellis, Maria Zambon
EPI_ISL_423403 hCoV-19/England/20140069104/2020 Europe / United Kingdom / England 2020-03-28 Respiratory Virus Unit, Microbiology Services Colindale, Public Health England Respiratory Virus Unit, Microbiology Services Colindale, Public Health England Monica Galiano, Shahjahan Miah, Angie Lackenby, Omolola Akinbami, Tiina Talts, Leena Bhaw, Richard Myers, Steven Platt, Kirstin Edwards, Jonathan Hubb, Joanna Ellis, Maria Zambon
EPI_ISL_423404 hCoV-19/England/20140069404/2020 Europe / United Kingdom / England 2020-03-30 Respiratory Virus Unit, Microbiology Services Colindale, Public Health England Respiratory Virus Unit, Microbiology Services Colindale, Public Health England Monica Galiano, Shahjahan Miah, Angie Lackenby, Omolola Akinbami, Tiina Talts, Leena Bhaw, Richard Myers, Steven Platt, Kirstin Edwards, Jonathan Hubb, Joanna Ellis, Maria Zambon
EPI_ISL_423405 hCoV-19/England/20140070004/2020 Europe / United Kingdom / England 2020-03-30 Respiratory Virus Unit, Microbiology Services Colindale, Public Health England Respiratory Virus Unit, Microbiology Services Colindale, Public Health England Monica Galiano, Shahjahan Miah, Angie Lackenby, Omolola Akinbami, Tiina Talts, Leena Bhaw, Richard Myers, Steven Platt, Kirstin Edwards, Jonathan Hubb, Joanna Ellis, Maria Zambon
EPI_ISL_423408 hCoV-19/England/20140070504/2020 Europe / United Kingdom / England 2020-03-30 Respiratory Virus Unit, Microbiology Services Colindale, Public Health England Respiratory Virus Unit, Microbiology Services Colindale, Public Health England Monica Galiano, Shahjahan Miah, Angie Lackenby, Omolola Akinbami, Tiina Talts, Leena Bhaw, Richard Myers, Steven Platt, Kirstin Edwards, Jonathan Hubb, Joanna Ellis, Maria Zambon
EPI_ISL_423409 hCoV-19/England/20140070604/2020 Europe / United Kingdom / England 2020-03-30 Respiratory Virus Unit, Microbiology Services Colindale, Public Health England Respiratory Virus Unit, Microbiology Services Colindale, Public Health England Monica Galiano, Shahjahan Miah, Angie Lackenby, Omolola Akinbami, Tiina Talts, Leena Bhaw, Richard Myers, Steven Platt, Kirstin Edwards, Jonathan Hubb, Joanna Ellis, Maria Zambon
EPI_ISL_423410 hCoV-19/England/20140070804/2020 Europe / United Kingdom / England 2020-03-30 Respiratory Virus Unit, Microbiology Services Colindale, Public Health England Respiratory Virus Unit, Microbiology Services Colindale, Public Health England Monica Galiano, Shahjahan Miah, Angie Lackenby, Omolola Akinbami, Tiina Talts, Leena Bhaw, Richard Myers, Steven Platt, Kirstin Edwards, Jonathan Hubb, Joanna Ellis,

Maria Zambon
EPI_ISL_423411 hCoV-19/England/20140071104/2020   Europe / United Kingdom / England   2020-03-28
Respiratory Virus Unit, Microbiology Services Colindale, Public Health England Respiratory Virus Unit,
Microbiology Services Colindale, Public Health England Monica Galiano, Shahjahan Miah, Angie Lackenby, Omolola
Akinbami, Tiina Talts, Leena Bhaw, Richard Myers, Steven Platt, Kirstin Edwards, Jonathan Hubb, Joanna Ellis,
Maria Zambon
EPI_ISL_423412 hCoV-19/England/20140071404/2020   Europe / United Kingdom / England   2020-03-29
Respiratory Virus Unit, Microbiology Services Colindale, Public Health England Respiratory Virus Unit,
Microbiology Services Colindale, Public Health England Monica Galiano, Shahjahan Miah, Angie Lackenby, Omolola
Akinbami, Tiina Talts, Leena Bhaw, Richard Myers, Steven Platt, Kirstin Edwards, Jonathan Hubb, Joanna Ellis,
Maria Zambon
EPI_ISL_423413 hCoV-19/England/20140071504/2020   Europe / United Kingdom / England   2020-03-29
Respiratory Virus Unit, Microbiology Services Colindale, Public Health England Respiratory Virus Unit,
Microbiology Services Colindale, Public Health England Monica Galiano, Shahjahan Miah, Angie Lackenby, Omolola
Akinbami, Tiina Talts, Leena Bhaw, Richard Myers, Steven Platt, Kirstin Edwards, Jonathan Hubb, Joanna Ellis,
Maria Zambon
EPI_ISL_423414 hCoV-19/England/20140071604/2020   Europe / United Kingdom / England   2020-03-27
Respiratory Virus Unit, Microbiology Services Colindale, Public Health England Respiratory Virus Unit,
Microbiology Services Colindale, Public Health England Monica Galiano, Shahjahan Miah, Angie Lackenby, Omolola
Akinbami, Tiina Talts, Leena Bhaw, Richard Myers, Steven Platt, Kirstin Edwards, Jonathan Hubb, Joanna Ellis,
Maria Zambon
EPI_ISL_423415 hCoV-19/England/20140071704/2020   Europe / United Kingdom / England   2020-03-29
Respiratory Virus Unit, Microbiology Services Colindale, Public Health England Respiratory Virus Unit,
Microbiology Services Colindale, Public Health England Monica Galiano, Shahjahan Miah, Angie Lackenby, Omolola
Akinbami, Tiina Talts, Leena Bhaw, Richard Myers, Steven Platt, Kirstin Edwards, Jonathan Hubb, Joanna Ellis,
Maria Zambon
EPI_ISL_423417 hCoV-19/England/20140072304/2020   Europe / United Kingdom / England   2020-03-28
Respiratory Virus Unit, Microbiology Services Colindale, Public Health England Respiratory Virus Unit,
Microbiology Services Colindale, Public Health England Monica Galiano, Shahjahan Miah, Angie Lackenby, Omolola
Akinbami, Tiina Talts, Leena Bhaw, Richard Myers, Steven Platt, Kirstin Edwards, Jonathan Hubb, Joanna Ellis,
Maria Zambon
EPI_ISL_423418 hCoV-19/England/20140072504/2020   Europe / United Kingdom / England   2020-03-28
Respiratory Virus Unit, Microbiology Services Colindale, Public Health England Respiratory Virus Unit,
Microbiology Services Colindale, Public Health England Monica Galiano, Shahjahan Miah, Angie Lackenby, Omolola
Akinbami, Tiina Talts, Leena Bhaw, Richard Myers, Steven Platt, Kirstin Edwards, Jonathan Hubb, Joanna Ellis,
Maria Zambon
EPI_ISL_423419 hCoV-19/England/20140073904/2020   Europe / United Kingdom / England   2020-03-30
Respiratory Virus Unit, Microbiology Services Colindale, Public Health England Respiratory Virus Unit,
Microbiology Services Colindale, Public Health England Monica Galiano, Shahjahan Miah, Angie Lackenby, Omolola
Akinbami, Tiina Talts, Leena Bhaw, Richard Myers, Steven Platt, Kirstin Edwards, Jonathan Hubb, Joanna Ellis,
Maria Zambon
EPI_ISL_423420 hCoV-19/England/20140074104/2020   Europe / United Kingdom / England   2020-03-29
Respiratory Virus Unit, Microbiology Services Colindale, Public Health England Respiratory Virus Unit,
Microbiology Services Colindale, Public Health England Monica Galiano, Shahjahan Miah, Angie Lackenby, Omolola
Akinbami, Tiina Talts, Leena Bhaw, Richard Myers, Steven Platt, Kirstin Edwards, Jonathan Hubb, Joanna Ellis,
Maria Zambon
EPI_ISL_423421 hCoV-19/England/20142002004/2020   Europe / United Kingdom / England   2020-03-30
Respiratory Virus Unit, Microbiology Services Colindale, Public Health England Respiratory Virus Unit,
Microbiology Services Colindale, Public Health England Monica Galiano, Shahjahan Miah, Angie Lackenby, Omolola
Akinbami, Tiina Talts, Leena Bhaw, Richard Myers, Steven Platt, Kirstin Edwards, Jonathan Hubb, Joanna Ellis,
Maria Zambon
EPI_ISL_423422 hCoV-19/England/20142002104/2020   Europe / United Kingdom / England   2020-03-30
Respiratory Virus Unit, Microbiology Services Colindale, Public Health England Respiratory Virus Unit,
Microbiology Services Colindale, Public Health England Monica Galiano, Shahjahan Miah, Angie Lackenby, Omolola
Akinbami, Tiina Talts, Leena Bhaw, Richard Myers, Steven Platt, Kirstin Edwards, Jonathan Hubb, Joanna Ellis,
Maria Zambon
EPI_ISL_423423 hCoV-19/England/20142002204/2020   Europe / United Kingdom / England   2020-03-30
Respiratory Virus Unit, Microbiology Services Colindale, Public Health England Respiratory Virus Unit,
Microbiology Services Colindale, Public Health England Monica Galiano, Shahjahan Miah, Angie Lackenby, Omolola
Akinbami, Tiina Talts, Leena Bhaw, Richard Myers, Steven Platt, Kirstin Edwards, Jonathan Hubb, Joanna Ellis,
Maria Zambon
EPI_ISL_423424 hCoV-19/England/20142002704/2020   Europe / United Kingdom / England   2020-03-30
Respiratory Virus Unit, Microbiology Services Colindale, Public Health England Respiratory Virus Unit,
Microbiology Services Colindale, Public Health England Monica Galiano, Shahjahan Miah, Angie Lackenby, Omolola
Akinbami, Tiina Talts, Leena Bhaw, Richard Myers, Steven Platt, Kirstin Edwards, Jonathan Hubb, Joanna Ellis,
Maria Zambon
EPI_ISL_423425 hCoV-19/England/20142002904/2020   Europe / United Kingdom / England   2020-03-30
Respiratory Virus Unit, Microbiology Services Colindale, Public Health England Respiratory Virus Unit,
Microbiology Services Colindale, Public Health England Monica Galiano, Shahjahan Miah, Angie Lackenby, Omolola
Akinbami, Tiina Talts, Leena Bhaw, Richard Myers, Steven Platt, Kirstin Edwards, Jonathan Hubb, Joanna Ellis,

Maria Zambon
EPI_ISL_423426 hCoV-19/England/20142003904/2020   Europe / United Kingdom / England   2020-03-30
Respiratory Virus Unit, Microbiology Services Colindale, Public Health England Respiratory Virus Unit, Microbiology Services Colindale, Public Health England Monica Galiano, Shahjahan Miah, Angie Lackenby, Omolola Akinbami, Tiina Talts, Leena Bhaw, Richard Myers, Steven Platt, Kirstin Edwards, Jonathan Hubb, Joanna Ellis, Maria Zambon
EPI_ISL_423428 hCoV-19/England/20142012304/2020   Europe / United Kingdom / England   2020-03-30
Respiratory Virus Unit, Microbiology Services Colindale, Public Health England Respiratory Virus Unit, Microbiology Services Colindale, Public Health England Monica Galiano, Shahjahan Miah, Angie Lackenby, Omolola Akinbami, Tiina Talts, Leena Bhaw, Richard Myers, Steven Platt, Kirstin Edwards, Jonathan Hubb, Joanna Ellis, Maria Zambon
EPI_ISL_423430 hCoV-19/England/20142014504/2020   Europe / United Kingdom / England   2020-03-31
Respiratory Virus Unit, Microbiology Services Colindale, Public Health England Respiratory Virus Unit, Microbiology Services Colindale, Public Health England Monica Galiano, Shahjahan Miah, Angie Lackenby, Omolola Akinbami, Tiina Talts, Leena Bhaw, Richard Myers, Steven Platt, Kirstin Edwards, Jonathan Hubb, Joanna Ellis, Maria Zambon
EPI_ISL_423432 hCoV-19/England/20142015104/2020   Europe / United Kingdom / England   2020-03-29
Respiratory Virus Unit, Microbiology Services Colindale, Public Health England Respiratory Virus Unit, Microbiology Services Colindale, Public Health England Monica Galiano, Shahjahan Miah, Angie Lackenby, Omolola Akinbami, Tiina Talts, Leena Bhaw, Richard Myers, Steven Platt, Kirstin Edwards, Jonathan Hubb, Joanna Ellis, Maria Zambon
EPI_ISL_423436 hCoV-19/England/20142016504/2020   Europe / United Kingdom / England   2020-03-30
Respiratory Virus Unit, Microbiology Services Colindale, Public Health England Respiratory Virus Unit, Microbiology Services Colindale, Public Health England Monica Galiano, Shahjahan Miah, Angie Lackenby, Omolola Akinbami, Tiina Talts, Leena Bhaw, Richard Myers, Steven Platt, Kirstin Edwards, Jonathan Hubb, Joanna Ellis, Maria Zambon
EPI_ISL_423437 hCoV-19/England/20142016704/2020   Europe / United Kingdom / England   2020-03-30
Respiratory Virus Unit, Microbiology Services Colindale, Public Health England Respiratory Virus Unit, Microbiology Services Colindale, Public Health England Monica Galiano, Shahjahan Miah, Angie Lackenby, Omolola Akinbami, Tiina Talts, Leena Bhaw, Richard Myers, Steven Platt, Kirstin Edwards, Jonathan Hubb, Joanna Ellis, Maria Zambon
EPI_ISL_423438 hCoV-19/England/20142018004/2020   Europe / United Kingdom / England   2020-03-30
Respiratory Virus Unit, Microbiology Services Colindale, Public Health England Respiratory Virus Unit, Microbiology Services Colindale, Public Health England Monica Galiano, Shahjahan Miah, Angie Lackenby, Omolola Akinbami, Tiina Talts, Leena Bhaw, Richard Myers, Steven Platt, Kirstin Edwards, Jonathan Hubb, Joanna Ellis, Maria Zambon
EPI_ISL_423440 hCoV-19/England/20142018604/2020   Europe / United Kingdom / England   2020-03-30
Respiratory Virus Unit, Microbiology Services Colindale, Public Health England Respiratory Virus Unit, Microbiology Services Colindale, Public Health England Monica Galiano, Shahjahan Miah, Angie Lackenby, Omolola Akinbami, Tiina Talts, Leena Bhaw, Richard Myers, Steven Platt, Kirstin Edwards, Jonathan Hubb, Joanna Ellis, Maria Zambon
EPI_ISL_423441 hCoV-19/England/20142018804/2020   Europe / United Kingdom / England   2020-03-30
Respiratory Virus Unit, Microbiology Services Colindale, Public Health England Respiratory Virus Unit, Microbiology Services Colindale, Public Health England Monica Galiano, Shahjahan Miah, Angie Lackenby, Omolola Akinbami, Tiina Talts, Leena Bhaw, Richard Myers, Steven Platt, Kirstin Edwards, Jonathan Hubb, Joanna Ellis, Maria Zambon
EPI_ISL_423442 hCoV-19/England/20142019004/2020   Europe / United Kingdom / England   2020-03-30
Respiratory Virus Unit, Microbiology Services Colindale, Public Health England Respiratory Virus Unit, Microbiology Services Colindale, Public Health England Monica Galiano, Shahjahan Miah, Angie Lackenby, Omolola Akinbami, Tiina Talts, Leena Bhaw, Richard Myers, Steven Platt, Kirstin Edwards, Jonathan Hubb, Joanna Ellis, Maria Zambon
EPI_ISL_423443 hCoV-19/England/20142019504/2020   Europe / United Kingdom / England   2020-03-30
Respiratory Virus Unit, Microbiology Services Colindale, Public Health England Respiratory Virus Unit, Microbiology Services Colindale, Public Health England Monica Galiano, Shahjahan Miah, Angie Lackenby, Omolola Akinbami, Tiina Talts, Leena Bhaw, Richard Myers, Steven Platt, Kirstin Edwards, Jonathan Hubb, Joanna Ellis, Maria Zambon
EPI_ISL_423444 hCoV-19/England/20142019704/2020   Europe / United Kingdom / England   2020-03-30
Respiratory Virus Unit, Microbiology Services Colindale, Public Health England Respiratory Virus Unit, Microbiology Services Colindale, Public Health England Monica Galiano, Shahjahan Miah, Angie Lackenby, Omolola Akinbami, Tiina Talts, Leena Bhaw, Richard Myers, Steven Platt, Kirstin Edwards, Jonathan Hubb, Joanna Ellis, Maria Zambon
EPI_ISL_423446 hCoV-19/England/20142021604/2020   Europe / United Kingdom / England   2020-03-29
Respiratory Virus Unit, Microbiology Services Colindale, Public Health England Respiratory Virus Unit, Microbiology Services Colindale, Public Health England Monica Galiano, Shahjahan Miah, Angie Lackenby, Omolola Akinbami, Tiina Talts, Leena Bhaw, Richard Myers, Steven Platt, Kirstin Edwards, Jonathan Hubb, Joanna Ellis, Maria Zambon
EPI_ISL_423447 hCoV-19/England/20142022104/2020   Europe / United Kingdom / England   2020-03-29
Respiratory Virus Unit, Microbiology Services Colindale, Public Health England Respiratory Virus Unit, Microbiology Services Colindale, Public Health England Monica Galiano, Shahjahan Miah, Angie Lackenby, Omolola Akinbami, Tiina Talts, Leena Bhaw, Richard Myers, Steven Platt, Kirstin Edwards, Jonathan Hubb, Joanna Ellis,

Maria Zambon
EPI_ISL_423448 hCoV-19/England/20142023704/2020 Europe / United Kingdom / England 2020-03-31 Respiratory Virus Unit, Microbiology Services Colindale, Public Health England Respiratory Virus Unit, Microbiology Services Colindale, Public Health England Monica Galiano, Shahjahan Miah, Angie Lackenby, Omolola Akinbami, Tiina Talts, Leena Bhaw, Richard Myers, Steven Platt, Kirstin Edwards, Jonathan Hubb, Joanna Ellis, Maria Zambon
EPI_ISL_423449 hCoV-19/England/20142023804/2020 Europe / United Kingdom / England 2020-03-29 Respiratory Virus Unit, Microbiology Services Colindale, Public Health England Respiratory Virus Unit, Microbiology Services Colindale, Public Health England Monica Galiano, Shahjahan Miah, Angie Lackenby, Omolola Akinbami, Tiina Talts, Leena Bhaw, Richard Myers, Steven Platt, Kirstin Edwards, Jonathan Hubb, Joanna Ellis, Maria Zambon
EPI_ISL_423450 hCoV-19/England/20142023904/2020 Europe / United Kingdom / England 2020-03-29 Respiratory Virus Unit, Microbiology Services Colindale, Public Health England Respiratory Virus Unit, Microbiology Services Colindale, Public Health England Monica Galiano, Shahjahan Miah, Angie Lackenby, Omolola Akinbami, Tiina Talts, Leena Bhaw, Richard Myers, Steven Platt, Kirstin Edwards, Jonathan Hubb, Joanna Ellis, Maria Zambon
EPI_ISL_423451 hCoV-19/England/20142024404/2020 Europe / United Kingdom / England 2020-03-29 Respiratory Virus Unit, Microbiology Services Colindale, Public Health England Respiratory Virus Unit, Microbiology Services Colindale, Public Health England Monica Galiano, Shahjahan Miah, Angie Lackenby, Omolola Akinbami, Tiina Talts, Leena Bhaw, Richard Myers, Steven Platt, Kirstin Edwards, Jonathan Hubb, Joanna Ellis, Maria Zambon
EPI_ISL_423452 hCoV-19/England/20142024704/2020 Europe / United Kingdom / England 2020-03-30 Respiratory Virus Unit, Microbiology Services Colindale, Public Health England Respiratory Virus Unit, Microbiology Services Colindale, Public Health England Monica Galiano, Shahjahan Miah, Angie Lackenby, Omolola Akinbami, Tiina Talts, Leena Bhaw, Richard Myers, Steven Platt, Kirstin Edwards, Jonathan Hubb, Joanna Ellis, Maria Zambon
EPI_ISL_423453 hCoV-19/England/20142026304/2020 Europe / United Kingdom / England 2020-03-29 Respiratory Virus Unit, Microbiology Services Colindale, Public Health England Respiratory Virus Unit, Microbiology Services Colindale, Public Health England Monica Galiano, Shahjahan Miah, Angie Lackenby, Omolola Akinbami, Tiina Talts, Leena Bhaw, Richard Myers, Steven Platt, Kirstin Edwards, Jonathan Hubb, Joanna Ellis, Maria Zambon
EPI_ISL_423455 hCoV-19/England/20142026804/2020 Europe / United Kingdom / England 2020-03-29 Respiratory Virus Unit, Microbiology Services Colindale, Public Health England Respiratory Virus Unit, Microbiology Services Colindale, Public Health England Monica Galiano, Shahjahan Miah, Angie Lackenby, Omolola Akinbami, Tiina Talts, Leena Bhaw, Richard Myers, Steven Platt, Kirstin Edwards, Jonathan Hubb, Joanna Ellis, Maria Zambon
EPI_ISL_423457 hCoV-19/England/20142053204/2020 Europe / United Kingdom / England 2020-03-28 Respiratory Virus Unit, Microbiology Services Colindale, Public Health England Respiratory Virus Unit, Microbiology Services Colindale, Public Health England Monica Galiano, Shahjahan Miah, Angie Lackenby, Omolola Akinbami, Tiina Talts, Leena Bhaw, Richard Myers, Steven Platt, Kirstin Edwards, Jonathan Hubb, Joanna Ellis, Maria Zambon
EPI_ISL_423458 hCoV-19/England/20142053804/2020 Europe / United Kingdom / England 2020-03-28 Respiratory Virus Unit, Microbiology Services Colindale, Public Health England Respiratory Virus Unit, Microbiology Services Colindale, Public Health England Monica Galiano, Shahjahan Miah, Angie Lackenby, Omolola Akinbami, Tiina Talts, Leena Bhaw, Richard Myers, Steven Platt, Kirstin Edwards, Jonathan Hubb, Joanna Ellis, Maria Zambon
EPI_ISL_423459 hCoV-19/England/20142078304/2020 Europe / United Kingdom / England 2020-03-30 Respiratory Virus Unit, Microbiology Services Colindale, Public Health England Respiratory Virus Unit, Microbiology Services Colindale, Public Health England Monica Galiano, Shahjahan Miah, Angie Lackenby, Omolola Akinbami, Tiina Talts, Leena Bhaw, Richard Myers, Steven Platt, Kirstin Edwards, Jonathan Hubb, Joanna Ellis, Maria Zambon
EPI_ISL_423460 hCoV-19/England/20142079504/2020 Europe / United Kingdom / England 2020-03-30 Respiratory Virus Unit, Microbiology Services Colindale, Public Health England Respiratory Virus Unit, Microbiology Services Colindale, Public Health England Monica Galiano, Shahjahan Miah, Angie Lackenby, Omolola Akinbami, Tiina Talts, Leena Bhaw, Richard Myers, Steven Platt, Kirstin Edwards, Jonathan Hubb, Joanna Ellis, Maria Zambon
EPI_ISL_423461 hCoV-19/England/20142079804/2020 Europe / United Kingdom / England 2020-03-30 Respiratory Virus Unit, Microbiology Services Colindale, Public Health England Respiratory Virus Unit, Microbiology Services Colindale, Public Health England Monica Galiano, Shahjahan Miah, Angie Lackenby, Omolola Akinbami, Tiina Talts, Leena Bhaw, Richard Myers, Steven Platt, Kirstin Edwards, Jonathan Hubb, Joanna Ellis, Maria Zambon
EPI_ISL_423463 hCoV-19/England/20142082704/2020 Europe / United Kingdom / England 2020-03-30 Respiratory Virus Unit, Microbiology Services Colindale, Public Health England Respiratory Virus Unit, Microbiology Services Colindale, Public Health England Monica Galiano, Shahjahan Miah, Angie Lackenby, Omolola Akinbami, Tiina Talts, Leena Bhaw, Richard Myers, Steven Platt, Kirstin Edwards, Jonathan Hubb, Joanna Ellis, Maria Zambon
EPI_ISL_423465 hCoV-19/England/20142083604/2020 Europe / United Kingdom / England 2020-03-30 Respiratory Virus Unit, Microbiology Services Colindale, Public Health England Respiratory Virus Unit, Microbiology Services Colindale, Public Health England Monica Galiano, Shahjahan Miah, Angie Lackenby, Omolola Akinbami, Tiina Talts, Leena Bhaw, Richard Myers, Steven Platt, Kirstin Edwards, Jonathan Hubb, Joanna Ellis,

Maria Zambon
EPI_ISL_423466 hCoV-19/England/20142083804/2020   Europe / United Kingdom / England   2020-03-30
Respiratory Virus Unit, Microbiology Services Colindale, Public Health England Respiratory Virus Unit, Microbiology Services Colindale, Public Health England Monica Galiano, Shahjahan Miah, Angie Lackenby, Omolola Akinbami, Tiina Talts, Leena Bhaw, Richard Myers, Steven Platt, Kirstin Edwards, Jonathan Hubb, Joanna Ellis, Maria Zambon
EPI_ISL_423467 hCoV-19/England/20142130404/2020   Europe / United Kingdom / England   2020-03-30
Respiratory Virus Unit, Microbiology Services Colindale, Public Health England Respiratory Virus Unit, Microbiology Services Colindale, Public Health England Monica Galiano, Shahjahan Miah, Angie Lackenby, Omolola Akinbami, Tiina Talts, Leena Bhaw, Richard Myers, Steven Platt, Kirstin Edwards, Jonathan Hubb, Joanna Ellis, Maria Zambon
EPI_ISL_423469 hCoV-19/England/20142131504/2020   Europe / United Kingdom / England   2020-03-31
Respiratory Virus Unit, Microbiology Services Colindale, Public Health England Respiratory Virus Unit, Microbiology Services Colindale, Public Health England Monica Galiano, Shahjahan Miah, Angie Lackenby, Omolola Akinbami, Tiina Talts, Leena Bhaw, Richard Myers, Steven Platt, Kirstin Edwards, Jonathan Hubb, Joanna Ellis, Maria Zambon
EPI_ISL_423472 hCoV-19/England/20144000204/2020   Europe / United Kingdom / England   2020-03-31
Respiratory Virus Unit, Microbiology Services Colindale, Public Health England Respiratory Virus Unit, Microbiology Services Colindale, Public Health England Monica Galiano, Shahjahan Miah, Angie Lackenby, Omolola Akinbami, Tiina Talts, Leena Bhaw, Richard Myers, Steven Platt, Kirstin Edwards, Jonathan Hubb, Joanna Ellis, Maria Zambon
EPI_ISL_423473 hCoV-19/England/20144000404/2020   Europe / United Kingdom / England   2020-03-31
Respiratory Virus Unit, Microbiology Services Colindale, Public Health England Respiratory Virus Unit, Microbiology Services Colindale, Public Health England Monica Galiano, Shahjahan Miah, Angie Lackenby, Omolola Akinbami, Tiina Talts, Leena Bhaw, Richard Myers, Steven Platt, Kirstin Edwards, Jonathan Hubb, Joanna Ellis, Maria Zambon
EPI_ISL_423476 hCoV-19/England/20144001204/2020   Europe / United Kingdom / England   2020-03-30
Respiratory Virus Unit, Microbiology Services Colindale, Public Health England Respiratory Virus Unit, Microbiology Services Colindale, Public Health England Monica Galiano, Shahjahan Miah, Angie Lackenby, Omolola Akinbami, Tiina Talts, Leena Bhaw, Richard Myers, Steven Platt, Kirstin Edwards, Jonathan Hubb, Joanna Ellis, Maria Zambon
EPI_ISL_423477 hCoV-19/England/20144001404/2020   Europe / United Kingdom / England   2020-03-30
Respiratory Virus Unit, Microbiology Services Colindale, Public Health England Respiratory Virus Unit, Microbiology Services Colindale, Public Health England Monica Galiano, Shahjahan Miah, Angie Lackenby, Omolola Akinbami, Tiina Talts, Leena Bhaw, Richard Myers, Steven Platt, Kirstin Edwards, Jonathan Hubb, Joanna Ellis, Maria Zambon
EPI_ISL_423479 hCoV-19/England/20144002904/2020   Europe / United Kingdom / England   2020-03-31
Respiratory Virus Unit, Microbiology Services Colindale, Public Health England Respiratory Virus Unit, Microbiology Services Colindale, Public Health England Monica Galiano, Shahjahan Miah, Angie Lackenby, Omolola Akinbami, Tiina Talts, Leena Bhaw, Richard Myers, Steven Platt, Kirstin Edwards, Jonathan Hubb, Joanna Ellis, Maria Zambon
EPI_ISL_423480 hCoV-19/England/20144003404/2020   Europe / United Kingdom / England   2020-03-31
Respiratory Virus Unit, Microbiology Services Colindale, Public Health England Respiratory Virus Unit, Microbiology Services Colindale, Public Health England Monica Galiano, Shahjahan Miah, Angie Lackenby, Omolola Akinbami, Tiina Talts, Leena Bhaw, Richard Myers, Steven Platt, Kirstin Edwards, Jonathan Hubb, Joanna Ellis, Maria Zambon
EPI_ISL_423481 hCoV-19/England/20144003604/2020   Europe / United Kingdom / England   2020-03-31
Respiratory Virus Unit, Microbiology Services Colindale, Public Health England Respiratory Virus Unit, Microbiology Services Colindale, Public Health England Monica Galiano, Shahjahan Miah, Angie Lackenby, Omolola Akinbami, Tiina Talts, Leena Bhaw, Richard Myers, Steven Platt, Kirstin Edwards, Jonathan Hubb, Joanna Ellis, Maria Zambon
EPI_ISL_423483 hCoV-19/England/20144003804/2020   Europe / United Kingdom / England   2020-04-01
Respiratory Virus Unit, Microbiology Services Colindale, Public Health England Respiratory Virus Unit, Microbiology Services Colindale, Public Health England Monica Galiano, Shahjahan Miah, Angie Lackenby, Omolola Akinbami, Tiina Talts, Leena Bhaw, Richard Myers, Steven Platt, Kirstin Edwards, Jonathan Hubb, Joanna Ellis, Maria Zambon
EPI_ISL_423486 hCoV-19/England/20144004504/2020   Europe / United Kingdom / England   2020-03-31
Respiratory Virus Unit, Microbiology Services Colindale, Public Health England Respiratory Virus Unit, Microbiology Services Colindale, Public Health England Monica Galiano, Shahjahan Miah, Angie Lackenby, Omolola Akinbami, Tiina Talts, Leena Bhaw, Richard Myers, Steven Platt, Kirstin Edwards, Jonathan Hubb, Joanna Ellis, Maria Zambon
EPI_ISL_423487 hCoV-19/England/20144004704/2020   Europe / United Kingdom / England   2020-03-31
Respiratory Virus Unit, Microbiology Services Colindale, Public Health England Respiratory Virus Unit, Microbiology Services Colindale, Public Health England Monica Galiano, Shahjahan Miah, Angie Lackenby, Omolola Akinbami, Tiina Talts, Leena Bhaw, Richard Myers, Steven Platt, Kirstin Edwards, Jonathan Hubb, Joanna Ellis, Maria Zambon
EPI_ISL_423488 hCoV-19/England/20144004804/2020   Europe / United Kingdom / England   2020-03-31
Respiratory Virus Unit, Microbiology Services Colindale, Public Health England Respiratory Virus Unit, Microbiology Services Colindale, Public Health England Monica Galiano, Shahjahan Miah, Angie Lackenby, Omolola Akinbami, Tiina Talts, Leena Bhaw, Richard Myers, Steven Platt, Kirstin Edwards, Jonathan Hubb, Joanna Ellis,

Maria Zambon
EPI_ISL_423491 hCoV-19/England/20144005904/2020 Europe / United Kingdom / England 2020-03-31 Respiratory Virus Unit, Microbiology Services Colindale, Public Health England Respiratory Virus Unit, Microbiology Services Colindale, Public Health England Monica Galiano, Shahjahan Miah, Angie Lackenby, Omolola Akinbami, Tiina Talts, Leena Bhaw, Richard Myers, Steven Platt, Kirstin Edwards, Jonathan Hubb, Joanna Ellis, Maria Zambon
EPI_ISL_423492 hCoV-19/England/20144006004/2020 Europe / United Kingdom / England 2020-03-31 Respiratory Virus Unit, Microbiology Services Colindale, Public Health England Respiratory Virus Unit, Microbiology Services Colindale, Public Health England Monica Galiano, Shahjahan Miah, Angie Lackenby, Omolola Akinbami, Tiina Talts, Leena Bhaw, Richard Myers, Steven Platt, Kirstin Edwards, Jonathan Hubb, Joanna Ellis, Maria Zambon
EPI_ISL_423493 hCoV-19/England/20144006304/2020 Europe / United Kingdom / England 2020-03-31 Respiratory Virus Unit, Microbiology Services Colindale, Public Health England Respiratory Virus Unit, Microbiology Services Colindale, Public Health England Monica Galiano, Shahjahan Miah, Angie Lackenby, Omolola Akinbami, Tiina Talts, Leena Bhaw, Richard Myers, Steven Platt, Kirstin Edwards, Jonathan Hubb, Joanna Ellis, Maria Zambon
EPI_ISL_423494 hCoV-19/England/20144006404/2020 Europe / United Kingdom / England 2020-03-31 Respiratory Virus Unit, Microbiology Services Colindale, Public Health England Respiratory Virus Unit, Microbiology Services Colindale, Public Health England Monica Galiano, Shahjahan Miah, Angie Lackenby, Omolola Akinbami, Tiina Talts, Leena Bhaw, Richard Myers, Steven Platt, Kirstin Edwards, Jonathan Hubb, Joanna Ellis, Maria Zambon
EPI_ISL_423495 hCoV-19/England/20144006704/2020 Europe / United Kingdom / England 2020-03-30 Respiratory Virus Unit, Microbiology Services Colindale, Public Health England Respiratory Virus Unit, Microbiology Services Colindale, Public Health England Monica Galiano, Shahjahan Miah, Angie Lackenby, Omolola Akinbami, Tiina Talts, Leena Bhaw, Richard Myers, Steven Platt, Kirstin Edwards, Jonathan Hubb, Joanna Ellis, Maria Zambon
EPI_ISL_423496 hCoV-19/England/20144006904/2020 Europe / United Kingdom / England 2020-03-30 Respiratory Virus Unit, Microbiology Services Colindale, Public Health England Respiratory Virus Unit, Microbiology Services Colindale, Public Health England Monica Galiano, Shahjahan Miah, Angie Lackenby, Omolola Akinbami, Tiina Talts, Leena Bhaw, Richard Myers, Steven Platt, Kirstin Edwards, Jonathan Hubb, Joanna Ellis, Maria Zambon
EPI_ISL_423497 hCoV-19/England/20144007604/2020 Europe / United Kingdom / England 2020-03-31 Respiratory Virus Unit, Microbiology Services Colindale, Public Health England Respiratory Virus Unit, Microbiology Services Colindale, Public Health England Monica Galiano, Shahjahan Miah, Angie Lackenby, Omolola Akinbami, Tiina Talts, Leena Bhaw, Richard Myers, Steven Platt, Kirstin Edwards, Jonathan Hubb, Joanna Ellis, Maria Zambon
EPI_ISL_423498 hCoV-19/England/20144008004/2020 Europe / United Kingdom / England 2020-03-31 Respiratory Virus Unit, Microbiology Services Colindale, Public Health England Respiratory Virus Unit, Microbiology Services Colindale, Public Health England Monica Galiano, Shahjahan Miah, Angie Lackenby, Omolola Akinbami, Tiina Talts, Leena Bhaw, Richard Myers, Steven Platt, Kirstin Edwards, Jonathan Hubb, Joanna Ellis, Maria Zambon
EPI_ISL_423499 hCoV-19/England/20144008204/2020 Europe / United Kingdom / England 2020-03-31 Respiratory Virus Unit, Microbiology Services Colindale, Public Health England Respiratory Virus Unit, Microbiology Services Colindale, Public Health England Monica Galiano, Shahjahan Miah, Angie Lackenby, Omolola Akinbami, Tiina Talts, Leena Bhaw, Richard Myers, Steven Platt, Kirstin Edwards, Jonathan Hubb, Joanna Ellis, Maria Zambon
EPI_ISL_423500 hCoV-19/England/20144008304/2020 Europe / United Kingdom / England 2020-03-31 Respiratory Virus Unit, Microbiology Services Colindale, Public Health England Respiratory Virus Unit, Microbiology Services Colindale, Public Health England Monica Galiano, Shahjahan Miah, Angie Lackenby, Omolola Akinbami, Tiina Talts, Leena Bhaw, Richard Myers, Steven Platt, Kirstin Edwards, Jonathan Hubb, Joanna Ellis, Maria Zambon
EPI_ISL_423502 hCoV-19/England/20144008904/2020 Europe / United Kingdom / England 2020-03-31 Respiratory Virus Unit, Microbiology Services Colindale, Public Health England Respiratory Virus Unit, Microbiology Services Colindale, Public Health England Monica Galiano, Shahjahan Miah, Angie Lackenby, Omolola Akinbami, Tiina Talts, Leena Bhaw, Richard Myers, Steven Platt, Kirstin Edwards, Jonathan Hubb, Joanna Ellis, Maria Zambon
EPI_ISL_423503 hCoV-19/England/20144009004/2020 Europe / United Kingdom / England 2020-03-31 Respiratory Virus Unit, Microbiology Services Colindale, Public Health England Respiratory Virus Unit, Microbiology Services Colindale, Public Health England Monica Galiano, Shahjahan Miah, Angie Lackenby, Omolola Akinbami, Tiina Talts, Leena Bhaw, Richard Myers, Steven Platt, Kirstin Edwards, Jonathan Hubb, Joanna Ellis, Maria Zambon
EPI_ISL_423504 hCoV-19/England/20144009104/2020 Europe / United Kingdom / England 2020-03-31 Respiratory Virus Unit, Microbiology Services Colindale, Public Health England Respiratory Virus Unit, Microbiology Services Colindale, Public Health England Monica Galiano, Shahjahan Miah, Angie Lackenby, Omolola Akinbami, Tiina Talts, Leena Bhaw, Richard Myers, Steven Platt, Kirstin Edwards, Jonathan Hubb, Joanna Ellis, Maria Zambon
EPI_ISL_423506 hCoV-19/England/20144011504/2020 Europe / United Kingdom / England 2020-03-30 Respiratory Virus Unit, Microbiology Services Colindale, Public Health England Respiratory Virus Unit, Microbiology Services Colindale, Public Health England Monica Galiano, Shahjahan Miah, Angie Lackenby, Omolola Akinbami, Tiina Talts, Leena Bhaw, Richard Myers, Steven Platt, Kirstin Edwards, Jonathan Hubb, Joanna Ellis,

Maria Zambon
EPI_ISL_423507 hCoV-19/England/20144012404/2020 Europe / United Kingdom / England 2020-03-30 Respiratory Virus Unit, Microbiology Services Colindale, Public Health England Respiratory Virus Unit, Microbiology Services Colindale, Public Health England Monica Galiano, Shahjahan Miah, Angie Lackenby, Omolola Akinbami, Tiina Talts, Leena Bhaw, Richard Myers, Steven Platt, Kirstin Edwards, Jonathan Hubb, Joanna Ellis, Maria Zambon
EPI_ISL_423508 hCoV-19/England/20144013704/2020 Europe / United Kingdom / England 2020-03-31 Respiratory Virus Unit, Microbiology Services Colindale, Public Health England Respiratory Virus Unit, Microbiology Services Colindale, Public Health England Monica Galiano, Shahjahan Miah, Angie Lackenby, Omolola Akinbami, Tiina Talts, Leena Bhaw, Richard Myers, Steven Platt, Kirstin Edwards, Jonathan Hubb, Joanna Ellis, Maria Zambon
EPI_ISL_423509 hCoV-19/England/20144014504/2020 Europe / United Kingdom / England 2020-03-31 Respiratory Virus Unit, Microbiology Services Colindale, Public Health England Respiratory Virus Unit, Microbiology Services Colindale, Public Health England Monica Galiano, Shahjahan Miah, Angie Lackenby, Omolola Akinbami, Tiina Talts, Leena Bhaw, Richard Myers, Steven Platt, Kirstin Edwards, Jonathan Hubb, Joanna Ellis, Maria Zambon
EPI_ISL_423512 hCoV-19/England/20144018904/2020 Europe / United Kingdom / England 2020-03-31 Respiratory Virus Unit, Microbiology Services Colindale, Public Health England Respiratory Virus Unit, Microbiology Services Colindale, Public Health England Monica Galiano, Shahjahan Miah, Angie Lackenby, Omolola Akinbami, Tiina Talts, Leena Bhaw, Richard Myers, Steven Platt, Kirstin Edwards, Jonathan Hubb, Joanna Ellis, Maria Zambon
EPI_ISL_424613 hCoV-19/Iceland/593/2020 Europe / Iceland / Reykjavik 2020-03-27 The National University Hospital of Iceland deCODE genetics Daniel F Gudbjartsson; Agnar Helgason; Hakon Jonsson; Olafur T Magnusson; Pall Melsted; Gudmundur L Norddahl; Jona Saemundsdottir; Asgeir Sigurdsson; Patrick Sulem; Arna B Agustsdottir; Berglind Eiriksdottir; Run Fridriksdottir; Elisabet E Gardarsdottir; Gudmundur Georgsson; Olafia S Gretarsdottir; Kjartan R Gudmundsson; Thora R Gunnarsdottir; Arnaldur Gylfason; Hilma Holm; Brynjar O Jensson; Aslaug Jonasdottir; Kamilla S Josefsdottir; Thordur Kristjansson; Droplaug N Magnusdottir; Louise le Roux; Gudrun Sigmundsdottir; Gardar Sveinbjornsson; Kristin E Sveinsdottir; Maney Sveinsdottir; Emil A Thorarensen; Bjarni Thorbjornsson; Gisli Masson; Ingileif Jonsdottir; Alma Moller; Thorolfur Gudnason; Karl G Kristinsson; Unnur Thorsteinsdottir; Kari Stefansson
EPI_ISL_424614 hCoV-19/Iceland/594/2020 Europe / Iceland / Reykjavik 2020-03-27 The National University Hospital of Iceland deCODE genetics Daniel F Gudbjartsson; Agnar Helgason; Hakon Jonsson; Olafur T Magnusson; Pall Melsted; Gudmundur L Norddahl; Jona Saemundsdottir; Asgeir Sigurdsson; Patrick Sulem; Arna B Agustsdottir; Berglind Eiriksdottir; Run Fridriksdottir; Elisabet E Gardarsdottir; Gudmundur Georgsson; Olafia S Gretarsdottir; Kjartan R Gudmundsson; Thora R Gunnarsdottir; Arnaldur Gylfason; Hilma Holm; Brynjar O Jensson; Aslaug Jonasdottir; Kamilla S Josefsdottir; Thordur Kristjansson; Droplaug N Magnusdottir; Louise le Roux; Gudrun Sigmundsdottir; Gardar Sveinbjornsson; Kristin E Sveinsdottir; Maney Sveinsdottir; Emil A Thorarensen; Bjarni Thorbjornsson; Gisli Masson; Ingileif Jonsdottir; Alma Moller; Thorolfur Gudnason; Karl G Kristinsson; Unnur Thorsteinsdottir; Kari Stefansson
EPI_ISL_424615 hCoV-19/Iceland/595/2020 Europe / Iceland / Reykjavik 2020-03-27 The National University Hospital of Iceland deCODE genetics Daniel F Gudbjartsson; Agnar Helgason; Hakon Jonsson; Olafur T Magnusson; Pall Melsted; Gudmundur L Norddahl; Jona Saemundsdottir; Asgeir Sigurdsson; Patrick Sulem; Arna B Agustsdottir; Berglind Eiriksdottir; Run Fridriksdottir; Elisabet E Gardarsdottir; Gudmundur Georgsson; Olafia S Gretarsdottir; Kjartan R Gudmundsson; Thora R Gunnarsdottir; Arnaldur Gylfason; Hilma Holm; Brynjar O Jensson; Aslaug Jonasdottir; Kamilla S Josefsdottir; Thordur Kristjansson; Droplaug N Magnusdottir; Louise le Roux; Gudrun Sigmundsdottir; Gardar Sveinbjornsson; Kristin E Sveinsdottir; Maney Sveinsdottir; Emil A Thorarensen; Bjarni Thorbjornsson; Gisli Masson; Ingileif Jonsdottir; Alma Moller; Thorolfur Gudnason; Karl G Kristinsson; Unnur Thorsteinsdottir; Kari Stefansson
EPI_ISL_424616 hCoV-19/Iceland/596/2020 Europe / Iceland / Reykjavik 2020-03-28 The National University Hospital of Iceland deCODE genetics Daniel F Gudbjartsson; Agnar Helgason; Hakon Jonsson; Olafur T Magnusson; Pall Melsted; Gudmundur L Norddahl; Jona Saemundsdottir; Asgeir Sigurdsson; Patrick Sulem; Arna B Agustsdottir; Berglind Eiriksdottir; Run Fridriksdottir; Elisabet E Gardarsdottir; Gudmundur Georgsson; Olafia S Gretarsdottir; Kjartan R Gudmundsson; Thora R Gunnarsdottir; Arnaldur Gylfason; Hilma Holm; Brynjar O Jensson; Aslaug Jonasdottir; Kamilla S Josefsdottir; Thordur Kristjansson; Droplaug N Magnusdottir; Louise le Roux; Gudrun Sigmundsdottir; Gardar Sveinbjornsson; Kristin E Sveinsdottir; Maney Sveinsdottir; Emil A Thorarensen; Bjarni Thorbjornsson; Gisli Masson; Ingileif Jonsdottir; Alma Moller; Thorolfur Gudnason; Karl G Kristinsson; Unnur Thorsteinsdottir; Kari Stefansson
EPI_ISL_424617 hCoV-19/Iceland/597/2020 Europe / Iceland / Reykjavik 2020-03-28 The National University Hospital of Iceland deCODE genetics Daniel F Gudbjartsson; Agnar Helgason; Hakon Jonsson; Olafur T Magnusson; Pall Melsted; Gudmundur L Norddahl; Jona Saemundsdottir; Asgeir Sigurdsson; Patrick Sulem; Arna B Agustsdottir; Berglind Eiriksdottir; Run Fridriksdottir; Elisabet E Gardarsdottir; Gudmundur Georgsson; Olafia S Gretarsdottir; Kjartan R Gudmundsson; Thora R Gunnarsdottir; Arnaldur Gylfason; Hilma Holm; Brynjar O Jensson; Aslaug Jonasdottir; Kamilla S Josefsdottir; Thordur Kristjansson; Droplaug N Magnusdottir; Louise le Roux; Gudrun Sigmundsdottir; Gardar Sveinbjornsson; Kristin E Sveinsdottir; Maney Sveinsdottir; Emil A Thorarensen; Bjarni Thorbjornsson; Gisli Masson; Ingileif Jonsdottir; Alma Moller; Thorolfur Gudnason; Karl G Kristinsson; Unnur Thorsteinsdottir; Kari Stefansson
EPI_ISL_424619 hCoV-19/Iceland/599/2020 Europe / Iceland / Reykjavik 2020-03-28 The National University Hospital of Iceland deCODE genetics Daniel F Gudbjartsson; Agnar Helgason; Hakon Jonsson; Olafur T Magnusson; Pall Melsted; Gudmundur L Norddahl; Jona Saemundsdottir; Asgeir Sigurdsson; Patrick Sulem; Arna B Agustsdottir; Berglind Eiriksdottir; Run Fridriksdottir; Elisabet E Gardarsdottir; Gudmundur Georgsson; Olafia

S Gretarsdottir; Kjartan R Gudmundsson; Thora R Gunnarsdottir; Arnaldur Gylfason; Hilma Holm; Brynjar O Jensson; Aslaug Jonasdottir; Kamilla S Josefsdottir; Thordur Kristjansson; Droplaug N Magnusdottir; Louise le Roux; Gudrun Sigmundsdottir; Gardar Sveinbjornsson; Kristin E Sveinsdottir; Maney Sveinsdottir; Emil A Thorarensen; Bjarni Thorbjornsson; Gisli Masson; Ingileif Jonsdottir; Alma Moller; Thorolfur Gudnason; Karl G Kristinsson; Unnur Thorsteinsdottir; Kari Stefansson
EPI_ISL_424621 hCoV-19/Iceland/601/2020 Europe / Iceland / Reykjavik 2020-03-28 The National University Hospital of Iceland deCODE genetics Daniel F Gudbjartsson; Agnar Helgason; Hakon Jonsson; Olafur T Magnusson; Pall Melsted; Gudmundur L Norddahl; Jona Saemundsdottir; Asgeir Sigurdsson; Patrick Sulem; Arna B Agustsdottir; Berglind Eiriksdottir; Run Fridriksdottir; Elisabet E Gardarsdottir; Gudmundur Georgsson; Olafia S Gretarsdottir; Kjartan R Gudmundsson; Thora R Gunnarsdottir; Arnaldur Gylfason; Hilma Holm; Brynjar O Jensson; Aslaug Jonasdottir; Kamilla S Josefsdottir; Thordur Kristjansson; Droplaug N Magnusdottir; Louise le Roux; Gudrun Sigmundsdottir; Gardar Sveinbjornsson; Kristin E Sveinsdottir; Maney Sveinsdottir; Emil A Thorarensen; Bjarni Thorbjornsson; Gisli Masson; Ingileif Jonsdottir; Alma Moller; Thorolfur Gudnason; Karl G Kristinsson; Unnur Thorsteinsdottir; Kari Stefansson
EPI_ISL_424622 hCoV-19/Iceland/602/2020 Europe / Iceland / Reykjavik 2020-03-28 The National University Hospital of Iceland deCODE genetics Daniel F Gudbjartsson; Agnar Helgason; Hakon Jonsson; Olafur T Magnusson; Pall Melsted; Gudmundur L Norddahl; Jona Saemundsdottir; Asgeir Sigurdsson; Patrick Sulem; Arna B Agustsdottir; Berglind Eiriksdottir; Run Fridriksdottir; Elisabet E Gardarsdottir; Gudmundur Georgsson; Olafia S Gretarsdottir; Kjartan R Gudmundsson; Thora R Gunnarsdottir; Arnaldur Gylfason; Hilma Holm; Brynjar O Jensson; Aslaug Jonasdottir; Kamilla S Josefsdottir; Thordur Kristjansson; Droplaug N Magnusdottir; Louise le Roux; Gudrun Sigmundsdottir; Gardar Sveinbjornsson; Kristin E Sveinsdottir; Maney Sveinsdottir; Emil A Thorarensen; Bjarni Thorbjornsson; Gisli Masson; Ingileif Jonsdottir; Alma Moller; Thorolfur Gudnason; Karl G Kristinsson; Unnur Thorsteinsdottir; Kari Stefansson
EPI_ISL_424624 hCoV-19/Iceland/604/2020 Europe / Iceland / Reykjavik 2020-03-28 The National University Hospital of Iceland deCODE genetics Daniel F Gudbjartsson; Agnar Helgason; Hakon Jonsson; Olafur T Magnusson; Pall Melsted; Gudmundur L Norddahl; Jona Saemundsdottir; Asgeir Sigurdsson; Patrick Sulem; Arna B Agustsdottir; Berglind Eiriksdottir; Run Fridriksdottir; Elisabet E Gardarsdottir; Gudmundur Georgsson; Olafia S Gretarsdottir; Kjartan R Gudmundsson; Thora R Gunnarsdottir; Arnaldur Gylfason; Hilma Holm; Brynjar O Jensson; Aslaug Jonasdottir; Kamilla S Josefsdottir; Thordur Kristjansson; Droplaug N Magnusdottir; Louise le Roux; Gudrun Sigmundsdottir; Gardar Sveinbjornsson; Kristin E Sveinsdottir; Maney Sveinsdottir; Emil A Thorarensen; Bjarni Thorbjornsson; Gisli Masson; Ingileif Jonsdottir; Alma Moller; Thorolfur Gudnason; Karl G Kristinsson; Unnur Thorsteinsdottir; Kari Stefansson
EPI_ISL_424626 hCoV-19/Mexico/CDMX-INER_04/2020 North America / Mexico / Mexico City 2020-03-15 Instituto Nacional de Enfermedades Respiratorias Instituto Nacional de Enfermedades Respiratorias Joel Armando Vázquez Pérez, Celia Boukadida, Santiago Avila Ríos, Mario Mújica Sánchez, José Arturo Martínez Orozco, Eduardo Becerril Vargas, Jorge Salas Hernández, Irma López Martínez, Lucia Hernández Rivas, Gisela Barrera Badillo, Edgar Mendieta Condado, Fabiola Garcés Ayala, Adnan Araiza Rodríguez, José Ernesto Ramírez González, Victor Hugo Borja Aburto, Concepción Grajales Muñiz, Cesar Raúl González Bonilla, Carolina González Torres, Francisco Javier Gaytán Cervantes, José Esteban Muñoz Medina, Guillermo M. Ruiz-Palacios, Pilar Ramos Cervantes, Violeta Ibarra Gonzalez, Fernando Ledesma Barrientos, Luis Alberto García Andrade, Alfredo Ponce de León Garduño, Blanca Taboada, Alejandro Sánchez, Pavel Isa, Ricardo Grande, Gloria Vázquez, Francisco Pulido, Carlos F. Arias.
EPI_ISL_424627 hCoV-19/Mexico/CDMX-INER_05/2020 North America / Mexico / Mexico City 2020-03-16 Instituto Nacional de Enfermedades Respiratorias Instituto Nacional de Enfermedades Respiratorias Joel Armando Vázquez Pérez, Celia Boukadida, Santiago Avila Ríos, Mario Mújica Sánchez, José Arturo Martínez Orozco, Eduardo Becerril Vargas, Jorge Salas Hernández, Irma López Martínez, Lucia Hernández Rivas, Gisela Barrera Badillo, Edgar Mendieta Condado, Fabiola Garcés Ayala, Adnan Araiza Rodríguez, José Ernesto Ramírez González, Victor Hugo Borja Aburto, Concepción Grajales Muñiz, Cesar Raúl González Bonilla, Carolina González Torres, Francisco Javier Gaytán Cervantes, José Esteban Muñoz Medina, Guillermo M. Ruiz-Palacios, Pilar Ramos Cervantes, Violeta Ibarra Gonzalez, Fernando Ledesma Barrientos, Luis Alberto García Andrade, Alfredo Ponce de León Garduño, Blanca Taboada, Alejandro Sánchez, Pavel Isa, Ricardo Grande, Gloria Vázquez, Francisco Pulido, Carlos F. Arias.
EPI_ISL_424629 hCoV-19/Belgium/ULG-10066/2020 Europe / Belgium / Liège 2020-04-03 Department of Clinical Microbiology GIGA Medical Genomics Keith Durkin, Maria Artesi, Sébastien Bontems, Raphaël Boreux, Cécile Meex, Pierrette Melin, Marie-Pierre Hayette, Vincent Bours.
EPI_ISL_424630 hCoV-19/Belgium/ULG-10067/2020 Europe / Belgium / Liège 2020-04-03 Department of Clinical Microbiology GIGA Medical Genomics Keith Durkin, Maria Artesi, Sébastien Bontems, Raphaël Boreux, Cécile Meex, Pierrette Melin, Marie-Pierre Hayette, Vincent Bours.
EPI_ISL_424631 hCoV-19/Belgium/ULG-10072/2020 Europe / Belgium / Liège 2020-04-03 Department of Clinical Microbiology GIGA Medical Genomics Keith Durkin, Maria Artesi, Sébastien Bontems, Raphaël Boreux, Cécile Meex, Pierrette Melin, Marie-Pierre Hayette, Vincent Bours.
EPI_ISL_424633 hCoV-19/Belgium/ULG-10069/2020 Europe / Belgium / Liège 2020-04-03 Department of Clinical Microbiology GIGA Medical Genomics Keith Durkin, Maria Artesi, Sébastien Bontems, Raphaël Boreux, Cécile Meex, Pierrette Melin, Marie-Pierre Hayette, Vincent Bours.
EPI_ISL_424636 hCoV-19/Belgium/ULG-10068/2020 Europe / Belgium / Liège 2020-04-03 Department of Clinical Microbiology GIGA Medical Genomics Keith Durkin, Maria Artesi, Sébastien Bontems, Raphaël Boreux, Cécile Meex, Pierrette Melin, Marie-Pierre Hayette, Vincent Bours.
EPI_ISL_424637 hCoV-19/Belgium/ULG-10078/2020 Europe / Belgium / Liège 2020-04-03 Department of Clinical Microbiology GIGA Medical Genomics Keith Durkin, Maria Artesi, Sébastien Bontems, Raphaël Boreux, Cécile Meex, Pierrette Melin, Marie-Pierre Hayette, Vincent Bours.

EPI_ISL_424640 hCoV-19/Belgium/ULG-10081/2020 Europe / Belgium / Liège 2020-04-03 Department of Clinical Microbiology GIGA Medical Genomics Keith Durkin, Maria Artesi, Sébastien Bontems, Raphaël Boreux, Cécile Meex, Pierrette Melin, Marie-Pierre Hayette, Vincent Bours.
EPI_ISL_424641 hCoV-19/Belgium/ULG-10080/2020 Europe / Belgium / Liège 2020-04-03 Department of Clinical Microbiology GIGA Medical Genomics Keith Durkin, Maria Artesi, Sébastien Bontems, Raphaël Boreux, Cécile Meex, Pierrette Melin, Marie-Pierre Hayette, Vincent Bours.
EPI_ISL_424642 hCoV-19/Belgium/ULG-10077/2020 Europe / Belgium / Liège 2020-04-04 Department of Clinical Microbiology GIGA Medical Genomics Keith Durkin, Maria Artesi, Sébastien Bontems, Raphaël Boreux, Cécile Meex, Pierrette Melin, Marie-Pierre Hayette, Vincent Bours.
EPI_ISL_424643 hCoV-19/Belgium/ULG-10089/2020 Europe / Belgium / Liège 2020-04-04 Department of Clinical Microbiology GIGA Medical Genomics Keith Durkin, Maria Artesi, Sébastien Bontems, Raphaël Boreux, Cécile Meex, Pierrette Melin, Marie-Pierre Hayette, Vincent Bours.
EPI_ISL_424644 hCoV-19/Belgium/ULG-10086/2020 Europe / Belgium / Liège 2020-04-04 Department of Clinical Microbiology GIGA Medical Genomics Keith Durkin, Maria Artesi, Sébastien Bontems, Raphaël Boreux, Cécile Meex, Pierrette Melin, Marie-Pierre Hayette, Vincent Bours.
EPI_ISL_424645 hCoV-19/Belgium/ULG-10085/2020 Europe / Belgium / Liège 2020-04-04 Department of Clinical Microbiology GIGA Medical Genomics Keith Durkin, Maria Artesi, Sébastien Bontems, Raphaël Boreux, Cécile Meex, Pierrette Melin, Marie-Pierre Hayette, Vincent Bours.
EPI_ISL_424646 hCoV-19/Belgium/ULG-10088/2020 Europe / Belgium / Liège 2020-04-04 Department of Clinical Microbiology GIGA Medical Genomics Keith Durkin, Maria Artesi, Sébastien Bontems, Raphaël Boreux, Cécile Meex, Pierrette Melin, Marie-Pierre Hayette, Vincent Bours.
EPI_ISL_424647 hCoV-19/Belgium/ULG-10092/2020 Europe / Belgium / Liège 2020-04-04 Department of Clinical Microbiology GIGA Medical Genomics Keith Durkin, Maria Artesi, Sébastien Bontems, Raphaël Boreux, Cécile Meex, Pierrette Melin, Marie-Pierre Hayette, Vincent Bours.
EPI_ISL_424649 hCoV-19/Belgium/ULG-10082/2020 Europe / Belgium / Liège 2020-04-04 Department of Clinical Microbiology GIGA Medical Genomics Keith Durkin, Maria Artesi, Sébastien Bontems, Raphaël Boreux, Cécile Meex, Pierrette Melin, Marie-Pierre Hayette, Vincent Bours.
EPI_ISL_424650 hCoV-19/Belgium/ULG-10094/2020 Europe / Belgium / Liège 2020-04-04 Department of Clinical Microbiology GIGA Medical Genomics Keith Durkin, Maria Artesi, Sébastien Bontems, Raphaël Boreux, Cécile Meex, Pierrette Melin, Marie-Pierre Hayette, Vincent Bours.
EPI_ISL_424651 hCoV-19/Belgium/ULG-10095/2020 Europe / Belgium / Liège 2020-04-05 Department of Clinical Microbiology GIGA Medical Genomics Keith Durkin, Maria Artesi, Sébastien Bontems, Raphaël Boreux, Cécile Meex, Pierrette Melin, Marie-Pierre Hayette, Vincent Bours.
EPI_ISL_424652 hCoV-19/Belgium/ULG-10055/2020 Europe / Belgium / Liège 2020-04-05 Department of Clinical Microbiology GIGA Medical Genomics Keith Durkin, Maria Artesi, Sébastien Bontems, Raphaël Boreux, Cécile Meex, Pierrette Melin, Marie-Pierre Hayette, Vincent Bours.
EPI_ISL_424653 hCoV-19/Belgium/ULG-10058/2020 Europe / Belgium / Liège 2020-04-05 Department of Clinical Microbiology GIGA Medical Genomics Keith Durkin, Maria Artesi, Sébastien Bontems, Raphaël Boreux, Cécile Meex, Pierrette Melin, Marie-Pierre Hayette, Vincent Bours.
EPI_ISL_424654 hCoV-19/Belgium/ULG-10057/2020 Europe / Belgium / Liège 2020-04-05 Department of Clinical Microbiology GIGA Medical Genomics Keith Durkin, Maria Artesi, Sébastien Bontems, Raphaël Boreux, Cécile Meex, Pierrette Melin, Marie-Pierre Hayette, Vincent Bours.
EPI_ISL_424655 hCoV-19/Belgium/ULG-10056/2020 Europe / Belgium / Liège 2020-04-05 Department of Clinical Microbiology GIGA Medical Genomics Keith Durkin, Maria Artesi, Sébastien Bontems, Raphaël Boreux, Cécile Meex, Pierrette Melin, Marie-Pierre Hayette, Vincent Bours.
EPI_ISL_424657 hCoV-19/Belgium/ULG-10052/2020 Europe / Belgium / Liège 2020-04-05 Department of Clinical Microbiology GIGA Medical Genomics Keith Durkin, Maria Artesi, Sébastien Bontems, Raphaël Boreux, Cécile Meex, Pierrette Melin, Marie-Pierre Hayette, Vincent Bours.
EPI_ISL_424658 hCoV-19/Belgium/ULG-10049/2020 Europe / Belgium / Liège 2020-04-05 Department of Clinical Microbiology GIGA Medical Genomics Keith Durkin, Maria Artesi, Sébastien Bontems, Raphaël Boreux, Cécile Meex, Pierrette Melin, Marie-Pierre Hayette, Vincent Bours.
EPI_ISL_424660 hCoV-19/Belgium/ULG-10051/2020 Europe / Belgium / Liège 2020-04-05 Department of Clinical Microbiology GIGA Medical Genomics Keith Durkin, Maria Artesi, Sébastien Bontems, Raphaël Boreux, Cécile Meex, Pierrette Melin, Marie-Pierre Hayette, Vincent Bours.
EPI_ISL_424661 hCoV-19/Belgium/ULG-10053/2020 Europe / Belgium / Liège 2020-04-05 Department of Clinical Microbiology GIGA Medical Genomics Keith Durkin, Maria Artesi, Sébastien Bontems, Raphaël Boreux, Cécile Meex, Pierrette Melin, Marie-Pierre Hayette, Vincent Bours.
EPI_ISL_424662 hCoV-19/Belgium/ULG-10061/2020 Europe / Belgium / Liège 2020-04-05 Department of Clinical Microbiology GIGA Medical Genomics Keith Durkin, Maria Artesi, Sébastien Bontems, Raphaël Boreux, Cécile Meex, Pierrette Melin, Marie-Pierre Hayette, Vincent Bours.
EPI_ISL_424664 hCoV-19/Belgium/ULG-10062/2020 Europe / Belgium / Liège 2020-04-05 Department of Clinical Microbiology GIGA Medical Genomics Keith Durkin, Maria Artesi, Sébastien Bontems, Raphaël Boreux, Cécile Meex, Pierrette Melin, Marie-Pierre Hayette, Vincent Bours.
EPI_ISL_424667 hCoV-19/Mexico/EdoMex-InDRE_03/2020 North America / Mexico / Estado de Mexico 2020-03-04 Laboratorio Estatal de Salud Publica del Estado de México Instituto de Diagnóstico y Referencia Epidemiológicos Irma López Martínez, José Ernesto Ramírez González, Lucia Hernández Rivas, Gisela Barrera Badillo, Edgar Mendieta Condado, Fabiola Garcés Ayala, Adnan Araiza Rodríguez, Celia Boukadida, Santiago Avila Ríos, Mario Mújica Sánchez, José Arturo Martínez Orozco,Eduardo Becerril Vargas, Joel Armando Vázquez Pérez, Victor Hugo Borja Aburto, Concepción Grajales Muñiz, Cesar Raúl González Bonilla, Carolina González Torres, Francisco Javier Gaytán Cervantes, José Esteban Muñoz Medina, Guillermo M. Ruiz-Palacios, Pilar Ramos

Cervantes, Violeta Ibarra Gonzalez, Fernando Ledesma Barrientos, Luis Alberto García Andrade, Alfredo Ponce de León Garduño, Blanca Taboada, Alejandro Sánchez, Pavel Isa, Ricardo Grande, Gloria Vázquez, Francisco Pulido, Carlos F. Arias.
EPI_ISL_424668 hCoV-19/USA/AZ-ASU2922/2020 North America / USA / Arizona 2020-03-16 Arizona State University Health Services Arizona State University Rabia Maqsood, LaRinda A. Holland, Emily A. Kaelin, Bereket Estifanos, Nicholas J. Mellor, Jason Steel, Lily I. Wu, Arvind Varsani, Rolf U. Halden, Brenda G. Hogue, Matthew Scotch, Efrem S. Lim
EPI_ISL_424669 hCoV-19/USA/AZ-ASU2923/2020 North America / USA / Arizona 2020-03-16 Arizona State University Health Services Arizona State University Rabia Maqsood, LaRinda A. Holland, Emily A. Kaelin, Bereket Estifanos, Nicholas J. Mellor, Jason Steel, Lily I. Wu, Arvind Varsani, Rolf U. Halden, Brenda G. Hogue, Matthew Scotch, Efrem S. Lim
EPI_ISL_424670 hCoV-19/Mexico/Queretaro-InDRE_04/2020 North America / Mexico / Queretaro 2020-03-10 Laboratorio Estatal de Salud Publica del Estado de Queretaro Instituto de Diagnóstico y Referencia Epidemiologicos Gisela Barrera Badillo, Irma López Martínez, Lucia Hernández Rivas, Edgar Mendieta Condado, Fabiola Garcés Ayala, Adnan Araiza Rodríguez, Celia Boukadida, Santiago Avila Ríos, Mario Mújica Sánchez, José Arturo Martínez Orozco,Eduardo Becerril Vargas, Joel Armando Vázquez Pérez, Victor Hugo Borja Aburto, Concepción Grajales Muñiz, Cesar Raúl González Bonilla, Carolina González Torres, Francisco Javier Gaytán Cervantes, José Esteban Muñoz Medina, Guillermo M. Ruiz-Palacios, Pilar Ramos Cervantes, Violeta Ibarra Gonzalez, Fernando Ledesma Barrientos, Luis Alberto García Andrade, Alfredo Ponce de León Garduño, Blanca Taboada, Alejandro Sánchez, Pavel Isa, Ricardo Grande, Gloria Vázquez, Francisco Pulido, Carlos F. Arias,José Ernesto Ramírez González
EPI_ISL_424671 hCoV-19/USA/AZ-ASU2936/2020 North America / USA / Arizona 2020-03-17 Arizona State University Health Services Arizona State University Rabia Maqsood, LaRinda A. Holland, Emily A. Kaelin, Bereket Estifanos, Nicholas J. Mellor, Jason Steel, Lily I. Wu, Arvind Varsani, Rolf U. Halden, Brenda G. Hogue, Matthew Scotch, Efrem S. Lim
EPI_ISL_424672 hCoV-19/Mexico/Puebla-InDRE_05/2020 North America / Mexico / Puebla 2020-03-11 Laboratorio Estatal de Salud Publica del Estado de Puebla Instituto de Diagnostico y Referencia Epidemiologicos Fabiola Garcés Ayala, Gisela Barrera Badillo, Irma López Martínez, Lucia Hernández Rivas, Edgar Mendieta Condado, Adnan Araiza Rodríguez, Celia Boukadida, Santiago Avila Ríos, Mario Mújica Sánchez, José Arturo Martínez Orozco,Eduardo Becerril Vargas, Joel Armando Vázquez Pérez, Victor Hugo Borja Aburto, Concepción Grajales Muñiz, Cesar Raúl González Bonilla, Carolina González Torres, Francisco Javier Gaytán Cervantes, José Esteban Muñoz Medina, Guillermo M. Ruiz-Palacios, Pilar Ramos Cervantes, Violeta Ibarra Gonzalez, Fernando Ledesma Barrientos, Luis Alberto García Andrade, Alfredo Ponce de León Garduño, Blanca Taboada, Alejandro Sánchez, Pavel Isa, Ricardo Grande, Gloria Vázquez, Francisco Pulido, Carlos F. Arias,José Ernesto Ramírez González
EPI_ISL_424673 hCoV-19/Mexico/CDMX-InDRE_06/2020 North America / Mexico / CDMX 2020-03-12 Instituto de Diagnostico y Referencia Epidemiologicos Instituto de Diagnostico y Referencia Epidemiologicos Adnan Araiza Rodríguez, Edgar Mendieta Condado,Fabiola Garcés Ayala, Gisela Barrera Badillo, Irma López Martínez, Lucia Hernández Rivas, Celia Boukadida, Santiago Avila Ríos, Mario Mújica Sánchez, José Arturo Martínez Orozco,Eduardo Becerril Vargas, Joel Armando Vázquez Pérez, Victor Hugo Borja Aburto, Concepción Grajales Muñiz, Cesar Raúl González Bonilla, Carolina González Torres, Francisco Javier Gaytán Cervantes, José Esteban Muñoz Medina, Guillermo M. Ruiz-Palacios, Pilar Ramos Cervantes, Violeta Ibarra Gonzalez, Fernando Ledesma Barrientos, Luis Alberto García Andrade, Alfredo Ponce de León Garduño, Blanca Taboada, Alejandro Sánchez, Pavel Isa, Ricardo Grande, Gloria Vázquez, Francisco Pulido, Carlos F. Arias,José Ernesto Ramírez González
EPI_ISL_424841 hCoV-19/USA/SC_3569/2020 North America / USA / South Carolina 2020-03-06 SC Dept of Health and Env. Control-Bureau of Laboratories Pathogen Discovery, Respiratory Viruses Branch, Division of Viral Diseases, Centers for Disease Control and Prevention Yan Li, Krista Queen, Clinton R. Paden, Rachel Marine, Anna Uehara, Ying Tao, Jing Zhang, Haibin Wang, Mary S. Keckler, Alison S. Laufer Halpin, Christopher A. Elkins, Suxiang Tong
EPI_ISL_424842 hCoV-19/USA/SC_3575/2020 North America / USA / South Carolina 2020-03-07 SC Dept of Health and Env. Control-Bureau of Laboratories Pathogen Discovery, Respiratory Viruses Branch, Division of Viral Diseases, Centers for Disease Control and Prevention Yan Li, Krista Queen, Clinton R. Paden, Rachel Marine, Anna Uehara, Ying Tao, Jing Zhang, Haibin Wang, Mary S. Keckler, Alison S. Laufer Halpin, Christopher A. Elkins, Suxiang Tong
EPI_ISL_424843 hCoV-19/USA/MA_0020/2020 North America / USA / Massachusetts 2020-03-05 MA State Public Health Laboratory Pathogen Discovery, Respiratory Viruses Branch, Division of Viral Diseases, Centers for Disease Control and Prevention Yan Li, Krista Queen, Clinton R. Paden, Rachel Marine, Anna Uehara, Ying Tao, Jing Zhang, Haibin Wang, Mary S. Keckler, Alison S. Laufer Halpin, Christopher A. Elkins, Suxiang Tong
EPI_ISL_424844 hCoV-19/USA/MA_3672/2020 North America / USA / Massachusetts 2020-03-06 MA State Public Health Laboratory Pathogen Discovery, Respiratory Viruses Branch, Division of Viral Diseases, Centers for Disease Control and Prevention Yan Li, Krista Queen, Clinton R. Paden, Rachel Marine, Anna Uehara, Ying Tao, Jing Zhang, Haibin Wang, Mary S. Keckler, Alison S. Laufer Halpin, Christopher A. Elkins, Suxiang Tong
EPI_ISL_424845 hCoV-19/USA/MA_3699/2020 North America / USA / Massachusetts 2020-03-06 MA State Public Health Laboratory Pathogen Discovery, Respiratory Viruses Branch, Division of Viral Diseases, Centers for Disease Control and Prevention Yan Li, Krista Queen, Clinton R. Paden, Rachel Marine, Anna Uehara, Ying Tao, Jing Zhang, Haibin Wang, Mary S. Keckler, Alison S. Laufer Halpin, Christopher A. Elkins, Suxiang Tong

EPI_ISL_424846 hCoV-19/USA/MA_3878/2020 North America / USA / Massachusetts 2020-03-06 MA State Public Health Laboratory Pathogen Discovery, Respiratory Viruses Branch, Division of Viral Diseases, Centers for Disease Control and Prevention Yan Li, Krista Queen, Clinton R. Paden, Rachel Marine, Anna Uehara, Ying Tao, Jing Zhang, Haibin Wang, Mary S. Keckler, Alison S. Laufer Halpin, Christopher A. Elkins, Suxiang Tong
EPI_ISL_424847 hCoV-19/USA/MA_4811/2020 North America / USA / Massachusetts 2020-03-07 MA State Public Health Laboratory Pathogen Discovery, Respiratory Viruses Branch, Division of Viral Diseases, Centers for Disease Control and Prevention Yan Li, Krista Queen, Clinton R. Paden, Rachel Marine, Anna Uehara, Ying Tao, Jing Zhang, Haibin Wang, Mary S. Keckler, Alison S. Laufer Halpin, Christopher A. Elkins, Suxiang Tong
EPI_ISL_424848 hCoV-19/USA/AZ_8132/2020 North America / USA / Arizona 2020-03-09 AZ SPHL, Arizona Department of Health Services Pathogen Discovery, Respiratory Viruses Branch, Division of Viral Diseases, Centers for Disease Control and Prevention Yan Li, Krista Queen, Clinton R. Paden, Rachel Marine, Anna Uehara, Ying Tao, Jing Zhang, Haibin Wang, Mary S. Keckler, Alison S. Laufer Halpin, Christopher A. Elkins, Suxiang Tong
EPI_ISL_424849 hCoV-19/USA/AZ_8135/2020 North America / USA / Arizona 2020-03-09 AZ SPHL, Arizona Department of Health Services Pathogen Discovery, Respiratory Viruses Branch, Division of Viral Diseases, Centers for Disease Control and Prevention Yan Li, Krista Queen, Clinton R. Paden, Rachel Marine, Anna Uehara, Ying Tao, Jing Zhang, Haibin Wang, Mary S. Keckler, Alison S. Laufer Halpin, Christopher A. Elkins, Suxiang Tong
EPI_ISL_424850 hCoV-19/USA/IL_0087/2020 North America / USA / Illinois 2020-03-07 IL Department of Public Health Chicago Laboratory Pathogen Discovery, Respiratory Viruses Branch, Division of Viral Diseases, Centers for Disease Control and Prevention Yan Li, Krista Queen, Clinton R. Paden, Rachel Marine, Anna Uehara, Ying Tao, Jing Zhang, Haibin Wang, Mary S. Keckler, Alison S. Laufer Halpin, Christopher A. Elkins, Suxiang Tong
EPI_ISL_424851 hCoV-19/USA/IL_0089/2020 North America / USA / Illinois 2020-03-07 IL Department of Public Health Chicago Laboratory Pathogen Discovery, Respiratory Viruses Branch, Division of Viral Diseases, Centers for Disease Control and Prevention Yan Li, Krista Queen, Clinton R. Paden, Rachel Marine, Anna Uehara, Ying Tao, Jing Zhang, Haibin Wang, Mary S. Keckler, Alison S. Laufer Halpin, Christopher A. Elkins, Suxiang Tong
EPI_ISL_424852 hCoV-19/USA/DC_0004/2020 North America / USA / District of Columbia 2020-03-06 DC Public Health Lab/ Dept. of Forensic Sciences Pathogen Discovery, Respiratory Viruses Branch, Division of Viral Diseases, Centers for Disease Control and Prevention Yan Li, Krista Queen, Clinton R. Paden, Rachel Marine, Anna Uehara, Ying Tao, Jing Zhang, Haibin Wang, Mary S. Keckler, Alison S. Laufer Halpin, Christopher A. Elkins, Suxiang Tong
EPI_ISL_424853 hCoV-19/USA/FL_9590/2020 North America / USA / Florida 2020-03-05 FL Bureau of Public Health Laboratories-Miami Pathogen Discovery, Respiratory Viruses Branch, Division of Viral Diseases, Centers for Disease Control and Prevention Yan Li, Krista Queen, Clinton R. Paden, Rachel Marine, Anna Uehara, Ying Tao, Jing Zhang, Haibin Wang, Mary S. Keckler, Alison S. Laufer Halpin, Christopher A. Elkins, Suxiang Tong
EPI_ISL_424854 hCoV-19/USA/FL_9655/2020 North America / USA / Florida 2020-03-05 FL Bureau of Public Health Laboratories-Miami Pathogen Discovery, Respiratory Viruses Branch, Division of Viral Diseases, Centers for Disease Control and Prevention Yan Li, Krista Queen, Clinton R. Paden, Rachel Marine, Anna Uehara, Ying Tao, Jing Zhang, Haibin Wang, Mary S. Keckler, Alison S. Laufer Halpin, Christopher A. Elkins, Suxiang Tong
EPI_ISL_425944 hCoV-19/Scotland/EDB214/2020 Europe / United Kingdom / Scotland 2020-03-26 Virology Department, Royal Infirmary of Edinburgh, NHS Lothian / School of Biological Sciences, University of Edinburgh / Institute of Genetics and Molecular Medicine, University of Edinburgh COVID-19 Genomics UK (COG-UK) Consortium McHugh M, Dewar R, Rooke S, Gallagher M, Balcaza C, O'Toole A, Hill V, McCrone JT, Colquhoun R, Yu X, Jackson B, Scher E, Rambaut A, Williams TC, Templeton K
EPI_ISL_425945 hCoV-19/Scotland/EDB216/2020 Europe / United Kingdom / Scotland 2020-03-26 Virology Department, Royal Infirmary of Edinburgh, NHS Lothian / School of Biological Sciences, University of Edinburgh / Institute of Genetics and Molecular Medicine, University of Edinburgh COVID-19 Genomics UK (COG-UK) Consortium McHugh M, Dewar R, Rooke S, Gallagher M, Balcaza C, O'Toole A, Hill V, McCrone JT, Colquhoun R, Yu X, Jackson B, Scher E, Rambaut A, Williams TC, Templeton K
EPI_ISL_425947 hCoV-19/Scotland/EDB219/2020 Europe / United Kingdom / Scotland 2020-03-26 Virology Department, Royal Infirmary of Edinburgh, NHS Lothian / School of Biological Sciences, University of Edinburgh / Institute of Genetics and Molecular Medicine, University of Edinburgh COVID-19 Genomics UK (COG-UK) Consortium McHugh M, Dewar R, Rooke S, Gallagher M, Balcaza C, O'Toole A, Hill V, McCrone JT, Colquhoun R, Yu X, Jackson B, Scher E, Rambaut A, Williams TC, Templeton K
EPI_ISL_425948 hCoV-19/Scotland/EDB222/2020 Europe / United Kingdom / Scotland 2020-03-26 Virology Department, Royal Infirmary of Edinburgh, NHS Lothian / School of Biological Sciences, University of Edinburgh / Institute of Genetics and Molecular Medicine, University of Edinburgh COVID-19 Genomics UK (COG-UK) Consortium McHugh M, Dewar R, Rooke S, Gallagher M, Balcaza C, O'Toole A, Hill V, McCrone JT, Colquhoun R, Yu X, Jackson B, Scher E, Rambaut A, Williams TC, Templeton K
EPI_ISL_425949 hCoV-19/Scotland/EDB226/2020 Europe / United Kingdom / Scotland 2020-03-26 Virology Department, Royal Infirmary of Edinburgh, NHS Lothian / School of Biological Sciences, University of Edinburgh / Institute of Genetics and Molecular Medicine, University of Edinburgh COVID-19 Genomics UK (COG-UK) Consortium McHugh M, Dewar R, Rooke S, Gallagher M, Balcaza C, O'Toole A, Hill V, McCrone JT, Colquhoun R, Yu X, Jackson B, Scher E, Rambaut A, Williams TC, Templeton K

EPI_ISL_425950 hCoV-19/Scotland/EDB227/2020 Europe / United Kingdom / Scotland 2020-03-26
Virology Department, Royal Infirmary of Edinburgh, NHS Lothian / School of Biological Sciences, University of Edinburgh / Institute of Genetics and Molecular Medicine, University of Edinburgh COVID-19 Genomics UK (COG-UK) Consortium McHugh M, Dewar R, Rooke S, Gallagher M, Balcaza C, O'Toole A, Hill V, McCrone JT, Colquhoun R, Yu X, Jackson B, Scher E, Rambaut A, Williams TC, Templeton K
EPI_ISL_425951 hCoV-19/Scotland/EDB232/2020 Europe / United Kingdom / Scotland 2020-03-27
Virology Department, Royal Infirmary of Edinburgh, NHS Lothian / School of Biological Sciences, University of Edinburgh / Institute of Genetics and Molecular Medicine, University of Edinburgh COVID-19 Genomics UK (COG-UK) Consortium McHugh M, Dewar R, Rooke S, Gallagher M, Balcaza C, O'Toole A, Hill V, McCrone JT, Colquhoun R, Yu X, Jackson B, Scher E, Rambaut A, Williams TC, Templeton K
EPI_ISL_425952 hCoV-19/Scotland/EDB234/2020 Europe / United Kingdom / Scotland 2020-03-27
Virology Department, Royal Infirmary of Edinburgh, NHS Lothian / School of Biological Sciences, University of Edinburgh / Institute of Genetics and Molecular Medicine, University of Edinburgh COVID-19 Genomics UK (COG-UK) Consortium McHugh M, Dewar R, Rooke S, Gallagher M, Balcaza C, O'Toole A, Hill V, McCrone JT, Colquhoun R, Yu X, Jackson B, Scher E, Rambaut A, Williams TC, Templeton K
EPI_ISL_425953 hCoV-19/Scotland/EDB245/2020 Europe / United Kingdom / Scotland 2020-03-26
Virology Department, Royal Infirmary of Edinburgh, NHS Lothian / School of Biological Sciences, University of Edinburgh / Institute of Genetics and Molecular Medicine, University of Edinburgh COVID-19 Genomics UK (COG-UK) Consortium McHugh M, Dewar R, Rooke S, Gallagher M, Balcaza C, O'Toole A, Hill V, McCrone JT, Colquhoun R, Yu X, Jackson B, Scher E, Rambaut A, Williams TC, Templeton K
EPI_ISL_425954 hCoV-19/Scotland/EDB248/2020 Europe / United Kingdom / Scotland 2020-03-27
Virology Department, Royal Infirmary of Edinburgh, NHS Lothian / School of Biological Sciences, University of Edinburgh / Institute of Genetics and Molecular Medicine, University of Edinburgh COVID-19 Genomics UK (COG-UK) Consortium McHugh M, Dewar R, Rooke S, Gallagher M, Balcaza C, O'Toole A, Hill V, McCrone JT, Colquhoun R, Yu X, Jackson B, Scher E, Rambaut A, Williams TC, Templeton K
EPI_ISL_425956 hCoV-19/Scotland/EDB254/2020 Europe / United Kingdom / Scotland 2020-03-26
Virology Department, Royal Infirmary of Edinburgh, NHS Lothian / School of Biological Sciences, University of Edinburgh / Institute of Genetics and Molecular Medicine, University of Edinburgh COVID-19 Genomics UK (COG-UK) Consortium McHugh M, Dewar R, Rooke S, Gallagher M, Balcaza C, O'Toole A, Hill V, McCrone JT, Colquhoun R, Yu X, Jackson B, Scher E, Rambaut A, Williams TC, Templeton K
EPI_ISL_425958 hCoV-19/Scotland/EDB270/2020 Europe / United Kingdom / Scotland 2020-03-27
Virology Department, Royal Infirmary of Edinburgh, NHS Lothian / School of Biological Sciences, University of Edinburgh / Institute of Genetics and Molecular Medicine, University of Edinburgh COVID-19 Genomics UK (COG-UK) Consortium McHugh M, Dewar R, Rooke S, Gallagher M, Balcaza C, O'Toole A, Hill V, McCrone JT, Colquhoun R, Yu X, Jackson B, Scher E, Rambaut A, Williams TC, Templeton K
EPI_ISL_425959 hCoV-19/Scotland/EDB273/2020 Europe / United Kingdom / Scotland 2020-03-26
Virology Department, Royal Infirmary of Edinburgh, NHS Lothian / School of Biological Sciences, University of Edinburgh / Institute of Genetics and Molecular Medicine, University of Edinburgh COVID-19 Genomics UK (COG-UK) Consortium McHugh M, Dewar R, Rooke S, Gallagher M, Balcaza C, O'Toole A, Hill V, McCrone JT, Colquhoun R, Yu X, Jackson B, Scher E, Rambaut A, Williams TC, Templeton K
EPI_ISL_425961 hCoV-19/Scotland/EDB275/2020 Europe / United Kingdom / Scotland 2020-03-26
Virology Department, Royal Infirmary of Edinburgh, NHS Lothian / School of Biological Sciences, University of Edinburgh / Institute of Genetics and Molecular Medicine, University of Edinburgh COVID-19 Genomics UK (COG-UK) Consortium McHugh M, Dewar R, Rooke S, Gallagher M, Balcaza C, O'Toole A, Hill V, McCrone JT, Colquhoun R, Yu X, Jackson B, Scher E, Rambaut A, Williams TC, Templeton K
EPI_ISL_425962 hCoV-19/Scotland/EDB278/2020 Europe / United Kingdom / Scotland 2020-03-27
Virology Department, Royal Infirmary of Edinburgh, NHS Lothian / School of Biological Sciences, University of Edinburgh / Institute of Genetics and Molecular Medicine, University of Edinburgh COVID-19 Genomics UK (COG-UK) Consortium McHugh M, Dewar R, Rooke S, Gallagher M, Balcaza C, O'Toole A, Hill V, McCrone JT, Colquhoun R, Yu X, Jackson B, Scher E, Rambaut A, Williams TC, Templeton K
EPI_ISL_425963 hCoV-19/Scotland/EDB283/2020 Europe / United Kingdom / Scotland 2020-03-27
Virology Department, Royal Infirmary of Edinburgh, NHS Lothian / School of Biological Sciences, University of Edinburgh / Institute of Genetics and Molecular Medicine, University of Edinburgh COVID-19 Genomics UK (COG-UK) Consortium McHugh M, Dewar R, Rooke S, Gallagher M, Balcaza C, O'Toole A, Hill V, McCrone JT, Colquhoun R, Yu X, Jackson B, Scher E, Rambaut A, Williams TC, Templeton K
EPI_ISL_425964 hCoV-19/Scotland/EDB287/2020 Europe / United Kingdom / Scotland 2020-03-27
Virology Department, Royal Infirmary of Edinburgh, NHS Lothian / School of Biological Sciences, University of Edinburgh / Institute of Genetics and Molecular Medicine, University of Edinburgh COVID-19 Genomics UK (COG-UK) Consortium McHugh M, Dewar R, Rooke S, Gallagher M, Balcaza C, O'Toole A, Hill V, McCrone JT, Colquhoun R, Yu X, Jackson B, Scher E, Rambaut A, Williams TC, Templeton K
EPI_ISL_425965 hCoV-19/Scotland/EDB288/2020 Europe / United Kingdom / Scotland 2020-03-27
Virology Department, Royal Infirmary of Edinburgh, NHS Lothian / School of Biological Sciences, University of Edinburgh / Institute of Genetics and Molecular Medicine, University of Edinburgh COVID-19 Genomics UK (COG-UK) Consortium McHugh M, Dewar R, Rooke S, Gallagher M, Balcaza C, O'Toole A, Hill V, McCrone JT, Colquhoun R, Yu X, Jackson B, Scher E, Rambaut A, Williams TC, Templeton K
EPI_ISL_425966 hCoV-19/Scotland/EDB297/2020 Europe / United Kingdom / Scotland 2020-03-28
Virology Department, Royal Infirmary of Edinburgh, NHS Lothian / School of Biological Sciences, University of Edinburgh / Institute of Genetics and Molecular Medicine, University of Edinburgh COVID-19 Genomics UK (COG-UK) Consortium McHugh M, Dewar R, Rooke S, Gallagher M, Balcaza C, O'Toole A, Hill V, McCrone JT, Colquhoun R, Yu X, Jackson B, Scher E, Rambaut A, Williams TC, Templeton K

EPI_ISL_425967 hCoV-19/Scotland/EDB300/2020 Europe / United Kingdom / Scotland 2020-03-29 Virology Department, Royal Infirmary of Edinburgh, NHS Lothian / School of Biological Sciences, University of Edinburgh / Institute of Genetics and Molecular Medicine, University of Edinburgh COVID-19 Genomics UK (COG-UK) Consortium McHugh M, Dewar R, Rooke S, Gallagher M, Balcaza C, O'Toole A, Hill V, McCrone JT, Colquhoun R, Yu X, Jackson B, Scher E, Rambaut A, Williams TC, Templeton K
EPI_ISL_425968 hCoV-19/Scotland/EDB314/2020 Europe / United Kingdom / Scotland 2020-03-28 Virology Department, Royal Infirmary of Edinburgh, NHS Lothian / School of Biological Sciences, University of Edinburgh / Institute of Genetics and Molecular Medicine, University of Edinburgh COVID-19 Genomics UK (COG-UK) Consortium McHugh M, Dewar R, Rooke S, Gallagher M, Balcaza C, O'Toole A, Hill V, McCrone JT, Colquhoun R, Yu X, Jackson B, Scher E, Rambaut A, Williams TC, Templeton K
EPI_ISL_425969 hCoV-19/Scotland/EDB318/2020 Europe / United Kingdom / Scotland 2020-03-28 Virology Department, Royal Infirmary of Edinburgh, NHS Lothian / School of Biological Sciences, University of Edinburgh / Institute of Genetics and Molecular Medicine, University of Edinburgh COVID-19 Genomics UK (COG-UK) Consortium McHugh M, Dewar R, Rooke S, Gallagher M, Balcaza C, O'Toole A, Hill V, McCrone JT, Colquhoun R, Yu X, Jackson B, Scher E, Rambaut A, Williams TC, Templeton K
EPI_ISL_425971 hCoV-19/Scotland/EDB325/2020 Europe / United Kingdom / Scotland 2020-03-28 Virology Department, Royal Infirmary of Edinburgh, NHS Lothian / School of Biological Sciences, University of Edinburgh / Institute of Genetics and Molecular Medicine, University of Edinburgh COVID-19 Genomics UK (COG-UK) Consortium McHugh M, Dewar R, Rooke S, Gallagher M, Balcaza C, O'Toole A, Hill V, McCrone JT, Colquhoun R, Yu X, Jackson B, Scher E, Rambaut A, Williams TC, Templeton K
EPI_ISL_425972 hCoV-19/Scotland/EDB326/2020 Europe / United Kingdom / Scotland 2020-03-28 Virology Department, Royal Infirmary of Edinburgh, NHS Lothian / School of Biological Sciences, University of Edinburgh / Institute of Genetics and Molecular Medicine, University of Edinburgh COVID-19 Genomics UK (COG-UK) Consortium McHugh M, Dewar R, Rooke S, Gallagher M, Balcaza C, O'Toole A, Hill V, McCrone JT, Colquhoun R, Yu X, Jackson B, Scher E, Rambaut A, Williams TC, Templeton K
EPI_ISL_425973 hCoV-19/Scotland/EDB327/2020 Europe / United Kingdom / Scotland 2020-03-28 Virology Department, Royal Infirmary of Edinburgh, NHS Lothian / School of Biological Sciences, University of Edinburgh / Institute of Genetics and Molecular Medicine, University of Edinburgh COVID-19 Genomics UK (COG-UK) Consortium McHugh M, Dewar R, Rooke S, Gallagher M, Balcaza C, O'Toole A, Hill V, McCrone JT, Colquhoun R, Yu X, Jackson B, Scher E, Rambaut A, Williams TC, Templeton K
EPI_ISL_425976 hCoV-19/Scotland/EDB342/2020 Europe / United Kingdom / Scotland 2020-03-30 Virology Department, Royal Infirmary of Edinburgh, NHS Lothian / School of Biological Sciences, University of Edinburgh / Institute of Genetics and Molecular Medicine, University of Edinburgh COVID-19 Genomics UK (COG-UK) Consortium McHugh M, Dewar R, Rooke S, Gallagher M, Balcaza C, O'Toole A, Hill V, McCrone JT, Colquhoun R, Yu X, Jackson B, Scher E, Rambaut A, Williams TC, Templeton K
EPI_ISL_425978 hCoV-19/Scotland/EDB354/2020 Europe / United Kingdom / Scotland 2020-03-30 Virology Department, Royal Infirmary of Edinburgh, NHS Lothian / School of Biological Sciences, University of Edinburgh / Institute of Genetics and Molecular Medicine, University of Edinburgh COVID-19 Genomics UK (COG-UK) Consortium McHugh M, Dewar R, Rooke S, Gallagher M, Balcaza C, O'Toole A, Hill V, McCrone JT, Colquhoun R, Yu X, Jackson B, Scher E, Rambaut A, Williams TC, Templeton K
EPI_ISL_425979 hCoV-19/Scotland/EDB355/2020 Europe / United Kingdom / Scotland 2020-03-29 Virology Department, Royal Infirmary of Edinburgh, NHS Lothian / School of Biological Sciences, University of Edinburgh / Institute of Genetics and Molecular Medicine, University of Edinburgh COVID-19 Genomics UK (COG-UK) Consortium McHugh M, Dewar R, Rooke S, Gallagher M, Balcaza C, O'Toole A, Hill V, McCrone JT, Colquhoun R, Yu X, Jackson B, Scher E, Rambaut A, Williams TC, Templeton K
EPI_ISL_425980 hCoV-19/Scotland/EDB356/2020 Europe / United Kingdom / Scotland 2020-03-30 Virology Department, Royal Infirmary of Edinburgh, NHS Lothian / School of Biological Sciences, University of Edinburgh / Institute of Genetics and Molecular Medicine, University of Edinburgh COVID-19 Genomics UK (COG-UK) Consortium McHugh M, Dewar R, Rooke S, Gallagher M, Balcaza C, O'Toole A, Hill V, McCrone JT, Colquhoun R, Yu X, Jackson B, Scher E, Rambaut A, Williams TC, Templeton K
EPI_ISL_425982 hCoV-19/Scotland/EDB363/2020 Europe / United Kingdom / Scotland 2020-03-29 Virology Department, Royal Infirmary of Edinburgh, NHS Lothian / School of Biological Sciences, University of Edinburgh / Institute of Genetics and Molecular Medicine, University of Edinburgh COVID-19 Genomics UK (COG-UK) Consortium McHugh M, Dewar R, Rooke S, Gallagher M, Balcaza C, O'Toole A, Hill V, McCrone JT, Colquhoun R, Yu X, Jackson B, Scher E, Rambaut A, Williams TC, Templeton K
EPI_ISL_425985 hCoV-19/Scotland/EDB373/2020 Europe / United Kingdom / Scotland 2020-03-30 Virology Department, Royal Infirmary of Edinburgh, NHS Lothian / School of Biological Sciences, University of Edinburgh / Institute of Genetics and Molecular Medicine, University of Edinburgh COVID-19 Genomics UK (COG-UK) Consortium McHugh M, Dewar R, Rooke S, Gallagher M, Balcaza C, O'Toole A, Hill V, McCrone JT, Colquhoun R, Yu X, Jackson B, Scher E, Rambaut A, Williams TC, Templeton K
EPI_ISL_425986 hCoV-19/Scotland/EDB374/2020 Europe / United Kingdom / Scotland 2020-03-30 Virology Department, Royal Infirmary of Edinburgh, NHS Lothian / School of Biological Sciences, University of Edinburgh / Institute of Genetics and Molecular Medicine, University of Edinburgh COVID-19 Genomics UK (COG-UK) Consortium McHugh M, Dewar R, Rooke S, Gallagher M, Balcaza C, O'Toole A, Hill V, McCrone JT, Colquhoun R, Yu X, Jackson B, Scher E, Rambaut A, Williams TC, Templeton K
EPI_ISL_425987 hCoV-19/Scotland/EDB378/2020 Europe / United Kingdom / Scotland 2020-03-30 Virology Department, Royal Infirmary of Edinburgh, NHS Lothian / School of Biological Sciences, University of Edinburgh / Institute of Genetics and Molecular Medicine, University of Edinburgh COVID-19 Genomics UK (COG-UK) Consortium McHugh M, Dewar R, Rooke S, Gallagher M, Balcaza C, O'Toole A, Hill V, McCrone JT, Colquhoun R, Yu X, Jackson B, Scher E, Rambaut A, Williams TC, Templeton K

EPI_ISL_425988 hCoV-19/Scotland/EDB379/2020 Europe / United Kingdom / Scotland 2020-03-30 Virology Department, Royal Infirmary of Edinburgh, NHS Lothian / School of Biological Sciences, University of Edinburgh / Institute of Genetics and Molecular Medicine, University of Edinburgh COVID-19 Genomics UK (COG-UK) Consortium McHugh M, Dewar R, Rooke S, Gallagher M, Balcaza C, O'Toole A, Hill V, McCrone JT, Colquhoun R, Yu X, Jackson B, Scher E, Rambaut A, Williams TC, Templeton K
EPI_ISL_425990 hCoV-19/Scotland/EDB383/2020 Europe / United Kingdom / Scotland 2020-03-30 Virology Department, Royal Infirmary of Edinburgh, NHS Lothian / School of Biological Sciences, University of Edinburgh / Institute of Genetics and Molecular Medicine, University of Edinburgh COVID-19 Genomics UK (COG-UK) Consortium McHugh M, Dewar R, Rooke S, Gallagher M, Balcaza C, O'Toole A, Hill V, McCrone JT, Colquhoun R, Yu X, Jackson B, Scher E, Rambaut A, Williams TC, Templeton K
EPI_ISL_425991 hCoV-19/Scotland/EDB386/2020 Europe / United Kingdom / Scotland 2020-03-30 Virology Department, Royal Infirmary of Edinburgh, NHS Lothian / School of Biological Sciences, University of Edinburgh / Institute of Genetics and Molecular Medicine, University of Edinburgh COVID-19 Genomics UK (COG-UK) Consortium McHugh M, Dewar R, Rooke S, Gallagher M, Balcaza C, O'Toole A, Hill V, McCrone JT, Colquhoun R, Yu X, Jackson B, Scher E, Rambaut A, Williams TC, Templeton K
EPI_ISL_425992 hCoV-19/Scotland/EDB389/2020 Europe / United Kingdom / Scotland 2020-03-30 Virology Department, Royal Infirmary of Edinburgh, NHS Lothian / School of Biological Sciences, University of Edinburgh / Institute of Genetics and Molecular Medicine, University of Edinburgh COVID-19 Genomics UK (COG-UK) Consortium McHugh M, Dewar R, Rooke S, Gallagher M, Balcaza C, O'Toole A, Hill V, McCrone JT, Colquhoun R, Yu X, Jackson B, Scher E, Rambaut A, Williams TC, Templeton K
EPI_ISL_425993 hCoV-19/Scotland/EDB390/2020 Europe / United Kingdom / Scotland 2020-03-30 Virology Department, Royal Infirmary of Edinburgh, NHS Lothian / School of Biological Sciences, University of Edinburgh / Institute of Genetics and Molecular Medicine, University of Edinburgh COVID-19 Genomics UK (COG-UK) Consortium McHugh M, Dewar R, Rooke S, Gallagher M, Balcaza C, O'Toole A, Hill V, McCrone JT, Colquhoun R, Yu X, Jackson B, Scher E, Rambaut A, Williams TC, Templeton K
EPI_ISL_425994 hCoV-19/Scotland/EDB391/2020 Europe / United Kingdom / Scotland 2020-03-30 Virology Department, Royal Infirmary of Edinburgh, NHS Lothian / School of Biological Sciences, University of Edinburgh / Institute of Genetics and Molecular Medicine, University of Edinburgh COVID-19 Genomics UK (COG-UK) Consortium McHugh M, Dewar R, Rooke S, Gallagher M, Balcaza C, O'Toole A, Hill V, McCrone JT, Colquhoun R, Yu X, Jackson B, Scher E, Rambaut A, Williams TC, Templeton K
EPI_ISL_425995 hCoV-19/Scotland/EDB392/2020 Europe / United Kingdom / Scotland 2020-03-30 Virology Department, Royal Infirmary of Edinburgh, NHS Lothian / School of Biological Sciences, University of Edinburgh / Institute of Genetics and Molecular Medicine, University of Edinburgh COVID-19 Genomics UK (COG-UK) Consortium McHugh M, Dewar R, Rooke S, Gallagher M, Balcaza C, O'Toole A, Hill V, McCrone JT, Colquhoun R, Yu X, Jackson B, Scher E, Rambaut A, Williams TC, Templeton K
EPI_ISL_425996 hCoV-19/Scotland/EDB396/2020 Europe / United Kingdom / Scotland 2020-03-30 Virology Department, Royal Infirmary of Edinburgh, NHS Lothian / School of Biological Sciences, University of Edinburgh / Institute of Genetics and Molecular Medicine, University of Edinburgh COVID-19 Genomics UK (COG-UK) Consortium McHugh M, Dewar R, Rooke S, Gallagher M, Balcaza C, O'Toole A, Hill V, McCrone JT, Colquhoun R, Yu X, Jackson B, Scher E, Rambaut A, Williams TC, Templeton K
EPI_ISL_425997 hCoV-19/Scotland/EDB397/2020 Europe / United Kingdom / Scotland 2020-03-30 Virology Department, Royal Infirmary of Edinburgh, NHS Lothian / School of Biological Sciences, University of Edinburgh / Institute of Genetics and Molecular Medicine, University of Edinburgh COVID-19 Genomics UK (COG-UK) Consortium McHugh M, Dewar R, Rooke S, Gallagher M, Balcaza C, O'Toole A, Hill V, McCrone JT, Colquhoun R, Yu X, Jackson B, Scher E, Rambaut A, Williams TC, Templeton K
EPI_ISL_425998 hCoV-19/Scotland/EDB399/2020 Europe / United Kingdom / Scotland 2020-03-30 Virology Department, Royal Infirmary of Edinburgh, NHS Lothian / School of Biological Sciences, University of Edinburgh / Institute of Genetics and Molecular Medicine, University of Edinburgh COVID-19 Genomics UK (COG-UK) Consortium McHugh M, Dewar R, Rooke S, Gallagher M, Balcaza C, O'Toole A, Hill V, McCrone JT, Colquhoun R, Yu X, Jackson B, Scher E, Rambaut A, Williams TC, Templeton K
EPI_ISL_425999 hCoV-19/Scotland/EDB401/2020 Europe / United Kingdom / Scotland 2020-03-29 Virology Department, Royal Infirmary of Edinburgh, NHS Lothian / School of Biological Sciences, University of Edinburgh / Institute of Genetics and Molecular Medicine, University of Edinburgh COVID-19 Genomics UK (COG-UK) Consortium McHugh M, Dewar R, Rooke S, Gallagher M, Balcaza C, O'Toole A, Hill V, McCrone JT, Colquhoun R, Yu X, Jackson B, Scher E, Rambaut A, Williams TC, Templeton K
EPI_ISL_427001 hCoV-19/Australia/VIC716/2020 Oceania / Australia / Victoria 2020-03-27 Victorian Infectious Diseases Reference Laboratory (VIDRL) Microbiological Diagnostic Unit Public Health Laboratory and Victorian Infectious Diseases Reference Laboratory, Doherty Institute Caly L., Seemann T., Sait, M., Schultz M., Druce J., Sherry, N.
EPI_ISL_427002 hCoV-19/Australia/VIC718/2020 Oceania / Australia / Victoria 2020-03-27 Victorian Infectious Diseases Reference Laboratory (VIDRL) Microbiological Diagnostic Unit Public Health Laboratory and Victorian Infectious Diseases Reference Laboratory, Doherty Institute Caly L., Seemann T., Sait, M., Schultz M., Druce J., Sherry, N.
EPI_ISL_427003 hCoV-19/Australia/VIC720/2020 Oceania / Australia / Victoria 2020-03-28 Victorian Infectious Diseases Reference Laboratory (VIDRL) Microbiological Diagnostic Unit Public Health Laboratory and Victorian Infectious Diseases Reference Laboratory, Doherty Institute Caly L., Seemann T., Sait, M., Schultz M., Druce J., Sherry, N.
EPI_ISL_427006 hCoV-19/Australia/VIC725/2020 Oceania / Australia / Victoria 2020-03-28 Victorian Infectious Diseases Reference Laboratory (VIDRL) Microbiological Diagnostic Unit Public Health Laboratory and Victorian Infectious Diseases Reference Laboratory, Doherty Institute Caly L., Seemann T.,

Sait, M., Schultz M., Druce J., Sherry, N.
EPI_ISL_427010 hCoV-19/Australia/VIC731/2020 Oceania / Australia / Victoria 2020-03-30 Victorian Infectious Diseases Reference Laboratory (VIDRL) Microbiological Diagnostic Unit Public Health Laboratory and Victorian Infectious Diseases Reference Laboratory, Doherty Institute Caly L., Seemann T., Sait, M., Schultz M., Druce J., Sherry, N.
EPI_ISL_427011 hCoV-19/Australia/VIC732/2020 Oceania / Australia / Victoria 2020-03-30 Victorian Infectious Diseases Reference Laboratory (VIDRL) Microbiological Diagnostic Unit Public Health Laboratory and Victorian Infectious Diseases Reference Laboratory, Doherty Institute Caly L., Seemann T., Sait, M., Schultz M., Druce J., Sherry, N.
EPI_ISL_427012 hCoV-19/Australia/VIC733/2020 Oceania / Australia / Victoria 2020-03-30 Victorian Infectious Diseases Reference Laboratory (VIDRL) Microbiological Diagnostic Unit Public Health Laboratory and Victorian Infectious Diseases Reference Laboratory, Doherty Institute Caly L., Seemann T., Sait, M., Schultz M., Druce J., Sherry, N.
EPI_ISL_427013 hCoV-19/Australia/VIC734/2020 Oceania / Australia / Victoria 2020-03-30 Victorian Infectious Diseases Reference Laboratory (VIDRL) Microbiological Diagnostic Unit Public Health Laboratory and Victorian Infectious Diseases Reference Laboratory, Doherty Institute Caly L., Seemann T., Sait, M., Schultz M., Druce J., Sherry, N.
EPI_ISL_427014 hCoV-19/Australia/VIC735/2020 Oceania / Australia / Victoria 2020-03-30 Victorian Infectious Diseases Reference Laboratory (VIDRL) Microbiological Diagnostic Unit Public Health Laboratory and Victorian Infectious Diseases Reference Laboratory, Doherty Institute Caly L., Seemann T., Sait, M., Schultz M., Druce J., Sherry, N.
EPI_ISL_427015 hCoV-19/Australia/VIC736/2020 Oceania / Australia / Victoria 2020-03-30 Victorian Infectious Diseases Reference Laboratory (VIDRL) Microbiological Diagnostic Unit Public Health Laboratory and Victorian Infectious Diseases Reference Laboratory, Doherty Institute Caly L., Seemann T., Sait, M., Schultz M., Druce J., Sherry, N.
EPI_ISL_427016 hCoV-19/Australia/VIC737/2020 Oceania / Australia / Victoria 2020-03-30 Victorian Infectious Diseases Reference Laboratory (VIDRL) Microbiological Diagnostic Unit Public Health Laboratory and Victorian Infectious Diseases Reference Laboratory, Doherty Institute Caly L., Seemann T., Sait, M., Schultz M., Druce J., Sherry, N.
EPI_ISL_427017 hCoV-19/Australia/VIC738/2020 Oceania / Australia / Victoria 2020-03-30 Victorian Infectious Diseases Reference Laboratory (VIDRL) Microbiological Diagnostic Unit Public Health Laboratory and Victorian Infectious Diseases Reference Laboratory, Doherty Institute Caly L., Seemann T., Sait, M., Schultz M., Druce J., Sherry, N.
EPI_ISL_427018 hCoV-19/Australia/VIC739/2020 Oceania / Australia / Victoria 2020-03-30 Victorian Infectious Diseases Reference Laboratory (VIDRL) Microbiological Diagnostic Unit Public Health Laboratory and Victorian Infectious Diseases Reference Laboratory, Doherty Institute Caly L., Seemann T., Sait, M., Schultz M., Druce J., Sherry, N.
EPI_ISL_427019 hCoV-19/Australia/VIC741/2020 Oceania / Australia / Victoria 2020-03-30 Victorian Infectious Diseases Reference Laboratory (VIDRL) Microbiological Diagnostic Unit Public Health Laboratory and Victorian Infectious Diseases Reference Laboratory, Doherty Institute Caly L., Seemann T., Sait, M., Schultz M., Druce J., Sherry, N.
EPI_ISL_427020 hCoV-19/Australia/VIC742/2020 Oceania / Australia / Victoria 2020-03-29 Victorian Infectious Diseases Reference Laboratory (VIDRL) Microbiological Diagnostic Unit Public Health Laboratory and Victorian Infectious Diseases Reference Laboratory, Doherty Institute Caly L., Seemann T., Sait, M., Schultz M., Druce J., Sherry, N.
EPI_ISL_427022 hCoV-19/Australia/VIC744/2020 Oceania / Australia / Victoria 2020-03-29 Victorian Infectious Diseases Reference Laboratory (VIDRL) Microbiological Diagnostic Unit Public Health Laboratory and Victorian Infectious Diseases Reference Laboratory, Doherty Institute Caly L., Seemann T., Sait, M., Schultz M., Druce J., Sherry, N.
EPI_ISL_427023 hCoV-19/Australia/VIC745/2020 Oceania / Australia / Victoria 2020-03-29 Victorian Infectious Diseases Reference Laboratory (VIDRL) Microbiological Diagnostic Unit Public Health Laboratory and Victorian Infectious Diseases Reference Laboratory, Doherty Institute Caly L., Seemann T., Sait, M., Schultz M., Druce J., Sherry, N.
EPI_ISL_427024 hCoV-19/Australia/VIC746/2020 Oceania / Australia / Victoria 2020-03-29 Victorian Infectious Diseases Reference Laboratory (VIDRL) Microbiological Diagnostic Unit Public Health Laboratory and Victorian Infectious Diseases Reference Laboratory, Doherty Institute Caly L., Seemann T., Sait, M., Schultz M., Druce J., Sherry, N.
EPI_ISL_427025 hCoV-19/Australia/VIC747/2020 Oceania / Australia / Victoria 2020-03-31 Victorian Infectious Diseases Reference Laboratory (VIDRL) Microbiological Diagnostic Unit Public Health Laboratory and Victorian Infectious Diseases Reference Laboratory, Doherty Institute Caly L., Seemann T., Sait, M., Schultz M., Druce J., Sherry, N.
EPI_ISL_427026 hCoV-19/Australia/VIC748/2020 Oceania / Australia / Victoria 2020-03-30 Victorian Infectious Diseases Reference Laboratory (VIDRL) Microbiological Diagnostic Unit Public Health Laboratory and Victorian Infectious Diseases Reference Laboratory, Doherty Institute Caly L., Seemann T., Sait, M., Schultz M., Druce J., Sherry, N.
EPI_ISL_427028 hCoV-19/Australia/VIC751/2020 Oceania / Australia / Victoria 2020-03-30 Victorian Infectious Diseases Reference Laboratory (VIDRL) Microbiological Diagnostic Unit Public Health Laboratory and Victorian Infectious Diseases Reference Laboratory, Doherty Institute Caly L., Seemann T., Sait, M., Schultz M., Druce J., Sherry, N.
EPI_ISL_427029 hCoV-19/Australia/VIC752/2020 Oceania / Australia / Victoria 2020-03-30 Victorian

Infectious Diseases Reference Laboratory (VIDRL)	Microbiological Diagnostic Unit Public Health Laboratory and Victorian Infectious Diseases Reference Laboratory, Doherty Institute	Caly L., Seemann T., Sait, M., Schultz M., Druce J., Sherry, N.
EPI_ISL_427031	hCoV-19/Australia/VIC754/2020	Oceania / Australia / Victoria	2020-03-30	Victorian Infectious Diseases Reference Laboratory (VIDRL)	Microbiological Diagnostic Unit Public Health Laboratory and Victorian Infectious Diseases Reference Laboratory, Doherty Institute	Caly L., Seemann T., Sait, M., Schultz M., Druce J., Sherry, N.
EPI_ISL_427034	hCoV-19/Australia/VIC757/2020	Oceania / Australia / Victoria	2020-03-31	Victorian Infectious Diseases Reference Laboratory (VIDRL)	Microbiological Diagnostic Unit Public Health Laboratory and Victorian Infectious Diseases Reference Laboratory, Doherty Institute	Caly L., Seemann T., Sait, M., Schultz M., Druce J., Sherry, N.
EPI_ISL_427038	hCoV-19/Australia/VIC762/2020	Oceania / Australia / Victoria	2020-03-29	Victorian Infectious Diseases Reference Laboratory (VIDRL)	Microbiological Diagnostic Unit Public Health Laboratory and Victorian Infectious Diseases Reference Laboratory, Doherty Institute	Caly L., Seemann T., Sait, M., Schultz M., Druce J., Sherry, N.
EPI_ISL_427040	hCoV-19/Australia/VIC764/2020	Oceania / Australia / Victoria	2020-03-29	Victorian Infectious Diseases Reference Laboratory (VIDRL)	Microbiological Diagnostic Unit Public Health Laboratory and Victorian Infectious Diseases Reference Laboratory, Doherty Institute	Caly L., Seemann T., Sait, M., Schultz M., Druce J., Sherry, N.
EPI_ISL_427041	hCoV-19/Australia/VIC765/2020	Oceania / Australia / Victoria	2020-03-29	Victorian Infectious Diseases Reference Laboratory (VIDRL)	Microbiological Diagnostic Unit Public Health Laboratory and Victorian Infectious Diseases Reference Laboratory, Doherty Institute	Caly L., Seemann T., Sait, M., Schultz M., Druce J., Sherry, N.
EPI_ISL_427043	hCoV-19/Greece/13/2020	Europe / Greece / Athens	2020-03-18	Laboratory of Microbiology, Medical School, National and Kapodistrian University of Athens	Laboratory of Biology, Department of Medicine, Democritus University of Thrace	Bampali,M., Dovrolis,N., Gatzidou,E., Froukala,E., Stavropoulou,A., Veletza,S., Tsakris,A., Spanakis,N. and Karakasiliotis,I.
EPI_ISL_427045	hCoV-19/Australia/VIC768/2020	Oceania / Australia / Victoria	2020-03-20	Victorian Infectious Diseases Reference Laboratory (VIDRL)	Microbiological Diagnostic Unit Public Health Laboratory and Victorian Infectious Diseases Reference Laboratory, Doherty Institute	Caly L., Seemann T., Sait, M., Schultz M., Druce J., Sherry, N.
EPI_ISL_427046	hCoV-19/Australia/VIC769/2020	Oceania / Australia / Victoria	2020-03-20	Victorian Infectious Diseases Reference Laboratory (VIDRL)	Microbiological Diagnostic Unit Public Health Laboratory and Victorian Infectious Diseases Reference Laboratory, Doherty Institute	Caly L., Seemann T., Sait, M., Schultz M., Druce J., Sherry, N.
EPI_ISL_427048	hCoV-19/Australia/VIC772/2020	Oceania / Australia / Victoria	2020-03-20	Victorian Infectious Diseases Reference Laboratory (VIDRL)	Microbiological Diagnostic Unit Public Health Laboratory and Victorian Infectious Diseases Reference Laboratory, Doherty Institute	Caly L., Seemann T., Sait, M., Schultz M., Druce J., Sherry, N.
EPI_ISL_427049	hCoV-19/Australia/VIC773/2020	Oceania / Australia / Victoria	2020-03-20	Victorian Infectious Diseases Reference Laboratory (VIDRL)	Microbiological Diagnostic Unit Public Health Laboratory and Victorian Infectious Diseases Reference Laboratory, Doherty Institute	Caly L., Seemann T., Sait, M., Schultz M., Druce J., Sherry, N.
EPI_ISL_427054	hCoV-19/Australia/VIC779/2020	Oceania / Australia / Victoria	2020-03-30	Microbiological Diagnostic Unit Public Health Laboratory	Microbiological Diagnostic Unit Public Health Laboratory	Seemann T., Schultz M., Sait, M., Sherry, N.
EPI_ISL_427055	hCoV-19/Australia/VIC780/2020	Oceania / Australia / Victoria	2020-03-30	Microbiological Diagnostic Unit Public Health Laboratory	Microbiological Diagnostic Unit Public Health Laboratory	Seemann T., Schultz M., Sait, M., Sherry, N.
EPI_ISL_427057	hCoV-19/Australia/VIC782/2020	Oceania / Australia / Victoria	2020-03-30	Microbiological Diagnostic Unit Public Health Laboratory	Microbiological Diagnostic Unit Public Health Laboratory	Seemann T., Schultz M., Sait, M., Sherry, N.
EPI_ISL_427058	hCoV-19/Australia/VIC783/2020	Oceania / Australia / Victoria	2020-03-30	Microbiological Diagnostic Unit Public Health Laboratory	Microbiological Diagnostic Unit Public Health Laboratory	Seemann T., Schultz M., Sait, M., Sherry, N.
EPI_ISL_427060	hCoV-19/Australia/VIC785/2020	Oceania / Australia / Victoria	2020-03-30	Microbiological Diagnostic Unit Public Health Laboratory	Microbiological Diagnostic Unit Public Health Laboratory	Seemann T., Schultz M., Sait, M., Sherry, N.
EPI_ISL_427061	hCoV-19/Australia/VIC786/2020	Oceania / Australia / Victoria	2020-03-30	Microbiological Diagnostic Unit Public Health Laboratory	Microbiological Diagnostic Unit Public Health Laboratory	Seemann T., Schultz M., Sait, M., Sherry, N.
EPI_ISL_427062	hCoV-19/Australia/VIC787/2020	Oceania / Australia / Victoria	2020-03-30	Microbiological Diagnostic Unit Public Health Laboratory	Microbiological Diagnostic Unit Public Health Laboratory	Seemann T., Schultz M., Sait, M., Sherry, N.
EPI_ISL_427063	hCoV-19/Australia/VIC788/2020	Oceania / Australia / Victoria	2020-03-31	Microbiological Diagnostic Unit Public Health Laboratory	Microbiological Diagnostic Unit Public Health Laboratory	Seemann T., Schultz M., Sait, M., Sherry, N.
EPI_ISL_427065	hCoV-19/Australia/VIC790/2020	Oceania / Australia / Victoria	2020-03-31	Microbiological Diagnostic Unit Public Health Laboratory	Microbiological Diagnostic Unit Public Health Laboratory	Seemann T., Schultz M., Sait, M., Sherry, N.

EPI_ISL_427068 hCoV-19/Australia/VIC793/2020 Oceania / Australia / Victoria 2020-03-31 Microbiological Diagnostic Unit Public Health Laboratory Microbiological Diagnostic Unit Public Health Laboratory Seemann T., Schultz M., Sait, M., Sherry, N.
EPI_ISL_427069 hCoV-19/Australia/VIC794/2020 Oceania / Australia / Victoria 2020-03-31 Microbiological Diagnostic Unit Public Health Laboratory Microbiological Diagnostic Unit Public Health Laboratory Seemann T., Schultz M., Sait, M., Sherry, N.
EPI_ISL_427070 hCoV-19/Australia/VIC796/2020 Oceania / Australia / Victoria 2020-03-31 Microbiological Diagnostic Unit Public Health Laboratory Microbiological Diagnostic Unit Public Health Laboratory Seemann T., Schultz M., Sait, M., Sherry, N.
EPI_ISL_427071 hCoV-19/Australia/VIC797/2020 Oceania / Australia / Victoria 2020-04-01 Microbiological Diagnostic Unit Public Health Laboratory Microbiological Diagnostic Unit Public Health Laboratory Seemann T., Schultz M., Sait, M., Sherry, N.
EPI_ISL_427072 hCoV-19/Australia/VIC798/2020 Oceania / Australia / Victoria 2020-04-01 Microbiological Diagnostic Unit Public Health Laboratory Microbiological Diagnostic Unit Public Health Laboratory Seemann T., Schultz M., Sait, M., Sherry, N.
EPI_ISL_427073 hCoV-19/Australia/VIC799/2020 Oceania / Australia / Victoria 2020-04-01 Microbiological Diagnostic Unit Public Health Laboratory Microbiological Diagnostic Unit Public Health Laboratory Seemann T., Schultz M., Sait, M., Sherry, N.
EPI_ISL_427074 hCoV-19/Australia/VIC800/2020 Oceania / Australia / Victoria 2020-04-01 Microbiological Diagnostic Unit Public Health Laboratory Microbiological Diagnostic Unit Public Health Laboratory Seemann T., Schultz M., Sait, M., Sherry, N.
EPI_ISL_427076 hCoV-19/Australia/VIC802/2020 Oceania / Australia / Victoria 2020-04-01 Microbiological Diagnostic Unit Public Health Laboratory Microbiological Diagnostic Unit Public Health Laboratory Seemann T., Schultz M., Sait, M., Sherry, N.
EPI_ISL_427079 hCoV-19/Australia/VIC805/2020 Oceania / Australia / Victoria 2020-03-16 Victorian Infectious Diseases Reference Laboratory (VIDRL) Microbiological Diagnostic Unit Public Health Laboratory and Victorian Infectious Diseases Reference Laboratory, Doherty Institute Caly L., Seemann T., Sait, M., Schultz M., Druce J., Sherry, N.
EPI_ISL_427080 hCoV-19/Australia/VIC806/2020 Oceania / Australia / Victoria 2020-03-23 Victorian Infectious Diseases Reference Laboratory (VIDRL) Microbiological Diagnostic Unit Public Health Laboratory and Victorian Infectious Diseases Reference Laboratory, Doherty Institute Caly L., Seemann T., Sait, M., Schultz M., Druce J., Sherry, N.
EPI_ISL_427081 hCoV-19/Australia/VIC807/2020 Oceania / Australia / Victoria 2020-03-24 Victorian Infectious Diseases Reference Laboratory (VIDRL) Microbiological Diagnostic Unit Public Health Laboratory and Victorian Infectious Diseases Reference Laboratory, Doherty Institute Caly L., Seemann T., Sait, M., Schultz M., Druce J., Sherry, N.
EPI_ISL_427082 hCoV-19/Australia/VIC808/2020 Oceania / Australia / Victoria 2020-03-28 Victorian Infectious Diseases Reference Laboratory (VIDRL) Microbiological Diagnostic Unit Public Health Laboratory and Victorian Infectious Diseases Reference Laboratory, Doherty Institute Caly L., Seemann T., Sait, M., Schultz M., Druce J., Sherry, N.
EPI_ISL_427085 hCoV-19/Australia/VIC812/2020 Oceania / Australia / Victoria 2020-04-02 Victorian Infectious Diseases Reference Laboratory (VIDRL) Microbiological Diagnostic Unit Public Health Laboratory and Victorian Infectious Diseases Reference Laboratory, Doherty Institute Caly L., Seemann T., Sait, M., Schultz M., Druce J., Sherry, N.
EPI_ISL_427092 hCoV-19/Australia/VIC820/2020 Oceania / Australia / Victoria 2020-04-03 Victorian Infectious Diseases Reference Laboratory (VIDRL) Microbiological Diagnostic Unit Public Health Laboratory and Victorian Infectious Diseases Reference Laboratory, Doherty Institute Caly L., Seemann T., Sait, M., Schultz M., Druce J., Sherry, N.
EPI_ISL_427093 hCoV-19/Australia/VIC821/2020 Oceania / Australia / Victoria 2020-04-03 Victorian Infectious Diseases Reference Laboratory (VIDRL) Microbiological Diagnostic Unit Public Health Laboratory and Victorian Infectious Diseases Reference Laboratory, Doherty Institute Caly L., Seemann T., Sait, M., Schultz M., Druce J., Sherry, N.
EPI_ISL_427094 hCoV-19/Australia/VIC823/2020 Oceania / Australia / Victoria 2020-04-03 Victorian Infectious Diseases Reference Laboratory (VIDRL) Microbiological Diagnostic Unit Public Health Laboratory and Victorian Infectious Diseases Reference Laboratory, Doherty Institute Caly L., Seemann T., Sait, M., Schultz M., Druce J., Sherry, N.
EPI_ISL_427095 hCoV-19/Australia/VIC824/2020 Oceania / Australia / Victoria 2020-04-03 Victorian Infectious Diseases Reference Laboratory (VIDRL) Microbiological Diagnostic Unit Public Health Laboratory and Victorian Infectious Diseases Reference Laboratory, Doherty Institute Caly L., Seemann T., Sait, M., Schultz M., Druce J., Sherry, N.
EPI_ISL_427096 hCoV-19/Australia/VIC828/2020 Oceania / Australia / Victoria 2020-04-03 Victorian Infectious Diseases Reference Laboratory (VIDRL) Microbiological Diagnostic Unit Public Health Laboratory and Victorian Infectious Diseases Reference Laboratory, Doherty Institute Caly L., Seemann T., Sait, M., Schultz M., Druce J., Sherry, N.
EPI_ISL_428300 hCoV-19/USA/WI-UW-204/2020 North America / USA / Wisconsin / Campbellsp 2020-03-25 University of Wisconsin-Madison AIDS Vaccine Research Laboratories University of Wisconsin-Madison AIDS Vaccine Research Laboratories Gage Moreno, Katarina Braun, et al. AIDS Vaccine Research Laboratories
EPI_ISL_428301 hCoV-19/USA/WI-UW-205/2020 North America / USA / Wisconsin / Milwaukee 2020-03-25 University of Wisconsin-Madison AIDS Vaccine Research Laboratories University of Wisconsin-Madison AIDS Vaccine Research Laboratories Gage Moreno, Katarina Braun, et al. AIDS Vaccine Research Laboratories

EPI_ISL_428302	hCoV-19/USA/WI-UW-206/2020	North America / USA / Wisconsin / Grafton	2020-03-25	University of Wisconsin-Madison AIDS Vaccine Research Laboratories	University of Wisconsin-Madison AIDS Vaccine Research Laboratories	Gage Moreno, Katarina Braun, et al. AIDS Vaccine Research Laboratories
EPI_ISL_428303	hCoV-19/USA/WI-UW-207/2020	North America / USA / Wisconsin / Milwaukee	2020-03-23	University of Wisconsin-Madison AIDS Vaccine Research Laboratories	University of Wisconsin-Madison AIDS Vaccine Research Laboratories	Gage Moreno, Katarina Braun, et al. AIDS Vaccine Research Laboratories
EPI_ISL_428304	hCoV-19/USA/WI-UW-208/2020	North America / USA / Wisconsin / Milwaukee	2020-03-25	University of Wisconsin-Madison AIDS Vaccine Research Laboratories	University of Wisconsin-Madison AIDS Vaccine Research Laboratories	Gage Moreno, Katarina Braun, et al. AIDS Vaccine Research Laboratories
EPI_ISL_428305	hCoV-19/USA/WI-UW-209/2020	North America / USA / Wisconsin / Milwaukee	2020-03-25	University of Wisconsin-Madison AIDS Vaccine Research Laboratories	University of Wisconsin-Madison AIDS Vaccine Research Laboratories	Gage Moreno, Katarina Braun, et al. AIDS Vaccine Research Laboratories
EPI_ISL_428306	hCoV-19/USA/WI-UW-210/2020	North America / USA / Wisconsin / Milwaukee	2020-03-24	University of Wisconsin-Madison AIDS Vaccine Research Laboratories	University of Wisconsin-Madison AIDS Vaccine Research Laboratories	Gage Moreno, Katarina Braun, et al. AIDS Vaccine Research Laboratories
EPI_ISL_428307	hCoV-19/USA/WI-UW-211/2020	North America / USA / Wisconsin / Milwaukee	2020-03-25	University of Wisconsin-Madison AIDS Vaccine Research Laboratories	University of Wisconsin-Madison AIDS Vaccine Research Laboratories	Gage Moreno, Katarina Braun, et al. AIDS Vaccine Research Laboratories
EPI_ISL_428308	hCoV-19/USA/WI-UW-212/2020	North America / USA / Wisconsin / Milwaukee	2020-03-25	University of Wisconsin-Madison AIDS Vaccine Research Laboratories	University of Wisconsin-Madison AIDS Vaccine Research Laboratories	Gage Moreno, Katarina Braun, et al. AIDS Vaccine Research Laboratories
EPI_ISL_428309	hCoV-19/USA/WI-UW-213/2020	North America / USA / Wisconsin / Milwaukee	2020-03-25	University of Wisconsin-Madison AIDS Vaccine Research Laboratories	University of Wisconsin-Madison AIDS Vaccine Research Laboratories	Gage Moreno, Katarina Braun, et al. AIDS Vaccine Research Laboratories
EPI_ISL_428310	hCoV-19/USA/WI-UW-214/2020	North America / USA / Wisconsin / Grafton	2020-03-25	University of Wisconsin-Madison AIDS Vaccine Research Laboratories	University of Wisconsin-Madison AIDS Vaccine Research Laboratories	Gage Moreno, Katarina Braun, et al. AIDS Vaccine Research Laboratories
EPI_ISL_428311	hCoV-19/USA/WI-UW-215/2020	North America / USA / Wisconsin / Milwaukee	2020-03-25	University of Wisconsin-Madison AIDS Vaccine Research Laboratories	University of Wisconsin-Madison AIDS Vaccine Research Laboratories	Gage Moreno, Katarina Braun, et al. AIDS Vaccine Research Laboratories
EPI_ISL_428312	hCoV-19/USA/WI-UW-216/2020	North America / USA / Wisconsin / Milwaukee	2020-03-25	University of Wisconsin-Madison AIDS Vaccine Research Laboratories	University of Wisconsin-Madison AIDS Vaccine Research Laboratories	Gage Moreno, Katarina Braun, et al. AIDS Vaccine Research Laboratories
EPI_ISL_428313	hCoV-19/USA/WI-UW-217/2020	North America / USA / Wisconsin / Milwaukee	2020-03-25	University of Wisconsin-Madison AIDS Vaccine Research Laboratories	University of Wisconsin-Madison AIDS Vaccine Research Laboratories	Gage Moreno, Katarina Braun, et al. AIDS Vaccine Research Laboratories
EPI_ISL_428314	hCoV-19/USA/WI-UW-218/2020	North America / USA / Wisconsin / Milwaukee	2020-03-25	University of Wisconsin-Madison AIDS Vaccine Research Laboratories	University of Wisconsin-Madison AIDS Vaccine Research Laboratories	Gage Moreno, Katarina Braun, et al. AIDS Vaccine Research Laboratories
EPI_ISL_428315	hCoV-19/USA/WI-UW-219/2020	North America / USA / Wisconsin / Milwaukee	2020-03-25	University of Wisconsin-Madison AIDS Vaccine Research Laboratories	University of Wisconsin-Madison AIDS Vaccine Research Laboratories	Gage Moreno, Katarina Braun, et al. AIDS Vaccine Research Laboratories
EPI_ISL_428316	hCoV-19/USA/WI-UW-220/2020	North America / USA / Wisconsin / Mequon	2020-03-25	University of Wisconsin-Madison AIDS Vaccine Research Laboratories	University of Wisconsin-Madison AIDS Vaccine Research Laboratories	Gage Moreno, Katarina Braun, et al. AIDS Vaccine Research Laboratories
EPI_ISL_428317	hCoV-19/USA/WI-UW-221/2020	North America / USA / Wisconsin / Milwaukee	2020-03-25	University of Wisconsin-Madison AIDS Vaccine Research Laboratories	University of Wisconsin-Madison AIDS Vaccine Research Laboratories	Gage Moreno, Katarina Braun, et al. AIDS Vaccine Research Laboratories
EPI_ISL_428318	hCoV-19/USA/WI-UW-222/2020	North America / USA / Wisconsin / Milwaukee	2020-03-25	University of Wisconsin-Madison AIDS Vaccine Research Laboratories	University of Wisconsin-Madison AIDS Vaccine Research Laboratories	Gage Moreno, Katarina Braun, et al. AIDS Vaccine Research Laboratories
EPI_ISL_428319	hCoV-19/USA/WI-UW-223/2020	North America / USA / Wisconsin / Milwaukee	2020-03-26	University of Wisconsin-Madison AIDS Vaccine Research Laboratories	University of Wisconsin-Madison AIDS Vaccine Research Laboratories	Gage Moreno, Katarina Braun, et al. AIDS Vaccine Research Laboratories
EPI_ISL_428320	hCoV-19/USA/WI-UW-224/2020	North America / USA / Wisconsin / Brookfield	2020-03-26	University of Wisconsin-Madison AIDS Vaccine Research Laboratories	University of Wisconsin-Madison AIDS Vaccine Research Laboratories	Gage Moreno, Katarina Braun, et al. AIDS Vaccine Research Laboratories
EPI_ISL_428321	hCoV-19/USA/WI-UW-225/2020	North America / USA / Wisconsin / Franklin	2020-03-26	University of Wisconsin-Madison AIDS Vaccine Research Laboratories	University of Wisconsin-Madison AIDS Vaccine Research Laboratories	Gage Moreno, Katarina Braun, et al. AIDS Vaccine Research Laboratories
EPI_ISL_428322	hCoV-19/USA/WI-UW-226/2020	North America / USA / Wisconsin / South Milwaukee	2020-03-26	University of Wisconsin-Madison AIDS Vaccine Research Laboratories	University of Wisconsin-Madison AIDS Vaccine Research Laboratories	Gage Moreno, Katarina Braun, et al. AIDS Vaccine Research Laboratories
EPI_ISL_428323	hCoV-19/USA/WI-UW-227/2020	North America / USA / Wisconsin / Milwaukee	2020-03-26	University of Wisconsin-Madison AIDS Vaccine Research Laboratories	University of Wisconsin-Madison AIDS Vaccine Research Laboratories	Gage Moreno, Katarina Braun, et al. AIDS Vaccine Research Laboratories
EPI_ISL_428324	hCoV-19/USA/WI-UW-228/2020	North America / USA / Wisconsin / Greenfield	2020-03-26	University of Wisconsin-Madison AIDS Vaccine Research Laboratories	University of Wisconsin-Madison AIDS Vaccine Research Laboratories	Gage Moreno, Katarina Braun, et al. AIDS Vaccine Research Laboratories
EPI_ISL_428325	hCoV-19/USA/WI-UW-229/2020	North America / USA / Wisconsin / Milwaukee	2020-03-26

University of Wisconsin-Madison AIDS Vaccine Research Laboratories	University of Wisconsin-Madison AIDS Vaccine Research Laboratories	Gage Moreno, Katarina Braun, et al. AIDS Vaccine Research Laboratories
EPI_ISL_428326	hCoV-19/USA/WI-UW-230/2020	North America / USA / Wisconsin / Milwaukee	2020-03-26	University of Wisconsin-Madison AIDS Vaccine Research Laboratories	University of Wisconsin-Madison AIDS Vaccine Research Laboratories	Gage Moreno, Katarina Braun, et al. AIDS Vaccine Research Laboratories
EPI_ISL_428327	hCoV-19/USA/WI-UW-231/2020	North America / USA / Wisconsin / Milwaukee	2020-03-26	University of Wisconsin-Madison AIDS Vaccine Research Laboratories	University of Wisconsin-Madison AIDS Vaccine Research Laboratories	Gage Moreno, Katarina Braun, et al. AIDS Vaccine Research Laboratories
EPI_ISL_428328	hCoV-19/USA/WI-UW-232/2020	North America / USA / Wisconsin / Glendale	2020-03-27	University of Wisconsin-Madison AIDS Vaccine Research Laboratories	University of Wisconsin-Madison AIDS Vaccine Research Laboratories	Gage Moreno, Katarina Braun, et al. AIDS Vaccine Research Laboratories
EPI_ISL_428329	hCoV-19/USA/WI-UW-233/2020	North America / USA / Wisconsin / Milwaukee	2020-03-27	University of Wisconsin-Madison AIDS Vaccine Research Laboratories	University of Wisconsin-Madison AIDS Vaccine Research Laboratories	Gage Moreno, Katarina Braun, et al. AIDS Vaccine Research Laboratories
EPI_ISL_428330	hCoV-19/USA/WI-UW-234/2020	North America / USA / Wisconsin / Milwaukee	2020-03-26	University of Wisconsin-Madison AIDS Vaccine Research Laboratories	University of Wisconsin-Madison AIDS Vaccine Research Laboratories	Gage Moreno, Katarina Braun, et al. AIDS Vaccine Research Laboratories
EPI_ISL_428331	hCoV-19/USA/WI-UW-235/2020	North America / USA / Wisconsin / Cudahy	2020-03-28	University of Wisconsin-Madison AIDS Vaccine Research Laboratories	University of Wisconsin-Madison AIDS Vaccine Research Laboratories	Gage Moreno, Katarina Braun, et al. AIDS Vaccine Research Laboratories
EPI_ISL_428332	hCoV-19/USA/WI-UW-236/2020	North America / USA / Wisconsin / Milwaukee	2020-03-27	University of Wisconsin-Madison AIDS Vaccine Research Laboratories	University of Wisconsin-Madison AIDS Vaccine Research Laboratories	Gage Moreno, Katarina Braun, et al. AIDS Vaccine Research Laboratories
EPI_ISL_428333	hCoV-19/USA/WI-UW-237/2020	North America / USA / Wisconsin / Whitefish	2020-03-27	University of Wisconsin-Madison AIDS Vaccine Research Laboratories	University of Wisconsin-Madison AIDS Vaccine Research Laboratories	Gage Moreno, Katarina Braun, et al. AIDS Vaccine Research Laboratories
EPI_ISL_428334	hCoV-19/USA/WI-UW-238/2020	North America / USA / Wisconsin / Milwaukee	2020-03-27	University of Wisconsin-Madison AIDS Vaccine Research Laboratories	University of Wisconsin-Madison AIDS Vaccine Research Laboratories	Gage Moreno, Katarina Braun, et al. AIDS Vaccine Research Laboratories
EPI_ISL_428335	hCoV-19/USA/WI-UW-239/2020	North America / USA / Wisconsin / Mequon	2020-03-27	University of Wisconsin-Madison AIDS Vaccine Research Laboratories	University of Wisconsin-Madison AIDS Vaccine Research Laboratories	Gage Moreno, Katarina Braun, et al. AIDS Vaccine Research Laboratories
EPI_ISL_428336	hCoV-19/USA/WI-UW-240/2020	North America / USA / Wisconsin / Milwaukee	2020-03-27	University of Wisconsin-Madison AIDS Vaccine Research Laboratories	University of Wisconsin-Madison AIDS Vaccine Research Laboratories	Gage Moreno, Katarina Braun, et al. AIDS Vaccine Research Laboratories
EPI_ISL_428337	hCoV-19/USA/WI-UW-241/2020	North America / USA / Wisconsin / Milwaukee	2020-03-27	University of Wisconsin-Madison AIDS Vaccine Research Laboratories	University of Wisconsin-Madison AIDS Vaccine Research Laboratories	Gage Moreno, Katarina Braun, et al. AIDS Vaccine Research Laboratories
EPI_ISL_428338	hCoV-19/USA/WI-UW-242/2020	North America / USA / Wisconsin / Milwaukee	2020-03-28	University of Wisconsin-Madison AIDS Vaccine Research Laboratories	University of Wisconsin-Madison AIDS Vaccine Research Laboratories	Gage Moreno, Katarina Braun, et al. AIDS Vaccine Research Laboratories
EPI_ISL_428339	hCoV-19/USA/WI-UW-243/2020	North America / USA / Wisconsin / Grafton	2020-03-28	University of Wisconsin-Madison AIDS Vaccine Research Laboratories	University of Wisconsin-Madison AIDS Vaccine Research Laboratories	Gage Moreno, Katarina Braun, et al. AIDS Vaccine Research Laboratories
EPI_ISL_428340	hCoV-19/USA/WI-UW-244/2020	North America / USA / Wisconsin / Thiensvill	2020-03-28	University of Wisconsin-Madison AIDS Vaccine Research Laboratories	University of Wisconsin-Madison AIDS Vaccine Research Laboratories	Gage Moreno, Katarina Braun, et al. AIDS Vaccine Research Laboratories
EPI_ISL_428341	hCoV-19/USA/WI-UW-245/2020	North America / USA / Wisconsin / Milwaukee	2020-03-28	University of Wisconsin-Madison AIDS Vaccine Research Laboratories	University of Wisconsin-Madison AIDS Vaccine Research Laboratories	Gage Moreno, Katarina Braun, et al. AIDS Vaccine Research Laboratories
EPI_ISL_428343	hCoV-19/USA/WI-UW-247/2020	North America / USA / Wisconsin / Port Washi	2020-03-28	University of Wisconsin-Madison AIDS Vaccine Research Laboratories	University of Wisconsin-Madison AIDS Vaccine Research Laboratories	Gage Moreno, Katarina Braun, et al. AIDS Vaccine Research Laboratories
EPI_ISL_428344	hCoV-19/USA/WI-UW-248/2020	North America / USA / Wisconsin / Milwaukee	2020-03-28	University of Wisconsin-Madison AIDS Vaccine Research Laboratories	University of Wisconsin-Madison AIDS Vaccine Research Laboratories	Gage Moreno, Katarina Braun, et al. AIDS Vaccine Research Laboratories
EPI_ISL_428345	hCoV-19/USA/WI-UW-249/2020	North America / USA / Wisconsin / Racine	2020-03-16	University of Wisconsin-Madison AIDS Vaccine Research Laboratories	University of Wisconsin-Madison AIDS Vaccine Research Laboratories	Gage Moreno, Katarina Braun, et al. AIDS Vaccine Research Laboratories
EPI_ISL_428346	hCoV-19/Turkey/GLAB-CoV012/2020 Europe / Turkey / Istanbul	2020-04-17	Genomic Laboratory (GLAB) (Conjoint lab of Health Directorate of Istanbul and Istanbul Technical University)	Genomic Laboratory (GLAB), Istanbul Technical University	Ilker Karacan, Tugba Kizilboga Akgun, Bugra Agaoglu, Gizem Alkurt, Jale Yildiz, Betsi Köse, Elifnaz Çelik, Arzu Irvem, Yasemin Kendir Demirkol, Ozlem Akgun Dogan, Mehtap Aydın, Levent Doganay, Gizem Dinler Doganay
EPI_ISL_428347	hCoV-19/France/IDF3384/2020	Europe / France / Ile De France / Longjumeau	2020-03-23	Service de Biologie Médicale - BP 125	National Reference Center for Viruses of Respiratory Infections, Institut Pasteur, Paris Mélanie Albert, Marion Barbet, Sylvie Behillil, Méline Bizard, Angela Brisebarre, Flora Donati, Etienne Simon-Lorière, Vincent Enouf, Maud Vanpeene, Sylvie van der Werf
EPI_ISL_428348	hCoV-19/France/GE3372/2020	Europe / France / Grand-est / Gondrecourt-le-chateau	2020-03-20	Maison de Santé du Val d'Ormois National Reference Center for Viruses of Respiratory Infections,

Institut Pasteur, Paris Mélanie Albert, Marion Barbet, Sylvie Behillil, Méline Bizard, Angela Brisebarre, Flora Donati, Etienne Simon-Lorière, Vincent Enouf, Maud Vanpeene, Sylvie van der Werf
EPI_ISL_428349 hCoV-19/France/IDF3386/2020 Europe / France / Ile De France / Longjumeau 2020-03-23 Service de Biologie Médicale - BP 125 National Reference Center for Viruses of Respiratory Infections, Institut Pasteur, Paris Mélanie Albert, Marion Barbet, Sylvie Behillil, Méline Bizard, Angela Brisebarre, Flora Donati, Etienne Simon-Lorière, Vincent Enouf, Maud Vanpeene, Sylvie van der Werf
EPI_ISL_428350 hCoV-19/France/HF3419/2020 Europe / France / Hauts De France / Chateau-thierry 2020-03-24 CH Jean de Navarre Laboratoire de Biologie National Reference Center for Viruses of Respiratory Infections, Institut Pasteur, Paris Mélanie Albert, Marion Barbet, Sylvie Behillil, Méline Bizard, Angela Brisebarre, Flora Donati, Etienne Simon-Lorière, Vincent Enouf, Maud Vanpeene, Sylvie van der Werf
EPI_ISL_428351 hCoV-19/France/IDF3509/2020 Europe / France / Ile De France / Orsay 2020-03-24 GH Nord Essonne Service de Biologie clinique National Reference Center for Viruses of Respiratory Infections, Institut Pasteur, Paris Mélanie Albert, Marion Barbet, Sylvie Behillil, Méline Bizard, Angela Brisebarre, Flora Donati, Etienne Simon-Lorière, Vincent Enouf, Maud Vanpeene, Sylvie van der Werf
EPI_ISL_428352 hCoV-19/France/IDF3518/2020 Europe / France / Ile De France / Orsay 2020-03-23 GH Nord Essonne Service de Biologie clinique National Reference Center for Viruses of Respiratory Infections, Institut Pasteur, Paris Mélanie Albert, Marion Barbet, Sylvie Behillil, Méline Bizard, Angela Brisebarre, Flora Donati, Etienne Simon-Lorière, Vincent Enouf, Maud Vanpeene, Sylvie van der Werf
EPI_ISL_428353 hCoV-19/France/HF3534/2020 Europe / France / Hauts De France / Compiegne 2020-03-24 CH Compiègne Laboratoire de Biologie National Reference Center for Viruses of Respiratory Infections, Institut Pasteur, Paris Mélanie Albert, Marion Barbet, Sylvie Behillil, Méline Bizard, Angela Brisebarre, Flora Donati, Etienne Simon-Lorière, Vincent Enouf, Maud Vanpeene, Sylvie van der Werf
EPI_ISL_428354 hCoV-19/France/IDF3577/2020 Europe / France / Ile De France / Longjumeau 2020-03-25 LABM GH nord Essonne de Longjumeau - BP 125 National Reference Center for Viruses of Respiratory Infections, Institut Pasteur, Paris Mélanie Albert, Marion Barbet, Sylvie Behillil, Méline Bizard, Angela Brisebarre, Flora Donati, Etienne Simon-Lorière, Vincent Enouf, Maud Vanpeene, Sylvie van der Werf
EPI_ISL_428355 hCoV-19/France/IDF2251/2020 Europe / France / Ile De France / Garches 2020-03-11 Institut Médico légal- Hop R. Poincaré National Reference Center for Viruses of Respiratory Infections, Institut Pasteur, Paris Mélanie Albert, Marion Barbet, Sylvie Behillil, Méline Bizard, Angela Brisebarre, Flora Donati, Etienne Simon-Lorière, Vincent Enouf, Maud Vanpeene, Sylvie van der Werf
EPI_ISL_428356 hCoV-19/France/IDF2252/2020 Europe / France / Ile De France / Garches 2020-03-11 Institut Médico légal- Hop R. Poincaré National Reference Center for Viruses of Respiratory Infections, Institut Pasteur, Paris Mélanie Albert, Marion Barbet, Sylvie Behillil, Méline Bizard, Angela Brisebarre, Flora Donati, Etienne Simon-Lorière, Vincent Enouf, Maud Vanpeene, Sylvie van der Werf
EPI_ISL_428357 hCoV-19/France/IDF2253/2020 Europe / France / Ile De France / Garches 2020-03-11 Institut Médico légal- Hop R. Poincaré National Reference Center for Viruses of Respiratory Infections, Institut Pasteur, Paris Mélanie Albert, Marion Barbet, Sylvie Behillil, Méline Bizard, Angela Brisebarre, Flora Donati, Etienne Simon-Lorière, Vincent Enouf, Maud Vanpeene, Sylvie van der Werf
EPI_ISL_428358 hCoV-19/France/HF3598/2020 Europe / France / Hauts De France / Chateau-thierry 2020-03-24 CH Jeanne de Navarre Laboratoire de Biologie National Reference Center for Viruses of Respiratory Infections, Institut Pasteur, Paris Mélanie Albert, Marion Barbet, Sylvie Behillil, Méline Bizard, Angela Brisebarre, Flora Donati, Etienne Simon-Lorière, Vincent Enouf, Maud Vanpeene, Sylvie van der Werf
EPI_ISL_428359 hCoV-19/France/HF3677/2020 Europe / France / Hauts De France / Compiegne 2020-03-23 CH Compiègne Laboratoire de Biologie National Reference Center for Viruses of Respiratory Infections, Institut Pasteur, Paris Mélanie Albert, Marion Barbet, Sylvie Behillil, Méline Bizard, Angela Brisebarre, Flora Donati, Etienne Simon-Lorière, Vincent Enouf, Maud Vanpeene, Sylvie van der Werf
EPI_ISL_428360 hCoV-19/France/HF3678/2020 Europe / France / Hauts De France / Compiegne 2020-03-25 CH Compiègne Laboratoire de Biologie National Reference Center for Viruses of Respiratory Infections, Institut Pasteur, Paris Mélanie Albert, Marion Barbet, Sylvie Behillil, Méline Bizard, Angela Brisebarre, Flora Donati, Etienne Simon-Lorière, Vincent Enouf, Maud Vanpeene, Sylvie van der Werf
EPI_ISL_428361 hCoV-19/France/IDF3703/2020 Europe / France / Ile De France / Longjumeau 2020-03-25 LABM GH nord Essonne de Longjumeau - BP 125 National Reference Center for Viruses of Respiratory Infections, Institut Pasteur, Paris Mélanie Albert, Marion Barbet, Sylvie Behillil, Méline Bizard, Angela Brisebarre, Flora Donati, Etienne Simon-Lorière, Vincent Enouf, Maud Vanpeene, Sylvie van der Werf
EPI_ISL_428363 hCoV-19/France/IDF3745/2020 Europe / France / Ile De France / Orsay 2020-03-26 GH Nord Essonne Service de Biologie clinique National Reference Center for Viruses of Respiratory Infections, Institut Pasteur, Paris Mélanie Albert, Marion Barbet, Sylvie Behillil, Méline Bizard, Angela Brisebarre, Flora Donati, Etienne Simon-Lorière, Vincent Enouf, Maud Vanpeene, Sylvie van der Werf
EPI_ISL_428365 hCoV-19/France/IDF3930/2020 Europe / France / Ile De France / Longjumeau 2020-03-26 LABM GH nord Essonne de Longjumeau - BP 125 National Reference Center for Viruses of Respiratory Infections, Institut Pasteur, Paris Mélanie Albert, Marion Barbet, Sylvie Behillil, Méline Bizard, Angela Brisebarre, Flora Donati, Etienne Simon-Lorière, Vincent Enouf, Maud Vanpeene, Sylvie van der Werf
EPI_ISL_428366 hCoV-19/France/HF4220/2020 Europe / France / Hauts De France / Chateau-thierry 2020-03-30 CH Jeanne de Navarre Laboratoire de Biologie National Reference Center for Viruses of Respiratory Infections, Institut Pasteur, Paris Mélanie Albert, Marion Barbet, Sylvie Behillil, Méline Bizard, Angela Brisebarre, Flora Donati, Etienne Simon-Lorière, Vincent Enouf, Maud Vanpeene, Sylvie van der Werf
EPI_ISL_428367 hCoV-19/France/N4427/2020 Europe / France / Normandie / Carentan 2020-03-23 Cabinet Médical National Reference Center for Viruses of Respiratory Infections, Institut Pasteur, Paris Mélanie Albert, Marion Barbet, Sylvie Behillil, Méline Bizard, Angela Brisebarre, Flora Donati, Etienne Simon-Lorière, Vincent Enouf, Maud Vanpeene, Sylvie van der Werf

EPI_ISL_428368 hCoV-19/Turkey/GLAB-CoV013/2020 Europe / Turkey / Istanbul 2020-04-16 Genomic Laboratory (GLAB) (Conjoint lab of Health Directorate of Istanbul and Istanbul Technical University) Genomic Laboratory (GLAB), Istanbul Technical University Ilker Karacan, Tugba Kizilboga Akgun, Bugra Agaoglu, Gizem Alkurt, Jale Yildiz, Betsi Köse, Elifnaz Çelik, Arzu Irvem, Yasemin Kendir Demirkol, Ozlem Akgun Dogan, Mehtap Aydın, Levent Doganay, Gizem Dinler Doganay
EPI_ISL_428385 hCoV-19/USA/CT-Yale-066/2020 North America / USA / Connecticut 2020-04-04 Yale COVID-19 Biorepository Grubaugh Lab - Yale School of Public Health Joseph Fauver, Tara Alpert, Anderson Brito, Anne Wyllie, Chantal Vogels, Mary Petrone, Chaney Kalinich, Isabel Ott, Arnau Casanovas, Catherine Muenker, Adam Moore, Alice Lu, Maria Tokuyama, Patrick Wong, Peiwen Lu, Saad Omer, Richard Martinello, Allison Nelson, Shelli Farhadian, Akiko Iwasaki, Charlese Dela Cruz, Albert Ko, Nathan Grubaugh
EPI_ISL_429350 hCoV-19/Denmark/ALAB-SSI121/2020 Europe / Denmark / Unknown 2020-03-08 Department of Virus and Microbiological Special Diagnostics, Statens Serum Institut, Copenhagen, Denmark, Artillerivej 5, 2300 Copenahgen S Albertsen lab, Department of Chemistry and Bioscience, Aalborg University, Denmark Rasmus Kirkegaard
EPI_ISL_429351 hCoV-19/Denmark/ALAB-SSI122/2020 Europe / Denmark / Unknown 2020-03-08 Department of Virus and Microbiological Special Diagnostics, Statens Serum Institut, Copenhagen, Denmark, Artillerivej 5, 2300 Copenahgen S Albertsen lab, Department of Chemistry and Bioscience, Aalborg University, Denmark Rasmus Kirkegaard
EPI_ISL_429352 hCoV-19/Denmark/ALAB-SSI123/2020 Europe / Denmark / Unknown 2020-03-08 Department of Virus and Microbiological Special Diagnostics, Statens Serum Institut, Copenhagen, Denmark, Artillerivej 5, 2300 Copenahgen S Albertsen lab, Department of Chemistry and Bioscience, Aalborg University, Denmark Rasmus Kirkegaard
EPI_ISL_429354 hCoV-19/Denmark/ALAB-SSI126/2020 Europe / Denmark / Unknown 2020-03-08 Department of Virus and Microbiological Special Diagnostics, Statens Serum Institut, Copenhagen, Denmark, Artillerivej 5, 2300 Copenahgen S Albertsen lab, Department of Chemistry and Bioscience, Aalborg University, Denmark Rasmus Kirkegaard
EPI_ISL_429370 hCoV-19/Denmark/ALAB-SSI154/2020 Europe / Denmark / Unknown 2020-03-09 Department of Virus and Microbiological Special Diagnostics, Statens Serum Institut, Copenhagen, Denmark, Artillerivej 5, 2300 Copenahgen S Albertsen lab, Department of Chemistry and Bioscience, Aalborg University, Denmark Rasmus Kirkegaard
EPI_ISL_429371 hCoV-19/Denmark/ALAB-SSI155/2020 Europe / Denmark / Unknown 2020-03-10 Department of Virus and Microbiological Special Diagnostics, Statens Serum Institut, Copenhagen, Denmark, Artillerivej 5, 2300 Copenahgen S Albertsen lab, Department of Chemistry and Bioscience, Aalborg University, Denmark Rasmus Kirkegaard
EPI_ISL_429372 hCoV-19/Denmark/ALAB-SSI156/2020 Europe / Denmark / Unknown 2020-03-10 Department of Virus and Microbiological Special Diagnostics, Statens Serum Institut, Copenhagen, Denmark, Artillerivej 5, 2300 Copenahgen S Albertsen lab, Department of Chemistry and Bioscience, Aalborg University, Denmark Rasmus Kirkegaard
EPI_ISL_429373 hCoV-19/Denmark/ALAB-SSI157/2020 Europe / Denmark / Unknown 2020-03-09 Department of Virus and Microbiological Special Diagnostics, Statens Serum Institut, Copenhagen, Denmark, Artillerivej 5, 2300 Copenahgen S Albertsen lab, Department of Chemistry and Bioscience, Aalborg University, Denmark Rasmus Kirkegaard
EPI_ISL_429374 hCoV-19/Denmark/ALAB-SSI158/2020 Europe / Denmark / Unknown 2020-03-09 Department of Virus and Microbiological Special Diagnostics, Statens Serum Institut, Copenhagen, Denmark, Artillerivej 5, 2300 Copenahgen S Albertsen lab, Department of Chemistry and Bioscience, Aalborg University, Denmark Rasmus Kirkegaard
EPI_ISL_429375 hCoV-19/Denmark/ALAB-SSI159/2020 Europe / Denmark / Unknown 2020-03-09 Department of Virus and Microbiological Special Diagnostics, Statens Serum Institut, Copenhagen, Denmark, Artillerivej 5, 2300 Copenahgen S Albertsen lab, Department of Chemistry and Bioscience, Aalborg University, Denmark Rasmus Kirkegaard
EPI_ISL_429376 hCoV-19/Denmark/ALAB-SSI160/2020 Europe / Denmark / Unknown 2020-03-09 Department of Virus and Microbiological Special Diagnostics, Statens Serum Institut, Copenhagen, Denmark, Artillerivej 5, 2300 Copenahgen S Albertsen lab, Department of Chemistry and Bioscience, Aalborg University, Denmark Rasmus Kirkegaard
EPI_ISL_429377 hCoV-19/Denmark/ALAB-SSI162/2020 Europe / Denmark / Unknown 2020-03-09 Department of Virus and Microbiological Special Diagnostics, Statens Serum Institut, Copenhagen, Denmark, Artillerivej 5, 2300 Copenahgen S Albertsen lab, Department of Chemistry and Bioscience, Aalborg University, Denmark Rasmus Kirkegaard
EPI_ISL_429378 hCoV-19/Denmark/ALAB-SSI163/2020 Europe / Denmark / Unknown 2020-03-09 Department of Virus and Microbiological Special Diagnostics, Statens Serum Institut, Copenhagen, Denmark, Artillerivej 5, 2300 Copenahgen S Albertsen lab, Department of Chemistry and Bioscience, Aalborg University, Denmark Rasmus Kirkegaard
EPI_ISL_429379 hCoV-19/Denmark/ALAB-SSI164/2020 Europe / Denmark / Unknown 2020-03-09 Department of Virus and Microbiological Special Diagnostics, Statens Serum Institut, Copenhagen, Denmark, Artillerivej 5, 2300 Copenahgen S Albertsen lab, Department of Chemistry and Bioscience, Aalborg University, Denmark Rasmus Kirkegaard
EPI_ISL_429381 hCoV-19/Denmark/ALAB-SSI167/2020 Europe / Denmark / Unknown 2020-03-09 Department of Virus and Microbiological Special Diagnostics, Statens Serum Institut, Copenhagen, Denmark, Artillerivej 5, 2300 Copenahgen S Albertsen lab, Department of Chemistry and Bioscience, Aalborg University, Denmark Rasmus Kirkegaard

EPI_ISL_429382 hCoV-19/Denmark/ALAB-SSI168/2020 Europe / Denmark / Unknown 2020-03-09 Department of Virus and Microbiological Special Diagnostics, Statens Serum Institut, Copenhagen, Denmark, Artillerivej 5, 2300 Copenahgen S Albertsen lab, Department of Chemistry and Bioscience, Aalborg University, Denmark Rasmus Kirkegaard
EPI_ISL_429383 hCoV-19/Denmark/ALAB-SSI169/2020 Europe / Denmark / Unknown 2020-03-09 Department of Virus and Microbiological Special Diagnostics, Statens Serum Institut, Copenhagen, Denmark, Artillerivej 5, 2300 Copenahgen S Albertsen lab, Department of Chemistry and Bioscience, Aalborg University, Denmark Rasmus Kirkegaard
EPI_ISL_429384 hCoV-19/Denmark/ALAB-SSI171/2020 Europe / Denmark / Unknown 2020-03-09 Department of Virus and Microbiological Special Diagnostics, Statens Serum Institut, Copenhagen, Denmark, Artillerivej 5, 2300 Copenahgen S Albertsen lab, Department of Chemistry and Bioscience, Aalborg University, Denmark Rasmus Kirkegaard
EPI_ISL_429385 hCoV-19/Denmark/ALAB-SSI172/2020 Europe / Denmark / Unknown 2020-03-09 Department of Virus and Microbiological Special Diagnostics, Statens Serum Institut, Copenhagen, Denmark, Artillerivej 5, 2300 Copenahgen S Albertsen lab, Department of Chemistry and Bioscience, Aalborg University, Denmark Rasmus Kirkegaard
EPI_ISL_429386 hCoV-19/Denmark/ALAB-SSI173/2020 Europe / Denmark / Unknown 2020-03-09 Department of Virus and Microbiological Special Diagnostics, Statens Serum Institut, Copenhagen, Denmark, Artillerivej 5, 2300 Copenahgen S Albertsen lab, Department of Chemistry and Bioscience, Aalborg University, Denmark Rasmus Kirkegaard
EPI_ISL_429387 hCoV-19/Denmark/ALAB-SSI174/2020 Europe / Denmark / Unknown 2020-03-09 Department of Virus and Microbiological Special Diagnostics, Statens Serum Institut, Copenhagen, Denmark, Artillerivej 5, 2300 Copenahgen S Albertsen lab, Department of Chemistry and Bioscience, Aalborg University, Denmark Rasmus Kirkegaard
EPI_ISL_429501 hCoV-19/Denmark/ALAB-SSI407/2020 Europe / Denmark / Unknown 2020-03-10 Department of Virus and Microbiological Special Diagnostics, Statens Serum Institut, Copenhagen, Denmark, Artillerivej 5, 2300 Copenahgen S Albertsen lab, Department of Chemistry and Bioscience, Aalborg University, Denmark Rasmus Kirkegaard
EPI_ISL_429502 hCoV-19/Denmark/ALAB-SSI408/2020 Europe / Denmark / Unknown 2020-03-10 Department of Virus and Microbiological Special Diagnostics, Statens Serum Institut, Copenhagen, Denmark, Artillerivej 5, 2300 Copenahgen S Albertsen lab, Department of Chemistry and Bioscience, Aalborg University, Denmark Rasmus Kirkegaard
EPI_ISL_429503 hCoV-19/Denmark/ALAB-SSI409/2020 Europe / Denmark / Unknown 2020-03-10 Department of Virus and Microbiological Special Diagnostics, Statens Serum Institut, Copenhagen, Denmark, Artillerivej 5, 2300 Copenahgen S Albertsen lab, Department of Chemistry and Bioscience, Aalborg University, Denmark Rasmus Kirkegaard
EPI_ISL_429505 hCoV-19/Denmark/ALAB-SSI411/2020 Europe / Denmark / Unknown 2020-03-10 Department of Virus and Microbiological Special Diagnostics, Statens Serum Institut, Copenhagen, Denmark, Artillerivej 5, 2300 Copenahgen S Albertsen lab, Department of Chemistry and Bioscience, Aalborg University, Denmark Rasmus Kirkegaard
EPI_ISL_429506 hCoV-19/Denmark/ALAB-SSI411A/2020 Europe / Denmark / Unknown 2020-03-23 Department of Virus and Microbiological Special Diagnostics, Statens Serum Institut, Copenhagen, Denmark, Artillerivej 5, 2300 Copenahgen S Albertsen lab, Department of Chemistry and Bioscience, Aalborg University, Denmark Rasmus Kirkegaard
EPI_ISL_429507 hCoV-19/Denmark/ALAB-SSI412/2020 Europe / Denmark / Unknown 2020-03-10 Department of Virus and Microbiological Special Diagnostics, Statens Serum Institut, Copenhagen, Denmark, Artillerivej 5, 2300 Copenahgen S Albertsen lab, Department of Chemistry and Bioscience, Aalborg University, Denmark Rasmus Kirkegaard
EPI_ISL_429600 hCoV-19/USA/WA-UW-4549/2020 North America / USA / Washington 2020-03-31 UW Virology Lab UW Virology Lab Pavitra Roychoudhury, Hong Xie, Keith Jerome, Alexander Greninger
EPI_ISL_429601 hCoV-19/USA/ID-UW-4552/2020 North America / USA / Idaho 2020-03-29 UW Virology Lab UW Virology Lab Pavitra Roychoudhury, Hong Xie, Keith Jerome, Alexander Greninger
EPI_ISL_429602 hCoV-19/USA/WA-UW-4564/2020 North America / USA / Washington 2020-03-30 UW Virology Lab UW Virology Lab Pavitra Roychoudhury, Hong Xie, Keith Jerome, Alexander Greninger
EPI_ISL_429603 hCoV-19/USA/WA-UW-4565/2020 North America / USA / Washington 2020-03-30 UW Virology Lab UW Virology Lab Pavitra Roychoudhury, Hong Xie, Keith Jerome, Alexander Greninger
EPI_ISL_429604 hCoV-19/USA/WA-UW-4568/2020 North America / USA / Washington 2020-03-29 UW Virology Lab UW Virology Lab Pavitra Roychoudhury, Hong Xie, Keith Jerome, Alexander Greninger
EPI_ISL_429605 hCoV-19/USA/WA-UW-4578/2020 North America / USA / Washington 2020-03-31 UW Virology Lab UW Virology Lab Pavitra Roychoudhury, Hong Xie, Keith Jerome, Alexander Greninger
EPI_ISL_429606 hCoV-19/USA/WA-UW-4579/2020 North America / USA / Washington 2020-03-31 UW Virology Lab UW Virology Lab Pavitra Roychoudhury, Hong Xie, Keith Jerome, Alexander Greninger
EPI_ISL_429608 hCoV-19/USA/WA-UW-4593/2020 North America / USA / Washington 2020-03-31 UW Virology Lab UW Virology Lab Pavitra Roychoudhury, Hong Xie, Keith Jerome, Alexander Greninger
EPI_ISL_429609 hCoV-19/USA/WA-UW-4594/2020 North America / USA / Washington 2020-03-31 UW Virology Lab UW Virology Lab Pavitra Roychoudhury, Hong Xie, Keith Jerome, Alexander Greninger
EPI_ISL_429610 hCoV-19/USA/ID-UW-4598/2020 North America / USA / Idaho 2020-03-30 UW Virology Lab UW Virology Lab Pavitra Roychoudhury, Hong Xie, Keith Jerome, Alexander Greninger
EPI_ISL_429611 hCoV-19/USA/WA-UW-4602/2020 North America / USA / Washington 2020-03-31 UW Virology Lab UW Virology Lab Pavitra Roychoudhury, Hong Xie, Keith Jerome, Alexander Greninger

EPI_ISL_429612 hCoV-19/USA/WA-UW-4609/2020 North America / USA / Washington 2020-03-26 UW Virology Lab UW Virology Lab Pavitra Roychoudhury, Hong Xie, Keith Jerome, Alexander Greninger
EPI_ISL_429613 hCoV-19/USA/WA-UW-4611/2020 North America / USA / Washington 2020-03-26 UW Virology Lab UW Virology Lab Pavitra Roychoudhury, Hong Xie, Keith Jerome, Alexander Greninger
EPI_ISL_429614 hCoV-19/USA/WA-UW-4612/2020 North America / USA / Washington 2020-03-26 UW Virology Lab UW Virology Lab Pavitra Roychoudhury, Hong Xie, Keith Jerome, Alexander Greninger
EPI_ISL_429615 hCoV-19/USA/WA-UW-4703/2020 North America / USA / Washington 2020-03-29 UW Virology Lab UW Virology Lab Pavitra Roychoudhury, Hong Xie, Keith Jerome, Alexander Greninger
EPI_ISL_429616 hCoV-19/USA/WA-UW-4732/2020 North America / USA / Washington 2020-03-29 UW Virology Lab UW Virology Lab Pavitra Roychoudhury, Hong Xie, Keith Jerome, Alexander Greninger
EPI_ISL_429617 hCoV-19/USA/WA-UW-4734/2020 North America / USA / Washington 2020-03-28 UW Virology Lab UW Virology Lab Pavitra Roychoudhury, Hong Xie, Keith Jerome, Alexander Greninger
EPI_ISL_429618 hCoV-19/USA/WA-UW-4741/2020 North America / USA / Washington 2020-03-25 UW Virology Lab UW Virology Lab Pavitra Roychoudhury, Hong Xie, Keith Jerome, Alexander Greninger
EPI_ISL_429621 hCoV-19/USA/WA-UW-4760/2020 North America / USA / Washington 2020-03-28 UW Virology Lab UW Virology Lab Pavitra Roychoudhury, Hong Xie, Keith Jerome, Alexander Greninger
EPI_ISL_429622 hCoV-19/USA/WA-UW-4763/2020 North America / USA / Washington 2020-03-27 UW Virology Lab UW Virology Lab Pavitra Roychoudhury, Hong Xie, Keith Jerome, Alexander Greninger
EPI_ISL_429623 hCoV-19/USA/WA-UW-4766/2020 North America / USA / Washington 2020-03-28 UW Virology Lab UW Virology Lab Pavitra Roychoudhury, Hong Xie, Keith Jerome, Alexander Greninger
EPI_ISL_429624 hCoV-19/USA/WA-UW-4769/2020 North America / USA / Washington 2020-03-27 UW Virology Lab UW Virology Lab Pavitra Roychoudhury, Hong Xie, Keith Jerome, Alexander Greninger
EPI_ISL_429625 hCoV-19/USA/WA-UW-4770/2020 North America / USA / Washington 2020-03-27 UW Virology Lab UW Virology Lab Pavitra Roychoudhury, Hong Xie, Keith Jerome, Alexander Greninger
EPI_ISL_429626 hCoV-19/USA/WA-UW-4775/2020 North America / USA / Washington 2020-03-28 UW Virology Lab UW Virology Lab Pavitra Roychoudhury, Hong Xie, Keith Jerome, Alexander Greninger
EPI_ISL_429627 hCoV-19/USA/WA-UW-4777/2020 North America / USA / Washington 2020-03-27 UW Virology Lab UW Virology Lab Pavitra Roychoudhury, Hong Xie, Keith Jerome, Alexander Greninger
EPI_ISL_429628 hCoV-19/USA/UN-UW-4645/2020 North America / USA 2020-04-06 UW Virology Lab UW Virology Lab Pavitra Roychoudhury, Hong Xie, Keith Jerome, Alexander Greninger
EPI_ISL_429629 hCoV-19/USA/WA-UW-4649/2020 North America / USA / Washington 2020-04-06 UW Virology Lab UW Virology Lab Pavitra Roychoudhury, Hong Xie, Keith Jerome, Alexander Greninger
EPI_ISL_429630 hCoV-19/USA/WA-UW-4677/2020 North America / USA / Washington 2020-04-05 UW Virology Lab UW Virology Lab Pavitra Roychoudhury, Hong Xie, Keith Jerome, Alexander Greninger
EPI_ISL_429631 hCoV-19/USA/WA-UW-4695/2020 North America / USA / Washington 2020-04-06 UW Virology Lab UW Virology Lab Pavitra Roychoudhury, Hong Xie, Keith Jerome, Alexander Greninger
EPI_ISL_429632 hCoV-19/USA/WA-UW-4705/2020 North America / USA / Washington 2020-04-06 UW Virology Lab UW Virology Lab Pavitra Roychoudhury, Hong Xie, Keith Jerome, Alexander Greninger
EPI_ISL_429634 hCoV-19/USA/WA-UW-4892/2020 North America / USA / Washington 2020-04-07 UW Virology Lab UW Virology Lab Pavitra Roychoudhury, Hong Xie, Keith Jerome, Alexander Greninger
EPI_ISL_429635 hCoV-19/USA/WA-UW-4897/2020 North America / USA / Washington 2020-04-08 UW Virology Lab UW Virology Lab Pavitra Roychoudhury, Hong Xie, Keith Jerome, Alexander Greninger
EPI_ISL_429636 hCoV-19/USA/WA-UW-4921/2020 North America / USA / Washington 2020-04-07 UW Virology Lab UW Virology Lab Pavitra Roychoudhury, Hong Xie, Keith Jerome, Alexander Greninger
EPI_ISL_429637 hCoV-19/USA/WA-UW-4925/2020 North America / USA / Washington 2020-04-07 UW Virology Lab UW Virology Lab Pavitra Roychoudhury, Hong Xie, Keith Jerome, Alexander Greninger
EPI_ISL_429638 hCoV-19/USA/WA-UW-4999/2020 North America / USA / Washington 2020-04-07 UW Virology Lab UW Virology Lab Pavitra Roychoudhury, Hong Xie, Keith Jerome, Alexander Greninger
EPI_ISL_429639 hCoV-19/USA/WA-UW-5025/2020 North America / USA / Washington 2020-04-07 UW Virology Lab UW Virology Lab Pavitra Roychoudhury, Hong Xie, Keith Jerome, Alexander Greninger
EPI_ISL_429641 hCoV-19/USA/CT-UW-5038/2020 North America / USA / Connecticut 2020-04-06 UW Virology Lab UW Virology Lab Pavitra Roychoudhury, Hong Xie, Keith Jerome, Alexander Greninger
EPI_ISL_429642 hCoV-19/USA/CT-UW-5043/2020 North America / USA / Connecticut 2020-04-06 UW Virology Lab UW Virology Lab Pavitra Roychoudhury, Hong Xie, Keith Jerome, Alexander Greninger
EPI_ISL_429643 hCoV-19/USA/UN-UW-5044/2020 North America / USA 2020-04-08 UW Virology Lab UW Virology Lab Pavitra Roychoudhury, Hong Xie, Keith Jerome, Alexander Greninger
EPI_ISL_429644 hCoV-19/USA/WA-UW-5065/2020 North America / USA / Washington 2020-04-08 UW Virology Lab UW Virology Lab Pavitra Roychoudhury, Hong Xie, Keith Jerome, Alexander Greninger
EPI_ISL_429645 hCoV-19/USA/CT-UW-5073/2020 North America / USA / Connecticut 2020-04-06 UW Virology Lab UW Virology Lab Pavitra Roychoudhury, Hong Xie, Keith Jerome, Alexander Greninger
EPI_ISL_429646 hCoV-19/USA/CT-UW-5086/2020 North America / USA / Connecticut 2020-04-06 UW Virology Lab UW Virology Lab Pavitra Roychoudhury, Hong Xie, Keith Jerome, Alexander Greninger
EPI_ISL_429647 hCoV-19/USA/WA-UW-5095/2020 North America / USA / Washington 2020-04-08 UW Virology Lab UW Virology Lab Pavitra Roychoudhury, Hong Xie, Keith Jerome, Alexander Greninger
EPI_ISL_429649 hCoV-19/USA/WA-UW-5144/2020 North America / USA / Washington 2020-04-02 UW Virology Lab UW Virology Lab Pavitra Roychoudhury, Hong Xie, Keith Jerome, Alexander Greninger
EPI_ISL_429650 hCoV-19/USA/WA-UW-5152/2020 North America / USA / Washington 2020-04-02 UW Virology Lab UW Virology Lab Pavitra Roychoudhury, Hong Xie, Keith Jerome, Alexander Greninger
EPI_ISL_429651 hCoV-19/USA/WA-UW-5158/2020 North America / USA / Washington 2020-04-02 UW Virology Lab UW Virology Lab Pavitra Roychoudhury, Hong Xie, Keith Jerome, Alexander Greninger

EPI_ISL_429652 hCoV-19/USA/WA-UW-5164/2020 North America / USA / Washington 2020-04-02 UW Virology Lab UW Virology Lab Pavitra Roychoudhury, Hong Xie, Keith Jerome, Alexander Greninger
EPI_ISL_429653 hCoV-19/USA/WA-UW-5166/2020 North America / USA / Washington 2020-04-02 UW Virology Lab UW Virology Lab Pavitra Roychoudhury, Hong Xie, Keith Jerome, Alexander Greninger
EPI_ISL_429654 hCoV-19/USA/WA-UW-5169/2020 North America / USA / Washington 2020-04-02 UW Virology Lab UW Virology Lab Pavitra Roychoudhury, Hong Xie, Keith Jerome, Alexander Greninger
EPI_ISL_429655 hCoV-19/USA/WA-UW-5170/2020 North America / USA / Washington 2020-04-02 UW Virology Lab UW Virology Lab Pavitra Roychoudhury, Hong Xie, Keith Jerome, Alexander Greninger
EPI_ISL_429656 hCoV-19/USA/UN-UW-5172/2020 North America / USA 2020-04-02 UW Virology Lab UW Virology Lab Pavitra Roychoudhury, Hong Xie, Keith Jerome, Alexander Greninger
EPI_ISL_429667 hCoV-19/Brazil/CV4/2020 South America / Brazil / Minas gerais 2020-03-09 Central Public Health Laboratory/Octávio Magalhães Institute (IOM) from the Ezequiel Dias Foundation (FUNED) Instituto Octávio Magalhães / Fundação Ezequiel Dias (IOM/Funed) Talita Adelino, Joilson Xavier, Marta Giovanetti, Vagner Fonseca, Marcos Vinícius Silva, Luiz Carlos Junior Alcantara, Marluce Aparecida Assunção Oliveira
EPI_ISL_429669 hCoV-19/Brazil/CV6/2020 South America / Brazil / Minas gerais 2020-03-13 Central Public Health Laboratory/Octávio Magalhães Institute (IOM) from the Ezequiel Dias Foundation (FUNED) Instituto Octávio Magalhães / Fundação Ezequiel Dias (IOM/Funed) Talita Adelino, Joilson Xavier, Marta Giovanetti, Vagner Fonseca, Marcos Vinícius Silva, Luiz Carlos Junior Alcantara, Marluce Aparecida Assunção Oliveira
EPI_ISL_429676 hCoV-19/Brazil/CV16/2020 South America / Brazil / Minas gerais 2020-03-16 Central Public Health Laboratory/Octávio Magalhães Institute (IOM) from the Ezequiel Dias Foundation (FUNED) Instituto Octávio Magalhães / Fundação Ezequiel Dias (IOM/Funed) Talita Adelino, Joilson Xavier, Marta Giovanetti, Vagner Fonseca, Marcos Vinícius Silva, Luiz Carlos Junior Alcantara, Marluce Aparecida Assunção Oliveira
EPI_ISL_429681 hCoV-19/Brazil/CV21/2020 South America / Brazil / Minas gerais 2020-03-16 Central Public Health Laboratory/Octávio Magalhães Institute (IOM) from the Ezequiel Dias Foundation (FUNED) Instituto Octávio Magalhães / Fundação Ezequiel Dias (IOM/Funed) Talita Adelino, Joilson Xavier, Marta Giovanetti, Vagner Fonseca, Marcos Vinícius Silva, Luiz Carlos Junior Alcantara, Marluce Aparecida Assunção Oliveira
EPI_ISL_429687 hCoV-19/Brazil/CV31/2020 South America / Brazil / Minas gerais 2020-03-17 Central Public Health Laboratory/Octávio Magalhães Institute (IOM) from the Ezequiel Dias Foundation (FUNED) Instituto Octávio Magalhães / Fundação Ezequiel Dias (IOM/Funed) Talita Adelino, Joilson Xavier, Marta Giovanetti, Vagner Fonseca, Marcos Vinícius Silva, Luiz Carlos Junior Alcantara, Marluce Aparecida Assunção Oliveira
EPI_ISL_429688 hCoV-19/Brazil/CV32/2020 South America / Brazil / Minas gerais 2020-03-17 Central Public Health Laboratory/Octávio Magalhães Institute (IOM) from the Ezequiel Dias Foundation (FUNED) Instituto Octávio Magalhães / Fundação Ezequiel Dias (IOM/Funed) Talita Adelino, Joilson Xavier, Marta Giovanetti, Vagner Fonseca, Marcos Vinícius Silva, Luiz Carlos Junior Alcantara, Marluce Aparecida Assunção Oliveira
EPI_ISL_429695 hCoV-19/Brazil/CV42/2020 South America / Brazil / Minas gerais 2020-03-20 Central Public Health Laboratory/Octávio Magalhães Institute (IOM) from the Ezequiel Dias Foundation (FUNED) Instituto Octávio Magalhães / Fundação Ezequiel Dias (IOM/Funed) Talita Adelino, Joilson Xavier, Marta Giovanetti, Vagner Fonseca, Marcos Vinícius Silva, Luiz Carlos Junior Alcantara, Marluce Aparecida Assunção Oliveira
EPI_ISL_430333 hCoV-19/USA/NY-NYUMC306/2020 North America / USA / New York / Brooklyn 2020-03-30 NYU Langone Health Departments of Pathology and Medicine, New York University School of Medicine Maria Aguero-Rosenfeld, Brendan Belovarac, Margaret Black, Ludovic Boytard, John Cadley, Paolo Cotzia, John Chen, Dacia Dimartino, Xiaojun Feng, Tatyana Gindin, Emily Guzman, Adriana Heguy, Megan Hogan, Emily Huang, George Jour, Lawrence H. Lin, Raven Luther, Andrew Lytle, Christian Marier, Matthew T. Maurano, Mark J. Mulligan, Peter Meyn, Raquel Ordonez Ciriza, Iman Osman, Jared Pinnell, Vanessa Raabe, Sitharam Ramaswami, Amy Rapkiewicz, Andre M. Ribeiro-dos-Santos, Marie Samanovic-Golden, Antonio Serrano, Guomiao Shen, Matija Snuderl, Theodore Vougiouklakis, Nick Vulpescu, Gael Westby, Paul Zappile, Yutong Zhang
EPI_ISL_430334 hCoV-19/USA/NY-NYUMC307/2020 North America / USA / New York / Brooklyn 2020-03-30 NYU Langone Health Departments of Pathology and Medicine, New York University School of Medicine Maria Aguero-Rosenfeld, Brendan Belovarac, Margaret Black, Ludovic Boytard, John Cadley, Paolo Cotzia, John Chen, Dacia Dimartino, Xiaojun Feng, Tatyana Gindin, Emily Guzman, Adriana Heguy, Megan Hogan, Emily Huang, George Jour, Lawrence H. Lin, Raven Luther, Andrew Lytle, Christian Marier, Matthew T. Maurano, Mark J. Mulligan, Peter Meyn, Raquel Ordonez Ciriza, Iman Osman, Jared Pinnell, Vanessa Raabe, Sitharam Ramaswami, Amy Rapkiewicz, Andre M. Ribeiro-dos-Santos, Marie Samanovic-Golden, Antonio Serrano, Guomiao Shen, Matija Snuderl, Theodore Vougiouklakis, Nick Vulpescu, Gael Westby, Paul Zappile, Yutong Zhang
EPI_ISL_430335 hCoV-19/USA/NY-NYUMC308/2020 North America / USA / New York / Brooklyn 2020-03-30 NYU Langone Health Departments of Pathology and Medicine, New York University School of Medicine Maria Aguero-Rosenfeld, Brendan Belovarac, Margaret Black, Ludovic Boytard, John Cadley, Paolo Cotzia, John Chen, Dacia Dimartino, Xiaojun Feng, Tatyana Gindin, Emily Guzman, Adriana Heguy, Megan Hogan, Emily Huang, George Jour, Lawrence H. Lin, Raven Luther, Andrew Lytle, Christian Marier, Matthew T. Maurano, Mark J. Mulligan, Peter Meyn, Raquel Ordonez Ciriza, Iman Osman, Jared Pinnell, Vanessa Raabe, Sitharam Ramaswami, Amy Rapkiewicz, Andre M. Ribeiro-dos-Santos, Marie Samanovic-Golden, Antonio Serrano, Guomiao Shen, Matija Snuderl, Theodore Vougiouklakis, Nick Vulpescu, Gael Westby, Paul Zappile, Yutong Zhang
EPI_ISL_430336 hCoV-19/USA/NY-NYUMC309/2020 North America / USA / New York / Manhattan 2020-03-30 NYU Langone Health Departments of Pathology and Medicine, New York University School of Medicine Maria Aguero-Rosenfeld, Brendan Belovarac, Margaret Black, Ludovic Boytard, John Cadley, Paolo Cotzia, John Chen,

Dacia Dimartino, Xiaojun Feng, Tatyana Gindin, Emily Guzman, Adriana Heguy, Megan Hogan, Emily Huang, George Jour, Lawrence H. Lin, Raven Luther, Andrew Lytle, Christian Marier, Matthew T. Maurano, Mark J. Mulligan, Peter Meyn, Raquel Ordonez Ciriza, Iman Osman, Jared Pinnell, Vanessa Raabe, Sitharam Ramaswami, Amy Rapkiewicz, Andre M. Ribeiro-dos-Santos, Marie Samanovic-Golden, Antonio Serrano, Guomiao Shen, Matija Snuderl, Theodore Vougiouklakis, Nick Vulpescu, Gael Westby, Paul Zappile, Yutong Zhang
EPI_ISL_430337 hCoV-19/USA/NY-NYUMC310/2020 North America / USA / New York / Manhattan 2020-03-30 NYU Langone Health Departments of Pathology and Medicine, New York University School of Medicine Maria Aguero-Rosenfeld, Brendan Belovarac, Margaret Black, Ludovic Boytard, John Cadley, Paolo Cotzia, John Chen, Dacia Dimartino, Xiaojun Feng, Tatyana Gindin, Emily Guzman, Adriana Heguy, Megan Hogan, Emily Huang, George Jour, Lawrence H. Lin, Raven Luther, Andrew Lytle, Christian Marier, Matthew T. Maurano, Mark J. Mulligan, Peter Meyn, Raquel Ordonez Ciriza, Iman Osman, Jared Pinnell, Vanessa Raabe, Sitharam Ramaswami, Amy Rapkiewicz, Andre M. Ribeiro-dos-Santos, Marie Samanovic-Golden, Antonio Serrano, Guomiao Shen, Matija Snuderl, Theodore Vougiouklakis, Nick Vulpescu, Gael Westby, Paul Zappile, Yutong Zhang
EPI_ISL_430338 hCoV-19/USA/NY-NYUMC311/2020 North America / USA / New York / Manhattan 2020-03-30 NYU Langone Health Departments of Pathology and Medicine, New York University School of Medicine Maria Aguero-Rosenfeld, Brendan Belovarac, Margaret Black, Ludovic Boytard, John Cadley, Paolo Cotzia, John Chen, Dacia Dimartino, Xiaojun Feng, Tatyana Gindin, Emily Guzman, Adriana Heguy, Megan Hogan, Emily Huang, George Jour, Lawrence H. Lin, Raven Luther, Andrew Lytle, Christian Marier, Matthew T. Maurano, Mark J. Mulligan, Peter Meyn, Raquel Ordonez Ciriza, Iman Osman, Jared Pinnell, Vanessa Raabe, Sitharam Ramaswami, Amy Rapkiewicz, Andre M. Ribeiro-dos-Santos, Marie Samanovic-Golden, Antonio Serrano, Guomiao Shen, Matija Snuderl, Theodore Vougiouklakis, Nick Vulpescu, Gael Westby, Paul Zappile, Yutong Zhang
EPI_ISL_430339 hCoV-19/USA/NY-NYUMC312/2020 North America / USA / New York / Manhattan 2020-03-30 NYU Langone Health Departments of Pathology and Medicine, New York University School of Medicine Maria Aguero-Rosenfeld, Brendan Belovarac, Margaret Black, Ludovic Boytard, John Cadley, Paolo Cotzia, John Chen, Dacia Dimartino, Xiaojun Feng, Tatyana Gindin, Emily Guzman, Adriana Heguy, Megan Hogan, Emily Huang, George Jour, Lawrence H. Lin, Raven Luther, Andrew Lytle, Christian Marier, Matthew T. Maurano, Mark J. Mulligan, Peter Meyn, Raquel Ordonez Ciriza, Iman Osman, Jared Pinnell, Vanessa Raabe, Sitharam Ramaswami, Amy Rapkiewicz, Andre M. Ribeiro-dos-Santos, Marie Samanovic-Golden, Antonio Serrano, Guomiao Shen, Matija Snuderl, Theodore Vougiouklakis, Nick Vulpescu, Gael Westby, Paul Zappile, Yutong Zhang
EPI_ISL_430343 hCoV-19/USA/NY-NYUMC316/2020 North America / USA / New York / Manhattan 2020-03-30 NYU Langone Health Departments of Pathology and Medicine, New York University School of Medicine Maria Aguero-Rosenfeld, Brendan Belovarac, Margaret Black, Ludovic Boytard, John Cadley, Paolo Cotzia, John Chen, Dacia Dimartino, Xiaojun Feng, Tatyana Gindin, Emily Guzman, Adriana Heguy, Megan Hogan, Emily Huang, George Jour, Lawrence H. Lin, Raven Luther, Andrew Lytle, Christian Marier, Matthew T. Maurano, Mark J. Mulligan, Peter Meyn, Raquel Ordonez Ciriza, Iman Osman, Jared Pinnell, Vanessa Raabe, Sitharam Ramaswami, Amy Rapkiewicz, Andre M. Ribeiro-dos-Santos, Marie Samanovic-Golden, Antonio Serrano, Guomiao Shen, Matija Snuderl, Theodore Vougiouklakis, Nick Vulpescu, Gael Westby, Paul Zappile, Yutong Zhang
EPI_ISL_430355 hCoV-19/USA/NY-NYUMC328/2020 North America / USA / New York / Nassau County 2020-04-02 NYU Langone Health Departments of Pathology and Medicine, New York University School of Medicine Maria Aguero-Rosenfeld, Brendan Belovarac, Margaret Black, Ludovic Boytard, John Cadley, Paolo Cotzia, John Chen, Dacia Dimartino, Xiaojun Feng, Tatyana Gindin, Emily Guzman, Adriana Heguy, Megan Hogan, Emily Huang, George Jour, Lawrence H. Lin, Raven Luther, Andrew Lytle, Christian Marier, Matthew T. Maurano, Mark J. Mulligan, Peter Meyn, Raquel Ordonez Ciriza, Iman Osman, Jared Pinnell, Vanessa Raabe, Sitharam Ramaswami, Amy Rapkiewicz, Andre M. Ribeiro-dos-Santos, Marie Samanovic-Golden, Antonio Serrano, Guomiao Shen, Matija Snuderl, Theodore Vougiouklakis, Nick Vulpescu, Gael Westby, Paul Zappile, Yutong Zhang
EPI_ISL_430356 hCoV-19/USA/NJ-NYUMC329/2020 North America / USA / New Jersey / Bergen County 2020-04-02 NYU Langone Health Departments of Pathology and Medicine, New York University School of Medicine Maria Aguero-Rosenfeld, Brendan Belovarac, Margaret Black, Ludovic Boytard, John Cadley, Paolo Cotzia, John Chen, Dacia Dimartino, Xiaojun Feng, Tatyana Gindin, Emily Guzman, Adriana Heguy, Megan Hogan, Emily Huang, George Jour, Lawrence H. Lin, Raven Luther, Andrew Lytle, Christian Marier, Matthew T. Maurano, Mark J. Mulligan, Peter Meyn, Raquel Ordonez Ciriza, Iman Osman, Jared Pinnell, Vanessa Raabe, Sitharam Ramaswami, Amy Rapkiewicz, Andre M. Ribeiro-dos-Santos, Marie Samanovic-Golden, Antonio Serrano, Guomiao Shen, Matija Snuderl, Theodore Vougiouklakis, Nick Vulpescu, Gael Westby, Paul Zappile, Yutong Zhang
EPI_ISL_430357 hCoV-19/USA/NY-NYUMC330/2020 North America / USA / New York / Brooklyn 2020-04-03 NYU Langone Health Departments of Pathology and Medicine, New York University School of Medicine Maria Aguero-Rosenfeld, Brendan Belovarac, Margaret Black, Ludovic Boytard, John Cadley, Paolo Cotzia, John Chen, Dacia Dimartino, Xiaojun Feng, Tatyana Gindin, Emily Guzman, Adriana Heguy, Megan Hogan, Emily Huang, George Jour, Lawrence H. Lin, Raven Luther, Andrew Lytle, Christian Marier, Matthew T. Maurano, Mark J. Mulligan, Peter Meyn, Raquel Ordonez Ciriza, Iman Osman, Jared Pinnell, Vanessa Raabe, Sitharam Ramaswami, Amy Rapkiewicz, Andre M. Ribeiro-dos-Santos, Marie Samanovic-Golden, Antonio Serrano, Guomiao Shen, Matija Snuderl, Theodore Vougiouklakis, Nick Vulpescu, Gael Westby, Paul Zappile, Yutong Zhang
EPI_ISL_430358 hCoV-19/USA/NY-NYUMC331/2020 North America / USA / New York / Manhattan 2020-04-13 NYU Langone Health Departments of Pathology and Medicine, New York University School of Medicine Maria Aguero-Rosenfeld, Brendan Belovarac, Margaret Black, Ludovic Boytard, John Cadley, Paolo Cotzia, John Chen, Dacia Dimartino, Xiaojun Feng, Tatyana Gindin, Emily Guzman, Adriana Heguy, Megan Hogan, Emily Huang, George Jour, Lawrence H. Lin, Raven Luther, Andrew Lytle, Christian Marier, Matthew T. Maurano, Mark J. Mulligan, Peter Meyn, Raquel Ordonez Ciriza, Iman Osman, Jared Pinnell, Vanessa Raabe, Sitharam Ramaswami, Amy Rapkiewicz, Andre M. Ribeiro-dos-Santos, Marie Samanovic-Golden, Antonio Serrano, Guomiao Shen, Matija Snuderl, Theodore Vougiouklakis, Nick Vulpescu, Gael Westby, Paul Zappile, Yutong Zhang
EPI_ISL_430359 hCoV-19/USA/NY-NYUMC332/2020 North America / USA / New York / Brooklyn 2020-04-08

NYU Langone Health	Departments of Pathology and Medicine, New York University School of Medicine	Maria Aguero-Rosenfeld, Brendan Belovarac, Margaret Black, Ludovic Boytard, John Cadley, Paolo Cotzia, John Chen, Dacia Dimartino, Xiaojun Feng, Tatyana Gindin, Emily Guzman, Adriana Heguy, Megan Hogan, Emily Huang, George Jour, Lawrence H. Lin, Raven Luther, Andrew Lytle, Christian Marier, Matthew T. Maurano, Mark J. Mulligan, Peter Meyn, Raquel Ordonez Ciriza, Iman Osman, Jared Pinnell, Vanessa Raabe, Sitharam Ramaswami, Amy Rapkiewicz, Andre M. Ribeiro-dos-Santos, Marie Samanovic-Golden, Antonio Serrano, Guomiao Shen, Matija Snuderl, Theodore Vougiouklakis, Nick Vulpescu, Gael Westby, Paul Zappile, Yutong Zhang
EPI_ISL_430360	hCoV-19/USA/NY-NYUMC333/2020	North America / USA / New York / Queens	2020-04-07	NYU Langone Health	Departments of Pathology and Medicine, New York University School of Medicine	Maria Aguero-Rosenfeld, Brendan Belovarac, Margaret Black, Ludovic Boytard, John Cadley, Paolo Cotzia, John Chen, Dacia Dimartino, Xiaojun Feng, Tatyana Gindin, Emily Guzman, Adriana Heguy, Megan Hogan, Emily Huang, George Jour, Lawrence H. Lin, Raven Luther, Andrew Lytle, Christian Marier, Matthew T. Maurano, Mark J. Mulligan, Peter Meyn, Raquel Ordonez Ciriza, Iman Osman, Jared Pinnell, Vanessa Raabe, Sitharam Ramaswami, Amy Rapkiewicz, Andre M. Ribeiro-dos-Santos, Marie Samanovic-Golden, Antonio Serrano, Guomiao Shen, Matija Snuderl, Theodore Vougiouklakis, Nick Vulpescu, Gael Westby, Paul Zappile, Yutong Zhang
EPI_ISL_430361	hCoV-19/USA/NY-NYUMC334/2020	North America / USA / New York / Brooklyn	2020-04-07	NYU Langone Health	Departments of Pathology and Medicine, New York University School of Medicine	Maria Aguero-Rosenfeld, Brendan Belovarac, Margaret Black, Ludovic Boytard, John Cadley, Paolo Cotzia, John Chen, Dacia Dimartino, Xiaojun Feng, Tatyana Gindin, Emily Guzman, Adriana Heguy, Megan Hogan, Emily Huang, George Jour, Lawrence H. Lin, Raven Luther, Andrew Lytle, Christian Marier, Matthew T. Maurano, Mark J. Mulligan, Peter Meyn, Raquel Ordonez Ciriza, Iman Osman, Jared Pinnell, Vanessa Raabe, Sitharam Ramaswami, Amy Rapkiewicz, Andre M. Ribeiro-dos-Santos, Marie Samanovic-Golden, Antonio Serrano, Guomiao Shen, Matija Snuderl, Theodore Vougiouklakis, Nick Vulpescu, Gael Westby, Paul Zappile, Yutong Zhang
EPI_ISL_430362	hCoV-19/USA/NY-NYUMC335/2020	North America / USA / New York / Brooklyn	2020-04-08	NYU Langone Health	Departments of Pathology and Medicine, New York University School of Medicine	Maria Aguero-Rosenfeld, Brendan Belovarac, Margaret Black, Ludovic Boytard, John Cadley, Paolo Cotzia, John Chen, Dacia Dimartino, Xiaojun Feng, Tatyana Gindin, Emily Guzman, Adriana Heguy, Megan Hogan, Emily Huang, George Jour, Lawrence H. Lin, Raven Luther, Andrew Lytle, Christian Marier, Matthew T. Maurano, Mark J. Mulligan, Peter Meyn, Raquel Ordonez Ciriza, Iman Osman, Jared Pinnell, Vanessa Raabe, Sitharam Ramaswami, Amy Rapkiewicz, Andre M. Ribeiro-dos-Santos, Marie Samanovic-Golden, Antonio Serrano, Guomiao Shen, Matija Snuderl, Theodore Vougiouklakis, Nick Vulpescu, Gael Westby, Paul Zappile, Yutong Zhang
EPI_ISL_430363	hCoV-19/USA/NY-NYUMC336/2020	North America / USA / New York / Brooklyn	2020-04-08	NYU Langone Health	Departments of Pathology and Medicine, New York University School of Medicine	Maria Aguero-Rosenfeld, Brendan Belovarac, Margaret Black, Ludovic Boytard, John Cadley, Paolo Cotzia, John Chen, Dacia Dimartino, Xiaojun Feng, Tatyana Gindin, Emily Guzman, Adriana Heguy, Megan Hogan, Emily Huang, George Jour, Lawrence H. Lin, Raven Luther, Andrew Lytle, Christian Marier, Matthew T. Maurano, Mark J. Mulligan, Peter Meyn, Raquel Ordonez Ciriza, Iman Osman, Jared Pinnell, Vanessa Raabe, Sitharam Ramaswami, Amy Rapkiewicz, Andre M. Ribeiro-dos-Santos, Marie Samanovic-Golden, Antonio Serrano, Guomiao Shen, Matija Snuderl, Theodore Vougiouklakis, Nick Vulpescu, Gael Westby, Paul Zappile, Yutong Zhang
EPI_ISL_430364	hCoV-19/USA/NY-NYUMC337/2020	North America / USA / New York / Brooklyn	2020-04-08	NYU Langone Health	Departments of Pathology and Medicine, New York University School of Medicine	Maria Aguero-Rosenfeld, Brendan Belovarac, Margaret Black, Ludovic Boytard, John Cadley, Paolo Cotzia, John Chen, Dacia Dimartino, Xiaojun Feng, Tatyana Gindin, Emily Guzman, Adriana Heguy, Megan Hogan, Emily Huang, George Jour, Lawrence H. Lin, Raven Luther, Andrew Lytle, Christian Marier, Matthew T. Maurano, Mark J. Mulligan, Peter Meyn, Raquel Ordonez Ciriza, Iman Osman, Jared Pinnell, Vanessa Raabe, Sitharam Ramaswami, Amy Rapkiewicz, Andre M. Ribeiro-dos-Santos, Marie Samanovic-Golden, Antonio Serrano, Guomiao Shen, Matija Snuderl, Theodore Vougiouklakis, Nick Vulpescu, Gael Westby, Paul Zappile, Yutong Zhang
EPI_ISL_430365	hCoV-19/USA/NY-NYUMC338/2020	North America / USA / New York / Brooklyn	2020-04-08	NYU Langone Health	Departments of Pathology and Medicine, New York University School of Medicine	Maria Aguero-Rosenfeld, Brendan Belovarac, Margaret Black, Ludovic Boytard, John Cadley, Paolo Cotzia, John Chen, Dacia Dimartino, Xiaojun Feng, Tatyana Gindin, Emily Guzman, Adriana Heguy, Megan Hogan, Emily Huang, George Jour, Lawrence H. Lin, Raven Luther, Andrew Lytle, Christian Marier, Matthew T. Maurano, Mark J. Mulligan, Peter Meyn, Raquel Ordonez Ciriza, Iman Osman, Jared Pinnell, Vanessa Raabe, Sitharam Ramaswami, Amy Rapkiewicz, Andre M. Ribeiro-dos-Santos, Marie Samanovic-Golden, Antonio Serrano, Guomiao Shen, Matija Snuderl, Theodore Vougiouklakis, Nick Vulpescu, Gael Westby, Paul Zappile, Yutong Zhang
EPI_ISL_430366	hCoV-19/USA/NY-NYUMC339/2020	North America / USA / New York / Brooklyn	2020-04-08	NYU Langone Health	Departments of Pathology and Medicine, New York University School of Medicine	Maria Aguero-Rosenfeld, Brendan Belovarac, Margaret Black, Ludovic Boytard, John Cadley, Paolo Cotzia, John Chen, Dacia Dimartino, Xiaojun Feng, Tatyana Gindin, Emily Guzman, Adriana Heguy, Megan Hogan, Emily Huang, George Jour, Lawrence H. Lin, Raven Luther, Andrew Lytle, Christian Marier, Matthew T. Maurano, Mark J. Mulligan, Peter Meyn, Raquel Ordonez Ciriza, Iman Osman, Jared Pinnell, Vanessa Raabe, Sitharam Ramaswami, Amy Rapkiewicz, Andre M. Ribeiro-dos-Santos, Marie Samanovic-Golden, Antonio Serrano, Guomiao Shen, Matija Snuderl, Theodore Vougiouklakis, Nick Vulpescu, Gael Westby, Paul Zappile, Yutong Zhang
EPI_ISL_430367	hCoV-19/USA/NY-NYUMC340/2020	North America / USA / New York / Brooklyn	2020-04-07	NYU Langone Health	Departments of Pathology and Medicine, New York University School of Medicine	Maria Aguero-Rosenfeld, Brendan Belovarac, Margaret Black, Ludovic Boytard, John Cadley, Paolo Cotzia, John Chen, Dacia Dimartino, Xiaojun Feng, Tatyana Gindin, Emily Guzman, Adriana Heguy, Megan Hogan, Emily Huang, George Jour, Lawrence H. Lin, Raven Luther, Andrew Lytle, Christian Marier, Matthew T. Maurano, Mark J. Mulligan, Peter Meyn, Raquel Ordonez Ciriza, Iman Osman, Jared Pinnell, Vanessa Raabe, Sitharam Ramaswami, Amy Rapkiewicz, Andre M. Ribeiro-dos-Santos, Marie Samanovic-Golden, Antonio Serrano, Guomiao Shen, Matija Snuderl,

Theodore Vougiouklakis, Nick Vulpescu, Gael Westby, Paul Zappile, Yutong Zhang
EPI_ISL_430368 hCoV-19/USA/NY-NYUMC341/2020 North America / USA / New York / Brooklyn 2020-04-08 NYU Langone Health Departments of Pathology and Medicine, New York University School of Medicine Maria Aguero-Rosenfeld, Brendan Belovarac, Margaret Black, Ludovic Boytard, John Cadley, Paolo Cotzia, John Chen, Dacia Dimartino, Xiaojun Feng, Tatyana Gindin, Emily Guzman, Adriana Heguy, Megan Hogan, Emily Huang, George Jour, Lawrence H. Lin, Raven Luther, Andrew Lytle, Christian Marier, Matthew T. Maurano, Mark J. Mulligan, Peter Meyn, Raquel Ordonez Ciriza, Iman Osman, Jared Pinnell, Vanessa Raabe, Sitharam Ramaswami, Amy Rapkiewicz, Andre M. Ribeiro-dos-Santos, Marie Samanovic-Golden, Antonio Serrano, Guomiao Shen, Matija Snuderl, Theodore Vougiouklakis, Nick Vulpescu, Gael Westby, Paul Zappile, Yutong Zhang
EPI_ISL_430369 hCoV-19/USA/NY-NYUMC342/2020 North America / USA / New York / Manhattan 2020-04-08 NYU Langone Health Departments of Pathology and Medicine, New York University School of Medicine Maria Aguero-Rosenfeld, Brendan Belovarac, Margaret Black, Ludovic Boytard, John Cadley, Paolo Cotzia, John Chen, Dacia Dimartino, Xiaojun Feng, Tatyana Gindin, Emily Guzman, Adriana Heguy, Megan Hogan, Emily Huang, George Jour, Lawrence H. Lin, Raven Luther, Andrew Lytle, Christian Marier, Matthew T. Maurano, Mark J. Mulligan, Peter Meyn, Raquel Ordonez Ciriza, Iman Osman, Jared Pinnell, Vanessa Raabe, Sitharam Ramaswami, Amy Rapkiewicz, Andre M. Ribeiro-dos-Santos, Marie Samanovic-Golden, Antonio Serrano, Guomiao Shen, Matija Snuderl, Theodore Vougiouklakis, Nick Vulpescu, Gael Westby, Paul Zappile, Yutong Zhang
EPI_ISL_430370 hCoV-19/USA/NY-NYUMC343/2020 North America / USA / New York / Queens 2020-04-08 NYU Langone Health Departments of Pathology and Medicine, New York University School of Medicine Maria Aguero-Rosenfeld, Brendan Belovarac, Margaret Black, Ludovic Boytard, John Cadley, Paolo Cotzia, John Chen, Dacia Dimartino, Xiaojun Feng, Tatyana Gindin, Emily Guzman, Adriana Heguy, Megan Hogan, Emily Huang, George Jour, Lawrence H. Lin, Raven Luther, Andrew Lytle, Christian Marier, Matthew T. Maurano, Mark J. Mulligan, Peter Meyn, Raquel Ordonez Ciriza, Iman Osman, Jared Pinnell, Vanessa Raabe, Sitharam Ramaswami, Amy Rapkiewicz, Andre M. Ribeiro-dos-Santos, Marie Samanovic-Golden, Antonio Serrano, Guomiao Shen, Matija Snuderl, Theodore Vougiouklakis, Nick Vulpescu, Gael Westby, Paul Zappile, Yutong Zhang
EPI_ISL_430371 hCoV-19/USA/NJ-NYUMC344/2020 North America / USA / New Jersey / Hudson County 2020-04-08 NYU Langone Health Departments of Pathology and Medicine, New York University School of Medicine Maria Aguero-Rosenfeld, Brendan Belovarac, Margaret Black, Ludovic Boytard, John Cadley, Paolo Cotzia, John Chen, Dacia Dimartino, Xiaojun Feng, Tatyana Gindin, Emily Guzman, Adriana Heguy, Megan Hogan, Emily Huang, George Jour, Lawrence H. Lin, Raven Luther, Andrew Lytle, Christian Marier, Matthew T. Maurano, Mark J. Mulligan, Peter Meyn, Raquel Ordonez Ciriza, Iman Osman, Jared Pinnell, Vanessa Raabe, Sitharam Ramaswami, Amy Rapkiewicz, Andre M. Ribeiro-dos-Santos, Marie Samanovic-Golden, Antonio Serrano, Guomiao Shen, Matija Snuderl, Theodore Vougiouklakis, Nick Vulpescu, Gael Westby, Paul Zappile, Yutong Zhang
EPI_ISL_430372 hCoV-19/USA/NY-NYUMC345/2020 North America / USA / New York / Brooklyn 2020-04-07 NYU Langone Health Departments of Pathology and Medicine, New York University School of Medicine Maria Aguero-Rosenfeld, Brendan Belovarac, Margaret Black, Ludovic Boytard, John Cadley, Paolo Cotzia, John Chen, Dacia Dimartino, Xiaojun Feng, Tatyana Gindin, Emily Guzman, Adriana Heguy, Megan Hogan, Emily Huang, George Jour, Lawrence H. Lin, Raven Luther, Andrew Lytle, Christian Marier, Matthew T. Maurano, Mark J. Mulligan, Peter Meyn, Raquel Ordonez Ciriza, Iman Osman, Jared Pinnell, Vanessa Raabe, Sitharam Ramaswami, Amy Rapkiewicz, Andre M. Ribeiro-dos-Santos, Marie Samanovic-Golden, Antonio Serrano, Guomiao Shen, Matija Snuderl, Theodore Vougiouklakis, Nick Vulpescu, Gael Westby, Paul Zappile, Yutong Zhang
EPI_ISL_430373 hCoV-19/USA/NY-NYUMC346/2020 North America / USA / New York / Brooklyn 2020-04-08 NYU Langone Health Departments of Pathology and Medicine, New York University School of Medicine Maria Aguero-Rosenfeld, Brendan Belovarac, Margaret Black, Ludovic Boytard, John Cadley, Paolo Cotzia, John Chen, Dacia Dimartino, Xiaojun Feng, Tatyana Gindin, Emily Guzman, Adriana Heguy, Megan Hogan, Emily Huang, George Jour, Lawrence H. Lin, Raven Luther, Andrew Lytle, Christian Marier, Matthew T. Maurano, Mark J. Mulligan, Peter Meyn, Raquel Ordonez Ciriza, Iman Osman, Jared Pinnell, Vanessa Raabe, Sitharam Ramaswami, Amy Rapkiewicz, Andre M. Ribeiro-dos-Santos, Marie Samanovic-Golden, Antonio Serrano, Guomiao Shen, Matija Snuderl, Theodore Vougiouklakis, Nick Vulpescu, Gael Westby, Paul Zappile, Yutong Zhang
EPI_ISL_430374 hCoV-19/USA/NY-NYUMC347/2020 North America / USA / New York / Brooklyn 2020-04-08 NYU Langone Health Departments of Pathology and Medicine, New York University School of Medicine Maria Aguero-Rosenfeld, Brendan Belovarac, Margaret Black, Ludovic Boytard, John Cadley, Paolo Cotzia, John Chen, Dacia Dimartino, Xiaojun Feng, Tatyana Gindin, Emily Guzman, Adriana Heguy, Megan Hogan, Emily Huang, George Jour, Lawrence H. Lin, Raven Luther, Andrew Lytle, Christian Marier, Matthew T. Maurano, Mark J. Mulligan, Peter Meyn, Raquel Ordonez Ciriza, Iman Osman, Jared Pinnell, Vanessa Raabe, Sitharam Ramaswami, Amy Rapkiewicz, Andre M. Ribeiro-dos-Santos, Marie Samanovic-Golden, Antonio Serrano, Guomiao Shen, Matija Snuderl, Theodore Vougiouklakis, Nick Vulpescu, Gael Westby, Paul Zappile, Yutong Zhang
EPI_ISL_430375 hCoV-19/USA/NY-NYUMC348/2020 North America / USA / New York / Brooklyn 2020-04-08 NYU Langone Health Departments of Pathology and Medicine, New York University School of Medicine Maria Aguero-Rosenfeld, Brendan Belovarac, Margaret Black, Ludovic Boytard, John Cadley, Paolo Cotzia, John Chen, Dacia Dimartino, Xiaojun Feng, Tatyana Gindin, Emily Guzman, Adriana Heguy, Megan Hogan, Emily Huang, George Jour, Lawrence H. Lin, Raven Luther, Andrew Lytle, Christian Marier, Matthew T. Maurano, Mark J. Mulligan, Peter Meyn, Raquel Ordonez Ciriza, Iman Osman, Jared Pinnell, Vanessa Raabe, Sitharam Ramaswami, Amy Rapkiewicz, Andre M. Ribeiro-dos-Santos, Marie Samanovic-Golden, Antonio Serrano, Guomiao Shen, Matija Snuderl, Theodore Vougiouklakis, Nick Vulpescu, Gael Westby, Paul Zappile, Yutong Zhang
EPI_ISL_430376 hCoV-19/USA/NY-NYUMC349/2020 North America / USA / New York / Brooklyn 2020-04-08 NYU Langone Health Departments of Pathology and Medicine, New York University School of Medicine Maria Aguero-Rosenfeld, Brendan Belovarac, Margaret Black, Ludovic Boytard, John Cadley, Paolo Cotzia, John Chen, Dacia Dimartino, Xiaojun Feng, Tatyana Gindin, Emily Guzman, Adriana Heguy, Megan Hogan, Emily Huang, George Jour, Lawrence H. Lin, Raven Luther, Andrew Lytle, Christian Marier, Matthew T. Maurano, Mark J. Mulligan,

Peter Meyn, Raquel Ordonez Ciriza, Iman Osman, Jared Pinnell, Vanessa Raabe, Sitharam Ramaswami, Amy Rapkiewicz, Andre M. Ribeiro-dos-Santos, Marie Samanovic-Golden, Antonio Serrano, Guomiao Shen, Matija Snuderl, Theodore Vougiouklakis, Nick Vulpescu, Gael Westby, Paul Zappile, Yutong Zhang
EPI_ISL_430600 hCoV-19/Australia/VIC1134/2020 Oceania / Australia / Victoria 2020-04-08 Victorian Infectious Diseases Reference Laboratory (VIDRL) Microbiological Diagnostic Unit Public Health Laboratory and Victorian Infectious Diseases Reference Laboratory, The Peter Doherty Institute for Infection and Immunity Caly L., Seemann T., Sait, M., Schultz M., Druce J., Sherry, N.
EPI_ISL_430601 hCoV-19/Australia/VIC1135/2020 Oceania / Australia / Victoria 2020-04-08 Victorian Infectious Diseases Reference Laboratory (VIDRL) Microbiological Diagnostic Unit Public Health Laboratory and Victorian Infectious Diseases Reference Laboratory, The Peter Doherty Institute for Infection and Immunity Caly L., Seemann T., Sait, M., Schultz M., Druce J., Sherry, N.
EPI_ISL_430602 hCoV-19/Australia/VIC1158/2020 Oceania / Australia / Victoria 2020-04-09 Victorian Infectious Diseases Reference Laboratory (VIDRL) Microbiological Diagnostic Unit Public Health Laboratory and Victorian Infectious Diseases Reference Laboratory, The Peter Doherty Institute for Infection and Immunity Caly L., Seemann T., Sait, M., Schultz M., Druce J., Sherry, N.
EPI_ISL_430604 hCoV-19/Australia/VIC1159/2020 Oceania / Australia / Victoria 2020-04-09 Victorian Infectious Diseases Reference Laboratory (VIDRL) Microbiological Diagnostic Unit Public Health Laboratory and Victorian Infectious Diseases Reference Laboratory, The Peter Doherty Institute for Infection and Immunity Caly L., Seemann T., Sait, M., Schultz M., Druce J., Sherry, N.
EPI_ISL_430605 hCoV-19/Australia/VIC1160/2020 Oceania / Australia / Victoria 2020-04-09 Victorian Infectious Diseases Reference Laboratory (VIDRL) Microbiological Diagnostic Unit Public Health Laboratory and Victorian Infectious Diseases Reference Laboratory, The Peter Doherty Institute for Infection and Immunity Caly L., Seemann T., Sait, M., Schultz M., Druce J., Sherry, N.
EPI_ISL_430607 hCoV-19/Australia/VIC1137/2020 Oceania / Australia / Victoria 2020-04-08 Victorian Infectious Diseases Reference Laboratory (VIDRL) Microbiological Diagnostic Unit Public Health Laboratory and Victorian Infectious Diseases Reference Laboratory, The Peter Doherty Institute for Infection and Immunity Caly L., Seemann T., Sait, M., Schultz M., Druce J., Sherry, N.
EPI_ISL_430609 hCoV-19/Australia/VIC1138/2020 Oceania / Australia / Victoria 2020-04-08 Victorian Infectious Diseases Reference Laboratory (VIDRL) Microbiological Diagnostic Unit Public Health Laboratory and Victorian Infectious Diseases Reference Laboratory, The Peter Doherty Institute for Infection and Immunity Caly L., Seemann T., Sait, M., Schultz M., Druce J., Sherry, N.
EPI_ISL_430610 hCoV-19/Australia/VIC1139/2020 Oceania / Australia / Victoria 2020-04-08 Victorian Infectious Diseases Reference Laboratory (VIDRL) Microbiological Diagnostic Unit Public Health Laboratory and Victorian Infectious Diseases Reference Laboratory, The Peter Doherty Institute for Infection and Immunity Caly L., Seemann T., Sait, M., Schultz M., Druce J., Sherry, N.
EPI_ISL_430614 hCoV-19/Australia/VIC1143/2020 Oceania / Australia / Victoria 2020-04-08 Victorian Infectious Diseases Reference Laboratory (VIDRL) Microbiological Diagnostic Unit Public Health Laboratory and Victorian Infectious Diseases Reference Laboratory, The Peter Doherty Institute for Infection and Immunity Caly L., Seemann T., Sait, M., Schultz M., Druce J., Sherry, N.
EPI_ISL_430615 hCoV-19/Australia/VIC1161/2020 Oceania / Australia / Victoria 2020-04-09 Victorian Infectious Diseases Reference Laboratory (VIDRL) Microbiological Diagnostic Unit Public Health Laboratory and Victorian Infectious Diseases Reference Laboratory, The Peter Doherty Institute for Infection and Immunity Caly L., Seemann T., Sait, M., Schultz M., Druce J., Sherry, N.
EPI_ISL_430616 hCoV-19/Australia/VIC1162/2020 Oceania / Australia / Victoria 2020-04-09 Victorian Infectious Diseases Reference Laboratory (VIDRL) Microbiological Diagnostic Unit Public Health Laboratory and Victorian Infectious Diseases Reference Laboratory, The Peter Doherty Institute for Infection and Immunity Caly L., Seemann T., Sait, M., Schultz M., Druce J., Sherry, N.
EPI_ISL_430618 hCoV-19/Australia/VIC1109/2020 Oceania / Australia / Victoria 2020-04-07 Victorian Infectious Diseases Reference Laboratory (VIDRL) Microbiological Diagnostic Unit Public Health Laboratory and Victorian Infectious Diseases Reference Laboratory, The Peter Doherty Institute for Infection and Immunity Caly L., Seemann T., Sait, M., Schultz M., Druce J., Sherry, N.
EPI_ISL_430619 hCoV-19/Australia/VIC1144/2020 Oceania / Australia / Victoria 2020-04-08 Victorian Infectious Diseases Reference Laboratory (VIDRL) Microbiological Diagnostic Unit Public Health Laboratory and Victorian Infectious Diseases Reference Laboratory, The Peter Doherty Institute for Infection and Immunity Caly L., Seemann T., Sait, M., Schultz M., Druce J., Sherry, N.
EPI_ISL_430623 hCoV-19/Australia/VIC1174/2020 Oceania / Australia / Victoria 2020-04-10 Victorian Infectious Diseases Reference Laboratory (VIDRL) Microbiological Diagnostic Unit Public Health Laboratory and Victorian Infectious Diseases Reference Laboratory, The Peter Doherty Institute for Infection and Immunity Caly L., Seemann T., Sait, M., Schultz M., Druce J., Sherry, N.
EPI_ISL_430632 hCoV-19/Australia/NT29/2020 Oceania / Australia / Northern Territory 2020 Royal Darwin Hospital Pathology Microbiological Diagnostic Unit Public Health Laboratory and Victorian Infectious Diseases Reference Laboratory, The Peter Doherty Institute for Infection and Immunity Meumann, E., Caly L., Seemann T., Sait, M., Schultz M., Druce J., Sherry, N.
EPI_ISL_430633 hCoV-19/Australia/NT30/2020 Oceania / Australia / Northern Territory 2020 Royal Darwin Hospital Pathology Microbiological Diagnostic Unit Public Health Laboratory and Victorian Infectious Diseases Reference Laboratory, The Peter Doherty Institute for Infection and Immunity Meumann, E., Caly L., Seemann T., Sait, M., Schultz M., Druce J., Sherry, N.
EPI_ISL_430635 hCoV-19/Australia/NT31/2020 Oceania / Australia / Northern Territory 2020 Royal Darwin Hospital Pathology Microbiological Diagnostic Unit Public Health Laboratory and Victorian Infectious Diseases Reference Laboratory, The Peter Doherty Institute for Infection and Immunity

Meumann, E., Caly L., Seemann T., Sait, M., Schultz M., Druce J., Sherry, N.
EPI_ISL_430638 hCoV-19/Australia/VIC1355/2020 Oceania / Australia / Victoria 2020-04-09 Victorian Infectious Diseases Reference Laboratory (VIDRL) Microbiological Diagnostic Unit Public Health Laboratory and Victorian Infectious Diseases Reference Laboratory, The Peter Doherty Institute for Infection and Immunity Caly L., Seemann T., Sait, M., Schultz M., Druce J., Sherry, N.
EPI_ISL_430639 hCoV-19/Australia/VIC942/2020 Oceania / Australia / Victoria 2020-03-26 Microbiological Diagnostic Unit Public Health Laboratory Microbiological Diagnostic Unit Public Health Laboratory Seemann T., Schultz M., Sait, M., Sherry, N.
EPI_ISL_430640 hCoV-19/Australia/VIC946/2020 Oceania / Australia / Victoria 2020-03-26 Microbiological Diagnostic Unit Public Health Laboratory Microbiological Diagnostic Unit Public Health Laboratory Seemann T., Schultz M., Sait, M., Sherry, N.
EPI_ISL_430645 hCoV-19/Australia/VIC953/2020 Oceania / Australia / Victoria 2020-03-26 Microbiological Diagnostic Unit Public Health Laboratory Microbiological Diagnostic Unit Public Health Laboratory Seemann T., Schultz M., Sait, M., Sherry, N.
EPI_ISL_430646 hCoV-19/Australia/VIC951/2020 Oceania / Australia / Victoria 2020-03-26 Microbiological Diagnostic Unit Public Health Laboratory Microbiological Diagnostic Unit Public Health Laboratory Seemann T., Schultz M., Sait, M., Sherry, N.
EPI_ISL_430647 hCoV-19/Australia/VIC948/2020 Oceania / Australia / Victoria 2020-03-26 Microbiological Diagnostic Unit Public Health Laboratory Microbiological Diagnostic Unit Public Health Laboratory Seemann T., Schultz M., Sait, M., Sherry, N.
EPI_ISL_430649 hCoV-19/Australia/VIC957/2020 Oceania / Australia / Victoria 2020-03-27 Microbiological Diagnostic Unit Public Health Laboratory Microbiological Diagnostic Unit Public Health Laboratory Seemann T., Schultz M., Sait, M., Sherry, N.
EPI_ISL_430653 hCoV-19/Australia/VIC966/2020 Oceania / Australia / Victoria 2020-03-27 Microbiological Diagnostic Unit Public Health Laboratory Microbiological Diagnostic Unit Public Health Laboratory Seemann T., Schultz M., Sait, M., Sherry, N.
EPI_ISL_430654 hCoV-19/Australia/VIC961/2020 Oceania / Australia / Victoria 2020-03-27 Microbiological Diagnostic Unit Public Health Laboratory Microbiological Diagnostic Unit Public Health Laboratory Seemann T., Schultz M., Sait, M., Sherry, N.
EPI_ISL_430655 hCoV-19/Australia/VIC964/2020 Oceania / Australia / Victoria 2020-03-27 Microbiological Diagnostic Unit Public Health Laboratory Microbiological Diagnostic Unit Public Health Laboratory Seemann T., Schultz M., Sait, M., Sherry, N.
EPI_ISL_430658 hCoV-19/Australia/VIC975/2020 Oceania / Australia / Victoria 2020-03-28 Microbiological Diagnostic Unit Public Health Laboratory Microbiological Diagnostic Unit Public Health Laboratory Seemann T., Schultz M., Sait, M., Sherry, N.
EPI_ISL_430659 hCoV-19/Australia/VIC974/2020 Oceania / Australia / Victoria 2020-03-28 Microbiological Diagnostic Unit Public Health Laboratory Microbiological Diagnostic Unit Public Health Laboratory Seemann T., Schultz M., Sait, M., Sherry, N.
EPI_ISL_430661 hCoV-19/Australia/VIC971/2020 Oceania / Australia / Victoria 2020-03-27 Microbiological Diagnostic Unit Public Health Laboratory Microbiological Diagnostic Unit Public Health Laboratory Seemann T., Schultz M., Sait, M., Sherry, N.
EPI_ISL_430663 hCoV-19/Australia/VIC959/2020 Oceania / Australia / Victoria 2020-03-27 Microbiological Diagnostic Unit Public Health Laboratory Microbiological Diagnostic Unit Public Health Laboratory Seemann T., Schultz M., Sait, M., Sherry, N.
EPI_ISL_430669 hCoV-19/Australia/VIC1056/2020 Oceania / Australia / Victoria 2020-04-03 Microbiological Diagnostic Unit Public Health Laboratory Microbiological Diagnostic Unit Public Health Laboratory Seemann T., Schultz M., Sait, M., Sherry, N.
EPI_ISL_430670 hCoV-19/Australia/VIC1067/2020 Oceania / Australia / Victoria 2020-04-04 Microbiological Diagnostic Unit Public Health Laboratory Microbiological Diagnostic Unit Public Health Laboratory Seemann T., Schultz M., Sait, M., Sherry, N.
EPI_ISL_430674 hCoV-19/Australia/VIC1178/2020 Oceania / Australia / Victoria 2020-04-10 Microbiological Diagnostic Unit Public Health Laboratory Microbiological Diagnostic Unit Public Health Laboratory Seemann T., Schultz M., Sait, M., Sherry, N.
EPI_ISL_430676 hCoV-19/Australia/VIC1281/2020 Oceania / Australia / Victoria 2020-04-08 Microbiological Diagnostic Unit Public Health Laboratory Microbiological Diagnostic Unit Public Health Laboratory Seemann T., Schultz M., Sait, M., Sherry, N.
EPI_ISL_430677 hCoV-19/Australia/VIC1300/2020 Oceania / Australia / Victoria 2020-04-10 Microbiological Diagnostic Unit Public Health Laboratory Microbiological Diagnostic Unit Public Health Laboratory Seemann T., Schultz M., Sait, M., Sherry, N.
EPI_ISL_430678 hCoV-19/Australia/VIC1279/2020 Oceania / Australia / Victoria 2020-04-07 Microbiological Diagnostic Unit Public Health Laboratory Microbiological Diagnostic Unit Public Health Laboratory Seemann T., Schultz M., Sait, M., Sherry, N.
EPI_ISL_430679 hCoV-19/Australia/VIC1301/2020 Oceania / Australia / Victoria 2020-04-10 Microbiological Diagnostic Unit Public Health Laboratory Microbiological Diagnostic Unit Public Health Laboratory Seemann T., Schultz M., Sait, M., Sherry, N.
EPI_ISL_430680 hCoV-19/Australia/VIC1302/2020 Oceania / Australia / Victoria 2020-04-10 Microbiological Diagnostic Unit Public Health Laboratory Microbiological Diagnostic Unit Public Health Laboratory Seemann T., Schultz M., Sait, M., Sherry, N.
EPI_ISL_430681 hCoV-19/Australia/VIC1314/2020 Oceania / Australia / Victoria 2020-04-11 Microbiological Diagnostic Unit Public Health Laboratory Microbiological Diagnostic Unit Public Health Laboratory

Seemann T., Schultz M., Sait, M., Sherry, N.
EPI_ISL_430682 hCoV-19/Australia/VIC1334/2020 Oceania / Australia / Victoria 2020-04-13 Microbiological Diagnostic Unit Public Health Laboratory Microbiological Diagnostic Unit Public Health Laboratory Seemann T., Schultz M., Sait, M., Sherry, N.
EPI_ISL_430683 hCoV-19/Australia/VIC1342/2020 Oceania / Australia / Victoria 2020-04-14 Microbiological Diagnostic Unit Public Health Laboratory Microbiological Diagnostic Unit Public Health Laboratory Seemann T., Schultz M., Sait, M., Sherry, N.
EPI_ISL_430684 hCoV-19/Australia/VIC1343/2020 Oceania / Australia / Victoria 2020-04-14 Microbiological Diagnostic Unit Public Health Laboratory Microbiological Diagnostic Unit Public Health Laboratory Seemann T., Schultz M., Sait, M., Sherry, N.
EPI_ISL_430686 hCoV-19/Australia/VIC1340/2020 Oceania / Australia / Victoria 2020-04-13 Microbiological Diagnostic Unit Public Health Laboratory Microbiological Diagnostic Unit Public Health Laboratory Seemann T., Schultz M., Sait, M., Sherry, N.
EPI_ISL_430689 hCoV-19/Australia/VIC1315/2020 Oceania / Australia / Victoria 2020-04-11 Victorian Infectious Diseases Reference Laboratory (VIDRL) Microbiological Diagnostic Unit Public Health Laboratory and Victorian Infectious Diseases Reference Laboratory, The Peter Doherty Institute for Infection and Immunity Caly L., Seemann T., Sait, M., Schultz M., Druce J., Sherry, N.
EPI_ISL_430690 hCoV-19/Australia/VIC1316/2020 Oceania / Australia / Victoria 2020-04-11 Victorian Infectious Diseases Reference Laboratory (VIDRL) Microbiological Diagnostic Unit Public Health Laboratory and Victorian Infectious Diseases Reference Laboratory, The Peter Doherty Institute for Infection and Immunity Caly L., Seemann T., Sait, M., Schultz M., Druce J., Sherry, N.
EPI_ISL_430691 hCoV-19/Australia/VIC1317/2020 Oceania / Australia / Victoria 2020-04-11 Victorian Infectious Diseases Reference Laboratory (VIDRL) Microbiological Diagnostic Unit Public Health Laboratory and Victorian Infectious Diseases Reference Laboratory, The Peter Doherty Institute for Infection and Immunity Caly L., Seemann T., Sait, M., Schultz M., Druce J., Sherry, N.
EPI_ISL_430693 hCoV-19/Australia/VIC1318/2020 Oceania / Australia / Victoria 2020-04-11 Victorian Infectious Diseases Reference Laboratory (VIDRL) Microbiological Diagnostic Unit Public Health Laboratory and Victorian Infectious Diseases Reference Laboratory, The Peter Doherty Institute for Infection and Immunity Caly L., Seemann T., Sait, M., Schultz M., Druce J., Sherry, N.
EPI_ISL_430694 hCoV-19/Australia/VIC1303/2020 Oceania / Australia / Victoria 2020-04-10 Victorian Infectious Diseases Reference Laboratory (VIDRL) Microbiological Diagnostic Unit Public Health Laboratory and Victorian Infectious Diseases Reference Laboratory, The Peter Doherty Institute for Infection and Immunity Caly L., Seemann T., Sait, M., Schultz M., Druce J., Sherry, N.
EPI_ISL_430695 hCoV-19/Australia/VIC1328/2020 Oceania / Australia / Victoria 2020-04-12 Victorian Infectious Diseases Reference Laboratory (VIDRL) Microbiological Diagnostic Unit Public Health Laboratory and Victorian Infectious Diseases Reference Laboratory, The Peter Doherty Institute for Infection and Immunity Caly L., Seemann T., Sait, M., Schultz M., Druce J., Sherry, N.
EPI_ISL_430696 hCoV-19/Australia/VIC1329/2020 Oceania / Australia / Victoria 2020-04-12 Victorian Infectious Diseases Reference Laboratory (VIDRL) Microbiological Diagnostic Unit Public Health Laboratory and Victorian Infectious Diseases Reference Laboratory, The Peter Doherty Institute for Infection and Immunity Caly L., Seemann T., Sait, M., Schultz M., Druce J., Sherry, N.
EPI_ISL_430697 hCoV-19/Australia/VIC1344/2020 Oceania / Australia / Victoria 2020-04-14 Victorian Infectious Diseases Reference Laboratory (VIDRL) Microbiological Diagnostic Unit Public Health Laboratory and Victorian Infectious Diseases Reference Laboratory, The Peter Doherty Institute for Infection and Immunity Caly L., Seemann T., Sait, M., Schultz M., Druce J., Sherry, N.
EPI_ISL_430698 hCoV-19/Australia/VIC1336/2020 Oceania / Australia / Victoria 2020-04-13 Victorian Infectious Diseases Reference Laboratory (VIDRL) Microbiological Diagnostic Unit Public Health Laboratory and Victorian Infectious Diseases Reference Laboratory, The Peter Doherty Institute for Infection and Immunity Caly L., Seemann T., Sait, M., Schultz M., Druce J., Sherry, N.
EPI_ISL_406798 hCoV-19/Wuhan/WH01/2019 Asia / China / Hubei / Wuhan 2019-12-26 General Hospital of Central Theater Command of People's Liberation Army of China BGI & Institute of Microbiology, Chinese Academy of Sciences & Shandong First Medical University & Shandong Academy of Medical Sciences & General Hospital of Central Theater Command of People's Liberation Army of China Weijun Chen, Yuhai Bi, Weifeng Shi and Zhenhong Hu
EPI_ISL_407976 hCoV-19/Belgium/GHB-03021/2020 Europe / Belgium / Leuven 2020-02-03 KU Leuven, Clinical and Epidemiological Virology KU Leuven, Clinical and Epidemiological Virology Bert Vanmechelen, Elke Wollants, Annabel Rector, Els Keyaerts, Lies Laenen, Marc Van Ranst, and Piet Maes
EPI_ISL_407987 hCoV-19/Singapore/2/2020 Asia / Singapore 2020-01-25 Singapore General Hospital Programme in Emerging Infectious Diseases, Duke-NUS Medical School Danielle E Anderson, Martin Linster, Yan Zhuang, Jayanthi Jayakumar, Kian Sing Chan, Lynette LE Oon, Jenny GH Low, Yvonne CF Su, Linfa Wang, Gavin JD Smith
EPI_ISL_407988 hCoV-19/Singapore/3/2020 Asia / Singapore 2020-02-01 National Centre for Infectious Diseases Programme in Emerging Infectious Diseases, Duke-NUS Medical School Danielle E Anderson, Martin Linster, Yan Zhuang, Jayanthi Jayakumar, David CB Lye, Yee Sin Leo, Barnaby E Young, Yvonne CF Su, Linfa Wang, Gavin JD Smith
EPI_ISL_412459 hCoV-19/Jingzhou/HBCDC-HB-01/2020 Asia / China / Hubei / Jingzhou 2020-01-08 Jingzhou Center for Disease Control and Prevention Hubei Provincial Center for Disease Control and Prevention Bin Fang, Xiang Li, Xiao Yu, Linlin Liu, Bo Yang, Faxian Zhan, Guojun Ye, Xixiang Huo, Junqiang Xu, Bo Yu, Kun Cai, Jing Li, Maoyi Chen,Jie Hu, Chunlin Mao, Yongzhong Jiang.
EPI_ISL_413729 hCoV-19/China/WF0015/2020 Asia / China 2020-02 Weifang Center for Disease Control and

Prevention	Weifang Center for Disease Control and Prevention & BGI-Shenzhen	Qing Nie, Xingguang Li, Erik M Volz, Han Fu, Haowei Wang, Xiaoyue Xi, Wei Chen, Dehui Liu, Yingying Chen, Mengmeng Tian, Wei Tan, Junjie Zai, Wanying Sun, Jiandong Li, Junhua Li
EPI_ISL_413746	hCoV-19/China/WF0016/2020	Asia / China	2020-02 Weifang Center for Disease Control and Prevention	Weifang Center for Disease Control and Prevention & BGI-Shenzhen	Qing Nie, Xingguang Li, Erik M Volz, Han Fu, Haowei Wang, Xiaoyue Xi, Wei Chen, Dehui Liu, Yingying Chen, Mengmeng Tian, Wei Tan, Junjie Zai, Wanying Sun, Jiandong Li, Junhua Li
EPI_ISL_413748	hCoV-19/China/WF0018/2020	Asia / China	2020-02 Weifang Center for Disease Control and Prevention	Weifang Center for Disease Control and Prevention & BGI-Shenzhen	Qing Nie, Xingguang Li, Erik M Volz, Han Fu, Haowei Wang, Xiaoyue Xi, Wei Chen, Dehui Liu, Yingying Chen, Mengmeng Tian, Wei Tan, Junjie Zai, Wanying Sun, Jiandong Li, Junhua Li
EPI_ISL_413749	hCoV-19/China/WF0019/2020	Asia / China	2020-02 Weifang Center for Disease Control and Prevention	Weifang Center for Disease Control and Prevention & BGI-Shenzhen	Qing Nie, Xingguang Li, Erik M Volz, Han Fu, Haowei Wang, Xiaoyue Xi, Wei Chen, Dehui Liu, Yingying Chen, Mengmeng Tian, Wei Tan, Junjie Zai, Wanying Sun, Jiandong Li, Junhua Li
EPI_ISL_413750	hCoV-19/China/WF0020/2020	Asia / China	2020-02 Weifang Center for Disease Control and Prevention	Weifang Center for Disease Control and Prevention & BGI-Shenzhen	Qing Nie, Xingguang Li, Erik M Volz, Han Fu, Haowei Wang, Xiaoyue Xi, Wei Chen, Dehui Liu, Yingying Chen, Mengmeng Tian, Wei Tan, Junjie Zai, Wanying Sun, Jiandong Li, Junhua Li
EPI_ISL_413751	hCoV-19/China/WF0021/2020	Asia / China	2020-02 Weifang Center for Disease Control and Prevention	Weifang Center for Disease Control and Prevention & BGI-Shenzhen	Qing Nie, Xingguang Li, Erik M Volz, Han Fu, Haowei Wang, Xiaoyue Xi, Wei Chen, Dehui Liu, Yingying Chen, Mengmeng Tian, Wei Tan, Junjie Zai, Wanying Sun, Jiandong Li, Junhua Li
EPI_ISL_413753	hCoV-19/China/WF0024/2020	Asia / China	2020-02 Weifang Center for Disease Control and Prevention	Weifang Center for Disease Control and Prevention & BGI-Shenzhen	Qing Nie, Xingguang Li, Erik M Volz, Han Fu, Haowei Wang, Xiaoyue Xi, Wei Chen, Dehui Liu, Yingying Chen, Mengmeng Tian, Wei Tan, Junjie Zai, Wanying Sun, Jiandong Li, Junhua Li
EPI_ISL_413761	hCoV-19/China/WF0026/2020	Asia / China	2020-02 Weifang Center for Disease Control and Prevention	Weifang Center for Disease Control and Prevention & BGI-Shenzhen	Qing Nie, Xingguang Li, Erik M Volz, Han Fu, Haowei Wang, Xiaoyue Xi, Wei Chen, Dehui Liu, Yingying Chen, Mengmeng Tian, Wei Tan, Junjie Zai, Wanying Sun, Jiandong Li, Junhua Li
EPI_ISL_413791	hCoV-19/China/WF0028/2020	Asia / China	2020-02 Weifang Center for Disease Control and Prevention	Weifang Center for Disease Control and Prevention & BGI-Shenzhen	Qing Nie, Xingguang Li, Erik M Volz, Han Fu, Haowei Wang, Xiaoyue Xi, Wei Chen, Dehui Liu, Yingying Chen, Mengmeng Tian, Wei Tan, Junjie Zai, Wanying Sun, Jiandong Li, Junhua Li
EPI_ISL_413809	hCoV-19/China/WF0029/2020	Asia / China	2020-02 Weifang Center for Disease Control and Prevention	Weifang Center for Disease Control and Prevention & BGI-Shenzhen	Qing Nie, Xingguang Li, Erik M Volz, Han Fu, Haowei Wang, Xiaoyue Xi, Wei Chen, Dehui Liu, Yingying Chen, Mengmeng Tian, Wei Tan, Junjie Zai, Wanying Sun, Jiandong Li, Junhua Li
EPI_ISL_413924	hCoV-19/USA/CA-CDPH-UC6/2020	North America / USA / California / Grand Princess cruise ship	2020-03-05	California Department of Public Health	Chiu Laboratory, University of California, San Francisco	Xianding Deng, Scot Federman, Chao-Yang Pan, Hugo Guevara,Wei Gu, Debra A. Wadford, and Charles Y. Chiu
EPI_ISL_413925	hCoV-19/USA/CA-CDPH-UC7/2020	North America / USA / California / Grand Princess cruise ship	2020-03-05	California Department of Public Health	Chiu Laboratory, University of California, San Francisco	Xianding Deng, Scot Federman, Chao-Yang Pan, Hugo Guevara,Wei Gu, Debra A. Wadford, and Charles Y. Chiu
EPI_ISL_413928	hCoV-19/USA/CA-CDPH-UC9/2020	North America / USA / California / Grand Princess cruise ship	2020-03-05	California Department of Public Health	Chiu Laboratory, University of California, San Francisco	Xianding Deng, Scot Federman, Chao-Yang Pan, Hugo Guevara,Wei Gu, Debra A. Wadford, and Charles Y. Chiu
EPI_ISL_413931	hCoV-19/USA/CA-CDPH-UC11/2020	North America / USA / California / Grand Princess cruise ship	2020-03-05	California Department of Public Health	Chiu Laboratory, University of California, San Francisco	Xianding Deng, Scot Federman, Chao-Yang Pan, Hugo Guevara,Wei Gu, Debra A. Wadford, and Charles Y. Chiu
EPI_ISL_416024	hCoV-19/Wales/PHW35/2020	Europe / United Kingdom / Wales 2020-03-11	Wales Specialist Virology Centre	Public Health Wales Microbiology Cardiff	Catherine Moore, Joanne Watkins, Sally Corden, Tom Connor
EPI_ISL_416026	hCoV-19/Wales/PHW21/2020	Europe / United Kingdom / Wales 2020-03-11	Wales Specialist Virology Centre	Public Health Wales Microbiology Cardiff	Catherine Moore, Joanne Watkins, Sally Corden, Tom Connor
EPI_ISL_416028	hCoV-19/Brazil/SPBR-07/2020	South America / Brazil / Sao Paulo / Sao Paulo	2020-03-03	National Influenza Center - Instituto Adolfo Lutz	Instituto Adolfo Lutz, Interdiciplinary Procedures Center, Strategic Laboratory	Claudio Tavares Sacchi, Claudia Regina Gonçalves, Carlos Henrique Camargo, Fabiana Cristina Pereira dos Santos, Daniela Bernardes Borges da Silva, Simone Guadagnucci Morillo, Adriano Abbud, Adriana Bugno, Maria do Carmo Sampaio Tavares Timenetsky, Terezinha Maria de Paiva
EPI_ISL_416029	hCoV-19/Brazil/SPBR-08/2020	South America / Brazil / Sao Paulo / Sao Paulo	2020-03-04	Laboiratório Fleury	Instituto Adolfo Lutz, Interdiciplinary Procedures Center, Strategic Laboratory Claudio Tavares Sacchi, Claudia Regina Gonçalves, Carlos Henrique Camargo, Fabiana Cristina Pereira dos Santos, Daniela Bernardes Borges da Silva, Simone Guadagnucci Morillo, Adriano Abbud, Adriana Bugno, Maria do Carmo Sampaio

Tavares Timenetsky, Terezinha Maria de Paiva
EPI_ISL_416031 hCoV-19/Brazil/SPBR-09/2020 South America / Brazil / Sao Paulo / Sao Paulo 2020-03-04 National Influenza Center - Instituto Adolfo Lutz Instituto Adolfo Lutz, Interdiciplinary Procedures Center, Strategic Laboratory Claudio Tavares Sacchi, Claudia Regina Gonçalves, Carlos Henrique Camargo, Fabiana Cristina Pereira dos Santos, Daniela Bernardes Borges da Silva, Simone Guadagnucci Morillo, Adriano Abbud, Adriana Bugno, Maria do Carmo Sampaio Tavares Timenetsky, Terezinha Maria de Paiva
EPI_ISL_416032 hCoV-19/Brazil/SPBR-10/2020 South America / Brazil / Distrito Federal / Brasilia 2020-03-04 National Influenza Center - Instituto Adolfo Lutz Instituto Adolfo Lutz, Interdiciplinary Procedures Center, Strategic Laboratory Claudio Tavares Sacchi, Claudia Regina Gonçalves, Carlos Henrique Camargo, Fabiana Cristina Pereira dos Santos, Daniela Bernardes Borges da Silva, Simone Guadagnucci Morillo, Adriano Abbud, Adriana Bugno, Maria do Carmo Sampaio Tavares Timenetsky, Terezinha Maria de Paiva
EPI_ISL_416033 hCoV-19/Brazil/SPBR-11/2020 South America / Brazil / Sao Paulo / Sao Paulo 2020-03-03 Hospital Israelita Albert Einstein Instituto Adolfo Lutz, Interdiciplinary Procedures Center, Strategic Laboratory Claudio Tavares Sacchi, Claudia Regina Gonçalves, Carlos Henrique Camargo, Erica Valessa Ramos Gomes, Fabiana Cristina Pereira dos Santos, Daniela Bernardes Borges da Silva, Simone Guadagnucci Morillo, Adriano Abbud, Adriana Bugno, Maria do Carmo Sampaio Tavares Timenetsky, Terezinha Maria de Paiva
EPI_ISL_416034 hCoV-19/Brazil/SPBR-12/2020 South America / Brazil / Sao Paulo / Sao Paulo 2020-03-04 Hospital Israelita Albert Einstein Instituto Adolfo Lutz, Interdiciplinary Procedures Center, Strategic Laboratory Claudio Tavares Sacchi, Claudia Regina Gonçalves, Carlos Henrique Camargo, Erica Valessa Ramos Gomes, Fabiana Cristina Pereira dos Santos, Daniela Bernardes Borges da Silva, Simone Guadagnucci Morillo, Adriano Abbud, Adriana Bugno, Maria do Carmo Sampaio Tavares Timenetsky, Terezinha Maria de Paiva
EPI_ISL_416035 hCoV-19/Brazil/SPBR-13/2020 South America / Brazil / Sao Paulo / Sao Paulo 2020-03-05 National Influenza Center - Instituto Adolfo Lutz Instituto Adolfo Lutz, Interdiciplinary Procedures Center, Strategic Laboratory Claudio Tavares Sacchi, Claudia Regina Gonçalves, Carlos Henrique Camargo, Erica Valessa Ramos Gomes, Fabiana Cristina Pereira dos Santos, Daniela Bernardes Borges da Silva, Simone Guadagnucci Morillo, Adriano Abbud, Adriana Bugno, Maria do Carmo Sampaio Tavares Timenetsky, Terezinha Maria de Paiva
EPI_ISL_416036 hCoV-19/Brazil/SPBR-14/2020 South America / Brazil / Sao Paulo / Sao Paulo 2020-03-05 National Influenza Center - Instituto Adolfo Lutz Instituto Adolfo Lutz, Interdiciplinary Procedures Center, Strategic Laboratory Claudio Tavares Sacchi, Claudia Regina Gonçalves, Carlos Henrique Camargo, Erica Valessa Ramos Gomes, Fabiana Cristina Pereira dos Santos, Daniela Bernardes Borges da Silva, Simone Guadagnucci Morillo, Adriano Abbud, Adriana Bugno, Maria do Carmo Sampaio Tavares Timenetsky, Terezinha Maria de Paiva
EPI_ISL_416140 hCoV-19/Denmark/SSI-05/2020 Europe / Denmark / Copenhagen 2020-03-02 Department of Virus and Microbiological Special diagnostics, Statens Serum Institut, Copenhagen, Denmark. Statens Serum Institute Morten Rasmussen, Maiken Worsoe Rosenstierne , Anders Fomsgaard
EPI_ISL_416141 hCoV-19/Denmark/SSI-09/2020 Europe / Denmark / Copenhagen 2020-03-03 Department of Virus and Microbiological Special diagnostics, Statens Serum Institut, Copenhagen, Denmark. Statens Serum Institute Morten Rasmussen, Maiken Worsoe Rosenstierne , Anders Fomsgaard
EPI_ISL_416142 hCoV-19/Denmark/SSI-01/2020 Europe / Denmark / Copenhagen 2020-02-26 Department of Virus and Microbiological Special diagnostics, Statens Serum Institut, Copenhagen, Denmark. Statens Serum Institute Morten Rasmussen, Maiken Worsoe Rosenstierne , Anders Fomsgaard
EPI_ISL_416143 hCoV-19/Denmark/SSI-02/2020 Europe / Denmark / Copenhagen 2020-02-28 Department of Virus and Microbiological Special diagnostics, Statens Serum Institut, Copenhagen, Denmark. ViFU Morten Rasmussen, Maiken Worsoe Rosenstierne , Anders Fomsgaard
EPI_ISL_416144 hCoV-19/Denmark/SSI-03/2020 Europe / Denmark / Copenhagen 2020-03-01 Department of Virus and Microbiological Special diagnostics, Statens Serum Institut, Copenhagen, Denmark. ViFU Morten Rasmussen, Maiken Worsoe Rosenstierne , Anders Fomsgaard
EPI_ISL_416153 hCoV-19/Denmark/SSI-04/2020 Europe / Denmark / Copenhagen 2020-03-02 Department of Virus and Microbiological Special diagnostics, Statens Serum Institut, Copenhagen, Denmark. ViFU Morten Rasmussen, Maiken Worsoe Rosenstierne , Anders Fomsgaard
EPI_ISL_417301 hCoV-19/England/20110000606/2020 Europe / United Kingdom / England 2020-03-06 Respiratory Virus Unit, Microbiology Services Colindale, Public Health England Respiratory Virus Unit, Microbiology Services Colindale, Public Health England Monica Galiano, Shahjahan Miah, Angie Lackenby, Omolola Akinbami, Tiina Talts, Leena Bhaw, Richard Myers, Steven Platt, Kirstin Edwards, Jonathan Hubb, Joanna Ellis, Maria Zambon
EPI_ISL_417302 hCoV-19/England/20110000706/2020 Europe / United Kingdom / England 2020-03-05 Respiratory Virus Unit, Microbiology Services Colindale, Public Health England Respiratory Virus Unit, Microbiology Services Colindale, Public Health England Monica Galiano, Shahjahan Miah, Angie Lackenby, Omolola Akinbami, Tiina Talts, Leena Bhaw, Richard Myers, Steven Platt, Kirstin Edwards, Jonathan Hubb, Joanna Ellis, Maria Zambon
EPI_ISL_417307 hCoV-19/England/20110023706/2020 Europe / United Kingdom / England 2020-03-08 Respiratory Virus Unit, Microbiology Services Colindale, Public Health England Respiratory Virus Unit, Microbiology Services Colindale, Public Health England Monica Galiano, Shahjahan Miah, Angie Lackenby, Omolola Akinbami, Tiina Talts, Leena Bhaw, Richard Myers, Steven Platt, Kirstin Edwards, Jonathan Hubb, Joanna Ellis, Maria Zambon
EPI_ISL_417311 hCoV-19/England/20110058706/2020 Europe / United Kingdom / England 2020-03-09 Respiratory Virus Unit, Microbiology Services Colindale, Public Health England Respiratory Virus Unit, Microbiology Services Colindale, Public Health England Monica Galiano, Shahjahan Miah, Angie Lackenby, Omolola Akinbami, Tiina Talts, Leena Bhaw, Richard Myers, Steven Platt, Kirstin Edwards, Jonathan Hubb, Joanna Ellis,

Maria Zambon
EPI_ISL_417312 hCoV-19/England/20110059306/2020 Europe / United Kingdom / England 2020-03-08 Respiratory Virus Unit, Microbiology Services Colindale, Public Health England Respiratory Virus Unit, Microbiology Services Colindale, Public Health England Monica Galiano, Shahjahan Miah, Angie Lackenby, Omolola Akinbami, Tiina Talts, Leena Bhaw, Richard Myers, Steven Platt, Kirstin Edwards, Jonathan Hubb, Joanna Ellis, Maria Zambon
EPI_ISL_417313 hCoV-19/England/20110097506/2020 Europe / United Kingdom / England 2020-03-08 Respiratory Virus Unit, Microbiology Services Colindale, Public Health England Respiratory Virus Unit, Microbiology Services Colindale, Public Health England Monica Galiano, Shahjahan Miah, Angie Lackenby, Omolola Akinbami, Tiina Talts, Leena Bhaw, Richard Myers, Steven Platt, Kirstin Edwards, Jonathan Hubb, Joanna Ellis, Maria Zambon
EPI_ISL_417314 hCoV-19/England/20112112106/2020 Europe / United Kingdom / England 2020-03-10 Respiratory Virus Unit, Microbiology Services Colindale, Public Health England Respiratory Virus Unit, Microbiology Services Colindale, Public Health England Monica Galiano, Shahjahan Miah, Angie Lackenby, Omolola Akinbami, Tiina Talts, Leena Bhaw, Richard Myers, Steven Platt, Kirstin Edwards, Jonathan Hubb, Joanna Ellis, Maria Zambon
EPI_ISL_417315 hCoV-19/England/20118044606/2020 Europe / United Kingdom / England 2020-03-13 Respiratory Virus Unit, Microbiology Services Colindale, Public Health England Respiratory Virus Unit, Microbiology Services Colindale, Public Health England Monica Galiano, Shahjahan Miah, Angie Lackenby, Omolola Akinbami, Tiina Talts, Leena Bhaw, Richard Myers, Steven Platt, Kirstin Edwards, Jonathan Hubb, Joanna Ellis, Maria Zambon
EPI_ISL_417317 hCoV-19/USA/CA-SCCPHD-UC13/2020 North America / USA / California / Santa Clara County 2020-03-02 Santa Clara County Public Health Department Chiu Laboratory, University of California, San Francisco Xianding Deng, Scot Federman, Wei Gu, Elsa Villarino, Brandon Bonin, Debra A. Wadford, and Charles Y. Chiu
EPI_ISL_417318 hCoV-19/USA/CA-SCCPHD-UC14/2020 North America / USA / California / Santa Clara County 2020-02-29 Santa Clara County Public Health Department Chiu Laboratory, University of California, San Francisco Xianding Deng, Scot Federman, Wei Gu, Elsa Villarino, Brandon Bonin, Debra A. Wadford, and Charles Y. Chiu
EPI_ISL_417320 hCoV-19/USA/CA-SCCPHD-UC16/2020 North America / USA / California / Santa Clara County 2020-03-04 Santa Clara County Public Health Department Chiu Laboratory, University of California, San Francisco Xianding Deng, Scot Federman, Wei Gu, Elsa Villarino, Brandon Bonin, Debra A. Wadford, and Charles Y. Chiu
EPI_ISL_417330 hCoV-19/USA/CA-CDPH-UC26/2020 North America / USA / California / San Francisco County 2020-03-13 Chiu Laboratory, University of California, San Francisco Chiu Laboratory, University of California, San Francisco Xianding Deng, Scot Federman, Wei Gu, and Charles Y. Chiu
EPI_ISL_417333 hCoV-19/France/Lyon_06042/2020 Europe / France / ARA 2020-03-04 Institut des Agents Infectieux (IAI), Hospices Civils de Lyon CNR Virus des Infections Respiratoires - France SUD Antonin Bal, Gregory Destras, Gwendolyne Burfin, Solenne Brun, Carine Moustaud, Raphaelle Lamy, Alexandre Gaymard, Maude Bouscambert-Duchamp, Florence Morfin-Sherpa, Martine Valette, Laurence Josset, Bruno Lina
EPI_ISL_417334 hCoV-19/France/Lyon_06056/2020 Europe / France / ARA 2020-03-04 Institut des Agents Infectieux (IAI), Hospices Civils de Lyon CNR Virus des Infections Respiratoires - France SUD Antonin Bal, Gregory Destras, Gwendolyne Burfin, Solenne Brun, Carine Moustaud, Raphaelle Lamy, Alexandre Gaymard, Maude Bouscambert-Duchamp, Florence Morfin-Sherpa, Martine Valette, Laurence Josset, Bruno Lina
EPI_ISL_417335 hCoV-19/France/Lyon_06573/2020 Europe / France / ARA 2020-03-06 Institut des Agents Infectieux (IAI), Hospices Civils de Lyon CNR Virus des Infections Respiratoires - France SUD Antonin Bal, Gregory Destras, Gwendolyne Burfin, Solenne Brun, Carine Moustaud, Raphaelle Lamy, Alexandre Gaymard, Maude Bouscambert-Duchamp, Florence Morfin-Sherpa, Martine Valette, Laurence Josset, Bruno Lina
EPI_ISL_417336 hCoV-19/France/Lyon_0668/2020 Europe / France / ARA 2020-03-06 Institut des Agents Infectieux (IAI), Hospices Civils de Lyon CNR Virus des Infections Respiratoires - France SUD Antonin Bal, Gregory Destras, Gwendolyne Burfin, Solenne Brun, Carine Moustaud, Raphaelle Lamy, Alexandre Gaymard, Maude Bouscambert-Duchamp, Florence Morfin-Sherpa, Martine Valette, Laurence Josset, Bruno Lina
EPI_ISL_417337 hCoV-19/France/Lyon_06625/2020 Europe / France / ARA 2020-03-07 Institut des Agents Infectieux (IAI), Hospices Civils de Lyon CNR Virus des Infections Respiratoires - France SUD Antonin Bal, Gregory Destras, Gwendolyne Burfin, Solenne Brun, Carine Moustaud, Raphaelle Lamy, Alexandre Gaymard, Maude Bouscambert-Duchamp, Florence Morfin-Sherpa, Martine Valette, Laurence Josset, Bruno Lina
EPI_ISL_417338 hCoV-19/France/Macon_06756/2020 Europe / France / ARA 2020-03-07 Centre Hospitalier de Macon CNR Virus des Infections Respiratoires - France SUD Antonin Bal, Gregory Destras, Gwendolyne Burfin, Solenne Brun, Carine Moustaud, Raphaelle Lamy, Alexandre Gaymard, Maude Bouscambert-Duchamp, Florence Morfin-Sherpa, Martine Valette, Laurence Josset, Bruno Lina
EPI_ISL_417339 hCoV-19/France/Lyon_06820/2020 Europe / France / ARA 2020-03-08 Institut des Agents Infectieux (IAI), Hospices Civils de Lyon CNR Virus des Infections Respiratoires - France SUD Antonin Bal, Gregory Destras, Gwendolyne Burfin, Solenne Brun, Carine Moustaud, Raphaelle Lamy, Alexandre Gaymard, Maude Bouscambert-Duchamp, Florence Morfin-Sherpa, Martine Valette, Laurence Josset, Bruno Lina
EPI_ISL_417340 hCoV-19/France/Bourg-en-Bresse_06813/2020 Europe / France / ARA 2020-03-07 Centre Hospitalier de Bourg en Bresse CNR Virus des Infections Respiratoires - France SUD Antonin Bal, Gregory Destras, Gwendolyne Burfin, Solenne Brun, Carine Moustaud, Raphaelle Lamy, Alexandre Gaymard, Maude Bouscambert-Duchamp, Florence Morfin-Sherpa, Martine Valette, Laurence Josset, Bruno Lina
EPI_ISL_417341 hCoV-19/USA/WA-UW192/2020 North America / USA / Washington 2020-03-13 UW Virology Lab UW Virology Lab Pavitra Roychoudhury, Hong Xie, Keith Jerome, Alexander Greninger

EPI_ISL_417342 hCoV-19/USA/WA-UW193/2020   North America / USA / Washington   2020-03-13   UW Virology Lab   UW Virology Lab Pavitra Roychoudhury, Hong Xie, Keith Jerome, Alexander Greninger
EPI_ISL_417343 hCoV-19/USA/WA-UW194/2020   North America / USA / Washington   2020-03-13   UW Virology Lab   UW Virology Lab Pavitra Roychoudhury, Hong Xie, Keith Jerome, Alexander Greninger
EPI_ISL_417344 hCoV-19/USA/WA-UW195/2020   North America / USA / Washington   2020-03-13   UW Virology Lab   UW Virology Lab Pavitra Roychoudhury, Hong Xie, Keith Jerome, Alexander Greninger
EPI_ISL_417345 hCoV-19/USA/WA-UW196/2020   North America / USA / Washington   2020-03-13   UW Virology Lab   UW Virology Lab Pavitra Roychoudhury, Hong Xie, Keith Jerome, Alexander Greninger
EPI_ISL_417346 hCoV-19/USA/WA-UW197/2020   North America / USA / Washington   2020-03-12   UW Virology Lab   UW Virology Lab Pavitra Roychoudhury, Hong Xie, Keith Jerome, Alexander Greninger
EPI_ISL_417347 hCoV-19/USA/WA-UW198/2020   North America / USA / Washington   2020-03-12   UW Virology Lab   UW Virology Lab Pavitra Roychoudhury, Hong Xie, Keith Jerome, Alexander Greninger
EPI_ISL_417348 hCoV-19/USA/WA-UW199/2020   North America / USA / Washington   2020-03-13   UW Virology Lab   UW Virology Lab Pavitra Roychoudhury, Hong Xie, Keith Jerome, Alexander Greninger
EPI_ISL_417349 hCoV-19/USA/WA-UW200/2020   North America / USA / Washington   2020-03-12   UW Virology Lab   UW Virology Lab Pavitra Roychoudhury, Hong Xie, Keith Jerome, Alexander Greninger
EPI_ISL_417350 hCoV-19/USA/WA-UW201/2020   North America / USA / Washington   2020-03-12   UW Virology Lab   UW Virology Lab Pavitra Roychoudhury, Hong Xie, Keith Jerome, Alexander Greninger
EPI_ISL_417351 hCoV-19/USA/WA-UW202/2020   North America / USA / Washington   2020-03-12   UW Virology Lab   UW Virology Lab Pavitra Roychoudhury, Hong Xie, Keith Jerome, Alexander Greninger
EPI_ISL_417352 hCoV-19/USA/WA-UW203/2020   North America / USA / Washington   2020-03-12   UW Virology Lab   UW Virology Lab Pavitra Roychoudhury, Hong Xie, Keith Jerome, Alexander Greninger
EPI_ISL_417353 hCoV-19/USA/WA-UW204/2020   North America / USA / Washington   2020-03-13   UW Virology Lab   UW Virology Lab Pavitra Roychoudhury, Hong Xie, Keith Jerome, Alexander Greninger
EPI_ISL_417354 hCoV-19/USA/WA-UW205/2020   North America / USA / Washington   2020-03-13   UW Virology Lab   UW Virology Lab Pavitra Roychoudhury, Hong Xie, Keith Jerome, Alexander Greninger
EPI_ISL_417355 hCoV-19/USA/WA-UW206/2020   North America / USA / Washington   2020-03-13   UW Virology Lab   UW Virology Lab Pavitra Roychoudhury, Hong Xie, Keith Jerome, Alexander Greninger
EPI_ISL_417356 hCoV-19/USA/WA-UW207/2020   North America / USA / Washington   2020-03-13   UW Virology Lab   UW Virology Lab Pavitra Roychoudhury, Hong Xie, Keith Jerome, Alexander Greninger
EPI_ISL_417358 hCoV-19/USA/WA-UW209/2020   North America / USA / Washington   2020-03-13   UW Virology Lab   UW Virology Lab Pavitra Roychoudhury, Hong Xie, Keith Jerome, Alexander Greninger
EPI_ISL_417359 hCoV-19/USA/WA-UW210/2020   North America / USA / Washington   2020-03-14   UW Virology Lab   UW Virology Lab Pavitra Roychoudhury, Hong Xie, Keith Jerome, Alexander Greninger
EPI_ISL_417360 hCoV-19/USA/WA-UW211/2020   North America / USA / Washington   2020-03-14   UW Virology Lab   UW Virology Lab Pavitra Roychoudhury, Hong Xie, Keith Jerome, Alexander Greninger
EPI_ISL_417361 hCoV-19/USA/WA-UW212/2020   North America / USA / Washington   2020-03-14   UW Virology Lab   UW Virology Lab Pavitra Roychoudhury, Hong Xie, Keith Jerome, Alexander Greninger
EPI_ISL_417362 hCoV-19/USA/CT-UW213/2020   North America / USA / Connecticut   2020-03-13   UW Virology Lab   UW Virology Lab Pavitra Roychoudhury, Hong Xie, Keith Jerome, Alexander Greninger
EPI_ISL_417363 hCoV-19/USA/WA-UW214/2020   North America / USA / Washington   2020-03-14   UW Virology Lab   UW Virology Lab Pavitra Roychoudhury, Hong Xie, Keith Jerome, Alexander Greninger
EPI_ISL_417364 hCoV-19/USA/WA-UW215/2020   North America / USA / Washington   2020-03-12   UW Virology Lab   UW Virology Lab Pavitra Roychoudhury, Hong Xie, Keith Jerome, Alexander Greninger
EPI_ISL_417365 hCoV-19/USA/WA-UW216/2020   North America / USA / Washington   2020-03-14   UW Virology Lab   UW Virology Lab Pavitra Roychoudhury, Hong Xie, Keith Jerome, Alexander Greninger
EPI_ISL_417366 hCoV-19/USA/WA-UW217/2020   North America / USA / Washington   2020-03-14   UW Virology Lab   UW Virology Lab Pavitra Roychoudhury, Hong Xie, Keith Jerome, Alexander Greninger
EPI_ISL_417367 hCoV-19/USA/WA-UW218/2020   North America / USA / Washington   2020-03-13   UW Virology Lab   UW Virology Lab Pavitra Roychoudhury, Hong Xie, Keith Jerome, Alexander Greninger
EPI_ISL_417368 hCoV-19/USA/UN-UW219/2020   North America / USA   2020-03-13   UW Virology Lab UW Virology Lab   Pavitra Roychoudhury, Hong Xie, Keith Jerome, Alexander Greninger
EPI_ISL_417369 hCoV-19/USA/WA-UW220/2020   North America / USA / Washington   2020-03-14   UW Virology Lab   UW Virology Lab Pavitra Roychoudhury, Hong Xie, Keith Jerome, Alexander Greninger
EPI_ISL_417390 hCoV-19/Australia/NSW27/2020   Oceania / Australia / New South Wales / Sydney 2020-03-08 Centre for Infectious Diseases and Microbiology Public Health   NSW Health Pathology - Institute of Clinical Pathology and Medical Research; Westmead Hospital; University of Sydney Sadsad R, Carter I, Rahman H, Holmes EC, O'Sullivan MV, Sintchenko V, Chen SC, Maddocks S, Kok J, Dwyer DE, Rockett R, Eden J-S, Lam C, Gray K, Timms V, Gall M and Arnott A for the 2019-nCoV Study Group
EPI_ISL_417391 hCoV-19/Australia/NSW28/2020   Oceania / Australia / New South Wales / Sydney 2020-03-08 Centre for Infectious Diseases and Microbiology Public Health   NSW Health Pathology - Institute of Clinical Pathology and Medical Research; Westmead Hospital; University of Sydney Carter I, Rahman H, Holmes EC, O'Sullivan MV, Sintchenko V, Chen SC, Maddocks S, Kok J, Dwyer DE, Rockett R, Eden J-S, Lam C, Gray K, Timms V, Gall M, Arnott A and Sadsad R for the 2019-nCoV Study Group
EPI_ISL_417392 hCoV-19/Australia/NSW31/2020   Oceania / Australia / New South Wales / Sydney 2020-03-10 Centre for Infectious Diseases and Microbiology Public Health   NSW Health Pathology - Institute of Clinical Pathology and Medical Research; Westmead Hospital; University of Sydney Rahman H, Holmes EC, O'Sullivan MV, Sintchenko V, Chen SC, Maddocks S, Kok J, Dwyer DE, Rockett R, Eden J-S, Lam C, Gray K, Timms V, Gall M, Arnott A, Sadsad R and Carter I for the 2019-nCoV Study Group
EPI_ISL_417393 hCoV-19/Australia/NSW35/2020   Oceania / Australia / New South Wales / Sydney 2020-03-08

Centre for Infectious Diseases and Microbiology Public Health NSW Health Pathology - Institute of Clinical Pathology and Medical Research; Westmead Hospital; University of Sydney Holmes EC, O'Sullivan MV, Sintchenko V, Chen SC, Maddocks S, Kok J, Dwyer DE, Rockett R, Eden J-S, Lam C, Gray K, Timms V, Gall M, Arnott A, Sadsad R, Carter I and Rahman H for the 2019-nCoV Study Group
EPI_ISL_417394 hCoV-19/Australia/NSW36/2020 Oceania / Australia / New South Wales / Sydney 2020-03-10 Centre for Infectious Diseases and Microbiology Public Health NSW Health Pathology - Institute of Clinical Pathology and Medical Research; Westmead Hospital; University of Sydney O'Sullivan MV, Sintchenko V, Chen SC, Maddocks S, Kok J, Dwyer DE, Rockett R, Eden J-S, Lam C, Gray K, Timms V, Gall M, Arnott A, Sadsad R, Carter I, Rahman H and Holmes EC for the 2019-nCoV Study Group
EPI_ISL_417395 hCoV-19/Australia/NSW37/2020 Oceania / Australia / New South Wales / Sydney 2020-03-10 Centre for Infectious Diseases and Microbiology Public Health NSW Health Pathology - Institute of Clinical Pathology and Medical Research; Westmead Hospital; University of Sydney Sintchenko V, Chen SC, Maddocks S, Kok J, Dwyer DE, Rockett R, Eden J-S, Lam C, Gray K, Timms V, Gall M, Arnott A, Sadsad R, Carter I, Rahman H, Holmes EC and O'Sullivan MV for the 2019-nCoV Study Group
EPI_ISL_417396 hCoV-19/Australia/NSW38/2020 Oceania / Australia / New South Wales / Sydney 2020-03-10 Centre for Infectious Diseases and Microbiology Public Health NSW Health Pathology - Institute of Clinical Pathology and Medical Research; Westmead Hospital; University of Sydney Chen SC, Maddocks S, Kok J, Dwyer DE, Rockett R, Eden J-S, Lam C, Gray K, Timms V, Gall M, Arnott A, Sadsad R, Carter I, Rahman H, Holmes EC, O'Sullivan MV and Sintchenko V for the 2019-nCoV Study Group
EPI_ISL_417397 hCoV-19/Australia/NSW39/2020 Oceania / Australia / New South Wales / Sydney 2020-03-11 Centre for Infectious Diseases and Microbiology Public Health NSW Health Pathology - Institute of Clinical Pathology and Medical Research; Westmead Hospital; University of Sydney Maddocks S, Kok J, Dwyer DE, Rockett R, Eden J-S, Lam C, Gray K, Timms V, Gall M, Arnott A, Sadsad R, Carter I, Rahman H, Holmes EC, O'Sullivan MV, Sintchenko V and Chen SC for the 2019-nCoV Study Group
EPI_ISL_417398 hCoV-19/Australia/NSW40/2020 Oceania / Australia / New South Wales / Sydney 2020-03-11 Centre for Infectious Diseases and Microbiology Public Health NSW Health Pathology - Institute of Clinical Pathology and Medical Research; Westmead Hospital; University of Sydney Kok J, Dwyer DE, Rockett R, Eden J-S, Lam C, Gray K, Timms V, Gall M, Arnott A, Sadsad R, Carter I, Rahman H, Holmes EC, O'Sullivan MV, Sintchenko V, Chen SC and Maddocks S for the 2019-nCoV Study Group
EPI_ISL_417399 hCoV-19/Australia/NSW41/2020 Oceania / Australia / New South Wales / Sydney 2020-03-10 Centre for Infectious Diseases and Microbiology Public Health NSW Health Pathology - Institute of Clinical Pathology and Medical Research; Westmead Hospital; University of Sydney Dwyer DE, Rockett R, Eden J-S, Lam C, Gray K, Timms V, Gall M, Arnott A, Sadsad R, Carter I, Rahman H, Holmes EC, O'Sullivan MV, Sintchenko V, Chen SC, Maddocks S and Kok J for the 2019-nCoV Study Group
EPI_ISL_417408 hCoV-19/Australia/NSW56/2020 Oceania / Australia / New South Wales / Sydney 2020-03-17 Centre for Infectious Diseases and Microbiology Public Health NSW Health Pathology - Institute of Clinical Pathology and Medical Research; Westmead Hospital; University of Sydney Carter I, Rahman H, Holmes EC, O'Sullivan MV, Sintchenko V, Chen SC, Maddocks S, Kok J, Dwyer DE, Rockett R, Eden J-S, Lam C, Gray K, Timms V, Gall M, Arnott A and Sadsad R for the 2019-nCoV Study Group
EPI_ISL_417409 hCoV-19/Australia/NSW57/2020 Oceania / Australia / New South Wales / Sydney 2020-03-16 Centre for Infectious Diseases and Microbiology Public Health NSW Health Pathology - Institute of Clinical Pathology and Medical Research; Westmead Hospital; University of Sydney Rahman H, Holmes EC, O'Sullivan MV, Sintchenko V, Chen SC, Maddocks S, Kok J, Dwyer DE, Rockett R, Eden J-S, Lam C, Gray K, Timms V, Gall M, Arnott A, Sadsad R and Carter I for the 2019-nCoV Study Group
EPI_ISL_417410 hCoV-19/Australia/NSW58/2020 Oceania / Australia / New South Wales / Sydney 2020-03-16 Centre for Infectious Diseases and Microbiology Public Health NSW Health Pathology - Institute of Clinical Pathology and Medical Research; Westmead Hospital; University of Sydney Holmes EC, O'Sullivan MV, Sintchenko V, Chen SC, Maddocks S, Kok J, Dwyer DE, Rockett R, Eden J-S, Lam C, Gray K, Timms V, Gall M, Arnott A, Sadsad R, Carter I and Rahman H for the 2019-nCoV Study Group
EPI_ISL_417411 hCoV-19/Australia/NSW61/2020 Oceania / Australia / New South Wales / Sydney 2020-03-17 Centre for Infectious Diseases and Microbiology Public Health NSW Health Pathology - Institute of Clinical Pathology and Medical Research; Westmead Hospital; University of Sydney O'Sullivan MV, Sintchenko V, Chen SC, Maddocks S, Kok J, Dwyer DE, Rockett R, Eden J-S, Lam C, Gray K, Timms V, Gall M, Arnott A, Sadsad R, Carter I, Rahman H and Holmes EC for the 2019-nCoV Study Group
EPI_ISL_417412 hCoV-19/Australia/NSW62/2020 Oceania / Australia / New South Wales / Sydney 2020-03-17 Centre for Infectious Diseases and Microbiology Public Health NSW Health Pathology - Institute of Clinical Pathology and Medical Research; Westmead Hospital; University of Sydney Sintchenko V, Chen SC, Maddocks S, Kok J, Dwyer DE, Rockett R, Eden J-S, Lam C, Gray K, Timms V, Gall M, Arnott A, Sadsad R, Carter I, Rahman H, Holmes EC and O'Sullivan MV for the 2019-nCoV Study Group
EPI_ISL_417418 hCoV-19/Italy/FVG-ICGEB_S1/2020 Europe / Italy / Friuli Venezia Giulia 2020-03-01 Laboratory of Molecular Virology International Center fro Genetic Engineering and Biotechnology (ICGEB) ARGO Open Lab Platform for Genome sequencing Licastro D, Rajasekharan S, Dal Monego S, Segat L, D'Agaro P, Marcello A
EPI_ISL_417419 hCoV-19/Italy/FVG-ICGEB_S5/2020 Europe / Italy / Friuli Venezia Giulia 2020-03-01 Laboratory of Molecular Virology International Center for Genetic Engineering and Biotechnology (ICGEB) ARGO Open Lab Platform for Genome sequencing Licastro D, Rajasekharan S, Dal Monego S, Segat L, D'Agaro P, Marcello A
EPI_ISL_417420 hCoV-19/NanChang/JX216/2020 Asia / China / NanChang 2020-03-23 Jiangxi province Center for Disease Control and Prevention Jiangxi province Center for Disease Control and Prevention Li jian Xiong

EPI_ISL_417421 hCoV-19/Italy/FVG-ICGEB_S8/2020 Europe / Italy / Friuli Venezia Giulia 2020-03-01 Laboratory of Molecular Virology International Center for Genetic Engineering and Biotechnology (ICGEB) ARGO Open Lab Platform for Genome sequencing Licastro D, Rajasekharan S, Dal Monego S, Segat L, D'Agaro P, Marcello A
EPI_ISL_417422 hCoV-19/Belgium/DHWM-03041/2020 Europe / Belgium / Dilbeek 2020-03-04 KU Leuven, Clinical and Epidemiological Virology KU Leuven, Clinical and Epidemiological Virology Joan Marti-Carerras, Tony Wawina, Bert Vanmechelen, Piet Maes
EPI_ISL_417423 hCoV-19/Italy/FVG-ICGEB_S9/2020 Europe / Italy / Friuli Venezia Giulia 2020-03-01 Laboratory of Molecular Virology International Center for Genetic Engineering and Biotechnology (ICGEB) ARGO Open Lab Platform for Genome sequencing Licastro D, Rajasekharan, Dal Monego S, Segat L, D'Agaro P, Marcello A
EPI_ISL_417424 hCoV-19/Belgium/KA-03042/2020 Europe / Belgium / Kasterlee 2020-03-04 KU Leuven, Clinical and Epidemiological Virology KU Leuven, Clinical and Epidemiological Virology Joan Marti-Carerras, Tony Wawina, Bert Vanmechelen, Piet Maes
EPI_ISL_417425 hCoV-19/Belgium/VBK-03061/2020 Europe / Belgium / Schoten 2020-03-06 KU Leuven, Clinical and Epidemiological Virology KU Leuven, Clinical and Epidemiological Virology Joan Marti-Carerras, Tony Wawina, Bert Vanmechelen, Piet Maes
EPI_ISL_417426 hCoV-19/Belgium/SQ-03043/2020 Europe / Belgium / Leuven 2020-03-04 KU Leuven, Clinical and Epidemiological Virology KU Leuven, Clinical and Epidemiological Virology Joan Marti-Carerras, Tony Wawina, Bert Vanmechelen, Piet Maes
EPI_ISL_417427 hCoV-19/Belgium/VI-03027/2020 Europe / Belgium / Sint-Pieters-Woluwe 2020-03-02 KU Leuven, Clinical and Epidemiological Virology KU Leuven, Clinical and Epidemiological Virology Joan Marti-Carerras, Tony Wawina, Bert Vanmechelen, Piet Maes
EPI_ISL_417428 hCoV-19/Belgium/FR-03029/2020 Europe / Belgium / Rixensart 2020-03-02 KU Leuven, Clinical and Epidemiological Virology KU Leuven, Clinical and Epidemiological Virology Joan Marti-Carerras, Tony Wawina, Bert Vanmechelen, Piet Maes
EPI_ISL_417429 hCoV-19/Belgium/MMJ-03034/2020 Europe / Belgium / Holsbeek 2020-03-03 KU Leuven, Clinical and Epidemiological Virology KU Leuven, Clinical and Epidemiological Virology Joan Marti-Carerras, Tony Wawina, Bert Vanmechelen, Piet Maes
EPI_ISL_417430 hCoV-19/Belgium/RT-03035/2020 Europe / Belgium / Vorst 2020-03-03 KU Leuven, Clinical and Epidemiological Virology KU Leuven, Clinical and Epidemiological Virology Joan Marti-Carerras, Tony Wawina, Bert Vanmechelen, Piet Maes
EPI_ISL_417437 hCoV-19/DRC/KN-0054/2020 Africa / Democratic Republic of the Congo / Kinshasa 2020-03-17 Viral Respiratory Lab, National Institute for Biomedical Research (INRB) Pathogen Sequencing Lab, National Institute for Biomedical Research (INRB) Placide Mbala-Kingebeni, Edith Nkwembe, Eddy Kinganda-Lusamaki, Amuri Aziza, Catherine Pratt, Matthias Pauthner, Josh Quick, Allison Black, James Hadfield, Trevor Bedford, Ian Goodfellow, Nick Loman, Kristian Andersen, Michael Wiley, Steve Ahuka-Mundeke, Jean-Jacques Muyembe Tamfum
EPI_ISL_417438 hCoV-19/DRC/KN-0058/2020 Africa / Democratic Republic of the Congo / Kinshasa 2020-03-17 Viral Respiratory Lab, National Institute for Biomedical Research (INRB) Pathogen Sequencing Lab, National Institute for Biomedical Research (INRB) Placide Mbala-Kingebeni, Edith Nkwembe, Eddy Kinganda-Lusamaki, Amuri Aziza, Catherine Pratt, Matthias Pauthner, Josh Quick, Allison Black, James Hadfield, Trevor Bedford, Ian Goodfellow, Nick Loman, Kristian Andersen, Michael Wiley, Steve Ahuka-Mundeke, Jean-Jacques Muyembe Tamfum
EPI_ISL_417440 hCoV-19/DRC/KN-0060/2020 Africa / Democratic Republic of the Congo / Kinshasa 2020-03-17 Viral Respiratory Lab, National Institute for Biomedical Research (INRB) Pathogen Sequencing Lab, National Institute for Biomedical Research (INRB) Placide Mbala-Kingebeni, Edith Nkwembe, Eddy Kinganda-Lusamaki, Amuri Aziza, Catherine Pratt, Matthias Pauthner, Josh Quick, Allison Black, James Hadfield, Trevor Bedford, Ian Goodfellow, Nick Loman, Kristian Andersen, Michael Wiley, Steve Ahuka-Mundeke, Jean-Jacques Muyembe Tamfum
EPI_ISL_417442 hCoV-19/DRC/KN-0072/2020 Africa / Democratic Republic of the Congo / Kinshasa 2020-03-18 Viral Respiratory Lab, National Institute for Biomedical Research (INRB) Pathogen Sequencing Lab, National Institute for Biomedical Research (INRB) Placide Mbala-Kingebeni, Edith Nkwembe, Eddy Kinganda-Lusamaki, Amuri Aziza, Catherine Pratt, Matthias Pauthner, Josh Quick, Allison Black, James Hadfield, Trevor Bedford, Ian Goodfellow, Nick Loman, Kristian Andersen, Michael Wiley, Steve Ahuka-Mundeke, Jean-Jacques Muyembe Tamfum
EPI_ISL_417443 hCoV-19/Hong Kong/XM-PII-S4/2020 Asia / Hong Kong 2020-01-22 State Key Laboratory for Emerging Infectious Diseases Department of Microbiology Li Ka Shing Faculty of Medicine The University of Hong Kong State Key Laboratory for Emerging Infectious Diseases Department of Microbiology Li Ka Shing Faculty of Medicine The University of Hong Kong Pui Wang, Siu-Ying Lau, Shaofeng Deng, Bobo Wing-Yee Mok, Wenjun Song, Kwok-Yung Yuen, Honglin Chen
EPI_ISL_417444 hCoV-19/Pakistan/Gilgit1/2020 Asia / Pakistan / Gilgit 2020-03-04 unknown Department of Healthcare Biotechnology Javed,A., Niazi,S.K., Ghani,E., Saqib,M., Janjua,H.A., Corman,V.M. and Zohaib,A.
EPI_ISL_417445 hCoV-19/Italy/UniMI01/2020 Europe / Italy / Lombardy / Milan 2020-02-24 Laboratory of Infectious Diseases, Department of Biomedical and Clinical Sciences L. Sacco, University of Milan Laboratory of Infectious Diseases, Department of Biomedical and Clinical Sciences L. Sacco, University of Milan Gianguglielmo Zehender, Alessia Lai, Annalisa Bergna, Luca Meroni, Agostino Riva, Claudia Balotta, Maciej Tarkowski, Arianna Gabrieli, Dario Bernacchia, Stefano Rusconi, Giuliano Rizzardini, Spinello Antinori, Massimo Galli
EPI_ISL_417447 hCoV-19/Italy/UniMI03/2020 Europe / Italy / Lombardy / Milan 2020-02-24

Laboratory of Infectious Diseases, Department of Biomedical and Clinical Sciences L. Sacco, University of Milan
Laboratory of Infectious Diseases, Department of Biomedical and Clinical Sciences L. Sacco, University of Milan
Gianguglielmo Zehender, Alessia Lai, Annalisa Bergna, Luca Meroni, Agostino Riva, Claudia Balotta, Maciej Tarkowski, Arianna Gabrieli, Dario Bernacchia, Stefano Rusconi, Giuliano Rizzardini, Spinello Antinori, Massimo Galli
EPI_ISL_417448 hCoV-19/USA/WA-UW234/2020 North America / USA / Washington 2020-03-14 UW Virology Lab UW Virology Lab Pavitra Roychoudhury, Hong Xie, Keith Jerome, Alexander Greninger
EPI_ISL_417449 hCoV-19/USA/WA-UW235/2020 North America / USA / Washington 2020-03-14 UW Virology Lab UW Virology Lab Pavitra Roychoudhury, Hong Xie, Keith Jerome, Alexander Greninger
EPI_ISL_417450 hCoV-19/USA/WA-UW236/2020 North America / USA / Washington 2020-03-14 UW Virology Lab UW Virology Lab Pavitra Roychoudhury, Hong Xie, Keith Jerome, Alexander Greninger
EPI_ISL_417451 hCoV-19/USA/WA-UW237/2020 North America / USA / Washington 2020-03-14 UW Virology Lab UW Virology Lab Pavitra Roychoudhury, Hong Xie, Keith Jerome, Alexander Greninger
EPI_ISL_417452 hCoV-19/USA/CT-UW238/2020 North America / USA / Connecticut 2020-03-12 UW Virology Lab UW Virology Lab Pavitra Roychoudhury, Hong Xie, Keith Jerome, Alexander Greninger
EPI_ISL_417453 hCoV-19/USA/WA-UW239/2020 North America / USA / Washington 2020-03-14 UW Virology Lab UW Virology Lab Pavitra Roychoudhury, Hong Xie, Keith Jerome, Alexander Greninger
EPI_ISL_417454 hCoV-19/USA/WA-UW240/2020 North America / USA / Washington 2020-03-14 UW Virology Lab UW Virology Lab Pavitra Roychoudhury, Hong Xie, Keith Jerome, Alexander Greninger
EPI_ISL_417455 hCoV-19/USA/WA-UW241/2020 North America / USA / Washington 2020-03-14 UW Virology Lab UW Virology Lab Pavitra Roychoudhury, Hong Xie, Keith Jerome, Alexander Greninger
EPI_ISL_417456 hCoV-19/USA/WA-UW242/2020 North America / USA / Washington 2020-03-13 UW Virology Lab UW Virology Lab Pavitra Roychoudhury, Hong Xie, Keith Jerome, Alexander Greninger
EPI_ISL_417457 hCoV-19/Germany/NRW-12/2020 Europe / Germany / North Rhine Westphalia / Duesseldorf 2020-03-10 Center of Medical Microbiology, Virology, and Hospital Hygiene, University of Duesseldorf Center of Medical Microbiology, Virology, and Hospital Hygiene, University of Duesseldorf Ortwin Adams, Marcel Andree, Alexander Dilthey, Torsten Feldt, Sandra Hauka, Torsten Houwaart, Björn-Erik Jensen, Detlef Kindgen-Milles, Malte Kohns Vasconcelos, Klaus Pfeffer, Tina Senff, Daniel Strelow, Jörg Timm, Andreas Walker, Tobias Wienemann
EPI_ISL_417458 hCoV-19/Germany/NRW-13/2020 Europe / Germany / North Rhine Westphalia / Duesseldorf 2020-03-11 Center of Medical Microbiology, Virology, and Hospital Hygiene, University of Duesseldorf Center of Medical Microbiology, Virology, and Hospital Hygiene, University of Duesseldorf Ortwin Adams, Marcel Andree, Alexander Dilthey, Torsten Feldt, Sandra Hauka, Torsten Houwaart, Björn-Erik Jensen, Detlef Kindgen-Milles, Malte Kohns Vasconcelos, Klaus Pfeffer, Tina Senff, Daniel Strelow, Jörg Timm, Andreas Walker, Tobias Wienemann
EPI_ISL_417459 hCoV-19/Germany/NRW-14/2020 Europe / Germany / North Rhine Westphalia / Duesseldorf 2020-03-11 Center of Medical Microbiology, Virology, and Hospital Hygiene, University of Duesseldorf Center of Medical Microbiology, Virology, and Hospital Hygiene, University of Duesseldorf Ortwin Adams, Marcel Andree, Alexander Dilthey, Torsten Feldt, Sandra Hauka, Torsten Houwaart, Björn-Erik Jensen, Detlef Kindgen-Milles, Malte Kohns Vasconcelos, Klaus Pfeffer, Tina Senff, Daniel Strelow, Jörg Timm, Andreas Walker, Tobias Wienemann
EPI_ISL_417460 hCoV-19/Germany/NRW-15/2020 Europe / Germany / North Rhine Westphalia / Duesseldorf 2020-03-11 Center of Medical Microbiology, Virology, and Hospital Hygiene, University of Duesseldorf Center of Medical Microbiology, Virology, and Hospital Hygiene, University of Duesseldorf Ortwin Adams, Marcel Andree, Alexander Dilthey, Torsten Feldt, Sandra Hauka, Torsten Houwaart, Björn-Erik Jensen, Detlef Kindgen-Milles, Malte Kohns Vasconcelos, Klaus Pfeffer, Tina Senff, Daniel Strelow, Jörg Timm, Andreas Walker, Tobias Wienemann
EPI_ISL_417461 hCoV-19/Germany/NRW-16/2020 Europe / Germany / North Rhine Westphalia / Duesseldorf 2020-03-11 Center of Medical Microbiology, Virology, and Hospital Hygiene, University of Duesseldorf Center of Medical Microbiology, Virology, and Hospital Hygiene, University of Duesseldorf Ortwin Adams, Marcel Andree, Alexander Dilthey, Torsten Feldt, Sandra Hauka, Torsten Houwaart, Björn-Erik Jensen, Detlef Kindgen-Milles, Malte Kohns Vasconcelos, Klaus Pfeffer, Tina Senff, Daniel Strelow, Jörg Timm, Andreas Walker, Tobias Wienemann
EPI_ISL_417462 hCoV-19/Germany/NRW-17/2020 Europe / Germany / North Rhine Westphalia / Duesseldorf 2020-03-11 Center of Medical Microbiology, Virology, and Hospital Hygiene, University of Duesseldorf Center of Medical Microbiology, Virology, and Hospital Hygiene, University of Duesseldorf Ortwin Adams, Marcel Andree, Alexander Dilthey, Torsten Feldt, Sandra Hauka, Torsten Houwaart, Björn-Erik Jensen, Detlef Kindgen-Milles, Malte Kohns Vasconcelos, Klaus Pfeffer, Tina Senff, Daniel Strelow, Jörg Timm, Andreas Walker, Tobias Wienemann
EPI_ISL_417463 hCoV-19/Germany/NRW-18/2020 Europe / Germany / North Rhine Westphalia / Duesseldorf 2020-03-13 Center of Medical Microbiology, Virology, and Hospital Hygiene, University of Duesseldorf Center of Medical Microbiology, Virology, and Hospital Hygiene, University of Duesseldorf Ortwin Adams, Marcel Andree, Alexander Dilthey, Torsten Feldt, Sandra Hauka, Torsten Houwaart, Björn-Erik Jensen, Detlef Kindgen-Milles, Malte Kohns Vasconcelos, Klaus Pfeffer, Tina Senff, Daniel Strelow, Jörg Timm, Andreas Walker, Tobias Wienemann
EPI_ISL_417464 hCoV-19/Germany/NRW-19/2020 Europe / Germany / North Rhine Westphalia / Duesseldorf 2020-03-14 Center of Medical Microbiology, Virology, and Hospital Hygiene, University of Duesseldorf Center of Medical Microbiology, Virology, and Hospital Hygiene, University of Duesseldorf Ortwin Adams, Marcel Andree, Alexander Dilthey, Torsten Feldt, Sandra Hauka, Torsten Houwaart, Björn-Erik Jensen, Detlef Kindgen-Milles, Malte Kohns Vasconcelos, Klaus Pfeffer, Tina Senff, Daniel Strelow, Jörg Timm, Andreas Walker, Tobias

Wienemann
EPI_ISL_417465 hCoV-19/Germany/NRW-20/2020 Europe / Germany / North Rhine Westphalia / Duesseldorf 2020-03-14 Center of Medical Microbiology, Virology, and Hospital Hygiene, University of Duesseldorf Center of Medical Microbiology, Virology, and Hospital Hygiene, University of Duesseldorf Ortwin Adams, Marcel Andree, Alexander Dilthey, Torsten Feldt, Sandra Hauka, Torsten Houwaart, Björn-Erik Jensen, Detlef Kindgen-Milles, Malte Kohns Vasconcelos, Klaus Pfeffer, Tina Senff, Daniel Strelow, Jörg Timm, Andreas Walker, Tobias Wienemann
EPI_ISL_417466 hCoV-19/Germany/NRW-21/2020 Europe / Germany / North Rhine Westphalia / Duesseldorf 2020-03-14 Center of Medical Microbiology, Virology, and Hospital Hygiene, University of Duesseldorf Center of Medical Microbiology, Virology, and Hospital Hygiene, University of Duesseldorf Ortwin Adams, Marcel Andree, Alexander Dilthey, Torsten Feldt, Sandra Hauka, Torsten Houwaart, Björn-Erik Jensen, Detlef Kindgen-Milles, Malte Kohns Vasconcelos, Klaus Pfeffer, Tina Senff, Daniel Strelow, Jörg Timm, Andreas Walker, Tobias Wienemann
EPI_ISL_417467 hCoV-19/Germany/NRW-22/2020 Europe / Germany / North Rhine Westphalia / Duesseldorf 2020-03-15 Center of Medical Microbiology, Virology, and Hospital Hygiene, University of Duesseldorf Center of Medical Microbiology, Virology, and Hospital Hygiene, University of Duesseldorf Ortwin Adams, Marcel Andree, Alexander Dilthey, Torsten Feldt, Sandra Hauka, Torsten Houwaart, Björn-Erik Jensen, Detlef Kindgen-Milles, Malte Kohns Vasconcelos, Klaus Pfeffer, Tina Senff, Daniel Strelow, Jörg Timm, Andreas Walker, Tobias Wienemann
EPI_ISL_417468 hCoV-19/Germany/NRW-23/2020 Europe / Germany / North Rhine Westphalia / Duesseldorf 2020-03-16 Center of Medical Microbiology, Virology, and Hospital Hygiene, University of Duesseldorf Center of Medical Microbiology, Virology, and Hospital Hygiene, University of Duesseldorf Ortwin Adams, Marcel Andree, Alexander Dilthey, Torsten Feldt, Sandra Hauka, Torsten Houwaart, Björn-Erik Jensen, Detlef Kindgen-Milles, Malte Kohns Vasconcelos, Klaus Pfeffer, Tina Senff, Daniel Strelow, Jörg Timm, Andreas Walker, Tobias Wienemann
EPI_ISL_417469 hCoV-19/USA/MN31-MDH31/2020 North America / USA / Minnesota 2020-03-12 Minnesota Department of Health, Public Health Laboratory Minnesota Department of Health, Public Health Laboratory Matt Plumb, Jake Garfin and Xiong Wang
EPI_ISL_417470 hCoV-19/USA/MN32-MDH32/2020 North America / USA / Minnesota 2020-03-12 Minnesota Department of Health, Public Health Laboratory Minnesota Department of Health, Public Health Laboratory Matt Plumb, Jake Garfin and Xiong Wang
EPI_ISL_417471 hCoV-19/USA/MN34-MDH34/2020 North America / USA / Minnesota 2020-03-12 Minnesota Department of Health, Public Health Laboratory Minnesota Department of Health, Public Health Laboratory Matt Plumb, Jake Garfin and Xiong Wang
EPI_ISL_417472 hCoV-19/USA/MN35-MDH35/2020 North America / USA / Minnesota 2020-03-12 Minnesota Department of Health, Public Health Laboratory Minnesota Department of Health, Public Health Laboratory Matt Plumb, Jake Garfin and Xiong Wang
EPI_ISL_417473 hCoV-19/USA/MN36-MDH36/2020 North America / USA / Minnesota 2020-03-13 Minnesota Department of Health, Public Health Laboratory Minnesota Department of Health, Public Health Laboratory Matt Plumb, Jake Garfin and Xiong Wang
EPI_ISL_417474 hCoV-19/USA/MN38-MDH38/2020 North America / USA / Minnesota 2020-03-12 Minnesota Department of Health, Public Health Laboratory Minnesota Department of Health, Public Health Laboratory Matt Plumb, Jake Garfin and Xiong Wang
EPI_ISL_417475 hCoV-19/USA/MN39-MDH39/2020 North America / USA / Minnesota 2020-03-12 Minnesota Department of Health, Public Health Laboratory Minnesota Department of Health, Public Health Laboratory Matt Plumb, Jake Garfin and Xiong Wang
EPI_ISL_417476 hCoV-19/USA/MN40-MDH40/2020 North America / USA / Minnesota 2020-03-12 Minnesota Department of Health, Public Health Laboratory Minnesota Department of Health, Public Health Laboratory Matt Plumb, Jake Garfin and Xiong Wang
EPI_ISL_417477 hCoV-19/USA/MN41-MDH41/2020 North America / USA / Minnesota 2020-03-12 Minnesota Department of Health, Public Health Laboratory Minnesota Department of Health, Public Health Laboratory Matt Plumb, Jake Garfin and Xiong Wang
EPI_ISL_417478 hCoV-19/USA/MN42-MDH42/2020 North America / USA / Minnesota 2020-03-13 Minnesota Department of Health, Public Health Laboratory Minnesota Department of Health, Public Health Laboratory Matt Plumb, Jake Garfin and Xiong Wang
EPI_ISL_417479 hCoV-19/USA/MN43-MDH43/2020 North America / USA / Minnesota 2020-03-12 Minnesota Department of Health, Public Health Laboratory Minnesota Department of Health, Public Health Laboratory Matt Plumb, Jake Garfin and Xiong Wang
EPI_ISL_417480 hCoV-19/USA/MN44-MDH44/2020 North America / USA / Minnesota 2020-03-12 Minnesota Department of Health, Public Health Laboratory Minnesota Department of Health, Public Health Laboratory Matt Plumb, Jake Garfin and Xiong Wang
EPI_ISL_417481 hCoV-19/Iceland/LUH-01/2020 Europe / Iceland / Reykjavik 2020-03-13 deCODE genetics deCODE genetics Daniel F Gudbjartsson, Agnar Helgason, Hakon Jonsson, Olafur T Magnusson, Pall Melsted, Gudmundur L Norddahl, Jona Saemundsdottir, Asgeir Sigurdsson, Patrick Sulem, Arna B Agustsdottir, Berglind Eiriksdottir, Run Fridriksdottir, Elisabet E Gardarsdottir, Gudmundur Georgsson, Olafia S Gretarsdottir, Kjartan R Gudmundsson, Thora R Gunnarsdottir, Arnaldur Gylfason, Hilma Holm, Brynjar O Jensson, Aslaug Jonasdottir, Kamilla S Josefsdottir, Thordur Kristjansson, Droplaug N Magnusdottir, Louise le Roux, Gudrun Sigmundsdottir, Gardar Sveinbjornsson, Kristin E Sveinsdottir, Maney Sveinsdottir, Emil A Thorarensen, Bjarni Thorbjornsson, Gisli Masson, Ingileif Jonsdottir, Alma Moller, Thorolfur Gudnason, Karl G Kristinsson, Unnur Thorsteinsdottir, Kari Stefansson

EPI_ISL_417483 hCoV-19/Norway/1379/2020 Europe / Norway / Oslo 2020-02-27 Oslo University Hospital, Department of Medical Microbiology Norwegian Institute of Public Health Kathrine Stene-Johansen, Kamilla Heddeland Instefjord, Hilde Elshaug, Karoline Bragstad, Olav Hungnes
EPI_ISL_417484 hCoV-19/Norway/1380/2020 Europe / Norway / Oslo 2020-02-26 Oslo University Hospital, Department of Medical Microbiology Norwegian Institute of Public Health, Department of Virology Kathrine Stene-Johansen, Kamilla Heddeland Instefjord, Hilde Elshaug, Karoline Bragstad, Olav Hungnes
EPI_ISL_417485 hCoV-19/Norway/1526/2020 Europe / Norway / Oslo 2020-03-02 University Hospital of Northern Norway, Department for Microbiology and Infectious Disease Control Norwegian Institute of Public Health, Department of Virology Kathrine Stene-Johansen, Kamilla Heddeland Instefjord, Hilde Elshaug, Karoline Bragstad, Olav Hungnes
EPI_ISL_417486 hCoV-19/Norway/1538/2020 Europe / Norway / Oslo 2020-03-01 Hospital of Southern Norway - Kristiansand, Department of Medical Microbiology Norwegian Institute of Public Health, Department of Virology Kathrine Stene-Johansen, Kamilla Heddeland Instefjord, Hilde Elshaug, Karoline Bragstad, Olav Hungnes
EPI_ISL_417487 hCoV-19/Norway/1539/2020 Europe / Norway / Oslo 2020-03-01 Hospital of Southern Norway - Kristiansand, Department of Medical Microbiology Norwegian Institute of Public Health, Department of Virology Kathrine Stene-Johansen, Kamilla Heddeland Instefjord, Hilde Elshaug, Karoline Bragstad, Olav Hungnes
EPI_ISL_417488 hCoV-19/Norway/1493/2020 Europe / Norway / Oslo 2020-02-29 Oslo University Hospital, Department of Medical Microbiology Norwegian Institute of Public Health, Department of Virology Kathrine Stene-Johansen, Kamilla Heddeland Instefjord, Hilde Elshaug, Karoline Bragstad, Olav Hungnes
EPI_ISL_417491 hCoV-19/Italy/UnivPM1/2020 Europe / Italy / Marche 2020-03-03 Virology Laboratory, Department of Biomedical Sciences and Public Health, University Politecnica delle Marche Virology and Legal Medicine Laboratories, Department of Biomedical Sciences and Public Health, University Politecnica delle Marche Bagnarelli,P., Caucci,S., Di Sante,L., Menzo,S., Alessandrini,F., Onofri,V., Turchi,C., Tagliabracci,A.
EPI_ISL_417492 hCoV-19/USA/MN45-MDH45/2020 North America / USA / Minnesota 2020-03-13 Minnesota Department of Health, Public Health Laboratory Minnesota Department of Health, Public Health Laboratory Matt Plumb, Jake Garfin and Xiong Wang
EPI_ISL_417493 hCoV-19/USA/MN46-MDH46/2020 North America / USA / Minnesota 2020-03-13 Minnesota Department of Health, Public Health Laboratory Minnesota Department of Health, Public Health Laboratory Matt Plumb, Jake Garfin and Xiong Wang
EPI_ISL_417494 hCoV-19/USA/MN47-MDH47/2020 North America / USA / Minnesota 2020-03-13 Minnesota Department of Health, Public Health Laboratory Minnesota Department of Health, Public Health Laboratory Matt Plumb, Jake Garfin and Xiong Wang
EPI_ISL_417495 hCoV-19/USA/MN48-MDH48/2020 North America / USA / Minnesota 2020-03-13 Minnesota Department of Health, Public Health Laboratory Minnesota Department of Health, Public Health Laboratory Matt Plumb, Jake Garfin and Xiong Wang
EPI_ISL_417496 hCoV-19/USA/MN51-MDH51/2020 North America / USA / Minnesota 2020-03-13 Minnesota Department of Health, Public Health Laboratory Minnesota Department of Health, Public Health Laboratory Matt Plumb, Jake Garfin and Xiong Wang
EPI_ISL_417497 hCoV-19/USA/MN52-MDH52/2020 North America / USA / Minnesota 2020-03-13 Minnesota Department of Health, Public Health Laboratory Minnesota Department of Health, Public Health Laboratory Matt Plumb, Jake Garfin and Xiong Wang
EPI_ISL_417498 hCoV-19/USA/MN53-MDH53/2020 North America / USA / Minnesota 2020-03-13 Minnesota Department of Health, Public Health Laboratory Minnesota Department of Health, Public Health Laboratory Matt Plumb, Jake Garfin and Xiong Wang
EPI_ISL_417499 hCoV-19/USA/MN55-MDH55/2020 North America / USA / Minnesota 2020-03-13 Minnesota Department of Health, Public Health Laboratory Minnesota Department of Health, Public Health Laboratory Matt Plumb, Jake Garfin and Xiong Wang
EPI_ISL_418624 hCoV-19/Belgium/ULG-6972/2020 Europe / Belgium / Liège 2020-03-15 Department of Clinical Microbiology GIGA Medical Genomics Keith Durkin, Maria Artesi, Sébastien Bontems, Raphaël Boreux, Cécile Meex, Pierrette Melin, Marie-Pierre Hayette, Vincent Bours.
EPI_ISL_418625 hCoV-19/Belgium/ULG-6688/2020 Europe / Belgium / Liège 2020-03-14 Department of Clinical Microbiology GIGA Medical Genomics Keith Durkin, Maria Artesi, Sébastien Bontems, Raphaël Boreux, Cécile Meex, Pierrette Melin, Marie-Pierre Hayette, Vincent Bours.
EPI_ISL_418626 hCoV-19/Belgium/ULG-7242/2020 Europe / Belgium / Liège 2020-03-16 Department of Clinical Microbiology GIGA Medical Genomics Keith Durkin, Maria Artesi, Sébastien Bontems, Raphaël Boreux, Cécile Meex, Pierrette Melin, Marie-Pierre Hayette, Vincent Bours.
EPI_ISL_418627 hCoV-19/Belgium/ULG-6743/2020 Europe / Belgium / Liège 2020-03-14 Department of Clinical Microbiology GIGA Medical Genomics Keith Durkin, Maria Artesi, Sébastien Bontems, Raphaël Boreux, Cécile Meex, Pierrette Melin, Marie-Pierre Hayette, Vincent Bours.
EPI_ISL_418628 hCoV-19/Belgium/ULG-7134/2020 Europe / Belgium / Liège 2020-03-16 Department of Clinical Microbiology GIGA Medical Genomics Keith Durkin, Maria Artesi, Sébastien Bontems, Raphaël Boreux, Cécile Meex, Pierrette Melin, Marie-Pierre Hayette, Vincent Bours.
EPI_ISL_418629 hCoV-19/Belgium/ULG-7773/2020 Europe / Belgium / Liège 2020-03-17 Department of Clinical Microbiology GIGA Medical Genomics Keith Durkin, Maria Artesi, Sébastien Bontems, Raphaël Boreux, Cécile Meex, Pierrette Melin, Marie-Pierre Hayette, Vincent Bours.
EPI_ISL_418630 hCoV-19/Belgium/ULG-7729/2020 Europe / Belgium / Liège 2020-03-17 Department of Clinical Microbiology GIGA Medical Genomics Keith Durkin, Maria Artesi, Sébastien Bontems, Raphaël Boreux, Cécile Meex, Pierrette Melin, Marie-Pierre Hayette, Vincent Bours.

EPI_ISL_418631 hCoV-19/Belgium/ULG-6649/2020 Europe / Belgium / Liège 2020-03-14 Department of Clinical Microbiology GIGA Medical Genomics Keith Durkin, Maria Artesi, Sébastien Bontems, Raphaël Boreux, Cécile Meex, Pierrette Melin, Marie-Pierre Hayette, Vincent Bours.
EPI_ISL_418632 hCoV-19/Belgium/ULG-7500/2020 Europe / Belgium / Liège 2020-03-16 Department of Clinical Microbiology GIGA Medical Genomics Keith Durkin, Maria Artesi, Sébastien Bontems, Raphaël Boreux, Cécile Meex, Pierrette Melin, Marie-Pierre Hayette, Vincent Bours.
EPI_ISL_418633 hCoV-19/Belgium/ULG-7626/2020 Europe / Belgium / Liège 2020-03-17 Department of Clinical Microbiology GIGA Medical Genomics Keith Durkin, Maria Artesi, Sébastien Bontems, Raphaël Boreux, Cécile Meex, Pierrette Melin, Marie-Pierre Hayette, Vincent Bours.
EPI_ISL_418634 hCoV-19/Belgium/ULG-8595/2020 Europe / Belgium / Liège 2020-03-19 Department of Clinical Microbiology GIGA Medical Genomics Keith Durkin, Maria Artesi, Sébastien Bontems, Raphaël Boreux, Cécile Meex, Pierrette Melin, Marie-Pierre Hayette, Vincent Bours.
EPI_ISL_418635 hCoV-19/Belgium/ULG-8532/2020 Europe / Belgium / Liège 2020-03-19 Department of Clinical Microbiology GIGA Medical Genomics Keith Durkin, Maria Artesi, Sébastien Bontems, Raphaël Boreux, Cécile Meex, Pierrette Melin, Marie-Pierre Hayette, Vincent Bours.
EPI_ISL_418636 hCoV-19/Belgium/ULG-8541/2020 Europe / Belgium / Liège 2020-03-19 Department of Clinical Microbiology GIGA Medical Genomics Keith Durkin, Maria Artesi, Sébastien Bontems, Raphaël Boreux, Cécile Meex, Pierrette Melin, Marie-Pierre Hayette, Vincent Bours.
EPI_ISL_418638 hCoV-19/Belgium/ULG-8578/2020 Europe / Belgium / Liège 2020-03-19 Department of Clinical Microbiology GIGA Medical Genomics Keith Durkin, Maria Artesi, Sébastien Bontems, Raphaël Boreux, Cécile Meex, Pierrette Melin, Marie-Pierre Hayette, Vincent Bours.
EPI_ISL_418639 hCoV-19/Belgium/ULG-8617/2020 Europe / Belgium / Liège 2020-03-19 Department of Clinical Microbiology GIGA Medical Genomics Keith Durkin, Maria Artesi, Sébastien Bontems, Raphaël Boreux, Cécile Meex, Pierrette Melin, Marie-Pierre Hayette, Vincent Bours.
EPI_ISL_418640 hCoV-19/Belgium/ULG-8634/2020 Europe / Belgium / Liège 2020-03-19 Department of Clinical Microbiology GIGA Medical Genomics Keith Durkin, Maria Artesi, Sébastien Bontems, Raphaël Boreux, Cécile Meex, Pierrette Melin, Marie-Pierre Hayette, Vincent Bours.
EPI_ISL_418645 hCoV-19/Belgium/ULG-9558/2020 Europe / Belgium / Liège 2020-03-21 Department of Clinical Microbiology GIGA Medical Genomics Keith Durkin, Maria Artesi, Sébastien Bontems, Raphaël Boreux, Cécile Meex, Pierrette Melin, Marie-Pierre Hayette, Vincent Bours.
EPI_ISL_418646 hCoV-19/Belgium/ULG-9572/2020 Europe / Belgium / Liège 2020-03-21 Department of Clinical Microbiology GIGA Medical Genomics Keith Durkin, Maria Artesi, Sébastien Bontems, Raphaël Boreux, Cécile Meex, Pierrette Melin, Marie-Pierre Hayette, Vincent Bours.
EPI_ISL_418648 hCoV-19/Belgium/ULG-9618/2020 Europe / Belgium / Liège 2020-03-22 Department of Clinical Microbiology GIGA Medical Genomics Keith Durkin, Maria Artesi, Sébastien Bontems, Raphaël Boreux, Cécile Meex, Pierrette Melin, Marie-Pierre Hayette, Vincent Bours.
EPI_ISL_418649 hCoV-19/Belgium/ULG-9619/2020 Europe / Belgium / Liège 2020-03-22 Department of Clinical Microbiology GIGA Medical Genomics Keith Durkin, Maria Artesi, Sébastien Bontems, Raphaël Boreux, Cécile Meex, Pierrette Melin, Marie-Pierre Hayette, Vincent Bours.
EPI_ISL_418650 hCoV-19/Belgium/ULG-9620/2020 Europe / Belgium / Liège 2020-03-22 Department of Clinical Microbiology GIGA Medical Genomics Keith Durkin, Maria Artesi, Sébastien Bontems, Raphaël Boreux, Cécile Meex, Pierrette Melin, Marie-Pierre Hayette, Vincent Bours.
EPI_ISL_418651 hCoV-19/Belgium/ULG-9634/2020 Europe / Belgium / Liège 2020-03-22 Department of Clinical Microbiology GIGA Medical Genomics Keith Durkin, Maria Artesi, Sébastien Bontems, Raphaël Boreux, Cécile Meex, Pierrette Melin, Marie-Pierre Hayette, Vincent Bours.
EPI_ISL_418652 hCoV-19/Belgium/ULG-9641/2020 Europe / Belgium / Liège 2020-03-22 Department of Clinical Microbiology GIGA Medical Genomics Keith Durkin, Maria Artesi, Sébastien Bontems, Raphaël Boreux, Cécile Meex, Pierrette Melin, Marie-Pierre Hayette, Vincent Bours.
EPI_ISL_418653 hCoV-19/Belgium/ULG-9647/2020 Europe / Belgium / Liège 2020-03-22 Department of Clinical Microbiology GIGA Medical Genomics Keith Durkin, Maria Artesi, Sébastien Bontems, Raphaël Boreux, Cécile Meex, Pierrette Melin, Marie-Pierre Hayette, Vincent Bours.
EPI_ISL_418654 hCoV-19/Belgium/ULG-9694/2020 Europe / Belgium / Liège 2020-03-22 Department of Clinical Microbiology GIGA Medical Genomics Keith Durkin, Maria Artesi, Sébastien Bontems, Raphaël Boreux, Cécile Meex, Pierrette Melin, Marie-Pierre Hayette, Vincent Bours.
EPI_ISL_418655 hCoV-19/Belgium/ULG-9714/2020 Europe / Belgium / Liège 2020-03-22 Department of Clinical Microbiology GIGA Medical Genomics Keith Durkin, Maria Artesi, Sébastien Bontems, Raphaël Boreux, Cécile Meex, Pierrette Melin, Marie-Pierre Hayette, Vincent Bours.
EPI_ISL_418656 hCoV-19/Belgium/ULG-9715/2020 Europe / Belgium / Liège 2020-03-22 Department of Clinical Microbiology GIGA Medical Genomics Keith Durkin, Maria Artesi, Sébastien Bontems, Raphaël Boreux, Cécile Meex, Pierrette Melin, Marie-Pierre Hayette, Vincent Bours.
EPI_ISL_418657 hCoV-19/Belgium/ULG-9716/2020 Europe / Belgium / Liège 2020-03-22 Department of Clinical Microbiology GIGA Medical Genomics Keith Durkin, Maria Artesi, Sébastien Bontems, Raphaël Boreux, Cécile Meex, Pierrette Melin, Marie-Pierre Hayette, Vincent Bours.
EPI_ISL_418659 hCoV-19/Belgium/ULG-9725/2020 Europe / Belgium / Liège 2020-03-22 Department of Clinical Microbiology GIGA Medical Genomics Keith Durkin, Maria Artesi, Sébastien Bontems, Raphaël Boreux, Cécile Meex, Pierrette Melin, Marie-Pierre Hayette, Vincent Bours.
EPI_ISL_418660 hCoV-19/Belgium/ULG-9732/2020 Europe / Belgium / Liège 2020-03-22 Department of Clinical Microbiology GIGA Medical Genomics Keith Durkin, Maria Artesi, Sébastien Bontems, Raphaël Boreux, Cécile Meex, Pierrette Melin, Marie-Pierre Hayette, Vincent Bours.
EPI_ISL_418661 hCoV-19/Belgium/ULG-9735/2020 Europe / Belgium / Liège 2020-03-22 Department of

Clinical Microbiology GIGA Medical Genomics Keith Durkin, Maria Artesi, Sébastien Bontems, Raphaël Boreux, Cécile Meex, Pierrette Melin, Marie-Pierre Hayette, Vincent Bours.
EPI_ISL_418663 hCoV-19/Belgium/ULG-9739/2020 Europe / Belgium / Liège 2020-03-22 Department of Clinical Microbiology GIGA Medical Genomics Keith Durkin, Maria Artesi, Sébastien Bontems, Raphaël Boreux, Cécile Meex, Pierrette Melin, Marie-Pierre Hayette, Vincent Bours.
EPI_ISL_418664 hCoV-19/Belgium/ULG-9741/2020 Europe / Belgium / Liège 2020-03-22 Department of Clinical Microbiology GIGA Medical Genomics Keith Durkin, Maria Artesi, Sébastien Bontems, Raphaël Boreux, Cécile Meex, Pierrette Melin, Marie-Pierre Hayette, Vincent Bours.
EPI_ISL_418667 hCoV-19/England/20109047803/2020 Europe / United Kingdom / England 2020-03-07 Respiratory Virus Unit, Microbiology Services Colindale, Public Health England Respiratory Virus Unit, Microbiology Services Colindale, Public Health England Monica Galiano, Shahjahan Miah, Angie Lackenby, Omolola Akinbami, Tiina Talts, Leena Bhaw, Richard Myers, Steven Platt, Kirstin Edwards, Jonathan Hubb, Joanna Ellis, Maria Zambon
EPI_ISL_418668 hCoV-19/England/20110136302/2020 Europe / United Kingdom / England 2020-03-09 Respiratory Virus Unit, Microbiology Services Colindale, Public Health England Respiratory Virus Unit, Microbiology Services Colindale, Public Health England Monica Galiano, Shahjahan Miah, Angie Lackenby, Omolola Akinbami, Tiina Talts, Leena Bhaw, Richard Myers, Steven Platt, Kirstin Edwards, Jonathan Hubb, Joanna Ellis, Maria Zambon
EPI_ISL_418669 hCoV-19/England/20116022902/2020 Europe / United Kingdom / England 2020-03-11 Respiratory Virus Unit, Microbiology Services Colindale, Public Health England Respiratory Virus Unit, Microbiology Services Colindale, Public Health England Monica Galiano, Shahjahan Miah, Angie Lackenby, Omolola Akinbami, Tiina Talts, Leena Bhaw, Richard Myers, Steven Platt, Kirstin Edwards, Jonathan Hubb, Joanna Ellis, Maria Zambon
EPI_ISL_418670 hCoV-19/England/20116025603/2020 Europe / United Kingdom / England 2020-03-11 Respiratory Virus Unit, Microbiology Services Colindale, Public Health England Respiratory Virus Unit, Microbiology Services Colindale, Public Health England Monica Galiano, Shahjahan Miah, Angie Lackenby, Omolola Akinbami, Tiina Talts, Leena Bhaw, Richard Myers, Steven Platt, Kirstin Edwards, Jonathan Hubb, Joanna Ellis, Maria Zambon
EPI_ISL_418671 hCoV-19/England/20119026102/2020 Europe / United Kingdom / England 2020-03-13 Respiratory Virus Unit, Microbiology Services Colindale, Public Health England Respiratory Virus Unit, Microbiology Services Colindale, Public Health England Monica Galiano, Shahjahan Miah, Angie Lackenby, Omolola Akinbami, Tiina Talts, Leena Bhaw, Richard Myers, Steven Platt, Kirstin Edwards, Jonathan Hubb, Joanna Ellis, Maria Zambon
EPI_ISL_418672 hCoV-19/England/20120002102/2020 Europe / United Kingdom / England 2020-03-15 Respiratory Virus Unit, Microbiology Services Colindale, Public Health England Respiratory Virus Unit, Microbiology Services Colindale, Public Health England Monica Galiano, Shahjahan Miah, Angie Lackenby, Omolola Akinbami, Tiina Talts, Leena Bhaw, Richard Myers, Steven Platt, Kirstin Edwards, Jonathan Hubb, Joanna Ellis, Maria Zambon
EPI_ISL_418673 hCoV-19/England/20122032802/2020 Europe / United Kingdom / England 2020-03-16 Respiratory Virus Unit, Microbiology Services Colindale, Public Health England Respiratory Virus Unit, Microbiology Services Colindale, Public Health England Monica Galiano, Shahjahan Miah, Angie Lackenby, Omolola Akinbami, Tiina Talts, Leena Bhaw, Richard Myers, Steven Platt, Kirstin Edwards, Jonathan Hubb, Joanna Ellis, Maria Zambon
EPI_ISL_418675 hCoV-19/Engiand/20122073102/2020 Europe / United Kingdom / England 2020-03-16 Respiratory Virus Unit, Microbiology Services Colindale, Public Health England Respiratory Virus Unit, Microbiology Services Colindale, Public Health England Monica Galiano, Shahjahan Miah, Angie Lackenby, Omolola Akinbami, Tiina Talts, Leena Bhaw, Richard Myers, Steven Platt, Kirstin Edwards, Jonathan Hubb, Joanna Ellis, Maria Zambon
EPI_ISL_418676 hCoV-19/England/20122074602/2020 Europe / United Kingdom / England 2020-03-10 Respiratory Virus Unit, Microbiology Services Colindale, Public Health England Respiratory Virus Unit, Microbiology Services Colindale, Public Health England Monica Galiano, Shahjahan Miah, Angie Lackenby, Omolola Akinbami, Tiina Talts, Leena Bhaw, Richard Myers, Steven Platt, Kirstin Edwards, Jonathan Hubb, Joanna Ellis, Maria Zambon
EPI_ISL_418677 hCoV-19/England/20122087702/2020 Europe / United Kingdom / England 2020-03-15 Respiratory Virus Unit, Microbiology Services Colindale, Public Health England Respiratory Virus Unit, Microbiology Services Colindale, Public Health England Monica Galiano, Shahjahan Miah, Angie Lackenby, Omolola Akinbami, Tiina Talts, Leena Bhaw, Richard Myers, Steven Platt, Kirstin Edwards, Jonathan Hubb, Joanna Ellis, Maria Zambon
EPI_ISL_418678 hCoV-19/England/20122088102/2020 Europe / United Kingdom / England 2020-03-16 Respiratory Virus Unit, Microbiology Services Colindale, Public Health England Respiratory Virus Unit, Microbiology Services Colindale, Public Health England Monica Galiano, Shahjahan Miah, Angie Lackenby, Omolola Akinbami, Tiina Talts, Leena Bhaw, Richard Myers, Steven Platt, Kirstin Edwards, Jonathan Hubb, Joanna Ellis, Maria Zambon
EPI_ISL_418679 hCoV-19/England/20122119202/2020 Europe / United Kingdom / England 2020-03-17 Respiratory Virus Unit, Microbiology Services Colindale, Public Health England Respiratory Virus Unit, Microbiology Services Colindale, Public Health England Monica Galiano, Shahjahan Miah, Angie Lackenby, Omolola Akinbami, Tiina Talts, Leena Bhaw, Richard Myers, Steven Platt, Kirstin Edwards, Jonathan Hubb, Joanna Ellis, Maria Zambon
EPI_ISL_418680 hCoV-19/England/20122119302/2020 Europe / United Kingdom / England 2020-03-17 Respiratory Virus Unit, Microbiology Services Colindale, Public Health England Respiratory Virus Unit,

Microbiology Services Colindale, Public Health England Monica Galiano, Shahjahan Miah, Angie Lackenby, Omolola Akinbami, Tiina Talts, Leena Bhaw, Richard Myers, Steven Platt, Kirstin Edwards, Jonathan Hubb, Joanna Ellis, Maria Zambon
EPI_ISL_418681 hCoV-19/England/20122119502/2020 Europe / United Kingdom / England 2020-03-17 Respiratory Virus Unit, Microbiology Services Colindale, Public Health England Respiratory Virus Unit, Microbiology Services Colindale, Public Health England Monica Galiano, Shahjahan Miah, Angie Lackenby, Omolola Akinbami, Tiina Talts, Leena Bhaw, Richard Myers, Steven Platt, Kirstin Edwards, Jonathan Hubb, Joanna Ellis, Maria Zambon
EPI_ISL_418684 hCoV-19/England/20124001302/2020 Europe / United Kingdom / England 2020-03-17 Respiratory Virus Unit, Microbiology Services Colindale, Public Health England Respiratory Virus Unit, Microbiology Services Colindale, Public Health England Monica Galiano, Shahjahan Miah, Angie Lackenby, Omolola Akinbami, Tiina Talts, Leena Bhaw, Richard Myers, Steven Platt, Kirstin Edwards, Jonathan Hubb, Joanna Ellis, Maria Zambon
EPI_ISL_418685 hCoV-19/England/20124001702/2020 Europe / United Kingdom / England 2020-03-17 Respiratory Virus Unit, Microbiology Services Colindale, Public Health England Respiratory Virus Unit, Microbiology Services Colindale, Public Health England Monica Galiano, Shahjahan Miah, Angie Lackenby, Omolola Akinbami, Tiina Talts, Leena Bhaw, Richard Myers, Steven Platt, Kirstin Edwards, Jonathan Hubb, Joanna Ellis, Maria Zambon
EPI_ISL_418686 hCoV-19/England/20124001902/2020 Europe / United Kingdom / England 2020-03-17 Respiratory Virus Unit, Microbiology Services Colindale, Public Health England Respiratory Virus Unit, Microbiology Services Colindale, Public Health England Monica Galiano, Shahjahan Miah, Angie Lackenby, Omolola Akinbami, Tiina Talts, Leena Bhaw, Richard Myers, Steven Platt, Kirstin Edwards, Jonathan Hubb, Joanna Ellis, Maria Zambon
EPI_ISL_418687 hCoV-19/England/20124002002/2020 Europe / United Kingdom / England 2020-03-17 Respiratory Virus Unit, Microbiology Services Colindale, Public Health England Respiratory Virus Unit, Microbiology Services Colindale, Public Health England Monica Galiano, Shahjahan Miah, Angie Lackenby, Omolola Akinbami, Tiina Talts, Leena Bhaw, Richard Myers, Steven Platt, Kirstin Edwards, Jonathan Hubb, Joanna Ellis, Maria Zambon
EPI_ISL_418688 hCoV-19/England/20124002102/2020 Europe / United Kingdom / England 2020-03-17 Respiratory Virus Unit, Microbiology Services Colindale, Public Health England Respiratory Virus Unit, Microbiology Services Colindale, Public Health England Monica Galiano, Shahjahan Miah, Angie Lackenby, Omolola Akinbami, Tiina Talts, Leena Bhaw, Richard Myers, Steven Platt, Kirstin Edwards, Jonathan Hubb, Joanna Ellis, Maria Zambon
EPI_ISL_418690 hCoV-19/England/20124003702/2020 Europe / United Kingdom / England 2020-03-17 Respiratory Virus Unit, Microbiology Services Colindale, Public Health England Respiratory Virus Unit, Microbiology Services Colindale, Public Health England Monica Galiano, Shahjahan Miah, Angie Lackenby, Omolola Akinbami, Tiina Talts, Leena Bhaw, Richard Myers, Steven Platt, Kirstin Edwards, Jonathan Hubb, Joanna Ellis, Maria Zambon
EPI_ISL_418691 hCoV-19/England/20124003902/2020 Europe / United Kingdom / England 2020-03-17 Respiratory Virus Unit, Microbiology Services Colindale, Public Health England Respiratory Virus Unit, Microbiology Services Colindale, Public Health England Monica Galiano, Shahjahan Miah, Angie Lackenby, Omolola Akinbami, Tiina Talts, Leena Bhaw, Richard Myers, Steven Platt, Kirstin Edwards, Jonathan Hubb, Joanna Ellis, Maria Zambon
EPI_ISL_418692 hCoV-19/England/20124004302/2020 Europe / United Kingdom / England 2020-03-17 Respiratory Virus Unit, Microbiology Services Colindale, Public Health England Respiratory Virus Unit, Microbiology Services Colindale, Public Health England Monica Galiano, Shahjahan Miah, Angie Lackenby, Omolola Akinbami, Tiina Talts, Leena Bhaw, Richard Myers, Steven Platt, Kirstin Edwards, Jonathan Hubb, Joanna Ellis, Maria Zambon
EPI_ISL_418694 hCoV-19/England/20124008902/2020 Europe / United Kingdom / England 2020-03-16 Respiratory Virus Unit, Microbiology Services Colindale, Public Health England Respiratory Virus Unit, Microbiology Services Colindale, Public Health England Monica Galiano, Shahjahan Miah, Angie Lackenby, Omolola Akinbami, Tiina Talts, Leena Bhaw, Richard Myers, Steven Platt, Kirstin Edwards, Jonathan Hubb, Joanna Ellis, Maria Zambon
EPI_ISL_418696 hCoV-19/England/20124009602/2020 Europe / United Kingdom / England 2020-03-18 Respiratory Virus Unit, Microbiology Services Colindale, Public Health England Respiratory Virus Unit, Microbiology Services Colindale, Public Health England Monica Galiano, Shahjahan Miah, Angie Lackenby, Omolola Akinbami, Tiina Talts, Leena Bhaw, Richard Myers, Steven Platt, Kirstin Edwards, Jonathan Hubb, Joanna Ellis, Maria Zambon
EPI_ISL_418698 hCoV-19/England/20124012702/2020 Europe / United Kingdom / England 2020-03-17 Respiratory Virus Unit, Microbiology Services Colindale, Public Health England Respiratory Virus Unit, Microbiology Services Colindale, Public Health England Monica Galiano, Shahjahan Miah, Angie Lackenby, Omolola Akinbami, Tiina Talts, Leena Bhaw, Richard Myers, Steven Platt, Kirstin Edwards, Jonathan Hubb, Joanna Ellis, Maria Zambon
EPI_ISL_418709 hCoV-19/England/20124034002/2020 Europe / United Kingdom / England 2020-03-16 Respiratory Virus Unit, Microbiology Services Colindale, Public Health England Respiratory Virus Unit, Microbiology Services Colindale, Public Health England Monica Galiano, Shahjahan Miah, Angie Lackenby, Omolola Akinbami, Tiina Talts, Leena Bhaw, Richard Myers, Steven Platt, Kirstin Edwards, Jonathan Hubb, Joanna Ellis, Maria Zambon
EPI_ISL_418711 hCoV-19/England/20124034702/2020 Europe / United Kingdom / England 2020-03-16 Respiratory Virus Unit, Microbiology Services Colindale, Public Health England Respiratory Virus Unit,

Microbiology Services Colindale, Public Health England Monica Galiano, Shahjahan Miah, Angie Lackenby, Omolola Akinbami, Tiina Talts, Leena Bhaw, Richard Myers, Steven Platt, Kirstin Edwards, Jonathan Hubb, Joanna Ellis, Maria Zambon
EPI_ISL_418715 hCoV-19/England/20124035802/2020 Europe / United Kingdom / England 2020-03-17 Respiratory Virus Unit, Microbiology Services Colindale, Public Health England Respiratory Virus Unit, Microbiology Services Colindale, Public Health England Monica Galiano, Shahjahan Miah, Angie Lackenby, Omolola Akinbami, Tiina Talts, Leena Bhaw, Richard Myers, Steven Platt, Kirstin Edwards, Jonathan Hubb, Joanna Ellis, Maria Zambon
EPI_ISL_418716 hCoV-19/England/20124036002/2020 Europe / United Kingdom / England 2020-03-17 Respiratory Virus Unit, Microbiology Services Colindale, Public Health England Respiratory Virus Unit, Microbiology Services Colindale, Public Health England Monica Galiano, Shahjahan Miah, Angie Lackenby, Omolola Akinbami, Tiina Talts, Leena Bhaw, Richard Myers, Steven Platt, Kirstin Edwards, Jonathan Hubb, Joanna Ellis, Maria Zambon
EPI_ISL_418739 hCoV-19/England/20124101102/2020 Europe / United Kingdom / England 2020-03-18 Respiratory Virus Unit, Microbiology Services Colindale, Public Health England Respiratory Virus Unit, Microbiology Services Colindale, Public Health England Monica Galiano, Shahjahan Miah, Angie Lackenby, Omolola Akinbami, Tiina Talts, Leena Bhaw, Richard Myers, Steven Platt, Kirstin Edwards, Jonathan Hubb, Joanna Ellis, Maria Zambon
EPI_ISL_418749 hCoV-19/England/20126006802/2020 Europe / United Kingdom / England 2020-03-18 Respiratory Virus Unit, Microbiology Services Colindale, Public Health England Respiratory Virus Unit, Microbiology Services Colindale, Public Health England Monica Galiano, Shahjahan Miah, Angie Lackenby, Omolola Akinbami, Tiina Talts, Leena Bhaw, Richard Myers, Steven Platt, Kirstin Edwards, Jonathan Hubb, Joanna Ellis, Maria Zambon
EPI_ISL_418751 hCoV-19/England/20126007102/2020 Europe / United Kingdom / England 2020-03-18 Respiratory Virus Unit, Microbiology Services Colindale, Public Health England Respiratory Virus Unit, Microbiology Services Colindale, Public Health England Monica Galiano, Shahjahan Miah, Angie Lackenby, Omolola Akinbami, Tiina Talts, Leena Bhaw, Richard Myers, Steven Platt, Kirstin Edwards, Jonathan Hubb, Joanna Ellis, Maria Zambon
EPI_ISL_418756 hCoV-19/England/20126012702/2020 Europe / United Kingdom / England 2020-03-17 Respiratory Virus Unit, Microbiology Services Colindale, Public Health England Respiratory Virus Unit, Microbiology Services Colindale, Public Health England Monica Galiano, Shahjahan Miah, Angie Lackenby, Omolola Akinbami, Tiina Talts, Leena Bhaw, Richard Myers, Steven Platt, Kirstin Edwards, Jonathan Hubb, Joanna Ellis, Maria Zambon
EPI_ISL_418764 hCoV-19/England/20126035502/2020 Europe / United Kingdom / England 2020-03-18 Respiratory Virus Unit, Microbiology Services Colindale, Public Health England Respiratory Virus Unit, Microbiology Services Colindale, Public Health England Monica Galiano, Shahjahan Miah, Angie Lackenby, Omolola Akinbami, Tiina Talts, Leena Bhaw, Richard Myers, Steven Platt, Kirstin Edwards, Jonathan Hubb, Joanna Ellis, Maria Zambon
EPI_ISL_418770 hCoV-19/England/20126096602/2020 Europe / United Kingdom / England 2020-03-18 Respiratory Virus Unit, Microbiology Services Colindale, Public Health England Respiratory Virus Unit, Microbiology Services Colindale, Public Health England Monica Galiano, Shahjahan Miah, Angie Lackenby, Omolola Akinbami, Tiina Talts, Leena Bhaw, Richard Myers, Steven Platt, Kirstin Edwards, Jonathan Hubb, Joanna Ellis, Maria Zambon
EPI_ISL_418771 hCoV-19/USA/WA-NH2/2020 North America / USA / Washington 2020-03-13 WA State Department of Health Pathogen Discovery, Respiratory Viruses Branch, Division of Viral Diseases, Centers for Disease Control and Prevention Jing Zhang, Ying Tao, Clinton R. Paden, Krista Queen, Anna Uehara, Yan Li, Haibin Wang, Jesica Jacobs, Denny Russell, Brian Hiatt, Jessica Gant, Suxiang Tong
EPI_ISL_418772 hCoV-19/USA/WA-NH3/2020 North America / USA / Washington 2020-03-13 WA State Department of Health Pathogen Discovery, Respiratory Viruses Branch, Division of Viral Diseases, Centers for Disease Control and Prevention Jing Zhang, Ying Tao, Clinton R. Paden, Krista Queen, Anna Uehara, Yan Li, Haibin Wang, Jesica Jacobs, Denny Russell, Brian Hiatt, Jessica Gant, Suxiang Tong
EPI_ISL_418773 hCoV-19/USA/WA-NH4/2020 North America / USA / Washington 2020-03-13 WA State Department of Health Pathogen Discovery, Respiratory Viruses Branch, Division of Viral Diseases, Centers for Disease Control and Prevention Jing Zhang, Ying Tao, Clinton R. Paden, Krista Queen, Anna Uehara, Yan Li, Haibin Wang, Jesica Jacobs, Denny Russell, Brian Hiatt, Jessica Gant, Suxiang Tong
EPI_ISL_418774 hCoV-19/USA/WA-NH5/2020 North America / USA / Washington 2020-03-13 WA State Department of Health Pathogen Discovery, Respiratory Viruses Branch, Division of Viral Diseases, Centers for Disease Control and Prevention Jing Zhang, Ying Tao, Clinton R. Paden, Krista Queen, Anna Uehara, Yan Li, Haibin Wang, Jesica Jacobs, Denny Russell, Brian Hiatt, Jessica Gant, Suxiang Tong
EPI_ISL_418775 hCoV-19/USA/WA-NH6/2020 North America / USA / Washington 2020-03-13 WA State Department of Health Pathogen Discovery, Respiratory Viruses Branch, Division of Viral Diseases, Centers for Disease Control and Prevention Jing Zhang, Ying Tao, Clinton R. Paden, Krista Queen, Anna Uehara, Yan Li, Haibin Wang, Jesica Jacobs, Denny Russell, Brian Hiatt, Jessica Gant, Suxiang Tong
EPI_ISL_418776 hCoV-19/USA/WA-NH7/2020 North America / USA / Washington 2020-03-13 WA State Department of Health Pathogen Discovery, Respiratory Viruses Branch, Division of Viral Diseases, Centers for Disease Control and Prevention Jing Zhang, Ying Tao, Clinton R. Paden, Krista Queen, Anna Uehara, Yan Li, Haibin Wang, Jesica Jacobs, Denny Russell, Brian Hiatt, Jessica Gant, Suxiang Tong
EPI_ISL_418777 hCoV-19/USA/WA-NH8/2020 North America / USA / Washington 2020-03-13 WA State Department of Health Pathogen Discovery, Respiratory Viruses Branch, Division of Viral Diseases, Centers for Disease Control and Prevention Jing Zhang, Ying Tao, Clinton R. Paden, Krista Queen, Anna Uehara, Yan Li,

Haibin Wang, Jesica Jacobs, Denny Russell, Brian Hiatt, Jessica Gant, Suxiang Tong
EPI_ISL_418778 hCoV-19/USA/WA-NH9/2020 North America / USA / Washington 2020-03-13 WA State Department of Health Pathogen Discovery, Respiratory Viruses Branch, Division of Viral Diseases, Centers for Disease Control and Prevention Ying Tao, Jing Zhang, Clinton R. Paden, Krista Queen, Anna Uehara, Yan Li, Haibin Wang, Jesica Jacobs, Denny Russell, Brian Hiatt, Jessica Gant, Suxiang Tong
EPI_ISL_418779 hCoV-19/USA/WA-NH10/2020 North America / USA / Washington 2020-03-13 WA State Department of Health Pathogen Discovery, Respiratory Viruses Branch, Division of Viral Diseases, Centers for Disease Control and Prevention Ying Tao, Jing Zhang, Clinton R. Paden, Krista Queen, Anna Uehara, Yan Li, Haibin Wang, Jesica Jacobs, Denny Russell, Brian Hiatt, Jessica Gant, Suxiang Tong
EPI_ISL_418780 hCoV-19/USA/WA-NH11/2020 North America / USA / Washington 2020-03-13 WA State Department of Health Pathogen Discovery, Respiratory Viruses Branch, Division of Viral Diseases, Centers for Disease Control and Prevention Ying Tao, Jing Zhang, Clinton R. Paden, Krista Queen, Anna Uehara, Yan Li, Haibin Wang, Jesica Jacobs, Denny Russell, Brian Hiatt, Jessica Gant, Suxiang Tong
EPI_ISL_418781 hCoV-19/USA/WA-NH12/2020 North America / USA / Washington 2020-03-13 WA State Department of Health Pathogen Discovery, Respiratory Viruses Branch, Division of Viral Diseases, Centers for Disease Control and Prevention Ying Tao, Jing Zhang, Clinton R. Paden, Krista Queen, Anna Uehara, Yan Li, Haibin Wang, Jesica Jacobs, Denny Russell, Brian Hiatt, Jessica Gant, Suxiang Tong
EPI_ISL_418782 hCoV-19/USA/WA-NH13/2020 North America / USA / Washington 2020-03-13 WA State Department of Health Pathogen Discovery, Respiratory Viruses Branch, Division of Viral Diseases, Centers for Disease Control and Prevention Ying Tao, Jing Zhang, Clinton R. Paden, Krista Queen, Anna Uehara, Yan Li, Haibin Wang, Jesica Jacobs, Denny Russell, Brian Hiatt, Jessica Gant, Suxiang Tong
EPI_ISL_418783 hCoV-19/USA/WA-NH14/2020 North America / USA / Washington 2020-03-13 WA State Department of Health Pathogen Discovery, Respiratory Viruses Branch, Division of Viral Diseases, Centers for Disease Control and Prevention Ying Tao, Jing Zhang, Clinton R. Paden, Krista Queen, Anna Uehara, Yan Li, Haibin Wang, Jesica Jacobs, Denny Russell, Brian Hiatt, Jessica Gant, Suxiang Tong
EPI_ISL_418784 hCoV-19/USA/WA-NH17/2020 North America / USA / Washington 2020-03-13 WA State Department of Health Pathogen Discovery, Respiratory Viruses Branch, Division of Viral Diseases, Centers for Disease Control and Prevention Ying Tao, Jing Zhang, Clinton R. Paden, Krista Queen, Anna Uehara, Yan Li, Haibin Wang, Jesica Jacobs, Denny Russell, Brian Hiatt, Jessica Gant, Suxiang Tong
EPI_ISL_418785 hCoV-19/USA/WA-NH18/2020 North America / USA / Washington 2020-03-13 WA State Department of Health Pathogen Discovery, Respiratory Viruses Branch, Division of Viral Diseases, Centers for Disease Control and Prevention Ying Tao, Jing Zhang, Clinton R. Paden, Krista Queen, Anna Uehara, Yan Li, Haibin Wang, Jesica Jacobs, Denny Russell, Brian Hiatt, Jessica Gant, Suxiang Tong
EPI_ISL_418786 hCoV-19/USA/WA-NH19/2020 North America / USA / Washington 2020-03-13 WA State Department of Health Pathogen Discovery, Respiratory Viruses Branch, Division of Viral Diseases, Centers for Disease Control and Prevention Ying Tao, Jing Zhang, Clinton R. Paden, Krista Queen, Anna Uehara, Yan Li, Haibin Wang, Jesica Jacobs, Denny Russell, Brian Hiatt, Jessica Gant, Suxiang Tong
EPI_ISL_418787 hCoV-19/USA/WA-NH20/2020 North America / USA / Washington 2020-03-13 WA State Department of Health Pathogen Discovery, Respiratory Viruses Branch, Division of Viral Diseases, Centers for Disease Control and Prevention Ying Tao, Jing Zhang, Clinton R. Paden, Krista Queen, Anna Uehara, Yan Li, Haibin Wang, Jesica Jacobs, Denny Russell, Brian Hiatt, Jessica Gant, Suxiang Tong
EPI_ISL_418788 hCoV-19/USA/WA-NH21/2020 North America / USA / Washington 2020-03-13 WA State Department of Health Pathogen Discovery, Respiratory Viruses Branch, Division of Viral Diseases, Centers for Disease Control and Prevention Ying Tao, Jing Zhang, Clinton R. Paden, Krista Queen, Anna Uehara, Yan Li, Haibin Wang, Jesica Jacobs, Denny Russell, Brian Hiatt, Jessica Gant, Suxiang Tong
EPI_ISL_418789 hCoV-19/USA/WA-NH22/2020 North America / USA / Washington 2020-03-13 WA State Department of Health Pathogen Discovery, Respiratory Viruses Branch, Division of Viral Diseases, Centers for Disease Control and Prevention Ying Tao, Jing Zhang, Clinton R. Paden, Krista Queen, Anna Uehara, Yan Li, Haibin Wang, Jesica Jacobs, Denny Russell, Brian Hiatt, Jessica Gant, Suxiang Tong
EPI_ISL_418790 hCoV-19/USA/WA-NH23/2020 North America / USA / Washington 2020-03-13 WA State Department of Health Pathogen Discovery, Respiratory Viruses Branch, Division of Viral Diseases, Centers for Disease Control and Prevention Ying Tao, Jing Zhang, Clinton R. Paden, Krista Queen, Anna Uehara, Yan Li, Haibin Wang, Jesica Jacobs, Denny Russell, Brian Hiatt, Jessica Gant, Suxiang Tong
EPI_ISL_418791 hCoV-19/USA/WA-NH24/2020 North America / USA / Washington 2020-03-13 WA State Department of Health Pathogen Discovery, Respiratory Viruses Branch, Division of Viral Diseases, Centers for Disease Control and Prevention Ying Tao, Jing Zhang, Clinton R. Paden, Krista Queen, Anna Uehara, Yan Li, Haibin Wang, Jesica Jacobs, Denny Russell, Brian Hiatt, Jessica Gant, Suxiang Tong
EPI_ISL_418792 hCoV-19/Belgium/GL-030546/2020 Europe / Belgium / Houthalen-Helchteren 2020-03-05 KU Leuven, Clinical and Epidemiological Virology KU Leuven, Clinical and Epidemiological Virology Bert Vanmechelen, Tony Wawina, Joan Marti-Carreras, Piet Maes
EPI_ISL_418793 hCoV-19/Belgium/SS-031047/2020 Europe / Belgium / Zonhoven 2020-03-10 KU Leuven, Clinical and Epidemiological Virology KU Leuven, Clinical and Epidemiological Virology Bert Vanmechelen, Tony Wawina, Joan Marti-Carreras, Piet Maes
EPI_ISL_418794 hCoV-19/Belgium/VHRA-030644/2020 Europe / Belgium / Nevele 2020-03-06 KU Leuven, Clinical and Epidemiological Virology KU Leuven, Clinical and Epidemiological Virology Bert Vanmechelen, Joan Marti-Carreras, Tony Wawina, Piet Maes
EPI_ISL_418795 hCoV-19/Belgium/NKR-030645/2020 Europe / Belgium / Ganshoren 2020-03-06 KU Leuven, Clinical and Epidemiological Virology KU Leuven, Clinical and Epidemiological Virology Bert Vanmechelen, Joan Marti-Carreras, Tony Wawina, Piet Maes
EPI_ISL_418796 hCoV-19/Belgium/VDLH-030548/2020 Europe / Belgium / Kalmthout 2020-03-05 KU

Leuven, Clinical and Epidemiological Virology   KU Leuven, Clinical and Epidemiological Virology   Bert Vanmechelen, Joan Marti-Carreras, Tony Wawina, Piet Maes
EPI_ISL_418798 hCoV-19/Belgium/GS-030549/2020 Europe / Belgium / Chaineux   2020-03-05   KU Leuven, Clinical and Epidemiological Virology   KU Leuven, Clinical and Epidemiological Virology   Bert Vanmechelen, Joan Marti-Carreras, Tony Wawina, Piet Maes
EPI_ISL_418799 hCoV-19/Australia/QLDID922/2020 Oceania / Australia / Queensland / Brisbane   2020-02-28   Mater Pathology Public Health Virology Laboratory   Bixing Huang, Alyssa Pyke, Amanda De Jong, Andrew Van Den Hurk, Carmel Taylor, David Warrilow, Doris Genge, Elisabeth Gamez, Glen Hewitson, Ian Maxwell Mackay, Inga Sultana, Jamie McMahon, Jean Barcelon, Judy Northill, Mitchell Finger, Natalie Simpson, Neelima Nair, Peter Burtonclay, Peter Moore, Sarah Wheatley, Sean Moody, Sonja Hall-Mendelin, Timothy Gardam, and Frederick Moore
EPI_ISL_419900 hCoV-19/Australia/VIC205/2020   Oceania / Australia / Victoria 2020-03-19   Victorian Infectious Diseases Reference Laboratory (VIDRL)   Victorian Infectious Diseases Reference Laboratory and Microbiological Diagnostic Unit Public Health Laboratory, Doherty Institute   Caly L., Seemann T., Sait, M., Schultz M., Druce J., Sherry, N.
EPI_ISL_419901 hCoV-19/Australia/VIC206/2020   Oceania / Australia / Victoria 2020-03-19   Victorian Infectious Diseases Reference Laboratory (VIDRL)   Victorian Infectious Diseases Reference Laboratory and Microbiological Diagnostic Unit Public Health Laboratory, Doherty Institute   Caly L., Seemann T., Sait, M., Schultz M., Druce J., Sherry, N.
EPI_ISL_419902 hCoV-19/Australia/VIC207/2020   Oceania / Australia / Victoria 2020-03-19   Victorian Infectious Diseases Reference Laboratory (VIDRL)   Victorian Infectious Diseases Reference Laboratory and Microbiological Diagnostic Unit Public Health Laboratory, Doherty Institute   Caly L., Seemann T., Sait, M., Schultz M., Druce J., Sherry, N.
EPI_ISL_419903 hCoV-19/Australia/VIC208/2020   Oceania / Australia / Victoria 2020-03-20   Victorian Infectious Diseases Reference Laboratory (VIDRL)   Victorian Infectious Diseases Reference Laboratory and Microbiological Diagnostic Unit Public Health Laboratory, Doherty Institute   Caly L., Seemann T., Sait, M., Schultz M., Druce J., Sherry, N.
EPI_ISL_419904 hCoV-19/Australia/VIC209/2020   Oceania / Australia / Victoria 2020-03-20   Victorian Infectious Diseases Reference Laboratory (VIDRL)   Victorian Infectious Diseases Reference Laboratory and Microbiological Diagnostic Unit Public Health Laboratory, Doherty Institute   Caly L., Seemann T., Sait, M., Schultz M., Druce J., Sherry, N.
EPI_ISL_419905 hCoV-19/Australia/VIC210/2020   Oceania / Australia / Victoria 2020-03-20   Victorian Infectious Diseases Reference Laboratory (VIDRL)   Victorian Infectious Diseases Reference Laboratory and Microbiological Diagnostic Unit Public Health Laboratory, Doherty Institute   Caly L., Seemann T., Sait, M., Schultz M., Druce J., Sherry, N.
EPI_ISL_419906 hCoV-19/Australia/VIC211/2020   Oceania / Australia / Victoria 2020-03-20   Victorian Infectious Diseases Reference Laboratory (VIDRL)   Victorian Infectious Diseases Reference Laboratory and Microbiological Diagnostic Unit Public Health Laboratory, Doherty Institute   Caly L., Seemann T., Sait, M., Schultz M., Druce J., Sherry, N.
EPI_ISL_419907 hCoV-19/Australia/VIC212/2020   Oceania / Australia / Victoria 2020-03-20   Victorian Infectious Diseases Reference Laboratory (VIDRL)   Victorian Infectious Diseases Reference Laboratory and Microbiological Diagnostic Unit Public Health Laboratory, Doherty Institute   Caly L., Seemann T., Sait, M., Schultz M., Druce J., Sherry, N.
EPI_ISL_419908 hCoV-19/Australia/VIC213/2020   Oceania / Australia / Victoria 2020-03-20   Victorian Infectious Diseases Reference Laboratory (VIDRL)   Victorian Infectious Diseases Reference Laboratory and Microbiological Diagnostic Unit Public Health Laboratory, Doherty Institute   Caly L., Seemann T., Sait, M., Schultz M., Druce J., Sherry, N.
EPI_ISL_419909 hCoV-19/Australia/VIC214/2020   Oceania / Australia / Victoria 2020-03-20   Victorian Infectious Diseases Reference Laboratory (VIDRL)   Victorian Infectious Diseases Reference Laboratory and Microbiological Diagnostic Unit Public Health Laboratory, Doherty Institute   Caly L., Seemann T., Sait, M., Schultz M., Druce J., Sherry, N.
EPI_ISL_419910 hCoV-19/Australia/VIC215/2020   Oceania / Australia / Victoria 2020-03-20   Victorian Infectious Diseases Reference Laboratory (VIDRL)   Victorian Infectious Diseases Reference Laboratory and Microbiological Diagnostic Unit Public Health Laboratory, Doherty Institute   Caly L., Seemann T., Sait, M., Schultz M., Druce J., Sherry, N.
EPI_ISL_419911 hCoV-19/Australia/VIC216/2020   Oceania / Australia / Victoria 2020-03-20   Victorian Infectious Diseases Reference Laboratory (VIDRL)   Victorian Infectious Diseases Reference Laboratory and Microbiological Diagnostic Unit Public Health Laboratory, Doherty Institute   Caly L., Seemann T., Sait, M., Schultz M., Druce J., Sherry, N.
EPI_ISL_419912 hCoV-19/Australia/VIC217/2020   Oceania / Australia / Victoria 2020-03-20   Victorian Infectious Diseases Reference Laboratory (VIDRL)   Victorian Infectious Diseases Reference Laboratory and Microbiological Diagnostic Unit Public Health Laboratory, Doherty Institute   Caly L., Seemann T., Sait, M., Schultz M., Druce J., Sherry, N.
EPI_ISL_419913 hCoV-19/Australia/VIC218/2020   Oceania / Australia / Victoria 2020-03-20   Victorian Infectious Diseases Reference Laboratory (VIDRL)   Victorian Infectious Diseases Reference Laboratory and Microbiological Diagnostic Unit Public Health Laboratory, Doherty Institute   Caly L., Seemann T., Sait, M., Schultz M., Druce J., Sherry, N.
EPI_ISL_419914 hCoV-19/Australia/VIC219/2020   Oceania / Australia / Victoria 2020-03-20   Victorian Infectious Diseases Reference Laboratory (VIDRL)   Victorian Infectious Diseases Reference Laboratory and Microbiological Diagnostic Unit Public Health Laboratory, Doherty Institute   Caly L., Seemann T., Sait, M., Schultz M., Druce J., Sherry, N.

EPI_ISL_419915 hCoV-19/Australia/VIC220/2020 Oceania / Australia / Victoria 2020-03-20 Victorian Infectious Diseases Reference Laboratory (VIDRL) Victorian Infectious Diseases Reference Laboratory and Microbiological Diagnostic Unit Public Health Laboratory, Doherty Institute Caly L., Seemann T., Sait, M., Schultz M., Druce J., Sherry, N.
EPI_ISL_419916 hCoV-19/Australia/VIC221/2020 Oceania / Australia / Victoria 2020-03-20 Victorian Infectious Diseases Reference Laboratory (VIDRL) Victorian Infectious Diseases Reference Laboratory and Microbiological Diagnostic Unit Public Health Laboratory, Doherty Institute Caly L., Seemann T., Sait, M., Schultz M., Druce J., Sherry, N.
EPI_ISL_419917 hCoV-19/Australia/VIC222/2020 Oceania / Australia / Victoria 2020-03-20 Victorian Infectious Diseases Reference Laboratory (VIDRL) Victorian Infectious Diseases Reference Laboratory and Microbiological Diagnostic Unit Public Health Laboratory, Doherty Institute Caly L., Seemann T., Sait, M., Schultz M., Druce J., Sherry, N.
EPI_ISL_419918 hCoV-19/Australia/VIC223/2020 Oceania / Australia / Victoria 2020-03-20 Victorian Infectious Diseases Reference Laboratory (VIDRL) Victorian Infectious Diseases Reference Laboratory and Microbiological Diagnostic Unit Public Health Laboratory, Doherty Institute Caly L., Seemann T., Sait, M., Schultz M., Druce J., Sherry, N.
EPI_ISL_419919 hCoV-19/Australia/VIC224/2020 Oceania / Australia / Victoria 2020-03-20 Victorian Infectious Diseases Reference Laboratory (VIDRL) Victorian Infectious Diseases Reference Laboratory and Microbiological Diagnostic Unit Public Health Laboratory, Doherty Institute Caly L., Seemann T., Sait, M., Schultz M., Druce J., Sherry, N.
EPI_ISL_419920 hCoV-19/Australia/VIC225/2020 Oceania / Australia / Victoria 2020-03-20 Victorian Infectious Diseases Reference Laboratory (VIDRL) Victorian Infectious Diseases Reference Laboratory and Microbiological Diagnostic Unit Public Health Laboratory, Doherty Institute Caly L., Seemann T., Sait, M., Schultz M., Druce J., Sherry, N.
EPI_ISL_419921 hCoV-19/Australia/VIC226/2020 Oceania / Australia / Victoria 2020-03-20 Victorian Infectious Diseases Reference Laboratory (VIDRL) Victorian Infectious Diseases Reference Laboratory and Microbiological Diagnostic Unit Public Health Laboratory, Doherty Institute Caly L., Seemann T., Sait, M., Schultz M., Druce J., Sherry, N.
EPI_ISL_419922 hCoV-19/Australia/VIC227/2020 Oceania / Australia / Victoria 2020-03-20 Victorian Infectious Diseases Reference Laboratory (VIDRL) Victorian Infectious Diseases Reference Laboratory and Microbiological Diagnostic Unit Public Health Laboratory, Doherty Institute Caly L., Seemann T., Sait, M., Schultz M., Druce J., Sherry, N.
EPI_ISL_419923 hCoV-19/Australia/VIC228/2020 Oceania / Australia / Victoria 2020-03-20 Victorian Infectious Diseases Reference Laboratory (VIDRL) Victorian Infectious Diseases Reference Laboratory and Microbiological Diagnostic Unit Public Health Laboratory, Doherty Institute Caly L., Seemann T., Sait, M., Schultz M., Druce J., Sherry, N.
EPI_ISL_419924 hCoV-19/Australia/VIC229/2020 Oceania / Australia / Victoria 2020-03-21 Victorian Infectious Diseases Reference Laboratory (VIDRL) Victorian Infectious Diseases Reference Laboratory and Microbiological Diagnostic Unit Public Health Laboratory, Doherty Institute Caly L., Seemann T., Sait, M., Schultz M., Druce J., Sherry, N.
EPI_ISL_419925 hCoV-19/Australia/VIC230/2020 Oceania / Australia / Victoria 2020-03-21 Victorian Infectious Diseases Reference Laboratory (VIDRL) Victorian Infectious Diseases Reference Laboratory and Microbiological Diagnostic Unit Public Health Laboratory, Doherty Institute Caly L., Seemann T., Sait, M., Schultz M., Druce J., Sherry, N.
EPI_ISL_419926 hCoV-19/Australia/VIC231/2020 Oceania / Australia / Victoria 2020-03-21 Victorian Infectious Diseases Reference Laboratory (VIDRL) Victorian Infectious Diseases Reference Laboratory and Microbiological Diagnostic Unit Public Health Laboratory, Doherty Institute Caly L., Seemann T., Sait, M., Schultz M., Druce J., Sherry, N.
EPI_ISL_419927 hCoV-19/Australia/VIC232/2020 Oceania / Australia / Victoria 2020-03-21 Victorian Infectious Diseases Reference Laboratory (VIDRL) Victorian Infectious Diseases Reference Laboratory and Microbiological Diagnostic Unit Public Health Laboratory, Doherty Institute Caly L., Seemann T., Sait, M., Schultz M., Druce J., Sherry, N.
EPI_ISL_419928 hCoV-19/Australia/VIC233/2020 Oceania / Australia / Victoria 2020-03-21 Victorian Infectious Diseases Reference Laboratory (VIDRL) Victorian Infectious Diseases Reference Laboratory and Microbiological Diagnostic Unit Public Health Laboratory, Doherty Institute Caly L., Seemann T., Sait, M., Schultz M., Druce J., Sherry, N.
EPI_ISL_419929 hCoV-19/Australia/VIC234/2020 Oceania / Australia / Victoria 2020-03-21 Victorian Infectious Diseases Reference Laboratory (VIDRL) Victorian Infectious Diseases Reference Laboratory and Microbiological Diagnostic Unit Public Health Laboratory, Doherty Institute Caly L., Seemann T., Sait, M., Schultz M., Druce J., Sherry, N.
EPI_ISL_419931 hCoV-19/Australia/VIC236/2020 Oceania / Australia / Victoria 2020-03-21 Victorian Infectious Diseases Reference Laboratory (VIDRL) Victorian Infectious Diseases Reference Laboratory and Microbiological Diagnostic Unit Public Health Laboratory, Doherty Institute Caly L., Seemann T., Sait, M., Schultz M., Druce J., Sherry, N.
EPI_ISL_419932 hCoV-19/Australia/VIC237/2020 Oceania / Australia / Victoria 2020-03-21 Victorian Infectious Diseases Reference Laboratory (VIDRL) Victorian Infectious Diseases Reference Laboratory and Microbiological Diagnostic Unit Public Health Laboratory, Doherty Institute Caly L., Seemann T., Sait, M., Schultz M., Druce J., Sherry, N.
EPI_ISL_419933 hCoV-19/Australia/VIC238/2020 Oceania / Australia / Victoria 2020-03-21 Victorian Infectious Diseases Reference Laboratory (VIDRL) Victorian Infectious Diseases Reference Laboratory and

Microbiological Diagnostic Unit Public Health Laboratory, Doherty Institute   Caly L., Seemann T., Sait, M., Schultz M., Druce J., Sherry, N.
EPI_ISL_419934 hCoV-19/Australia/VIC239/2020   Oceania / Australia / Victoria 2020-03-21   Victorian Infectious Diseases Reference Laboratory (VIDRL)   Victorian Infectious Diseases Reference Laboratory and Microbiological Diagnostic Unit Public Health Laboratory, Doherty Institute   Caly L., Seemann T., Sait, M., Schultz M., Druce J., Sherry, N.
EPI_ISL_419935 hCoV-19/Australia/VIC240/2020   Oceania / Australia / Victoria 2020-03-21   Victorian Infectious Diseases Reference Laboratory (VIDRL)   Victorian Infectious Diseases Reference Laboratory and Microbiological Diagnostic Unit Public Health Laboratory, Doherty Institute   Caly L., Seemann T., Sait, M., Schultz M., Druce J., Sherry, N.
EPI_ISL_419936 hCoV-19/Australia/VIC241/2020   Oceania / Australia / Victoria 2020-03-21   Victorian Infectious Diseases Reference Laboratory (VIDRL)   Victorian Infectious Diseases Reference Laboratory and Microbiological Diagnostic Unit Public Health Laboratory, Doherty Institute   Caly L., Seemann T., Sait, M., Schultz M., Druce J., Sherry, N.
EPI_ISL_419937 hCoV-19/Australia/VIC242/2020   Oceania / Australia / Victoria 2020-03-21   Victorian Infectious Diseases Reference Laboratory (VIDRL)   Victorian Infectious Diseases Reference Laboratory and Microbiological Diagnostic Unit Public Health Laboratory, Doherty Institute   Caly L., Seemann T., Sait, M., Schultz M., Druce J., Sherry, N.
EPI_ISL_419938 hCoV-19/Australia/VIC243/2020   Oceania / Australia / Victoria 2020-03-21   Victorian Infectious Diseases Reference Laboratory (VIDRL)   Victorian Infectious Diseases Reference Laboratory and Microbiological Diagnostic Unit Public Health Laboratory, Doherty Institute   Caly L., Seemann T., Sait, M., Schultz M., Druce J., Sherry, N.
EPI_ISL_419939 hCoV-19/Australia/VIC244/2020   Oceania / Australia / Victoria 2020-03-21   Victorian Infectious Diseases Reference Laboratory (VIDRL)   Victorian Infectious Diseases Reference Laboratory and Microbiological Diagnostic Unit Public Health Laboratory, Doherty Institute   Caly L., Seemann T., Sait, M., Schultz M., Druce J., Sherry, N.
EPI_ISL_419940 hCoV-19/Australia/VIC245/2020   Oceania / Australia / Victoria 2020-03-21   Victorian Infectious Diseases Reference Laboratory (VIDRL)   Victorian Infectious Diseases Reference Laboratory and Microbiological Diagnostic Unit Public Health Laboratory, Doherty Institute   Caly L., Seemann T., Sait, M., Schultz M., Druce J., Sherry, N.
EPI_ISL_419941 hCoV-19/Australia/VIC246/2020   Oceania / Australia / Victoria 2020-03-21   Victorian Infectious Diseases Reference Laboratory (VIDRL)   Victorian Infectious Diseases Reference Laboratory and Microbiological Diagnostic Unit Public Health Laboratory, Doherty Institute   Caly L., Seemann T., Sait, M., Schultz M., Druce J., Sherry, N.
EPI_ISL_419942 hCoV-19/Australia/VIC247/2020   Oceania / Australia / Victoria 2020-03-21   Victorian Infectious Diseases Reference Laboratory (VIDRL)   Victorian Infectious Diseases Reference Laboratory and Microbiological Diagnostic Unit Public Health Laboratory, Doherty Institute   Caly L., Seemann T., Sait, M., Schultz M., Druce J., Sherry, N.
EPI_ISL_419943 hCoV-19/Australia/VIC248/2020   Oceania / Australia / Victoria 2020-03-21   Victorian Infectious Diseases Reference Laboratory (VIDRL)   Victorian Infectious Diseases Reference Laboratory and Microbiological Diagnostic Unit Public Health Laboratory, Doherty Institute   Caly L., Seemann T., Sait, M., Schultz M., Druce J., Sherry, N.
EPI_ISL_419944 hCoV-19/Australia/VIC249/2020   Oceania / Australia / Victoria 2020-03-21   Victorian Infectious Diseases Reference Laboratory (VIDRL)   Victorian Infectious Diseases Reference Laboratory and Microbiological Diagnostic Unit Public Health Laboratory, Doherty Institute   Caly L., Seemann T., Sait, M., Schultz M., Druce J., Sherry, N.
EPI_ISL_419945 hCoV-19/Australia/VIC250/2020   Oceania / Australia / Victoria 2020-03-21   Victorian Infectious Diseases Reference Laboratory (VIDRL)   Victorian Infectious Diseases Reference Laboratory and Microbiological Diagnostic Unit Public Health Laboratory, Doherty Institute   Caly L., Seemann T., Sait, M., Schultz M., Druce J., Sherry, N.
EPI_ISL_419946 hCoV-19/Australia/VIC251/2020   Oceania / Australia / Victoria 2020-03-21   Victorian Infectious Diseases Reference Laboratory (VIDRL)   Victorian Infectious Diseases Reference Laboratory and Microbiological Diagnostic Unit Public Health Laboratory, Doherty Institute   Caly L., Seemann T., Sait, M., Schultz M., Druce J., Sherry, N.
EPI_ISL_419947 hCoV-19/Australia/VIC252/2020   Oceania / Australia / Victoria 2020-03-21   Victorian Infectious Diseases Reference Laboratory (VIDRL)   Victorian Infectious Diseases Reference Laboratory and Microbiological Diagnostic Unit Public Health Laboratory, Doherty Institute   Caly L., Seemann T., Sait, M., Schultz M., Druce J., Sherry, N.
EPI_ISL_419948 hCoV-19/Australia/VIC253/2020   Oceania / Australia / Victoria 2020-03-21   Victorian Infectious Diseases Reference Laboratory (VIDRL)   Victorian Infectious Diseases Reference Laboratory and Microbiological Diagnostic Unit Public Health Laboratory, Doherty Institute   Caly L., Seemann T., Sait, M., Schultz M., Druce J., Sherry, N.
EPI_ISL_419949 hCoV-19/Australia/VIC254/2020   Oceania / Australia / Victoria 2020-03-21   Victorian Infectious Diseases Reference Laboratory (VIDRL)   Victorian Infectious Diseases Reference Laboratory and Microbiological Diagnostic Unit Public Health Laboratory, Doherty Institute   Caly L., Seemann T., Sait, M., Schultz M., Druce J., Sherry, N.
EPI_ISL_419950 hCoV-19/Australia/VIC257/2020   Oceania / Australia / Victoria 2020-03-21   Victorian Infectious Diseases Reference Laboratory (VIDRL)   Victorian Infectious Diseases Reference Laboratory and Microbiological Diagnostic Unit Public Health Laboratory, Doherty Institute   Caly L., Seemann T., Sait, M., Schultz M., Druce J., Sherry, N.

EPI_ISL_419951 hCoV-19/Australia/VIC258/2020 Oceania / Australia / Victoria 2020-03-21 Victorian Infectious Diseases Reference Laboratory (VIDRL) Victorian Infectious Diseases Reference Laboratory and Microbiological Diagnostic Unit Public Health Laboratory, Doherty Institute Caly L., Seemann T., Sait, M., Schultz M., Druce J., Sherry, N.
EPI_ISL_419952 hCoV-19/Australia/VIC259/2020 Oceania / Australia / Victoria 2020-03-21 Victorian Infectious Diseases Reference Laboratory (VIDRL) Victorian Infectious Diseases Reference Laboratory and Microbiological Diagnostic Unit Public Health Laboratory, Doherty Institute Caly L., Seemann T., Sait, M., Schultz M., Druce J., Sherry, N.
EPI_ISL_419953 hCoV-19/Australia/VIC260/2020 Oceania / Australia / Victoria 2020-03-21 Victorian Infectious Diseases Reference Laboratory (VIDRL) Victorian Infectious Diseases Reference Laboratory and Microbiological Diagnostic Unit Public Health Laboratory, Doherty Institute Caly L., Seemann T., Sait, M., Schultz M., Druce J., Sherry, N.
EPI_ISL_419954 hCoV-19/Australia/VIC261/2020 Oceania / Australia / Victoria 2020-03-21 Victorian Infectious Diseases Reference Laboratory (VIDRL) Victorian Infectious Diseases Reference Laboratory and Microbiological Diagnostic Unit Public Health Laboratory, Doherty Institute Caly L., Seemann T., Sait, M., Schultz M., Druce J., Sherry, N.
EPI_ISL_419955 hCoV-19/Australia/VIC262/2020 Oceania / Australia / Victoria 2020-03-21 Victorian Infectious Diseases Reference Laboratory (VIDRL) Victorian Infectious Diseases Reference Laboratory and Microbiological Diagnostic Unit Public Health Laboratory, Doherty Institute Caly L., Seemann T., Sait, M., Schultz M., Druce J., Sherry, N.
EPI_ISL_419956 hCoV-19/Australia/VIC263/2020 Oceania / Australia / Victoria 2020-03-21 Victorian Infectious Diseases Reference Laboratory (VIDRL) Victorian Infectious Diseases Reference Laboratory and Microbiological Diagnostic Unit Public Health Laboratory, Doherty Institute Caly L., Seemann T., Sait, M., Schultz M., Druce J., Sherry, N.
EPI_ISL_419957 hCoV-19/Australia/VIC264/2020 Oceania / Australia / Victoria 2020-03-22 Victorian Infectious Diseases Reference Laboratory (VIDRL) Victorian Infectious Diseases Reference Laboratory and Microbiological Diagnostic Unit Public Health Laboratory, Doherty Institute Caly L., Seemann T., Sait, M., Schultz M., Druce J., Sherry, N.
EPI_ISL_419958 hCoV-19/Australia/VIC265/2020 Oceania / Australia / Victoria 2020-03-22 Victorian Infectious Diseases Reference Laboratory (VIDRL) Victorian Infectious Diseases Reference Laboratory and Microbiological Diagnostic Unit Public Health Laboratory, Doherty Institute Caly L., Seemann T., Sait, M., Schultz M., Druce J., Sherry, N.
EPI_ISL_419959 hCoV-19/Australia/VIC266/2020 Oceania / Australia / Victoria 2020-03-22 Victorian Infectious Diseases Reference Laboratory (VIDRL) Victorian Infectious Diseases Reference Laboratory and Microbiological Diagnostic Unit Public Health Laboratory, Doherty Institute Caly L., Seemann T., Sait, M., Schultz M., Druce J., Sherry, N.
EPI_ISL_419960 hCoV-19/Australia/VIC267/2020 Oceania / Australia / Victoria 2020-03-22 Victorian Infectious Diseases Reference Laboratory (VIDRL) Victorian Infectious Diseases Reference Laboratory and Microbiological Diagnostic Unit Public Health Laboratory, Doherty Institute Caly L., Seemann T., Sait, M., Schultz M., Druce J., Sherry, N.
EPI_ISL_419961 hCoV-19/Australia/VIC268/2020 Oceania / Australia / Victoria 2020-03-22 Victorian Infectious Diseases Reference Laboratory (VIDRL) Victorian Infectious Diseases Reference Laboratory and Microbiological Diagnostic Unit Public Health Laboratory, Doherty Institute Caly L., Seemann T., Sait, M., Schultz M., Druce J., Sherry, N.
EPI_ISL_419962 hCoV-19/Australia/VIC269/2020 Oceania / Australia / Victoria 2020-03-22 Victorian Infectious Diseases Reference Laboratory (VIDRL) Victorian Infectious Diseases Reference Laboratory and Microbiological Diagnostic Unit Public Health Laboratory, Doherty Institute Caly L., Seemann T., Sait, M., Schultz M., Druce J., Sherry, N.
EPI_ISL_419963 hCoV-19/Australia/VIC270/2020 Oceania / Australia / Victoria 2020-03-22 Victorian Infectious Diseases Reference Laboratory (VIDRL) Victorian Infectious Diseases Reference Laboratory and Microbiological Diagnostic Unit Public Health Laboratory, Doherty Institute Caly L., Seemann T., Sait, M., Schultz M., Druce J., Sherry, N.
EPI_ISL_419964 hCoV-19/Australia/VIC271/2020 Oceania / Australia / Victoria 2020-03-22 Victorian Infectious Diseases Reference Laboratory (VIDRL) Victorian Infectious Diseases Reference Laboratory and Microbiological Diagnostic Unit Public Health Laboratory, Doherty Institute Caly L., Seemann T., Sait, M., Schultz M., Druce J., Sherry, N.
EPI_ISL_419965 hCoV-19/Australia/VIC272/2020 Oceania / Australia / Victoria 2020-03-22 Victorian Infectious Diseases Reference Laboratory (VIDRL) Victorian Infectious Diseases Reference Laboratory and Microbiological Diagnostic Unit Public Health Laboratory, Doherty Institute Caly L., Seemann T., Sait, M., Schultz M., Druce J., Sherry, N.
EPI_ISL_419966 hCoV-19/Australia/VIC273/2020 Oceania / Australia / Victoria 2020-03-22 Victorian Infectious Diseases Reference Laboratory (VIDRL) Victorian Infectious Diseases Reference Laboratory and Microbiological Diagnostic Unit Public Health Laboratory, Doherty Institute Caly L., Seemann T., Sait, M., Schultz M., Druce J., Sherry, N.
EPI_ISL_419967 hCoV-19/Australia/VIC274/2020 Oceania / Australia / Victoria 2020-03-22 Victorian Infectious Diseases Reference Laboratory (VIDRL) Victorian Infectious Diseases Reference Laboratory and Microbiological Diagnostic Unit Public Health Laboratory, Doherty Institute Caly L., Seemann T., Sait, M., Schultz M., Druce J., Sherry, N.
EPI_ISL_419968 hCoV-19/Australia/VIC275/2020 Oceania / Australia / Victoria 2020-03-22 Victorian Infectious Diseases Reference Laboratory (VIDRL) Victorian Infectious Diseases Reference Laboratory and

Microbiological Diagnostic Unit Public Health Laboratory, Doherty Institute	Caly L., Seemann T., Sait, M., Schultz M., Druce J., Sherry, N.
EPI_ISL_419969	hCoV-19/Australia/VIC276/2020	Oceania / Australia / Victoria	2020-03-22	Victorian Infectious Diseases Reference Laboratory (VIDRL)	Victorian Infectious Diseases Reference Laboratory and Microbiological Diagnostic Unit Public Health Laboratory, Doherty Institute	Caly L., Seemann T., Sait, M., Schultz M., Druce J., Sherry, N.
EPI_ISL_419970	hCoV-19/Australia/VIC277/2020	Oceania / Australia / Victoria	2020-03-22	Victorian Infectious Diseases Reference Laboratory (VIDRL)	Victorian Infectious Diseases Reference Laboratory and Microbiological Diagnostic Unit Public Health Laboratory, Doherty Institute	Caly L., Seemann T., Sait, M., Schultz M., Druce J., Sherry, N.
EPI_ISL_419971	hCoV-19/Australia/VIC278/2020	Oceania / Australia / Victoria	2020-03-22	Victorian Infectious Diseases Reference Laboratory (VIDRL)	Victorian Infectious Diseases Reference Laboratory and Microbiological Diagnostic Unit Public Health Laboratory, Doherty Institute	Caly L., Seemann T., Sait, M., Schultz M., Druce J., Sherry, N.
EPI_ISL_419972	hCoV-19/Australia/VIC279/2020	Oceania / Australia / Victoria	2020-03-22	Victorian Infectious Diseases Reference Laboratory (VIDRL)	Victorian Infectious Diseases Reference Laboratory and Microbiological Diagnostic Unit Public Health Laboratory, Doherty Institute	Caly L., Seemann T., Sait, M., Schultz M., Druce J., Sherry, N.
EPI_ISL_419973	hCoV-19/Australia/VIC280/2020	Oceania / Australia / Victoria	2020-03-22	Victorian Infectious Diseases Reference Laboratory (VIDRL)	Victorian Infectious Diseases Reference Laboratory and Microbiological Diagnostic Unit Public Health Laboratory, Doherty Institute	Caly L., Seemann T., Sait, M., Schultz M., Druce J., Sherry, N.
EPI_ISL_419974	hCoV-19/Australia/VIC283/2020	Oceania / Australia / Victoria	2020-03-22	Victorian Infectious Diseases Reference Laboratory (VIDRL)	Victorian Infectious Diseases Reference Laboratory and Microbiological Diagnostic Unit Public Health Laboratory, Doherty Institute	Caly L., Seemann T., Sait, M., Schultz M., Druce J., Sherry, N.
EPI_ISL_419975	hCoV-19/Australia/VIC284/2020	Oceania / Australia / Victoria	2020-03-22	Victorian Infectious Diseases Reference Laboratory (VIDRL)	Victorian Infectious Diseases Reference Laboratory and Microbiological Diagnostic Unit Public Health Laboratory, Doherty Institute	Caly L., Seemann T., Sait, M., Schultz M., Druce J., Sherry, N.
EPI_ISL_419976	hCoV-19/Australia/VIC285/2020	Oceania / Australia / Victoria	2020-03-22	Victorian Infectious Diseases Reference Laboratory (VIDRL)	Victorian Infectious Diseases Reference Laboratory and Microbiological Diagnostic Unit Public Health Laboratory, Doherty Institute	Caly L., Seemann T., Sait, M., Schultz M., Druce J., Sherry, N.
EPI_ISL_419977	hCoV-19/Australia/VIC286/2020	Oceania / Australia / Victoria	2020-03-22	Victorian Infectious Diseases Reference Laboratory (VIDRL)	Victorian Infectious Diseases Reference Laboratory and Microbiological Diagnostic Unit Public Health Laboratory, Doherty Institute	Caly L., Seemann T., Sait, M., Schultz M., Druce J., Sherry, N.
EPI_ISL_419978	hCoV-19/Australia/VIC287/2020	Oceania / Australia / Victoria	2020-03-22	Victorian Infectious Diseases Reference Laboratory (VIDRL)	Victorian Infectious Diseases Reference Laboratory and Microbiological Diagnostic Unit Public Health Laboratory, Doherty Institute	Caly L., Seemann T., Sait, M., Schultz M., Druce J., Sherry, N.
EPI_ISL_419979	hCoV-19/Australia/VIC288/2020	Oceania / Australia / Victoria	2020-03-22	Victorian Infectious Diseases Reference Laboratory (VIDRL)	Victorian Infectious Diseases Reference Laboratory and Microbiological Diagnostic Unit Public Health Laboratory, Doherty Institute	Caly L., Seemann T., Sait, M., Schultz M., Druce J., Sherry, N.
EPI_ISL_419980	hCoV-19/Australia/VIC289/2020	Oceania / Australia / Victoria	2020-03-22	Microbiological Diagnostic Unit Public Health Laboratory	Microbiological Diagnostic Unit Public Health Laboratory	Seemann T., Schultz M., Sait, M., Sherry, N.
EPI_ISL_419982	hCoV-19/Australia/VIC291/2020	Oceania / Australia / Victoria	2020-03-23	Victorian Infectious Diseases Reference Laboratory (VIDRL)	Victorian Infectious Diseases Reference Laboratory and Microbiological Diagnostic Unit Public Health Laboratory, Doherty Institute	Caly L., Seemann T., Sait, M., Schultz M., Druce J., Sherry, N.
EPI_ISL_419983	hCoV-19/Australia/VIC292/2020	Oceania / Australia / Victoria	2020-03-23	Victorian Infectious Diseases Reference Laboratory (VIDRL)	Victorian Infectious Diseases Reference Laboratory and Microbiological Diagnostic Unit Public Health Laboratory, Doherty Institute	Caly L., Seemann T., Sait, M., Schultz M., Druce J., Sherry, N.
EPI_ISL_419984	hCoV-19/Australia/VIC293/2020	Oceania / Australia / Victoria	2020-03-23	Victorian Infectious Diseases Reference Laboratory (VIDRL)	Victorian Infectious Diseases Reference Laboratory and Microbiological Diagnostic Unit Public Health Laboratory, Doherty Institute	Caly L., Seemann T., Sait, M., Schultz M., Druce J., Sherry, N.
EPI_ISL_419985	hCoV-19/Australia/VIC294/2020	Oceania / Australia / Victoria	2020-03-23	Victorian Infectious Diseases Reference Laboratory (VIDRL)	Victorian Infectious Diseases Reference Laboratory and Microbiological Diagnostic Unit Public Health Laboratory, Doherty Institute	Caly L., Seemann T., Sait, M., Schultz M., Druce J., Sherry, N.
EPI_ISL_419986	hCoV-19/Australia/VIC295/2020	Oceania / Australia / Victoria	2020-03-23	Victorian Infectious Diseases Reference Laboratory (VIDRL)	Victorian Infectious Diseases Reference Laboratory and Microbiological Diagnostic Unit Public Health Laboratory, Doherty Institute	Caly L., Seemann T., Sait, M., Schultz M., Druce J., Sherry, N.
EPI_ISL_419987	hCoV-19/Australia/VIC296/2020	Oceania / Australia / Victoria	2020-03-23	Victorian

Infectious Diseases Reference Laboratory (VIDRL)	Victorian Infectious Diseases Reference Laboratory and Microbiological Diagnostic Unit Public Health Laboratory, Doherty Institute	Caly L., Seemann T., Sait, M., Schultz M., Druce J., Sherry, N.
EPI_ISL_419988	hCoV-19/Australia/VIC297/2020	Oceania / Australia / Victoria	2020-03-23	Victorian Infectious Diseases Reference Laboratory (VIDRL)	Victorian Infectious Diseases Reference Laboratory and Microbiological Diagnostic Unit Public Health Laboratory, Doherty Institute	Caly L., Seemann T., Sait, M., Schultz M., Druce J., Sherry, N.
EPI_ISL_419989	hCoV-19/Australia/VIC298/2020	Oceania / Australia / Victoria	2020-03-23	Victorian Infectious Diseases Reference Laboratory (VIDRL)	Victorian Infectious Diseases Reference Laboratory and Microbiological Diagnostic Unit Public Health Laboratory, Doherty Institute	Caly L., Seemann T., Sait, M., Schultz M., Druce J., Sherry, N.
EPI_ISL_419990	hCoV-19/Australia/VIC299/2020	Oceania / Australia / Victoria	2020-03-23	Victorian Infectious Diseases Reference Laboratory (VIDRL)	Victorian Infectious Diseases Reference Laboratory and Microbiological Diagnostic Unit Public Health Laboratory, Doherty Institute	Caly L., Seemann T., Sait, M., Schultz M., Druce J., Sherry, N.
EPI_ISL_419991	hCoV-19/Australia/VIC300/2020	Oceania / Australia / Victoria	2020-03-23	Victorian Infectious Diseases Reference Laboratory (VIDRL)	Victorian Infectious Diseases Reference Laboratory and Microbiological Diagnostic Unit Public Health Laboratory, Doherty Institute	Caly L., Seemann T., Sait, M., Schultz M., Druce J., Sherry, N.
EPI_ISL_419992	hCoV-19/Australia/VIC301/2020	Oceania / Australia / Victoria	2020-03-23	Victorian Infectious Diseases Reference Laboratory (VIDRL)	Victorian Infectious Diseases Reference Laboratory and Microbiological Diagnostic Unit Public Health Laboratory, Doherty Institute	Caly L., Seemann T., Sait, M., Schultz M., Druce J., Sherry, N.
EPI_ISL_419993	hCoV-19/Australia/VIC302/2020	Oceania / Australia / Victoria	2020-03-23	Victorian Infectious Diseases Reference Laboratory (VIDRL)	Victorian Infectious Diseases Reference Laboratory and Microbiological Diagnostic Unit Public Health Laboratory, Doherty Institute	Caly L., Seemann T., Sait, M., Schultz M., Druce J., Sherry, N.
EPI_ISL_419994	hCoV-19/Australia/VIC303/2020	Oceania / Australia / Victoria	2020-03-23	Victorian Infectious Diseases Reference Laboratory (VIDRL)	Victorian Infectious Diseases Reference Laboratory and Microbiological Diagnostic Unit Public Health Laboratory, Doherty Institute	Caly L., Seemann T., Sait, M., Schultz M., Druce J., Sherry, N.
EPI_ISL_419995	hCoV-19/Australia/VIC304/2020	Oceania / Australia / Victoria	2020-03-23	Victorian Infectious Diseases Reference Laboratory (VIDRL)	Victorian Infectious Diseases Reference Laboratory and Microbiological Diagnostic Unit Public Health Laboratory, Doherty Institute	Caly L., Seemann T., Sait, M., Schultz M., Druce J., Sherry, N.
EPI_ISL_419996	hCoV-19/Australia/VIC305/2020	Oceania / Australia / Victoria	2020-03-23	Victorian Infectious Diseases Reference Laboratory (VIDRL)	Victorian Infectious Diseases Reference Laboratory and Microbiological Diagnostic Unit Public Health Laboratory, Doherty Institute	Caly L., Seemann T., Sait, M., Schultz M., Druce J., Sherry, N.
EPI_ISL_419997	hCoV-19/Australia/VIC306/2020	Oceania / Australia / Victoria	2020-03-23	Victorian Infectious Diseases Reference Laboratory (VIDRL)	Victorian Infectious Diseases Reference Laboratory and Microbiological Diagnostic Unit Public Health Laboratory, Doherty Institute	Caly L., Seemann T., Sait, M., Schultz M., Druce J., Sherry, N.
EPI_ISL_419998	hCoV-19/Australia/VIC307/2020	Oceania / Australia / Victoria	2020-03-23	Victorian Infectious Diseases Reference Laboratory (VIDRL)	Victorian Infectious Diseases Reference Laboratory and Microbiological Diagnostic Unit Public Health Laboratory, Doherty Institute	Caly L., Seemann T., Sait, M., Schultz M., Druce J., Sherry, N.
EPI_ISL_419999	hCoV-19/Australia/VIC308/2020	Oceania / Australia / Victoria	2020-03-23	Microbiological Diagnostic Unit Public Health Laboratory	Microbiological Diagnostic Unit Public Health Laboratory	Seemann T., Schultz M., Sait, M., Sherry, N.
EPI_ISL_420901	hCoV-19/Germany/BAV-V2010840/2020	Europe / Germany / Munich	2020-03-12	Max von Pettenkofer Institute, Virology, National Reference Center for Retroviruses, LMU Munich	Laboratory for Functional Genome Analysis, Dept. Genomics, Gene Center of the LMU Munich	Max Muenchhoff, Stefan Krebs, Alexander Graf, Ashok Varadharajan, Oliver Keppler, Helmut Blum
EPI_ISL_420902	hCoV-19/Germany/BAV-V2011376/2020	Europe / Germany / Munich	2020-03-13	Max von Pettenkofer Institute, Virology, National Reference Center for Retroviruses, LMU Munich	Laboratory for Functional Genome Analysis, Dept. Genomics, Gene Center of the LMU Munich	Max Muenchhoff, Stefan Krebs, Alexander Graf, Ashok Varadharajan, Oliver Keppler, Helmut Blum
EPI_ISL_420903	hCoV-19/Germany/BAV-V2011395/2020	Europe / Germany / Munich	2020-03-14	Max von Pettenkofer Institute, Virology, National Reference Center for Retroviruses, LMU Munich	Laboratory for Functional Genome Analysis, Dept. Genomics, Gene Center of the LMU Munich	Max Muenchhoff, Stefan Krebs, Alexander Graf, Ashok Varadharajan, Oliver Keppler, Helmut Blum
EPI_ISL_420906	hCoV-19/Germany/BAV-V2011673/2020	Europe / Germany / Munich	2020-03-16	Max von Pettenkofer Institute, Virology, National Reference Center for Retroviruses, LMU Munich	Laboratory for Functional Genome Analysis, Dept. Genomics, Gene Center of the LMU Munich	Max Muenchhoff, Stefan Krebs, Alexander Graf, Ashok Varadharajan, Oliver Keppler, Helmut Blum
EPI_ISL_420907	hCoV-19/Germany/BAV-V2011675/2020	Europe / Germany / Munich	2020-03-16	Max von Pettenkofer Institute, Virology, National Reference Center for Retroviruses, LMU Munich	Laboratory for Functional Genome Analysis, Dept. Genomics, Gene Center of the LMU Munich	Max Muenchhoff, Stefan Krebs, Alexander Graf, Ashok Varadharajan, Oliver Keppler, Helmut Blum

EPI_ISL_420910 hCoV-19/England/20136087804/2020 Europe / United Kingdom / England 2020-03-25 Respiratory Virus Unit, Microbiology Services Colindale, Public Health England Respiratory Virus Unit, Microbiology Services Colindale, Public Health England Monica Galiano, Shahjahan Miah, Angie Lackenby, Omolola Akinbami, Tiina Talts, Leena Bhaw, Richard Myers, Steven Platt, Kirstin Edwards, Jonathan Hubb, Joanna Ellis, Maria Zambon
EPI_ISL_420912 hCoV-19/Germany/BAV-V2012856/2020 Europe / Germany / Munich 2020-03-21 Max von Pettenkofer Institute, Virology, National Reference Center for Retroviruses, LMU Munich Laboratory for Functional Genome Analysis, Dept. Genomics, Gene Center of the LMU Munich Max Muenchhoff, Stefan Krebs, Alexander Graf, Ashok Varadharajan, Oliver Keppler, Helmut Blum
EPI_ISL_420929 hCoV-19/Wales/PHWC-25005/2020 Europe / United Kingdom / Wales 2020-03-20 Wales Specialist Virology Centre Public Health Wales Microbiology Cardiff Catherine Moore, Joanne Watkins, Sally Corden, Malorie Perry, Simon Cottrell Sara Rey, Matt Bull, Tom Connor
EPI_ISL_420937 hCoV-19/Wales/PHWC-24CB7/2020 Europe / United Kingdom / Wales 2020-03-21 Wales Specialist Virology Centre Public Health Wales Microbiology Cardiff Catherine Moore, Joanne Watkins, Sally Corden, Malorie Perry, Simon Cottrell Sara Rey, Matt Bull, Tom Connor
EPI_ISL_420940 hCoV-19/Wales/PHWC-24DD2/2020 Europe / United Kingdom / Wales 2020-03-21 Wales Specialist Virology Centre Public Health Wales Microbiology Cardiff Catherine Moore, Joanne Watkins, Sally Corden, Malorie Perry, Simon Cottrell Sara Rey, Matt Bull, Tom Connor
EPI_ISL_420943 hCoV-19/Wales/PHWC-24F36/2020 Europe / United Kingdom / Wales 2020-03-21 Wales Specialist Virology Centre Public Health Wales Microbiology Cardiff Catherine Moore, Joanne Watkins, Sally Corden, Malorie Perry, Simon Cottrell Sara Rey, Matt Bull, Tom Connor
EPI_ISL_420944 hCoV-19/Wales/PHWC-24FFA/2020 Europe / United Kingdom / Wales 2020-03-21 Wales Specialist Virology Centre Public Health Wales Microbiology Cardiff Catherine Moore, Joanne Watkins, Sally Corden, Malorie Perry, Simon Cottrell Sara Rey, Matt Bull, Tom Connor
EPI_ISL_420949 hCoV-19/Wales/PHWC-2522D/2020 Europe / United Kingdom / Wales 2020-03-21 Wales Specialist Virology Centre Public Health Wales Microbiology Cardiff Catherine Moore, Joanne Watkins, Sally Corden, Malorie Perry, Simon Cottrell Sara Rey, Matt Bull, Tom Connor
EPI_ISL_420951 hCoV-19/Wales/PHWC-24CF3/2020 Europe / United Kingdom / Wales 2020-03-22 Wales Specialist Virology Centre Public Health Wales Microbiology Cardiff Catherine Moore, Joanne Watkins, Sally Corden, Malorie Perry, Simon Cottrell Sara Rey, Matt Bull, Tom Connor
EPI_ISL_420960 hCoV-19/Wales/PHWC-24F72/2020 Europe / United Kingdom / Wales 2020-03-22 Wales Specialist Virology Centre Public Health Wales Microbiology Cardiff Catherine Moore, Joanne Watkins, Sally Corden, Malorie Perry, Simon Cottrell Sara Rey, Matt Bull, Tom Connor
EPI_ISL_420961 hCoV-19/Wales/PHWC-24F81/2020 Europe / United Kingdom / Wales 2020-03-22 Wales Specialist Virology Centre Public Health Wales Microbiology Cardiff Catherine Moore, Joanne Watkins, Sally Corden, Malorie Perry, Simon Cottrell Sara Rey, Matt Bull, Tom Connor
EPI_ISL_420969 hCoV-19/Wales/PHWC-25111/2020 Europe / United Kingdom / Wales 2020-03-22 Wales Specialist Virology Centre Public Health Wales Microbiology Cardiff Catherine Moore, Joanne Watkins, Sally Corden, Malorie Perry, Simon Cottrell Sara Rey, Matt Bull, Tom Connor
EPI_ISL_420970 hCoV-19/Wales/PHWC-25120/2020 Europe / United Kingdom / Wales 2020-03-22 Wales Specialist Virology Centre Public Health Wales Microbiology Cardiff Catherine Moore, Joanne Watkins, Sally Corden, Malorie Perry, Simon Cottrell Sara Rey, Matt Bull, Tom Connor
EPI_ISL_420974 hCoV-19/Wales/PHWC-2516C/2020 Europe / United Kingdom / Wales 2020-03-22 Wales Specialist Virology Centre Public Health Wales Microbiology Cardiff Catherine Moore, Joanne Watkins, Sally Corden, Malorie Perry, Simon Cottrell Sara Rey, Matt Bull, Tom Connor
EPI_ISL_420976 hCoV-19/Wales/PHWC-251B7/2020 Europe / United Kingdom / Wales 2020-03-22 Wales Specialist Virology Centre Public Health Wales Microbiology Cardiff Catherine Moore, Joanne Watkins, Sally Corden, Malorie Perry, Simon Cottrell Sara Rey, Matt Bull, Tom Connor
EPI_ISL_420977 hCoV-19/Wales/PHWC-2523C/2020 Europe / United Kingdom / Wales 2020-03-22 Wales Specialist Virology Centre Public Health Wales Microbiology Cardiff Catherine Moore, Joanne Watkins, Sally Corden, Malorie Perry, Simon Cottrell Sara Rey, Matt Bull, Tom Connor
EPI_ISL_420978 hCoV-19/Wales/PHWC-253B1/2020 Europe / United Kingdom / Wales 2020-03-22 Wales Specialist Virology Centre Public Health Wales Microbiology Cardiff Catherine Moore, Joanne Watkins, Sally Corden, Malorie Perry, Simon Cottrell Sara Rey, Matt Bull, Tom Connor
EPI_ISL_420982 hCoV-19/Wales/PHWC-251D5/2020 Europe / United Kingdom / Wales 2020-03-23 Wales Specialist Virology Centre Public Health Wales Microbiology Cardiff Catherine Moore, Joanne Watkins, Sally Corden, Malorie Perry, Simon Cottrell Sara Rey, Matt Bull, Tom Connor
EPI_ISL_420985 hCoV-19/Wales/PHWC-252B4/2020 Europe / United Kingdom / Wales 2020-03-23 Wales Specialist Virology Centre Public Health Wales Microbiology Cardiff Catherine Moore, Joanne Watkins, Sally Corden, Malorie Perry, Simon Cottrell Sara Rey, Matt Bull, Tom Connor
EPI_ISL_420988 hCoV-19/Wales/PHWC-253DF/2020 Europe / United Kingdom / Wales 2020-03-23 Wales Specialist Virology Centre Public Health Wales Microbiology Cardiff Catherine Moore, Joanne Watkins, Sally Corden, Malorie Perry, Simon Cottrell Sara Rey, Matt Bull, Tom Connor
EPI_ISL_420990 hCoV-19/Wales/PHWC-25418/2020 Europe / United Kingdom / Wales 2020-03-23 Wales Specialist Virology Centre Public Health Wales Microbiology Cardiff Catherine Moore, Joanne Watkins, Sally Corden, Malorie Perry, Simon Cottrell Sara Rey, Matt Bull, Tom Connor
EPI_ISL_420994 hCoV-19/Wales/PHWC-25463/2020 Europe / United Kingdom / Wales 2020-03-23 Wales Specialist Virology Centre Public Health Wales Microbiology Cardiff Catherine Moore, Joanne Watkins, Sally Corden, Malorie Perry, Simon Cottrell Sara Rey, Matt Bull, Tom Connor
EPI_ISL_420995 hCoV-19/Wales/PHWC-25472/2020 Europe / United Kingdom / Wales 2020-03-23 Wales

Specialist Virology Centre	Public Health Wales Microbiology Cardiff	Catherine Moore, Joanne Watkins, Sally Corden, Malorie Perry, Simon Cottrell Sara Rey, Matt Bull, Tom Connor
EPI_ISL_420998	hCoV-19/Wales/PHWC-254AF/2020	Europe / United Kingdom / Wales 2020-03-23	Wales Specialist Virology Centre	Public Health Wales Microbiology Cardiff	Catherine Moore, Joanne Watkins, Sally Corden, Malorie Perry, Simon Cottrell Sara Rey, Matt Bull, Tom Connor
EPI_ISL_423100	hCoV-19/England/20134018902/2020	Europe / United Kingdom / England	2020-03-24	Respiratory Virus Unit, Microbiology Services Colindale, Public Health England	Respiratory Virus Unit, Microbiology Services Colindale, Public Health England	Monica Galiano, Shahjahan Miah, Angie Lackenby, Omolola Akinbami, Tiina Talts, Leena Bhaw, Richard Myers, Steven Platt, Kirstin Edwards, Jonathan Hubb, Joanna Ellis, Maria Zambon
EPI_ISL_423101	hCoV-19/England/20134019102/2020	Europe / United Kingdom / England	2020-03-24	Respiratory Virus Unit, Microbiology Services Colindale, Public Health England	Respiratory Virus Unit, Microbiology Services Colindale, Public Health England	Monica Galiano, Shahjahan Miah, Angie Lackenby, Omolola Akinbami, Tiina Talts, Leena Bhaw, Richard Myers, Steven Platt, Kirstin Edwards, Jonathan Hubb, Joanna Ellis, Maria Zambon
EPI_ISL_423102	hCoV-19/England/20134019202/2020	Europe / United Kingdom / England	2020-03-24	Respiratory Virus Unit, Microbiology Services Colindale, Public Health England	Respiratory Virus Unit, Microbiology Services Colindale, Public Health England	Monica Galiano, Shahjahan Miah, Angie Lackenby, Omolola Akinbami, Tiina Talts, Leena Bhaw, Richard Myers, Steven Platt, Kirstin Edwards, Jonathan Hubb, Joanna Ellis, Maria Zambon
EPI_ISL_423104	hCoV-19/England/20134019402/2020	Europe / United Kingdom / England	2020-03-23	Respiratory Virus Unit, Microbiology Services Colindale, Public Health England	Respiratory Virus Unit, Microbiology Services Colindale, Public Health England	Monica Galiano, Shahjahan Miah, Angie Lackenby, Omolola Akinbami, Tiina Talts, Leena Bhaw, Richard Myers, Steven Platt, Kirstin Edwards, Jonathan Hubb, Joanna Ellis, Maria Zambon
EPI_ISL_423105	hCoV-19/England/20134019502/2020	Europe / United Kingdom / England	2020-03-23	Respiratory Virus Unit, Microbiology Services Colindale, Public Health England	Respiratory Virus Unit, Microbiology Services Colindale, Public Health England	Monica Galiano, Shahjahan Miah, Angie Lackenby, Omolola Akinbami, Tiina Talts, Leena Bhaw, Richard Myers, Steven Platt, Kirstin Edwards, Jonathan Hubb, Joanna Ellis, Maria Zambon
EPI_ISL_423107	hCoV-19/England/20134019904/2020	Europe / United Kingdom / England	2020-03-23	Respiratory Virus Unit, Microbiology Services Colindale, Public Health England	Respiratory Virus Unit, Microbiology Services Colindale, Public Health England	Monica Galiano, Shahjahan Miah, Angie Lackenby, Omolola Akinbami, Tiina Talts, Leena Bhaw, Richard Myers, Steven Platt, Kirstin Edwards, Jonathan Hubb, Joanna Ellis, Maria Zambon
EPI_ISL_423109	hCoV-19/England/20134020104/2020	Europe / United Kingdom / England	2020-03-23	Respiratory Virus Unit, Microbiology Services Colindale, Public Health England	Respiratory Virus Unit, Microbiology Services Colindale, Public Health England	Monica Galiano, Shahjahan Miah, Angie Lackenby, Omolola Akinbami, Tiina Talts, Leena Bhaw, Richard Myers, Steven Platt, Kirstin Edwards, Jonathan Hubb, Joanna Ellis, Maria Zambon
EPI_ISL_423110	hCoV-19/England/20134020504/2020	Europe / United Kingdom / England	2020-03-24	Respiratory Virus Unit, Microbiology Services Colindale, Public Health England	Respiratory Virus Unit, Microbiology Services Colindale, Public Health England	Monica Galiano, Shahjahan Miah, Angie Lackenby, Omolola Akinbami, Tiina Talts, Leena Bhaw, Richard Myers, Steven Platt, Kirstin Edwards, Jonathan Hubb, Joanna Ellis, Maria Zambon
EPI_ISL_423111	hCoV-19/England/20134020904/2020	Europe / United Kingdom / England	2020-03-24	Respiratory Virus Unit, Microbiology Services Colindale, Public Health England	Respiratory Virus Unit, Microbiology Services Colindale, Public Health England	Monica Galiano, Shahjahan Miah, Angie Lackenby, Omolola Akinbami, Tiina Talts, Leena Bhaw, Richard Myers, Steven Platt, Kirstin Edwards, Jonathan Hubb, Joanna Ellis, Maria Zambon
EPI_ISL_423112	hCoV-19/England/20134021004/2020	Europe / United Kingdom / England	2020-03-24	Respiratory Virus Unit, Microbiology Services Colindale, Public Health England	Respiratory Virus Unit, Microbiology Services Colindale, Public Health England	Monica Galiano, Shahjahan Miah, Angie Lackenby, Omolola Akinbami, Tiina Talts, Leena Bhaw, Richard Myers, Steven Platt, Kirstin Edwards, Jonathan Hubb, Joanna Ellis, Maria Zambon
EPI_ISL_423113	hCoV-19/England/20134021104/2020	Europe / United Kingdom / England	2020-03-24	Respiratory Virus Unit, Microbiology Services Colindale, Public Health England	Respiratory Virus Unit, Microbiology Services Colindale, Public Health England	Monica Galiano, Shahjahan Miah, Angie Lackenby, Omolola Akinbami, Tiina Talts, Leena Bhaw, Richard Myers, Steven Platt, Kirstin Edwards, Jonathan Hubb, Joanna Ellis, Maria Zambon
EPI_ISL_423117	hCoV-19/England/20134021804/2020	Europe / United Kingdom / England	2020-03-24	Respiratory Virus Unit, Microbiology Services Colindale, Public Health England	Respiratory Virus Unit, Microbiology Services Colindale, Public Health England	Monica Galiano, Shahjahan Miah, Angie Lackenby, Omolola Akinbami, Tiina Talts, Leena Bhaw, Richard Myers, Steven Platt, Kirstin Edwards, Jonathan Hubb, Joanna Ellis, Maria Zambon
EPI_ISL_423118	hCoV-19/England/20134021904/2020	Europe / United Kingdom / England	2020-03-23	Respiratory Virus Unit, Microbiology Services Colindale, Public Health England	Respiratory Virus Unit, Microbiology Services Colindale, Public Health England	Monica Galiano, Shahjahan Miah, Angie Lackenby, Omolola Akinbami, Tiina Talts, Leena Bhaw, Richard Myers, Steven Platt, Kirstin Edwards, Jonathan Hubb, Joanna Ellis, Maria Zambon

EPI_ISL_423119 hCoV-19/England/20134022104/2020 Europe / United Kingdom / England 2020-03-25 Respiratory Virus Unit, Microbiology Services Colindale, Public Health England Respiratory Virus Unit, Microbiology Services Colindale, Public Health England Monica Galiano, Shahjahan Miah, Angie Lackenby, Omolola Akinbami, Tiina Talts, Leena Bhaw, Richard Myers, Steven Platt, Kirstin Edwards, Jonathan Hubb, Joanna Ellis, Maria Zambon
EPI_ISL_423121 hCoV-19/England/20134023004/2020 Europe / United Kingdom / England 2020-03-24 Respiratory Virus Unit, Microbiology Services Colindale, Public Health England Respiratory Virus Unit, Microbiology Services Colindale, Public Health England Monica Galiano, Shahjahan Miah, Angie Lackenby, Omolola Akinbami, Tiina Talts, Leena Bhaw, Richard Myers, Steven Platt, Kirstin Edwards, Jonathan Hubb, Joanna Ellis, Maria Zambon
EPI_ISL_423122 hCoV-19/England/20134023104/2020 Europe / United Kingdom / England 2020-03-24 Respiratory Virus Unit, Microbiology Services Colindale, Public Health England Respiratory Virus Unit, Microbiology Services Colindale, Public Health England Monica Galiano, Shahjahan Miah, Angie Lackenby, Omolola Akinbami, Tiina Talts, Leena Bhaw, Richard Myers, Steven Platt, Kirstin Edwards, Jonathan Hubb, Joanna Ellis, Maria Zambon
EPI_ISL_423123 hCoV-19/England/20134023204/2020 Europe / United Kingdom / England 2020-03-24 Respiratory Virus Unit, Microbiology Services Colindale, Public Health England Respiratory Virus Unit, Microbiology Services Colindale, Public Health England Monica Galiano, Shahjahan Miah, Angie Lackenby, Omolola Akinbami, Tiina Talts, Leena Bhaw, Richard Myers, Steven Platt, Kirstin Edwards, Jonathan Hubb, Joanna Ellis, Maria Zambon
EPI_ISL_423124 hCoV-19/England/20134023704/2020 Europe / United Kingdom / England 2020-03-24 Respiratory Virus Unit, Microbiology Services Colindale, Public Health England Respiratory Virus Unit, Microbiology Services Colindale, Public Health England Monica Galiano, Shahjahan Miah, Angie Lackenby, Omolola Akinbami, Tiina Talts, Leena Bhaw, Richard Myers, Steven Platt, Kirstin Edwards, Jonathan Hubb, Joanna Ellis, Maria Zambon
EPI_ISL_423126 hCoV-19/England/20134024104/2020 Europe / United Kingdom / England 2020-03-24 Respiratory Virus Unit, Microbiology Services Colindale, Public Health England Respiratory Virus Unit, Microbiology Services Colindale, Public Health England Monica Galiano, Shahjahan Miah, Angie Lackenby, Omolola Akinbami, Tiina Talts, Leena Bhaw, Richard Myers, Steven Platt, Kirstin Edwards, Jonathan Hubb, Joanna Ellis, Maria Zambon
EPI_ISL_423128 hCoV-19/England/20134024504/2020 Europe / United Kingdom / England 2020-03-23 Respiratory Virus Unit, Microbiology Services Colindale, Public Health England Respiratory Virus Unit, Microbiology Services Colindale, Public Health England Monica Galiano, Shahjahan Miah, Angie Lackenby, Omolola Akinbami, Tiina Talts, Leena Bhaw, Richard Myers, Steven Platt, Kirstin Edwards, Jonathan Hubb, Joanna Ellis, Maria Zambon
EPI_ISL_423129 hCoV-19/England/20134025204/2020 Europe / United Kingdom / England 2020-03-23 Respiratory Virus Unit, Microbiology Services Colindale, Public Health England Respiratory Virus Unit, Microbiology Services Colindale, Public Health England Monica Galiano, Shahjahan Miah, Angie Lackenby, Omolola Akinbami, Tiina Talts, Leena Bhaw, Richard Myers, Steven Platt, Kirstin Edwards, Jonathan Hubb, Joanna Ellis, Maria Zambon
EPI_ISL_423130 hCoV-19/England/20134025504/2020 Europe / United Kingdom / England 2020-03-24 Respiratory Virus Unit, Microbiology Services Colindale, Public Health England Respiratory Virus Unit, Microbiology Services Colindale, Public Health England Monica Galiano, Shahjahan Miah, Angie Lackenby, Omolola Akinbami, Tiina Talts, Leena Bhaw, Richard Myers, Steven Platt, Kirstin Edwards, Jonathan Hubb, Joanna Ellis, Maria Zambon
EPI_ISL_423131 hCoV-19/England/20134025704/2020 Europe / United Kingdom / England 2020-03-24 Respiratory Virus Unit, Microbiology Services Colindale, Public Health England Respiratory Virus Unit, Microbiology Services Colindale, Public Health England Monica Galiano, Shahjahan Miah, Angie Lackenby, Omolola Akinbami, Tiina Talts, Leena Bhaw, Richard Myers, Steven Platt, Kirstin Edwards, Jonathan Hubb, Joanna Ellis, Maria Zambon
EPI_ISL_423132 hCoV-19/England/20134025904/2020 Europe / United Kingdom / England 2020-03-24 Respiratory Virus Unit, Microbiology Services Colindale, Public Health England Respiratory Virus Unit, Microbiology Services Colindale, Public Health England Monica Galiano, Shahjahan Miah, Angie Lackenby, Omolola Akinbami, Tiina Talts, Leena Bhaw, Richard Myers, Steven Platt, Kirstin Edwards, Jonathan Hubb, Joanna Ellis, Maria Zambon
EPI_ISL_423134 hCoV-19/England/20134026904/2020 Europe / United Kingdom / England 2020-03-23 Respiratory Virus Unit, Microbiology Services Colindale, Public Health England Respiratory Virus Unit, Microbiology Services Colindale, Public Health England Monica Galiano, Shahjahan Miah, Angie Lackenby, Omolola Akinbami, Tiina Talts, Leena Bhaw, Richard Myers, Steven Platt, Kirstin Edwards, Jonathan Hubb, Joanna Ellis, Maria Zambon
EPI_ISL_423136 hCoV-19/England/20134027504/2020 Europe / United Kingdom / England 2020-03-23 Respiratory Virus Unit, Microbiology Services Colindale, Public Health England Respiratory Virus Unit, Microbiology Services Colindale, Public Health England Monica Galiano, Shahjahan Miah, Angie Lackenby, Omolola Akinbami, Tiina Talts, Leena Bhaw, Richard Myers, Steven Platt, Kirstin Edwards, Jonathan Hubb, Joanna Ellis, Maria Zambon
EPI_ISL_423142 hCoV-19/England/20134047404/2020 Europe / United Kingdom / England 2020-03-24 Respiratory Virus Unit, Microbiology Services Colindale, Public Health England Respiratory Virus Unit, Microbiology Services Colindale, Public Health England Monica Galiano, Shahjahan Miah, Angie Lackenby, Omolola Akinbami, Tiina Talts, Leena Bhaw, Richard Myers, Steven Platt, Kirstin Edwards, Jonathan Hubb, Joanna Ellis, Maria Zambon

EPI_ISL_423144 hCoV-19/England/20134059604/2020   Europe / United Kingdom / England   2020-03-24
Respiratory Virus Unit, Microbiology Services Colindale, Public Health England Respiratory Virus Unit, Microbiology Services Colindale, Public Health England Monica Galiano, Shahjahan Miah, Angie Lackenby, Omolola Akinbami, Tiina Talts, Leena Bhaw, Richard Myers, Steven Platt, Kirstin Edwards, Jonathan Hubb, Joanna Ellis, Maria Zambon
EPI_ISL_423145 hCoV-19/England/20134059704/2020   Europe / United Kingdom / England   2020-03-24
Respiratory Virus Unit, Microbiology Services Colindale, Public Health England Respiratory Virus Unit, Microbiology Services Colindale, Public Health England Monica Galiano, Shahjahan Miah, Angie Lackenby, Omolola Akinbami, Tiina Talts, Leena Bhaw, Richard Myers, Steven Platt, Kirstin Edwards, Jonathan Hubb, Joanna Ellis, Maria Zambon
EPI_ISL_423147 hCoV-19/England/20134060504/2020   Europe / United Kingdom / England   2020-03-23
Respiratory Virus Unit, Microbiology Services Colindale, Public Health England Respiratory Virus Unit, Microbiology Services Colindale, Public Health England Monica Galiano, Shahjahan Miah, Angie Lackenby, Omolola Akinbami, Tiina Talts, Leena Bhaw, Richard Myers, Steven Platt, Kirstin Edwards, Jonathan Hubb, Joanna Ellis, Maria Zambon
EPI_ISL_423148 hCoV-19/England/20134060804/2020   Europe / United Kingdom / England   2020-03-24
Respiratory Virus Unit, Microbiology Services Colindale, Public Health England Respiratory Virus Unit, Microbiology Services Colindale, Public Health England Monica Galiano, Shahjahan Miah, Angie Lackenby, Omolola Akinbami, Tiina Talts, Leena Bhaw, Richard Myers, Steven Platt, Kirstin Edwards, Jonathan Hubb, Joanna Ellis, Maria Zambon
EPI_ISL_423149 hCoV-19/England/20134060904/2020   Europe / United Kingdom / England   2020-03-21
Respiratory Virus Unit, Microbiology Services Colindale, Public Health England Respiratory Virus Unit, Microbiology Services Colindale, Public Health England Monica Galiano, Shahjahan Miah, Angie Lackenby, Omolola Akinbami, Tiina Talts, Leena Bhaw, Richard Myers, Steven Platt, Kirstin Edwards, Jonathan Hubb, Joanna Ellis, Maria Zambon
EPI_ISL_423150 hCoV-19/England/20134064004/2020   Europe / United Kingdom / England   2020-03-23
Respiratory Virus Unit, Microbiology Services Colindale, Public Health England Respiratory Virus Unit, Microbiology Services Colindale, Public Health England Monica Galiano, Shahjahan Miah, Angie Lackenby, Omolola Akinbami, Tiina Talts, Leena Bhaw, Richard Myers, Steven Platt, Kirstin Edwards, Jonathan Hubb, Joanna Ellis, Maria Zambon
EPI_ISL_423151 hCoV-19/England/20134064204/2020   Europe / United Kingdom / England   2020-03-24
Respiratory Virus Unit, Microbiology Services Colindale, Public Health England Respiratory Virus Unit, Microbiology Services Colindale, Public Health England Monica Galiano, Shahjahan Miah, Angie Lackenby, Omolola Akinbami, Tiina Talts, Leena Bhaw, Richard Myers, Steven Platt, Kirstin Edwards, Jonathan Hubb, Joanna Ellis, Maria Zambon
EPI_ISL_423152 hCoV-19/England/20134064904/2020   Europe / United Kingdom / England   2020-03-24
Respiratory Virus Unit, Microbiology Services Colindale, Public Health England Respiratory Virus Unit, Microbiology Services Colindale, Public Health England Monica Galiano, Shahjahan Miah, Angie Lackenby, Omolola Akinbami, Tiina Talts, Leena Bhaw, Richard Myers, Steven Platt, Kirstin Edwards, Jonathan Hubb, Joanna Ellis, Maria Zambon
EPI_ISL_423154 hCoV-19/England/20134067304/2020   Europe / United Kingdom / England   2020-03-24
Respiratory Virus Unit, Microbiology Services Colindale, Public Health England Respiratory Virus Unit, Microbiology Services Colindale, Public Health England Monica Galiano, Shahjahan Miah, Angie Lackenby, Omolola Akinbami, Tiina Talts, Leena Bhaw, Richard Myers, Steven Platt, Kirstin Edwards, Jonathan Hubb, Joanna Ellis, Maria Zambon
EPI_ISL_423156 hCoV-19/England/20134067904/2020   Europe / United Kingdom / England   2020-03-24
Respiratory Virus Unit, Microbiology Services Colindale, Public Health England Respiratory Virus Unit, Microbiology Services Colindale, Public Health England Monica Galiano, Shahjahan Miah, Angie Lackenby, Omolola Akinbami, Tiina Talts, Leena Bhaw, Richard Myers, Steven Platt, Kirstin Edwards, Jonathan Hubb, Joanna Ellis, Maria Zambon
EPI_ISL_423157 hCoV-19/England/20134068504/2020   Europe / United Kingdom / England   2020-03-24
Respiratory Virus Unit, Microbiology Services Colindale, Public Health England Respiratory Virus Unit, Microbiology Services Colindale, Public Health England Monica Galiano, Shahjahan Miah, Angie Lackenby, Omolola Akinbami, Tiina Talts, Leena Bhaw, Richard Myers, Steven Platt, Kirstin Edwards, Jonathan Hubb, Joanna Ellis, Maria Zambon
EPI_ISL_423158 hCoV-19/England/20134068604/2020   Europe / United Kingdom / England   2020-03-24
Respiratory Virus Unit, Microbiology Services Colindale, Public Health England Respiratory Virus Unit, Microbiology Services Colindale, Public Health England Monica Galiano, Shahjahan Miah, Angie Lackenby, Omolola Akinbami, Tiina Talts, Leena Bhaw, Richard Myers, Steven Platt, Kirstin Edwards, Jonathan Hubb, Joanna Ellis, Maria Zambon
EPI_ISL_423159 hCoV-19/England/20134068704/2020   Europe / United Kingdom / England   2020-03-24
Respiratory Virus Unit, Microbiology Services Colindale, Public Health England Respiratory Virus Unit, Microbiology Services Colindale, Public Health England Monica Galiano, Shahjahan Miah, Angie Lackenby, Omolola Akinbami, Tiina Talts, Leena Bhaw, Richard Myers, Steven Platt, Kirstin Edwards, Jonathan Hubb, Joanna Ellis, Maria Zambon
EPI_ISL_423160 hCoV-19/England/20134068904/2020   Europe / United Kingdom / England   2020-03-24
Respiratory Virus Unit, Microbiology Services Colindale, Public Health England Respiratory Virus Unit, Microbiology Services Colindale, Public Health England Monica Galiano, Shahjahan Miah, Angie Lackenby, Omolola Akinbami, Tiina Talts, Leena Bhaw, Richard Myers, Steven Platt, Kirstin Edwards, Jonathan Hubb, Joanna Ellis, Maria Zambon

EPI_ISL_423161 hCoV-19/England/20134069004/2020   Europe / United Kingdom / England   2020-03-24
Respiratory Virus Unit, Microbiology Services Colindale, Public Health England Respiratory Virus Unit, Microbiology Services Colindale, Public Health England Monica Galiano, Shahjahan Miah, Angie Lackenby, Omolola Akinbami, Tiina Talts, Leena Bhaw, Richard Myers, Steven Platt, Kirstin Edwards, Jonathan Hubb, Joanna Ellis, Maria Zambon
EPI_ISL_423162 hCoV-19/England/20134071504/2020   Europe / United Kingdom / England   2020-03-24
Respiratory Virus Unit, Microbiology Services Colindale, Public Health England Respiratory Virus Unit, Microbiology Services Colindale, Public Health England Monica Galiano, Shahjahan Miah, Angie Lackenby, Omolola Akinbami, Tiina Talts, Leena Bhaw, Richard Myers, Steven Platt, Kirstin Edwards, Jonathan Hubb, Joanna Ellis, Maria Zambon
EPI_ISL_423163 hCoV-19/England/20134071804/2020   Europe / United Kingdom / England   2020-03-23
Respiratory Virus Unit, Microbiology Services Colindale, Public Health England Respiratory Virus Unit, Microbiology Services Colindale, Public Health England Monica Galiano, Shahjahan Miah, Angie Lackenby, Omolola Akinbami, Tiina Talts, Leena Bhaw, Richard Myers, Steven Platt, Kirstin Edwards, Jonathan Hubb, Joanna Ellis, Maria Zambon
EPI_ISL_423170 hCoV-19/England/20134077504/2020   Europe / United Kingdom / England   2020-03-24
Respiratory Virus Unit, Microbiology Services Colindale, Public Health England Respiratory Virus Unit, Microbiology Services Colindale, Public Health England Monica Galiano, Shahjahan Miah, Angie Lackenby, Omolola Akinbami, Tiina Talts, Leena Bhaw, Richard Myers, Steven Platt, Kirstin Edwards, Jonathan Hubb, Joanna Ellis, Maria Zambon
EPI_ISL_423172 hCoV-19/England/20134078004/2020   Europe / United Kingdom / England   2020-03-24
Respiratory Virus Unit, Microbiology Services Colindale, Public Health England Respiratory Virus Unit, Microbiology Services Colindale, Public Health England Monica Galiano, Shahjahan Miah, Angie Lackenby, Omolola Akinbami, Tiina Talts, Leena Bhaw, Richard Myers, Steven Platt, Kirstin Edwards, Jonathan Hubb, Joanna Ellis, Maria Zambon
EPI_ISL_423173 hCoV-19/England/20134078204/2020   Europe / United Kingdom / England   2020-03-24
Respiratory Virus Unit, Microbiology Services Colindale, Public Health England Respiratory Virus Unit, Microbiology Services Colindale, Public Health England Monica Galiano, Shahjahan Miah, Angie Lackenby, Omolola Akinbami, Tiina Talts, Leena Bhaw, Richard Myers, Steven Platt, Kirstin Edwards, Jonathan Hubb, Joanna Ellis, Maria Zambon
EPI_ISL_423174 hCoV-19/England/20134078404/2020   Europe / United Kingdom / England   2020-03-24
Respiratory Virus Unit, Microbiology Services Colindale, Public Health England Respiratory Virus Unit, Microbiology Services Colindale, Public Health England Monica Galiano, Shahjahan Miah, Angie Lackenby, Omolola Akinbami, Tiina Talts, Leena Bhaw, Richard Myers, Steven Platt, Kirstin Edwards, Jonathan Hubb, Joanna Ellis, Maria Zambon
EPI_ISL_423175 hCoV-19/England/20134078604/2020   Europe / United Kingdom / England   2020-03-24
Respiratory Virus Unit, Microbiology Services Colindale, Public Health England Respiratory Virus Unit, Microbiology Services Colindale, Public Health England Monica Galiano, Shahjahan Miah, Angie Lackenby, Omolola Akinbami, Tiina Talts, Leena Bhaw, Richard Myers, Steven Platt, Kirstin Edwards, Jonathan Hubb, Joanna Ellis, Maria Zambon
EPI_ISL_423176 hCoV-19/England/20134078804/2020   Europe / United Kingdom / England   2020-03-24
Respiratory Virus Unit, Microbiology Services Colindale, Public Health England Respiratory Virus Unit, Microbiology Services Colindale, Public Health England Monica Galiano, Shahjahan Miah, Angie Lackenby, Omolola Akinbami, Tiina Talts, Leena Bhaw, Richard Myers, Steven Platt, Kirstin Edwards, Jonathan Hubb, Joanna Ellis, Maria Zambon
EPI_ISL_423177 hCoV-19/England/20134078904/2020   Europe / United Kingdom / England   2020-03-24
Respiratory Virus Unit, Microbiology Services Colindale, Public Health England Respiratory Virus Unit, Microbiology Services Colindale, Public Health England Monica Galiano, Shahjahan Miah, Angie Lackenby, Omolola Akinbami, Tiina Talts, Leena Bhaw, Richard Myers, Steven Platt, Kirstin Edwards, Jonathan Hubb, Joanna Ellis, Maria Zambon
EPI_ISL_423178 hCoV-19/England/20134079504/2020   Europe / United Kingdom / England   2020-03-24
Respiratory Virus Unit, Microbiology Services Colindale, Public Health England Respiratory Virus Unit, Microbiology Services Colindale, Public Health England Monica Galiano, Shahjahan Miah, Angie Lackenby, Omolola Akinbami, Tiina Talts, Leena Bhaw, Richard Myers, Steven Platt, Kirstin Edwards, Jonathan Hubb, Joanna Ellis, Maria Zambon
EPI_ISL_423181 hCoV-19/England/20134081904/2020   Europe / United Kingdom / England   2020-03-20
Respiratory Virus Unit, Microbiology Services Colindale, Public Health England Respiratory Virus Unit, Microbiology Services Colindale, Public Health England Monica Galiano, Shahjahan Miah, Angie Lackenby, Omolola Akinbami, Tiina Talts, Leena Bhaw, Richard Myers, Steven Platt, Kirstin Edwards, Jonathan Hubb, Joanna Ellis, Maria Zambon
EPI_ISL_423193 hCoV-19/England/201340875/2020 Europe / United Kingdom / England   2020-03-24
Respiratory Virus Unit, Microbiology Services Colindale, Public Health England Respiratory Virus Unit, Microbiology Services Colindale, Public Health England Monica Galiano, Shahjahan Miah, Angie Lackenby, Omolola Akinbami, Tiina Talts, Leena Bhaw, Richard Myers, Steven Platt, Kirstin Edwards, Jonathan Hubb, Joanna Ellis, Maria Zambon
EPI_ISL_423194 hCoV-19/England/201360001/2020 Europe / United Kingdom / England   2020-03-25
Respiratory Virus Unit, Microbiology Services Colindale, Public Health England Respiratory Virus Unit, Microbiology Services Colindale, Public Health England Monica Galiano, Shahjahan Miah, Angie Lackenby, Omolola Akinbami, Tiina Talts, Leena Bhaw, Richard Myers, Steven Platt, Kirstin Edwards, Jonathan Hubb, Joanna Ellis, Maria Zambon

EPI_ISL_423196 hCoV-19/England/201360015/2020 Europe / United Kingdom / England   2020-03-24
Respiratory Virus Unit, Microbiology Services Colindale, Public Health England Respiratory Virus Unit, Microbiology Services Colindale, Public Health England Monica Galiano, Shahjahan Miah, Angie Lackenby, Omolola Akinbami, Tiina Talts, Leena Bhaw, Richard Myers, Steven Platt, Kirstin Edwards, Jonathan Hubb, Joanna Ellis, Maria Zambon
EPI_ISL_423199 hCoV-19/England/201360037/2020 Europe / United Kingdom / England   2020-03-24
Respiratory Virus Unit, Microbiology Services Colindale, Public Health England Respiratory Virus Unit, Microbiology Services Colindale, Public Health England Monica Galiano, Shahjahan Miah, Angie Lackenby, Omolola Akinbami, Tiina Talts, Leena Bhaw, Richard Myers, Steven Platt, Kirstin Edwards, Jonathan Hubb, Joanna Ellis, Maria Zambon
EPI_ISL_423250 hCoV-19/England/201360220/2020 Europe / United Kingdom / England   2020-03-25
Respiratory Virus Unit, Microbiology Services Colindale, Public Health England Respiratory Virus Unit, Microbiology Services Colindale, Public Health England Monica Galiano, Shahjahan Miah, Angie Lackenby, Omolola Akinbami, Tiina Talts, Leena Bhaw, Richard Myers, Steven Platt, Kirstin Edwards, Jonathan Hubb, Joanna Ellis, Maria Zambon
EPI_ISL_423251 hCoV-19/England/201360221/2020 Europe / United Kingdom / England   2020-03-25
Respiratory Virus Unit, Microbiology Services Colindale, Public Health England Respiratory Virus Unit, Microbiology Services Colindale, Public Health England Monica Galiano, Shahjahan Miah, Angie Lackenby, Omolola Akinbami, Tiina Talts, Leena Bhaw, Richard Myers, Steven Platt, Kirstin Edwards, Jonathan Hubb, Joanna Ellis, Maria Zambon
EPI_ISL_423253 hCoV-19/England/201360223/2020 Europe / United Kingdom / England   2020-03-25
Respiratory Virus Unit, Microbiology Services Colindale, Public Health England Respiratory Virus Unit, Microbiology Services Colindale, Public Health England Monica Galiano, Shahjahan Miah, Angie Lackenby, Omolola Akinbami, Tiina Talts, Leena Bhaw, Richard Myers, Steven Platt, Kirstin Edwards, Jonathan Hubb, Joanna Ellis, Maria Zambon
EPI_ISL_423254 hCoV-19/England/201360224/2020 Europe / United Kingdom / England   2020-03-25
Respiratory Virus Unit, Microbiology Services Colindale, Public Health England Respiratory Virus Unit, Microbiology Services Colindale, Public Health England Monica Galiano, Shahjahan Miah, Angie Lackenby, Omolola Akinbami, Tiina Talts, Leena Bhaw, Richard Myers, Steven Platt, Kirstin Edwards, Jonathan Hubb, Joanna Ellis, Maria Zambon
EPI_ISL_423255 hCoV-19/England/201360229/2020 Europe / United Kingdom / England   2020-03-25
Respiratory Virus Unit, Microbiology Services Colindale, Public Health England Respiratory Virus Unit, Microbiology Services Colindale, Public Health England Monica Galiano, Shahjahan Miah, Angie Lackenby, Omolola Akinbami, Tiina Talts, Leena Bhaw, Richard Myers, Steven Platt, Kirstin Edwards, Jonathan Hubb, Joanna Ellis, Maria Zambon
EPI_ISL_423256 hCoV-19/England/201360236/2020 Europe / United Kingdom / England   2020-03-25
Respiratory Virus Unit, Microbiology Services Colindale, Public Health England Respiratory Virus Unit, Microbiology Services Colindale, Public Health England Monica Galiano, Shahjahan Miah, Angie Lackenby, Omolola Akinbami, Tiina Talts, Leena Bhaw, Richard Myers, Steven Platt, Kirstin Edwards, Jonathan Hubb, Joanna Ellis, Maria Zambon
EPI_ISL_423258 hCoV-19/England/201360249/2020 Europe / United Kingdom / England   2020-03-23
Respiratory Virus Unit, Microbiology Services Colindale, Public Health England Respiratory Virus Unit, Microbiology Services Colindale, Public Health England Monica Galiano, Shahjahan Miah, Angie Lackenby, Omolola Akinbami, Tiina Talts, Leena Bhaw, Richard Myers, Steven Platt, Kirstin Edwards, Jonathan Hubb, Joanna Ellis, Maria Zambon
EPI_ISL_423259 hCoV-19/England/201360251/2020 Europe / United Kingdom / England   2020-03-24
Respiratory Virus Unit, Microbiology Services Colindale, Public Health England Respiratory Virus Unit, Microbiology Services Colindale, Public Health England Monica Galiano, Shahjahan Miah, Angie Lackenby, Omolola Akinbami, Tiina Talts, Leena Bhaw, Richard Myers, Steven Platt, Kirstin Edwards, Jonathan Hubb, Joanna Ellis, Maria Zambon
EPI_ISL_423261 hCoV-19/England/201360276/2020 Europe / United Kingdom / England   2020-03-25
Respiratory Virus Unit, Microbiology Services Colindale, Public Health England Respiratory Virus Unit, Microbiology Services Colindale, Public Health England Monica Galiano, Shahjahan Miah, Angie Lackenby, Omolola Akinbami, Tiina Talts, Leena Bhaw, Richard Myers, Steven Platt, Kirstin Edwards, Jonathan Hubb, Joanna Ellis, Maria Zambon
EPI_ISL_423262 hCoV-19/England/201360277/2020 Europe / United Kingdom / England   2020-03-25
Respiratory Virus Unit, Microbiology Services Colindale, Public Health England Respiratory Virus Unit, Microbiology Services Colindale, Public Health England Monica Galiano, Shahjahan Miah, Angie Lackenby, Omolola Akinbami, Tiina Talts, Leena Bhaw, Richard Myers, Steven Platt, Kirstin Edwards, Jonathan Hubb, Joanna Ellis, Maria Zambon
EPI_ISL_423263 hCoV-19/England/201360281/2020 Europe / United Kingdom / England   2020-03-26
Respiratory Virus Unit, Microbiology Services Colindale, Public Health England Respiratory Virus Unit, Microbiology Services Colindale, Public Health England Monica Galiano, Shahjahan Miah, Angie Lackenby, Omolola Akinbami, Tiina Talts, Leena Bhaw, Richard Myers, Steven Platt, Kirstin Edwards, Jonathan Hubb, Joanna Ellis, Maria Zambon
EPI_ISL_423264 hCoV-19/England/201360317/2020 Europe / United Kingdom / England   2020-03-25
Respiratory Virus Unit, Microbiology Services Colindale, Public Health England Respiratory Virus Unit, Microbiology Services Colindale, Public Health England Monica Galiano, Shahjahan Miah, Angie Lackenby, Omolola Akinbami, Tiina Talts, Leena Bhaw, Richard Myers, Steven Platt, Kirstin Edwards, Jonathan Hubb, Joanna Ellis, Maria Zambon

EPI_ISL_423265 hCoV-19/England/201360318/2020 Europe / United Kingdom / England 2020-03-25 Respiratory Virus Unit, Microbiology Services Colindale, Public Health England Respiratory Virus Unit, Microbiology Services Colindale, Public Health England Monica Galiano, Shahjahan Miah, Angie Lackenby, Omolola Akinbami, Tiina Talts, Leena Bhaw, Richard Myers, Steven Platt, Kirstin Edwards, Jonathan Hubb, Joanna Ellis, Maria Zambon
EPI_ISL_423266 hCoV-19/England/201360538/2020 Europe / United Kingdom / England 2020-03-25 Respiratory Virus Unit, Microbiology Services Colindale, Public Health England Respiratory Virus Unit, Microbiology Services Colindale, Public Health England Monica Galiano, Shahjahan Miah, Angie Lackenby, Omolola Akinbami, Tiina Talts, Leena Bhaw, Richard Myers, Steven Platt, Kirstin Edwards, Jonathan Hubb, Joanna Ellis, Maria Zambon
EPI_ISL_423269 hCoV-19/England/20138007104/2020 Europe / United Kingdom / England 2020-03-27 Respiratory Virus Unit, Microbiology Services Colindale, Public Health England Respiratory Virus Unit, Microbiology Services Colindale, Public Health England Monica Galiano, Shahjahan Miah, Angie Lackenby, Omolola Akinbami, Tiina Talts, Leena Bhaw, Richard Myers, Steven Platt, Kirstin Edwards, Jonathan Hubb, Joanna Ellis, Maria Zambon
EPI_ISL_423288 hCoV-19/England/20138017804/2020 Europe / United Kingdom / England 2020-03-26 Respiratory Virus Unit, Microbiology Services Colindale, Public Health England Respiratory Virus Unit, Microbiology Services Colindale, Public Health England Monica Galiano, Shahjahan Miah, Angie Lackenby, Omolola Akinbami, Tiina Talts, Leena Bhaw, Richard Myers, Steven Platt, Kirstin Edwards, Jonathan Hubb, Joanna Ellis, Maria Zambon
EPI_ISL_424400 hCoV-19/Iceland/376/2020 Europe / Iceland / Reykjavik 2020-03-19 The National University Hospital of Iceland deCODE genetics Daniel F Gudbjartsson; Agnar Helgason; Hakon Jonsson; Olafur T Magnusson; Pall Melsted; Gudmundur L Norddahl; Jona Saemundsdottir; Asgeir Sigurdsson; Patrick Sulem; Arna B Agustsdottir; Berglind Eiriksdottir; Run Fridriksdottir; Elisabet E Gardarsdottir; Gudmundur Georgsson; Olafia S Gretarsdottir; Kjartan R Gudmundsson; Thora R Gunnarsdottir; Arnaldur Gylfason; Hilma Holm; Brynjar O Jensson; Aslaug Jonasdottir; Kamilla S Josefsdottir; Thordur Kristjansson; Droplaug N Magnusdottir; Louise le Roux; Gudrun Sigmundsdottir; Gardar Sveinbjornsson; Kristin E Sveinsdottir; Maney Sveinsdottir; Emil A Thorarensen; Bjarni Thorbjornsson; Gisli Masson; Ingileif Jonsdottir; Alma Moller; Thorolfur Gudnason; Karl G Kristinsson; Unnur Thorsteinsdottir; Kari Stefansson
EPI_ISL_424401 hCoV-19/Iceland/377/2020 Europe / Iceland / Reykjavik 2020-03-19 The National University Hospital of Iceland deCODE genetics Daniel F Gudbjartsson; Agnar Helgason; Hakon Jonsson; Olafur T Magnusson; Pall Melsted; Gudmundur L Norddahl; Jona Saemundsdottir; Asgeir Sigurdsson; Patrick Sulem; Arna B Agustsdottir; Berglind Eiriksdottir; Run Fridriksdottir; Elisabet E Gardarsdottir; Gudmundur Georgsson; Olafia S Gretarsdottir; Kjartan R Gudmundsson; Thora R Gunnarsdottir; Arnaldur Gylfason; Hilma Holm; Brynjar O Jensson; Aslaug Jonasdottir; Kamilla S Josefsdottir; Thordur Kristjansson; Droplaug N Magnusdottir; Louise le Roux; Gudrun Sigmundsdottir; Gardar Sveinbjornsson; Kristin E Sveinsdottir; Maney Sveinsdottir; Emil A Thorarensen; Bjarni Thorbjornsson; Gisli Masson; Ingileif Jonsdottir; Alma Moller; Thorolfur Gudnason; Karl G Kristinsson; Unnur Thorsteinsdottir; Kari Stefansson
EPI_ISL_424402 hCoV-19/Iceland/378/2020 Europe / Iceland / Reykjavik 2020-03-19 The National University Hospital of Iceland deCODE genetics Daniel F Gudbjartsson; Agnar Helgason; Hakon Jonsson; Olafur T Magnusson; Pall Melsted; Gudmundur L Norddahl; Jona Saemundsdottir; Asgeir Sigurdsson; Patrick Sulem; Arna B Agustsdottir; Berglind Eiriksdottir; Run Fridriksdottir; Elisabet E Gardarsdottir; Gudmundur Georgsson; Olafia S Gretarsdottir; Kjartan R Gudmundsson; Thora R Gunnarsdottir; Arnaldur Gylfason; Hilma Holm; Brynjar O Jensson; Aslaug Jonasdottir; Kamilla S Josefsdottir; Thordur Kristjansson; Droplaug N Magnusdottir; Louise le Roux; Gudrun Sigmundsdottir; Gardar Sveinbjornsson; Kristin E Sveinsdottir; Maney Sveinsdottir; Emil A Thorarensen; Bjarni Thorbjornsson; Gisli Masson; Ingileif Jonsdottir; Alma Moller; Thorolfur Gudnason; Karl G Kristinsson; Unnur Thorsteinsdottir; Kari Stefansson
EPI_ISL_424403 hCoV-19/Iceland/379/2020 Europe / Iceland / Reykjavik 2020-03-18 The National University Hospital of Iceland deCODE genetics Daniel F Gudbjartsson; Agnar Helgason; Hakon Jonsson; Olafur T Magnusson; Pall Melsted; Gudmundur L Norddahl; Jona Saemundsdottir; Asgeir Sigurdsson; Patrick Sulem; Arna B Agustsdottir; Berglind Eiriksdottir; Run Fridriksdottir; Elisabet E Gardarsdottir; Gudmundur Georgsson; Olafia S Gretarsdottir; Kjartan R Gudmundsson; Thora R Gunnarsdottir; Arnaldur Gylfason; Hilma Holm; Brynjar O Jensson; Aslaug Jonasdottir; Kamilla S Josefsdottir; Thordur Kristjansson; Droplaug N Magnusdottir; Louise le Roux; Gudrun Sigmundsdottir; Gardar Sveinbjornsson; Kristin E Sveinsdottir; Maney Sveinsdottir; Emil A Thorarensen; Bjarni Thorbjornsson; Gisli Masson; Ingileif Jonsdottir; Alma Moller; Thorolfur Gudnason; Karl G Kristinsson; Unnur Thorsteinsdottir; Kari Stefansson
EPI_ISL_424404 hCoV-19/Iceland/380/2020 Europe / Iceland / Reykjavik 2020-03-19 The National University Hospital of Iceland deCODE genetics Daniel F Gudbjartsson; Agnar Helgason; Hakon Jonsson; Olafur T Magnusson; Pall Melsted; Gudmundur L Norddahl; Jona Saemundsdottir; Asgeir Sigurdsson; Patrick Sulem; Arna B Agustsdottir; Berglind Eiriksdottir; Run Fridriksdottir; Elisabet E Gardarsdottir; Gudmundur Georgsson; Olafia S Gretarsdottir; Kjartan R Gudmundsson; Thora R Gunnarsdottir; Arnaldur Gylfason; Hilma Holm; Brynjar O Jensson; Aslaug Jonasdottir; Kamilla S Josefsdottir; Thordur Kristjansson; Droplaug N Magnusdottir; Louise le Roux; Gudrun Sigmundsdottir; Gardar Sveinbjornsson; Kristin E Sveinsdottir; Maney Sveinsdottir; Emil A Thorarensen; Bjarni Thorbjornsson; Gisli Masson; Ingileif Jonsdottir; Alma Moller; Thorolfur Gudnason; Karl G Kristinsson; Unnur Thorsteinsdottir; Kari Stefansson
EPI_ISL_424405 hCoV-19/Iceland/381/2020 Europe / Iceland / Reykjavik 2020-03-19 The National University Hospital of Iceland deCODE genetics Daniel F Gudbjartsson; Agnar Helgason; Hakon Jonsson; Olafur T Magnusson; Pall Melsted; Gudmundur L Norddahl; Jona Saemundsdottir; Asgeir Sigurdsson; Patrick Sulem; Arna B Agustsdottir; Berglind Eiriksdottir; Run Fridriksdottir; Elisabet E Gardarsdottir; Gudmundur Georgsson; Olafia S Gretarsdottir; Kjartan R Gudmundsson; Thora R Gunnarsdottir; Arnaldur Gylfason; Hilma Holm; Brynjar O

Jensson; Aslaug Jonasdottir; Kamilla S Josefsdottir; Thordur Kristjansson; Droplaug N Magnusdottir; Louise le Roux; Gudrun Sigmundsdottir; Gardar Sveinbjornsson; Kristin E Sveinsdottir; Maney Sveinsdottir; Emil A Thorarensen; Bjarni Thorbjornsson; Gisli Masson; Ingileif Jonsdottir; Alma Moller; Thorolfur Gudnason; Karl G Kristinsson; Unnur Thorsteinsdottir; Kari Stefansson
EPI_ISL_424406 hCoV-19/Iceland/382/2020 Europe / Iceland / Reykjavik 2020-03-19 The National University Hospital of Iceland deCODE genetics Daniel F Gudbjartsson; Agnar Helgason; Hakon Jonsson; Olafur T Magnusson; Pall Melsted; Gudmundur L Norddahl; Jona Saemundsdottir; Asgeir Sigurdsson; Patrick Sulem; Arna B Agustsdottir; Berglind Eiriksdottir; Run Fridriksdottir; Elisabet E Gardarsdottir; Gudmundur Georgsson; Olafia S Gretarsdottir; Kjartan R Gudmundsson; Thora R Gunnarsdottir; Arnaldur Gylfason; Hilma Holm; Brynjar O Jensson; Aslaug Jonasdottir; Kamilla S Josefsdottir; Thordur Kristjansson; Droplaug N Magnusdottir; Louise le Roux; Gudrun Sigmundsdottir; Gardar Sveinbjornsson; Kristin E Sveinsdottir; Maney Sveinsdottir; Emil A Thorarensen; Bjarni Thorbjornsson; Gisli Masson; Ingileif Jonsdottir; Alma Moller; Thorolfur Gudnason; Karl G Kristinsson; Unnur Thorsteinsdottir; Kari Stefansson
EPI_ISL_424407 hCoV-19/Iceland/383/2020 Europe / Iceland / Reykjavik 2020-03-19 The National University Hospital of Iceland deCODE genetics Daniel F Gudbjartsson; Agnar Helgason; Hakon Jonsson; Olafur T Magnusson; Pall Melsted; Gudmundur L Norddahl; Jona Saemundsdottir; Asgeir Sigurdsson; Patrick Sulem; Arna B Agustsdottir; Berglind Eiriksdottir; Run Fridriksdottir; Elisabet E Gardarsdottir; Gudmundur Georgsson; Olafia S Gretarsdottir; Kjartan R Gudmundsson; Thora R Gunnarsdottir; Arnaldur Gylfason; Hilma Holm; Brynjar O Jensson; Aslaug Jonasdottir; Kamilla S Josefsdottir; Thordur Kristjansson; Droplaug N Magnusdottir; Louise le Roux; Gudrun Sigmundsdottir; Gardar Sveinbjornsson; Kristin E Sveinsdottir; Maney Sveinsdottir; Emil A Thorarensen; Bjarni Thorbjornsson; Gisli Masson; Ingileif Jonsdottir; Alma Moller; Thorolfur Gudnason; Karl G Kristinsson; Unnur Thorsteinsdottir; Kari Stefansson
EPI_ISL_424410 hCoV-19/Iceland/386/2020 Europe / Iceland / Reykjavik 2020-03-19 The National University Hospital of Iceland deCODE genetics Daniel F Gudbjartsson; Agnar Helgason; Hakon Jonsson; Olafur T Magnusson; Pall Melsted; Gudmundur L Norddahl; Jona Saemundsdottir; Asgeir Sigurdsson; Patrick Sulem; Arna B Agustsdottir; Berglind Eiriksdottir; Run Fridriksdottir; Elisabet E Gardarsdottir; Gudmundur Georgsson; Olafia S Gretarsdottir; Kjartan R Gudmundsson; Thora R Gunnarsdottir; Arnaldur Gylfason; Hilma Holm; Brynjar O Jensson; Aslaug Jonasdottir; Kamilla S Josefsdottir; Thordur Kristjansson; Droplaug N Magnusdottir; Louise le Roux; Gudrun Sigmundsdottir; Gardar Sveinbjornsson; Kristin E Sveinsdottir; Maney Sveinsdottir; Emil A Thorarensen; Bjarni Thorbjornsson; Gisli Masson; Ingileif Jonsdottir; Alma Moller; Thorolfur Gudnason; Karl G Kristinsson; Unnur Thorsteinsdottir; Kari Stefansson
EPI_ISL_424411 hCoV-19/Iceland/387/2020 Europe / Iceland / Reykjavik 2020-03-19 The National University Hospital of Iceland deCODE genetics Daniel F Gudbjartsson; Agnar Helgason; Hakon Jonsson; Olafur T Magnusson; Pall Melsted; Gudmundur L Norddahl; Jona Saemundsdottir; Asgeir Sigurdsson; Patrick Sulem; Arna B Agustsdottir; Berglind Eiriksdottir; Run Fridriksdottir; Elisabet E Gardarsdottir; Gudmundur Georgsson; Olafia S Gretarsdottir; Kjartan R Gudmundsson; Thora R Gunnarsdottir; Arnaldur Gylfason; Hilma Holm; Brynjar O Jensson; Aslaug Jonasdottir; Kamilla S Josefsdottir; Thordur Kristjansson; Droplaug N Magnusdottir; Louise le Roux; Gudrun Sigmundsdottir; Gardar Sveinbjornsson; Kristin E Sveinsdottir; Maney Sveinsdottir; Emil A Thorarensen; Bjarni Thorbjornsson; Gisli Masson; Ingileif Jonsdottir; Alma Moller; Thorolfur Gudnason; Karl G Kristinsson; Unnur Thorsteinsdottir; Kari Stefansson
EPI_ISL_424412 hCoV-19/Iceland/388/2020 Europe / Iceland / Reykjavik 2020-03-19 The National University Hospital of Iceland deCODE genetics Daniel F Gudbjartsson; Agnar Helgason; Hakon Jonsson; Olafur T Magnusson; Pall Melsted; Gudmundur L Norddahl; Jona Saemundsdottir; Asgeir Sigurdsson; Patrick Sulem; Arna B Agustsdottir; Berglind Eiriksdottir; Run Fridriksdottir; Elisabet E Gardarsdottir; Gudmundur Georgsson; Olafia S Gretarsdottir; Kjartan R Gudmundsson; Thora R Gunnarsdottir; Arnaldur Gylfason; Hilma Holm; Brynjar O Jensson; Aslaug Jonasdottir; Kamilla S Josefsdottir; Thordur Kristjansson; Droplaug N Magnusdottir; Louise le Roux; Gudrun Sigmundsdottir; Gardar Sveinbjornsson; Kristin E Sveinsdottir; Maney Sveinsdottir; Emil A Thorarensen; Bjarni Thorbjornsson; Gisli Masson; Ingileif Jonsdottir; Alma Moller; Thorolfur Gudnason; Karl G Kristinsson; Unnur Thorsteinsdottir; Kari Stefansson
EPI_ISL_424413 hCoV-19/Iceland/389/2020 Europe / Iceland / Reykjavik 2020-03-19 The National University Hospital of Iceland deCODE genetics Daniel F Gudbjartsson; Agnar Helgason; Hakon Jonsson; Olafur T Magnusson; Pall Melsted; Gudmundur L Norddahl; Jona Saemundsdottir; Asgeir Sigurdsson; Patrick Sulem; Arna B Agustsdottir; Berglind Eiriksdottir; Run Fridriksdottir; Elisabet E Gardarsdottir; Gudmundur Georgsson; Olafia S Gretarsdottir; Kjartan R Gudmundsson; Thora R Gunnarsdottir; Arnaldur Gylfason; Hilma Holm; Brynjar O Jensson; Aslaug Jonasdottir; Kamilla S Josefsdottir; Thordur Kristjansson; Droplaug N Magnusdottir; Louise le Roux; Gudrun Sigmundsdottir; Gardar Sveinbjornsson; Kristin E Sveinsdottir; Maney Sveinsdottir; Emil A Thorarensen; Bjarni Thorbjornsson; Gisli Masson; Ingileif Jonsdottir; Alma Moller; Thorolfur Gudnason; Karl G Kristinsson; Unnur Thorsteinsdottir; Kari Stefansson
EPI_ISL_424414 hCoV-19/Iceland/390/2020 Europe / Iceland / Reykjavik 2020-03-19 The National University Hospital of Iceland deCODE genetics Daniel F Gudbjartsson; Agnar Helgason; Hakon Jonsson; Olafur T Magnusson; Pall Melsted; Gudmundur L Norddahl; Jona Saemundsdottir; Asgeir Sigurdsson; Patrick Sulem; Arna B Agustsdottir; Berglind Eiriksdottir; Run Fridriksdottir; Elisabet E Gardarsdottir; Gudmundur Georgsson; Olafia S Gretarsdottir; Kjartan R Gudmundsson; Thora R Gunnarsdottir; Arnaldur Gylfason; Hilma Holm; Brynjar O Jensson; Aslaug Jonasdottir; Kamilla S Josefsdottir; Thordur Kristjansson; Droplaug N Magnusdottir; Louise le Roux; Gudrun Sigmundsdottir; Gardar Sveinbjornsson; Kristin E Sveinsdottir; Maney Sveinsdottir; Emil A Thorarensen; Bjarni Thorbjornsson; Gisli Masson; Ingileif Jonsdottir; Alma Moller; Thorolfur Gudnason; Karl G Kristinsson; Unnur Thorsteinsdottir; Kari Stefansson
EPI_ISL_424415 hCoV-19/Iceland/391/2020 Europe / Iceland / Reykjavik 2020-03-19 The National University Hospital of Iceland deCODE genetics Daniel F Gudbjartsson; Agnar Helgason; Hakon Jonsson; Olafur T Magnusson; Pall Melsted; Gudmundur L Norddahl; Jona Saemundsdottir; Asgeir Sigurdsson; Patrick Sulem; Arna B

Agustsdottir; Berglind Eiriksdottir; Run Fridriksdottir; Elisabet E Gardarsdottir; Gudmundur Georgsson; Olafia S Gretarsdottir; Kjartan R Gudmundsson; Thora R Gunnarsdottir; Arnaldur Gylfason; Hilma Holm; Brynjar O Jensson; Aslaug Jonasdottir; Kamilla S Josefsdottir; Thordur Kristjansson; Droplaug N Magnusdottir; Louise le Roux; Gudrun Sigmundsdottir; Gardar Sveinbjornsson; Kristin E Sveinsdottir; Maney Sveinsdottir; Emil A Thorarensen; Bjarni Thorbjornsson; Gisli Masson; Ingileif Jonsdottir; Alma Moller; Thorolfur Gudnason; Karl G Kristinsson; Unnur Thorsteinsdottir; Kari Stefansson
EPI_ISL_424418 hCoV-19/Iceland/394/2020 Europe / Iceland / Reykjavik 2020-03-19 The National University Hospital of Iceland deCODE genetics Daniel F Gudbjartsson; Agnar Helgason; Hakon Jonsson; Olafur T Magnusson; Pall Melsted; Gudmundur L Norddahl; Jona Saemundsdottir; Asgeir Sigurdsson; Patrick Sulem; Arna B Agustsdottir; Berglind Eiriksdottir; Run Fridriksdottir; Elisabet E Gardarsdottir; Gudmundur Georgsson; Olafia S Gretarsdottir; Kjartan R Gudmundsson; Thora R Gunnarsdottir; Arnaldur Gylfason; Hilma Holm; Brynjar O Jensson; Aslaug Jonasdottir; Kamilla S Josefsdottir; Thordur Kristjansson; Droplaug N Magnusdottir; Louise le Roux; Gudrun Sigmundsdottir; Gardar Sveinbjornsson; Kristin E Sveinsdottir; Maney Sveinsdottir; Emil A Thorarensen; Bjarni Thorbjornsson; Gisli Masson; Ingileif Jonsdottir; Alma Moller; Thorolfur Gudnason; Karl G Kristinsson; Unnur Thorsteinsdottir; Kari Stefansson
EPI_ISL_424419 hCoV-19/Iceland/395/2020 Europe / Iceland / Reykjavik 2020-03-19 The National University Hospital of Iceland deCODE genetics Daniel F Gudbjartsson; Agnar Helgason; Hakon Jonsson; Olafur T Magnusson; Pall Melsted; Gudmundur L Norddahl; Jona Saemundsdottir; Asgeir Sigurdsson; Patrick Sulem; Arna B Agustsdottir; Berglind Eiriksdottir; Run Fridriksdottir; Elisabet E Gardarsdottir; Gudmundur Georgsson; Olafia S Gretarsdottir; Kjartan R Gudmundsson; Thora R Gunnarsdottir; Arnaldur Gylfason; Hilma Holm; Brynjar O Jensson; Aslaug Jonasdottir; Kamilla S Josefsdottir; Thordur Kristjansson; Droplaug N Magnusdottir; Louise le Roux; Gudrun Sigmundsdottir; Gardar Sveinbjornsson; Kristin E Sveinsdottir; Maney Sveinsdottir; Emil A Thorarensen; Bjarni Thorbjornsson; Gisli Masson; Ingileif Jonsdottir; Alma Moller; Thorolfur Gudnason; Karl G Kristinsson; Unnur Thorsteinsdottir; Kari Stefansson
EPI_ISL_424422 hCoV-19/Iceland/398/2020 Europe / Iceland / Reykjavik 2020-03-19 The National University Hospital of Iceland deCODE genetics Daniel F Gudbjartsson; Agnar Helgason; Hakon Jonsson; Olafur T Magnusson; Pall Melsted; Gudmundur L Norddahl; Jona Saemundsdottir; Asgeir Sigurdsson; Patrick Sulem; Arna B Agustsdottir; Berglind Eiriksdottir; Run Fridriksdottir; Elisabet E Gardarsdottir; Gudmundur Georgsson; Olafia S Gretarsdottir; Kjartan R Gudmundsson; Thora R Gunnarsdottir; Arnaldur Gylfason; Hilma Holm; Brynjar O Jensson; Aslaug Jonasdottir; Kamilla S Josefsdottir; Thordur Kristjansson; Droplaug N Magnusdottir; Louise le Roux; Gudrun Sigmundsdottir; Gardar Sveinbjornsson; Kristin E Sveinsdottir; Maney Sveinsdottir; Emil A Thorarensen; Bjarni Thorbjornsson; Gisli Masson; Ingileif Jonsdottir; Alma Moller; Thorolfur Gudnason; Karl G Kristinsson; Unnur Thorsteinsdottir; Kari Stefansson
EPI_ISL_424423 hCoV-19/Iceland/399/2020 Europe / Iceland / Reykjavik 2020-03-19 The National University Hospital of Iceland deCODE genetics Daniel F Gudbjartsson; Agnar Helgason; Hakon Jonsson; Olafur T Magnusson; Pall Melsted; Gudmundur L Norddahl; Jona Saemundsdottir; Asgeir Sigurdsson; Patrick Sulem; Arna B Agustsdottir; Berglind Eiriksdottir; Run Fridriksdottir; Elisabet E Gardarsdottir; Gudmundur Georgsson; Olafia S Gretarsdottir; Kjartan R Gudmundsson; Thora R Gunnarsdottir; Arnaldur Gylfason; Hilma Holm; Brynjar O Jensson; Aslaug Jonasdottir; Kamilla S Josefsdottir; Thordur Kristjansson; Droplaug N Magnusdottir; Louise le Roux; Gudrun Sigmundsdottir; Gardar Sveinbjornsson; Kristin E Sveinsdottir; Maney Sveinsdottir; Emil A Thorarensen; Bjarni Thorbjornsson; Gisli Masson; Ingileif Jonsdottir; Alma Moller; Thorolfur Gudnason; Karl G Kristinsson; Unnur Thorsteinsdottir; Kari Stefansson
EPI_ISL_424424 hCoV-19/Iceland/400/2020 Europe / Iceland / Reykjavik 2020-03-19 The National University Hospital of Iceland deCODE genetics Daniel F Gudbjartsson; Agnar Helgason; Hakon Jonsson; Olafur T Magnusson; Pall Melsted; Gudmundur L Norddahl; Jona Saemundsdottir; Asgeir Sigurdsson; Patrick Sulem; Arna B Agustsdottir; Berglind Eiriksdottir; Run Fridriksdottir; Elisabet E Gardarsdottir; Gudmundur Georgsson; Olafia S Gretarsdottir; Kjartan R Gudmundsson; Thora R Gunnarsdottir; Arnaldur Gylfason; Hilma Holm; Brynjar O Jensson; Aslaug Jonasdottir; Kamilla S Josefsdottir; Thordur Kristjansson; Droplaug N Magnusdottir; Louise le Roux; Gudrun Sigmundsdottir; Gardar Sveinbjornsson; Kristin E Sveinsdottir; Maney Sveinsdottir; Emil A Thorarensen; Bjarni Thorbjornsson; Gisli Masson; Ingileif Jonsdottir; Alma Moller; Thorolfur Gudnason; Karl G Kristinsson; Unnur Thorsteinsdottir; Kari Stefansson
EPI_ISL_424425 hCoV-19/Iceland/401/2020 Europe / Iceland / Reykjavik 2020-03-19 The National University Hospital of Iceland deCODE genetics Daniel F Gudbjartsson; Agnar Helgason; Hakon Jonsson; Olafur T Magnusson; Pall Melsted; Gudmundur L Norddahl; Jona Saemundsdottir; Asgeir Sigurdsson; Patrick Sulem; Arna B Agustsdottir; Berglind Eiriksdottir; Run Fridriksdottir; Elisabet E Gardarsdottir; Gudmundur Georgsson; Olafia S Gretarsdottir; Kjartan R Gudmundsson; Thora R Gunnarsdottir; Arnaldur Gylfason; Hilma Holm; Brynjar O Jensson; Aslaug Jonasdottir; Kamilla S Josefsdottir; Thordur Kristjansson; Droplaug N Magnusdottir; Louise le Roux; Gudrun Sigmundsdottir; Gardar Sveinbjornsson; Kristin E Sveinsdottir; Maney Sveinsdottir; Emil A Thorarensen; Bjarni Thorbjornsson; Gisli Masson; Ingileif Jonsdottir; Alma Moller; Thorolfur Gudnason; Karl G Kristinsson; Unnur Thorsteinsdottir; Kari Stefansson
EPI_ISL_424426 hCoV-19/Iceland/402/2020 Europe / Iceland / Reykjavik 2020-03-19 The National University Hospital of Iceland deCODE genetics Daniel F Gudbjartsson; Agnar Helgason; Hakon Jonsson; Olafur T Magnusson; Pall Melsted; Gudmundur L Norddahl; Jona Saemundsdottir; Asgeir Sigurdsson; Patrick Sulem; Arna B Agustsdottir; Berglind Eiriksdottir; Run Fridriksdottir; Elisabet E Gardarsdottir; Gudmundur Georgsson; Olafia S Gretarsdottir; Kjartan R Gudmundsson; Thora R Gunnarsdottir; Arnaldur Gylfason; Hilma Holm; Brynjar O Jensson; Aslaug Jonasdottir; Kamilla S Josefsdottir; Thordur Kristjansson; Droplaug N Magnusdottir; Louise le Roux; Gudrun Sigmundsdottir; Gardar Sveinbjornsson; Kristin E Sveinsdottir; Maney Sveinsdottir; Emil A Thorarensen; Bjarni Thorbjornsson; Gisli Masson; Ingileif Jonsdottir; Alma Moller; Thorolfur Gudnason; Karl G Kristinsson; Unnur Thorsteinsdottir; Kari Stefansson
EPI_ISL_424427 hCoV-19/Iceland/403/2020 Europe / Iceland / Reykjavik 2020-03-19 The National

University Hospital of Iceland deCODE genetics Daniel F Gudbjartsson; Agnar Helgason; Hakon Jonsson; Olafur T Magnusson; Pall Melsted; Gudmundur L Norddahl; Jona Saemundsdottir; Asgeir Sigurdsson; Patrick Sulem; Arna B Agustsdottir; Berglind Eiriksdottir; Run Fridriksdottir; Elisabet E Gardarsdottir; Gudmundur Georgsson; Olafia S Gretarsdottir; Kjartan R Gudmundsson; Thora R Gunnarsdottir; Arnaldur Gylfason; Hilma Holm; Brynjar O Jensson; Aslaug Jonasdottir; Kamilla S Josefsdottir; Thordur Kristjansson; Droplaug N Magnusdottir; Louise le Roux; Gudrun Sigmundsdottir; Gardar Sveinbjornsson; Kristin E Sveinsdottir; Maney Sveinsdottir; Emil A Thorarensen; Bjarni Thorbjornsson; Gisli Masson; Ingileif Jonsdottir; Alma Moller; Thorolfur Gudnason; Karl G Kristinsson; Unnur Thorsteinsdottir; Kari Stefansson
EPI_ISL_424429 hCoV-19/Iceland/405/2020 Europe / Iceland / Reykjavik 2020-03-19 The National University Hospital of Iceland deCODE genetics Daniel F Gudbjartsson; Agnar Helgason; Hakon Jonsson; Olafur T Magnusson; Pall Melsted; Gudmundur L Norddahl; Jona Saemundsdottir; Asgeir Sigurdsson; Patrick Sulem; Arna B Agustsdottir; Berglind Eiriksdottir; Run Fridriksdottir; Elisabet E Gardarsdottir; Gudmundur Georgsson; Olafia S Gretarsdottir; Kjartan R Gudmundsson; Thora R Gunnarsdottir; Arnaldur Gylfason; Hilma Holm; Brynjar O Jensson; Aslaug Jonasdottir; Kamilla S Josefsdottir; Thordur Kristjansson; Droplaug N Magnusdottir; Louise le Roux; Gudrun Sigmundsdottir; Gardar Sveinbjornsson; Kristin E Sveinsdottir; Maney Sveinsdottir; Emil A Thorarensen; Bjarni Thorbjornsson; Gisli Masson; Ingileif Jonsdottir; Alma Moller; Thorolfur Gudnason; Karl G Kristinsson; Unnur Thorsteinsdottir; Kari Stefansson
EPI_ISL_424433 hCoV-19/Iceland/409/2020 Europe / Iceland / Reykjavik 2020-03-19 The National University Hospital of Iceland deCODE genetics Daniel F Gudbjartsson; Agnar Helgason; Hakon Jonsson; Olafur T Magnusson; Pall Melsted; Gudmundur L Norddahl; Jona Saemundsdottir; Asgeir Sigurdsson; Patrick Sulem; Arna B Agustsdottir; Berglind Eiriksdottir; Run Fridriksdottir; Elisabet E Gardarsdottir; Gudmundur Georgsson; Olafia S Gretarsdottir; Kjartan R Gudmundsson; Thora R Gunnarsdottir; Arnaldur Gylfason; Hilma Holm; Brynjar O Jensson; Aslaug Jonasdottir; Kamilla S Josefsdottir; Thordur Kristjansson; Droplaug N Magnusdottir; Louise le Roux; Gudrun Sigmundsdottir; Gardar Sveinbjornsson; Kristin E Sveinsdottir; Maney Sveinsdottir; Emil A Thorarensen; Bjarni Thorbjornsson; Gisli Masson; Ingileif Jonsdottir; Alma Moller; Thorolfur Gudnason; Karl G Kristinsson; Unnur Thorsteinsdottir; Kari Stefansson
EPI_ISL_424434 hCoV-19/Iceland/410/2020 Europe / Iceland / Reykjavik 2020-03-19 The National University Hospital of Iceland deCODE genetics Daniel F Gudbjartsson; Agnar Helgason; Hakon Jonsson; Olafur T Magnusson; Pall Melsted; Gudmundur L Norddahl; Jona Saemundsdottir; Asgeir Sigurdsson; Patrick Sulem; Arna B Agustsdottir; Berglind Eiriksdottir; Run Fridriksdottir; Elisabet E Gardarsdottir; Gudmundur Georgsson; Olafia S Gretarsdottir; Kjartan R Gudmundsson; Thora R Gunnarsdottir; Arnaldur Gylfason; Hilma Holm; Brynjar O Jensson; Aslaug Jonasdottir; Kamilla S Josefsdottir; Thordur Kristjansson; Droplaug N Magnusdottir; Louise le Roux; Gudrun Sigmundsdottir; Gardar Sveinbjornsson; Kristin E Sveinsdottir; Maney Sveinsdottir; Emil A Thorarensen; Bjarni Thorbjornsson; Gisli Masson; Ingileif Jonsdottir; Alma Moller; Thorolfur Gudnason; Karl G Kristinsson; Unnur Thorsteinsdottir; Kari Stefansson
EPI_ISL_424435 hCoV-19/Iceland/411/2020 Europe / Iceland / Reykjavik 2020-03-19 The National University Hospital of Iceland deCODE genetics Daniel F Gudbjartsson; Agnar Helgason; Hakon Jonsson; Olafur T Magnusson; Pall Melsted; Gudmundur L Norddahl; Jona Saemundsdottir; Asgeir Sigurdsson; Patrick Sulem; Arna B Agustsdottir; Berglind Eiriksdottir; Run Fridriksdottir; Elisabet E Gardarsdottir; Gudmundur Georgsson; Olafia S Gretarsdottir; Kjartan R Gudmundsson; Thora R Gunnarsdottir; Arnaldur Gylfason; Hilma Holm; Brynjar O Jensson; Aslaug Jonasdottir; Kamilla S Josefsdottir; Thordur Kristjansson; Droplaug N Magnusdottir; Louise le Roux; Gudrun Sigmundsdottir; Gardar Sveinbjornsson; Kristin E Sveinsdottir; Maney Sveinsdottir; Emil A Thorarensen; Bjarni Thorbjornsson; Gisli Masson; Ingileif Jonsdottir; Alma Moller; Thorolfur Gudnason; Karl G Kristinsson; Unnur Thorsteinsdottir; Kari Stefansson
EPI_ISL_424436 hCoV-19/Iceland/412/2020 Europe / Iceland / Reykjavik 2020-03-19 The National University Hospital of Iceland deCODE genetics Daniel F Gudbjartsson; Agnar Helgason; Hakon Jonsson; Olafur T Magnusson; Pall Melsted; Gudmundur L Norddahl; Jona Saemundsdottir; Asgeir Sigurdsson; Patrick Sulem; Arna B Agustsdottir; Berglind Eiriksdottir; Run Fridriksdottir; Elisabet E Gardarsdottir; Gudmundur Georgsson; Olafia S Gretarsdottir; Kjartan R Gudmundsson; Thora R Gunnarsdottir; Arnaldur Gylfason; Hilma Holm; Brynjar O Jensson; Aslaug Jonasdottir; Kamilla S Josefsdottir; Thordur Kristjansson; Droplaug N Magnusdottir; Louise le Roux; Gudrun Sigmundsdottir; Gardar Sveinbjornsson; Kristin E Sveinsdottir; Maney Sveinsdottir; Emil A Thorarensen; Bjarni Thorbjornsson; Gisli Masson; Ingileif Jonsdottir; Alma Moller; Thorolfur Gudnason; Karl G Kristinsson; Unnur Thorsteinsdottir; Kari Stefansson
EPI_ISL_424437 hCoV-19/Iceland/413/2020 Europe / Iceland / Reykjavik 2020-03-19 The National University Hospital of Iceland deCODE genetics Daniel F Gudbjartsson; Agnar Helgason; Hakon Jonsson; Olafur T Magnusson; Pall Melsted; Gudmundur L Norddahl; Jona Saemundsdottir; Asgeir Sigurdsson; Patrick Sulem; Arna B Agustsdottir; Berglind Eiriksdottir; Run Fridriksdottir; Elisabet E Gardarsdottir; Gudmundur Georgsson; Olafia S Gretarsdottir; Kjartan R Gudmundsson; Thora R Gunnarsdottir; Arnaldur Gylfason; Hilma Holm; Brynjar O Jensson; Aslaug Jonasdottir; Kamilla S Josefsdottir; Thordur Kristjansson; Droplaug N Magnusdottir; Louise le Roux; Gudrun Sigmundsdottir; Gardar Sveinbjornsson; Kristin E Sveinsdottir; Maney Sveinsdottir; Emil A Thorarensen; Bjarni Thorbjornsson; Gisli Masson; Ingileif Jonsdottir; Alma Moller; Thorolfur Gudnason; Karl G Kristinsson; Unnur Thorsteinsdottir; Kari Stefansson
EPI_ISL_424438 hCoV-19/Iceland/414/2020 Europe / Iceland / Reykjavik 2020-03-19 The National University Hospital of Iceland deCODE genetics Daniel F Gudbjartsson; Agnar Helgason; Hakon Jonsson; Olafur T Magnusson; Pall Melsted; Gudmundur L Norddahl; Jona Saemundsdottir; Asgeir Sigurdsson; Patrick Sulem; Arna B Agustsdottir; Berglind Eiriksdottir; Run Fridriksdottir; Elisabet E Gardarsdottir; Gudmundur Georgsson; Olafia S Gretarsdottir; Kjartan R Gudmundsson; Thora R Gunnarsdottir; Arnaldur Gylfason; Hilma Holm; Brynjar O Jensson; Aslaug Jonasdottir; Kamilla S Josefsdottir; Thordur Kristjansson; Droplaug N Magnusdottir; Louise le Roux; Gudrun Sigmundsdottir; Gardar Sveinbjornsson; Kristin E Sveinsdottir; Maney Sveinsdottir; Emil A Thorarensen; Bjarni Thorbjornsson; Gisli Masson; Ingileif Jonsdottir; Alma Moller; Thorolfur Gudnason; Karl G

Kristinsson; Unnur Thorsteinsdottir; Kari Stefansson
EPI_ISL_424439 hCoV-19/Iceland/415/2020 Europe / Iceland / Reykjavik 2020-03-19 The National University Hospital of Iceland deCODE genetics Daniel F Gudbjartsson; Agnar Helgason; Hakon Jonsson; Olafur T Magnusson; Pall Melsted; Gudmundur L Norddahl; Jona Saemundsdottir; Asgeir Sigurdsson; Patrick Sulem; Arna B Agustsdottir; Berglind Eiriksdottir; Run Fridriksdottir; Elisabet E Gardarsdottir; Gudmundur Georgsson; Olafia S Gretarsdottir; Kjartan R Gudmundsson; Thora R Gunnarsdottir; Arnaldur Gylfason; Hilma Holm; Brynjar O Jensson; Aslaug Jonasdottir; Kamilla S Josefsdottir; Thordur Kristjansson; Droplaug N Magnusdottir; Louise le Roux; Gudrun Sigmundsdottir; Gardar Sveinbjornsson; Kristin E Sveinsdottir; Maney Sveinsdottir; Emil A Thorarensen; Bjarni Thorbjornsson; Gisli Masson; Ingileif Jonsdottir; Alma Moller; Thorolfur Gudnason; Karl G Kristinsson; Unnur Thorsteinsdottir; Kari Stefansson
EPI_ISL_424440 hCoV-19/Iceland/416/2020 Europe / Iceland / Reykjavik 2020-03-19 The National University Hospital of Iceland deCODE genetics Daniel F Gudbjartsson; Agnar Helgason; Hakon Jonsson; Olafur T Magnusson; Pall Melsted; Gudmundur L Norddahl; Jona Saemundsdottir; Asgeir Sigurdsson; Patrick Sulem; Arna B Agustsdottir; Berglind Eiriksdottir; Run Fridriksdottir; Elisabet E Gardarsdottir; Gudmundur Georgsson; Olafia S Gretarsdottir; Kjartan R Gudmundsson; Thora R Gunnarsdottir; Arnaldur Gylfason; Hilma Holm; Brynjar O Jensson; Aslaug Jonasdottir; Kamilla S Josefsdottir; Thordur Kristjansson; Droplaug N Magnusdottir; Louise le Roux; Gudrun Sigmundsdottir; Gardar Sveinbjornsson; Kristin E Sveinsdottir; Maney Sveinsdottir; Emil A Thorarensen; Bjarni Thorbjornsson; Gisli Masson; Ingileif Jonsdottir; Alma Moller; Thorolfur Gudnason; Karl G Kristinsson; Unnur Thorsteinsdottir; Kari Stefansson
EPI_ISL_424441 hCoV-19/Iceland/417/2020 Europe / Iceland / Reykjavik 2020-03-19 The National University Hospital of Iceland deCODE genetics Daniel F Gudbjartsson; Agnar Helgason; Hakon Jonsson; Olafur T Magnusson; Pall Melsted; Gudmundur L Norddahl; Jona Saemundsdottir; Asgeir Sigurdsson; Patrick Sulem; Arna B Agustsdottir; Berglind Eiriksdottir; Run Fridriksdottir; Elisabet E Gardarsdottir; Gudmundur Georgsson; Olafia S Gretarsdottir; Kjartan R Gudmundsson; Thora R Gunnarsdottir; Arnaldur Gylfason; Hilma Holm; Brynjar O Jensson; Aslaug Jonasdottir; Kamilla S Josefsdottir; Thordur Kristjansson; Droplaug N Magnusdottir; Louise le Roux; Gudrun Sigmundsdottir; Gardar Sveinbjornsson; Kristin E Sveinsdottir; Maney Sveinsdottir; Emil A Thorarensen; Bjarni Thorbjornsson; Gisli Masson; Ingileif Jonsdottir; Alma Moller; Thorolfur Gudnason; Karl G Kristinsson; Unnur Thorsteinsdottir; Kari Stefansson
EPI_ISL_424442 hCoV-19/Iceland/418/2020 Europe / Iceland / Reykjavik 2020-03-19 The National University Hospital of Iceland deCODE genetics Daniel F Gudbjartsson; Agnar Helgason; Hakon Jonsson; Olafur T Magnusson; Pall Melsted; Gudmundur L Norddahl; Jona Saemundsdottir; Asgeir Sigurdsson; Patrick Sulem; Arna B Agustsdottir; Berglind Eiriksdottir; Run Fridriksdottir; Elisabet E Gardarsdottir; Gudmundur Georgsson; Olafia S Gretarsdottir; Kjartan R Gudmundsson; Thora R Gunnarsdottir; Arnaldur Gylfason; Hilma Holm; Brynjar O Jensson; Aslaug Jonasdottir; Kamilla S Josefsdottir; Thordur Kristjansson; Droplaug N Magnusdottir; Louise le Roux; Gudrun Sigmundsdottir; Gardar Sveinbjornsson; Kristin E Sveinsdottir; Maney Sveinsdottir; Emil A Thorarensen; Bjarni Thorbjornsson; Gisli Masson; Ingileif Jonsdottir; Alma Moller; Thorolfur Gudnason; Karl G Kristinsson; Unnur Thorsteinsdottir; Kari Stefansson
EPI_ISL_424443 hCoV-19/Iceland/419/2020 Europe / Iceland / Reykjavik 2020-03-19 The National University Hospital of Iceland deCODE genetics Daniel F Gudbjartsson; Agnar Helgason; Hakon Jonsson; Olafur T Magnusson; Pall Melsted; Gudmundur L Norddahl; Jona Saemundsdottir; Asgeir Sigurdsson; Patrick Sulem; Arna B Agustsdottir; Berglind Eiriksdottir; Run Fridriksdottir; Elisabet E Gardarsdottir; Gudmundur Georgsson; Olafia S Gretarsdottir; Kjartan R Gudmundsson; Thora R Gunnarsdottir; Arnaldur Gylfason; Hilma Holm; Brynjar O Jensson; Aslaug Jonasdottir; Kamilla S Josefsdottir; Thordur Kristjansson; Droplaug N Magnusdottir; Louise le Roux; Gudrun Sigmundsdottir; Gardar Sveinbjornsson; Kristin E Sveinsdottir; Maney Sveinsdottir; Emil A Thorarensen; Bjarni Thorbjornsson; Gisli Masson; Ingileif Jonsdottir; Alma Moller; Thorolfur Gudnason; Karl G Kristinsson; Unnur Thorsteinsdottir; Kari Stefansson
EPI_ISL_424445 hCoV-19/Iceland/421/2020 Europe / Iceland / Reykjavik 2020-03-19 The National University Hospital of Iceland deCODE genetics Daniel F Gudbjartsson; Agnar Helgason; Hakon Jonsson; Olafur T Magnusson; Pall Melsted; Gudmundur L Norddahl; Jona Saemundsdottir; Asgeir Sigurdsson; Patrick Sulem; Arna B Agustsdottir; Berglind Eiriksdottir; Run Fridriksdottir; Elisabet E Gardarsdottir; Gudmundur Georgsson; Olafia S Gretarsdottir; Kjartan R Gudmundsson; Thora R Gunnarsdottir; Arnaldur Gylfason; Hilma Holm; Brynjar O Jensson; Aslaug Jonasdottir; Kamilla S Josefsdottir; Thordur Kristjansson; Droplaug N Magnusdottir; Louise le Roux; Gudrun Sigmundsdottir; Gardar Sveinbjornsson; Kristin E Sveinsdottir; Maney Sveinsdottir; Emil A Thorarensen; Bjarni Thorbjornsson; Gisli Masson; Ingileif Jonsdottir; Alma Moller; Thorolfur Gudnason; Karl G Kristinsson; Unnur Thorsteinsdottir; Kari Stefansson
EPI_ISL_424446 hCoV-19/Iceland/422/2020 Europe / Iceland / Reykjavik 2020-03-19 The National University Hospital of Iceland deCODE genetics Daniel F Gudbjartsson; Agnar Helgason; Hakon Jonsson; Olafur T Magnusson; Pall Melsted; Gudmundur L Norddahl; Jona Saemundsdottir; Asgeir Sigurdsson; Patrick Sulem; Arna B Agustsdottir; Berglind Eiriksdottir; Run Fridriksdottir; Elisabet E Gardarsdottir; Gudmundur Georgsson; Olafia S Gretarsdottir; Kjartan R Gudmundsson; Thora R Gunnarsdottir; Arnaldur Gylfason; Hilma Holm; Brynjar O Jensson; Aslaug Jonasdottir; Kamilla S Josefsdottir; Thordur Kristjansson; Droplaug N Magnusdottir; Louise le Roux; Gudrun Sigmundsdottir; Gardar Sveinbjornsson; Kristin E Sveinsdottir; Maney Sveinsdottir; Emil A Thorarensen; Bjarni Thorbjornsson; Gisli Masson; Ingileif Jonsdottir; Alma Moller; Thorolfur Gudnason; Karl G Kristinsson; Unnur Thorsteinsdottir; Kari Stefansson
EPI_ISL_424447 hCoV-19/Iceland/423/2020 Europe / Iceland / Reykjavik 2020-03-19 The National University Hospital of Iceland deCODE genetics Daniel F Gudbjartsson; Agnar Helgason; Hakon Jonsson; Olafur T Magnusson; Pall Melsted; Gudmundur L Norddahl; Jona Saemundsdottir; Asgeir Sigurdsson; Patrick Sulem; Arna B Agustsdottir; Berglind Eiriksdottir; Run Fridriksdottir; Elisabet E Gardarsdottir; Gudmundur Georgsson; Olafia S Gretarsdottir; Kjartan R Gudmundsson; Thora R Gunnarsdottir; Arnaldur Gylfason; Hilma Holm; Brynjar O Jensson; Aslaug Jonasdottir; Kamilla S Josefsdottir; Thordur Kristjansson; Droplaug N Magnusdottir; Louise le

Roux; Gudrun Sigmundsdottir; Gardar Sveinbjornsson; Kristin E Sveinsdottir; Maney Sveinsdottir; Emil A Thorarensen; Bjarni Thorbjornsson; Gisli Masson; Ingileif Jonsdottir; Alma Moller; Thorolfur Gudnason; Karl G Kristinsson; Unnur Thorsteinsdottir; Kari Stefansson
EPI_ISL_424448 hCoV-19/Iceland/424/2020 Europe / Iceland / Reykjavik 2020-03-20 The National University Hospital of Iceland deCODE genetics Daniel F Gudbjartsson; Agnar Helgason; Hakon Jonsson; Olafur T Magnusson; Pall Melsted; Gudmundur L Norddahl; Jona Saemundsdottir; Asgeir Sigurdsson; Patrick Sulem; Arna B Agustsdottir; Berglind Eiriksdottir; Run Fridriksdottir; Elisabet E Gardarsdottir; Gudmundur Georgsson; Olafia S Gretarsdottir; Kjartan R Gudmundsson; Thora R Gunnarsdottir; Arnaldur Gylfason; Hilma Holm; Brynjar O Jensson; Aslaug Jonasdottir; Kamilla S Josefsdottir; Thordur Kristjansson; Droplaug N Magnusdottir; Louise le Roux; Gudrun Sigmundsdottir; Gardar Sveinbjornsson; Kristin E Sveinsdottir; Maney Sveinsdottir; Emil A Thorarensen; Bjarni Thorbjornsson; Gisli Masson; Ingileif Jonsdottir; Alma Moller; Thorolfur Gudnason; Karl G Kristinsson; Unnur Thorsteinsdottir; Kari Stefansson
EPI_ISL_424449 hCoV-19/Iceland/425/2020 Europe / Iceland / Reykjavik 2020-03-20 The National University Hospital of Iceland deCODE genetics Daniel F Gudbjartsson; Agnar Helgason; Hakon Jonsson; Olafur T Magnusson; Pall Melsted; Gudmundur L Norddahl; Jona Saemundsdottir; Asgeir Sigurdsson; Patrick Sulem; Arna B Agustsdottir; Berglind Eiriksdottir; Run Fridriksdottir; Elisabet E Gardarsdottir; Gudmundur Georgsson; Olafia S Gretarsdottir; Kjartan R Gudmundsson; Thora R Gunnarsdottir; Arnaldur Gylfason; Hilma Holm; Brynjar O Jensson; Aslaug Jonasdottir; Kamilla S Josefsdottir; Thordur Kristjansson; Droplaug N Magnusdottir; Louise le Roux; Gudrun Sigmundsdottir; Gardar Sveinbjornsson; Kristin E Sveinsdottir; Maney Sveinsdottir; Emil A Thorarensen; Bjarni Thorbjornsson; Gisli Masson; Ingileif Jonsdottir; Alma Moller; Thorolfur Gudnason; Karl G Kristinsson; Unnur Thorsteinsdottir; Kari Stefansson
EPI_ISL_424450 hCoV-19/Iceland/426/2020 Europe / Iceland / Reykjavik 2020-03-20 The National University Hospital of Iceland deCODE genetics Daniel F Gudbjartsson; Agnar Helgason; Hakon Jonsson; Olafur T Magnusson; Pall Melsted; Gudmundur L Norddahl; Jona Saemundsdottir; Asgeir Sigurdsson; Patrick Sulem; Arna B Agustsdottir; Berglind Eiriksdottir; Run Fridriksdottir; Elisabet E Gardarsdottir; Gudmundur Georgsson; Olafia S Gretarsdottir; Kjartan R Gudmundsson; Thora R Gunnarsdottir; Arnaldur Gylfason; Hilma Holm; Brynjar O Jensson; Aslaug Jonasdottir; Kamilla S Josefsdottir; Thordur Kristjansson; Droplaug N Magnusdottir; Louise le Roux; Gudrun Sigmundsdottir; Gardar Sveinbjornsson; Kristin E Sveinsdottir; Maney Sveinsdottir; Emil A Thorarensen; Bjarni Thorbjornsson; Gisli Masson; Ingileif Jonsdottir; Alma Moller; Thorolfur Gudnason; Karl G Kristinsson; Unnur Thorsteinsdottir; Kari Stefansson
EPI_ISL_424451 hCoV-19/Iceland/427/2020 Europe / Iceland / Reykjavik 2020-03-20 The National University Hospital of Iceland deCODE genetics Daniel F Gudbjartsson; Agnar Helgason; Hakon Jonsson; Olafur T Magnusson; Pall Melsted; Gudmundur L Norddahl; Jona Saemundsdottir; Asgeir Sigurdsson; Patrick Sulem; Arna B Agustsdottir; Berglind Eiriksdottir; Run Fridriksdottir; Elisabet E Gardarsdottir; Gudmundur Georgsson; Olafia S Gretarsdottir; Kjartan R Gudmundsson; Thora R Gunnarsdottir; Arnaldur Gylfason; Hilma Holm; Brynjar O Jensson; Aslaug Jonasdottir; Kamilla S Josefsdottir; Thordur Kristjansson; Droplaug N Magnusdottir; Louise le Roux; Gudrun Sigmundsdottir; Gardar Sveinbjornsson; Kristin E Sveinsdottir; Maney Sveinsdottir; Emil A Thorarensen; Bjarni Thorbjornsson; Gisli Masson; Ingileif Jonsdottir; Alma Moller; Thorolfur Gudnason; Karl G Kristinsson; Unnur Thorsteinsdottir; Kari Stefansson
EPI_ISL_424452 hCoV-19/Iceland/428/2020 Europe / Iceland / Reykjavik 2020-03-20 The National University Hospital of Iceland deCODE genetics Daniel F Gudbjartsson; Agnar Helgason; Hakon Jonsson; Olafur T Magnusson; Pall Melsted; Gudmundur L Norddahl; Jona Saemundsdottir; Asgeir Sigurdsson; Patrick Sulem; Arna B Agustsdottir; Berglind Eiriksdottir; Run Fridriksdottir; Elisabet E Gardarsdottir; Gudmundur Georgsson; Olafia S Gretarsdottir; Kjartan R Gudmundsson; Thora R Gunnarsdottir; Arnaldur Gylfason; Hilma Holm; Brynjar O Jensson; Aslaug Jonasdottir; Kamilla S Josefsdottir; Thordur Kristjansson; Droplaug N Magnusdottir; Louise le Roux; Gudrun Sigmundsdottir; Gardar Sveinbjornsson; Kristin E Sveinsdottir; Maney Sveinsdottir; Emil A Thorarensen; Bjarni Thorbjornsson; Gisli Masson; Ingileif Jonsdottir; Alma Moller; Thorolfur Gudnason; Karl G Kristinsson; Unnur Thorsteinsdottir; Kari Stefansson
EPI_ISL_424454 hCoV-19/Iceland/430/2020 Europe / Iceland / Reykjavik 2020-03-20 The National University Hospital of Iceland deCODE genetics Daniel F Gudbjartsson; Agnar Helgason; Hakon Jonsson; Olafur T Magnusson; Pall Melsted; Gudmundur L Norddahl; Jona Saemundsdottir; Asgeir Sigurdsson; Patrick Sulem; Arna B Agustsdottir; Berglind Eiriksdottir; Run Fridriksdottir; Elisabet E Gardarsdottir; Gudmundur Georgsson; Olafia S Gretarsdottir; Kjartan R Gudmundsson; Thora R Gunnarsdottir; Arnaldur Gylfason; Hilma Holm; Brynjar O Jensson; Aslaug Jonasdottir; Kamilla S Josefsdottir; Thordur Kristjansson; Droplaug N Magnusdottir; Louise le Roux; Gudrun Sigmundsdottir; Gardar Sveinbjornsson; Kristin E Sveinsdottir; Maney Sveinsdottir; Emil A Thorarensen; Bjarni Thorbjornsson; Gisli Masson; Ingileif Jonsdottir; Alma Moller; Thorolfur Gudnason; Karl G Kristinsson; Unnur Thorsteinsdottir; Kari Stefansson
EPI_ISL_424455 hCoV-19/Iceland/431/2020 Europe / Iceland / Reykjavik 2020-03-20 The National University Hospital of Iceland deCODE genetics Daniel F Gudbjartsson; Agnar Helgason; Hakon Jonsson; Olafur T Magnusson; Pall Melsted; Gudmundur L Norddahl; Jona Saemundsdottir; Asgeir Sigurdsson; Patrick Sulem; Arna B Agustsdottir; Berglind Eiriksdottir; Run Fridriksdottir; Elisabet E Gardarsdottir; Gudmundur Georgsson; Olafia S Gretarsdottir; Kjartan R Gudmundsson; Thora R Gunnarsdottir; Arnaldur Gylfason; Hilma Holm; Brynjar O Jensson; Aslaug Jonasdottir; Kamilla S Josefsdottir; Thordur Kristjansson; Droplaug N Magnusdottir; Louise le Roux; Gudrun Sigmundsdottir; Gardar Sveinbjornsson; Kristin E Sveinsdottir; Maney Sveinsdottir; Emil A Thorarensen; Bjarni Thorbjornsson; Gisli Masson; Ingileif Jonsdottir; Alma Moller; Thorolfur Gudnason; Karl G Kristinsson; Unnur Thorsteinsdottir; Kari Stefansson
EPI_ISL_424456 hCoV-19/Iceland/432/2020 Europe / Iceland / Reykjavik 2020-03-20 The National University Hospital of Iceland deCODE genetics Daniel F Gudbjartsson; Agnar Helgason; Hakon Jonsson; Olafur T Magnusson; Pall Melsted; Gudmundur L Norddahl; Jona Saemundsdottir; Asgeir Sigurdsson; Patrick Sulem; Arna B Agustsdottir; Berglind Eiriksdottir; Run Fridriksdottir; Elisabet E Gardarsdottir; Gudmundur Georgsson; Olafia

S Gretarsdottir; Kjartan R Gudmundsson; Thora R Gunnarsdottir; Arnaldur Gylfason; Hilma Holm; Brynjar O Jensson; Aslaug Jonasdottir; Kamilla S Josefsdottir; Thordur Kristjansson; Droplaug N Magnusdottir; Louise le Roux; Gudrun Sigmundsdottir; Gardar Sveinbjornsson; Kristin E Sveinsdottir; Maney Sveinsdottir; Emil A Thorarensen; Bjarni Thorbjornsson; Gisli Masson; Ingileif Jonsdottir; Alma Moller; Thorolfur Gudnason; Karl G Kristinsson; Unnur Thorsteinsdottir; Kari Stefansson
EPI_ISL_424457 hCoV-19/Iceland/433/2020 Europe / Iceland / Reykjavik 2020-03-20 The National University Hospital of Iceland deCODE genetics Daniel F Gudbjartsson; Agnar Helgason; Hakon Jonsson; Olafur T Magnusson; Pall Melsted; Gudmundur L Norddahl; Jona Saemundsdottir; Asgeir Sigurdsson; Patrick Sulem; Arna B Agustsdottir; Berglind Eiriksdottir; Run Fridriksdottir; Elisabet E Gardarsdottir; Gudmundur Georgsson; Olafia S Gretarsdottir; Kjartan R Gudmundsson; Thora R Gunnarsdottir; Arnaldur Gylfason; Hilma Holm; Brynjar O Jensson; Aslaug Jonasdottir; Kamilla S Josefsdottir; Thordur Kristjansson; Droplaug N Magnusdottir; Louise le Roux; Gudrun Sigmundsdottir; Gardar Sveinbjornsson; Kristin E Sveinsdottir; Maney Sveinsdottir; Emil A Thorarensen; Bjarni Thorbjornsson; Gisli Masson; Ingileif Jonsdottir; Alma Moller; Thorolfur Gudnason; Karl G Kristinsson; Unnur Thorsteinsdottir; Kari Stefansson
EPI_ISL_424459 hCoV-19/Iceland/435/2020 Europe / Iceland / Reykjavik 2020-03-20 The National University Hospital of Iceland deCODE genetics Daniel F Gudbjartsson; Agnar Helgason; Hakon Jonsson; Olafur T Magnusson; Pall Melsted; Gudmundur L Norddahl; Jona Saemundsdottir; Asgeir Sigurdsson; Patrick Sulem; Arna B Agustsdottir; Berglind Eiriksdottir; Run Fridriksdottir; Elisabet E Gardarsdottir; Gudmundur Georgsson; Olafia S Gretarsdottir; Kjartan R Gudmundsson; Thora R Gunnarsdottir; Arnaldur Gylfason; Hilma Holm; Brynjar O Jensson; Aslaug Jonasdottir; Kamilla S Josefsdottir; Thordur Kristjansson; Droplaug N Magnusdottir; Louise le Roux; Gudrun Sigmundsdottir; Gardar Sveinbjornsson; Kristin E Sveinsdottir; Maney Sveinsdottir; Emil A Thorarensen; Bjarni Thorbjornsson; Gisli Masson; Ingileif Jonsdottir; Alma Moller; Thorolfur Gudnason; Karl G Kristinsson; Unnur Thorsteinsdottir; Kari Stefansson
EPI_ISL_424460 hCoV-19/Iceland/436/2020 Europe / Iceland / Reykjavik 2020-03-20 The National University Hospital of Iceland deCODE genetics Daniel F Gudbjartsson; Agnar Helgason; Hakon Jonsson; Olafur T Magnusson; Pall Melsted; Gudmundur L Norddahl; Jona Saemundsdottir; Asgeir Sigurdsson; Patrick Sulem; Arna B Agustsdottir; Berglind Eiriksdottir; Run Fridriksdottir; Elisabet E Gardarsdottir; Gudmundur Georgsson; Olafia S Gretarsdottir; Kjartan R Gudmundsson; Thora R Gunnarsdottir; Arnaldur Gylfason; Hilma Holm; Brynjar O Jensson; Aslaug Jonasdottir; Kamilla S Josefsdottir; Thordur Kristjansson; Droplaug N Magnusdottir; Louise le Roux; Gudrun Sigmundsdottir; Gardar Sveinbjornsson; Kristin E Sveinsdottir; Maney Sveinsdottir; Emil A Thorarensen; Bjarni Thorbjornsson; Gisli Masson; Ingileif Jonsdottir; Alma Moller; Thorolfur Gudnason; Karl G Kristinsson; Unnur Thorsteinsdottir; Kari Stefansson
EPI_ISL_424461 hCoV-19/Iceland/437/2020 Europe / Iceland / Reykjavik 2020-03-20 The National University Hospital of Iceland deCODE genetics Daniel F Gudbjartsson; Agnar Helgason; Hakon Jonsson; Olafur T Magnusson; Pall Melsted; Gudmundur L Norddahl; Jona Saemundsdottir; Asgeir Sigurdsson; Patrick Sulem; Arna B Agustsdottir; Berglind Eiriksdottir; Run Fridriksdottir; Elisabet E Gardarsdottir; Gudmundur Georgsson; Olafia S Gretarsdottir; Kjartan R Gudmundsson; Thora R Gunnarsdottir; Arnaldur Gylfason; Hilma Holm; Brynjar O Jensson; Aslaug Jonasdottir; Kamilla S Josefsdottir; Thordur Kristjansson; Droplaug N Magnusdottir; Louise le Roux; Gudrun Sigmundsdottir; Gardar Sveinbjornsson; Kristin E Sveinsdottir; Maney Sveinsdottir; Emil A Thorarensen; Bjarni Thorbjornsson; Gisli Masson; Ingileif Jonsdottir; Alma Moller; Thorolfur Gudnason; Karl G Kristinsson; Unnur Thorsteinsdottir; Kari Stefansson
EPI_ISL_424462 hCoV-19/Iceland/438/2020 Europe / Iceland / Reykjavik 2020-03-20 The National University Hospital of Iceland deCODE genetics Daniel F Gudbjartsson; Agnar Helgason; Hakon Jonsson; Olafur T Magnusson; Pall Melsted; Gudmundur L Norddahl; Jona Saemundsdottir; Asgeir Sigurdsson; Patrick Sulem; Arna B Agustsdottir; Berglind Eiriksdottir; Run Fridriksdottir; Elisabet E Gardarsdottir; Gudmundur Georgsson; Olafia S Gretarsdottir; Kjartan R Gudmundsson; Thora R Gunnarsdottir; Arnaldur Gylfason; Hilma Holm; Brynjar O Jensson; Aslaug Jonasdottir; Kamilla S Josefsdottir; Thordur Kristjansson; Droplaug N Magnusdottir; Louise le Roux; Gudrun Sigmundsdottir; Gardar Sveinbjornsson; Kristin E Sveinsdottir; Maney Sveinsdottir; Emil A Thorarensen; Bjarni Thorbjornsson; Gisli Masson; Ingileif Jonsdottir; Alma Moller; Thorolfur Gudnason; Karl G Kristinsson; Unnur Thorsteinsdottir; Kari Stefansson
EPI_ISL_424463 hCoV-19/Iceland/439/2020 Europe / Iceland / Reykjavik 2020-03-20 The National University Hospital of Iceland deCODE genetics Daniel F Gudbjartsson; Agnar Helgason; Hakon Jonsson; Olafur T Magnusson; Pall Melsted; Gudmundur L Norddahl; Jona Saemundsdottir; Asgeir Sigurdsson; Patrick Sulem; Arna B Agustsdottir; Berglind Eiriksdottir; Run Fridriksdottir; Elisabet E Gardarsdottir; Gudmundur Georgsson; Olafia S Gretarsdottir; Kjartan R Gudmundsson; Thora R Gunnarsdottir; Arnaldur Gylfason; Hilma Holm; Brynjar O Jensson; Aslaug Jonasdottir; Kamilla S Josefsdottir; Thordur Kristjansson; Droplaug N Magnusdottir; Louise le Roux; Gudrun Sigmundsdottir; Gardar Sveinbjornsson; Kristin E Sveinsdottir; Maney Sveinsdottir; Emil A Thorarensen; Bjarni Thorbjornsson; Gisli Masson; Ingileif Jonsdottir; Alma Moller; Thorolfur Gudnason; Karl G Kristinsson; Unnur Thorsteinsdottir; Kari Stefansson
EPI_ISL_424465 hCoV-19/Iceland/441/2020 Europe / Iceland / Reykjavik 2020-03-20 The National University Hospital of Iceland deCODE genetics Daniel F Gudbjartsson; Agnar Helgason; Hakon Jonsson; Olafur T Magnusson; Pall Melsted; Gudmundur L Norddahl; Jona Saemundsdottir; Asgeir Sigurdsson; Patrick Sulem; Arna B Agustsdottir; Berglind Eiriksdottir; Run Fridriksdottir; Elisabet E Gardarsdottir; Gudmundur Georgsson; Olafia S Gretarsdottir; Kjartan R Gudmundsson; Thora R Gunnarsdottir; Arnaldur Gylfason; Hilma Holm; Brynjar O Jensson; Aslaug Jonasdottir; Kamilla S Josefsdottir; Thordur Kristjansson; Droplaug N Magnusdottir; Louise le Roux; Gudrun Sigmundsdottir; Gardar Sveinbjornsson; Kristin E Sveinsdottir; Maney Sveinsdottir; Emil A Thorarensen; Bjarni Thorbjornsson; Gisli Masson; Ingileif Jonsdottir; Alma Moller; Thorolfur Gudnason; Karl G Kristinsson; Unnur Thorsteinsdottir; Kari Stefansson
EPI_ISL_424466 hCoV-19/Iceland/442/2020 Europe / Iceland / Reykjavik 2020-03-20 The National University Hospital of Iceland deCODE genetics Daniel F Gudbjartsson; Agnar Helgason; Hakon Jonsson; Olafur T

Magnusson; Pall Melsted; Gudmundur L Norddahl; Jona Saemundsdottir; Asgeir Sigurdsson; Patrick Sulem; Arna B Agustsdottir; Berglind Eiriksdottir; Run Fridriksdottir; Elisabet E Gardarsdottir; Gudmundur Georgsson; Olafia S Gretarsdottir; Kjartan R Gudmundsson; Thora R Gunnarsdottir; Arnaldur Gylfason; Hilma Holm; Brynjar O Jensson; Aslaug Jonasdottir; Kamilla S Josefsdottir; Thordur Kristjansson; Droplaug N Magnusdottir; Louise le Roux; Gudrun Sigmundsdottir; Gardar Sveinbjornsson; Kristin E Sveinsdottir; Maney Sveinsdottir; Emil A Thorarensen; Bjarni Thorbjornsson; Gisli Masson; Ingileif Jonsdottir; Alma Moller; Thorolfur Gudnason; Karl G Kristinsson; Unnur Thorsteinsdottir; Kari Stefansson
EPI_ISL_424467 hCoV-19/Iceland/443/2020 Europe / Iceland / Reykjavik 2020-03-20 The National University Hospital of Iceland deCODE genetics Daniel F Gudbjartsson; Agnar Helgason; Hakon Jonsson; Olafur T Magnusson; Pall Melsted; Gudmundur L Norddahl; Jona Saemundsdottir; Asgeir Sigurdsson; Patrick Sulem; Arna B Agustsdottir; Berglind Eiriksdottir; Run Fridriksdottir; Elisabet E Gardarsdottir; Gudmundur Georgsson; Olafia S Gretarsdottir; Kjartan R Gudmundsson; Thora R Gunnarsdottir; Arnaldur Gylfason; Hilma Holm; Brynjar O Jensson; Aslaug Jonasdottir; Kamilla S Josefsdottir; Thordur Kristjansson; Droplaug N Magnusdottir; Louise le Roux; Gudrun Sigmundsdottir; Gardar Sveinbjornsson; Kristin E Sveinsdottir; Maney Sveinsdottir; Emil A Thorarensen; Bjarni Thorbjornsson; Gisli Masson; Ingileif Jonsdottir; Alma Moller; Thorolfur Gudnason; Karl G Kristinsson; Unnur Thorsteinsdottir; Kari Stefansson
EPI_ISL_424468 hCoV-19/Iceland/444/2020 Europe / Iceland / Reykjavik 2020-03-20 The National University Hospital of Iceland deCODE genetics Daniel F Gudbjartsson; Agnar Helgason; Hakon Jonsson; Olafur T Magnusson; Pall Melsted; Gudmundur L Norddahl; Jona Saemundsdottir; Asgeir Sigurdsson; Patrick Sulem; Arna B Agustsdottir; Berglind Eiriksdottir; Run Fridriksdottir; Elisabet E Gardarsdottir; Gudmundur Georgsson; Olafia S Gretarsdottir; Kjartan R Gudmundsson; Thora R Gunnarsdottir; Arnaldur Gylfason; Hilma Holm; Brynjar O Jensson; Aslaug Jonasdottir; Kamilla S Josefsdottir; Thordur Kristjansson; Droplaug N Magnusdottir; Louise le Roux; Gudrun Sigmundsdottir; Gardar Sveinbjornsson; Kristin E Sveinsdottir; Maney Sveinsdottir; Emil A Thorarensen; Bjarni Thorbjornsson; Gisli Masson; Ingileif Jonsdottir; Alma Moller; Thorolfur Gudnason; Karl G Kristinsson; Unnur Thorsteinsdottir; Kari Stefansson
EPI_ISL_424469 hCoV-19/Iceland/445/2020 Europe / Iceland / Reykjavik 2020-03-20 The National University Hospital of Iceland deCODE genetics Daniel F Gudbjartsson; Agnar Helgason; Hakon Jonsson; Olafur T Magnusson; Pall Melsted; Gudmundur L Norddahl; Jona Saemundsdottir; Asgeir Sigurdsson; Patrick Sulem; Arna B Agustsdottir; Berglind Eiriksdottir; Run Fridriksdottir; Elisabet E Gardarsdottir; Gudmundur Georgsson; Olafia S Gretarsdottir; Kjartan R Gudmundsson; Thora R Gunnarsdottir; Arnaldur Gylfason; Hilma Holm; Brynjar O Jensson; Aslaug Jonasdottir; Kamilla S Josefsdottir; Thordur Kristjansson; Droplaug N Magnusdottir; Louise le Roux; Gudrun Sigmundsdottir; Gardar Sveinbjornsson; Kristin E Sveinsdottir; Maney Sveinsdottir; Emil A Thorarensen; Bjarni Thorbjornsson; Gisli Masson; Ingileif Jonsdottir; Alma Moller; Thorolfur Gudnason; Karl G Kristinsson; Unnur Thorsteinsdottir; Kari Stefansson
EPI_ISL_424470 hCoV-19/Iceland/446/2020 Europe / Iceland / Reykjavik 2020-03-20 The National University Hospital of Iceland deCODE genetics Daniel F Gudbjartsson; Agnar Helgason; Hakon Jonsson; Olafur T Magnusson; Pall Melsted; Gudmundur L Norddahl; Jona Saemundsdottir; Asgeir Sigurdsson; Patrick Sulem; Arna B Agustsdottir; Berglind Eiriksdottir; Run Fridriksdottir; Elisabet E Gardarsdottir; Gudmundur Georgsson; Olafia S Gretarsdottir; Kjartan R Gudmundsson; Thora R Gunnarsdottir; Arnaldur Gylfason; Hilma Holm; Brynjar O Jensson; Aslaug Jonasdottir; Kamilla S Josefsdottir; Thordur Kristjansson; Droplaug N Magnusdottir; Louise le Roux; Gudrun Sigmundsdottir; Gardar Sveinbjornsson; Kristin E Sveinsdottir; Maney Sveinsdottir; Emil A Thorarensen; Bjarni Thorbjornsson; Gisli Masson; Ingileif Jonsdottir; Alma Moller; Thorolfur Gudnason; Karl G Kristinsson; Unnur Thorsteinsdottir; Kari Stefansson
EPI_ISL_424471 hCoV-19/Iceland/447/2020 Europe / Iceland / Reykjavik 2020-03-20 The National University Hospital of Iceland deCODE genetics Daniel F Gudbjartsson; Agnar Helgason; Hakon Jonsson; Olafur T Magnusson; Pall Melsted; Gudmundur L Norddahl; Jona Saemundsdottir; Asgeir Sigurdsson; Patrick Sulem; Arna B Agustsdottir; Berglind Eiriksdottir; Run Fridriksdottir; Elisabet E Gardarsdottir; Gudmundur Georgsson; Olafia S Gretarsdottir; Kjartan R Gudmundsson; Thora R Gunnarsdottir; Arnaldur Gylfason; Hilma Holm; Brynjar O Jensson; Aslaug Jonasdottir; Kamilla S Josefsdottir; Thordur Kristjansson; Droplaug N Magnusdottir; Louise le Roux; Gudrun Sigmundsdottir; Gardar Sveinbjornsson; Kristin E Sveinsdottir; Maney Sveinsdottir; Emil A Thorarensen; Bjarni Thorbjornsson; Gisli Masson; Ingileif Jonsdottir; Alma Moller; Thorolfur Gudnason; Karl G Kristinsson; Unnur Thorsteinsdottir; Kari Stefansson
EPI_ISL_424472 hCoV-19/Iceland/448/2020 Europe / Iceland / Reykjavik 2020-03-20 The National University Hospital of Iceland deCODE genetics Daniel F Gudbjartsson; Agnar Helgason; Hakon Jonsson; Olafur T Magnusson; Pall Melsted; Gudmundur L Norddahl; Jona Saemundsdottir; Asgeir Sigurdsson; Patrick Sulem; Arna B Agustsdottir; Berglind Eiriksdottir; Run Fridriksdottir; Elisabet E Gardarsdottir; Gudmundur Georgsson; Olafia S Gretarsdottir; Kjartan R Gudmundsson; Thora R Gunnarsdottir; Arnaldur Gylfason; Hilma Holm; Brynjar O Jensson; Aslaug Jonasdottir; Kamilla S Josefsdottir; Thordur Kristjansson; Droplaug N Magnusdottir; Louise le Roux; Gudrun Sigmundsdottir; Gardar Sveinbjornsson; Kristin E Sveinsdottir; Maney Sveinsdottir; Emil A Thorarensen; Bjarni Thorbjornsson; Gisli Masson; Ingileif Jonsdottir; Alma Moller; Thorolfur Gudnason; Karl G Kristinsson; Unnur Thorsteinsdottir; Kari Stefansson
EPI_ISL_424473 hCoV-19/Iceland/449/2020 Europe / Iceland / Reykjavik 2020-03-20 The National University Hospital of Iceland deCODE genetics Daniel F Gudbjartsson; Agnar Helgason; Hakon Jonsson; Olafur T Magnusson; Pall Melsted; Gudmundur L Norddahl; Jona Saemundsdottir; Asgeir Sigurdsson; Patrick Sulem; Arna B Agustsdottir; Berglind Eiriksdottir; Run Fridriksdottir; Elisabet E Gardarsdottir; Gudmundur Georgsson; Olafia S Gretarsdottir; Kjartan R Gudmundsson; Thora R Gunnarsdottir; Arnaldur Gylfason; Hilma Holm; Brynjar O Jensson; Aslaug Jonasdottir; Kamilla S Josefsdottir; Thordur Kristjansson; Droplaug N Magnusdottir; Louise le Roux; Gudrun Sigmundsdottir; Gardar Sveinbjornsson; Kristin E Sveinsdottir; Maney Sveinsdottir; Emil A Thorarensen; Bjarni Thorbjornsson; Gisli Masson; Ingileif Jonsdottir; Alma Moller; Thorolfur Gudnason; Karl G Kristinsson; Unnur Thorsteinsdottir; Kari Stefansson

EPI_ISL_424474 hCoV-19/Iceland/450/2020 Europe / Iceland / Reykjavik 2020-03-20 The National University Hospital of Iceland deCODE genetics Daniel F Gudbjartsson; Agnar Helgason; Hakon Jonsson; Olafur T Magnusson; Pall Melsted; Gudmundur L Norddahl; Jona Saemundsdottir; Asgeir Sigurdsson; Patrick Sulem; Arna B Agustsdottir; Berglind Eiriksdottir; Run Fridriksdottir; Elisabet E Gardarsdottir; Gudmundur Georgsson; Olafia S Gretarsdottir; Kjartan R Gudmundsson; Thora R Gunnarsdottir; Arnaldur Gylfason; Hilma Holm; Brynjar O Jensson; Aslaug Jonasdottir; Kamilla S Josefsdottir; Thordur Kristjansson; Droplaug N Magnusdottir; Louise le Roux; Gudrun Sigmundsdottir; Gardar Sveinbjornsson; Kristin E Sveinsdottir; Maney Sveinsdottir; Emil A Thorarensen; Bjarni Thorbjornsson; Gisli Masson; Ingileif Jonsdottir; Alma Moller; Thorolfur Gudnason; Karl G Kristinsson; Unnur Thorsteinsdottir; Kari Stefansson
EPI_ISL_424475 hCoV-19/Iceland/451/2020 Europe / Iceland / Reykjavik 2020-03-20 The National University Hospital of Iceland deCODE genetics Daniel F Gudbjartsson; Agnar Helgason; Hakon Jonsson; Olafur T Magnusson; Pall Melsted; Gudmundur L Norddahl; Jona Saemundsdottir; Asgeir Sigurdsson; Patrick Sulem; Arna B Agustsdottir; Berglind Eiriksdottir; Run Fridriksdottir; Elisabet E Gardarsdottir; Gudmundur Georgsson; Olafia S Gretarsdottir; Kjartan R Gudmundsson; Thora R Gunnarsdottir; Arnaldur Gylfason; Hilma Holm; Brynjar O Jensson; Aslaug Jonasdottir; Kamilla S Josefsdottir; Thordur Kristjansson; Droplaug N Magnusdottir; Louise le Roux; Gudrun Sigmundsdottir; Gardar Sveinbjornsson; Kristin E Sveinsdottir; Maney Sveinsdottir; Emil A Thorarensen; Bjarni Thorbjornsson; Gisli Masson; Ingileif Jonsdottir; Alma Moller; Thorolfur Gudnason; Karl G Kristinsson; Unnur Thorsteinsdottir; Kari Stefansson
EPI_ISL_424476 hCoV-19/Iceland/452/2020 Europe / Iceland / Reykjavik 2020-03-19 The National University Hospital of Iceland deCODE genetics Daniel F Gudbjartsson; Agnar Helgason; Hakon Jonsson; Olafur T Magnusson; Pall Melsted; Gudmundur L Norddahl; Jona Saemundsdottir; Asgeir Sigurdsson; Patrick Sulem; Arna B Agustsdottir; Berglind Eiriksdottir; Run Fridriksdottir; Elisabet E Gardarsdottir; Gudmundur Georgsson; Olafia S Gretarsdottir; Kjartan R Gudmundsson; Thora R Gunnarsdottir; Arnaldur Gylfason; Hilma Holm; Brynjar O Jensson; Aslaug Jonasdottir; Kamilla S Josefsdottir; Thordur Kristjansson; Droplaug N Magnusdottir; Louise le Roux; Gudrun Sigmundsdottir; Gardar Sveinbjornsson; Kristin E Sveinsdottir; Maney Sveinsdottir; Emil A Thorarensen; Bjarni Thorbjornsson; Gisli Masson; Ingileif Jonsdottir; Alma Moller; Thorolfur Gudnason; Karl G Kristinsson; Unnur Thorsteinsdottir; Kari Stefansson
EPI_ISL_424477 hCoV-19/Iceland/454/2020 Europe / Iceland / Reykjavik 2020-03-20 The National University Hospital of Iceland deCODE genetics Daniel F Gudbjartsson; Agnar Helgason; Hakon Jonsson; Olafur T Magnusson; Pall Melsted; Gudmundur L Norddahl; Jona Saemundsdottir; Asgeir Sigurdsson; Patrick Sulem; Arna B Agustsdottir; Berglind Eiriksdottir; Run Fridriksdottir; Elisabet E Gardarsdottir; Gudmundur Georgsson; Olafia S Gretarsdottir; Kjartan R Gudmundsson; Thora R Gunnarsdottir; Arnaldur Gylfason; Hilma Holm; Brynjar O Jensson; Aslaug Jonasdottir; Kamilla S Josefsdottir; Thordur Kristjansson; Droplaug N Magnusdottir; Louise le Roux; Gudrun Sigmundsdottir; Gardar Sveinbjornsson; Kristin E Sveinsdottir; Maney Sveinsdottir; Emil A Thorarensen; Bjarni Thorbjornsson; Gisli Masson; Ingileif Jonsdottir; Alma Moller; Thorolfur Gudnason; Karl G Kristinsson; Unnur Thorsteinsdottir; Kari Stefansson
EPI_ISL_424478 hCoV-19/Iceland/455/2020 Europe / Iceland / Reykjavik 2020-03-20 The National University Hospital of Iceland deCODE genetics Daniel F Gudbjartsson; Agnar Helgason; Hakon Jonsson; Olafur T Magnusson; Pall Melsted; Gudmundur L Norddahl; Jona Saemundsdottir; Asgeir Sigurdsson; Patrick Sulem; Arna B Agustsdottir; Berglind Eiriksdottir; Run Fridriksdottir; Elisabet E Gardarsdottir; Gudmundur Georgsson; Olafia S Gretarsdottir; Kjartan R Gudmundsson; Thora R Gunnarsdottir; Arnaldur Gylfason; Hilma Holm; Brynjar O Jensson; Aslaug Jonasdottir; Kamilla S Josefsdottir; Thordur Kristjansson; Droplaug N Magnusdottir; Louise le Roux; Gudrun Sigmundsdottir; Gardar Sveinbjornsson; Kristin E Sveinsdottir; Maney Sveinsdottir; Emil A Thorarensen; Bjarni Thorbjornsson; Gisli Masson; Ingileif Jonsdottir; Alma Moller; Thorolfur Gudnason; Karl G Kristinsson; Unnur Thorsteinsdottir; Kari Stefansson
EPI_ISL_424479 hCoV-19/Iceland/456/2020 Europe / Iceland / Reykjavik 2020-03-20 The National University Hospital of Iceland deCODE genetics Daniel F Gudbjartsson; Agnar Helgason; Hakon Jonsson; Olafur T Magnusson; Pall Melsted; Gudmundur L Norddahl; Jona Saemundsdottir; Asgeir Sigurdsson; Patrick Sulem; Arna B Agustsdottir; Berglind Eiriksdottir; Run Fridriksdottir; Elisabet E Gardarsdottir; Gudmundur Georgsson; Olafia S Gretarsdottir; Kjartan R Gudmundsson; Thora R Gunnarsdottir; Arnaldur Gylfason; Hilma Holm; Brynjar O Jensson; Aslaug Jonasdottir; Kamilla S Josefsdottir; Thordur Kristjansson; Droplaug N Magnusdottir; Louise le Roux; Gudrun Sigmundsdottir; Gardar Sveinbjornsson; Kristin E Sveinsdottir; Maney Sveinsdottir; Emil A Thorarensen; Bjarni Thorbjornsson; Gisli Masson; Ingileif Jonsdottir; Alma Moller; Thorolfur Gudnason; Karl G Kristinsson; Unnur Thorsteinsdottir; Kari Stefansson
EPI_ISL_424480 hCoV-19/Iceland/457/2020 Europe / Iceland / Reykjavik 2020-03-20 The National University Hospital of Iceland deCODE genetics Daniel F Gudbjartsson; Agnar Helgason; Hakon Jonsson; Olafur T Magnusson; Pall Melsted; Gudmundur L Norddahl; Jona Saemundsdottir; Asgeir Sigurdsson; Patrick Sulem; Arna B Agustsdottir; Berglind Eiriksdottir; Run Fridriksdottir; Elisabet E Gardarsdottir; Gudmundur Georgsson; Olafia S Gretarsdottir; Kjartan R Gudmundsson; Thora R Gunnarsdottir; Arnaldur Gylfason; Hilma Holm; Brynjar O Jensson; Aslaug Jonasdottir; Kamilla S Josefsdottir; Thordur Kristjansson; Droplaug N Magnusdottir; Louise le Roux; Gudrun Sigmundsdottir; Gardar Sveinbjornsson; Kristin E Sveinsdottir; Maney Sveinsdottir; Emil A Thorarensen; Bjarni Thorbjornsson; Gisli Masson; Ingileif Jonsdottir; Alma Moller; Thorolfur Gudnason; Karl G Kristinsson; Unnur Thorsteinsdottir; Kari Stefansson
EPI_ISL_424482 hCoV-19/Iceland/459/2020 Europe / Iceland / Reykjavik 2020-03-20 The National University Hospital of Iceland deCODE genetics Daniel F Gudbjartsson; Agnar Helgason; Hakon Jonsson; Olafur T Magnusson; Pall Melsted; Gudmundur L Norddahl; Jona Saemundsdottir; Asgeir Sigurdsson; Patrick Sulem; Arna B Agustsdottir; Berglind Eiriksdottir; Run Fridriksdottir; Elisabet E Gardarsdottir; Gudmundur Georgsson; Olafia S Gretarsdottir; Kjartan R Gudmundsson; Thora R Gunnarsdottir; Arnaldur Gylfason; Hilma Holm; Brynjar O Jensson; Aslaug Jonasdottir; Kamilla S Josefsdottir; Thordur Kristjansson; Droplaug N Magnusdottir; Louise le Roux; Gudrun Sigmundsdottir; Gardar Sveinbjornsson; Kristin E Sveinsdottir; Maney Sveinsdottir; Emil A

Thorarensen; Bjarni Thorbjornsson; Gisli Masson; Ingileif Jonsdottir; Alma Moller; Thorolfur Gudnason; Karl G Kristinsson; Unnur Thorsteinsdottir; Kari Stefansson
EPI_ISL_424483 hCoV-19/Iceland/460/2020 Europe / Iceland / Reykjavik 2020-03-20 The National University Hospital of Iceland deCODE genetics Daniel F Gudbjartsson; Agnar Helgason; Hakon Jonsson; Olafur T Magnusson; Pall Melsted; Gudmundur L Norddahl; Jona Saemundsdottir; Asgeir Sigurdsson; Patrick Sulem; Arna B Agustsdottir; Berglind Eiriksdottir; Run Fridriksdottir; Elisabet E Gardarsdottir; Gudmundur Georgsson; Olafia S Gretarsdottir; Kjartan R Gudmundsson; Thora R Gunnarsdottir; Arnaldur Gylfason; Hilma Holm; Brynjar O Jensson; Aslaug Jonasdottir; Kamilla S Josefsdottir; Thordur Kristjansson; Droplaug N Magnusdottir; Louise le Roux; Gudrun Sigmundsdottir; Gardar Sveinbjornsson; Kristin E Sveinsdottir; Maney Sveinsdottir; Emil A Thorarensen; Bjarni Thorbjornsson; Gisli Masson; Ingileif Jonsdottir; Alma Moller; Thorolfur Gudnason; Karl G Kristinsson; Unnur Thorsteinsdottir; Kari Stefansson
EPI_ISL_424484 hCoV-19/Iceland/461/2020 Europe / Iceland / Reykjavik 2020-03-20 The National University Hospital of Iceland deCODE genetics Daniel F Gudbjartsson; Agnar Helgason; Hakon Jonsson; Olafur T Magnusson; Pall Melsted; Gudmundur L Norddahl; Jona Saemundsdottir; Asgeir Sigurdsson; Patrick Sulem; Arna B Agustsdottir; Berglind Eiriksdottir; Run Fridriksdottir; Elisabet E Gardarsdottir; Gudmundur Georgsson; Olafia S Gretarsdottir; Kjartan R Gudmundsson; Thora R Gunnarsdottir; Arnaldur Gylfason; Hilma Holm; Brynjar O Jensson; Aslaug Jonasdottir; Kamilla S Josefsdottir; Thordur Kristjansson; Droplaug N Magnusdottir; Louise le Roux; Gudrun Sigmundsdottir; Gardar Sveinbjornsson; Kristin E Sveinsdottir; Maney Sveinsdottir; Emil A Thorarensen; Bjarni Thorbjornsson; Gisli Masson; Ingileif Jonsdottir; Alma Moller; Thorolfur Gudnason; Karl G Kristinsson; Unnur Thorsteinsdottir; Kari Stefansson
EPI_ISL_424485 hCoV-19/Iceland/462/2020 Europe / Iceland / Reykjavik 2020-03-20 The National University Hospital of Iceland deCODE genetics Daniel F Gudbjartsson; Agnar Helgason; Hakon Jonsson; Olafur T Magnusson; Pall Melsted; Gudmundur L Norddahl; Jona Saemundsdottir; Asgeir Sigurdsson; Patrick Sulem; Arna B Agustsdottir; Berglind Eiriksdottir; Run Fridriksdottir; Elisabet E Gardarsdottir; Gudmundur Georgsson; Olafia S Gretarsdottir; Kjartan R Gudmundsson; Thora R Gunnarsdottir; Arnaldur Gylfason; Hilma Holm; Brynjar O Jensson; Aslaug Jonasdottir; Kamilla S Josefsdottir; Thordur Kristjansson; Droplaug N Magnusdottir; Louise le Roux; Gudrun Sigmundsdottir; Gardar Sveinbjornsson; Kristin E Sveinsdottir; Maney Sveinsdottir; Emil A Thorarensen; Bjarni Thorbjornsson; Gisli Masson; Ingileif Jonsdottir; Alma Moller; Thorolfur Gudnason; Karl G Kristinsson; Unnur Thorsteinsdottir; Kari Stefansson
EPI_ISL_424486 hCoV-19/Iceland/463/2020 Europe / Iceland / Reykjavik 2020-03-20 The National University Hospital of Iceland deCODE genetics Daniel F Gudbjartsson; Agnar Helgason; Hakon Jonsson; Olafur T Magnusson; Pall Melsted; Gudmundur L Norddahl; Jona Saemundsdottir; Asgeir Sigurdsson; Patrick Sulem; Arna B Agustsdottir; Berglind Eiriksdottir; Run Fridriksdottir; Elisabet E Gardarsdottir; Gudmundur Georgsson; Olafia S Gretarsdottir; Kjartan R Gudmundsson; Thora R Gunnarsdottir; Arnaldur Gylfason; Hilma Holm; Brynjar O Jensson; Aslaug Jonasdottir; Kamilla S Josefsdottir; Thordur Kristjansson; Droplaug N Magnusdottir; Louise le Roux; Gudrun Sigmundsdottir; Gardar Sveinbjornsson; Kristin E Sveinsdottir; Maney Sveinsdottir; Emil A Thorarensen; Bjarni Thorbjornsson; Gisli Masson; Ingileif Jonsdottir; Alma Moller; Thorolfur Gudnason; Karl G Kristinsson; Unnur Thorsteinsdottir; Kari Stefansson
EPI_ISL_424487 hCoV-19/Iceland/464/2020 Europe / Iceland / Reykjavik 2020-03-20 The National University Hospital of Iceland deCODE genetics Daniel F Gudbjartsson; Agnar Helgason; Hakon Jonsson; Olafur T Magnusson; Pall Melsted; Gudmundur L Norddahl; Jona Saemundsdottir; Asgeir Sigurdsson; Patrick Sulem; Arna B Agustsdottir; Berglind Eiriksdottir; Run Fridriksdottir; Elisabet E Gardarsdottir; Gudmundur Georgsson; Olafia S Gretarsdottir; Kjartan R Gudmundsson; Thora R Gunnarsdottir; Arnaldur Gylfason; Hilma Holm; Brynjar O Jensson; Aslaug Jonasdottir; Kamilla S Josefsdottir; Thordur Kristjansson; Droplaug N Magnusdottir; Louise le Roux; Gudrun Sigmundsdottir; Gardar Sveinbjornsson; Kristin E Sveinsdottir; Maney Sveinsdottir; Emil A Thorarensen; Bjarni Thorbjornsson; Gisli Masson; Ingileif Jonsdottir; Alma Moller; Thorolfur Gudnason; Karl G Kristinsson; Unnur Thorsteinsdottir; Kari Stefansson
EPI_ISL_424489 hCoV-19/Iceland/466/2020 Europe / Iceland / Reykjavik 2020-03-20 The National University Hospital of Iceland deCODE genetics Daniel F Gudbjartsson; Agnar Helgason; Hakon Jonsson; Olafur T Magnusson; Pall Melsted; Gudmundur L Norddahl; Jona Saemundsdottir; Asgeir Sigurdsson; Patrick Sulem; Arna B Agustsdottir; Berglind Eiriksdottir; Run Fridriksdottir; Elisabet E Gardarsdottir; Gudmundur Georgsson; Olafia S Gretarsdottir; Kjartan R Gudmundsson; Thora R Gunnarsdottir; Arnaldur Gylfason; Hilma Holm; Brynjar O Jensson; Aslaug Jonasdottir; Kamilla S Josefsdottir; Thordur Kristjansson; Droplaug N Magnusdottir; Louise le Roux; Gudrun Sigmundsdottir; Gardar Sveinbjornsson; Kristin E Sveinsdottir; Maney Sveinsdottir; Emil A Thorarensen; Bjarni Thorbjornsson; Gisli Masson; Ingileif Jonsdottir; Alma Moller; Thorolfur Gudnason; Karl G Kristinsson; Unnur Thorsteinsdottir; Kari Stefansson
EPI_ISL_424490 hCoV-19/Iceland/467/2020 Europe / Iceland / Reykjavik 2020-03-20 The National University Hospital of Iceland deCODE genetics Daniel F Gudbjartsson; Agnar Helgason; Hakon Jonsson; Olafur T Magnusson; Pall Melsted; Gudmundur L Norddahl; Jona Saemundsdottir; Asgeir Sigurdsson; Patrick Sulem; Arna B Agustsdottir; Berglind Eiriksdottir; Run Fridriksdottir; Elisabet E Gardarsdottir; Gudmundur Georgsson; Olafia S Gretarsdottir; Kjartan R Gudmundsson; Thora R Gunnarsdottir; Arnaldur Gylfason; Hilma Holm; Brynjar O Jensson; Aslaug Jonasdottir; Kamilla S Josefsdottir; Thordur Kristjansson; Droplaug N Magnusdottir; Louise le Roux; Gudrun Sigmundsdottir; Gardar Sveinbjornsson; Kristin E Sveinsdottir; Maney Sveinsdottir; Emil A Thorarensen; Bjarni Thorbjornsson; Gisli Masson; Ingileif Jonsdottir; Alma Moller; Thorolfur Gudnason; Karl G Kristinsson; Unnur Thorsteinsdottir; Kari Stefansson
EPI_ISL_424491 hCoV-19/Iceland/468/2020 Europe / Iceland / Reykjavik 2020-03-20 The National University Hospital of Iceland deCODE genetics Daniel F Gudbjartsson; Agnar Helgason; Hakon Jonsson; Olafur T Magnusson; Pall Melsted; Gudmundur L Norddahl; Jona Saemundsdottir; Asgeir Sigurdsson; Patrick Sulem; Arna B Agustsdottir; Berglind Eiriksdottir; Run Fridriksdottir; Elisabet E Gardarsdottir; Gudmundur Georgsson; Olafia S Gretarsdottir; Kjartan R Gudmundsson; Thora R Gunnarsdottir; Arnaldur Gylfason; Hilma Holm; Brynjar O

Jensson; Aslaug Jonasdottir; Kamilla S Josefsdottir; Thordur Kristjansson; Droplaug N Magnusdottir; Louise le Roux; Gudrun Sigmundsdottir; Gardar Sveinbjornsson; Kristin E Sveinsdottir; Maney Sveinsdottir; Emil A Thorarensen; Bjarni Thorbjornsson; Gisli Masson; Ingileif Jonsdottir; Alma Moller; Thorolfur Gudnason; Karl G Kristinsson; Unnur Thorsteinsdottir; Kari Stefansson
EPI_ISL_424492 hCoV-19/Iceland/469/2020 Europe / Iceland / Reykjavik 2020-03-21 The National University Hospital of Iceland deCODE genetics Daniel F Gudbjartsson; Agnar Helgason; Hakon Jonsson; Olafur T Magnusson; Pall Melsted; Gudmundur L Norddahl; Jona Saemundsdottir; Asgeir Sigurdsson; Patrick Sulem; Arna B Agustsdottir; Berglind Eiriksdottir; Run Fridriksdottir; Elisabet E Gardarsdottir; Gudmundur Georgsson; Olafia S Gretarsdottir; Kjartan R Gudmundsson; Thora R Gunnarsdottir; Arnaldur Gylfason; Hilma Holm; Brynjar O Jensson; Aslaug Jonasdottir; Kamilla S Josefsdottir; Thordur Kristjansson; Droplaug N Magnusdottir; Louise le Roux; Gudrun Sigmundsdottir; Gardar Sveinbjornsson; Kristin E Sveinsdottir; Maney Sveinsdottir; Emil A Thorarensen; Bjarni Thorbjornsson; Gisli Masson; Ingileif Jonsdottir; Alma Moller; Thorolfur Gudnason; Karl G Kristinsson; Unnur Thorsteinsdottir; Kari Stefansson
EPI_ISL_424493 hCoV-19/Iceland/470/2020 Europe / Iceland / Reykjavik 2020-03-21 The National University Hospital of Iceland deCODE genetics Daniel F Gudbjartsson; Agnar Helgason; Hakon Jonsson; Olafur T Magnusson; Pall Melsted; Gudmundur L Norddahl; Jona Saemundsdottir; Asgeir Sigurdsson; Patrick Sulem; Arna B Agustsdottir; Berglind Eiriksdottir; Run Fridriksdottir; Elisabet E Gardarsdottir; Gudmundur Georgsson; Olafia S Gretarsdottir; Kjartan R Gudmundsson; Thora R Gunnarsdottir; Arnaldur Gylfason; Hilma Holm; Brynjar O Jensson; Aslaug Jonasdottir; Kamilla S Josefsdottir; Thordur Kristjansson; Droplaug N Magnusdottir; Louise le Roux; Gudrun Sigmundsdottir; Gardar Sveinbjornsson; Kristin E Sveinsdottir; Maney Sveinsdottir; Emil A Thorarensen; Bjarni Thorbjornsson; Gisli Masson; Ingileif Jonsdottir; Alma Moller; Thorolfur Gudnason; Karl G Kristinsson; Unnur Thorsteinsdottir; Kari Stefansson
EPI_ISL_424494 hCoV-19/Iceland/471/2020 Europe / Iceland / Reykjavik 2020-03-20 The National University Hospital of Iceland deCODE genetics Daniel F Gudbjartsson; Agnar Helgason; Hakon Jonsson; Olafur T Magnusson; Pall Melsted; Gudmundur L Norddahl; Jona Saemundsdottir; Asgeir Sigurdsson; Patrick Sulem; Arna B Agustsdottir; Berglind Eiriksdottir; Run Fridriksdottir; Elisabet E Gardarsdottir; Gudmundur Georgsson; Olafia S Gretarsdottir; Kjartan R Gudmundsson; Thora R Gunnarsdottir; Arnaldur Gylfason; Hilma Holm; Brynjar O Jensson; Aslaug Jonasdottir; Kamilla S Josefsdottir; Thordur Kristjansson; Droplaug N Magnusdottir; Louise le Roux; Gudrun Sigmundsdottir; Gardar Sveinbjornsson; Kristin E Sveinsdottir; Maney Sveinsdottir; Emil A Thorarensen; Bjarni Thorbjornsson; Gisli Masson; Ingileif Jonsdottir; Alma Moller; Thorolfur Gudnason; Karl G Kristinsson; Unnur Thorsteinsdottir; Kari Stefansson
EPI_ISL_424495 hCoV-19/Iceland/472/2020 Europe / Iceland / Reykjavik 2020-03-20 The National University Hospital of Iceland deCODE genetics Daniel F Gudbjartsson; Agnar Helgason; Hakon Jonsson; Olafur T Magnusson; Pall Melsted; Gudmundur L Norddahl; Jona Saemundsdottir; Asgeir Sigurdsson; Patrick Sulem; Arna B Agustsdottir; Berglind Eiriksdottir; Run Fridriksdottir; Elisabet E Gardarsdottir; Gudmundur Georgsson; Olafia S Gretarsdottir; Kjartan R Gudmundsson; Thora R Gunnarsdottir; Arnaldur Gylfason; Hilma Holm; Brynjar O Jensson; Aslaug Jonasdottir; Kamilla S Josefsdottir; Thordur Kristjansson; Droplaug N Magnusdottir; Louise le Roux; Gudrun Sigmundsdottir; Gardar Sveinbjornsson; Kristin E Sveinsdottir; Maney Sveinsdottir; Emil A Thorarensen; Bjarni Thorbjornsson; Gisli Masson; Ingileif Jonsdottir; Alma Moller; Thorolfur Gudnason; Karl G Kristinsson; Unnur Thorsteinsdottir; Kari Stefansson
EPI_ISL_424496 hCoV-19/Iceland/473/2020 Europe / Iceland / Reykjavik 2020-03-20 The National University Hospital of Iceland deCODE genetics Daniel F Gudbjartsson; Agnar Helgason; Hakon Jonsson; Olafur T Magnusson; Pall Melsted; Gudmundur L Norddahl; Jona Saemundsdottir; Asgeir Sigurdsson; Patrick Sulem; Arna B Agustsdottir; Berglind Eiriksdottir; Run Fridriksdottir; Elisabet E Gardarsdottir; Gudmundur Georgsson; Olafia S Gretarsdottir; Kjartan R Gudmundsson; Thora R Gunnarsdottir; Arnaldur Gylfason; Hilma Holm; Brynjar O Jensson; Aslaug Jonasdottir; Kamilla S Josefsdottir; Thordur Kristjansson; Droplaug N Magnusdottir; Louise le Roux; Gudrun Sigmundsdottir; Gardar Sveinbjornsson; Kristin E Sveinsdottir; Maney Sveinsdottir; Emil A Thorarensen; Bjarni Thorbjornsson; Gisli Masson; Ingileif Jonsdottir; Alma Moller; Thorolfur Gudnason; Karl G Kristinsson; Unnur Thorsteinsdottir; Kari Stefansson
EPI_ISL_424498 hCoV-19/Iceland/475/2020 Europe / Iceland / Reykjavik 2020-03-20 The National University Hospital of Iceland deCODE genetics Daniel F Gudbjartsson; Agnar Helgason; Hakon Jonsson; Olafur T Magnusson; Pall Melsted; Gudmundur L Norddahl; Jona Saemundsdottir; Asgeir Sigurdsson; Patrick Sulem; Arna B Agustsdottir; Berglind Eiriksdottir; Run Fridriksdottir; Elisabet E Gardarsdottir; Gudmundur Georgsson; Olafia S Gretarsdottir; Kjartan R Gudmundsson; Thora R Gunnarsdottir; Arnaldur Gylfason; Hilma Holm; Brynjar O Jensson; Aslaug Jonasdottir; Kamilla S Josefsdottir; Thordur Kristjansson; Droplaug N Magnusdottir; Louise le Roux; Gudrun Sigmundsdottir; Gardar Sveinbjornsson; Kristin E Sveinsdottir; Maney Sveinsdottir; Emil A Thorarensen; Bjarni Thorbjornsson; Gisli Masson; Ingileif Jonsdottir; Alma Moller; Thorolfur Gudnason; Karl G Kristinsson; Unnur Thorsteinsdottir; Kari Stefansson
EPI_ISL_424499 hCoV-19/Iceland/476/2020 Europe / Iceland / Reykjavik 2020-03-20 The National University Hospital of Iceland deCODE genetics Daniel F Gudbjartsson; Agnar Helgason; Hakon Jonsson; Olafur T Magnusson; Pall Melsted; Gudmundur L Norddahl; Jona Saemundsdottir; Asgeir Sigurdsson; Patrick Sulem; Arna B Agustsdottir; Berglind Eiriksdottir; Run Fridriksdottir; Elisabet E Gardarsdottir; Gudmundur Georgsson; Olafia S Gretarsdottir; Kjartan R Gudmundsson; Thora R Gunnarsdottir; Arnaldur Gylfason; Hilma Holm; Brynjar O Jensson; Aslaug Jonasdottir; Kamilla S Josefsdottir; Thordur Kristjansson; Droplaug N Magnusdottir; Louise le Roux; Gudrun Sigmundsdottir; Gardar Sveinbjornsson; Kristin E Sveinsdottir; Maney Sveinsdottir; Emil A Thorarensen; Bjarni Thorbjornsson; Gisli Masson; Ingileif Jonsdottir; Alma Moller; Thorolfur Gudnason; Karl G Kristinsson; Unnur Thorsteinsdottir; Kari Stefansson
EPI_ISL_424580 hCoV-19/Iceland/560/2020 Europe / Iceland / Reykjavik 2020-03-27 The National University Hospital of Iceland deCODE genetics Daniel F Gudbjartsson; Agnar Helgason; Hakon Jonsson; Olafur T Magnusson; Pall Melsted; Gudmundur L Norddahl; Jona Saemundsdottir; Asgeir Sigurdsson; Patrick Sulem; Arna B

Agustsdottir; Berglind Eiriksdottir; Run Fridriksdottir; Elisabet E Gardarsdottir; Gudmundur Georgsson; Olafia S Gretarsdottir; Kjartan R Gudmundsson; Thora R Gunnarsdottir; Arnaldur Gylfason; Hilma Holm; Brynjar O Jensson; Aslaug Jonasdottir; Kamilla S Josefsdottir; Thordur Kristjansson; Droplaug N Magnusdottir; Louise le Roux; Gudrun Sigmundsdottir; Gardar Sveinbjornsson; Kristin E Sveinsdottir; Maney Sveinsdottir; Emil A Thorarensen; Bjarni Thorbjornsson; Gisli Masson; Ingileif Jonsdottir; Alma Moller; Thorolfur Gudnason; Karl G Kristinsson; Unnur Thorsteinsdottir; Kari Stefansson
EPI_ISL_424581 hCoV-19/Iceland/561/2020 Europe / Iceland / Reykjavik 2020-03-28 The National University Hospital of Iceland deCODE genetics Daniel F Gudbjartsson; Agnar Helgason; Hakon Jonsson; Olafur T Magnusson; Pall Melsted; Gudmundur L Norddahl; Jona Saemundsdottir; Asgeir Sigurdsson; Patrick Sulem; Arna B Agustsdottir; Berglind Eiriksdottir; Run Fridriksdottir; Elisabet E Gardarsdottir; Gudmundur Georgsson; Olafia S Gretarsdottir; Kjartan R Gudmundsson; Thora R Gunnarsdottir; Arnaldur Gylfason; Hilma Holm; Brynjar O Jensson; Aslaug Jonasdottir; Kamilla S Josefsdottir; Thordur Kristjansson; Droplaug N Magnusdottir; Louise le Roux; Gudrun Sigmundsdottir; Gardar Sveinbjornsson; Kristin E Sveinsdottir; Maney Sveinsdottir; Emil A Thorarensen; Bjarni Thorbjornsson; Gisli Masson; Ingileif Jonsdottir; Alma Moller; Thorolfur Gudnason; Karl G Kristinsson; Unnur Thorsteinsdottir; Kari Stefansson
EPI_ISL_424582 hCoV-19/Iceland/562/2020 Europe / Iceland / Reykjavik 2020-03-27 The National University Hospital of Iceland deCODE genetics Daniel F Gudbjartsson; Agnar Helgason; Hakon Jonsson; Olafur T Magnusson; Pall Melsted; Gudmundur L Norddahl; Jona Saemundsdottir; Asgeir Sigurdsson; Patrick Sulem; Arna B Agustsdottir; Berglind Eiriksdottir; Run Fridriksdottir; Elisabet E Gardarsdottir; Gudmundur Georgsson; Olafia S Gretarsdottir; Kjartan R Gudmundsson; Thora R Gunnarsdottir; Arnaldur Gylfason; Hilma Holm; Brynjar O Jensson; Aslaug Jonasdottir; Kamilla S Josefsdottir; Thordur Kristjansson; Droplaug N Magnusdottir; Louise le Roux; Gudrun Sigmundsdottir; Gardar Sveinbjornsson; Kristin E Sveinsdottir; Maney Sveinsdottir; Emil A Thorarensen; Bjarni Thorbjornsson; Gisli Masson; Ingileif Jonsdottir; Alma Moller; Thorolfur Gudnason; Karl G Kristinsson; Unnur Thorsteinsdottir; Kari Stefansson
EPI_ISL_424584 hCoV-19/Iceland/564/2020 Europe / Iceland / Reykjavik 2020-03-28 The National University Hospital of Iceland deCODE genetics Daniel F Gudbjartsson; Agnar Helgason; Hakon Jonsson; Olafur T Magnusson; Pall Melsted; Gudmundur L Norddahl; Jona Saemundsdottir; Asgeir Sigurdsson; Patrick Sulem; Arna B Agustsdottir; Berglind Eiriksdottir; Run Fridriksdottir; Elisabet E Gardarsdottir; Gudmundur Georgsson; Olafia S Gretarsdottir; Kjartan R Gudmundsson; Thora R Gunnarsdottir; Arnaldur Gylfason; Hilma Holm; Brynjar O Jensson; Aslaug Jonasdottir; Kamilla S Josefsdottir; Thordur Kristjansson; Droplaug N Magnusdottir; Louise le Roux; Gudrun Sigmundsdottir; Gardar Sveinbjornsson; Kristin E Sveinsdottir; Maney Sveinsdottir; Emil A Thorarensen; Bjarni Thorbjornsson; Gisli Masson; Ingileif Jonsdottir; Alma Moller; Thorolfur Gudnason; Karl G Kristinsson; Unnur Thorsteinsdottir; Kari Stefansson
EPI_ISL_424585 hCoV-19/Iceland/565/2020 Europe / Iceland / Reykjavik 2020-03-28 The National University Hospital of Iceland deCODE genetics Daniel F Gudbjartsson; Agnar Helgason; Hakon Jonsson; Olafur T Magnusson; Pall Melsted; Gudmundur L Norddahl; Jona Saemundsdottir; Asgeir Sigurdsson; Patrick Sulem; Arna B Agustsdottir; Berglind Eiriksdottir; Run Fridriksdottir; Elisabet E Gardarsdottir; Gudmundur Georgsson; Olafia S Gretarsdottir; Kjartan R Gudmundsson; Thora R Gunnarsdottir; Arnaldur Gylfason; Hilma Holm; Brynjar O Jensson; Aslaug Jonasdottir; Kamilla S Josefsdottir; Thordur Kristjansson; Droplaug N Magnusdottir; Louise le Roux; Gudrun Sigmundsdottir; Gardar Sveinbjornsson; Kristin E Sveinsdottir; Maney Sveinsdottir; Emil A Thorarensen; Bjarni Thorbjornsson; Gisli Masson; Ingileif Jonsdottir; Alma Moller; Thorolfur Gudnason; Karl G Kristinsson; Unnur Thorsteinsdottir; Kari Stefansson
EPI_ISL_424588 hCoV-19/Iceland/568/2020 Europe / Iceland / Reykjavik 2020-03-28 The National University Hospital of Iceland deCODE genetics Daniel F Gudbjartsson; Agnar Helgason; Hakon Jonsson; Olafur T Magnusson; Pall Melsted; Gudmundur L Norddahl; Jona Saemundsdottir; Asgeir Sigurdsson; Patrick Sulem; Arna B Agustsdottir; Berglind Eiriksdottir; Run Fridriksdottir; Elisabet E Gardarsdottir; Gudmundur Georgsson; Olafia S Gretarsdottir; Kjartan R Gudmundsson; Thora R Gunnarsdottir; Arnaldur Gylfason; Hilma Holm; Brynjar O Jensson; Aslaug Jonasdottir; Kamilla S Josefsdottir; Thordur Kristjansson; Droplaug N Magnusdottir; Louise le Roux; Gudrun Sigmundsdottir; Gardar Sveinbjornsson; Kristin E Sveinsdottir; Maney Sveinsdottir; Emil A Thorarensen; Bjarni Thorbjornsson; Gisli Masson; Ingileif Jonsdottir; Alma Moller; Thorolfur Gudnason; Karl G Kristinsson; Unnur Thorsteinsdottir; Kari Stefansson
EPI_ISL_424589 hCoV-19/Iceland/569/2020 Europe / Iceland / Reykjavik 2020-03-28 The National University Hospital of Iceland deCODE genetics Daniel F Gudbjartsson; Agnar Helgason; Hakon Jonsson; Olafur T Magnusson; Pall Melsted; Gudmundur L Norddahl; Jona Saemundsdottir; Asgeir Sigurdsson; Patrick Sulem; Arna B Agustsdottir; Berglind Eiriksdottir; Run Fridriksdottir; Elisabet E Gardarsdottir; Gudmundur Georgsson; Olafia S Gretarsdottir; Kjartan R Gudmundsson; Thora R Gunnarsdottir; Arnaldur Gylfason; Hilma Holm; Brynjar O Jensson; Aslaug Jonasdottir; Kamilla S Josefsdottir; Thordur Kristjansson; Droplaug N Magnusdottir; Louise le Roux; Gudrun Sigmundsdottir; Gardar Sveinbjornsson; Kristin E Sveinsdottir; Maney Sveinsdottir; Emil A Thorarensen; Bjarni Thorbjornsson; Gisli Masson; Ingileif Jonsdottir; Alma Moller; Thorolfur Gudnason; Karl G Kristinsson; Unnur Thorsteinsdottir; Kari Stefansson
EPI_ISL_424590 hCoV-19/Iceland/570/2020 Europe / Iceland / Reykjavik 2020-03-28 The National University Hospital of Iceland deCODE genetics Daniel F Gudbjartsson; Agnar Helgason; Hakon Jonsson; Olafur T Magnusson; Pall Melsted; Gudmundur L Norddahl; Jona Saemundsdottir; Asgeir Sigurdsson; Patrick Sulem; Arna B Agustsdottir; Berglind Eiriksdottir; Run Fridriksdottir; Elisabet E Gardarsdottir; Gudmundur Georgsson; Olafia S Gretarsdottir; Kjartan R Gudmundsson; Thora R Gunnarsdottir; Arnaldur Gylfason; Hilma Holm; Brynjar O Jensson; Aslaug Jonasdottir; Kamilla S Josefsdottir; Thordur Kristjansson; Droplaug N Magnusdottir; Louise le Roux; Gudrun Sigmundsdottir; Gardar Sveinbjornsson; Kristin E Sveinsdottir; Maney Sveinsdottir; Emil A Thorarensen; Bjarni Thorbjornsson; Gisli Masson; Ingileif Jonsdottir; Alma Moller; Thorolfur Gudnason; Karl G Kristinsson; Unnur Thorsteinsdottir; Kari Stefansson
EPI_ISL_424591 hCoV-19/Iceland/571/2020 Europe / Iceland / Reykjavik 2020-03-28 The National

University Hospital of Iceland deCODE genetics Daniel F Gudbjartsson; Agnar Helgason; Hakon Jonsson; Olafur T Magnusson; Pall Melsted; Gudmundur L Norddahl; Jona Saemundsdottir; Asgeir Sigurdsson; Patrick Sulem; Arna B Agustsdottir; Berglind Eiriksdottir; Run Fridriksdottir; Elisabet E Gardarsdottir; Gudmundur Georgsson; Olafia S Gretarsdottir; Kjartan R Gudmundsson; Thora R Gunnarsdottir; Arnaldur Gylfason; Hilma Holm; Brynjar O Jensson; Aslaug Jonasdottir; Kamilla S Josefsdottir; Thordur Kristjansson; Droplaug N Magnusdottir; Louise le Roux; Gudrun Sigmundsdottir; Gardar Sveinbjornsson; Kristin E Sveinsdottir; Maney Sveinsdottir; Emil A Thorarensen; Bjarni Thorbjornsson; Gisli Masson; Ingileif Jonsdottir; Alma Moller; Thorolfur Gudnason; Karl G Kristinsson; Unnur Thorsteinsdottir; Kari Stefansson
EPI_ISL_424592 hCoV-19/Iceland/572/2020 Europe / Iceland / Reykjavik 2020-03-28 The National University Hospital of Iceland deCODE genetics Daniel F Gudbjartsson; Agnar Helgason; Hakon Jonsson; Olafur T Magnusson; Pall Melsted; Gudmundur L Norddahl; Jona Saemundsdottir; Asgeir Sigurdsson; Patrick Sulem; Arna B Agustsdottir; Berglind Eiriksdottir; Run Fridriksdottir; Elisabet E Gardarsdottir; Gudmundur Georgsson; Olafia S Gretarsdottir; Kjartan R Gudmundsson; Thora R Gunnarsdottir; Arnaldur Gylfason; Hilma Holm; Brynjar O Jensson; Aslaug Jonasdottir; Kamilla S Josefsdottir; Thordur Kristjansson; Droplaug N Magnusdottir; Louise le Roux; Gudrun Sigmundsdottir; Gardar Sveinbjornsson; Kristin E Sveinsdottir; Maney Sveinsdottir; Emil A Thorarensen; Bjarni Thorbjornsson; Gisli Masson; Ingileif Jonsdottir; Alma Moller; Thorolfur Gudnason; Karl G Kristinsson; Unnur Thorsteinsdottir; Kari Stefansson
EPI_ISL_424593 hCoV-19/Iceland/573/2020 Europe / Iceland / Reykjavik 2020-03-28 The National University Hospital of Iceland deCODE genetics Daniel F Gudbjartsson; Agnar Helgason; Hakon Jonsson; Olafur T Magnusson; Pall Melsted; Gudmundur L Norddahl; Jona Saemundsdottir; Asgeir Sigurdsson; Patrick Sulem; Arna B Agustsdottir; Berglind Eiriksdottir; Run Fridriksdottir; Elisabet E Gardarsdottir; Gudmundur Georgsson; Olafia S Gretarsdottir; Kjartan R Gudmundsson; Thora R Gunnarsdottir; Arnaldur Gylfason; Hilma Holm; Brynjar O Jensson; Aslaug Jonasdottir; Kamilla S Josefsdottir; Thordur Kristjansson; Droplaug N Magnusdottir; Louise le Roux; Gudrun Sigmundsdottir; Gardar Sveinbjornsson; Kristin E Sveinsdottir; Maney Sveinsdottir; Emil A Thorarensen; Bjarni Thorbjornsson; Gisli Masson; Ingileif Jonsdottir; Alma Moller; Thorolfur Gudnason; Karl G Kristinsson; Unnur Thorsteinsdottir; Kari Stefansson
EPI_ISL_424595 hCoV-19/Iceland/575/2020 Europe / Iceland / Reykjavik 2020-03-28 The National University Hospital of Iceland deCODE genetics Daniel F Gudbjartsson; Agnar Helgason; Hakon Jonsson; Olafur T Magnusson; Pall Melsted; Gudmundur L Norddahl; Jona Saemundsdottir; Asgeir Sigurdsson; Patrick Sulem; Arna B Agustsdottir; Berglind Eiriksdottir; Run Fridriksdottir; Elisabet E Gardarsdottir; Gudmundur Georgsson; Olafia S Gretarsdottir; Kjartan R Gudmundsson; Thora R Gunnarsdottir; Arnaldur Gylfason; Hilma Holm; Brynjar O Jensson; Aslaug Jonasdottir; Kamilla S Josefsdottir; Thordur Kristjansson; Droplaug N Magnusdottir; Louise le Roux; Gudrun Sigmundsdottir; Gardar Sveinbjornsson; Kristin E Sveinsdottir; Maney Sveinsdottir; Emil A Thorarensen; Bjarni Thorbjornsson; Gisli Masson; Ingileif Jonsdottir; Alma Moller; Thorolfur Gudnason; Karl G Kristinsson; Unnur Thorsteinsdottir; Kari Stefansson
EPI_ISL_424596 hCoV-19/Iceland/576/2020 Europe / Iceland / Reykjavik 2020-03-28 The National University Hospital of Iceland deCODE genetics Daniel F Gudbjartsson; Agnar Helgason; Hakon Jonsson; Olafur T Magnusson; Pall Melsted; Gudmundur L Norddahl; Jona Saemundsdottir; Asgeir Sigurdsson; Patrick Sulem; Arna B Agustsdottir; Berglind Eiriksdottir; Run Fridriksdottir; Elisabet E Gardarsdottir; Gudmundur Georgsson; Olafia S Gretarsdottir; Kjartan R Gudmundsson; Thora R Gunnarsdottir; Arnaldur Gylfason; Hilma Holm; Brynjar O Jensson; Aslaug Jonasdottir; Kamilla S Josefsdottir; Thordur Kristjansson; Droplaug N Magnusdottir; Louise le Roux; Gudrun Sigmundsdottir; Gardar Sveinbjornsson; Kristin E Sveinsdottir; Maney Sveinsdottir; Emil A Thorarensen; Bjarni Thorbjornsson; Gisli Masson; Ingileif Jonsdottir; Alma Moller; Thorolfur Gudnason; Karl G Kristinsson; Unnur Thorsteinsdottir; Kari Stefansson
EPI_ISL_424597 hCoV-19/Iceland/577/2020 Europe / Iceland / Reykjavik 2020-03-27 The National University Hospital of Iceland deCODE genetics Daniel F Gudbjartsson; Agnar Helgason; Hakon Jonsson; Olafur T Magnusson; Pall Melsted; Gudmundur L Norddahl; Jona Saemundsdottir; Asgeir Sigurdsson; Patrick Sulem; Arna B Agustsdottir; Berglind Eiriksdottir; Run Fridriksdottir; Elisabet E Gardarsdottir; Gudmundur Georgsson; Olafia S Gretarsdottir; Kjartan R Gudmundsson; Thora R Gunnarsdottir; Arnaldur Gylfason; Hilma Holm; Brynjar O Jensson; Aslaug Jonasdottir; Kamilla S Josefsdottir; Thordur Kristjansson; Droplaug N Magnusdottir; Louise le Roux; Gudrun Sigmundsdottir; Gardar Sveinbjornsson; Kristin E Sveinsdottir; Maney Sveinsdottir; Emil A Thorarensen; Bjarni Thorbjornsson; Gisli Masson; Ingileif Jonsdottir; Alma Moller; Thorolfur Gudnason; Karl G Kristinsson; Unnur Thorsteinsdottir; Kari Stefansson
EPI_ISL_424598 hCoV-19/Iceland/578/2020 Europe / Iceland / Reykjavik 2020-03-27 The National University Hospital of Iceland deCODE genetics Daniel F Gudbjartsson; Agnar Helgason; Hakon Jonsson; Olafur T Magnusson; Pall Melsted; Gudmundur L Norddahl; Jona Saemundsdottir; Asgeir Sigurdsson; Patrick Sulem; Arna B Agustsdottir; Berglind Eiriksdottir; Run Fridriksdottir; Elisabet E Gardarsdottir; Gudmundur Georgsson; Olafia S Gretarsdottir; Kjartan R Gudmundsson; Thora R Gunnarsdottir; Arnaldur Gylfason; Hilma Holm; Brynjar O Jensson; Aslaug Jonasdottir; Kamilla S Josefsdottir; Thordur Kristjansson; Droplaug N Magnusdottir; Louise le Roux; Gudrun Sigmundsdottir; Gardar Sveinbjornsson; Kristin E Sveinsdottir; Maney Sveinsdottir; Emil A Thorarensen; Bjarni Thorbjornsson; Gisli Masson; Ingileif Jonsdottir; Alma Moller; Thorolfur Gudnason; Karl G Kristinsson; Unnur Thorsteinsdottir; Kari Stefansson
EPI_ISL_424599 hCoV-19/Iceland/579/2020 Europe / Iceland / Reykjavik 2020-03-27 The National University Hospital of Iceland deCODE genetics Daniel F Gudbjartsson; Agnar Helgason; Hakon Jonsson; Olafur T Magnusson; Pall Melsted; Gudmundur L Norddahl; Jona Saemundsdottir; Asgeir Sigurdsson; Patrick Sulem; Arna B Agustsdottir; Berglind Eiriksdottir; Run Fridriksdottir; Elisabet E Gardarsdottir; Gudmundur Georgsson; Olafia S Gretarsdottir; Kjartan R Gudmundsson; Thora R Gunnarsdottir; Arnaldur Gylfason; Hilma Holm; Brynjar O Jensson; Aslaug Jonasdottir; Kamilla S Josefsdottir; Thordur Kristjansson; Droplaug N Magnusdottir; Louise le Roux; Gudrun Sigmundsdottir; Gardar Sveinbjornsson; Kristin E Sveinsdottir; Maney Sveinsdottir; Emil A Thorarensen; Bjarni Thorbjornsson; Gisli Masson; Ingileif Jonsdottir; Alma Moller; Thorolfur Gudnason; Karl G Kristinsson;

Kristinsson; Unnur Thorsteinsdottir; Kari Stefansson
EPI_ISL_424601 hCoV-19/Iceland/581/2020 Europe / Iceland / Reykjavik 2020-03-27 The National University Hospital of Iceland deCODE genetics Daniel F Gudbjartsson; Agnar Helgason; Hakon Jonsson; Olafur T Magnusson; Pall Melsted; Gudmundur L Norddahl; Jona Saemundsdottir; Asgeir Sigurdsson; Patrick Sulem; Arna B Agustsdottir; Berglind Eiriksdottir; Run Fridriksdottir; Elisabet E Gardarsdottir; Gudmundur Georgsson; Olafia S Gretarsdottir; Kjartan R Gudmundsson; Thora R Gunnarsdottir; Arnaldur Gylfason; Hilma Holm; Brynjar O Jensson; Aslaug Jonasdottir; Kamilla S Josefsdottir; Thordur Kristjansson; Droplaug N Magnusdottir; Louise le Roux; Gudrun Sigmundsdottir; Gardar Sveinbjornsson; Kristin E Sveinsdottir; Maney Sveinsdottir; Emil A Thorarensen; Bjarni Thorbjornsson; Gisli Masson; Ingileif Jonsdottir; Alma Moller; Thorolfur Gudnason; Karl G Kristinsson; Unnur Thorsteinsdottir; Kari Stefansson
EPI_ISL_424602 hCoV-19/Iceland/582/2020 Europe / Iceland / Reykjavik 2020-03-27 The National University Hospital of Iceland deCODE genetics Daniel F Gudbjartsson; Agnar Helgason; Hakon Jonsson; Olafur T Magnusson; Pall Melsted; Gudmundur L Norddahl; Jona Saemundsdottir; Asgeir Sigurdsson; Patrick Sulem; Arna B Agustsdottir; Berglind Eiriksdottir; Run Fridriksdottir; Elisabet E Gardarsdottir; Gudmundur Georgsson; Olafia S Gretarsdottir; Kjartan R Gudmundsson; Thora R Gunnarsdottir; Arnaldur Gylfason; Hilma Holm; Brynjar O Jensson; Aslaug Jonasdottir; Kamilla S Josefsdottir; Thordur Kristjansson; Droplaug N Magnusdottir; Louise le Roux; Gudrun Sigmundsdottir; Gardar Sveinbjornsson; Kristin E Sveinsdottir; Maney Sveinsdottir; Emil A Thorarensen; Bjarni Thorbjornsson; Gisli Masson; Ingileif Jonsdottir; Alma Moller; Thorolfur Gudnason; Karl G Kristinsson; Unnur Thorsteinsdottir; Kari Stefansson
EPI_ISL_424603 hCoV-19/Iceland/583/2020 Europe / Iceland / Reykjavik 2020-03-27 The National University Hospital of Iceland deCODE genetics Daniel F Gudbjartsson; Agnar Helgason; Hakon Jonsson; Olafur T Magnusson; Pall Melsted; Gudmundur L Norddahl; Jona Saemundsdottir; Asgeir Sigurdsson; Patrick Sulem; Arna B Agustsdottir; Berglind Eiriksdottir; Run Fridriksdottir; Elisabet E Gardarsdottir; Gudmundur Georgsson; Olafia S Gretarsdottir; Kjartan R Gudmundsson; Thora R Gunnarsdottir; Arnaldur Gylfason; Hilma Holm; Brynjar O Jensson; Aslaug Jonasdottir; Kamilla S Josefsdottir; Thordur Kristjansson; Droplaug N Magnusdottir; Louise le Roux; Gudrun Sigmundsdottir; Gardar Sveinbjornsson; Kristin E Sveinsdottir; Maney Sveinsdottir; Emil A Thorarensen; Bjarni Thorbjornsson; Gisli Masson; Ingileif Jonsdottir; Alma Moller; Thorolfur Gudnason; Karl G Kristinsson; Unnur Thorsteinsdottir; Kari Stefansson
EPI_ISL_424604 hCoV-19/Iceland/584/2020 Europe / Iceland / Reykjavik 2020-03-27 The National University Hospital of Iceland deCODE genetics Daniel F Gudbjartsson; Agnar Helgason; Hakon Jonsson; Olafur T Magnusson; Pall Melsted; Gudmundur L Norddahl; Jona Saemundsdottir; Asgeir Sigurdsson; Patrick Sulem; Arna B Agustsdottir; Berglind Eiriksdottir; Run Fridriksdottir; Elisabet E Gardarsdottir; Gudmundur Georgsson; Olafia S Gretarsdottir; Kjartan R Gudmundsson; Thora R Gunnarsdottir; Arnaldur Gylfason; Hilma Holm; Brynjar O Jensson; Aslaug Jonasdottir; Kamilla S Josefsdottir; Thordur Kristjansson; Droplaug N Magnusdottir; Louise le Roux; Gudrun Sigmundsdottir; Gardar Sveinbjornsson; Kristin E Sveinsdottir; Maney Sveinsdottir; Emil A Thorarensen; Bjarni Thorbjornsson; Gisli Masson; Ingileif Jonsdottir; Alma Moller; Thorolfur Gudnason; Karl G Kristinsson; Unnur Thorsteinsdottir; Kari Stefansson
EPI_ISL_424605 hCoV-19/Iceland/585/2020 Europe / Iceland / Reykjavik 2020-03-27 The National University Hospital of Iceland deCODE genetics Daniel F Gudbjartsson; Agnar Helgason; Hakon Jonsson; Olafur T Magnusson; Pall Melsted; Gudmundur L Norddahl; Jona Saemundsdottir; Asgeir Sigurdsson; Patrick Sulem; Arna B Agustsdottir; Berglind Eiriksdottir; Run Fridriksdottir; Elisabet E Gardarsdottir; Gudmundur Georgsson; Olafia S Gretarsdottir; Kjartan R Gudmundsson; Thora R Gunnarsdottir; Arnaldur Gylfason; Hilma Holm; Brynjar O Jensson; Aslaug Jonasdottir; Kamilla S Josefsdottir; Thordur Kristjansson; Droplaug N Magnusdottir; Louise le Roux; Gudrun Sigmundsdottir; Gardar Sveinbjornsson; Kristin E Sveinsdottir; Maney Sveinsdottir; Emil A Thorarensen; Bjarni Thorbjornsson; Gisli Masson; Ingileif Jonsdottir; Alma Moller; Thorolfur Gudnason; Karl G Kristinsson; Unnur Thorsteinsdottir; Kari Stefansson
EPI_ISL_424606 hCoV-19/Iceland/586/2020 Europe / Iceland / Reykjavik 2020-03-28 The National University Hospital of Iceland deCODE genetics Daniel F Gudbjartsson; Agnar Helgason; Hakon Jonsson; Olafur T Magnusson; Pall Melsted; Gudmundur L Norddahl; Jona Saemundsdottir; Asgeir Sigurdsson; Patrick Sulem; Arna B Agustsdottir; Berglind Eiriksdottir; Run Fridriksdottir; Elisabet E Gardarsdottir; Gudmundur Georgsson; Olafia S Gretarsdottir; Kjartan R Gudmundsson; Thora R Gunnarsdottir; Arnaldur Gylfason; Hilma Holm; Brynjar O Jensson; Aslaug Jonasdottir; Kamilla S Josefsdottir; Thordur Kristjansson; Droplaug N Magnusdottir; Louise le Roux; Gudrun Sigmundsdottir; Gardar Sveinbjornsson; Kristin E Sveinsdottir; Maney Sveinsdottir; Emil A Thorarensen; Bjarni Thorbjornsson; Gisli Masson; Ingileif Jonsdottir; Alma Moller; Thorolfur Gudnason; Karl G Kristinsson; Unnur Thorsteinsdottir; Kari Stefansson
EPI_ISL_424609 hCoV-19/Iceland/589/2020 Europe / Iceland / Reykjavik 2020-03-29 deCODE genetics deCODE genetics Daniel F Gudbjartsson; Agnar Helgason; Hakon Jonsson; Olafur T Magnusson; Pall Melsted; Gudmundur L Norddahl; Jona Saemundsdottir; Asgeir Sigurdsson; Patrick Sulem; Arna B Agustsdottir; Berglind Eiriksdottir; Run Fridriksdottir; Elisabet E Gardarsdottir; Gudmundur Georgsson; Olafia S Gretarsdottir; Kjartan R Gudmundsson; Thora R Gunnarsdottir; Arnaldur Gylfason; Hilma Holm; Brynjar O Jensson; Aslaug Jonasdottir; Kamilla S Josefsdottir; Thordur Kristjansson; Droplaug N Magnusdottir; Louise le Roux; Gudrun Sigmundsdottir; Gardar Sveinbjornsson; Kristin E Sveinsdottir; Maney Sveinsdottir; Emil A Thorarensen; Bjarni Thorbjornsson; Gisli Masson; Ingileif Jonsdottir; Alma Moller; Thorolfur Gudnason; Karl G Kristinsson; Unnur Thorsteinsdottir; Kari Stefansson
EPI_ISL_424610 hCoV-19/Iceland/590/2020 Europe / Iceland / Reykjavik 2020-03-26 The National University Hospital of Iceland deCODE genetics Daniel F Gudbjartsson; Agnar Helgason; Hakon Jonsson; Olafur T Magnusson; Pall Melsted; Gudmundur L Norddahl; Jona Saemundsdottir; Asgeir Sigurdsson; Patrick Sulem; Arna B Agustsdottir; Berglind Eiriksdottir; Run Fridriksdottir; Elisabet E Gardarsdottir; Gudmundur Georgsson; Olafia S Gretarsdottir; Kjartan R Gudmundsson; Thora R Gunnarsdottir; Arnaldur Gylfason; Hilma Holm; Brynjar O Jensson; Aslaug Jonasdottir; Kamilla S Josefsdottir; Thordur Kristjansson; Droplaug N Magnusdottir; Louise le

Roux; Gudrun Sigmundsdottir; Gardar Sveinbjornsson; Kristin E Sveinsdottir; Maney Sveinsdottir; Emil A Thorarensen; Bjarni Thorbjornsson; Gisli Masson; Ingileif Jonsdottir; Alma Moller; Thorolfur Gudnason; Karl G Kristinsson; Unnur Thorsteinsdottir; Kari Stefansson
EPI_ISL_424612 hCoV-19/Iceland/592/2020 Europe / Iceland / Reykjavik 2020-03-27 The National University Hospital of Iceland deCODE genetics Daniel F Gudbjartsson; Agnar Helgason; Hakon Jonsson; Olafur T Magnusson; Pall Melsted; Gudmundur L Norddahl; Jona Saemundsdottir; Asgeir Sigurdsson; Patrick Sulem; Arna B Agustsdottir; Berglind Eiriksdottir; Run Fridriksdottir; Elisabet E Gardarsdottir; Gudmundur Georgsson; Olafia S Gretarsdottir; Kjartan R Gudmundsson; Thora R Gunnarsdottir; Arnaldur Gylfason; Hilma Holm; Brynjar O Jensson; Aslaug Jonasdottir; Kamilla S Josefsdottir; Thordur Kristjansson; Droplaug N Magnusdottir; Louise le Roux; Gudrun Sigmundsdottir; Gardar Sveinbjornsson; Kristin E Sveinsdottir; Maney Sveinsdottir; Emil A Thorarensen; Bjarni Thorbjornsson; Gisli Masson; Ingileif Jonsdottir; Alma Moller; Thorolfur Gudnason; Karl G Kristinsson; Unnur Thorsteinsdottir; Kari Stefansson
EPI_ISL_425702 hCoV-19/Scotland/CVR163/2020 Europe / United Kingdom / Scotland 2020-03-17 West of Scotland Specialist Virology Centre, NHSGGC / MRC-University of Glasgow Centre for Virus Research COVID-19 Genomics UK (COG-UK) Consortium Ana da Silva Filipe, Kathy Smollett, Stephen Carmichael, Natasha Johnson, Daniel Mair, Lily Tong, Jenna Nichols; Sarah McDonald; Richard Orton, Joseph Hughes, Sreenu Vattipally, David L Robertson; Kathy Li, Natasha Jesudason, Rajiv Shah, James Shepherd, Antonia Ho, Emma Thomson; Alasdair MacLean, Rory Gunson.
EPI_ISL_425703 hCoV-19/Scotland/CVR164/2020 Europe / United Kingdom / Scotland 2020-03-17 West of Scotland Specialist Virology Centre, NHSGGC / MRC-University of Glasgow Centre for Virus Research COVID-19 Genomics UK (COG-UK) Consortium Ana da Silva Filipe, Kathy Smollett, Stephen Carmichael, Natasha Johnson, Daniel Mair, Lily Tong, Jenna Nichols; Sarah McDonald; Richard Orton, Joseph Hughes, Sreenu Vattipally, David L Robertson; Kathy Li, Natasha Jesudason, Rajiv Shah, James Shepherd, Antonia Ho, Emma Thomson; Alasdair MacLean, Rory Gunson.
EPI_ISL_425704 hCoV-19/Scotland/CVR165/2020 Europe / United Kingdom / Scotland 2020-03-17 West of Scotland Specialist Virology Centre, NHSGGC / MRC-University of Glasgow Centre for Virus Research COVID-19 Genomics UK (COG-UK) Consortium Ana da Silva Filipe, Kathy Smollett, Stephen Carmichael, Natasha Johnson, Daniel Mair, Lily Tong, Jenna Nichols; Sarah McDonald; Richard Orton, Joseph Hughes, Sreenu Vattipally, David L Robertson; Kathy Li, Natasha Jesudason, Rajiv Shah, James Shepherd, Antonia Ho, Emma Thomson; Alasdair MacLean, Rory Gunson.
EPI_ISL_425705 hCoV-19/Scotland/CVR166/2020 Europe / United Kingdom / Scotland 2020-03-17 West of Scotland Specialist Virology Centre, NHSGGC / MRC-University of Glasgow Centre for Virus Research COVID-19 Genomics UK (COG-UK) Consortium Ana da Silva Filipe, Kathy Smollett, Stephen Carmichael, Natasha Johnson, Daniel Mair, Lily Tong, Jenna Nichols; Sarah McDonald; Richard Orton, Joseph Hughes, Sreenu Vattipally, David L Robertson; Kathy Li, Natasha Jesudason, Rajiv Shah, James Shepherd, Antonia Ho, Emma Thomson; Alasdair MacLean, Rory Gunson.
EPI_ISL_425707 hCoV-19/Scotland/CVR168/2020 Europe / United Kingdom / Scotland 2020-03-17 West of Scotland Specialist Virology Centre, NHSGGC / MRC-University of Glasgow Centre for Virus Research COVID-19 Genomics UK (COG-UK) Consortium Ana da Silva Filipe, Kathy Smollett, Stephen Carmichael, Natasha Johnson, Daniel Mair, Lily Tong, Jenna Nichols; Sarah McDonald; Richard Orton, Joseph Hughes, Sreenu Vattipally, David L Robertson; Kathy Li, Natasha Jesudason, Rajiv Shah, James Shepherd, Antonia Ho, Emma Thomson; Alasdair MacLean, Rory Gunson.
EPI_ISL_425708 hCoV-19/Scotland/CVR169/2020 Europe / United Kingdom / Scotland 2020-03-17 West of Scotland Specialist Virology Centre, NHSGGC / MRC-University of Glasgow Centre for Virus Research COVID-19 Genomics UK (COG-UK) Consortium Ana da Silva Filipe, Kathy Smollett, Stephen Carmichael, Natasha Johnson, Daniel Mair, Lily Tong, Jenna Nichols; Sarah McDonald; Richard Orton, Joseph Hughes, Sreenu Vattipally, David L Robertson; Kathy Li, Natasha Jesudason, Rajiv Shah, James Shepherd, Antonia Ho, Emma Thomson; Alasdair MacLean, Rory Gunson.
EPI_ISL_425709 hCoV-19/Scotland/CVR17/2020 Europe / United Kingdom / Scotland 2020-03-09 West of Scotland Specialist Virology Centre, NHSGGC / MRC-University of Glasgow Centre for Virus Research COVID-19 Genomics UK (COG-UK) Consortium Ana da Silva Filipe, Kathy Smollett, Stephen Carmichael, Natasha Johnson, Daniel Mair, Lily Tong, Jenna Nichols; Sarah McDonald; Richard Orton, Joseph Hughes, Sreenu Vattipally, David L Robertson; Kathy Li, Natasha Jesudason, Rajiv Shah, James Shepherd, Antonia Ho, Emma Thomson; Alasdair MacLean, Rory Gunson.
EPI_ISL_425711 hCoV-19/Scotland/CVR171/2020 Europe / United Kingdom / Scotland 2020-03-17 West of Scotland Specialist Virology Centre, NHSGGC / MRC-University of Glasgow Centre for Virus Research COVID-19 Genomics UK (COG-UK) Consortium Ana da Silva Filipe, Kathy Smollett, Stephen Carmichael, Natasha Johnson, Daniel Mair, Lily Tong, Jenna Nichols; Sarah McDonald; Richard Orton, Joseph Hughes, Sreenu Vattipally, David L Robertson; Kathy Li, Natasha Jesudason, Rajiv Shah, James Shepherd, Antonia Ho, Emma Thomson; Alasdair MacLean, Rory Gunson.
EPI_ISL_425713 hCoV-19/Scotland/CVR173/2020 Europe / United Kingdom / Scotland 2020-03-17 West of Scotland Specialist Virology Centre, NHSGGC / MRC-University of Glasgow Centre for Virus Research COVID-19 Genomics UK (COG-UK) Consortium Ana da Silva Filipe, Kathy Smollett, Stephen Carmichael, Natasha Johnson, Daniel Mair, Lily Tong, Jenna Nichols; Sarah McDonald; Richard Orton, Joseph Hughes, Sreenu Vattipally, David L Robertson; Kathy Li, Natasha Jesudason, Rajiv Shah, James Shepherd, Antonia Ho, Emma Thomson; Alasdair MacLean, Rory Gunson.
EPI_ISL_425715 hCoV-19/Scotland/CVR175/2020 Europe / United Kingdom / Scotland 2020-03-17 West of Scotland Specialist Virology Centre, NHSGGC / MRC-University of Glasgow Centre for Virus Research COVID-19 Genomics UK (COG-UK) Consortium Ana da Silva Filipe, Kathy Smollett, Stephen Carmichael, Natasha Johnson, Daniel Mair, Lily Tong, Jenna Nichols; Sarah McDonald; Richard Orton, Joseph Hughes, Sreenu

Vattipally, David L Robertson; Kathy Li, Natasha Jesudason, Rajiv Shah, James Shepherd, Antonia Ho, Emma Thomson; Alasdair MacLean, Rory Gunson.
EPI_ISL_425716 hCoV-19/Scotland/CVR176/2020 Europe / United Kingdom / Scotland 2020-03-17 West of Scotland Specialist Virology Centre, NHSGGC / MRC-University of Glasgow Centre for Virus Research COVID-19 Genomics UK (COG-UK) Consortium Ana da Silva Filipe, Kathy Smollett, Stephen Carmichael, Natasha Johnson, Daniel Mair, Lily Tong, Jenna Nichols; Sarah McDonald; Richard Orton, Joseph Hughes, Sreenu Vattipally, David L Robertson; Kathy Li, Natasha Jesudason, Rajiv Shah, James Shepherd, Antonia Ho, Emma Thomson; Alasdair MacLean, Rory Gunson.
EPI_ISL_425717 hCoV-19/Scotland/CVR177/2020 Europe / United Kingdom / Scotland 2020-03-17 West of Scotland Specialist Virology Centre, NHSGGC / MRC-University of Glasgow Centre for Virus Research COVID-19 Genomics UK (COG-UK) Consortium Ana da Silva Filipe, Kathy Smollett, Stephen Carmichael, Natasha Johnson, Daniel Mair, Lily Tong, Jenna Nichols; Sarah McDonald; Richard Orton, Joseph Hughes, Sreenu Vattipally, David L Robertson; Kathy Li, Natasha Jesudason, Rajiv Shah, James Shepherd, Antonia Ho, Emma Thomson; Alasdair MacLean, Rory Gunson.
EPI_ISL_425720 hCoV-19/Scotland/CVR18/2020 Europe / United Kingdom / Scotland 2020-03-12 West of Scotland Specialist Virology Centre, NHSGGC / MRC-University of Glasgow Centre for Virus Research COVID-19 Genomics UK (COG-UK) Consortium Ana da Silva Filipe, Kathy Smollett, Stephen Carmichael, Natasha Johnson, Daniel Mair, Lily Tong, Jenna Nichols; Sarah McDonald; Richard Orton, Joseph Hughes, Sreenu Vattipally, David L Robertson; Kathy Li, Natasha Jesudason, Rajiv Shah, James Shepherd, Antonia Ho, Emma Thomson; Alasdair MacLean, Rory Gunson.
EPI_ISL_425721 hCoV-19/Scotland/CVR180/2020 Europe / United Kingdom / Scotland 2020-03-17 West of Scotland Specialist Virology Centre, NHSGGC / MRC-University of Glasgow Centre for Virus Research COVID-19 Genomics UK (COG-UK) Consortium Ana da Silva Filipe, Kathy Smollett, Stephen Carmichael, Natasha Johnson, Daniel Mair, Lily Tong, Jenna Nichols; Sarah McDonald; Richard Orton, Joseph Hughes, Sreenu Vattipally, David L Robertson; Kathy Li, Natasha Jesudason, Rajiv Shah, James Shepherd, Antonia Ho, Emma Thomson; Alasdair MacLean, Rory Gunson.
EPI_ISL_425722 hCoV-19/Scotland/CVR183/2020 Europe / United Kingdom / Scotland 2020-03-18 West of Scotland Specialist Virology Centre, NHSGGC / MRC-University of Glasgow Centre for Virus Research COVID-19 Genomics UK (COG-UK) Consortium Ana da Silva Filipe, Kathy Smollett, Stephen Carmichael, Natasha Johnson, Daniel Mair, Lily Tong, Jenna Nichols; Sarah McDonald; Richard Orton, Joseph Hughes, Sreenu Vattipally, David L Robertson; Kathy Li, Natasha Jesudason, Rajiv Shah, James Shepherd, Antonia Ho, Emma Thomson; Alasdair MacLean, Rory Gunson.
EPI_ISL_425724 hCoV-19/Scotland/CVR19/2020 Europe / United Kingdom / Scotland 2020-03-12 West of Scotland Specialist Virology Centre, NHSGGC / MRC-University of Glasgow Centre for Virus Research COVID-19 Genomics UK (COG-UK) Consortium Ana da Silva Filipe, Kathy Smollett, Stephen Carmichael, Natasha Johnson, Daniel Mair, Lily Tong, Jenna Nichols; Sarah McDonald; Richard Orton, Joseph Hughes, Sreenu Vattipally, David L Robertson; Kathy Li, Natasha Jesudason, Rajiv Shah, James Shepherd, Antonia Ho, Emma Thomson; Alasdair MacLean, Rory Gunson.
EPI_ISL_425725 hCoV-19/Scotland/CVR190/2020 Europe / United Kingdom / Scotland 2020-03-18 West of Scotland Specialist Virology Centre, NHSGGC / MRC-University of Glasgow Centre for Virus Research COVID-19 Genomics UK (COG-UK) Consortium Ana da Silva Filipe, Kathy Smollett, Stephen Carmichael, Natasha Johnson, Daniel Mair, Lily Tong, Jenna Nichols; Sarah McDonald; Richard Orton, Joseph Hughes, Sreenu Vattipally, David L Robertson; Kathy Li, Natasha Jesudason, Rajiv Shah, James Shepherd, Antonia Ho, Emma Thomson; Alasdair MacLean, Rory Gunson.
EPI_ISL_425726 hCoV-19/Scotland/CVR191/2020 Europe / United Kingdom / Scotland 2020-03-18 West of Scotland Specialist Virology Centre, NHSGGC / MRC-University of Glasgow Centre for Virus Research COVID-19 Genomics UK (COG-UK) Consortium Ana da Silva Filipe, Kathy Smollett, Stephen Carmichael, Natasha Johnson, Daniel Mair, Lily Tong, Jenna Nichols; Sarah McDonald; Richard Orton, Joseph Hughes, Sreenu Vattipally, David L Robertson; Kathy Li, Natasha Jesudason, Rajiv Shah, James Shepherd, Antonia Ho, Emma Thomson; Alasdair MacLean, Rory Gunson.
EPI_ISL_425727 hCoV-19/Scotland/CVR195/2020 Europe / United Kingdom / Scotland 2020-03-18 West of Scotland Specialist Virology Centre, NHSGGC / MRC-University of Glasgow Centre for Virus Research COVID-19 Genomics UK (COG-UK) Consortium Ana da Silva Filipe, Kathy Smollett, Stephen Carmichael, Natasha Johnson, Daniel Mair, Lily Tong, Jenna Nichols; Sarah McDonald; Richard Orton, Joseph Hughes, Sreenu Vattipally, David L Robertson; Kathy Li, Natasha Jesudason, Rajiv Shah, James Shepherd, Antonia Ho, Emma Thomson; Alasdair MacLean, Rory Gunson.
EPI_ISL_425728 hCoV-19/Scotland/CVR196/2020 Europe / United Kingdom / Scotland 2020-03-19 West of Scotland Specialist Virology Centre, NHSGGC / MRC-University of Glasgow Centre for Virus Research COVID-19 Genomics UK (COG-UK) Consortium Ana da Silva Filipe, Kathy Smollett, Stephen Carmichael, Natasha Johnson, Daniel Mair, Lily Tong, Jenna Nichols; Sarah McDonald; Richard Orton, Joseph Hughes, Sreenu Vattipally, David L Robertson; Kathy Li, Natasha Jesudason, Rajiv Shah, James Shepherd, Antonia Ho, Emma Thomson; Alasdair MacLean, Rory Gunson.
EPI_ISL_425729 hCoV-19/Scotland/CVR199/2020 Europe / United Kingdom / Scotland 2020-03-18 West of Scotland Specialist Virology Centre, NHSGGC / MRC-University of Glasgow Centre for Virus Research COVID-19 Genomics UK (COG-UK) Consortium Ana da Silva Filipe, Kathy Smollett, Stephen Carmichael, Natasha Johnson, Daniel Mair, Lily Tong, Jenna Nichols; Sarah McDonald; Richard Orton, Joseph Hughes, Sreenu Vattipally, David L Robertson; Kathy Li, Natasha Jesudason, Rajiv Shah, James Shepherd, Antonia Ho, Emma Thomson; Alasdair MacLean, Rory Gunson.
EPI_ISL_425730 hCoV-19/Scotland/CVR20/2020 Europe / United Kingdom / Scotland 2020-03-13 West of Scotland Specialist Virology Centre, NHSGGC / MRC-University of Glasgow Centre for Virus Research COVID-

19 Genomics UK (COG-UK) Consortium Ana da Silva Filipe, Kathy Smollett, Stephen Carmichael, Natasha Johnson, Daniel Mair, Lily Tong, Jenna Nichols; Sarah McDonald; Richard Orton, Joseph Hughes, Sreenu Vattipally, David L Robertson; Kathy Li, Natasha Jesudason, Rajiv Shah, James Shepherd, Antonia Ho, Emma Thomson; Alasdair MacLean, Rory Gunson.
EPI_ISL_425731 hCoV-19/Scotland/CVR200/2020 Europe / United Kingdom / Scotland 2020-03-17 West of Scotland Specialist Virology Centre, NHSGGC / MRC-University of Glasgow Centre for Virus Research COVID-19 Genomics UK (COG-UK) Consortium Ana da Silva Filipe, Kathy Smollett, Stephen Carmichael, Natasha Johnson, Daniel Mair, Lily Tong, Jenna Nichols; Sarah McDonald; Richard Orton, Joseph Hughes, Sreenu Vattipally, David L Robertson; Kathy Li, Natasha Jesudason, Rajiv Shah, James Shepherd, Antonia Ho, Emma Thomson; Alasdair MacLean, Rory Gunson.
EPI_ISL_425732 hCoV-19/Scotland/CVR201/2020 Europe / United Kingdom / Scotland 2020-03-18 West of Scotland Specialist Virology Centre, NHSGGC / MRC-University of Glasgow Centre for Virus Research COVID-19 Genomics UK (COG-UK) Consortium Ana da Silva Filipe, Kathy Smollett, Stephen Carmichael, Natasha Johnson, Daniel Mair, Lily Tong, Jenna Nichols; Sarah McDonald; Richard Orton, Joseph Hughes, Sreenu Vattipally, David L Robertson; Kathy Li, Natasha Jesudason, Rajiv Shah, James Shepherd, Antonia Ho, Emma Thomson; Alasdair MacLean, Rory Gunson.
EPI_ISL_425733 hCoV-19/Scotland/CVR202/2020 Europe / United Kingdom / Scotland 2020-03-19 West of Scotland Specialist Virology Centre, NHSGGC / MRC-University of Glasgow Centre for Virus Research COVID-19 Genomics UK (COG-UK) Consortium Ana da Silva Filipe, Kathy Smollett, Stephen Carmichael, Natasha Johnson, Daniel Mair, Lily Tong, Jenna Nichols; Sarah McDonald; Richard Orton, Joseph Hughes, Sreenu Vattipally, David L Robertson; Kathy Li, Natasha Jesudason, Rajiv Shah, James Shepherd, Antonia Ho, Emma Thomson; Alasdair MacLean, Rory Gunson.
EPI_ISL_425734 hCoV-19/Scotland/CVR203/2020 Europe / United Kingdom / Scotland 2020-03-18 West of Scotland Specialist Virology Centre, NHSGGC / MRC-University of Glasgow Centre for Virus Research COVID-19 Genomics UK (COG-UK) Consortium Ana da Silva Filipe, Kathy Smollett, Stephen Carmichael, Natasha Johnson, Daniel Mair, Lily Tong, Jenna Nichols; Sarah McDonald; Richard Orton, Joseph Hughes, Sreenu Vattipally, David L Robertson; Kathy Li, Natasha Jesudason, Rajiv Shah, James Shepherd, Antonia Ho, Emma Thomson; Alasdair MacLean, Rory Gunson.
EPI_ISL_425735 hCoV-19/Scotland/CVR205/2020 Europe / United Kingdom / Scotland 2020-03-19 West of Scotland Specialist Virology Centre, NHSGGC / MRC-University of Glasgow Centre for Virus Research COVID-19 Genomics UK (COG-UK) Consortium Ana da Silva Filipe, Kathy Smollett, Stephen Carmichael, Natasha Johnson, Daniel Mair, Lily Tong, Jenna Nichols; Sarah McDonald; Richard Orton, Joseph Hughes, Sreenu Vattipally, David L Robertson; Kathy Li, Natasha Jesudason, Rajiv Shah, James Shepherd, Antonia Ho, Emma Thomson; Alasdair MacLean, Rory Gunson.
EPI_ISL_425736 hCoV-19/Scotland/CVR206/2020 Europe / United Kingdom / Scotland 2020-03-16 West of Scotland Specialist Virology Centre, NHSGGC / MRC-University of Glasgow Centre for Virus Research COVID-19 Genomics UK (COG-UK) Consortium Ana da Silva Filipe, Kathy Smollett, Stephen Carmichael, Natasha Johnson, Daniel Mair, Lily Tong, Jenna Nichols; Sarah McDonald; Richard Orton, Joseph Hughes, Sreenu Vattipally, David L Robertson; Kathy Li, Natasha Jesudason, Rajiv Shah, James Shepherd, Antonia Ho, Emma Thomson; Alasdair MacLean, Rory Gunson.
EPI_ISL_425737 hCoV-19/Scotland/CVR207/2020 Europe / United Kingdom / Scotland 2020-03-18 West of Scotland Specialist Virology Centre, NHSGGC / MRC-University of Glasgow Centre for Virus Research COVID-19 Genomics UK (COG-UK) Consortium Ana da Silva Filipe, Kathy Smollett, Stephen Carmichael, Natasha Johnson, Daniel Mair, Lily Tong, Jenna Nichols; Sarah McDonald; Richard Orton, Joseph Hughes, Sreenu Vattipally, David L Robertson; Kathy Li, Natasha Jesudason, Rajiv Shah, James Shepherd, Antonia Ho, Emma Thomson; Alasdair MacLean, Rory Gunson.
EPI_ISL_425739 hCoV-19/Scotland/CVR21/2020 Europe / United Kingdom / Scotland 2020-03-13 West of Scotland Specialist Virology Centre, NHSGGC / MRC-University of Glasgow Centre for Virus Research COVID-19 Genomics UK (COG-UK) Consortium Ana da Silva Filipe, Kathy Smollett, Stephen Carmichael, Natasha Johnson, Daniel Mair, Lily Tong, Jenna Nichols; Sarah McDonald; Richard Orton, Joseph Hughes, Sreenu Vattipally, David L Robertson; Kathy Li, Natasha Jesudason, Rajiv Shah, James Shepherd, Antonia Ho, Emma Thomson; Alasdair MacLean, Rory Gunson.
EPI_ISL_425741 hCoV-19/Scotland/CVR212/2020 Europe / United Kingdom / Scotland 2020-03-18 West of Scotland Specialist Virology Centre, NHSGGC / MRC-University of Glasgow Centre for Virus Research COVID-19 Genomics UK (COG-UK) Consortium Ana da Silva Filipe, Kathy Smollett, Stephen Carmichael, Natasha Johnson, Daniel Mair, Lily Tong, Jenna Nichols; Sarah McDonald; Richard Orton, Joseph Hughes, Sreenu Vattipally, David L Robertson; Kathy Li, Natasha Jesudason, Rajiv Shah, James Shepherd, Antonia Ho, Emma Thomson; Alasdair MacLean, Rory Gunson.
EPI_ISL_425742 hCoV-19/Scotland/CVR213/2020 Europe / United Kingdom / Scotland 2020-03-19 West of Scotland Specialist Virology Centre, NHSGGC / MRC-University of Glasgow Centre for Virus Research COVID-19 Genomics UK (COG-UK) Consortium Ana da Silva Filipe, Kathy Smollett, Stephen Carmichael, Natasha Johnson, Daniel Mair, Lily Tong, Jenna Nichols; Sarah McDonald; Richard Orton, Joseph Hughes, Sreenu Vattipally, David L Robertson; Kathy Li, Natasha Jesudason, Rajiv Shah, James Shepherd, Antonia Ho, Emma Thomson; Alasdair MacLean, Rory Gunson.
EPI_ISL_425743 hCoV-19/Scotland/CVR215/2020 Europe / United Kingdom / Scotland 2020-03-18 West of Scotland Specialist Virology Centre, NHSGGC / MRC-University of Glasgow Centre for Virus Research COVID-19 Genomics UK (COG-UK) Consortium Ana da Silva Filipe, Kathy Smollett, Stephen Carmichael, Natasha Johnson, Daniel Mair, Lily Tong, Jenna Nichols; Sarah McDonald; Richard Orton, Joseph Hughes, Sreenu Vattipally, David L Robertson; Kathy Li, Natasha Jesudason, Rajiv Shah, James Shepherd, Antonia Ho, Emma Thomson; Alasdair MacLean, Rory Gunson.

EPI_ISL_425744 hCoV-19/Scotland/CVR216/2020 Europe / United Kingdom / Scotland 2020-03-19 West of Scotland Specialist Virology Centre, NHSGGC / MRC-University of Glasgow Centre for Virus Research COVID-19 Genomics UK (COG-UK) Consortium Ana da Silva Filipe, Kathy Smollett, Stephen Carmichael, Natasha Johnson, Daniel Mair, Lily Tong, Jenna Nichols; Sarah McDonald; Richard Orton, Joseph Hughes, Sreenu Vattipally, David L Robertson; Kathy Li, Natasha Jesudason, Rajiv Shah, James Shepherd, Antonia Ho, Emma Thomson; Alasdair MacLean, Rory Gunson.
EPI_ISL_425745 hCoV-19/Scotland/CVR218/2020 Europe / United Kingdom / Scotland 2020-03-19 West of Scotland Specialist Virology Centre, NHSGGC / MRC-University of Glasgow Centre for Virus Research COVID-19 Genomics UK (COG-UK) Consortium Ana da Silva Filipe, Kathy Smollett, Stephen Carmichael, Natasha Johnson, Daniel Mair, Lily Tong, Jenna Nichols; Sarah McDonald; Richard Orton, Joseph Hughes, Sreenu Vattipally, David L Robertson; Kathy Li, Natasha Jesudason, Rajiv Shah, James Shepherd, Antonia Ho, Emma Thomson; Alasdair MacLean, Rory Gunson.
EPI_ISL_425746 hCoV-19/Scotland/CVR219/2020 Europe / United Kingdom / Scotland 2020-03-19 West of Scotland Specialist Virology Centre, NHSGGC / MRC-University of Glasgow Centre for Virus Research COVID-19 Genomics UK (COG-UK) Consortium Ana da Silva Filipe, Kathy Smollett, Stephen Carmichael, Natasha Johnson, Daniel Mair, Lily Tong, Jenna Nichols; Sarah McDonald; Richard Orton, Joseph Hughes, Sreenu Vattipally, David L Robertson; Kathy Li, Natasha Jesudason, Rajiv Shah, James Shepherd, Antonia Ho, Emma Thomson; Alasdair MacLean, Rory Gunson.
EPI_ISL_425747 hCoV-19/Scotland/CVR220/2020 Europe / United Kingdom / Scotland 2020-03-18 West of Scotland Specialist Virology Centre, NHSGGC / MRC-University of Glasgow Centre for Virus Research COVID-19 Genomics UK (COG-UK) Consortium Ana da Silva Filipe, Kathy Smollett, Stephen Carmichael, Natasha Johnson, Daniel Mair, Lily Tong, Jenna Nichols; Sarah McDonald; Richard Orton, Joseph Hughes, Sreenu Vattipally, David L Robertson; Kathy Li, Natasha Jesudason, Rajiv Shah, James Shepherd, Antonia Ho, Emma Thomson; Alasdair MacLean, Rory Gunson.
EPI_ISL_425749 hCoV-19/Scotland/CVR23/2020 Europe / United Kingdom / Scotland 2020-03-12 West of Scotland Specialist Virology Centre, NHSGGC / MRC-University of Glasgow Centre for Virus Research COVID-19 Genomics UK (COG-UK) Consortium Ana da Silva Filipe, Kathy Smollett, Stephen Carmichael, Natasha Johnson, Daniel Mair, Lily Tong, Jenna Nichols; Sarah McDonald; Richard Orton, Joseph Hughes, Sreenu Vattipally, David L Robertson; Kathy Li, Natasha Jesudason, Rajiv Shah, James Shepherd, Antonia Ho, Emma Thomson; Alasdair MacLean, Rory Gunson.
EPI_ISL_425750 hCoV-19/Scotland/CVR235/2020 Europe / United Kingdom / Scotland 2020-03-19 West of Scotland Specialist Virology Centre, NHSGGC / MRC-University of Glasgow Centre for Virus Research COVID-19 Genomics UK (COG-UK) Consortium Ana da Silva Filipe, Kathy Smollett, Stephen Carmichael, Natasha Johnson, Daniel Mair, Lily Tong, Jenna Nichols; Sarah McDonald; Richard Orton, Joseph Hughes, Sreenu Vattipally, David L Robertson; Kathy Li, Natasha Jesudason, Rajiv Shah, James Shepherd, Antonia Ho, Emma Thomson; Alasdair MacLean, Rory Gunson.
EPI_ISL_425751 hCoV-19/Scotland/CVR236/2020 Europe / United Kingdom / Scotland 2020-03-19 West of Scotland Specialist Virology Centre, NHSGGC / MRC-University of Glasgow Centre for Virus Research COVID-19 Genomics UK (COG-UK) Consortium Ana da Silva Filipe, Kathy Smollett, Stephen Carmichael, Natasha Johnson, Daniel Mair, Lily Tong, Jenna Nichols; Sarah McDonald; Richard Orton, Joseph Hughes, Sreenu Vattipally, David L Robertson; Kathy Li, Natasha Jesudason, Rajiv Shah, James Shepherd, Antonia Ho, Emma Thomson; Alasdair MacLean, Rory Gunson.
EPI_ISL_425752 hCoV-19/Scotland/CVR24/2020 Europe / United Kingdom / Scotland 2020-03-12 West of Scotland Specialist Virology Centre, NHSGGC / MRC-University of Glasgow Centre for Virus Research COVID-19 Genomics UK (COG-UK) Consortium Ana da Silva Filipe, Kathy Smollett, Stephen Carmichael, Natasha Johnson, Daniel Mair, Lily Tong, Jenna Nichols; Sarah McDonald; Richard Orton, Joseph Hughes, Sreenu Vattipally, David L Robertson; Kathy Li, Natasha Jesudason, Rajiv Shah, James Shepherd, Antonia Ho, Emma Thomson; Alasdair MacLean, Rory Gunson.
EPI_ISL_425754 hCoV-19/Scotland/CVR264/2020 Europe / United Kingdom / Scotland 2020-03-20 West of Scotland Specialist Virology Centre, NHSGGC / MRC-University of Glasgow Centre for Virus Research COVID-19 Genomics UK (COG-UK) Consortium Ana da Silva Filipe, Kathy Smollett, Stephen Carmichael, Natasha Johnson, Daniel Mair, Lily Tong, Jenna Nichols; Sarah McDonald; Richard Orton, Joseph Hughes, Sreenu Vattipally, David L Robertson; Kathy Li, Natasha Jesudason, Rajiv Shah, James Shepherd, Antonia Ho, Emma Thomson; Alasdair MacLean, Rory Gunson.
EPI_ISL_425755 hCoV-19/Scotland/CVR268/2020 Europe / United Kingdom / Scotland 2020-03-19 West of Scotland Specialist Virology Centre, NHSGGC / MRC-University of Glasgow Centre for Virus Research COVID-19 Genomics UK (COG-UK) Consortium Ana da Silva Filipe, Kathy Smollett, Stephen Carmichael, Natasha Johnson, Daniel Mair, Lily Tong, Jenna Nichols; Sarah McDonald; Richard Orton, Joseph Hughes, Sreenu Vattipally, David L Robertson; Kathy Li, Natasha Jesudason, Rajiv Shah, James Shepherd, Antonia Ho, Emma Thomson; Alasdair MacLean, Rory Gunson.
EPI_ISL_425756 hCoV-19/Scotland/CVR27/2020 Europe / United Kingdom / Scotland 2020-03-12 West of Scotland Specialist Virology Centre, NHSGGC / MRC-University of Glasgow Centre for Virus Research COVID-19 Genomics UK (COG-UK) Consortium Ana da Silva Filipe, Kathy Smollett, Stephen Carmichael, Natasha Johnson, Daniel Mair, Lily Tong, Jenna Nichols; Sarah McDonald; Richard Orton, Joseph Hughes, Sreenu Vattipally, David L Robertson; Kathy Li, Natasha Jesudason, Rajiv Shah, James Shepherd, Antonia Ho, Emma Thomson; Alasdair MacLean, Rory Gunson.
EPI_ISL_425757 hCoV-19/Scotland/CVR28/2020 Europe / United Kingdom / Scotland 2020-03-12 West of Scotland Specialist Virology Centre, NHSGGC / MRC-University of Glasgow Centre for Virus Research COVID-19 Genomics UK (COG-UK) Consortium Ana da Silva Filipe, Kathy Smollett, Stephen Carmichael, Natasha Johnson, Daniel Mair, Lily Tong, Jenna Nichols; Sarah McDonald; Richard Orton, Joseph Hughes, Sreenu

Vattipally, David L Robertson; Kathy Li, Natasha Jesudason, Rajiv Shah, James Shepherd, Antonia Ho, Emma Thomson; Alasdair MacLean, Rory Gunson.
EPI_ISL_425758 hCoV-19/Scotland/CVR29/2020 Europe / United Kingdom / Scotland 2020-03-11 West of Scotland Specialist Virology Centre, NHSGGC / MRC-University of Glasgow Centre for Virus Research COVID-19 Genomics UK (COG-UK) Consortium Ana da Silva Filipe, Kathy Smollett, Stephen Carmichael, Natasha Johnson, Daniel Mair, Lily Tong, Jenna Nichols; Sarah McDonald; Richard Orton, Joseph Hughes, Sreenu Vattipally, David L Robertson; Kathy Li, Natasha Jesudason, Rajiv Shah, James Shepherd, Antonia Ho, Emma Thomson; Alasdair MacLean, Rory Gunson.
EPI_ISL_425759 hCoV-19/Scotland/CVR30/2020 Europe / United Kingdom / Scotland 2020-03-13 West of Scotland Specialist Virology Centre, NHSGGC / MRC-University of Glasgow Centre for Virus Research COVID-19 Genomics UK (COG-UK) Consortium Ana da Silva Filipe, Kathy Smollett, Stephen Carmichael, Natasha Johnson, Daniel Mair, Lily Tong, Jenna Nichols; Sarah McDonald; Richard Orton, Joseph Hughes, Sreenu Vattipally, David L Robertson; Kathy Li, Natasha Jesudason, Rajiv Shah, James Shepherd, Antonia Ho, Emma Thomson; Alasdair MacLean, Rory Gunson.
EPI_ISL_425760 hCoV-19/Scotland/CVR31/2020 Europe / United Kingdom / Scotland 2020-03-13 West of Scotland Specialist Virology Centre, NHSGGC / MRC-University of Glasgow Centre for Virus Research COVID-19 Genomics UK (COG-UK) Consortium Ana da Silva Filipe, Kathy Smollett, Stephen Carmichael, Natasha Johnson, Daniel Mair, Lily Tong, Jenna Nichols; Sarah McDonald; Richard Orton, Joseph Hughes, Sreenu Vattipally, David L Robertson; Kathy Li, Natasha Jesudason, Rajiv Shah, James Shepherd, Antonia Ho, Emma Thomson; Alasdair MacLean, Rory Gunson.
EPI_ISL_425761 hCoV-19/Scotland/CVR32/2020 Europe / United Kingdom / Scotland 2020-03-13 West of Scotland Specialist Virology Centre, NHSGGC / MRC-University of Glasgow Centre for Virus Research COVID-19 Genomics UK (COG-UK) Consortium Ana da Silva Filipe, Kathy Smollett, Stephen Carmichael, Natasha Johnson, Daniel Mair, Lily Tong, Jenna Nichols; Sarah McDonald; Richard Orton, Joseph Hughes, Sreenu Vattipally, David L Robertson; Kathy Li, Natasha Jesudason, Rajiv Shah, James Shepherd, Antonia Ho, Emma Thomson; Alasdair MacLean, Rory Gunson.
EPI_ISL_425762 hCoV-19/Scotland/CVR33/2020 Europe / United Kingdom / Scotland 2020-03-13 West of Scotland Specialist Virology Centre, NHSGGC / MRC-University of Glasgow Centre for Virus Research COVID-19 Genomics UK (COG-UK) Consortium Ana da Silva Filipe, Kathy Smollett, Stephen Carmichael, Natasha Johnson, Daniel Mair, Lily Tong, Jenna Nichols; Sarah McDonald; Richard Orton, Joseph Hughes, Sreenu Vattipally, David L Robertson; Kathy Li, Natasha Jesudason, Rajiv Shah, James Shepherd, Antonia Ho, Emma Thomson; Alasdair MacLean, Rory Gunson.
EPI_ISL_425763 hCoV-19/Scotland/CVR34/2020 Europe / United Kingdom / Scotland 2020-03-12 West of Scotland Specialist Virology Centre, NHSGGC / MRC-University of Glasgow Centre for Virus Research COVID-19 Genomics UK (COG-UK) Consortium Ana da Silva Filipe, Kathy Smollett, Stephen Carmichael, Natasha Johnson, Daniel Mair, Lily Tong, Jenna Nichols; Sarah McDonald; Richard Orton, Joseph Hughes, Sreenu Vattipally, David L Robertson; Kathy Li, Natasha Jesudason, Rajiv Shah, James Shepherd, Antonia Ho, Emma Thomson; Alasdair MacLean, Rory Gunson.
EPI_ISL_425764 hCoV-19/Scotland/CVR35/2020 Europe / United Kingdom / Scotland 2020-03-13 West of Scotland Specialist Virology Centre, NHSGGC / MRC-University of Glasgow Centre for Virus Research COVID-19 Genomics UK (COG-UK) Consortium Ana da Silva Filipe, Kathy Smollett, Stephen Carmichael, Natasha Johnson, Daniel Mair, Lily Tong, Jenna Nichols; Sarah McDonald; Richard Orton, Joseph Hughes, Sreenu Vattipally, David L Robertson; Kathy Li, Natasha Jesudason, Rajiv Shah, James Shepherd, Antonia Ho, Emma Thomson; Alasdair MacLean, Rory Gunson.
EPI_ISL_425765 hCoV-19/Scotland/CVR36/2020 Europe / United Kingdom / Scotland 2020-03-13 West of Scotland Specialist Virology Centre, NHSGGC / MRC-University of Glasgow Centre for Virus Research COVID-19 Genomics UK (COG-UK) Consortium Ana da Silva Filipe, Kathy Smollett, Stephen Carmichael, Natasha Johnson, Daniel Mair, Lily Tong, Jenna Nichols; Sarah McDonald; Richard Orton, Joseph Hughes, Sreenu Vattipally, David L Robertson; Kathy Li, Natasha Jesudason, Rajiv Shah, James Shepherd, Antonia Ho, Emma Thomson; Alasdair MacLean, Rory Gunson.
EPI_ISL_425766 hCoV-19/Scotland/CVR37/2020 Europe / United Kingdom / Scotland 2020-03-13 West of Scotland Specialist Virology Centre, NHSGGC / MRC-University of Glasgow Centre for Virus Research COVID-19 Genomics UK (COG-UK) Consortium Ana da Silva Filipe, Kathy Smollett, Stephen Carmichael, Natasha Johnson, Daniel Mair, Lily Tong, Jenna Nichols; Sarah McDonald; Richard Orton, Joseph Hughes, Sreenu Vattipally, David L Robertson; Kathy Li, Natasha Jesudason, Rajiv Shah, James Shepherd, Antonia Ho, Emma Thomson; Alasdair MacLean, Rory Gunson.
EPI_ISL_425767 hCoV-19/Scotland/CVR38/2020 Europe / United Kingdom / Scotland 2020-03-13 West of Scotland Specialist Virology Centre, NHSGGC / MRC-University of Glasgow Centre for Virus Research COVID-19 Genomics UK (COG-UK) Consortium Ana da Silva Filipe, Kathy Smollett, Stephen Carmichael, Natasha Johnson, Daniel Mair, Lily Tong, Jenna Nichols; Sarah McDonald; Richard Orton, Joseph Hughes, Sreenu Vattipally, David L Robertson; Kathy Li, Natasha Jesudason, Rajiv Shah, James Shepherd, Antonia Ho, Emma Thomson; Alasdair MacLean, Rory Gunson.
EPI_ISL_425768 hCoV-19/Scotland/CVR40/2020 Europe / United Kingdom / Scotland 2020-03-13 West of Scotland Specialist Virology Centre, NHSGGC / MRC-University of Glasgow Centre for Virus Research COVID-19 Genomics UK (COG-UK) Consortium Ana da Silva Filipe, Kathy Smollett, Stephen Carmichael, Natasha Johnson, Daniel Mair, Lily Tong, Jenna Nichols; Sarah McDonald; Richard Orton, Joseph Hughes, Sreenu Vattipally, David L Robertson; Kathy Li, Natasha Jesudason, Rajiv Shah, James Shepherd, Antonia Ho, Emma Thomson; Alasdair MacLean, Rory Gunson.
EPI_ISL_425769 hCoV-19/Scotland/CVR41/2020 Europe / United Kingdom / Scotland 2020-03-13 West of Scotland Specialist Virology Centre, NHSGGC / MRC-University of Glasgow Centre for Virus Research COVID-

19 Genomics UK (COG-UK) Consortium Ana da Silva Filipe, Kathy Smollett, Stephen Carmichael, Natasha Johnson, Daniel Mair, Lily Tong, Jenna Nichols; Sarah McDonald; Richard Orton, Joseph Hughes, Sreenu Vattipally, David L Robertson; Kathy Li, Natasha Jesudason, Rajiv Shah, James Shepherd, Antonia Ho, Emma Thomson; Alasdair MacLean, Rory Gunson.
EPI_ISL_425770 hCoV-19/Scotland/CVR42/2020 Europe / United Kingdom / Scotland 2020-03-13 West of Scotland Specialist Virology Centre, NHSGGC / MRC-University of Glasgow Centre for Virus Research COVID-19 Genomics UK (COG-UK) Consortium Ana da Silva Filipe, Kathy Smollett, Stephen Carmichael, Natasha Johnson, Daniel Mair, Lily Tong, Jenna Nichols; Sarah McDonald; Richard Orton, Joseph Hughes, Sreenu Vattipally, David L Robertson; Kathy Li, Natasha Jesudason, Rajiv Shah, James Shepherd, Antonia Ho, Emma Thomson; Alasdair MacLean, Rory Gunson.
EPI_ISL_425772 hCoV-19/Scotland/CVR44/2020 Europe / United Kingdom / Scotland 2020-03-13 West of Scotland Specialist Virology Centre, NHSGGC / MRC-University of Glasgow Centre for Virus Research COVID-19 Genomics UK (COG-UK) Consortium Ana da Silva Filipe, Kathy Smollett, Stephen Carmichael, Natasha Johnson, Daniel Mair, Lily Tong, Jenna Nichols; Sarah McDonald; Richard Orton, Joseph Hughes, Sreenu Vattipally, David L Robertson; Kathy Li, Natasha Jesudason, Rajiv Shah, James Shepherd, Antonia Ho, Emma Thomson; Alasdair MacLean, Rory Gunson.
EPI_ISL_425773 hCoV-19/Scotland/CVR45/2020 Europe / United Kingdom / Scotland 2020-03-13 West of Scotland Specialist Virology Centre, NHSGGC / MRC-University of Glasgow Centre for Virus Research COVID-19 Genomics UK (COG-UK) Consortium Ana da Silva Filipe, Kathy Smollett, Stephen Carmichael, Natasha Johnson, Daniel Mair, Lily Tong, Jenna Nichols; Sarah McDonald; Richard Orton, Joseph Hughes, Sreenu Vattipally, David L Robertson; Kathy Li, Natasha Jesudason, Rajiv Shah, James Shepherd, Antonia Ho, Emma Thomson; Alasdair MacLean, Rory Gunson.
EPI_ISL_425774 hCoV-19/Scotland/CVR46/2020 Europe / United Kingdom / Scotland 2020-03-13 West of Scotland Specialist Virology Centre, NHSGGC / MRC-University of Glasgow Centre for Virus Research COVID-19 Genomics UK (COG-UK) Consortium Ana da Silva Filipe, Kathy Smollett, Stephen Carmichael, Natasha Johnson, Daniel Mair, Lily Tong, Jenna Nichols; Sarah McDonald; Richard Orton, Joseph Hughes, Sreenu Vattipally, David L Robertson; Kathy Li, Natasha Jesudason, Rajiv Shah, James Shepherd, Antonia Ho, Emma Thomson; Alasdair MacLean, Rory Gunson.
EPI_ISL_425775 hCoV-19/Scotland/CVR462/2020 Europe / United Kingdom / Scotland 2020-03-23 West of Scotland Specialist Virology Centre, NHSGGC / MRC-University of Glasgow Centre for Virus Research COVID-19 Genomics UK (COG-UK) Consortium Ana da Silva Filipe, Kathy Smollett, Stephen Carmichael, Natasha Johnson, Daniel Mair, Lily Tong, Jenna Nichols; Sarah McDonald; Richard Orton, Joseph Hughes, Sreenu Vattipally, David L Robertson; Kathy Li, Natasha Jesudason, Rajiv Shah, James Shepherd, Antonia Ho, Emma Thomson; Alasdair MacLean, Rory Gunson.
EPI_ISL_425776 hCoV-19/Scotland/CVR47/2020 Europe / United Kingdom / Scotland 2020-03-13 West of Scotland Specialist Virology Centre, NHSGGC / MRC-University of Glasgow Centre for Virus Research COVID-19 Genomics UK (COG-UK) Consortium Ana da Silva Filipe, Kathy Smollett, Stephen Carmichael, Natasha Johnson, Daniel Mair, Lily Tong, Jenna Nichols; Sarah McDonald; Richard Orton, Joseph Hughes, Sreenu Vattipally, David L Robertson; Kathy Li, Natasha Jesudason, Rajiv Shah, James Shepherd, Antonia Ho, Emma Thomson; Alasdair MacLean, Rory Gunson.
EPI_ISL_425777 hCoV-19/Scotland/CVR49/2020 Europe / United Kingdom / Scotland 2020-03-13 West of Scotland Specialist Virology Centre, NHSGGC / MRC-University of Glasgow Centre for Virus Research COVID-19 Genomics UK (COG-UK) Consortium Ana da Silva Filipe, Kathy Smollett, Stephen Carmichael, Natasha Johnson, Daniel Mair, Lily Tong, Jenna Nichols; Sarah McDonald; Richard Orton, Joseph Hughes, Sreenu Vattipally, David L Robertson; Kathy Li, Natasha Jesudason, Rajiv Shah, James Shepherd, Antonia Ho, Emma Thomson; Alasdair MacLean, Rory Gunson.
EPI_ISL_425778 hCoV-19/Scotland/CVR50/2020 Europe / United Kingdom / Scotland 2020-03-13 West of Scotland Specialist Virology Centre, NHSGGC / MRC-University of Glasgow Centre for Virus Research COVID-19 Genomics UK (COG-UK) Consortium Ana da Silva Filipe, Kathy Smollett, Stephen Carmichael, Natasha Johnson, Daniel Mair, Lily Tong, Jenna Nichols; Sarah McDonald; Richard Orton, Joseph Hughes, Sreenu Vattipally, David L Robertson; Kathy Li, Natasha Jesudason, Rajiv Shah, James Shepherd, Antonia Ho, Emma Thomson; Alasdair MacLean, Rory Gunson.
EPI_ISL_425779 hCoV-19/Scotland/CVR51/2020 Europe / United Kingdom / Scotland 2020-03-13 West of Scotland Specialist Virology Centre, NHSGGC / MRC-University of Glasgow Centre for Virus Research COVID-19 Genomics UK (COG-UK) Consortium Ana da Silva Filipe, Kathy Smollett, Stephen Carmichael, Natasha Johnson, Daniel Mair, Lily Tong, Jenna Nichols; Sarah McDonald; Richard Orton, Joseph Hughes, Sreenu Vattipally, David L Robertson; Kathy Li, Natasha Jesudason, Rajiv Shah, James Shepherd, Antonia Ho, Emma Thomson; Alasdair MacLean, Rory Gunson.
EPI_ISL_425780 hCoV-19/Scotland/CVR52/2020 Europe / United Kingdom / Scotland 2020-03-14 West of Scotland Specialist Virology Centre, NHSGGC / MRC-University of Glasgow Centre for Virus Research COVID-19 Genomics UK (COG-UK) Consortium Ana da Silva Filipe, Kathy Smollett, Stephen Carmichael, Natasha Johnson, Daniel Mair, Lily Tong, Jenna Nichols; Sarah McDonald; Richard Orton, Joseph Hughes, Sreenu Vattipally, David L Robertson; Kathy Li, Natasha Jesudason, Rajiv Shah, James Shepherd, Antonia Ho, Emma Thomson; Alasdair MacLean, Rory Gunson.
EPI_ISL_425782 hCoV-19/Scotland/CVR53/2020 Europe / United Kingdom / Scotland 2020-03-14 West of Scotland Specialist Virology Centre, NHSGGC / MRC-University of Glasgow Centre for Virus Research COVID-19 Genomics UK (COG-UK) Consortium Ana da Silva Filipe, Kathy Smollett, Stephen Carmichael, Natasha Johnson, Daniel Mair, Lily Tong, Jenna Nichols; Sarah McDonald; Richard Orton, Joseph Hughes, Sreenu Vattipally, David L Robertson; Kathy Li, Natasha Jesudason, Rajiv Shah, James Shepherd, Antonia Ho, Emma Thomson; Alasdair MacLean, Rory Gunson.

EPI_ISL_425783 hCoV-19/Scotland/CVR54/2020 Europe / United Kingdom / Scotland 2020-03-13 West of Scotland Specialist Virology Centre, NHSGGC / MRC-University of Glasgow Centre for Virus Research COVID-19 Genomics UK (COG-UK) Consortium Ana da Silva Filipe, Kathy Smollett, Stephen Carmichael, Natasha Johnson, Daniel Mair, Lily Tong, Jenna Nichols; Sarah McDonald; Richard Orton, Joseph Hughes, Sreenu Vattipally, David L Robertson; Kathy Li, Natasha Jesudason, Rajiv Shah, James Shepherd, Antonia Ho, Emma Thomson; Alasdair MacLean, Rory Gunson.
EPI_ISL_425784 hCoV-19/Scotland/CVR55/2020 Europe / United Kingdom / Scotland 2020-03-13 West of Scotland Specialist Virology Centre, NHSGGC / MRC-University of Glasgow Centre for Virus Research COVID-19 Genomics UK (COG-UK) Consortium Ana da Silva Filipe, Kathy Smollett, Stephen Carmichael, Natasha Johnson, Daniel Mair, Lily Tong, Jenna Nichols; Sarah McDonald; Richard Orton, Joseph Hughes, Sreenu Vattipally, David L Robertson; Kathy Li, Natasha Jesudason, Rajiv Shah, James Shepherd, Antonia Ho, Emma Thomson; Alasdair MacLean, Rory Gunson.
EPI_ISL_425785 hCoV-19/Scotland/CVR56/2020 Europe / United Kingdom / Scotland 2020-03-12 West of Scotland Specialist Virology Centre, NHSGGC / MRC-University of Glasgow Centre for Virus Research COVID-19 Genomics UK (COG-UK) Consortium Ana da Silva Filipe, Kathy Smollett, Stephen Carmichael, Natasha Johnson, Daniel Mair, Lily Tong, Jenna Nichols; Sarah McDonald; Richard Orton, Joseph Hughes, Sreenu Vattipally, David L Robertson; Kathy Li, Natasha Jesudason, Rajiv Shah, James Shepherd, Antonia Ho, Emma Thomson; Alasdair MacLean, Rory Gunson.
EPI_ISL_425786 hCoV-19/Scotland/CVR562/2020 Europe / United Kingdom / Scotland 2020-03-23 West of Scotland Specialist Virology Centre, NHSGGC / MRC-University of Glasgow Centre for Virus Research COVID-19 Genomics UK (COG-UK) Consortium Ana da Silva Filipe, Kathy Smollett, Stephen Carmichael, Natasha Johnson, Daniel Mair, Lily Tong, Jenna Nichols; Sarah McDonald; Richard Orton, Joseph Hughes, Sreenu Vattipally, David L Robertson; Kathy Li, Natasha Jesudason, Rajiv Shah, James Shepherd, Antonia Ho, Emma Thomson; Alasdair MacLean, Rory Gunson.
EPI_ISL_425787 hCoV-19/Scotland/CVR57/2020 Europe / United Kingdom / Scotland 2020-03-12 West of Scotland Specialist Virology Centre, NHSGGC / MRC-University of Glasgow Centre for Virus Research COVID-19 Genomics UK (COG-UK) Consortium Ana da Silva Filipe, Kathy Smollett, Stephen Carmichael, Natasha Johnson, Daniel Mair, Lily Tong, Jenna Nichols; Sarah McDonald; Richard Orton, Joseph Hughes, Sreenu Vattipally, David L Robertson; Kathy Li, Natasha Jesudason, Rajiv Shah, James Shepherd, Antonia Ho, Emma Thomson; Alasdair MacLean, Rory Gunson.
EPI_ISL_425788 hCoV-19/Scotland/CVR58/2020 Europe / United Kingdom / Scotland 2020-03-12 West of Scotland Specialist Virology Centre, NHSGGC / MRC-University of Glasgow Centre for Virus Research COVID-19 Genomics UK (COG-UK) Consortium Ana da Silva Filipe, Kathy Smollett, Stephen Carmichael, Natasha Johnson, Daniel Mair, Lily Tong, Jenna Nichols; Sarah McDonald; Richard Orton, Joseph Hughes, Sreenu Vattipally, David L Robertson; Kathy Li, Natasha Jesudason, Rajiv Shah, James Shepherd, Antonia Ho, Emma Thomson; Alasdair MacLean, Rory Gunson.
EPI_ISL_425789 hCoV-19/Scotland/CVR60/2020 Europe / United Kingdom / Scotland 2020-03-12 West of Scotland Specialist Virology Centre, NHSGGC / MRC-University of Glasgow Centre for Virus Research COVID-19 Genomics UK (COG-UK) Consortium Ana da Silva Filipe, Kathy Smollett, Stephen Carmichael, Natasha Johnson, Daniel Mair, Lily Tong, Jenna Nichols; Sarah McDonald; Richard Orton, Joseph Hughes, Sreenu Vattipally, David L Robertson; Kathy Li, Natasha Jesudason, Rajiv Shah, James Shepherd, Antonia Ho, Emma Thomson; Alasdair MacLean, Rory Gunson.
EPI_ISL_425790 hCoV-19/Scotland/CVR62/2020 Europe / United Kingdom / Scotland 2020-03-12 West of Scotland Specialist Virology Centre, NHSGGC / MRC-University of Glasgow Centre for Virus Research COVID-19 Genomics UK (COG-UK) Consortium Ana da Silva Filipe, Kathy Smollett, Stephen Carmichael, Natasha Johnson, Daniel Mair, Lily Tong, Jenna Nichols; Sarah McDonald; Richard Orton, Joseph Hughes, Sreenu Vattipally, David L Robertson; Kathy Li, Natasha Jesudason, Rajiv Shah, James Shepherd, Antonia Ho, Emma Thomson; Alasdair MacLean, Rory Gunson.
EPI_ISL_425791 hCoV-19/Scotland/CVR63/2020 Europe / United Kingdom / Scotland 2020-03-13 West of Scotland Specialist Virology Centre, NHSGGC / MRC-University of Glasgow Centre for Virus Research COVID-19 Genomics UK (COG-UK) Consortium Ana da Silva Filipe, Kathy Smollett, Stephen Carmichael, Natasha Johnson, Daniel Mair, Lily Tong, Jenna Nichols; Sarah McDonald; Richard Orton, Joseph Hughes, Sreenu Vattipally, David L Robertson; Kathy Li, Natasha Jesudason, Rajiv Shah, James Shepherd, Antonia Ho, Emma Thomson; Alasdair MacLean, Rory Gunson.
EPI_ISL_425792 hCoV-19/Scotland/CVR632/2020 Europe / United Kingdom / Scotland 2020-03-25 West of Scotland Specialist Virology Centre, NHSGGC / MRC-University of Glasgow Centre for Virus Research COVID-19 Genomics UK (COG-UK) Consortium Ana da Silva Filipe, Kathy Smollett, Stephen Carmichael, Natasha Johnson, Daniel Mair, Lily Tong, Jenna Nichols; Sarah McDonald; Richard Orton, Joseph Hughes, Sreenu Vattipally, David L Robertson; Kathy Li, Natasha Jesudason, Rajiv Shah, James Shepherd, Antonia Ho, Emma Thomson; Alasdair MacLean, Rory Gunson.
EPI_ISL_425793 hCoV-19/Scotland/CVR634/2020 Europe / United Kingdom / Scotland 2020-03-25 West of Scotland Specialist Virology Centre, NHSGGC / MRC-University of Glasgow Centre for Virus Research COVID-19 Genomics UK (COG-UK) Consortium Ana da Silva Filipe, Kathy Smollett, Stephen Carmichael, Natasha Johnson, Daniel Mair, Lily Tong, Jenna Nichols; Sarah McDonald; Richard Orton, Joseph Hughes, Sreenu Vattipally, David L Robertson; Kathy Li, Natasha Jesudason, Rajiv Shah, James Shepherd, Antonia Ho, Emma Thomson; Alasdair MacLean, Rory Gunson.
EPI_ISL_425794 hCoV-19/Scotland/CVR635/2020 Europe / United Kingdom / Scotland 2020-03-25 West of Scotland Specialist Virology Centre, NHSGGC / MRC-University of Glasgow Centre for Virus Research COVID-19 Genomics UK (COG-UK) Consortium Ana da Silva Filipe, Kathy Smollett, Stephen Carmichael, Natasha Johnson, Daniel Mair, Lily Tong, Jenna Nichols; Sarah McDonald; Richard Orton, Joseph Hughes, Sreenu

Vattipally, David L Robertson; Kathy Li, Natasha Jesudason, Rajiv Shah, James Shepherd, Antonia Ho, Emma Thomson; Alasdair MacLean, Rory Gunson.
EPI_ISL_425795 hCoV-19/Scotland/CVR64/2020 Europe / United Kingdom / Scotland 2020-03-03 West of Scotland Specialist Virology Centre, NHSGGC / MRC-University of Glasgow Centre for Virus Research COVID-19 Genomics UK (COG-UK) Consortium Ana da Silva Filipe, Kathy Smollett, Stephen Carmichael, Natasha Johnson, Daniel Mair, Lily Tong, Jenna Nichols; Sarah McDonald; Richard Orton, Joseph Hughes, Sreenu Vattipally, David L Robertson; Kathy Li, Natasha Jesudason, Rajiv Shah, James Shepherd, Antonia Ho, Emma Thomson; Alasdair MacLean, Rory Gunson.
EPI_ISL_425796 hCoV-19/Scotland/CVR65/2020 Europe / United Kingdom / Scotland 2020-03-06 West of Scotland Specialist Virology Centre, NHSGGC / MRC-University of Glasgow Centre for Virus Research COVID-19 Genomics UK (COG-UK) Consortium Ana da Silva Filipe, Kathy Smollett, Stephen Carmichael, Natasha Johnson, Daniel Mair, Lily Tong, Jenna Nichols; Sarah McDonald; Richard Orton, Joseph Hughes, Sreenu Vattipally, David L Robertson; Kathy Li, Natasha Jesudason, Rajiv Shah, James Shepherd, Antonia Ho, Emma Thomson; Alasdair MacLean, Rory Gunson.
EPI_ISL_425797 hCoV-19/Scotland/CVR66/2020 Europe / United Kingdom / Scotland 2020-03-06 West of Scotland Specialist Virology Centre, NHSGGC / MRC-University of Glasgow Centre for Virus Research COVID-19 Genomics UK (COG-UK) Consortium Ana da Silva Filipe, Kathy Smollett, Stephen Carmichael, Natasha Johnson, Daniel Mair, Lily Tong, Jenna Nichols; Sarah McDonald; Richard Orton, Joseph Hughes, Sreenu Vattipally, David L Robertson; Kathy Li, Natasha Jesudason, Rajiv Shah, James Shepherd, Antonia Ho, Emma Thomson; Alasdair MacLean, Rory Gunson.
EPI_ISL_425798 hCoV-19/Scotland/CVR67/2020 Europe / United Kingdom / Scotland 2020-03-07 West of Scotland Specialist Virology Centre, NHSGGC / MRC-University of Glasgow Centre for Virus Research COVID-19 Genomics UK (COG-UK) Consortium Ana da Silva Filipe, Kathy Smollett, Stephen Carmichael, Natasha Johnson, Daniel Mair, Lily Tong, Jenna Nichols; Sarah McDonald; Richard Orton, Joseph Hughes, Sreenu Vattipally, David L Robertson; Kathy Li, Natasha Jesudason, Rajiv Shah, James Shepherd, Antonia Ho, Emma Thomson; Alasdair MacLean, Rory Gunson.
EPI_ISL_425799 hCoV-19/Scotland/CVR68/2020 Europe / United Kingdom / Scotland 2020-03-09 West of Scotland Specialist Virology Centre, NHSGGC / MRC-University of Glasgow Centre for Virus Research COVID-19 Genomics UK (COG-UK) Consortium Ana da Silva Filipe, Kathy Smollett, Stephen Carmichael, Natasha Johnson, Daniel Mair, Lily Tong, Jenna Nichols; Sarah McDonald; Richard Orton, Joseph Hughes, Sreenu Vattipally, David L Robertson; Kathy Li, Natasha Jesudason, Rajiv Shah, James Shepherd, Antonia Ho, Emma Thomson; Alasdair MacLean, Rory Gunson.
EPI_ISL_425800 hCoV-19/Scotland/CVR69/2020 Europe / United Kingdom / Scotland 2020-03-10 West of Scotland Specialist Virology Centre, NHSGGC / MRC-University of Glasgow Centre for Virus Research COVID-19 Genomics UK (COG-UK) Consortium Ana da Silva Filipe, Kathy Smollett, Stephen Carmichael, Natasha Johnson, Daniel Mair, Lily Tong, Jenna Nichols; Sarah McDonald; Richard Orton, Joseph Hughes, Sreenu Vattipally, David L Robertson; Kathy Li, Natasha Jesudason, Rajiv Shah, James Shepherd, Antonia Ho, Emma Thomson; Alasdair MacLean, Rory Gunson.
EPI_ISL_425801 hCoV-19/Scotland/CVR70/2020 Europe / United Kingdom / Scotland 2020-03-09 West of Scotland Specialist Virology Centre, NHSGGC / MRC-University of Glasgow Centre for Virus Research COVID-19 Genomics UK (COG-UK) Consortium Ana da Silva Filipe, Kathy Smollett, Stephen Carmichael, Natasha Johnson, Daniel Mair, Lily Tong, Jenna Nichols; Sarah McDonald; Richard Orton, Joseph Hughes, Sreenu Vattipally, David L Robertson; Kathy Li, Natasha Jesudason, Rajiv Shah, James Shepherd, Antonia Ho, Emma Thomson; Alasdair MacLean, Rory Gunson.
EPI_ISL_425802 hCoV-19/Scotland/CVR71/2020 Europe / United Kingdom / Scotland 2020-03-06 West of Scotland Specialist Virology Centre, NHSGGC / MRC-University of Glasgow Centre for Virus Research COVID-19 Genomics UK (COG-UK) Consortium Ana da Silva Filipe, Kathy Smollett, Stephen Carmichael, Natasha Johnson, Daniel Mair, Lily Tong, Jenna Nichols; Sarah McDonald; Richard Orton, Joseph Hughes, Sreenu Vattipally, David L Robertson; Kathy Li, Natasha Jesudason, Rajiv Shah, James Shepherd, Antonia Ho, Emma Thomson; Alasdair MacLean, Rory Gunson.
EPI_ISL_425803 hCoV-19/Scotland/CVR72/2020 Europe / United Kingdom / Scotland 2020-03-04 West of Scotland Specialist Virology Centre, NHSGGC / MRC-University of Glasgow Centre for Virus Research COVID-19 Genomics UK (COG-UK) Consortium Ana da Silva Filipe, Kathy Smollett, Stephen Carmichael, Natasha Johnson, Daniel Mair, Lily Tong, Jenna Nichols; Sarah McDonald; Richard Orton, Joseph Hughes, Sreenu Vattipally, David L Robertson; Kathy Li, Natasha Jesudason, Rajiv Shah, James Shepherd, Antonia Ho, Emma Thomson; Alasdair MacLean, Rory Gunson.
EPI_ISL_425804 hCoV-19/Scotland/CVR74/2020 Europe / United Kingdom / Scotland 2020-03-07 West of Scotland Specialist Virology Centre, NHSGGC / MRC-University of Glasgow Centre for Virus Research COVID-19 Genomics UK (COG-UK) Consortium Ana da Silva Filipe, Kathy Smollett, Stephen Carmichael, Natasha Johnson, Daniel Mair, Lily Tong, Jenna Nichols; Sarah McDonald; Richard Orton, Joseph Hughes, Sreenu Vattipally, David L Robertson; Kathy Li, Natasha Jesudason, Rajiv Shah, James Shepherd, Antonia Ho, Emma Thomson; Alasdair MacLean, Rory Gunson.
EPI_ISL_425805 hCoV-19/Scotland/CVR75/2020 Europe / United Kingdom / Scotland 2020-03-06 West of Scotland Specialist Virology Centre, NHSGGC / MRC-University of Glasgow Centre for Virus Research COVID-19 Genomics UK (COG-UK) Consortium Ana da Silva Filipe, Kathy Smollett, Stephen Carmichael, Natasha Johnson, Daniel Mair, Lily Tong, Jenna Nichols; Sarah McDonald; Richard Orton, Joseph Hughes, Sreenu Vattipally, David L Robertson; Kathy Li, Natasha Jesudason, Rajiv Shah, James Shepherd, Antonia Ho, Emma Thomson; Alasdair MacLean, Rory Gunson.
EPI_ISL_425806 hCoV-19/Scotland/CVR76/2020 Europe / United Kingdom / Scotland 2020-03-05 West of Scotland Specialist Virology Centre, NHSGGC / MRC-University of Glasgow Centre for Virus Research COVID-

19 Genomics UK (COG-UK) Consortium Ana da Silva Filipe, Kathy Smollett, Stephen Carmichael, Natasha Johnson, Daniel Mair, Lily Tong, Jenna Nichols; Sarah McDonald; Richard Orton, Joseph Hughes, Sreenu Vattipally, David L Robertson; Kathy Li, Natasha Jesudason, Rajiv Shah, James Shepherd, Antonia Ho, Emma Thomson; Alasdair MacLean, Rory Gunson.
EPI_ISL_425807 hCoV-19/Scotland/CVR77/2020 Europe / United Kingdom / Scotland 2020-03-03 West of Scotland Specialist Virology Centre, NHSGGC / MRC-University of Glasgow Centre for Virus Research COVID-19 Genomics UK (COG-UK) Consortium Ana da Silva Filipe, Kathy Smollett, Stephen Carmichael, Natasha Johnson, Daniel Mair, Lily Tong, Jenna Nichols; Sarah McDonald; Richard Orton, Joseph Hughes, Sreenu Vattipally, David L Robertson; Kathy Li, Natasha Jesudason, Rajiv Shah, James Shepherd, Antonia Ho, Emma Thomson; Alasdair MacLean, Rory Gunson.
EPI_ISL_425808 hCoV-19/Scotland/CVR78/2020 Europe / United Kingdom / Scotland 2020-03-06 West of Scotland Specialist Virology Centre, NHSGGC / MRC-University of Glasgow Centre for Virus Research COVID-19 Genomics UK (COG-UK) Consortium Ana da Silva Filipe, Kathy Smollett, Stephen Carmichael, Natasha Johnson, Daniel Mair, Lily Tong, Jenna Nichols; Sarah McDonald; Richard Orton, Joseph Hughes, Sreenu Vattipally, David L Robertson; Kathy Li, Natasha Jesudason, Rajiv Shah, James Shepherd, Antonia Ho, Emma Thomson; Alasdair MacLean, Rory Gunson.
EPI_ISL_425809 hCoV-19/Scotland/CVR79/2020 Europe / United Kingdom / Scotland 2020-03-09 West of Scotland Specialist Virology Centre, NHSGGC / MRC-University of Glasgow Centre for Virus Research COVID-19 Genomics UK (COG-UK) Consortium Ana da Silva Filipe, Kathy Smollett, Stephen Carmichael, Natasha Johnson, Daniel Mair, Lily Tong, Jenna Nichols; Sarah McDonald; Richard Orton, Joseph Hughes, Sreenu Vattipally, David L Robertson; Kathy Li, Natasha Jesudason, Rajiv Shah, James Shepherd, Antonia Ho, Emma Thomson; Alasdair MacLean, Rory Gunson.
EPI_ISL_425810 hCoV-19/Scotland/CVR80/2020 Europe / United Kingdom / Scotland 2020-03-05 West of Scotland Specialist Virology Centre, NHSGGC / MRC-University of Glasgow Centre for Virus Research COVID-19 Genomics UK (COG-UK) Consortium Ana da Silva Filipe, Kathy Smollett, Stephen Carmichael, Natasha Johnson, Daniel Mair, Lily Tong, Jenna Nichols; Sarah McDonald; Richard Orton, Joseph Hughes, Sreenu Vattipally, David L Robertson; Kathy Li, Natasha Jesudason, Rajiv Shah, James Shepherd, Antonia Ho, Emma Thomson; Alasdair MacLean, Rory Gunson.
EPI_ISL_425811 hCoV-19/Scotland/CVR83/2020 Europe / United Kingdom / Scotland 2020-03-09 West of Scotland Specialist Virology Centre, NHSGGC / MRC-University of Glasgow Centre for Virus Research COVID-19 Genomics UK (COG-UK) Consortium Ana da Silva Filipe, Kathy Smollett, Stephen Carmichael, Natasha Johnson, Daniel Mair, Lily Tong, Jenna Nichols; Sarah McDonald; Richard Orton, Joseph Hughes, Sreenu Vattipally, David L Robertson; Kathy Li, Natasha Jesudason, Rajiv Shah, James Shepherd, Antonia Ho, Emma Thomson; Alasdair MacLean, Rory Gunson.
EPI_ISL_425812 hCoV-19/Scotland/CVR85/2020 Europe / United Kingdom / Scotland 2020-03-09 West of Scotland Specialist Virology Centre, NHSGGC / MRC-University of Glasgow Centre for Virus Research COVID-19 Genomics UK (COG-UK) Consortium Ana da Silva Filipe, Kathy Smollett, Stephen Carmichael, Natasha Johnson, Daniel Mair, Lily Tong, Jenna Nichols; Sarah McDonald; Richard Orton, Joseph Hughes, Sreenu Vattipally, David L Robertson; Kathy Li, Natasha Jesudason, Rajiv Shah, James Shepherd, Antonia Ho, Emma Thomson; Alasdair MacLean, Rory Gunson.
EPI_ISL_425813 hCoV-19/Scotland/CVR89/2020 Europe / United Kingdom / Scotland 2020-03-12 West of Scotland Specialist Virology Centre, NHSGGC / MRC-University of Glasgow Centre for Virus Research COVID-19 Genomics UK (COG-UK) Consortium Ana da Silva Filipe, Kathy Smollett, Stephen Carmichael, Natasha Johnson, Daniel Mair, Lily Tong, Jenna Nichols; Sarah McDonald; Richard Orton, Joseph Hughes, Sreenu Vattipally, David L Robertson; Kathy Li, Natasha Jesudason, Rajiv Shah, James Shepherd, Antonia Ho, Emma Thomson; Alasdair MacLean, Rory Gunson.
EPI_ISL_425815 hCoV-19/Scotland/CVR91/2020 Europe / United Kingdom / Scotland 2020-03-12 West of Scotland Specialist Virology Centre, NHSGGC / MRC-University of Glasgow Centre for Virus Research COVID-19 Genomics UK (COG-UK) Consortium Ana da Silva Filipe, Kathy Smollett, Stephen Carmichael, Natasha Johnson, Daniel Mair, Lily Tong, Jenna Nichols; Sarah McDonald; Richard Orton, Joseph Hughes, Sreenu Vattipally, David L Robertson; Kathy Li, Natasha Jesudason, Rajiv Shah, James Shepherd, Antonia Ho, Emma Thomson; Alasdair MacLean, Rory Gunson.
EPI_ISL_425816 hCoV-19/Scotland/CVR96/2020 Europe / United Kingdom / Scotland 2020-03-14 West of Scotland Specialist Virology Centre, NHSGGC / MRC-University of Glasgow Centre for Virus Research COVID-19 Genomics UK (COG-UK) Consortium Ana da Silva Filipe, Kathy Smollett, Stephen Carmichael, Natasha Johnson, Daniel Mair, Lily Tong, Jenna Nichols; Sarah McDonald; Richard Orton, Joseph Hughes, Sreenu Vattipally, David L Robertson; Kathy Li, Natasha Jesudason, Rajiv Shah, James Shepherd, Antonia Ho, Emma Thomson; Alasdair MacLean, Rory Gunson.
EPI_ISL_425817 hCoV-19/Scotland/CVR97/2020 Europe / United Kingdom / Scotland 2020-03-13 West of Scotland Specialist Virology Centre, NHSGGC / MRC-University of Glasgow Centre for Virus Research COVID-19 Genomics UK (COG-UK) Consortium Ana da Silva Filipe, Kathy Smollett, Stephen Carmichael, Natasha Johnson, Daniel Mair, Lily Tong, Jenna Nichols; Sarah McDonald; Richard Orton, Joseph Hughes, Sreenu Vattipally, David L Robertson; Kathy Li, Natasha Jesudason, Rajiv Shah, James Shepherd, Antonia Ho, Emma Thomson; Alasdair MacLean, Rory Gunson.
EPI_ISL_425818 hCoV-19/Scotland/CVR99/2020 Europe / United Kingdom / Scotland 2020-03-14 West of Scotland Specialist Virology Centre, NHSGGC / MRC-University of Glasgow Centre for Virus Research COVID-19 Genomics UK (COG-UK) Consortium Ana da Silva Filipe, Kathy Smollett, Stephen Carmichael, Natasha Johnson, Daniel Mair, Lily Tong, Jenna Nichols; Sarah McDonald; Richard Orton, Joseph Hughes, Sreenu Vattipally, David L Robertson; Kathy Li, Natasha Jesudason, Rajiv Shah, James Shepherd, Antonia Ho, Emma Thomson; Alasdair MacLean, Rory Gunson.

EPI_ISL_425819 hCoV-19/Scotland/EDB002/2020   Europe / United Kingdom / Scotland   2020-03-05
Virology Department, Royal Infirmary of Edinburgh, NHS Lothian / School of Biological Sciences, University of Edinburgh / Institute of Genetics and Molecular Medicine, University of Edinburgh   COVID-19 Genomics UK (COG-UK) Consortium   McHugh M, Dewar R, Rooke S, Gallagher M, Balcaza C, O'Toole A, Hill V, McCrone JT, Colquhoun R, Yu X, Jackson B, Scher E, Rambaut A, Williams TC, Templeton K
EPI_ISL_425820 hCoV-19/Scotland/EDB014/2020   Europe / United Kingdom / Scotland   2020-03-11
Virology Department, Royal Infirmary of Edinburgh, NHS Lothian / School of Biological Sciences, University of Edinburgh / Institute of Genetics and Molecular Medicine, University of Edinburgh   COVID-19 Genomics UK (COG-UK) Consortium   McHugh M, Dewar R, Rooke S, Gallagher M, Balcaza C, O'Toole A, Hill V, McCrone JT, Colquhoun R, Yu X, Jackson B, Scher E, Rambaut A, Williams TC, Templeton K
EPI_ISL_425821 hCoV-19/Scotland/EDB015/2020   Europe / United Kingdom / Scotland   2020-03-11
Virology Department, Royal Infirmary of Edinburgh, NHS Lothian / School of Biological Sciences, University of Edinburgh / Institute of Genetics and Molecular Medicine, University of Edinburgh   COVID-19 Genomics UK (COG-UK) Consortium   McHugh M, Dewar R, Rooke S, Gallagher M, Balcaza C, O'Toole A, Hill V, McCrone JT, Colquhoun R, Yu X, Jackson B, Scher E, Rambaut A, Williams TC, Templeton K
EPI_ISL_425822 hCoV-19/Scotland/EDB016/2020   Europe / United Kingdom / Scotland   2020-03-11
Virology Department, Royal Infirmary of Edinburgh, NHS Lothian / School of Biological Sciences, University of Edinburgh / Institute of Genetics and Molecular Medicine, University of Edinburgh   COVID-19 Genomics UK (COG-UK) Consortium   McHugh M, Dewar R, Rooke S, Gallagher M, Balcaza C, O'Toole A, Hill V, McCrone JT, Colquhoun R, Yu X, Jackson B, Scher E, Rambaut A, Williams TC, Templeton K
EPI_ISL_425900 hCoV-19/Scotland/EDB113/2020   Europe / United Kingdom / Scotland   2020-03-22
Virology Department, Royal Infirmary of Edinburgh, NHS Lothian / School of Biological Sciences, University of Edinburgh / Institute of Genetics and Molecular Medicine, University of Edinburgh   COVID-19 Genomics UK (COG-UK) Consortium   McHugh M, Dewar R, Rooke S, Gallagher M, Balcaza C, O'Toole A, Hill V, McCrone JT, Colquhoun R, Yu X, Jackson B, Scher E, Rambaut A, Williams TC, Templeton K
EPI_ISL_425902 hCoV-19/Scotland/EDB118/2020   Europe / United Kingdom / Scotland   2020-03-23
Virology Department, Royal Infirmary of Edinburgh, NHS Lothian / School of Biological Sciences, University of Edinburgh / Institute of Genetics and Molecular Medicine, University of Edinburgh   COVID-19 Genomics UK (COG-UK) Consortium   McHugh M, Dewar R, Rooke S, Gallagher M, Balcaza C, O'Toole A, Hill V, McCrone JT, Colquhoun R, Yu X, Jackson B, Scher E, Rambaut A, Williams TC, Templeton K
EPI_ISL_425904 hCoV-19/Scotland/EDB120/2020   Europe / United Kingdom / Scotland   2020-03-22
Virology Department, Royal Infirmary of Edinburgh, NHS Lothian / School of Biological Sciences, University of Edinburgh / Institute of Genetics and Molecular Medicine, University of Edinburgh   COVID-19 Genomics UK (COG-UK) Consortium   McHugh M, Dewar R, Rooke S, Gallagher M, Balcaza C, O'Toole A, Hill V, McCrone JT, Colquhoun R, Yu X, Jackson B, Scher E, Rambaut A, Williams TC, Templeton K
EPI_ISL_425906 hCoV-19/Scotland/EDB124/2020   Europe / United Kingdom / Scotland   2020-03-18
Virology Department, Royal Infirmary of Edinburgh, NHS Lothian / School of Biological Sciences, University of Edinburgh / Institute of Genetics and Molecular Medicine, University of Edinburgh   COVID-19 Genomics UK (COG-UK) Consortium   McHugh M, Dewar R, Rooke S, Gallagher M, Balcaza C, O'Toole A, Hill V, McCrone JT, Colquhoun R, Yu X, Jackson B, Scher E, Rambaut A, Williams TC, Templeton K
EPI_ISL_425907 hCoV-19/Scotland/EDB125/2020   Europe / United Kingdom / Scotland   2020-03-23
Virology Department, Royal Infirmary of Edinburgh, NHS Lothian / School of Biological Sciences, University of Edinburgh / Institute of Genetics and Molecular Medicine, University of Edinburgh   COVID-19 Genomics UK (COG-UK) Consortium   McHugh M, Dewar R, Rooke S, Gallagher M, Balcaza C, O'Toole A, Hill V, McCrone JT, Colquhoun R, Yu X, Jackson B, Scher E, Rambaut A, Williams TC, Templeton K
EPI_ISL_425911 hCoV-19/Scotland/EDB132/2020   Europe / United Kingdom / Scotland   2020-03-23
Virology Department, Royal Infirmary of Edinburgh, NHS Lothian / School of Biological Sciences, University of Edinburgh / Institute of Genetics and Molecular Medicine, University of Edinburgh   COVID-19 Genomics UK (COG-UK) Consortium   McHugh M, Dewar R, Rooke S, Gallagher M, Balcaza C, O'Toole A, Hill V, McCrone JT, Colquhoun R, Yu X, Jackson B, Scher E, Rambaut A, Williams TC, Templeton K
EPI_ISL_425912 hCoV-19/Scotland/EDB142/2020   Europe / United Kingdom / Scotland   2020-03-23
Virology Department, Royal Infirmary of Edinburgh, NHS Lothian / School of Biological Sciences, University of Edinburgh / Institute of Genetics and Molecular Medicine, University of Edinburgh   COVID-19 Genomics UK (COG-UK) Consortium   McHugh M, Dewar R, Rooke S, Gallagher M, Balcaza C, O'Toole A, Hill V, McCrone JT, Colquhoun R, Yu X, Jackson B, Scher E, Rambaut A, Williams TC, Templeton K
EPI_ISL_425916 hCoV-19/Scotland/EDB148/2020   Europe / United Kingdom / Scotland   2020-03-23
Virology Department, Royal Infirmary of Edinburgh, NHS Lothian / School of Biological Sciences, University of Edinburgh / Institute of Genetics and Molecular Medicine, University of Edinburgh   COVID-19 Genomics UK (COG-UK) Consortium   McHugh M, Dewar R, Rooke S, Gallagher M, Balcaza C, O'Toole A, Hill V, McCrone JT, Colquhoun R, Yu X, Jackson B, Scher E, Rambaut A, Williams TC, Templeton K
EPI_ISL_425917 hCoV-19/Scotland/EDB149/2020   Europe / United Kingdom / Scotland   2020-03-22
Virology Department, Royal Infirmary of Edinburgh, NHS Lothian / School of Biological Sciences, University of Edinburgh / Institute of Genetics and Molecular Medicine, University of Edinburgh   COVID-19 Genomics UK (COG-UK) Consortium   McHugh M, Dewar R, Rooke S, Gallagher M, Balcaza C, O'Toole A, Hill V, McCrone JT, Colquhoun R, Yu X, Jackson B, Scher E, Rambaut A, Williams TC, Templeton K
EPI_ISL_425918 hCoV-19/Scotland/EDB150/2020   Europe / United Kingdom / Scotland   2020-03-24
Virology Department, Royal Infirmary of Edinburgh, NHS Lothian / School of Biological Sciences, University of Edinburgh / Institute of Genetics and Molecular Medicine, University of Edinburgh   COVID-19 Genomics UK (COG-UK) Consortium   McHugh M, Dewar R, Rooke S, Gallagher M, Balcaza C, O'Toole A, Hill V, McCrone JT, Colquhoun R, Yu X, Jackson B, Scher E, Rambaut A, Williams TC, Templeton K

EPI_ISL_425919 hCoV-19/Scotland/EDB151/2020   Europe / United Kingdom / Scotland   2020-03-24
Virology Department, Royal Infirmary of Edinburgh, NHS Lothian / School of Biological Sciences, University of Edinburgh / Institute of Genetics and Molecular Medicine, University of Edinburgh   COVID-19 Genomics UK (COG-UK) Consortium   McHugh M, Dewar R, Rooke S, Gallagher M, Balcaza C, O'Toole A, Hill V, McCrone JT, Colquhoun R, Yu X, Jackson B, Scher E, Rambaut A, Williams TC, Templeton K
EPI_ISL_425921 hCoV-19/Scotland/EDB156/2020   Europe / United Kingdom / Scotland   2020-03-23
Virology Department, Royal Infirmary of Edinburgh, NHS Lothian / School of Biological Sciences, University of Edinburgh / Institute of Genetics and Molecular Medicine, University of Edinburgh   COVID-19 Genomics UK (COG-UK) Consortium   McHugh M, Dewar R, Rooke S, Gallagher M, Balcaza C, O'Toole A, Hill V, McCrone JT, Colquhoun R, Yu X, Jackson B, Scher E, Rambaut A, Williams TC, Templeton K
EPI_ISL_425922 hCoV-19/Scotland/EDB157/2020   Europe / United Kingdom / Scotland   2020-03-23
Virology Department, Royal Infirmary of Edinburgh, NHS Lothian / School of Biological Sciences, University of Edinburgh / Institute of Genetics and Molecular Medicine, University of Edinburgh   COVID-19 Genomics UK (COG-UK) Consortium   McHugh M, Dewar R, Rooke S, Gallagher M, Balcaza C, O'Toole A, Hill V, McCrone JT, Colquhoun R, Yu X, Jackson B, Scher E, Rambaut A, Williams TC, Templeton K
EPI_ISL_425924 hCoV-19/Scotland/EDB162/2020   Europe / United Kingdom / Scotland   2020-03-24
Virology Department, Royal Infirmary of Edinburgh, NHS Lothian / School of Biological Sciences, University of Edinburgh / Institute of Genetics and Molecular Medicine, University of Edinburgh   COVID-19 Genomics UK (COG-UK) Consortium   McHugh M, Dewar R, Rooke S, Gallagher M, Balcaza C, O'Toole A, Hill V, McCrone JT, Colquhoun R, Yu X, Jackson B, Scher E, Rambaut A, Williams TC, Templeton K
EPI_ISL_425925 hCoV-19/Scotland/EDB168/2020   Europe / United Kingdom / Scotland   2020-03-24
Virology Department, Royal Infirmary of Edinburgh, NHS Lothian / School of Biological Sciences, University of Edinburgh / Institute of Genetics and Molecular Medicine, University of Edinburgh   COVID-19 Genomics UK (COG-UK) Consortium   McHugh M, Dewar R, Rooke S, Gallagher M, Balcaza C, O'Toole A, Hill V, McCrone JT, Colquhoun R, Yu X, Jackson B, Scher E, Rambaut A, Williams TC, Templeton K
EPI_ISL_425927 hCoV-19/Scotland/EDB181/2020   Europe / United Kingdom / Scotland   2020-03-24
Virology Department, Royal Infirmary of Edinburgh, NHS Lothian / School of Biological Sciences, University of Edinburgh / Institute of Genetics and Molecular Medicine, University of Edinburgh   COVID-19 Genomics UK (COG-UK) Consortium   McHugh M, Dewar R, Rooke S, Gallagher M, Balcaza C, O'Toole A, Hill V, McCrone JT, Colquhoun R, Yu X, Jackson B, Scher E, Rambaut A, Williams TC, Templeton K
EPI_ISL_425933 hCoV-19/Scotland/EDB189/2020   Europe / United Kingdom / Scotland   2020-03-25
Virology Department, Royal Infirmary of Edinburgh, NHS Lothian / School of Biological Sciences, University of Edinburgh / Institute of Genetics and Molecular Medicine, University of Edinburgh   COVID-19 Genomics UK (COG-UK) Consortium   McHugh M, Dewar R, Rooke S, Gallagher M, Balcaza C, O'Toole A, Hill V, McCrone JT, Colquhoun R, Yu X, Jackson B, Scher E, Rambaut A, Williams TC, Templeton K
EPI_ISL_425937 hCoV-19/Scotland/EDB194/2020   Europe / United Kingdom / Scotland   2020-03-25
Virology Department, Royal Infirmary of Edinburgh, NHS Lothian / School of Biological Sciences, University of Edinburgh / Institute of Genetics and Molecular Medicine, University of Edinburgh   COVID-19 Genomics UK (COG-UK) Consortium   McHugh M, Dewar R, Rooke S, Gallagher M, Balcaza C, O'Toole A, Hill V, McCrone JT, Colquhoun R, Yu X, Jackson B, Scher E, Rambaut A, Williams TC, Templeton K
EPI_ISL_425939 hCoV-19/Scotland/EDB198/2020   Europe / United Kingdom / Scotland   2020-03-25
Virology Department, Royal Infirmary of Edinburgh, NHS Lothian / School of Biological Sciences, University of Edinburgh / Institute of Genetics and Molecular Medicine, University of Edinburgh   COVID-19 Genomics UK (COG-UK) Consortium   McHugh M, Dewar R, Rooke S, Gallagher M, Balcaza C, O'Toole A, Hill V, McCrone JT, Colquhoun R, Yu X, Jackson B, Scher E, Rambaut A, Williams TC, Templeton K
EPI_ISL_425942 hCoV-19/Scotland/EDB204/2020   Europe / United Kingdom / Scotland   2020-03-26
Virology Department, Royal Infirmary of Edinburgh, NHS Lothian / School of Biological Sciences, University of Edinburgh / Institute of Genetics and Molecular Medicine, University of Edinburgh   COVID-19 Genomics UK (COG-UK) Consortium   McHugh M, Dewar R, Rooke S, Gallagher M, Balcaza C, O'Toole A, Hill V, McCrone JT, Colquhoun R, Yu X, Jackson B, Scher E, Rambaut A, Williams TC, Templeton K
EPI_ISL_425943 hCoV-19/Scotland/EDB212/2020   Europe / United Kingdom / Scotland   2020-03-26
Virology Department, Royal Infirmary of Edinburgh, NHS Lothian / School of Biological Sciences, University of Edinburgh / Institute of Genetics and Molecular Medicine, University of Edinburgh   COVID-19 Genomics UK (COG-UK) Consortium   McHugh M, Dewar R, Rooke S, Gallagher M, Balcaza C, O'Toole A, Hill V, McCrone JT, Colquhoun R, Yu X, Jackson B, Scher E, Rambaut A, Williams TC, Templeton K
EPI_ISL_428148 hCoV-19/Sweden/RV-FOI-4/2020   Europe / Sweden / Västerbotten 2020-03-18   Klinisk mikrobiologi, Region Västerbotten   Unit for Biological Agents, Department for CBRN Defence and Security, Swedish Defence Research Agency FOI bioinformatics team
EPI_ISL_429300 hCoV-19/Denmark/ALAB-HH46/2020 Europe / Denmark / Unknown   2020-03-13   Department of Clinical Microbiology, Copenhagen University Hospital, Hvidovre, Kettegaard Alle 30, 2650 Hvidovre.   Albertsen lab, Department of Chemistry and Bioscience, Aalborg University, Denmark   Rasmus Kirkegaard
EPI_ISL_429301 hCoV-19/Denmark/ALAB-HH49/2020 Europe / Denmark / Unknown   2020-03-16   Department of Clinical Microbiology, Copenhagen University Hospital, Hvidovre, Kettegaard Alle 30, 2650 Hvidovre.   Albertsen lab, Department of Chemistry and Bioscience, Aalborg University, Denmark   Rasmus Kirkegaard
EPI_ISL_429302 hCoV-19/Denmark/ALAB-HH50/2020 Europe / Denmark / Unknown   2020-03-16   Department of Clinical Microbiology, Copenhagen University Hospital, Hvidovre, Kettegaard Alle 30, 2650 Hvidovre.   Albertsen lab, Department of Chemistry and Bioscience, Aalborg University, Denmark   Rasmus Kirkegaard
EPI_ISL_429303 hCoV-19/Denmark/ALAB-HH51/2020 Europe / Denmark / Unknown   2020-03-16   Department of Clinical Microbiology, Copenhagen University Hospital, Hvidovre, Kettegaard Alle 30, 2650 Hvidovre.   Albertsen lab, Department of Chemistry and Bioscience, Aalborg University, Denmark   Rasmus Kirkegaard

EPI_ISL_429304 hCoV-19/Denmark/ALAB-HH56/2020 Europe / Denmark / Unknown 2020-03-17 Department of Clinical Microbiology, Copenhagen University Hospital, Hvidovre, Kettegaard Alle 30, 2650 Hvidovre. Albertsen lab, Department of Chemistry and Bioscience, Aalborg University, Denmark Rasmus Kirkegaard
EPI_ISL_429305 hCoV-19/Denmark/ALAB-HH57/2020 Europe / Denmark / Unknown 2020-03-17 Department of Clinical Microbiology, Copenhagen University Hospital, Hvidovre, Kettegaard Alle 30, 2650 Hvidovre. Albertsen lab, Department of Chemistry and Bioscience, Aalborg University, Denmark Rasmus Kirkegaard
EPI_ISL_429306 hCoV-19/Denmark/ALAB-HH60/2020 Europe / Denmark / Unknown 2020-03-17 Department of Clinical Microbiology, Copenhagen University Hospital, Hvidovre, Kettegaard Alle 30, 2650 Hvidovre. Albertsen lab, Department of Chemistry and Bioscience, Aalborg University, Denmark Rasmus Kirkegaard
EPI_ISL_429307 hCoV-19/Denmark/ALAB-HH61/2020 Europe / Denmark / Unknown 2020-03-17 Department of Clinical Microbiology, Copenhagen University Hospital, Hvidovre, Kettegaard Alle 30, 2650 Hvidovre. Albertsen lab, Department of Chemistry and Bioscience, Aalborg University, Denmark Rasmus Kirkegaard
EPI_ISL_429308 hCoV-19/Denmark/ALAB-HH62/2020 Europe / Denmark / Unknown 2020-03-17 Department of Clinical Microbiology, Copenhagen University Hospital, Hvidovre, Kettegaard Alle 30, 2650 Hvidovre. Albertsen lab, Department of Chemistry and Bioscience, Aalborg University, Denmark Rasmus Kirkegaard
EPI_ISL_429309 hCoV-19/Denmark/ALAB-HH63/2020 Europe / Denmark / Unknown 2020-03-18 Department of Clinical Microbiology, Copenhagen University Hospital, Hvidovre, Kettegaard Alle 30, 2650 Hvidovre. Albertsen lab, Department of Chemistry and Bioscience, Aalborg University, Denmark Rasmus Kirkegaard
EPI_ISL_429310 hCoV-19/Denmark/ALAB-HH64/2020 Europe / Denmark / Unknown 2020-03-17 Department of Clinical Microbiology, Copenhagen University Hospital, Hvidovre, Kettegaard Alle 30, 2650 Hvidovre. Albertsen lab, Department of Chemistry and Bioscience, Aalborg University, Denmark Rasmus Kirkegaard
EPI_ISL_429311 hCoV-19/Denmark/ALAB-HH65/2020 Europe / Denmark / Unknown 2020-03-17 Department of Clinical Microbiology, Copenhagen University Hospital, Hvidovre, Kettegaard Alle 30, 2650 Hvidovre. Albertsen lab, Department of Chemistry and Bioscience, Aalborg University, Denmark Rasmus Kirkegaard
EPI_ISL_429312 hCoV-19/Denmark/ALAB-HH66/2020 Europe / Denmark / Unknown 2020-03-18 Department of Clinical Microbiology, Copenhagen University Hospital, Hvidovre, Kettegaard Alle 30, 2650 Hvidovre. Albertsen lab, Department of Chemistry and Bioscience, Aalborg University, Denmark Rasmus Kirkegaard
EPI_ISL_429315 hCoV-19/Denmark/ALAB-HH74/2020 Europe / Denmark / Unknown 2020-03-18 Department of Clinical Microbiology, Copenhagen University Hospital, Hvidovre, Kettegaard Alle 30, 2650 Hvidovre. Albertsen lab, Department of Chemistry and Bioscience, Aalborg University, Denmark Rasmus Kirkegaard
EPI_ISL_429316 hCoV-19/Denmark/ALAB-HH75/2020 Europe / Denmark / Unknown 2020-03-18 Department of Clinical Microbiology, Copenhagen University Hospital, Hvidovre, Kettegaard Alle 30, 2650 Hvidovre. Albertsen lab, Department of Chemistry and Bioscience, Aalborg University, Denmark Rasmus Kirkegaard
EPI_ISL_429318 hCoV-19/Denmark/ALAB-HH77/2020 Europe / Denmark / Unknown 2020-03-16 Department of Clinical Microbiology, Copenhagen University Hospital, Hvidovre, Kettegaard Alle 30, 2650 Hvidovre. Albertsen lab, Department of Chemistry and Bioscience, Aalborg University, Denmark Rasmus Kirkegaard
EPI_ISL_429321 hCoV-19/Denmark/ALAB-HH80/2020 Europe / Denmark / Unknown 2020-03-16 Department of Clinical Microbiology, Copenhagen University Hospital, Hvidovre, Kettegaard Alle 30, 2650 Hvidovre. Albertsen lab, Department of Chemistry and Bioscience, Aalborg University, Denmark Rasmus Kirkegaard
EPI_ISL_429322 hCoV-19/Denmark/ALAB-HH81/2020 Europe / Denmark / Unknown 2020-03-16 Department of Clinical Microbiology, Copenhagen University Hospital, Hvidovre, Kettegaard Alle 30, 2650 Hvidovre. Albertsen lab, Department of Chemistry and Bioscience, Aalborg University, Denmark Rasmus Kirkegaard
EPI_ISL_429323 hCoV-19/Denmark/ALAB-HH82/2020 Europe / Denmark / Unknown 2020-03-15 Department of Clinical Microbiology, Copenhagen University Hospital, Hvidovre, Kettegaard Alle 30, 2650 Hvidovre. Albertsen lab, Department of Chemistry and Bioscience, Aalborg University, Denmark Rasmus Kirkegaard
EPI_ISL_429324 hCoV-19/Denmark/ALAB-HH83/2020 Europe / Denmark / Unknown 2020-03-16 Department of Clinical Microbiology, Copenhagen University Hospital, Hvidovre, Kettegaard Alle 30, 2650 Hvidovre. Albertsen lab, Department of Chemistry and Bioscience, Aalborg University, Denmark Rasmus Kirkegaard
EPI_ISL_429325 hCoV-19/Denmark/ALAB-HH84/2020 Europe / Denmark / Unknown 2020-03-16 Department of Clinical Microbiology, Copenhagen University Hospital, Hvidovre, Kettegaard Alle 30, 2650 Hvidovre. Albertsen lab, Department of Chemistry and Bioscience, Aalborg University, Denmark Rasmus Kirkegaard
EPI_ISL_429327 hCoV-19/Denmark/ALAB-HH87/2020 Europe / Denmark / Unknown 2020-03-15 Department of Clinical Microbiology, Copenhagen University Hospital, Hvidovre, Kettegaard Alle 30, 2650 Hvidovre. Albertsen lab, Department of Chemistry and Bioscience, Aalborg University, Denmark Rasmus Kirkegaard
EPI_ISL_429328 hCoV-19/Denmark/ALAB-HH88/2020 Europe / Denmark / Unknown 2020-03-15 Department of Clinical Microbiology, Copenhagen University Hospital, Hvidovre, Kettegaard Alle 30, 2650 Hvidovre. Albertsen lab, Department of Chemistry and Bioscience, Aalborg University, Denmark Rasmus Kirkegaard
EPI_ISL_429329 hCoV-19/Denmark/ALAB-HH89/2020 Europe / Denmark / Unknown 2020-03-15 Department of Clinical Microbiology, Copenhagen University Hospital, Hvidovre, Kettegaard Alle 30, 2650 Hvidovre. Albertsen lab, Department of Chemistry and Bioscience, Aalborg University, Denmark Rasmus Kirkegaard
EPI_ISL_429331 hCoV-19/Denmark/ALAB-HH92/2020 Europe / Denmark / Unknown 2020-03-15 Department of Clinical Microbiology, Copenhagen University Hospital, Hvidovre, Kettegaard Alle 30, 2650 Hvidovre. Albertsen lab, Department of Chemistry and Bioscience, Aalborg University, Denmark Rasmus Kirkegaard
EPI_ISL_429335 hCoV-19/Denmark/ALAB-SSI103/2020 Europe / Denmark / Unknown 2020-03-02 Department of Virus and Microbiological Special Diagnostics, Statens Serum Institut, Copenhagen, Denmark, Artillerivej 5, 2300 Copenahgen S Albertsen lab, Department of Chemistry and Bioscience, Aalborg University, Denmark Rasmus Kirkegaard
EPI_ISL_429337 hCoV-19/Denmark/ALAB-SSI105/2020 Europe / Denmark / Unknown 2020-03-08 Department of Virus and Microbiological Special Diagnostics, Statens Serum Institut, Copenhagen, Denmark, Artillerivej 5, 2300 Copenahgen S Albertsen lab, Department of Chemistry and Bioscience, Aalborg

University, Denmark	Rasmus Kirkegaard
EPI_ISL_429339	hCoV-19/Denmark/ALAB-SSI107/2020	Europe / Denmark / Unknown	2020-03-07
Department of Virus and Microbiological Special Diagnostics, Statens Serum Institut, Copenhagen, Denmark, Artillerivej 5, 2300 Copenahgen S	Albertsen lab, Department of Chemistry and Bioscience, Aalborg University, Denmark	Rasmus Kirkegaard
EPI_ISL_429340	hCoV-19/Denmark/ALAB-SSI108/2020	Europe / Denmark / Unknown	2020-03-07
Department of Virus and Microbiological Special Diagnostics, Statens Serum Institut, Copenhagen, Denmark, Artillerivej 5, 2300 Copenahgen S	Albertsen lab, Department of Chemistry and Bioscience, Aalborg University, Denmark	Rasmus Kirkegaard
EPI_ISL_429341	hCoV-19/Denmark/ALAB-SSI109/2020	Europe / Denmark / Unknown	2020-03-04
Department of Virus and Microbiological Special Diagnostics, Statens Serum Institut, Copenhagen, Denmark, Artillerivej 5, 2300 Copenahgen S	Albertsen lab, Department of Chemistry and Bioscience, Aalborg University, Denmark	Rasmus Kirkegaard
EPI_ISL_429342	hCoV-19/Denmark/ALAB-SSI110/2020	Europe / Denmark / Unknown	2020-03-08
Department of Virus and Microbiological Special Diagnostics, Statens Serum Institut, Copenhagen, Denmark, Artillerivej 5, 2300 Copenahgen S	Albertsen lab, Department of Chemistry and Bioscience, Aalborg University, Denmark	Rasmus Kirkegaard
EPI_ISL_429343	hCoV-19/Denmark/ALAB-SSI111/2020	Europe / Denmark / Unknown	2020-03-09
Department of Virus and Microbiological Special Diagnostics, Statens Serum Institut, Copenhagen, Denmark, Artillerivej 5, 2300 Copenahgen S	Albertsen lab, Department of Chemistry and Bioscience, Aalborg University, Denmark	Rasmus Kirkegaard
EPI_ISL_429344	hCoV-19/Denmark/ALAB-SSI113/2020	Europe / Denmark / Unknown	2020-03-08
Department of Virus and Microbiological Special Diagnostics, Statens Serum Institut, Copenhagen, Denmark, Artillerivej 5, 2300 Copenahgen S	Albertsen lab, Department of Chemistry and Bioscience, Aalborg University, Denmark	Rasmus Kirkegaard
EPI_ISL_429345	hCoV-19/Denmark/ALAB-SSI114/2020	Europe / Denmark / Unknown	2020-03-09
Department of Virus and Microbiological Special Diagnostics, Statens Serum Institut, Copenhagen, Denmark, Artillerivej 5, 2300 Copenahgen S	Albertsen lab, Department of Chemistry and Bioscience, Aalborg University, Denmark	Rasmus Kirkegaard
EPI_ISL_429346	hCoV-19/Denmark/ALAB-SSI117/2020	Europe / Denmark / Unknown	2020-03-06
Department of Virus and Microbiological Special Diagnostics, Statens Serum Institut, Copenhagen, Denmark, Artillerivej 5, 2300 Copenahgen S	Albertsen lab, Department of Chemistry and Bioscience, Aalborg University, Denmark	Rasmus Kirkegaard
EPI_ISL_429348	hCoV-19/Denmark/ALAB-SSI119/2020	Europe / Denmark / Unknown	2020-03-08
Department of Virus and Microbiological Special Diagnostics, Statens Serum Institut, Copenhagen, Denmark, Artillerivej 5, 2300 Copenahgen S	Albertsen lab, Department of Chemistry and Bioscience, Aalborg University, Denmark	Rasmus Kirkegaard
EPI_ISL_429349	hCoV-19/Denmark/ALAB-SSI120/2020	Europe / Denmark / Unknown	2020-03-08
Department of Virus and Microbiological Special Diagnostics, Statens Serum Institut, Copenhagen, Denmark, Artillerivej 5, 2300 Copenahgen S	Albertsen lab, Department of Chemistry and Bioscience, Aalborg University, Denmark	Rasmus Kirkegaard
EPI_ISL_429353	hCoV-19/Denmark/ALAB-SSI125/2020	Europe / Denmark / Unknown	2020-03-08
Department of Virus and Microbiological Special Diagnostics, Statens Serum Institut, Copenhagen, Denmark, Artillerivej 5, 2300 Copenahgen S	Albertsen lab, Department of Chemistry and Bioscience, Aalborg University, Denmark	Rasmus Kirkegaard
EPI_ISL_429355	hCoV-19/Denmark/ALAB-SSI128/2020	Europe / Denmark / Unknown	2020-03-08
Department of Virus and Microbiological Special Diagnostics, Statens Serum Institut, Copenhagen, Denmark, Artillerivej 5, 2300 Copenahgen S	Albertsen lab, Department of Chemistry and Bioscience, Aalborg University, Denmark	Rasmus Kirkegaard
EPI_ISL_429356	hCoV-19/Denmark/ALAB-SSI129/2020	Europe / Denmark / Unknown	2020-03-08
Department of Virus and Microbiological Special Diagnostics, Statens Serum Institut, Copenhagen, Denmark, Artillerivej 5, 2300 Copenahgen S	Albertsen lab, Department of Chemistry and Bioscience, Aalborg University, Denmark	Rasmus Kirkegaard
EPI_ISL_429357	hCoV-19/Denmark/ALAB-SSI132/2020	Europe / Denmark / Unknown	2020-03-08
Department of Virus and Microbiological Special Diagnostics, Statens Serum Institut, Copenhagen, Denmark, Artillerivej 5, 2300 Copenahgen S	Albertsen lab, Department of Chemistry and Bioscience, Aalborg University, Denmark	Rasmus Kirkegaard
EPI_ISL_429358	hCoV-19/Denmark/ALAB-SSI139/2020	Europe / Denmark / Unknown	2020-03-09
Department of Virus and Microbiological Special Diagnostics, Statens Serum Institut, Copenhagen, Denmark, Artillerivej 5, 2300 Copenahgen S	Albertsen lab, Department of Chemistry and Bioscience, Aalborg University, Denmark	Rasmus Kirkegaard
EPI_ISL_429360	hCoV-19/Denmark/ALAB-SSI142/2020	Europe / Denmark / Unknown	2020-03-09
Department of Virus and Microbiological Special Diagnostics, Statens Serum Institut, Copenhagen, Denmark, Artillerivej 5, 2300 Copenahgen S	Albertsen lab, Department of Chemistry and Bioscience, Aalborg University, Denmark	Rasmus Kirkegaard
EPI_ISL_429361	hCoV-19/Denmark/ALAB-SSI143/2020	Europe / Denmark / Unknown	2020-03-09
Department of Virus and Microbiological Special Diagnostics, Statens Serum Institut, Copenhagen, Denmark, Artillerivej 5, 2300 Copenahgen S	Albertsen lab, Department of Chemistry and Bioscience, Aalborg University, Denmark	Rasmus Kirkegaard
EPI_ISL_429364	hCoV-19/Denmark/ALAB-SSI146/2020	Europe / Denmark / Unknown	2020-03-09

Department of Virus and Microbiological Special Diagnostics, Statens Serum Institut, Copenhagen, Denmark, Artillerivej 5, 2300 Copenahgen S	Albertsen lab, Department of Chemistry and Bioscience, Aalborg University, Denmark	Rasmus Kirkegaard
EPI_ISL_429368	hCoV-19/Denmark/ALAB-SSI150/2020	Europe / Denmark / Unknown	2020-03-09	Department of Virus and Microbiological Special Diagnostics, Statens Serum Institut, Copenhagen, Denmark, Artillerivej 5, 2300 Copenahgen S	Albertsen lab, Department of Chemistry and Bioscience, Aalborg University, Denmark	Rasmus Kirkegaard
EPI_ISL_429369	hCoV-19/Denmark/ALAB-SSI151/2020	Europe / Denmark / Unknown	2020-03-09	Department of Virus and Microbiological Special Diagnostics, Statens Serum Institut, Copenhagen, Denmark, Artillerivej 5, 2300 Copenahgen S	Albertsen lab, Department of Chemistry and Bioscience, Aalborg University, Denmark	Rasmus Kirkegaard
EPI_ISL_429395	hCoV-19/Denmark/ALAB-SSI194/2020	Europe / Denmark / Unknown	2020-03-09	Department of Virus and Microbiological Special Diagnostics, Statens Serum Institut, Copenhagen, Denmark, Artillerivej 5, 2300 Copenahgen S	Albertsen lab, Department of Chemistry and Bioscience, Aalborg University, Denmark	Rasmus Kirkegaard
EPI_ISL_429396	hCoV-19/Denmark/ALAB-SSI195/2020	Europe / Denmark / Unknown	2020-03-09	Department of Virus and Microbiological Special Diagnostics, Statens Serum Institut, Copenhagen, Denmark, Artillerivej 5, 2300 Copenahgen S	Albertsen lab, Department of Chemistry and Bioscience, Aalborg University, Denmark	Rasmus Kirkegaard
EPI_ISL_429399	hCoV-19/Denmark/ALAB-SSI201/2020	Europe / Denmark / Unknown	2020-03-09	Department of Virus and Microbiological Special Diagnostics, Statens Serum Institut, Copenhagen, Denmark, Artillerivej 5, 2300 Copenahgen S	Albertsen lab, Department of Chemistry and Bioscience, Aalborg University, Denmark	Rasmus Kirkegaard
EPI_ISL_429402	hCoV-19/Denmark/ALAB-SSI204/2020	Europe / Denmark / Unknown	2020-03-09	Department of Virus and Microbiological Special Diagnostics, Statens Serum Institut, Copenhagen, Denmark, Artillerivej 5, 2300 Copenahgen S	Albertsen lab, Department of Chemistry and Bioscience, Aalborg University, Denmark	Rasmus Kirkegaard
EPI_ISL_429404	hCoV-19/Denmark/ALAB-SSI207/2020	Europe / Denmark / Unknown	2020-03-10	Department of Virus and Microbiological Special Diagnostics, Statens Serum Institut, Copenhagen, Denmark, Artillerivej 5, 2300 Copenahgen S	Albertsen lab, Department of Chemistry and Bioscience, Aalborg University, Denmark	Rasmus Kirkegaard
EPI_ISL_429405	hCoV-19/Denmark/ALAB-SSI209/2020	Europe / Denmark / Unknown	2020-03-10	Department of Virus and Microbiological Special Diagnostics, Statens Serum Institut, Copenhagen, Denmark, Artillerivej 5, 2300 Copenahgen S	Albertsen lab, Department of Chemistry and Bioscience, Aalborg University, Denmark	Rasmus Kirkegaard
EPI_ISL_429406	hCoV-19/Denmark/ALAB-SSI213/2020	Europe / Denmark / Unknown	2020-03-11	Department of Virus and Microbiological Special Diagnostics, Statens Serum Institut, Copenhagen, Denmark, Artillerivej 5, 2300 Copenahgen S	Albertsen lab, Department of Chemistry and Bioscience, Aalborg University, Denmark	Rasmus Kirkegaard
EPI_ISL_429407	hCoV-19/Denmark/ALAB-SSI215/2020	Europe / Denmark / Unknown	2020-03-11	Department of Virus and Microbiological Special Diagnostics, Statens Serum Institut, Copenhagen, Denmark, Artillerivej 5, 2300 Copenahgen S	Albertsen lab, Department of Chemistry and Bioscience, Aalborg University, Denmark	Rasmus Kirkegaard
EPI_ISL_429408	hCoV-19/Denmark/ALAB-SSI216/2020	Europe / Denmark / Unknown	2020-03-10	Department of Virus and Microbiological Special Diagnostics, Statens Serum Institut, Copenhagen, Denmark, Artillerivej 5, 2300 Copenahgen S	Albertsen lab, Department of Chemistry and Bioscience, Aalborg University, Denmark	Rasmus Kirkegaard
EPI_ISL_429409	hCoV-19/Denmark/ALAB-SSI219/2020	Europe / Denmark / Unknown	2020-03-10	Department of Virus and Microbiological Special Diagnostics, Statens Serum Institut, Copenhagen, Denmark, Artillerivej 5, 2300 Copenahgen S	Albertsen lab, Department of Chemistry and Bioscience, Aalborg University, Denmark	Rasmus Kirkegaard
EPI_ISL_429410	hCoV-19/Denmark/ALAB-SSI221/2020	Europe / Denmark / Unknown	2020-03-10	Department of Virus and Microbiological Special Diagnostics, Statens Serum Institut, Copenhagen, Denmark, Artillerivej 5, 2300 Copenahgen S	Albertsen lab, Department of Chemistry and Bioscience, Aalborg University, Denmark	Rasmus Kirkegaard
EPI_ISL_429414	hCoV-19/Denmark/ALAB-SSI235/2020	Europe / Denmark / Unknown	2020-03-09	Department of Virus and Microbiological Special Diagnostics, Statens Serum Institut, Copenhagen, Denmark, Artillerivej 5, 2300 Copenahgen S	Albertsen lab, Department of Chemistry and Bioscience, Aalborg University, Denmark	Rasmus Kirkegaard
EPI_ISL_429420	hCoV-19/Denmark/ALAB-SSI258/2020	Europe / Denmark / Unknown	2020-03-10	Department of Virus and Microbiological Special Diagnostics, Statens Serum Institut, Copenhagen, Denmark, Artillerivej 5, 2300 Copenahgen S	Albertsen lab, Department of Chemistry and Bioscience, Aalborg University, Denmark	Rasmus Kirkegaard
EPI_ISL_429421	hCoV-19/Denmark/ALAB-SSI259/2020	Europe / Denmark / Unknown	2020-03-10	Department of Virus and Microbiological Special Diagnostics, Statens Serum Institut, Copenhagen, Denmark, Artillerivej 5, 2300 Copenahgen S	Albertsen lab, Department of Chemistry and Bioscience, Aalborg University, Denmark	Rasmus Kirkegaard
EPI_ISL_429424	hCoV-19/Denmark/ALAB-SSI262/2020	Europe / Denmark / Unknown	2020-03-10	Department of Virus and Microbiological Special Diagnostics, Statens Serum Institut, Copenhagen, Denmark, Artillerivej 5, 2300 Copenahgen S	Albertsen lab, Department of Chemistry and Bioscience, Aalborg

University, Denmark	Rasmus Kirkegaard
EPI_ISL_429425	hCoV-19/Denmark/ALAB-SSI263/2020	Europe / Denmark / Unknown	2020-03-10	Department of Virus and Microbiological Special Diagnostics, Statens Serum Institut, Copenhagen, Denmark, Artillerivej 5, 2300 Copenahgen S	Albertsen lab, Department of Chemistry and Bioscience, Aalborg University, Denmark	Rasmus Kirkegaard
EPI_ISL_429426	hCoV-19/Denmark/ALAB-SSI264/2020	Europe / Denmark / Unknown	2020-03-10	Department of Virus and Microbiological Special Diagnostics, Statens Serum Institut, Copenhagen, Denmark, Artillerivej 5, 2300 Copenahgen S	Albertsen lab, Department of Chemistry and Bioscience, Aalborg University, Denmark	Rasmus Kirkegaard
EPI_ISL_429427	hCoV-19/Denmark/ALAB-SSI265/2020	Europe / Denmark / Unknown	2020-03-10	Department of Virus and Microbiological Special Diagnostics, Statens Serum Institut, Copenhagen, Denmark, Artillerivej 5, 2300 Copenahgen S	Albertsen lab, Department of Chemistry and Bioscience, Aalborg University, Denmark	Rasmus Kirkegaard
EPI_ISL_429428	hCoV-19/Denmark/ALAB-SSI268/2020	Europe / Denmark / Unknown	2020-03-10	Department of Virus and Microbiological Special Diagnostics, Statens Serum Institut, Copenhagen, Denmark, Artillerivej 5, 2300 Copenahgen S	Albertsen lab, Department of Chemistry and Bioscience, Aalborg University, Denmark	Rasmus Kirkegaard
EPI_ISL_429431	hCoV-19/Denmark/ALAB-SSI271/2020	Europe / Denmark / Unknown	2020-03-10	Department of Virus and Microbiological Special Diagnostics, Statens Serum Institut, Copenhagen, Denmark, Artillerivej 5, 2300 Copenahgen S	Albertsen lab, Department of Chemistry and Bioscience, Aalborg University, Denmark	Rasmus Kirkegaard
EPI_ISL_429433	hCoV-19/Denmark/ALAB-SSI272/2020	Europe / Denmark / Unknown	2020-03-10	Department of Virus and Microbiological Special Diagnostics, Statens Serum Institut, Copenhagen, Denmark, Artillerivej 5, 2300 Copenahgen S	Albertsen lab, Department of Chemistry and Bioscience, Aalborg University, Denmark	Rasmus Kirkegaard
EPI_ISL_429434	hCoV-19/Denmark/ALAB-SSI273/2020	Europe / Denmark / Unknown	2020-03-10	Department of Virus and Microbiological Special Diagnostics, Statens Serum Institut, Copenhagen, Denmark, Artillerivej 5, 2300 Copenahgen S	Albertsen lab, Department of Chemistry and Bioscience, Aalborg University, Denmark	Rasmus Kirkegaard
EPI_ISL_429439	hCoV-19/Denmark/ALAB-SSI279/2020	Europe / Denmark / Unknown	2020-03-10	Department of Virus and Microbiological Special Diagnostics, Statens Serum Institut, Copenhagen, Denmark, Artillerivej 5, 2300 Copenahgen S	Albertsen lab, Department of Chemistry and Bioscience, Aalborg University, Denmark	Rasmus Kirkegaard
EPI_ISL_429440	hCoV-19/Denmark/ALAB-SSI280/2020	Europe / Denmark / Unknown	2020-03-10	Department of Virus and Microbiological Special Diagnostics, Statens Serum Institut, Copenhagen, Denmark, Artillerivej 5, 2300 Copenahgen S	Albertsen lab, Department of Chemistry and Bioscience, Aalborg University, Denmark	Rasmus Kirkegaard
EPI_ISL_429447	hCoV-19/Denmark/ALAB-SSI29/2020 Europe / Denmark / Unknown	2020-03-03	Department of Virus and Microbiological Special Diagnostics, Statens Serum Institut, Copenhagen, Denmark, Artillerivej 5, 2300 Copenahgen S	Albertsen lab, Department of Chemistry and Bioscience, Aalborg University, Denmark	Rasmus Kirkegaard
EPI_ISL_429448	hCoV-19/Denmark/ALAB-SSI293/2020	Europe / Denmark / Unknown	2020-03-10	Department of Virus and Microbiological Special Diagnostics, Statens Serum Institut, Copenhagen, Denmark, Artillerivej 5, 2300 Copenahgen S	Albertsen lab, Department of Chemistry and Bioscience, Aalborg University, Denmark	Rasmus Kirkegaard
EPI_ISL_429449	hCoV-19/Denmark/ALAB-SSI298/2020	Europe / Denmark / Unknown	2020-03-10	Department of Virus and Microbiological Special Diagnostics, Statens Serum Institut, Copenhagen, Denmark, Artillerivej 5, 2300 Copenahgen S	Albertsen lab, Department of Chemistry and Bioscience, Aalborg University, Denmark	Rasmus Kirkegaard
EPI_ISL_429450	hCoV-19/Denmark/ALAB-SSI299/2020	Europe / Denmark / Unknown	2020-03-10	Department of Virus and Microbiological Special Diagnostics, Statens Serum Institut, Copenhagen, Denmark, Artillerivej 5, 2300 Copenahgen S	Albertsen lab, Department of Chemistry and Bioscience, Aalborg University, Denmark	Rasmus Kirkegaard
EPI_ISL_429451	hCoV-19/Denmark/ALAB-SSI300/2020	Europe / Denmark / Unknown	2020-03-10	Department of Virus and Microbiological Special Diagnostics, Statens Serum Institut, Copenhagen, Denmark, Artillerivej 5, 2300 Copenahgen S	Albertsen lab, Department of Chemistry and Bioscience, Aalborg University, Denmark	Rasmus Kirkegaard
EPI_ISL_429452	hCoV-19/Denmark/ALAB-SSI301/2020	Europe / Denmark / Unknown	2020-03-10	Department of Virus and Microbiological Special Diagnostics, Statens Serum Institut, Copenhagen, Denmark, Artillerivej 5, 2300 Copenahgen S	Albertsen lab, Department of Chemistry and Bioscience, Aalborg University, Denmark	Rasmus Kirkegaard
EPI_ISL_429453	hCoV-19/Denmark/ALAB-SSI302/2020	Europe / Denmark / Unknown	2020-03-10	Department of Virus and Microbiological Special Diagnostics, Statens Serum Institut, Copenhagen, Denmark, Artillerivej 5, 2300 Copenahgen S	Albertsen lab, Department of Chemistry and Bioscience, Aalborg University, Denmark	Rasmus Kirkegaard
EPI_ISL_429460	hCoV-19/Denmark/ALAB-SSI346/2020	Europe / Denmark / Unknown	2020-03-23	Department of Virus and Microbiological Special Diagnostics, Statens Serum Institut, Copenhagen, Denmark, Artillerivej 5, 2300 Copenahgen S	Albertsen lab, Department of Chemistry and Bioscience, Aalborg University, Denmark	Rasmus Kirkegaard
EPI_ISL_429461	hCoV-19/Denmark/ALAB-SSI347/2020	Europe / Denmark / Unknown	2020-03-23

Department of Virus and Microbiological Special Diagnostics, Statens Serum Institut, Copenhagen, Denmark, Artillerivej 5, 2300 Copenahgen S	Albertsen lab, Department of Chemistry and Bioscience, Aalborg University, Denmark	Rasmus Kirkegaard
EPI_ISL_429463	hCoV-19/Denmark/ALAB-SSI349/2020	Europe / Denmark / Unknown	2020-03-23	Department of Virus and Microbiological Special Diagnostics, Statens Serum Institut, Copenhagen, Denmark, Artillerivej 5, 2300 Copenahgen S	Albertsen lab, Department of Chemistry and Bioscience, Aalborg University, Denmark	Rasmus Kirkegaard
EPI_ISL_429464	hCoV-19/Denmark/ALAB-SSI351/2020	Europe / Denmark / Unknown	2020-03-23	Department of Virus and Microbiological Special Diagnostics, Statens Serum Institut, Copenhagen, Denmark, Artillerivej 5, 2300 Copenahgen S	Albertsen lab, Department of Chemistry and Bioscience, Aalborg University, Denmark	Rasmus Kirkegaard
EPI_ISL_429465	hCoV-19/Denmark/ALAB-SSI352/2020	Europe / Denmark / Unknown	2020-03-23	Department of Virus and Microbiological Special Diagnostics, Statens Serum Institut, Copenhagen, Denmark, Artillerivej 5, 2300 Copenahgen S	Albertsen lab, Department of Chemistry and Bioscience, Aalborg University, Denmark	Rasmus Kirkegaard
EPI_ISL_429466	hCoV-19/Denmark/ALAB-SSI354/2020	Europe / Denmark / Unknown	2020-03-23	Department of Virus and Microbiological Special Diagnostics, Statens Serum Institut, Copenhagen, Denmark, Artillerivej 5, 2300 Copenahgen S	Albertsen lab, Department of Chemistry and Bioscience, Aalborg University, Denmark	Rasmus Kirkegaard
EPI_ISL_429468	hCoV-19/Denmark/ALAB-SSI357/2020	Europe / Denmark / Unknown	2020-03-23	Department of Virus and Microbiological Special Diagnostics, Statens Serum Institut, Copenhagen, Denmark, Artillerivej 5, 2300 Copenahgen S	Albertsen lab, Department of Chemistry and Bioscience, Aalborg University, Denmark	Rasmus Kirkegaard
EPI_ISL_429469	hCoV-19/Denmark/ALAB-SSI358/2020	Europe / Denmark / Unknown	2020-03-24	Department of Virus and Microbiological Special Diagnostics, Statens Serum Institut, Copenhagen, Denmark, Artillerivej 5, 2300 Copenahgen S	Albertsen lab, Department of Chemistry and Bioscience, Aalborg University, Denmark	Rasmus Kirkegaard
EPI_ISL_429471	hCoV-19/Denmark/ALAB-SSI361/2020	Europe / Denmark / Unknown	2020-03-23	Department of Virus and Microbiological Special Diagnostics, Statens Serum Institut, Copenhagen, Denmark, Artillerivej 5, 2300 Copenahgen S	Albertsen lab, Department of Chemistry and Bioscience, Aalborg University, Denmark	Rasmus Kirkegaard
EPI_ISL_429472	hCoV-19/Denmark/ALAB-SSI364/2020	Europe / Denmark / Unknown	2020-03-23	Department of Virus and Microbiological Special Diagnostics, Statens Serum Institut, Copenhagen, Denmark, Artillerivej 5, 2300 Copenahgen S	Albertsen lab, Department of Chemistry and Bioscience, Aalborg University, Denmark	Rasmus Kirkegaard
EPI_ISL_429473	hCoV-19/Denmark/ALAB-SSI365/2020	Europe / Denmark / Unknown	2020-03-23	Department of Virus and Microbiological Special Diagnostics, Statens Serum Institut, Copenhagen, Denmark, Artillerivej 5, 2300 Copenahgen S	Albertsen lab, Department of Chemistry and Bioscience, Aalborg University, Denmark	Rasmus Kirkegaard
EPI_ISL_429474	hCoV-19/Denmark/ALAB-SSI366/2020	Europe / Denmark / Unknown	2020-03-23	Department of Virus and Microbiological Special Diagnostics, Statens Serum Institut, Copenhagen, Denmark, Artillerivej 5, 2300 Copenahgen S	Albertsen lab, Department of Chemistry and Bioscience, Aalborg University, Denmark	Rasmus Kirkegaard
EPI_ISL_429476	hCoV-19/Denmark/ALAB-SSI370/2020	Europe / Denmark / Unknown	2020-03-23	Department of Virus and Microbiological Special Diagnostics, Statens Serum Institut, Copenhagen, Denmark, Artillerivej 5, 2300 Copenahgen S	Albertsen lab, Department of Chemistry and Bioscience, Aalborg University, Denmark	Rasmus Kirkegaard
EPI_ISL_429477	hCoV-19/Denmark/ALAB-SSI371/2020	Europe / Denmark / Unknown	2020-03-23	Department of Virus and Microbiological Special Diagnostics, Statens Serum Institut, Copenhagen, Denmark, Artillerivej 5, 2300 Copenahgen S	Albertsen lab, Department of Chemistry and Bioscience, Aalborg University, Denmark	Rasmus Kirkegaard
EPI_ISL_429478	hCoV-19/Denmark/ALAB-SSI372/2020	Europe / Denmark / Unknown	2020-03-23	Department of Virus and Microbiological Special Diagnostics, Statens Serum Institut, Copenhagen, Denmark, Artillerivej 5, 2300 Copenahgen S	Albertsen lab, Department of Chemistry and Bioscience, Aalborg University, Denmark	Rasmus Kirkegaard
EPI_ISL_429479	hCoV-19/Denmark/ALAB-SSI374/2020	Europe / Denmark / Unknown	2020-03-22	Department of Virus and Microbiological Special Diagnostics, Statens Serum Institut, Copenhagen, Denmark, Artillerivej 5, 2300 Copenahgen S	Albertsen lab, Department of Chemistry and Bioscience, Aalborg University, Denmark	Rasmus Kirkegaard
EPI_ISL_429480	hCoV-19/Denmark/ALAB-SSI375/2020	Europe / Denmark / Unknown	2020-03-22	Department of Virus and Microbiological Special Diagnostics, Statens Serum Institut, Copenhagen, Denmark, Artillerivej 5, 2300 Copenahgen S	Albertsen lab, Department of Chemistry and Bioscience, Aalborg University, Denmark	Rasmus Kirkegaard
EPI_ISL_429481	hCoV-19/Denmark/ALAB-SSI376/2020	Europe / Denmark / Unknown	2020-03-22	Department of Virus and Microbiological Special Diagnostics, Statens Serum Institut, Copenhagen, Denmark, Artillerivej 5, 2300 Copenahgen S	Albertsen lab, Department of Chemistry and Bioscience, Aalborg University, Denmark	Rasmus Kirkegaard
EPI_ISL_429482	hCoV-19/Denmark/ALAB-SSI377/2020	Europe / Denmark / Unknown	2020-03-22	Department of Virus and Microbiological Special Diagnostics, Statens Serum Institut, Copenhagen, Denmark, Artillerivej 5, 2300 Copenahgen S	Albertsen lab, Department of Chemistry and Bioscience, Aalborg

University, Denmark Rasmus Kirkegaard
EPI_ISL_429483 hCoV-19/Denmark/ALAB-SSI383/2020 Europe / Denmark / Unknown 2020-03-24 Department of Virus and Microbiological Special Diagnostics, Statens Serum Institut, Copenhagen, Denmark, Artillerivej 5, 2300 Copenahgen S Albertsen lab, Department of Chemistry and Bioscience, Aalborg University, Denmark Rasmus Kirkegaard
EPI_ISL_429484 hCoV-19/Denmark/ALAB-SSI384/2020 Europe / Denmark / Unknown 2020-03-24 Department of Virus and Microbiological Special Diagnostics, Statens Serum Institut, Copenhagen, Denmark, Artillerivej 5, 2300 Copenahgen S Albertsen lab, Department of Chemistry and Bioscience, Aalborg University, Denmark Rasmus Kirkegaard
EPI_ISL_429485 hCoV-19/Denmark/ALAB-SSI385/2020 Europe / Denmark / Unknown 2020-03-24 Department of Virus and Microbiological Special Diagnostics, Statens Serum Institut, Copenhagen, Denmark, Artillerivej 5, 2300 Copenahgen S Albertsen lab, Department of Chemistry and Bioscience, Aalborg University, Denmark Rasmus Kirkegaard
EPI_ISL_429486 hCoV-19/Denmark/ALAB-SSI390/2020 Europe / Denmark / Unknown 2020-03-24 Department of Virus and Microbiological Special Diagnostics, Statens Serum Institut, Copenhagen, Denmark, Artillerivej 5, 2300 Copenahgen S Albertsen lab, Department of Chemistry and Bioscience, Aalborg University, Denmark Rasmus Kirkegaard
EPI_ISL_429488 hCoV-19/Denmark/ALAB-SSI393/2020 Europe / Denmark / Unknown 2020-03-24 Department of Virus and Microbiological Special Diagnostics, Statens Serum Institut, Copenhagen, Denmark, Artillerivej 5, 2300 Copenahgen S Albertsen lab, Department of Chemistry and Bioscience, Aalborg University, Denmark Rasmus Kirkegaard
EPI_ISL_429489 hCoV-19/Denmark/ALAB-SSI394/2020 Europe / Denmark / Unknown 2020-03-24 Department of Virus and Microbiological Special Diagnostics, Statens Serum Institut, Copenhagen, Denmark, Artillerivej 5, 2300 Copenahgen S Albertsen lab, Department of Chemistry and Bioscience, Aalborg University, Denmark Rasmus Kirkegaard
EPI_ISL_429490 hCoV-19/Denmark/ALAB-SSI395/2020 Europe / Denmark / Unknown 2020-03-23 Department of Virus and Microbiological Special Diagnostics, Statens Serum Institut, Copenhagen, Denmark, Artillerivej 5, 2300 Copenahgen S Albertsen lab, Department of Chemistry and Bioscience, Aalborg University, Denmark Rasmus Kirkegaard
EPI_ISL_429492 hCoV-19/Denmark/ALAB-SSI399/2020 Europe / Denmark / Unknown 2020-03-23 Department of Virus and Microbiological Special Diagnostics, Statens Serum Institut, Copenhagen, Denmark, Artillerivej 5, 2300 Copenahgen S Albertsen lab, Department of Chemistry and Bioscience, Aalborg University, Denmark Rasmus Kirkegaard
EPI_ISL_429493 hCoV-19/Denmark/ALAB-SSI400/2020 Europe / Denmark / Unknown 2020-03-23 Department of Virus and Microbiological Special Diagnostics, Statens Serum Institut, Copenhagen, Denmark, Artillerivej 5, 2300 Copenahgen S Albertsen lab, Department of Chemistry and Bioscience, Aalborg University, Denmark Rasmus Kirkegaard
EPI_ISL_429495 hCoV-19/Denmark/ALAB-SSI402/2020 Europe / Denmark / Unknown 2020-03-09 Department of Virus and Microbiological Special Diagnostics, Statens Serum Institut, Copenhagen, Denmark, Artillerivej 5, 2300 Copenahgen S Albertsen lab, Department of Chemistry and Bioscience, Aalborg University, Denmark Rasmus Kirkegaard
EPI_ISL_429496 hCoV-19/Denmark/ALAB-SSI403A/2020 Europe / Denmark / Unknown 2020-03-23 Department of Virus and Microbiological Special Diagnostics, Statens Serum Institut, Copenhagen, Denmark, Artillerivej 5, 2300 Copenahgen S Albertsen lab, Department of Chemistry and Bioscience, Aalborg University, Denmark Rasmus Kirkegaard
EPI_ISL_429497 hCoV-19/Denmark/ALAB-SSI404/2020 Europe / Denmark / Unknown 2020-03-08 Department of Virus and Microbiological Special Diagnostics, Statens Serum Institut, Copenhagen, Denmark, Artillerivej 5, 2300 Copenahgen S Albertsen lab, Department of Chemistry and Bioscience, Aalborg University, Denmark Rasmus Kirkegaard
EPI_ISL_429499 hCoV-19/Denmark/ALAB-SSI405/2020 Europe / Denmark / Unknown 2020-03-08 Department of Virus and Microbiological Special Diagnostics, Statens Serum Institut, Copenhagen, Denmark, Artillerivej 5, 2300 Copenahgen S Albertsen lab, Department of Chemistry and Bioscience, Aalborg University, Denmark Rasmus Kirkegaard
EPI_ISL_430323 hCoV-19/USA/NY-NYUMC296/2020 North America / USA / New York / Queens 2020-04-10 NYU Langone Health Departments of Pathology and Medicine, New York University School of Medicine Maria Aguero-Rosenfeld, Brendan Belovarac, Margaret Black, Ludovic Boytard, John Cadley, Paolo Cotzia, John Chen, Dacia Dimartino, Xiaojun Feng, Tatyana Gindin, Emily Guzman, Adriana Heguy, Megan Hogan, Emily Huang, George Jour, Lawrence H. Lin, Raven Luther, Andrew Lytle, Christian Marier, Matthew T. Maurano, Mark J. Mulligan, Peter Meyn, Raquel Ordonez Ciriza, Iman Osman, Jared Pinnell, Vanessa Raabe, Sitharam Ramaswami, Amy Rapkiewicz, Andre M. Ribeiro-dos-Santos, Marie Samanovic-Golden, Antonio Serrano, Guomiao Shen, Matija Snuderl, Theodore Vougiouklakis, Nick Vulpescu, Gael Westby, Paul Zappile, Yutong Zhang
EPI_ISL_430324 hCoV-19/USA/NY-NYUMC297/2020 North America / USA / New York / Westchester County 2020-04-10 NYU Langone Health Departments of Pathology and Medicine, New York University School of Medicine Maria Aguero-Rosenfeld, Brendan Belovarac, Margaret Black, Ludovic Boytard, John Cadley, Paolo Cotzia, John Chen, Dacia Dimartino, Xiaojun Feng, Tatyana Gindin, Emily Guzman, Adriana Heguy, Megan Hogan, Emily Huang, George Jour, Lawrence H. Lin, Raven Luther, Andrew Lytle, Christian Marier, Matthew T. Maurano, Mark J. Mulligan, Peter Meyn, Raquel Ordonez Ciriza, Iman Osman, Jared Pinnell, Vanessa Raabe, Sitharam Ramaswami, Amy Rapkiewicz, Andre M. Ribeiro-dos-Santos, Marie Samanovic-Golden, Antonio Serrano, Guomiao Shen, Matija Snuderl, Theodore Vougiouklakis, Nick Vulpescu, Gael Westby, Paul Zappile, Yutong Zhang
EPI_ISL_430325 hCoV-19/USA/NY-NYUMC298/2020 North America / USA / New York / Brooklyn 2020-04-08

NYU Langone Health Departments of Pathology and Medicine, New York University School of Medicine Maria Aguero-Rosenfeld, Brendan Belovarac, Margaret Black, Ludovic Boytard, John Cadley, Paolo Cotzia, John Chen, Dacia Dimartino, Xiaojun Feng, Tatyana Gindin, Emily Guzman, Adriana Heguy, Megan Hogan, Emily Huang, George Jour, Lawrence H. Lin, Raven Luther, Andrew Lytle, Christian Marier, Matthew T. Maurano, Mark J. Mulligan, Peter Meyn, Raquel Ordonez Ciriza, Iman Osman, Jared Pinnell, Vanessa Raabe, Sitharam Ramaswami, Amy Rapkiewicz, Andre M. Ribeiro-dos-Santos, Marie Samanovic-Golden, Antonio Serrano, Guomiao Shen, Matija Snuderl, Theodore Vougiouklakis, Nick Vulpescu, Gael Westby, Paul Zappile, Yutong Zhang
EPI_ISL_430326 hCoV-19/USA/NY-NYUMC299/2020 North America / USA / New York / Brooklyn 2020-03-30 NYU Langone Health Departments of Pathology and Medicine, New York University School of Medicine Maria Aguero-Rosenfeld, Brendan Belovarac, Margaret Black, Ludovic Boytard, John Cadley, Paolo Cotzia, John Chen, Dacia Dimartino, Xiaojun Feng, Tatyana Gindin, Emily Guzman, Adriana Heguy, Megan Hogan, Emily Huang, George Jour, Lawrence H. Lin, Raven Luther, Andrew Lytle, Christian Marier, Matthew T. Maurano, Mark J. Mulligan, Peter Meyn, Raquel Ordonez Ciriza, Iman Osman, Jared Pinnell, Vanessa Raabe, Sitharam Ramaswami, Amy Rapkiewicz, Andre M. Ribeiro-dos-Santos, Marie Samanovic-Golden, Antonio Serrano, Guomiao Shen, Matija Snuderl, Theodore Vougiouklakis, Nick Vulpescu, Gael Westby, Paul Zappile, Yutong Zhang
EPI_ISL_430327 hCoV-19/USA/NY-NYUMC300/2020 North America / USA / New York / Manhattan 2020-03-30 NYU Langone Health Departments of Pathology and Medicine, New York University School of Medicine Maria Aguero-Rosenfeld, Brendan Belovarac, Margaret Black, Ludovic Boytard, John Cadley, Paolo Cotzia, John Chen, Dacia Dimartino, Xiaojun Feng, Tatyana Gindin, Emily Guzman, Adriana Heguy, Megan Hogan, Emily Huang, George Jour, Lawrence H. Lin, Raven Luther, Andrew Lytle, Christian Marier, Matthew T. Maurano, Mark J. Mulligan, Peter Meyn, Raquel Ordonez Ciriza, Iman Osman, Jared Pinnell, Vanessa Raabe, Sitharam Ramaswami, Amy Rapkiewicz, Andre M. Ribeiro-dos-Santos, Marie Samanovic-Golden, Antonio Serrano, Guomiao Shen, Matija Snuderl, Theodore Vougiouklakis, Nick Vulpescu, Gael Westby, Paul Zappile, Yutong Zhang
EPI_ISL_430328 hCoV-19/USA/NY-NYUMC301/2020 North America / USA / New York / Brooklyn 2020-03-30 NYU Langone Health Departments of Pathology and Medicine, New York University School of Medicine Maria Aguero-Rosenfeld, Brendan Belovarac, Margaret Black, Ludovic Boytard, John Cadley, Paolo Cotzia, John Chen, Dacia Dimartino, Xiaojun Feng, Tatyana Gindin, Emily Guzman, Adriana Heguy, Megan Hogan, Emily Huang, George Jour, Lawrence H. Lin, Raven Luther, Andrew Lytle, Christian Marier, Matthew T. Maurano, Mark J. Mulligan, Peter Meyn, Raquel Ordonez Ciriza, Iman Osman, Jared Pinnell, Vanessa Raabe, Sitharam Ramaswami, Amy Rapkiewicz, Andre M. Ribeiro-dos-Santos, Marie Samanovic-Golden, Antonio Serrano, Guomiao Shen, Matija Snuderl, Theodore Vougiouklakis, Nick Vulpescu, Gael Westby, Paul Zappile, Yutong Zhang
EPI_ISL_430329 hCoV-19/USA/NY-NYUMC302/2020 North America / USA / New York / Staten Island 2020-03-30 NYU Langone Health Departments of Pathology and Medicine, New York University School of Medicine Maria Aguero-Rosenfeld, Brendan Belovarac, Margaret Black, Ludovic Boytard, John Cadley, Paolo Cotzia, John Chen, Dacia Dimartino, Xiaojun Feng, Tatyana Gindin, Emily Guzman, Adriana Heguy, Megan Hogan, Emily Huang, George Jour, Lawrence H. Lin, Raven Luther, Andrew Lytle, Christian Marier, Matthew T. Maurano, Mark J. Mulligan, Peter Meyn, Raquel Ordonez Ciriza, Iman Osman, Jared Pinnell, Vanessa Raabe, Sitharam Ramaswami, Amy Rapkiewicz, Andre M. Ribeiro-dos-Santos, Marie Samanovic-Golden, Antonio Serrano, Guomiao Shen, Matija Snuderl, Theodore Vougiouklakis, Nick Vulpescu, Gael Westby, Paul Zappile, Yutong Zhang
EPI_ISL_430330 hCoV-19/USA/NY-NYUMC303/2020 North America / USA / New York / Nassau County 2020-03-30 NYU Langone Health Departments of Pathology and Medicine, New York University School of Medicine Maria Aguero-Rosenfeld, Brendan Belovarac, Margaret Black, Ludovic Boytard, John Cadley, Paolo Cotzia, John Chen, Dacia Dimartino, Xiaojun Feng, Tatyana Gindin, Emily Guzman, Adriana Heguy, Megan Hogan, Emily Huang, George Jour, Lawrence H. Lin, Raven Luther, Andrew Lytle, Christian Marier, Matthew T. Maurano, Mark J. Mulligan, Peter Meyn, Raquel Ordonez Ciriza, Iman Osman, Jared Pinnell, Vanessa Raabe, Sitharam Ramaswami, Amy Rapkiewicz, Andre M. Ribeiro-dos-Santos, Marie Samanovic-Golden, Antonio Serrano, Guomiao Shen, Matija Snuderl, Theodore Vougiouklakis, Nick Vulpescu, Gael Westby, Paul Zappile, Yutong Zhang
EPI_ISL_430331 hCoV-19/USA/NY-NYUMC304/2020 North America / USA / New York / Manhattan 2020-03-30 NYU Langone Health Departments of Pathology and Medicine, New York University School of Medicine Maria Aguero-Rosenfeld, Brendan Belovarac, Margaret Black, Ludovic Boytard, John Cadley, Paolo Cotzia, John Chen, Dacia Dimartino, Xiaojun Feng, Tatyana Gindin, Emily Guzman, Adriana Heguy, Megan Hogan, Emily Huang, George Jour, Lawrence H. Lin, Raven Luther, Andrew Lytle, Christian Marier, Matthew T. Maurano, Mark J. Mulligan, Peter Meyn, Raquel Ordonez Ciriza, Iman Osman, Jared Pinnell, Vanessa Raabe, Sitharam Ramaswami, Amy Rapkiewicz, Andre M. Ribeiro-dos-Santos, Marie Samanovic-Golden, Antonio Serrano, Guomiao Shen, Matija Snuderl, Theodore Vougiouklakis, Nick Vulpescu, Gael Westby, Paul Zappile, Yutong Zhang
EPI_ISL_430332 hCoV-19/USA/NY-NYUMC305/2020 North America / USA / New York / Brooklyn 2020-03-30 NYU Langone Health Departments of Pathology and Medicine, New York University School of Medicine Maria Aguero-Rosenfeld, Brendan Belovarac, Margaret Black, Ludovic Boytard, John Cadley, Paolo Cotzia, John Chen, Dacia Dimartino, Xiaojun Feng, Tatyana Gindin, Emily Guzman, Adriana Heguy, Megan Hogan, Emily Huang, George Jour, Lawrence H. Lin, Raven Luther, Andrew Lytle, Christian Marier, Matthew T. Maurano, Mark J. Mulligan, Peter Meyn, Raquel Ordonez Ciriza, Iman Osman, Jared Pinnell, Vanessa Raabe, Sitharam Ramaswami, Amy Rapkiewicz, Andre M. Ribeiro-dos-Santos, Marie Samanovic-Golden, Antonio Serrano, Guomiao Shen, Matija Snuderl, Theodore Vougiouklakis, Nick Vulpescu, Gael Westby, Paul Zappile, Yutong Zhang
EPI_ISL_430340 hCoV-19/USA/NY-NYUMC313/2020 North America / USA / New York / Westchester County 2020-03-30 NYU Langone Health Departments of Pathology and Medicine, New York University School of Medicine Maria Aguero-Rosenfeld, Brendan Belovarac, Margaret Black, Ludovic Boytard, John Cadley, Paolo Cotzia, John Chen, Dacia Dimartino, Xiaojun Feng, Tatyana Gindin, Emily Guzman, Adriana Heguy, Megan Hogan, Emily Huang, George Jour, Lawrence H. Lin, Raven Luther, Andrew Lytle, Christian Marier, Matthew T. Maurano, Mark J. Mulligan, Peter Meyn, Raquel Ordonez Ciriza, Iman Osman, Jared Pinnell, Vanessa Raabe, Sitharam Ramaswami, Amy Rapkiewicz, Andre M. Ribeiro-dos-Santos, Marie Samanovic-Golden, Antonio Serrano, Guomiao Shen, Matija Snuderl,

Theodore Vougiouklakis, Nick Vulpescu, Gael Westby, Paul Zappile, Yutong Zhang
EPI_ISL_430341 hCoV-19/USA/NY-NYUMC314/2020 North America / USA / New York / Brooklyn 2020-03-30 NYU Langone Health Departments of Pathology and Medicine, New York University School of Medicine Maria Aguero-Rosenfeld, Brendan Belovarac, Margaret Black, Ludovic Boytard, John Cadley, Paolo Cotzia, John Chen, Dacia Dimartino, Xiaojun Feng, Tatyana Gindin, Emily Guzman, Adriana Heguy, Megan Hogan, Emily Huang, George Jour, Lawrence H. Lin, Raven Luther, Andrew Lytle, Christian Marier, Matthew T. Maurano, Mark J. Mulligan, Peter Meyn, Raquel Ordonez Ciriza, Iman Osman, Jared Pinnell, Vanessa Raabe, Sitharam Ramaswami, Amy Rapkiewicz, Andre M. Ribeiro-dos-Santos, Marie Samanovic-Golden, Antonio Serrano, Guomiao Shen, Matija Snuderl, Theodore Vougiouklakis, Nick Vulpescu, Gael Westby, Paul Zappile, Yutong Zhang
EPI_ISL_430342 hCoV-19/USA/NY-NYUMC315/2020 North America / USA / New York / Queens 2020-03-30 NYU Langone Health Departments of Pathology and Medicine, New York University School of Medicine Maria Aguero-Rosenfeld, Brendan Belovarac, Margaret Black, Ludovic Boytard, John Cadley, Paolo Cotzia, John Chen, Dacia Dimartino, Xiaojun Feng, Tatyana Gindin, Emily Guzman, Adriana Heguy, Megan Hogan, Emily Huang, George Jour, Lawrence H. Lin, Raven Luther, Andrew Lytle, Christian Marier, Matthew T. Maurano, Mark J. Mulligan, Peter Meyn, Raquel Ordonez Ciriza, Iman Osman, Jared Pinnell, Vanessa Raabe, Sitharam Ramaswami, Amy Rapkiewicz, Andre M. Ribeiro-dos-Santos, Marie Samanovic-Golden, Antonio Serrano, Guomiao Shen, Matija Snuderl, Theodore Vougiouklakis, Nick Vulpescu, Gael Westby, Paul Zappile, Yutong Zhang
EPI_ISL_430344 hCoV-19/USA/NY-NYUMC317/2020 North America / USA / New York / Brooklyn 2020-03-30 NYU Langone Health Departments of Pathology and Medicine, New York University School of Medicine Maria Aguero-Rosenfeld, Brendan Belovarac, Margaret Black, Ludovic Boytard, John Cadley, Paolo Cotzia, John Chen, Dacia Dimartino, Xiaojun Feng, Tatyana Gindin, Emily Guzman, Adriana Heguy, Megan Hogan, Emily Huang, George Jour, Lawrence H. Lin, Raven Luther, Andrew Lytle, Christian Marier, Matthew T. Maurano, Mark J. Mulligan, Peter Meyn, Raquel Ordonez Ciriza, Iman Osman, Jared Pinnell, Vanessa Raabe, Sitharam Ramaswami, Amy Rapkiewicz, Andre M. Ribeiro-dos-Santos, Marie Samanovic-Golden, Antonio Serrano, Guomiao Shen, Matija Snuderl, Theodore Vougiouklakis, Nick Vulpescu, Gael Westby, Paul Zappile, Yutong Zhang
EPI_ISL_430345 hCoV-19/USA/NY-NYUMC318/2020 North America / USA / New York / Manhattan 2020-03-30 NYU Langone Health Departments of Pathology and Medicine, New York University School of Medicine Maria Aguero-Rosenfeld, Brendan Belovarac, Margaret Black, Ludovic Boytard, John Cadley, Paolo Cotzia, John Chen, Dacia Dimartino, Xiaojun Feng, Tatyana Gindin, Emily Guzman, Adriana Heguy, Megan Hogan, Emily Huang, George Jour, Lawrence H. Lin, Raven Luther, Andrew Lytle, Christian Marier, Matthew T. Maurano, Mark J. Mulligan, Peter Meyn, Raquel Ordonez Ciriza, Iman Osman, Jared Pinnell, Vanessa Raabe, Sitharam Ramaswami, Amy Rapkiewicz, Andre M. Ribeiro-dos-Santos, Marie Samanovic-Golden, Antonio Serrano, Guomiao Shen, Matija Snuderl, Theodore Vougiouklakis, Nick Vulpescu, Gael Westby, Paul Zappile, Yutong Zhang
EPI_ISL_430346 hCoV-19/USA/NY-NYUMC319/2020 North America / USA / New York / Brooklyn 2020-03-30 NYU Langone Health Departments of Pathology and Medicine, New York University School of Medicine Maria Aguero-Rosenfeld, Brendan Belovarac, Margaret Black, Ludovic Boytard, John Cadley, Paolo Cotzia, John Chen, Dacia Dimartino, Xiaojun Feng, Tatyana Gindin, Emily Guzman, Adriana Heguy, Megan Hogan, Emily Huang, George Jour, Lawrence H. Lin, Raven Luther, Andrew Lytle, Christian Marier, Matthew T. Maurano, Mark J. Mulligan, Peter Meyn, Raquel Ordonez Ciriza, Iman Osman, Jared Pinnell, Vanessa Raabe, Sitharam Ramaswami, Amy Rapkiewicz, Andre M. Ribeiro-dos-Santos, Marie Samanovic-Golden, Antonio Serrano, Guomiao Shen, Matija Snuderl, Theodore Vougiouklakis, Nick Vulpescu, Gael Westby, Paul Zappile, Yutong Zhang
EPI_ISL_430347 hCoV-19/USA/NY-NYUMC320/2020 North America / USA / New York / Manhattan 2020-03-30 NYU Langone Health Departments of Pathology and Medicine, New York University School of Medicine Maria Aguero-Rosenfeld, Brendan Belovarac, Margaret Black, Ludovic Boytard, John Cadley, Paolo Cotzia, John Chen, Dacia Dimartino, Xiaojun Feng, Tatyana Gindin, Emily Guzman, Adriana Heguy, Megan Hogan, Emily Huang, George Jour, Lawrence H. Lin, Raven Luther, Andrew Lytle, Christian Marier, Matthew T. Maurano, Mark J. Mulligan, Peter Meyn, Raquel Ordonez Ciriza, Iman Osman, Jared Pinnell, Vanessa Raabe, Sitharam Ramaswami, Amy Rapkiewicz, Andre M. Ribeiro-dos-Santos, Marie Samanovic-Golden, Antonio Serrano, Guomiao Shen, Matija Snuderl, Theodore Vougiouklakis, Nick Vulpescu, Gael Westby, Paul Zappile, Yutong Zhang
EPI_ISL_430348 hCoV-19/USA/NY-NYUMC321/2020 North America / USA / New York / Manhattan 2020-03-30 NYU Langone Health Departments of Pathology and Medicine, New York University School of Medicine Maria Aguero-Rosenfeld, Brendan Belovarac, Margaret Black, Ludovic Boytard, John Cadley, Paolo Cotzia, John Chen, Dacia Dimartino, Xiaojun Feng, Tatyana Gindin, Emily Guzman, Adriana Heguy, Megan Hogan, Emily Huang, George Jour, Lawrence H. Lin, Raven Luther, Andrew Lytle, Christian Marier, Matthew T. Maurano, Mark J. Mulligan, Peter Meyn, Raquel Ordonez Ciriza, Iman Osman, Jared Pinnell, Vanessa Raabe, Sitharam Ramaswami, Amy Rapkiewicz, Andre M. Ribeiro-dos-Santos, Marie Samanovic-Golden, Antonio Serrano, Guomiao Shen, Matija Snuderl, Theodore Vougiouklakis, Nick Vulpescu, Gael Westby, Paul Zappile, Yutong Zhang
EPI_ISL_430349 hCoV-19/USA/NY-NYUMC322/2020 North America / USA / New York / Staten Island 2020-03-30 NYU Langone Health Departments of Pathology and Medicine, New York University School of Medicine Maria Aguero-Rosenfeld, Brendan Belovarac, Margaret Black, Ludovic Boytard, John Cadley, Paolo Cotzia, John Chen, Dacia Dimartino, Xiaojun Feng, Tatyana Gindin, Emily Guzman, Adriana Heguy, Megan Hogan, Emily Huang, George Jour, Lawrence H. Lin, Raven Luther, Andrew Lytle, Christian Marier, Matthew T. Maurano, Mark J. Mulligan, Peter Meyn, Raquel Ordonez Ciriza, Iman Osman, Jared Pinnell, Vanessa Raabe, Sitharam Ramaswami, Amy Rapkiewicz, Andre M. Ribeiro-dos-Santos, Marie Samanovic-Golden, Antonio Serrano, Guomiao Shen, Matija Snuderl, Theodore Vougiouklakis, Nick Vulpescu, Gael Westby, Paul Zappile, Yutong Zhang
EPI_ISL_430350 hCoV-19/USA/NY-NYUMC323/2020 North America / USA / New York / Queens 2020-04-02 NYU Langone Health Departments of Pathology and Medicine, New York University School of Medicine Maria Aguero-Rosenfeld, Brendan Belovarac, Margaret Black, Ludovic Boytard, John Cadley, Paolo Cotzia, John Chen, Dacia Dimartino, Xiaojun Feng, Tatyana Gindin, Emily Guzman, Adriana Heguy, Megan Hogan, Emily Huang, George Jour, Lawrence H. Lin, Raven Luther, Andrew Lytle, Christian Marier, Matthew T. Maurano, Mark J. Mulligan, Peter

Meyn, Raquel Ordonez Ciriza, Iman Osman, Jared Pinnell, Vanessa Raabe, Sitharam Ramaswami, Amy Rapkiewicz, Andre M. Ribeiro-dos-Santos, Marie Samanovic-Golden, Antonio Serrano, Guomiao Shen, Matija Snuderl, Theodore Vougiouklakis, Nick Vulpescu, Gael Westby, Paul Zappile, Yutong Zhang
EPI_ISL_430351 hCoV-19/USA/NY-NYUMC324/2020 North America / USA / New York / Brooklyn 2020-03-31 NYU Langone Health Departments of Pathology and Medicine, New York University School of Medicine Maria Aguero-Rosenfeld, Brendan Belovarac, Margaret Black, Ludovic Boytard, John Cadley, Paolo Cotzia, John Chen, Dacia Dimartino, Xiaojun Feng, Tatyana Gindin, Emily Guzman, Adriana Heguy, Megan Hogan, Emily Huang, George Jour, Lawrence H. Lin, Raven Luther, Andrew Lytle, Christian Marier, Matthew T. Maurano, Mark J. Mulligan, Peter Meyn, Raquel Ordonez Ciriza, Iman Osman, Jared Pinnell, Vanessa Raabe, Sitharam Ramaswami, Amy Rapkiewicz, Andre M. Ribeiro-dos-Santos, Marie Samanovic-Golden, Antonio Serrano, Guomiao Shen, Matija Snuderl, Theodore Vougiouklakis, Nick Vulpescu, Gael Westby, Paul Zappile, Yutong Zhang
EPI_ISL_430352 hCoV-19/USA/NY-NYUMC325/2020 North America / USA / New York / Suffolk County 2020-04-01 NYU Langone Health Departments of Pathology and Medicine, New York University School of Medicine Maria Aguero-Rosenfeld, Brendan Belovarac, Margaret Black, Ludovic Boytard, John Cadley, Paolo Cotzia, John Chen, Dacia Dimartino, Xiaojun Feng, Tatyana Gindin, Emily Guzman, Adriana Heguy, Megan Hogan, Emily Huang, George Jour, Lawrence H. Lin, Raven Luther, Andrew Lytle, Christian Marier, Matthew T. Maurano, Mark J. Mulligan, Peter Meyn, Raquel Ordonez Ciriza, Iman Osman, Jared Pinnell, Vanessa Raabe, Sitharam Ramaswami, Amy Rapkiewicz, Andre M. Ribeiro-dos-Santos, Marie Samanovic-Golden, Antonio Serrano, Guomiao Shen, Matija Snuderl, Theodore Vougiouklakis, Nick Vulpescu, Gael Westby, Paul Zappile, Yutong Zhang
EPI_ISL_430353 hCoV-19/USA/NY-NYUMC326/2020 North America / USA / New York / Brooklyn 2020-04-01 NYU Langone Health Departments of Pathology and Medicine, New York University School of Medicine Maria Aguero-Rosenfeld, Brendan Belovarac, Margaret Black, Ludovic Boytard, John Cadley, Paolo Cotzia, John Chen, Dacia Dimartino, Xiaojun Feng, Tatyana Gindin, Emily Guzman, Adriana Heguy, Megan Hogan, Emily Huang, George Jour, Lawrence H. Lin, Raven Luther, Andrew Lytle, Christian Marier, Matthew T. Maurano, Mark J. Mulligan, Peter Meyn, Raquel Ordonez Ciriza, Iman Osman, Jared Pinnell, Vanessa Raabe, Sitharam Ramaswami, Amy Rapkiewicz, Andre M. Ribeiro-dos-Santos, Marie Samanovic-Golden, Antonio Serrano, Guomiao Shen, Matija Snuderl, Theodore Vougiouklakis, Nick Vulpescu, Gael Westby, Paul Zappile, Yutong Zhang
EPI_ISL_430354 hCoV-19/USA/NY-NYUMC327/2020 North America / USA / New York / Nassau County 2020-04-03 NYU Langone Health Departments of Pathology and Medicine, New York University School of Medicine Maria Aguero-Rosenfeld, Brendan Belovarac, Margaret Black, Ludovic Boytard, John Cadley, Paolo Cotzia, John Chen, Dacia Dimartino, Xiaojun Feng, Tatyana Gindin, Emily Guzman, Adriana Heguy, Megan Hogan, Emily Huang, George Jour, Lawrence H. Lin, Raven Luther, Andrew Lytle, Christian Marier, Matthew T. Maurano, Mark J. Mulligan, Peter Meyn, Raquel Ordonez Ciriza, Iman Osman, Jared Pinnell, Vanessa Raabe, Sitharam Ramaswami, Amy Rapkiewicz, Andre M. Ribeiro-dos-Santos, Marie Samanovic-Golden, Antonio Serrano, Guomiao Shen, Matija Snuderl, Theodore Vougiouklakis, Nick Vulpescu, Gael Westby, Paul Zappile, Yutong Zhang
EPI_ISL_430377 hCoV-19/USA/NY-NYUMC350/2020 North America / USA / New York / Brooklyn 2020-04-08 NYU Langone Health Departments of Pathology and Medicine, New York University School of Medicine Maria Aguero-Rosenfeld, Brendan Belovarac, Margaret Black, Ludovic Boytard, John Cadley, Paolo Cotzia, John Chen, Dacia Dimartino, Xiaojun Feng, Tatyana Gindin, Emily Guzman, Adriana Heguy, Megan Hogan, Emily Huang, George Jour, Lawrence H. Lin, Raven Luther, Andrew Lytle, Christian Marier, Matthew T. Maurano, Mark J. Mulligan, Peter Meyn, Raquel Ordonez Ciriza, Iman Osman, Jared Pinnell, Vanessa Raabe, Sitharam Ramaswami, Amy Rapkiewicz, Andre M. Ribeiro-dos-Santos, Marie Samanovic-Golden, Antonio Serrano, Guomiao Shen, Matija Snuderl, Theodore Vougiouklakis, Nick Vulpescu, Gael Westby, Paul Zappile, Yutong Zhang
EPI_ISL_430378 hCoV-19/USA/NY-NYUMC351/2020 North America / USA / New York / Brooklyn 2020-04-07 NYU Langone Health Departments of Pathology and Medicine, New York University School of Medicine Maria Aguero-Rosenfeld, Brendan Belovarac, Margaret Black, Ludovic Boytard, John Cadley, Paolo Cotzia, John Chen, Dacia Dimartino, Xiaojun Feng, Tatyana Gindin, Emily Guzman, Adriana Heguy, Megan Hogan, Emily Huang, George Jour, Lawrence H. Lin, Raven Luther, Andrew Lytle, Christian Marier, Matthew T. Maurano, Mark J. Mulligan, Peter Meyn, Raquel Ordonez Ciriza, Iman Osman, Jared Pinnell, Vanessa Raabe, Sitharam Ramaswami, Amy Rapkiewicz, Andre M. Ribeiro-dos-Santos, Marie Samanovic-Golden, Antonio Serrano, Guomiao Shen, Matija Snuderl, Theodore Vougiouklakis, Nick Vulpescu, Gael Westby, Paul Zappile, Yutong Zhang
EPI_ISL_430379 hCoV-19/USA/NY-NYUMC352/2020 North America / USA / New York / Brooklyn 2020-04-07 NYU Langone Health Departments of Pathology and Medicine, New York University School of Medicine Maria Aguero-Rosenfeld, Brendan Belovarac, Margaret Black, Ludovic Boytard, John Cadley, Paolo Cotzia, John Chen, Dacia Dimartino, Xiaojun Feng, Tatyana Gindin, Emily Guzman, Adriana Heguy, Megan Hogan, Emily Huang, George Jour, Lawrence H. Lin, Raven Luther, Andrew Lytle, Christian Marier, Matthew T. Maurano, Mark J. Mulligan, Peter Meyn, Raquel Ordonez Ciriza, Iman Osman, Jared Pinnell, Vanessa Raabe, Sitharam Ramaswami, Amy Rapkiewicz, Andre M. Ribeiro-dos-Santos, Marie Samanovic-Golden, Antonio Serrano, Guomiao Shen, Matija Snuderl, Theodore Vougiouklakis, Nick Vulpescu, Gael Westby, Paul Zappile, Yutong Zhang
EPI_ISL_430380 hCoV-19/USA/NY-NYUMC353/2020 North America / USA / New York / Queens 2020-04-08 NYU Langone Health Departments of Pathology and Medicine, New York University School of Medicine Maria Aguero-Rosenfeld, Brendan Belovarac, Margaret Black, Ludovic Boytard, John Cadley, Paolo Cotzia, John Chen, Dacia Dimartino, Xiaojun Feng, Tatyana Gindin, Emily Guzman, Adriana Heguy, Megan Hogan, Emily Huang, George Jour, Lawrence H. Lin, Raven Luther, Andrew Lytle, Christian Marier, Matthew T. Maurano, Mark J. Mulligan, Peter Meyn, Raquel Ordonez Ciriza, Iman Osman, Jared Pinnell, Vanessa Raabe, Sitharam Ramaswami, Amy Rapkiewicz, Andre M. Ribeiro-dos-Santos, Marie Samanovic-Golden, Antonio Serrano, Guomiao Shen, Matija Snuderl, Theodore Vougiouklakis, Nick Vulpescu, Gael Westby, Paul Zappile, Yutong Zhang
EPI_ISL_430381 hCoV-19/USA/NY-NYUMC354/2020 North America / USA / New York / Brooklyn 2020-04-07 NYU Langone Health Departments of Pathology and Medicine, New York University School of Medicine Maria Aguero-Rosenfeld, Brendan Belovarac, Margaret Black, Ludovic Boytard, John Cadley, Paolo Cotzia, John Chen,

Dacia Dimartino, Xiaojun Feng, Tatyana Gindin, Emily Guzman, Adriana Heguy, Megan Hogan, Emily Huang, George Jour, Lawrence H. Lin, Raven Luther, Andrew Lytle, Christian Marier, Matthew T. Maurano, Mark J. Mulligan, Peter Meyn, Raquel Ordonez Ciriza, Iman Osman, Jared Pinnell, Vanessa Raabe, Sitharam Ramaswami, Amy Rapkiewicz, Andre M. Ribeiro-dos-Santos, Marie Samanovic-Golden, Antonio Serrano, Guomiao Shen, Matija Snuderl, Theodore Vougiouklakis, Nick Vulpescu, Gael Westby, Paul Zappile, Yutong Zhang
EPI_ISL_430382 hCoV-19/USA/NY-NYUMC355/2020 North America / USA / New York / Brooklyn 2020-04-09 NYU Langone Health Departments of Pathology and Medicine, New York University School of Medicine Maria Aguero-Rosenfeld, Brendan Belovarac, Margaret Black, Ludovic Boytard, John Cadley, Paolo Cotzia, John Chen, Dacia Dimartino, Xiaojun Feng, Tatyana Gindin, Emily Guzman, Adriana Heguy, Megan Hogan, Emily Huang, George Jour, Lawrence H. Lin, Raven Luther, Andrew Lytle, Christian Marier, Matthew T. Maurano, Mark J. Mulligan, Peter Meyn, Raquel Ordonez Ciriza, Iman Osman, Jared Pinnell, Vanessa Raabe, Sitharam Ramaswami, Amy Rapkiewicz, Andre M. Ribeiro-dos-Santos, Marie Samanovic-Golden, Antonio Serrano, Guomiao Shen, Matija Snuderl, Theodore Vougiouklakis, Nick Vulpescu, Gael Westby, Paul Zappile, Yutong Zhang
EPI_ISL_430383 hCoV-19/USA/NY-NYUMC356/2020 North America / USA / New York / Brooklyn 2020-04-08 NYU Langone Health Departments of Pathology and Medicine, New York University School of Medicine Maria Aguero-Rosenfeld, Brendan Belovarac, Margaret Black, Ludovic Boytard, John Cadley, Paolo Cotzia, John Chen, Dacia Dimartino, Xiaojun Feng, Tatyana Gindin, Emily Guzman, Adriana Heguy, Megan Hogan, Emily Huang, George Jour, Lawrence H. Lin, Raven Luther, Andrew Lytle, Christian Marier, Matthew T. Maurano, Mark J. Mulligan, Peter Meyn, Raquel Ordonez Ciriza, Iman Osman, Jared Pinnell, Vanessa Raabe, Sitharam Ramaswami, Amy Rapkiewicz, Andre M. Ribeiro-dos-Santos, Marie Samanovic-Golden, Antonio Serrano, Guomiao Shen, Matija Snuderl, Theodore Vougiouklakis, Nick Vulpescu, Gael Westby, Paul Zappile, Yutong Zhang
EPI_ISL_430384 hCoV-19/USA/NY-NYUMC357/2020 North America / USA / New York / Brooklyn 2020-04-09 NYU Langone Health Departments of Pathology and Medicine, New York University School of Medicine Maria Aguero-Rosenfeld, Brendan Belovarac, Margaret Black, Ludovic Boytard, John Cadley, Paolo Cotzia, John Chen, Dacia Dimartino, Xiaojun Feng, Tatyana Gindin, Emily Guzman, Adriana Heguy, Megan Hogan, Emily Huang, George Jour, Lawrence H. Lin, Raven Luther, Andrew Lytle, Christian Marier, Matthew T. Maurano, Mark J. Mulligan, Peter Meyn, Raquel Ordonez Ciriza, Iman Osman, Jared Pinnell, Vanessa Raabe, Sitharam Ramaswami, Amy Rapkiewicz, Andre M. Ribeiro-dos-Santos, Marie Samanovic-Golden, Antonio Serrano, Guomiao Shen, Matija Snuderl, Theodore Vougiouklakis, Nick Vulpescu, Gael Westby, Paul Zappile, Yutong Zhang
EPI_ISL_430385 hCoV-19/USA/NY-NYUMC358/2020 North America / USA / New York / Brooklyn 2020-04-08 NYU Langone Health Departments of Pathology and Medicine, New York University School of Medicine Maria Aguero-Rosenfeld, Brendan Belovarac, Margaret Black, Ludovic Boytard, John Cadley, Paolo Cotzia, John Chen, Dacia Dimartino, Xiaojun Feng, Tatyana Gindin, Emily Guzman, Adriana Heguy, Megan Hogan, Emily Huang, George Jour, Lawrence H. Lin, Raven Luther, Andrew Lytle, Christian Marier, Matthew T. Maurano, Mark J. Mulligan, Peter Meyn, Raquel Ordonez Ciriza, Iman Osman, Jared Pinnell, Vanessa Raabe, Sitharam Ramaswami, Amy Rapkiewicz, Andre M. Ribeiro-dos-Santos, Marie Samanovic-Golden, Antonio Serrano, Guomiao Shen, Matija Snuderl, Theodore Vougiouklakis, Nick Vulpescu, Gael Westby, Paul Zappile, Yutong Zhang
EPI_ISL_430386 hCoV-19/USA/NY-NYUMC359/2020 North America / USA / New York / Brooklyn 2020-04-08 NYU Langone Health Departments of Pathology and Medicine, New York University School of Medicine Maria Aguero-Rosenfeld, Brendan Belovarac, Margaret Black, Ludovic Boytard, John Cadley, Paolo Cotzia, John Chen, Dacia Dimartino, Xiaojun Feng, Tatyana Gindin, Emily Guzman, Adriana Heguy, Megan Hogan, Emily Huang, George Jour, Lawrence H. Lin, Raven Luther, Andrew Lytle, Christian Marier, Matthew T. Maurano, Mark J. Mulligan, Peter Meyn, Raquel Ordonez Ciriza, Iman Osman, Jared Pinnell, Vanessa Raabe, Sitharam Ramaswami, Amy Rapkiewicz, Andre M. Ribeiro-dos-Santos, Marie Samanovic-Golden, Antonio Serrano, Guomiao Shen, Matija Snuderl, Theodore Vougiouklakis, Nick Vulpescu, Gael Westby, Paul Zappile, Yutong Zhang
EPI_ISL_430387 hCoV-19/USA/NY-NYUMC360/2020 North America / USA / New York / Brooklyn 2020-04-08 NYU Langone Health Departments of Pathology and Medicine, New York University School of Medicine Maria Aguero-Rosenfeld, Brendan Belovarac, Margaret Black, Ludovic Boytard, John Cadley, Paolo Cotzia, John Chen, Dacia Dimartino, Xiaojun Feng, Tatyana Gindin, Emily Guzman, Adriana Heguy, Megan Hogan, Emily Huang, George Jour, Lawrence H. Lin, Raven Luther, Andrew Lytle, Christian Marier, Matthew T. Maurano, Mark J. Mulligan, Peter Meyn, Raquel Ordonez Ciriza, Iman Osman, Jared Pinnell, Vanessa Raabe, Sitharam Ramaswami, Amy Rapkiewicz, Andre M. Ribeiro-dos-Santos, Marie Samanovic-Golden, Antonio Serrano, Guomiao Shen, Matija Snuderl, Theodore Vougiouklakis, Nick Vulpescu, Gael Westby, Paul Zappile, Yutong Zhang
EPI_ISL_430388 hCoV-19/USA/NY-NYUMC361/2020 North America / USA / New York / Queens 2020-04-09 NYU Langone Health Departments of Pathology and Medicine, New York University School of Medicine Maria Aguero-Rosenfeld, Brendan Belovarac, Margaret Black, Ludovic Boytard, John Cadley, Paolo Cotzia, John Chen, Dacia Dimartino, Xiaojun Feng, Tatyana Gindin, Emily Guzman, Adriana Heguy, Megan Hogan, Emily Huang, George Jour, Lawrence H. Lin, Raven Luther, Andrew Lytle, Christian Marier, Matthew T. Maurano, Mark J. Mulligan, Peter Meyn, Raquel Ordonez Ciriza, Iman Osman, Jared Pinnell, Vanessa Raabe, Sitharam Ramaswami, Amy Rapkiewicz, Andre M. Ribeiro-dos-Santos, Marie Samanovic-Golden, Antonio Serrano, Guomiao Shen, Matija Snuderl, Theodore Vougiouklakis, Nick Vulpescu, Gael Westby, Paul Zappile, Yutong Zhang
EPI_ISL_430389 hCoV-19/USA/NY-NYUMC362/2020 North America / USA / New York / Brooklyn 2020-04-08 NYU Langone Health Departments of Pathology and Medicine, New York University School of Medicine Maria Aguero-Rosenfeld, Brendan Belovarac, Margaret Black, Ludovic Boytard, John Cadley, Paolo Cotzia, John Chen, Dacia Dimartino, Xiaojun Feng, Tatyana Gindin, Emily Guzman, Adriana Heguy, Megan Hogan, Emily Huang, George Jour, Lawrence H. Lin, Raven Luther, Andrew Lytle, Christian Marier, Matthew T. Maurano, Mark J. Mulligan, Peter Meyn, Raquel Ordonez Ciriza, Iman Osman, Jared Pinnell, Vanessa Raabe, Sitharam Ramaswami, Amy Rapkiewicz, Andre M. Ribeiro-dos-Santos, Marie Samanovic-Golden, Antonio Serrano, Guomiao Shen, Matija Snuderl, Theodore Vougiouklakis, Nick Vulpescu, Gael Westby, Paul Zappile, Yutong Zhang
EPI_ISL_430390 hCoV-19/USA/NY-NYUMC363/2020 North America / USA / New York / Brooklyn 2020-04-08

NYU Langone Health	Departments of Pathology and Medicine, New York University School of Medicine	Maria Aguero-Rosenfeld, Brendan Belovarac, Margaret Black, Ludovic Boytard, John Cadley, Paolo Cotzia, John Chen, Dacia Dimartino, Xiaojun Feng, Tatyana Gindin, Emily Guzman, Adriana Heguy, Megan Hogan, Emily Huang, George Jour, Lawrence H. Lin, Raven Luther, Andrew Lytle, Christian Marier, Matthew T. Maurano, Mark J. Mulligan, Peter Meyn, Raquel Ordonez Ciriza, Iman Osman, Jared Pinnell, Vanessa Raabe, Sitharam Ramaswami, Amy Rapkiewicz, Andre M. Ribeiro-dos-Santos, Marie Samanovic-Golden, Antonio Serrano, Guomiao Shen, Matija Snuderl, Theodore Vougiouklakis, Nick Vulpescu, Gael Westby, Paul Zappile, Yutong Zhang
EPI_ISL_430391	hCoV-19/USA/NY-NYUMC364/2020	North America / USA / New York / Queens	2020-04-09	NYU Langone Health	Departments of Pathology and Medicine, New York University School of Medicine	Maria Aguero-Rosenfeld, Brendan Belovarac, Margaret Black, Ludovic Boytard, John Cadley, Paolo Cotzia, John Chen, Dacia Dimartino, Xiaojun Feng, Tatyana Gindin, Emily Guzman, Adriana Heguy, Megan Hogan, Emily Huang, George Jour, Lawrence H. Lin, Raven Luther, Andrew Lytle, Christian Marier, Matthew T. Maurano, Mark J. Mulligan, Peter Meyn, Raquel Ordonez Ciriza, Iman Osman, Jared Pinnell, Vanessa Raabe, Sitharam Ramaswami, Amy Rapkiewicz, Andre M. Ribeiro-dos-Santos, Marie Samanovic-Golden, Antonio Serrano, Guomiao Shen, Matija Snuderl, Theodore Vougiouklakis, Nick Vulpescu, Gael Westby, Paul Zappile, Yutong Zhang
EPI_ISL_430392	hCoV-19/USA/NY-NYUMC365/2020	North America / USA / New York / Brooklyn	2020-04-08	NYU Langone Health	Departments of Pathology and Medicine, New York University School of Medicine	Maria Aguero-Rosenfeld, Brendan Belovarac, Margaret Black, Ludovic Boytard, John Cadley, Paolo Cotzia, John Chen, Dacia Dimartino, Xiaojun Feng, Tatyana Gindin, Emily Guzman, Adriana Heguy, Megan Hogan, Emily Huang, George Jour, Lawrence H. Lin, Raven Luther, Andrew Lytle, Christian Marier, Matthew T. Maurano, Mark J. Mulligan, Peter Meyn, Raquel Ordonez Ciriza, Iman Osman, Jared Pinnell, Vanessa Raabe, Sitharam Ramaswami, Amy Rapkiewicz, Andre M. Ribeiro-dos-Santos, Marie Samanovic-Golden, Antonio Serrano, Guomiao Shen, Matija Snuderl, Theodore Vougiouklakis, Nick Vulpescu, Gael Westby, Paul Zappile, Yutong Zhang
EPI_ISL_430393	hCoV-19/USA/NY-NYUMC366/2020	North America / USA / New York / Manhattan	2020-04-09	NYU Langone Health	Departments of Pathology and Medicine, New York University School of Medicine	Maria Aguero-Rosenfeld, Brendan Belovarac, Margaret Black, Ludovic Boytard, John Cadley, Paolo Cotzia, John Chen, Dacia Dimartino, Xiaojun Feng, Tatyana Gindin, Emily Guzman, Adriana Heguy, Megan Hogan, Emily Huang, George Jour, Lawrence H. Lin, Raven Luther, Andrew Lytle, Christian Marier, Matthew T. Maurano, Mark J. Mulligan, Peter Meyn, Raquel Ordonez Ciriza, Iman Osman, Jared Pinnell, Vanessa Raabe, Sitharam Ramaswami, Amy Rapkiewicz, Andre M. Ribeiro-dos-Santos, Marie Samanovic-Golden, Antonio Serrano, Guomiao Shen, Matija Snuderl, Theodore Vougiouklakis, Nick Vulpescu, Gael Westby, Paul Zappile, Yutong Zhang
EPI_ISL_430394	hCoV-19/USA/NJ-NYUMC367/2020	North America / USA / New Jersey / Hudson County	2020-04-09	NYU Langone Health	Departments of Pathology and Medicine, New York University School of Medicine	Maria Aguero-Rosenfeld, Brendan Belovarac, Margaret Black, Ludovic Boytard, John Cadley, Paolo Cotzia, John Chen, Dacia Dimartino, Xiaojun Feng, Tatyana Gindin, Emily Guzman, Adriana Heguy, Megan Hogan, Emily Huang, George Jour, Lawrence H. Lin, Raven Luther, Andrew Lytle, Christian Marier, Matthew T. Maurano, Mark J. Mulligan, Peter Meyn, Raquel Ordonez Ciriza, Iman Osman, Jared Pinnell, Vanessa Raabe, Sitharam Ramaswami, Amy Rapkiewicz, Andre M. Ribeiro-dos-Santos, Marie Samanovic-Golden, Antonio Serrano, Guomiao Shen, Matija Snuderl, Theodore Vougiouklakis, Nick Vulpescu, Gael Westby, Paul Zappile, Yutong Zhang
EPI_ISL_430395	hCoV-19/USA/NY-NYUMC368/2020	North America / USA / New York / Brooklyn	2020-04-09	NYU Langone Health	Departments of Pathology and Medicine, New York University School of Medicine	Maria Aguero-Rosenfeld, Brendan Belovarac, Margaret Black, Ludovic Boytard, John Cadley, Paolo Cotzia, John Chen, Dacia Dimartino, Xiaojun Feng, Tatyana Gindin, Emily Guzman, Adriana Heguy, Megan Hogan, Emily Huang, George Jour, Lawrence H. Lin, Raven Luther, Andrew Lytle, Christian Marier, Matthew T. Maurano, Mark J. Mulligan, Peter Meyn, Raquel Ordonez Ciriza, Iman Osman, Jared Pinnell, Vanessa Raabe, Sitharam Ramaswami, Amy Rapkiewicz, Andre M. Ribeiro-dos-Santos, Marie Samanovic-Golden, Antonio Serrano, Guomiao Shen, Matija Snuderl, Theodore Vougiouklakis, Nick Vulpescu, Gael Westby, Paul Zappile, Yutong Zhang
EPI_ISL_430396	hCoV-19/USA/NY-NYUMC369/2020	North America / USA / New York / Nassau County	2020-04-10	NYU Langone Health	Departments of Pathology and Medicine, New York University School of Medicine	Maria Aguero-Rosenfeld, Brendan Belovarac, Margaret Black, Ludovic Boytard, John Cadley, Paolo Cotzia, John Chen, Dacia Dimartino, Xiaojun Feng, Tatyana Gindin, Emily Guzman, Adriana Heguy, Megan Hogan, Emily Huang, George Jour, Lawrence H. Lin, Raven Luther, Andrew Lytle, Christian Marier, Matthew T. Maurano, Mark J. Mulligan, Peter Meyn, Raquel Ordonez Ciriza, Iman Osman, Jared Pinnell, Vanessa Raabe, Sitharam Ramaswami, Amy Rapkiewicz, Andre M. Ribeiro-dos-Santos, Marie Samanovic-Golden, Antonio Serrano, Guomiao Shen, Matija Snuderl, Theodore Vougiouklakis, Nick Vulpescu, Gael Westby, Paul Zappile, Yutong Zhang
EPI_ISL_430397	hCoV-19/USA/NY-NYUMC370/2020	North America / USA / New York / Nassau County	2020-04-09	NYU Langone Health	Departments of Pathology and Medicine, New York University School of Medicine	Maria Aguero-Rosenfeld, Brendan Belovarac, Margaret Black, Ludovic Boytard, John Cadley, Paolo Cotzia, John Chen, Dacia Dimartino, Xiaojun Feng, Tatyana Gindin, Emily Guzman, Adriana Heguy, Megan Hogan, Emily Huang, George Jour, Lawrence H. Lin, Raven Luther, Andrew Lytle, Christian Marier, Matthew T. Maurano, Mark J. Mulligan, Peter Meyn, Raquel Ordonez Ciriza, Iman Osman, Jared Pinnell, Vanessa Raabe, Sitharam Ramaswami, Amy Rapkiewicz, Andre M. Ribeiro-dos-Santos, Marie Samanovic-Golden, Antonio Serrano, Guomiao Shen, Matija Snuderl, Theodore Vougiouklakis, Nick Vulpescu, Gael Westby, Paul Zappile, Yutong Zhang
EPI_ISL_430398	hCoV-19/USA/NY-NYUMC371/2020	North America / USA / New York / Nassau County	2020-04-10	NYU Langone Health	Departments of Pathology and Medicine, New York University School of Medicine	Maria Aguero-Rosenfeld, Brendan Belovarac, Margaret Black, Ludovic Boytard, John Cadley, Paolo Cotzia, John Chen, Dacia Dimartino, Xiaojun Feng, Tatyana Gindin, Emily Guzman, Adriana Heguy, Megan Hogan, Emily Huang, George Jour, Lawrence H. Lin, Raven Luther, Andrew Lytle, Christian Marier, Matthew T. Maurano, Mark J. Mulligan, Peter Meyn, Raquel Ordonez Ciriza, Iman Osman, Jared Pinnell, Vanessa Raabe, Sitharam Ramaswami, Amy Rapkiewicz, Andre M. Ribeiro-dos-Santos, Marie Samanovic-Golden, Antonio Serrano, Guomiao Shen, Matija Snuderl,

Theodore Vougiouklakis, Nick Vulpescu, Gael Westby, Paul Zappile, Yutong Zhang
EPI_ISL_430399 hCoV-19/USA/NY-NYUMC372/2020 North America / USA / New York / Nassau County 2020-04-10 NYU Langone Health Departments of Pathology and Medicine, New York University School of Medicine Maria Aguero-Rosenfeld, Brendan Belovarac, Margaret Black, Ludovic Boytard, John Cadley, Paolo Cotzia, John Chen, Dacia Dimartino, Xiaojun Feng, Tatyana Gindin, Emily Guzman, Adriana Heguy, Megan Hogan, Emily Huang, George Jour, Lawrence H. Lin, Raven Luther, Andrew Lytle, Christian Marier, Matthew T. Maurano, Mark J. Mulligan, Peter Meyn, Raquel Ordonez Ciriza, Iman Osman, Jared Pinnell, Vanessa Raabe, Sitharam Ramaswami, Amy Rapkiewicz, Andre M. Ribeiro-dos-Santos, Marie Samanovic-Golden, Antonio Serrano, Guomiao Shen, Matija Snuderl, Theodore Vougiouklakis, Nick Vulpescu, Gael Westby, Paul Zappile, Yutong Zhang
EPI_ISL_430400 hCoV-19/USA/NY-NYUMC373/2020 North America / USA / New York / Brooklyn 2020-04-10 NYU Langone Health Departments of Pathology and Medicine, New York University School of Medicine Maria Aguero-Rosenfeld, Brendan Belovarac, Margaret Black, Ludovic Boytard, John Cadley, Paolo Cotzia, John Chen, Dacia Dimartino, Xiaojun Feng, Tatyana Gindin, Emily Guzman, Adriana Heguy, Megan Hogan, Emily Huang, George Jour, Lawrence H. Lin, Raven Luther, Andrew Lytle, Christian Marier, Matthew T. Maurano, Mark J. Mulligan, Peter Meyn, Raquel Ordonez Ciriza, Iman Osman, Jared Pinnell, Vanessa Raabe, Sitharam Ramaswami, Amy Rapkiewicz, Andre M. Ribeiro-dos-Santos, Marie Samanovic-Golden, Antonio Serrano, Guomiao Shen, Matija Snuderl, Theodore Vougiouklakis, Nick Vulpescu, Gael Westby, Paul Zappile, Yutong Zhang
EPI_ISL_430401 hCoV-19/USA/CT-NYUMC374/2020 North America / USA / Connecticut / Fairfield County 2020-04-10 NYU Langone Health Departments of Pathology and Medicine, New York University School of Medicine Maria Aguero-Rosenfeld, Brendan Belovarac, Margaret Black, Ludovic Boytard, John Cadley, Paolo Cotzia, John Chen, Dacia Dimartino, Xiaojun Feng, Tatyana Gindin, Emily Guzman, Adriana Heguy, Megan Hogan, Emily Huang, George Jour, Lawrence H. Lin, Raven Luther, Andrew Lytle, Christian Marier, Matthew T. Maurano, Mark J. Mulligan, Peter Meyn, Raquel Ordonez Ciriza, Iman Osman, Jared Pinnell, Vanessa Raabe, Sitharam Ramaswami, Amy Rapkiewicz, Andre M. Ribeiro-dos-Santos, Marie Samanovic-Golden, Antonio Serrano, Guomiao Shen, Matija Snuderl, Theodore Vougiouklakis, Nick Vulpescu, Gael Westby, Paul Zappile, Yutong Zhang
EPI_ISL_430402 hCoV-19/USA/NY-NYUMC375/2020 North America / USA / New York / Brooklyn 2020-04-10 NYU Langone Health Departments of Pathology and Medicine, New York University School of Medicine Maria Aguero-Rosenfeld, Brendan Belovarac, Margaret Black, Ludovic Boytard, John Cadley, Paolo Cotzia, John Chen, Dacia Dimartino, Xiaojun Feng, Tatyana Gindin, Emily Guzman, Adriana Heguy, Megan Hogan, Emily Huang, George Jour, Lawrence H. Lin, Raven Luther, Andrew Lytle, Christian Marier, Matthew T. Maurano, Mark J. Mulligan, Peter Meyn, Raquel Ordonez Ciriza, Iman Osman, Jared Pinnell, Vanessa Raabe, Sitharam Ramaswami, Amy Rapkiewicz, Andre M. Ribeiro-dos-Santos, Marie Samanovic-Golden, Antonio Serrano, Guomiao Shen, Matija Snuderl, Theodore Vougiouklakis, Nick Vulpescu, Gael Westby, Paul Zappile, Yutong Zhang
EPI_ISL_430403 hCoV-19/USA/NJ-NYUMC376/2020 North America / USA / New York 2020-04-10 NYU Langone Health Departments of Pathology and Medicine, New York University School of Medicine Maria Aguero-Rosenfeld, Brendan Belovarac, Margaret Black, Ludovic Boytard, John Cadley, Paolo Cotzia, John Chen, Dacia Dimartino, Xiaojun Feng, Tatyana Gindin, Emily Guzman, Adriana Heguy, Megan Hogan, Emily Huang, George Jour, Lawrence H. Lin, Raven Luther, Andrew Lytle, Christian Marier, Matthew T. Maurano, Mark J. Mulligan, Peter Meyn, Raquel Ordonez Ciriza, Iman Osman, Jared Pinnell, Vanessa Raabe, Sitharam Ramaswami, Amy Rapkiewicz, Andre M. Ribeiro-dos-Santos, Marie Samanovic-Golden, Antonio Serrano, Guomiao Shen, Matija Snuderl, Theodore Vougiouklakis, Nick Vulpescu, Gael Westby, Paul Zappile, Yutong Zhang
EPI_ISL_430404 hCoV-19/USA/NY-NYUMC377/2020 North America / USA / New York / Brooklyn 2020-04-10 NYU Langone Health Departments of Pathology and Medicine, New York University School of Medicine Maria Aguero-Rosenfeld, Brendan Belovarac, Margaret Black, Ludovic Boytard, John Cadley, Paolo Cotzia, John Chen, Dacia Dimartino, Xiaojun Feng, Tatyana Gindin, Emily Guzman, Adriana Heguy, Megan Hogan, Emily Huang, George Jour, Lawrence H. Lin, Raven Luther, Andrew Lytle, Christian Marier, Matthew T. Maurano, Mark J. Mulligan, Peter Meyn, Raquel Ordonez Ciriza, Iman Osman, Jared Pinnell, Vanessa Raabe, Sitharam Ramaswami, Amy Rapkiewicz, Andre M. Ribeiro-dos-Santos, Marie Samanovic-Golden, Antonio Serrano, Guomiao Shen, Matija Snuderl, Theodore Vougiouklakis, Nick Vulpescu, Gael Westby, Paul Zappile, Yutong Zhang
EPI_ISL_430405 hCoV-19/USA/NJ-NYUMC378/2020 North America / USA / New Jersey / Hudson County 2020-04-10 NYU Langone Health Departments of Pathology and Medicine, New York University School of Medicine Maria Aguero-Rosenfeld, Brendan Belovarac, Margaret Black, Ludovic Boytard, John Cadley, Paolo Cotzia, John Chen, Dacia Dimartino, Xiaojun Feng, Tatyana Gindin, Emily Guzman, Adriana Heguy, Megan Hogan, Emily Huang, George Jour, Lawrence H. Lin, Raven Luther, Andrew Lytle, Christian Marier, Matthew T. Maurano, Mark J. Mulligan, Peter Meyn, Raquel Ordonez Ciriza, Iman Osman, Jared Pinnell, Vanessa Raabe, Sitharam Ramaswami, Amy Rapkiewicz, Andre M. Ribeiro-dos-Santos, Marie Samanovic-Golden, Antonio Serrano, Guomiao Shen, Matija Snuderl, Theodore Vougiouklakis, Nick Vulpescu, Gael Westby, Paul Zappile, Yutong Zhang
EPI_ISL_430406 hCoV-19/USA/NY-NYUMC379/2020 North America / USA / New York / Manhattan 2020-04-10 NYU Langone Health Departments of Pathology and Medicine, New York University School of Medicine Maria Aguero-Rosenfeld, Brendan Belovarac, Margaret Black, Ludovic Boytard, John Cadley, Paolo Cotzia, John Chen, Dacia Dimartino, Xiaojun Feng, Tatyana Gindin, Emily Guzman, Adriana Heguy, Megan Hogan, Emily Huang, George Jour, Lawrence H. Lin, Raven Luther, Andrew Lytle, Christian Marier, Matthew T. Maurano, Mark J. Mulligan, Peter Meyn, Raquel Ordonez Ciriza, Iman Osman, Jared Pinnell, Vanessa Raabe, Sitharam Ramaswami, Amy Rapkiewicz, Andre M. Ribeiro-dos-Santos, Marie Samanovic-Golden, Antonio Serrano, Guomiao Shen, Matija Snuderl, Theodore Vougiouklakis, Nick Vulpescu, Gael Westby, Paul Zappile, Yutong Zhang
EPI_ISL_430407 hCoV-19/USA/NY-NYUMC380/2020 North America / USA / New York / Brooklyn 2020-04-10 NYU Langone Health Departments of Pathology and Medicine, New York University School of Medicine Maria Aguero-Rosenfeld, Brendan Belovarac, Margaret Black, Ludovic Boytard, John Cadley, Paolo Cotzia, John Chen, Dacia Dimartino, Xiaojun Feng, Tatyana Gindin, Emily Guzman, Adriana Heguy, Megan Hogan, Emily Huang, George Jour, Lawrence H. Lin, Raven Luther, Andrew Lytle, Christian Marier, Matthew T. Maurano, Mark J. Mulligan,

Peter Meyn, Raquel Ordonez Ciriza, Iman Osman, Jared Pinnell, Vanessa Raabe, Sitharam Ramaswami, Amy Rapkiewicz, Andre M. Ribeiro-dos-Santos, Marie Samanovic-Golden, Antonio Serrano, Guomiao Shen, Matija Snuderl, Theodore Vougiouklakis, Nick Vulpescu, Gael Westby, Paul Zappile, Yutong Zhang
EPI_ISL_430408 hCoV-19/USA/NY-NYUMC381/2020 North America / USA / New York / Nassau County 2020-04-09 NYU Langone Health Departments of Pathology and Medicine, New York University School of Medicine Maria Aguero-Rosenfeld, Brendan Belovarac, Margaret Black, Ludovic Boytard, John Cadley, Paolo Cotzia, John Chen, Dacia Dimartino, Xiaojun Feng, Tatyana Gindin, Emily Guzman, Adriana Heguy, Megan Hogan, Emily Huang, George Jour, Lawrence H. Lin, Raven Luther, Andrew Lytle, Christian Marier, Matthew T. Maurano, Mark J. Mulligan, Peter Meyn, Raquel Ordonez Ciriza, Iman Osman, Jared Pinnell, Vanessa Raabe, Sitharam Ramaswami, Amy Rapkiewicz, Andre M. Ribeiro-dos-Santos, Marie Samanovic-Golden, Antonio Serrano, Guomiao Shen, Matija Snuderl, Theodore Vougiouklakis, Nick Vulpescu, Gael Westby, Paul Zappile, Yutong Zhang
EPI_ISL_430409 hCoV-19/USA/NY-NYUMC382/2020 North America / USA / New York / Brooklyn 2020-04-10 NYU Langone Health Departments of Pathology and Medicine, New York University School of Medicine Maria Aguero-Rosenfeld, Brendan Belovarac, Margaret Black, Ludovic Boytard, John Cadley, Paolo Cotzia, John Chen, Dacia Dimartino, Xiaojun Feng, Tatyana Gindin, Emily Guzman, Adriana Heguy, Megan Hogan, Emily Huang, George Jour, Lawrence H. Lin, Raven Luther, Andrew Lytle, Christian Marier, Matthew T. Maurano, Mark J. Mulligan, Peter Meyn, Raquel Ordonez Ciriza, Iman Osman, Jared Pinnell, Vanessa Raabe, Sitharam Ramaswami, Amy Rapkiewicz, Andre M. Ribeiro-dos-Santos, Marie Samanovic-Golden, Antonio Serrano, Guomiao Shen, Matija Snuderl, Theodore Vougiouklakis, Nick Vulpescu, Gael Westby, Paul Zappile, Yutong Zhang
EPI_ISL_430410 hCoV-19/USA/NY-NYUMC383/2020 North America / USA / New York / Brooklyn 2020-04-10 NYU Langone Health Departments of Pathology and Medicine, New York University School of Medicine Maria Aguero-Rosenfeld, Brendan Belovarac, Margaret Black, Ludovic Boytard, John Cadley, Paolo Cotzia, John Chen, Dacia Dimartino, Xiaojun Feng, Tatyana Gindin, Emily Guzman, Adriana Heguy, Megan Hogan, Emily Huang, George Jour, Lawrence H. Lin, Raven Luther, Andrew Lytle, Christian Marier, Matthew T. Maurano, Mark J. Mulligan, Peter Meyn, Raquel Ordonez Ciriza, Iman Osman, Jared Pinnell, Vanessa Raabe, Sitharam Ramaswami, Amy Rapkiewicz, Andre M. Ribeiro-dos-Santos, Marie Samanovic-Golden, Antonio Serrano, Guomiao Shen, Matija Snuderl, Theodore Vougiouklakis, Nick Vulpescu, Gael Westby, Paul Zappile, Yutong Zhang
EPI_ISL_430411 hCoV-19/USA/NY-NYUMC384/2020 North America / USA / New York / Brooklyn 2020-04-09 NYU Langone Health Departments of Pathology and Medicine, New York University School of Medicine Maria Aguero-Rosenfeld, Brendan Belovarac, Margaret Black, Ludovic Boytard, John Cadley, Paolo Cotzia, John Chen, Dacia Dimartino, Xiaojun Feng, Tatyana Gindin, Emily Guzman, Adriana Heguy, Megan Hogan, Emily Huang, George Jour, Lawrence H. Lin, Raven Luther, Andrew Lytle, Christian Marier, Matthew T. Maurano, Mark J. Mulligan, Peter Meyn, Raquel Ordonez Ciriza, Iman Osman, Jared Pinnell, Vanessa Raabe, Sitharam Ramaswami, Amy Rapkiewicz, Andre M. Ribeiro-dos-Santos, Marie Samanovic-Golden, Antonio Serrano, Guomiao Shen, Matija Snuderl, Theodore Vougiouklakis, Nick Vulpescu, Gael Westby, Paul Zappile, Yutong Zhang
EPI_ISL_430412 hCoV-19/USA/NY-NYUMC385/2020 North America / USA / New York / Staten Island 2020-04-10 NYU Langone Health Departments of Pathology and Medicine, New York University School of Medicine Maria Aguero-Rosenfeld, Brendan Belovarac, Margaret Black, Ludovic Boytard, John Cadley, Paolo Cotzia, John Chen, Dacia Dimartino, Xiaojun Feng, Tatyana Gindin, Emily Guzman, Adriana Heguy, Megan Hogan, Emily Huang, George Jour, Lawrence H. Lin, Raven Luther, Andrew Lytle, Christian Marier, Matthew T. Maurano, Mark J. Mulligan, Peter Meyn, Raquel Ordonez Ciriza, Iman Osman, Jared Pinnell, Vanessa Raabe, Sitharam Ramaswami, Amy Rapkiewicz, Andre M. Ribeiro-dos-Santos, Marie Samanovic-Golden, Antonio Serrano, Guomiao Shen, Matija Snuderl, Theodore Vougiouklakis, Nick Vulpescu, Gael Westby, Paul Zappile, Yutong Zhang
EPI_ISL_430413 hCoV-19/USA/NY-NYUMC386/2020 North America / USA / New York / Nassau County 2020-04-09 NYU Langone Health Departments of Pathology and Medicine, New York University School of Medicine Maria Aguero-Rosenfeld, Brendan Belovarac, Margaret Black, Ludovic Boytard, John Cadley, Paolo Cotzia, John Chen, Dacia Dimartino, Xiaojun Feng, Tatyana Gindin, Emily Guzman, Adriana Heguy, Megan Hogan, Emily Huang, George Jour, Lawrence H. Lin, Raven Luther, Andrew Lytle, Christian Marier, Matthew T. Maurano, Mark J. Mulligan, Peter Meyn, Raquel Ordonez Ciriza, Iman Osman, Jared Pinnell, Vanessa Raabe, Sitharam Ramaswami, Amy Rapkiewicz, Andre M. Ribeiro-dos-Santos, Marie Samanovic-Golden, Antonio Serrano, Guomiao Shen, Matija Snuderl, Theodore Vougiouklakis, Nick Vulpescu, Gael Westby, Paul Zappile, Yutong Zhang
EPI_ISL_430414 hCoV-19/USA/NY-NYUMC387/2020 North America / USA / New York / Rockland County 2020-04-10 NYU Langone Health Departments of Pathology and Medicine, New York University School of Medicine Maria Aguero-Rosenfeld, Brendan Belovarac, Margaret Black, Ludovic Boytard, John Cadley, Paolo Cotzia, John Chen, Dacia Dimartino, Xiaojun Feng, Tatyana Gindin, Emily Guzman, Adriana Heguy, Megan Hogan, Emily Huang, George Jour, Lawrence H. Lin, Raven Luther, Andrew Lytle, Christian Marier, Matthew T. Maurano, Mark J. Mulligan, Peter Meyn, Raquel Ordonez Ciriza, Iman Osman, Jared Pinnell, Vanessa Raabe, Sitharam Ramaswami, Amy Rapkiewicz, Andre M. Ribeiro-dos-Santos, Marie Samanovic-Golden, Antonio Serrano, Guomiao Shen, Matija Snuderl, Theodore Vougiouklakis, Nick Vulpescu, Gael Westby, Paul Zappile, Yutong Zhang
EPI_ISL_430415 hCoV-19/USA/NY-NYUMC388/2020 North America / USA / New York / Manhattan 2020-04-10 NYU Langone Health Departments of Pathology and Medicine, New York University School of Medicine Maria Aguero-Rosenfeld, Brendan Belovarac, Margaret Black, Ludovic Boytard, John Cadley, Paolo Cotzia, John Chen, Dacia Dimartino, Xiaojun Feng, Tatyana Gindin, Emily Guzman, Adriana Heguy, Megan Hogan, Emily Huang, George Jour, Lawrence H. Lin, Raven Luther, Andrew Lytle, Christian Marier, Matthew T. Maurano, Mark J. Mulligan, Peter Meyn, Raquel Ordonez Ciriza, Iman Osman, Jared Pinnell, Vanessa Raabe, Sitharam Ramaswami, Amy Rapkiewicz, Andre M. Ribeiro-dos-Santos, Marie Samanovic-Golden, Antonio Serrano, Guomiao Shen, Matija Snuderl, Theodore Vougiouklakis, Nick Vulpescu, Gael Westby, Paul Zappile, Yutong Zhang
EPI_ISL_430416 hCoV-19/USA/NY-NYUMC389/2020 North America / USA / New York / Manhattan 2020-04-13 NYU Langone Health Departments of Pathology and Medicine, New York University School of Medicine Maria Aguero-Rosenfeld, Brendan Belovarac, Margaret Black, Ludovic Boytard, John Cadley, Paolo Cotzia, John Chen,

Dacia Dimartino, Xiaojun Feng, Tatyana Gindin, Emily Guzman, Adriana Heguy, Megan Hogan, Emily Huang, George Jour, Lawrence H. Lin, Raven Luther, Andrew Lytle, Christian Marier, Matthew T. Maurano, Mark J. Mulligan, Peter Meyn, Raquel Ordonez Ciriza, Iman Osman, Jared Pinnell, Vanessa Raabe, Sitharam Ramaswami, Amy Rapkiewicz, Andre M. Ribeiro-dos-Santos, Marie Samanovic-Golden, Antonio Serrano, Guomiao Shen, Matija Snuderl, Theodore Vougiouklakis, Nick Vulpescu, Gael Westby, Paul Zappile, Yutong Zhang
EPI_ISL_430417 hCoV-19/USA/NY-NYUMC390/2020 North America / USA / New York / Nassau County 2020-04-13 NYU Langone Health Departments of Pathology and Medicine, New York University School of Medicine Maria Aguero-Rosenfeld, Brendan Belovarac, Margaret Black, Ludovic Boytard, John Cadley, Paolo Cotzia, John Chen, Dacia Dimartino, Xiaojun Feng, Tatyana Gindin, Emily Guzman, Adriana Heguy, Megan Hogan, Emily Huang, George Jour, Lawrence H. Lin, Raven Luther, Andrew Lytle, Christian Marier, Matthew T. Maurano, Mark J. Mulligan, Peter Meyn, Raquel Ordonez Ciriza, Iman Osman, Jared Pinnell, Vanessa Raabe, Sitharam Ramaswami, Amy Rapkiewicz, Andre M. Ribeiro-dos-Santos, Marie Samanovic-Golden, Antonio Serrano, Guomiao Shen, Matija Snuderl, Theodore Vougiouklakis, Nick Vulpescu, Gael Westby, Paul Zappile, Yutong Zhang
EPI_ISL_430418 hCoV-19/USA/NY-NYUMC391/2020 North America / USA / New York / Nassau County 2020-04-13 NYU Langone Health Departments of Pathology and Medicine, New York University School of Medicine Maria Aguero-Rosenfeld, Brendan Belovarac, Margaret Black, Ludovic Boytard, John Cadley, Paolo Cotzia, John Chen, Dacia Dimartino, Xiaojun Feng, Tatyana Gindin, Emily Guzman, Adriana Heguy, Megan Hogan, Emily Huang, George Jour, Lawrence H. Lin, Raven Luther, Andrew Lytle, Christian Marier, Matthew T. Maurano, Mark J. Mulligan, Peter Meyn, Raquel Ordonez Ciriza, Iman Osman, Jared Pinnell, Vanessa Raabe, Sitharam Ramaswami, Amy Rapkiewicz, Andre M. Ribeiro-dos-Santos, Marie Samanovic-Golden, Antonio Serrano, Guomiao Shen, Matija Snuderl, Theodore Vougiouklakis, Nick Vulpescu, Gael Westby, Paul Zappile, Yutong Zhang
EPI_ISL_430419 hCoV-19/USA/NY-NYUMC392/2020 North America / USA / New York / Nassau County 2020-04-13 NYU Langone Health Departments of Pathology and Medicine, New York University School of Medicine Maria Aguero-Rosenfeld, Brendan Belovarac, Margaret Black, Ludovic Boytard, John Cadley, Paolo Cotzia, John Chen, Dacia Dimartino, Xiaojun Feng, Tatyana Gindin, Emily Guzman, Adriana Heguy, Megan Hogan, Emily Huang, George Jour, Lawrence H. Lin, Raven Luther, Andrew Lytle, Christian Marier, Matthew T. Maurano, Mark J. Mulligan, Peter Meyn, Raquel Ordonez Ciriza, Iman Osman, Jared Pinnell, Vanessa Raabe, Sitharam Ramaswami, Amy Rapkiewicz, Andre M. Ribeiro-dos-Santos, Marie Samanovic-Golden, Antonio Serrano, Guomiao Shen, Matija Snuderl, Theodore Vougiouklakis, Nick Vulpescu, Gael Westby, Paul Zappile, Yutong Zhang
EPI_ISL_430420 hCoV-19/USA/NY-NYUMC393/2020 North America / USA / New York / Staten Island 2020-04-13 NYU Langone Health Departments of Pathology and Medicine, New York University School of Medicine Maria Aguero-Rosenfeld, Brendan Belovarac, Margaret Black, Ludovic Boytard, John Cadley, Paolo Cotzia, John Chen, Dacia Dimartino, Xiaojun Feng, Tatyana Gindin, Emily Guzman, Adriana Heguy, Megan Hogan, Emily Huang, George Jour, Lawrence H. Lin, Raven Luther, Andrew Lytle, Christian Marier, Matthew T. Maurano, Mark J. Mulligan, Peter Meyn, Raquel Ordonez Ciriza, Iman Osman, Jared Pinnell, Vanessa Raabe, Sitharam Ramaswami, Amy Rapkiewicz, Andre M. Ribeiro-dos-Santos, Marie Samanovic-Golden, Antonio Serrano, Guomiao Shen, Matija Snuderl, Theodore Vougiouklakis, Nick Vulpescu, Gael Westby, Paul Zappile, Yutong Zhang
EPI_ISL_430421 hCoV-19/USA/NY-NYUMC394/2020 North America / USA / New York / Brooklyn 2020-04-13 NYU Langone Health Departments of Pathology and Medicine, New York University School of Medicine Maria Aguero-Rosenfeld, Brendan Belovarac, Margaret Black, Ludovic Boytard, John Cadley, Paolo Cotzia, John Chen, Dacia Dimartino, Xiaojun Feng, Tatyana Gindin, Emily Guzman, Adriana Heguy, Megan Hogan, Emily Huang, George Jour, Lawrence H. Lin, Raven Luther, Andrew Lytle, Christian Marier, Matthew T. Maurano, Mark J. Mulligan, Peter Meyn, Raquel Ordonez Ciriza, Iman Osman, Jared Pinnell, Vanessa Raabe, Sitharam Ramaswami, Amy Rapkiewicz, Andre M. Ribeiro-dos-Santos, Marie Samanovic-Golden, Antonio Serrano, Guomiao Shen, Matija Snuderl, Theodore Vougiouklakis, Nick Vulpescu, Gael Westby, Paul Zappile, Yutong Zhang
EPI_ISL_430422 hCoV-19/USA/NY-NYUMC395/2020 North America / USA / New York / Queens 2020-04-13 NYU Langone Health Departments of Pathology and Medicine, New York University School of Medicine Maria Aguero-Rosenfeld, Brendan Belovarac, Margaret Black, Ludovic Boytard, John Cadley, Paolo Cotzia, John Chen, Dacia Dimartino, Xiaojun Feng, Tatyana Gindin, Emily Guzman, Adriana Heguy, Megan Hogan, Emily Huang, George Jour, Lawrence H. Lin, Raven Luther, Andrew Lytle, Christian Marier, Matthew T. Maurano, Mark J. Mulligan, Peter Meyn, Raquel Ordonez Ciriza, Iman Osman, Jared Pinnell, Vanessa Raabe, Sitharam Ramaswami, Amy Rapkiewicz, Andre M. Ribeiro-dos-Santos, Marie Samanovic-Golden, Antonio Serrano, Guomiao Shen, Matija Snuderl, Theodore Vougiouklakis, Nick Vulpescu, Gael Westby, Paul Zappile, Yutong Zhang
EPI_ISL_430423 hCoV-19/USA/NY-NYUMC396/2020 North America / USA / New York / Queens 2020-04-11 NYU Langone Health Departments of Pathology and Medicine, New York University School of Medicine Maria Aguero-Rosenfeld, Brendan Belovarac, Margaret Black, Ludovic Boytard, John Cadley, Paolo Cotzia, John Chen, Dacia Dimartino, Xiaojun Feng, Tatyana Gindin, Emily Guzman, Adriana Heguy, Megan Hogan, Emily Huang, George Jour, Lawrence H. Lin, Raven Luther, Andrew Lytle, Christian Marier, Matthew T. Maurano, Mark J. Mulligan, Peter Meyn, Raquel Ordonez Ciriza, Iman Osman, Jared Pinnell, Vanessa Raabe, Sitharam Ramaswami, Amy Rapkiewicz, Andre M. Ribeiro-dos-Santos, Marie Samanovic-Golden, Antonio Serrano, Guomiao Shen, Matija Snuderl, Theodore Vougiouklakis, Nick Vulpescu, Gael Westby, Paul Zappile, Yutong Zhang
EPI_ISL_430424 hCoV-19/USA/NY-NYUMC397/2020 North America / USA / New York / Brooklyn 2020-04-13 NYU Langone Health Departments of Pathology and Medicine, New York University School of Medicine Maria Aguero-Rosenfeld, Brendan Belovarac, Margaret Black, Ludovic Boytard, John Cadley, Paolo Cotzia, John Chen, Dacia Dimartino, Xiaojun Feng, Tatyana Gindin, Emily Guzman, Adriana Heguy, Megan Hogan, Emily Huang, George Jour, Lawrence H. Lin, Raven Luther, Andrew Lytle, Christian Marier, Matthew T. Maurano, Mark J. Mulligan, Peter Meyn, Raquel Ordonez Ciriza, Iman Osman, Jared Pinnell, Vanessa Raabe, Sitharam Ramaswami, Amy Rapkiewicz, Andre M. Ribeiro-dos-Santos, Marie Samanovic-Golden, Antonio Serrano, Guomiao Shen, Matija Snuderl, Theodore Vougiouklakis, Nick Vulpescu, Gael Westby, Paul Zappile, Yutong Zhang
EPI_ISL_430425 hCoV-19/USA/NY-NYUMC398/2020 North America / USA / New York / Manhattan 2020-04-13

NYU Langone Health	Departments of Pathology and Medicine, New York University School of Medicine	Maria Aguero-Rosenfeld, Brendan Belovarac, Margaret Black, Ludovic Boytard, John Cadley, Paolo Cotzia, John Chen, Dacia Dimartino, Xiaojun Feng, Tatyana Gindin, Emily Guzman, Adriana Heguy, Megan Hogan, Emily Huang, George Jour, Lawrence H. Lin, Raven Luther, Andrew Lytle, Christian Marier, Matthew T. Maurano, Mark J. Mulligan, Peter Meyn, Raquel Ordonez Ciriza, Iman Osman, Jared Pinnell, Vanessa Raabe, Sitharam Ramaswami, Amy Rapkiewicz, Andre M. Ribeiro-dos-Santos, Marie Samanovic-Golden, Antonio Serrano, Guomiao Shen, Matija Snuderl, Theodore Vougiouklakis, Nick Vulpescu, Gael Westby, Paul Zappile, Yutong Zhang
EPI_ISL_430426	hCoV-19/USA/NY-NYUMC399/2020	North America / USA / New York / Manhattan	2020-04-13	NYU Langone Health	Departments of Pathology and Medicine, New York University School of Medicine	Maria Aguero-Rosenfeld, Brendan Belovarac, Margaret Black, Ludovic Boytard, John Cadley, Paolo Cotzia, John Chen, Dacia Dimartino, Xiaojun Feng, Tatyana Gindin, Emily Guzman, Adriana Heguy, Megan Hogan, Emily Huang, George Jour, Lawrence H. Lin, Raven Luther, Andrew Lytle, Christian Marier, Matthew T. Maurano, Mark J. Mulligan, Peter Meyn, Raquel Ordonez Ciriza, Iman Osman, Jared Pinnell, Vanessa Raabe, Sitharam Ramaswami, Amy Rapkiewicz, Andre M. Ribeiro-dos-Santos, Marie Samanovic-Golden, Antonio Serrano, Guomiao Shen, Matija Snuderl, Theodore Vougiouklakis, Nick Vulpescu, Gael Westby, Paul Zappile, Yutong Zhang
EPI_ISL_430427	hCoV-19/USA/NY-NYUMC400/2020	North America / USA / New York / Nassau County	2020-04-13	NYU Langone Health	Departments of Pathology and Medicine, New York University School of Medicine	Maria Aguero-Rosenfeld, Brendan Belovarac, Margaret Black, Ludovic Boytard, John Cadley, Paolo Cotzia, John Chen, Dacia Dimartino, Xiaojun Feng, Tatyana Gindin, Emily Guzman, Adriana Heguy, Megan Hogan, Emily Huang, George Jour, Lawrence H. Lin, Raven Luther, Andrew Lytle, Christian Marier, Matthew T. Maurano, Mark J. Mulligan, Peter Meyn, Raquel Ordonez Ciriza, Iman Osman, Jared Pinnell, Vanessa Raabe, Sitharam Ramaswami, Amy Rapkiewicz, Andre M. Ribeiro-dos-Santos, Marie Samanovic-Golden, Antonio Serrano, Guomiao Shen, Matija Snuderl, Theodore Vougiouklakis, Nick Vulpescu, Gael Westby, Paul Zappile, Yutong Zhang
EPI_ISL_430428	hCoV-19/USA/NJ-NYUMC401/2020	North America / USA / New Jersey / Hudson County	2020-04-13	NYU Langone Health	Departments of Pathology and Medicine, New York University School of Medicine	Maria Aguero-Rosenfeld, Brendan Belovarac, Margaret Black, Ludovic Boytard, John Cadley, Paolo Cotzia, John Chen, Dacia Dimartino, Xiaojun Feng, Tatyana Gindin, Emily Guzman, Adriana Heguy, Megan Hogan, Emily Huang, George Jour, Lawrence H. Lin, Raven Luther, Andrew Lytle, Christian Marier, Matthew T. Maurano, Mark J. Mulligan, Peter Meyn, Raquel Ordonez Ciriza, Iman Osman, Jared Pinnell, Vanessa Raabe, Sitharam Ramaswami, Amy Rapkiewicz, Andre M. Ribeiro-dos-Santos, Marie Samanovic-Golden, Antonio Serrano, Guomiao Shen, Matija Snuderl, Theodore Vougiouklakis, Nick Vulpescu, Gael Westby, Paul Zappile, Yutong Zhang
EPI_ISL_430429	hCoV-19/USA/NY-NYUMC402/2020	North America / USA / New York / Brooklyn	2020-04-13	NYU Langone Health	Departments of Pathology and Medicine, New York University School of Medicine	Maria Aguero-Rosenfeld, Brendan Belovarac, Margaret Black, Ludovic Boytard, John Cadley, Paolo Cotzia, John Chen, Dacia Dimartino, Xiaojun Feng, Tatyana Gindin, Emily Guzman, Adriana Heguy, Megan Hogan, Emily Huang, George Jour, Lawrence H. Lin, Raven Luther, Andrew Lytle, Christian Marier, Matthew T. Maurano, Mark J. Mulligan, Peter Meyn, Raquel Ordonez Ciriza, Iman Osman, Jared Pinnell, Vanessa Raabe, Sitharam Ramaswami, Amy Rapkiewicz, Andre M. Ribeiro-dos-Santos, Marie Samanovic-Golden, Antonio Serrano, Guomiao Shen, Matija Snuderl, Theodore Vougiouklakis, Nick Vulpescu, Gael Westby, Paul Zappile, Yutong Zhang
EPI_ISL_430430	hCoV-19/USA/NY-NYUMC403/2020	North America / USA / New York / Bronx	2020-04-13	NYU Langone Health	Departments of Pathology and Medicine, New York University School of Medicine	Maria Aguero-Rosenfeld, Brendan Belovarac, Margaret Black, Ludovic Boytard, John Cadley, Paolo Cotzia, John Chen, Dacia Dimartino, Xiaojun Feng, Tatyana Gindin, Emily Guzman, Adriana Heguy, Megan Hogan, Emily Huang, George Jour, Lawrence H. Lin, Raven Luther, Andrew Lytle, Christian Marier, Matthew T. Maurano, Mark J. Mulligan, Peter Meyn, Raquel Ordonez Ciriza, Iman Osman, Jared Pinnell, Vanessa Raabe, Sitharam Ramaswami, Amy Rapkiewicz, Andre M. Ribeiro-dos-Santos, Marie Samanovic-Golden, Antonio Serrano, Guomiao Shen, Matija Snuderl, Theodore Vougiouklakis, Nick Vulpescu, Gael Westby, Paul Zappile, Yutong Zhang
EPI_ISL_430431	hCoV-19/USA/NY-NYUMC404/2020	North America / USA / New York / Nassau County	2020-04-13	NYU Langone Health	Departments of Pathology and Medicine, New York University School of Medicine	Maria Aguero-Rosenfeld, Brendan Belovarac, Margaret Black, Ludovic Boytard, John Cadley, Paolo Cotzia, John Chen, Dacia Dimartino, Xiaojun Feng, Tatyana Gindin, Emily Guzman, Adriana Heguy, Megan Hogan, Emily Huang, George Jour, Lawrence H. Lin, Raven Luther, Andrew Lytle, Christian Marier, Matthew T. Maurano, Mark J. Mulligan, Peter Meyn, Raquel Ordonez Ciriza, Iman Osman, Jared Pinnell, Vanessa Raabe, Sitharam Ramaswami, Amy Rapkiewicz, Andre M. Ribeiro-dos-Santos, Marie Samanovic-Golden, Antonio Serrano, Guomiao Shen, Matija Snuderl, Theodore Vougiouklakis, Nick Vulpescu, Gael Westby, Paul Zappile, Yutong Zhang
EPI_ISL_430432	hCoV-19/USA/NY-NYUMC405/2020	North America / USA / New York / Staten Island	2020-04-13	NYU Langone Health	Departments of Pathology and Medicine, New York University School of Medicine	Maria Aguero-Rosenfeld, Brendan Belovarac, Margaret Black, Ludovic Boytard, John Cadley, Paolo Cotzia, John Chen, Dacia Dimartino, Xiaojun Feng, Tatyana Gindin, Emily Guzman, Adriana Heguy, Megan Hogan, Emily Huang, George Jour, Lawrence H. Lin, Raven Luther, Andrew Lytle, Christian Marier, Matthew T. Maurano, Mark J. Mulligan, Peter Meyn, Raquel Ordonez Ciriza, Iman Osman, Jared Pinnell, Vanessa Raabe, Sitharam Ramaswami, Amy Rapkiewicz, Andre M. Ribeiro-dos-Santos, Marie Samanovic-Golden, Antonio Serrano, Guomiao Shen, Matija Snuderl, Theodore Vougiouklakis, Nick Vulpescu, Gael Westby, Paul Zappile, Yutong Zhang
EPI_ISL_430433	hCoV-19/USA/NY-NYUMC406/2020	North America / USA / New York / Queens	2020-04-13	NYU Langone Health	Departments of Pathology and Medicine, New York University School of Medicine	Maria Aguero-Rosenfeld, Brendan Belovarac, Margaret Black, Ludovic Boytard, John Cadley, Paolo Cotzia, John Chen, Dacia Dimartino, Xiaojun Feng, Tatyana Gindin, Emily Guzman, Adriana Heguy, Megan Hogan, Emily Huang, George Jour, Lawrence H. Lin, Raven Luther, Andrew Lytle, Christian Marier, Matthew T. Maurano, Mark J. Mulligan, Peter Meyn, Raquel Ordonez Ciriza, Iman Osman, Jared Pinnell, Vanessa Raabe, Sitharam Ramaswami, Amy Rapkiewicz, Andre M. Ribeiro-dos-Santos, Marie Samanovic-Golden, Antonio Serrano, Guomiao Shen, Matija Snuderl, Theodore

Vougiouklakis, Nick Vulpescu, Gael Westby, Paul Zappile, Yutong Zhang
EPI_ISL_430434 hCoV-19/USA/NY-NYUMC407/2020 North America / USA / New York / Queens 2020-04-12 NYU Langone Health Departments of Pathology and Medicine, New York University School of Medicine Maria Aguero-Rosenfeld, Brendan Belovarac, Margaret Black, Ludovic Boytard, John Cadley, Paolo Cotzia, John Chen, Dacia Dimartino, Xiaojun Feng, Tatyana Gindin, Emily Guzman, Adriana Heguy, Megan Hogan, Emily Huang, George Jour, Lawrence H. Lin, Raven Luther, Andrew Lytle, Christian Marier, Matthew T. Maurano, Mark J. Mulligan, Peter Meyn, Raquel Ordonez Ciriza, Iman Osman, Jared Pinnell, Vanessa Raabe, Sitharam Ramaswami, Amy Rapkiewicz, Andre M. Ribeiro-dos-Santos, Marie Samanovic-Golden, Antonio Serrano, Guomiao Shen, Matija Snuderl, Theodore Vougiouklakis, Nick Vulpescu, Gael Westby, Paul Zappile, Yutong Zhang
EPI_ISL_430439 hCoV-19/Malaysia/IMR_WC1177/2020 Asia / Malaysia 2020-03-05 Institute for Medical Research, Infectious Disease Research Centre, National Institutes of Health, Ministry of Health Malaysia Institute for Medical Research Infectious Disease Research Centre, National Institutes of Health, Ministry of Health Malaysia Suppiah.J, Mohd-Zawawi.Z, Kalyanasundram.J, Azizan.M-A, Mat-Sharani.S, Hisham.H-A, Tan.L-P, Abdul-Wahid.M-Z, Mohd-Zain.R, Ahmad.N, Thayan.R
EPI_ISL_430440 hCoV-19/Malaysia/IMR_WC1170/2020 Asia / Malaysia 2020-03-05 Institute for Medical Research, Infectious Disease Research Centre, National Institutes of Health, Ministry of Health Malaysia Institute for Medical Research, Infectious Disease Research Centre, National Institutes of Health, Ministry of Health Malaysia Suppiah.J, Mohd-Zawawi.Z, Kalyanasundram.J, Azizan.M-A, Mat-Sharani.S, Hisham.H-A, Tan.L-P, Abdul-Wahid.M-Z, Tengku-Abd-Rashid.T-R, Mohd-Zain.R, Ahmad.N, Thayan.R
EPI_ISL_430441 hCoV-19/Malaysia/IMR_WC1097/2020 Asia / Malaysia 2020-02-29 Institute for Medical Research, Infectious Disease Research Centre, National Institutes of Health, Ministry of Health Malaysia Institute for Medical Research, Infectious Disease Research Centre, National Institutes of Health, Ministry of Health Malaysia Suppiah.J, Mohd-Zawawi.Z, Kalyanasundram.J, Azizan.M-A, Mat-Sharani.S, Hisham.H-A, Tan.L-P, Abdul-Wahid.M-Z, Tengku-Rogayah.TAR, Mohd-Zain.R, Ahmad.N, Thayan.R
EPI_ISL_430442 hCoV-19/Malaysia/IMR_WC1098/2020 Asia / Malaysia 2020-02-29 Institute for Medical Research, Infectious Disease Research Centre, National Institutes of Health, Ministry of Health Malaysia Institute for Medical Research, Infectious Disease Research Centre, National Institutes of Health, Ministry of Health Malaysia Suppiah.J, Mohd-Zawawi.Z, Kalyanasundram.J, Azizan.M-A, Mat-Sharani.S, Hisham.H-A, Tan.L-P, Abdul-Wahid.M-Z, Tengku-Rogayah.TAR, Mohd-Zain.R, Ahmad.N, Thayan.R
EPI_ISL_430443 hCoV-19/Malaysia/IMR_WC085/2020 Asia / Malaysia 2020-01-28 Institute for Medical Research, Infectious Disease Research Centre, National Institutes of Health, Ministry of Health Malaysia Institute for Medical Research, Infectious Disease Research Centre, National Institutes of Health, Ministry of Health Malaysia Suppiah.J, Mohd-Zawawi.Z, Kalyanasundram.J, Azizan.M-A, Mat-Sharani.S, Hisham.H-A, Tan.L-P, Abdul-Wahid.M-Z, Tengku-Rogayah.TAR, Mohd-Zain.R, Ahmad.N, Thayan.R
EPI_ISL_430444 hCoV-19/Malaysia/IMR_WC627/2020 Asia / Malaysia 2020-02-12 Institute for Medical Research, Infectious Disease Research Centre, National Institutes of Health, Ministry of Health Malaysia Institute for Medical Research, Infectious Disease Research Centre, National Institutes of Health, Ministry of Health Malaysia Suppiah.J, Mohd-Zawawi.Z, Kalyanasundram.J, Azizan.M-A, Mat-Sharani.S, Hisham.H-A, Tan.L-P, Abdul-Wahid.M-Z, Tengku-Rogayah.TAR, Mohd-Zain.R, Ahmad.N, Thayan.R
EPI_ISL_430464 hCoV-19/India/S3/2020 Asia / India / West Bengal / Kolkata 2020-03-21 ICMR-National Institute of Cholera and Enteric Diseases National Institute of Biomedical Genomics Arindam Maitra, Mamta Chawla Sarkar, Sreedhar Chinnaswamy, Hasina Banu, Ananya Chatterjee, Shanta Dutta, Saumitra Das
EPI_ISL_430465 hCoV-19/India/S5/2020 Asia / India / West Bengal / Darjeeling 2020-03-28 ICMR-National Institute of Cholera and Enteric Diseases National Institute of Biomedical Genomics Arindam Maitra, Mamta Chawla Sarkar, Sreedhar Chinnaswamy, Hasina Banu, Ananya Chatterjee, Shanta Dutta, Saumitra Das
EPI_ISL_430466 hCoV-19/India/S6/2020 Asia / India / West Bengal / Tehatta 2020-03-26 ICMR-National Institute of Cholera and Enteric Diseases National Institute of Biomedical Genomics Arindam Maitra, Mamta Chawla Sarkar, Sreedhar Chinnaswamy, Hasina Banu, Ananya Chatterjee, Shanta Dutta, Saumitra Das
EPI_ISL_430467 hCoV-19/India/S11/2020 Asia / India / West Bengal / East Medinipur 2020-04-03 ICMR-National Institute of Cholera and Enteric Diseases National Institute of Biomedical Genomics Arindam Maitra, Mamta Chawla Sarkar, Sreedhar Chinnaswamy, Hasina Banu, Ananya Chatterjee, Shanta Dutta, Saumitra Das
EPI_ISL_430468 hCoV-19/India/S2/2020 Asia / India / West_Bengal / Kolkata 2020-03-21 ICMR-National Institute of Cholera and Enteric Diseases National Institute of Biomedical Genomics Arindam Maitra, Mamta Chawla Sarkar, Sreedhar Chinnaswamy, Hasina Banu, Ananya Chatterjee, Shanta Dutta, Saumitra Das
EPI_ISL_430469 hCoV-19/Greece/127_HPI/2020 Europe / Greece / Athens 2020-02-29 Hellenic Pasteur Institute, Public Health Laboratories Hellenic Pasteur Institute, Public Health Laboratories, Unit of Bioinformatics and Applied Genomics Vasiliki Pogka, Timokratis Karamitros, Athanasios Kossyvakis, Antonios Kalliaropoulos, Horefti Elina, Evangelidou Maria, Androniki Voulgari-Kokota, Aspasia Kontou, Andreas Mentis
EPI_ISL_430471 hCoV-19/Australia/VIC935/2020 Oceania / Australia / Victoria 2020-03-25 Microbiological Diagnostic Unit Public Health Laboratory Microbiological Diagnostic Unit Public Health Laboratory Seemann T., Schultz M., Sait, M., Sherry, N.
EPI_ISL_430472 hCoV-19/Australia/VIC938/2020 Oceania / Australia / Victoria 2020-03-25 Microbiological Diagnostic Unit Public Health Laboratory Microbiological Diagnostic Unit Public Health Laboratory Seemann T., Schultz M., Sait, M., Sherry, N.
EPI_ISL_430473 hCoV-19/Australia/VIC1008/2020 Oceania / Australia / Victoria 2020-03-31 Victorian Infectious Diseases Reference Laboratory (VIDRL) Microbiological Diagnostic Unit Public Health Laboratory and Victorian Infectious Diseases Reference Laboratory, The Peter Doherty Institute for Infection and Immunity Caly L., Seemann T., Sait, M., Schultz M., Druce J., Sherry, N.
EPI_ISL_430476 hCoV-19/Australia/VIC1016/2020 Oceania / Australia / Victoria 2020-04-01 Victorian Infectious Diseases Reference Laboratory (VIDRL) Microbiological Diagnostic Unit Public Health

Laboratory and Victorian Infectious Diseases Reference Laboratory, The Peter Doherty Institute for Infection and Immunity Caly L., Seemann T., Sait, M., Schultz M., Druce J., Sherry, N.
EPI_ISL_430478 hCoV-19/Australia/VIC994/2020 Oceania / Australia / Victoria 2020-03-31 Victorian Infectious Diseases Reference Laboratory (VIDRL) Microbiological Diagnostic Unit Public Health Laboratory and Victorian Infectious Diseases Reference Laboratory, The Peter Doherty Institute for Infection and Immunity Caly L., Seemann T., Sait, M., Schultz M., Druce J., Sherry, N.
EPI_ISL_430479 hCoV-19/Australia/VIC995/2020 Oceania / Australia / Victoria 2020-03-31 Victorian Infectious Diseases Reference Laboratory (VIDRL) Microbiological Diagnostic Unit Public Health Laboratory and Victorian Infectious Diseases Reference Laboratory, The Peter Doherty Institute for Infection and Immunity Caly L., Seemann T., Sait, M., Schultz M., Druce J., Sherry, N.
EPI_ISL_430481 hCoV-19/Australia/VIC997/2020 Oceania / Australia / Victoria 2020-03-31 Victorian Infectious Diseases Reference Laboratory (VIDRL) Microbiological Diagnostic Unit Public Health Laboratory and Victorian Infectious Diseases Reference Laboratory, The Peter Doherty Institute for Infection and Immunity Caly L., Seemann T., Sait, M., Schultz M., Druce J., Sherry, N.
EPI_ISL_430482 hCoV-19/Australia/VIC998/2020 Oceania / Australia / Victoria 2020-03-31 Victorian Infectious Diseases Reference Laboratory (VIDRL) Microbiological Diagnostic Unit Public Health Laboratory and Victorian Infectious Diseases Reference Laboratory, The Peter Doherty Institute for Infection and Immunity Caly L., Seemann T., Sait, M., Schultz M., Druce J., Sherry, N.
EPI_ISL_430484 hCoV-19/Australia/VIC1000/2020 Oceania / Australia / Victoria 2020-03-31 Victorian Infectious Diseases Reference Laboratory (VIDRL) Microbiological Diagnostic Unit Public Health Laboratory and Victorian Infectious Diseases Reference Laboratory, The Peter Doherty Institute for Infection and Immunity Caly L., Seemann T., Sait, M., Schultz M., Druce J., Sherry, N.
EPI_ISL_430486 hCoV-19/Australia/VIC1002/2020 Oceania / Australia / Victoria 2020-03-31 Victorian Infectious Diseases Reference Laboratory (VIDRL) Microbiological Diagnostic Unit Public Health Laboratory and Victorian Infectious Diseases Reference Laboratory, The Peter Doherty Institute for Infection and Immunity Caly L., Seemann T., Sait, M., Schultz M., Druce J., Sherry, N.
EPI_ISL_430490 hCoV-19/Australia/VIC1018/2020 Oceania / Australia / Victoria 2020-04-01 Victorian Infectious Diseases Reference Laboratory (VIDRL) Microbiological Diagnostic Unit Public Health Laboratory and Victorian Infectious Diseases Reference Laboratory, The Peter Doherty Institute for Infection and Immunity Caly L., Seemann T., Sait, M., Schultz M., Druce J., Sherry, N.
EPI_ISL_430498 hCoV-19/Australia/VIC1208/2020 Oceania / Australia / Victoria 2020-04-01 Victorian Infectious Diseases Reference Laboratory (VIDRL) Microbiological Diagnostic Unit Public Health Laboratory and Victorian Infectious Diseases Reference Laboratory, The Peter Doherty Institute for Infection and Immunity Caly L., Seemann T., Sait, M., Schultz M., Druce J., Sherry, N.
EPI_ISL_406716 hCoV-19/Wuhan/WHU01/2020 Asia / China / Hubei / Wuhan 2020-01-02 unknown State Key Laboratory of Virology, Wuhan University Chen,L., Liu,W., Zhang,Q., Xu,K., Ye,G., Wu,W., Sun,Z., Liu,F., Wu,K., Mei,Y., Zhang,W., Chen,Y., Li,Y., Shi,M., Lan,K. and Liu,Y.
EPI_ISL_406717 hCoV-19/Wuhan/WHU02/2020 Asia / China / Hubei / Wuhan 2020-01-02 unknown State Key Laboratory of Virology, Wuhan University Chen,L., Liu,W., Zhang,Q., Xu,K., Ye,G., Wu,W., Sun,Z., Liu,F., Wu,K., Mei,Y., Zhang,W., Chen,Y., Li,Y., Shi,M., Lan,K. and Liu,Y.
EPI_ISL_410486 hCoV-19/France/RA739/2020 Europe / France / Rhone-Alpes / Contamines 2020-02-08 CNR Virus des Infections Respiratoires - France SUD CNR Virus des Infections Respiratoires - France SUD Bal, Antonin; Destras, Gregory; Gaymard, Alexandre; Bouscambert-Duchamp, Maude; Cheynet, Valérie; Brengel-Pesce, Karen; Morfin-Sherpa, Florence; Valette, Martine; Josset, Laurence; Lina, Bruno.
EPI_ISL_410531 hCoV-19/Japan/NA-20-05-1/2020 Asia / Japan / Nara 2020-01-25 Dept. of Pathology, National Institute of Infectious Diseases Pathogen Genomics Center, National Institute of Infectious Diseases Tsuyoshi Sekizuka, Harutaka Katano, Shutoku Matsuyama, Naganori Nao, Kazuya Shirato, Motoi Suzuki, Hideki Hasegawa, Takaji Wakita, Makoto Takeda, Tadaki Suzuki, Makoto Kuroda
EPI_ISL_410532 hCoV-19/Japan/OS-20-07-1/2020 Asia / Japan / Osaka 2020-01-23 Dept. of Pathology, National Institute of Infectious Diseases Pathogen Genomics Center, National Institute of Infectious Diseases Tsuyoshi Sekizuka, Harutaka Katano, Shutoku Matsuyama, Naganori Nao, Kazuya Shirato, Motoi Suzuki, Hideki Hasegawa, Takaji Wakita, Makoto Takeda, Tadaki Suzuki, Makoto Kuroda
EPI_ISL_410535 hCoV-19/Singapore/4/2020 Asia / Singapore 2020-02-03 National Centre for Infectious Diseases Programme in Emerging Infectious Diseases, Duke-NUS Medical School Danielle E Anderson, Martin Linster, Yan Zhuang, Jayanthi Jayakumar, David CB Lye, Yee Sin Leo, Barnaby E Young, Yvonne CF Su, Gavin JD Smith
EPI_ISL_410536 hCoV-19/Singapore/5/2020 Asia / Singapore 2020-02-06 Singapore General Hospital, Molecular Laboratory, Division of Pathology Programme in Emerging Infectious Diseases, Duke-NUS Medical School Danielle E Anderson, Martin Linster, Yan Zhuang, Jayanthi Jayakumar, Kian Sing Chan, Lynette LE Oon, Shirin Kalimuddin, Jenny GH Low, Yvonne CF Su, Gavin JD Smith
EPI_ISL_410537 hCoV-19/Singapore/6/2020 Asia / Singapore 2020-02-09 Singapore General Hospital, Molecular Laboratory, Division of Pathology Programme in Emerging Infectious Diseases, Duke-NUS Medical School Danielle E Anderson, Martin Linster, Yan Zhuang, Jayanthi Jayakumar, Kian Sing Chan, Lynette LE Oon, Shirin Kalimuddin, Jenny GH Low, Yvonne CF Su, Gavin JD Smith
EPI_ISL_410545 hCoV-19/Italy/INMI1-isl/2020 Europe / Italy / Rome 2020-01-29 INMI Lazzaro Spallanzani IRCCS Laboratory of Virology, INMI Lazzaro Spallanzani IRCCS Maria R. Capobianchi, Cesare E. M. Gruber, Martina Rueca, Barbara Bartolini, Francesco Messina, Emanuela Giombini, Francesca Colavita, Concetta Castilletti, Eleonora Lalle, Fabrizio Carletti, Emanuele Nicastri, Giuseppe Ippolito.
EPI_ISL_410546 hCoV-19/Italy/INMI1-cs/2020 Europe / Italy / Rome 2020-01-31 INMI Lazzaro Spallanzani IRCCS Laboratory of Virology, INMI Lazzaro Spallanzani IRCCS Maria R. Capobianchi, Cesare E.

M. Gruber, Martina Rueca, Fabrizio Carletti, Barbara Bartolini, Francesco Messina, Emanuela Giombini, Francesca Colavita, Concetta Castilletti, Eleonora Lalle, Emanuele Nicastri, Giuseppe Ippolito.
EPI_ISL_411218 hCoV-19/France/IDF0571/2020 Europe / France / Ile-de-France / Paris 2020-02-02 Department of Infectious and Tropical Diseases, Bichat Claude Bernard Hospital, Paris Laboratoire Virpath, CIRI U111, UCBL1, INSERM, CNRS, ENS Lyon Olivier Terrier, Aurélien Traversier, Julien Fouret, Yazdan Yazdanpanah, Xavier Lescure, Catherine Legras-Lachuer, Alexandre Gaymard, Bruno Lina, Manuel Rosa-Calatrava
EPI_ISL_411219 hCoV-19/France/IDF0386-islP1/2020 Europe / France / Ile-de-France / Paris 2020-01-28 Department of Infectious and Tropical Diseases, Bichat Claude Bernard Hospital, Paris Laboratoire Virpath, CIRI U111, UCBL1, INSERM, CNRS, ENS Lyon Olivier Terrier, Aurélien Traversier, Julien Fouret, Yazdan Yazdanpanah, Xavier Lescure, Alexandre Gaymard, Bruno Lina, Manuel Rosa-Calatrava
EPI_ISL_411220 hCoV-19/France/IDF0386-islP3/2020 Europe / France / Ile-de-France / Paris 2020-01-28 Department of Infectious and Tropical Diseases, Bichat Claude Bernard Hospital, Paris Laboratoire Virpath, CIRI U111, UCBL1, INSERM, CNRS, ENS Lyon Olivier Terrier, Aurélien Traversier, Julien Fouret, Yazdan Yazdanpanah, Xavier Lescure, Alexandre Gaymard, Bruno Lina, Manuel Rosa-Calatrava
EPI_ISL_411950 hCoV-19/Jiangsu/JS01/2020 Asia / China / Jiangsu 2020-01-23 NHC Key laboratory of Enteric Pathogenic Microbiology, Institute of Pathogenic Microbiology Jiangsu Provincial Center for Disease Control & Prevention Lunbiao Cui,Kangchen Zhao,Xiaojuan Zhu,Yiyue Ge,Tao Wu,Bin Wu,Yin Chen,Fengcai Zhu,Baoli Zhu,Ming Wu
EPI_ISL_411951 hCoV-19/Sweden/01/2020 Europe / Sweden 2020-02-07 unknown Unit for Laboratory Development and Technology Transfer, Public Health Agency of Sweden Bengner,M., Palmerus,M., Lindsjo,O., Lind Karlberg,M., Monteil,V., Appelberg,S., Brave,A., Muradrasoli,S. and Tegmark-Wisell,K.
EPI_ISL_411952 hCoV-19/Jiangsu/JS02/2020 Asia / China / Jiangsu 2020-01-24 NHC Key laboratory of Enteric Pathogenic Microbiology, Institute of Pathogenic Microbiology Jiangsu Provincial Center for Disease Control & Prevention Kangchen Zhao, Xiaojuan Zhu, Lunbiao Cui, Tao Wu, Yiyue Ge, Bin Wu, Yin Chen, Fengcai Zhu, Baoli Zhu, Ming Wu
EPI_ISL_411953 hCoV-19/Jiangsu/JS03/2020 Asia / China / Jiangsu 2020-01-24 NHC Key laboratory of Enteric Pathogenic Microbiology, Institute of Pathogenic Microbiology Jiangsu Provincial Center for Disease Control & Prevention Kangchen Zhao, Xiaojuan Zhu, Lunbiao Cui, Tao Wu, Yiyue Ge, Bin Wu, Yin Chen, Fengcai Zhu, Baoli Zhu, Ming Wu
EPI_ISL_411954 hCoV-19/USA/CA7/2020 North America / USA / California 2020-02-06 California Department of Public Health Pathogen Discovery, Respiratory Viruses Branch, Division of Viral Diseases, Centers for Dieases Control and Prevention Krista Queen, Anna Uehara, Jing Zhang, Yan Li, Ying Tao, Clinton R. Paden, Haibin Wang, Shifaq Kamili, Xiaoyan Lu, Brian Lynch, Senthil Kumar K. Sakthivel, Brett L. Whitaker, Lijuan Wang, Janna' R. Murray, Susan I. Gerber, Stephen Lindstrom, Suxiang Tong
EPI_ISL_411955 hCoV-19/USA/CA8/2020 North America / USA / California 2020-02-10 California Department of Public Health Pathogen Discovery, Respiratory Viruses Branch, Division of Viral Diseases, Centers for Dieases Control and Prevention Krista Queen, Anna Uehara, Jing Zhang, Yan Li, Ying Tao, Clinton R. Paden, Haibin Wang, Shifaq Kamili, Xiaoyan Lu, Brian Lynch, Senthil Kumar K. Sakthivel, Brett L. Whitaker, Lijuan Wang, Janna' R. Murray, Susan I. Gerber, Stephen Lindstrom, Suxiang Tong
EPI_ISL_411956 hCoV-19/USA/TX1/2020 North America / USA / Texas 2020-02-11 Texas Department of State Health Services Pathogen Discovery, Respiratory Viruses Branch, Division of Viral Diseases, Centers for Dieases Control and Prevention Krista Queen, Anna Uehara, Jing Zhang, Yan Li, Ying Tao, Clinton R. Paden, Haibin Wang, Shifaq Kamili, Xiaoyan Lu, Brian Lynch, Senthil Kumar K. Sakthivel, Brett L. Whitaker, Lijuan Wang, Janna' R. Murray, Susan I. Gerber, Stephen Lindstrom, Suxiang Tong
EPI_ISL_411957 hCoV-19/China/WH-09/2020 Asia / China 2020-01-08 unknown Key Laboratory of Human Diseases, Comparative Medicine, Institute of Laboratory Animal Science Linlin,B., Lili,R., Shuran,G., Jiangning,L., Feifei,Q., Qi,L., Fengdi,L., Jing,X., Wei,D., Pin,Y., Yanfeng,X., Yajin,Q., Hong,G., Qiang,W., Mingya,L., Guanpeng,W., Shunyi,W., Zhiqi,S., Li,G., Lan,C., Conghui,W., Ying,W., Xinming,W., Yan,X., Qi,J. and Chuan,Q.
EPI_ISL_413851 hCoV-19/Guangdong/2020XN4373-P0039/2020 Asia / China / Guangdong 2020-01-30 Guangdong Provincial Institution of Public Health, Guangdong Provinical Center for Disease Control and Prevention Guangdong Provincial Institution of Public Health Jing Lu, Louis du Plessis, Liu Zhe, Jiufeng Sun, Sarah François, Huifang Lin, Moritz Kraemer, Jingju Peng, Qianlin Xiong, Runyu Yuan, Lilian Zeng, Pingping Zhou, Chuming Liang, Tao Liu, Wei Li, Juan Su, Huanying Zheng, Kang Min, Song Tie, Bo Peng, Shisong Fang, Wenzhe Su, Kuibiao Li, Ruilin Sun, Ru bai, Xi Tang, Minfeng Liang, Nuno Faria, Josh Quick, Andrew Rambaut, Verity Hill, Wenjun Ma, Nick Loman, Oliver Pybus, Changwen Ke
EPI_ISL_413852 hCoV-19/Guangdong/2020XN4433-P0040/2020 Asia / China / Guangdong 2020-01-30 Guangdong Provincial Institution of Public Health, Guangdong Provinical Center for Disease Control and Prevention Guangdong Provincial Institution of Public Health Jing Lu, Louis du Plessis, Liu Zhe, Jiufeng Sun, Sarah François, Huifang Lin, Moritz Kraemer, Jingju Peng, Qianlin Xiong, Runyu Yuan, Lilian Zeng, Pingping Zhou, Chuming Liang, Tao Liu, Wei Li, Juan Su, Huanying Zheng, Kang Min, Song Tie, Bo Peng, Shisong Fang, Wenzhe Su, Kuibiao Li, Ruilin Sun, Ru bai, Xi Tang, Minfeng Liang, Nuno Faria, Josh Quick, Andrew Rambaut, Verity Hill, Wenjun Ma, Nick Loman, Oliver Pybus, Changwen Ke
EPI_ISL_413853 hCoV-19/Guangdong/2020XN4243-P0035/2020 Asia / China / Guangdong 2020-01-30 Guangdong Provincial Institution of Public Health, Guangdong Provinical Center for Disease Control and Prevention Guangdong Provincial Institution of Public Health Jing Lu, Louis du Plessis, Liu Zhe, Jiufeng Sun, Sarah François, Huifang Lin, Moritz Kraemer, Jingju Peng, Qianlin Xiong, Runyu Yuan, Lilian Zeng, Pingping Zhou, Chuming Liang, Tao Liu, Wei Li, Juan Su, Huanying Zheng, Kang Min, Song Tie, Bo Peng, Shisong Fang, Wenzhe Su, Kuibiao Li, Ruilin Sun, Ru bai, Xi Tang, Minfeng Liang, Nuno Faria, Josh Quick, Andrew Rambaut, Verity Hill, Wenjun Ma, Nick Loman, Oliver Pybus, Changwen Ke

EPI_ISL_413854 hCoV-19/Guangdong/2020XN4475-P0042/2020 Asia / China / Guangdong 2020-01-30
Guangdong Provincial Institution of Public Health, Guangdong Provinical Center for Disease Control and Prevention Guangdong Provincial Institution of Public Health Jing Lu, Louis du Plessis, Liu Zhe, Jiufeng Sun, Sarah François, Huifang Lin, Moritz Kraemer, Jingju Peng, Qianlin Xiong, Runyu Yuan, Lilian Zeng, Pingping Zhou, Chuming Liang, Tao Liu, Wei Li, Juan Su, Huanying Zheng, Kang Min, Song Tie, Bo Peng, Shisong Fang, Wenzhe Su, Kuibiao Li, Ruilin Sun, Ru bai, Xi Tang, Minfeng Liang, Nuno Faria, Josh Quick, Andrew Rambaut, Verity Hill, Wenjun Ma, Nick Loman, Oliver Pybus, Changwen Ke
EPI_ISL_413855 hCoV-19/Guangdong/GD2020234-P0023/2020 Asia / China / Guangdong 2020-02-07
Guangdong Provincial Institution of Public Health, Guangdong Provinical Center for Disease Control and Prevention Guangdong Provincial Institution of Public Health Jing Lu, Louis du Plessis, Liu Zhe, Jiufeng Sun, Sarah François, Huifang Lin, Moritz Kraemer, Jingju Peng, Qianlin Xiong, Runyu Yuan, Lilian Zeng, Pingping Zhou, Chuming Liang, Tao Liu, Wei Li, Juan Su, Huanying Zheng, Kang Min, Song Tie, Bo Peng, Shisong Fang, Wenzhe Su, Kuibiao Li, Ruilin Sun, Ru bai, Xi Tang, Minfeng Liang, Nuno Faria, Josh Quick, Andrew Rambaut, Verity Hill, Wenjun Ma, Nick Loman, Oliver Pybus, Changwen Ke
EPI_ISL_413856 hCoV-19/Guangdong/GD2020233-P0027/2020 Asia / China / Guangdong 2020-02-07
Guangdong Provincial Institution of Public Health, Guangdong Provinical Center for Disease Control and Prevention Guangdong Provincial Institution of Public Health Jing Lu, Louis du Plessis, Liu Zhe, Jiufeng Sun, Sarah François, Huifang Lin, Moritz Kraemer, Jingju Peng, Qianlin Xiong, Runyu Yuan, Lilian Zeng, Pingping Zhou, Chuming Liang, Tao Liu, Wei Li, Juan Su, Huanying Zheng, Kang Min, Song Tie, Bo Peng, Shisong Fang, Wenzhe Su, Kuibiao Li, Ruilin Sun, Ru bai, Xi Tang, Minfeng Liang, Nuno Faria, Josh Quick, Andrew Rambaut, Verity Hill, Wenjun Ma, Nick Loman, Oliver Pybus, Changwen Ke
EPI_ISL_413857 hCoV-19/Guangdong/2020XN4448-P0002/2020 Asia / China / Guangdong 2020-01-31
Guangdong Provincial Institution of Public Health, Guangdong Provinical Center for Disease Control and Prevention Guangdong Provincial Institution of Public Health Jing Lu, Louis du Plessis, Liu Zhe, Jiufeng Sun, Sarah François, Huifang Lin, Moritz Kraemer, Jingju Peng, Qianlin Xiong, Runyu Yuan, Lilian Zeng, Pingping Zhou, Chuming Liang, Tao Liu, Wei Li, Juan Su, Huanying Zheng, Kang Min, Song Tie, Bo Peng, Shisong Fang, Wenzhe Su, Kuibiao Li, Ruilin Sun, Ru bai, Xi Tang, Minfeng Liang, Nuno Faria, Josh Quick, Andrew Rambaut, Verity Hill, Wenjun Ma, Nick Loman, Oliver Pybus, Changwen Ke
EPI_ISL_413858 hCoV-19/Guangdong/2020XN4459-P0041/2020 Asia / China / Guangdong 2020-01-30
Guangdong Provincial Institution of Public Health, Guangdong Provinical Center for Disease Control and Prevention Guangdong Provincial Institution of Public Health Jing Lu, Louis du Plessis, Liu Zhe, Jiufeng Sun, Sarah François, Huifang Lin, Moritz Kraemer, Jingju Peng, Qianlin Xiong, Runyu Yuan, Lilian Zeng, Pingping Zhou, Chuming Liang, Tao Liu, Wei Li, Juan Su, Huanying Zheng, Kang Min, Song Tie, Bo Peng, Shisong Fang, Wenzhe Su, Kuibiao Li, Ruilin Sun, Ru bai, Xi Tang, Minfeng Liang, Nuno Faria, Josh Quick, Andrew Rambaut, Verity Hill, Wenjun Ma, Nick Loman, Oliver Pybus, Changwen Ke
EPI_ISL_413860 hCoV-19/Guangdong/2020XN4273-P0036/2020 Asia / China / Guangdong 2020-01-30
Guangdong Provincial Institution of Public Health, Guangdong Provinical Center for Disease Control and Prevention Guangdong Provincial Institution of Public Health Jing Lu, Louis du Plessis, Liu Zhe, Jiufeng Sun, Sarah François, Huifang Lin, Moritz Kraemer, Jingju Peng, Qianlin Xiong, Runyu Yuan, Lilian Zeng, Pingping Zhou, Chuming Liang, Tao Liu, Wei Li, Juan Su, Huanying Zheng, Kang Min, Song Tie, Bo Peng, Shisong Fang, Wenzhe Su, Kuibiao Li, Ruilin Sun, Ru bai, Xi Tang, Minfeng Liang, Nuno Faria, Josh Quick, Andrew Rambaut, Verity Hill, Wenjun Ma, Nick Loman, Oliver Pybus, Changwen Ke
EPI_ISL_413861 hCoV-19/Guangdong/GD2020080-P0010/2020 Asia / China / Guangdong 2020-02-01
Guangdong Provincial Institution of Public Health, Guangdong Provinical Center for Disease Control and Prevention Guangdong Provincial Institution of Public Health Jing Lu, Louis du Plessis, Liu Zhe, Jiufeng Sun, Sarah François, Huifang Lin, Moritz Kraemer, Jingju Peng, Qianlin Xiong, Runyu Yuan, Lilian Zeng, Pingping Zhou, Chuming Liang, Tao Liu, Wei Li, Juan Su, Huanying Zheng, Kang Min, Song Tie, Bo Peng, Shisong Fang, Wenzhe Su, Kuibiao Li, Ruilin Sun, Ru bai, Xi Tang, Minfeng Liang, Nuno Faria, Josh Quick, Andrew Rambaut, Verity Hill, Wenjun Ma, Nick Loman, Oliver Pybus, Changwen Ke
EPI_ISL_413862 hCoV-19/Guangdong/GD2020086-P0021/2020 Asia / China / Guangdong 2020-02-01
Guangdong Provincial Institution of Public Health, Guangdong Provinical Center for Disease Control and Prevention Guangdong Provincial Institution of Public Health Jing Lu, Louis du Plessis, Liu Zhe, Jiufeng Sun, Sarah François, Huifang Lin, Moritz Kraemer, Jingju Peng, Qianlin Xiong, Runyu Yuan, Lilian Zeng, Pingping Zhou, Chuming Liang, Tao Liu, Wei Li, Juan Su, Huanying Zheng, Kang Min, Song Tie, Bo Peng, Shisong Fang, Wenzhe Su, Kuibiao Li, Ruilin Sun, Ru bai, Xi Tang, Minfeng Liang, Nuno Faria, Josh Quick, Andrew Rambaut, Verity Hill, Wenjun Ma, Nick Loman, Oliver Pybus, Changwen Ke
EPI_ISL_413863 hCoV-19/Guangdong/GD2020087-P0008/2020 Asia / China / Guangdong 2020-02-01
Guangdong Provincial Institution of Public Health, Guangdong Provinical Center for Disease Control and Prevention Guangdong Provincial Institution of Public Health Jing Lu, Louis du Plessis, Liu Zhe, Jiufeng Sun, Sarah François, Huifang Lin, Moritz Kraemer, Jingju Peng, Qianlin Xiong, Runyu Yuan, Lilian Zeng, Pingping Zhou, Chuming Liang, Tao Liu, Wei Li, Juan Su, Huanying Zheng, Kang Min, Song Tie, Bo Peng, Shisong Fang, Wenzhe Su, Kuibiao Li, Ruilin Sun, Ru bai, Xi Tang, Minfeng Liang, Nuno Faria, Josh Quick, Andrew Rambaut, Verity Hill, Wenjun Ma, Nick Loman, Oliver Pybus, Changwen Ke
EPI_ISL_413864 hCoV-19/Guangdong/GD2020246-P0028/2020 Asia / China / Guangdong 2020-02-09
Guangdong Provincial Institution of Public Health, Guangdong Provinical Center for Disease Control and Prevention Guangdong Provincial Institution of Public Health Jing Lu, Louis du Plessis, Liu Zhe, Jiufeng Sun, Sarah François, Huifang Lin, Moritz Kraemer, Jingju Peng, Qianlin Xiong, Runyu Yuan, Lilian Zeng, Pingping Zhou, Chuming Liang, Tao Liu, Wei Li, Juan Su, Huanying Zheng, Kang Min, Song Tie, Bo Peng, Shisong Fang, Wenzhe Su, Kuibiao Li, Ruilin Sun, Ru bai, Xi Tang, Minfeng Liang, Nuno Faria, Josh Quick, Andrew Rambaut, Verity Hill, Wenjun Ma, Nick Loman, Oliver Pybus, Changwen Ke

EPI_ISL_413867 hCoV-19/Guangdong/GDSZ202015-P0019/2020 Asia / China / Guangdong 2020-02-05 Guangdong Provincial Institution of Public Health, Guangdong Provinical Center for Disease Control and Prevention Guangdong Provincial Institution of Public Health Jing Lu, Louis du Plessis, Liu Zhe, Jiufeng Sun, Sarah François, Huifang Lin, Moritz Kraemer, Jingju Peng, Qianlin Xiong, Runyu Yuan, Lilian Zeng, Pingping Zhou, Chuming Liang, Tao Liu, Wei Li, Juan Su, Huanying Zheng, Kang Min, Song Tie, Bo Peng, Shisong Fang, Wenzhe Su, Kuibiao Li, Ruilin Sun, Ru bai, Xi Tang, Minfeng Liang, Nuno Faria, Josh Quick, Andrew Rambaut, Verity Hill, Wenjun Ma, Nick Loman, Oliver Pybus, Changwen Ke

EPI_ISL_414005 hCoV-19/England/200940527/2020 Europe / United Kingdom / England 2020-02-25 Respiratory Virus Unit, Microbiology Services Colindale, Public Health England Respiratory Virus Unit, Microbiology Services Colindale, Public Health England Monica Galiano, Shahjahan Miah, Angie Lackenby, Omolola Akinbami, Tiina Talts, Leena Bhaw, Richard Myers, Steven Platt, Kirstin Edwards, Jonathan Hubb, Joanna Ellis, Maria Zambon

EPI_ISL_414006 hCoV-19/England/200990724/2020 Europe / United Kingdom / England 2020-02-28 Respiratory Virus Unit, Microbiology Services Colindale, Public Health England Respiratory Virus Unit, Microbiology Services Colindale, Public Health England Monica Galiano, Shahjahan Miah, Angie Lackenby, Omolola Akinbami, Tiina Talts, Leena Bhaw, Richard Myers, Steven Platt, Kirstin Edwards, Jonathan Hubb, Joanna Ellis, Maria Zambon

EPI_ISL_414007 hCoV-19/England/200990725/2020 Europe / United Kingdom / England 2020-02-28 Respiratory Virus Unit, Microbiology Services Colindale, Public Health England Respiratory Virus Unit, Microbiology Services Colindale, Public Health England Monica Galiano, Shahjahan Miah, Angie Lackenby, Omolola Akinbami, Tiina Talts, Leena Bhaw, Richard Myers, Steven Platt, Kirstin Edwards, Jonathan Hubb, Joanna Ellis, Maria Zambon

EPI_ISL_414008 hCoV-19/England/200960041/2020 Europe / United Kingdom / England 2020-02-27 Respiratory Virus Unit, Microbiology Services Colindale, Public Health England Respiratory Virus Unit, Microbiology Services Colindale, Public Health England Monica Galiano, Shahjahan Miah, Angie Lackenby, Omolola Akinbami, Tiina Talts, Leena Bhaw, Richard Myers, Steven Platt, Kirstin Edwards, Jonathan Hubb, Joanna Ellis, Maria Zambon

EPI_ISL_414009 hCoV-19/England/200960515/2020 Europe / United Kingdom / England 2020-02-25 Respiratory Virus Unit, Microbiology Services Colindale, Public Health England Respiratory Virus Unit, Microbiology Services Colindale, Public Health England Monica Galiano, Shahjahan Miah, Angie Lackenby, Omolola Akinbami, Tiina Talts, Leena Bhaw, Richard Myers, Steven Platt, Kirstin Edwards, Jonathan Hubb, Joanna Ellis, Maria Zambon

EPI_ISL_414010 hCoV-19/England/200981386/2020 Europe / United Kingdom / England 2020-02-26 Respiratory Virus Unit, Microbiology Services Colindale, Public Health England Respiratory Virus Unit, Microbiology Services Colindale, Public Health England Monica Galiano, Shahjahan Miah, Angie Lackenby, Omolola Akinbami, Tiina Talts, Leena Bhaw, Richard Myers, Steven Platt, Kirstin Edwards, Jonathan Hubb, Joanna Ellis, Maria Zambon

EPI_ISL_414011 hCoV-19/England/200990006/2020 Europe / United Kingdom / England 2020-02-26 Respiratory Virus Unit, Microbiology Services Colindale, Public Health England Respiratory Virus Unit, Microbiology Services Colindale, Public Health England Monica Galiano, Shahjahan Miah, Angie Lackenby, Omolola Akinbami, Tiina Talts, Leena Bhaw, Richard Myers, Steven Platt, Kirstin Edwards, Jonathan Hubb, Joanna Ellis, Maria Zambon

EPI_ISL_414012 hCoV-19/England/200990723/2020 Europe / United Kingdom / England 2020-02-27 Respiratory Virus Unit, Microbiology Services Colindale, Public Health England Respiratory Virus Unit, Microbiology Services Colindale, Public Health England Monica Galiano, Shahjahan Miah, Angie Lackenby, Omolola Akinbami, Tiina Talts, Leena Bhaw, Richard Myers, Steven Platt, Kirstin Edwards, Jonathan Hubb, Joanna Ellis, Maria Zambon

EPI_ISL_414013 hCoV-19/England/201000003/2020 Europe / United Kingdom / England 2020-03-01 Respiratory Virus Unit, Microbiology Services Colindale, Public Health England Respiratory Virus Unit, Microbiology Services Colindale, Public Health England Monica Galiano, Shahjahan Miah, Angie Lackenby, Omolola Akinbami, Tiina Talts, Leena Bhaw, Richard Myers, Steven Platt, Kirstin Edwards, Jonathan Hubb, Joanna Ellis, Maria Zambon

EPI_ISL_414014 hCoV-19/Brazil/SPBR-03/2020 South America / Brazil / Sao Paulo 2020-03-02 Hospital Israelita Albert Einstein Instituto Adolfo Lutz, Interdiciplinary Procedures Center, Strategic Laboratory Claudio Tavares Sacchi, Claudia Regina Gonçalves, Katia Correia dos Santos, Carlos Henrique Camargo, Maria do Carmo Sampaito Tavares Timenetsky, Terezinha Maria de Paiva, Ester Cerdeira Sabino

EPI_ISL_414015 hCoV-19/Brazil/SPBR-06/2020 South America / Brazil / Sao Paulo / Sao Paulo 2020-02-29 Hospital São Joaquim Beneficencia Portuguesa Instituto Adolfo Lutz, Interdiciplinary Procedures Center, Strategic Laboratory Claudio Tavares Sacchi, Claudia Regina Gonçalves, SimoneGuadagnucci Morillo, Carlos Henrique Camargo, Maria do Carmo Sampaito Tavares Timenetsky, Fabiana Cristina Pereira dos Santos Terezinha Maria de Paiva, Ester Cerdeira Sabino

EPI_ISL_414016 hCoV-19/Brazil/SPBR-05/2020 South America / Brazil / Sao Paulo / Sao Paulo 2020-02-29 Hospital São Joaquim Beneficencia Portuguesa Instituto Adolfo Lutz, Interdiciplinary Procedures Center, Strategic Laboratory Claudio Tavares Sacchi, Claudia Regina Gonçalves, Audrey Cilli, Carlos Henrique Camargo, Maria do Carmo Sampaito Tavares Timenetsky, Daniela Bernardes Borges da Silva, Terezinha Maria de Paiva, Ester Cerdeira Sabino

EPI_ISL_414017 hCoV-19/Brazil/SPBR-04/2020 South America / Brazil / Sao Paulo 2020-03-04 Hospital São Joaquim Beneficencia Portuguesa Instituto Adolfo Lutz, Interdiciplinary Procedures Center, Strategic Laboratory Claudio Tavares Sacchi, Claudia Regina Gonçalves, Fabiana Cristina Pereira dos Santos, Carlos Henrique Camargo, Maria do Carmo Sampaito Tavares Timenetsky, Daniela Bernardes Borges da Silva,

Terezinha Maria de Paiva, Ester Cerdeira Sabino
EPI_ISL_414019 hCoV-19/Switzerland/GE3121/2020 Europe / Switzerland / Geneva 2020-02-27 Laboratoire de Virologie, HUG Swiss National Reference Centre for Influenza LAUBSCHER Florian et al.
EPI_ISL_414020 hCoV-19/Switzerland/GE5373/2020 Europe / Switzerland / Geneva 2020-02-27 Laboratoire de Virologie, HUG Swiss National Reference Centre for Influenza LAUBSCHER Florian et al.
EPI_ISL_414021 hCoV-19/Switzerland/BL0902/2020 Europe / Switzerland / Basel 2020-02-27 Laboratoire de Virologie, HUG Swiss National Reference Centre for Influenza LAUBSCHER Florian et al.
EPI_ISL_414022 hCoV-19/Switzerland/GE9586/2020 Europe / Switzerland / Geneva 2020-02-27 Laboratoire de Virologie, HUG Swiss National Reference Centre for Influenza LAUBSCHER Florian et al.
EPI_ISL_414023 hCoV-19/Switzerland/VD5615/2020 Europe / Switzerland / Vaud 2020-03-01 Laboratoire de Virologie, HUG Swiss National Reference Centre for Influenza LAUBSCHER Florian et al.
EPI_ISL_414027 hCoV-19/Scotland/CVR05/2020 Europe / United Kingdom / Scotland 2020-03-04 West of Scotland Specialist Virology Centre, NHSGGC MRC-University of Glasgow Centre for Virus Research Emma Thomson, Antonia Ho; Kathy Smollett, Daniel Mair, Stephen Carmichael, Ana da Silva Filipe; Richard Orton, David L Robertson; Alasdair MacLean, Rory Gunson.
EPI_ISL_414040 hCoV-19/England/200641094/2020 Europe / United Kingdom / England 2020-02-05 Respiratory Virus Unit, Microbiology Services Colindale, Public Health England Respiratory Virus Unit, Microbiology Services Colindale, Public Health England Monica Galiano, Shahjahan Miah, Angie Lackenby, Omolola Akinbami, Tiina Talts, Leena Bhaw, Richard Myers, Steven Platt, Kirstin Edwards, Jonathan Hubb, Joanna Ellis, Maria Zambon
EPI_ISL_414041 hCoV-19/England/200690245/2020 Europe / United Kingdom / England 2020-02-08 Respiratory Virus Unit, Microbiology Services Colindale, Public Health England Respiratory Virus Unit, Microbiology Services Colindale, Public Health England Monica Galiano, Shahjahan Miah, Angie Lackenby, Omolola Akinbami, Tiina Talts, Leena Bhaw, Richard Myers, Steven Platt, Kirstin Edwards, Jonathan Hubb, Joanna Ellis, Maria Zambon
EPI_ISL_414042 hCoV-19/England/200690300/2020 Europe / United Kingdom / England 2020-02-08 Respiratory Virus Unit, Microbiology Services Colindale, Public Health England Respiratory Virus Unit, Microbiology Services Colindale, Public Health England Monica Galiano, Shahjahan Miah, Angie Lackenby, Omolola Akinbami, Tiina Talts, Leena Bhaw, Richard Myers, Steven Platt, Kirstin Edwards, Jonathan Hubb, Joanna Ellis, Maria Zambon
EPI_ISL_414043 hCoV-19/England/200690306/2020 Europe / United Kingdom / England 2020-02-07 Respiratory Virus Unit, Microbiology Services Colindale, Public Health England Respiratory Virus Unit, Microbiology Services Colindale, Public Health England Monica Galiano, Shahjahan Miah, Angie Lackenby, Omolola Akinbami, Tiina Talts, Leena Bhaw, Richard Myers, Steven Platt, Kirstin Edwards, Jonathan Hubb, Joanna Ellis, Maria Zambon
EPI_ISL_414044 hCoV-19/England/200690756/2020 Europe / United Kingdom / England 2020-02-08 Respiratory Virus Unit, Microbiology Services Colindale, Public Health England Respiratory Virus Unit, Microbiology Services Colindale, Public Health England Monica Galiano, Shahjahan Miah, Angie Lackenby, Omolola Akinbami, Tiina Talts, Leena Bhaw, Richard Myers, Steven Platt, Kirstin Edwards, Jonathan Hubb, Joanna Ellis, Maria Zambon
EPI_ISL_414045 hCoV-19/Brazil/RJ-314/2020 South America / Brazil / Rio de Janeiro / Rio de Janeiro 2020-03-04 LACEN RJ - Laboratório Central de Saúde Pública Noel Nutels Instituto Oswaldo Cruz FIOCRUZ - Laboratory of Respiratory Viruses and Measles (LVRS) Paola Resende, Alisson Fabri, Joilson Xavier, Sunando Roy, Fernando Motta, Aline Mattos, Milene Miranda, Cristiana Garcia, Braulia Caetano, Maria Ogrzewalska, Jonathan Lopes, Luciana Appolinario, Maria Nóbrega, Marilda Siqueira
EPI_ISL_416600 hCoV-19/Japan/DP0438/2020 Asia / Japan / unknown 2020-02-16 Japanese Quarantine Stations Pathogen Genomics Center, National Institute of Infectious Diseases Tsuyoshi Sekizuka, Kentaro Itokawa, Rina Tanaka, Masanori Hashino, Tsutomu Kageyama, Shinji Saito, Ikuyo Takayama, Hideki Hasegawa, Takuri Takahashi, Hajime Kamiya, Takuya Yamagishi, Motoi Suzuki, Takaji Wakita, Makoto Kuroda
EPI_ISL_416601 hCoV-19/Japan/DP0457/2020 Asia / Japan / unknown 2020-02-16 Japanese Quarantine Stations Pathogen Genomics Center, National Institute of Infectious Diseases Tsuyoshi Sekizuka, Kentaro Itokawa, Rina Tanaka, Masanori Hashino, Tsutomu Kageyama, Shinji Saito, Ikuyo Takayama, Hideki Hasegawa, Takuri Takahashi, Hajime Kamiya, Takuya Yamagishi, Motoi Suzuki, Takaji Wakita, Makoto Kuroda
EPI_ISL_416602 hCoV-19/Japan/DP0462/2020 Asia / Japan / unknown 2020-02-16 Japanese Quarantine Stations Pathogen Genomics Center, National Institute of Infectious Diseases Tsuyoshi Sekizuka, Kentaro Itokawa, Rina Tanaka, Masanori Hashino, Tsutomu Kageyama, Shinji Saito, Ikuyo Takayama, Hideki Hasegawa, Takuri Takahashi, Hajime Kamiya, Takuya Yamagishi, Motoi Suzuki, Takaji Wakita, Makoto Kuroda
EPI_ISL_416603 hCoV-19/Japan/DP0464/2020 Asia / Japan / unknown 2020-02-16 Japanese Quarantine Stations Pathogen Genomics Center, National Institute of Infectious Diseases Tsuyoshi Sekizuka, Kentaro Itokawa, Rina Tanaka, Masanori Hashino, Tsutomu Kageyama, Shinji Saito, Ikuyo Takayama, Hideki Hasegawa, Takuri Takahashi, Hajime Kamiya, Takuya Yamagishi, Motoi Suzuki, Takaji Wakita, Makoto Kuroda
EPI_ISL_416604 hCoV-19/Japan/DP0476/2020 Asia / Japan / unknown 2020-02-16 Japanese Quarantine Stations Pathogen Genomics Center, National Institute of Infectious Diseases Tsuyoshi Sekizuka, Kentaro Itokawa, Rina Tanaka, Masanori Hashino, Tsutomu Kageyama, Shinji Saito, Ikuyo Takayama, Hideki Hasegawa, Takuri Takahashi, Hajime Kamiya, Takuya Yamagishi, Motoi Suzuki, Takaji Wakita, Makoto Kuroda
EPI_ISL_416605 hCoV-19/Japan/DP0481/2020 Asia / Japan / unknown 2020-02-16 Japanese Quarantine Stations Pathogen Genomics Center, National Institute of Infectious Diseases Tsuyoshi Sekizuka, Kentaro Itokawa, Rina Tanaka, Masanori Hashino, Tsutomu Kageyama, Shinji Saito, Ikuyo Takayama, Hideki Hasegawa, Takuri Takahashi, Hajime Kamiya, Takuya Yamagishi, Motoi Suzuki, Takaji Wakita, Makoto Kuroda
EPI_ISL_416606 hCoV-19/Japan/DP0482/2020 Asia / Japan / unknown 2020-02-16 Japanese Quarantine

Stations	Pathogen Genomics Center, National Institute of Infectious Diseases	Tsuyoshi Sekizuka, Kentaro Itokawa, Rina Tanaka, Masanori Hashino, Tsutomu Kageyama, Shinji Saito, Ikuyo Takayama, Hideki Hasegawa, Takuri Takahashi, Hajime Kamiya, Takuya Yamagishi, Motoi Suzuki, Takaji Wakita, Makoto Kuroda
EPI_ISL_416607	hCoV-19/Japan/DP0543/2020	Asia / Japan / unknown	2020-02-17	Japanese Quarantine Stations	Pathogen Genomics Center, National Institute of Infectious Diseases	Tsuyoshi Sekizuka, Kentaro Itokawa, Rina Tanaka, Masanori Hashino, Tsutomu Kageyama, Shinji Saito, Ikuyo Takayama, Hideki Hasegawa, Takuri Takahashi, Hajime Kamiya, Takuya Yamagishi, Motoi Suzuki, Takaji Wakita, Makoto Kuroda
EPI_ISL_416608	hCoV-19/Japan/DP0544/2020	Asia / Japan / unknown	2020-02-17	Japanese Quarantine Stations	Pathogen Genomics Center, National Institute of Infectious Diseases	Tsuyoshi Sekizuka, Kentaro Itokawa, Rina Tanaka, Masanori Hashino, Tsutomu Kageyama, Shinji Saito, Ikuyo Takayama, Hideki Hasegawa, Takuri Takahashi, Hajime Kamiya, Takuya Yamagishi, Motoi Suzuki, Takaji Wakita, Makoto Kuroda
EPI_ISL_416609	hCoV-19/Japan/DP0568/2020	Asia / Japan / unknown	2020-02-17	Japanese Quarantine Stations	Pathogen Genomics Center, National Institute of Infectious Diseases	Tsuyoshi Sekizuka, Kentaro Itokawa, Rina Tanaka, Masanori Hashino, Tsutomu Kageyama, Shinji Saito, Ikuyo Takayama, Hideki Hasegawa, Takuri Takahashi, Hajime Kamiya, Takuya Yamagishi, Motoi Suzuki, Takaji Wakita, Makoto Kuroda
EPI_ISL_416610	hCoV-19/Japan/DP0588/2020	Asia / Japan / unknown	2020-02-17	Japanese Quarantine Stations	Pathogen Genomics Center, National Institute of Infectious Diseases	Tsuyoshi Sekizuka, Kentaro Itokawa, Rina Tanaka, Masanori Hashino, Tsutomu Kageyama, Shinji Saito, Ikuyo Takayama, Hideki Hasegawa, Takuri Takahashi, Hajime Kamiya, Takuya Yamagishi, Motoi Suzuki, Takaji Wakita, Makoto Kuroda
EPI_ISL_416611	hCoV-19/Japan/DP0644/2020	Asia / Japan / unknown	2020-02-17	Japanese Quarantine Stations	Pathogen Genomics Center, National Institute of Infectious Diseases	Tsuyoshi Sekizuka, Kentaro Itokawa, Rina Tanaka, Masanori Hashino, Tsutomu Kageyama, Shinji Saito, Ikuyo Takayama, Hideki Hasegawa, Takuri Takahashi, Hajime Kamiya, Takuya Yamagishi, Motoi Suzuki, Takaji Wakita, Makoto Kuroda
EPI_ISL_416612	hCoV-19/Japan/DP0645/2020	Asia / Japan / unknown	2020-02-17	Japanese Quarantine Stations	Pathogen Genomics Center, National Institute of Infectious Diseases	Tsuyoshi Sekizuka, Kentaro Itokawa, Rina Tanaka, Masanori Hashino, Tsutomu Kageyama, Shinji Saito, Ikuyo Takayama, Hideki Hasegawa, Takuri Takahashi, Hajime Kamiya, Takuya Yamagishi, Motoi Suzuki, Takaji Wakita, Makoto Kuroda
EPI_ISL_416613	hCoV-19/Japan/DP0654/2020	Asia / Japan / unknown	2020-02-17	Japanese Quarantine Stations	Pathogen Genomics Center, National Institute of Infectious Diseases	Tsuyoshi Sekizuka, Kentaro Itokawa, Rina Tanaka, Masanori Hashino, Tsutomu Kageyama, Shinji Saito, Ikuyo Takayama, Hideki Hasegawa, Takuri Takahashi, Hajime Kamiya, Takuya Yamagishi, Motoi Suzuki, Takaji Wakita, Makoto Kuroda
EPI_ISL_416614	hCoV-19/Japan/DP0687/2020	Asia / Japan / unknown	2020-02-17	Japanese Quarantine Stations	Pathogen Genomics Center, National Institute of Infectious Diseases	Tsuyoshi Sekizuka, Kentaro Itokawa, Rina Tanaka, Masanori Hashino, Tsutomu Kageyama, Shinji Saito, Ikuyo Takayama, Hideki Hasegawa, Takuri Takahashi, Hajime Kamiya, Takuya Yamagishi, Motoi Suzuki, Takaji Wakita, Makoto Kuroda
EPI_ISL_416615	hCoV-19/Japan/DP0690/2020	Asia / Japan / unknown	2020-02-17	Japanese Quarantine Stations	Pathogen Genomics Center, National Institute of Infectious Diseases	Tsuyoshi Sekizuka, Kentaro Itokawa, Rina Tanaka, Masanori Hashino, Tsutomu Kageyama, Shinji Saito, Ikuyo Takayama, Hideki Hasegawa, Takuri Takahashi, Hajime Kamiya, Takuya Yamagishi, Motoi Suzuki, Takaji Wakita, Makoto Kuroda
EPI_ISL_416617	hCoV-19/Japan/DP0699/2020	Asia / Japan / unknown	2020-02-17	Japanese Quarantine Stations	Pathogen Genomics Center, National Institute of Infectious Diseases	Tsuyoshi Sekizuka, Kentaro Itokawa, Rina Tanaka, Masanori Hashino, Tsutomu Kageyama, Shinji Saito, Ikuyo Takayama, Hideki Hasegawa, Takuri Takahashi, Hajime Kamiya, Takuya Yamagishi, Motoi Suzuki, Takaji Wakita, Makoto Kuroda
EPI_ISL_416618	hCoV-19/Japan/DP0700/2020	Asia / Japan / unknown	2020-02-17	Japanese Quarantine Stations	Pathogen Genomics Center, National Institute of Infectious Diseases	Tsuyoshi Sekizuka, Kentaro Itokawa, Rina Tanaka, Masanori Hashino, Tsutomu Kageyama, Shinji Saito, Ikuyo Takayama, Hideki Hasegawa, Takuri Takahashi, Hajime Kamiya, Takuya Yamagishi, Motoi Suzuki, Takaji Wakita, Makoto Kuroda
EPI_ISL_416619	hCoV-19/Japan/DP0703/2020	Asia / Japan / unknown	2020-02-17	Japanese Quarantine Stations	Pathogen Genomics Center, National Institute of Infectious Diseases	Tsuyoshi Sekizuka, Kentaro Itokawa, Rina Tanaka, Masanori Hashino, Tsutomu Kageyama, Shinji Saito, Ikuyo Takayama, Hideki Hasegawa, Takuri Takahashi, Hajime Kamiya, Takuya Yamagishi, Motoi Suzuki, Takaji Wakita, Makoto Kuroda
EPI_ISL_416620	hCoV-19/Japan/DP0724/2020	Asia / Japan / unknown	2020-02-17	Japanese Quarantine Stations	Pathogen Genomics Center, National Institute of Infectious Diseases	Tsuyoshi Sekizuka, Kentaro Itokawa, Rina Tanaka, Masanori Hashino, Tsutomu Kageyama, Shinji Saito, Ikuyo Takayama, Hideki Hasegawa, Takuri Takahashi, Hajime Kamiya, Takuya Yamagishi, Motoi Suzuki, Takaji Wakita, Makoto Kuroda
EPI_ISL_416621	hCoV-19/Japan/DP0743/2020	Asia / Japan / unknown	2020-02-17	Japanese Quarantine Stations	Pathogen Genomics Center, National Institute of Infectious Diseases	Tsuyoshi Sekizuka, Kentaro Itokawa, Rina Tanaka, Masanori Hashino, Tsutomu Kageyama, Shinji Saito, Ikuyo Takayama, Hideki Hasegawa, Takuri Takahashi, Hajime Kamiya, Takuya Yamagishi, Motoi Suzuki, Takaji Wakita, Makoto Kuroda
EPI_ISL_416622	hCoV-19/Japan/DP0752/2020	Asia / Japan / unknown	2020-02-17	Japanese Quarantine Stations	Pathogen Genomics Center, National Institute of Infectious Diseases	Tsuyoshi Sekizuka, Kentaro Itokawa, Rina Tanaka, Masanori Hashino, Tsutomu Kageyama, Shinji Saito, Ikuyo Takayama, Hideki Hasegawa, Takuri Takahashi, Hajime Kamiya, Takuya Yamagishi, Motoi Suzuki, Takaji Wakita, Makoto Kuroda
EPI_ISL_416624	hCoV-19/Japan/DP0764/2020	Asia / Japan / unknown	2020-02-17	Japanese Quarantine Stations	Pathogen Genomics Center, National Institute of Infectious Diseases	Tsuyoshi Sekizuka, Kentaro Itokawa, Rina Tanaka, Masanori Hashino, Tsutomu Kageyama, Shinji Saito, Ikuyo Takayama, Hideki Hasegawa, Takuri Takahashi, Hajime Kamiya, Takuya Yamagishi, Motoi Suzuki, Takaji Wakita, Makoto Kuroda
EPI_ISL_416625	hCoV-19/Japan/DP0765/2020	Asia / Japan / unknown	2020-02-17	Japanese Quarantine Stations	Pathogen Genomics Center, National Institute of Infectious Diseases	Tsuyoshi Sekizuka, Kentaro Itokawa, Rina Tanaka, Masanori Hashino, Tsutomu Kageyama, Shinji Saito, Ikuyo Takayama, Hideki

Hasegawa, Takuri Takahashi, Hajime Kamiya, Takuya Yamagishi, Motoi Suzuki, Takaji Wakita, Makoto Kuroda
EPI_ISL_416626 hCoV-19/Japan/DP0779/2020 Asia / Japan / unknown 2020-02-17 Japanese Quarantine Stations Pathogen Genomics Center, National Institute of Infectious Diseases Tsuyoshi Sekizuka, Kentaro Itokawa, Rina Tanaka, Masanori Hashino, Tsutomu Kageyama, Shinji Saito, Ikuyo Takayama, Hideki Hasegawa, Takuri Takahashi, Hajime Kamiya, Takuya Yamagishi, Motoi Suzuki, Takaji Wakita, Makoto Kuroda
EPI_ISL_416627 hCoV-19/Japan/DP0785/2020 Asia / Japan / unknown 2020-02-17 Japanese Quarantine Stations Pathogen Genomics Center, National Institute of Infectious Diseases Tsuyoshi Sekizuka, Kentaro Itokawa, Rina Tanaka, Masanori Hashino, Tsutomu Kageyama, Shinji Saito, Ikuyo Takayama, Hideki Hasegawa, Takuri Takahashi, Hajime Kamiya, Takuya Yamagishi, Motoi Suzuki, Takaji Wakita, Makoto Kuroda
EPI_ISL_416628 hCoV-19/Japan/DP0786/2020 Asia / Japan / unknown 2020-02-17 Japanese Quarantine Stations Pathogen Genomics Center, National Institute of Infectious Diseases Tsuyoshi Sekizuka, Kentaro Itokawa, Rina Tanaka, Masanori Hashino, Tsutomu Kageyama, Shinji Saito, Ikuyo Takayama, Hideki Hasegawa, Takuri Takahashi, Hajime Kamiya, Takuya Yamagishi, Motoi Suzuki, Takaji Wakita, Makoto Kuroda
EPI_ISL_416629 hCoV-19/Japan/DP0802/2020 Asia / Japan / unknown 2020-02-17 Japanese Quarantine Stations Pathogen Genomics Center, National Institute of Infectious Diseases Tsuyoshi Sekizuka, Kentaro Itokawa, Rina Tanaka, Masanori Hashino, Tsutomu Kageyama, Shinji Saito, Ikuyo Takayama, Hideki Hasegawa, Takuri Takahashi, Hajime Kamiya, Takuya Yamagishi, Motoi Suzuki, Takaji Wakita, Makoto Kuroda
EPI_ISL_416630 hCoV-19/Japan/DP0803/2020 Asia / Japan / unknown 2020-02-17 Japanese Quarantine Stations Pathogen Genomics Center, National Institute of Infectious Diseases Tsuyoshi Sekizuka, Kentaro Itokawa, Rina Tanaka, Masanori Hashino, Tsutomu Kageyama, Shinji Saito, Ikuyo Takayama, Hideki Hasegawa, Takuri Takahashi, Hajime Kamiya, Takuya Yamagishi, Motoi Suzuki, Takaji Wakita, Makoto Kuroda
EPI_ISL_416631 hCoV-19/Japan/DP0804/2020 Asia / Japan / unknown 2020-02-17 Japanese Quarantine Stations Pathogen Genomics Center, National Institute of Infectious Diseases Tsuyoshi Sekizuka, Kentaro Itokawa, Rina Tanaka, Masanori Hashino, Tsutomu Kageyama, Shinji Saito, Ikuyo Takayama, Hideki Hasegawa, Takuri Takahashi, Hajime Kamiya, Takuya Yamagishi, Motoi Suzuki, Takaji Wakita, Makoto Kuroda
EPI_ISL_416632 hCoV-19/Japan/DP0827/2020 Asia / Japan / unknown 2020-02-17 Japanese Quarantine Stations Pathogen Genomics Center, National Institute of Infectious Diseases Tsuyoshi Sekizuka, Kentaro Itokawa, Rina Tanaka, Masanori Hashino, Tsutomu Kageyama, Shinji Saito, Ikuyo Takayama, Hideki Hasegawa, Takuri Takahashi, Hajime Kamiya, Takuya Yamagishi, Motoi Suzuki, Takaji Wakita, Makoto Kuroda
EPI_ISL_416633 hCoV-19/Japan/DP0880/2020 Asia / Japan / unknown 2020-02-17 Japanese Quarantine Stations Pathogen Genomics Center, National Institute of Infectious Diseases Tsuyoshi Sekizuka, Kentaro Itokawa, Rina Tanaka, Masanori Hashino, Tsutomu Kageyama, Shinji Saito, Ikuyo Takayama, Hideki Hasegawa, Takuri Takahashi, Hajime Kamiya, Takuya Yamagishi, Motoi Suzuki, Takaji Wakita, Makoto Kuroda
EPI_ISL_416634 hCoV-19/Japan/DP0890/2020 Asia / Japan / unknown 2020-02-17 Japanese Quarantine Stations Pathogen Genomics Center, National Institute of Infectious Diseases Tsuyoshi Sekizuka, Kentaro Itokawa, Rina Tanaka, Masanori Hashino, Tsutomu Kageyama, Shinji Saito, Ikuyo Takayama, Hideki Hasegawa, Takuri Takahashi, Hajime Kamiya, Takuya Yamagishi, Motoi Suzuki, Takaji Wakita, Makoto Kuroda
EPI_ISL_416635 hCoV-19/USA/WA-UW97/2020 North America / USA / Washington 2020-03-12 UW Virology Lab UW Virology Lab Pavitra Roychoudhury, Hong Xie, Keith Jerome, Alexander Greninger
EPI_ISL_416636 hCoV-19/USA/WA-UW98/2020 North America / USA / Washington 2020-03-12 UW Virology Lab UW Virology Lab Pavitra Roychoudhury, Hong Xie, Keith Jerome, Alexander Greninger
EPI_ISL_416637 hCoV-19/USA/WA-UW99/2020 North America / USA / Washington 2020-03-12 UW Virology Lab UW Virology Lab Pavitra Roychoudhury, Hong Xie, Keith Jerome, Alexander Greninger
EPI_ISL_416638 hCoV-19/USA/WA-UW100/2020 North America / USA / Washington 2020-03-12 UW Virology Lab UW Virology Lab Pavitra Roychoudhury, Hong Xie, Keith Jerome, Alexander Greninger
EPI_ISL_416639 hCoV-19/USA/WA-UW101/2020 North America / USA / Washington 2020-03-10 UW Virology Lab UW Virology Lab Pavitra Roychoudhury, Hong Xie, Keith Jerome, Alexander Greninger
EPI_ISL_416641 hCoV-19/USA/WA-UW103/2020 North America / USA / Washington 2020-03-11 UW Virology Lab UW Virology Lab Pavitra Roychoudhury, Hong Xie, Keith Jerome, Alexander Greninger
EPI_ISL_416642 hCoV-19/USA/WA-UW104/2020 North America / USA / Washington 2020-03-11 UW Virology Lab UW Virology Lab Pavitra Roychoudhury, Hong Xie, Keith Jerome, Alexander Greninger
EPI_ISL_416643 hCoV-19/USA/WA-UW105/2020 North America / USA / Washington 2020-03-11 UW Virology Lab UW Virology Lab Pavitra Roychoudhury, Hong Xie, Keith Jerome, Alexander Greninger
EPI_ISL_416644 hCoV-19/USA/WA-UW106/2020 North America / USA / Washington 2020-03-11 UW Virology Lab UW Virology Lab Pavitra Roychoudhury, Hong Xie, Keith Jerome, Alexander Greninger
EPI_ISL_416646 hCoV-19/USA/WA-UW108/2020 North America / USA / Washington 2020-03-11 UW Virology Lab UW Virology Lab Pavitra Roychoudhury, Hong Xie, Keith Jerome, Alexander Greninger
EPI_ISL_416647 hCoV-19/USA/WA-UW109/2020 North America / USA / Washington 2020-03-11 UW Virology Lab UW Virology Lab Pavitra Roychoudhury, Hong Xie, Keith Jerome, Alexander Greninger
EPI_ISL_416648 hCoV-19/USA/WA-UW110/2020 North America / USA / Washington 2020-03-11 UW Virology Lab UW Virology Lab Pavitra Roychoudhury, Hong Xie, Keith Jerome, Alexander Greninger
EPI_ISL_416649 hCoV-19/USA/WA-UW111/2020 North America / USA / Washington 2020-03-11 UW Virology Lab UW Virology Lab Pavitra Roychoudhury, Hong Xie, Keith Jerome, Alexander Greninger
EPI_ISL_416650 hCoV-19/USA/WA-UW112/2020 North America / USA 2020-03-10 UW Virology Lab UW Virology Lab Pavitra Roychoudhury, Hong Xie, Keith Jerome, Alexander Greninger
EPI_ISL_416651 hCoV-19/USA/WA-UW113/2020 North America / USA 2020-03-11 UW Virology Lab UW Virology Lab Pavitra Roychoudhury, Hong Xie, Keith Jerome, Alexander Greninger
EPI_ISL_416652 hCoV-19/USA/WA-UW114/2020 North America / USA / Washington 2020-03-11 UW Virology Lab UW Virology Lab Pavitra Roychoudhury, Hong Xie, Keith Jerome, Alexander Greninger
EPI_ISL_416653 hCoV-19/USA/WA-UW115/2020 North America / USA / Washington 2020-03-11 UW

Virology Lab	UW Virology Lab Pavitra Roychoudhury, Hong Xie, Keith Jerome, Alexander Greninger
EPI_ISL_416654	hCoV-19/USA/WA-UW116/2020	North America / USA / Washington	2020-03-11	UW
Virology Lab	UW Virology Lab Pavitra Roychoudhury, Hong Xie, Keith Jerome, Alexander Greninger
EPI_ISL_416655	hCoV-19/USA/WA-UW117/2020	North America / USA / Washington	2020-03-11	UW
Virology Lab	UW Virology Lab Pavitra Roychoudhury, Hong Xie, Keith Jerome, Alexander Greninger
EPI_ISL_416656	hCoV-19/USA/WA-UW118/2020	North America / USA / Washington	2020-03-11	UW
Virology Lab	UW Virology Lab Pavitra Roychoudhury, Hong Xie, Keith Jerome, Alexander Greninger
EPI_ISL_416657	hCoV-19/USA/WA-UW119/2020	North America / USA / Washington	2020-03-11	UW
Virology Lab	UW Virology Lab Pavitra Roychoudhury, Hong Xie, Keith Jerome, Alexander Greninger
EPI_ISL_416658	hCoV-19/USA/WA-UW120/2020	North America / USA / Washington	2020-03-11	UW
Virology Lab	UW Virology Lab Pavitra Roychoudhury, Hong Xie, Keith Jerome, Alexander Greninger
EPI_ISL_416659	hCoV-19/USA/WA-UW121/2020	North America / USA / Washington	2020-03-11	UW
Virology Lab	UW Virology Lab Pavitra Roychoudhury, Hong Xie, Keith Jerome, Alexander Greninger
EPI_ISL_416660	hCoV-19/USA/WA-UW122/2020	North America / USA	2020-03-10	UW Virology Lab UW
Virology Lab	Pavitra Roychoudhury, Hong Xie, Keith Jerome, Alexander Greninger
EPI_ISL_416661	hCoV-19/USA/WA-UW123/2020	North America / USA / Washington	2020-03-12	UW
Virology Lab	UW Virology Lab Pavitra Roychoudhury, Hong Xie, Keith Jerome, Alexander Greninger
EPI_ISL_416662	hCoV-19/USA/WA-UW124/2020	North America / USA / Washington	2020-03-12	UW
Virology Lab	UW Virology Lab Pavitra Roychoudhury, Hong Xie, Keith Jerome, Alexander Greninger
EPI_ISL_416663	hCoV-19/USA/WA-UW125/2020	North America / USA / Washington	2020-03-12	UW
Virology Lab	UW Virology Lab Pavitra Roychoudhury, Hong Xie, Keith Jerome, Alexander Greninger
EPI_ISL_416664	hCoV-19/USA/WA-UW126/2020	North America / USA / Washington	2020-03-12	UW
Virology Lab	UW Virology Lab Pavitra Roychoudhury, Hong Xie, Keith Jerome, Alexander Greninger
EPI_ISL_416665	hCoV-19/USA/WA-UW127/2020	North America / USA / Washington	2020-03-12	UW
Virology Lab	UW Virology Lab Pavitra Roychoudhury, Hong Xie, Keith Jerome, Alexander Greninger
EPI_ISL_416666	hCoV-19/USA/WA-UW128/2020	North America / USA / Washington	2020-03-12	UW
Virology Lab	UW Virology Lab Pavitra Roychoudhury, Hong Xie, Keith Jerome, Alexander Greninger
EPI_ISL_416667	hCoV-19/USA/WA-UW129/2020	North America / USA / Washington	2020-03-12	UW
Virology Lab	UW Virology Lab Pavitra Roychoudhury, Hong Xie, Keith Jerome, Alexander Greninger
EPI_ISL_416668	hCoV-19/USA/WA-UW130/2020	North America / USA / Washington	2020-03-11	UW
Virology Lab	UW Virology Lab Pavitra Roychoudhury, Hong Xie, Keith Jerome, Alexander Greninger
EPI_ISL_416669	hCoV-19/USA/WA-UW131/2020	North America / USA / Washington	2020-03-11	UW
Virology Lab	UW Virology Lab Pavitra Roychoudhury, Hong Xie, Keith Jerome, Alexander Greninger
EPI_ISL_416671	hCoV-19/USA/WA-UW133/2020	North America / USA / Washington	2020-03-12	UW
Virology Lab	UW Virology Lab Pavitra Roychoudhury, Hong Xie, Keith Jerome, Alexander Greninger
EPI_ISL_416672	hCoV-19/USA/WA-UW134/2020	North America / USA	2020-03-10	UW Virology Lab UW
Virology Lab	Pavitra Roychoudhury, Hong Xie, Keith Jerome, Alexander Greninger
EPI_ISL_416673	hCoV-19/USA/OR-UW135/2020	North America / USA / Oregon	2020-03-11	UW Virology Lab
UW Virology Lab Pavitra Roychoudhury, Hong Xie, Keith Jerome, Alexander Greninger
EPI_ISL_416674	hCoV-19/USA/WA-UW136/2020	North America / USA	2020-03-11	UW Virology Lab UW
Virology Lab	Pavitra Roychoudhury, Hong Xie, Keith Jerome, Alexander Greninger
EPI_ISL_416675	hCoV-19/USA/WA-UW137/2020	North America / USA	2020-03-11	UW Virology Lab UW
Virology Lab	Pavitra Roychoudhury, Hong Xie, Keith Jerome, Alexander Greninger
EPI_ISL_416676	hCoV-19/USA/WA-UW138/2020	North America / USA	2020-03-11	UW Virology Lab UW
Virology Lab	Pavitra Roychoudhury, Hong Xie, Keith Jerome, Alexander Greninger
EPI_ISL_416677	hCoV-19/USA/WA-UW139/2020	North America / USA / Washington	2020-03-12	UW
Virology Lab	UW Virology Lab Pavitra Roychoudhury, Hong Xie, Keith Jerome, Alexander Greninger
EPI_ISL_416678	hCoV-19/USA/WA-UW140/2020	North America / USA / Washington	2020-03-11	UW
Virology Lab	UW Virology Lab Pavitra Roychoudhury, Hong Xie, Keith Jerome, Alexander Greninger
EPI_ISL_416679	hCoV-19/USA/WA-UW141/2020	North America / USA / Washington	2020-03-11	UW
Virology Lab	UW Virology Lab Pavitra Roychoudhury, Hong Xie, Keith Jerome, Alexander Greninger
EPI_ISL_416680	hCoV-19/USA/WA-UW142/2020	North America / USA / Washington	2020-03-11	UW
Virology Lab	UW Virology Lab Pavitra Roychoudhury, Hong Xie, Keith Jerome, Alexander Greninger
EPI_ISL_416681	hCoV-19/USA/WA-UW143/2020	North America / USA / Washington	2020-03-11	UW
Virology Lab	UW Virology Lab Pavitra Roychoudhury, Hong Xie, Keith Jerome, Alexander Greninger
EPI_ISL_416682	hCoV-19/USA/WA-UW144/2020	North America / USA	2020-03-09	UW Virology Lab UW
Virology Lab	Pavitra Roychoudhury, Hong Xie, Keith Jerome, Alexander Greninger
EPI_ISL_416683	hCoV-19/USA/WA-UW145/2020	North America / USA / Washington	2020-03-15	UW
Virology Lab	UW Virology Lab Pavitra Roychoudhury, Hong Xie, Keith Jerome, Alexander Greninger
EPI_ISL_416684	hCoV-19/USA/WA-UW146/2020	North America / USA / Washington	2020-03-14	UW
Virology Lab	UW Virology Lab Pavitra Roychoudhury, Hong Xie, Keith Jerome, Alexander Greninger
EPI_ISL_416685	hCoV-19/USA/WA-UW147/2020	North America / USA / Washington	2020-03-15	UW
Virology Lab	UW Virology Lab Pavitra Roychoudhury, Hong Xie, Keith Jerome, Alexander Greninger
EPI_ISL_416686	hCoV-19/USA/WA-UW148/2020	North America / USA / Washington	2020-03-14	UW
Virology Lab	UW Virology Lab Pavitra Roychoudhury, Hong Xie, Keith Jerome, Alexander Greninger
EPI_ISL_416687	hCoV-19/USA/WA-UW149/2020	North America / USA / Washington	2020-03-14	UW
Virology Lab	UW Virology Lab Pavitra Roychoudhury, Hong Xie, Keith Jerome, Alexander Greninger
EPI_ISL_416688	hCoV-19/USA/WA-UW150/2020	North America / USA / Washington	2020-03-14	UW
Virology Lab	UW Virology Lab Pavitra Roychoudhury, Hong Xie, Keith Jerome, Alexander Greninger
EPI_ISL_416689	hCoV-19/USA/WA-UW151/2020	North America / USA / Washington	2020-03-14	UW

Virology Lab	UW Virology Lab Pavitra Roychoudhury, Hong Xie, Keith Jerome, Alexander Greninger
EPI_ISL_416690	hCoV-19/USA/WA-UW152/2020	North America / USA / Washington	2020-03-13	UW Virology Lab	UW Virology Lab Pavitra Roychoudhury, Hong Xie, Keith Jerome, Alexander Greninger
EPI_ISL_416691	hCoV-19/USA/WA-UW153/2020	North America / USA / Washington	2020-03-13	UW Virology Lab	UW Virology Lab Pavitra Roychoudhury, Hong Xie, Keith Jerome, Alexander Greninger
EPI_ISL_416692	hCoV-19/USA/WA-UW154/2020	North America / USA / Washington	2020-03-14	UW Virology Lab	UW Virology Lab Pavitra Roychoudhury, Hong Xie, Keith Jerome, Alexander Greninger
EPI_ISL_416693	hCoV-19/USA/WA-UW155/2020	North America / USA / Washington	2020-03-12	UW Virology Lab	UW Virology Lab Pavitra Roychoudhury, Hong Xie, Keith Jerome, Alexander Greninger
EPI_ISL_416694	hCoV-19/USA/WA-UW156/2020	North America / USA / Washington	2020-03-13	UW Virology Lab	UW Virology Lab Pavitra Roychoudhury, Hong Xie, Keith Jerome, Alexander Greninger
EPI_ISL_416696	hCoV-19/USA/CT-UW158/2020	North America / USA / Connecticut	2020-03-13	UW Virology Lab	UW Virology Lab Pavitra Roychoudhury, Hong Xie, Keith Jerome, Alexander Greninger
EPI_ISL_416697	hCoV-19/USA/CT-UW159/2020	North America / USA / Connecticut	2020-03-13	UW Virology Lab	UW Virology Lab Pavitra Roychoudhury, Hong Xie, Keith Jerome, Alexander Greninger
EPI_ISL_416698	hCoV-19/USA/WA-UW160/2020	North America / USA / Washington	2020-03-13	UW Virology Lab	UW Virology Lab Pavitra Roychoudhury, Hong Xie, Keith Jerome, Alexander Greninger
EPI_ISL_416699	hCoV-19/USA/WA-UW161/2020	North America / USA / Washington	2020-03-13	UW Virology Lab	UW Virology Lab Pavitra Roychoudhury, Hong Xie, Keith Jerome, Alexander Greninger
EPI_ISL_417400	hCoV-19/Australia/NSW44/2020	Oceania / Australia / New South Wales / Sydney	2020-03-10	Centre for Infectious Diseases and Microbiology Public Health	NSW Health Pathology - Institute of Clinical Pathology and Medical Research; Westmead Hospital; University of Sydney Rockett R, Eden J-S, Lam C, Gray K, Timms V, Gall M, Arnott A, Sadsad R, Carter I, Rahman H, Holmes EC, O'Sullivan MV, Sintchenko V, Chen SC, Maddocks S, Kok J and Dwyer DE for the 2019-nCoV Study Group
EPI_ISL_417401	hCoV-19/Australia/NSW45/2020	Oceania / Australia / New South Wales / Sydney	2020-03-13	Centre for Infectious Diseases and Microbiology Public Health	NSW Health Pathology - Institute of Clinical Pathology and Medical Research; Westmead Hospital; University of Sydney Eden J-S, Lam C, Gray K, Timms V, Gall M, Arnott A, Sadsad R, Carter I, Rahman H, Holmes EC, O'Sullivan MV, Sintchenko V, Chen SC, Maddocks S, Kok J, Dwyer DE and Rockett R for the 2019-nCoV Study Group
EPI_ISL_417402	hCoV-19/Australia/NSW47/2020	Oceania / Australia / New South Wales / Sydney	2020-03-13	Centre for Infectious Diseases and Microbiology Public Health	NSW Health Pathology - Institute of Clinical Pathology and Medical Research; Westmead Hospital; University of Sydney Lam C, Gray K, Timms V, Gall M, Arnott A, Sadsad R, Carter I, Rahman H, Holmes EC, O'Sullivan MV, Sintchenko V, Chen SC, Maddocks S, Kok J, Dwyer DE, Rockett R and Eden J-S for the 2019-nCoV Study Group
EPI_ISL_417403	hCoV-19/Australia/NSW48/2020	Oceania / Australia / New South Wales / Sydney	2020-03-14	Centre for Infectious Diseases and Microbiology Public Health	NSW Health Pathology - Institute of Clinical Pathology and Medical Research; Westmead Hospital; University of Sydney Gray K, Timms V, Gall M, Arnott A, Sadsad R, Carter I, Rahman H, Holmes EC, O'Sullivan MV, Sintchenko V, Chen SC, Maddocks S, Kok J, Dwyer DE, Rockett R, Eden J-S and Lam C for the 2019-nCoV Study Group
EPI_ISL_417404	hCoV-19/Australia/NSW50/2020	Oceania / Australia / New South Wales / Sydney	2020-03-12	Centre for Infectious Diseases and Microbiology Public Health	NSW Health Pathology - Institute of Clinical Pathology and Medical Research; Westmead Hospital; University of Sydney Timms V, Gall M, Arnott A, Sadsad R, Carter I, Rahman H, Holmes EC, O'Sullivan MV, Sintchenko V, Chen SC, Maddocks S, Kok J, Dwyer DE, Rockett R, Eden J-S, Lam C and Gray K for the 2019-nCoV Study Group
EPI_ISL_417405	hCoV-19/Australia/NSW52/2020	Oceania / Australia / New South Wales / Sydney	2020-03-11	Centre for Infectious Diseases and Microbiology Public Health	NSW Health Pathology - Institute of Clinical Pathology and Medical Research; Westmead Hospital; University of Sydney Gall M, Arnott A, Sadsad R, Carter I, Rahman H, Holmes EC, O'Sullivan MV, Sintchenko V, Chen SC, Maddocks S, Kok J, Dwyer DE, Rockett R, Eden J-S, Lam C, Gray K and Timms V for the 2019-nCoV Study Group
EPI_ISL_417406	hCoV-19/Australia/NSW53/2020	Oceania / Australia / New South Wales / Sydney	2020-03-11	Centre for Infectious Diseases and Microbiology Public Health	NSW Health Pathology - Institute of Clinical Pathology and Medical Research; Westmead Hospital; University of Sydney Arnott A, Sadsad R, Carter I, Rahman H, Holmes EC, O'Sullivan MV, Sintchenko V, Chen SC, Maddocks S, Kok J, Dwyer DE, Rockett R, Eden J-S, Lam C, Gray K, Timms V and Gall M for the 2019-nCoV Study Group
EPI_ISL_417407	hCoV-19/Australia/NSW54/2020	Oceania / Australia / New South Wales / Sydney	2020-03-14	Centre for Infectious Diseases and Microbiology Public Health	NSW Health Pathology - Institute of Clinical Pathology and Medical Research; Westmead Hospital; University of Sydney Sadsad R, Carter I, Rahman H, Holmes EC, O'Sullivan MV, Sintchenko V, Chen SC, Maddocks S, Kok J, Dwyer DE, Rockett R, Eden J-S, Lam C, Gray K, Timms V, Gall M and Arnott A for the 2019-nCoV Study Group
EPI_ISL_417917	hCoV-19/Malaysia/189332/2020	Asia / Malaysia / Kuala Lumpur	2020-03-20	Department of Medical Microbiology, University Malaya Medical Centre	Department of Medical Microbiology	Yoong Min CHONG, Sasheela PONNAMPALAVANAR, Sharifah Faridah SYED OMAR, Adeeba KAMARULZAMAN,Vijayan MUNUSAMY, Chee Kuan WONG, Cindy Shuan Ju TEH, I-Ching SAM, Yoke Fun Chan, University Malaya Medical Centre COVID Team
EPI_ISL_417918	hCoV-19/Malaysia/188407/2020	Asia / Malaysia / Kuala Lumpur	2020-03-18	Department of Medical Microbiology, University Malaya Medical Centre	Department of Medical Microbiology, Faculty of Medicine, University of Malaya	Yoong Min CHONG, Sasheela PONNAMPALAVANAR, Sharifah Faridah SYED OMAR, Adeeba KAMARULZAMAN,Vijayan MUNUSAMY, Chee Kuan WONG, Cindy Shuan Ju TEH, I-Ching SAM, Yoke Fun Chan, University Malaya Medical Centre COVID Team
EPI_ISL_417921	hCoV-19/Italy/INMI3/2020	Europe / Italy / Rome	2020-03-01	INMI Lazzaro Spallanzani IRCCS	Laboratory of Virology, INMI Lazzaro Spallanzani IRCCS	Martina Rueca, Barbara

Bartolini, Francesco Messina, Cesare E. M. Gruber, Emanuela Giombini, Maria R. Capobianchi, Fabrizio Carletti, Francesca Colavita, Concetta Castilletti, Eleonora Lalle, Daniele Lapa, Giuseppe Ippolito.
EPI_ISL_417922 hCoV-19/Italy/INMI4/2020 Europe / Italy / Rome 2020-02-28 INMI Lazzaro Spallanzani IRCCS Laboratory of Virology, INMI Lazzaro Spallanzani IRCCS Cesare E. M. Gruber, Martina Rueca, Barbara Bartolini, Francesco Messina, Emanuela Giombini, Maria R. Capobianchi, Fabrizio Carletti, Francesca Colavita, Concetta Castilletti, Eleonora Lalle, Daniele Lapa, Giuseppe Ippolito.
EPI_ISL_417923 hCoV-19/Italy/INMI5/2020 Europe / Italy / Rome 2020-03-04 INMI Lazzaro Spallanzani IRCCS Laboratory of Virology, INMI Lazzaro Spallanzani IRCCS Francesco Messina, Barbara Bartolini, Martina Rueca, Cesare E. M. Gruber, Emanuela Giombini, Maria R. Capobianchi, Fabrizio Carletti, Francesca Colavita, Concetta Castilletti, Eleonora Lalle, Daniele Lapa, Giuseppe Ippolito.
EPI_ISL_417924 hCoV-19/Colombia/INS-79256/2020 South America / Colombia / Antioquia 2020-03-11 Secretaría de Salud Medellín Instituto Nacional de Salud, Universidad Cooperativa de Colombia, Instituto Alexander von Humboldt, Imperial College-London, London School of Hygiene & Tropical Medicine Marcela Mercado-Reyes, Katherine Laiton-Donato, Diego A. Álvarez-Díaz, Carlos Franco-Muñoz, Jose A. Usme-Ciro, Gloria Puerto, Nicolás D. Franco-Sierra, Mailyn A. Gonzalez, Zulma M. Cucunubá, Christian Julian Villabona-Arenas, Liz Villabona-Arenas, Sussy Echeverria-Londoño, Astrid C. Flórez, Sergio Gomez Rangel, Luz Dary Rodriguez, Juliana Barbosa, Erika Ospitia, Diana Marcela Walteros-Acero, Martha Lucia Ospina Martinez
EPI_ISL_417931 hCoV-19/USA/CA-CZB05/2020 North America / USA / California / San Francisco 2020-03-18 UCSF Clinical Microbiology Laboratory Chan-Zuckerberg Biohub Shaun Arevalo, Josh Batson, Olga Botvinnik, Gloria Castaneda, Angela Detweiler, David Dynerman, Samantha Hao, Jack Kamm, Amy Kistler, G. Renuka Kumar, Chaz Langelier, Lucy Li, Steve Miller, Lusajo Mwakibete, Norma Neff, Angela Pisco, Maira Phelps, Michelle Tan, Chunyu Zhao
EPI_ISL_417932 hCoV-19/USA/CA-CZB06/2020 North America / USA / California / San Francisco 2020-03-18 UCSF Clinical Microbiology Laboratory Chan-Zuckerberg Biohub Shaun Arevalo, Josh Batson, Olga Botvinnik, Gloria Castaneda, Angela Detweiler, David Dynerman, Samantha Hao, Jack Kamm, Amy Kistler, G. Renuka Kumar, Chaz Langelier, Lucy Li, Steve Miller, Lusajo Mwakibete, Norma Neff, Angela Pisco, Maira Phelps, Michelle Tan, Chunyu Zhao
EPI_ISL_417933 hCoV-19/USA/CA-CZB07/2020 North America / USA / California / San Francisco 2020-03-18 UCSF Clinical Microbiology Laboratory Chan-Zuckerberg Biohub Shaun Arevalo, Josh Batson, Olga Botvinnik, Gloria Castaneda, Angela Detweiler, David Dynerman, Samantha Hao, Jack Kamm, Amy Kistler, G. Renuka Kumar, Chaz Langelier, Lucy Li, Steve Miller, Lusajo Mwakibete, Norma Neff, Angela Pisco, Maira Phelps, Michelle Tan, Chunyu Zhao
EPI_ISL_417935 hCoV-19/USA/CA-CZB011a/2020 North America / USA / California / San Francisco 2020-03-18 UCSF Clinical Microbiology Laboratory Chan-Zuckerberg Biohub Shaun Arevalo, Josh Batson, Olga Botvinnik, Gloria Castaneda, Angela Detweiler, David Dynerman, Samantha Hao, Jack Kamm, Amy Kistler, G. Renuka Kumar, Chaz Langelier, Lucy Li, Steve Miller, Lusajo Mwakibete, Norma Neff, Angela Pisco, Maira Phelps, Michelle Tan, Chunyu Zhao
EPI_ISL_417937 hCoV-19/USA/CA-CZB013/2020 North America / USA / California / San Francisco 2020-03-18 UCSF Clinical Microbiology Laboratory Chan-Zuckerberg Biohub Shaun Arevalo, Josh Batson, Olga Botvinnik, Gloria Castaneda, Angela Detweiler, David Dynerman, Samantha Hao, Jack Kamm, Amy Kistler, G. Renuka Kumar, Chaz Langelier, Lucy Li, Steve Miller, Lusajo Mwakibete, Norma Neff, Angela Pisco, Maira Phelps, Michelle Tan, Chunyu Zhao
EPI_ISL_417938 hCoV-19/USA/CA-CZB014/2020 North America / USA / California / San Francisco 2020-03-18 UCSF Clinical Microbiology Laboratory Chan-Zuckerberg Biohub Shaun Arevalo, Josh Batson, Olga Botvinnik, Gloria Castaneda, Angela Detweiler, David Dynerman, Samantha Hao, Jack Kamm, Amy Kistler, G. Renuka Kumar, Chaz Langelier, Lucy Li, Steve Miller, Lusajo Mwakibete, Norma Neff, Angela Pisco, Maira Phelps, Michelle Tan, Chunyu Zhao
EPI_ISL_417939 hCoV-19/USA/CA-CZB015a/2020 North America / USA / California / San Francisco 2020-03-18 UCSF Clinical Microbiology Laboratory Chan-Zuckerberg Biohub Shaun Arevalo, Josh Batson, Olga Botvinnik, Gloria Castaneda, Angela Detweiler, David Dynerman, Samantha Hao, Jack Kamm, Amy Kistler, G. Renuka Kumar, Chaz Langelier, Lucy Li, Steve Miller, Lusajo Mwakibete, Norma Neff, Angela Pisco, Maira Phelps, Michelle Tan, Chunyu Zhao
EPI_ISL_417941 hCoV-19/DRC/73/2020 Africa / Democratic Republic of the Congo / Kinshasa 2020-03-18 Viral Respiratory Lab, National Institute for Biomedical Research (INRB) Pathogen Sequencing Lab, National Institute for Biomedical Research (INRB) Placide Mbala-Kingebeni, Edith Nkwembe, Eddy Kinganda-Lusamaki, Amuri Aziza, Catherine Pratt, Matthias Pauthner, Josh Quick, Allison Black, James Hadfield, Trevor Bedford, Ian Goodfellow, Nick Loman, Kristian Andersen, Michael Wiley, Steve Ahuka-Mundeke, Jean-Jacques Muyembe Tamfum
EPI_ISL_417942 hCoV-19/DRC/80/2020 Africa / Democratic Republic of the Congo / Kinshasa 2020-03-18 Viral Respiratory Lab, National Institute for Biomedical Research (INRB) Pathogen Sequencing Lab, National Institute for Biomedical Research (INRB) Placide Mbala-Kingebeni, Edith Nkwembe, Eddy Kinganda-Lusamaki, Amuri Aziza, Catherine Pratt, Matthias Pauthner, Josh Quick, Allison Black, James Hadfield, Trevor Bedford, Ian Goodfellow, Nick Loman, Kristian Andersen, Michael Wiley, Steve Ahuka-Mundeke, Jean-Jacques Muyembe Tamfum
EPI_ISL_417944 hCoV-19/DRC/81/2020 Africa / Democratic Republic of the Congo / Kinshasa 2020-03-18 Viral Respiratory Lab, National Institute for Biomedical Research (INRB) Pathogen Sequencing Lab, National Institute for Biomedical Research (INRB) Placide Mbala-Kingebeni, Edith Nkwembe, Eddy Kinganda-Lusamaki, Amuri Aziza, Catherine Pratt, Matthias Pauthner, Josh Quick, Allison Black, James Hadfield, Trevor Bedford, Ian Goodfellow, Nick Loman, Kristian Andersen, Michael Wiley, Steve Ahuka-Mundeke, Jean-Jacques Muyembe Tamfum

EPI_ISL_417946 hCoV-19/DRC/82/2020 Africa / Democratic Republic of the Congo / Kinshasa 2020-03-18 Viral Respiratory Lab, National Institute for Biomedical Research (INRB) Pathogen Sequencing Lab, National Institute for Biomedical Research (INRB) Placide Mbala-Kingebeni, Edith Nkwembe, Eddy Kinganda-Lusamaki, Amuri Aziza, Catherine Pratt, Matthias Pauthner, Josh Quick, Allison Black, James Hadfield, Trevor Bedford, Ian Goodfellow, Nick Loman, Kristian Andersen, Michael Wiley, Steve Ahuka-Mundeke, Jean-Jacques Muyembe Tamfum
EPI_ISL_417947 hCoV-19/DRC/94/2020 Africa / Democratic Republic of the Congo / Kinshasa 2020-03-19 Viral Respiratory Lab, National Institute for Biomedical Research (INRB) Pathogen Sequencing Lab, National Institute for Biomedical Research (INRB) Placide Mbala-Kingebeni, Edith Nkwembe, Eddy Kinganda-Lusamaki, Amuri Aziza, Catherine Pratt, Matthias Pauthner, Josh Quick, Allison Black, James Hadfield, Trevor Bedford, Ian Goodfellow, Nick Loman, Kristian Andersen, Michael Wiley, Steve Ahuka-Mundeke, Jean-Jacques Muyembe Tamfum
EPI_ISL_417948 hCoV-19/DRC/108/2020 Africa / Democratic Republic of the Congo / Kinshasa 2020-03-19 Viral Respiratory Lab, National Institute for Biomedical Research (INRB) Pathogen Sequencing Lab, National Institute for Biomedical Research (INRB) Placide Mbala-Kingebeni, Edith Nkwembe, Eddy Kinganda-Lusamaki, Amuri Aziza, Catherine Pratt, Matthias Pauthner, Josh Quick, Allison Black, James Hadfield, Trevor Bedford, Ian Goodfellow, Nick Loman, Kristian Andersen, Michael Wiley, Steve Ahuka-Mundeke, Jean-Jacques Muyembe Tamfum
EPI_ISL_417950 hCoV-19/DRC/158/2020 Africa / Democratic Republic of the Congo / Kinshasa 2020-03-20 Viral Respiratory Lab, National Institute for Biomedical Research (INRB) Pathogen Sequencing Lab, National Institute for Biomedical Research (INRB) Placide Mbala-Kingebeni, Edith Nkwembe, Eddy Kinganda-Lusamaki, Amuri Aziza, Catherine Pratt, Matthias Pauthner, Josh Quick, Allison Black, James Hadfield, Trevor Bedford, Ian Goodfellow, Nick Loman, Kristian Andersen, Michael Wiley, Steve Ahuka-Mundeke, Jean-Jacques Muyembe Tamfum
EPI_ISL_417954 hCoV-19/Spain/Madrid_H3_10/2020 Europe / Spain / Madrid 2020-03-12 Hospital Universitario 12 de Octubre Hospital Universitario La Paz Elias Dahdouh, Sara González, Fernando Lázaro, Esther Viedma, Natalia Stella, Julio García, Juan Carlos Galán, Rafael Cantón, Mª Dolores Folgueira, Rafael Delgado, Jesús Mingorance
EPI_ISL_417955 hCoV-19/DRC/191/2020 Africa / Democratic Republic of the Congo / Kinshasa 2020-03-21 Viral Respiratory Lab, National Institute for Biomedical Research (INRB) Pathogen Sequencing Lab, National Institute for Biomedical Research (INRB) Placide Mbala-Kingebeni, Edith Nkwembe, Eddy Kinganda-Lusamaki, Amuri Aziza, Catherine Pratt, Matthias Pauthner, Josh Quick, Allison Black, James Hadfield, Trevor Bedford, Ian Goodfellow, Nick Loman, Kristian Andersen, Michael Wiley, Steve Ahuka-Mundeke, Jean-Jacques Muyembe Tamfum
EPI_ISL_417956 hCoV-19/Spain/Madrid_H5_34/2020 Europe / Spain / Madrid 2020-03-11 Hospital Universitario 12 de Octubre Hospital Universitario La Paz Elias Dahdouh, Sara González, Fernando Lázaro, Esther Viedma, Natalia Stella, Julio García, Juan Carlos Galán, Rafael Cantón, Mª Dolores Folgueira, Rafael Delgado, Jesús Mingorance
EPI_ISL_417957 hCoV-19/Spain/Madrid_H7_36/2020 Europe / Spain / Madrid 2020-03-12 Hospital Universitario 12 de Octubre Hospital Universitario La Paz Elias Dahdouh, Sara González, Fernando Lázaro, Esther Viedma, Natalia Stella, Julio García, Juan Carlos Galán, Rafael Cantón, Mª Dolores Folgueira, Rafael Delgado, Jesús Mingorance
EPI_ISL_417958 hCoV-19/USA/UT-010/2020 North America / USA / Utah 2020-03-10 Utah Public Health Laboratory Utah Public Health Laboratory Erin Young, Kelly Oakeson
EPI_ISL_417960 hCoV-19/USA/UT-012/2020 North America / USA / Utah 2020-03-12 Utah Public Health Laboratory Utah Public Health Laboratory Erin Young, Kelly Oakeson
EPI_ISL_417961 hCoV-19/Spain/Madrid_H9_38/2020 Europe / Spain / Madrid 2020-03-12 Hospital Universitario 12 de Octubre Hospital Universitario La Paz Elias Dahdouh, Sara González, Fernando Lázaro, Esther Viedma, Natalia Stella, Julio García, Juan Carlos Galán, Rafael Cantón, Mª Dolores Folgueira, Rafael Delgado, Jesús Mingorance
EPI_ISL_417963 hCoV-19/Spain/Madrid_H10_39/2020 Europe / Spain / Madrid 2020-03-12 Hospital Universitario 12 de Octubre Hospital Universitario La Paz Elias Dahdouh, Sara González, Fernando Lázaro, Esther Viedma, Natalia Stella, Julio García, Juan Carlos Galán, Rafael Cantón, Mª Dolores Folgueira, Rafael Delgado, Jesús Mingorance
EPI_ISL_417964 hCoV-19/USA/UT-014/2020 North America / USA / Utah 2020-03-13 Utah Public Health Laboratory Utah Public Health Laboratory Erin Young, Kelly Oakeson
EPI_ISL_417966 hCoV-19/USA/UT-016/2020 North America / USA / Utah 2020-03-13 Utah Public Health Laboratory Utah Public Health Laboratory Erin Young, Kelly Oakeson
EPI_ISL_417967 hCoV-19/Spain/Madrid_H11_40/2020 Europe / Spain / Madrid 2020-03-12 Hospital Universitario 12 de Octubre Hospital Universitario La Paz Elias Dahdouh, Sara González, Fernando Lázaro, Esther Viedma, Natalia Stella, Julio García, Juan Carlos Galán, Rafael Cantón, Mª Dolores Folgueira, Rafael Delgado, Jesús Mingorance
EPI_ISL_417969 hCoV-19/Spain/Madrid_LP10_12/2020 Europe / Spain / Madrid 2020-03-09 Hospital Universitario La Paz Hospital Universitario La Paz Elias Dahdouh, Sara González, Fernando Lázaro, Esther Viedma, Natalia Stella, Julio García, Juan Carlos Galán, Rafael Cantón, Mª Dolores Folgueira, Rafael Delgado, Jesús Mingorance
EPI_ISL_417971 hCoV-19/USA/UT-028/2020 North America / USA / Utah 2020-03-19 Utah Public Health Laboratory Utah Public Health Laboratory Erin Young, Kelly Oakeson
EPI_ISL_417972 hCoV-19/Spain/Madrid_LP12_21/2020 Europe / Spain / Madrid 2020-03-09 Hospital Universitario La Paz Hospital Universitario La Paz Elias Dahdouh, Sara González, Fernando Lázaro, Esther

Viedma, Natalia Stella, Julio García, Juan Carlos Galán, Rafael Cantón, Mª Dolores Folgueira, Rafael Delgado, Jesús Mingorance
EPI_ISL_417973 hCoV-19/USA/UT-031/2020 North America / USA / Utah 2020-03-19 Utah Public Health Laboratory Utah Public Health Laboratory Erin Young, Kelly Oakeson
EPI_ISL_417974 hCoV-19/USA/UT-032/2020 North America / USA / Utah 2020-03-19 Utah Public Health Laboratory Utah Public Health Laboratory Erin Young, Kelly Oakeson
EPI_ISL_417975 hCoV-19/Spain/Madrid_LP14_3/2020 Europe / Spain / Madrid 2020-03-09 Hospital Universitario La Paz Hospital Universitario La Paz Elias Dahdouh, Sara González, Fernando Lázaro, Esther Viedma, Natalia Stella, Julio García, Juan Carlos Galán, Rafael Cantón, Mª Dolores Folgueira, Rafael Delgado, Jesús Mingorance
EPI_ISL_417976 hCoV-19/USA/UT-033/2020 North America / USA / Utah 2020-03-19 Utah Public Health Laboratory Utah Public Health Laboratory Erin Young, Kelly Oakeson
EPI_ISL_417977 hCoV-19/USA/UT-034/2020 North America / USA / Utah 2020-03-20 Utah Public Health Laboratory Utah Public Health Laboratory Erin Young, Kelly Oakeson
EPI_ISL_417978 hCoV-19/Spain/Madrid_LP15_4/2020 Europe / Spain / Madrid 2020-03-09 Hospital Universitario La Paz Hospital Universitario La Paz Elias Dahdouh, Sara González, Fernando Lázaro, Esther Viedma, Natalia Stella, Julio García, Juan Carlos Galán, Rafael Cantón, Mª Dolores Folgueira, Rafael Delgado, Jesús Mingorance
EPI_ISL_417979 hCoV-19/Spain/Madrid_R2_15/2020 Europe / Spain / Madrid 2020-03-03 Hospital Universitario Ramón y Cajal Hospital Universitario La Paz Elias Dahdouh, Sara González, Fernando Lázaro, Esther Viedma, Natalia Stella, Julio García, Juan Carlos Galán, Rafael Cantón, Mª Dolores Folgueira, Rafael Delgado, Jesús Mingorance
EPI_ISL_417980 hCoV-19/Spain/Madrid_R5_8/2020 Europe / Spain / Madrid 2020-03-03 Hospital Universitario Ramón y Cajal Hospital Universitario La Paz Elias Dahdouh, Sara González, Fernando Lázaro, Esther Viedma, Natalia Stella, Julio García, Juan Carlos Galán, Rafael Cantón, Mª Dolores Folgueira, Rafael Delgado, Jesús Mingorance
EPI_ISL_417981 hCoV-19/Spain/Madrid_R10_33/2020 Europe / Spain / Madrid 2020-03-02 Hospital Universitario Ramón y Cajal Hospital Universitario La Paz Elias Dahdouh, Sara González, Fernando Lázaro, Esther Viedma, Natalia Stella, Julio García, Juan Carlos Galán, Rafael Cantón, Mª Dolores Folgueira, Rafael Delgado, Jesús Mingorance
EPI_ISL_417986 hCoV-19/Portugal/PT0001b/2020 Europe / Portugal 2020-03-03 Centro Hospitalar e Universitario de Sao Joao, Porto Instituto Nacional de Saude (INSA) Guiomar et al
EPI_ISL_417987 hCoV-19/Portugal/PT0003/2020 Europe / Portugal 2020-03-03 Centro Hospitalar e Universitario de Sao Joao, Porto Instituto Nacional de Saude (INSA) Guiomar et al
EPI_ISL_417988 hCoV-19/Portugal/PT0004/2020 Europe / Portugal 2020-03-05 CHULC - H Curry Cabral Instituto Nacional de Saude (INSA) Guiomar et al
EPI_ISL_417989 hCoV-19/Portugal/PT0005/2020 Europe / Portugal 2020-03-04 Centro Hospitalar e Universitario de Sao Joao, Porto Instituto Nacional de Saude (INSA) Guiomar et al
EPI_ISL_417990 hCoV-19/Portugal/PT0006a/2020 Europe / Portugal 2020-03-06 CHULC - H Curry Cabral Instituto Nacional de Saude (INSA) Guiomar et al
EPI_ISL_417991 hCoV-19/Portugal/PT0006b/2020 Europe / Portugal 2020-03-08 CHULC - H Curry Cabral Instituto Nacional de Saude (INSA) Guiomar et al
EPI_ISL_417992 hCoV-19/Portugal/PT0007/2020 Europe / Portugal 2020-03-07 CHULC - H D Estefania Instituto Nacional de Saude (INSA) Guiomar et al
EPI_ISL_417993 hCoV-19/Portugal/PT0008/2020 Europe / Portugal 2020-03-08 CHULC - H D Estefania Instituto Nacional de Saude (INSA) Guiomar et al
EPI_ISL_417994 hCoV-19/Portugal/PT0009/2020 Europe / Portugal 2020-03-08 CHULC - H Curry Cabral Instituto Nacional de Saude (INSA) Guiomar et al
EPI_ISL_417995 hCoV-19/Portugal/PT0010/2020 Europe / Portugal 2020-03-08 CHULC - H Curry Cabral Instituto Nacional de Saude (INSA) Guiomar et al
EPI_ISL_417996 hCoV-19/Portugal/PT0011/2020 Europe / Portugal 2020-03-08 CHULC - H Curry Cabral Instituto Nacional de Saude (INSA) Guiomar et al
EPI_ISL_417997 hCoV-19/Portugal/PT0012/2020 Europe / Portugal 2020-03-07 Centro Hospital do Porto, E.P.E. - H. Geral de Santo Antonio Instituto Nacional de Saude (INSA) Guiomar et al
EPI_ISL_417998 hCoV-19/Portugal/PT0013/2020 Europe / Portugal 2020-03-08 Centro Hospital do Porto, E.P.E. - H. Geral de Santo Antonio Instituto Nacional de Saude (INSA) Guiomar et al
EPI_ISL_417999 hCoV-19/Portugal/PT0014/2020 Europe / Portugal 2020-03-07 Centro Hospital do Porto, E.P.E. - H. Geral de Santo Antonio Instituto Nacional de Saude (INSA) Guiomar et al
EPI_ISL_418700 hCoV-19/England/20124013202/2020 Europe / United Kingdom / England 2020-03-17 Respiratory Virus Unit, Microbiology Services Colindale, Public Health England Respiratory Virus Unit, Microbiology Services Colindale, Public Health England Monica Galiano, Shahjahan Miah, Angie Lackenby, Omolola Akinbami, Tiina Talts, Leena Bhaw, Richard Myers, Steven Platt, Kirstin Edwards, Jonathan Hubb, Joanna Ellis, Maria Zambon
EPI_ISL_418701 hCoV-19/England/20124020402/2020 Europe / United Kingdom / England 2020-03-17 Respiratory Virus Unit, Microbiology Services Colindale, Public Health England Respiratory Virus Unit, Microbiology Services Colindale, Public Health England Monica Galiano, Shahjahan Miah, Angie Lackenby, Omolola Akinbami, Tiina Talts, Leena Bhaw, Richard Myers, Steven Platt, Kirstin Edwards, Jonathan Hubb, Joanna Ellis, Maria Zambon
EPI_ISL_418702 hCoV-19/England/20124020802/2020 Europe / United Kingdom / England 2020-03-16 Respiratory Virus Unit, Microbiology Services Colindale, Public Health England Respiratory Virus Unit,

Microbiology Services Colindale, Public Health England Monica Galiano, Shahjahan Miah, Angie Lackenby, Omolola Akinbami, Tiina Talts, Leena Bhaw, Richard Myers, Steven Platt, Kirstin Edwards, Jonathan Hubb, Joanna Ellis, Maria Zambon
EPI_ISL_418704 hCoV-19/England/20124021402/2020 Europe / United Kingdom / England 2020-03-17 Respiratory Virus Unit, Microbiology Services Colindale, Public Health England Respiratory Virus Unit, Microbiology Services Colindale, Public Health England Monica Galiano, Shahjahan Miah, Angie Lackenby, Omolola Akinbami, Tiina Talts, Leena Bhaw, Richard Myers, Steven Platt, Kirstin Edwards, Jonathan Hubb, Joanna Ellis, Maria Zambon
EPI_ISL_418706 hCoV-19/England/20124029802/2020 Europe / United Kingdom / England 2020-03-17 Respiratory Virus Unit, Microbiology Services Colindale, Public Health England Respiratory Virus Unit, Microbiology Services Colindale, Public Health England Monica Galiano, Shahjahan Miah, Angie Lackenby, Omolola Akinbami, Tiina Talts, Leena Bhaw, Richard Myers, Steven Platt, Kirstin Edwards, Jonathan Hubb, Joanna Ellis, Maria Zambon
EPI_ISL_418707 hCoV-19/England/20124030003/2020 Europe / United Kingdom / England 2020-03-17 Respiratory Virus Unit, Microbiology Services Colindale, Public Health England Respiratory Virus Unit, Microbiology Services Colindale, Public Health England Monica Galiano, Shahjahan Miah, Angie Lackenby, Omolola Akinbami, Tiina Talts, Leena Bhaw, Richard Myers, Steven Platt, Kirstin Edwards, Jonathan Hubb, Joanna Ellis, Maria Zambon
EPI_ISL_418708 hCoV-19/England/20124030802/2020 Europe / United Kingdom / England 2020-03-17 Respiratory Virus Unit, Microbiology Services Colindale, Public Health England Respiratory Virus Unit, Microbiology Services Colindale, Public Health England Monica Galiano, Shahjahan Miah, Angie Lackenby, Omolola Akinbami, Tiina Talts, Leena Bhaw, Richard Myers, Steven Platt, Kirstin Edwards, Jonathan Hubb, Joanna Ellis, Maria Zambon
EPI_ISL_418718 hCoV-19/England/20124036902/2020 Europe / United Kingdom / England 2020-03-18 Respiratory Virus Unit, Microbiology Services Colindale, Public Health England Respiratory Virus Unit, Microbiology Services Colindale, Public Health England Monica Galiano, Shahjahan Miah, Angie Lackenby, Omolola Akinbami, Tiina Talts, Leena Bhaw, Richard Myers, Steven Platt, Kirstin Edwards, Jonathan Hubb, Joanna Ellis, Maria Zambon
EPI_ISL_418720 hCoV-19/England/20124037302/2020 Europe / United Kingdom / England 2020-03-17 Respiratory Virus Unit, Microbiology Services Colindale, Public Health England Respiratory Virus Unit, Microbiology Services Colindale, Public Health England Monica Galiano, Shahjahan Miah, Angie Lackenby, Omolola Akinbami, Tiina Talts, Leena Bhaw, Richard Myers, Steven Platt, Kirstin Edwards, Jonathan Hubb, Joanna Ellis, Maria Zambon
EPI_ISL_418722 hCoV-19/England/20124048202/2020 Europe / United Kingdom / England 2020-03-17 Respiratory Virus Unit, Microbiology Services Colindale, Public Health England Respiratory Virus Unit, Microbiology Services Colindale, Public Health England Monica Galiano, Shahjahan Miah, Angie Lackenby, Omolola Akinbami, Tiina Talts, Leena Bhaw, Richard Myers, Steven Platt, Kirstin Edwards, Jonathan Hubb, Joanna Ellis, Maria Zambon
EPI_ISL_418723 hCoV-19/England/20124049402/2020 Europe / United Kingdom / England 2020-03-16 Respiratory Virus Unit, Microbiology Services Colindale, Public Health England Respiratory Virus Unit, Microbiology Services Colindale, Public Health England Monica Galiano, Shahjahan Miah, Angie Lackenby, Omolola Akinbami, Tiina Talts, Leena Bhaw, Richard Myers, Steven Platt, Kirstin Edwards, Jonathan Hubb, Joanna Ellis, Maria Zambon
EPI_ISL_418729 hCoV-19/England/20124095502/2020 Europe / United Kingdom / England 2020-03-18 Respiratory Virus Unit, Microbiology Services Colindale, Public Health England Respiratory Virus Unit, Microbiology Services Colindale, Public Health England Monica Galiano, Shahjahan Miah, Angie Lackenby, Omolola Akinbami, Tiina Talts, Leena Bhaw, Richard Myers, Steven Platt, Kirstin Edwards, Jonathan Hubb, Joanna Ellis, Maria Zambon
EPI_ISL_418733 hCoV-19/England/20124096802/2020 Europe / United Kingdom / England 2020-03-18 Respiratory Virus Unit, Microbiology Services Colindale, Public Health England Respiratory Virus Unit, Microbiology Services Colindale, Public Health England Monica Galiano, Shahjahan Miah, Angie Lackenby, Omolola Akinbami, Tiina Talts, Leena Bhaw, Richard Myers, Steven Platt, Kirstin Edwards, Jonathan Hubb, Joanna Ellis, Maria Zambon
EPI_ISL_418734 hCoV-19/England/20124097102/2020 Europe / United Kingdom / England 2020-03-13 Respiratory Virus Unit, Microbiology Services Colindale, Public Health England Respiratory Virus Unit, Microbiology Services Colindale, Public Health England Monica Galiano, Shahjahan Miah, Angie Lackenby, Omolola Akinbami, Tiina Talts, Leena Bhaw, Richard Myers, Steven Platt, Kirstin Edwards, Jonathan Hubb, Joanna Ellis, Maria Zambon
EPI_ISL_418737 hCoV-19/England/20124100002/2020 Europe / United Kingdom / England 2020-03-17 Respiratory Virus Unit, Microbiology Services Colindale, Public Health England Respiratory Virus Unit, Microbiology Services Colindale, Public Health England Monica Galiano, Shahjahan Miah, Angie Lackenby, Omolola Akinbami, Tiina Talts, Leena Bhaw, Richard Myers, Steven Platt, Kirstin Edwards, Jonathan Hubb, Joanna Ellis, Maria Zambon
EPI_ISL_419000 hCoV-19/Singapore/21/2020 Asia / Singapore 2020-02-13 National Public Health Laboratory, National Centre for Infectious Diseases National Public Health Laboratory, National Centre for Infectious Diseases Mak TM, Octavia S, Cui L, Lin RTP
EPI_ISL_419001 hCoV-19/Singapore/19/2020 Asia / Singapore 2020-03-02 National Public Health Laboratory, National Centre for Infectious Diseases National Public Health Laboratory, National Centre for Infectious Diseases Mak TM, Octavia S, Cui L, Lin RTP
EPI_ISL_421171 hCoV-19/Spain/Madrid_H12_1301/2020 Europe / Spain/ Madrid 2020-03-05 Hospital

Universitario 12 de Octubre	Hospital Universitario 12 de Octubre	Esther Viedma, Sara González, Elias Dahdouh, Raúl Recio, Fernando Lázaro, Julio García, Mª Dolores Folgueira, Jesús Mingorance, Rafael Delgado
EPI_ISL_421172	hCoV-19/Spain/Madrid_H12_1502/2020	Europe / Spain/ Madrid	2020-03-09	Hospital Universitario 12 de Octubre	Hospital Universitario 12 de Octubre	Esther Viedma, Sara González, Elias Dahdouh, Raúl Recio, Fernando Lázaro, Julio García, Mª Dolores Folgueira, Jesús Mingorance, Rafael Delgado
EPI_ISL_421173	hCoV-19/Spain/Madrid_H12_1703/2020	Europe / Spain / Madrid	2020-03-08	Hospital Universitario 12 de Octubre	Hospital Universitario 12 de Octubre	Esther Viedma, Sara González, Elias Dahdouh, Raúl Recio, Fernando Lázaro, Julio García, Mª Dolores Folgueira, Jesús Mingorance, Rafael Delgado
EPI_ISL_421174	hCoV-19/Spain/Madrid_H12_1804/2021	Europe / Spain / Madrid	2020-03-29	Hospital Universitario 12 de Octubre	Hospital Universitario 12 de Octubre	Esther Viedma, Sara González, Elias Dahdouh, Raúl Recio, Fernando Lázaro, Julio García, Mª Dolores Folgueira, Jesús Mingorance, Rafael Delgado
EPI_ISL_421175	hCoV-19/Spain/Madrid_H12_1905/2020	Europe / Spain / Madrid	2020-03-29	Hospital Universitario 12 de Octubre	Hospital Universitario 12 de Octubre	Esther Viedma, Sara González, Elias Dahdouh, Raúl Recio, Fernando Lázaro, Julio García, Mª Dolores Folgueira, Jesús Mingorance, Rafael Delgado
EPI_ISL_421176	hCoV-19/Spain/Madrid_H12_2006/2020	Europe / Spain / Madrid	2020-03-27	Hospital Universitario 12 de Octubre	Hospital Universitario 12 de Octubre	Esther Viedma, Sara González, Elias Dahdouh, Raúl Recio, Fernando Lázaro, Julio García, Mª Dolores Folgueira, Jesús Mingorance, Rafael Delgado
EPI_ISL_421177	hCoV-19/Spain/Madrid_H12_2107/2020	Europe / Spain / Madrid	2020-03-27	Hospital Universitario 12 de Octubre	Hospital Universitario 12 de Octubre	Esther Viedma, Sara González, Elias Dahdouh, Raúl Recio, Fernando Lázaro, Julio García, Mª Dolores Folgueira, Jesús Mingorance, Rafael Delgado
EPI_ISL_421178	hCoV-19/Spain/Madrid_H12_2208/2020	Europe / Spain / Madrid	2020-03-27	Hospital Universitario 12 de Octubre	Hospital Universitario 12 de Octubre	Esther Viedma, Sara González, Elias Dahdouh, Raúl Recio, Fernando Lázaro, Julio García, Mª Dolores Folgueira, Jesús Mingorance, Rafael Delgado
EPI_ISL_421179	hCoV-19/Spain/Madrid_H12_2309/2020	Europe / Spain / Madrid	2020-03-27	Hospital Universitario 12 de Octubre	Hospital Universitario 12 de Octubre	Esther Viedma, Sara González, Elias Dahdouh, Raúl Recio, Fernando Lázaro, Julio García, Mª Dolores Folgueira, Jesús Mingorance, Rafael Delgado
EPI_ISL_421180	hCoV-19/Spain/Madrid_H12_2410/2020	Europe / Spain / Madrid	2020-03-27	Hospital Universitario 12 de Octubre	Hospital Universitario 12 de Octubre	Esther Viedma, Sara González, Elias Dahdouh, Raúl Recio, Fernando Lázaro, Julio García, Mª Dolores Folgueira, Jesús Mingorance, Rafael Delgado
EPI_ISL_421182	hCoV-19/Belgium/ULG-10001/2020	Europe / Belgium / Liège	2020-03-30	Department of Clinical Microbiology	GIGA Medical Genomics	Keith Durkin, Maria Artesi, Sébastien Bontems, Raphaël Boreux, Cécile Meex, Pierrette Melin, Marie-Pierre Hayette, Vincent Bours.
EPI_ISL_421183	hCoV-19/Belgium/ULG-10003/2020	Europe / Belgium / Liège	2020-03-30	Department of Clinical Microbiology	GIGA Medical Genomics	Keith Durkin, Maria Artesi, Sébastien Bontems, Raphaël Boreux, Cécile Meex, Pierrette Melin, Marie-Pierre Hayette, Vincent Bours.
EPI_ISL_421184	hCoV-19/Belgium/ULG-10004/2020	Europe / Belgium / Liège	2020-03-31	Department of Clinical Microbiology	GIGA Medical Genomics	Keith Durkin, Maria Artesi, Sébastien Bontems, Raphaël Boreux, Cécile Meex, Pierrette Melin, Marie-Pierre Hayette, Vincent Bours.
EPI_ISL_421185	hCoV-19/Belgium/ULG-10006/2020	Europe / Belgium / Liège	2020-03-30	Department of Clinical Microbiology	GIGA Medical Genomics	Keith Durkin, Maria Artesi, Sébastien Bontems, Raphaël Boreux, Cécile Meex, Pierrette Melin, Marie-Pierre Hayette, Vincent Bours.
EPI_ISL_421186	hCoV-19/Belgium/ULG-10007/2020	Europe / Belgium / Liège	2020-03-30	Department of Clinical Microbiology	GIGA Medical Genomics	Keith Durkin, Maria Artesi, Sébastien Bontems, Raphaël Boreux, Cécile Meex, Pierrette Melin, Marie-Pierre Hayette, Vincent Bours.
EPI_ISL_421187	hCoV-19/Belgium/ULG-10008/2020	Europe / Belgium / Liège	2020-03-30	Department of Clinical Microbiology	GIGA Medical Genomics	Keith Durkin, Maria Artesi, Sébastien Bontems, Raphaël Boreux, Cécile Meex, Pierrette Melin, Marie-Pierre Hayette, Vincent Bours.
EPI_ISL_421188	hCoV-19/Belgium/ULG-10009/2020	Europe / Belgium / Liège	2020-03-30	Department of Clinical Microbiology	GIGA Medical Genomics	Keith Durkin, Maria Artesi, Sébastien Bontems, Raphaël Boreux, Cécile Meex, Pierrette Melin, Marie-Pierre Hayette, Vincent Bours.
EPI_ISL_421189	hCoV-19/Belgium/ULG-10011/2020	Europe / Belgium / Liège	2020-03-30	Department of Clinical Microbiology	GIGA Medical Genomics	Keith Durkin, Maria Artesi, Sébastien Bontems, Raphaël Boreux, Cécile Meex, Pierrette Melin, Marie-Pierre Hayette, Vincent Bours.
EPI_ISL_421190	hCoV-19/Belgium/ULG-10012/2020	Europe / Belgium / Liège	2020-03-31	Department of Clinical Microbiology	GIGA Medical Genomics	Keith Durkin, Maria Artesi, Sébastien Bontems, Raphaël Boreux, Cécile Meex, Pierrette Melin, Marie-Pierre Hayette, Vincent Bours.
EPI_ISL_421191	hCoV-19/Belgium/ULG-10013/2020	Europe / Belgium / Liège	2020-03-30	Department of Clinical Microbiology	GIGA Medical Genomics	Keith Durkin, Maria Artesi, Sébastien Bontems, Raphaël Boreux, Cécile Meex, Pierrette Melin, Marie-Pierre Hayette, Vincent Bours.
EPI_ISL_421192	hCoV-19/Belgium/ULG-10014/2020	Europe / Belgium / Liège	2020-03-30	Department of Clinical Microbiology	GIGA Medical Genomics	Keith Durkin, Maria Artesi, Sébastien Bontems, Raphaël Boreux, Cécile Meex, Pierrette Melin, Marie-Pierre Hayette, Vincent Bours.
EPI_ISL_421193	hCoV-19/Belgium/ULG-10015/2020	Europe / Belgium / Liège	2020-03-31	Department of Clinical Microbiology	GIGA Medical Genomics	Keith Durkin, Maria Artesi, Sébastien Bontems, Raphaël Boreux, Cécile Meex, Pierrette Melin, Marie-Pierre Hayette, Vincent Bours.
EPI_ISL_421194	hCoV-19/Belgium/ULG-10016/2020	Europe / Belgium / Liège	2020-03-30	Department of Clinical Microbiology	GIGA Medical Genomics	Keith Durkin, Maria Artesi, Sébastien Bontems, Raphaël Boreux, Cécile Meex, Pierrette Melin, Marie-Pierre Hayette, Vincent Bours.
EPI_ISL_421195	hCoV-19/Belgium/ULG-10017/2020	Europe / Belgium / Liège	2020-03-30	Department of Clinical Microbiology	GIGA Medical Genomics	Keith Durkin, Maria Artesi, Sébastien Bontems, Raphaël Boreux,

Cécile Meex, Pierrette Melin, Marie-Pierre Hayette, Vincent Bours.
EPI_ISL_421196 hCoV-19/Belgium/ULG-10018/2020 Europe / Belgium / Liège 2020-03-30 Department of Clinical Microbiology GIGA Medical Genomics Keith Durkin, Maria Artesi, Sébastien Bontems, Raphaël Boreux, Cécile Meex, Pierrette Melin, Marie-Pierre Hayette, Vincent Bours.
EPI_ISL_421198 hCoV-19/Belgium/ULG-10024/2020 Europe / Belgium / Liège 2020-03-30 Department of Clinical Microbiology GIGA Medical Genomics Keith Durkin, Maria Artesi, Sébastien Bontems, Raphaël Boreux, Cécile Meex, Pierrette Melin, Marie-Pierre Hayette, Vincent Bours.
EPI_ISL_421200 hCoV-19/Belgium/ULG-10026/2020 Europe / Belgium / Liège 2020-03-31 Department of Clinical Microbiology GIGA Medical Genomics Keith Durkin, Maria Artesi, Sébastien Bontems, Raphaël Boreux, Cécile Meex, Pierrette Melin, Marie-Pierre Hayette, Vincent Bours.
EPI_ISL_421201 hCoV-19/Belgium/ULG-10027/2020 Europe / Belgium / Liège 2020-03-31 Department of Clinical Microbiology GIGA Medical Genomics Keith Durkin, Maria Artesi, Sébastien Bontems, Raphaël Boreux, Cécile Meex, Pierrette Melin, Marie-Pierre Hayette, Vincent Bours.
EPI_ISL_421202 hCoV-19/Belgium/ULG-10028/2020 Europe / Belgium / Liège 2020-03-30 Department of Clinical Microbiology GIGA Medical Genomics Keith Durkin, Maria Artesi, Sébastien Bontems, Raphaël Boreux, Cécile Meex, Pierrette Melin, Marie-Pierre Hayette, Vincent Bours.
EPI_ISL_421203 hCoV-19/Belgium/ULG-10029/2020 Europe / Belgium / Liège 2020-03-30 Department of Clinical Microbiology GIGA Medical Genomics Keith Durkin, Maria Artesi, Sébastien Bontems, Raphaël Boreux, Cécile Meex, Pierrette Melin, Marie-Pierre Hayette, Vincent Bours.
EPI_ISL_421204 hCoV-19/Belgium/ULG-10030/2020 Europe / Belgium / Liège 2020-03-31 Department of Clinical Microbiology GIGA Medical Genomics Keith Durkin, Maria Artesi, Sébastien Bontems, Raphaël Boreux, Cécile Meex, Pierrette Melin, Marie-Pierre Hayette, Vincent Bours.
EPI_ISL_421205 hCoV-19/Belgium/ULG-10031/2020 Europe / Belgium / Liège 2020-03-31 Department of Clinical Microbiology GIGA Medical Genomics Keith Durkin, Maria Artesi, Sébastien Bontems, Raphaël Boreux, Cécile Meex, Pierrette Melin, Marie-Pierre Hayette, Vincent Bours.
EPI_ISL_421206 hCoV-19/Belgium/ULG-10032/2020 Europe / Belgium / Liège 2020-03-31 Department of Clinical Microbiology GIGA Medical Genomics Keith Durkin, Maria Artesi, Sébastien Bontems, Raphaël Boreux, Cécile Meex, Pierrette Melin, Marie-Pierre Hayette, Vincent Bours.
EPI_ISL_421207 hCoV-19/Belgium/ULG-10033/2020 Europe / Belgium / Liège 2020-03-31 Department of Clinical Microbiology GIGA Medical Genomics Keith Durkin, Maria Artesi, Sébastien Bontems, Raphaël Boreux, Cécile Meex, Pierrette Melin, Marie-Pierre Hayette, Vincent Bours.
EPI_ISL_421210 hCoV-19/Belgium/ULG-10043/2020 Europe / Belgium / Liège 2020-03-31 Department of Clinical Microbiology GIGA Medical Genomics Keith Durkin, Maria Artesi, Sébastien Bontems, Raphaël Boreux, Cécile Meex, Pierrette Melin, Marie-Pierre Hayette, Vincent Bours.
EPI_ISL_421212 hCoV-19/Belgium/ULG-10045/2020 Europe / Belgium / Liège 2020-03-31 Department of Clinical Microbiology GIGA Medical Genomics Keith Durkin, Maria Artesi, Sébastien Bontems, Raphaël Boreux, Cécile Meex, Pierrette Melin, Marie-Pierre Hayette, Vincent Bours.
EPI_ISL_421213 hCoV-19/Belgium/ULG-10046/2020 Europe / Belgium / Liège 2020-03-31 Department of Clinical Microbiology GIGA Medical Genomics Keith Durkin, Maria Artesi, Sébastien Bontems, Raphaël Boreux, Cécile Meex, Pierrette Melin, Marie-Pierre Hayette, Vincent Bours.
EPI_ISL_421214 hCoV-19/Belgium/ULG-10047/2020 Europe / Belgium / Liège 2020-03-30 Department of Clinical Microbiology GIGA Medical Genomics Keith Durkin, Maria Artesi, Sébastien Bontems, Raphaël Boreux, Cécile Meex, Pierrette Melin, Marie-Pierre Hayette, Vincent Bours.
EPI_ISL_421221 hCoV-19/Hangzhou/HZCDC6789/2020 Asia / China / Hangzhou 2020-03-15 Hangzhou Center for Diseases Control and Prevention Hangzhou Center for Diseases Control and Prevention Jun Li, Haoqiu Wang, Lingfeng Mao, Hua Yu, Xinfen Yu, Zhou Sun, Xin Qian, Shuchang Chen, Junfang Chen, Xuchu Wang
EPI_ISL_421222 hCoV-19/Hangzhou/HZCDC6706/2020 Asia / China / Hangzhou 2020-03-14 Hangzhou Center for Diseases Control and Prevention Hangzhou Center for Diseases Control and Prevention Jun Li, Haoqiu Wang, Lingfeng Mao, Hua Yu, Xinfen Yu, Zhou Sun, Xin Qian, Shuchang Chen, Junfang Chen, Xuchu Wang
EPI_ISL_421224 hCoV-19/Hangzhou/HZCDC0162/2020 Asia / China / Hangzhou 2020-01-23 Hangzhou Center for Diseases Control and Prevention Hangzhou Center for Diseases Control and Prevention Jun Li, Haoqiu Wang, Lingfeng Mao, Hua Yu, Xinfen Yu, Zhou Sun, Xin Qian, Shuchang Chen, Junfang Chen, Xuchu Wang
EPI_ISL_421300 hCoV-19/USA/WI-UW-42/2020 North America / USA / Wisconsin / Madison 2020-03-18 University of Wisconsin-Madison AIDS Vaccine Research Laboratories University of Wisconsin-Madison AIDS Vaccine Research Laboratories Gage Moreno, Katarina Braun, et al. AIDS Vaccine Research Laboratories
EPI_ISL_421301 hCoV-19/USA/WI-UW-43/2020 North America / USA / Wisconsin / Waunakee 2020-03-19 University of Wisconsin-Madison AIDS Vaccine Research Laboratories University of Wisconsin-Madison AIDS Vaccine Research Laboratories Gage Moreno, Katarina Braun, et al. AIDS Vaccine Research Laboratories
EPI_ISL_421302 hCoV-19/USA/WI-UW-44/2020 North America / USA / Wisconsin / Madison 2020-03-17 University of Wisconsin-Madison AIDS Vaccine Research Laboratories University of Wisconsin-Madison AIDS Vaccine Research Laboratories Gage Moreno, Katarina Braun, et al. AIDS Vaccine Research Laboratories
EPI_ISL_421304 hCoV-19/USA/WI-UW-46/2020 North America / USA / Wisconsin / Belleville 2020-03-22 University of Wisconsin-Madison AIDS Vaccine Research Laboratories University of Wisconsin-Madison AIDS Vaccine Research Laboratories Gage Moreno, Katarina Braun, et al. AIDS Vaccine Research Laboratories
EPI_ISL_421305 hCoV-19/USA/WI-UW-47/2020 North America / USA / Wisconsin / Chippewa Falls 2020-03-19 University of Wisconsin-Madison AIDS Vaccine Research Laboratories University of Wisconsin-Madison AIDS Vaccine Research Laboratories Gage Moreno, Katarina Braun, et al. AIDS Vaccine Research Laboratories
EPI_ISL_421306 hCoV-19/USA/WI-UW-48/2020 North America / USA / Wisconsin / DeForest 2020-03-25 University of Wisconsin-Madison AIDS Vaccine Research Laboratories University of Wisconsin-Madison AIDS Vaccine Research Laboratories Gage Moreno, Katarina Braun, et al. AIDS Vaccine Research Laboratories

EPI_ISL_421307 hCoV-19/USA/WI-UW-49/2020 North America / USA / Wisconsin / Sun Prarie 2020-03-25
University of Wisconsin-Madison AIDS Vaccine Research Laboratories University of Wisconsin-Madison AIDS
Vaccine Research Laboratories Gage Moreno, Katarina Braun, et al. AIDS Vaccine Research Laboratories
EPI_ISL_421308 hCoV-19/USA/WI-UW-50/2020 North America / USA / Wisconsin / Brooklyn 2020-03-25
University of Wisconsin-Madison AIDS Vaccine Research Laboratories University of Wisconsin-Madison AIDS
Vaccine Research Laboratories Gage Moreno, Katarina Braun, et al. AIDS Vaccine Research Laboratories
EPI_ISL_421309 hCoV-19/USA/WI-UW-51/2020 North America / USA / Wisconsin / Madison 2020-03-20
University of Wisconsin-Madison AIDS Vaccine Research Laboratories University of Wisconsin-Madison AIDS
Vaccine Research Laboratories Gage Moreno, Katarina Braun, et al. AIDS Vaccine Research Laboratories
EPI_ISL_421310 hCoV-19/USA/WI-UW-52/2020 North America / USA / Wisconsin / Madison 2020-03-18
University of Wisconsin-Madison AIDS Vaccine Research Laboratories University of Wisconsin-Madison AIDS
Vaccine Research Laboratories Gage Moreno, Katarina Braun, et al. AIDS Vaccine Research Laboratories
EPI_ISL_421311 hCoV-19/USA/WI-UW-53/2020 North America / USA / Wisconsin / Madison 2020-03-18
University of Wisconsin-Madison AIDS Vaccine Research Laboratories University of Wisconsin-Madison AIDS
Vaccine Research Laboratories Gage Moreno, Katarina Braun, et al. AIDS Vaccine Research Laboratories
EPI_ISL_421312 hCoV-19/USA/WI-UW-54/2020 North America / USA / Wisconsin / Monona 2020-03-20
University of Wisconsin-Madison AIDS Vaccine Research Laboratories University of Wisconsin-Madison AIDS
Vaccine Research Laboratories Gage Moreno, Katarina Braun, et al. AIDS Vaccine Research Laboratories
EPI_ISL_421313 hCoV-19/USA/WI-UW-55/2020 North America / USA / Wisconsin / Madison 2020-03-23
University of Wisconsin-Madison AIDS Vaccine Research Laboratories University of Wisconsin-Madison AIDS
Vaccine Research Laboratories Gage Moreno, Katarina Braun, et al. AIDS Vaccine Research Laboratories
EPI_ISL_421314 hCoV-19/USA/WI-UW-56/2020 North America / USA / Wisconsin / Waunakee 2020-03-18
University of Wisconsin-Madison AIDS Vaccine Research Laboratories University of Wisconsin-Madison AIDS
Vaccine Research Laboratories Gage Moreno, Katarina Braun, et al. AIDS Vaccine Research Laboratories
EPI_ISL_421315 hCoV-19/USA/WI-UW-57/2020 North America / USA / Wisconsin / Blanchardville 2020-
03-19 University of Wisconsin-Madison AIDS Vaccine Research Laboratories University of Wisconsin-Madison
AIDS Vaccine Research Laboratories Gage Moreno, Katarina Braun, et al. AIDS Vaccine Research Laboratories
EPI_ISL_421316 hCoV-19/USA/WI-UW-58/2020 North America / USA / Wisconsin / Madison 2020-03-23
University of Wisconsin-Madison AIDS Vaccine Research Laboratories University of Wisconsin-Madison AIDS
Vaccine Research Laboratories Gage Moreno, Katarina Braun, et al. AIDS Vaccine Research Laboratories
EPI_ISL_421317 hCoV-19/USA/WI-UW-59/2020 North America / USA / Wisconsin / DeForest 2020-03-24
University of Wisconsin-Madison AIDS Vaccine Research Laboratories University of Wisconsin-Madison AIDS
Vaccine Research Laboratories Gage Moreno, Katarina Braun, et al. AIDS Vaccine Research Laboratories
EPI_ISL_421318 hCoV-19/USA/WI-UW-60/2020 North America / USA / Wisconsin / Madison 2020-03-19
University of Wisconsin-Madison AIDS Vaccine Research Laboratories University of Wisconsin-Madison AIDS
Vaccine Research Laboratories Gage Moreno, Katarina Braun, et al. AIDS Vaccine Research Laboratories
EPI_ISL_421319 hCoV-19/USA/WI-UW-61/2020 North America / USA / Wisconsin / Stoughton 2020-03-23
University of Wisconsin-Madison AIDS Vaccine Research Laboratories University of Wisconsin-Madison AIDS
Vaccine Research Laboratories Gage Moreno, Katarina Braun, et al. AIDS Vaccine Research Laboratories
EPI_ISL_421320 hCoV-19/USA/WI-UW-62/2020 North America / USA / Wisconsin / Madison 2020-03-25
University of Wisconsin-Madison AIDS Vaccine Research Laboratories University of Wisconsin-Madison AIDS
Vaccine Research Laboratories Gage Moreno, Katarina Braun, et al. AIDS Vaccine Research Laboratories
EPI_ISL_421321 hCoV-19/USA/WI-UW-63/2020 North America / USA / Wisconsin / Sun Prarie 2020-03-24
University of Wisconsin-Madison AIDS Vaccine Research Laboratories University of Wisconsin-Madison AIDS
Vaccine Research Laboratories Gage Moreno, Katarina Braun, et al. AIDS Vaccine Research Laboratories
EPI_ISL_421322 hCoV-19/USA/WI-UW-64/2020 North America / USA / Wisconsin / Verona 2020-03-24
University of Wisconsin-Madison AIDS Vaccine Research Laboratories University of Wisconsin-Madison AIDS
Vaccine Research Laboratories Gage Moreno, Katarina Braun, et al. AIDS Vaccine Research Laboratories
EPI_ISL_421323 hCoV-19/USA/WI-UW-65/2020 North America / USA / Wisconsin / Madison 2020-03-22
University of Wisconsin-Madison AIDS Vaccine Research Laboratories University of Wisconsin-Madison AIDS
Vaccine Research Laboratories Gage Moreno, Katarina Braun, et al. AIDS Vaccine Research Laboratories
EPI_ISL_422435 hCoV-19/Singapore/42/2020 Asia / Singapore 2020-03-31 National Public Health
Laboratory, National Centre for Infectious Diseases National Public Health Laboratory, National Centre for
Infectious Diseases Mak TM, Octavia S, Cui L, Lin RTP
EPI_ISL_422437 hCoV-19/Italy/VR_20COV21-26/2020 Europe / Italy / Veneto / Verona 2020-03-25
ULSS9 Distretto di Bussolengo Istituto Zooprofilattico Sperimentale delle Venezie Adelaide Milani,
Alessia Schivo, Annalisa Salviato, Erika Giorgia Quaranta, Gianpiero Zamperin, Ambra Pastori, Bianca Zecchin,
Alice Fusaro, Calogero Terregino, Antonia Ricci
EPI_ISL_422438 hCoV-19/Italy/VR_20COV21-37/2020 Europe / Italy / Veneto / Verona 2020-03-25
ULSS9 Distretto di Bussolengo Istituto Zooprofilattico Sperimentale delle Venezie Adelaide Milani,
Alessia Schivo, Annalisa Salviato, Erika Giorgia Quaranta, Gianpiero Zamperin, Ambra Pastori, Bianca Zecchin,
Alice Fusaro, Calogero Terregino, Antonia Ricci
EPI_ISL_422453 hCoV-19/USA/WI-GMF-00498/2020 North America / USA / Wisconsin / Adams County 2020-03-31
Gundersen Molecular Diagnostics Laboratory Kabara Cancer Research Institute Craig S. Richmond &
Paraic A. Kenny
EPI_ISL_422459 hCoV-19/USA/WI-GMF-00557/2020 North America / USA / Wisconsin / Monroe County 2020-04-02
Gundersen Molecular Diagnostics Laboratory Kabara Cancer Research Institute Craig S. Richmond &
Paraic A. Kenny
EPI_ISL_422461 hCoV-19/USA/WI-GMF-00707/2020 North America / USA / Iowa / Allamakee County 2020-04-06
Gundersen Molecular Diagnostics Laboratory Kabara Cancer Research Institute Craig S. Richmond,

Paraic A. Kenny
EPI_ISL_422462 hCoV-19/USA/WI-GMF-00744/2020 North America / USA / Iowa / Winneshiek County 2020-04-03 Gundersen Molecular Diagnostics Laboratory Kabara Cancer Research Institute Craig S. Richmond, Paraic A. Kenny
EPI_ISL_422463 hCoV-19/USA/WI-GMF-00588/2020 North America / USA / Minnesota / Winona County 2020-04-06 Gundersen Molecular Diagnostics Laboratory Kabara Cancer Research Institute Craig S. Richmond, Paraic A. Kenny
EPI_ISL_422465 hCoV-19/USA/WI-GMF-00441/2020 North America / USA / Wisconsin / Jackson County 2020-03-28 Gundersen Molecular Diagnostics Laboratory Kabara Cancer Research Institute Craig S. Richmond, Paraic A. Kenny
EPI_ISL_422488 hCoV-19/USA/NY-PV09045/2020 North America / USA / New York / Manhattan 2020-03-21 MSHS Clinical Microbiology Laboratories MSHS Pathogen Surveillance Program Ana S. Gonzalez-Reiche, Mitchell Sullivan, Ajay Obla, Gopi Patel, Emilia Sordillo, Melissa Gitman, Alberto Paniz-mondolfi, Matthew Hernandez, Shelcie Fabre, Jose Polanco, Zenab Khan, Bremy Albuquerque, Jayeeta Dutta, Juan Soto, Shwetha Sridhar Hara, Ying-Chih Wang, Melissa Smith, Robert Sebra, Lisa Miorin, Wen-chun Liu, Randy Albrecht, Judith Aberg, Florian Krammer, Adolfo Garcia-Sarstre, Viviana Simon, Harm van Bakel
EPI_ISL_422489 hCoV-19/USA/NY-PV09057/2020 North America / USA / New York / Brooklyn 2020-03-21 MSHS Clinical Microbiology Laboratories MSHS Pathogen Surveillance Program Ana S. Gonzalez-Reiche, Mitchell Sullivan, Ajay Obla, Gopi Patel, Emilia Sordillo, Melissa Gitman, Alberto Paniz-mondolfi, Matthew Hernandez, Shelcie Fabre, Jose Polanco, Zenab Khan, Bremy Albuquerque, Jayeeta Dutta, Juan Soto, Shwetha Sridhar Hara, Ying-Chih Wang, Melissa Smith, Robert Sebra, Lisa Miorin, Wen-chun Liu, Randy Albrecht, Judith Aberg, Florian Krammer, Adolfo Garcia-Sarstre, Viviana Simon, Harm van Bakel
EPI_ISL_422490 hCoV-19/USA/NY-PV09069/2020 North America / USA / New York / Brooklyn 2020-03-20 MSHS Clinical Microbiology Laboratories MSHS Pathogen Surveillance Program Ana S. Gonzalez-Reiche, Mitchell Sullivan, Ajay Obla, Gopi Patel, Emilia Sordillo, Melissa Gitman, Alberto Paniz-mondolfi, Matthew Hernandez, Shelcie Fabre, Jose Polanco, Zenab Khan, Bremy Albuquerque, Jayeeta Dutta, Juan Soto, Shwetha Sridhar Hara, Ying-Chih Wang, Melissa Smith, Robert Sebra, Lisa Miorin, Wen-chun Liu, Randy Albrecht, Judith Aberg, Florian Krammer, Adolfo Garcia-Sarstre, Viviana Simon, Harm van Bakel
EPI_ISL_422491 hCoV-19/USA/NY-PV09115/2020 North America / USA / New York / Nassau County 2020-03-19 MSHS Clinical Microbiology Laboratories MSHS Pathogen Surveillance Program Ana S. Gonzalez-Reiche, Mitchell Sullivan, Ajay Obla, Gopi Patel, Emilia Sordillo, Melissa Gitman, Alberto Paniz-mondolfi, Matthew Hernandez, Shelcie Fabre, Jose Polanco, Zenab Khan, Bremy Albuquerque, Jayeeta Dutta, Juan Soto, Shwetha Sridhar Hara, Ying-Chih Wang, Melissa Smith, Robert Sebra, Lisa Miorin, Wen-chun Liu, Randy Albrecht, Judith Aberg, Florian Krammer, Adolfo Garcia-Sarstre, Viviana Simon, Harm van Bakel
EPI_ISL_422492 hCoV-19/USA/NY-PV09116/2020 North America / USA / New York / Nassau County 2020-03-19 MSHS Clinical Microbiology Laboratories MSHS Pathogen Surveillance Program Ana S. Gonzalez-Reiche, Mitchell Sullivan, Ajay Obla, Gopi Patel, Emilia Sordillo, Melissa Gitman, Alberto Paniz-mondolfi, Matthew Hernandez, Shelcie Fabre, Jose Polanco, Zenab Khan, Bremy Albuquerque, Jayeeta Dutta, Juan Soto, Shwetha Sridhar Hara, Ying-Chih Wang, Melissa Smith, Robert Sebra, Lisa Miorin, Wen-chun Liu, Randy Albrecht, Judith Aberg, Florian Krammer, Adolfo Garcia-Sarstre, Viviana Simon, Harm van Bakel
EPI_ISL_422494 hCoV-19/USA/NY-PV09119/2020 North America / USA / New York / Manhattan 2020-03-19 MSHS Clinical Microbiology Laboratories MSHS Pathogen Surveillance Program Ana S. Gonzalez-Reiche, Mitchell Sullivan, Ajay Obla, Gopi Patel, Emilia Sordillo, Melissa Gitman, Alberto Paniz-mondolfi, Matthew Hernandez, Shelcie Fabre, Jose Polanco, Zenab Khan, Bremy Albuquerque, Jayeeta Dutta, Juan Soto, Shwetha Sridhar Hara, Ying-Chih Wang, Melissa Smith, Robert Sebra, Lisa Miorin, Wen-chun Liu, Randy Albrecht, Judith Aberg, Florian Krammer, Adolfo Garcia-Sarstre, Viviana Simon, Harm van Bakel
EPI_ISL_422495 hCoV-19/USA/NY-PV09120/2020 North America / USA / New York / Brooklyn 2020-03-19 MSHS Clinical Microbiology Laboratories MSHS Pathogen Surveillance Program Ana S. Gonzalez-Reiche, Mitchell Sullivan, Ajay Obla, Gopi Patel, Emilia Sordillo, Melissa Gitman, Alberto Paniz-mondolfi, Matthew Hernandez, Shelcie Fabre, Jose Polanco, Zenab Khan, Bremy Albuquerque, Jayeeta Dutta, Juan Soto, Shwetha Sridhar Hara, Ying-Chih Wang, Melissa Smith, Robert Sebra, Lisa Miorin, Wen-chun Liu, Randy Albrecht, Judith Aberg, Florian Krammer, Adolfo Garcia-Sarstre, Viviana Simon, Harm van Bakel
EPI_ISL_422496 hCoV-19/USA/NY-PV09121/2020 North America / USA / New York / Nassau County 2020-03-19 MSHS Clinical Microbiology Laboratories MSHS Pathogen Surveillance Program Ana S. Gonzalez-Reiche, Mitchell Sullivan, Ajay Obla, Gopi Patel, Emilia Sordillo, Melissa Gitman, Alberto Paniz-mondolfi, Matthew Hernandez, Shelcie Fabre, Jose Polanco, Zenab Khan, Bremy Albuquerque, Jayeeta Dutta, Juan Soto, Shwetha Sridhar Hara, Ying-Chih Wang, Melissa Smith, Robert Sebra, Lisa Miorin, Wen-chun Liu, Randy Albrecht, Judith Aberg, Florian Krammer, Adolfo Garcia-Sarstre, Viviana Simon, Harm van Bakel
EPI_ISL_422497 hCoV-19/USA/NY-PV09122/2020 North America / USA / New York / Nassau County 2020-03-19 MSHS Clinical Microbiology Laboratories MSHS Pathogen Surveillance Program Ana S. Gonzalez-Reiche, Mitchell Sullivan, Ajay Obla, Gopi Patel, Emilia Sordillo, Melissa Gitman, Alberto Paniz-mondolfi, Matthew Hernandez, Shelcie Fabre, Jose Polanco, Zenab Khan, Bremy Albuquerque, Jayeeta Dutta, Juan Soto, Shwetha Sridhar Hara, Ying-Chih Wang, Melissa Smith, Robert Sebra, Lisa Miorin, Wen-chun Liu, Randy Albrecht, Judith Aberg, Florian Krammer, Adolfo Garcia-Sarstre, Viviana Simon, Harm van Bakel
EPI_ISL_422498 hCoV-19/USA/NY-PV09123/2020 North America / USA / New York / Brooklyn 2020-03-19 MSHS Clinical Microbiology Laboratories MSHS Pathogen Surveillance Program Ana S. Gonzalez-Reiche, Mitchell Sullivan, Ajay Obla, Gopi Patel, Emilia Sordillo, Melissa Gitman, Alberto Paniz-mondolfi, Matthew Hernandez, Shelcie Fabre, Jose Polanco, Zenab Khan, Bremy Albuquerque, Jayeeta Dutta, Juan Soto, Shwetha Sridhar Hara, Ying-Chih Wang, Melissa Smith, Robert Sebra, Lisa Miorin, Wen-chun Liu, Randy Albrecht, Judith Aberg, Florian Krammer, Adolfo Garcia-Sarstre, Viviana Simon, Harm van Bakel

EPI_ISL_422499 hCoV-19/USA/NY-PV09125/2020 North America / USA / New York / Queens 2020-03-19 MSHS Clinical Microbiology Laboratories MSHS Pathogen Surveillance Program Ana S. Gonzalez-Reiche, Mitchell Sullivan, Ajay Obla, Gopi Patel, Emilia Sordillo, Melissa Gitman, Alberto Paniz-mondolfi, Matthew Hernandez, Shelcie Fabre, Jose Polanco, Zenab Khan, Bremy Albuquerque, Jayeeta Dutta, Juan Soto, Shwetha Sridhar Hara, Ying-Chih Wang, Melissa Smith, Robert Sebra, Lisa Miorin, Wen-chun Liu, Randy Albrecht, Judith Aberg, Florian Krammer, Adolfo Garcia-Sarstre, Viviana Simon, Harm van Bakel
EPI_ISL_422500 hCoV-19/USA/NY-PV09126/2020 North America / USA / New York / Nassau County 2020-03-19 MSHS Clinical Microbiology Laboratories MSHS Pathogen Surveillance Program Ana S. Gonzalez-Reiche, Mitchell Sullivan, Ajay Obla, Gopi Patel, Emilia Sordillo, Melissa Gitman, Alberto Paniz-mondolfi, Matthew Hernandez, Shelcie Fabre, Jose Polanco, Zenab Khan, Bremy Albuquerque, Jayeeta Dutta, Juan Soto, Shwetha Sridhar Hara, Ying-Chih Wang, Melissa Smith, Robert Sebra, Lisa Miorin, Wen-chun Liu, Randy Albrecht, Judith Aberg, Florian Krammer, Adolfo Garcia-Sarstre, Viviana Simon, Harm van Bakel
EPI_ISL_422501 hCoV-19/USA/NY-PV09127/2020 North America / USA / New York / Brooklyn 2020-03-19 MSHS Clinical Microbiology Laboratories MSHS Pathogen Surveillance Program Ana S. Gonzalez-Reiche, Mitchell Sullivan, Ajay Obla, Gopi Patel, Emilia Sordillo, Melissa Gitman, Alberto Paniz-mondolfi, Matthew Hernandez, Shelcie Fabre, Jose Polanco, Zenab Khan, Bremy Albuquerque, Jayeeta Dutta, Juan Soto, Shwetha Sridhar Hara, Ying-Chih Wang, Melissa Smith, Robert Sebra, Lisa Miorin, Wen-chun Liu, Randy Albrecht, Judith Aberg, Florian Krammer, Adolfo Garcia-Sarstre, Viviana Simon, Harm van Bakel
EPI_ISL_422502 hCoV-19/USA/NY-PV09128/2020 North America / USA / New York / Manhattan 2020-03-19 MSHS Clinical Microbiology Laboratories MSHS Pathogen Surveillance Program Ana S. Gonzalez-Reiche, Mitchell Sullivan, Ajay Obla, Gopi Patel, Emilia Sordillo, Melissa Gitman, Alberto Paniz-mondolfi, Matthew Hernandez, Shelcie Fabre, Jose Polanco, Zenab Khan, Bremy Albuquerque, Jayeeta Dutta, Juan Soto, Shwetha Sridhar Hara, Ying-Chih Wang, Melissa Smith, Robert Sebra, Lisa Miorin, Wen-chun Liu, Randy Albrecht, Judith Aberg, Florian Krammer, Adolfo Garcia-Sarstre, Viviana Simon, Harm van Bakel
EPI_ISL_422503 hCoV-19/USA/NY-PV09129/2020 North America / USA / New York / Nassau County 2020-03-19 MSHS Clinical Microbiology Laboratories MSHS Pathogen Surveillance Program Ana S. Gonzalez-Reiche, Mitchell Sullivan, Ajay Obla, Gopi Patel, Emilia Sordillo, Melissa Gitman, Alberto Paniz-mondolfi, Matthew Hernandez, Shelcie Fabre, Jose Polanco, Zenab Khan, Bremy Albuquerque, Jayeeta Dutta, Juan Soto, Shwetha Sridhar Hara, Ying-Chih Wang, Melissa Smith, Robert Sebra, Lisa Miorin, Wen-chun Liu, Randy Albrecht, Judith Aberg, Florian Krammer, Adolfo Garcia-Sarstre, Viviana Simon, Harm van Bakel
EPI_ISL_422504 hCoV-19/USA/NY-PV09130/2020 North America / USA / New York / Manhattan 2020-03-19 MSHS Clinical Microbiology Laboratories MSHS Pathogen Surveillance Program Ana S. Gonzalez-Reiche, Mitchell Sullivan, Ajay Obla, Gopi Patel, Emilia Sordillo, Melissa Gitman, Alberto Paniz-mondolfi, Matthew Hernandez, Shelcie Fabre, Jose Polanco, Zenab Khan, Bremy Albuquerque, Jayeeta Dutta, Juan Soto, Shwetha Sridhar Hara, Ying-Chih Wang, Melissa Smith, Robert Sebra, Lisa Miorin, Wen-chun Liu, Randy Albrecht, Judith Aberg, Florian Krammer, Adolfo Garcia-Sarstre, Viviana Simon, Harm van Bakel
EPI_ISL_422505 hCoV-19/USA/NY-PV09132/2020 North America / USA / New York / Manhattan 2020-03-19 MSHS Clinical Microbiology Laboratories MSHS Pathogen Surveillance Program Ana S. Gonzalez-Reiche, Mitchell Sullivan, Ajay Obla, Gopi Patel, Emilia Sordillo, Melissa Gitman, Alberto Paniz-mondolfi, Matthew Hernandez, Shelcie Fabre, Jose Polanco, Zenab Khan, Bremy Albuquerque, Jayeeta Dutta, Juan Soto, Shwetha Sridhar Hara, Ying-Chih Wang, Melissa Smith, Robert Sebra, Lisa Miorin, Wen-chun Liu, Randy Albrecht, Judith Aberg, Florian Krammer, Adolfo Garcia-Sarstre, Viviana Simon, Harm van Bakel
EPI_ISL_422506 hCoV-19/USA/NY-PV09134/2020 North America / USA / New York / Manhattan 2020-03-19 MSHS Clinical Microbiology Laboratories MSHS Pathogen Surveillance Program Ana S. Gonzalez-Reiche, Mitchell Sullivan, Ajay Obla, Gopi Patel, Emilia Sordillo, Melissa Gitman, Alberto Paniz-mondolfi, Matthew Hernandez, Shelcie Fabre, Jose Polanco, Zenab Khan, Bremy Albuquerque, Jayeeta Dutta, Juan Soto, Shwetha Sridhar Hara, Ying-Chih Wang, Melissa Smith, Robert Sebra, Lisa Miorin, Wen-chun Liu, Randy Albrecht, Judith Aberg, Florian Krammer, Adolfo Garcia-Sarstre, Viviana Simon, Harm van Bakel
EPI_ISL_422507 hCoV-19/USA/NY-PV09135/2020 North America / USA / New York / Nassau County 2020-03-20 MSHS Clinical Microbiology Laboratories MSHS Pathogen Surveillance Program Ana S. Gonzalez-Reiche, Mitchell Sullivan, Ajay Obla, Gopi Patel, Emilia Sordillo, Melissa Gitman, Alberto Paniz-mondolfi, Matthew Hernandez, Shelcie Fabre, Jose Polanco, Zenab Khan, Bremy Albuquerque, Jayeeta Dutta, Juan Soto, Shwetha Sridhar Hara, Ying-Chih Wang, Melissa Smith, Robert Sebra, Lisa Miorin, Wen-chun Liu, Randy Albrecht, Judith Aberg, Florian Krammer, Adolfo Garcia-Sarstre, Viviana Simon, Harm van Bakel
EPI_ISL_422508 hCoV-19/USA/NY-PV09137/2020 North America / USA / New York / Nassau County 2020-03-20 MSHS Clinical Microbiology Laboratories MSHS Pathogen Surveillance Program Ana S. Gonzalez-Reiche, Mitchell Sullivan, Ajay Obla, Gopi Patel, Emilia Sordillo, Melissa Gitman, Alberto Paniz-mondolfi, Matthew Hernandez, Shelcie Fabre, Jose Polanco, Zenab Khan, Bremy Albuquerque, Jayeeta Dutta, Juan Soto, Shwetha Sridhar Hara, Ying-Chih Wang, Melissa Smith, Robert Sebra, Lisa Miorin, Wen-chun Liu, Randy Albrecht, Judith Aberg, Florian Krammer, Adolfo Garcia-Sarstre, Viviana Simon, Harm van Bakel
EPI_ISL_422509 hCoV-19/USA/NY-PV09138/2020 North America / USA / New York / Manhattan 2020-03-20 MSHS Clinical Microbiology Laboratories MSHS Pathogen Surveillance Program Ana S. Gonzalez-Reiche, Mitchell Sullivan, Ajay Obla, Gopi Patel, Emilia Sordillo, Melissa Gitman, Alberto Paniz-mondolfi, Matthew Hernandez, Shelcie Fabre, Jose Polanco, Zenab Khan, Bremy Albuquerque, Jayeeta Dutta, Juan Soto, Shwetha Sridhar Hara, Ying-Chih Wang, Melissa Smith, Robert Sebra, Lisa Miorin, Wen-chun Liu, Randy Albrecht, Judith Aberg, Florian Krammer, Adolfo Garcia-Sarstre, Viviana Simon, Harm van Bakel
EPI_ISL_422510 hCoV-19/USA/NY-PV09139/2020 North America / USA / New York / Queens 2020-03-20 MSHS Clinical Microbiology Laboratories MSHS Pathogen Surveillance Program Ana S. Gonzalez-Reiche, Mitchell Sullivan, Ajay Obla, Gopi Patel, Emilia Sordillo, Melissa Gitman, Alberto Paniz-mondolfi, Matthew Hernandez, Shelcie Fabre, Jose Polanco, Zenab Khan, Bremy Albuquerque, Jayeeta Dutta, Juan Soto, Shwetha

Sridhar Hara, Ying-Chih Wang, Melissa Smith, Robert Sebra, Lisa Miorin, Wen-chun Liu, Randy Albrecht, Judith Aberg, Florian Krammer, Adolfo Garcia-Sarstre, Viviana Simon, Harm van Bakel
EPI_ISL_422511 hCoV-19/USA/NY-PV09140/2020 North America / USA / New York / Manhattan 2020-03-20 MSHS Clinical Microbiology Laboratories MSHS Pathogen Surveillance Program Ana S. Gonzalez-Reiche, Mitchell Sullivan, Ajay Obla, Gopi Patel, Emilia Sordillo, Melissa Gitman, Alberto Paniz-mondolfi, Matthew Hernandez, Shelcie Fabre, Jose Polanco, Zenab Khan, Bremy Albuquerque, Jayeeta Dutta, Juan Soto, Shwetha Sridhar Hara, Ying-Chih Wang, Melissa Smith, Robert Sebra, Lisa Miorin, Wen-chun Liu, Randy Albrecht, Judith Aberg, Florian Krammer, Adolfo Garcia-Sarstre, Viviana Simon, Harm van Bakel
EPI_ISL_422513 hCoV-19/USA/NY-PV09142/2020 North America / USA / New York / Manhattan 2020-03-20 MSHS Clinical Microbiology Laboratories MSHS Pathogen Surveillance Program Ana S. Gonzalez-Reiche, Mitchell Sullivan, Ajay Obla, Gopi Patel, Emilia Sordillo, Melissa Gitman, Alberto Paniz-mondolfi, Matthew Hernandez, Shelcie Fabre, Jose Polanco, Zenab Khan, Bremy Albuquerque, Jayeeta Dutta, Juan Soto, Shwetha Sridhar Hara, Ying-Chih Wang, Melissa Smith, Robert Sebra, Lisa Miorin, Wen-chun Liu, Randy Albrecht, Judith Aberg, Florian Krammer, Adolfo Garcia-Sarstre, Viviana Simon, Harm van Bakel
EPI_ISL_422514 hCoV-19/USA/NY-PV09143/2020 North America / USA / New York / Manhattan 2020-03-20 MSHS Clinical Microbiology Laboratories MSHS Pathogen Surveillance Program Ana S. Gonzalez-Reiche, Mitchell Sullivan, Ajay Obla, Gopi Patel, Emilia Sordillo, Melissa Gitman, Alberto Paniz-mondolfi, Matthew Hernandez, Shelcie Fabre, Jose Polanco, Zenab Khan, Bremy Albuquerque, Jayeeta Dutta, Juan Soto, Shwetha Sridhar Hara, Ying-Chih Wang, Melissa Smith, Robert Sebra, Lisa Miorin, Wen-chun Liu, Randy Albrecht, Judith Aberg, Florian Krammer, Adolfo Garcia-Sarstre, Viviana Simon, Harm van Bakel
EPI_ISL_422515 hCoV-19/USA/NY-PV09144/2020 North America / USA / New York / Manhattan 2020-03-20 MSHS Clinical Microbiology Laboratories MSHS Pathogen Surveillance Program Ana S. Gonzalez-Reiche, Mitchell Sullivan, Ajay Obla, Gopi Patel, Emilia Sordillo, Melissa Gitman, Alberto Paniz-mondolfi, Matthew Hernandez, Shelcie Fabre, Jose Polanco, Zenab Khan, Bremy Albuquerque, Jayeeta Dutta, Juan Soto, Shwetha Sridhar Hara, Ying-Chih Wang, Melissa Smith, Robert Sebra, Lisa Miorin, Wen-chun Liu, Randy Albrecht, Judith Aberg, Florian Krammer, Adolfo Garcia-Sarstre, Viviana Simon, Harm van Bakel
EPI_ISL_422516 hCoV-19/USA/NY-PV09145/2020 North America / USA / New York / Queens 2020-03-20 MSHS Clinical Microbiology Laboratories MSHS Pathogen Surveillance Program Ana S. Gonzalez-Reiche, Mitchell Sullivan, Ajay Obla, Gopi Patel, Emilia Sordillo, Melissa Gitman, Alberto Paniz-mondolfi, Matthew Hernandez, Shelcie Fabre, Jose Polanco, Zenab Khan, Bremy Albuquerque, Jayeeta Dutta, Juan Soto, Shwetha Sridhar Hara, Ying-Chih Wang, Melissa Smith, Robert Sebra, Lisa Miorin, Wen-chun Liu, Randy Albrecht, Judith Aberg, Florian Krammer, Adolfo Garcia-Sarstre, Viviana Simon, Harm van Bakel
EPI_ISL_422517 hCoV-19/USA/NY-PV09147/2020 North America / USA / New York / Manhattan 2020-03-20 MSHS Clinical Microbiology Laboratories MSHS Pathogen Surveillance Program Ana S. Gonzalez-Reiche, Mitchell Sullivan, Ajay Obla, Gopi Patel, Emilia Sordillo, Melissa Gitman, Alberto Paniz-mondolfi, Matthew Hernandez, Shelcie Fabre, Jose Polanco, Zenab Khan, Bremy Albuquerque, Jayeeta Dutta, Juan Soto, Shwetha Sridhar Hara, Ying-Chih Wang, Melissa Smith, Robert Sebra, Lisa Miorin, Wen-chun Liu, Randy Albrecht, Judith Aberg, Florian Krammer, Adolfo Garcia-Sarstre, Viviana Simon, Harm van Bakel
EPI_ISL_422519 hCoV-19/USA/NY-PV09151/2020 North America / USA / New York / Brooklyn 2020-03-22 MSHS Clinical Microbiology Laboratories MSHS Pathogen Surveillance Program Ana S. Gonzalez-Reiche, Mitchell Sullivan, Ajay Obla, Gopi Patel, Emilia Sordillo, Melissa Gitman, Alberto Paniz-mondolfi, Matthew Hernandez, Shelcie Fabre, Jose Polanco, Zenab Khan, Bremy Albuquerque, Jayeeta Dutta, Juan Soto, Shwetha Sridhar Hara, Ying-Chih Wang, Melissa Smith, Robert Sebra, Lisa Miorin, Wen-chun Liu, Randy Albrecht, Judith Aberg, Florian Krammer, Adolfo Garcia-Sarstre, Viviana Simon, Harm van Bakel
EPI_ISL_422520 hCoV-19/USA/NY-PV09152/2020 North America / USA / New York / Brooklyn 2020-03-21 MSHS Clinical Microbiology Laboratories MSHS Pathogen Surveillance Program Ana S. Gonzalez-Reiche, Mitchell Sullivan, Ajay Obla, Gopi Patel, Emilia Sordillo, Melissa Gitman, Alberto Paniz-mondolfi, Matthew Hernandez, Shelcie Fabre, Jose Polanco, Zenab Khan, Bremy Albuquerque, Jayeeta Dutta, Juan Soto, Shwetha Sridhar Hara, Ying-Chih Wang, Melissa Smith, Robert Sebra, Lisa Miorin, Wen-chun Liu, Randy Albrecht, Judith Aberg, Florian Krammer, Adolfo Garcia-Sarstre, Viviana Simon, Harm van Bakel
EPI_ISL_422521 hCoV-19/USA/NY-PV09153/2020 North America / USA / New York / Brooklyn 2020-03-22 MSHS Clinical Microbiology Laboratories MSHS Pathogen Surveillance Program Ana S. Gonzalez-Reiche, Mitchell Sullivan, Ajay Obla, Gopi Patel, Emilia Sordillo, Melissa Gitman, Alberto Paniz-mondolfi, Matthew Hernandez, Shelcie Fabre, Jose Polanco, Zenab Khan, Bremy Albuquerque, Jayeeta Dutta, Juan Soto, Shwetha Sridhar Hara, Ying-Chih Wang, Melissa Smith, Robert Sebra, Lisa Miorin, Wen-chun Liu, Randy Albrecht, Judith Aberg, Florian Krammer, Adolfo Garcia-Sarstre, Viviana Simon, Harm van Bakel
EPI_ISL_422522 hCoV-19/USA/NY-PV09154/2020 North America / USA / New York / Manhattan 2020-03-22 MSHS Clinical Microbiology Laboratories MSHS Pathogen Surveillance Program Ana S. Gonzalez-Reiche, Mitchell Sullivan, Ajay Obla, Gopi Patel, Emilia Sordillo, Melissa Gitman, Alberto Paniz-mondolfi, Matthew Hernandez, Shelcie Fabre, Jose Polanco, Zenab Khan, Bremy Albuquerque, Jayeeta Dutta, Juan Soto, Shwetha Sridhar Hara, Ying-Chih Wang, Melissa Smith, Robert Sebra, Lisa Miorin, Wen-chun Liu, Randy Albrecht, Judith Aberg, Florian Krammer, Adolfo Garcia-Sarstre, Viviana Simon, Harm van Bakel
EPI_ISL_422523 hCoV-19/USA/NY-PV09156/2020 North America / USA / New York / Bronx 2020-03-21 MSHS Clinical Microbiology Laboratories MSHS Pathogen Surveillance Program Ana S. Gonzalez-Reiche, Mitchell Sullivan, Ajay Obla, Gopi Patel, Emilia Sordillo, Melissa Gitman, Alberto Paniz-mondolfi, Matthew Hernandez, Shelcie Fabre, Jose Polanco, Zenab Khan, Bremy Albuquerque, Jayeeta Dutta, Juan Soto, Shwetha Sridhar Hara, Ying-Chih Wang, Melissa Smith, Robert Sebra, Lisa Miorin, Wen-chun Liu, Randy Albrecht, Judith Aberg, Florian Krammer, Adolfo Garcia-Sarstre, Viviana Simon, Harm van Bakel
EPI_ISL_422524 hCoV-19/USA/NY-PV09157/2020 North America / USA / New York / Manhattan 2020-03-22 MSHS Clinical Microbiology Laboratories MSHS Pathogen Surveillance Program Ana S. Gonzalez-Reiche,

Mitchell Sullivan, Ajay Obla, Gopi Patel, Emilia Sordillo, Melissa Gitman, Alberto Paniz-mondolfi, Matthew Hernandez, Shelcie Fabre, Jose Polanco, Zenab Khan, Bremy Albuquerque, Jayeeta Dutta, Juan Soto, Shwetha Sridhar Hara, Ying-Chih Wang, Melissa Smith, Robert Sebra, Lisa Miorin, Wen-chun Liu, Randy Albrecht, Judith Aberg, Florian Krammer, Adolfo Garcia-Sarstre, Viviana Simon, Harm van Bakel
EPI_ISL_422525 hCoV-19/USA/NY-PV09158/2020 North America / USA / New York / Brooklyn 2020-03-22 MSHS Clinical Microbiology Laboratories MSHS Pathogen Surveillance Program Ana S. Gonzalez-Reiche, Mitchell Sullivan, Ajay Obla, Gopi Patel, Emilia Sordillo, Melissa Gitman, Alberto Paniz-mondolfi, Matthew Hernandez, Shelcie Fabre, Jose Polanco, Zenab Khan, Bremy Albuquerque, Jayeeta Dutta, Juan Soto, Shwetha Sridhar Hara, Ying-Chih Wang, Melissa Smith, Robert Sebra, Lisa Miorin, Wen-chun Liu, Randy Albrecht, Judith Aberg, Florian Krammer, Adolfo Garcia-Sarstre, Viviana Simon, Harm van Bakel
EPI_ISL_422527 hCoV-19/USA/NY-PV09163/2020 North America / USA / New York / Brooklyn 2020-03-21 MSHS Clinical Microbiology Laboratories MSHS Pathogen Surveillance Program Ana S. Gonzalez-Reiche, Mitchell Sullivan, Ajay Obla, Gopi Patel, Emilia Sordillo, Melissa Gitman, Alberto Paniz-mondolfi, Matthew Hernandez, Shelcie Fabre, Jose Polanco, Zenab Khan, Bremy Albuquerque, Jayeeta Dutta, Juan Soto, Shwetha Sridhar Hara, Ying-Chih Wang, Melissa Smith, Robert Sebra, Lisa Miorin, Wen-chun Liu, Randy Albrecht, Judith Aberg, Florian Krammer, Adolfo Garcia-Sarstre, Viviana Simon, Harm van Bakel
EPI_ISL_422528 hCoV-19/USA/NY-PV09164/2020 North America / USA / New York / Brooklyn 2020-03-21 MSHS Clinical Microbiology Laboratories MSHS Pathogen Surveillance Program Ana S. Gonzalez-Reiche, Mitchell Sullivan, Ajay Obla, Gopi Patel, Emilia Sordillo, Melissa Gitman, Alberto Paniz-mondolfi, Matthew Hernandez, Shelcie Fabre, Jose Polanco, Zenab Khan, Bremy Albuquerque, Jayeeta Dutta, Juan Soto, Shwetha Sridhar Hara, Ying-Chih Wang, Melissa Smith, Robert Sebra, Lisa Miorin, Wen-chun Liu, Randy Albrecht, Judith Aberg, Florian Krammer, Adolfo Garcia-Sarstre, Viviana Simon, Harm van Bakel
EPI_ISL_422529 hCoV-19/USA/NY-PV09165/2020 North America / USA / New York / Brooklyn 2020-03-22 MSHS Clinical Microbiology Laboratories MSHS Pathogen Surveillance Program Ana S. Gonzalez-Reiche, Mitchell Sullivan, Ajay Obla, Gopi Patel, Emilia Sordillo, Melissa Gitman, Alberto Paniz-mondolfi, Matthew Hernandez, Shelcie Fabre, Jose Polanco, Zenab Khan, Bremy Albuquerque, Jayeeta Dutta, Juan Soto, Shwetha Sridhar Hara, Ying-Chih Wang, Melissa Smith, Robert Sebra, Lisa Miorin, Wen-chun Liu, Randy Albrecht, Judith Aberg, Florian Krammer, Adolfo Garcia-Sarstre, Viviana Simon, Harm van Bakel
EPI_ISL_422530 hCoV-19/USA/NY-PV09168/2020 North America / USA / New York / Manhattan 2020-03-22 MSHS Clinical Microbiology Laboratories MSHS Pathogen Surveillance Program Ana S. Gonzalez-Reiche, Mitchell Sullivan, Ajay Obla, Gopi Patel, Emilia Sordillo, Melissa Gitman, Alberto Paniz-mondolfi, Matthew Hernandez, Shelcie Fabre, Jose Polanco, Zenab Khan, Bremy Albuquerque, Jayeeta Dutta, Juan Soto, Shwetha Sridhar Hara, Ying-Chih Wang, Melissa Smith, Robert Sebra, Lisa Miorin, Wen-chun Liu, Randy Albrecht, Judith Aberg, Florian Krammer, Adolfo Garcia-Sarstre, Viviana Simon, Harm van Bakel
EPI_ISL_422531 hCoV-19/USA/NY-PV09169/2020 North America / USA / New York / Manhattan 2020-03-19 MSHS Clinical Microbiology Laboratories MSHS Pathogen Surveillance Program Ana S. Gonzalez-Reiche, Mitchell Sullivan, Ajay Obla, Gopi Patel, Emilia Sordillo, Melissa Gitman, Alberto Paniz-mondolfi, Matthew Hernandez, Shelcie Fabre, Jose Polanco, Zenab Khan, Bremy Albuquerque, Jayeeta Dutta, Juan Soto, Shwetha Sridhar Hara, Ying-Chih Wang, Melissa Smith, Robert Sebra, Lisa Miorin, Wen-chun Liu, Randy Albrecht, Judith Aberg, Florian Krammer, Adolfo Garcia-Sarstre, Viviana Simon, Harm van Bakel
EPI_ISL_422532 hCoV-19/USA/NY-PV09170/2020 North America / USA / New York / Brooklyn 2020-03-19 MSHS Clinical Microbiology Laboratories MSHS Pathogen Surveillance Program Ana S. Gonzalez-Reiche, Mitchell Sullivan, Ajay Obla, Gopi Patel, Emilia Sordillo, Melissa Gitman, Alberto Paniz-mondolfi, Matthew Hernandez, Shelcie Fabre, Jose Polanco, Zenab Khan, Bremy Albuquerque, Jayeeta Dutta, Juan Soto, Shwetha Sridhar Hara, Ying-Chih Wang, Melissa Smith, Robert Sebra, Lisa Miorin, Wen-chun Liu, Randy Albrecht, Judith Aberg, Florian Krammer, Adolfo Garcia-Sarstre, Viviana Simon, Harm van Bakel
EPI_ISL_422533 hCoV-19/USA/NY-PV09171/2020 North America / USA / New York / Manhattan 2020-03-19 MSHS Clinical Microbiology Laboratories MSHS Pathogen Surveillance Program Ana S. Gonzalez-Reiche, Mitchell Sullivan, Ajay Obla, Gopi Patel, Emilia Sordillo, Melissa Gitman, Alberto Paniz-mondolfi, Matthew Hernandez, Shelcie Fabre, Jose Polanco, Zenab Khan, Bremy Albuquerque, Jayeeta Dutta, Juan Soto, Shwetha Sridhar Hara, Ying-Chih Wang, Melissa Smith, Robert Sebra, Lisa Miorin, Wen-chun Liu, Randy Albrecht, Judith Aberg, Florian Krammer, Adolfo Garcia-Sarstre, Viviana Simon, Harm van Bakel
EPI_ISL_422534 hCoV-19/USA/NY-PV09172/2020 North America / USA / New York / Queens 2020-03-19 MSHS Clinical Microbiology Laboratories MSHS Pathogen Surveillance Program Ana S. Gonzalez-Reiche, Mitchell Sullivan, Ajay Obla, Gopi Patel, Emilia Sordillo, Melissa Gitman, Alberto Paniz-mondolfi, Matthew Hernandez, Shelcie Fabre, Jose Polanco, Zenab Khan, Bremy Albuquerque, Jayeeta Dutta, Juan Soto, Shwetha Sridhar Hara, Ying-Chih Wang, Melissa Smith, Robert Sebra, Lisa Miorin, Wen-chun Liu, Randy Albrecht, Judith Aberg, Florian Krammer, Adolfo Garcia-Sarstre, Viviana Simon, Harm van Bakel
EPI_ISL_422535 hCoV-19/USA/NY-PV09176/2020 North America / USA / New York / Brooklyn 2020-03-19 MSHS Clinical Microbiology Laboratories MSHS Pathogen Surveillance Program Ana S. Gonzalez-Reiche, Mitchell Sullivan, Ajay Obla, Gopi Patel, Emilia Sordillo, Melissa Gitman, Alberto Paniz-mondolfi, Matthew Hernandez, Shelcie Fabre, Jose Polanco, Zenab Khan, Bremy Albuquerque, Jayeeta Dutta, Juan Soto, Shwetha Sridhar Hara, Ying-Chih Wang, Melissa Smith, Robert Sebra, Lisa Miorin, Wen-chun Liu, Randy Albrecht, Judith Aberg, Florian Krammer, Adolfo Garcia-Sarstre, Viviana Simon, Harm van Bakel
EPI_ISL_422536 hCoV-19/USA/NY-PV09177/2020 North America / USA / New York / Brooklyn 2020-03-19 MSHS Clinical Microbiology Laboratories MSHS Pathogen Surveillance Program Ana S. Gonzalez-Reiche, Mitchell Sullivan, Ajay Obla, Gopi Patel, Emilia Sordillo, Melissa Gitman, Alberto Paniz-mondolfi, Matthew Hernandez, Shelcie Fabre, Jose Polanco, Zenab Khan, Bremy Albuquerque, Jayeeta Dutta, Juan Soto, Shwetha Sridhar Hara, Ying-Chih Wang, Melissa Smith, Robert Sebra, Lisa Miorin, Wen-chun Liu, Randy Albrecht, Judith Aberg, Florian Krammer, Adolfo Garcia-Sarstre, Viviana Simon, Harm van Bakel

EPI_ISL_422537 hCoV-19/USA/NY-PV09178/2020 North America / USA / New York / Manhattan 2020-03-19 MSHS Clinical Microbiology Laboratories MSHS Pathogen Surveillance Program Ana S. Gonzalez-Reiche, Mitchell Sullivan, Ajay Obla, Gopi Patel, Emilia Sordillo, Melissa Gitman, Alberto Paniz-mondolfi, Matthew Hernandez, Shelcie Fabre, Jose Polanco, Zenab Khan, Bremy Albuquerque, Jayeeta Dutta, Juan Soto, Shwetha Sridhar Hara, Ying-Chih Wang, Melissa Smith, Robert Sebra, Lisa Miorin, Wen-chun Liu, Randy Albrecht, Judith Aberg, Florian Krammer, Adolfo Garcia-Sarstre, Viviana Simon, Harm van Bakel
EPI_ISL_422539 hCoV-19/USA/NY-PV09180/2020 North America / USA / New York / Bronx 2020-03-19 MSHS Clinical Microbiology Laboratories MSHS Pathogen Surveillance Program Ana S. Gonzalez-Reiche, Mitchell Sullivan, Ajay Obla, Gopi Patel, Emilia Sordillo, Melissa Gitman, Alberto Paniz-mondolfi, Matthew Hernandez, Shelcie Fabre, Jose Polanco, Zenab Khan, Bremy Albuquerque, Jayeeta Dutta, Juan Soto, Shwetha Sridhar Hara, Ying-Chih Wang, Melissa Smith, Robert Sebra, Lisa Miorin, Wen-chun Liu, Randy Albrecht, Judith Aberg, Florian Krammer, Adolfo Garcia-Sarstre, Viviana Simon, Harm van Bakel
EPI_ISL_422540 hCoV-19/USA/NY-PV09181/2020 North America / USA / New York / Manhattan 2020-03-19 MSHS Clinical Microbiology Laboratories MSHS Pathogen Surveillance Program Ana S. Gonzalez-Reiche, Mitchell Sullivan, Ajay Obla, Gopi Patel, Emilia Sordillo, Melissa Gitman, Alberto Paniz-mondolfi, Matthew Hernandez, Shelcie Fabre, Jose Polanco, Zenab Khan, Bremy Albuquerque, Jayeeta Dutta, Juan Soto, Shwetha Sridhar Hara, Ying-Chih Wang, Melissa Smith, Robert Sebra, Lisa Miorin, Wen-chun Liu, Randy Albrecht, Judith Aberg, Florian Krammer, Adolfo Garcia-Sarstre, Viviana Simon, Harm van Bakel
EPI_ISL_422541 hCoV-19/USA/NY-PV09182/2020 North America / USA / New York / Manhattan 2020-03-19 MSHS Clinical Microbiology Laboratories MSHS Pathogen Surveillance Program Ana S. Gonzalez-Reiche, Mitchell Sullivan, Ajay Obla, Gopi Patel, Emilia Sordillo, Melissa Gitman, Alberto Paniz-mondolfi, Matthew Hernandez, Shelcie Fabre, Jose Polanco, Zenab Khan, Bremy Albuquerque, Jayeeta Dutta, Juan Soto, Shwetha Sridhar Hara, Ying-Chih Wang, Melissa Smith, Robert Sebra, Lisa Miorin, Wen-chun Liu, Randy Albrecht, Judith Aberg, Florian Krammer, Adolfo Garcia-Sarstre, Viviana Simon, Harm van Bakel
EPI_ISL_422542 hCoV-19/USA/NY-PV09183/2020 North America / USA / New York / Bronx 2020-03-19 MSHS Clinical Microbiology Laboratories MSHS Pathogen Surveillance Program Ana S. Gonzalez-Reiche, Mitchell Sullivan, Ajay Obla, Gopi Patel, Emilia Sordillo, Melissa Gitman, Alberto Paniz-mondolfi, Matthew Hernandez, Shelcie Fabre, Jose Polanco, Zenab Khan, Bremy Albuquerque, Jayeeta Dutta, Juan Soto, Shwetha Sridhar Hara, Ying-Chih Wang, Melissa Smith, Robert Sebra, Lisa Miorin, Wen-chun Liu, Randy Albrecht, Judith Aberg, Florian Krammer, Adolfo Garcia-Sarstre, Viviana Simon, Harm van Bakel
EPI_ISL_422543 hCoV-19/USA/NY-PV09186/2020 North America / USA / New York / Bronx 2020-03-20 MSHS Clinical Microbiology Laboratories MSHS Pathogen Surveillance Program Ana S. Gonzalez-Reiche, Mitchell Sullivan, Ajay Obla, Gopi Patel, Emilia Sordillo, Melissa Gitman, Alberto Paniz-mondolfi, Matthew Hernandez, Shelcie Fabre, Jose Polanco, Zenab Khan, Bremy Albuquerque, Jayeeta Dutta, Juan Soto, Shwetha Sridhar Hara, Ying-Chih Wang, Melissa Smith, Robert Sebra, Lisa Miorin, Wen-chun Liu, Randy Albrecht, Judith Aberg, Florian Krammer, Adolfo Garcia-Sarstre, Viviana Simon, Harm van Bakel
EPI_ISL_422544 hCoV-19/USA/NY-PV09187/2020 North America / USA / New York / Westchester County 2020-03-20 MSHS Clinical Microbiology Laboratories MSHS Pathogen Surveillance Program Ana S. Gonzalez-Reiche, Mitchell Sullivan, Ajay Obla, Gopi Patel, Emilia Sordillo, Melissa Gitman, Alberto Paniz-mondolfi, Matthew Hernandez, Shelcie Fabre, Jose Polanco, Zenab Khan, Bremy Albuquerque, Jayeeta Dutta, Juan Soto, Shwetha Sridhar Hara, Ying-Chih Wang, Melissa Smith, Robert Sebra, Lisa Miorin, Wen-chun Liu, Randy Albrecht, Judith Aberg, Florian Krammer, Adolfo Garcia-Sarstre, Viviana Simon, Harm van Bakel
EPI_ISL_422546 hCoV-19/USA/NY-PV09189/2020 North America / USA / New York / Queens 2020-03-19 MSHS Clinical Microbiology Laboratories MSHS Pathogen Surveillance Program Ana S. Gonzalez-Reiche, Mitchell Sullivan, Ajay Obla, Gopi Patel, Emilia Sordillo, Melissa Gitman, Alberto Paniz-mondolfi, Matthew Hernandez, Shelcie Fabre, Jose Polanco, Zenab Khan, Bremy Albuquerque, Jayeeta Dutta, Juan Soto, Shwetha Sridhar Hara, Ying-Chih Wang, Melissa Smith, Robert Sebra, Lisa Miorin, Wen-chun Liu, Randy Albrecht, Judith Aberg, Florian Krammer, Adolfo Garcia-Sarstre, Viviana Simon, Harm van Bakel
EPI_ISL_422547 hCoV-19/USA/NY-PV09190/2020 North America / USA / New York / Manhattan 2020-03-20 MSHS Clinical Microbiology Laboratories MSHS Pathogen Surveillance Program Ana S. Gonzalez-Reiche, Mitchell Sullivan, Ajay Obla, Gopi Patel, Emilia Sordillo, Melissa Gitman, Alberto Paniz-mondolfi, Matthew Hernandez, Shelcie Fabre, Jose Polanco, Zenab Khan, Bremy Albuquerque, Jayeeta Dutta, Juan Soto, Shwetha Sridhar Hara, Ying-Chih Wang, Melissa Smith, Robert Sebra, Lisa Miorin, Wen-chun Liu, Randy Albrecht, Judith Aberg, Florian Krammer, Adolfo Garcia-Sarstre, Viviana Simon, Harm van Bakel
EPI_ISL_422548 hCoV-19/USA/NY-PV09192/2020 North America / USA / New York / Bronx 2020-03-20 MSHS Clinical Microbiology Laboratories MSHS Pathogen Surveillance Program Ana S. Gonzalez-Reiche, Mitchell Sullivan, Ajay Obla, Gopi Patel, Emilia Sordillo, Melissa Gitman, Alberto Paniz-mondolfi, Matthew Hernandez, Shelcie Fabre, Jose Polanco, Zenab Khan, Bremy Albuquerque, Jayeeta Dutta, Juan Soto, Shwetha Sridhar Hara, Ying-Chih Wang, Melissa Smith, Robert Sebra, Lisa Miorin, Wen-chun Liu, Randy Albrecht, Judith Aberg, Florian Krammer, Adolfo Garcia-Sarstre, Viviana Simon, Harm van Bakel
EPI_ISL_422549 hCoV-19/USA/NY-PV09193/2020 North America / USA / New York / Manhattan 2020-03-20 MSHS Clinical Microbiology Laboratories MSHS Pathogen Surveillance Program Ana S. Gonzalez-Reiche, Mitchell Sullivan, Ajay Obla, Gopi Patel, Emilia Sordillo, Melissa Gitman, Alberto Paniz-mondolfi, Matthew Hernandez, Shelcie Fabre, Jose Polanco, Zenab Khan, Bremy Albuquerque, Jayeeta Dutta, Juan Soto, Shwetha Sridhar Hara, Ying-Chih Wang, Melissa Smith, Robert Sebra, Lisa Miorin, Wen-chun Liu, Randy Albrecht, Judith Aberg, Florian Krammer, Adolfo Garcia-Sarstre, Viviana Simon, Harm van Bakel
EPI_ISL_422550 hCoV-19/USA/NY-PV09194/2020 North America / USA / New York / Bronx 2020-03-20 MSHS Clinical Microbiology Laboratories MSHS Pathogen Surveillance Program Ana S. Gonzalez-Reiche, Mitchell Sullivan, Ajay Obla, Gopi Patel, Emilia Sordillo, Melissa Gitman, Alberto Paniz-mondolfi, Matthew Hernandez, Shelcie Fabre, Jose Polanco, Zenab Khan, Bremy Albuquerque, Jayeeta Dutta, Juan Soto, Shwetha

Sridhar Hara, Ying-Chih Wang, Melissa Smith, Robert Sebra, Lisa Miorin, Wen-chun Liu, Randy Albrecht, Judith Aberg, Florian Krammer, Adolfo Garcia-Sarstre, Viviana Simon, Harm van Bakel
EPI_ISL_422551 hCoV-19/USA/NY-PV09195/2020 North America / USA / New York / Bronx 2020-03-20 MSHS Clinical Microbiology Laboratories MSHS Pathogen Surveillance Program Ana S. Gonzalez-Reiche, Mitchell Sullivan, Ajay Obla, Gopi Patel, Emilia Sordillo, Melissa Gitman, Alberto Paniz-mondolfi, Matthew Hernandez, Shelcie Fabre, Jose Polanco, Zenab Khan, Bremy Albuquerque, Jayeeta Dutta, Juan Soto, Shwetha Sridhar Hara, Ying-Chih Wang, Melissa Smith, Robert Sebra, Lisa Miorin, Wen-chun Liu, Randy Albrecht, Judith Aberg, Florian Krammer, Adolfo Garcia-Sarstre, Viviana Simon, Harm van Bakel
EPI_ISL_422552 hCoV-19/USA/NY-PV09197/2020 North America / USA / New York / Manhattan 2020-03-20 MSHS Clinical Microbiology Laboratories MSHS Pathogen Surveillance Program Ana S. Gonzalez-Reiche, Mitchell Sullivan, Ajay Obla, Gopi Patel, Emilia Sordillo, Melissa Gitman, Alberto Paniz-mondolfi, Matthew Hernandez, Shelcie Fabre, Jose Polanco, Zenab Khan, Bremy Albuquerque, Jayeeta Dutta, Juan Soto, Shwetha Sridhar Hara, Ying-Chih Wang, Melissa Smith, Robert Sebra, Lisa Miorin, Wen-chun Liu, Randy Albrecht, Judith Aberg, Florian Krammer, Adolfo Garcia-Sarstre, Viviana Simon, Harm van Bakel
EPI_ISL_422553 hCoV-19/USA/NY-PV09198/2020 North America / USA / New York / Manhattan 2020-03-20 MSHS Clinical Microbiology Laboratories MSHS Pathogen Surveillance Program Ana S. Gonzalez-Reiche, Mitchell Sullivan, Ajay Obla, Gopi Patel, Emilia Sordillo, Melissa Gitman, Alberto Paniz-mondolfi, Matthew Hernandez, Shelcie Fabre, Jose Polanco, Zenab Khan, Bremy Albuquerque, Jayeeta Dutta, Juan Soto, Shwetha Sridhar Hara, Ying-Chih Wang, Melissa Smith, Robert Sebra, Lisa Miorin, Wen-chun Liu, Randy Albrecht, Judith Aberg, Florian Krammer, Adolfo Garcia-Sarstre, Viviana Simon, Harm van Bakel
EPI_ISL_422554 hCoV-19/USA/NY-PV09199/2020 North America / USA / New York / Brooklyn 2020-03-21 MSHS Clinical Microbiology Laboratories MSHS Pathogen Surveillance Program Ana S. Gonzalez-Reiche, Mitchell Sullivan, Ajay Obla, Gopi Patel, Emilia Sordillo, Melissa Gitman, Alberto Paniz-mondolfi, Matthew Hernandez, Shelcie Fabre, Jose Polanco, Zenab Khan, Bremy Albuquerque, Jayeeta Dutta, Juan Soto, Shwetha Sridhar Hara, Ying-Chih Wang, Melissa Smith, Robert Sebra, Lisa Miorin, Wen-chun Liu, Randy Albrecht, Judith Aberg, Florian Krammer, Adolfo Garcia-Sarstre, Viviana Simon, Harm van Bakel
EPI_ISL_422555 hCoV-19/USA/NY-PV09200/2020 North America / USA / New York / Manhattan 2020-03-22 MSHS Clinical Microbiology Laboratories MSHS Pathogen Surveillance Program Ana S. Gonzalez-Reiche, Mitchell Sullivan, Ajay Obla, Gopi Patel, Emilia Sordillo, Melissa Gitman, Alberto Paniz-mondolfi, Matthew Hernandez, Shelcie Fabre, Jose Polanco, Zenab Khan, Bremy Albuquerque, Jayeeta Dutta, Juan Soto, Shwetha Sridhar Hara, Ying-Chih Wang, Melissa Smith, Robert Sebra, Lisa Miorin, Wen-chun Liu, Randy Albrecht, Judith Aberg, Florian Krammer, Adolfo Garcia-Sarstre, Viviana Simon, Harm van Bakel
EPI_ISL_422600 hCoV-19/Netherlands/NA_291/2020 Europe / Netherlands 2020-04-01 Dutch COVID-19 response team Erasmus Medical Center Bas Oude Munnink, David Nieuwenhuijse, Reina Sikkema, Claudia Schapendonk, Irina Chestakova, Anne van der Linden, Theo Bestebroer, Stefan van Nieuwkoop, Mark Pronk, Pascal Lexmond, Corien Swaan, Manon Haverkate, Madelief Mollers, Mart Stein, Sandra Kengne Kamga Mobou, Jeroen van Kampen, Jolanda Voermans, Aura Timen, Corine GeurtsvanKessel, Annemiek van der Eijk, Richard Molenkamp, Marion Koopmans, on behalf of the Dutch national COVID-19 response team.
EPI_ISL_422601 hCoV-19/Netherlands/NA_292/2020 Europe / Netherlands 2020-04-01 Dutch COVID-19 response team Erasmus Medical Center Bas Oude Munnink, David Nieuwenhuijse, Reina Sikkema, Claudia Schapendonk, Irina Chestakova, Anne van der Linden, Theo Bestebroer, Stefan van Nieuwkoop, Mark Pronk, Pascal Lexmond, Corien Swaan, Manon Haverkate, Madelief Mollers, Mart Stein, Sandra Kengne Kamga Mobou, Jeroen van Kampen, Jolanda Voermans, Aura Timen, Corine GeurtsvanKessel, Annemiek van der Eijk, Richard Molenkamp, Marion Koopmans, on behalf of the Dutch national COVID-19 response team.
EPI_ISL_422602 hCoV-19/Netherlands/NA_293/2020 Europe / Netherlands 2020-04-01 Dutch COVID-19 response team Erasmus Medical Center Bas Oude Munnink, David Nieuwenhuijse, Reina Sikkema, Claudia Schapendonk, Irina Chestakova, Anne van der Linden, Theo Bestebroer, Stefan van Nieuwkoop, Mark Pronk, Pascal Lexmond, Corien Swaan, Manon Haverkate, Madelief Mollers, Mart Stein, Sandra Kengne Kamga Mobou, Jeroen van Kampen, Jolanda Voermans, Aura Timen, Corine GeurtsvanKessel, Annemiek van der Eijk, Richard Molenkamp, Marion Koopmans, on behalf of the Dutch national COVID-19 response team.
EPI_ISL_422603 hCoV-19/Netherlands/NA_294/2020 Europe / Netherlands 2020-04-01 Dutch COVID-19 response team Erasmus Medical Center Bas Oude Munnink, David Nieuwenhuijse, Reina Sikkema, Claudia Schapendonk, Irina Chestakova, Anne van der Linden, Theo Bestebroer, Stefan van Nieuwkoop, Mark Pronk, Pascal Lexmond, Corien Swaan, Manon Haverkate, Madelief Mollers, Mart Stein, Sandra Kengne Kamga Mobou, Jeroen van Kampen, Jolanda Voermans, Aura Timen, Corine GeurtsvanKessel, Annemiek van der Eijk, Richard Molenkamp, Marion Koopmans, on behalf of the Dutch national COVID-19 response team.
EPI_ISL_422604 hCoV-19/Netherlands/NA_295/2020 Europe / Netherlands 2020-04-01 Dutch COVID-19 response team Erasmus Medical Center Bas Oude Munnink, David Nieuwenhuijse, Reina Sikkema, Claudia Schapendonk, Irina Chestakova, Anne van der Linden, Theo Bestebroer, Stefan van Nieuwkoop, Mark Pronk, Pascal Lexmond, Corien Swaan, Manon Haverkate, Madelief Mollers, Mart Stein, Sandra Kengne Kamga Mobou, Jeroen van Kampen, Jolanda Voermans, Aura Timen, Corine GeurtsvanKessel, Annemiek van der Eijk, Richard Molenkamp, Marion Koopmans, on behalf of the Dutch national COVID-19 response team.
EPI_ISL_422605 hCoV-19/Netherlands/NA_296/2020 Europe / Netherlands 2020-04-01 Dutch COVID-19 response team Erasmus Medical Center Bas Oude Munnink, David Nieuwenhuijse, Reina Sikkema, Claudia Schapendonk, Irina Chestakova, Anne van der Linden, Theo Bestebroer, Stefan van Nieuwkoop, Mark Pronk, Pascal Lexmond, Corien Swaan, Manon Haverkate, Madelief Mollers, Mart Stein, Sandra Kengne Kamga Mobou, Jeroen van Kampen, Jolanda Voermans, Aura Timen, Corine GeurtsvanKessel, Annemiek van der Eijk, Richard Molenkamp, Marion Koopmans, on behalf of the Dutch national COVID-19 response team.
EPI_ISL_422606 hCoV-19/Netherlands/NA_297/2020 Europe / Netherlands 2020-04-01 Dutch COVID-19 response team Erasmus Medical Center Bas Oude Munnink, David Nieuwenhuijse, Reina Sikkema, Claudia Schapendonk,

Irina Chestakova, Anne van der Linden, Theo Bestebroer, Stefan van Nieuwkoop, Mark Pronk, Pascal Lexmond, Corien Swaan, Manon Haverkate, Madelief Mollers, Mart Stein, Sandra Kengne Kamga Mobou, Jeroen van Kampen, Jolanda Voermans, Aura Timen, Corine GeurtsvanKessel, Annemiek van der Eijk, Richard Molenkamp, Marion Koopmans, on behalf of the Dutch national COVID-19 response team.
EPI_ISL_422607 hCoV-19/Netherlands/NA_298/2020 Europe / Netherlands 2020-04-01 Dutch COVID-19 response team Erasmus Medical Center Bas Oude Munnink, David Nieuwenhuijse, Reina Sikkema, Claudia Schapendonk, Irina Chestakova, Anne van der Linden, Theo Bestebroer, Stefan van Nieuwkoop, Mark Pronk, Pascal Lexmond, Corien Swaan, Manon Haverkate, Madelief Mollers, Mart Stein, Sandra Kengne Kamga Mobou, Jeroen van Kampen, Jolanda Voermans, Aura Timen, Corine GeurtsvanKessel, Annemiek van der Eijk, Richard Molenkamp, Marion Koopmans, on behalf of the Dutch national COVID-19 response team.
EPI_ISL_422608 hCoV-19/Netherlands/NA_299/2020 Europe / Netherlands 2020-04-01 Dutch COVID-19 response team Erasmus Medical Center Bas Oude Munnink, David Nieuwenhuijse, Reina Sikkema, Claudia Schapendonk, Irina Chestakova, Anne van der Linden, Theo Bestebroer, Stefan van Nieuwkoop, Mark Pronk, Pascal Lexmond, Corien Swaan, Manon Haverkate, Madelief Mollers, Mart Stein, Sandra Kengne Kamga Mobou, Jeroen van Kampen, Jolanda Voermans, Aura Timen, Corine GeurtsvanKessel, Annemiek van der Eijk, Richard Molenkamp, Marion Koopmans, on behalf of the Dutch national COVID-19 response team.
EPI_ISL_422609 hCoV-19/Netherlands/NA_300/2020 Europe / Netherlands 2020-04-01 Dutch COVID-19 response team Erasmus Medical Center Bas Oude Munnink, David Nieuwenhuijse, Reina Sikkema, Claudia Schapendonk, Irina Chestakova, Anne van der Linden, Theo Bestebroer, Stefan van Nieuwkoop, Mark Pronk, Pascal Lexmond, Corien Swaan, Manon Haverkate, Madelief Mollers, Mart Stein, Sandra Kengne Kamga Mobou, Jeroen van Kampen, Jolanda Voermans, Aura Timen, Corine GeurtsvanKessel, Annemiek van der Eijk, Richard Molenkamp, Marion Koopmans, on behalf of the Dutch national COVID-19 response team.
EPI_ISL_422610 hCoV-19/Netherlands/NA_301/2020 Europe / Netherlands 2020-04-01 Dutch COVID-19 response team Erasmus Medical Center Bas Oude Munnink, David Nieuwenhuijse, Reina Sikkema, Claudia Schapendonk, Irina Chestakova, Anne van der Linden, Theo Bestebroer, Stefan van Nieuwkoop, Mark Pronk, Pascal Lexmond, Corien Swaan, Manon Haverkate, Madelief Mollers, Mart Stein, Sandra Kengne Kamga Mobou, Jeroen van Kampen, Jolanda Voermans, Aura Timen, Corine GeurtsvanKessel, Annemiek van der Eijk, Richard Molenkamp, Marion Koopmans, on behalf of the Dutch national COVID-19 response team.
EPI_ISL_422611 hCoV-19/Netherlands/NA_302/2020 Europe / Netherlands 2020-04-01 Dutch COVID-19 response team Erasmus Medical Center Bas Oude Munnink, David Nieuwenhuijse, Reina Sikkema, Claudia Schapendonk, Irina Chestakova, Anne van der Linden, Theo Bestebroer, Stefan van Nieuwkoop, Mark Pronk, Pascal Lexmond, Corien Swaan, Manon Haverkate, Madelief Mollers, Mart Stein, Sandra Kengne Kamga Mobou, Jeroen van Kampen, Jolanda Voermans, Aura Timen, Corine GeurtsvanKessel, Annemiek van der Eijk, Richard Molenkamp, Marion Koopmans, on behalf of the Dutch national COVID-19 response team.
EPI_ISL_422612 hCoV-19/Netherlands/NA_303/2020 Europe / Netherlands 2020-04-01 Dutch COVID-19 response team Erasmus Medical Center Bas Oude Munnink, David Nieuwenhuijse, Reina Sikkema, Claudia Schapendonk, Irina Chestakova, Anne van der Linden, Theo Bestebroer, Stefan van Nieuwkoop, Mark Pronk, Pascal Lexmond, Corien Swaan, Manon Haverkate, Madelief Mollers, Mart Stein, Sandra Kengne Kamga Mobou, Jeroen van Kampen, Jolanda Voermans, Aura Timen, Corine GeurtsvanKessel, Annemiek van der Eijk, Richard Molenkamp, Marion Koopmans, on behalf of the Dutch national COVID-19 response team.
EPI_ISL_422615 hCoV-19/Netherlands/NA_306/2020 Europe / Netherlands 2020-04-01 Dutch COVID-19 response team Erasmus Medical Center Bas Oude Munnink, David Nieuwenhuijse, Reina Sikkema, Claudia Schapendonk, Irina Chestakova, Anne van der Linden, Theo Bestebroer, Stefan van Nieuwkoop, Mark Pronk, Pascal Lexmond, Corien Swaan, Manon Haverkate, Madelief Mollers, Mart Stein, Sandra Kengne Kamga Mobou, Jeroen van Kampen, Jolanda Voermans, Aura Timen, Corine GeurtsvanKessel, Annemiek van der Eijk, Richard Molenkamp, Marion Koopmans, on behalf of the Dutch national COVID-19 response team.
EPI_ISL_422616 hCoV-19/Netherlands/NA_307/2020 Europe / Netherlands 2020-04-02 Dutch COVID-19 response team Erasmus Medical Center Bas Oude Munnink, David Nieuwenhuijse, Reina Sikkema, Claudia Schapendonk, Irina Chestakova, Anne van der Linden, Theo Bestebroer, Stefan van Nieuwkoop, Mark Pronk, Pascal Lexmond, Corien Swaan, Manon Haverkate, Madelief Mollers, Mart Stein, Sandra Kengne Kamga Mobou, Jeroen van Kampen, Jolanda Voermans, Aura Timen, Corine GeurtsvanKessel, Annemiek van der Eijk, Richard Molenkamp, Marion Koopmans, on behalf of the Dutch national COVID-19 response team.
EPI_ISL_422619 hCoV-19/Netherlands/NA_310/2020 Europe / Netherlands 2020-04-02 Dutch COVID-19 response team Erasmus Medical Center Bas Oude Munnink, David Nieuwenhuijse, Reina Sikkema, Claudia Schapendonk, Irina Chestakova, Anne van der Linden, Theo Bestebroer, Stefan van Nieuwkoop, Mark Pronk, Pascal Lexmond, Corien Swaan, Manon Haverkate, Madelief Mollers, Mart Stein, Sandra Kengne Kamga Mobou, Jeroen van Kampen, Jolanda Voermans, Aura Timen, Corine GeurtsvanKessel, Annemiek van der Eijk, Richard Molenkamp, Marion Koopmans, on behalf of the Dutch national COVID-19 response team.
EPI_ISL_422620 hCoV-19/Netherlands/NA_311/2020 Europe / Netherlands 2020-04-02 Dutch COVID-19 response team Erasmus Medical Center Bas Oude Munnink, David Nieuwenhuijse, Reina Sikkema, Claudia Schapendonk, Irina Chestakova, Anne van der Linden, Theo Bestebroer, Stefan van Nieuwkoop, Mark Pronk, Pascal Lexmond, Corien Swaan, Manon Haverkate, Madelief Mollers, Mart Stein, Sandra Kengne Kamga Mobou, Jeroen van Kampen, Jolanda Voermans, Aura Timen, Corine GeurtsvanKessel, Annemiek van der Eijk, Richard Molenkamp, Marion Koopmans, on behalf of the Dutch national COVID-19 response team.
EPI_ISL_422621 hCoV-19/Netherlands/NA_312/2020 Europe / Netherlands 2020-04-02 Dutch COVID-19 response team Erasmus Medical Center Bas Oude Munnink, David Nieuwenhuijse, Reina Sikkema, Claudia Schapendonk, Irina Chestakova, Anne van der Linden, Theo Bestebroer, Stefan van Nieuwkoop, Mark Pronk, Pascal Lexmond, Corien Swaan, Manon Haverkate, Madelief Mollers, Mart Stein, Sandra Kengne Kamga Mobou, Jeroen van Kampen, Jolanda Voermans, Aura Timen, Corine GeurtsvanKessel, Annemiek van der Eijk, Richard Molenkamp, Marion Koopmans, on behalf of the Dutch national COVID-19 response team.

EPI_ISL_422622 hCoV-19/Netherlands/NA_313/2020 Europe / Netherlands 2020-04-02 Dutch COVID-19 response team Erasmus Medical Center Bas Oude Munnink, David Nieuwenhuijse, Reina Sikkema, Claudia Schapendonk, Irina Chestakova, Anne van der Linden, Theo Bestebroer, Stefan van Nieuwkoop, Mark Pronk, Pascal Lexmond, Corien Swaan, Manon Haverkate, Madelief Mollers, Mart Stein, Sandra Kengne Kamga Mobou, Jeroen van Kampen, Jolanda Voermans, Aura Timen, Corine GeurtsvanKessel, Annemiek van der Eijk, Richard Molenkamp, Marion Koopmans, on behalf of the Dutch national COVID-19 response team.
EPI_ISL_422623 hCoV-19/Netherlands/NA_314/2020 Europe / Netherlands 2020-04-02 Dutch COVID-19 response team Erasmus Medical Center Bas Oude Munnink, David Nieuwenhuijse, Reina Sikkema, Claudia Schapendonk, Irina Chestakova, Anne van der Linden, Theo Bestebroer, Stefan van Nieuwkoop, Mark Pronk, Pascal Lexmond, Corien Swaan, Manon Haverkate, Madelief Mollers, Mart Stein, Sandra Kengne Kamga Mobou, Jeroen van Kampen, Jolanda Voermans, Aura Timen, Corine GeurtsvanKessel, Annemiek van der Eijk, Richard Molenkamp, Marion Koopmans, on behalf of the Dutch national COVID-19 response team.
EPI_ISL_422625 hCoV-19/Netherlands/NA_316/2020 Europe / Netherlands 2020-04-02 Dutch COVID-19 response team Erasmus Medical Center Bas Oude Munnink, David Nieuwenhuijse, Reina Sikkema, Claudia Schapendonk, Irina Chestakova, Anne van der Linden, Theo Bestebroer, Stefan van Nieuwkoop, Mark Pronk, Pascal Lexmond, Corien Swaan, Manon Haverkate, Madelief Mollers, Mart Stein, Sandra Kengne Kamga Mobou, Jeroen van Kampen, Jolanda Voermans, Aura Timen, Corine GeurtsvanKessel, Annemiek van der Eijk, Richard Molenkamp, Marion Koopmans, on behalf of the Dutch national COVID-19 response team.
EPI_ISL_422626 hCoV-19/Netherlands/NA_317/2020 Europe / Netherlands 2020-04-02 Dutch COVID-19 response team Erasmus Medical Center Bas Oude Munnink, David Nieuwenhuijse, Reina Sikkema, Claudia Schapendonk, Irina Chestakova, Anne van der Linden, Theo Bestebroer, Stefan van Nieuwkoop, Mark Pronk, Pascal Lexmond, Corien Swaan, Manon Haverkate, Madelief Mollers, Mart Stein, Sandra Kengne Kamga Mobou, Jeroen van Kampen, Jolanda Voermans, Aura Timen, Corine GeurtsvanKessel, Annemiek van der Eijk, Richard Molenkamp, Marion Koopmans, on behalf of the Dutch national COVID-19 response team.
EPI_ISL_422628 hCoV-19/Netherlands/ZuidHolland_100/2020 Europe / Netherlands / Zuid Holland 2020-03-31 Dutch COVID-19 response team Erasmus Medical Center Bas Oude Munnink, David Nieuwenhuijse, Reina Sikkema, Claudia Schapendonk, Irina Chestakova, Anne van der Linden, Theo Bestebroer, Stefan van Nieuwkoop, Mark Pronk, Pascal Lexmond, Corien Swaan, Manon Haverkate, Madelief Mollers, Mart Stein, Sandra Kengne Kamga Mobou, Jeroen van Kampen, Jolanda Voermans, Aura Timen, Corine GeurtsvanKessel, Annemiek van der Eijk, Richard Molenkamp, Marion Koopmans, on behalf of the Dutch national COVID-19 response team.
EPI_ISL_422629 hCoV-19/Netherlands/ZuidHolland_101/2020 Europe / Netherlands / Zuid Holland 2020-03-31 Dutch COVID-19 response team Erasmus Medical Center Bas Oude Munnink, David Nieuwenhuijse, Reina Sikkema, Claudia Schapendonk, Irina Chestakova, Anne van der Linden, Theo Bestebroer, Stefan van Nieuwkoop, Mark Pronk, Pascal Lexmond, Corien Swaan, Manon Haverkate, Madelief Mollers, Mart Stein, Sandra Kengne Kamga Mobou, Jeroen van Kampen, Jolanda Voermans, Aura Timen, Corine GeurtsvanKessel, Annemiek van der Eijk, Richard Molenkamp, Marion Koopmans, on behalf of the Dutch national COVID-19 response team.
EPI_ISL_422630 hCoV-19/Netherlands/ZuidHolland_94/2020 Europe / Netherlands / Zuid Holland 2020-03-31 Dutch COVID-19 response team Erasmus Medical Center Bas Oude Munnink, David Nieuwenhuijse, Reina Sikkema, Claudia Schapendonk, Irina Chestakova, Anne van der Linden, Theo Bestebroer, Stefan van Nieuwkoop, Mark Pronk, Pascal Lexmond, Corien Swaan, Manon Haverkate, Madelief Mollers, Mart Stein, Sandra Kengne Kamga Mobou, Jeroen van Kampen, Jolanda Voermans, Aura Timen, Corine GeurtsvanKessel, Annemiek van der Eijk, Richard Molenkamp, Marion Koopmans, on behalf of the Dutch national COVID-19 response team.
EPI_ISL_422631 hCoV-19/Netherlands/ZuidHolland_95/2020 Europe / Netherlands / Zuid Holland 2020-03-31 Dutch COVID-19 response team Erasmus Medical Center Bas Oude Munnink, David Nieuwenhuijse, Reina Sikkema, Claudia Schapendonk, Irina Chestakova, Anne van der Linden, Theo Bestebroer, Stefan van Nieuwkoop, Mark Pronk, Pascal Lexmond, Corien Swaan, Manon Haverkate, Madelief Mollers, Mart Stein, Sandra Kengne Kamga Mobou, Jeroen van Kampen, Jolanda Voermans, Aura Timen, Corine GeurtsvanKessel, Annemiek van der Eijk, Richard Molenkamp, Marion Koopmans, on behalf of the Dutch national COVID-19 response team.
EPI_ISL_422632 hCoV-19/Netherlands/ZuidHolland_96/2020 Europe / Netherlands / Zuid Holland 2020-03-30 Dutch COVID-19 response team Erasmus Medical Center Bas Oude Munnink, David Nieuwenhuijse, Reina Sikkema, Claudia Schapendonk, Irina Chestakova, Anne van der Linden, Theo Bestebroer, Stefan van Nieuwkoop, Mark Pronk, Pascal Lexmond, Corien Swaan, Manon Haverkate, Madelief Mollers, Mart Stein, Sandra Kengne Kamga Mobou, Jeroen van Kampen, Jolanda Voermans, Aura Timen, Corine GeurtsvanKessel, Annemiek van der Eijk, Richard Molenkamp, Marion Koopmans, on behalf of the Dutch national COVID-19 response team.
EPI_ISL_422633 hCoV-19/Netherlands/ZuidHolland_97/2020 Europe / Netherlands / Zuid Holland 2020-03-31 Dutch COVID-19 response team Erasmus Medical Center Bas Oude Munnink, David Nieuwenhuijse, Reina Sikkema, Claudia Schapendonk, Irina Chestakova, Anne van der Linden, Theo Bestebroer, Stefan van Nieuwkoop, Mark Pronk, Pascal Lexmond, Corien Swaan, Manon Haverkate, Madelief Mollers, Mart Stein, Sandra Kengne Kamga Mobou, Jeroen van Kampen, Jolanda Voermans, Aura Timen, Corine GeurtsvanKessel, Annemiek van der Eijk, Richard Molenkamp, Marion Koopmans, on behalf of the Dutch national COVID-19 response team.
EPI_ISL_422635 hCoV-19/Netherlands/ZuidHolland_99/2020 Europe / Netherlands / Zuid Holland 2020-03-31 Dutch COVID-19 response team Erasmus Medical Center Bas Oude Munnink, David Nieuwenhuijse, Reina Sikkema, Claudia Schapendonk, Irina Chestakova, Anne van der Linden, Theo Bestebroer, Stefan van Nieuwkoop, Mark Pronk, Pascal Lexmond, Corien Swaan, Manon Haverkate, Madelief Mollers, Mart Stein, Sandra Kengne Kamga Mobou, Jeroen van Kampen, Jolanda Voermans, Aura Timen, Corine GeurtsvanKessel, Annemiek van der Eijk, Richard Molenkamp, Marion Koopmans, on behalf of the Dutch national COVID-19 response team.
EPI_ISL_422636 hCoV-19/Czech Republic/2741/2020 Europe / Czech Republic / Prague 2020-03-08 The National Institute of Public Health Center for Epidemiology and Microbiology State Veterinary Institute Prague Alexander Nagy, Helena Jirincova, Klara Labska, Ludmila Novakova, Olga Storkanova, Dusan Trnka, Jaromira Vecerova

EPI_ISL_422638 hCoV-19/Netherlands/Gelderland_10/2020 Europe / Netherlands / Gelderland 2020-03-20 Dutch COVID-19 response team Erasmus Medical Center Bas Oude Munnink, David Nieuwenhuijse, Reina Sikkema, Claudia Schapendonk, Irina Chestakova, Anne van der Linden, Theo Bestebroer, Stefan van Nieuwkoop, Mark Pronk, Pascal Lexmond, Corien Swaan, Manon Haverkate, Madelief Mollers, Mart Stein, Sandra Kengne Kamga Mobou, Jeroen van Kampen, Jolanda Voermans, Aura Timen, Corine GeurtsvanKessel, Annemiek van der Eijk, Richard Molenkamp, Marion Koopmans, on behalf of the Dutch national COVID-19 response team.
EPI_ISL_422639 hCoV-19/Netherlands/Gelderland_11/2020 Europe / Netherlands / Gelderland 2020-03-21 Dutch COVID-19 response team Erasmus Medical Center Bas Oude Munnink, David Nieuwenhuijse, Reina Sikkema, Claudia Schapendonk, Irina Chestakova, Anne van der Linden, Theo Bestebroer, Stefan van Nieuwkoop, Mark Pronk, Pascal Lexmond, Corien Swaan, Manon Haverkate, Madelief Mollers, Mart Stein, Sandra Kengne Kamga Mobou, Jeroen van Kampen, Jolanda Voermans, Aura Timen, Corine GeurtsvanKessel, Annemiek van der Eijk, Richard Molenkamp, Marion Koopmans, on behalf of the Dutch national COVID-19 response team.
EPI_ISL_422640 hCoV-19/Netherlands/Gelderland_12/2020 Europe / Netherlands / Gelderland 2020-03-20 Dutch COVID-19 response team Erasmus Medical Center Bas Oude Munnink, David Nieuwenhuijse, Reina Sikkema, Claudia Schapendonk, Irina Chestakova, Anne van der Linden, Theo Bestebroer, Stefan van Nieuwkoop, Mark Pronk, Pascal Lexmond, Corien Swaan, Manon Haverkate, Madelief Mollers, Mart Stein, Sandra Kengne Kamga Mobou, Jeroen van Kampen, Jolanda Voermans, Aura Timen, Corine GeurtsvanKessel, Annemiek van der Eijk, Richard Molenkamp, Marion Koopmans, on behalf of the Dutch national COVID-19 response team.
EPI_ISL_422641 hCoV-19/Netherlands/Gelderland_5/2020 Europe / Netherlands / Gelderland 2020-03-10 Dutch COVID-19 response team Erasmus Medical Center Bas Oude Munnink, David Nieuwenhuijse, Reina Sikkema, Claudia Schapendonk, Irina Chestakova, Anne van der Linden, Theo Bestebroer, Stefan van Nieuwkoop, Mark Pronk, Pascal Lexmond, Corien Swaan, Manon Haverkate, Madelief Mollers, Mart Stein, Sandra Kengne Kamga Mobou, Jeroen van Kampen, Jolanda Voermans, Aura Timen, Corine GeurtsvanKessel, Annemiek van der Eijk, Richard Molenkamp, Marion Koopmans, on behalf of the Dutch national COVID-19 response team.
EPI_ISL_422642 hCoV-19/Netherlands/Gelderland_6/2020 Europe / Netherlands / Gelderland 2020-03-09 Dutch COVID-19 response team Erasmus Medical Center Bas Oude Munnink, David Nieuwenhuijse, Reina Sikkema, Claudia Schapendonk, Irina Chestakova, Anne van der Linden, Theo Bestebroer, Stefan van Nieuwkoop, Mark Pronk, Pascal Lexmond, Corien Swaan, Manon Haverkate, Madelief Mollers, Mart Stein, Sandra Kengne Kamga Mobou, Jeroen van Kampen, Jolanda Voermans, Aura Timen, Corine GeurtsvanKessel, Annemiek van der Eijk, Richard Molenkamp, Marion Koopmans, on behalf of the Dutch national COVID-19 response team.
EPI_ISL_422643 hCoV-19/Netherlands/Gelderland_7/2020 Europe / Netherlands / Gelderland 2020-03-11 Dutch COVID-19 response team Erasmus Medical Center Bas Oude Munnink, David Nieuwenhuijse, Reina Sikkema, Claudia Schapendonk, Irina Chestakova, Anne van der Linden, Theo Bestebroer, Stefan van Nieuwkoop, Mark Pronk, Pascal Lexmond, Corien Swaan, Manon Haverkate, Madelief Mollers, Mart Stein, Sandra Kengne Kamga Mobou, Jeroen van Kampen, Jolanda Voermans, Aura Timen, Corine GeurtsvanKessel, Annemiek van der Eijk, Richard Molenkamp, Marion Koopmans, on behalf of the Dutch national COVID-19 response team.
EPI_ISL_422644 hCoV-19/Netherlands/Gelderland_8/2020 Europe / Netherlands / Gelderland 2020-03-13 Dutch COVID-19 response team Erasmus Medical Center Bas Oude Munnink, David Nieuwenhuijse, Reina Sikkema, Claudia Schapendonk, Irina Chestakova, Anne van der Linden, Theo Bestebroer, Stefan van Nieuwkoop, Mark Pronk, Pascal Lexmond, Corien Swaan, Manon Haverkate, Madelief Mollers, Mart Stein, Sandra Kengne Kamga Mobou, Jeroen van Kampen, Jolanda Voermans, Aura Timen, Corine GeurtsvanKessel, Annemiek van der Eijk, Richard Molenkamp, Marion Koopmans, on behalf of the Dutch national COVID-19 response team.
EPI_ISL_422645 hCoV-19/Netherlands/Gelderland_9/2020 Europe / Netherlands / Gelderland 2020-03-18 Dutch COVID-19 response team Erasmus Medical Center Bas Oude Munnink, David Nieuwenhuijse, Reina Sikkema, Claudia Schapendonk, Irina Chestakova, Anne van der Linden, Theo Bestebroer, Stefan van Nieuwkoop, Mark Pronk, Pascal Lexmond, Corien Swaan, Manon Haverkate, Madelief Mollers, Mart Stein, Sandra Kengne Kamga Mobou, Jeroen van Kampen, Jolanda Voermans, Aura Timen, Corine GeurtsvanKessel, Annemiek van der Eijk, Richard Molenkamp, Marion Koopmans, on behalf of the Dutch national COVID-19 response team.
EPI_ISL_422646 hCoV-19/Netherlands/Limburg_10/2020 Europe / Netherlands / Limburg 2020-03-20 Dutch COVID-19 response team Erasmus Medical Center Bas Oude Munnink, David Nieuwenhuijse, Reina Sikkema, Claudia Schapendonk, Irina Chestakova, Anne van der Linden, Theo Bestebroer, Stefan van Nieuwkoop, Mark Pronk, Pascal Lexmond, Corien Swaan, Manon Haverkate, Madelief Mollers, Mart Stein, Sandra Kengne Kamga Mobou, Jeroen van Kampen, Jolanda Voermans, Aura Timen, Corine GeurtsvanKessel, Annemiek van der Eijk, Richard Molenkamp, Marion Koopmans, on behalf of the Dutch national COVID-19 response team.
EPI_ISL_422647 hCoV-19/Netherlands/Limburg_8/2020 Europe / Netherlands / Limburg 2020-03-19 Dutch COVID-19 response team Erasmus Medical Center Bas Oude Munnink, David Nieuwenhuijse, Reina Sikkema, Claudia Schapendonk, Irina Chestakova, Anne van der Linden, Theo Bestebroer, Stefan van Nieuwkoop, Mark Pronk, Pascal Lexmond, Corien Swaan, Manon Haverkate, Madelief Mollers, Mart Stein, Sandra Kengne Kamga Mobou, Jeroen van Kampen, Jolanda Voermans, Aura Timen, Corine GeurtsvanKessel, Annemiek van der Eijk, Richard Molenkamp, Marion Koopmans, on behalf of the Dutch national COVID-19 response team.
EPI_ISL_422648 hCoV-19/Netherlands/Limburg_9/2020 Europe / Netherlands / Limburg 2020-03-20 Dutch COVID-19 response team Erasmus Medical Center Bas Oude Munnink, David Nieuwenhuijse, Reina Sikkema, Claudia Schapendonk, Irina Chestakova, Anne van der Linden, Theo Bestebroer, Stefan van Nieuwkoop, Mark Pronk, Pascal Lexmond, Corien Swaan, Manon Haverkate, Madelief Mollers, Mart Stein, Sandra Kengne Kamga Mobou, Jeroen van Kampen, Jolanda Voermans, Aura Timen, Corine GeurtsvanKessel, Annemiek van der Eijk, Richard Molenkamp, Marion Koopmans, on behalf of the Dutch national COVID-19 response team.
EPI_ISL_422649 hCoV-19/Netherlands/NA_100/2020 Europe / Netherlands 2020-03-18 Dutch COVID-19 response team Erasmus Medical Center Bas Oude Munnink, David Nieuwenhuijse, Reina Sikkema, Claudia Schapendonk, Irina Chestakova, Anne van der Linden, Theo Bestebroer, Stefan van Nieuwkoop, Mark Pronk, Pascal Lexmond, Corien Swaan, Manon Haverkate, Madelief Mollers, Mart Stein, Sandra Kengne Kamga Mobou, Jeroen van Kampen,

Jolanda Voermans, Aura Timen, Corine GeurtsvanKessel, Annemiek van der Eijk, Richard Molenkamp, Marion Koopmans, on behalf of the Dutch national COVID-19 response team.
EPI_ISL_422650 hCoV-19/Netherlands/NA_101/2020 Europe / Netherlands 2020-03-18 Dutch COVID-19 response team Erasmus Medical Center Bas Oude Munnink, David Nieuwenhuijse, Reina Sikkema, Claudia Schapendonk, Irina Chestakova, Anne van der Linden, Theo Bestebroer, Stefan van Nieuwkoop, Mark Pronk, Pascal Lexmond, Corien Swaan, Manon Haverkate, Madelief Mollers, Mart Stein, Sandra Kengne Kamga Mobou, Jeroen van Kampen, Jolanda Voermans, Aura Timen, Corine GeurtsvanKessel, Annemiek van der Eijk, Richard Molenkamp, Marion Koopmans, on behalf of the Dutch national COVID-19 response team.
EPI_ISL_422651 hCoV-19/Netherlands/NA_102/2020 Europe / Netherlands 2020-03-18 Dutch COVID-19 response team Erasmus Medical Center Bas Oude Munnink, David Nieuwenhuijse, Reina Sikkema, Claudia Schapendonk, Irina Chestakova, Anne van der Linden, Theo Bestebroer, Stefan van Nieuwkoop, Mark Pronk, Pascal Lexmond, Corien Swaan, Manon Haverkate, Madelief Mollers, Mart Stein, Sandra Kengne Kamga Mobou, Jeroen van Kampen, Jolanda Voermans, Aura Timen, Corine GeurtsvanKessel, Annemiek van der Eijk, Richard Molenkamp, Marion Koopmans, on behalf of the Dutch national COVID-19 response team.
EPI_ISL_422652 hCoV-19/Netherlands/NA_103/2020 Europe / Netherlands 2020-03-18 Dutch COVID-19 response team Erasmus Medical Center Bas Oude Munnink, David Nieuwenhuijse, Reina Sikkema, Claudia Schapendonk, Irina Chestakova, Anne van der Linden, Theo Bestebroer, Stefan van Nieuwkoop, Mark Pronk, Pascal Lexmond, Corien Swaan, Manon Haverkate, Madelief Mollers, Mart Stein, Sandra Kengne Kamga Mobou, Jeroen van Kampen, Jolanda Voermans, Aura Timen, Corine GeurtsvanKessel, Annemiek van der Eijk, Richard Molenkamp, Marion Koopmans, on behalf of the Dutch national COVID-19 response team.
EPI_ISL_422653 hCoV-19/Netherlands/NA_104/2020 Europe / Netherlands 2020-03-18 Dutch COVID-19 response team Erasmus Medical Center Bas Oude Munnink, David Nieuwenhuijse, Reina Sikkema, Claudia Schapendonk, Irina Chestakova, Anne van der Linden, Theo Bestebroer, Stefan van Nieuwkoop, Mark Pronk, Pascal Lexmond, Corien Swaan, Manon Haverkate, Madelief Mollers, Mart Stein, Sandra Kengne Kamga Mobou, Jeroen van Kampen, Jolanda Voermans, Aura Timen, Corine GeurtsvanKessel, Annemiek van der Eijk, Richard Molenkamp, Marion Koopmans, on behalf of the Dutch national COVID-19 response team.
EPI_ISL_423202 hCoV-19/England/201360052/2020 Europe / United Kingdom / England 2020-03-24 Respiratory Virus Unit, Microbiology Services Colindale, Public Health England Respiratory Virus Unit, Microbiology Services Colindale, Public Health England Monica Galiano, Shahjahan Miah, Angie Lackenby, Omolola Akinbami, Tiina Talts, Leena Bhaw, Richard Myers, Steven Platt, Kirstin Edwards, Jonathan Hubb, Joanna Ellis, Maria Zambon
EPI_ISL_423203 hCoV-19/England/201360053/2020 Europe / United Kingdom / England 2020-03-24 Respiratory Virus Unit, Microbiology Services Colindale, Public Health England Respiratory Virus Unit, Microbiology Services Colindale, Public Health England Monica Galiano, Shahjahan Miah, Angie Lackenby, Omolola Akinbami, Tiina Talts, Leena Bhaw, Richard Myers, Steven Platt, Kirstin Edwards, Jonathan Hubb, Joanna Ellis, Maria Zambon
EPI_ISL_423204 hCoV-19/England/201360055/2020 Europe / United Kingdom / England 2020-03-26 Respiratory Virus Unit, Microbiology Services Colindale, Public Health England Respiratory Virus Unit, Microbiology Services Colindale, Public Health England Monica Galiano, Shahjahan Miah, Angie Lackenby, Omolola Akinbami, Tiina Talts, Leena Bhaw, Richard Myers, Steven Platt, Kirstin Edwards, Jonathan Hubb, Joanna Ellis, Maria Zambon
EPI_ISL_423205 hCoV-19/England/201360057/2020 Europe / United Kingdom / England 2020-03-25 Respiratory Virus Unit, Microbiology Services Colindale, Public Health England Respiratory Virus Unit, Microbiology Services Colindale, Public Health England Monica Galiano, Shahjahan Miah, Angie Lackenby, Omolola Akinbami, Tiina Talts, Leena Bhaw, Richard Myers, Steven Platt, Kirstin Edwards, Jonathan Hubb, Joanna Ellis, Maria Zambon
EPI_ISL_423208 hCoV-19/England/201360069/2020 Europe / United Kingdom / England 2020-03-24 Respiratory Virus Unit, Microbiology Services Colindale, Public Health England Respiratory Virus Unit, Microbiology Services Colindale, Public Health England Monica Galiano, Shahjahan Miah, Angie Lackenby, Omolola Akinbami, Tiina Talts, Leena Bhaw, Richard Myers, Steven Platt, Kirstin Edwards, Jonathan Hubb, Joanna Ellis, Maria Zambon
EPI_ISL_423209 hCoV-19/England/201360071/2020 Europe / United Kingdom / England 2020-03-24 Respiratory Virus Unit, Microbiology Services Colindale, Public Health England Respiratory Virus Unit, Microbiology Services Colindale, Public Health England Monica Galiano, Shahjahan Miah, Angie Lackenby, Omolola Akinbami, Tiina Talts, Leena Bhaw, Richard Myers, Steven Platt, Kirstin Edwards, Jonathan Hubb, Joanna Ellis, Maria Zambon
EPI_ISL_423210 hCoV-19/England/201360075/2020 Europe / United Kingdom / England 2020-03-27 Respiratory Virus Unit, Microbiology Services Colindale, Public Health England Respiratory Virus Unit, Microbiology Services Colindale, Public Health England Monica Galiano, Shahjahan Miah, Angie Lackenby, Omolola Akinbami, Tiina Talts, Leena Bhaw, Richard Myers, Steven Platt, Kirstin Edwards, Jonathan Hubb, Joanna Ellis, Maria Zambon
EPI_ISL_423214 hCoV-19/England/201360119/2020 Europe / United Kingdom / England 2020-03-25 Respiratory Virus Unit, Microbiology Services Colindale, Public Health England Respiratory Virus Unit, Microbiology Services Colindale, Public Health England Monica Galiano, Shahjahan Miah, Angie Lackenby, Omolola Akinbami, Tiina Talts, Leena Bhaw, Richard Myers, Steven Platt, Kirstin Edwards, Jonathan Hubb, Joanna Ellis, Maria Zambon
EPI_ISL_423215 hCoV-19/England/20136012004/2020 Europe / United Kingdom / England 2020-03-25 Respiratory Virus Unit, Microbiology Services Colindale, Public Health England Respiratory Virus Unit, Microbiology Services Colindale, Public Health England Monica Galiano, Shahjahan Miah, Angie Lackenby, Omolola Akinbami, Tiina Talts, Leena Bhaw, Richard Myers, Steven Platt, Kirstin Edwards, Jonathan Hubb, Joanna Ellis,

Maria Zambon
EPI_ISL_423216 hCoV-19/England/201360121/2020 Europe / United Kingdom / England 2020-03-25 Respiratory Virus Unit, Microbiology Services Colindale, Public Health England Respiratory Virus Unit, Microbiology Services Colindale, Public Health England Monica Galiano, Shahjahan Miah, Angie Lackenby, Omolola Akinbami, Tiina Talts, Leena Bhaw, Richard Myers, Steven Platt, Kirstin Edwards, Jonathan Hubb, Joanna Ellis, Maria Zambon
EPI_ISL_423218 hCoV-19/England/201360126/2020 Europe / United Kingdom / England 2020-03-25 Respiratory Virus Unit, Microbiology Services Colindale, Public Health England Respiratory Virus Unit, Microbiology Services Colindale, Public Health England Monica Galiano, Shahjahan Miah, Angie Lackenby, Omolola Akinbami, Tiina Talts, Leena Bhaw, Richard Myers, Steven Platt, Kirstin Edwards, Jonathan Hubb, Joanna Ellis, Maria Zambon
EPI_ISL_423219 hCoV-19/England/201360127/2020 Europe / United Kingdom / England 2020-03-25 Respiratory Virus Unit, Microbiology Services Colindale, Public Health England Respiratory Virus Unit, Microbiology Services Colindale, Public Health England Monica Galiano, Shahjahan Miah, Angie Lackenby, Omolola Akinbami, Tiina Talts, Leena Bhaw, Richard Myers, Steven Platt, Kirstin Edwards, Jonathan Hubb, Joanna Ellis, Maria Zambon
EPI_ISL_423221 hCoV-19/England/201360131/2020 Europe / United Kingdom / England 2020-03-24 Respiratory Virus Unit, Microbiology Services Colindale, Public Health England Respiratory Virus Unit, Microbiology Services Colindale, Public Health England Monica Galiano, Shahjahan Miah, Angie Lackenby, Omolola Akinbami, Tiina Talts, Leena Bhaw, Richard Myers, Steven Platt, Kirstin Edwards, Jonathan Hubb, Joanna Ellis, Maria Zambon
EPI_ISL_423223 hCoV-19/England/201360136/2020 Europe / United Kingdom / England 2020-03-25 Respiratory Virus Unit, Microbiology Services Colindale, Public Health England Respiratory Virus Unit, Microbiology Services Colindale, Public Health England Monica Galiano, Shahjahan Miah, Angie Lackenby, Omolola Akinbami, Tiina Talts, Leena Bhaw, Richard Myers, Steven Platt, Kirstin Edwards, Jonathan Hubb, Joanna Ellis, Maria Zambon
EPI_ISL_423224 hCoV-19/England/201360137/2020 Europe / United Kingdom / England 2020-03-26 Respiratory Virus Unit, Microbiology Services Colindale, Public Health England Respiratory Virus Unit, Microbiology Services Colindale, Public Health England Monica Galiano, Shahjahan Miah, Angie Lackenby, Omolola Akinbami, Tiina Talts, Leena Bhaw, Richard Myers, Steven Platt, Kirstin Edwards, Jonathan Hubb, Joanna Ellis, Maria Zambon
EPI_ISL_423227 hCoV-19/England/201360143/2020 Europe / United Kingdom / England 2020-03-24 Respiratory Virus Unit, Microbiology Services Colindale, Public Health England Respiratory Virus Unit, Microbiology Services Colindale, Public Health England Monica Galiano, Shahjahan Miah, Angie Lackenby, Omolola Akinbami, Tiina Talts, Leena Bhaw, Richard Myers, Steven Platt, Kirstin Edwards, Jonathan Hubb, Joanna Ellis, Maria Zambon
EPI_ISL_423228 hCoV-19/England/201360171/2020 Europe / United Kingdom / England 2020-03-25 Respiratory Virus Unit, Microbiology Services Colindale, Public Health England Respiratory Virus Unit, Microbiology Services Colindale, Public Health England Monica Galiano, Shahjahan Miah, Angie Lackenby, Omolola Akinbami, Tiina Talts, Leena Bhaw, Richard Myers, Steven Platt, Kirstin Edwards, Jonathan Hubb, Joanna Ellis, Maria Zambon
EPI_ISL_423231 hCoV-19/England/201360178/2020 Europe / United Kingdom / England 2020-03-24 Respiratory Virus Unit, Microbiology Services Colindale, Public Health England Respiratory Virus Unit, Microbiology Services Colindale, Public Health England Monica Galiano, Shahjahan Miah, Angie Lackenby, Omolola Akinbami, Tiina Talts, Leena Bhaw, Richard Myers, Steven Platt, Kirstin Edwards, Jonathan Hubb, Joanna Ellis, Maria Zambon
EPI_ISL_423232 hCoV-19/England/201360181/2020 Europe / United Kingdom / England 2020-03-26 Respiratory Virus Unit, Microbiology Services Colindale, Public Health England Respiratory Virus Unit, Microbiology Services Colindale, Public Health England Monica Galiano, Shahjahan Miah, Angie Lackenby, Omolola Akinbami, Tiina Talts, Leena Bhaw, Richard Myers, Steven Platt, Kirstin Edwards, Jonathan Hubb, Joanna Ellis, Maria Zambon
EPI_ISL_423233 hCoV-19/England/201360188/2020 Europe / United Kingdom / England 2020-03-24 Respiratory Virus Unit, Microbiology Services Colindale, Public Health England Respiratory Virus Unit, Microbiology Services Colindale, Public Health England Monica Galiano, Shahjahan Miah, Angie Lackenby, Omolola Akinbami, Tiina Talts, Leena Bhaw, Richard Myers, Steven Platt, Kirstin Edwards, Jonathan Hubb, Joanna Ellis, Maria Zambon
EPI_ISL_423235 hCoV-19/England/201360191/2020 Europe / United Kingdom / England 2020-03-24 Respiratory Virus Unit, Microbiology Services Colindale, Public Health England Respiratory Virus Unit, Microbiology Services Colindale, Public Health England Monica Galiano, Shahjahan Miah, Angie Lackenby, Omolola Akinbami, Tiina Talts, Leena Bhaw, Richard Myers, Steven Platt, Kirstin Edwards, Jonathan Hubb, Joanna Ellis, Maria Zambon
EPI_ISL_423236 hCoV-19/England/201360198/2020 Europe / United Kingdom / England 2020-03-24 Respiratory Virus Unit, Microbiology Services Colindale, Public Health England Respiratory Virus Unit, Microbiology Services Colindale, Public Health England Monica Galiano, Shahjahan Miah, Angie Lackenby, Omolola Akinbami, Tiina Talts, Leena Bhaw, Richard Myers, Steven Platt, Kirstin Edwards, Jonathan Hubb, Joanna Ellis, Maria Zambon
EPI_ISL_423237 hCoV-19/England/201360200/2020 Europe / United Kingdom / England 2020-03-24 Respiratory Virus Unit, Microbiology Services Colindale, Public Health England Respiratory Virus Unit, Microbiology Services Colindale, Public Health England Monica Galiano, Shahjahan Miah, Angie Lackenby, Omolola Akinbami, Tiina Talts, Leena Bhaw, Richard Myers, Steven Platt, Kirstin Edwards, Jonathan Hubb, Joanna Ellis,

Maria Zambon
EPI_ISL_423238 hCoV-19/England/201360201/2020 Europe / United Kingdom / England 2020-03-24 Respiratory Virus Unit, Microbiology Services Colindale, Public Health England Respiratory Virus Unit, Microbiology Services Colindale, Public Health England Monica Galiano, Shahjahan Miah, Angie Lackenby, Omolola Akinbami, Tiina Talts, Leena Bhaw, Richard Myers, Steven Platt, Kirstin Edwards, Jonathan Hubb, Joanna Ellis, Maria Zambon
EPI_ISL_423241 hCoV-19/England/201360206/2020 Europe / United Kingdom / England 2020-03-24 Respiratory Virus Unit, Microbiology Services Colindale, Public Health England Respiratory Virus Unit, Microbiology Services Colindale, Public Health England Monica Galiano, Shahjahan Miah, Angie Lackenby, Omolola Akinbami, Tiina Talts, Leena Bhaw, Richard Myers, Steven Platt, Kirstin Edwards, Jonathan Hubb, Joanna Ellis, Maria Zambon
EPI_ISL_423242 hCoV-19/England/201360210/2020 Europe / United Kingdom / England 2020-03-25 Respiratory Virus Unit, Microbiology Services Colindale, Public Health England Respiratory Virus Unit, Microbiology Services Colindale, Public Health England Monica Galiano, Shahjahan Miah, Angie Lackenby, Omolola Akinbami, Tiina Talts, Leena Bhaw, Richard Myers, Steven Platt, Kirstin Edwards, Jonathan Hubb, Joanna Ellis, Maria Zambon
EPI_ISL_423243 hCoV-19/England/201360211/2020 Europe / United Kingdom / England 2020-03-25 Respiratory Virus Unit, Microbiology Services Colindale, Public Health England Respiratory Virus Unit, Microbiology Services Colindale, Public Health England Monica Galiano, Shahjahan Miah, Angie Lackenby, Omolola Akinbami, Tiina Talts, Leena Bhaw, Richard Myers, Steven Platt, Kirstin Edwards, Jonathan Hubb, Joanna Ellis, Maria Zambon
EPI_ISL_423244 hCoV-19/England/201360213/2020 Europe / United Kingdom / England 2020-03-25 Respiratory Virus Unit, Microbiology Services Colindale, Public Health England Respiratory Virus Unit, Microbiology Services Colindale, Public Health England Monica Galiano, Shahjahan Miah, Angie Lackenby, Omolola Akinbami, Tiina Talts, Leena Bhaw, Richard Myers, Steven Platt, Kirstin Edwards, Jonathan Hubb, Joanna Ellis, Maria Zambon
EPI_ISL_423246 hCoV-19/England/201360215/2020 Europe / United Kingdom / England 2020-03-25 Respiratory Virus Unit, Microbiology Services Colindale, Public Health England Respiratory Virus Unit, Microbiology Services Colindale, Public Health England Monica Galiano, Shahjahan Miah, Angie Lackenby, Omolola Akinbami, Tiina Talts, Leena Bhaw, Richard Myers, Steven Platt, Kirstin Edwards, Jonathan Hubb, Joanna Ellis, Maria Zambon
EPI_ISL_423248 hCoV-19/England/201360217/2020 Europe / United Kingdom / England 2020-03-25 Respiratory Virus Unit, Microbiology Services Colindale, Public Health England Respiratory Virus Unit, Microbiology Services Colindale, Public Health England Monica Galiano, Shahjahan Miah, Angie Lackenby, Omolola Akinbami, Tiina Talts, Leena Bhaw, Richard Myers, Steven Platt, Kirstin Edwards, Jonathan Hubb, Joanna Ellis, Maria Zambon
EPI_ISL_423769 hCoV-19/England/20132031404/2020 Europe / United Kingdom / England 2020-03-22 Respiratory Virus Unit, Microbiology Services Colindale, Public Health England Respiratory Virus Unit, Microbiology Services Colindale, Public Health England Monica Galiano, Shahjahan Miah, Angie Lackenby, Omolola Akinbami, Tiina Talts, Leena Bhaw, Richard Myers, Steven Platt, Kirstin Edwards, Jonathan Hubb, Joanna Ellis, Maria Zambon
EPI_ISL_423771 hCoV-19/England/20132032504/2020 Europe / United Kingdom / England 2020-03-24 Respiratory Virus Unit, Microbiology Services Colindale, Public Health England Respiratory Virus Unit, Microbiology Services Colindale, Public Health England Monica Galiano, Shahjahan Miah, Angie Lackenby, Omolola Akinbami, Tiina Talts, Leena Bhaw, Richard Myers, Steven Platt, Kirstin Edwards, Jonathan Hubb, Joanna Ellis, Maria Zambon
EPI_ISL_423772 hCoV-19/England/20132032604/2020 Europe / United Kingdom / England 2020-03-22 Respiratory Virus Unit, Microbiology Services Colindale, Public Health England Respiratory Virus Unit, Microbiology Services Colindale, Public Health England Monica Galiano, Shahjahan Miah, Angie Lackenby, Omolola Akinbami, Tiina Talts, Leena Bhaw, Richard Myers, Steven Platt, Kirstin Edwards, Jonathan Hubb, Joanna Ellis, Maria Zambon
EPI_ISL_423773 hCoV-19/England/20132032704/2020 Europe / United Kingdom / England 2020-03-24 Respiratory Virus Unit, Microbiology Services Colindale, Public Health England Respiratory Virus Unit, Microbiology Services Colindale, Public Health England Monica Galiano, Shahjahan Miah, Angie Lackenby, Omolola Akinbami, Tiina Talts, Leena Bhaw, Richard Myers, Steven Platt, Kirstin Edwards, Jonathan Hubb, Joanna Ellis, Maria Zambon
EPI_ISL_423774 hCoV-19/England/20132032804/2020 Europe / United Kingdom / England 2020-03-22 Respiratory Virus Unit, Microbiology Services Colindale, Public Health England Respiratory Virus Unit, Microbiology Services Colindale, Public Health England Monica Galiano, Shahjahan Miah, Angie Lackenby, Omolola Akinbami, Tiina Talts, Leena Bhaw, Richard Myers, Steven Platt, Kirstin Edwards, Jonathan Hubb, Joanna Ellis, Maria Zambon
EPI_ISL_423775 hCoV-19/England/20132032904/2020 Europe / United Kingdom / England 2020-03-22 Respiratory Virus Unit, Microbiology Services Colindale, Public Health England Respiratory Virus Unit, Microbiology Services Colindale, Public Health England Monica Galiano, Shahjahan Miah, Angie Lackenby, Omolola Akinbami, Tiina Talts, Leena Bhaw, Richard Myers, Steven Platt, Kirstin Edwards, Jonathan Hubb, Joanna Ellis, Maria Zambon
EPI_ISL_423778 hCoV-19/England/20132035704/2020 Europe / United Kingdom / England 2020-03-24 Respiratory Virus Unit, Microbiology Services Colindale, Public Health England Respiratory Virus Unit, Microbiology Services Colindale, Public Health England Monica Galiano, Shahjahan Miah, Angie Lackenby, Omolola Akinbami, Tiina Talts, Leena Bhaw, Richard Myers, Steven Platt, Kirstin Edwards, Jonathan Hubb, Joanna Ellis,

Maria Zambon
EPI_ISL_423779 hCoV-19/England/20132035904/2020 Europe / United Kingdom / England 2020-03-23 Respiratory Virus Unit, Microbiology Services Colindale, Public Health England Respiratory Virus Unit, Microbiology Services Colindale, Public Health England Monica Galiano, Shahjahan Miah, Angie Lackenby, Omolola Akinbami, Tiina Talts, Leena Bhaw, Richard Myers, Steven Platt, Kirstin Edwards, Jonathan Hubb, Joanna Ellis, Maria Zambon
EPI_ISL_423782 hCoV-19/England/20132047304/2020 Europe / United Kingdom / England 2020-03-23 Respiratory Virus Unit, Microbiology Services Colindale, Public Health England Respiratory Virus Unit, Microbiology Services Colindale, Public Health England Monica Galiano, Shahjahan Miah, Angie Lackenby, Omolola Akinbami, Tiina Talts, Leena Bhaw, Richard Myers, Steven Platt, Kirstin Edwards, Jonathan Hubb, Joanna Ellis, Maria Zambon
EPI_ISL_423783 hCoV-19/England/20132047404/2020 Europe / United Kingdom / England 2020-03-24 Respiratory Virus Unit, Microbiology Services Colindale, Public Health England Respiratory Virus Unit, Microbiology Services Colindale, Public Health England Monica Galiano, Shahjahan Miah, Angie Lackenby, Omolola Akinbami, Tiina Talts, Leena Bhaw, Richard Myers, Steven Platt, Kirstin Edwards, Jonathan Hubb, Joanna Ellis, Maria Zambon
EPI_ISL_423786 hCoV-19/England/20132054604/2020 Europe / United Kingdom / England 2020-03-23 Respiratory Virus Unit, Microbiology Services Colindale, Public Health England Respiratory Virus Unit, Microbiology Services Colindale, Public Health England Monica Galiano, Shahjahan Miah, Angie Lackenby, Omolola Akinbami, Tiina Talts, Leena Bhaw, Richard Myers, Steven Platt, Kirstin Edwards, Jonathan Hubb, Joanna Ellis, Maria Zambon
EPI_ISL_423787 hCoV-19/England/20132054704/2020 Europe / United Kingdom / England 2020-03-23 Respiratory Virus Unit, Microbiology Services Colindale, Public Health England Respiratory Virus Unit, Microbiology Services Colindale, Public Health England Monica Galiano, Shahjahan Miah, Angie Lackenby, Omolola Akinbami, Tiina Talts, Leena Bhaw, Richard Myers, Steven Platt, Kirstin Edwards, Jonathan Hubb, Joanna Ellis, Maria Zambon
EPI_ISL_423791 hCoV-19/England/20132066804/2020 Europe / United Kingdom / England 2020-03-23 Respiratory Virus Unit, Microbiology Services Colindale, Public Health England Respiratory Virus Unit, Microbiology Services Colindale, Public Health England Monica Galiano, Shahjahan Miah, Angie Lackenby, Omolola Akinbami, Tiina Talts, Leena Bhaw, Richard Myers, Steven Platt, Kirstin Edwards, Jonathan Hubb, Joanna Ellis, Maria Zambon
EPI_ISL_423796 hCoV-19/England/20132068204/2020 Europe / United Kingdom / England 2020-03-23 Respiratory Virus Unit, Microbiology Services Colindale, Public Health England Respiratory Virus Unit, Microbiology Services Colindale, Public Health England Monica Galiano, Shahjahan Miah, Angie Lackenby, Omolola Akinbami, Tiina Talts, Leena Bhaw, Richard Myers, Steven Platt, Kirstin Edwards, Jonathan Hubb, Joanna Ellis, Maria Zambon
EPI_ISL_423797 hCoV-19/England/20132068304/2020 Europe / United Kingdom / England 2020-03-23 Respiratory Virus Unit, Microbiology Services Colindale, Public Health England Respiratory Virus Unit, Microbiology Services Colindale, Public Health England Monica Galiano, Shahjahan Miah, Angie Lackenby, Omolola Akinbami, Tiina Talts, Leena Bhaw, Richard Myers, Steven Platt, Kirstin Edwards, Jonathan Hubb, Joanna Ellis, Maria Zambon
EPI_ISL_423798 hCoV-19/England/20132069004/2020 Europe / United Kingdom / England 2020-03-23 Respiratory Virus Unit, Microbiology Services Colindale, Public Health England Respiratory Virus Unit, Microbiology Services Colindale, Public Health England Monica Galiano, Shahjahan Miah, Angie Lackenby, Omolola Akinbami, Tiina Talts, Leena Bhaw, Richard Myers, Steven Platt, Kirstin Edwards, Jonathan Hubb, Joanna Ellis, Maria Zambon
EPI_ISL_423799 hCoV-19/England/20132069104/2020 Europe / United Kingdom / England 2020-03-23 Respiratory Virus Unit, Microbiology Services Colindale, Public Health England Respiratory Virus Unit, Microbiology Services Colindale, Public Health England Monica Galiano, Shahjahan Miah, Angie Lackenby, Omolola Akinbami, Tiina Talts, Leena Bhaw, Richard Myers, Steven Platt, Kirstin Edwards, Jonathan Hubb, Joanna Ellis, Maria Zambon
EPI_ISL_423803 hCoV-19/England/20132080004/2020 Europe / United Kingdom / England 2020-03-24 Respiratory Virus Unit, Microbiology Services Colindale, Public Health England Respiratory Virus Unit, Microbiology Services Colindale, Public Health England Monica Galiano, Shahjahan Miah, Angie Lackenby, Omolola Akinbami, Tiina Talts, Leena Bhaw, Richard Myers, Steven Platt, Kirstin Edwards, Jonathan Hubb, Joanna Ellis, Maria Zambon
EPI_ISL_423808 hCoV-19/England/20132080804/2020 Europe / United Kingdom / England 2020-03-23 Respiratory Virus Unit, Microbiology Services Colindale, Public Health England Respiratory Virus Unit, Microbiology Services Colindale, Public Health England Monica Galiano, Shahjahan Miah, Angie Lackenby, Omolola Akinbami, Tiina Talts, Leena Bhaw, Richard Myers, Steven Platt, Kirstin Edwards, Jonathan Hubb, Joanna Ellis, Maria Zambon
EPI_ISL_423809 hCoV-19/England/20132081104/2020 Europe / United Kingdom / England 2020-03-24 Respiratory Virus Unit, Microbiology Services Colindale, Public Health England Respiratory Virus Unit, Microbiology Services Colindale, Public Health England Monica Galiano, Shahjahan Miah, Angie Lackenby, Omolola Akinbami, Tiina Talts, Leena Bhaw, Richard Myers, Steven Platt, Kirstin Edwards, Jonathan Hubb, Joanna Ellis, Maria Zambon
[truncated: 367,277 more chars]
