# Supplementary figures and images for: Emergence of novel SARS-CoV-2 variants in the Netherlands
Source: Sci Rep. 2021 Mar 23;11:6625. doi: 10.1038/s41598-021-85363-7 (PMC7988010; doi:10.1038/s41598-021-85363-7)

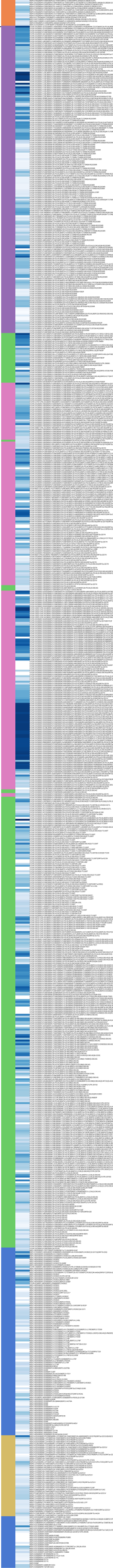[illegible]

Supplement: Supplementary file 5 — Supplementary Information 5. [file 41598_2021_85363_MOESM5_ESM.pdf]
